# Signatures of gene transfer in the parallel evolution of osmotrophic specialization in eukaryotes

**DOI:** 10.1038/s41559-026-03054-w

**Published:** 2026-05-25

**Authors:** Eduard Ocaña-Pallarès, Thomas A. Richards, Toni Gabaldón, Gergely J. Szöllősi

**Affiliations:** 1 UOC-TECH, Universitat Oberta de Catalunya, Barcelona, Spain; 2 Department of Biological Physics, Eötvös Loránd University, Budapest, Hungary; 3 BSC-CNS, Barcelona Supercomputing Center, Barcelona, Spain; 4 Institute for Research in Biomedicine, The Barcelona Institute of Science and Technology, Barcelona, Spain; 5 Departament de Medicina i Ciències de la Vida, Institut de Biologia Evolutiva (CSIC-UPF), Universitat Pompeu Fabra, Barcelona, Spain; 6 Department of Biology, University of Oxford, Oxford, UK; 7 Catalan Institution for Research and Advanced Studies, Barcelona, Spain; 8 Centro de Investigación Biomédica En Red de Enfermedades Infecciosas, Barcelona, Spain; 9 Model-Based Evolutionary Genomics Unit, Okinawa Institute of Science and Technology Graduate University, Okinawa, Japan; 10 HUN-REN Centre for Ecological Research, Institute of Evolution, Budapest, Hungary

**Keywords:** Molecular evolution, Comparative genomics, Phylogenetics

## Abstract

Recurrent transitions in feeding strategies have shaped the eukaryotic tree of life, as unrelated groups independently evolved similar solutions to common ecological challenges. One of the most interesting yet poorly studied of these shifts is the transition towards osmotrophy. We reconstructed the evolution of four eukaryotic groups that specialized in osmotrophy through convergent evolution. Here we show that these groups arose most likely during the Tonian period (1,000–720 million years ago) or slightly before, and possess a genetic toolkit enriched in shared metabolic functions. We report signatures that are compatible with horizontal gene transfer encompassing at least 20% of this toolkit. Phylogenetic reconciliation analyses show that this fraction of the toolkit ranks in the upper percentiles for inferred horizontal gene transfers, particularly in the period in which the osmotrophic groups originated. Moreover, analyses of the total gene content using supervised phylogenetic screening identified 166 gene tree topologies that are supportive of transfer events involving distantly related eukaryotic osmotrophs. These data include transfer highways between Fungi and Pseudofungi and between Labyrinthulea and Teretosporea. Our work thus unravels the evolutionary history of four independent transitions towards specialization in osmotrophy within the eukaryotes, supporting a role of gene transfer in the evolution of these groups.

## Abstract (web summary)

Comparative genomics of 86 eukaryotic species reveals a shared genetic toolkit enriched in metabolic functions among osmotrophs that was shaped by early horizontal gene transfer and more recent transfers among distantly related eukaryotic osmotrophs.

## Main

The evolutionary history of eukaryotes is punctuated by major trophic transitions, where shared ecologies have driven distant lineages to evolve analogous forms and functions^1,2^. One of the most interesting yet poorly studied of these shifts is the transition from a phagotrophic ancestral state of engulfing prey^3–6^ to specialized osmotrophy^7,8^. Osmotrophy is a form of heterotrophic nutrition based on the absorption of nutrients directly from the environment, often via extracellular digestion of complex molecules, without relying on phagocytosis^7^. While this strategy is to some extent present in many organisms, some groups have independently evolved a series of adaptations that allow them to specialize in this trophic mode^7,9^.

Among eukaryotes, Fungi^10^ are a classic example of forms that specialize in osmotrophy, yet other groups such as Teretosporea^11–13^, Pseudofungi^14,15^ and Labyrinthulea^16,17^ are morphologically similar to Fungi because they also specialize in osmotrophy. On the one hand, Fungi and the Teretosporea clade (Ichthyosporea and Corallochytrea) belong to the Opisthokonta group from the Amorphea division of eukaryotes^13^. Members of Teretosporea are frequently found as parasites or symbionts of various animals^12,18^, although free-living representatives have been described^11,12,19^. Although some Teretosporea were historically misclassified^11,20,21^ owing to fungal-like morphology, they are phylogenetically closer to animals than to Fungi^13^. On the other hand, Labyrinthulea^16,17^ and Pseudofungi^14,15^ belong to the Stramenopiles^22^ group from the Diaphoretickes division of eukaryotes^6^. Labyrinthulea includes saprotrophic species as well as symbionts of algae, marine plants and animals^16^. Similar to ichthyosporeans^19^, some species have been described as parasites, commensals or mutualists of invertebrates^16^. Regarding Pseudofungi (Hyphochytriomycota and Oomycota)^14,15,23^, Hyphochytriomycota are widespread in occurrence, and most are saprotrophs or parasites^15^. Oomycetes are numerous in marine, freshwater and terrestrial ecosystems, where they occur as widespread saprotrophs or parasites, and the early diverging groups are almost exclusively marine^15^. We refer to these four groups (Fungi, Teretosporea, Pseudofungi and Labyrinthulea), which represent a broad diversity of osmotrophic forms, hereafter as ‘osmotrophic groups’.

Historically, the shared phenotypic traits among the four osmotrophic groups have led to frequent taxonomic misidentifications and the misclassification of various species (for example, refs. ^11,20,21,24,25^). These phenotypic similarities include a range of functionally connected traits that allowed species from these groups to specialize into an osmotrophic lifestyle: (1) robust cell wall structures encapsulating the cell and enabling maintenance of high intracellular turgor^7^, (2) heterotrophy via specialized absorptive nutrition, with adapted secretomes for external digestion of large compound chains^7,26–28^, (3) hyphae and hyphae-like structures, which often coevolve with specialized osmotrophic lifestyles. They are particularly common in Fungi^29^ and in some Pseudofungi^15^, and can also be observed in some Teretosporea^19,21^. Some osmotrophs form simpler filamentous structures (for example, anucleated rhizoids in chytrid fungi^30,31^), and some Labyrinthulea evolved ectoplasmic nets for nutritional intake^32^. Hyphae and hyphae-like structures provide functional advantages under spatially heterogeneous nutrient conditions, improving nutrient absorption and enzyme secretion^33^. In addition, (4) multinucleated coenocytic/syncytial development is often a characteristic of these groups^19,31^. While multinucleated development is neither specific nor strictly necessary for osmotrophy, it allows some organisms with an expanding and networked cytoplasm (for example, hyphal growth) to maintain a closer relationship between cell volume and nuclear density^34^.

Osmotrophs do not feed by internalizing particles such as prey cells. Instead, they are specialized in absorbing liberated compounds directly from the environment for metabolism^7^. However, specializing in osmotrophy extends beyond simple absorptive feeding; it involves complex adaptations driven by specific ecological pressures^7^. Because osmotrophic organisms do not have private access to phagocytosed food, they must compete for public goods in the environment^8^. In such competitive settings, osmotrophs are expected to require a diversified metabolic network, which maximizes the utilization of available but variant metabolites. Previous works reported that horizontal gene transfer (HGT) from fungi facilitated the specialization of some pseudofungi towards a plant parasitic osmotrophic lifestyle^8,35^. A later study reported a complex pattern of HGT in the evolution of the nitrate assimilation pathway involving all four distinct osmotrophic groups^36^. To clarify the role of HGT and the evolutionary history of these groups, we used comparative genomics, phylogenomics and molecular clock methods to reconstruct and date the trajectory of gene content evolution which accompanied these four parallel transitions towards osmotrophy within the eukaryotes.

## Results

### Origin and age of the osmotrophic groups

**Fig. 1:**
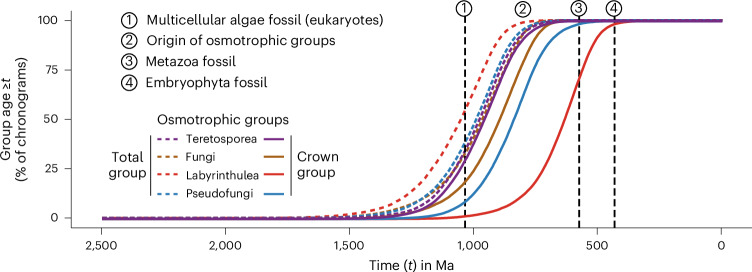
Timing the origin of Teretosporea, Fungi, Labyrinthulea and Pseudofungi, four eukaryotic groups that experienced parallel transitions towards osmotrophic specialization. The curves represent the proportion of post-burn-in sampled chronograms from relaxed molecular clock analyses (*n* = 32,379) in which the estimated age of a group is greater than or equal to the time indicated on the *x* axis. The dashed black lines show the ages of old bona fide fossils of multicellular eukaryotic algae (*Bangiomorpha pubescens*, ~1,030 Ma), animals (*Charnia masoni*, ~575 Ma) and vascular plants (*Cooksonia barrandei*, ~430 Ma), as described in the key.

Little is known about the origins of osmotrophic specialization in eukaryotes. Aiming to obtain a temporal framework for this study, we performed relaxed molecular clock analyses to date the origin of the four osmotrophic groups on a species phylogeny (see ‘Species tree reconstruction and molecular clock analyses’ section in Methods). A major shift towards osmotrophic specializations must have occurred between the last common ancestor of each osmotrophic group (crown group) and the onset of the stem branch leading to each osmotrophic group (total group). The age curves found for the four osmotrophic groups show substantial overlap, both for total group and for crown-group ages (Fig. 1). For total group ages, the curves reach half of the fraction of sampled chronograms at 1,052, 990, 977 and 966 million years ago (Ma) for Labyrinthulea, Pseudofungi, Teretosporea and Fungi, respectively, and for crown-group ages, 957, 896 and 839 Ma for Teretosporea, Fungi and Pseudofungi, respectively, and 634 Ma for Labyrinthulea. While Labyrinthulea appear younger in crown-group age than the other osmotrophic groups, this could be owing to the limited genome sequence data for this group (our study incorporates sequence data for four Labyrinthulea species, and the gap between total and crown-group ages for this group is compatible with the possibility of unsampled/unsequenced lineages). Leaving aside this uncertainty, the substantial overlap in total group ages for the four osmotrophic groups and the crown-group ages for three osmotrophic groups suggests that osmotrophic specialization may have evolved convergently in the eukaryotes during a similar geological period, most likely near the beginning of the last billion years, predating the emergence of crown-group metazoans and the embryophytes (Fig. 1; we elaborate on this result in Discussion).

### Osmoclusters—genomic convergence between the osmotrophic groups

**Fig. 2:**
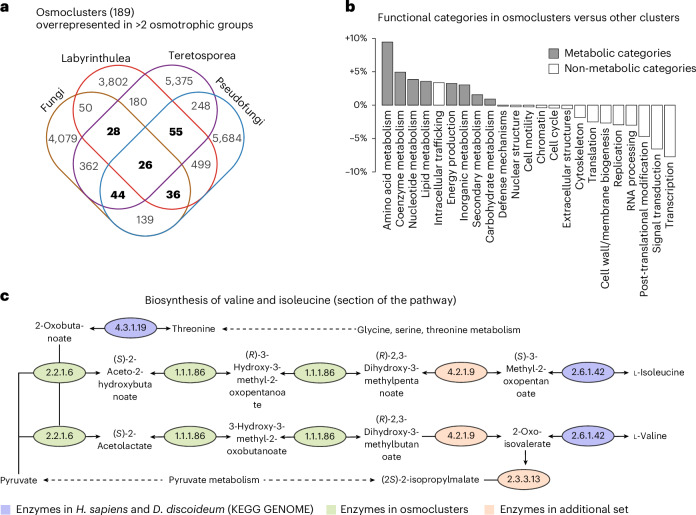
Functional information on the 189 osmoclusters. **a**, The number of significantly overrepresented gene families clusters in osmotrophic groups. The numbers in bold, which sum to 189, correspond to clusters overrepresented in more than two osmotrophic groups (osmoclusters; see Methods for further details). **b**, A differential representation of COG categories with functional information in osmoclusters (*n* = 189) versus other clusters (*n* = 256,642). The positive values represent functional categories that are overrepresented in the osmoclusters. For example, 14.6% of functional category annotations for osmoclusters are related to ‘amino acid metabolism’, while only 5.1% are related to ‘amino acid metabolism’ in the other clusters, a positive net difference of 9.5%. **c**, Example of the extended coverage of the metabolic network provided by the osmoclusters compared with two heterotrophs that do not specialize in osmotrophy. See Supplementary Information 1 for additional full pathway representations for *Homo sapiens*, and Supplementary Information 2 for the amoeba *Dictyostelium discoideum*. The images shown in Supplementary Information 1 and 2 were generated using the ‘KEGG Mapper – Reconstruct’ tool. We thank Kanehisa Laboratories for permission to publish results from KEGG and KEGG images^82^. Enzymes in the additional set refer to enzymatic annotations retrieved in the additional set of 94 clusters, which were also found to be overrepresented in the osmotrophic groups when the photosynthetic organisms are excluded from the background set of species.

Next, we investigated whether the phenotypic convergence of these four eukaryotic lineages towards osmotrophy was accompanied by convergence in gene content. We grouped extant protein sequences into clusters, used as proxies for gene families. Then we performed statistical tests to identify clusters overrepresented in copy number in the osmotrophic groups compared with the rest of species (Methods). Given that the four osmotrophic groups are distributed in two distinct eukaryotic divisions^37^ (Amorphea and Diaphoretickes, two groups in each), clusters overrepresented in three or more osmotrophic groups probably represent convergent adaptations putatively related to osmotrophic function. This process identified 189 clusters enriched in three or more of the osmotrophic groups. We therefore use the term ‘osmoclusters’ for these 189 clusters (Fig. 2a).

Interestingly, the osmoclusters include the MIP aquaporin family and a wide array of transporters for various compounds, including a sugar transporter, secondary active sulphate transmembrane transporter and nucleoside transmembrane transporter (Supplementary Table 2). For the control of ion balance (a requirement for high cellular turgor linked to osmotrophy), the osmoclusters include genes such as the ZIP zinc transporter, Na^+^/H^+^ antiporter, Na^+^/Pi cotransporter, divalent cation transporter, Mg^2+^ ion transporter and the ion channel regulatory protein UNC-93. Specialized regulatory pathways such as the cellular response to phosphate starvation and the negative regulation of TOR signalling, which controls cell growth in response to nutrient availability^38^, are also present among the osmoclusters. This functional repertoire demonstrates an expanded genetic toolkit for osmotic regulation and for nutrient acquisition, which is likely to be necessary for utilization of extracellular metabolites and the prevention of the loss of valuable resources to competitors^7^.

The osmoclusters also include components for cell surface activities and enzymes for extracellular digestion, including serine-type endopeptidase and peptidase family C69, as well as other depolymerizing enzymes such as glycoside-hydrolase family GH114, cellulase and pectate lyase. Gene families involved in cell wall functions are also represented, including the chitin-binding domain type 3 genes and components for the attachment of GPI anchors to proteins for presentation on the cell surface. Finally, functional annotations of osmoclusters also include polarized growth, including proteins supporting intracellular transport and movement such as dynamitin.

**Extended Data Fig. 1:**
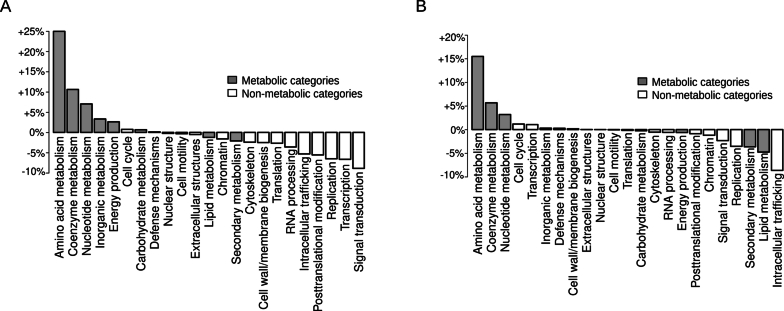
Differential representation of COG categories with functional information between pairs of clusters sets. (**a**) Test set vs background clusters. Test set here corresponds to the additional set of 94 clusters that satisfy the conditions for being defined as osmoclusters when photosynthetic species are excluded from the background set of species in the overrepresentation tests (see Results section ‘Osmoclusters: genomic convergence between the osmotrophic group’). (**b**) Same Test set as in panel A vs osmoclusters.

Beyond the diversity of functions described, the main functional categories that are proportionally overrepresented in osmoclusters compared with non-osmoclusters are largely biased towards metabolism (Fig. 2b). Consistently, genes annotated as enzymes also show an increased representation compared with non-osmoclusters (28% versus 15%; Methods). Metabolism being a core component of osmoclusters is unsurprising. Osmotrophs do not have private access to phagocytized food particles such as prey cells, which are rich sources of organic matter and complex metabolites. In limiting nutrient conditions, the ability to assimilate diverse substrates including simpler metabolites can provide an advantage over others^7^. As such, we would expect osmotrophs to possess an expanded anabolic metabolic network. Beyond carbon source acquisition, shared similarities may exist on a metabolic level between the osmotrophic groups and the photosynthetic groups. A documented example is the pathway to assimilate nitrate, an inorganic nitrogen source, which in eukaryotes is only present in osmotrophic and photosynthetic groups, and which has spread across eukaryotes through HGT^36,39,40^. This case is not unique, as an additional set of 94 clusters is retrieved as osmoclusters (Supplementary Table 10) if the photosynthetic groups are excluded from the background set of species in the statistical tests. This second set of clusters is also functionally biased towards metabolic network function (Extended Data Fig. 1a), with ‘amino acid metabolism’ again highly represented (Extended Data Fig. 1b).

When exploring osmoclusters, including this additional set of clusters (Supplementary Tables 1 and 11), amino acid metabolism and metabolism of vitamins and cofactors are the most highly represented metabolic processes. Regarding amino acid metabolism, we identified 4/4 enzymatic steps connecting pyruvate/2-oxobutanoate to the precursors of ʟ-valine and l-isoleucine (see p. 1 in the Supplementary Information 1, which corresponds to p. 3 in the unified Supplementary Information file), 8/10 steps connecting phosphoribosyl pyrophosphate to ʟ-histidine (p. 2), 4/4 steps connecting 2-oxobutanoate to ʟ-homocysteine (p. 3), 2/3 steps connecting l-aspartate to l-homoserine (p. 4) and 6/6 steps connecting prephenate and ʟ-tryptophan (p. 5). In agreement with the importance of amino acid biosynthesis among osmotrophic groups, inorganic nitrogen assimilation is also represented (for example, row 58 in Supplementary Table 1). Interestingly, this also demonstrates that transformation between amino acids and amino acid precursors is important, indicating that osmotrophs are enriched with metabolic flexibility, enabling them to navigate differing metabolite availability. A similar pattern is present in the vitamin biosynthesis metabolic network; osmoclusters include 5/6 steps connecting guanosine triphosphate to riboflavin (vitamin B2, p. 6), 3/3 steps connecting 8-amino-7-oxonanoate to biotin (vitamin B7, p. 7) and 3/3 steps connecting 7,8-dihydroneopterin to 7,8-dihydropteroate, a precursor of folate (vitamin B9, p. 8). Most of the sections described connect regions of the metabolic network that are absent in heterotrophs that do not specialize in osmotrophy (Fig. 2c), such as *Homo sapiens* (Supplementary Information 1) or the amoeba *Dictyostelium discoideum* (Supplementary Information 2).

### Origin and expansion of the osmoclusters

**Fig. 3:**
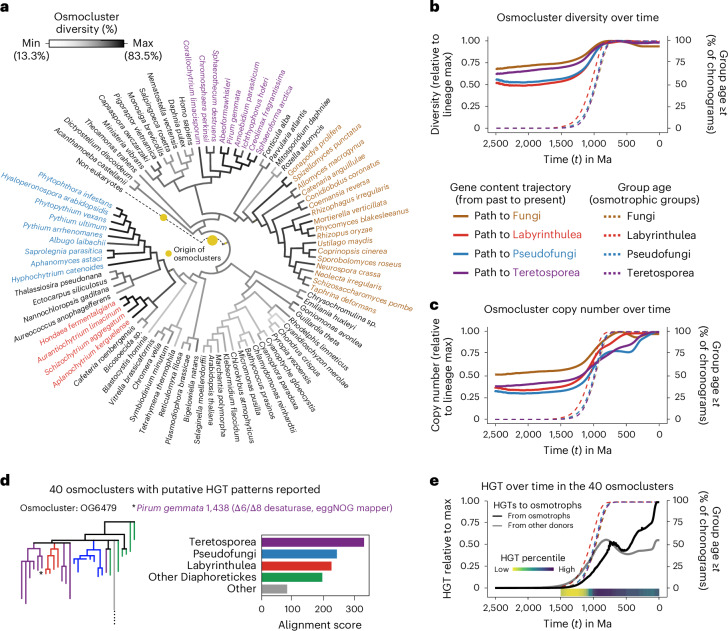
Evolutionary history of the osmoclusters. **a**, The osmocluster content across eukaryotes, measured as the fraction of osmoclusters found in each lineage (branches). Four colours other than black have been chosen to indicate the clades corresponding to the four osmotrophic groups (see the legend). The terminal branches are extant species (*n* = 86), whereas internal branches (*n* = 85) correspond to inferred ancestral gene contents (Methods). The size of the yellow circles is proportional to the fraction of osmoclusters that originated in each branch/ancestor. Osmoclusters mostly originated in ‘non-eukaryotes’ and in the root of the species phylogeny (Supplementary Table 3). As such, only the diameter of the circles from both the deepest branches are sufficiently large to be visible. See Extended Data Fig. 3 for a version based on an extended dataset of 121 species under three alternative roots of the eukaryotic phylogeny, and see Extended Data Fig. 4 for a version including taxonomic information for each species. **b**,**c**, The osmocluster content (**b**) and osmocluster copy number (**c**) over time based on the inferred gene contents from the lineage paths leading to each osmotrophic group. The raw values were normalized to the maximum value observed for each lineage. **d**, An example of an HGT candidate, among a total of 40 osmoclusters in which we identified patterns of HGT (full list and case by case inspection in Supplementary Information 3). In this example, a sequence from Teretosporea (Amorphea) shows a twofold higher alignment score against Diaphoretickes (Pseudofungi, Labyrinthulea or other Diaphoretickes) than against any other sequence, including the Amorphea division of eukaryotes (‘Other’). **e**, The inferred distribution of HGT acquisitions for the 40 osmoclusters over time in the lineages leading to the extant representatives of the four osmotrophic groups. The bottom colour bar indicates the HGT percentile of the 40 osmoclusters compared with a background distribution of equivalent gene family sets at each timepoint (Methods). High percentiles represent time units where the 40 osmoclusters have a stronger HGT signal than other clusters.

**Extended Data Fig. 2:**
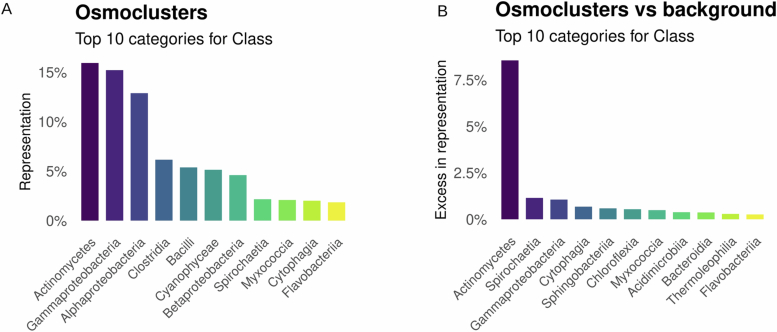
Non-eukaryotic representation in the osmoclusters. Extended Data Fig. 2. **a**) Top 10 most represented ‘Class’ categories on a taxonomic level among the non-eukaryotic species represented in osmoclusters. For each osmocluster, the representation of each ‘Class’ was calculated by dividing the number of species belonging to that ‘Class’ by the total number of non-eukaryotic species represented in the cluster with valid taxonomic assignments. These relative proportions were then averaged across all osmoclusters. Only clusters in which at least one non-eukaryotic sequence was present were considered. Taxonomic information was obtained from NCBI Taxonomy files. **b**) We computed the same measurements as for A, but in 100 iterations of random samplings of clusters among the list of non-osmoclusters with representations of non-eukaryotic sequences, and we subtracted the relative represenation found in the osmoclusters vs that found on average in the randomly sampled sets of clusters.

By employing phylogenetic reconciliation methods^41^, we reconstructed the evolutionary history of the osmoclusters. First, we found that most osmoclusters have a deep origin in the tree of life (see yellow circles in Fig. 3a), which is consistent with the presence of non-eukaryotic sequences within these clusters. Specifically, we found Actinomycetes (15.9%), Gammaproteobacteria (15.2%) and Alphaproteobacteria (12.8%) as the three predominant taxonomic groups among the non-eukaryotic sequences represented in the osmoclusters (Extended Data Fig. 2a). However, only Actinomycetes are represented more than expected (+8.5%) when we compare these values with the proportions obtained from random samples of gene family clusters (Extended Data Fig. 2b). Because Actinomycetes include species that are also specialized in osmotrophic growth^42^, this suggests that osmoclusters are probably important for osmotrophic functions beyond the eukaryotes.

**Extended Data Fig. 3:**
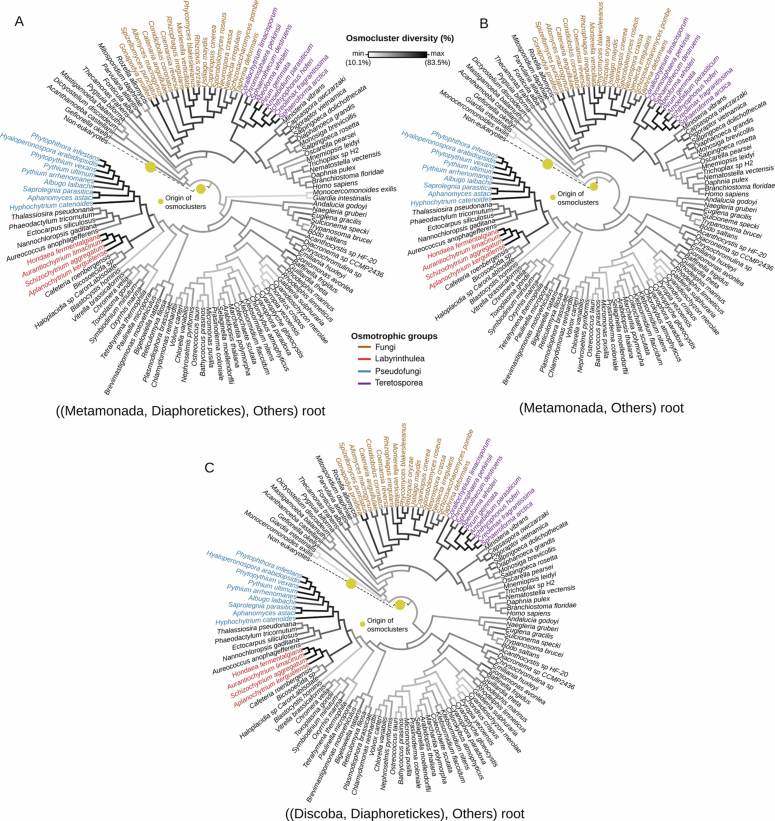
Re-analyses of Fig. 3a under an extended taxon sampling of 122 species. (**a-c**) Each of the three panels represent a re-analysis done under one plausible root for the eukaryotic phylogeny. As in Fig. 3a, in each of the three panels, osmocluster content across eukaryotes was measured as the fraction of osmoclusters found in each lineage (branches). Terminal branches are extant species, whereas internal branches correspond to inferred ancestral gene contents (see Methods). The size of the yellow circles is proportional to the fraction of osmoclusters that originated in each branch/ancestor.

**Extended Data Fig. 4:**
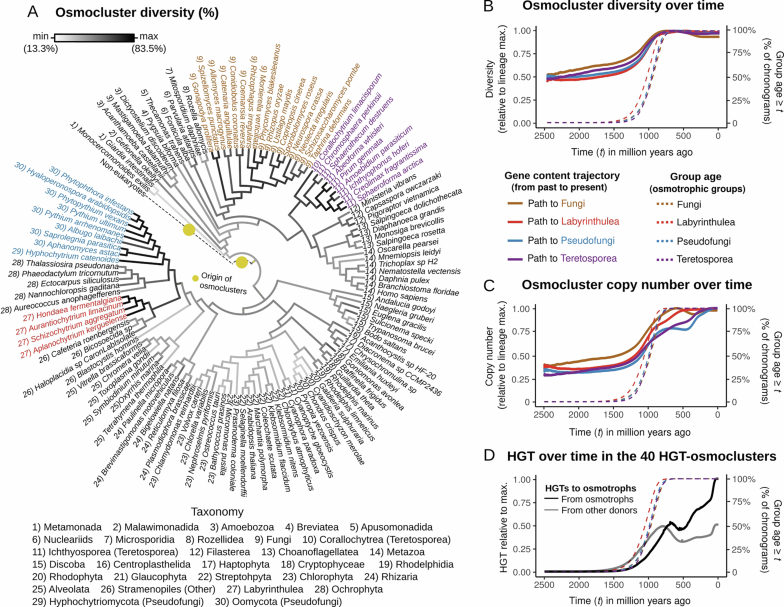
Evolutionary history of the osmoclusters: Re-analyses of Fig. 3, confirming the results retrieved with the original taxon sampling of 86 species, now with an extended taxon sampling of 122 species. (**a**) Osmocluster content across eukaryotes, measured as the fraction of osmoclusters found in each lineage (branches). Among the three tested roots under the extended dataset (Extended Data Fig. 3), here we employed the ‘((Discoba, Diaphoretickes), Others) root’ and represent the retrieved trends into the original species tree, so that data results can be directly compared to Fig. 3a. We chose this particular root as it is the most concordant with the results reported by Williamson et al. 2025 (https://doi.org/10.1038/s41586-025-08709-5). Terminal branches are extant species, whereas internal branches correspond to inferred ancestral gene contents (see Methods). The size of the yellow circles is proportional to the fraction of osmoclusters that originated in each branch/ancestor. Osmoclusters mostly originated in “Non-eukaryotes” and in the root of the species phylogeny. As such, only the diameter of the circles from both branches is sufficiently large to be visible. (**b**) Osmocluster content and (**c**) osmocluster copy number over time based on the inferred gene contents from the lineage paths leading to each osmotrophic group. (**d**) For the 40 osmoclusters in which we identified signatures of potential HGT events, inferred distribution of HGT acquisitions over time in the lineages leading to the extant representatives of the four osmotrophic groups.

Despite osmoclusters being ancient gene families (Fig. 3a), our ancestral reconstruction shows that the genomic repertoire of osmoclusters fluctuated both in the diversity (fraction of osmoclusters represented in the genome; Fig. 3a,b) and abundance (mean genomic copy number of osmoclusters; Fig. 3c) in the evolutionary path leading to the osmotrophic groups. These results are robust to alternative roots of the eukaryotic phylogeny and to changes in the taxon sampling (Extended Data Figs. 3 and 4). In particular, osmocluster diversity increased and reached its peak in parallel to the origin of each osmotrophic group (Fig. 3b). Concordantly, osmocluster abundance also increased with the emergence of the osmotrophic groups, and it continued increasing during the diversification of these groups (Fig. 3c). These data are consistent with the hypothesis that the osmoclusters encompass gene families that have been important for the evolution of the osmotrophic lifestyle in these groups.

### Horizontal gene transfers in osmoclusters

Gene duplications and gene losses are major mechanisms driving gene content changes in eukaryotes^43,44^. However, the old origin of the osmoclusters (Fig. 3a), combined with the reported increase in the genomic diversity of osmoclosters in the osmotrophic groups (Fig. 3b), suggests a potential role for HGT in the evolutionary history of these gene families. We therefore explored the role of HGT in the osmoclusters and in the osmotrophic groups. Our first approach consisted of a manual inspection of the gene tree phylogeny of each osmocluster, together with additional data visualization, including sequence similarity analyses (Supplementary Information 3). On the basis of this combined data, we identified 40 (~20%) osmoclusters showing phylogenetic patterns suggestive of HGT events involving distantly related taxa (see example in Fig. 3d). In a previous study, we reported phylogenetic evidence for at least two HGTs of the nitrate assimilation pathway involving the four osmotrophic groups^36^. As a validation of our methodology, this previously reported case of HGT is represented among the 40 HGT osmoclusters identified (osmocluster OG5471; Supplementary Information 3).

Signatures of non-vertical evolution can be robust even when phylogenetic resolution is incomplete. For instance, Fig. 3d supports HGT to Teretosporea from Diaphoretickes, despite uncertainty regarding the donor group. Phylogenetic reconciliation tools address this quantitatively by accounting for uncertainty in both the gene tree and the history of duplication, transfer, loss and speciation events^41^. Applying this methodology to osmocluster OG6479 (Fig. 3d), we retrieved an average of 1.76 HGTs from Diaphoretickes to Teretosporea across all sampled reconciliations. Collectively, these results support the hypothesized HGT to Teretosporea for this osmocluster, despite ambiguity regarding the precise lineages involved (60 possible HGT donor–receptor lineage pairs involving Diaphoretickes and Teretosporea were retrieved for OG6479; see raw reconciliation results in Supplementary Data).

Expanding this approach, we aggregated and dated the HGT signals for the 40 osmoclusters where candidate HGT topologies were previously identified (Fig. 3e). We identified two main patterns: first, the earliest HGTs are derived from non-osmotrophic donor groups (that is, HGTs from other eukaryotes or from non-eukaryotic donors). This, together with the above observation that the diversity of the osmoclusters increased in parallel to the origin of the osmotrophic groups, is consistent with the hypothesis that HGT may have allowed early representatives of these groups to expand their repertoire of osmotrophy-related functions. Second, our results show an increase in HGT as the osmotrophic groups diversified, with most mid- and late HGTs corresponding to HGTs between osmotrophs (Fig. 3e). Aiming to get insights into the magnitude of HGT between osmotrophic groups, we next inspected HGT across all gene families present in the genomes of the osmotrophic groups. We note that the previously analysed osmoclusters would not fully sample such patterns.

### Horizontal gene transfers beyond the osmoclusters

**Fig. 4:**
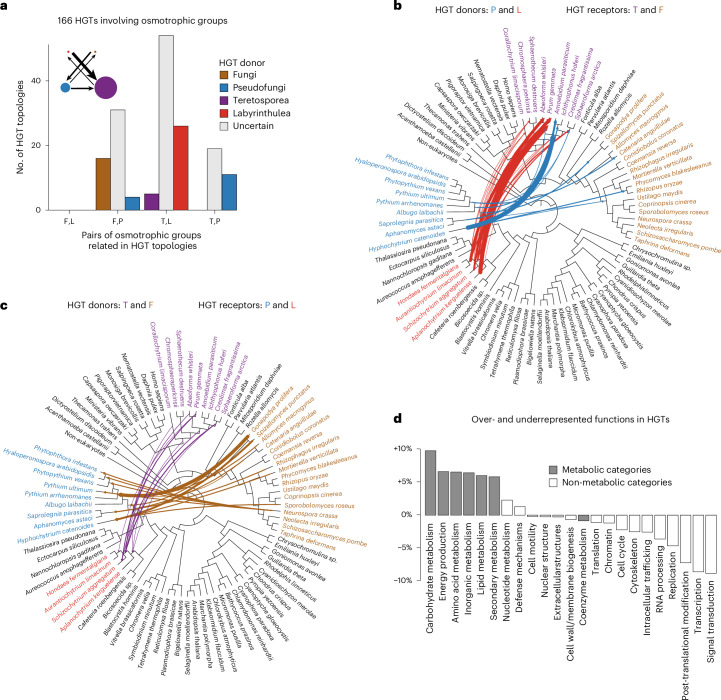
Exploration of HGT in all gene families (*n* = 256,831) present in the genomes of the osmotrophic groups. **a**, The sum of validated HGTs consisting of gene tree clades that include sequences from two distantly related osmotrophic groups. **b**,**c**, The phylogenetic distribution of the inferred HGTs with donor and receptor information (Supplementary Table 5). **b** shows HGTs from Pseudofungi and Labyrinthulea to Teretosporea and Fungi, and vice versa for **c**. **d**, A differential representation of COG categories with functional information in the 163 clusters containing the 166 HGT topologies versus other clusters. Positive values represent functional categories that demonstrate increased representation in the HGT clusters. For example, 17.06% of functional category annotations for the HGT clusters are classified as ‘carbohydrate metabolism’, while only 7.32% are present in non-HGT clusters, a net difference of 9.74%. F, Fungi; L, Labyrinthulea; P, Pseudofungi; T, Teretosporea.

We performed a large-scale HGT screen that allowed us to provide supervised quantitative assessments of HGTs involving the four osmotrophic groups. This consisted of reconstructing a phylogeny for every gene family cluster that contained osmotrophic group taxa. We then collapsed every poorly supported clade in the gene trees and identified tree topologies showing phylogenetic associations between sequences from distantly related osmotrophic groups (Supplementary Information 4). We visually inspected them case by case and validated each of them with sequence similarity analyses. This process identified 166 gene tree topologies derived from 163 gene families, which we refer to as HGT topologies (Fig. 4). Aiming to minimize false positives by not considering cases that could be easily confounded with differential gene loss patterns^43^, we only considered HGT topologies involving pairs of phylogenetically distant osmotrophic groups belonging to distinct eukaryotic divisions (four pairs in total; Fig. 4a). Through careful visual inspections of the phylogenies, we could map HGT donor and receptor information for 62 HGT topologies. On the basis of this, we identified two clear HGT paths between osmotrophic groups (Fig. 4a). First, Fungi have been a major HGT donor to Pseudofungi (16 Fungi-to-Pseudofungi HGTs). This is consistent with previous findings that identified HGTs in this direction of genes that function in osmotrophic plant parasitic lifestyles^35,45^ (for example, clusters OG858 and OG3350 correspond to ‘pectinesterase’ and ‘galacturan 1,4-alpha-galacturonidase’, both involved in pectin degradation from plant cell walls; Supplementary Table 4). Second, we identified, to our knowledge, a previously unreported HGT pattern from Labyrinthulea to Teretosporea (26 cases).

The identified HGTs are spread across the species phylogeny (Fig. 4b,c), suggesting that HGT has been an ongoing process in the evolution of these osmotrophic groups. Species coexistence in shared environments could have been a factor underpinning the two main HGT paths detected: Labyrinthulea-to-Teretosporea (most sequenced species are from aquatic environments^19,32^), and Fungi-to-Pseudofungi (most sequenced species are from terrestrial environments^9,15^). Regarding the 26 Labyrinthulea-to-Teretosporea HGTs, the *Abeoforma whisleri* + *Pirum gemmata* clade from Teretosporea received 21 HGTs (7 to the *Pirum* branch, 4 to the *Abeoforma* branch and 10 to the ancestor of this clade—labelled with the ‘I93’ notation in Supplementary Table 5). Regarding the 16 Fungi-to-Pseudofungi HGTs, 4 were transferred from Pezizomycotina (represented by *Neurospora crassa*), and 6.5 from Chytridiomycota (represented by *Spizellomyces punctatus* and *Gonapodya prolifera*). To a lesser extent, Pseudofungi and Teretosporea also transferred genes. Overall, 8.5 out of the 11 HGTs from Pseudofungi to distinct nodes of Teretosporea arise from the *Hyphochytrium catenoides* branch, and the 5 HGTs from Terestosporea were all transferred to Labyrinthulea taxa (fractional values result from HGT cases where possible donor–receptor groups are equally plausible; Supplementary Table 5). Notably, neither Fungi-to-Labyrinthulea nor Labyrinthulea-to-Fungi HGTs were identified (Fig. 4b,c), possibly because the sequenced species from both groups are mainly from terrestrial (Fungi) and from marine (Labyrinthulea) environments, meaning our genome sampling of these two groups was restricted to species with non-overlapping ecologies.

Metabolism is overrepresented in the 163 gene families with HGT topologies compared with all other gene families sampled (Fig. 4d). Some transferred genes are tightly connected in the metabolism. For example, (1) the nitrate transporter and the nitrite reductase (OG935 and OG5471 in Supplementary Table 4), transferred between Teretosporea and Pseudofungi, (2) two enzymes involved in pectin degradation, transferred between Fungi and Pseudofungi (mentioned above), and (3) three enzymes from the postsqualene cholesterol biosynthesis pathway (cholestenol delta-isomerase, sterol-4α-carboxylate 3-dehydrogenase and Δ24-sterol reductase; OG17590, OG19444 and OG3191, respectively), transferred between Labyrinthulea and Teretosporea. Altogether, the 163 gene families cover 68 different pathways (Supplementary Table 4), suggesting a general contribution to metabolism. Among the 163 gene families with HGT cases, 110 are overrepresented in copy number in at least one osmotrophic group, and 48 in at least two distantly related osmotrophic groups (Supplementary Table 4). The HGT cases found are thus representing gene families that are probably playing important functions in osmotrophic groups.

## Discussion

To study the genetic footprints that underpinned specialization to an osmotrophic lifestyle, we compared four eukaryotic groups, Fungi, Labyrinthulea, Teretosporea and Pseudofungi, which include a broad range of species that evolved an array of osmotrophic adaptations^7,11–17^. Comparative genomics identified genetic convergence in 189 gene families (here referred to as osmoclusters). These 189 gene families demonstrated a clear functional trend, involved in osmotrophy-related functions, including nutrient uptake, ion regulation and anabolic metabolism.

To investigate the origins of osmotrophic specialization in eukaryotes, we dated the evolution of the four osmotrophic groups, revealing that these groups emerged within a similar timeframe (Fig. 1), most likely during the Tonian period (1,000–720 Ma). It is challenging to put the retrieved age estimates into ecological context given uncertainties in the geochemical record for this period. Specifically, it is still unclear how limiting the oxygen levels were during this period or how this relates to the diversification of the ‘crown-group’ eukaryotes^46^. Furthermore, there is a limited availability of fossils that can be confidently mapped to crown-group eukaryotes, particularly from before the Neoproterozoic period^47^, which limits the resolution of relaxed molecular clock analyses^48^. Acknowledging these limitations, our results suggest that the emergence of the osmotrophic groups may have either coincided with, or slightly preceded, the time when eukaryotes would have started to become ecologically dominant, according to lipid fossil markers^49^, possibly linked to a rise in oxygen levels, a change that may have played a role in the diversification of crown-group eukaryotes^50^. An additional condition that may have been important was concurrent changes in marine chemistry, which have been proposed to be linked with the rise of eukaryotic algae during the Tonian period^51,52^.

Although many extant osmotrophs have been described as symbionts of animals or plants^19,32^, an origin of the osmotrophic groups related to these two multicellular groups can be discarded as all four osmotrophic groups are older than established fossils of animals^53^ (~575 Ma) and vascular plants^54^ (~430 Ma) (Fig. 1), and are older than recently published estimates for the age of the Metazoa^55^ (613–593 Ma) and the Embryophyta clades^56^ (515–493 Ma). Notwithstanding, it is likely that the ecosystems in which the osmotrophic groups emerged already possessed multicellular eukaryotic forms (for example, *Bangiomorpha pubescens*, a multicellular rhodophyte fossil of ~1 billion years ago (Ga)^57^). In this regard, phylogenies of the osmotrophic groups provide support that the deepest nodes within these clades most likely represent marine or freshwater lineages^11,15,19,32,58^. Altogether, we hypothesize that these groups most likely emerged in aquatic environments enriched in organic matter and composed of communities that, despite still being dominated by prokaryotic forms^59^ (perhaps cyanobacterial mats), included an increasing representation of eukaryotes^49^, some of which probably presented multicellular forms.

Given the broad representation of osmotrophic forms represented in the four groups sampled, important changes towards osmotrophy probably occurred in parallel to the origin of these groups. The expansion of osmoclusters identified at the onset of these groups supports this hypothesis. Yet, osmocluster copy numbers continued to expand throughout the diversification of the four groups. One possible explanation is that osmotrophic specialization was a gradual process that continued during the diversification of these lineages. In the particular case of Teretosporea (Ichthyosporea + Corallochytrea), the early branching species *Syssomonas multiformis*^60^ was characterized as a flagellated predator, in contrast to the rest of Tereosporea. Unfortunately, sequence data from *S. multiformis* could not be incorporated into our study owing to a high level of sequence contamination^61^. Phagotrophy in *S. multiformis* could indicate that the last common ancestor of Teretosporea was only partly specialized in osmotrophy, with full transitions occurring later and separately within this group: in Ichthyosporea^19^ and in Corallochytrea (more specifically in *Corallochytrium*^11,13^, the sister lineage to *S. multiformis*^60^). While some studies have recovered alternative topologies in which Collarochytrea and Ichthyosporea are not monophyletic^60,62^, the species tree from this work and others^63,64^ provide support for the Teretosporea hypothesis^13^.

Previous research has reported several examples of HGT into the eukaryotes^35,65–68^. While most studies have focused on HGT from bacteria to eukaryotes (for example, refs. ^69,70^), phylogenetic evidence of HGT between eukaryotes has also been published (for example, refs. ^36,71^). Fungi is probably one of the main eukaryotic groups for which studies of HGT have been published^70,72^. On the basis of previous reports of HGT involving Fungi and Pseudofungi^73^, HGT was proposed as a potential source of genes present in osmotrophs^8,74^. Our screen for HGT in the osmoclusters consisted of an inspection of gene tree topologies together with sequence similarity analyses, a process which identified 40 osmoclusters presenting topologies that are compatible with HGT events. Phylogenetic reconciliations were then employed to identify predominant trends of HGT in these osmoclusters, showing an increase of HGT between eukaryotic osmotrophs concurrent with the diversification of these groups (Fig. 3e). Phylogenetic reconciliation analyses also showed that these 40 osmoclusters rank in the upper percentiles for inferred HGTs compared with other clusters, particularly in the period in which the osmotrophic groups originated (Fig. 3e). Then, to explore the occurrence of HGT also beyond the osmoclusters, we screened for HGT topologies in the whole set of gene family clusters. This analysis also encompassed a rigorous inspection of the candidate HGT topologies, validating each case with independent sequence similarity analyses to test that phylogenetic associations were not gene tree artifacts (Supplementary Information 4; Methods). This HGT screening identified 166 HGT topologies involving phylogenetically unrelated osmotrophic groups—a number that is not only likely to be an underestimation of HGTs due to methodological constraints but also because this analysis focused exclusively on HGTs only involving distantly related osmotrophic groups. This finding, combined with the observed functional trends found in the HGTs (that is, overrepresentation of metabolism, as expected^75^; Fig. 4d), as well as the observed phylogenetic trends (that is, distinct HGT highways involving Labyrinthulea to Teretosporea and Fungi to Pseudofungi, a pattern unlikely to result from methodological errors; Fig. 4a–c) together indicates that our approach captured true evolutionary signals.

In conclusion, we establish that the independent transitions towards specialized osmotrophy by four distinct eukaryotic groups most likely coincided or slightly preceded the Tonian period (1,000–720 Ma). We identify that this case of phenotypic convergence was accompanied by convergence at the gene content level in a set of gene families involved in osmotrophic functions, which we referred to as osmoclusters. In eukaryotes, gene duplications and losses represent the main sources of gene content changes. Despite HGT not being a main evolutionary driver as it is in prokaryotes, HGT acted in concert with standard vertical evolutionary processes to facilitate the spread of genes among the osmotrophic groups. Taken together, our work provides a detailed genomic perspective on a prominent convergent trophic transition in eukaryotes, revealing signatures of gene transfer in the parallel evolution of osmotrophic specialization in eukaryotes.

## Methods

### Protein sequences datasets

We prepared a species protein sequence dataset specifically for this study. As described in the main text, the four transitions towards osmotrophy occurred in the eukaryotic groups Opisthokonta ((1) Fungi and (2) Teretsoporea) and Stramenopiles ((3) Pseudofungi and (4) Labyrinthulea). Opisthokonta and Stramenopiles belong, respectively, to the major eukaryotic divisions Amorphea and Diaphoretickes. Taking this into consideration, the dataset employed in this study includes 1,363,672 protein sequences from 86 eukaryotic species within both divisions of eukaryotes. The use of proteome datasets derived from genome projects was prioritized over proteome predictions derived from only transcriptome data. Proteomes from transcriptomic projects were only used for groups with very few genomic data available (for example, Glaucophyta), to expand the representation of certain osmotrophic groups (for example, Teretosporea), or to sample clades that branch close to the osmotrophic groups in the eukaryotic phylogeny (for example, Opalazoa). Owing to computational constraints, we prioritized proteome data from a broad and balanced taxonomic representation within the eukaryotic divisions Amorphea and Diaphoretickes. Both eukaryotic divisions include most of the known eukaryotic diversity, and the availability of genomic data from the groups that branch outside both divisions is poor, and in many cases, the phylogenetic position of these taxa is poorly resolved. The purpose of this study was to explore gene content changes that occurred near the emergence of the osmotrophic groups and during their diversification. Since the osmotrophic groups branch late within the Amorphea and Diaphoreticles divisions, we expected that the absence of species representation outside both divisions would not be problematic for our analyses. Still, we tested this hypothesis, in addition to the impact of extending the taxonomic representation of some groups, by re-running some of our main analyses under an extended dataset (see the Methods section ‘Re-analyses with an extended dataset’ below).

### Gene family clusters

We aligned the dataset proteins in an all-against-all fashion with BLASTP^76^ version 2.10.1+, using an E-value of 1 × 10^−3^, enabling the soft_masking option and keeping the 500 best-scoring targets per each query. BLASTP alignments were then used as input for the MCL^77^ clustering software version 14-137, which groups genes into clusters on the basis of a sequence similarity network constructed from the all-against-all BLASTP alignments. MCL was run with an inflation value of -I 2, using the −log_10_-transformed BLASTP E-values as a similarity metric. MCL output clusters with fewer than four members were discarded for downstream analyses. Clusters with >750 members were also discarded as they are unlikely to produce reliable alignments for phylogenetic inferences. The cluster definition file and the sequences included in the clusters are available in Supplementary Data.

### Osmoclusters

We identified gene family clusters that are potentially important for osmotrophic specialization on the basis of the logic that these should be overrepresented in copy number in the osmotrophic groups compared with other species. For every gene family cluster tested (we excluded clusters found in only one species), we performed four statistical tests (Mann–Whitney *U* test, using the mannwhitneyu function from SciPy^78^ 1.10.1), one per osmotrophic group, to evaluate whether the copy number distribution of the given osmotrophic group (for example, Fungi) in the cluster is significantly greater than that of the background set of species (the background species were all the eukaryotes sampled in our study that do not belong to any of the four osmotrophic groups). For each osmotrophic group, the overrepresentation tests were performed on those clusters that are represented in at least one species from the group. In total, we identified 4,764, 13,816, 4,676 and 6,731 families overrepresented in Fungi, Teretosporea, Labyrinthulea and Pseudofungi, respectively, compared with the background set of species (adjusted *P* value <0.05; adjusted *P* values were computed with the Benjamini–Hochberg procedure using the fdrcorrection function from statsmodels^79^ 0.13.5). Among these families overrepresented in at least one osmotrophic group, 189 of them are overrepresented in three or more of them, that is, in at least one osmotrophic group from Amorphea and at least one osmotrophic group from Diaphoretickes. We refer to this set of clusters as osmoclusters. We also performed an additional round of the same statistical analyses but excluding the photosynthetic species from the background set of species when performing the overrepresentation tests. Note that, sensu stricto, we did not consider as osmoclusters the overrepresented clusters resulting from this second round of analyses, since these are likely to be shared gene content signatures that are unlikely to be specific to the osmotrophic groups but also shared with the photosynthetic groups (see ‘Osmoclusters—genomic convergence between the osmotrophic groups’ section in the Results). The results from the two rounds of statistical tests are available in Supplementary Data.

### Functional annotation of the gene family clusters and metabolic pathways

To produce functional annotations of the clusters (related to Fig. 2b,c and Fig. 4d), we first annotated all the sequences contained in the clusters. To annotate enzyme information and functional category information, we run eggNOG-mapper^80^ version 2.0.1 on local mode. Among the retrieved annotations, we used the Cluster of Orthologous Groups (COG) functional categories^81^ and the KEGG Orthology Groups^82^. We considered as enzymes those KEGG Orthology Groups with associated Enzyme Commission numbers. Once individual sequences were annotated, for each cluster, we transferred the annotations made for their sequence cluster members by weightening every annotation found in at least one member of the cluster by the relative fraction of cluster members for which that specific annotation was detected, as performed in ref. ^63^. When transferring sequence annotations to clusters, sequences that were not annotated as enzymes were categorized as ‘No_enzyme’ before aggregating annotations on a cluster level. Regarding the transferring COG functional category annotations to clusters, for sequences with more than one COG category annotated, the contribution of each category was first divided by the amount of COG categories annotated for that sequence. All COG categories representing known functional information were considered.

We controlled for the potential interaction between cluster annotations and cluster size (number of sequence representatives of a cluster) when comparing the fraction of sequences annotated as enzymes in osmoclusters versus other clusters. In particular, we grouped gene family clusters into size bins ([1,2), [2,3), [3,4), [4,5), [5,10), [10,15), [15,20), [20,30), [30,50), [50,75), [75,100), [100,125), [125,150), [150,200), [200,300), [300,400), [400,500), [500,700), [700,1,000), [1,000,∞)), and performed 1,000 simulations, randomly sampling clusters from the set of clusters that are not osmocluster (non-osmoclusters) from every size bin so that in every simulation each bin is equally represented in the osmoclusters and in the subsampled set of clusters. During the cluster sampling process, only clusters that are not among osmoclusters, or clusters that were not previously sampled in the simulation round were considered. We then computed the average of the 1,000 values retrieved from each simulation to retrieve a single value representative of the whole range of interactions. The relative fraction of sequences with enzyme annotations corresponds to the opposite of the fraction of sequences without enzymatic annotations (85% in the non-osmoclusters clusters and 72% in osmoclusters). KEGG PATHWAY maps (related to Fig. 2c) were reconstructed using the online tool https://www.genome.jp/kegg/mapper/reconstruct.html (details in Supplementary Information 1 and 2).

### Species tree reconstruction and molecular clock analyses

The species tree used in the reconciliation analyses was constructed in the following manner. We first explored the filtered clusters with the aim of identifying a reliable subset from which to construct a supermatrix for the phylogenetic inference. In particular, we targeted those clusters having broad taxonomic distributions but at the same time few sequences per species to minimize paralogy problems. This led to 214 clusters that are present in at least 78 of the 86 species sampled and with a maximum of 100 sequences per cluster. Of these, we kept the 130 showing the highest ‘mean residue score’ from the heads or tails approach, which has been proposed as a measure of uncertainty in multiple sequence alignments^83^.

Before being concatenated into a supermatrix, these 130 clusters were cleaned of inparalogs and potential outparalogs (false cluster members), leaving one sequence per taxon. First, to remove inparalogs, in the cases when a given taxa had more than one representative in the cluster, we kept for each taxa the representative that aligned on average with the highest average BLAST score to the rest of the members of that cluster. By doing this, we should expect to retain the less divergent paralogs while removing the more divergent ones. Then, to remove potential outparalogs, we ranked the remaining members of each taxon in all the clusters by the mean BLAST score-based similarity that each member shows to the other members of the corresponding cluster, and then we excluded the 20 most divergent members for every taxon. By doing this, we expect to remove most, if not all, the outparalogs at the expense of having discarded only a very minor fraction of the dataset.

**Extended Data Fig. 5:**
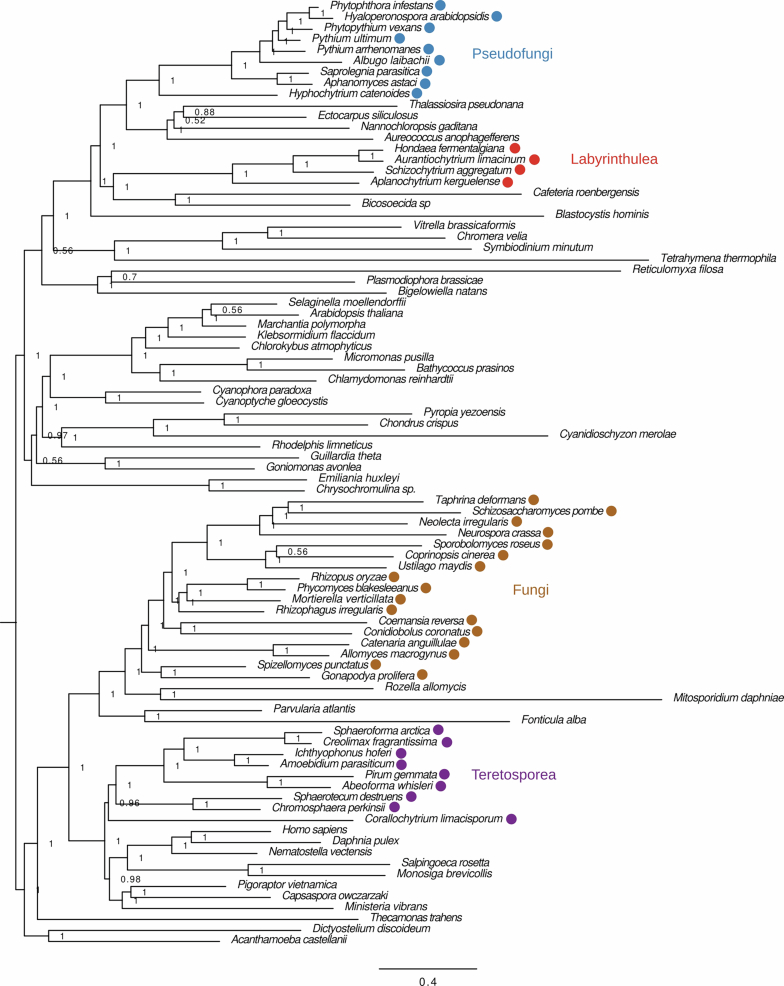
Maximum clade credibility tree retrieved from the two Phylobayes-MPI chains run under the CAT+GTR+Gamma(4) model on the multiple sequence alignment species concatenate, as described in Material and methods. Circles indicate species belonging to the osmotrophic groups.

After the cleaning process, clusters were aligned separately before being concatenated into a single supermatrix (alignment and alignment trimming were also carried out with MAFFT^84^ version 7.407 using the ‘-linsi’ option, and with trimAl^85^ 1.2rev59 using the ‘-gappyout’ option, respectively). The resulting supermatrix includes 86 taxa and 31,888 positions, with a median of 13.08% missing data. The phylogenetic inference was performed using Phylobayes-MPI^86^ version 1.8 under the CAT + GTR + Gamma(4) model, with two chains running for more than 13,000 generations until an effective size larger than 50 was reached for every parameter represented in the trace file. A consensus tree was built from the two chains with a burn-in of 25% (Extended Data Fig. 5). The sampled species tree topologies by each chain are available in Supplementary Data. The consensus tree recovered (Extended Data Fig. 5) is consistent with the current view of the eukaryotic species tree^87^, and the topology demonstrates robust support with high posterior probability values retrieved throughout, including the deepest parts of the tree (Extended Data Fig. 5). In addition, the consensus-tree topology was found to be better in the gene tree–species tree reconciliation analyses than three other alternative topologies, each of which included alternative topological resolutions for poorly supported bipartitions within Basidiomycota, Ochrophyta and Haptophyta (Supplementary Table 9). In particular, we performed different reconciliation analyses, each time using an alternative species tree, and the consensus-tree topology obtained the highest value for the sum of log-likelihoods (Supplementary Table 9). We thus used consensus-tree topology for the molecular dating and for the reconciliation analyses based on the original, pre-extended taxon sampling (see the Methods section ‘Re-analyses with an extended dataset’ below).

Molecular dating of the species tree was performed with Phylobayes 4.1c, again under the CAT + GTR + G4 model and using the same alignment and the topology obtained in the species tree reconstruction process. We ran two chains for each of the following relaxed molecular clock models (log-normal auto-correlated^88^ and uncorrelated gamma^89^) until at least 10,000 trees were sampled by each chain (30% of burn-in). All chains were run, imposing a set of fossil calibrations (Supplementary Table 7) under the soft constraints option and using a birth–death prior on divergence times. The sampled chronograms are available in Supplementary Data.

### Ancestral gene content estimation by phylogenetic reconciliation

**Extended Data Fig. 6:**
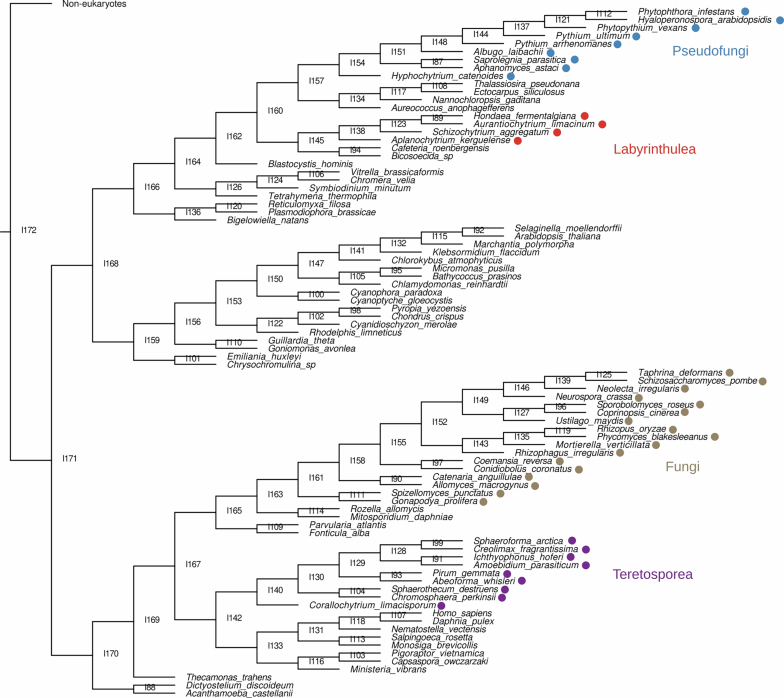
Topology of the species tree used for the phylogenomic gene tree-species tree reconciliation analyses done with the software ALE. Internal node names are also shown. Circles indicate species belonging to the osmogroups.

We inferred the gene contents of every ancestral lineage in our phylogeny by means of gene tree–species tree reconciliation^41^. This analysis required the species tree (see section above) and a sample of gene trees for every gene family cluster. Before performing gene tree sampling for every cluster, we first incorporated non-eukaryotic homologues into the clusters, as performed in ref. ^63^. We employed DIAMOND^90^ version 0.9.24 with the -more-sensitive parameter and an E-value threshold of 1 × 10^−5^. First, all eukaryotic sequences from the protein dataset of this study (euk_db) were aligned against the reference non-eukaryotic database used in ref. ^63^, which included the UniProt reference proteomes for Bacteria, Archaea and Virus (release 2016_02). This constituted the forward alignment approach. Next, sequences from prok_db that aligned to euk_db were subjected to a reverse alignment back against euk_db. Alignments with query and target coverages below 75% were discarded. In addition, any hit where the highest-scoring euk_db target for a given prok_db query belonged to a different cluster than the best-scoring euk_db query for that same prok_db sequence in the forward alignment was also removed. Following these filtering steps, we incorporated only the best-scoring prok_db query for each euk_db target sequence into the clusters (for example, if a cluster contained 300 euk_db sequences and the same prok_db sequence was the top hit for all of them, only that single prok_db sequence was added, resulting in a cluster with 300 euk_db sequences and 1 prok_db sequence). After having incorporated the non-eukaryotic homologues into the gene family clusters, we proceeded to sample gene trees for each cluster. We first performed multiple sequence alignments using MAFFT version 7.407 (‘-linsi’ option). Alignments were trimmed with trimAl 1.2rev59 (‘-gappyout’ option). The 1,000 optimized ultrafast bootstrap replicates were then sampled from each trimmed alignment using IQ-TREE^91^ version 1.6.12 under the LG + G4 model (Shimodaira–Hasegawa approximate likelihood-ratio test (SH-aLRT) bootstrap values were also computed). The sampled bootstrap replicates were used as gene tree samples to be reconciled against the species tree using the phylogenetic reconciliation software ALE^41^ (ALEml_undated from ALE version 1.0). In addition, in ref. ^63^, the species tree incorporated a pseudospecies branch representing all non-eukaryotic sequences, and transfers to eukaryotes from non-eukaryotes and transfers between eukaryotes were considered in the reconciliation model (Extended Data Fig. 6).

### Dating the genomic content of osmoclusters over time

We computed the mean osmocluster count per million years (My) time unit in the evolutionary path going from the root of our eukaryotic tree to the extant representatives of each osmotrophic group. In particular, for each osmotrophic group, we first computed the mean osmoclusters count in every branch found between the terminal nodes of the group and the root of the tree (both included). From this, we computed the mean osmocluster count for every time unit by performing the weighted arithmetic mean of the mean osmoclusters’ count of every branch under consideration, using as weights the fraction of post-burn-in chronograms supporting the existence of every lineage (branch) in the corresponding My time unit.

### Dating transfer signal inferred with ALE for a subset of osmoclusters

The output of ALE includes lineage-to-lineage transfer information. We collected this information for the subset of osmoclusters in which our manual screening identified signatures of HGT cases (see ‘Manually supervised identification of osmoclusters with putative HGT patterns’ section). Even in gene trees with topologies that are strongly suggestive of HGT patterns, sometimes it becomes challenging to establish the lineages that were involved in the HGT events owing to uncertainty, particularly if transfers occurred deep in evolutionary time (for example, ref. ^36^). In such cases, when there are different combinations of lineages that could have been involved in the HGT as donors–receptors, probabilistic reconciliation methods such as ALE can help in dealing with uncertainty in a probabilistic manner. ALE samples gene trees that have been reconciled against the species tree. From the sampled reconciliation scenarios, we can obtain the probability of pairs of donor–receptor lineages to have been involved in a HGT by taking into account the distribution of gene tree topologies consistent with the bootstrap samples obtained during the gene tree reconstruction step. By doing so, we could add up the results from the phylogenetic reconciliation analyses on the 40 osmoclusters in which we previously identified potential HGT topologies (see ‘Manually supervised identification of osmoclusters with putative HGT patterns’), and we aggregated the signal to get insights into an approximate distribution of the HGT signal over evolutionary time. For that, we dated the transfer events inferred with ALE (Fig. 3e) using the dates provided by the molecular dating analysis of the species tree.

The logic behind dating transfer events is the following. Every transfer must have happened in the period of time in which the respective donor and receptor lineages (branches) coexisted/overlapped in the chronogram. The dating software reports ages for nodes, not for branches. In a chronogram, every branch is delimited by two nodes (here referred to as birth and death nodes), and the age of a branch is defined by the ages of the respective birth and death nodes (here referred to as birth and death ages). Note that the model of ALE considers the possibility that transfers can come either from sampled lineages or from unsampled/extinct donor lineages descending from sampled lineages^92^. Because of this, when ALE outputs a given lineage as a transfer donor (for example, the lineage corresponding to the immediate ancestor of Fungi), this implies that the transfer could have occurred at any time between the birth of the donor lineage until the present. For example, if ALE reports as donor lineage the last common ancestor of Fungi, this means that the receptor could have acquired the gene either from the immediate branch preceding this node or from any unsampled/extinct lineage that may have diverged from the immediate branch preceding and became extinct at some point. Therefore, transfer ages are not constrained by the death age of the donor branches. In fact, they are delimited by two values: (1) the age of the death of the receptor lineage and (2) the birth age of either the donor or the receptor (whichever is the youngest).

On the basis of the information mentioned above, we dated transfers with the following approach: for every chronogram, we evaluated every branch in the role of donor and in the role of receptor. In the role of donor, a branch existed in the period of time delimited by its birth age and current time (0 My). In the role of receptor, a branch existed in the period of time delimited by its birth and death ages. We then checked the period of time in which every possible donor–receptor pair could have coexisted in every chronogram. After inspecting all chronograms, every possible donor–receptor pair has a value for every time unit (million years, from 0 My to the oldest root age found in the chronograms), which corresponds to the relative fraction of chronograms supporting the possibility that the two lineages could have coexisted in that specific time. We then normalized the distribution of values obtained for every donor–receptor pair by dividing the value of every time unit by the sum of values of all time units. Every transfer event was then dated according to the normalized distribution obtained for the corresponding donor–receptor pair. In particular, we obtained the probability of every transfer to have occurred in every million year time unit by multiplying the corresponding transfer frequency value (output from ALE) by the normalized value obtained for that time unit for the corresponding donor–receptor pair.

For Fig. 3e, we aggregated dated transfer events into two types: HGTs to osmotrophic groups originating from (1) osmotrophic groups or (2) other donors. In both cases, the osmotrophic lineages included all extant representatives and their preceding ancestral lineages. We also ranked the total HGT signal of these 40 osmoclusters against 100 background sets of 40 clusters, which were randomly sampled to maintain the same representation of cluster size bins as in the 40 osmoclusters (cluster size bins are defined in the ‘Functional annotation of the gene family clusters and metabolic pathways’ section). This yielded 101 values per time unit (one corresponding to the 40 osmoclusters and one for each background set of clusters), allowing us to identify the relative rank of the 40 osmoclusters at each timepoint (Supplementary Table 12). This analysis was performed for the 1,500–0 Ma period, as the transfer signal identified is residual before that period (Fig. 3e).

### Re-analyses with an extended dataset

The availability of protein species data, on the one hand, and the computational requirements of the analyses performed, on the other, are two important factors influencing the size and the set of species to be included in a given comparative genomics analyses. Regarding the latter point, the all-versus-all BLASTP alignments used to build the gene family clusters, the relaxed molecular clock analyses used to obtain age estimates for the osmotrophic groups and the gene tree–species tree reconciliation analyses used to estimate ancestral gene contents are particularly computationally taxing. We prioritized a balanced representation of as much phylogenetic diversity as possible, not only within the osmotrophic groups, but also among different clades from Amorphea and Diaphoretickes, the two divisions of eukaryotes in which the four osmotrophic groups belong to. Our taxon sampling allowed the execution of state-of-the-art methods; for example, we could employ BLASTP, the baseline all-against-all protein alignment tool, we could run sophisticated phylogenetic models for the inference of the species topology and the relaxed molecular clock analysis and we could perform ancestral state reconstruction by employing maximum-likelihood phylogenetic reconciliation tools. We explored whether we could identify signatures that the patterns seen in our results could have been driven to some extent by a limited representation in our taxon sampling. For that, we re-ran those analyses that are less computationally prohibitive with an extended taxon sampling that incorporates 35 additional species’ proteomes for a total of 121 represented proteomes. Apart from providing an expanded taxonomic coverage of previously represented groups, this extended dataset also incorporated proteome data from Discoba, Metamonada and Malawimonadida, all of which branch outside the Amorphea and Diaphoretickes divisions of eukaryotes (Supplementary Table 8). Proteome data for the extended dataset was selected from ‘The Comparative Set’, which consists of 196 species chosen by the developers of EukProt (version 3) on the basis of BUSCO completion and phylogenetic importance^93^. Among the set of species that could be selected, we discarded those with reported evidence of contamination^61^. We also avoided incorporating proteomes coming from transcriptomic data that showed high values of sequence redundancy according to the duplicated BUSCO score. For the re-analyses run under this extended taxon sampling (Extended Data Figs. 3 and 4), we had to incorporate sequences from the extended proteomes into the original gene family clusters, as well as incorporate the extended species set into the reconstructed species tree. Regarding the extension of the clusters, we did so by performing BLASTP alignments of the extended proteomes against a joined database with the rest of the species’ proteomes, (parameters: -evalue 1e-3 -max_target_seqs 250), assigning each sequence to the previously defined gene family clusters on the basis of the best hit criteria. Regarding the species tree extension, we identified the most likely phylogenetic placements of the extended taxa by performing a comprehensive screening of the available literature (Supplementary Table 8).

### Manually supervised identification of osmoclusters with putative HGT patterns

We performed a thorough evaluation of each osmocluster, one by one, to retrieve a curated subset of osmoclusters showing putative HGT patterns in their tree topologies. When HGT takes place, it is expected to leave signatures in the genome. The younger the HGT event is, the higher the degree of conservation of HGT signatures will be in the genomes of extant species, and vice versa. For old HGT events, it can become challenging to identify the exact identity of the donor and receptor lineages involved, but there may still be incongruent phylogenetic signals supporting the occurrence of the HGT. The evaluation of HGT cases in osmoclusters consisted of checking in parallel three types of information for each osmocluster: (1) in the gene tree (poorly supported bipartitions with <80% SH-aLRT bootstrap value were collapsed), we searched for clades involving sequences from distantly related osmotrophic groups (that is, osmotrophic groups from Amorphea branching closely related to osmotrophic groups from Diaphoretickes; these are less likely to be explained by gene losses, which are pervasive in evolution^43,94^). Gene trees incorporated sequences from the extended species proteomes; (2) in the taxonomic distribution of the cluster, the more biased the distribution is towards osmotrophic groups, the less likely is that the observed distribution could be explained by differential duplication and loss patterns rather than by HGT; (3) sequence similarity searches between each sequence in the osmocluster and every sequence included in our study were also considered as gene-tree-independent evidence. This third source of information is important to validate that the potential HGT trends in the gene tree are not artefacts derived from errors in the gene family clustering or in the phylogenetic reconstruction processes. Alignments for (3) were performed with DIAMOND version 2.0.14.152, aligning every query sequence against a concatenated database of the eukaryotic and non-eukaryotic sequences included in our study, including the extended eukaryotic species proteomes (-evalue 1e-3 -more-sensitive). See Supplementary Information 3 for a detailed explanation on how these three sources of information were crossed to evaluate putative HGT patterns in osmoclusters, including a specific report for each osmocluster case identified.

### Semi-automated quantification of HGT topologies in all clusters

Beyond osmoclusters, we also explored the whole set of gene family clusters for highly supported HGT clades involving sequences from distantly related osmotrophic groups. We used the ETE^95^ version 3 toolkit to search for clades including only sequences from two osmotrophic groups, each belonging to a distinct eukaryotic division: Pseudofungi–Fungi, Pseudofungi–Teretosporea, Labyrinthulea–Fungi and Labyrinthulea–Teretosporea. We did not consider pairs of osmotrophic groups’ pairs belonging to the same eukaryotic division to minimize chances of false-positive HGT inferences that could be confounded by complex duplication and loss patterns. Before clade inspection, poorly supported clades from the phylogenies (<80% SH-aLRT bootstrap support) were collapsed and turned into polytomies. A total of 1,267 clades in 1,029 clusters passed this automated filter step. In 442 of these 1,267 clades, we found at least one sequence from each of the two osmotrophic groups involved in the clade that performed the best hit in the Diamond alignments carried out for the previous section to a sequence belonging to the other osmotrophic group included in the clade (ignoring in any case hits involving sequences belonging to the same taxonomic category). To provide a specific example, in a clade incorporating sequences from Pseudofungi and Fungi, at least one sequence from Pseudofungi must return its best-alignment score against a sequence from Fungi (excluding hits from Pseudofungi), and in addition, at least one sequence from Fungi must return a best-alignment score against a sequence from Pseudofungi (excluding hits from Fungi). The remaining 442 clades, representing 398 clusters, were manually inspected in detail, exploring (i) the topology of the gene tree for the corresponding cluster and (ii) the taxonomic distribution of the best hits in Diamond alignments for the sequences from the two osmotrophic groups involved in the clade. After a case-by-case inspection, 175 clades from 172 clusters were considered as strongly suggestive of HGTs involving taxa from distantly related osmotrophic groups. Overall, 166 of these clades representing 163 clusters were validated after a re-analysis, which consisted of recomputing the gene trees and the Diamond alignments on the basis of the extended taxon sampling (Supplementary Information 4). On the basis of a rigorous inspection of the taxonomic composition of the sister branches to the HGT clades, and of the taxonomic patterns of the Diamond alignment results, among the 166 cases of HGT, we could confidently establish which osmotrophic group acted as the HGT donor for 62 cases. For these 62 cases, when there was uncertainty in the specific lineages involved as HGT donors–receptors within each osmotrophic group, HGT counts were fractionated and assigned to each plausible donor–receptor scenario identified.

### Reporting summary

Further information on research design is available in the Nature Portfolio Reporting Summary linked to this article.

## Supplementary information

Supplementary Information Supplementary Information 1–4.

Reporting Summary

Peer Review File

Supplementary Tables Supplementary Tables 1–12.

## Data Availability

Supplementary Data are available via figshare at 10.6084/m9.figshare.28759352 (ref. ^96^).
