# Supplementary material for: Signatures of gene transfer in the parallel evolution of osmotrophic specialization in eukaryotes
Source: Nat Ecol Evol. 2026 May 25;10(7):1342–55. doi: 10.1038/s41559-026-03054-w (PMC13345962; doi:10.1038/s41559-026-03054-w)
Supplement: Supplementary file 1 — Supplementary Information 1–4. [file 41559_2026_3054_MOESM1_ESM.pdf]

nature ecology & evolution

Supplementary information

https://doi.org/10.1038/s41559-026-03054-w

Signatures of gene transfer in the parallel evolution of osmotrophic specialization in eukaryotes

In the format provided by the authors and unedited

Supplementary Information 1 to 4

- Supplementary Information 1 – pg. 2 in PDF file

KEGG pathway map representations including enzymes in *Homo sapiens* and enzymes in the osmoclusters. Referenced in the ‘Osmoclusters: genomic convergence between the osmotrophic groups’ section from the main text

- Supplementary Information 2 – pg. 14 in PDF file

KEGG pathway map representations including enzymes in *Dictyostelium discoideum* and enzymes in the osmoclusters. Referenced in the ‘Osmoclusters: genomic convergence between the osmotrophic groups’ section from the main text

- Supplementary Information 3 – pg. 26 in PDF file

Report of the positive cases identified in the supervised inspection of putative HGT patterns in osmoclusters. Referenced in the ‘Horizontal gene transfers in osmoclusters’ section from the main text

- Supplementary Information 4 – pg. 74 in PDF file

Supporting files (phylogenetic tree and Diamond allignment score plots) for the 166 cases of HGT topologies identified by analysing the whole set of gene families present in osmotrophic groups. Referenced in the ‘Horizontal gene transfers beyond osmoclusters’ section from the main text

Supplementary Information 1

KEGG pathway map representations including enzymes in *Homo sapiens* and enzymes in the osmoclusters. Referenced in the ‘Osmoclusters: genomic convergence between the osmotrophic groups’ section from the main text

KEGG PATHWAY maps (related to Fig. 2C) were reconstructed using the online tool https://www.genome.jp/kegg/mapper/reconstruct.html. We thank Kanehisa Laboratories for permission to publish results from KEGG and KEGG images (Kanehisa et al. 2000). For this, we uploaded a text file including a concatenation of three lists of KO (KEGG ORTHOLOGY) ids: (1) KO ids that can be confidently be considered to be present among osmoclusters (at least >=50% of sequences from at least one osmocluster were annotated with that KO id), (2) KO ids that for which annotation presence is less certain but likely (at least >=25% of sequences from at least one osmocluster were annotated with that KO id), (3-4) Same as (1) and (2), but for additional set of clusters that are identified as overrepresented in osmotrophic groups compared to background heterotrophic species, but excluding photosynthetic organisms from the background set of species in the overrepresentation tests, and (5) all KO ids annotated to *Homo sapiens* proteome in the KEGG website on the 24th of February 2025. We used purple, pink, orange, light blue and green color codes for lists 1 to 5, respectively.

VALINE, LEUCINE AND ISOLEUCINE BIOSYNTHESIS

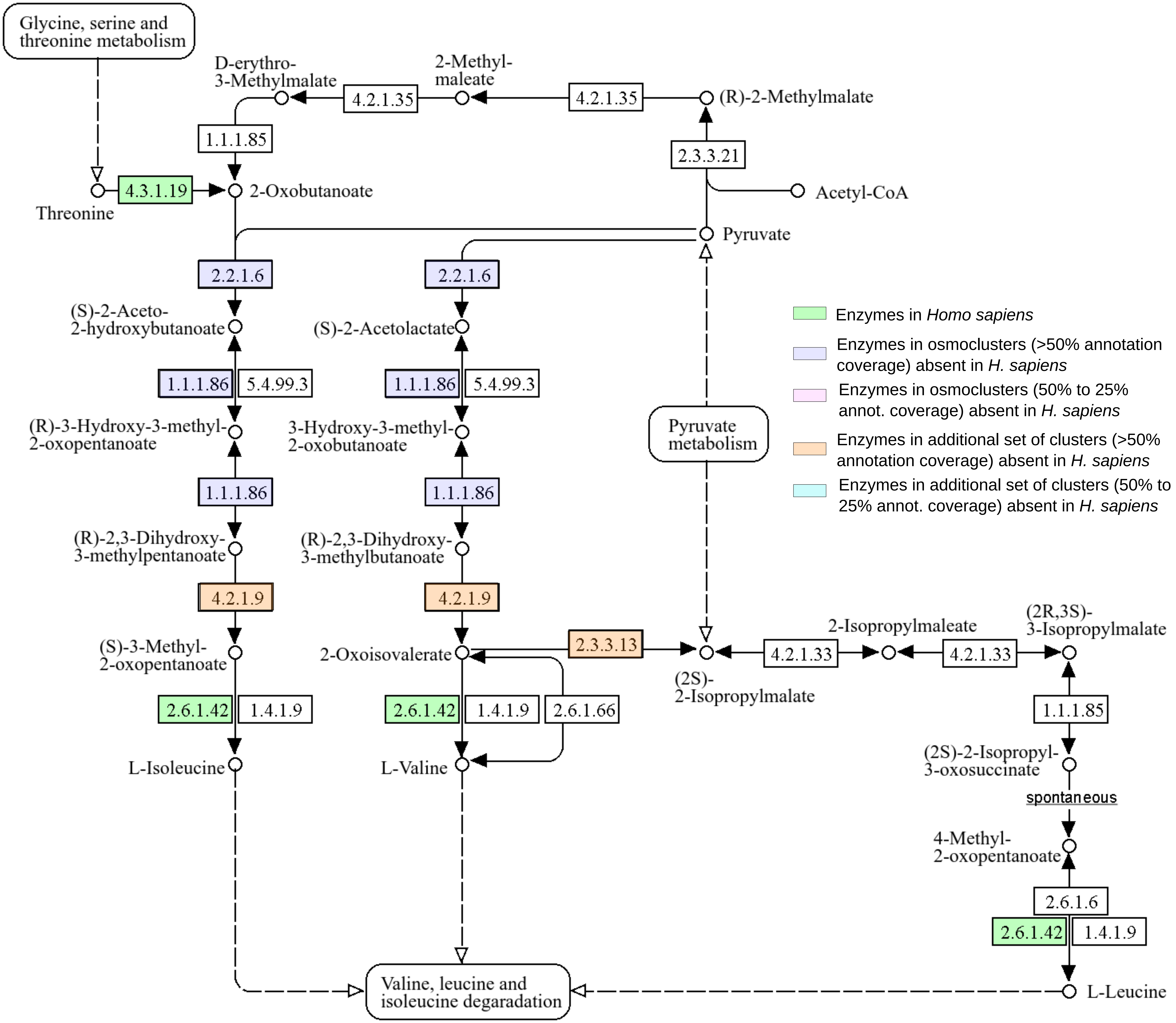

00290 12/27/21
(c) Kanehisa Laboratories

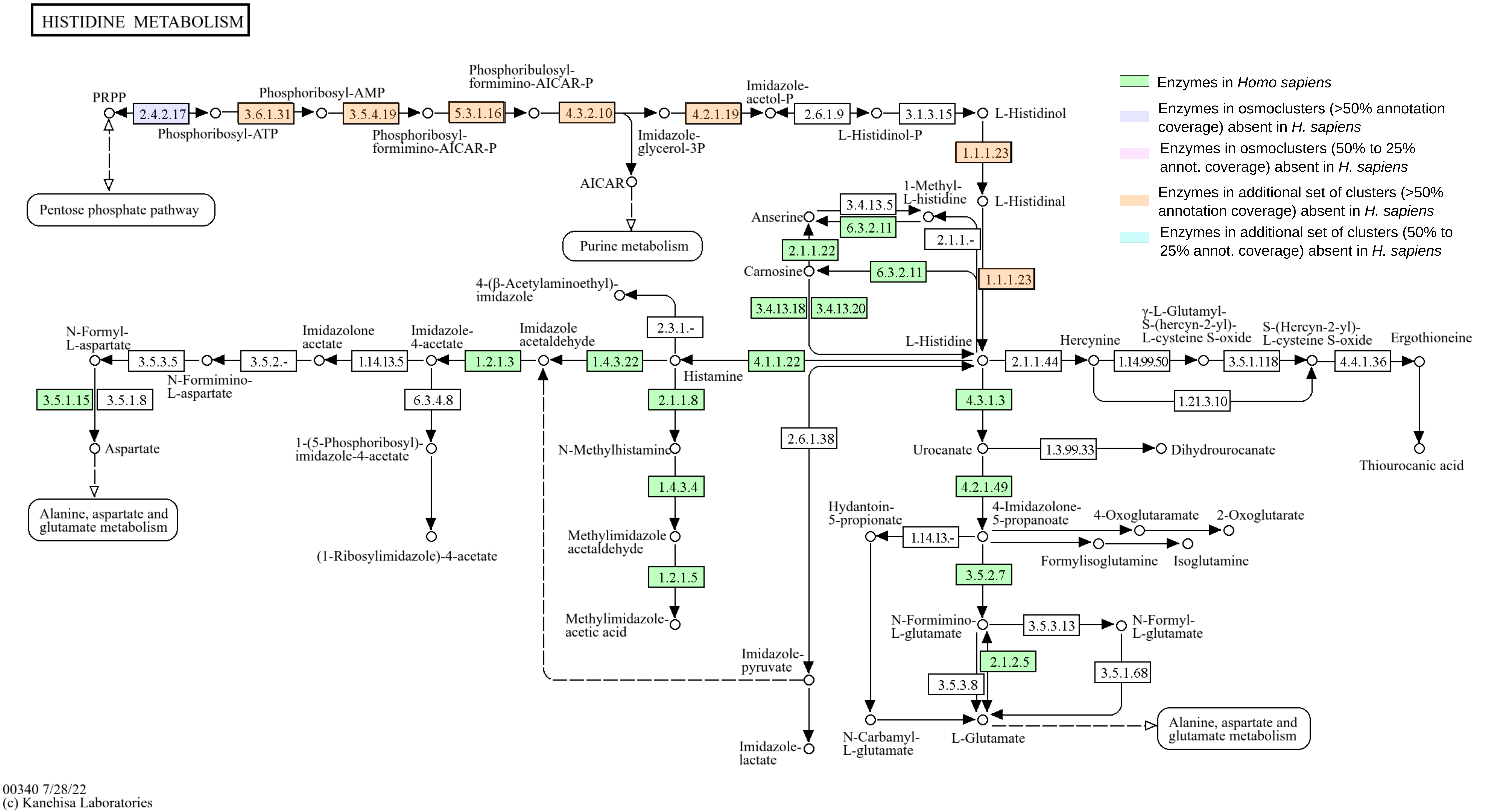
HISTIDINE METABOLISM
Enzymes in Homo sapiens
Enzymes in osmoclusters (>50% annotation coverage) absent in H. sapiens
Enzymes in osmoclusters (50% to 25% annot. coverage) absent in H. sapiens
Enzymes in additional set of clusters (>50% annotation coverage) absent in H. sapiens
Enzymes in additional set of clusters (50% to 25% annot. coverage) absent in H. sapiens
PRPP
2.4.2.17
Phosphoribosyl-ATP
3.6.1.31
Phosphoribosyl-AMP
3.5.4.19
Phosphoribosyl-formimino-AICAR-P
5.3.1.16
Phosphoribulosyl-formimino-AICAR-P
4.3.2.10
Imidazole-glycerol-3P
AICAR
Purine metabolism
4.2.1.19
Imidazole-acetol-P
2.6.1.9
L-Histidinol-P
3.1.3.15
L-Histidinol
1.1.1.23
L-Histidinal
1.1.1.23
Pentose phosphate pathway
1-Methyl-L-histidine
3.4.13.5
6.3.2.11
Anserine
2.1.1.-
2.1.1.22
Carnosine
6.3.2.11
3.4.13.18
3.4.13.20
4-(β-Acetylaminoethyl)-imidazole
2.3.1.-
L-Histidine
4.1.1.22
Histamine
1.4.3.22
Imidazole acetaldehyde
1.2.1.3
Imidazole-4-acetate
1.14.13.5
Imidazolone acetate
3.5.2.-
N-Formimino-L-aspartate
3.5.3.5
N-Formyl-L-aspartate
3.5.1.15
3.5.1.8
Aspartate
Alanine, aspartate and glutamate metabolism
6.3.4.8
1-(5-Phosphoribosyl)-imidazole-4-acetate
(1-Ribosylimidazole)-4-acetate
2.1.1.8
N-Methylhistamine
1.4.3.4
Methylimidazole acetaldehyde
1.2.1.5
Methylimidazole-acetic acid
2.6.1.38
Imidazole-pyruvate
Imidazole-lactate
2.1.1.44
Hercynine
1.14.99.50
γ-L-Glutamyl-S-(hercyn-2-yl)-L-cysteine S-oxide
3.5.1.118
S-(Hercyn-2-yl)-L-cysteine S-oxide
4.4.1.36
Ergothioneine
1.21.3.10
Thiourocanic acid
4.3.1.3
Urocanate
1.3.99.33
Dihydrourocanate
4.2.1.49
4-Imidazolone-5-propanoate
4-Oxoglutaramate
2-Oxoglutarate
Formylisoglutamine
Isoglutamine
Hydantoin-5-propionate
1.14.13.-
3.5.2.7
N-Formimino-L-glutamate
3.5.3.13
N-Formyl-L-glutamate
2.1.2.5
3.5.3.8
3.5.1.68
N-Carbamyl-L-glutamate
L-Glutamate
Alanine, aspartate and glutamate metabolism
00340 7/28/22
(c) Kanehisa Laboratories

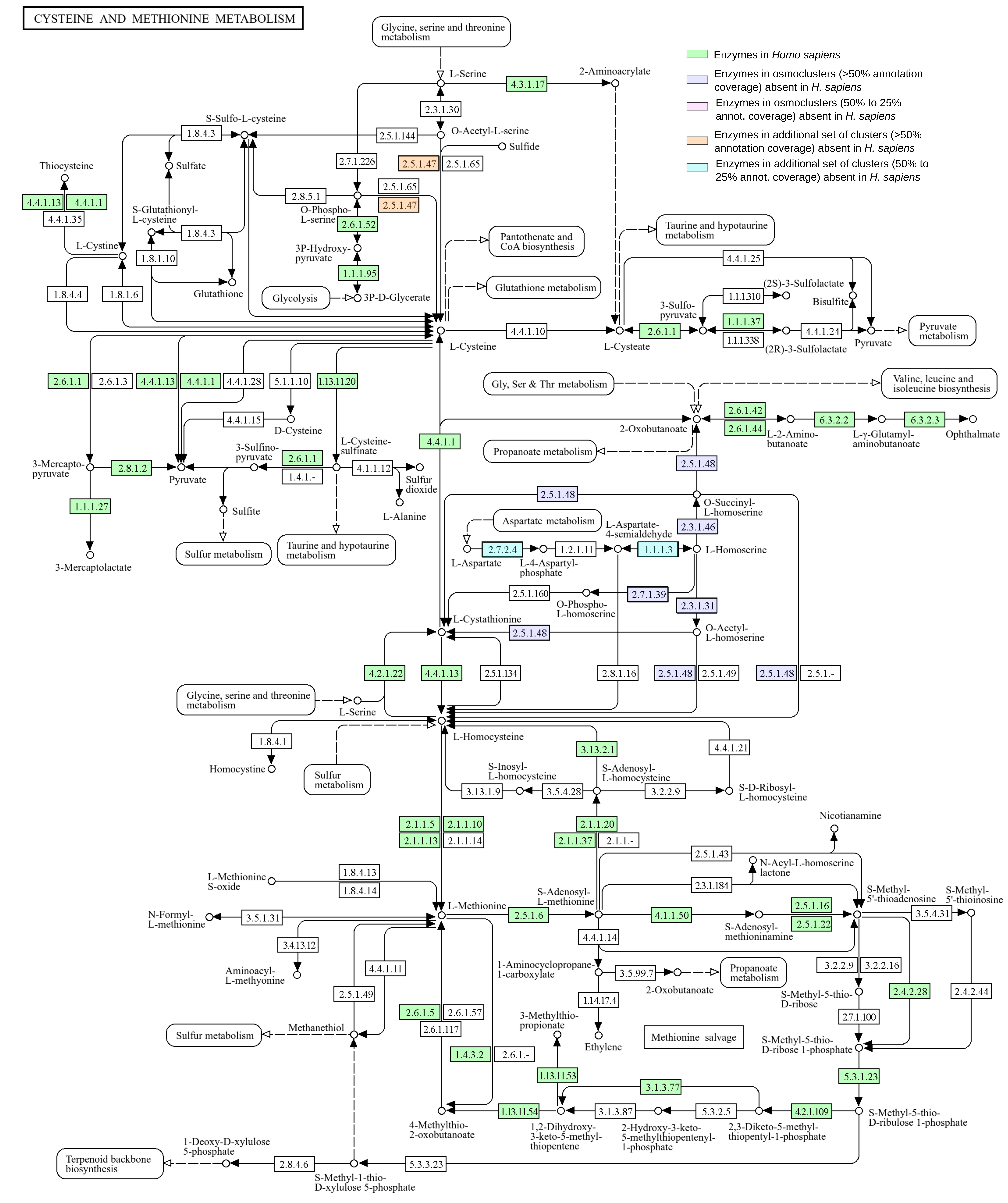

00270 1/10/25

(c) Kanehisa Laboratories

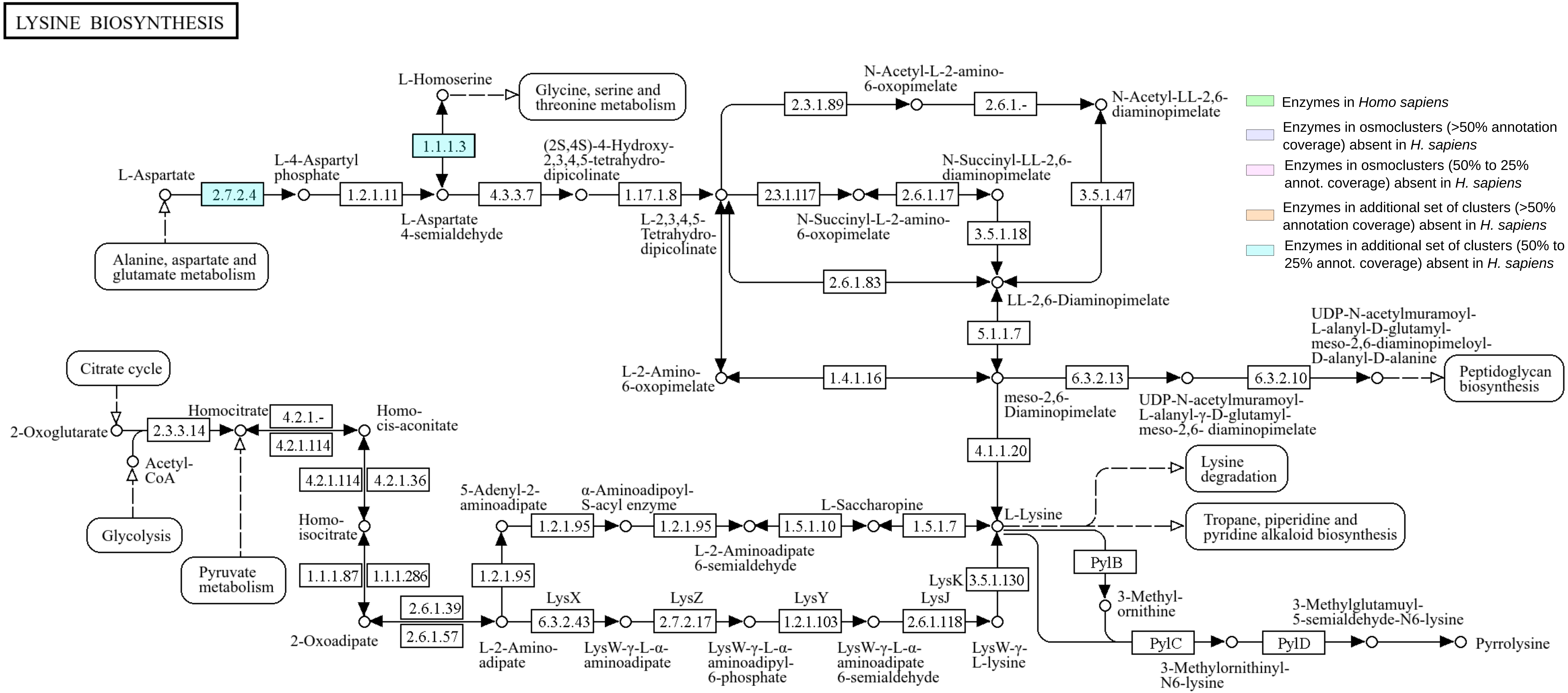

00300 7/29/22
(c) Kanehisa Laboratories

PHENYLALANINE, TYROSINE AND TRYPTOPHAN BIOSYNTHESIS

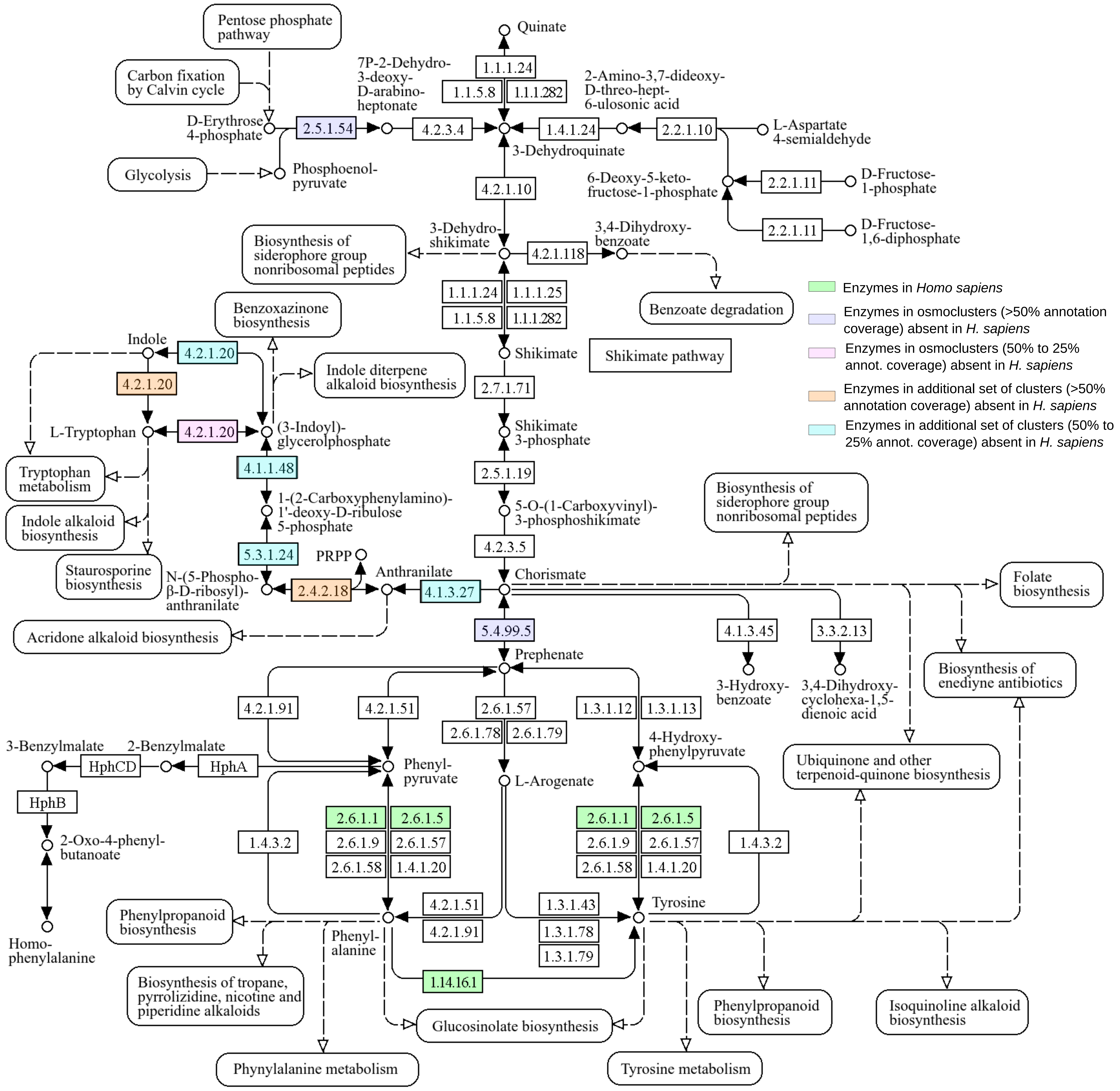

00400 6/10/24

(c) Kanehisa Laboratories

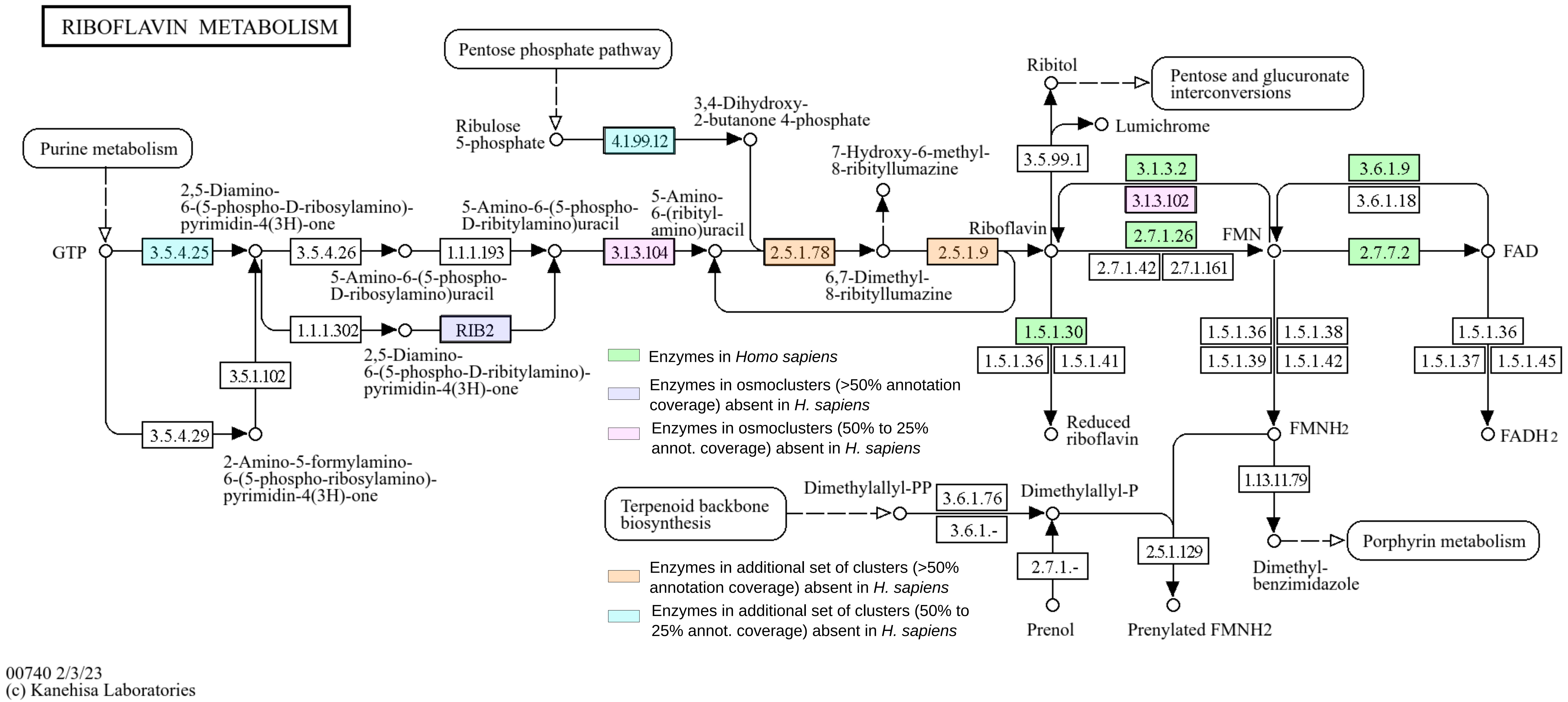
RIBOFLAVIN METABOLISM
Pentose phosphate pathway
Purine metabolism
Ribulose 5-phosphate
4.1.99.12
3,4-Dihydroxy-2-butanone 4-phosphate
GTP
3.5.4.25
2,5-Diamino-6-(5-phospho-D-ribosylamino)-pyrimidin-4(3H)-one
3.5.4.26
5-Amino-6-(5-phospho-D-ribosylamino)uracil
1.1.1.193
5-Amino-6-(5-phospho-D-ribitylamino)uracil
3.1.3.104
5-Amino-6-(ribityl-amino)uracil
2.5.1.78
7-Hydroxy-6-methyl-8-ribityllumazine
6,7-Dimethyl-8-ribityllumazine
2.5.1.9
Riboflavin
1.1.1.302
RIB2
2,5-Diamino-6-(5-phospho-D-ribitylamino)-pyrimidin-4(3H)-one
3.5.1.102
3.5.4.29
2-Amino-5-formylamino-6-(5-phospho-ribosylamino)-pyrimidin-4(3H)-one
Ribitol
Pentose and glucuronate interconversions
Lumichrome
3.5.99.1
3.1.3.2
3.1.3.102
2.7.1.26
2.7.1.42
2.7.1.161
FMN
3.6.1.9
3.6.1.18
2.7.7.2
FAD
1.5.1.30
1.5.1.36
1.5.1.41
Reduced riboflavin
1.5.1.36
1.5.1.38
1.5.1.39
1.5.1.42
FMNH2
1.5.1.36
1.5.1.37
1.5.1.45
FADH2
1.13.11.79
Dimethyl-benzimidazole
Porphyrin metabolism
Terpenoid backbone biosynthesis
Dimethylallyl-PP
3.6.1.76
3.6.1.-
Dimethylallyl-P
2.7.1.-
Prenol
2.5.1.129
Prenylated FMNH2
Enzymes in Homo sapiens
Enzymes in osmoclusters (>50% annotation coverage) absent in H. sapiens
Enzymes in osmoclusters (50% to 25% annot. coverage) absent in H. sapiens
Enzymes in additional set of clusters (>50% annotation coverage) absent in H. sapiens
Enzymes in additional set of clusters (50% to 25% annot. coverage) absent in H. sapiens
00740 2/3/23
(c) Kanehisa Laboratories

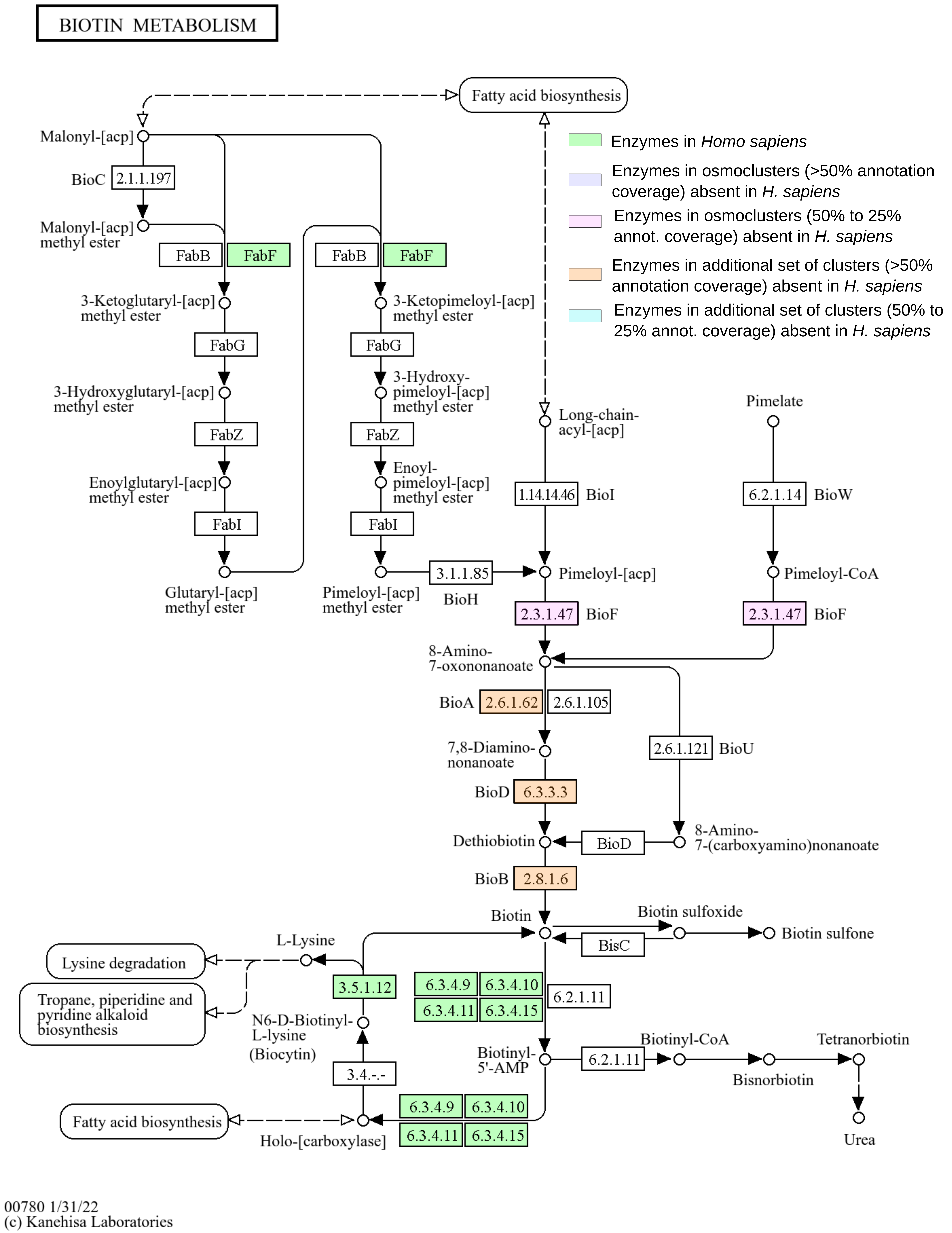
BIOTIN METABOLISM
Fatty acid biosynthesis
Enzymes in Homo sapiens
Enzymes in osmoclusters (>50% annotation coverage) absent in H. sapiens
Enzymes in osmoclusters (50% to 25% annot. coverage) absent in H. sapiens
Enzymes in additional set of clusters (>50% annotation coverage) absent in H. sapiens
Enzymes in additional set of clusters (50% to 25% annot. coverage) absent in H. sapiens
Malonyl-[acp]
BioC
2.1.1.197
Malonyl-[acp] methyl ester
FabB
FabF
FabB
FabF
3-Ketoglutaryl-[acp] methyl ester
3-Ketopimeloyl-[acp] methyl ester
FabG
FabG
3-Hydroxyglutaryl-[acp] methyl ester
3-Hydroxy-pimeloyl-[acp] methyl ester
FabZ
FabZ
Enoylglutaryl-[acp] methyl ester
Enoyl-pimeloyl-[acp] methyl ester
FabI
FabI
Glutaryl-[acp] methyl ester
Pimeloyl-[acp] methyl ester
3.1.1.85
BioH
Long-chain-acyl-[acp]
1.14.14.46
BioI
Pimelate
6.2.1.14
BioW
Pimeloyl-[acp]
Pimeloyl-CoA
2.3.1.47
BioF
2.3.1.47
BioF
8-Amino-7-oxononanoate
BioA
2.6.1.62
2.6.1.105
7,8-Diamino-nonanoate
2.6.1.121
BioU
BioD
6.3.3.3
Dethiobiotin
BioD
8-Amino-7-(carboxyamino)nonanoate
BioB
2.8.1.6
Biotin
Biotin sulfoxide
Biotin sulfone
BisC
L-Lysine
Lysine degradation
Tropane, piperidine and pyridine alkaloid biosynthesis
3.5.1.12
6.3.4.9
6.3.4.10
6.3.4.11
6.3.4.15
6.2.1.11
N6-D-Biotinyl-L-lysine (Biocytin)
Biotinyl-5'-AMP
6.2.1.11
Biotinyl-CoA
Tetranorbiotin
Bisnorbiotin
3.4.-.-
6.3.4.9
6.3.4.10
6.3.4.11
6.3.4.15
Fatty acid biosynthesis
Holo-[carboxylase]
Urea
00780 1/31/22
(c) Kanehisa Laboratories

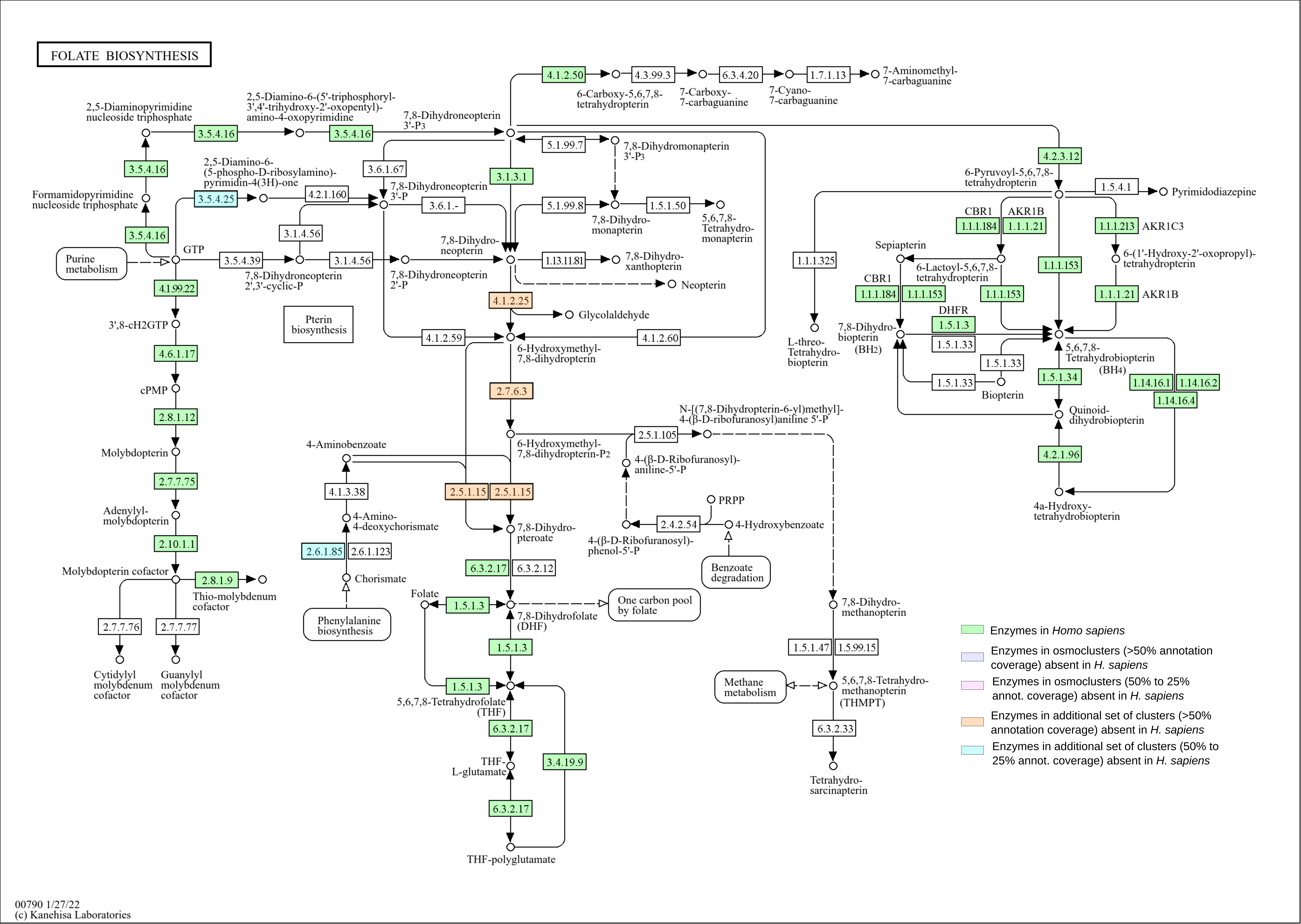

FOLATE BIOSYNTHESIS
2,5-Diaminopyrimidine nucleoside triphosphate
3.5.4.16
2,5-Diamino-6-(5'-triphosphoryl-3',4'-trihydroxy-2'-oxopentyl)-amino-4-oxopyrimidine
3.5.4.16
7,8-Dihydroneopterin 3'-P3
4.1.2.50
6-Carboxy-5,6,7,8-tetrahydropterin
4.3.99.3
7-Carboxy-7-carbaguanine
6.3.4.20
7-Cyano-7-carbaguanine
1.7.1.13
7-Aminomethyl-7-carbaguanine
5.1.99.7
7,8-Dihydromonapterin 3'-P3
3.1.3.1
3.6.1.67
7,8-Dihydroneopterin 3'-P
3.6.1.-
2,5-Diamino-6-(5-phospho-D-ribosylamino)-pyrimidin-4(3H)-one
3.5.4.25
4.2.1.160
Formamidopyrimidine nucleoside triphosphate
3.5.4.16
3.5.4.16
Purine metabolism
GTP
3.5.4.39
7,8-Dihydroneopterin 2',3'-cyclic-P
3.1.4.56
3.1.4.56
7,8-Dihydroneopterin 2'-P
7,8-Dihydroneopterin
5.1.99.8
7,8-Dihydromonapterin
1.5.1.50
5,6,7,8-Tetrahydromonapterin
1.13.11.81
7,8-Dihydroxanthopterin
Neopterin
4.1.2.25
Glycolaldehyde
4.1.99.22
3',8-cH2GTP
Pterin biosynthesis
4.1.2.59
4.1.2.60
6-Hydroxymethyl-7,8-dihydropterin
4.6.1.17
cPMP
2.7.6.3
2.8.1.12
Molybdopterin
6-Hydroxymethyl-7,8-dihydropterin-P2
2.5.1.105
N-[(7,8-Dihydropterin-6-yl)methyl]-4-(β-D-ribofuranosyl)aniline 5'-P
4-(β-D-Ribofuranosyl)-aniline-5'-P
4-Aminobenzoate
4.1.3.38
2.5.1.15
2.5.1.15
2.7.7.75
Adenylyl-molybdopterin
4-Amino-4-deoxychorismate
7,8-Dihydropteroate
PRPP
2.4.2.54
4-Hydroxybenzoate
4-(β-D-Ribofuranosyl)-phenol-5'-P
Benzoate degradation
2.10.1.1
2.6.1.85
2.6.1.123
Chorismate
6.3.2.17
6.3.2.12
Molybdopterin cofactor
2.8.1.9
Thio-molybdenum cofactor
Phenylalanine biosynthesis
Folate
1.5.1.3
7,8-Dihydrofolate (DHF)
One carbon pool by folate
7,8-Dihydromethanopterin
2.7.7.76
2.7.7.77
Cytidylyl molybdenum cofactor
Guanylyl molybdenum cofactor
1.5.1.3
1.5.1.3
5,6,7,8-Tetrahydrofolate (THF)
1.5.1.47
1.5.99.15
Methane metabolism
5,6,7,8-Tetrahydromethanopterin (THMPT)
6.3.2.33
Tetrahydrosarcinapterin
6.3.2.17
THF-L-glutamate
3.4.19.9
6.3.2.17
THF-polyglutamate
4.2.3.12
6-Pyruvoyl-5,6,7,8-tetrahydropterin
1.5.4.1
Pyrimidodiazepine
CBR1
1.1.1.184
AKR1B
1.1.1.21
1.1.1.213
AKR1C3
Sepiapterin
6-Lactoyl-5,6,7,8-tetrahydropterin
6-(1'-Hydroxy-2'-oxopropyl)-tetrahydropterin
1.1.1.325
1.1.1.153
CBR1
1.1.1.184
1.1.1.153
1.1.1.153
1.1.1.21
AKR1B
DHFR
1.5.1.3
L-threo-Tetrahydrobiopterin
7,8-Dihydrobiopterin (BH2)
1.5.1.33
1.5.1.33
1.5.1.33
Biopterin
5,6,7,8-Tetrahydrobiopterin (BH4)
1.5.1.34
1.14.16.1
1.14.16.2
1.14.16.4
Quinoid-dihydrobiopterin
4.2.1.96
4a-Hydroxytetrahydrobiopterin
Enzymes in Homo sapiens
Enzymes in osmoclusters (>50% annotation coverage) absent in H. sapiens
Enzymes in osmoclusters (50% to 25% annot. coverage) absent in H. sapiens
Enzymes in additional set of clusters (>50% annotation coverage) absent in H. sapiens
Enzymes in additional set of clusters (50% to 25% annot. coverage) absent in H. sapiens
00790 1/27/22
(c) Kanehisa Laboratories

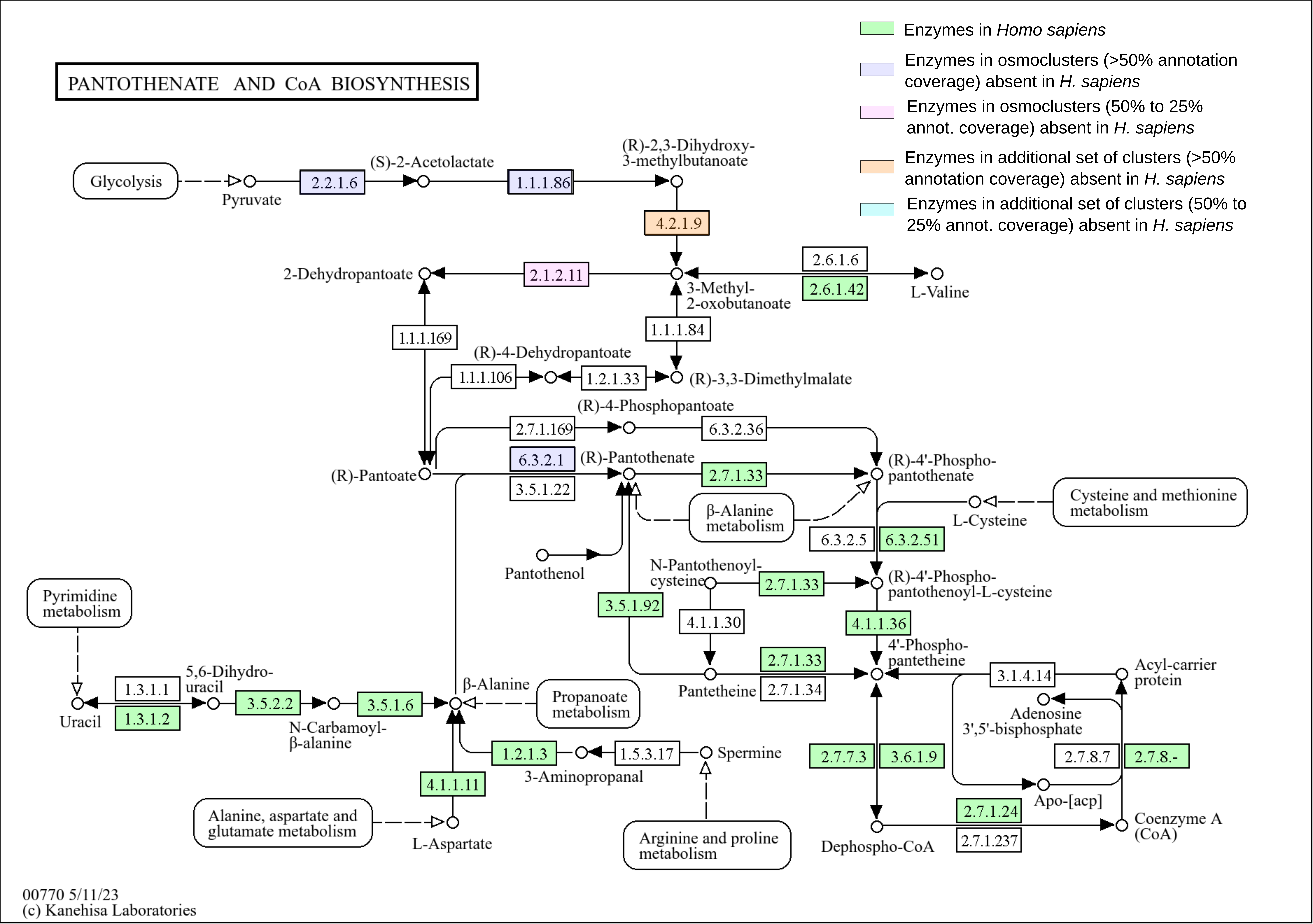

Enzymes in Homo sapiens
Enzymes in osmoclusters (>50% annotation coverage) absent in H. sapiens
Enzymes in osmoclusters (50% to 25% annot. coverage) absent in H. sapiens
Enzymes in additional set of clusters (>50% annotation coverage) absent in H. sapiens
Enzymes in additional set of clusters (50% to 25% annot. coverage) absent in H. sapiens
PANTOTHENATE AND CoA BIOSYNTHESIS
Glycolysis
Pyruvate
2.2.1.6
(S)-2-Acetolactate
1.1.1.86
(R)-2,3-Dihydroxy-3-methylbutanoate
4.2.1.9
2-Dehydropantoate
2.1.2.11
3-Methyl-2-oxobutanoate
2.6.1.6
2.6.1.42
L-Valine
1.1.1.169
1.1.1.84
(R)-4-Dehydropantoate
1.1.1.106
1.2.1.33
(R)-3,3-Dimethylmalate
(R)-4-Phosphopantoate
2.7.1.169
6.3.2.36
(R)-Pantoate
6.3.2.1
3.5.1.22
(R)-Pantothenate
2.7.1.33
(R)-4'-Phospho-pantothenate
β-Alanine metabolism
L-Cysteine
Cysteine and methionine metabolism
6.3.2.5
6.3.2.51
Pantothenol
N-Pantothenoyl-cysteine
2.7.1.33
(R)-4'-Phospho-pantothenoyl-L-cysteine
3.5.1.92
4.1.1.30
4.1.1.36
4'-Phospho-pantetheine
2.7.1.33
Pantetheine
2.7.1.34
3.1.4.14
Acyl-carrier protein
Adenosine 3',5'-bisphosphate
Pyrimidine metabolism
Uracil
1.3.1.1
1.3.1.2
5,6-Dihydro-uracil
3.5.2.2
N-Carbamoyl-β-alanine
3.5.1.6
β-Alanine
Propanoate metabolism
1.2.1.3
1.5.3.17
Spermine
3-Aminopropanal
4.1.1.11
Alanine, aspartate and glutamate metabolism
L-Aspartate
Arginine and proline metabolism
2.7.7.3
3.6.1.9
2.7.8.7
2.7.8.-
Apo-[acp]
2.7.1.24
2.7.1.237
Dephospho-CoA
Coenzyme A (CoA)
00770 5/11/23
(c) Kanehisa Laboratories

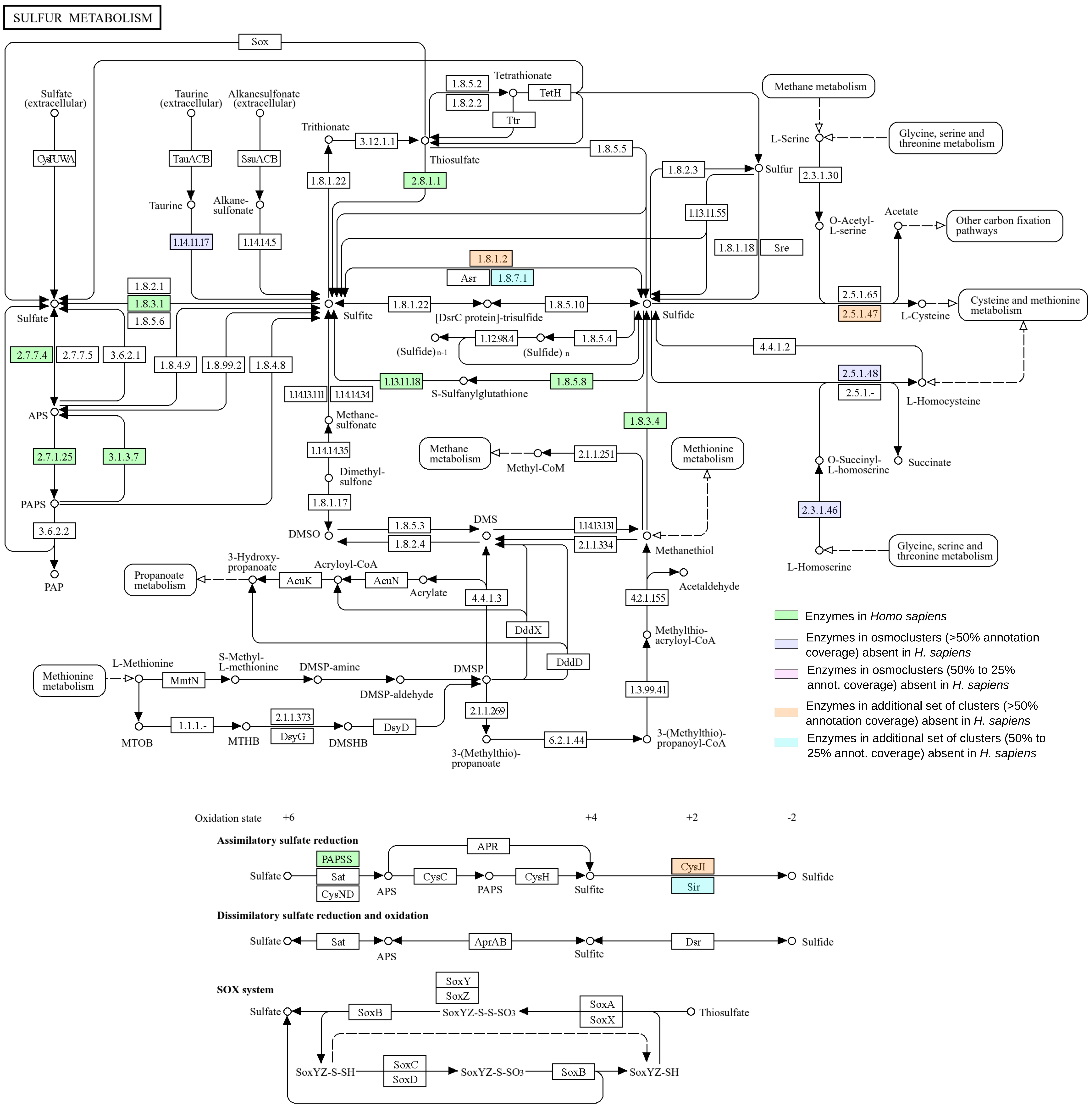

00920 1/22/25
(c) Kanehisa Laboratories

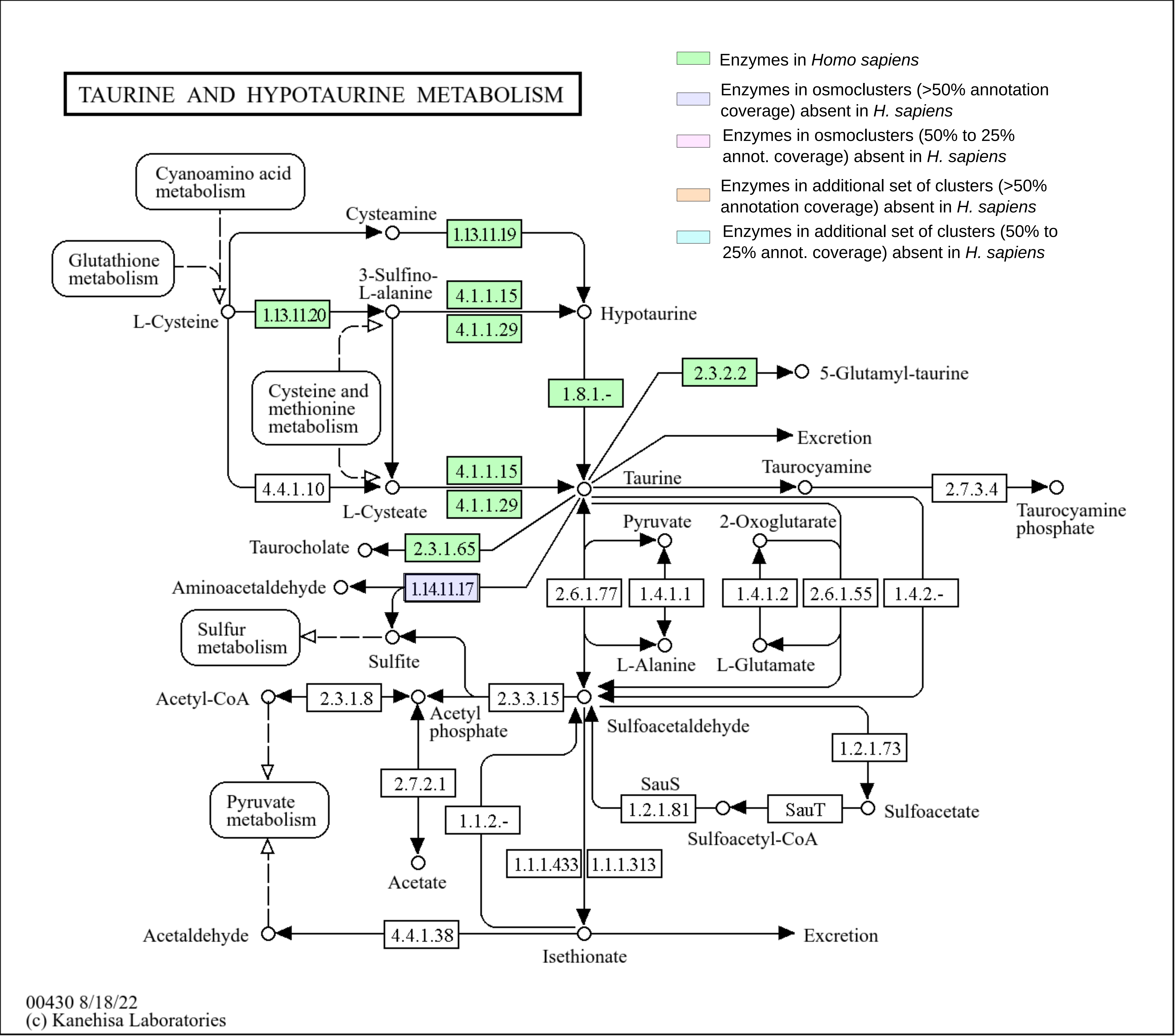

TAURINE AND HYPOTAURINE METABOLISM
Enzymes in *Homo sapiens*
Enzymes in osmoclusters (>50% annotation coverage) absent in *H. sapiens*
Enzymes in osmoclusters (50% to 25% annot. coverage) absent in *H. sapiens*
Enzymes in additional set of clusters (>50% annotation coverage) absent in *H. sapiens*
Enzymes in additional set of clusters (50% to 25% annot. coverage) absent in *H. sapiens*
Cyanoamino acid metabolism
Glutathione metabolism
Cysteamine
1.13.11.19
3-Sulfino-L-alanine
4.1.1.15
4.1.1.29
L-Cysteine
1.13.11.20
Hypotaurine
Cysteine and methionine metabolism
2.3.2.2
5-Glutamyl-taurine
1.8.1.-
Excretion
Taurocyamine
Taurine
4.4.1.10
4.1.1.15
4.1.1.29
2.7.3.4
L-Cysteate
Taurocyamine phosphate
Pyruvate
2-Oxoglutarate
Taurocholate
2.3.1.65
Aminoacetaldehyde
1.14.11.17
2.6.1.77
1.4.1.1
1.4.1.2
2.6.1.55
1.4.2.-
Sulfur metabolism
Sulfite
L-Alanine
L-Glutamate
Acetyl-CoA
2.3.1.8
2.3.3.15
Acetyl phosphate
Sulfoacetaldehyde
1.2.1.73
SauS
Pyruvate metabolism
2.7.2.1
1.1.2.-
1.2.1.81
SauT
Sulfoacetate
Sulfoacetyl-CoA
Acetate
1.1.1.433
1.1.1.313
Acetaldehyde
4.4.1.38
Isethionate
Excretion
00430 8/18/22
(c) Kanehisa Laboratories

Supplementary Information 2

KEGG pathway map representations including enzymes in *Dictyostelium discoideum* and enzymes in the osmoclusters. Referenced in the ‘Osmoclusters: genomic convergence between the osmotrophic groups’ section from the main text

KEGG PATHWAY maps (related to Fig. 2C) were reconstructed using the online tool https://www.genome.jp/kegg/mapper/reconstruct.html. We thank Kanehisa Laboratories for permission to publish results from KEGG and KEGG images (Kanehisa et al. 2000). For this, we uploaded a text file including a concatenation of three lists of KO (KEGG ORTHOLOGY) ids: (1) KO ids that can be confidently be considered to be present among osmoclusters (at least >=50% of sequences from at least one osmocluster were annotated with that KO id), (2) KO ids that for which annotation presence is less certain but likely (at least >=25% of sequences from at least one osmocluster were annotated with that KO id), (3-4) Same as (1) and (2), but for additional set of clusters that are identified as overrepresented in osmotrophic groups compared to background heterotrophic species, but excluding photosynthetic organisms from the background set of species in the overrepresentation tests, and (5) all KO ids annotated to *Dictyostelium discoideum* proteome in the KEGG website on the 24th of February 2025. We used purple, pink, orange, light blue and green color codes for lists 1 to 5, respectively.

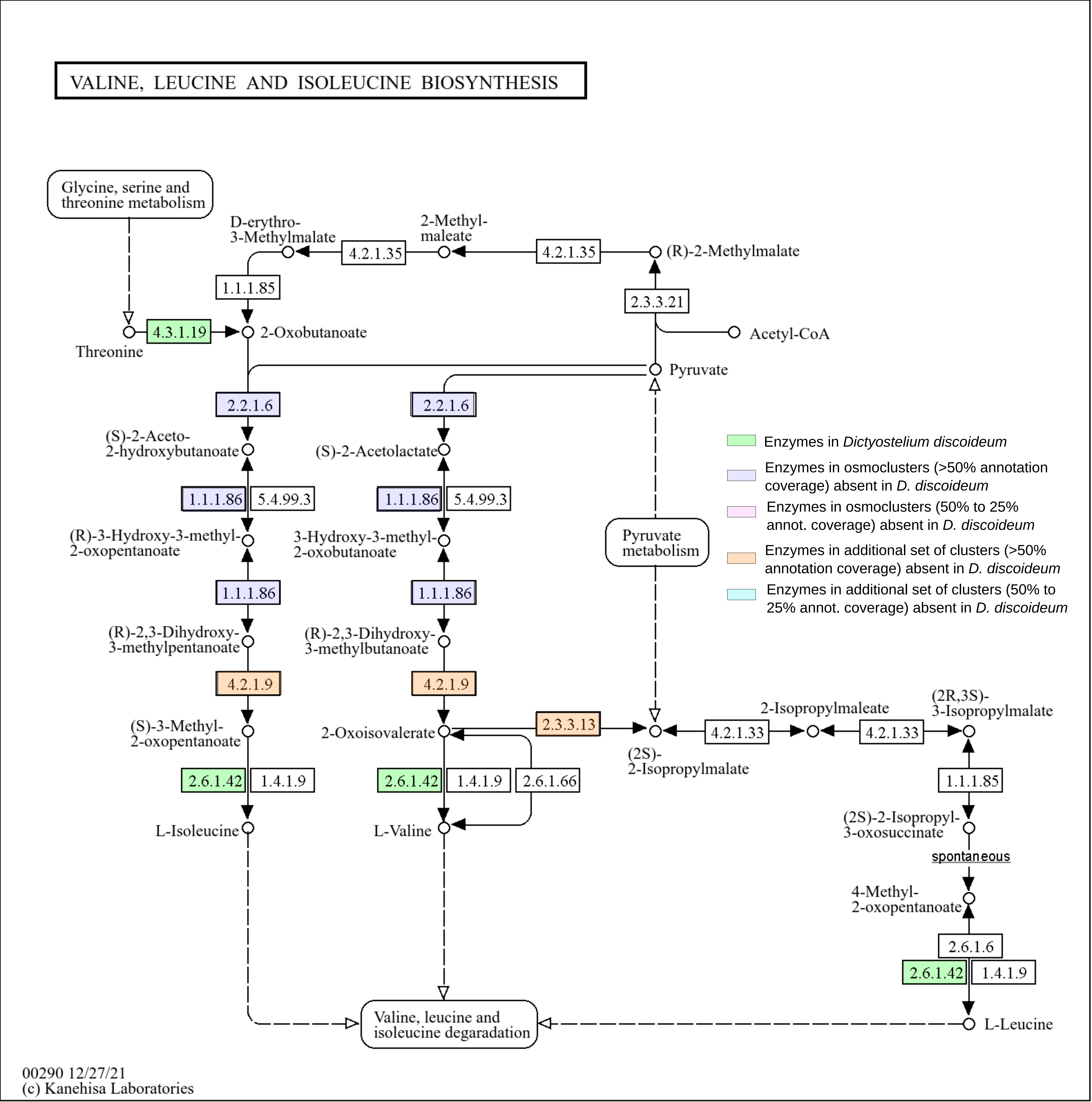
VALINE, LEUCINE AND ISOLEUCINE BIOSYNTHESIS
Glycine, serine and threonine metabolism
D-erythro-3-Methylmalate
4.2.1.35
2-Methyl-maleate
4.2.1.35
(R)-2-Methylmalate
1.1.1.85
2.3.3.21
Threonine
4.3.1.19
2-Oxobutanoate
Acetyl-CoA
Pyruvate
2.2.1.6
2.2.1.6
(S)-2-Aceto-2-hydroxybutanoate
(S)-2-Acetolactate
1.1.1.86
5.4.99.3
1.1.1.86
5.4.99.3
(R)-3-Hydroxy-3-methyl-2-oxopentanoate
3-Hydroxy-3-methyl-2-oxobutanoate
Pyruvate metabolism
1.1.1.86
1.1.1.86
(R)-2,3-Dihydroxy-3-methylpentanoate
(R)-2,3-Dihydroxy-3-methylbutanoate
4.2.1.9
4.2.1.9
(S)-3-Methyl-2-oxopentanoate
2-Oxoisovalerate
2.3.3.13
(2S)-2-Isopropylmalate
4.2.1.33
2-Isopropylmaleate
4.2.1.33
(2R,3S)-3-Isopropylmalate
2.6.1.42
1.4.1.9
2.6.1.42
1.4.1.9
2.6.1.66
1.1.1.85
L-Isoleucine
L-Valine
(2S)-2-Isopropyl-3-oxosuccinate
spontaneous
4-Methyl-2-oxopentanoate
2.6.1.6
2.6.1.42
1.4.1.9
Valine, leucine and isoleucine degaradation
L-Leucine
00290 12/27/21
(c) Kanehisa Laboratories
Enzymes in Dictyostelium discoideum
Enzymes in osmoclusters (>50% annotation coverage) absent in D. discoideum
Enzymes in osmoclusters (50% to 25% annot. coverage) absent in D. discoideum
Enzymes in additional set of clusters (>50% annotation coverage) absent in D. discoideum
Enzymes in additional set of clusters (50% to 25% annot. coverage) absent in D. discoideum

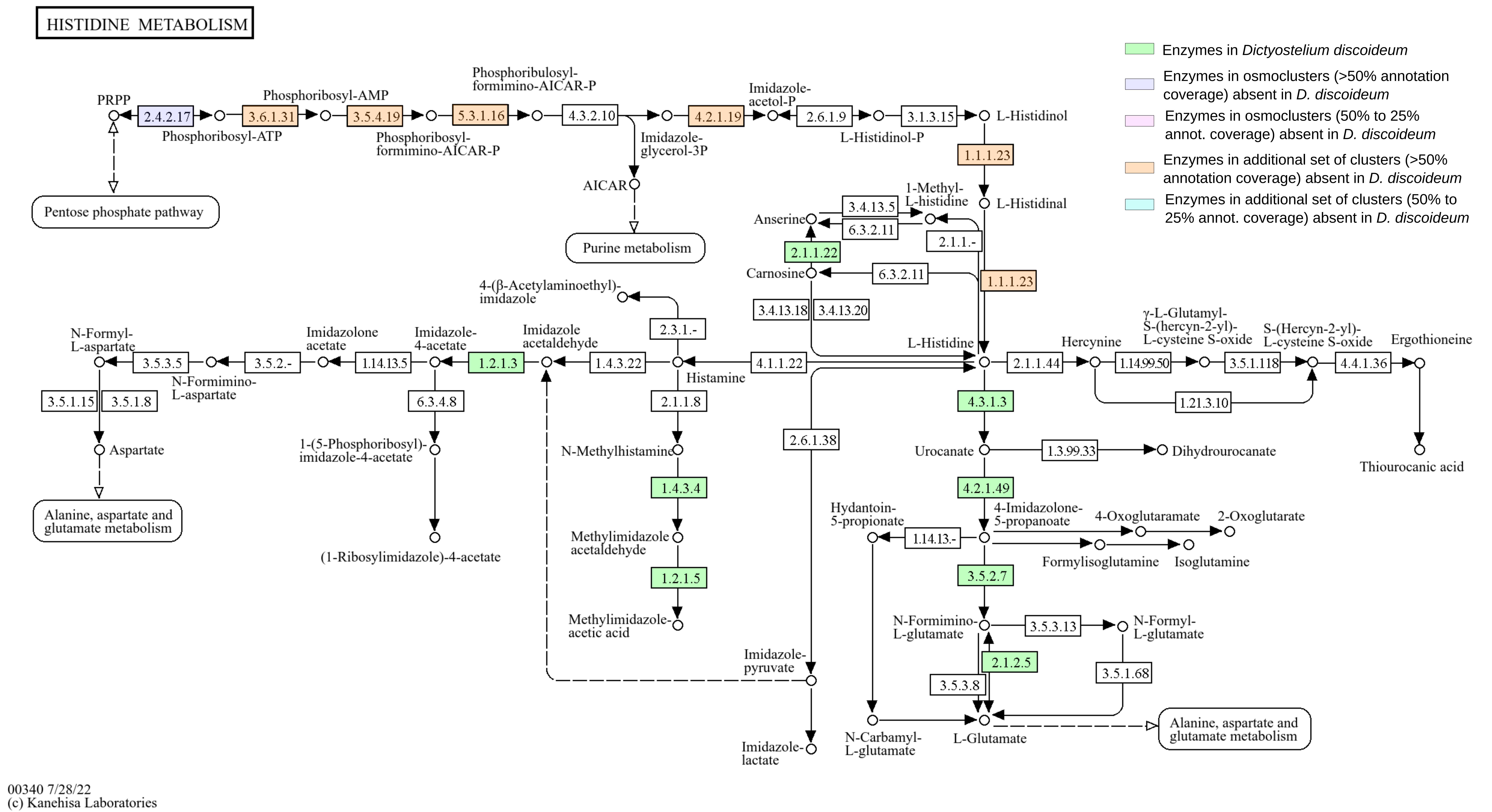
HISTIDINE METABOLISM
Enzymes in Dictyostelium discoideum
Enzymes in osmoclusters (>50% annotation coverage) absent in D. discoideum
Enzymes in osmoclusters (50% to 25% annot. coverage) absent in D. discoideum
Enzymes in additional set of clusters (>50% annotation coverage) absent in D. discoideum
Enzymes in additional set of clusters (50% to 25% annot. coverage) absent in D. discoideum
PRPP
2.4.2.17
Phosphoribosyl-ATP
3.6.1.31
Phosphoribosyl-AMP
3.5.4.19
Phosphoribosyl-formimino-AICAR-P
5.3.1.16
Phosphoribulosyl-formimino-AICAR-P
4.3.2.10
Imidazole-glycerol-3P
4.2.1.19
Imidazole-acetol-P
2.6.1.9
L-Histidinol-P
3.1.3.15
L-Histidinol
1.1.1.23
L-Histidinal
1.1.1.23
AICAR
Purine metabolism
Pentose phosphate pathway
Anserine
3.4.13.5
1-Methyl-L-histidine
6.3.2.11
2.1.1.-
2.1.1.22
Carnosine
6.3.2.11
3.4.13.18
3.4.13.20
4-(β-Acetylaminoethyl)-imidazole
2.3.1.-
L-Histidine
4.1.1.22
Histamine
1.4.3.22
Imidazole acetaldehyde
1.2.1.3
Imidazole-4-acetate
1.14.13.5
Imidazolone acetate
3.5.2.-
N-Formimino-L-aspartate
3.5.3.5
N-Formyl-L-aspartate
3.5.1.15
3.5.1.8
Aspartate
Alanine, aspartate and glutamate metabolism
6.3.4.8
1-(5-Phosphoribosyl)-imidazole-4-acetate
(1-Ribosylimidazole)-4-acetate
2.1.1.8
N-Methylhistamine
1.4.3.4
Methylimidazole acetaldehyde
1.2.1.5
Methylimidazole-acetic acid
2.6.1.38
Imidazole-pyruvate
Imidazole-lactate
2.1.1.44
Hercynine
1.14.99.50
γ-L-Glutamyl-S-(hercyn-2-yl)-L-cysteine S-oxide
3.5.1.118
S-(Hercyn-2-yl)-L-cysteine S-oxide
4.4.1.36
Ergothioneine
1.21.3.10
Thiourocanic acid
4.3.1.3
Urocanate
1.3.99.33
Dihydrourocanate
4.2.1.49
4-Imidazolone-5-propanoate
4-Oxoglutaramate
2-Oxoglutarate
Formylisoglutamine
Isoglutamine
Hydantoin-5-propionate
1.14.13.-
3.5.2.7
N-Formimino-L-glutamate
3.5.3.13
N-Formyl-L-glutamate
2.1.2.5
3.5.3.8
3.5.1.68
N-Carbamyl-L-glutamate
L-Glutamate
Alanine, aspartate and glutamate metabolism
00340 7/28/22
(c) Kanehisa Laboratories

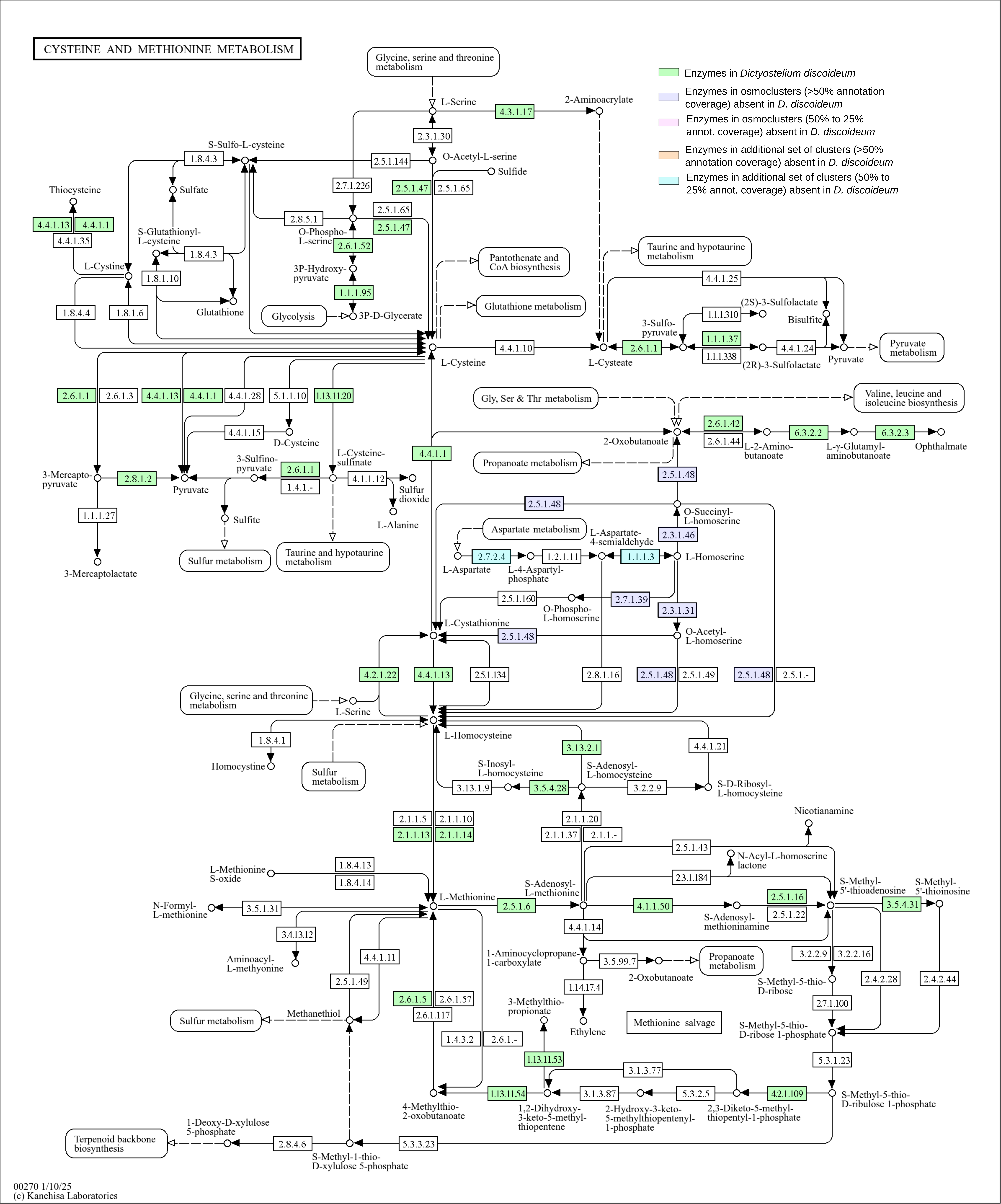

CYSTEINE AND METHIONINE METABOLISM
Enzymes in Dictyostelium discoideum
Enzymes in osmoclusters (>50% annotation coverage) absent in D. discoideum
Enzymes in osmoclusters (50% to 25% annot. coverage) absent in D. discoideum
Enzymes in additional set of clusters (>50% annotation coverage) absent in D. discoideum
Enzymes in additional set of clusters (50% to 25% annot. coverage) absent in D. discoideum
Glycine, serine and threonine metabolism
L-Serine
4.3.1.17
2-Aminoacrylate
2.3.1.30
O-Acetyl-L-serine
Sulfide
S-Sulfo-L-cysteine
1.8.4.3
2.5.1.144
2.7.1.226
2.5.1.47
2.5.1.65
Thiocysteine
Sulfate
4.4.1.13
4.4.1.1
4.4.1.35
2.8.5.1
2.5.1.65
2.5.1.47
S-Glutathionyl-L-cysteine
O-Phospho-L-serine
2.6.1.52
L-Cystine
1.8.4.3
3P-Hydroxy-pyruvate
Pantothenate and CoA biosynthesis
Taurine and hypotaurine metabolism
1.8.1.10
1.1.1.95
4.4.1.25
Glutathione
Glutathione metabolism
(2S)-3-Sulfolactate
1.8.4.4
1.8.1.6
Glycolysis
3P-D-Glycerate
1.1.1.310
3-Sulfo-pyruvate
Bisulfite
1.1.1.37
4.4.1.10
2.6.1.1
4.4.1.24
Pyruvate metabolism
L-Cysteine
L-Cysteate
1.1.1.338
(2R)-3-Sulfolactate
Pyruvate
2.6.1.1
2.6.1.3
4.4.1.13
4.4.1.1
4.4.1.28
5.1.1.10
1.13.11.20
Gly, Ser & Thr metabolism
Valine, leucine and isoleucine biosynthesis
4.4.1.15
D-Cysteine
2.6.1.42
6.3.2.2
6.3.2.3
2.6.1.44
2-Oxobutanoate
L-2-Amino-butanoate
L-γ-Glutamyl-aminobutanoate
Ophthalmate
4.4.1.1
3-Sulfino-pyruvate
L-Cysteine-sulfinate
Propanoate metabolism
3-Mercapto-pyruvate
2.8.1.2
Pyruvate
2.6.1.1
4.1.1.12
1.4.1.-
Sulfur dioxide
2.5.1.48
2.5.1.48
O-Succinyl-L-homoserine
1.1.1.27
Sulfite
L-Alanine
Aspartate metabolism
L-Aspartate-4-semialdehyde
2.3.1.46
Sulfur metabolism
Taurine and hypotaurine metabolism
2.7.2.4
1.2.1.11
1.1.1.3
L-Homoserine
3-Mercaptolactate
L-Aspartate
L-4-Aspartyl-phosphate
2.5.1.160
2.7.1.39
O-Phospho-L-homoserine
2.3.1.31
L-Cystathionine
O-Acetyl-L-homoserine
2.5.1.48
4.2.1.22
4.4.1.13
2.5.1.134
2.8.1.16
2.5.1.48
2.5.1.49
2.5.1.48
2.5.1.-
Glycine, serine and threonine metabolism
L-Serine
L-Homocysteine
1.8.4.1
3.13.2.1
4.4.1.21
Homocystine
Sulfur metabolism
S-Inosyl-L-homocysteine
S-Adenosyl-L-homocysteine
3.13.1.9
3.5.4.28
3.2.2.9
S-D-Ribosyl-L-homocysteine
2.1.1.5
2.1.1.10
2.1.1.20
Nicotianamine
2.1.1.13
2.1.1.14
2.1.1.37
2.1.1.-
2.5.1.43
N-Acyl-L-homoserine lactone
L-Methionine S-oxide
1.8.4.13
1.8.4.14
2.3.1.184
S-Adenosyl-L-methionine
S-Methyl-5'-thioadenosine
S-Methyl-5'-thioinosine
N-Formyl-L-methionine
3.5.1.31
L-Methionine
2.5.1.6
4.1.1.50
2.5.1.16
2.5.1.22
3.5.4.31
S-Adenosyl-methioninamine
3.4.13.12
4.4.1.14
Aminoacyl-L-methyonine
4.4.1.11
1-Aminocyclopropane-1-carboxylate
3.5.99.7
Propanoate metabolism
2-Oxobutanoate
3.2.2.9
3.2.2.16
2.5.1.49
S-Methyl-5-thio-D-ribose
2.4.2.28
2.4.2.44
1.14.17.4
2.6.1.5
2.6.1.57
2.6.1.117
3-Methylthio-propionate
2.7.1.100
Sulfur metabolism
Methanethiol
Ethylene
Methionine salvage
S-Methyl-5-thio-D-ribose 1-phosphate
1.4.3.2
2.6.1.-
1.13.11.53
5.3.1.23
3.1.3.77
1.13.11.54
3.1.3.87
5.3.2.5
4.2.1.109
S-Methyl-5-thio-D-ribulose 1-phosphate
4-Methylthio-2-oxobutanoate
1,2-Dihydroxy-3-keto-5-methyl-thiopentene
2-Hydroxy-3-keto-5-methylthiopentenyl-1-phosphate
2,3-Diketo-5-methyl-thiopentyl-1-phosphate
1-Deoxy-D-xylulose 5-phosphate
Terpenoid backbone biosynthesis
2.8.4.6
5.3.3.23
S-Methyl-1-thio-D-xylulose 5-phosphate
00270 1/10/25
(c) Kanehisa Laboratories

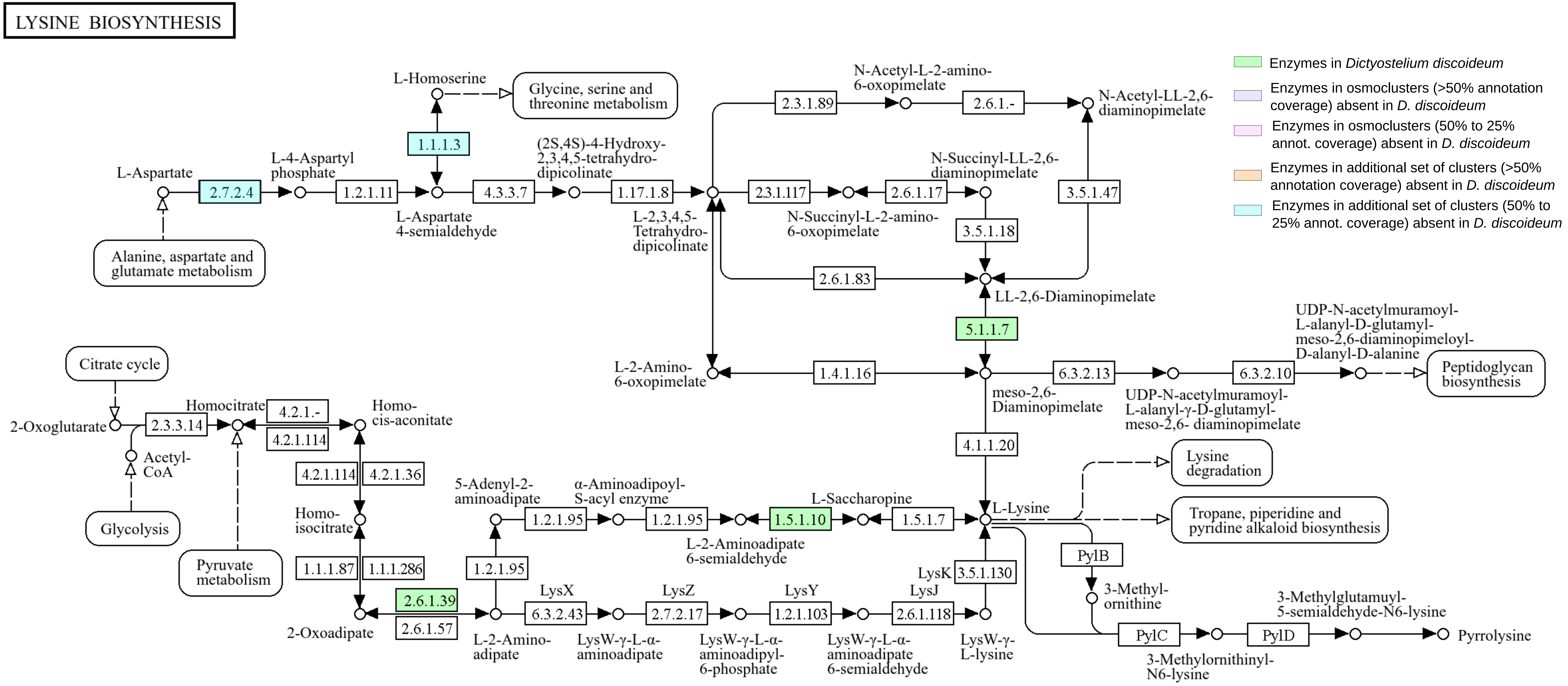

00300 7/29/22
(c) Kanehisa Laboratories

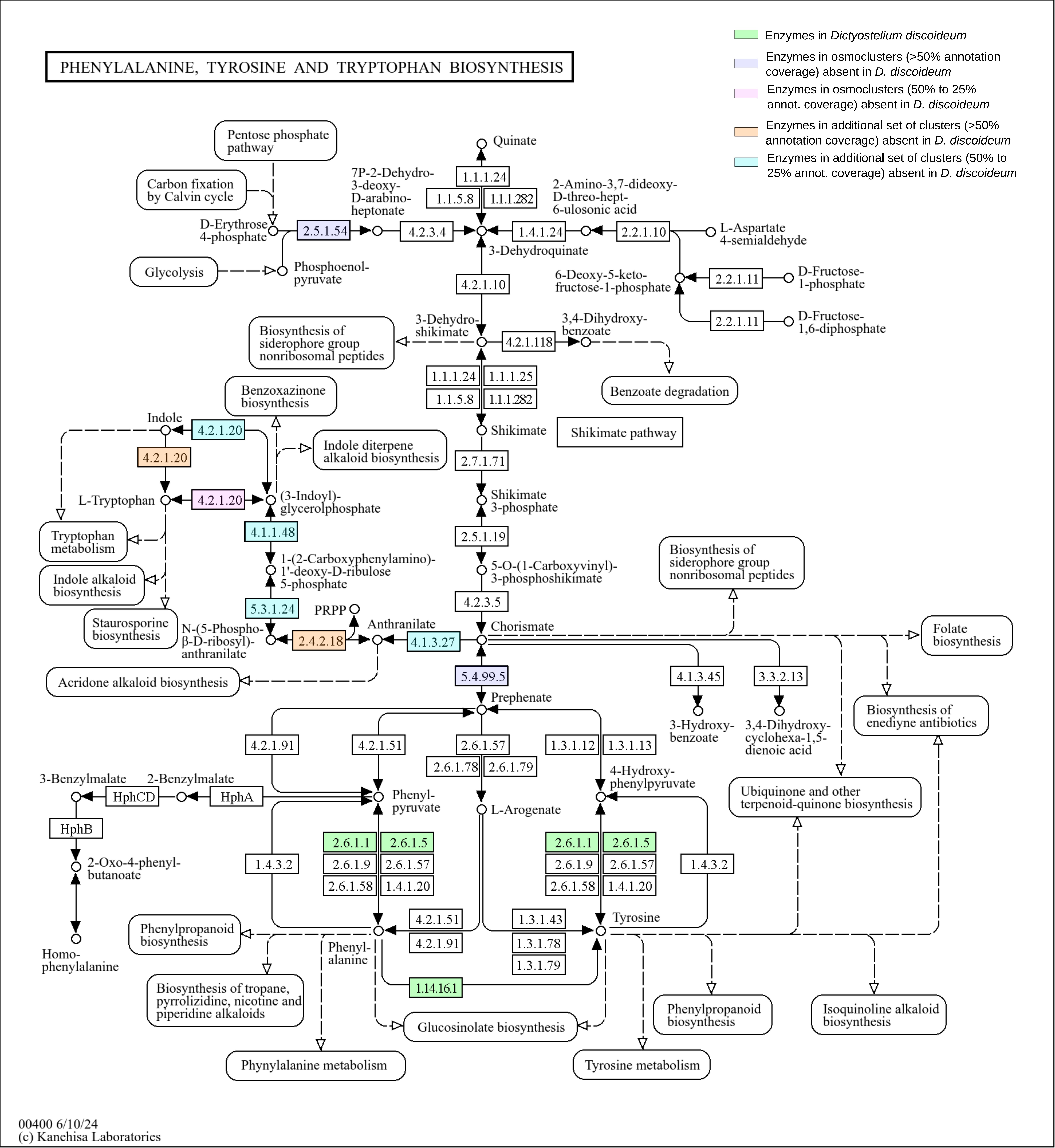

Enzymes in Dictyostelium discoideum
Enzymes in osmoclusters (>50% annotation coverage) absent in D. discoideum
Enzymes in osmoclusters (50% to 25% annot. coverage) absent in D. discoideum
Enzymes in additional set of clusters (>50% annotation coverage) absent in D. discoideum
Enzymes in additional set of clusters (50% to 25% annot. coverage) absent in D. discoideum
PHENYLALANINE, TYROSINE AND TRYPTOPHAN BIOSYNTHESIS
Pentose phosphate pathway
Carbon fixation by Calvin cycle
Glycolysis
D-Erythrose 4-phosphate
Phosphoenol-pyruvate
2.5.1.54
7P-2-Dehydro-3-deoxy-D-arabino-heptonate
4.2.3.4
Quinate
1.1.1.24
1.1.5.8
1.1.1.282
3-Dehydroquinate
1.4.1.24
2-Amino-3,7-dideoxy-D-threo-hept-6-ulosonic acid
2.2.1.10
L-Aspartate 4-semialdehyde
6-Deoxy-5-keto-fructose-1-phosphate
2.2.1.11
D-Fructose-1-phosphate
2.2.1.11
D-Fructose-1,6-diphosphate
4.2.1.10
3-Dehydro-shikimate
Biosynthesis of siderophore group nonribosomal peptides
4.2.1.118
3,4-Dihydroxy-benzoate
Benzoate degradation
1.1.1.24
1.1.1.25
1.1.5.8
1.1.1.282
Shikimate
Shikimate pathway
2.7.1.71
Shikimate 3-phosphate
2.5.1.19
5-O-(1-Carboxyvinyl)-3-phosphoshikimate
4.2.3.5
Chorismate
Benzoxazinone biosynthesis
Indole diterpene alkaloid biosynthesis
Indole
4.2.1.20
4.2.1.20
L-Tryptophan
4.2.1.20
(3-Indoyl)-glycerolphosphate
Tryptophan metabolism
Indole alkaloid biosynthesis
Staurosporine biosynthesis
4.1.1.48
1-(2-Carboxyphenylamino)-1'-deoxy-D-ribulose 5-phosphate
5.3.1.24
PRPP
N-(5-Phospho-β-D-ribosyl)-anthranilate
2.4.2.18
Anthranilate
4.1.3.27
Acridone alkaloid biosynthesis
Biosynthesis of siderophore group nonribosomal peptides
Folate biosynthesis
5.4.99.5
4.1.3.45
3.3.2.13
Prephenate
3-Hydroxy-benzoate
3,4-Dihydroxy-cyclohexa-1,5-dienoic acid
Biosynthesis of enediyne antibiotics
4.2.1.91
4.2.1.51
2.6.1.57
2.6.1.78
2.6.1.79
1.3.1.12
1.3.1.13
4-Hydroxy-phenylpyruvate
Ubiquinone and other terpenoid-quinone biosynthesis
3-Benzylmalate
2-Benzylmalate
HphCD
HphA
HphB
Phenyl-pyruvate
L-Arogenate
2-Oxo-4-phenyl-butanoate
Homo-phenylalanine
2.6.1.1
2.6.1.5
2.6.1.9
2.6.1.57
2.6.1.58
1.4.1.20
1.4.3.2
2.6.1.1
2.6.1.5
2.6.1.9
2.6.1.57
2.6.1.58
1.4.1.20
1.4.3.2
4.2.1.51
4.2.1.91
1.3.1.43
1.3.1.78
1.3.1.79
Tyrosine
Phenylpropanoid biosynthesis
Phenyl-alanine
1.14.16.1
Biosynthesis of tropane, pyrrolizidine, nicotine and piperidine alkaloids
Glucosinolate biosynthesis
Phenylpropanoid biosynthesis
Isoquinoline alkaloid biosynthesis
Phynylalanine metabolism
Tyrosine metabolism
00400 6/10/24
(c) Kanehisa Laboratories

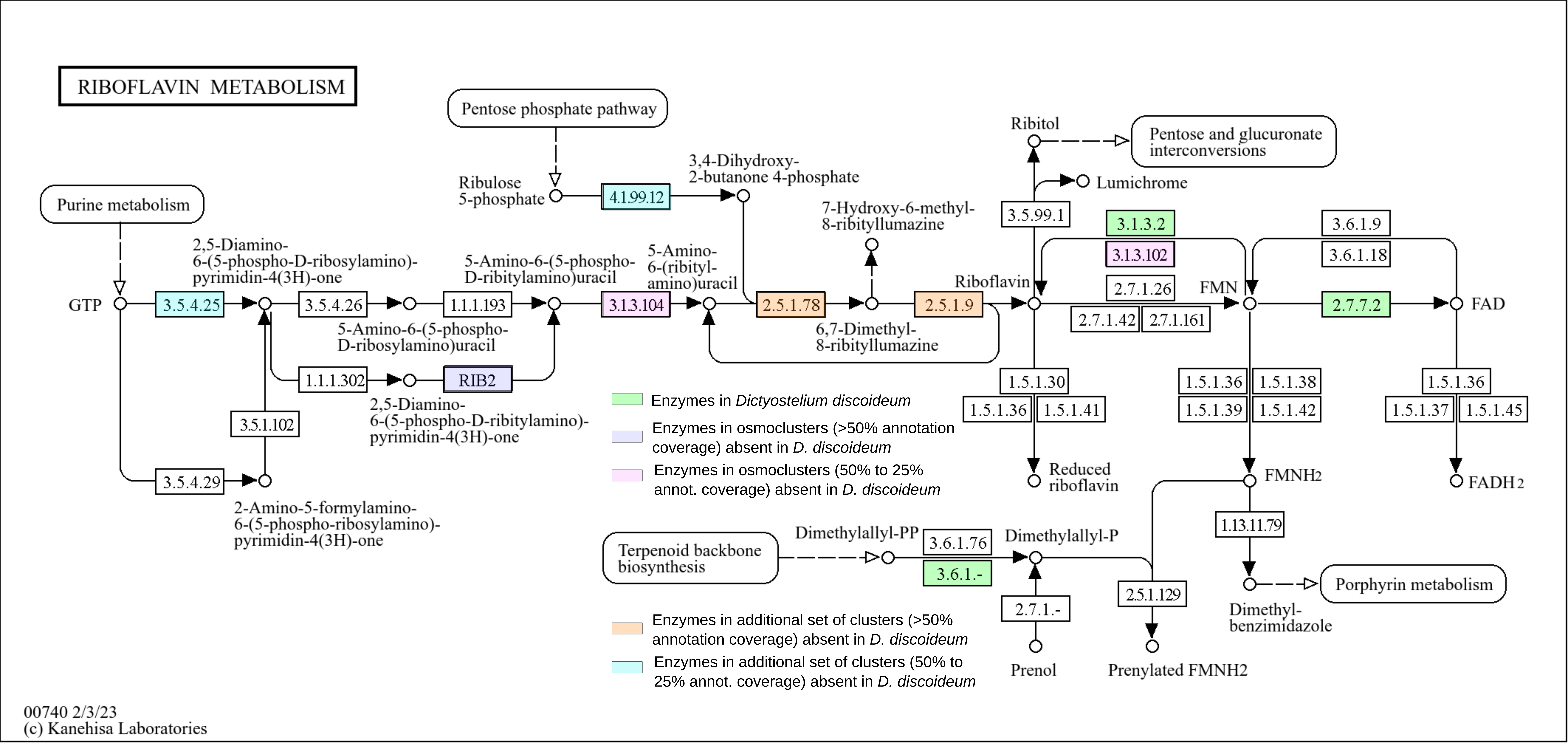

RIBOFLAVIN METABOLISM
Pentose phosphate pathway
Purine metabolism
Ribulose 5-phosphate
4.1.99.12
3,4-Dihydroxy-2-butanone 4-phosphate
7-Hydroxy-6-methyl-8-ribityllumazine
Ribitol
Pentose and glucuronate interconversions
Lumichrome
3.5.99.1
3.1.3.2
3.1.3.102
3.6.1.9
3.6.1.18
2,5-Diamino-6-(5-phospho-D-ribosylamino)-pyrimidin-4(3H)-one
5-Amino-6-(5-phospho-D-ribitylamino)uracil
5-Amino-6-(ribitylamino)uracil
Riboflavin
FMN
FAD
GTP
3.5.4.25
3.5.4.26
1.1.1.193
3.1.3.104
2.5.1.78
2.5.1.9
2.7.1.26
2.7.1.42
2.7.1.161
2.7.7.2
5-Amino-6-(5-phospho-D-ribosylamino)uracil
6,7-Dimethyl-8-ribityllumazine
1.1.1.302
RIB2
2,5-Diamino-6-(5-phospho-D-ribitylamino)-pyrimidin-4(3H)-one
3.5.1.102
3.5.4.29
2-Amino-5-formylamino-6-(5-phospho-ribosylamino)-pyrimidin-4(3H)-one
1.5.1.30
1.5.1.36
1.5.1.41
Reduced riboflavin
1.5.1.36
1.5.1.38
1.5.1.39
1.5.1.42
FMNH2
1.5.1.36
1.5.1.37
1.5.1.45
FADH2
Enzymes in Dictyostelium discoideum
Enzymes in osmoclusters (>50% annotation coverage) absent in D. discoideum
Enzymes in osmoclusters (50% to 25% annot. coverage) absent in D. discoideum
Terpenoid backbone biosynthesis
Dimethylallyl-PP
3.6.1.76
3.6.1.-
Dimethylallyl-P
2.7.1.-
Prenol
2.5.1.129
Prenylated FMNH2
1.13.11.79
Dimethyl-benzimidazole
Porphyrin metabolism
Enzymes in additional set of clusters (>50% annotation coverage) absent in D. discoideum
Enzymes in additional set of clusters (50% to 25% annot. coverage) absent in D. discoideum
00740 2/3/23
(c) Kanehisa Laboratories

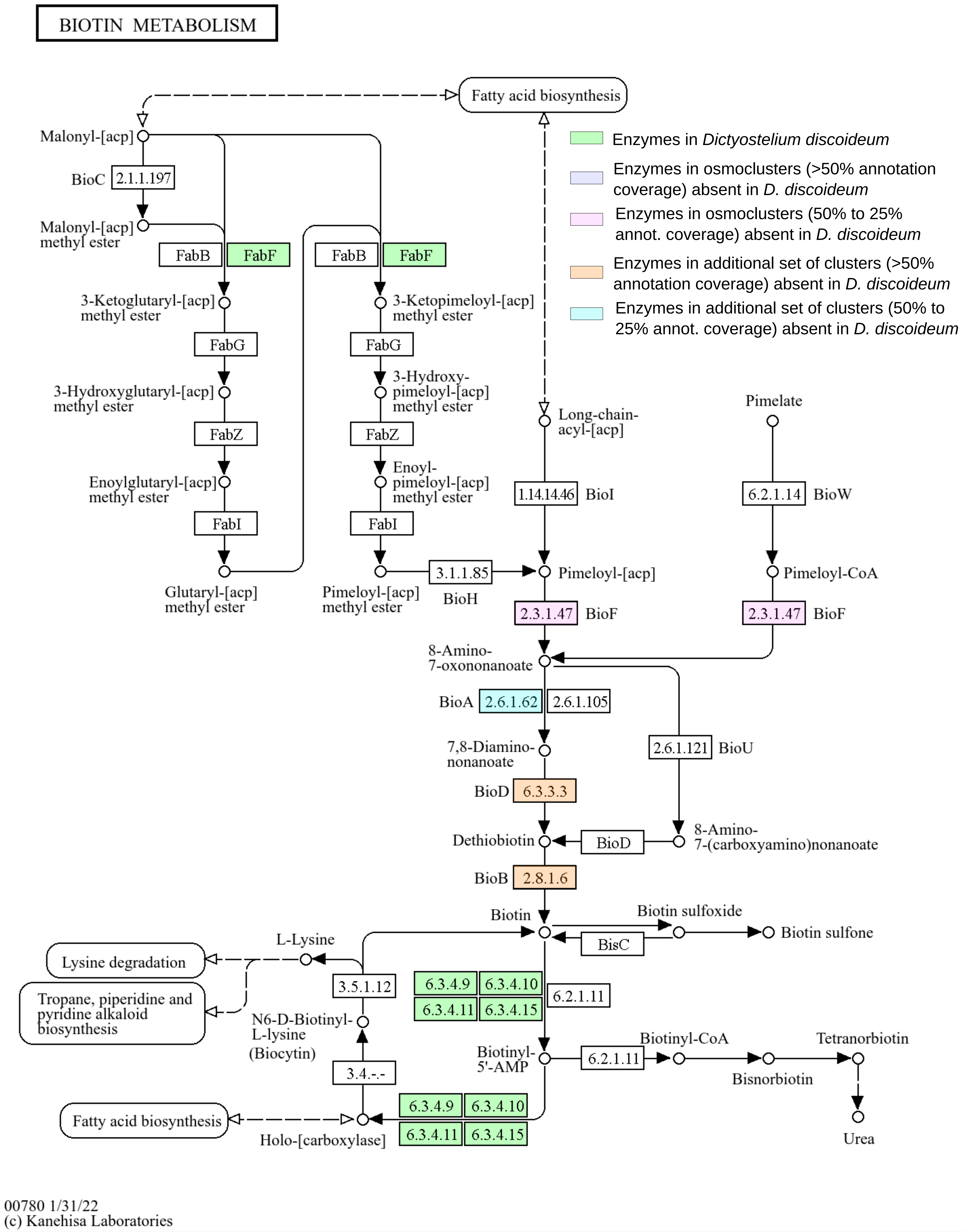
BIOTIN METABOLISM
Fatty acid biosynthesis
Enzymes in Dictyostelium discoideum
Enzymes in osmoclusters (>50% annotation coverage) absent in D. discoideum
Enzymes in osmoclusters (50% to 25% annot. coverage) absent in D. discoideum
Enzymes in additional set of clusters (>50% annotation coverage) absent in D. discoideum
Enzymes in additional set of clusters (50% to 25% annot. coverage) absent in D. discoideum
Malonyl-[acp]
BioC
2.1.1.197
Malonyl-[acp] methyl ester
FabB
FabF
FabB
FabF
3-Ketoglutaryl-[acp] methyl ester
3-Ketopimeloyl-[acp] methyl ester
FabG
FabG
3-Hydroxyglutaryl-[acp] methyl ester
3-Hydroxy-pimeloyl-[acp] methyl ester
FabZ
FabZ
Enoylglutaryl-[acp] methyl ester
Enoyl-pimeloyl-[acp] methyl ester
FabI
FabI
Glutaryl-[acp] methyl ester
Pimeloyl-[acp] methyl ester
3.1.1.85
BioH
Long-chain-acyl-[acp]
1.14.14.46
BioI
Pimelate
6.2.1.14
BioW
Pimeloyl-[acp]
Pimeloyl-CoA
2.3.1.47
BioF
2.3.1.47
BioF
8-Amino-7-oxononanoate
BioA
2.6.1.62
2.6.1.105
7,8-Diamino-nonanoate
2.6.1.121
BioU
BioD
6.3.3.3
Dethiobiotin
BioD
8-Amino-7-(carboxyamino)nonanoate
BioB
2.8.1.6
Biotin
Biotin sulfoxide
Biotin sulfone
BisC
L-Lysine
Lysine degradation
Tropane, piperidine and pyridine alkaloid biosynthesis
3.5.1.12
6.3.4.9
6.3.4.10
6.3.4.11
6.3.4.15
6.2.1.11
N6-D-Biotinyl-L-lysine (Biocytin)
3.4.-.-
Biotinyl-5'-AMP
6.2.1.11
Biotinyl-CoA
Bisnorbiotin
Tetranorbiotin
Urea
Fatty acid biosynthesis
Holo-[carboxylase]
6.3.4.9
6.3.4.10
6.3.4.11
6.3.4.15
00780 1/31/22
(c) Kanehisa Laboratories

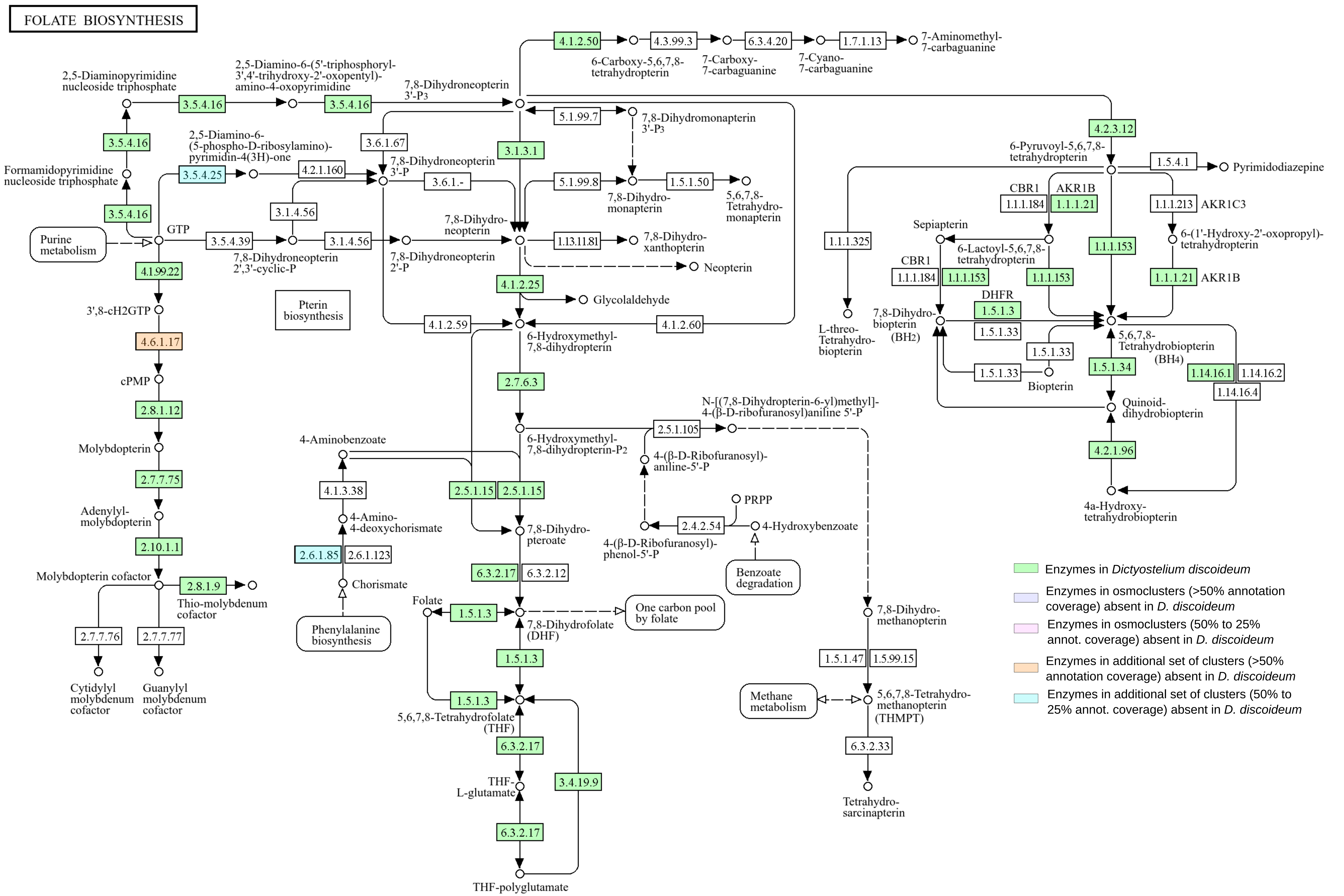

00790 1/27/22

(c) Kanehisa Laboratories

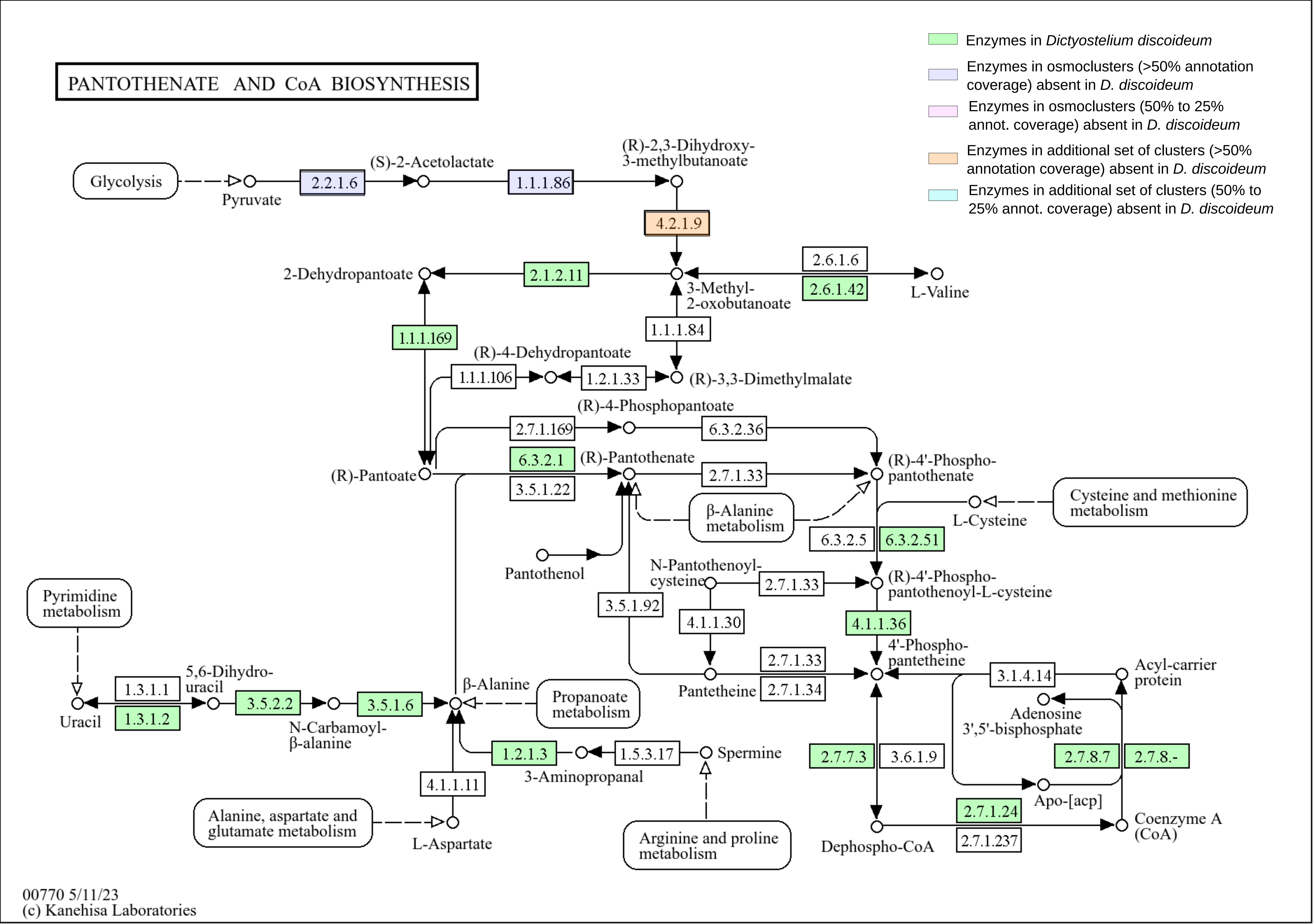

PANTOTHENATE AND CoA BIOSYNTHESIS
Enzymes in Dictyostelium discoideum
Enzymes in osmoclusters (>50% annotation coverage) absent in D. discoideum
Enzymes in osmoclusters (50% to 25% annot. coverage) absent in D. discoideum
Enzymes in additional set of clusters (>50% annotation coverage) absent in D. discoideum
Enzymes in additional set of clusters (50% to 25% annot. coverage) absent in D. discoideum
Glycolysis
Pyruvate
2.2.1.6
(S)-2-Acetolactate
1.1.1.86
(R)-2,3-Dihydroxy-3-methylbutanoate
4.2.1.9
2-Dehydropantoate
2.1.2.11
3-Methyl-2-oxobutanoate
2.6.1.6
2.6.1.42
L-Valine
1.1.1.169
1.1.1.84
(R)-4-Dehydropantoate
1.1.1.106
1.2.1.33
(R)-3,3-Dimethylmalate
(R)-4-Phosphopantoate
2.7.1.169
6.3.2.36
6.3.2.1
(R)-Pantothenate
(R)-Pantoate
3.5.1.22
2.7.1.33
(R)-4'-Phosphopantothenate
β-Alanine metabolism
Cysteine and methionine metabolism
L-Cysteine
6.3.2.5
6.3.2.51
Pantothenol
N-Pantothenoyl-cysteine
2.7.1.33
(R)-4'-Phosphopantothenoyl-L-cysteine
3.5.1.92
4.1.1.30
4.1.1.36
4'-Phosphopantetheine
Pyrimidine metabolism
2.7.1.33
Pantetheine
2.7.1.34
3.1.4.14
Acyl-carrier protein
1.3.1.1
5,6-Dihydrouracil
β-Alanine
Propanoate metabolism
Uracil
1.3.1.2
3.5.2.2
N-Carbamoyl-β-alanine
3.5.1.6
Adenosine 3',5'-bisphosphate
1.2.1.3
1.5.3.17
Spermine
3-Aminopropanal
2.7.7.3
3.6.1.9
2.7.8.7
2.7.8.-
4.1.1.11
Apo-[acp]
Alanine, aspartate and glutamate metabolism
L-Aspartate
Arginine and proline metabolism
2.7.1.24
Dephospho-CoA
2.7.1.237
Coenzyme A (CoA)
00770 5/11/23
(c) Kanehisa Laboratories

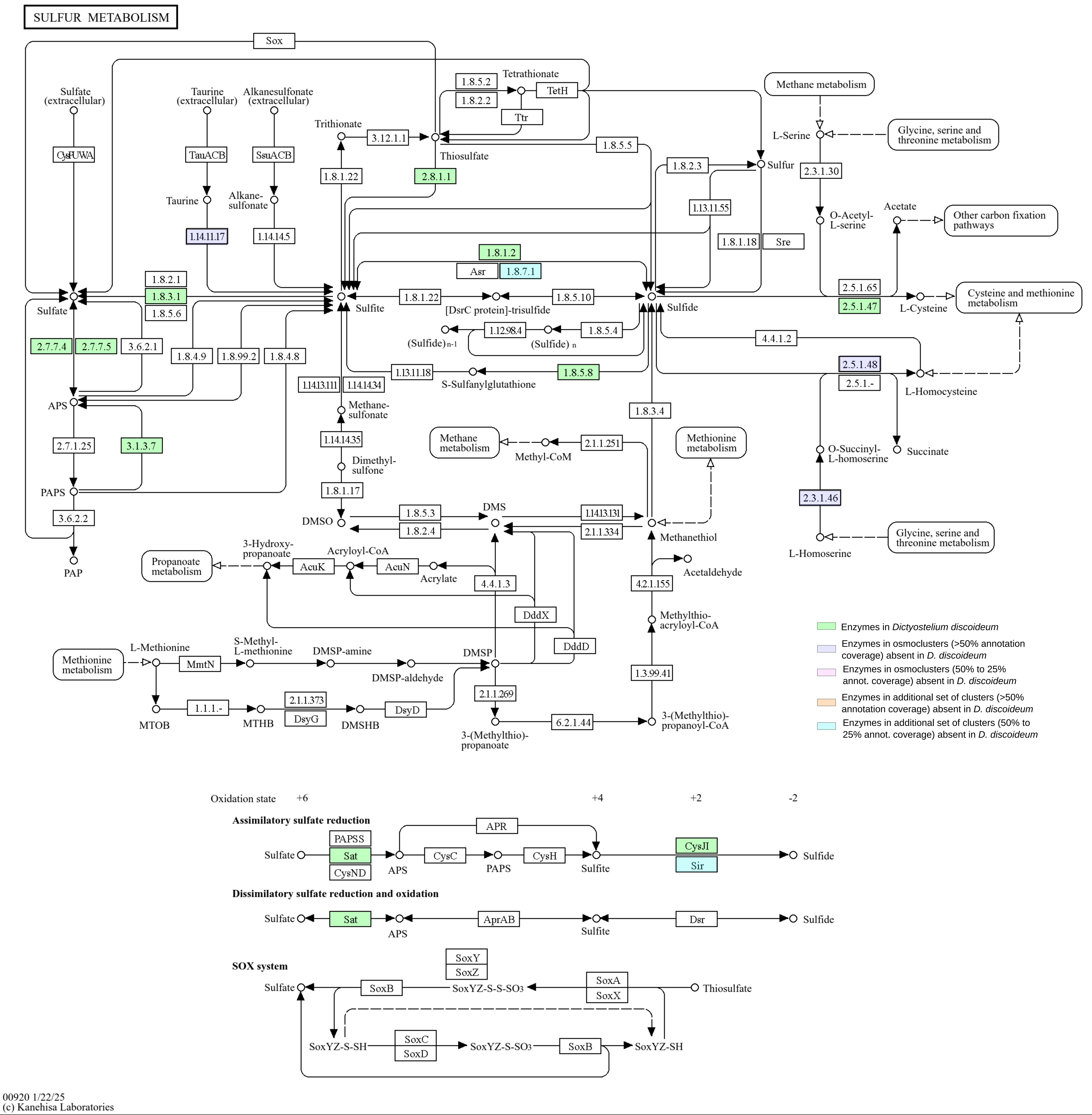
SULFUR METABOLISM
Sox
Sulfate (extracellular)
Taurine (extracellular)
Alkanesulfonate (extracellular)
CysPUWA
TauACB
SsuACB
Taurine
Alkane-sulfonate
1.14.11.17
1.14.14.5
Tetrathionate
1.8.5.2
1.8.2.2
TetH
Ttr
Trithionate
3.12.1.1
Thiosulfate
2.8.1.1
1.8.1.22
1.8.5.5
1.8.2.3
Sulfur
1.13.11.55
1.8.1.18
Sre
Methane metabolism
L-Serine
Glycine, serine and threonine metabolism
2.3.1.30
O-Acetyl-L-serine
Acetate
Other carbon fixation pathways
1.8.1.2
Asr
1.8.7.1
1.8.2.1
1.8.3.1
1.8.5.6
Sulfate
Sulfite
1.8.1.22
[DsrC protein]-trisulfide
1.8.5.10
Sulfide
2.5.1.65
2.5.1.47
L-Cysteine
Cysteine and methionine metabolism
2.7.7.4
2.7.7.5
3.6.2.1
1.8.4.9
1.8.99.2
1.8.4.8
(Sulfide)n-1
1.12.98.4
(Sulfide) n
1.8.5.4
4.4.1.2
1.13.11.18
S-Sulfanylglutathione
1.8.5.8
2.5.1.48
2.5.1.-
L-Homocysteine
1.14.13.111
1.14.14.34
Methane-sulfonate
APS
1.8.3.4
2.7.1.25
3.1.3.7
1.14.14.35
Methane metabolism
Methyl-CoM
2.1.1.251
Methionine metabolism
O-Succinyl-L-homoserine
Succinate
Dimethyl-sulfone
PAPS
1.8.1.17
2.3.1.46
DMSO
1.8.5.3
1.8.2.4
DMS
1.14.13.131
2.1.1.334
Methanethiol
3.6.2.2
PAP
L-Homoserine
Glycine, serine and threonine metabolism
3-Hydroxy-propanoate
Propanoate metabolism
Acryloyl-CoA
AcuK
AcuN
Acrylate
4.4.1.3
4.2.1.155
Acetaldehyde
DddX
Methylthio-acryloyl-CoA
DddD
Methionine metabolism
L-Methionine
S-Methyl-L-methionine
DMSP-amine
DMSP
MmtN
DMSP-aldehyde
1.3.99.41
MTOB
1.1.1.-
MTHB
2.1.1.373
DsyG
DMSHB
DsyD
2.1.1.269
3-(Methylthio)-propanoate
6.2.1.44
3-(Methylthio)-propanoyl-CoA
Enzymes in *Dictyostelium discoideum*
Enzymes in osmoclusters (>50% annotation coverage) absent in *D. discoideum*
Enzymes in osmoclusters (50% to 25% annot. coverage) absent in *D. discoideum*
Enzymes in additional set of clusters (>50% annotation coverage) absent in *D. discoideum*
Enzymes in additional set of clusters (50% to 25% annot. coverage) absent in *D. discoideum*
Oxidation state +6 +4 +2 -2
Assimilatory sulfate reduction
Sulfate
PAPSS
Sat
CysND
APS
APR
CysC
PAPS
CysH
Sulfite
CysJI
Sir
Sulfide
Dissimilatory sulfate reduction and oxidation
Sulfate
Sat
APS
AprAB
Sulfite
Dsr
Sulfide
SOX system
Sulfate
SoxB
SoxY
SoxZ
SoxYZ-S-S-SO3
SoxA
SoxX
Thiosulfate
SoxYZ-S-SH
SoxC
SoxD
SoxYZ-S-SO3
SoxB
SoxYZ-SH
00920 1/22/25
(c) Kanehisa Laboratories

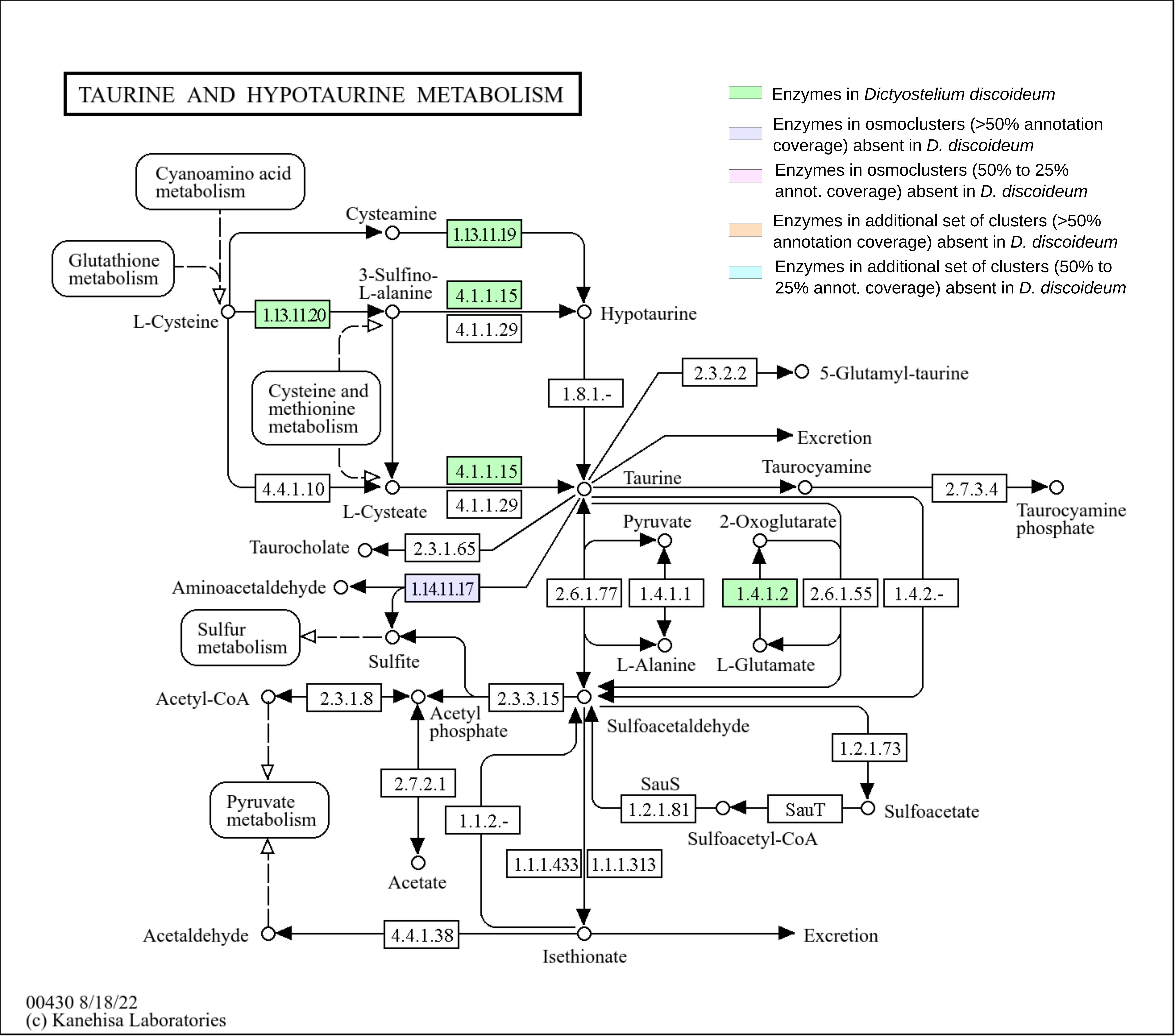
TAURINE AND HYPOTAURINE METABOLISM
Enzymes in Dictyostelium discoideum
Enzymes in osmoclusters (>50% annotation coverage) absent in D. discoideum
Enzymes in osmoclusters (50% to 25% annot. coverage) absent in D. discoideum
Enzymes in additional set of clusters (>50% annotation coverage) absent in D. discoideum
Enzymes in additional set of clusters (50% to 25% annot. coverage) absent in D. discoideum
Cyanoamino acid metabolism
Glutathione metabolism
Cysteamine
1.13.11.19
L-Cysteine
1.13.11.20
3-Sulfino-L-alanine
4.1.1.15
4.1.1.29
Hypotaurine
Cysteine and methionine metabolism
1.8.1.-
2.3.2.2
5-Glutamyl-taurine
Excretion
4.4.1.10
L-Cysteate
4.1.1.15
4.1.1.29
Taurine
Taurocyamine
2.7.3.4
Taurocyamine phosphate
Taurocholate
2.3.1.65
Pyruvate
2-Oxoglutarate
Aminoacetaldehyde
1.14.11.17
2.6.1.77
1.4.1.1
1.4.1.2
2.6.1.55
1.4.2.-
Sulfur metabolism
Sulfite
L-Alanine
L-Glutamate
Acetyl-CoA
2.3.1.8
Acetyl phosphate
2.3.3.15
Sulfoacetaldehyde
1.2.1.73
Pyruvate metabolism
2.7.2.1
SauS
1.2.1.81
SauT
Sulfoacetate
1.1.2.-
Sulfoacetyl-CoA
Acetate
1.1.1.433
1.1.1.313
Acetaldehyde
4.4.1.38
Isethionate
Excretion
00430 8/18/22
(c) Kanehisa Laboratories

Supplementary Information 3

Report of the positive cases identified in the supervised inspection of putative HGT patterns in osmoclusters. Referenced in the ‘Horizontal gene transfers in osmoclusters’ section from the main text

See ‘Manually supervised identification of osmoclusters with putative HGT patterns’ section in Material and methods for further details

Instructions to understand these slides

- Each slide represents an osmocluster showing signatures of Horizontal Gene Transfer (HGT) events involving at least some of the osmogroups and/or some of its/their ancestor/s along the evolutionary history of the gene family represented by the osmoclusters
  - “Osmogroup” is here used as an abbreviation for “osmogrouphic group”. This term shouldn't be confused with the term “osmocluster”, which is used along the manuscript, and refers to the gene family clusters that are found to be overrepresented in copy number in the osmogroups.
  - Cluster/gene/gene family terms are used as synonyms of osmocluster along the slides.

- Each slide includes two types of visualizations:
  1) A phylogenetic tree of the osmocluster, in which poorly supported bipartitions with <80% SH-aLRT bootstrap support were collapsed and turned into polytomies.
  2) Barplot/s showing the best score in alignments involving the cluster sequence indicated in the plot title (query sequence) against a database consisting of all eukaryotic and non-eukaryotic sequence used in this study. In particular, the height of the bar represents the best score against each of the categories in which the included species were grouped. Alignments were done with the software Diamond, which is a computationally faster alternative to Blast.

Instructions to understand these slides

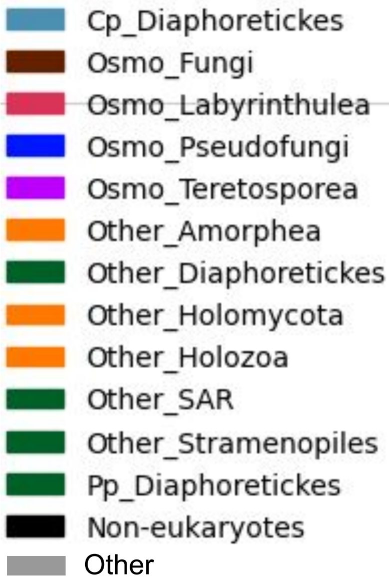

- The categories chosen to group taxa were the four osmogroups (‘Osmo_Fungi’, ‘Osmo_Labyrinthulea’, ‘Osmo_Pseudofungi’, ‘Osmo_Teretosporea’), as well as other categories that represent the rest of species. These other categories are the following (see ‘Group info 2’ column in Supplementary Table 6):
    - The non-osmogroup categories within Amorphea are ‘Other_Holozoa’, which include all the holozoan species apart from Teretosporea, ‘Other_Holomycota’, which include all the holomycotan species apart from Fungi, and ‘Other_Amorphea’, which include the rest of Amorphea species.
    - The non-osmogroup categories within Diaphoretickes are ‘Pp_Diaphoretickes’, which include the Diaphoretickes species with primary plastids (plastids derived from a primary endosymbiosis), ‘Cp_Diaphoretickes’, which include the Diaphoretickes species with complex plastids (plastids derived from an endosymbiosis with an eukaryotic alga), ‘Other_Stramenopiles’, which include the Stramenopiles species that are not in the previously mentioned categories, ‘Other_SAR’, which include the SAR species that are not in the previously mentioned categories, and ‘Other_Diaphoretickes’, which include the Diaphoretickes species that are not in the previously mentioned categories.
    - The ‘Other’ category represents taxa that branches outside Amorphea and Diaphoretickes. This taxa was included as part of the extended taxon sampling.
    - The ‘Non-eukaryotes’ category represents non-eukaryotic sequences.
- The bars of the barplots, as well as the sequence names of the tree, are colored based on the color code here represented.

Instructions to understand these slides

- Beyond the topology, the phylogenetic tree offers three additional sources of colour information:

  1) Each sequence label is colored according to the category to which each species have been assigned. Information on the category assigned to each species is available in Supplementary Table 6

Osmo Pseudofungi-Hyphochytrium catenoides 4628 OG5419
Other Diaphoretickes-Goniomonas avonlea 16394 OG5419
Pp Diaphoretickes-Pyropia yezoensis 8039 OG5419
Osmo Fungi-Spizellomyces punctatus 8548 OG5419
Other Diaphoretickes-Goniomonas avonlea 17276 OG5419
Pp Diaphoretickes-Chondrus crispus 9075 OG5419
Pp Diaphoretickes-Chondrus crispus 2655 OG5419
Osmo Fungi-Gonapodya prolifera 4513 OG5419
Osmo Teretosporea-Amoebidium parasiticum 16794 OG5419
Osmo Teretosporea-Ichthyophonus hoferi 5377 OG5419
Osmo Teretosporea-Pirum gemmata 21208 OG5419
Osmo Teretosporea-Abeoforma whisleri 17290 OG5419
Osmo Teretosporea-Abeoforma whisleri 19800 OG5419
Osmo Teretosporea-Abeoforma whisleri 15960 OG5419
Osmo Teretosporea-Abeoforma whisleri 1517 OG5419
Osmo Fungi-Spizellomyces punctatus 9214 OG5419
Osmo Fungi-Coprinopsis cinerea 3110 OG5419

Instructions to understand these slides

- Beyond the topology, the phylogenetic tree offers three additional sources of colour information:

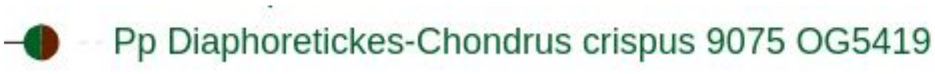

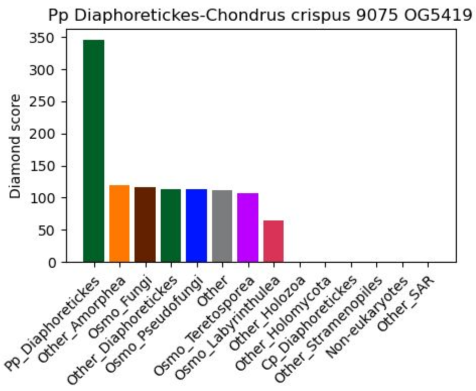

2) For the branch corresponding to each eukaryotic sequence, a pie chart displaying a summary information on the Diamond alignment is represented. Every pie chart can have maximum two areas. Each area occupies a fraction of the chart which is proportional to the best Diamond alignment score of alignments involving the represented sequence and sequences from species belonging to the categories considered by each of the areas. By default, for those species belonging to categories corresponding to Diaphoretickes taxa, the first area considers every category within Diaphoretickes except the category where the species is included, and the second area considers every category within Amorphea, Other and ‘Non-eukaryotes’. And vice-versa by default for those categories corresponding to Amorphea taxa. For example, for the ‘*Chondrus crispus* 9075’ sequence, the first area corresponds to ‘Other_Diaphoretickes’ from Diaphoretickes (note that ‘Pp_Diaphoretickes is ignored, as this is the category corresponding to this species), and the second area corresponds to ‘Osmo_Fungi’ from Amorphea. Both areas have similar size since both represent similar alignment scores.

Instructions to understand these slides

- Beyond the topology, the phylogenetic tree offers three additional sources of colour information:

2) Regarding the categories considered by each area, exceptions were applied only in the case of osmogroups. The specific categories considered by each of the two areas for each osmogroup are shown below:

Osmo_Fungi	Prokaryotes,Cp_Diaphoretickes,Osmo_Labyrinthulea,Osmo_Pseudofungi,Other_Diaphoretickes,Other_SAR,Other_Stramenopiles,Pp_Diaphoretickes,Other	Other_Amorphea,Other_Holomycota,Other_Holozoa
Osmo_Labyrinthulea	Prokaryotes,Osmo_Fungi,Osmo_Teretosporea,Other_Amorphea,Other_Holomycota,Other_Holozoa,Other	Cp_Diaphoretickes,Other_Diaphoretickes,Other_SAR,Other_Stramenopiles,Pp_Diaphoretickes
Osmo_Pseudofungi	Prokaryotes,Osmo_Fungi,Osmo_Teretosporea,Other_Amorphea,Other_Holomycota,Other_Holozoa,Other	Cp_Diaphoretickes,Other_Diaphoretickes,Other_SAR,Other_Stramenopiles,Pp_Diaphoretickes
Osmo_Teretosporea	Prokaryotes,Cp_Diaphoretickes,Osmo_Labyrinthulea,Osmo_Pseudofungi,Other_Diaphoretickes,Other_SAR,Other_Stramenopiles,Pp_Diaphoretickes,Other	Other_Amorphea,Other_Holomycota,Other_Holozoa

- Basically, the only difference from the default behaviour is that the osmogroups belonging to the same eukaryotic division are ignored. For example, for ‘Osmo_Fungi’ (Amorphea), the category ‘Osmo_Teretosporea’ (Amorphea) is ignored. This was done because we observed that in multiple occasions, when a cluster is present in a given osmogroup, within the same eukaryotic division, the cluster tends to be present only in the other osmogroup from that same division. By applying this modification, it became easier to visually identify if the cluster is present also in some other category from the same division apart from the other osmogroup.

Instructions to understand these slides

- Beyond the topology, the phylogenetic tree offers three additional sources of colour information:

  3) For osmogroup sequence,s the corresponding branch of the tree is colored based proportionally to the score difference between the score represented by the first area and the score represented by the second area. Examples:

  - ‘*P. gemmata* 19730’, from ‘Osmo_Teretosporea’, doesn’t align with any other Amorphea sequence. Hence, only a single area is represented corresponding to the best scoring category outside Amorphea (‘Osmo_Pseudofungi’)
  - ‘*A. kerguelense* 80126’, from ‘Osmo_Labyrinthulea’, the score against the best category outside Diaphoretickes (‘Osmo_Teretosporea’, purple) is much higher than the best scoring category within Diaphoretickes (‘Cp_Diaphoretickes’, light blue) excluding ‘Osmo_Pseudofungi’, the other osmogroup from Diaphoretickes. This indicates that this Labyrinthulea sequence is much more similar to Teretosporea sequences, an osmogroup from Amorphea, than to any other non-osmogroup sequence from Diaphoretickes.

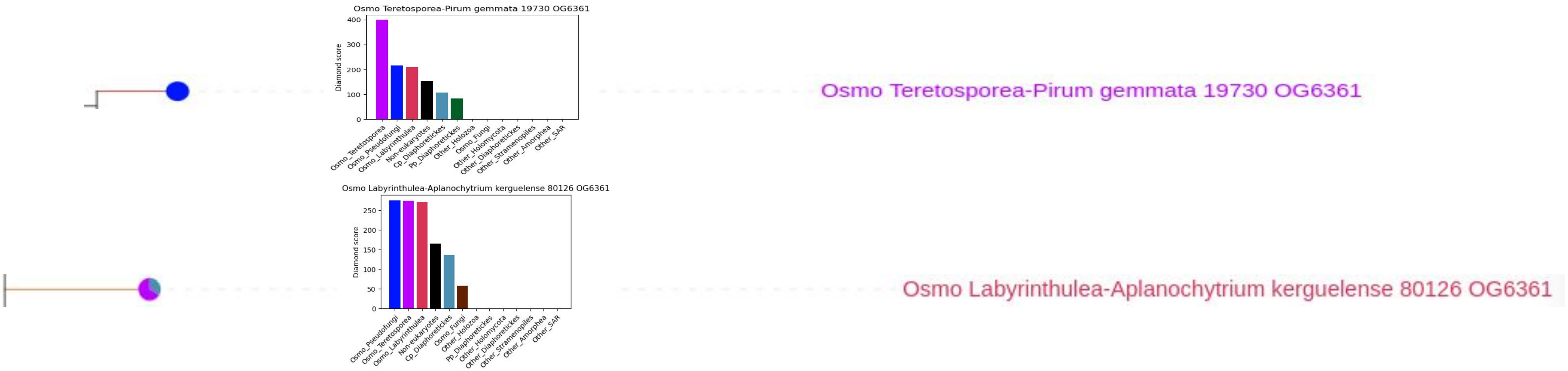

The report starts in the next slide. Note that we labeled each slide as ‘Likely HGT’ or ‘Highly likely HGT’. ‘Highly likely HGT’ are those cases in which the combination of the phylogenetic information, the taxonomic distribution of the gene family/gene/cluster/osmocluster (all these terms are here employed as synonyms), as well as the Diamond alignment results, altogether provide robust support to claim that a HGT occurred along the evolutionary history of the osmocluster. When we labeled it as ‘Likely HGT’, it is usually because the alignment scores from the Diamond alignment results are low (e.g., <= 100), which could indicate limited sequence conservation, a factor that can be limiting in providing sufficient resolution for the reconstruction of the evolutionary history of the gene family under consideration

Highly likely HGT

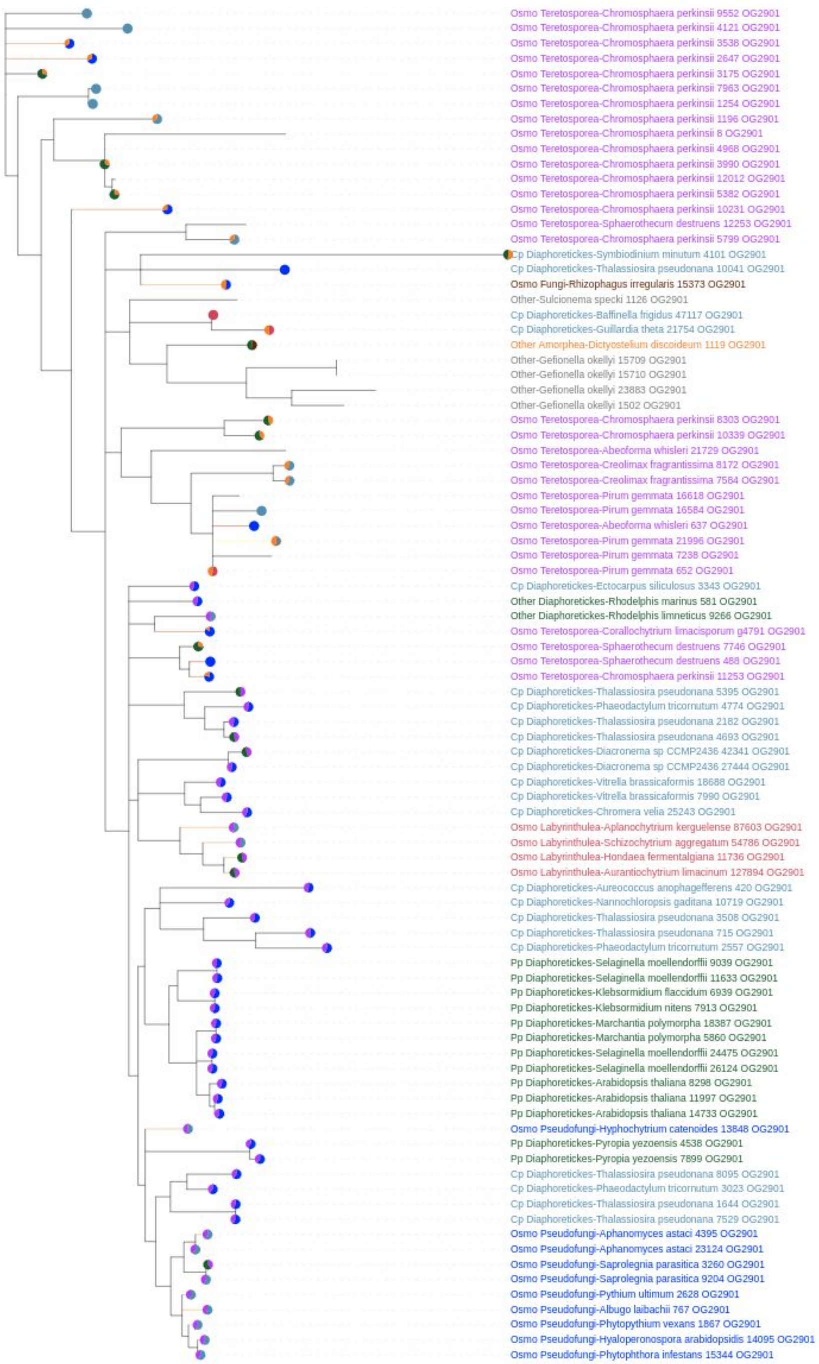

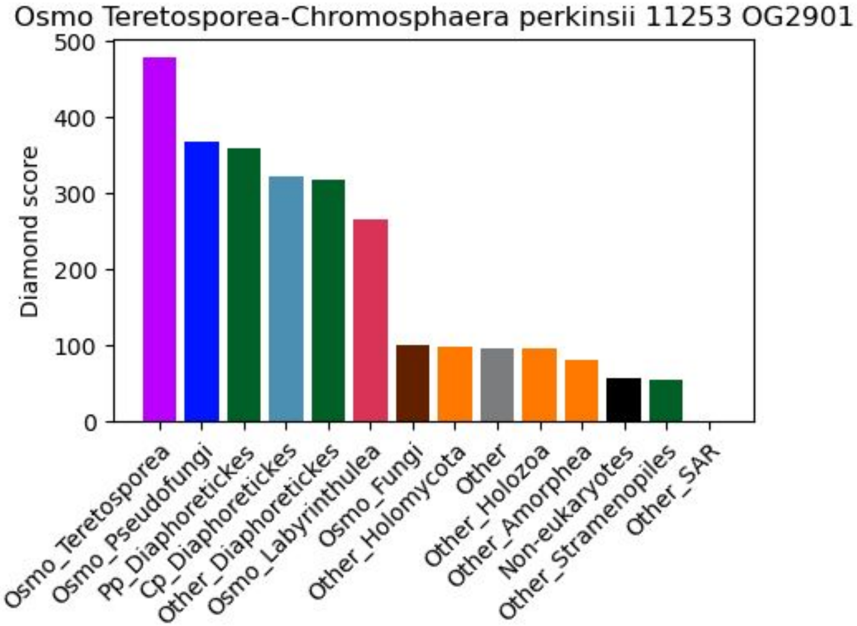

OG2901 The taxonomic distribution of this cluster is clearly biased to Diaphoretickes. In Amorphea, it is only clearly represented in Teretosporea. The sequences from the osmogroup Teretosporea were very likely transferred from Diaphoretickes, probably from Pseudofungi, given the taxonomic distribution and the Diamond alignment results.

- (0); P (0.69); cellular response to phosphate starvation (0.23)

Likely HGT

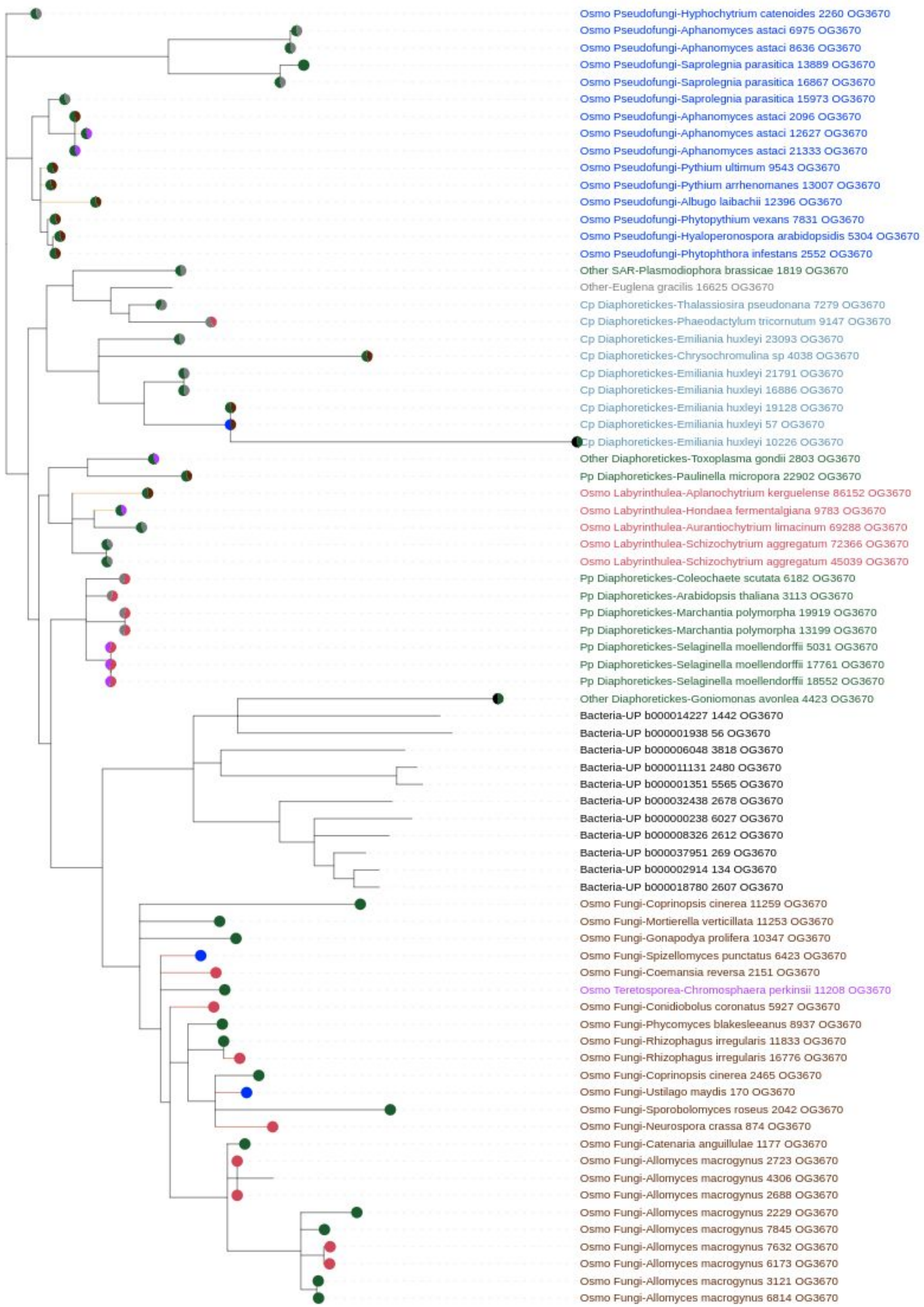

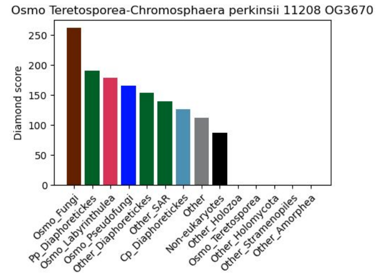

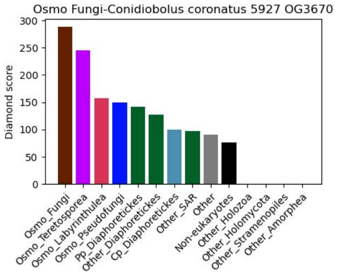

OG3670 In Amorphea, this cluster is restricted to the two osmogroups from Amorphea: Fungi and *C. perkinsii* (Teretosporea). In Diaphoretickes, this sequence is clearly more widespread. Based on the given taxonomic distribution and on the retrieved topology, it is very likely that the osmogroups from Amorphea acquired this gene through HGT. Based on the monophyly of the bacterial sequences, and also based on the Diamond alignment results, it is very likely that there was a single HGT from Diaphoretickes to Fungi, and then *C. perkinsii* would have acquired this gene through a HGT from Fungi. An alternative plausible scenario is one HGT from Diaphoretickes to Fungi and another to *C. perkinsii*.

- (0); P (0.03); Peptidase M50B-like (0.65)

Likely HGT

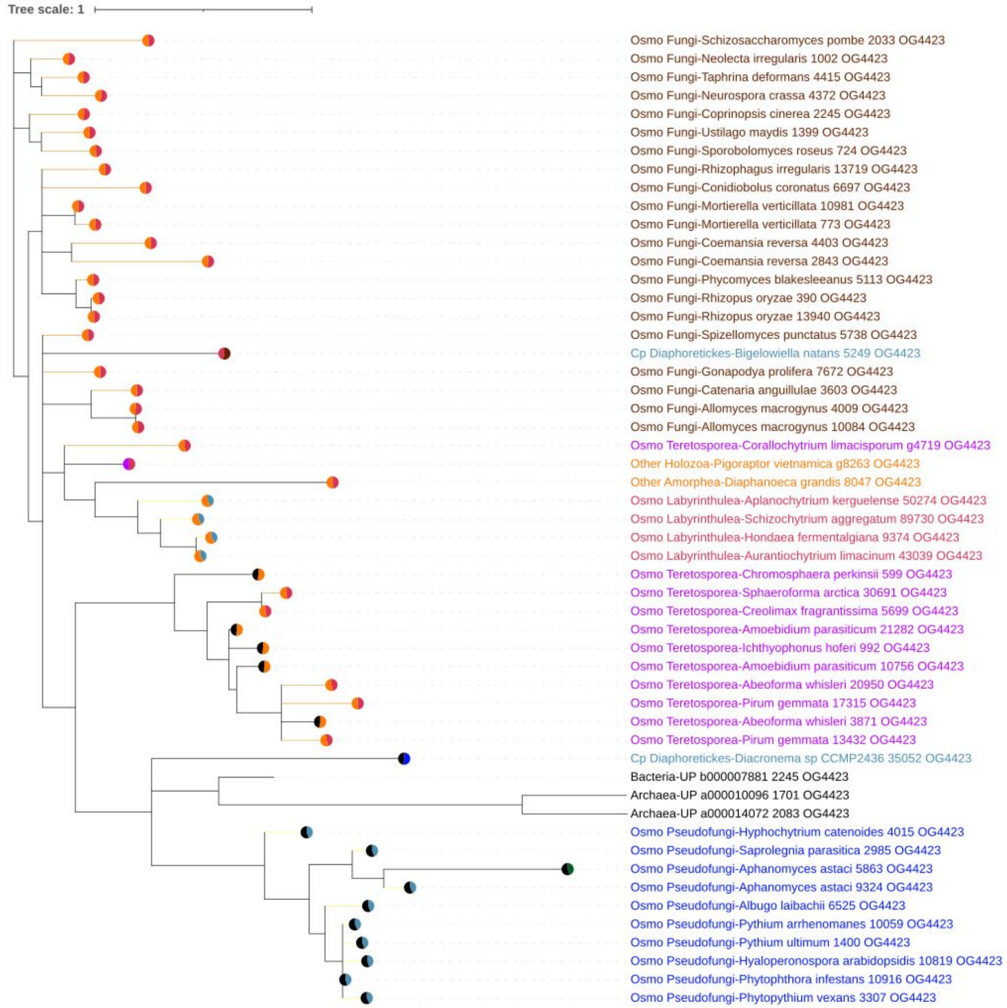

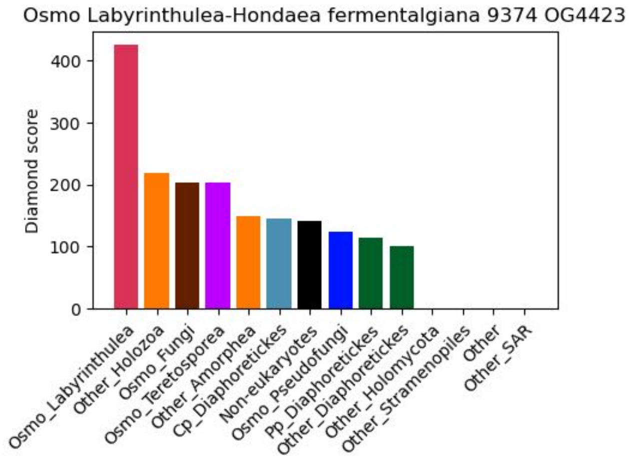

OG4423 The distribution of the cluster is taxonomically patchy. Mainly only sequences from osmogroups are represented. This is remarkable considering that the osmogroups from Amorphea are very phylogenetically unrelated to those from Diaphoretickes. It seems that a transfer from Amorphea to Diaphoretickes likely occurred at some point. The most evident hint for this are Labyrinthulea sequences, which show substantially higher alignment score with sequences from Amorphea (Holozoa, Fungi, Teretosporea) than with sequences from Diaphoretickes.

ILV6 (1.0); E (1.0); acetolactate synthase activity (0.31)

Highly likely HGT

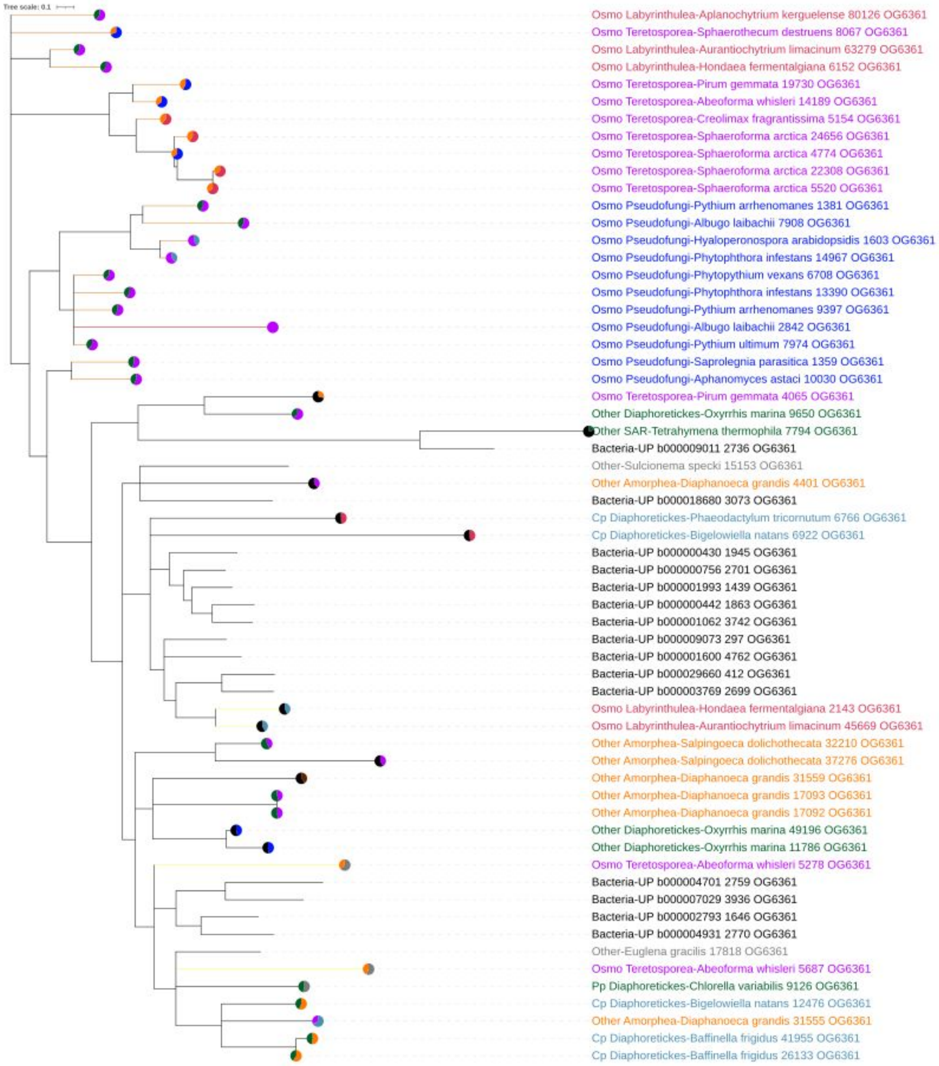

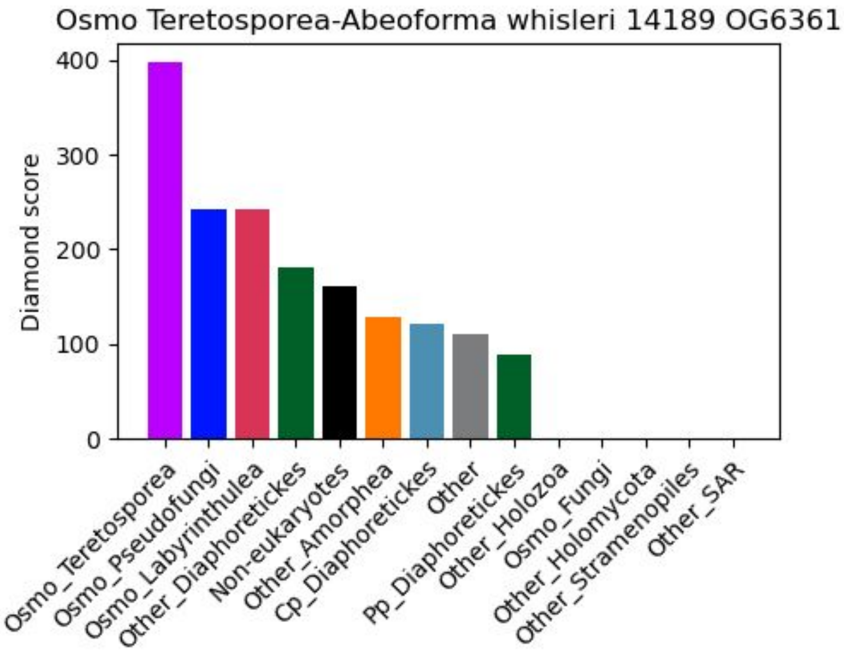

OG6361 The distribution is very biased towards osmogroups. It is likely that there was a transfer between the osmogroups from Diaphoretickes (Pseudofungi, Labyrinthulea) and Teretosporea. Note how Teretosporea sequences show a clearly higher alignment score in alignments with Diaphoretickes osmogroups than to bacterial sequences.

mmuM (0.1); E (0.73); Source PGD (0.43)

Likely HGT

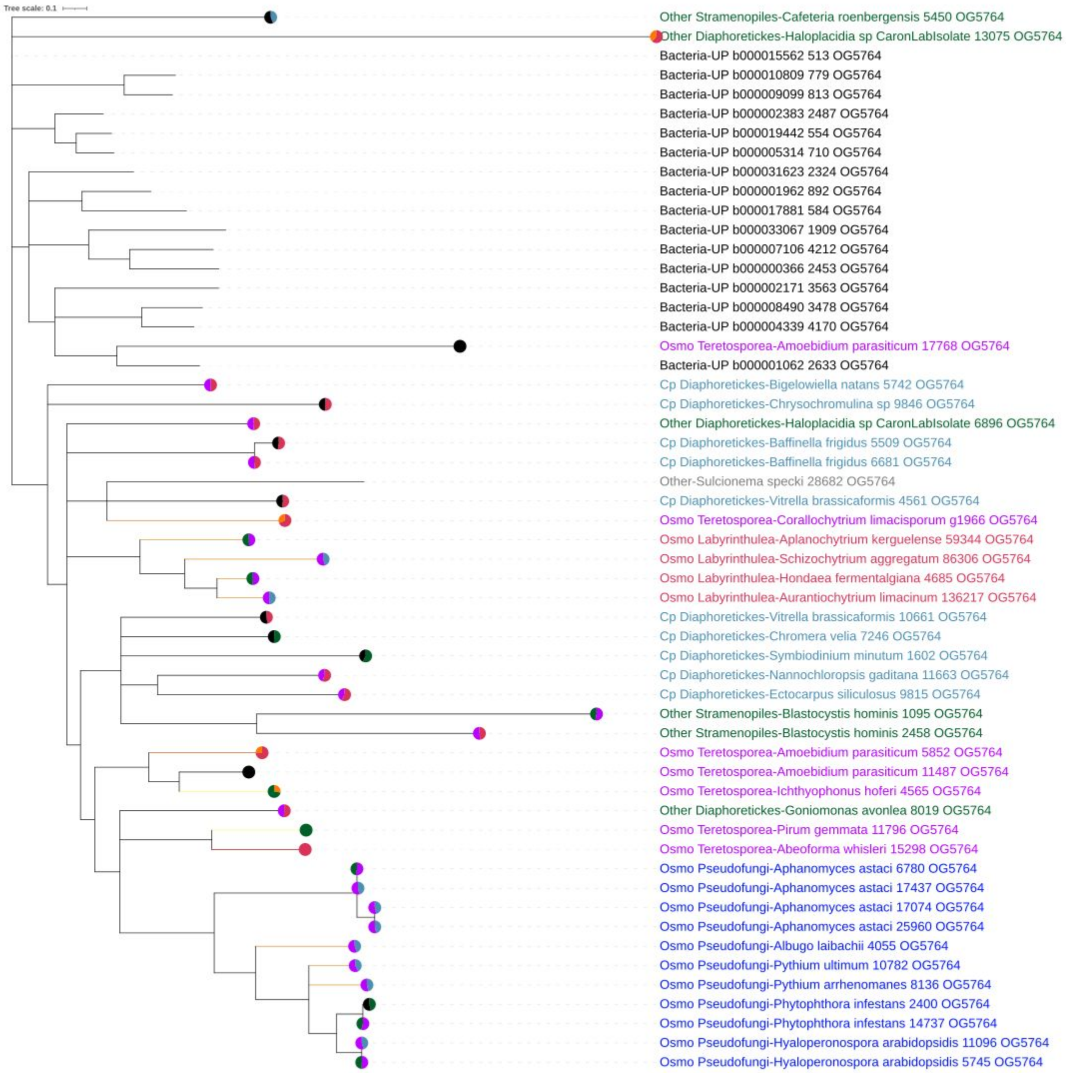

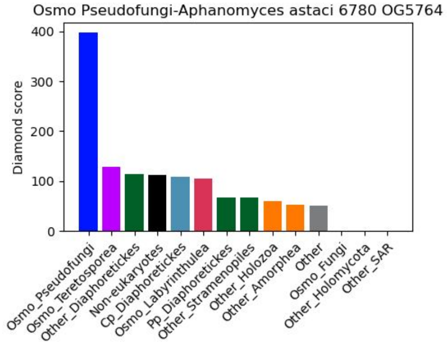

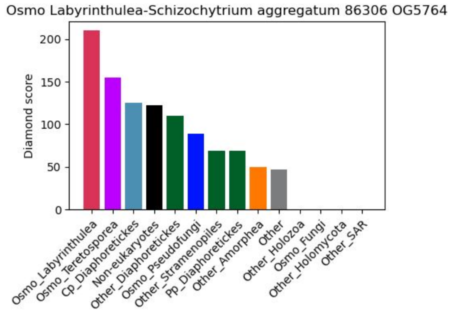

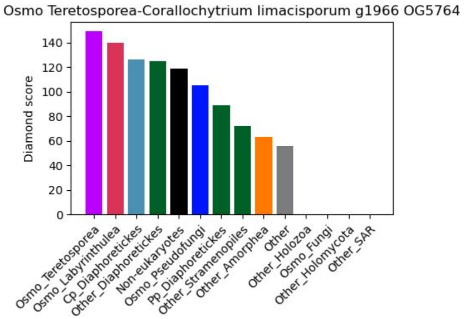

OG5764 Patchy distribution but complex phylogeny. The patchy taxonomic distribution of the cluster, enriched in taxa from Diaphoretickes, but with the inclusion of Teretosporea, suggests that Teretosporea may have acquired the cluster from Diaphoretickes. Alternatively, Bacteria transferred multiple times this gene family to eukaryotes

yigB (0.15); H (0.03); Haloacid dehalogenase-like hydrolase (0.29)

Likely HGT

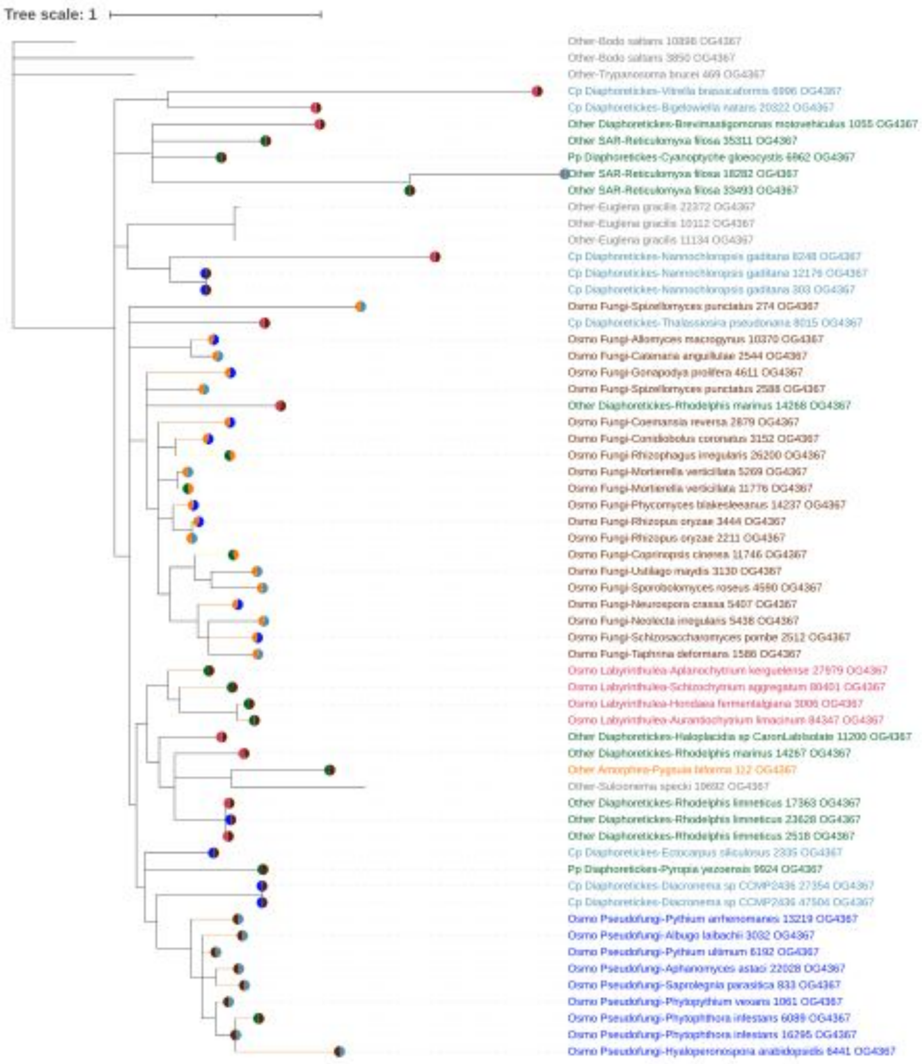

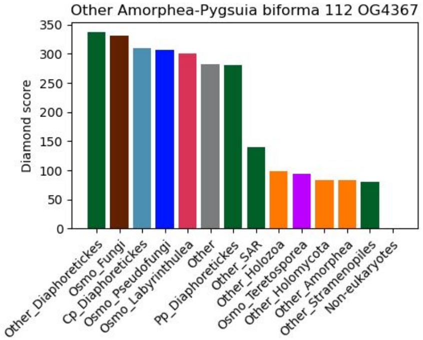

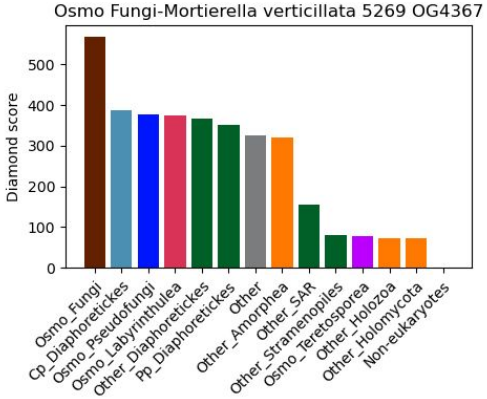

OG4367 Patchy distribution. The cluster is found in Diaphoretickes and in Fungi. Fungi clearly acquired this gene from the Diaphoretickes side of eukaryotes. While within Amorphea, the cluster is also found in *Pygsuia biforma*, this amorphean is phylogenetically distant to Fungi, suggesting most likely independent acquisitions of this gene at least twice in Amorphea.

YHM1 (0.96); C (0.88); Mitochondrial carrier protein (0.46)

Highly likely HGT

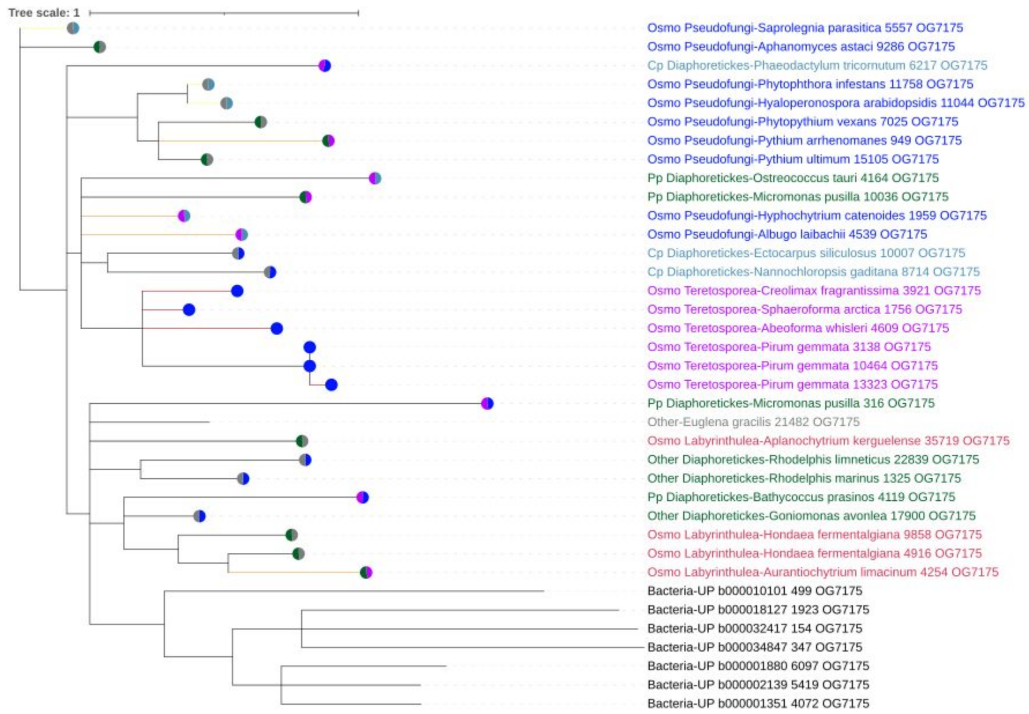

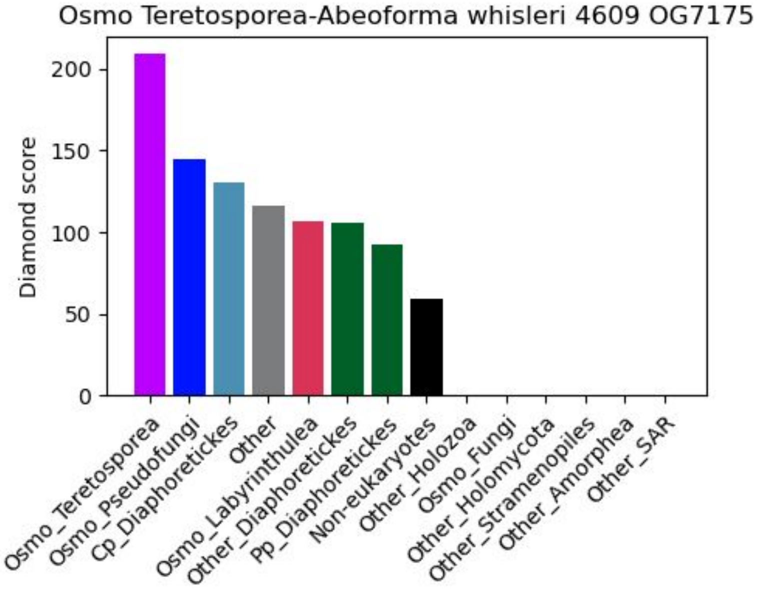

OG7175 Patchy distribution. Within Amorphea, the cluster is only found in Teretosporea. Teretosporea most likely acquired the gene from the Diaphoretickes, probably Pseudofungi.

- (0); H (0.96); Cytidylyltransferase-like (0.85)

Highly likely HGT

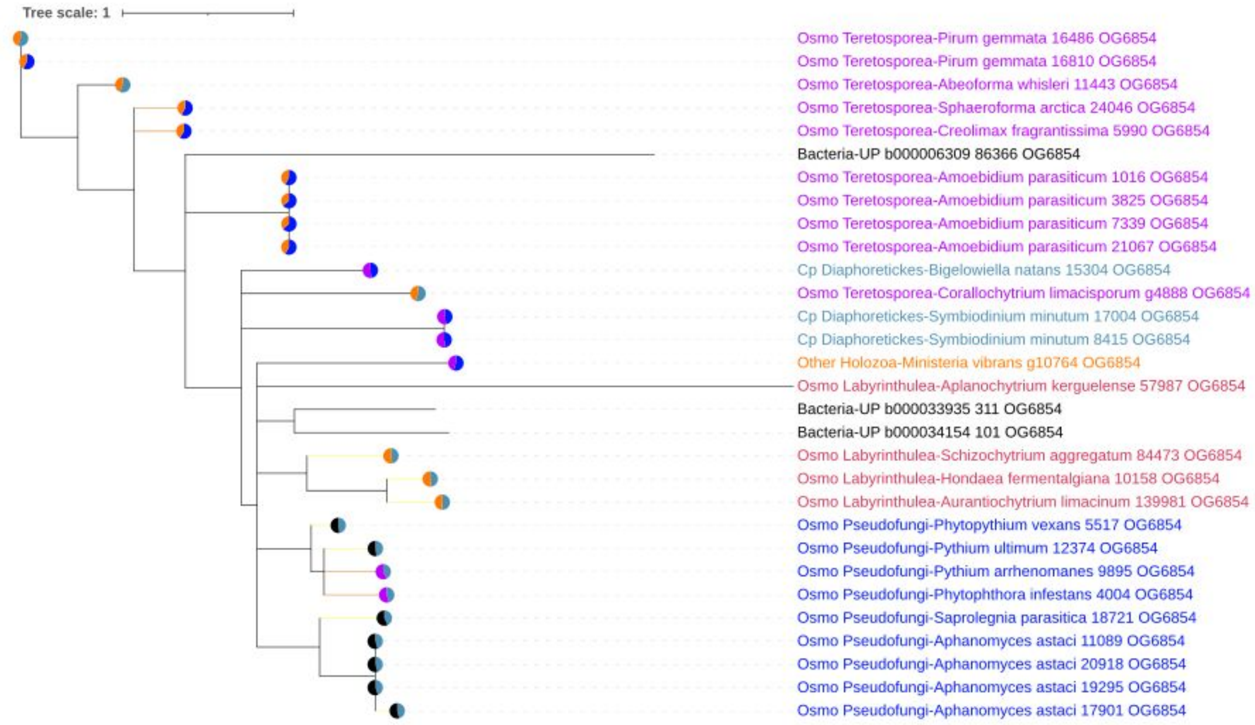

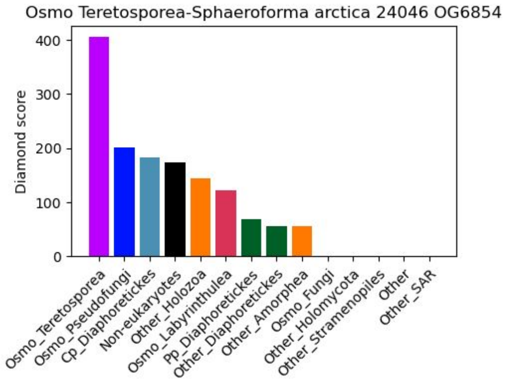

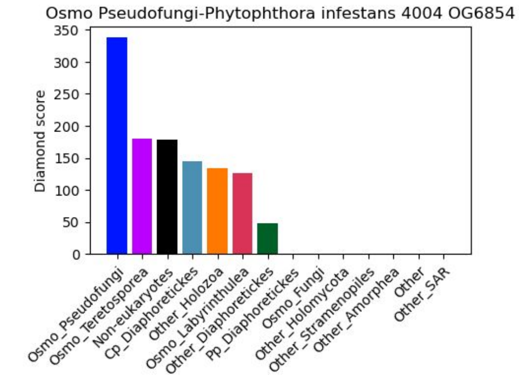

OG6854 Patchy distribution, largely biased to osmogroups. Found in the three osmogroups and absent from Fungi. At least one transfer from Bacteria to eukaryotes, probably more than one. This convoluted topology could be explained either by multiple HGTs from Bacteria to osmogroups, or secondary HGTs involving osmogroups. In any case, the absence of HGT in the evolution of this gene family is highly unlikely.

kinesin-3D (0.04); Z (0.04); Succinylglutamate desuccinylase / Aspartoacylase family (0.78)

Highly likely HGT

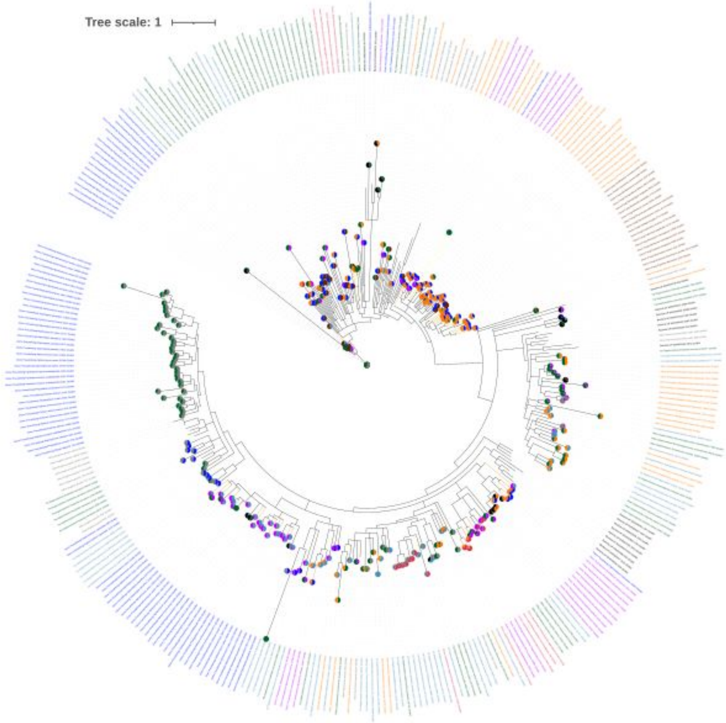

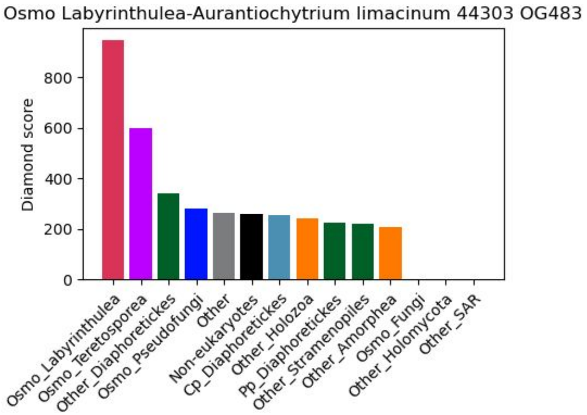

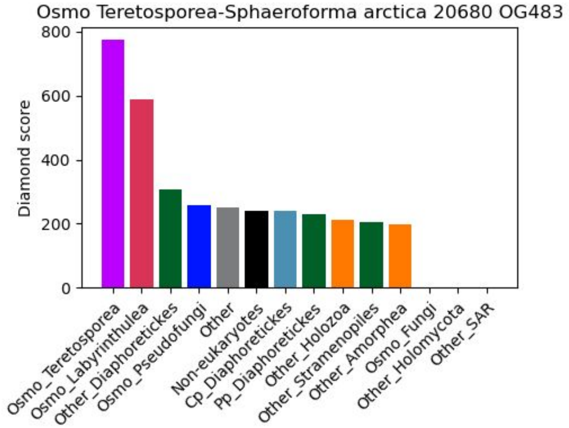

OG483 This is a widely distributed cluster. Notwithstanding this, there are some topologies suggestive of HGT events involving osmogroups. The clearest case is the one represented in this slide, involving Labyrinthulea and Teretosporea

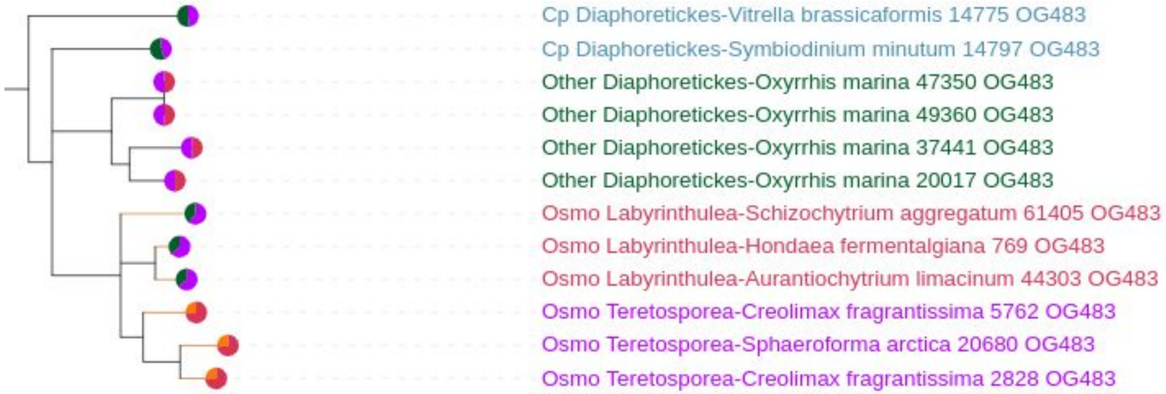

ARPC2 (0.22); E (0.62); Peptidase family C69 (0.48)

Highly likely HGT

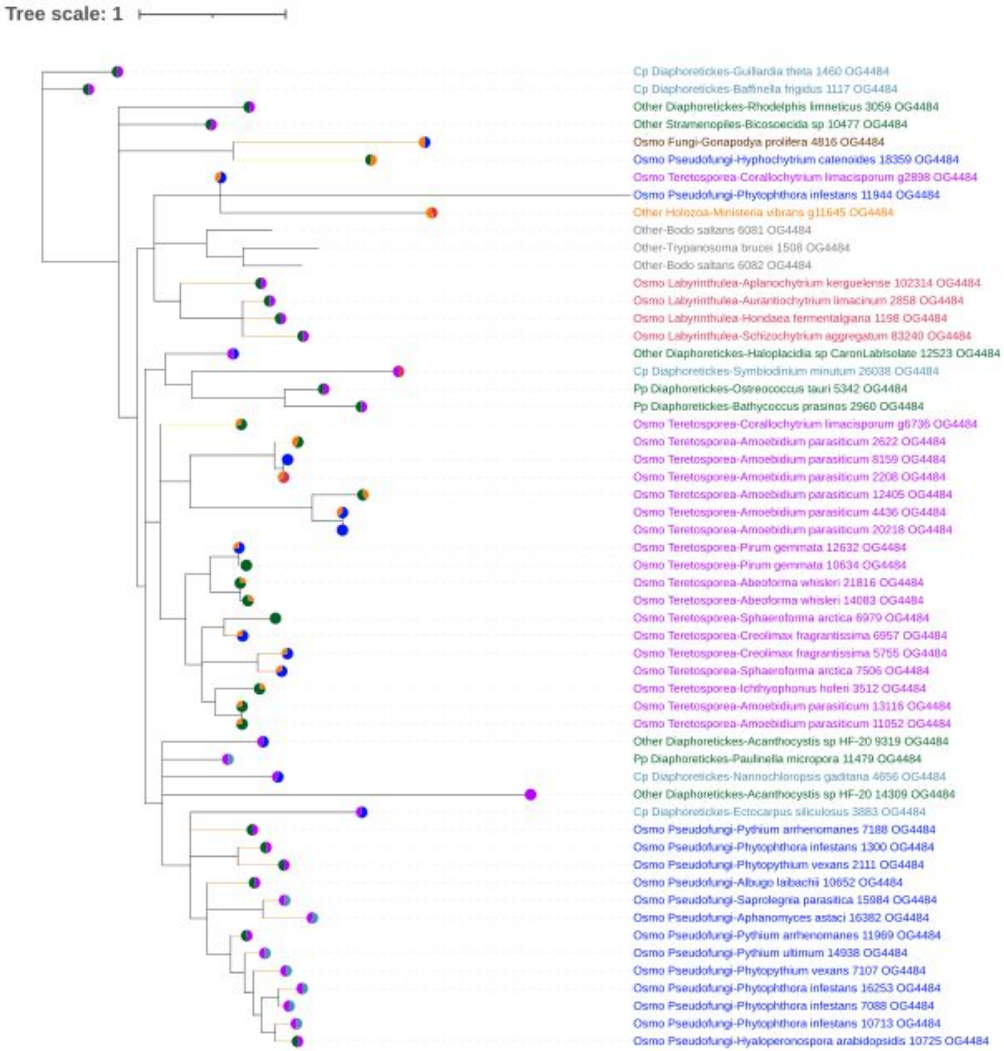

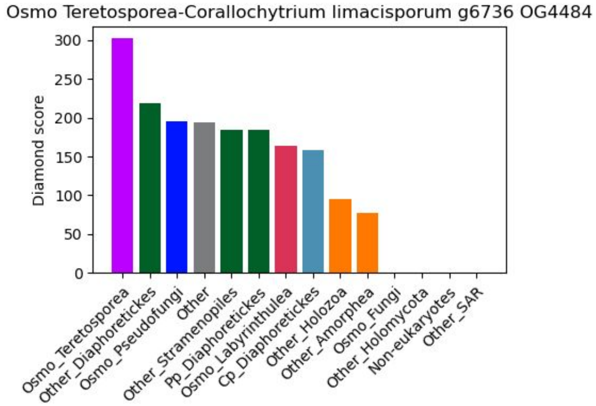

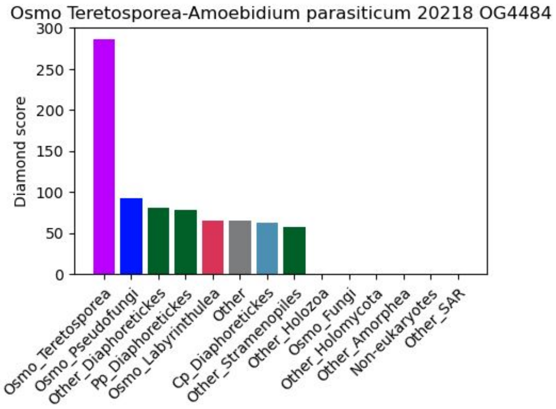

OG4484 The taxonomic distribution of this cluster is biased towards Diaphoretickes. While the topology is convoluted, the taxonomic distribution and the Diamond results strongly suggests that Teretosporea incorporated the gene family represented by this cluster from outside Amorphea, most likely from Diaphoretickes, probably from Pseudofungi

PNPLA2,,,PNPLA1 (0.02); U (0.28); triglyceride catabolic process (0.81)

Highly likely HGT

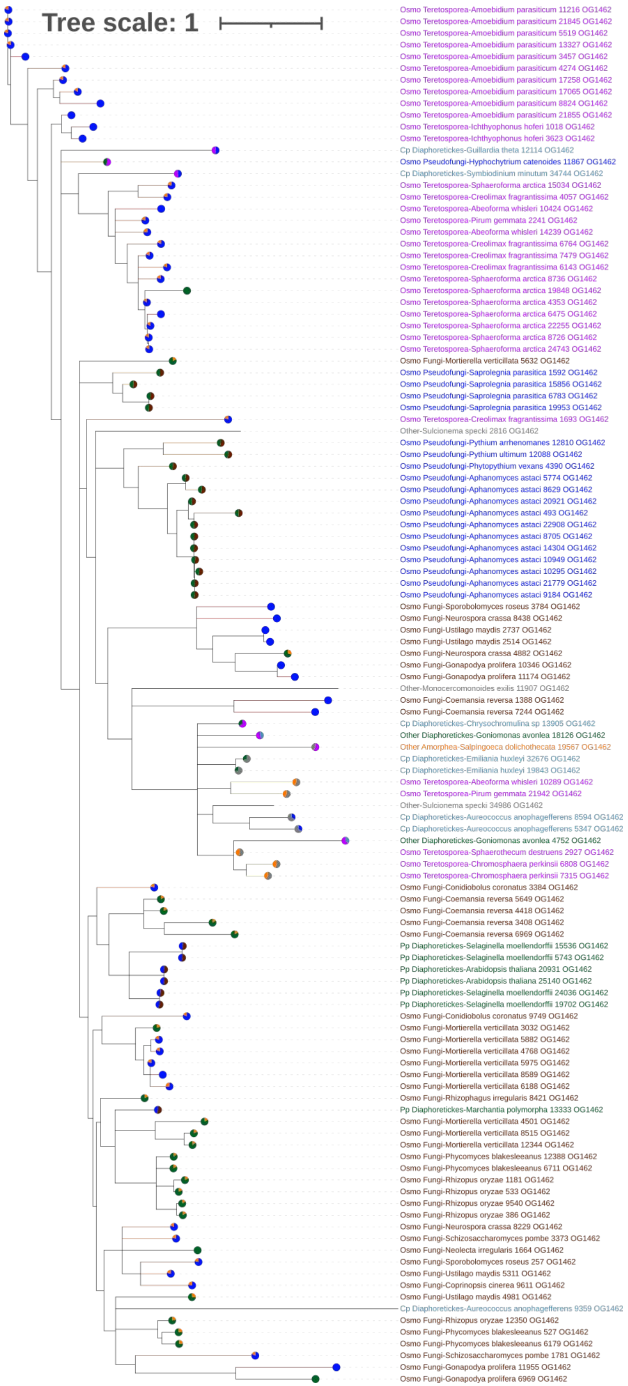

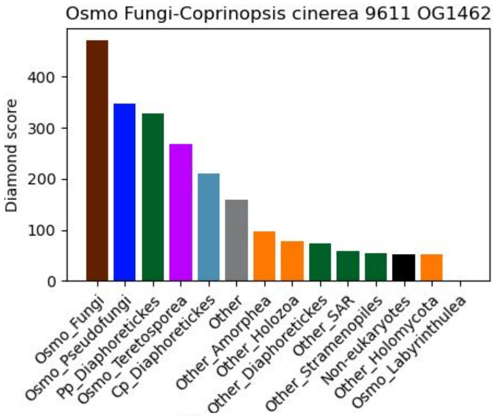

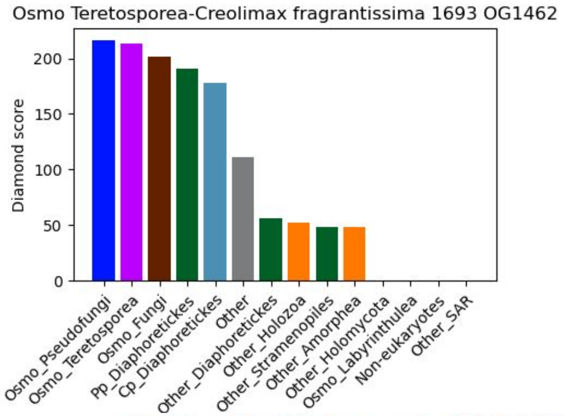

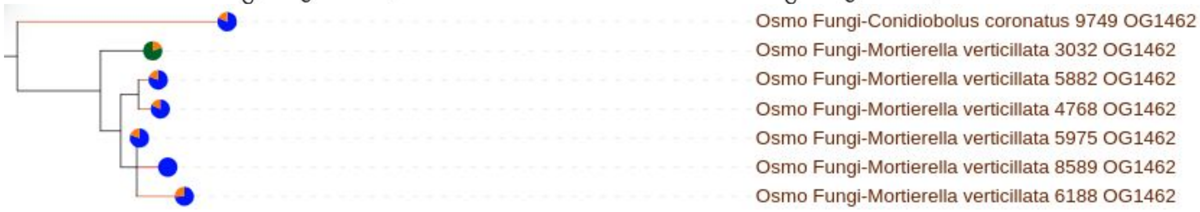

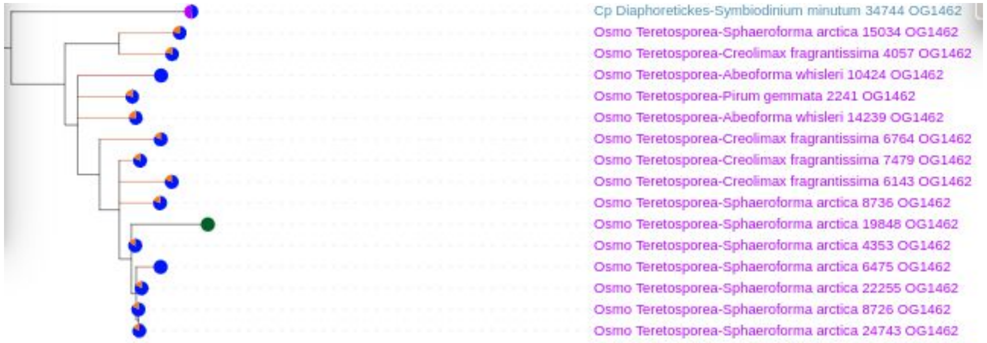

OG1462 The topology of the tree and the Diamond results congruently suggest HGT acquisitions of this cluster by the osmogroups from Amorphea (Fungi and Teretosporea) from outside Amorphea, most likely from Pseudofungi.

- (0); A (0.02); Ion channel regulatory protein UNC-93 (0.32)

Likely HGT

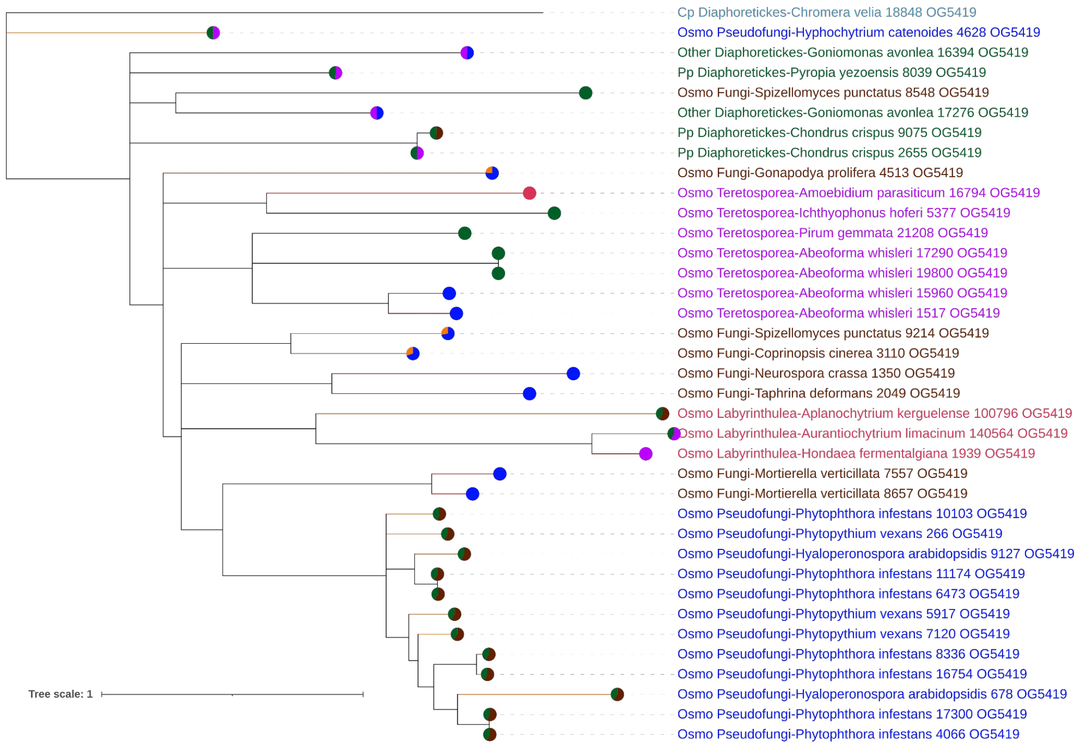

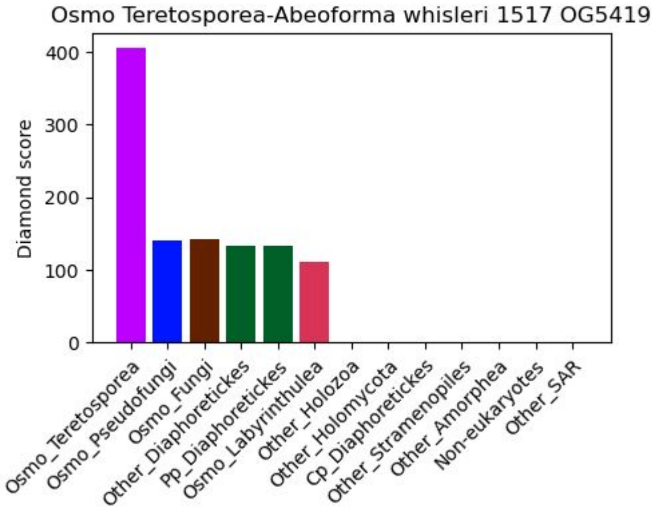

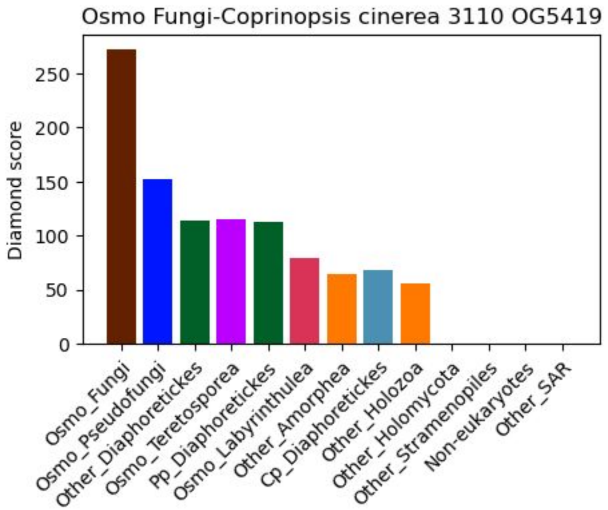

OG5419 The topology of the tree is highly convoluted, but the taxonomic distribution and the Diamond alignment scores are altogether strongly suggestive of HGT being an explanatory factor behind the biased taxonomic distribution of this cluster. While for the fungal sequence represented in the second barplot, the plot suggests potential homologs from the Amorphea side of eukaryotes (orange bars) that could be missing from the cluster (alternatively, these could be outparalog sequences), the difference in score for example between ‘Other_Amorphea’ and ‘Osmo_Pseudofungi’ to the fungal sequence represented in the barplot strongly suggests that the fungal sequence (and also the teretosporean sequence represented above) is most likely coming from Diaphoretickes (horizontal origin) than from Amorphea (vertical origin). Still, we labeled this case as ‘Likely HGT’ and not as ‘Highly likely HGT’ due to the fact that the Diamond scores (<100) are potentially limiting

- (0); - (0):Protein kinase C conserved region 2 (CalB) (0.54)

Highly likely HGT

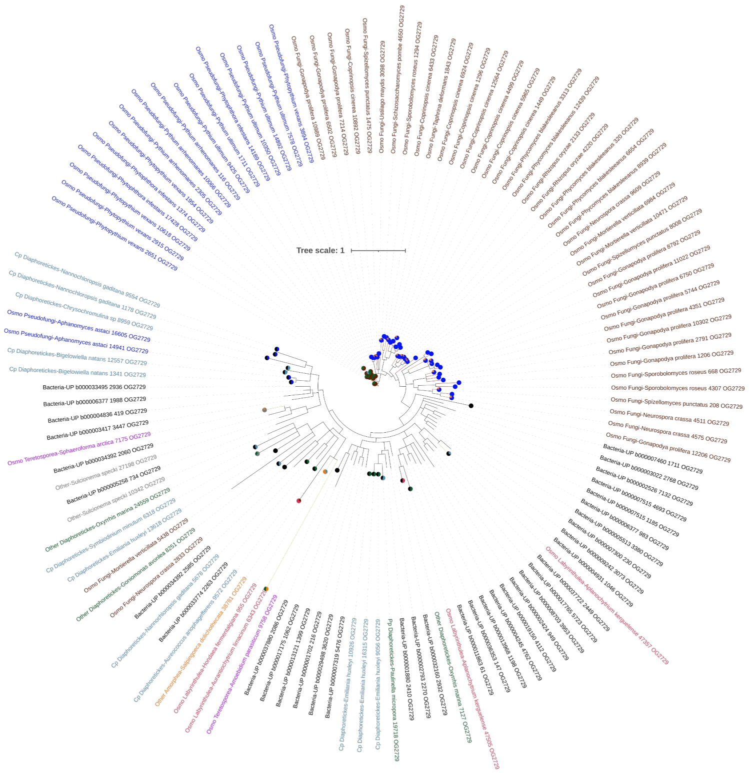

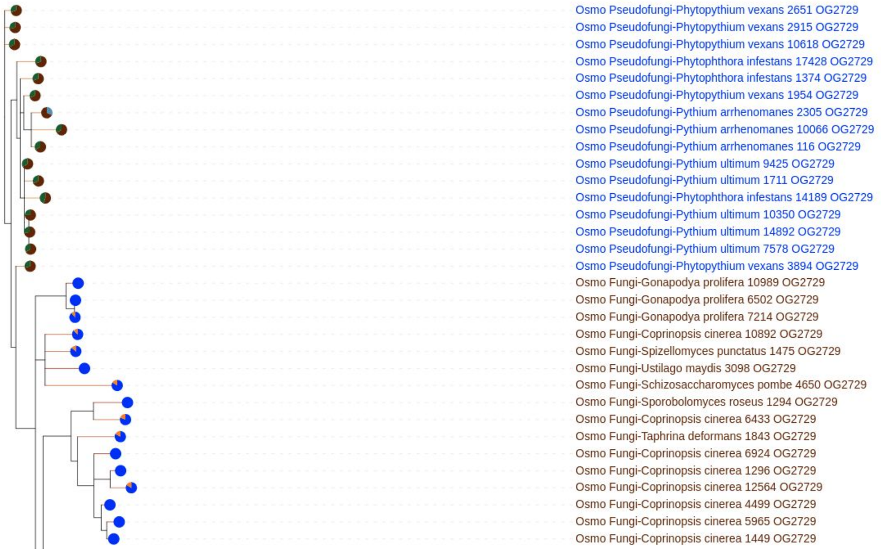

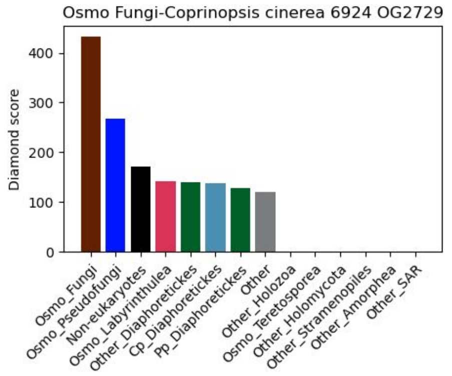

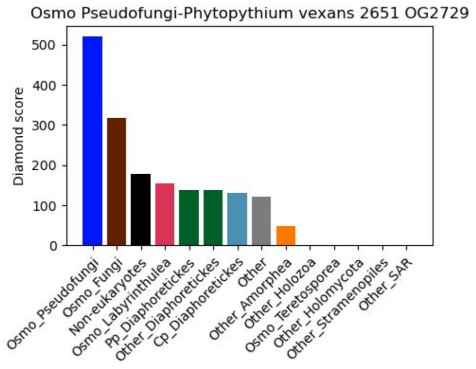

OG2729 The topology and the Diamond alignment results strongly support a HGT involving Fungi and Pseudofungi.

tauD (0.05); E (0.5); Taurine catabolism dioxygenase TauD, TfdA family (0.38)

Highly likely HGT

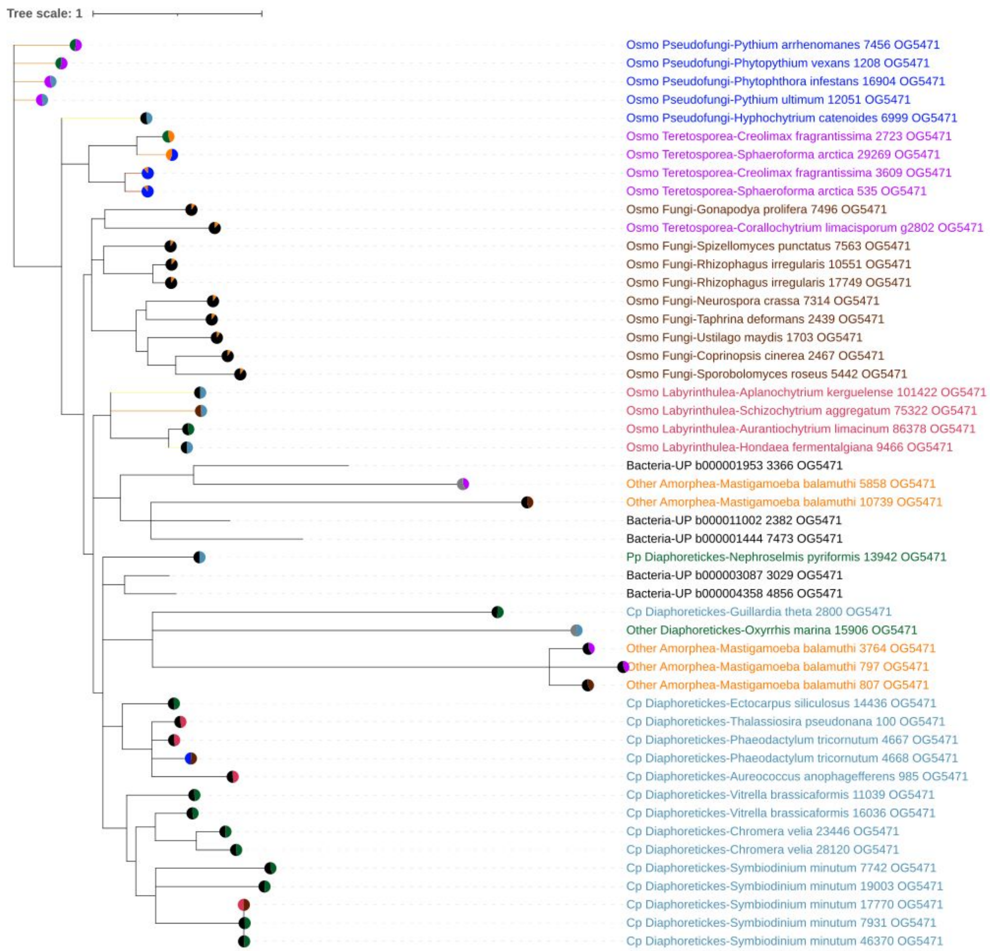

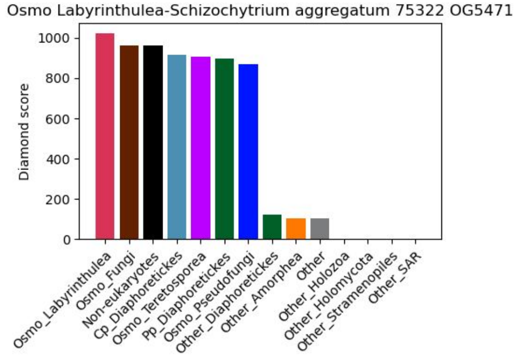

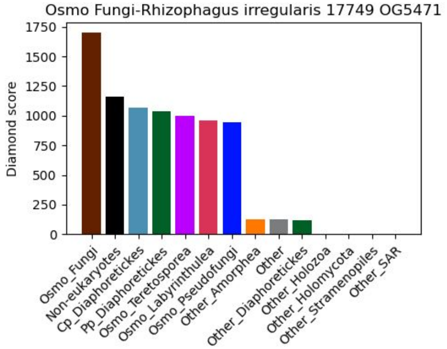

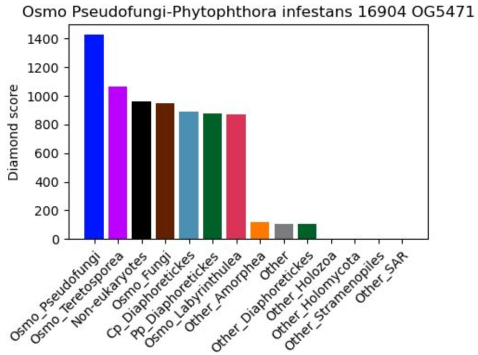

OG5471 The distribution of the cluster is patchy and biased to osmogroups. It is present only in the four osmogroups, in some other Diaphoretickes lineages (algae with complex plastids). (The sequences from *Mastigamoeba balamuthi* are probably erroneous cluster members, see long branches). Bacterial sequences are not monophyletic, suggesting potential multiple HGT acquisitions from Bacteria to osmogroups or to the ancestors of the osmogroups ancestors. Still, Diamond alignment results and the topology give support to a HGT involving Teretosporea and Pseudofungi

nirB (0.33); C (0.39); nitrite reductase (NAD(P)H) large subunit (0.28)

Likely HGT

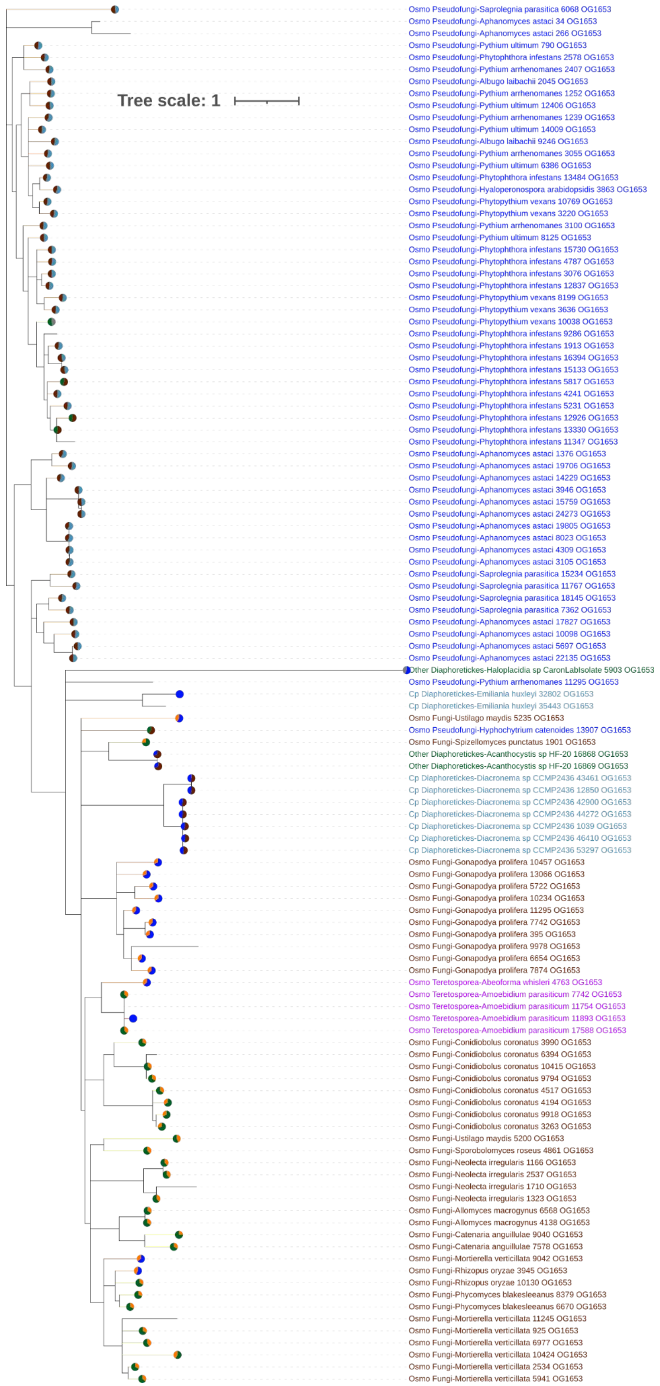

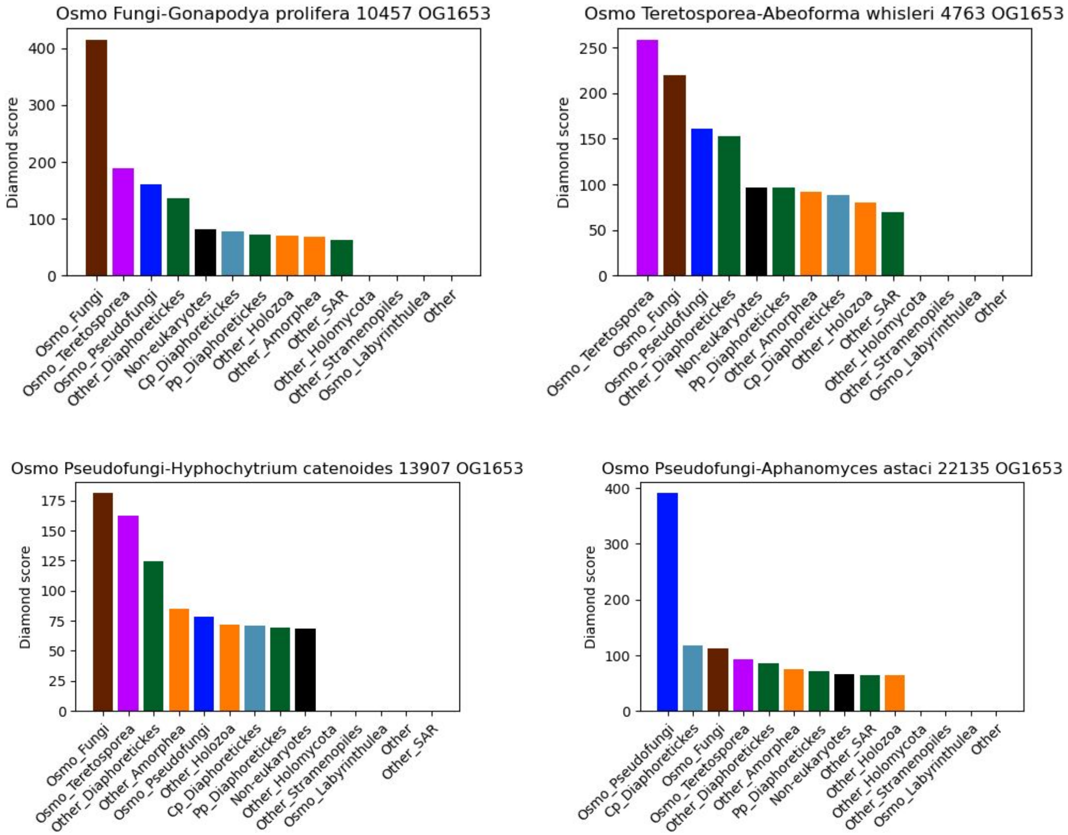

OG1653 The topology of this cluster is very patchy. It is found in three of the four osmogroups. The taxonomic distribution and Diamond alignment patterns suggest HGT patterns involving the osmogroups from Amorphea with Diaphoretickes taxa, most likely Pseudofungi.

MNN1 (0.22); G (0.18); Mannosyltransferase putative (0.47)

Highly likely HGT

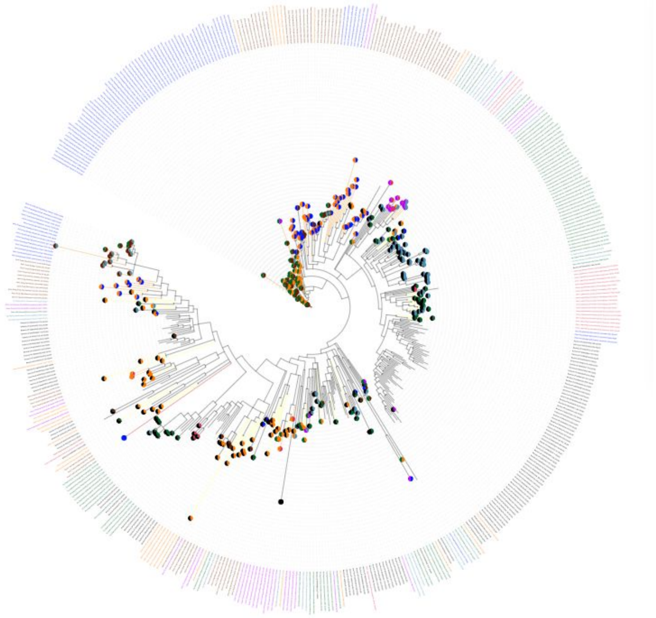

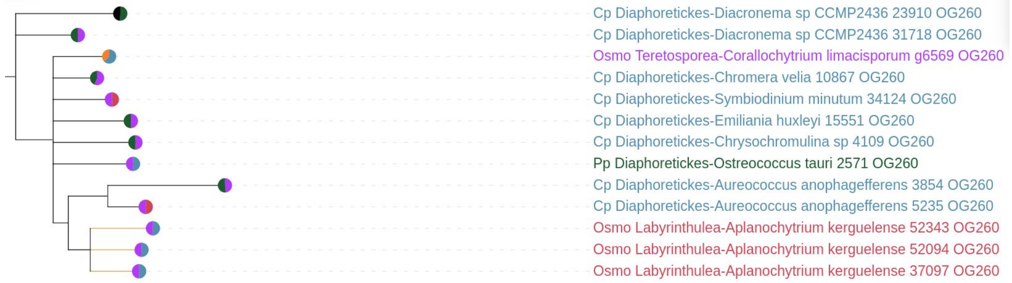

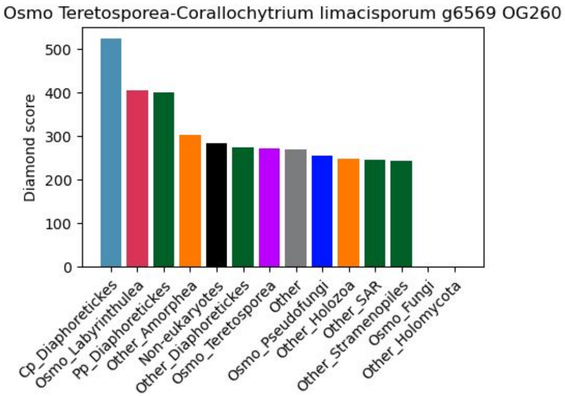

OG260 This is a huge cluster, widely distributed. Still, there are some clades which are strongly suggestive of HGT involving osmogroups. The case above is one sequence from Teretosporea, from the species *C. limacisporum*, robustly branching within a Diaphoretickes clade. On the other hand, there is also a clade including Fungi and Pseudofungi. In both cases, the Diamond alignment results support the hypothesized transfer events.

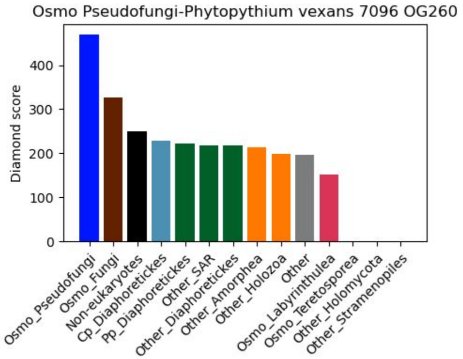

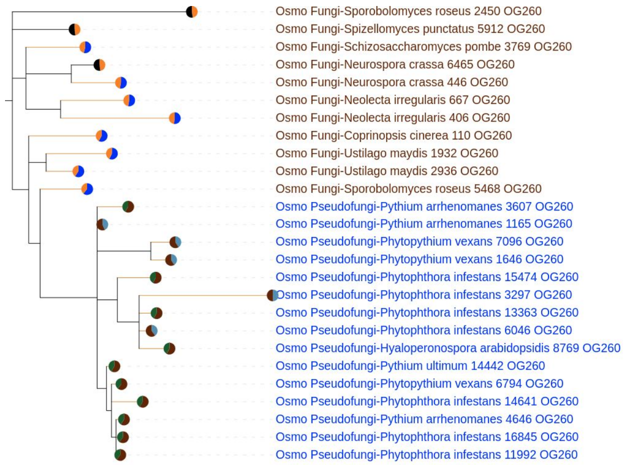

ADH1 (0.07); Q (0.67): Alcohol dehydrogenase GroES-like domain (0.27)

Highly likely HGT

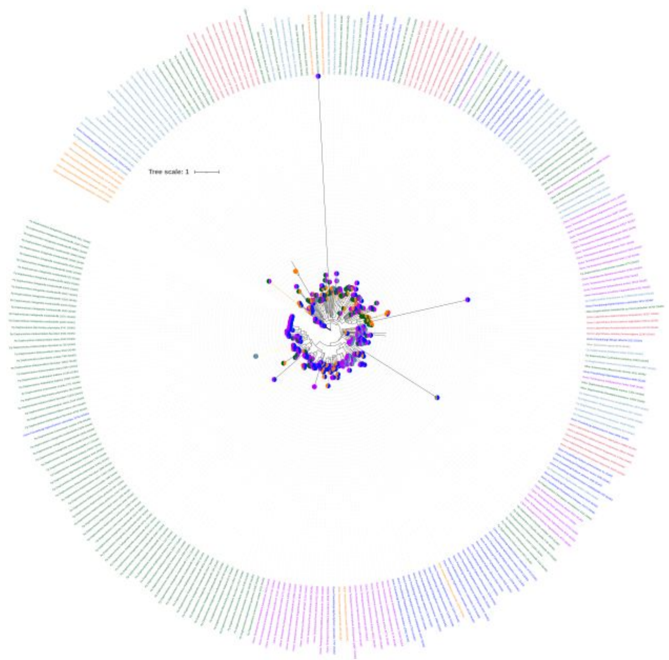

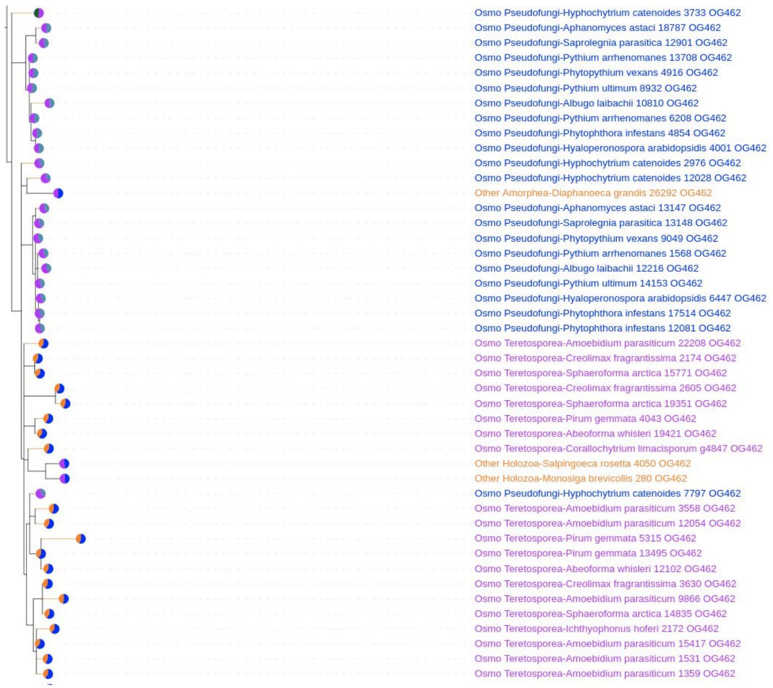

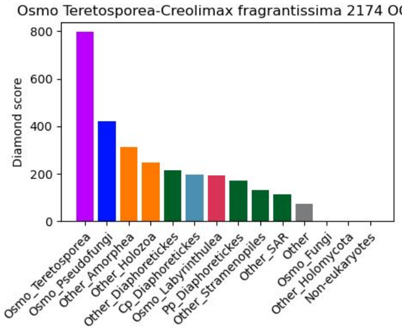

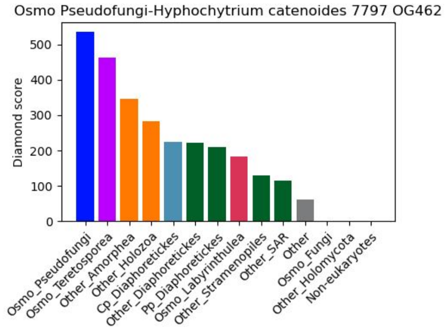

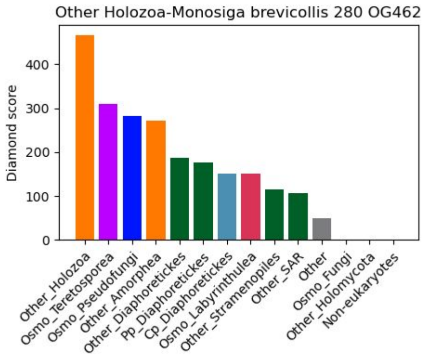

OG462 Clusters with a quite wide distribution across Diaphoretickes, but with a restricted distribution in Amorphea. In Amorphea, it is only present in the osmogroup Teretosporea (Holozoa), and in Choanoflagellatea (Holozoa). The topology and Diamond alignment results strongly suggests that the sequences from Teretosporea and Choanoflagellates most likely originated from HGTs from Dipahoretickes, likely from Pseudofungi. The number of transfers occurred is uncertain.

TCF25 (0.02); T (0.34); Protein of unknown function (DUF1336) (0.46)

Likely HGT

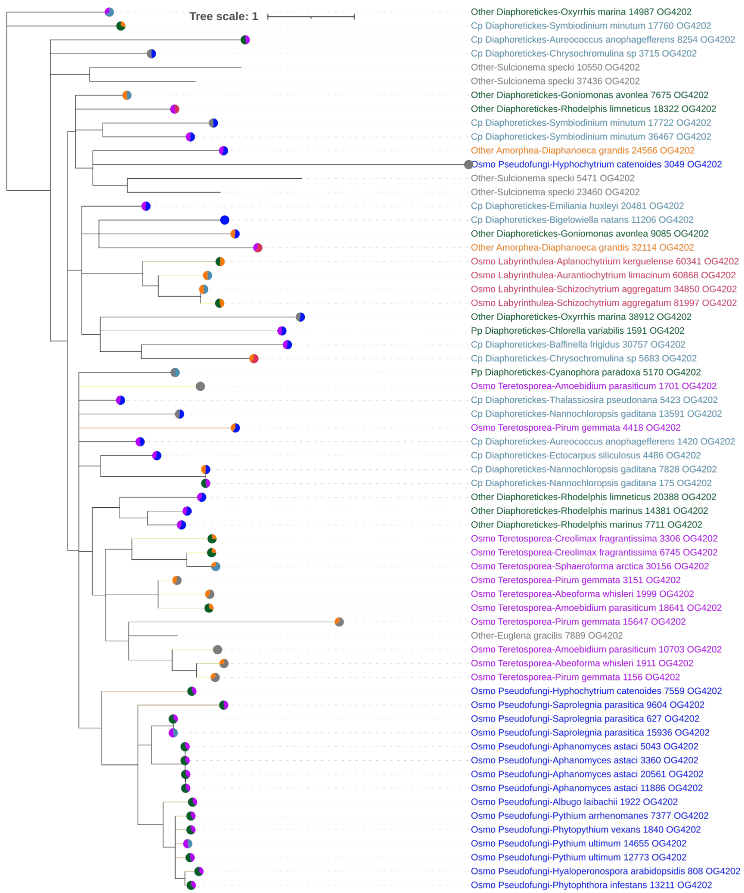

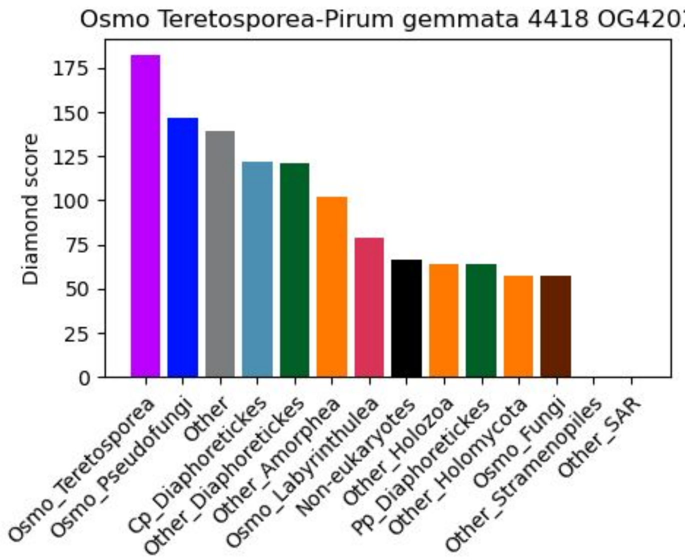

OG4202 The patchy distribution of the cluster, with Teretosporea and the choanoflagellate species *D. grandis* being the only representatives of the cluster in Holozoa (apart from the choanoflagellate species *D. grandis*), suggests HGT acquisition/s of this cluster by Teretosporea and by *D. grandis* (or just one HGT to the last common ancestor of Holozoa) from outside Amorphea, most likely from Pseudofungi

WDFY2 (0.02); U (0.41); negative regulation of epidermal growth factor-activated receptor activity (0.35)

Highly likely HGT

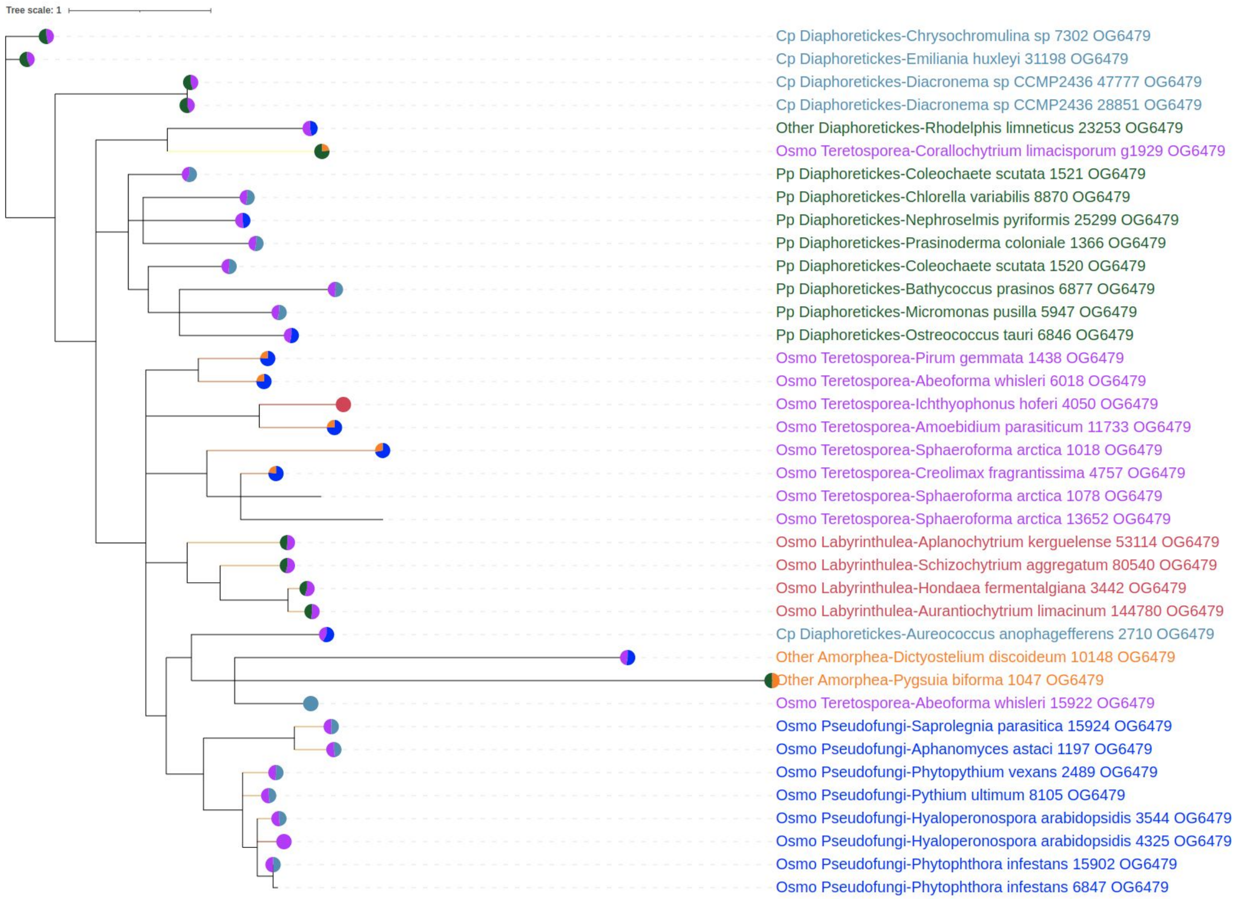

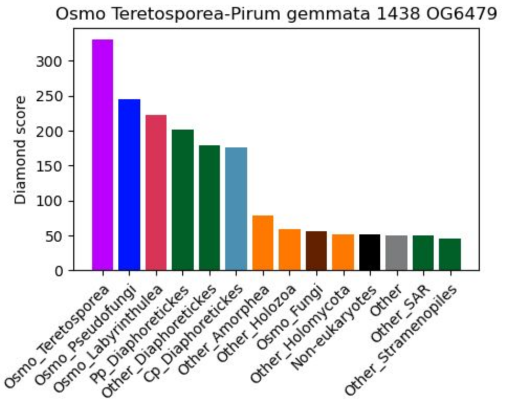

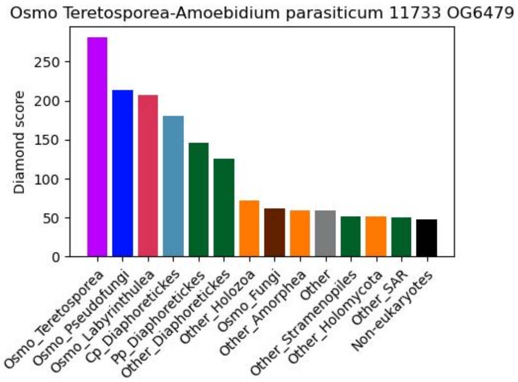

OG6479 Biased taxonomic distribution. In Amorphea, this cluster seems to be only in Teretosporea, and in two isolated amorphean taxa (which in fact present long branches, suggesting that these may perhaps be erroneous cluster members). The patchy distribution and the Diamond alignment results suggests HGT/s to Teretosporea, probably from Pseudofungi

AMACR (0.15); I (0.56); CoA-transferase family III (0.34)

Highly likely HGT

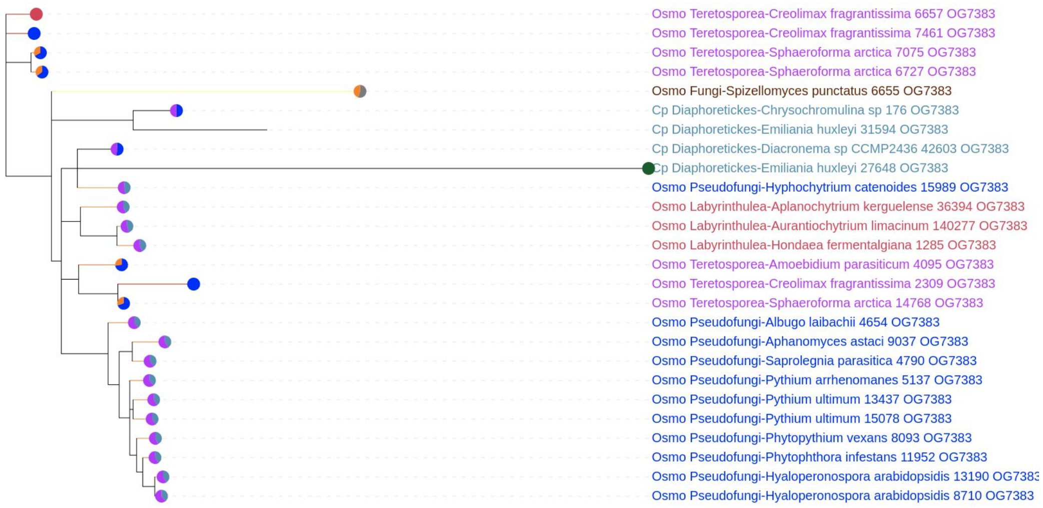

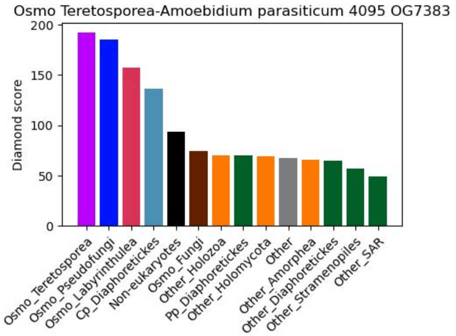

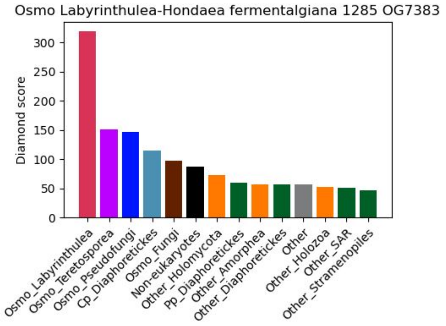

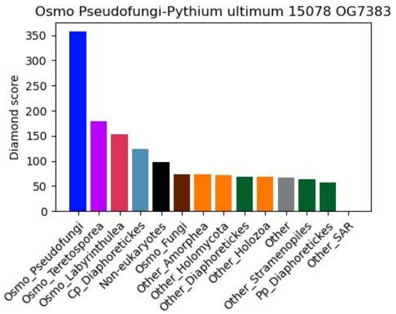

OG7383 This cluster has a taxonomically biased distribution. It has representatives in the four osmogroups, although the copy from Fungi (*S. punctatus*) is quite divergent and could be an outlier member of this cluster. The patchiness of the topology, as well as the Diamond alignment results, suggest HGTs involving osmogroups. Based on the taxonomic distribution, the most likely scenario is that Teretosporea acquired the gene from Diaphoretickes, probably from Pseudofungi.

- (0); - (0); Fatty acid hydroxylase superfamily (0.88)

Highly likely HGT

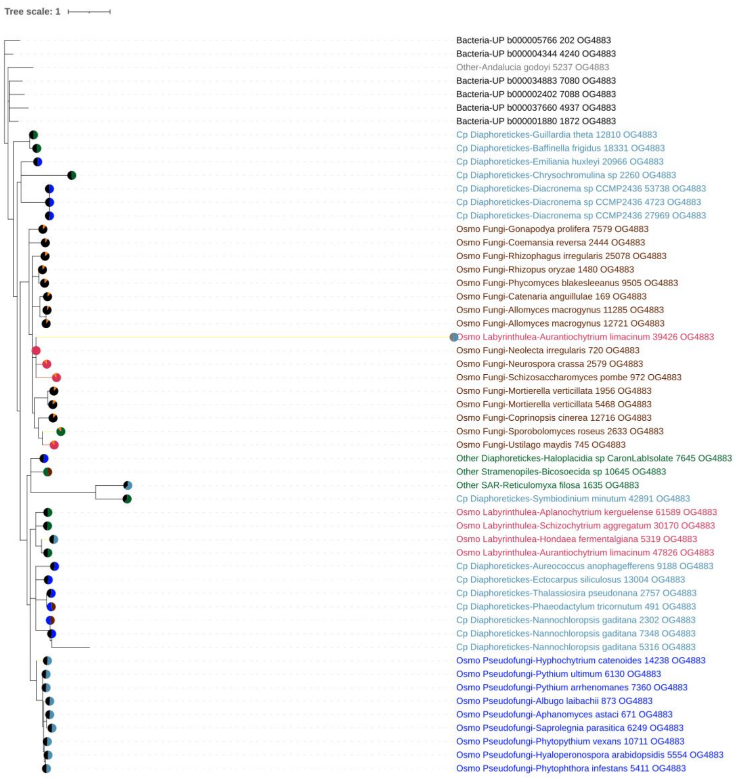

OG4883 In eukaryotes, this cluster is almost exclusive to Diaphoretickes, although it is also present in Fungi (Amorphea). Diamond alignment results suggest that Diaphoretickes acquired this gene family from Bacteria, and this would explain its presence in Pseudofungi and in Labyrinthulea. On the other hand, Fungi most likely acquired the gene by HGT, either from Bacteria or from Diaphoretickes

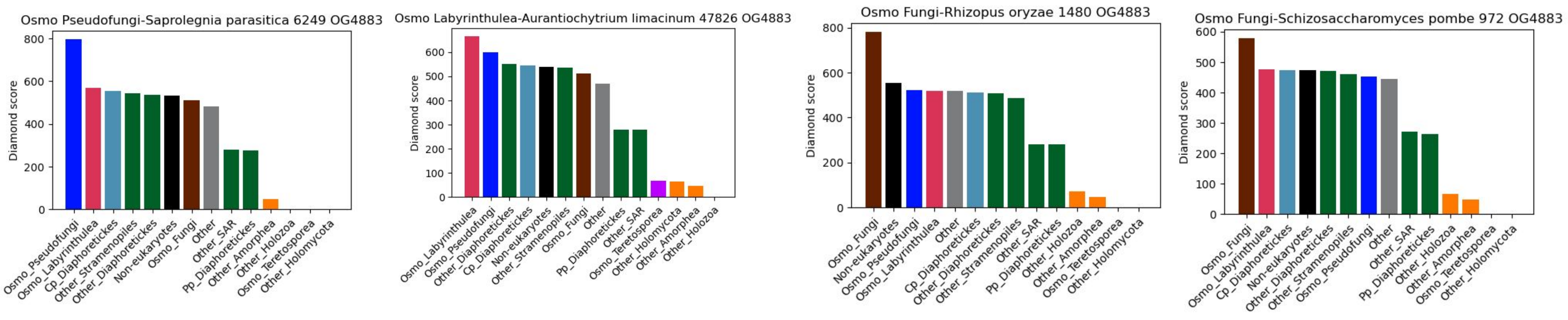

MA20_04470 (0.05); H (0.93); GTP cyclohydrolase N terminal (0.5)

Likely HGT

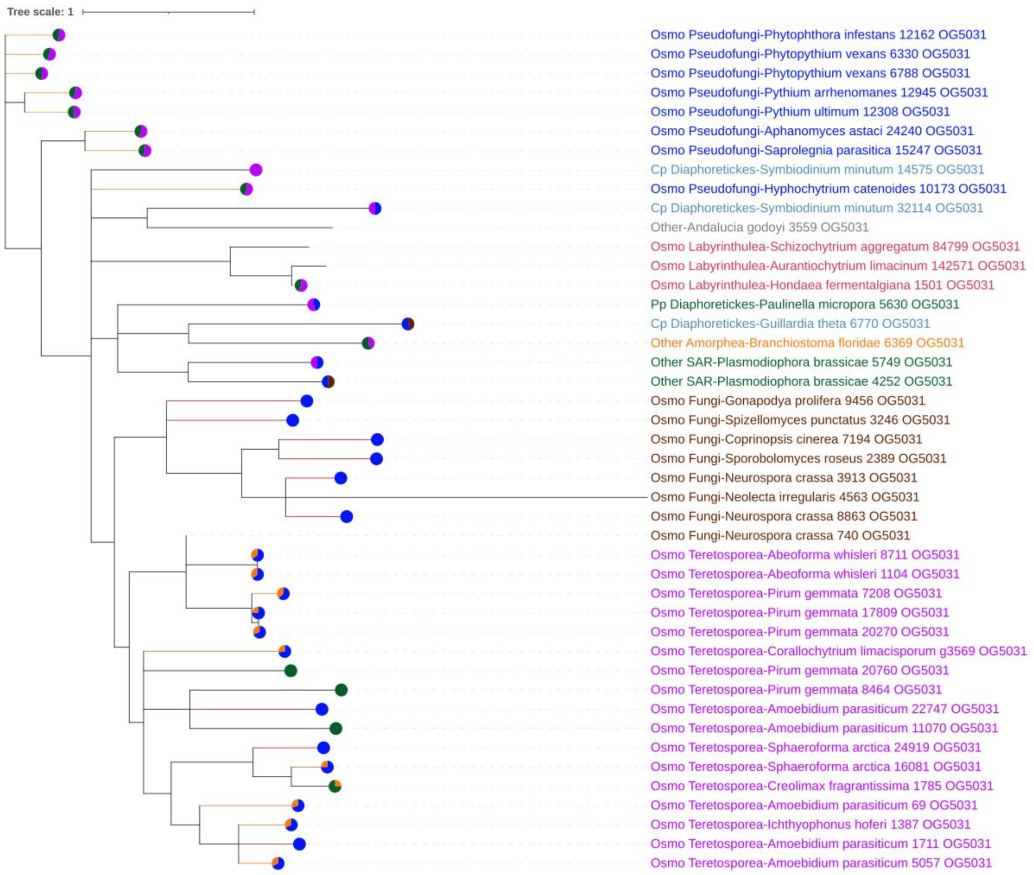

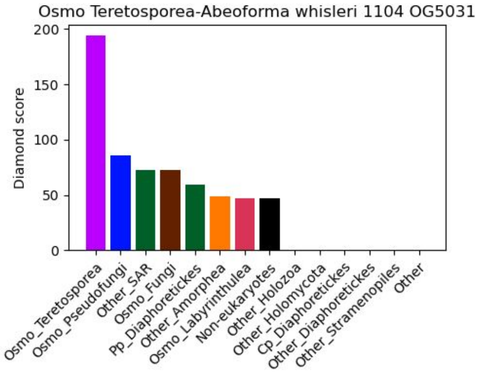

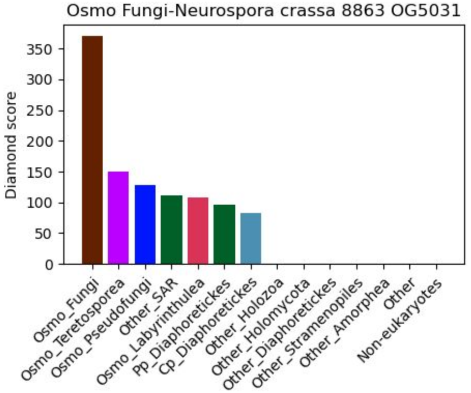

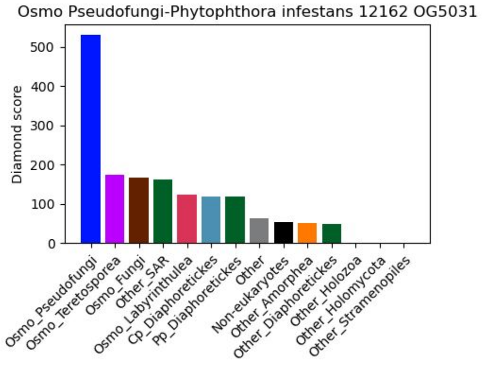

OG5031 The distribution of this cluster is largely biased to osmogroups. The patchiness of the taxonomic distribution, the topology, and the Diamond alignment results suggest HGT/s involving the osmogroups or the ancestors of the osmogroups. If there were only just one transfer, this would imply that the sequences in Teretosporea and Fungi have been vertically inherited from their last shared common ancestor (Opisthokonta). Still, we labeled this as a ‘Likely HGT’ instead of ‘Highly likely HGT’ since the Diamond alignment scores are generally lower

- (0); (0); LCCL domain-containing protein (0.39)

Highly likely HGT

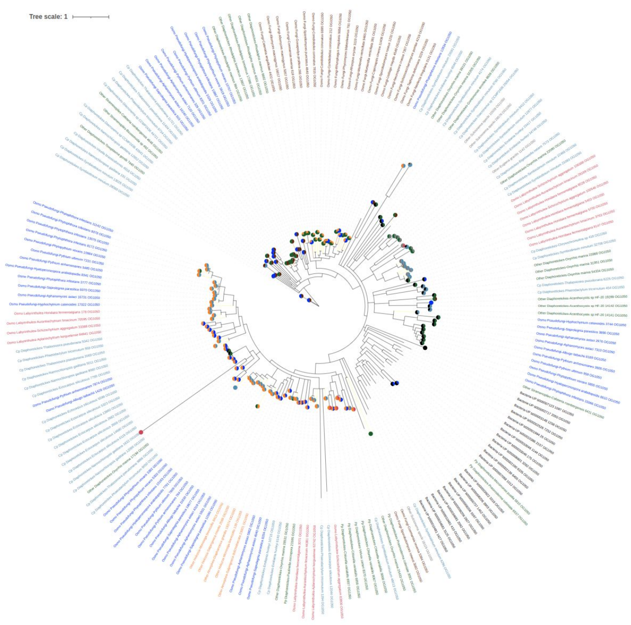

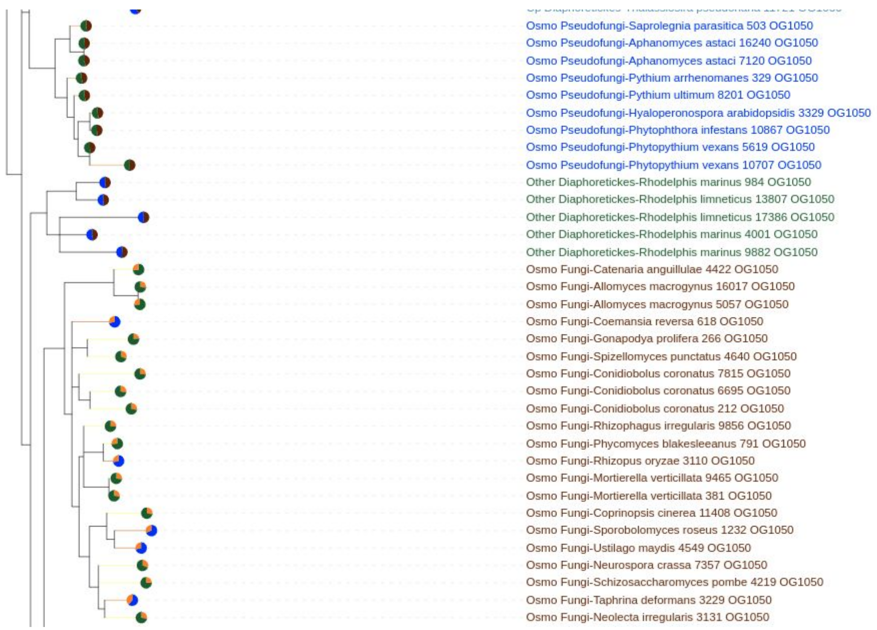

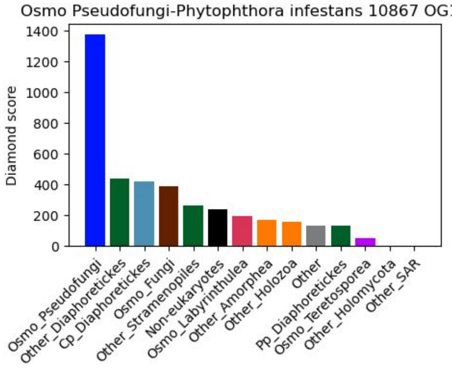

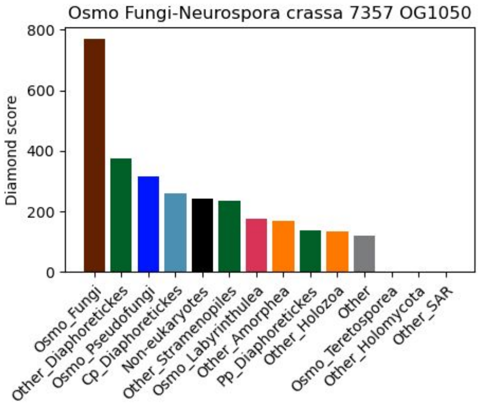

OG1050 The taxonomic distribution of this cluster is strongly biased to Diaphoretickes. The clade above -represented in the rightmost part of the slide-, in combination with the Diamond alignment results, suggests that Fungi acquired the gene from Diaphoretickes. Since the HGT would have been to LCA Fungi (based on the widespread fungal representation within the clade), this could explain the limited resolution to establish the specific HGT donor lineage from Diaphoretickes to Fungi

MA20_04470 (0.05); H (0.93); GTP cyclohydrolase N terminal (0.5)

Likely HGT

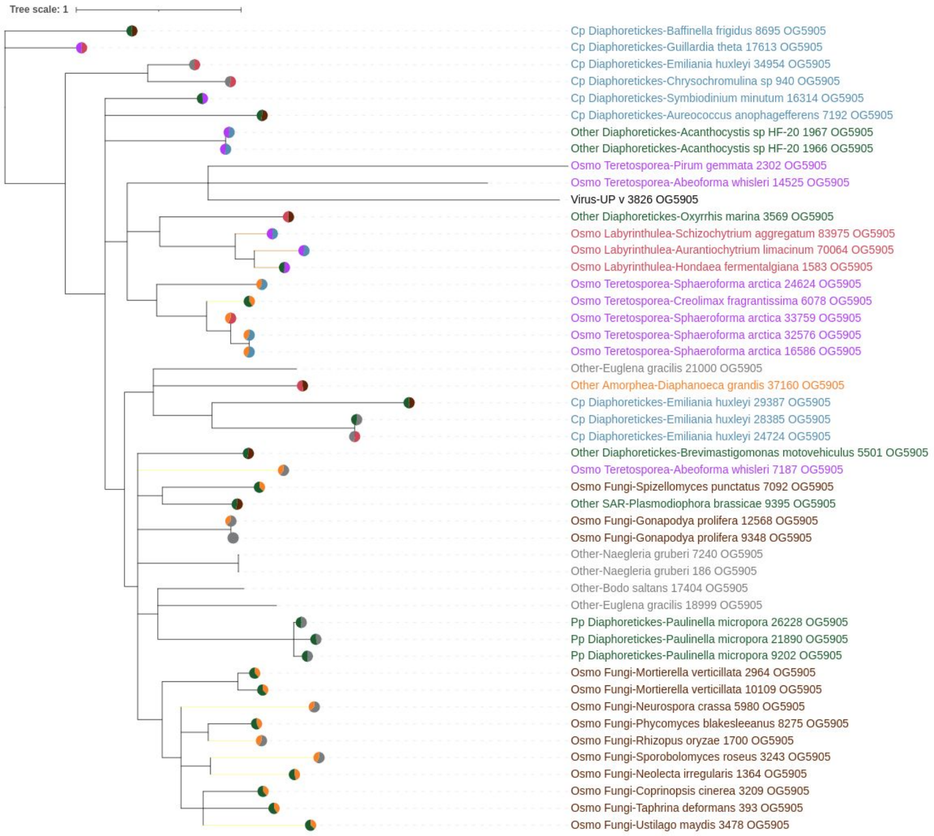

OG5905 This cluster is present in all osmogroups but Pseudofungi. Besides, it is also found in other Diaphoretickes. Within Amorphea, it is found in the two osmogroups (Fungi and Teretosporea), and in one choanoflagellate species (*D. grandis*). The patchy distribution of this family in Amorphea, which is in the line with the observed Diamond alignment results, suggest the potential occurrence of HGTs from Diaphoretickes to Amorphea. Still, we labeled this as a 'Likely HGT' instead of 'Highly likely HGT' since the Diamond alignment scores are generally lower

- (0); - (0); Mitochondrial 18 KDa protein (MTP18) (0.7)

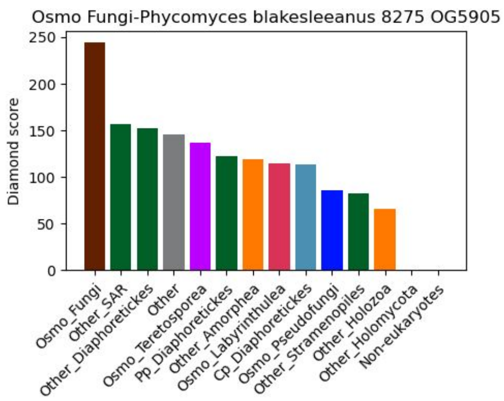

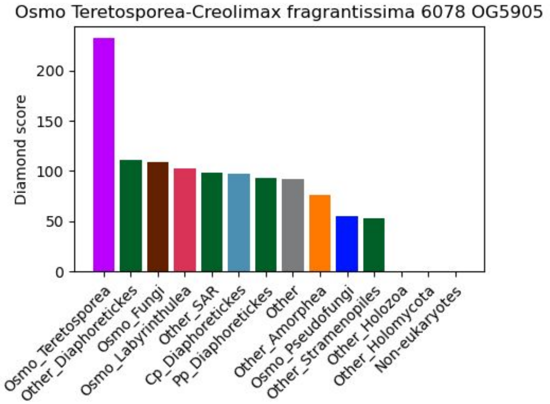

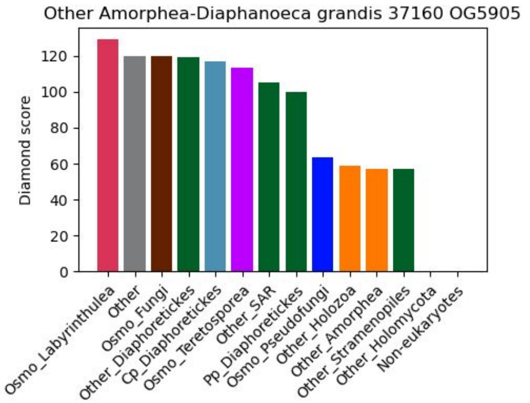

Likely HGT

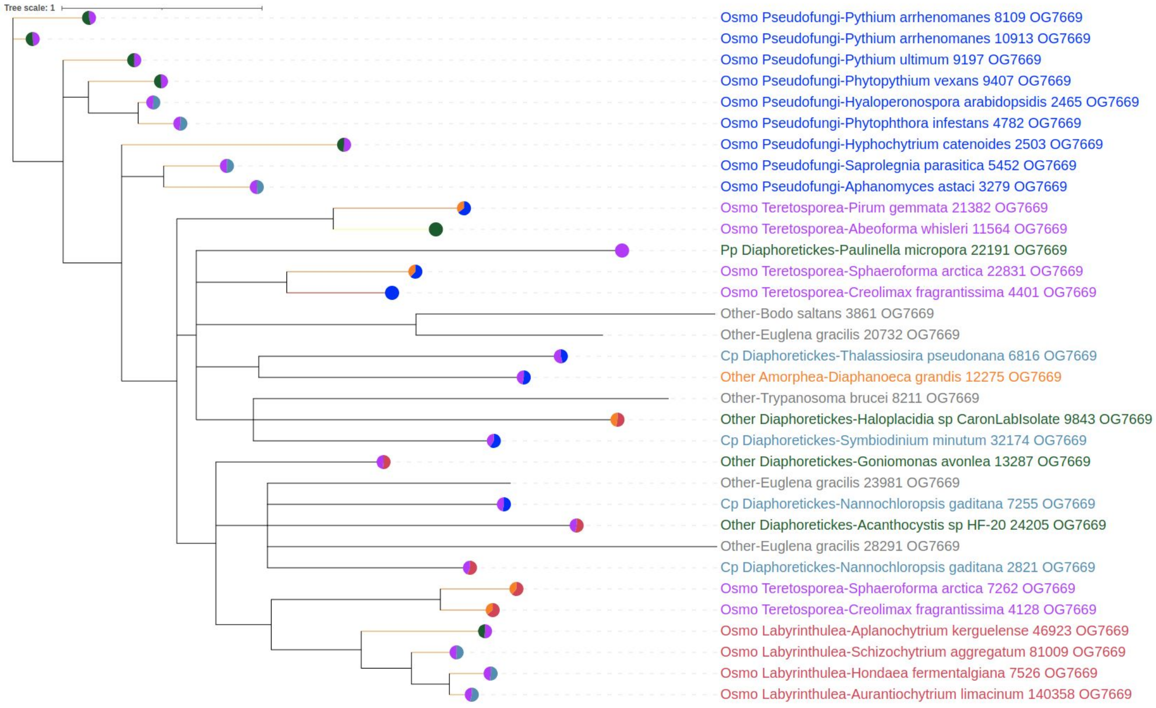

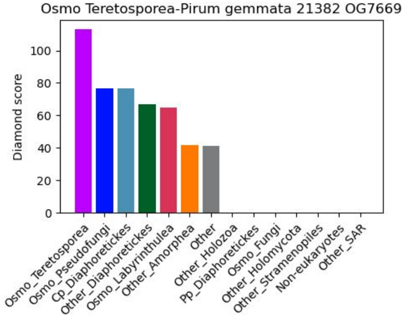

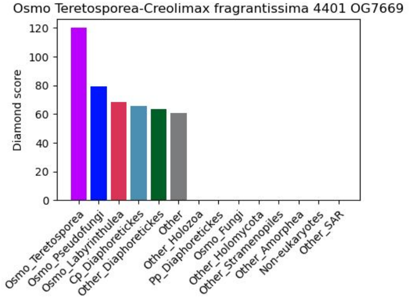

OG7669 The taxonomic distribution of the cluster is very patchy and biased towards osmogroups. In Amorphea, it is only present in Teretosporea and in one choanoflagellate species (*D. grandis*). The Diamond alignment results are concordant with the patchy distribution and both support HGTs involving osmogroup taxa. While the alignment score is overall low, which does not allow to discard potential false positive issues, the fact that the taxonomic distribution is so biased to osmogroups leaves as unlikely the possibility that the presence of this sequence in Teretosporea is explained by vertical inheritance from a shared ancestor with the Diaphoretickes (i.e., from the last shared eukaryotic common ancestor). Still, we labeled this as a ‘Likely HGT’ instead of ‘Highly likely HGT’ since the Diamond alignment scores are generally lower

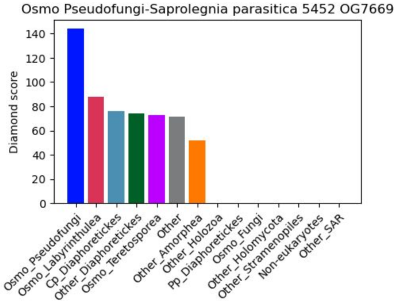

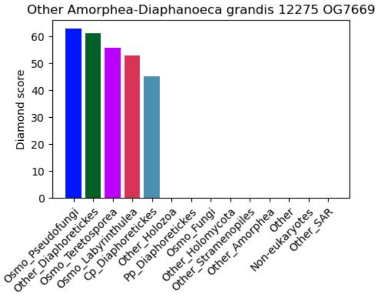

- (0): - (0): Source PGD (0.38)

Likely HGT

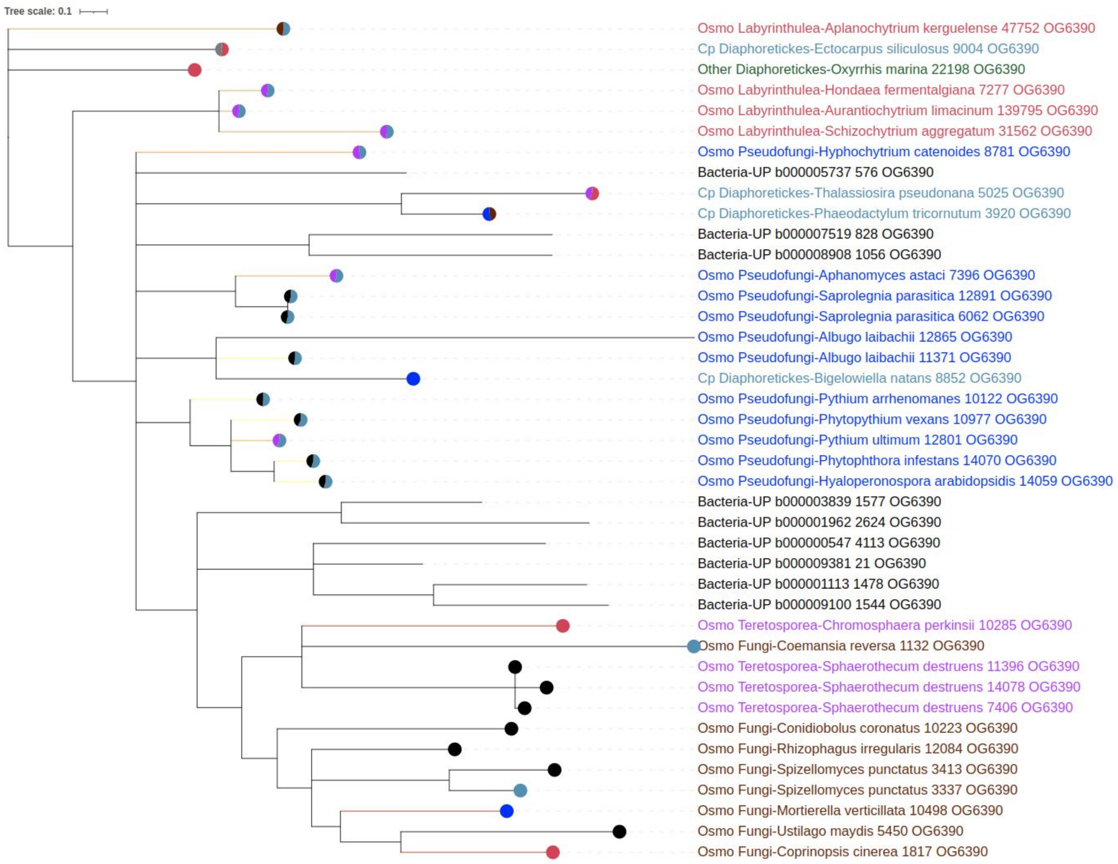

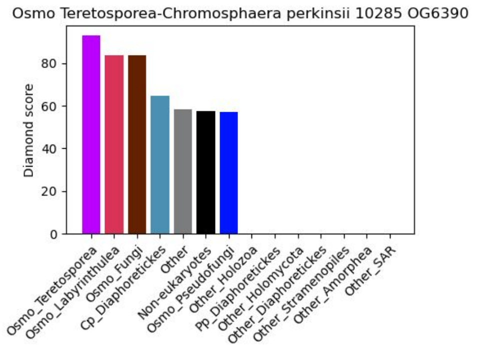

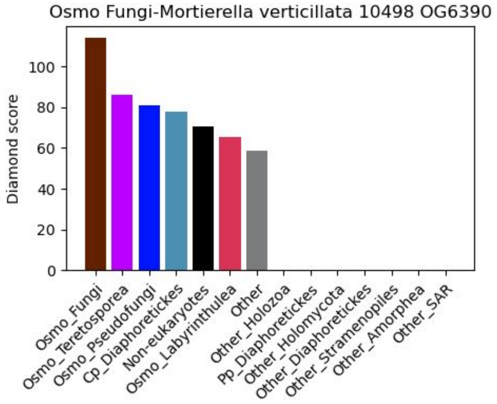

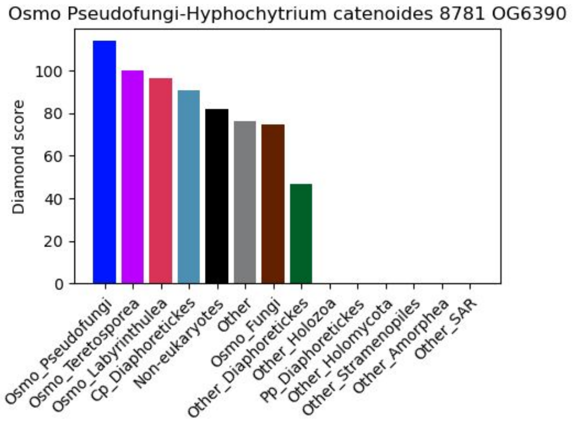

OG6390 The distribution of the cluster is strongly biased towards osmogroups. In Amorphea, it is only present in osmogroups. The low level of sequence conservation limits the possibility to confidently propose HGTs involving osmogroups, as Bacteria could have been acted as a donor instead. In any case, the taxonomic distribution and Diamond results suggests that this gene was convergently acquired by osmogroups and/or some of their ancestors, with independence on whether the donor was Bacteria or some other osmogroup/eukaryote.

- (0); - (0); DinB superfamily (0.17)

Likely HGT

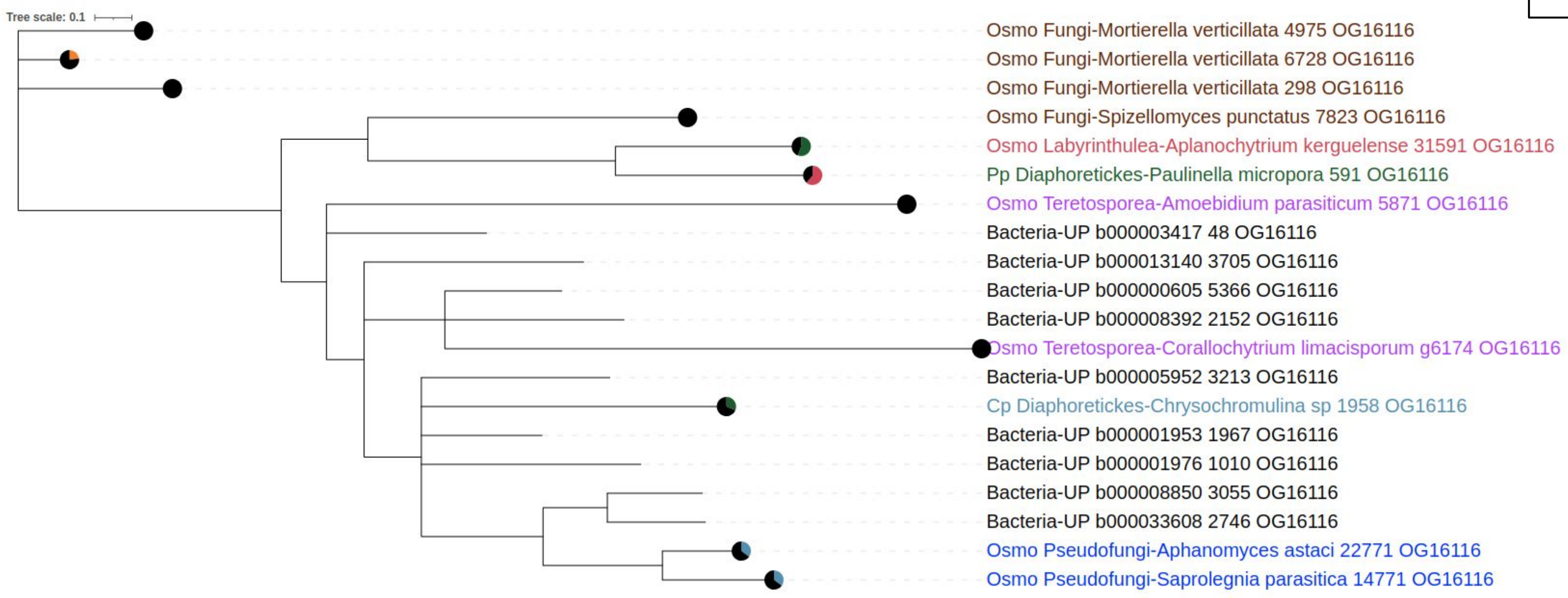

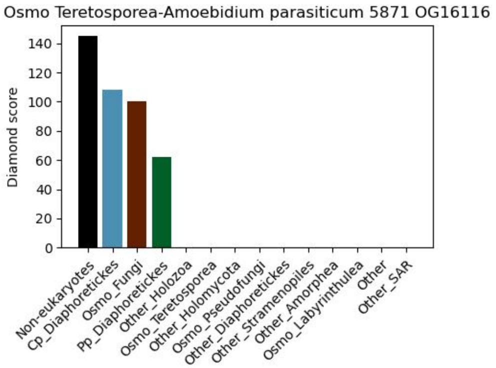

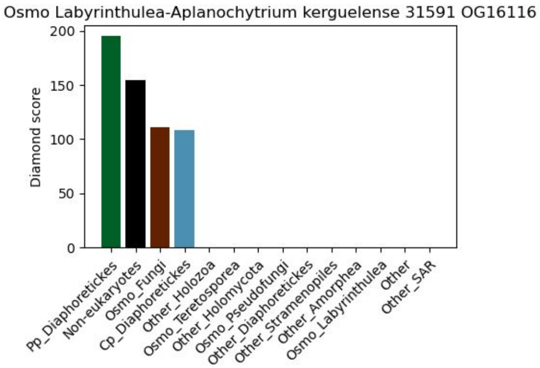

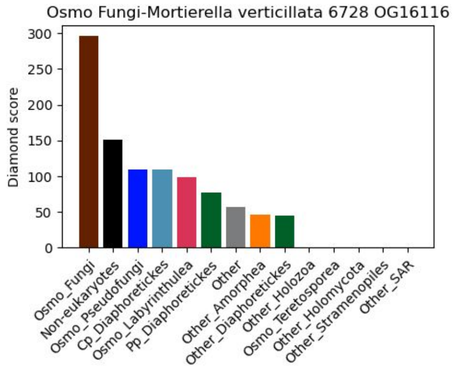

OG16116 This cluster was probably transferred multiple times from Bacteria to specific taxa from osmogroups.

yfcF (0.3); O (1.0); Glutathione S-transferase, N-terminal domain (0.6)

Highly likely HGT

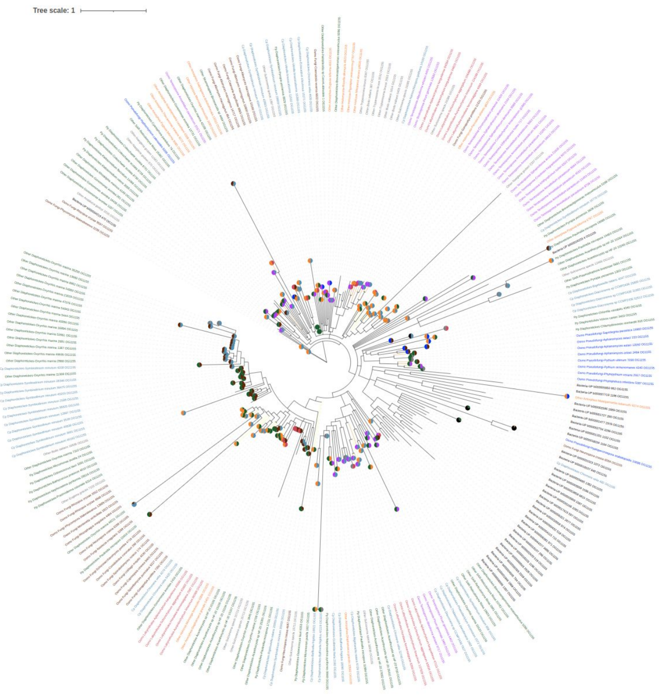

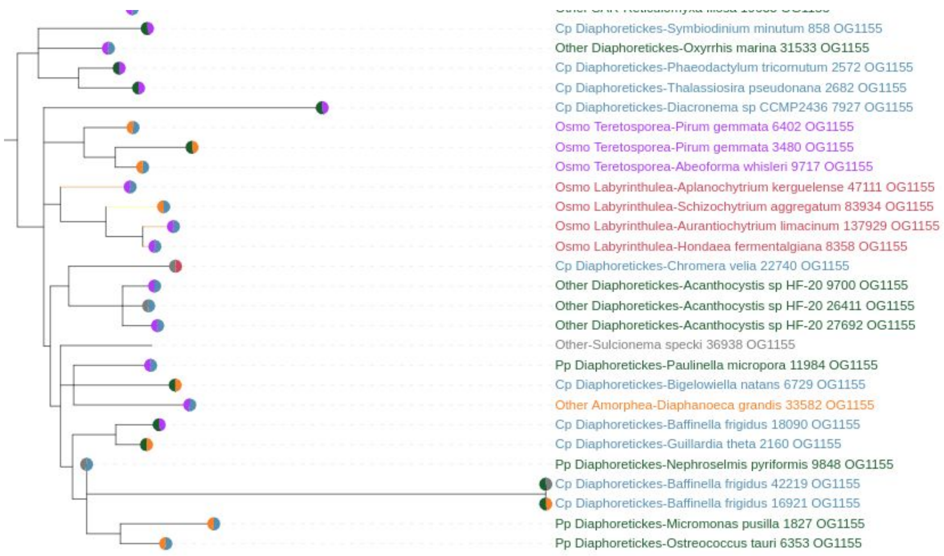

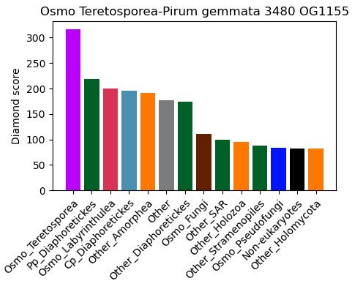

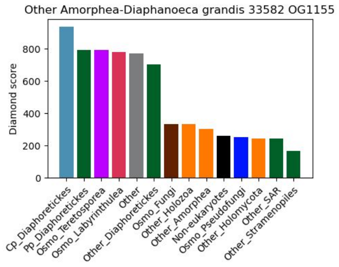

OG1155 A medium size gene family. One clade of the phylogeny shows a pattern which is strongly suggestive of HGT from Diaphoretickes to the *Pirum*+*Abeoforma* clade from Teretosporea (Amorphea), and to one choanoflagellate species (*Diaphanoeca grandis*), also from Amorphea. This trend is corroborated by the Diamond alignments. It is more clearly observed in the '*Diaphanoeca grandis*' alignment barplot, as it can be seen that it aligns with a much higher score with everything outside amorphea except for the teretosporean taxa which are within that same clade.

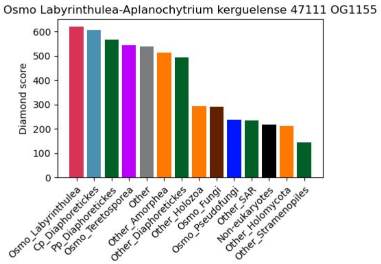

OSM2 (0.23) C (0.96) fumarate reductase (NADH) activity (0.27)

Likely HGT

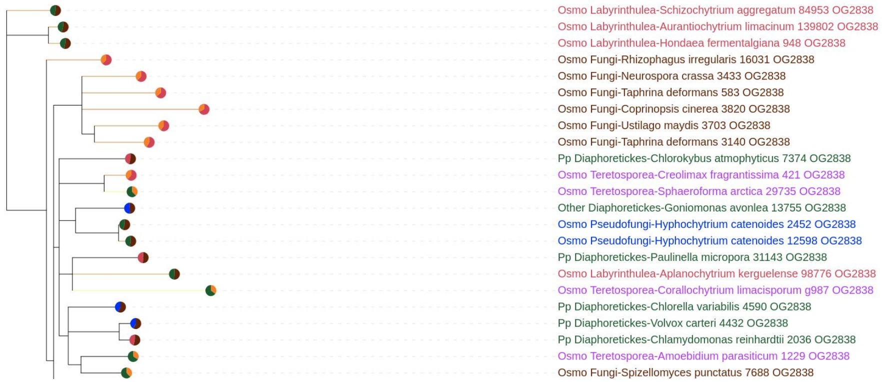

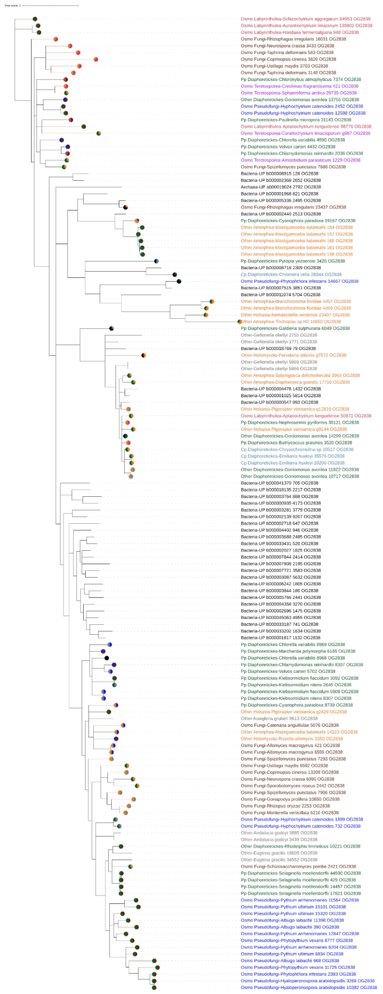

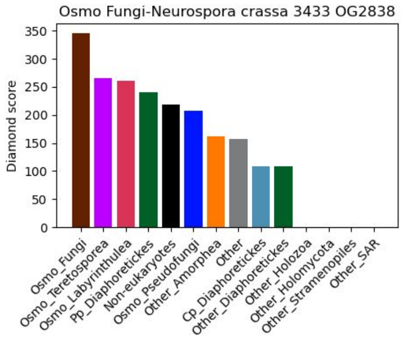

OG2838 A quite widespread gene family, which has a clade consisting of a possibly HGT path between the osmogroup Labyrinthulea (Diaphoretickes) and taxa from two osmogroups from Amorphea (Fungi and Teretosporea). Diamond alignment results are aligned with the described potential HGT events

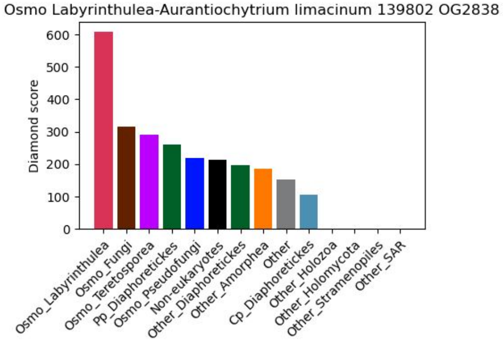

fdhA (0.04) Q (0.81) Alcohol dehydrogenase GroES-like domain (0.36)

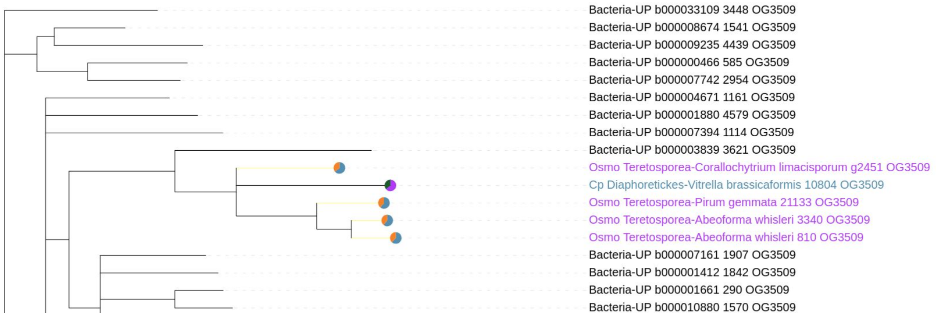

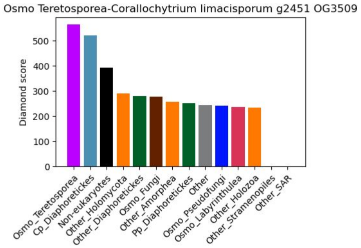

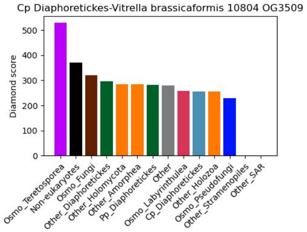

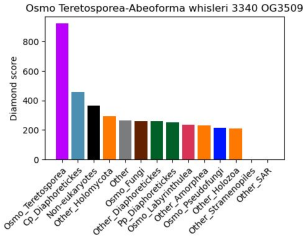

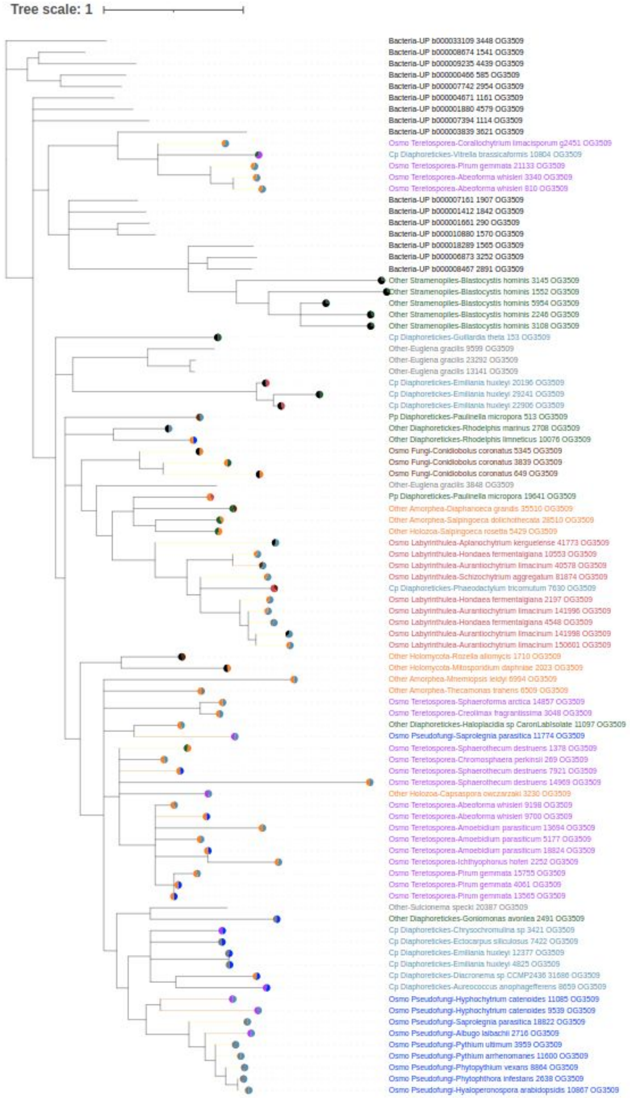

OG3509 This gene family is quite widespread in eukaryotes. Still, above there is a clade of sequences, mostly from Bacteria, which include a subclade of eukaryotic sequences. This subclade includes 4 sequences from 3 species from Teretosporea (Amorphea), and one sequence from *V. brassicaformis* (Diaphoretickes). It is likely that Bacteria transferred this gene to Teretosporea, and then there was a second HGT from Teretosporea to a lineage related to *V. brassicaformis*

nhaC (0.19) C (0.94) Na+/H+ antiporter family (0.75)

Likely HGT

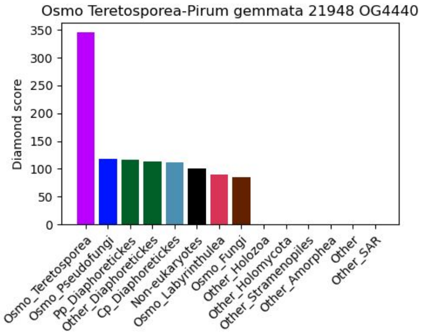

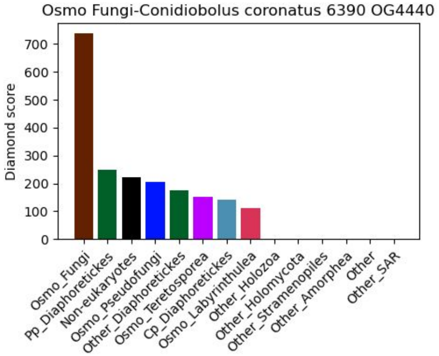

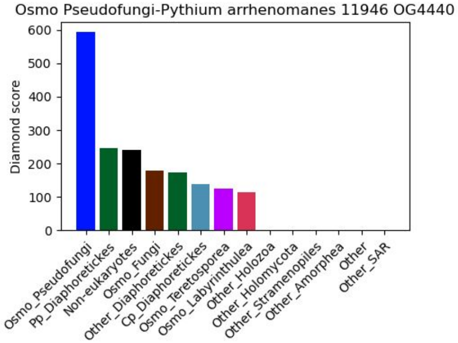

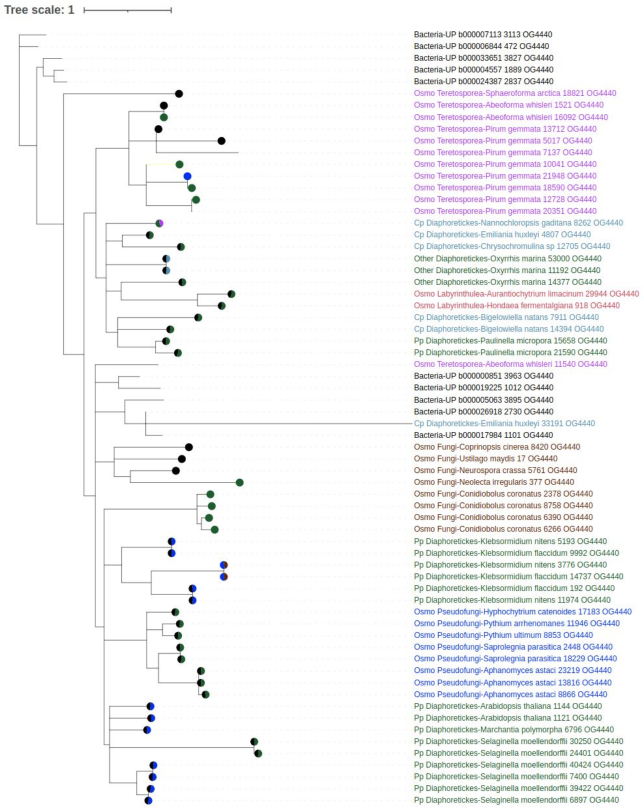

OG4440 This gene family is quite widely distributed across Diaphoretickes. Within Amorphea, the distribution is quite narrow: it is only found in Teretosporea and in Fungi. HGTs either from Diaphoretickes or from Bacteria to species from the two osmogroups from Amorphea is thus quite likely.

- (0) - (0) PFAM Peptide N-acetyl-beta-D-glucosaminyl asparaginase amidase A (0.56)

Highly likely HGT

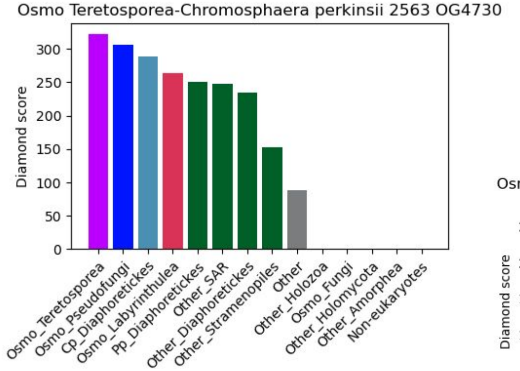

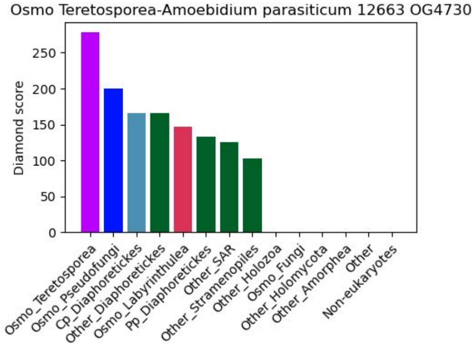

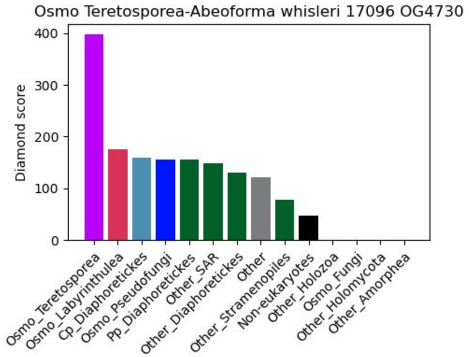

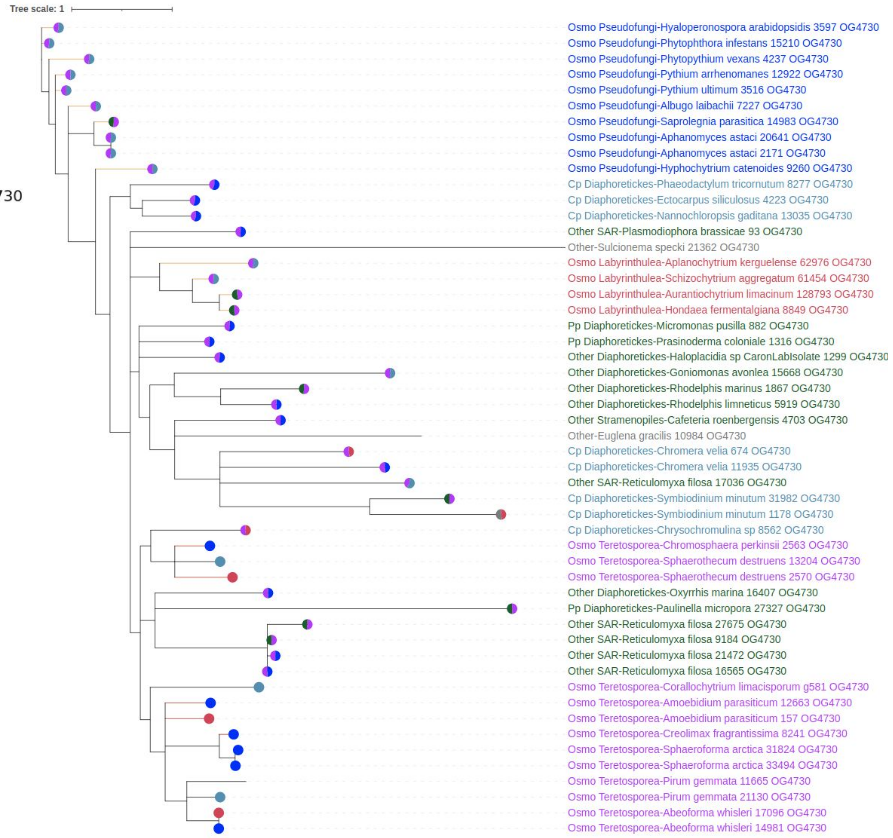

OG4730 This gene family is quite widely distributed in Diaphoretickes. In Amorphea, it is only found in Teretosporea. Teretosporea very likely acquired this gene from Diaphoretickes, possibly through Pseudofungi, Labyrinthulea, or some common ancestor of both osmogroups

- (0) - (0) A distinct subfamily of CDD/CDA-like deaminases (0.7)

Highly likely HGT

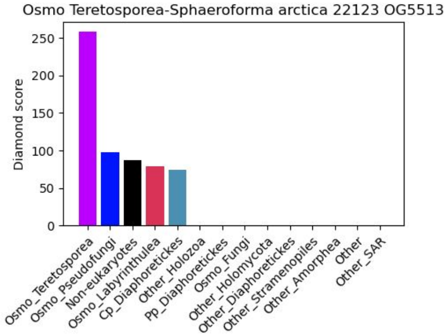

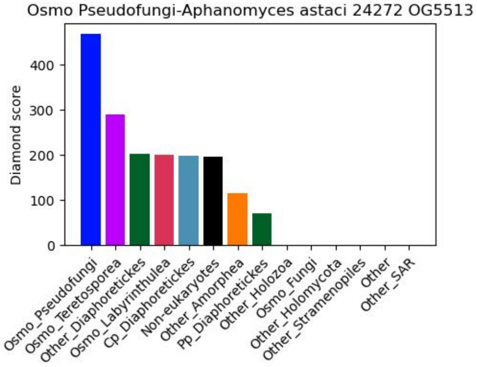

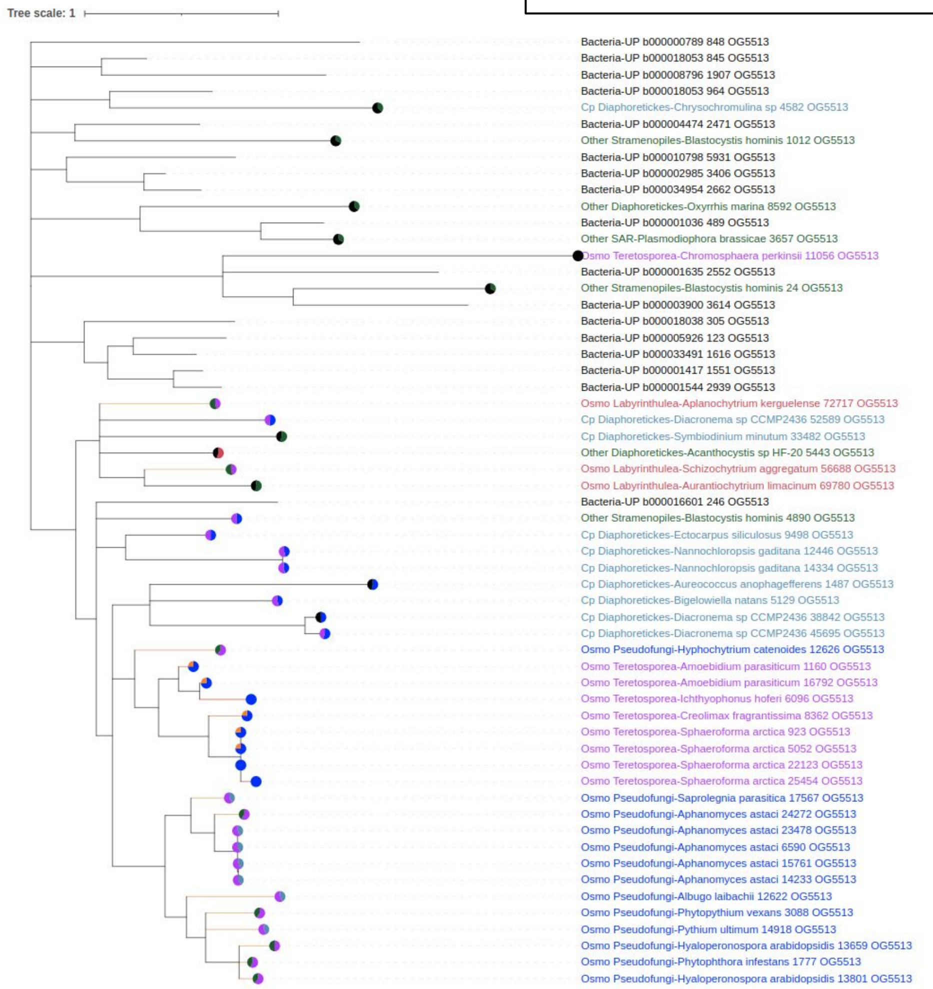

OG5513 The taxonomic distribution of this gene is quite patchy. At the same time, Bacteria is distributed across the phylogeny, instead of branching within a single clade. It is thus likely that this gene has been transferred multiple times from Bacteria to eukaryotes. Still, the Diamond alignment results and the bottom of the phylogeny strongly suggest that Teretosporea acquired this gene from Diaphoretickes through HGT, most likely from Pseudofungi

GNE (0.47) G (0.53) UDP-N-acetylglucosamine 2-epimerase activity (0.33)

Highly likely HGT

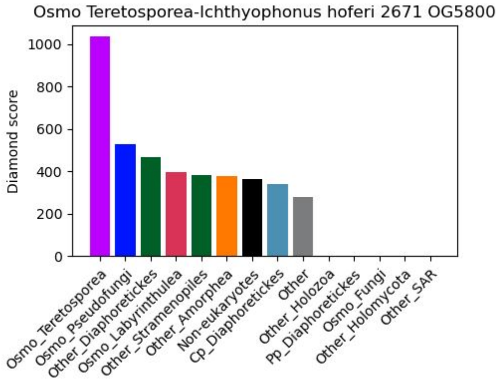

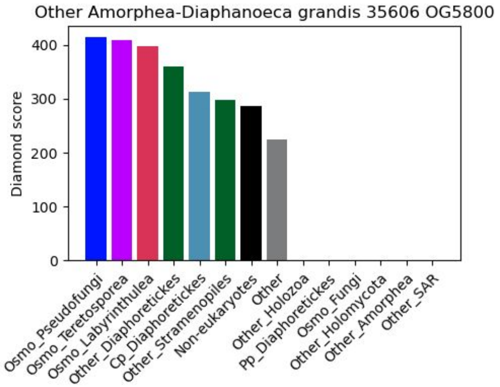

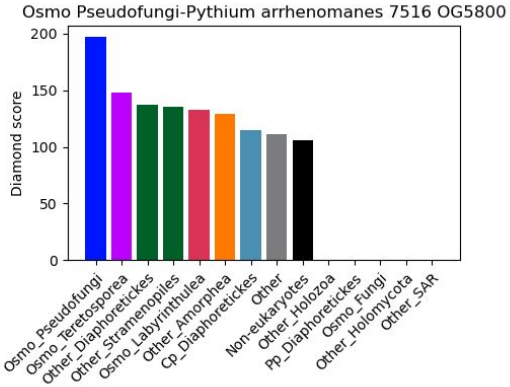

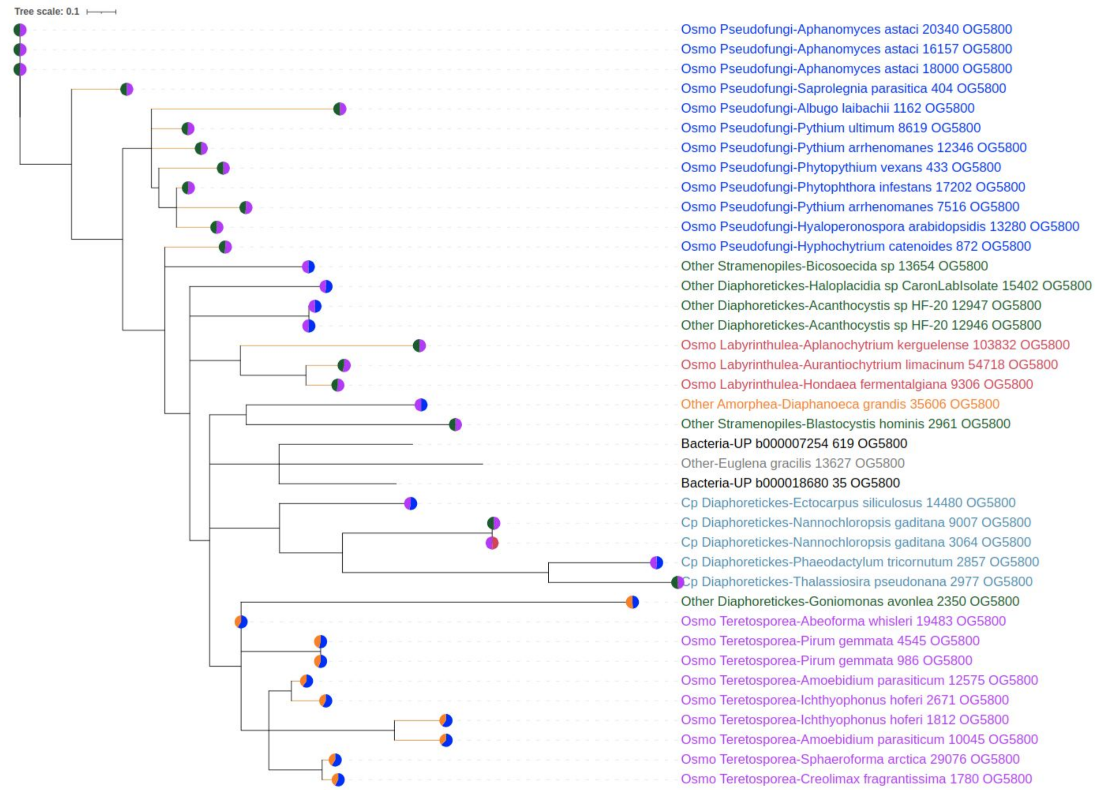

OG5800 This gene family is more widely distributed in Diaphoretickes than in Amorphea. In Amorphea, it is only found in Teretosporea and in one choanoflagellate species. It is likely that the gene was transferred from Diaphoretickes either twice to Teretosporea and to a lineage related to the choanoflagellate species (*D. grandis*), or it was transferred once to the last common ancestor of Teretosporea and choanoflagellates (Holozoa), most likely from either Pseudofungi, Labyrinthulea, or a common ancestor of both groups. Bacteria being the HGT donor is possible but less likely based on the topology and the Diamond alignment results

GID8 (0.06) L (0.06) Domain of unknown function (DUF4954) (0.26)

Highly likely HGT

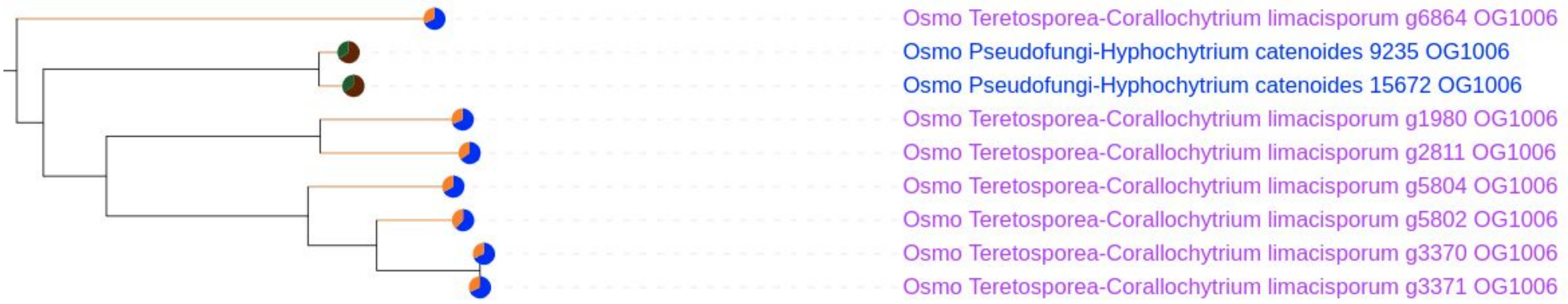

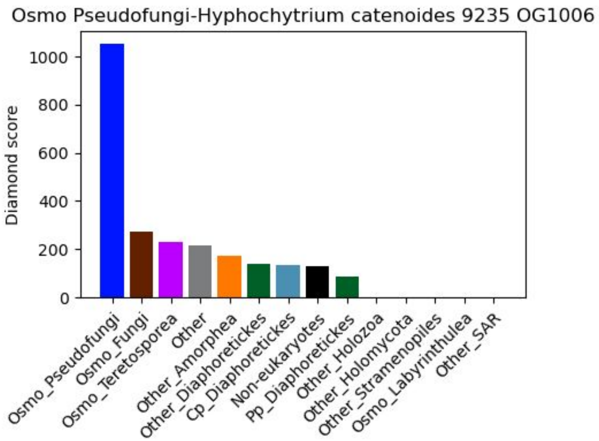

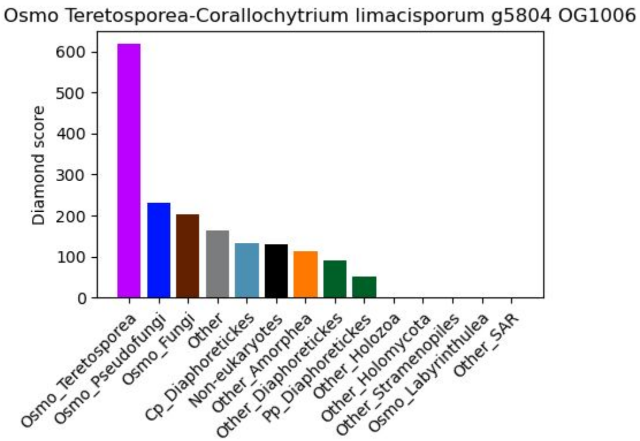

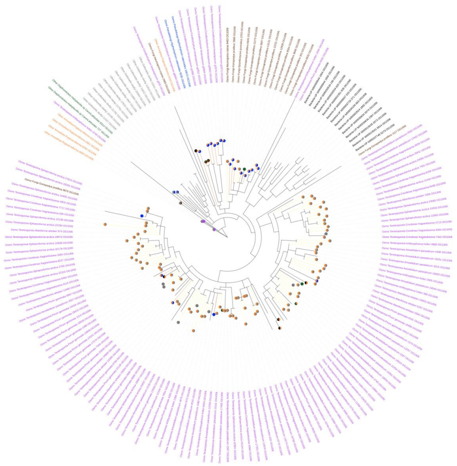

OG1006 This is a patchily distributed gene family, with a substantial expansion in Teretosporea. There is a clade which suggest a HGT from a lineage related to *C. limacisporum* (Teretosporea) to a lineage related to *H. catenoides* (Pseudofungi). The HGT acquisition of this gene by *H. catenoides* is highly likely given that beyond this phylogenetic pattern, this gene is not found in the relative lineages of *H. catenoides*.

- (1.0) S (1.0) Sphaeroforma arctica JP610 (0.75)

Likely HGT

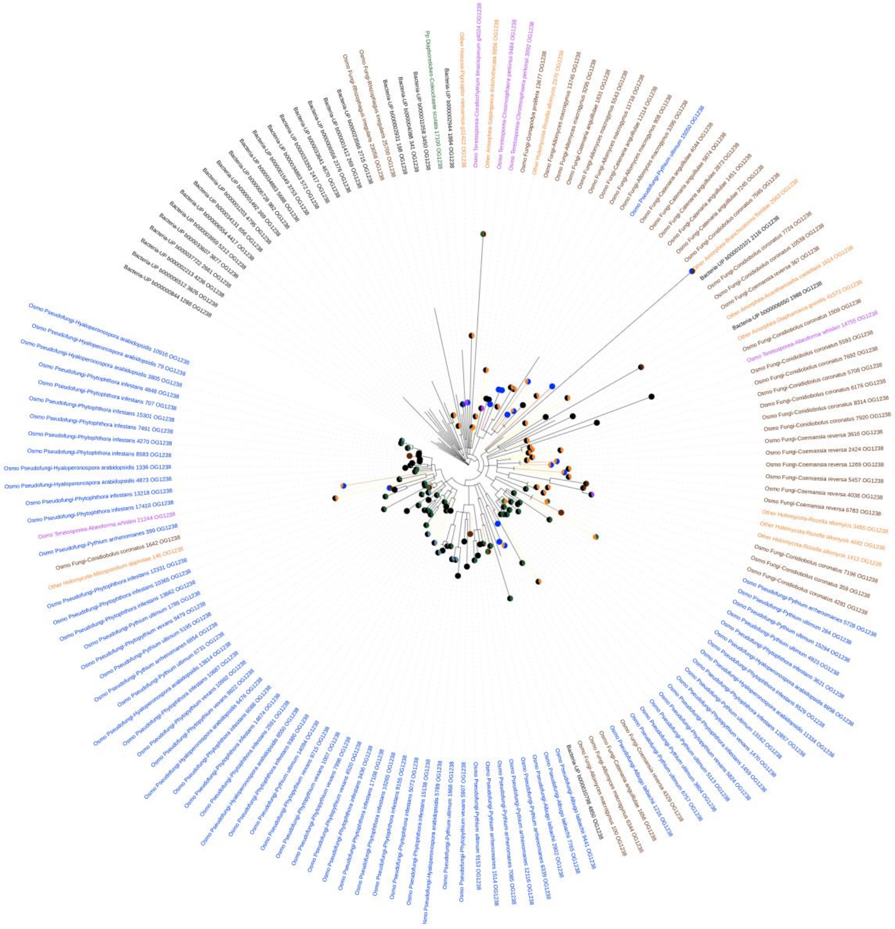

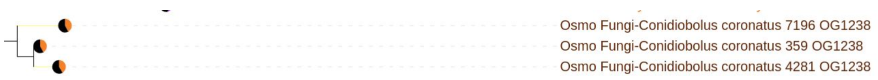

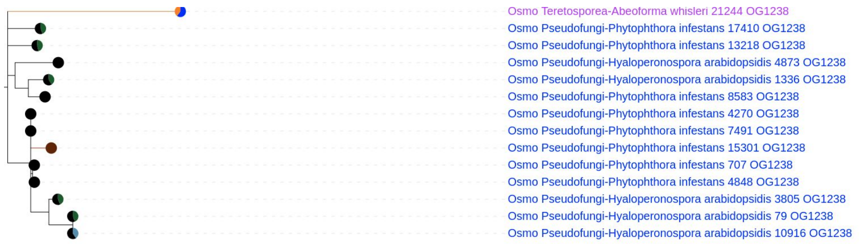

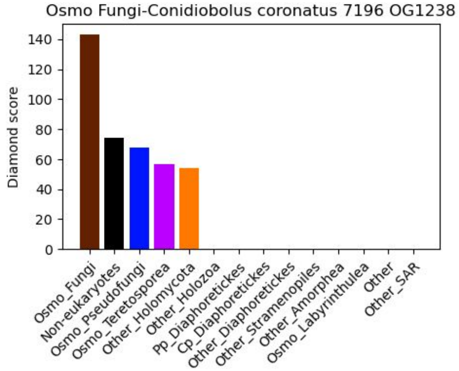

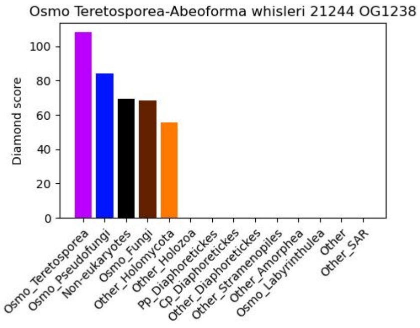

OG1238 This gene family is mainly present, and in high copy numbers, in the osmogroups Pseudofungi and Fungi. It is also present in Teretosporea and in a few other species. Bacterial sequences are not monophyletic in the phylogeny. The phylogeny and the Diamond alignment suggests HGTs, possibly from Bacteria multiple times to eukaryotes. We consider this as a ‘Likely HGT’ but not a ‘Highly likely HGT’ case due to the low Diamond score values limits the resolution to fully clarify the evolutionary history of this gene family

- (0.99) S (0.89) Cysteine-rich secretory protein family (0.58)

Highly likely HGT

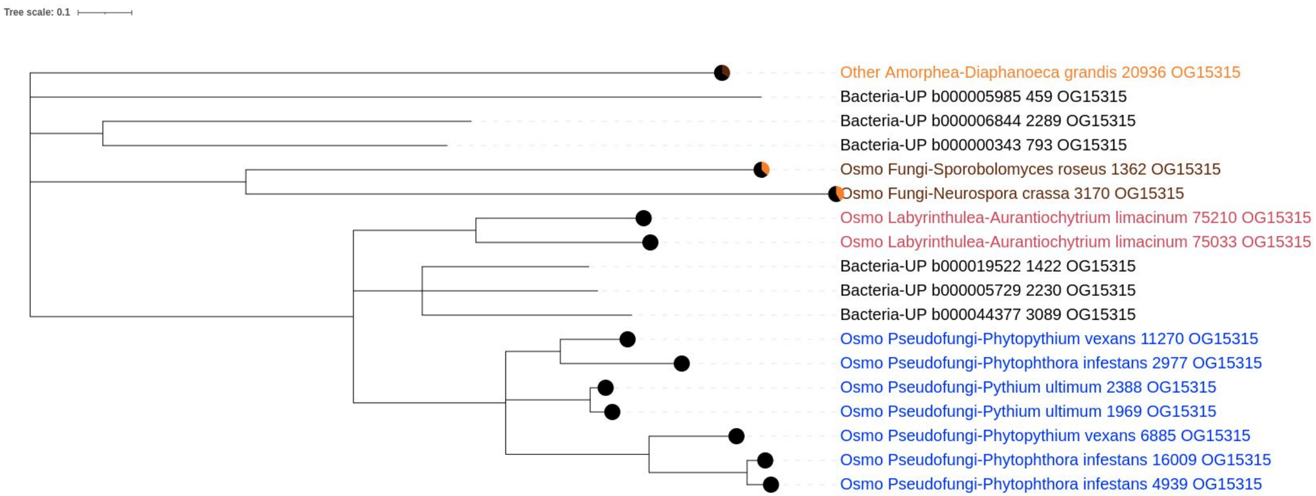

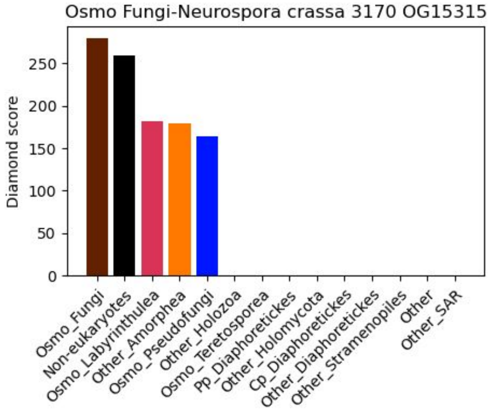

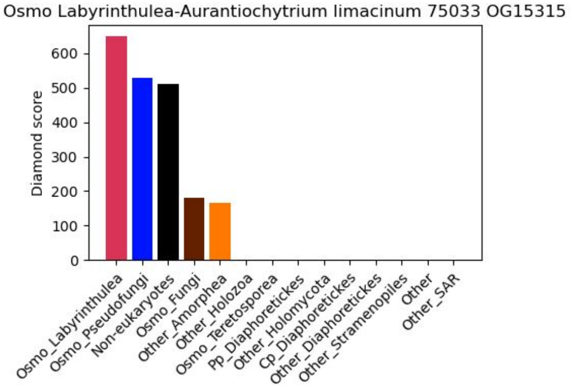

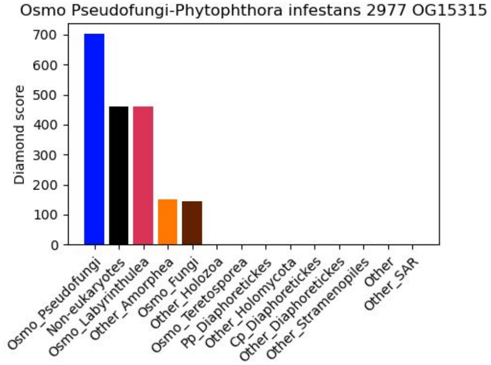

OG15315 This gene family is patchily distributed. It has likely been transferred different times from Bacteria to subclades within three different osmotrophic groups.

- (1.0) G (0.55) Tannase and feruloyl esterase (0.82)

Highly likely HGT

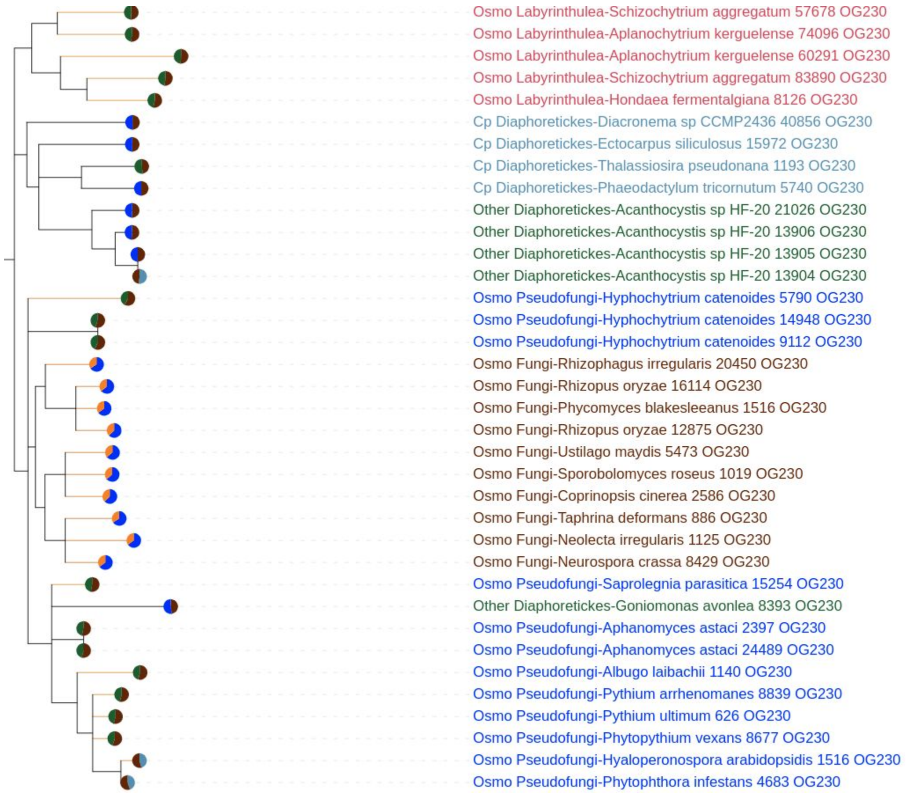

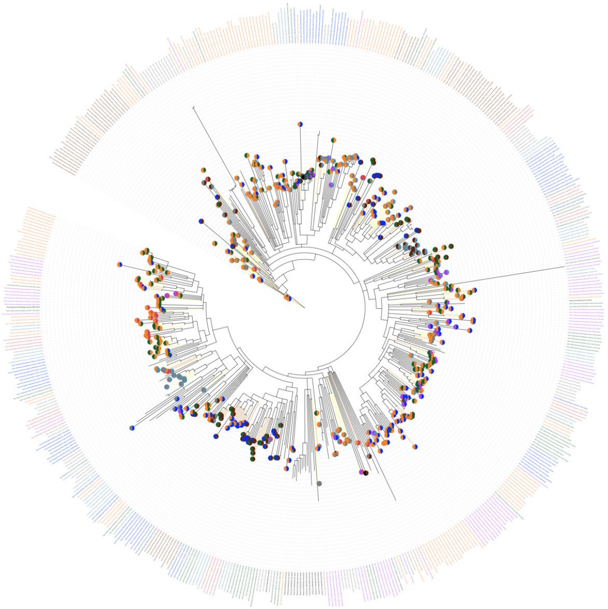

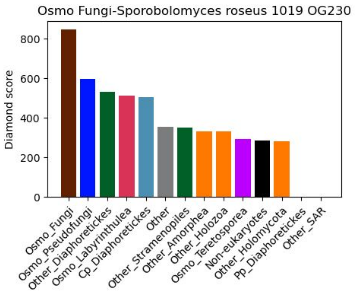

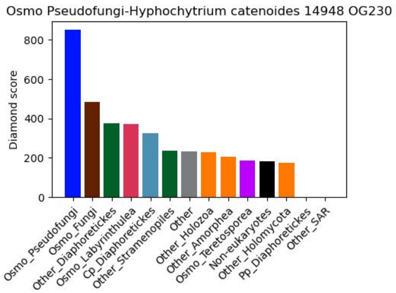

OG230 This gene family is widely distributed. Still, there is a paralog clade which seem to have been acquired by Fungi clearly from Diaphoretickes, most likely from Pseudofungi (*H. catenoides*' lineage?).

- (0.45) I (0.97) Belongs to the carnitine choline acetyltransferase family (0.35)

Likely HGT

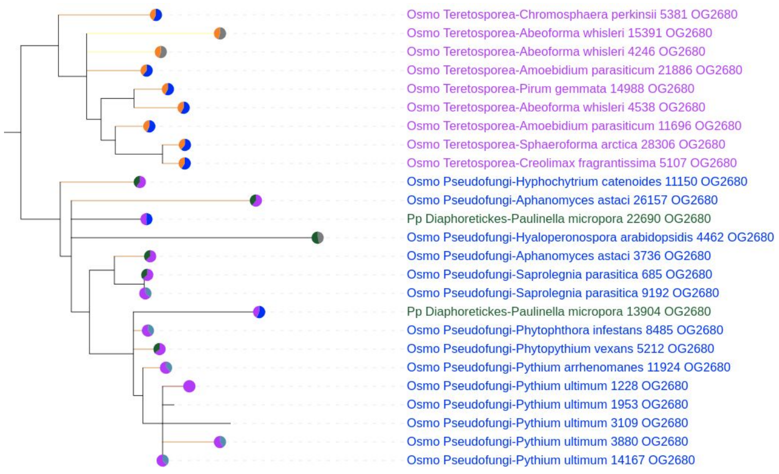

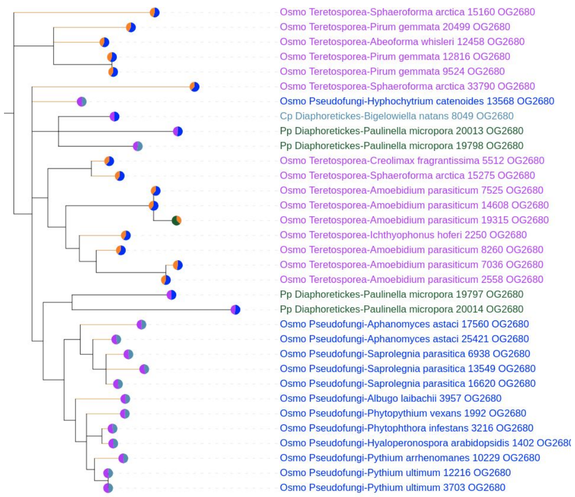

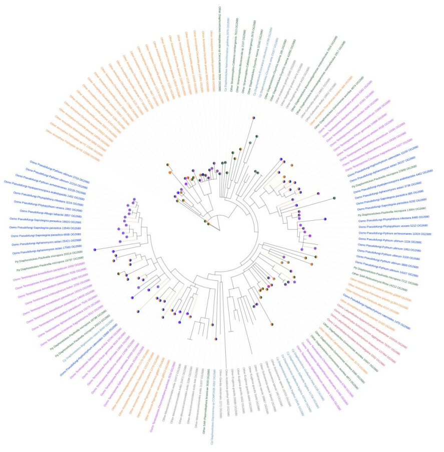

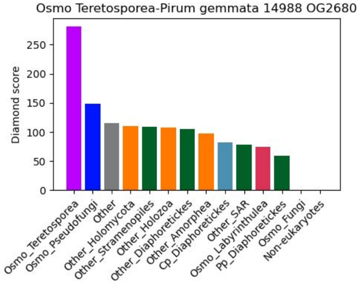

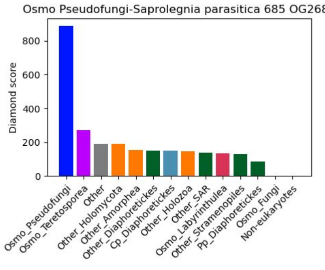

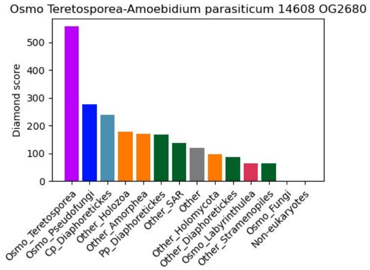

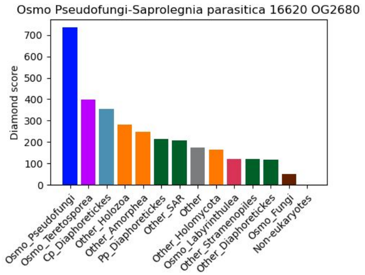

OG2680 This gene family is quite widely distributed. Still, there are two clades of sequences from Teretosporea which seem to be related to sequences from Diaphoretickes; a HGT involving Teretosporea and Pseudofungi likely took place. We labeled it not as a ‘Highly likely HGT’ but as ‘Likely HGT’ since the Diamond score difference is not so high such as to completely discard that these phylogenetic associations could be due to stochastic higher sequence similarity between the Pseudofungi and the Teretosporea sequences

- (1.0) S (1.0) Transmembrane amino acid transporter protein (0.77)

Likely HGT

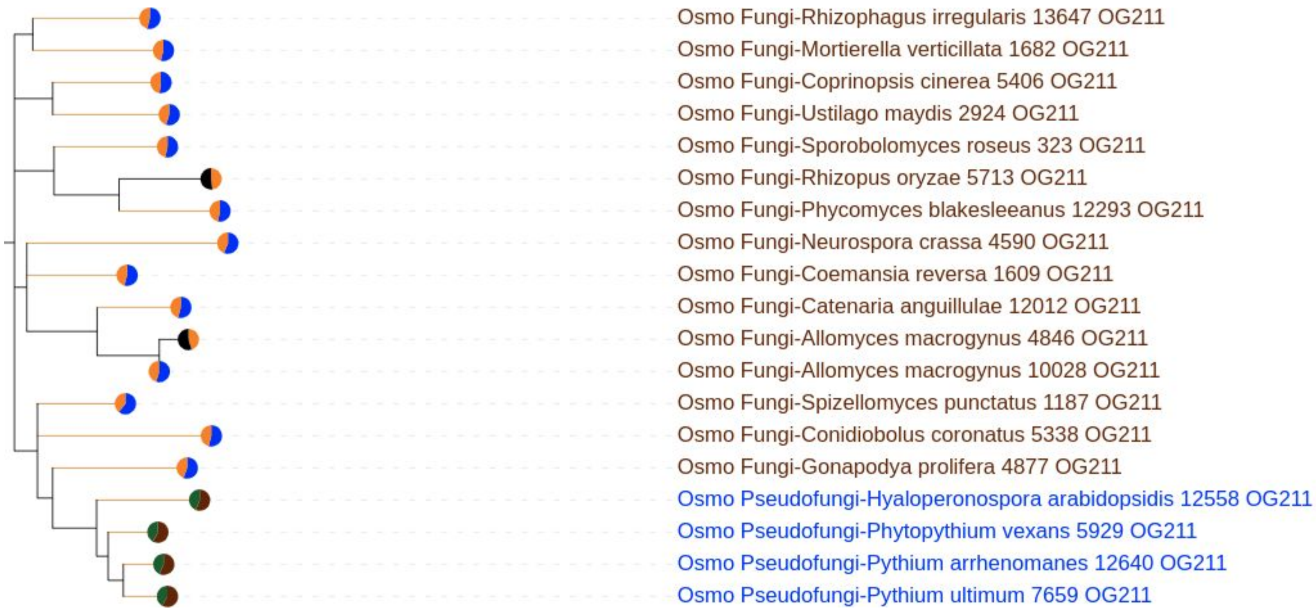

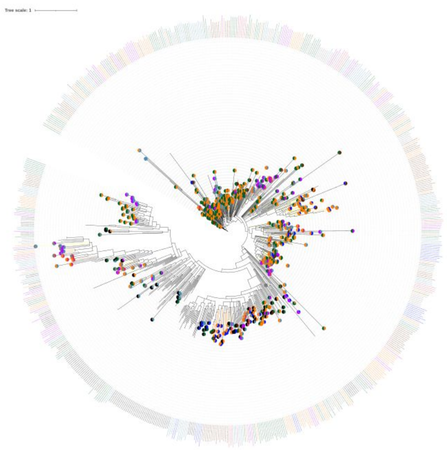

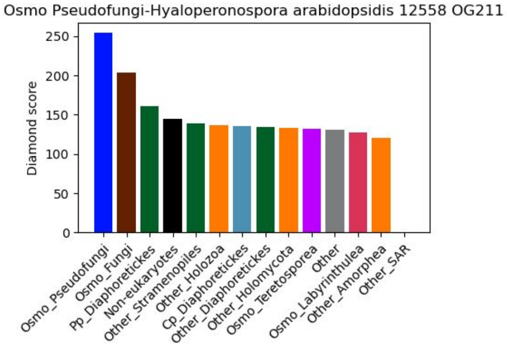

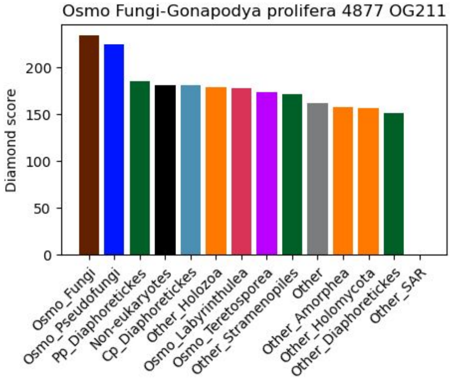

OG211 This gene family is widely distributed. Still, there is a clade involving sequence from Pseudofungi with Fungi. A HGT from *G. prolifera* lineage (Fungi) to Pseudofungi is quite likely, although we don't consider this a 'Highly likely' case due that the differences with in Diamond score with other taxonomic categories are not clearly differentiated sequences

- (0.44) K (0.61) Sir2 family (0.24)

Supplementary Information 4

Supporting files (phylogenetic tree and Diamond allignment score plots) for the 166 cases of HGT topologies identified by analysing the whole set of gene families present in osmotrophic groups. Referenced in the 'Horizontal gene transfers beyond osmoclusters' section from the main text

Two supporting files are displayed for each of the 166 cases, one after the other.

The first display corresponds to the phylogenetic tree of the corresponding cluster. In the tree, the sequence names from the clade which is considered to contain a HGT topology are colored according to the osmotrophic groups to which they belong to (red-Labyrinthulea, blue-Pseudofungi, purple-Teretsoporea, brown-Fungi).

These same colors are used for the second display item, which corresponds to dot plots showing the top Diamond alignment score of each sequence in the HGT topology clade against each of the taxonomic groupings displayed in the X axis. Dots corresponding to taxonomic groupings from Amorphea which do not belong to osmotrophic groups are colored in orange, and dots corresponding to taxonomic groupings from Diaphoretickes which do not belong to osmotrophic groups are colored in dark green. Dots corresponding to taxonomic groupings from outside Amorphea and Diaphoretickes are colored in grey, and dots corresponding to non-eukaryotes are colored in black. Each sequence in the HGT clade has a dot in each taxonomic grouping according to the given Diamond best alignment score against sequences belonging to species from the given taxonomic grouping (the score is zero if no alignment was reported for the given query sequence and a sequence from the given taxonomic grouping). The species taxonomic groupings used for these plots correspond to 'Group info 2' column in Supplementary Table 6. Since each HGT clade includes sequences from two distantly-related osmotrophic groups, the Diamond alignment score information is split in two subplots, each one showing the information for the sequences belonging to one of the two osmotrophic groups in the HGT clade.

See 'Semi-automated quantification of HGT topologies in all clusters' section in Material and methods for further details

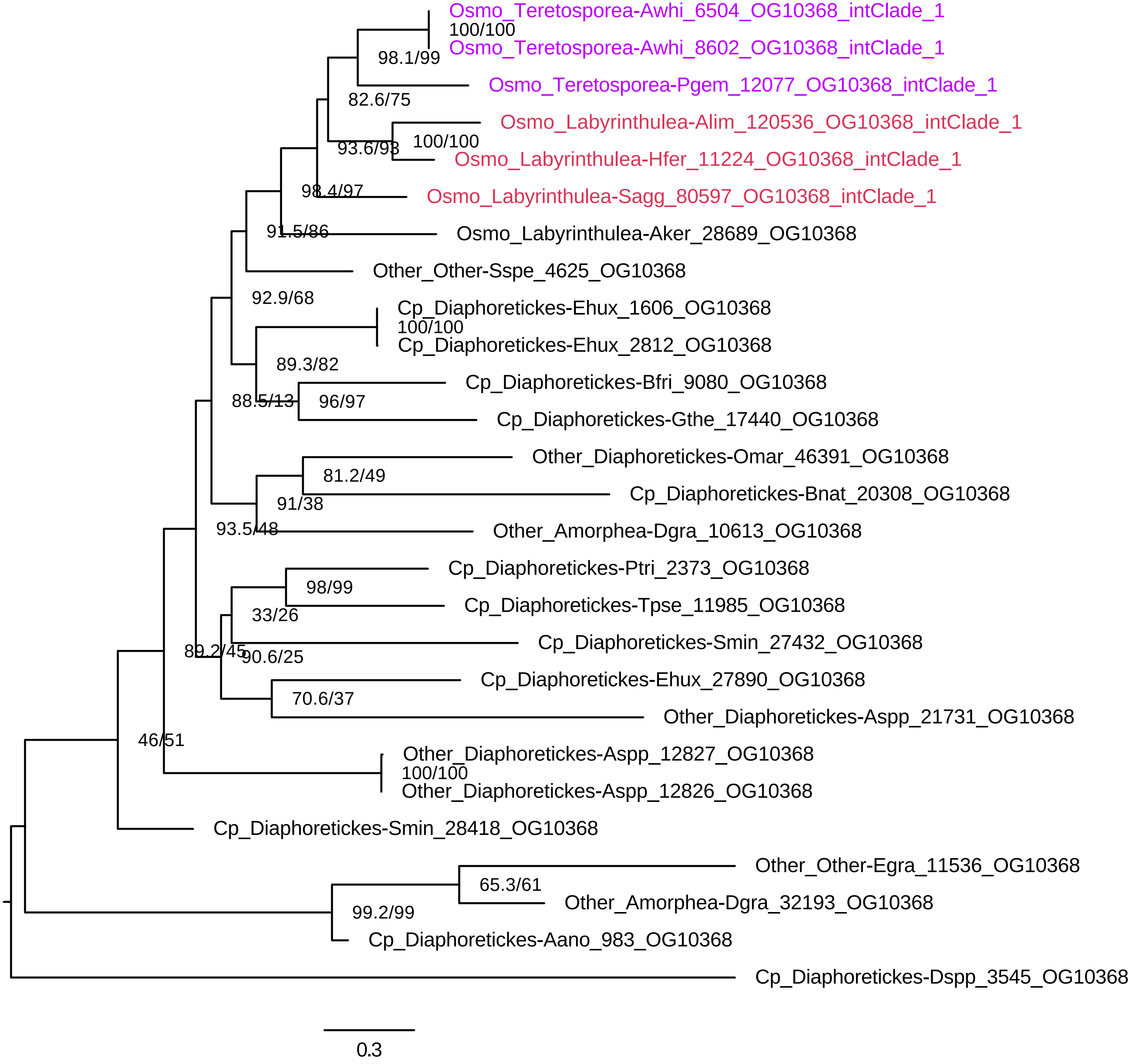

Osmo_Teretosporea-Awhi_6504_OG10368_intClade_1
100/100
Osmo_Teretosporea-Awhi_8602_OG10368_intClade_1
98.1/99
Osmo_Teretosporea-Pgem_12077_OG10368_intClade_1
82.6/75
Osmo_Labyrinthulea-Alim_120536_OG10368_intClade_1
100/100
93.6/98
Osmo_Labyrinthulea-Hfer_11224_OG10368_intClade_1
98.4/97
Osmo_Labyrinthulea-Sagg_80597_OG10368_intClade_1
91.5/86
Osmo_Labyrinthulea-Aker_28689_OG10368
Other_Other-Sspe_4625_OG10368
92.9/68
Cp_Diaphoretickes-Ehux_1606_OG10368
100/100
Cp_Diaphoretickes-Ehux_2812_OG10368
89.3/82
Cp_Diaphoretickes-Bfri_9080_OG10368
88.5/13
96/97
Cp_Diaphoretickes-Gthe_17440_OG10368
Other_Diaphoretickes-Omar_46391_OG10368
81.2/49
Cp_Diaphoretickes-Bnat_20308_OG10368
91/38
93.5/48
Other_Amorphea-Dgra_10613_OG10368
Cp_Diaphoretickes-Ptri_2373_OG10368
98/99
Cp_Diaphoretickes-Tpse_11985_OG10368
33/26
Cp_Diaphoretickes-Smin_27432_OG10368
89.2/45
90.6/25
Cp_Diaphoretickes-Ehux_27890_OG10368
70.6/37
Other_Diaphoretickes-Aspp_21731_OG10368
46/51
Other_Diaphoretickes-Aspp_12827_OG10368
100/100
Other_Diaphoretickes-Aspp_12826_OG10368
Cp_Diaphoretickes-Smin_28418_OG10368
Other_Other-Egra_11536_OG10368
65.3/61
Other_Amorphea-Dgra_32193_OG10368
99.2/99
Cp_Diaphoretickes-Aano_983_OG10368
Cp_Diaphoretickes-Dspp_3545_OG10368
0.3

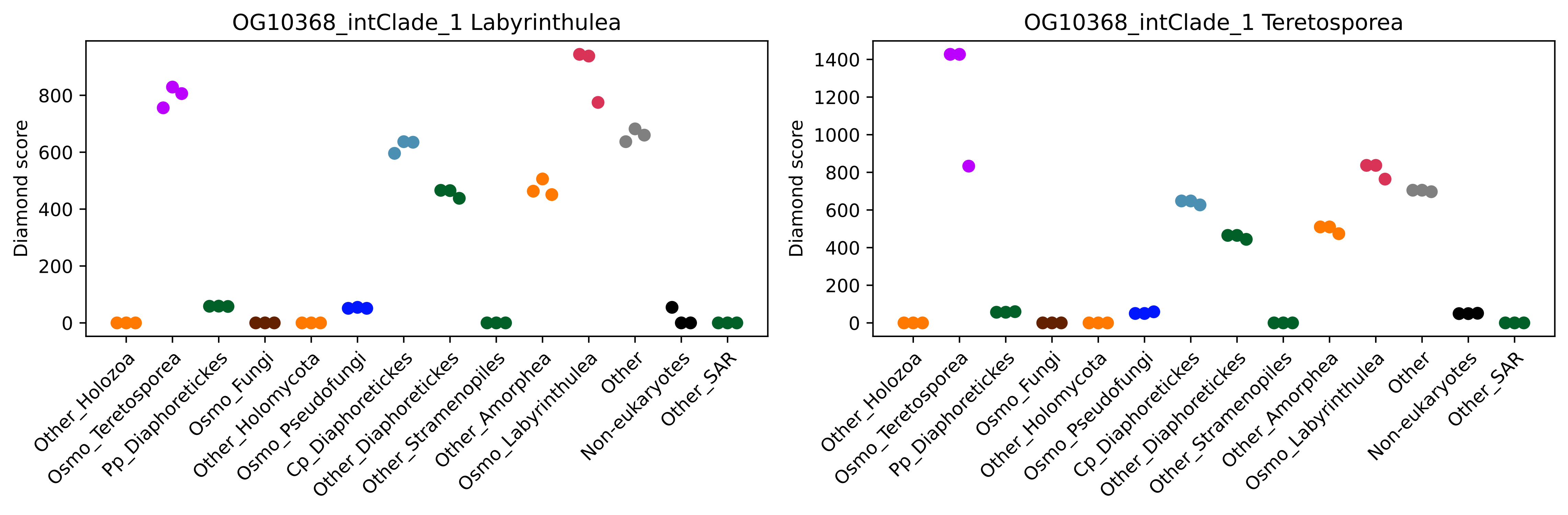
OG10368_intClade_1 Labyrinthulea
OG10368_intClade_1 Teretosporea
Diamond score
0
200
400
600
800
1000
1200
1400
Other_Holozoa
Osmo_Teretosporea
Pp_Diaphoretickes
Osmo_Fungi
Other_Holomycota
Osmo_Pseudofungi
Cp_Diaphoretickes
Other_Diaphoretickes
Other_Stramenopiles
Other_Amorphea
Osmo_Labyrinthulea
Other
Non-eukaryotes
Other_SAR

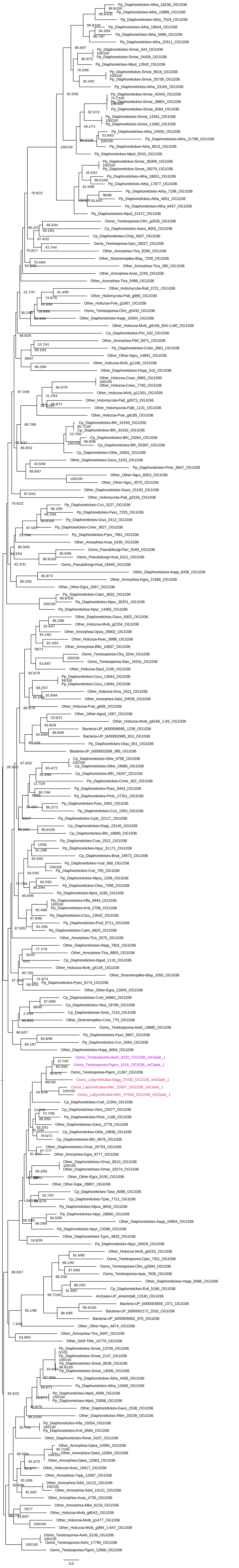
0.3

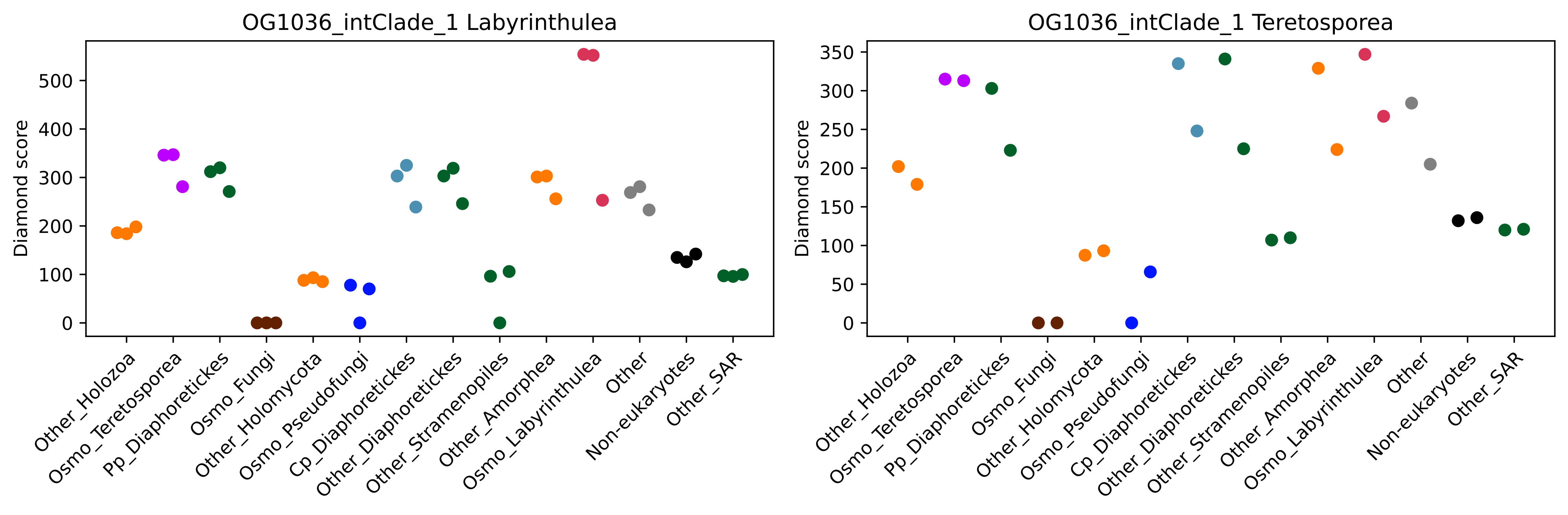
OG1036_intClade_1 Labyrinthulea
OG1036_intClade_1 Teretosporea
Diamond score
0
100
200
300
400
500
0
50
100
150
200
250
300
350
Other_Holozoa
Osmo_Teretosporea
Pp_Diaphoretickes
Osmo_Fungi
Other_Holomycota
Osmo_Pseudofungi
Cp_Diaphoretickes
Other_Diaphoretickes
Other_Stramenopiles
Other_Amorphea
Osmo_Labyrinthulea
Other
Non-eukaryotes
Other_SAR

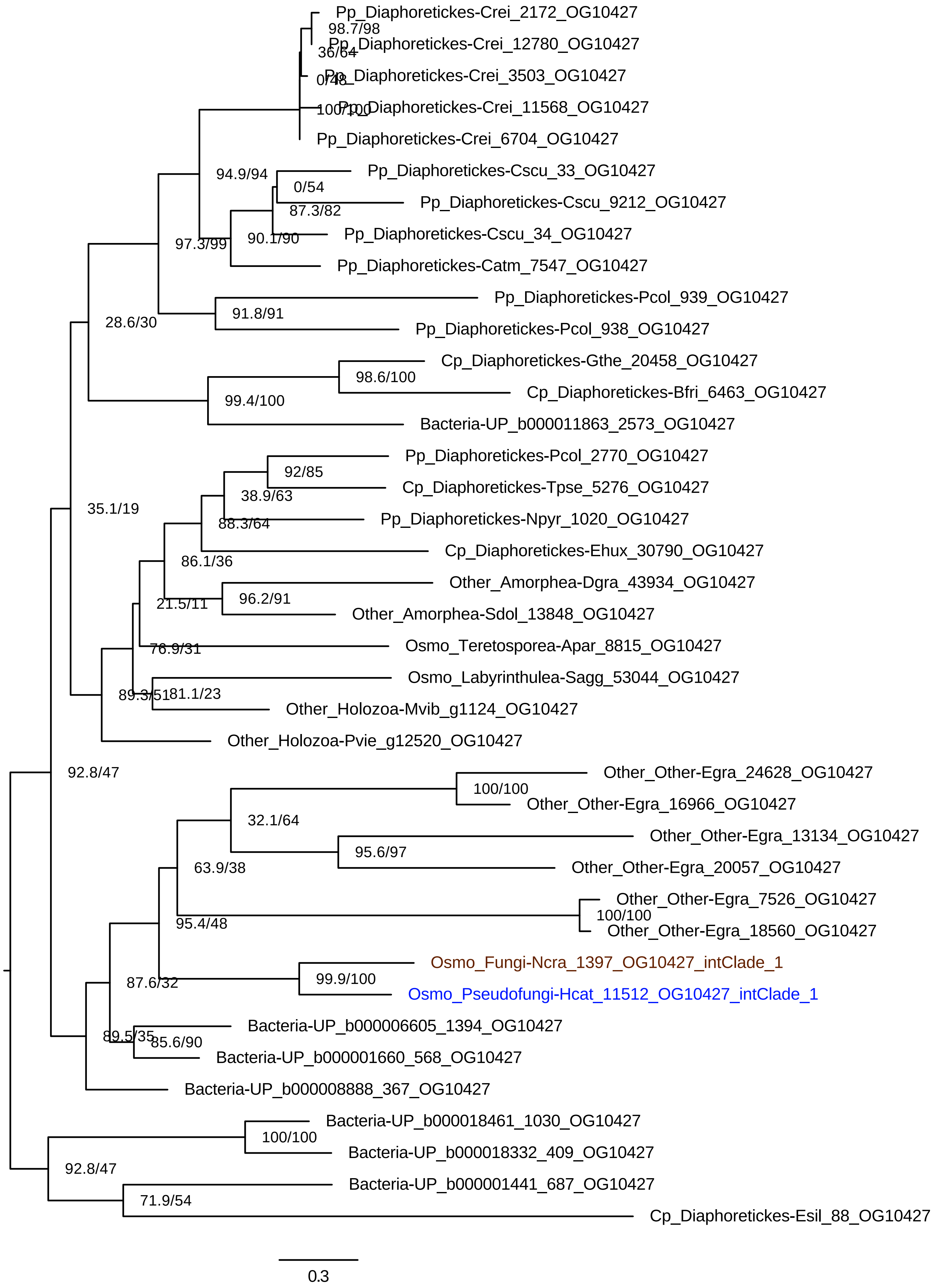
Pp_Diaphoretickes-Crei_2172_OG10427
98.7/98
Pp_Diaphoretickes-Crei_12780_OG10427
36/64
Pp_Diaphoretickes-Crei_3503_OG10427
0/48
Pp_Diaphoretickes-Crei_11568_OG10427
100/100
Pp_Diaphoretickes-Crei_6704_OG10427
Pp_Diaphoretickes-Cscu_33_OG10427
94.9/94
0/54
Pp_Diaphoretickes-Cscu_9212_OG10427
87.3/82
Pp_Diaphoretickes-Cscu_34_OG10427
97.3/99
90.1/90
Pp_Diaphoretickes-Catm_7547_OG10427
Pp_Diaphoretickes-Pcol_939_OG10427
91.8/91
28.6/30
Pp_Diaphoretickes-Pcol_938_OG10427
Cp_Diaphoretickes-Gthe_20458_OG10427
98.6/100
Cp_Diaphoretickes-Bfri_6463_OG10427
99.4/100
Bacteria-UP_b000011863_2573_OG10427
Pp_Diaphoretickes-Pcol_2770_OG10427
92/85
Cp_Diaphoretickes-Tpse_5276_OG10427
38.9/63
35.1/19
Pp_Diaphoretickes-Npyr_1020_OG10427
88.3/64
Cp_Diaphoretickes-Ehux_30790_OG10427
86.1/36
Other_Amorphea-Dgra_43934_OG10427
96.2/91
21.5/11
Other_Amorphea-Sdol_13848_OG10427
Osmo_Teretosporea-Apar_8815_OG10427
76.9/31
Osmo_Labyrinthulea-Sagg_53044_OG10427
89.3/51
81.1/23
Other_Holozoa-Mvib_g1124_OG10427
Other_Holozoa-Pvie_g12520_OG10427
92.8/47
Other_Other-Egra_24628_OG10427
100/100
Other_Other-Egra_16966_OG10427
32.1/64
Other_Other-Egra_13134_OG10427
95.6/97
63.9/38
Other_Other-Egra_20057_OG10427
Other_Other-Egra_7526_OG10427
100/100
95.4/48
Other_Other-Egra_18560_OG10427
Osmo_Fungi-Ncra_1397_OG10427_intClade_1
87.6/32
99.9/100
Osmo_Pseudofungi-Hcat_11512_OG10427_intClade_1
Bacteria-UP_b000006605_1394_OG10427
89.5/35
85.6/90
Bacteria-UP_b000001660_568_OG10427
Bacteria-UP_b000008888_367_OG10427
Bacteria-UP_b000018461_1030_OG10427
100/100
Bacteria-UP_b000018332_409_OG10427
92.8/47
Bacteria-UP_b000001441_687_OG10427
71.9/54
Cp_Diaphoretickes-Esil_88_OG10427
0.3

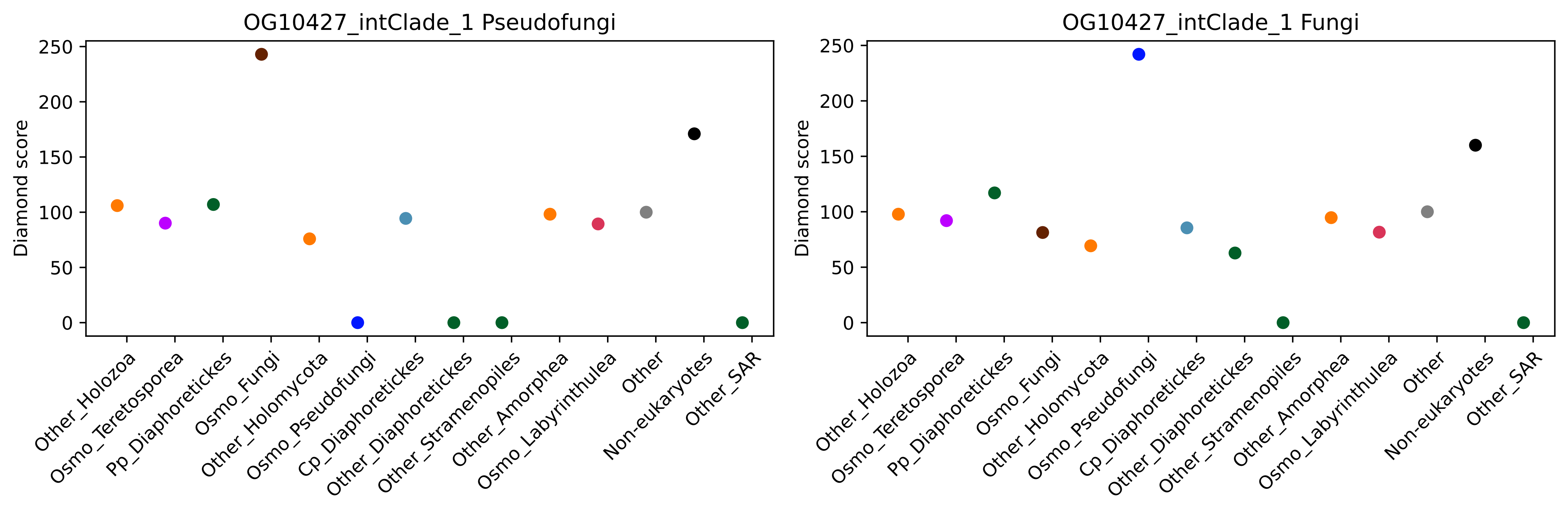
OG10427_intClade_1 Pseudofungi
OG10427_intClade_1 Fungi
Diamond score
0
50
100
150
200
250
Other_Holozoa
Osmo_Teretosporea
Pp_Diaphoretickes
Osmo_Fungi
Other_Holomycota
Osmo_Pseudofungi
Cp_Diaphoretickes
Other_Diaphoretickes
Other_Stramenopiles
Other_Amorphea
Osmo_Labyrinthulea
Other
Non-eukaryotes
Other_SAR

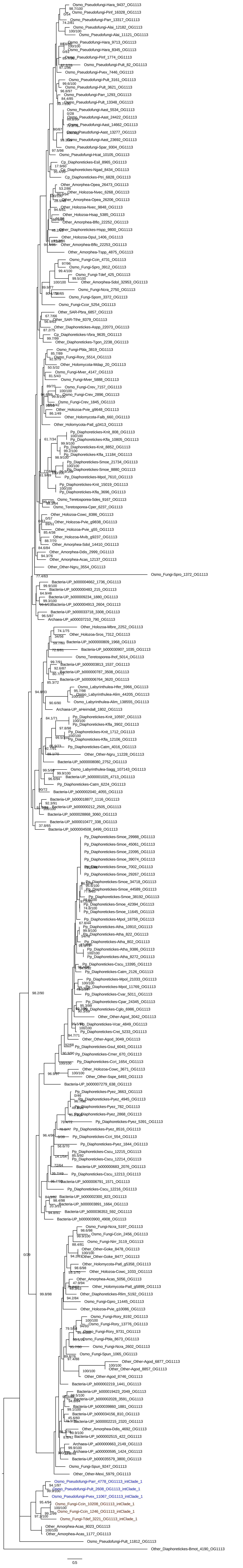

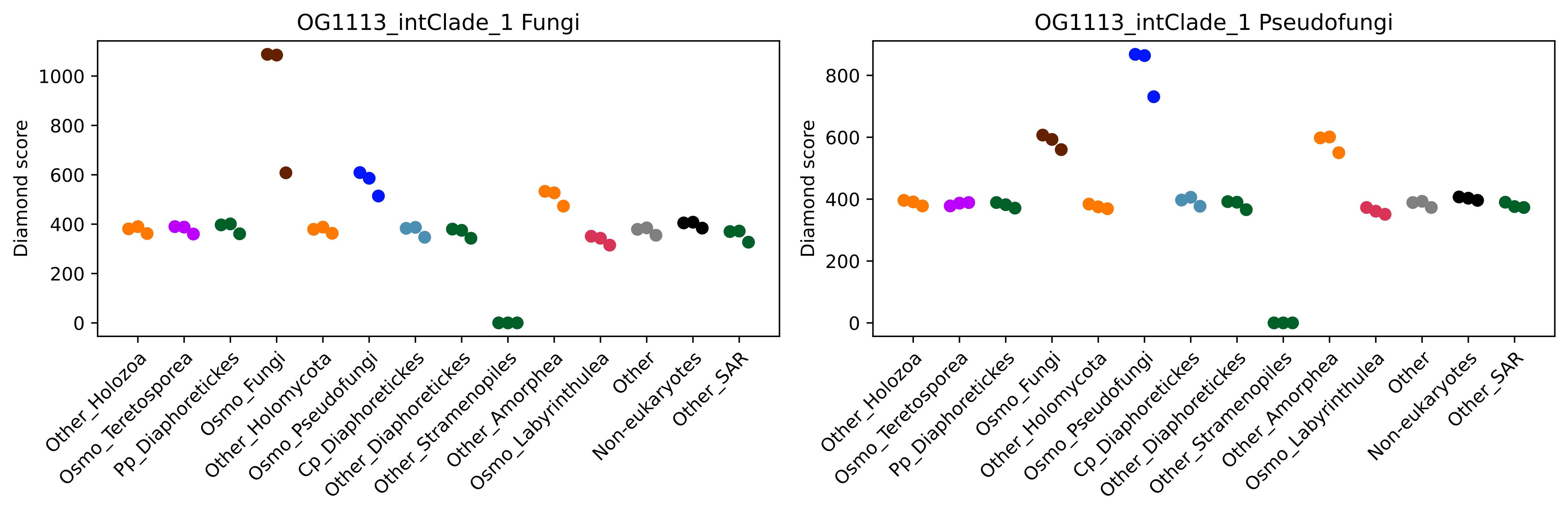

OG1113_intClade_1 Fungi
OG1113_intClade_1 Pseudofungi
Diamond score
0
200
400
600
800
1000
Other_Holozoa
Osmo_Teretosporea
Pp_Diaphoretickes
Osmo_Fungi
Other_Holomycota
Osmo_Pseudofungi
Cp_Diaphoretickes
Other_Diaphoretickes
Other_Stramenopiles
Other_Amorphea
Osmo_Labyrinthulea
Other
Non-eukaryotes
Other_SAR

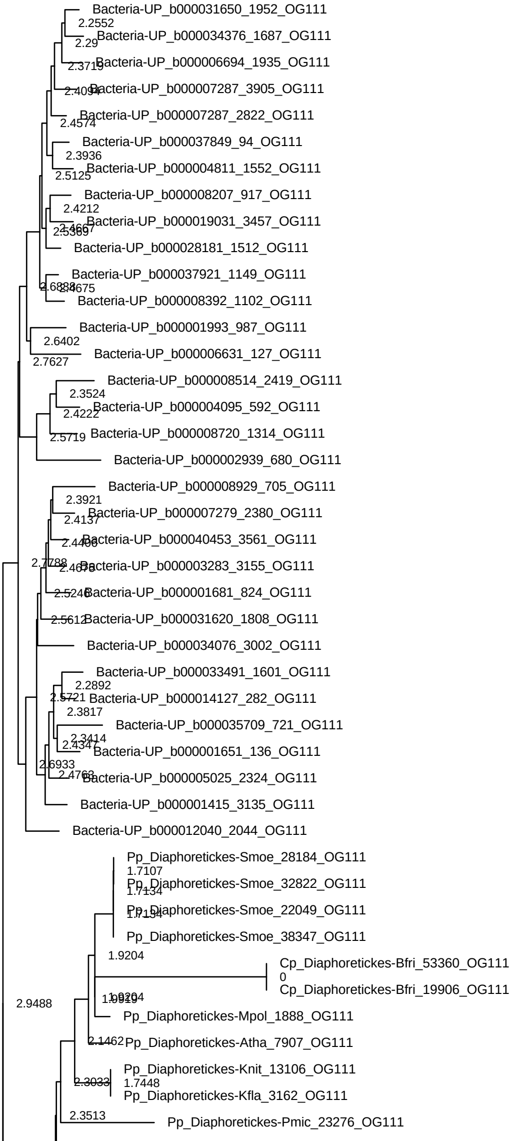

Bacteria-UP_b000031650_1952_OG111
2.2552
Bacteria-UP_b000034376_1687_OG111
2.29
Bacteria-UP_b000006694_1935_OG111
Bacteria-UP_b000007287_3905_OG111
Bacteria-UP_b000007287_2822_OG111
2.4574
Bacteria-UP_b000037849_94_OG111
2.3936
Bacteria-UP_b000004811_1552_OG111
2.5125
Bacteria-UP_b000008207_917_OG111
2.4212
Bacteria-UP_b000019031_3457_OG111
Bacteria-UP_b000028181_1512_OG111
Bacteria-UP_b000037921_1149_OG111
Bacteria-UP_b000008392_1102_OG111
Bacteria-UP_b000001993_987_OG111
2.6402
Bacteria-UP_b000006631_127_OG111
2.7627
Bacteria-UP_b000008514_2419_OG111
2.3524
Bacteria-UP_b000004095_592_OG111
2.4222
Bacteria-UP_b000008720_1314_OG111
2.5719
Bacteria-UP_b000002939_680_OG111
Bacteria-UP_b000008929_705_OG111
2.3921
Bacteria-UP_b000007279_2380_OG111
2.4137
Bacteria-UP_b000040453_3561_OG111
Bacteria-UP_b000003283_3155_OG111
Bacteria-UP_b000001681_824_OG111
Bacteria-UP_b000031620_1808_OG111
2.5612
Bacteria-UP_b000034076_3002_OG111
Bacteria-UP_b000033491_1601_OG111
2.2892
Bacteria-UP_b000014127_282_OG111
2.5721
2.3817
Bacteria-UP_b000035709_721_OG111
2.3414
2.4347
Bacteria-UP_b000001651_136_OG111
2.6933
Bacteria-UP_b000005025_2324_OG111
Bacteria-UP_b000001415_3135_OG111
Bacteria-UP_b000012040_2044_OG111
Pp_Diaphoretickes-Smoe_28184_OG111
1.7107
Pp_Diaphoretickes-Smoe_32822_OG111
Pp_Diaphoretickes-Smoe_22049_OG111
Pp_Diaphoretickes-Smoe_38347_OG111
1.9204
Cp_Diaphoretickes-Bfri_53360_OG111
0
Cp_Diaphoretickes-Bfri_19906_OG111
2.9488
Pp_Diaphoretickes-Mpol_1888_OG111
2.1462
Pp_Diaphoretickes-Atha_7907_OG111
Pp_Diaphoretickes-Knit_13106_OG111
2.3033
1.7448
Pp_Diaphoretickes-Kfla_3162_OG111
2.3513
Pp_Diaphoretickes-Pmic_23276_OG111

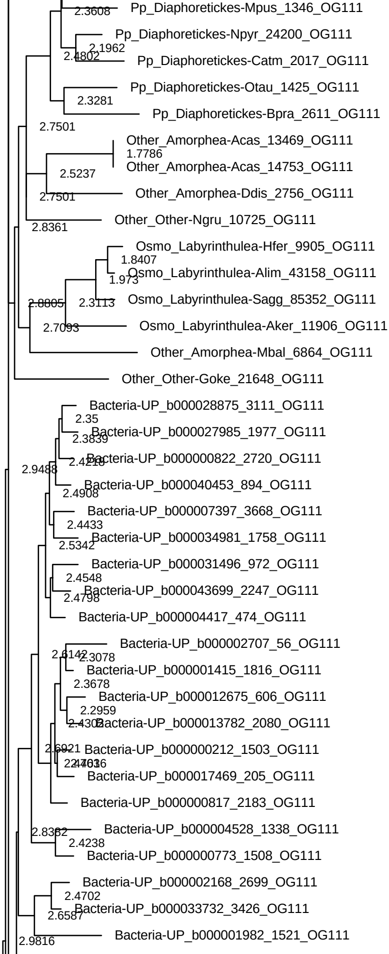

Pp_Diaphoretickes-Mpus_1346_OG111
2.3608
Pp_Diaphoretickes-Npyr_24200_OG111
2.1962
2.4802
Pp_Diaphoretickes-Catm_2017_OG111
Pp_Diaphoretickes-Otau_1425_OG111
2.3281
Pp_Diaphoretickes-Bpra_2611_OG111
2.7501
Other_Amorphea-Acas_13469_OG111
1.7786
Other_Amorphea-Acas_14753_OG111
2.5237
Other_Amorphea-Ddis_2756_OG111
2.7501
Other_Other-Ngru_10725_OG111
2.8361
Osmo_Labyrinthulea-Hfer_9905_OG111
1.8407
Osmo_Labyrinthulea-Alim_43158_OG111
1.973
2.8805
2.3113
Osmo_Labyrinthulea-Sagg_85352_OG111
2.7093
Osmo_Labyrinthulea-Aker_11906_OG111
Other_Amorphea-Mbal_6864_OG111
Other_Other-Goke_21648_OG111
Bacteria-UP_b000028875_3111_OG111
2.35
Bacteria-UP_b000027985_1977_OG111
2.3839
2.9488
2.4218
Bacteria-UP_b000000822_2720_OG111
Bacteria-UP_b000040453_894_OG111
2.4908
Bacteria-UP_b000007397_3668_OG111
2.4433
Bacteria-UP_b000034981_1758_OG111
2.5342
Bacteria-UP_b000031496_972_OG111
2.4548
Bacteria-UP_b000043699_2247_OG111
2.4798
Bacteria-UP_b000004417_474_OG111
Bacteria-UP_b000002707_56_OG111
2.6142
2.3078
Bacteria-UP_b000001415_1816_OG111
2.3678
Bacteria-UP_b000012675_606_OG111
2.2959
2.4302
Bacteria-UP_b000013782_2080_OG111
2.6921
Bacteria-UP_b000000212_1503_OG111
Bacteria-UP_b000017469_205_OG111
Bacteria-UP_b000000817_2183_OG111
2.8382
Bacteria-UP_b000004528_1338_OG111
2.4238
Bacteria-UP_b000000773_1508_OG111
Bacteria-UP_b000002168_2699_OG111
2.4702
Bacteria-UP_b000033732_3426_OG111
2.6587
Bacteria-UP_b000001982_1521_OG111
2.9816

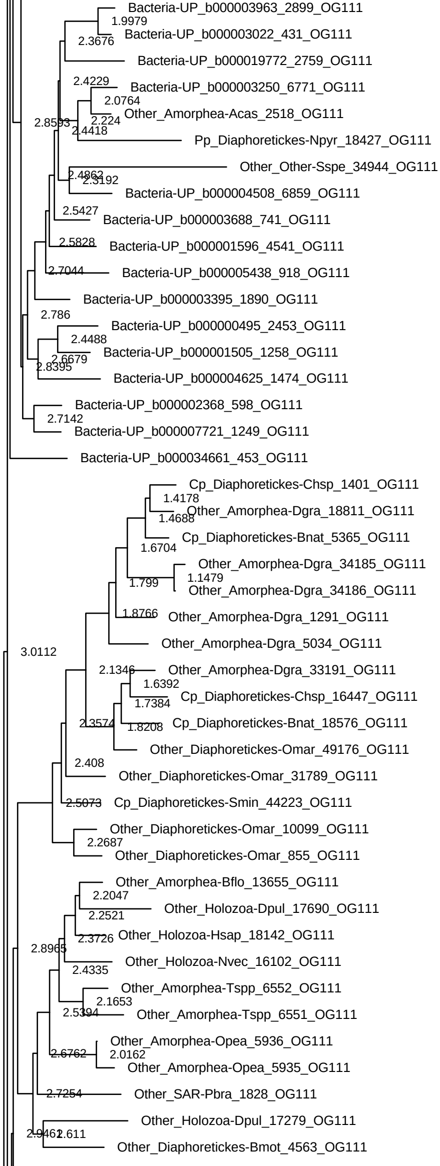

Bacteria-UP_b000003963_2899_OG111
1.9979
Bacteria-UP_b000003022_431_OG111
2.3676
Bacteria-UP_b000019772_2759_OG111
2.4229
Bacteria-UP_b000003250_6771_OG111
2.0764
Other_Amorphea-Acas_2518_OG111
2.8593
2.224
2.4418
Pp_Diaphoretickes-Npyr_18427_OG111
Other_Other-Sspe_34944_OG111
2.4862
2.3192
Bacteria-UP_b000004508_6859_OG111
2.5427
Bacteria-UP_b000003688_741_OG111
2.5828
Bacteria-UP_b000001596_4541_OG111
2.7044
Bacteria-UP_b000005438_918_OG111
Bacteria-UP_b000003395_1890_OG111
2.786
Bacteria-UP_b000000495_2453_OG111
2.4488
Bacteria-UP_b000001505_1258_OG111
2.6679
2.8395
Bacteria-UP_b000004625_1474_OG111
Bacteria-UP_b000002368_598_OG111
2.7142
Bacteria-UP_b000007721_1249_OG111
Bacteria-UP_b000034661_453_OG111
Cp_Diaphoretickes-Chsp_1401_OG111
1.4178
Other_Amorphea-Dgra_18811_OG111
1.4688
Cp_Diaphoretickes-Bnat_5365_OG111
1.6704
Other_Amorphea-Dgra_34185_OG111
1.1479
1.799
Other_Amorphea-Dgra_34186_OG111
1.8766
Other_Amorphea-Dgra_1291_OG111
Other_Amorphea-Dgra_5034_OG111
3.0112
2.1346
Other_Amorphea-Dgra_33191_OG111
1.6392
Cp_Diaphoretickes-Chsp_16447_OG111
1.7384
2.3574
1.8208
Cp_Diaphoretickes-Bnat_18576_OG111
Other_Diaphoretickes-Omar_49176_OG111
2.408
Other_Diaphoretickes-Omar_31789_OG111
2.5073
Cp_Diaphoretickes-Smin_44223_OG111
Other_Diaphoretickes-Omar_10099_OG111
2.2687
Other_Diaphoretickes-Omar_855_OG111
Other_Amorphea-Bflo_13655_OG111
2.2047
Other_Holozoa-Dpul_17690_OG111
2.2521
Other_Holozoa-Hsap_18142_OG111
2.3726
2.8965
Other_Holozoa-Nvec_16102_OG111
2.4335
Other_Amorphea-Tspp_6552_OG111
2.1653
2.5394
Other_Amorphea-Tspp_6551_OG111
Other_Amorphea-Opea_5936_OG111
2.6762
2.0162
Other_Amorphea-Opea_5935_OG111
2.7254
Other_SAR-Pbra_1828_OG111
Other_Holozoa-Dpul_17279_OG111
2.9461
2.611
Other_Diaphoretickes-Bmot_4563_OG111

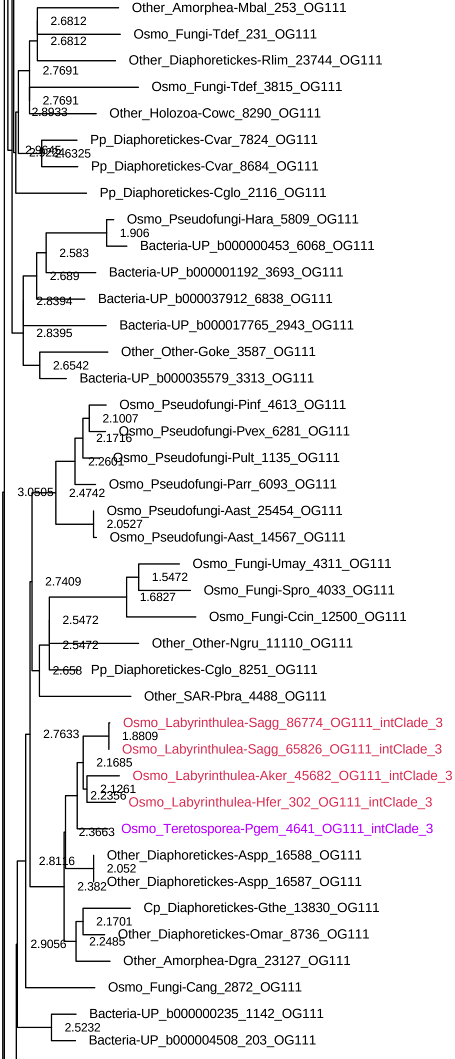
Other_Amorphea-Mbal_253_OG111
Osmo_Fungi-Tdef_231_OG111
Other_Diaphoretickes-Rlim_23744_OG111
Osmo_Fungi-Tdef_3815_OG111
Other_Holozoa-Cowc_8290_OG111
Pp_Diaphoretickes-Cvar_7824_OG111
Pp_Diaphoretickes-Cvar_8684_OG111
Pp_Diaphoretickes-Cglo_2116_OG111
Osmo_Pseudofungi-Hara_5809_OG111
Bacteria-UP_b000000453_6068_OG111
Bacteria-UP_b000001192_3693_OG111
Bacteria-UP_b000037912_6838_OG111
Bacteria-UP_b000017765_2943_OG111
Other_Other-Goke_3587_OG111
Bacteria-UP_b000035579_3313_OG111
Osmo_Pseudofungi-Pinf_4613_OG111
Osmo_Pseudofungi-Pvex_6281_OG111
Osmo_Pseudofungi-Pult_1135_OG111
Osmo_Pseudofungi-Parr_6093_OG111
Osmo_Pseudofungi-Aast_25454_OG111
Osmo_Pseudofungi-Aast_14567_OG111
Osmo_Fungi-Umay_4311_OG111
Osmo_Fungi-Spro_4033_OG111
Osmo_Fungi-Ccin_12500_OG111
Other_Other-Ngru_11110_OG111
Pp_Diaphoretickes-Cglo_8251_OG111
Other_SAR-Pbra_4488_OG111
Osmo_Labyrinthulea-Sagg_86774_OG111_intClade_3
Osmo_Labyrinthulea-Sagg_65826_OG111_intClade_3
Osmo_Labyrinthulea-Aker_45682_OG111_intClade_3
Osmo_Labyrinthulea-Hfer_302_OG111_intClade_3
Osmo_Teretosporea-Pgem_4641_OG111_intClade_3
Other_Diaphoretickes-Aspp_16588_OG111
Other_Diaphoretickes-Aspp_16587_OG111
Cp_Diaphoretickes-Gthe_13830_OG111
Other_Diaphoretickes-Omar_8736_OG111
Other_Amorphea-Dgra_23127_OG111
Osmo_Fungi-Cang_2872_OG111
Bacteria-UP_b000000235_1142_OG111
Bacteria-UP_b000004508_203_OG111

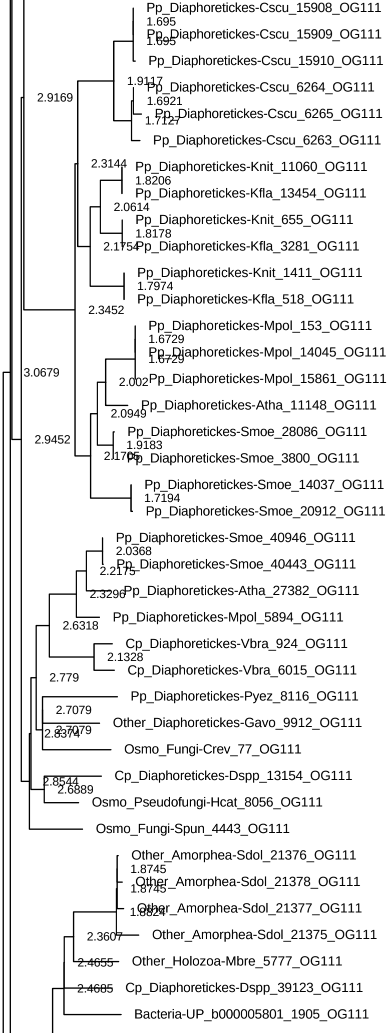
Pp_Diaphoretickes-Cscu_15908_OG111
1.695
Pp_Diaphoretickes-Cscu_15909_OG111
1.695
Pp_Diaphoretickes-Cscu_15910_OG111
1.9117
Pp_Diaphoretickes-Cscu_6264_OG111
2.9169
1.6921
Pp_Diaphoretickes-Cscu_6265_OG111
1.7127
Pp_Diaphoretickes-Cscu_6263_OG111
2.3144
Pp_Diaphoretickes-Knit_11060_OG111
1.8206
Pp_Diaphoretickes-Kfla_13454_OG111
2.0614
Pp_Diaphoretickes-Knit_655_OG111
1.8178
2.1754
Pp_Diaphoretickes-Kfla_3281_OG111
Pp_Diaphoretickes-Knit_1411_OG111
1.7974
Pp_Diaphoretickes-Kfla_518_OG111
2.3452
Pp_Diaphoretickes-Mpol_153_OG111
1.6729
Pp_Diaphoretickes-Mpol_14045_OG111
1.6729
3.0679
2.002
Pp_Diaphoretickes-Mpol_15861_OG111
Pp_Diaphoretickes-Atha_11148_OG111
2.0949
Pp_Diaphoretickes-Smoe_28086_OG111
2.9452
1.9183
2.1705
Pp_Diaphoretickes-Smoe_3800_OG111
Pp_Diaphoretickes-Smoe_14037_OG111
1.7194
Pp_Diaphoretickes-Smoe_20912_OG111
Pp_Diaphoretickes-Smoe_40946_OG111
2.0368
Pp_Diaphoretickes-Smoe_40443_OG111
2.2175
2.3296
Pp_Diaphoretickes-Atha_27382_OG111
Pp_Diaphoretickes-Mpol_5894_OG111
2.6318
Cp_Diaphoretickes-Vbra_924_OG111
2.1328
Cp_Diaphoretickes-Vbra_6015_OG111
2.779
Pp_Diaphoretickes-Pyez_8116_OG111
2.7079
Other_Diaphoretickes-Gavo_9912_OG111
2.7079
2.8374
Osmo_Fungi-Crev_77_OG111
Cp_Diaphoretickes-Dspp_13154_OG111
2.8544
2.6889
Osmo_Pseudofungi-Hcat_8056_OG111
Osmo_Fungi-Spun_4443_OG111
Other_Amorphea-Sdol_21376_OG111
1.8745
Other_Amorphea-Sdol_21378_OG111
1.8745
Other_Amorphea-Sdol_21377_OG111
1.8824
2.3607
Other_Amorphea-Sdol_21375_OG111
2.4655
Other_Holozoa-Mbre_5777_OG111
2.4685
Cp_Diaphoretickes-Dspp_39123_OG111
Bacteria-UP_b000005801_1905_OG111

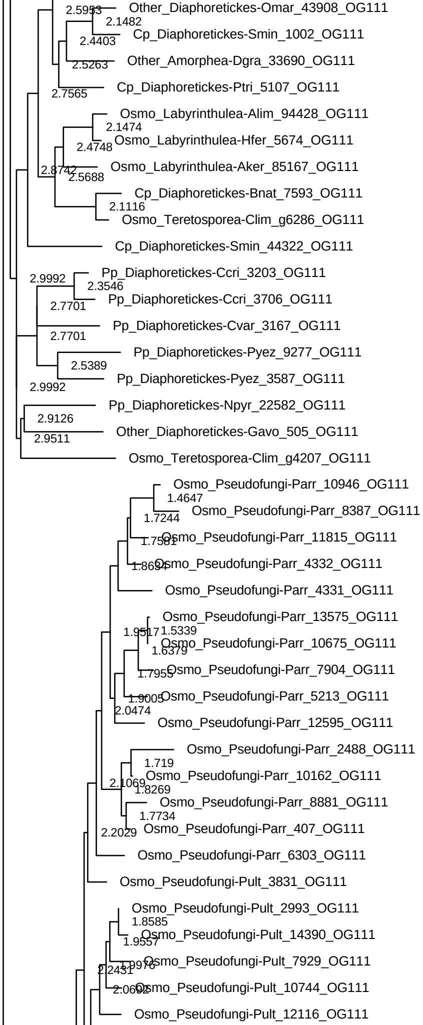
Other_Diaphoretickes-Omar_43908_OG111
2.5953
2.1482
Cp_Diaphoretickes-Smin_1002_OG111
2.4403
Other_Amorphea-Dgra_33690_OG111
2.5263
Cp_Diaphoretickes-Ptri_5107_OG111
2.7565
Osmo_Labyrinthulea-Alim_94428_OG111
2.1474
Osmo_Labyrinthulea-Hfer_5674_OG111
2.4748
Osmo_Labyrinthulea-Aker_85167_OG111
2.8742
2.5688
Cp_Diaphoretickes-Bnat_7593_OG111
2.1116
Osmo_Teretosporea-Clim_g6286_OG111
Cp_Diaphoretickes-Smin_44322_OG111
Pp_Diaphoretickes-Ccri_3203_OG111
2.9992
2.3546
Pp_Diaphoretickes-Ccri_3706_OG111
2.7701
Pp_Diaphoretickes-Cvar_3167_OG111
2.7701
Pp_Diaphoretickes-Pyez_9277_OG111
2.5389
Pp_Diaphoretickes-Pyez_3587_OG111
2.9992
Pp_Diaphoretickes-Npyr_22582_OG111
2.9126
Other_Diaphoretickes-Gavo_505_OG111
2.9511
Osmo_Teretosporea-Clim_g4207_OG111
Osmo_Pseudofungi-Parr_10946_OG111
1.4647
Osmo_Pseudofungi-Parr_8387_OG111
1.7244
Osmo_Pseudofungi-Parr_11815_OG111
1.7581
Osmo_Pseudofungi-Parr_4332_OG111
1.8634
Osmo_Pseudofungi-Parr_4331_OG111
Osmo_Pseudofungi-Parr_13575_OG111
1.9517
1.5339
Osmo_Pseudofungi-Parr_10675_OG111
1.6379
Osmo_Pseudofungi-Parr_7904_OG111
1.7955
Osmo_Pseudofungi-Parr_5213_OG111
1.9005
2.0474
Osmo_Pseudofungi-Parr_12595_OG111
Osmo_Pseudofungi-Parr_2488_OG111
1.719
Osmo_Pseudofungi-Parr_10162_OG111
2.1069
1.8269
Osmo_Pseudofungi-Parr_8881_OG111
1.7734
Osmo_Pseudofungi-Parr_407_OG111
2.2029
Osmo_Pseudofungi-Parr_6303_OG111
Osmo_Pseudofungi-Pult_3831_OG111
Osmo_Pseudofungi-Pult_2993_OG111
1.8585
Osmo_Pseudofungi-Pult_14390_OG111
1.9557
Osmo_Pseudofungi-Pult_7929_OG111
1.9976
2.2431
Osmo_Pseudofungi-Pult_10744_OG111
2.0692
Osmo_Pseudofungi-Pult_12116_OG111

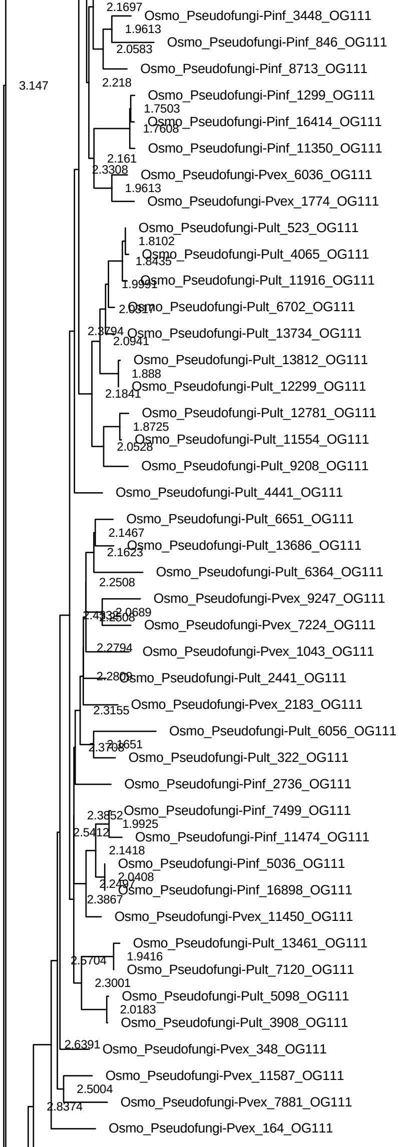
2.1697
Osmo_Pseudofungi-Pinf_3448_OG111
1.9613
Osmo_Pseudofungi-Pinf_846_OG111
2.0583
Osmo_Pseudofungi-Pinf_8713_OG111
3.147
2.218
Osmo_Pseudofungi-Pinf_1299_OG111
1.7503
Osmo_Pseudofungi-Pinf_16414_OG111
1.7608
Osmo_Pseudofungi-Pinf_11350_OG111
2.161
2.3308
Osmo_Pseudofungi-Pvex_6036_OG111
1.9613
Osmo_Pseudofungi-Pvex_1774_OG111
Osmo_Pseudofungi-Pult_523_OG111
1.8102
Osmo_Pseudofungi-Pult_4065_OG111
1.8435
Osmo_Pseudofungi-Pult_11916_OG111
1.9991
Osmo_Pseudofungi-Pult_6702_OG111
2.0317
2.3794
Osmo_Pseudofungi-Pult_13734_OG111
2.0941
Osmo_Pseudofungi-Pult_13812_OG111
1.888
Osmo_Pseudofungi-Pult_12299_OG111
2.1841
Osmo_Pseudofungi-Pult_12781_OG111
1.8725
Osmo_Pseudofungi-Pult_11554_OG111
2.0528
Osmo_Pseudofungi-Pult_9208_OG111
Osmo_Pseudofungi-Pult_4441_OG111
Osmo_Pseudofungi-Pult_6651_OG111
2.1467
Osmo_Pseudofungi-Pult_13686_OG111
2.1623
Osmo_Pseudofungi-Pult_6364_OG111
2.2508
Osmo_Pseudofungi-Pvex_9247_OG111
2.0689
2.2508
Osmo_Pseudofungi-Pvex_7224_OG111
2.2794
Osmo_Pseudofungi-Pvex_1043_OG111
2.2809
Osmo_Pseudofungi-Pult_2441_OG111
2.3155
Osmo_Pseudofungi-Pvex_2183_OG111
Osmo_Pseudofungi-Pult_6056_OG111
2.1651
2.3708
Osmo_Pseudofungi-Pult_322_OG111
Osmo_Pseudofungi-Pinf_2736_OG111
2.3852
Osmo_Pseudofungi-Pinf_7499_OG111
1.9925
2.5412
Osmo_Pseudofungi-Pinf_11474_OG111
2.1418
Osmo_Pseudofungi-Pinf_5036_OG111
2.0408
2.2497
Osmo_Pseudofungi-Pinf_16898_OG111
2.3867
Osmo_Pseudofungi-Pvex_11450_OG111
Osmo_Pseudofungi-Pult_13461_OG111
2.5704
1.9416
Osmo_Pseudofungi-Pult_7120_OG111
2.3001
Osmo_Pseudofungi-Pult_5098_OG111
2.0183
Osmo_Pseudofungi-Pult_3908_OG111
2.6391
Osmo_Pseudofungi-Pvex_348_OG111
Osmo_Pseudofungi-Pvex_11587_OG111
2.5004
Osmo_Pseudofungi-Pvex_7881_OG111
2.8374
Osmo_Pseudofungi-Pvex_164_OG111

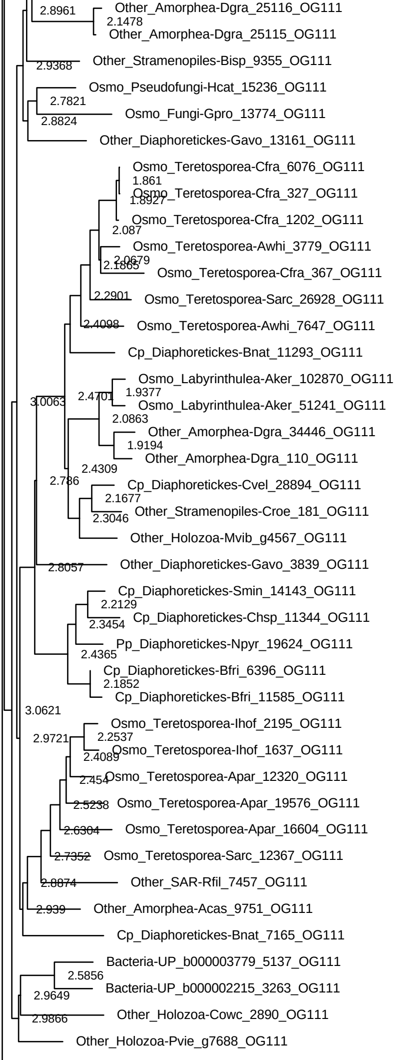

2.8961
Other_Amorphea-Dgra_25116_OG111
2.1478
Other_Amorphea-Dgra_25115_OG111
2.9368
Other_Stramenopiles-Bisp_9355_OG111
Osmo_Pseudofungi-Hcat_15236_OG111
2.7821
Osmo_Fungi-Gpro_13774_OG111
2.8824
Other_Diaphoretickes-Gavo_13161_OG111
Osmo_Teretosporea-Cfra_6076_OG111
1.861
Osmo_Teretosporea-Cfra_327_OG111
1.8927
Osmo_Teretosporea-Cfra_1202_OG111
2.087
Osmo_Teretosporea-Awhi_3779_OG111
2.0679
2.1865
Osmo_Teretosporea-Cfra_367_OG111
2.2901
Osmo_Teretosporea-Sarc_26928_OG111
2.4098
Osmo_Teretosporea-Awhi_7647_OG111
Cp_Diaphoretickes-Bnat_11293_OG111
Osmo_Labyrinthulea-Aker_102870_OG111
1.9377
3.0063
2.4701
Osmo_Labyrinthulea-Aker_51241_OG111
2.0863
Other_Amorphea-Dgra_34446_OG111
1.9194
Other_Amorphea-Dgra_110_OG111
2.4309
2.786
Cp_Diaphoretickes-Cvel_28894_OG111
2.1677
Other_Stramenopiles-Croe_181_OG111
2.3046
Other_Holozoa-Mvib_g4567_OG111
2.8057
Other_Diaphoretickes-Gavo_3839_OG111
Cp_Diaphoretickes-Smin_14143_OG111
2.2129
Cp_Diaphoretickes-Chsp_11344_OG111
2.3454
Pp_Diaphoretickes-Npyr_19624_OG111
2.4365
Cp_Diaphoretickes-Bfri_6396_OG111
2.1852
Cp_Diaphoretickes-Bfri_11585_OG111
3.0621
Osmo_Teretosporea-Ihof_2195_OG111
2.9721
2.2537
Osmo_Teretosporea-Ihof_1637_OG111
2.4089
2.454
Osmo_Teretosporea-Apar_12320_OG111
2.5238
Osmo_Teretosporea-Apar_19576_OG111
2.6304
Osmo_Teretosporea-Apar_16604_OG111
2.7352
Osmo_Teretosporea-Sarc_12367_OG111
2.8874
Other_SAR-Rfil_7457_OG111
2.939
Other_Amorphea-Acas_9751_OG111
Cp_Diaphoretickes-Bnat_7165_OG111
Bacteria-UP_b000003779_5137_OG111
2.5856
Bacteria-UP_b000002215_3263_OG111
2.9649
2.9866
Other_Holozoa-Cowc_2890_OG111
Other_Holozoa-Pvie_g7688_OG111

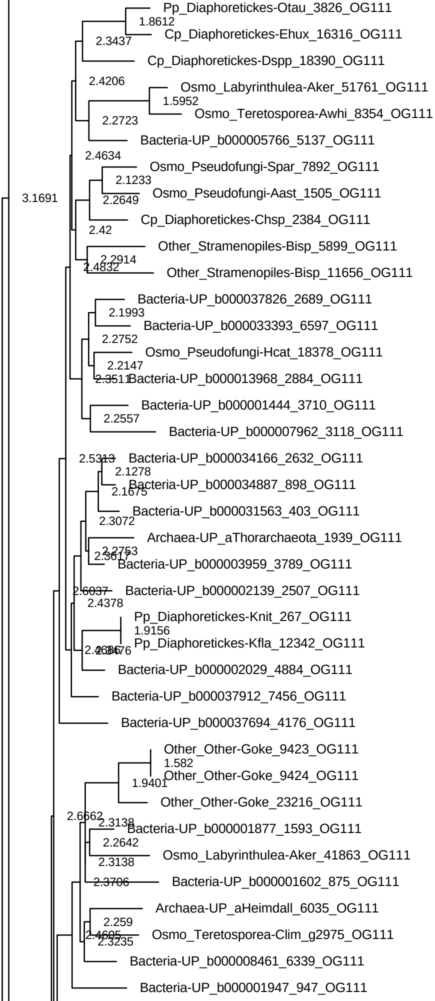

Pp_Diaphoretickes-Otau_3826_OG111
1.8612
Cp_Diaphoretickes-Ehux_16316_OG111
2.3437
Cp_Diaphoretickes-Dspp_18390_OG111
2.4206
Osmo_Labyrinthulea-Aker_51761_OG111
1.5952
Osmo_Teretosporea-Awhi_8354_OG111
2.2723
Bacteria-UP_b000005766_5137_OG111
2.4634
Osmo_Pseudofungi-Spar_7892_OG111
2.1233
3.1691
Osmo_Pseudofungi-Aast_1505_OG111
2.2649
Cp_Diaphoretickes-Chsp_2384_OG111
2.42
Other_Stramenopiles-Bisp_5899_OG111
2.2914
2.4832
Other_Stramenopiles-Bisp_11656_OG111
Bacteria-UP_b000037826_2689_OG111
2.1993
Bacteria-UP_b000033393_6597_OG111
2.2752
Osmo_Pseudofungi-Hcat_18378_OG111
2.2147
2.3511
Bacteria-UP_b000013968_2884_OG111
Bacteria-UP_b000001444_3710_OG111
2.2557
Bacteria-UP_b000007962_3118_OG111
2.5313
Bacteria-UP_b000034166_2632_OG111
2.1278
Bacteria-UP_b000034887_898_OG111
2.1675
Bacteria-UP_b000031563_403_OG111
2.3072
Archaea-UP_aThorarchaeota_1939_OG111
2.2753
2.3617
Bacteria-UP_b000003959_3789_OG111
2.6037
Bacteria-UP_b000002139_2507_OG111
2.4378
Pp_Diaphoretickes-Knit_267_OG111
1.9156
Pp_Diaphoretickes-Kfla_12342_OG111
Bacteria-UP_b000002029_4884_OG111
Bacteria-UP_b000037912_7456_OG111
Bacteria-UP_b000037694_4176_OG111
Other_Other-Goke_9423_OG111
1.582
Other_Other-Goke_9424_OG111
1.9401
Other_Other-Goke_23216_OG111
2.6662
2.3138
Bacteria-UP_b000001877_1593_OG111
2.2642
Osmo_Labyrinthulea-Aker_41863_OG111
2.3138
2.3706
Bacteria-UP_b000001602_875_OG111
Archaea-UP_aHeimdall_6035_OG111
2.259
2.4605
Osmo_Teretosporea-Clim_g2975_OG111
2.3235
Bacteria-UP_b000008461_6339_OG111
Bacteria-UP_b000001947_947_OG111

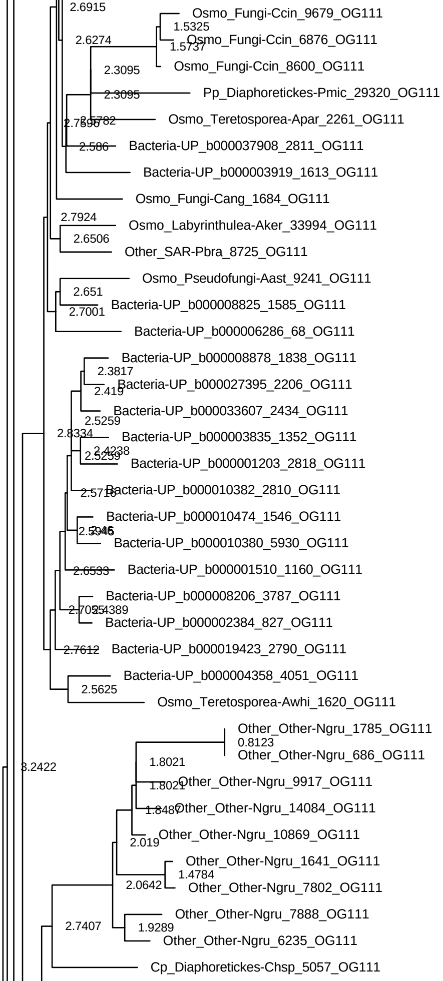

2.6915
Osmo_Fungi-Ccin_9679_OG111
1.5325
Osmo_Fungi-Ccin_6876_OG111
1.5737
2.6274
Osmo_Fungi-Ccin_8600_OG111
2.3095
Pp_Diaphoretickes-Pmic_29320_OG111
2.3095
Osmo_Teretosporea-Apar_2261_OG111
2.5782
Bacteria-UP_b000037908_2811_OG111
2.586
Bacteria-UP_b000003919_1613_OG111
Osmo_Fungi-Cang_1684_OG111
2.7924
Osmo_Labyrinthulea-Aker_33994_OG111
2.6506
Other_SAR-Pbra_8725_OG111
Osmo_Pseudofungi-Aast_9241_OG111
2.651
Bacteria-UP_b000008825_1585_OG111
2.7001
Bacteria-UP_b000006286_68_OG111
Bacteria-UP_b000008878_1838_OG111
2.3817
Bacteria-UP_b000027395_2206_OG111
2.419
Bacteria-UP_b000033607_2434_OG111
2.5259
2.8334
Bacteria-UP_b000003835_1352_OG111
2.4238
2.5259
Bacteria-UP_b000001203_2818_OG111
Bacteria-UP_b000010382_2810_OG111
Bacteria-UP_b000010474_1546_OG111
Bacteria-UP_b000010380_5930_OG111
2.6533
Bacteria-UP_b000001510_1160_OG111
Bacteria-UP_b000008206_3787_OG111
2.4389
Bacteria-UP_b000002384_827_OG111
2.7612
Bacteria-UP_b000019423_2790_OG111
Bacteria-UP_b000004358_4051_OG111
2.5625
Osmo_Teretosporea-Awhi_1620_OG111
Other_Other-Ngru_1785_OG111
0.8123
Other_Other-Ngru_686_OG111
3.2422
1.8021
Other_Other-Ngru_9917_OG111
1.8021
Other_Other-Ngru_14084_OG111
1.8487
Other_Other-Ngru_10869_OG111
2.019
Other_Other-Ngru_1641_OG111
1.4784
2.0642
Other_Other-Ngru_7802_OG111
Other_Other-Ngru_7888_OG111
2.7407
1.9289
Other_Other-Ngru_6235_OG111
Cp_Diaphoretickes-Chsp_5057_OG111

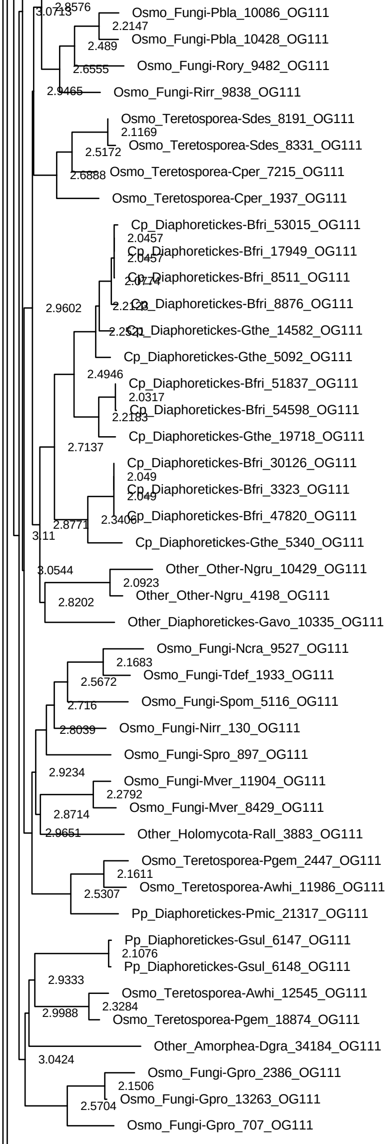
3.0713
2.8576
Osmo_Fungi-Pbla_10086_OG111
2.2147
Osmo_Fungi-Pbla_10428_OG111
2.489
Osmo_Fungi-Rory_9482_OG111
2.6555
2.9465
Osmo_Fungi-Rirr_9838_OG111
Osmo_Teretosporea-Sdes_8191_OG111
2.1169
Osmo_Teretosporea-Sdes_8331_OG111
2.5172
Osmo_Teretosporea-Cper_7215_OG111
2.6888
Osmo_Teretosporea-Cper_1937_OG111
Cp_Diaphoretickes-Bfri_53015_OG111
2.0457
Cp_Diaphoretickes-Bfri_17949_OG111
2.0457
Cp_Diaphoretickes-Bfri_8511_OG111
2.0774
2.9602
2.2123
Cp_Diaphoretickes-Bfri_8876_OG111
2.2521
Cp_Diaphoretickes-Gthe_14582_OG111
Cp_Diaphoretickes-Gthe_5092_OG111
2.4946
Cp_Diaphoretickes-Bfri_51837_OG111
2.0317
Cp_Diaphoretickes-Bfri_54598_OG111
2.2183
Cp_Diaphoretickes-Gthe_19718_OG111
2.7137
Cp_Diaphoretickes-Bfri_30126_OG111
2.049
Cp_Diaphoretickes-Bfri_3323_OG111
2.049
Cp_Diaphoretickes-Bfri_47820_OG111
2.3406
2.8771
3.11
Cp_Diaphoretickes-Gthe_5340_OG111
3.0544
Other_Other-Ngru_10429_OG111
2.0923
Other_Other-Ngru_4198_OG111
2.8202
Other_Diaphoretickes-Gavo_10335_OG111
Osmo_Fungi-Ncra_9527_OG111
2.1683
Osmo_Fungi-Tdef_1933_OG111
2.5672
Osmo_Fungi-Spom_5116_OG111
2.716
2.8039
Osmo_Fungi-Nirr_130_OG111
Osmo_Fungi-Spro_897_OG111
2.9234
Osmo_Fungi-Mver_11904_OG111
2.2792
Osmo_Fungi-Mver_8429_OG111
2.8714
2.9651
Other_Holomycota-Rall_3883_OG111
Osmo_Teretosporea-Pgem_2447_OG111
2.1611
Osmo_Teretosporea-Awhi_11986_OG111
2.5307
Pp_Diaphoretickes-Pmic_21317_OG111
Pp_Diaphoretickes-Gsul_6147_OG111
2.1076
Pp_Diaphoretickes-Gsul_6148_OG111
2.9333
Osmo_Teretosporea-Awhi_12545_OG111
2.3284
2.9988
Osmo_Teretosporea-Pgem_18874_OG111
Other_Amorphea-Dgra_34184_OG111
3.0424
Osmo_Fungi-Gpro_2386_OG111
2.1506
Osmo_Fungi-Gpro_13263_OG111
2.5704
Osmo_Fungi-Gpro_707_OG111

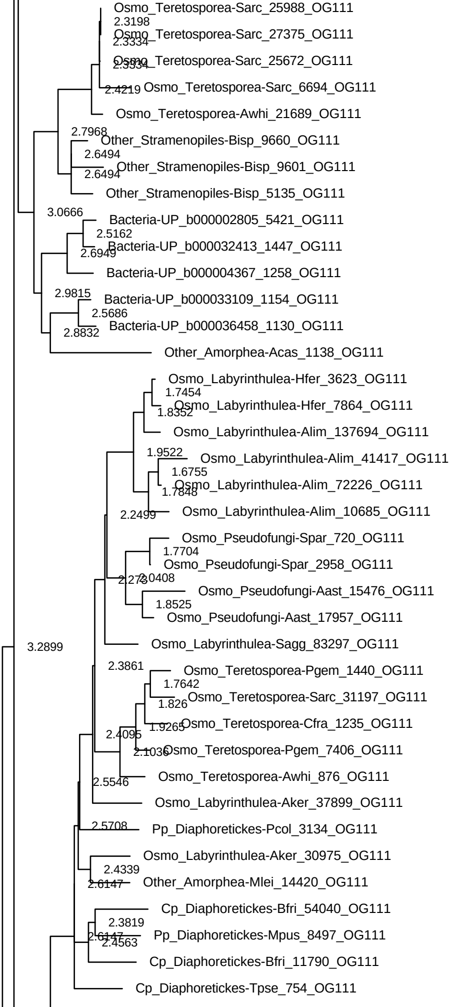

Osmo_Teretosporea-Sarc_25988_OG111
2.3198
Osmo_Teretosporea-Sarc_27375_OG111
2.3334
Osmo_Teretosporea-Sarc_25672_OG111
2.3334
2.4219
Osmo_Teretosporea-Sarc_6694_OG111
Osmo_Teretosporea-Awhi_21689_OG111
2.7968
Other_Stramenopiles-Bisp_9660_OG111
2.6494
Other_Stramenopiles-Bisp_9601_OG111
2.6494
Other_Stramenopiles-Bisp_5135_OG111
3.0666
Bacteria-UP_b000002805_5421_OG111
2.5162
Bacteria-UP_b000032413_1447_OG111
2.6949
Bacteria-UP_b000004367_1258_OG111
2.9815
Bacteria-UP_b000033109_1154_OG111
2.5686
Bacteria-UP_b000036458_1130_OG111
2.8832
Other_Amorphea-Acas_1138_OG111
Osmo_Labyrinthulea-Hfer_3623_OG111
1.7454
Osmo_Labyrinthulea-Hfer_7864_OG111
1.8352
Osmo_Labyrinthulea-Alim_137694_OG111
1.9522
Osmo_Labyrinthulea-Alim_41417_OG111
1.6755
Osmo_Labyrinthulea-Alim_72226_OG111
1.7848
2.2499
Osmo_Labyrinthulea-Alim_10685_OG111
Osmo_Pseudofungi-Spar_720_OG111
1.7704
Osmo_Pseudofungi-Spar_2958_OG111
2.273
2.0408
Osmo_Pseudofungi-Aast_15476_OG111
1.8525
Osmo_Pseudofungi-Aast_17957_OG111
3.2899
Osmo_Labyrinthulea-Sagg_83297_OG111
2.3861
Osmo_Teretosporea-Pgem_1440_OG111
1.7642
Osmo_Teretosporea-Sarc_31197_OG111
1.826
Osmo_Teretosporea-Cfra_1235_OG111
1.9265
2.4095
Osmo_Teretosporea-Pgem_7406_OG111
2.1036
Osmo_Teretosporea-Awhi_876_OG111
2.5546
Osmo_Labyrinthulea-Aker_37899_OG111
2.5708
Pp_Diaphoretickes-Pcol_3134_OG111
Osmo_Labyrinthulea-Aker_30975_OG111
2.4339
2.6147
Other_Amorphea-Mlei_14420_OG111
Cp_Diaphoretickes-Bfri_54040_OG111
2.3819
2.6147
Pp_Diaphoretickes-Mpus_8497_OG111
2.4563
Cp_Diaphoretickes-Bfri_11790_OG111
Cp_Diaphoretickes-Tpse_754_OG111

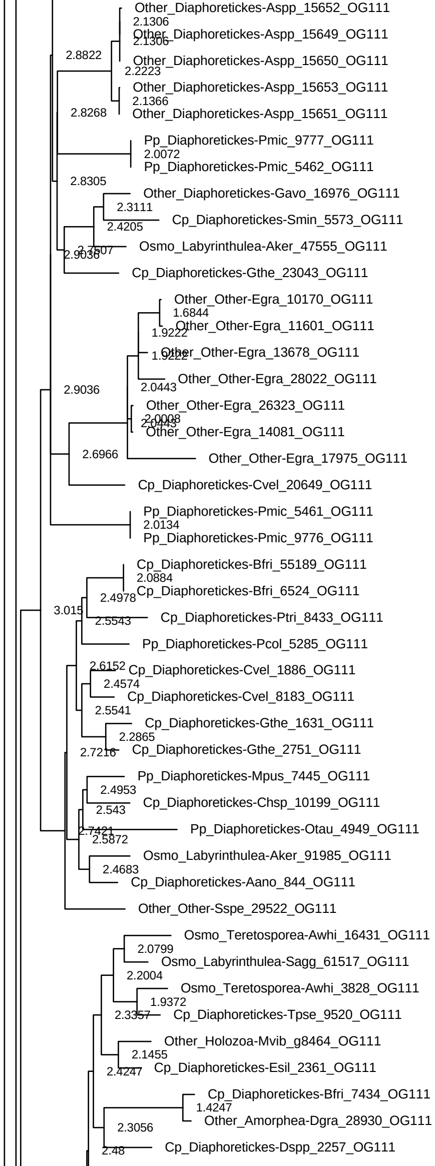
Other_Diaphoretickes-Aspp_15652_OG111
2.1306
Other_Diaphoretickes-Aspp_15649_OG111
2.1306
2.8822
Other_Diaphoretickes-Aspp_15650_OG111
2.2223
Other_Diaphoretickes-Aspp_15653_OG111
2.1366
2.8268
Other_Diaphoretickes-Aspp_15651_OG111
Pp_Diaphoretickes-Pmic_9777_OG111
2.0072
Pp_Diaphoretickes-Pmic_5462_OG111
2.8305
Other_Diaphoretickes-Gavo_16976_OG111
2.3111
Cp_Diaphoretickes-Smin_5573_OG111
2.4205
Osmo_Labyrinthulea-Aker_47555_OG111
2.7507
2.9036
Cp_Diaphoretickes-Gthe_23043_OG111
Other_Other-Egra_10170_OG111
1.6844
Other_Other-Egra_11601_OG111
1.9222
Other_Other-Egra_13678_OG111
1.9222
Other_Other-Egra_28022_OG111
2.0443
2.9036
Other_Other-Egra_26323_OG111
2.0008
2.0443
Other_Other-Egra_14081_OG111
2.6966
Other_Other-Egra_17975_OG111
Cp_Diaphoretickes-Cvel_20649_OG111
Pp_Diaphoretickes-Pmic_5461_OG111
2.0134
Pp_Diaphoretickes-Pmic_9776_OG111
Cp_Diaphoretickes-Bfri_55189_OG111
2.0884
Cp_Diaphoretickes-Bfri_6524_OG111
2.4978
3.015
Cp_Diaphoretickes-Ptri_8433_OG111
2.5543
Pp_Diaphoretickes-Pcol_5285_OG111
2.6152
Cp_Diaphoretickes-Cvel_1886_OG111
2.4574
Cp_Diaphoretickes-Cvel_8183_OG111
2.5541
Cp_Diaphoretickes-Gthe_1631_OG111
2.2865
2.7216
Cp_Diaphoretickes-Gthe_2751_OG111
Pp_Diaphoretickes-Mpus_7445_OG111
2.4953
Cp_Diaphoretickes-Chsp_10199_OG111
2.543
2.7421
Pp_Diaphoretickes-Otau_4949_OG111
2.5872
Osmo_Labyrinthulea-Aker_91985_OG111
2.4683
Cp_Diaphoretickes-Aano_844_OG111
Other_Other-Sspe_29522_OG111
Osmo_Teretosporea-Awhi_16431_OG111
2.0799
Osmo_Labyrinthulea-Sagg_61517_OG111
2.2004
Osmo_Teretosporea-Awhi_3828_OG111
1.9372
2.3357
Cp_Diaphoretickes-Tpse_9520_OG111
Other_Holozoa-Mvib_g8464_OG111
2.1455
2.4247
Cp_Diaphoretickes-Esil_2361_OG111
Cp_Diaphoretickes-Bfri_7434_OG111
1.4247
Other_Amorphea-Dgra_28930_OG111
2.3056
Cp_Diaphoretickes-Dspp_2257_OG111
2.48

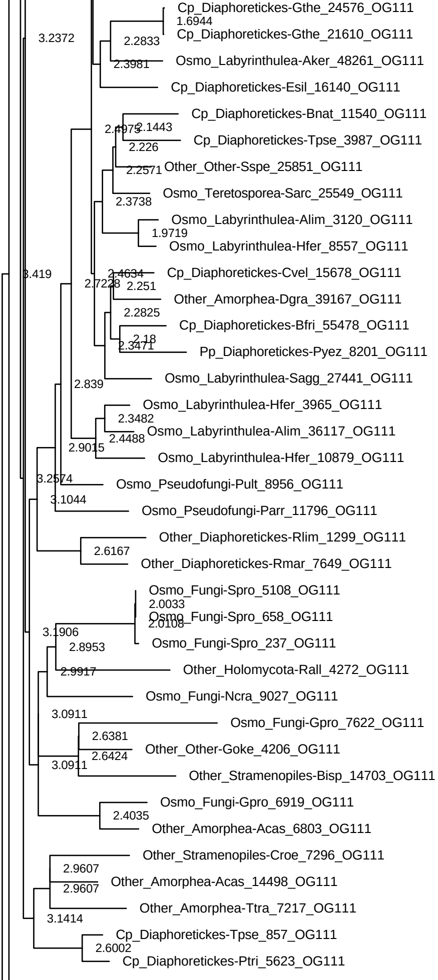
Cp_Diaphoretickes-Gthe_24576_OG111
1.6944
Cp_Diaphoretickes-Gthe_21610_OG111
3.2372
2.2833
Osmo_Labyrinthulea-Aker_48261_OG111
2.3981
Cp_Diaphoretickes-Esil_16140_OG111
Cp_Diaphoretickes-Bnat_11540_OG111
2.4975
2.1443
Cp_Diaphoretickes-Tpse_3987_OG111
2.226
Other_Other-Sspe_25851_OG111
2.2571
Osmo_Teretosporea-Sarc_25549_OG111
2.3738
Osmo_Labyrinthulea-Alim_3120_OG111
1.9719
Osmo_Labyrinthulea-Hfer_8557_OG111
3.419
2.4634
Cp_Diaphoretickes-Cvel_15678_OG111
2.7228
2.251
Other_Amorphea-Dgra_39167_OG111
2.2825
Cp_Diaphoretickes-Bfri_55478_OG111
2.18
2.3471
Pp_Diaphoretickes-Pyez_8201_OG111
Osmo_Labyrinthulea-Sagg_27441_OG111
2.839
Osmo_Labyrinthulea-Hfer_3965_OG111
2.3482
Osmo_Labyrinthulea-Alim_36117_OG111
2.4488
2.9015
Osmo_Labyrinthulea-Hfer_10879_OG111
3.2574
Osmo_Pseudofungi-Pult_8956_OG111
3.1044
Osmo_Pseudofungi-Parr_11796_OG111
Other_Diaphoretickes-Rlim_1299_OG111
2.6167
Other_Diaphoretickes-Rmar_7649_OG111
Osmo_Fungi-Spro_5108_OG111
2.0033
Osmo_Fungi-Spro_658_OG111
2.0108
3.1906
Osmo_Fungi-Spro_237_OG111
2.8953
2.9917
Other_Holomycota-Rall_4272_OG111
Osmo_Fungi-Ncra_9027_OG111
3.0911
Osmo_Fungi-Gpro_7622_OG111
2.6381
Other_Other-Goke_4206_OG111
2.6424
3.0911
Other_Stramenopiles-Bisp_14703_OG111
Osmo_Fungi-Gpro_6919_OG111
2.4035
Other_Amorphea-Acas_6803_OG111
Other_Stramenopiles-Croe_7296_OG111
2.9607
Other_Amorphea-Acas_14498_OG111
2.9607
Other_Amorphea-Ttra_7217_OG111
3.1414
Cp_Diaphoretickes-Tpse_857_OG111
2.6002
Cp_Diaphoretickes-Ptri_5623_OG111

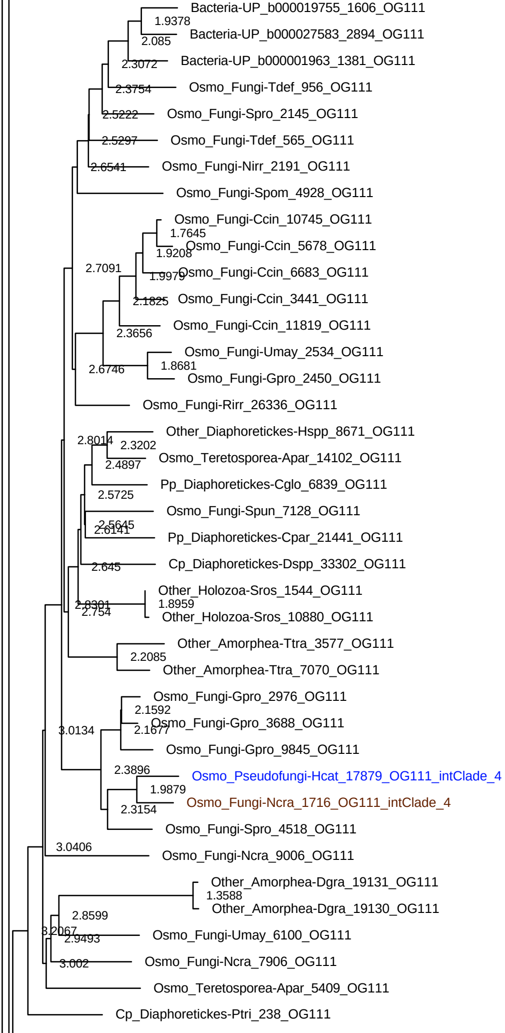
Bacteria-UP_b000019755_1606_OG111
1.9378
Bacteria-UP_b000027583_2894_OG111
2.085
Bacteria-UP_b000001963_1381_OG111
2.3072
Osmo_Fungi-Tdef_956_OG111
2.3754
Osmo_Fungi-Spro_2145_OG111
2.5222
Osmo_Fungi-Tdef_565_OG111
2.5297
Osmo_Fungi-Nirr_2191_OG111
2.6541
Osmo_Fungi-Spom_4928_OG111
Osmo_Fungi-Ccin_10745_OG111
1.7645
Osmo_Fungi-Ccin_5678_OG111
1.9208
2.7091
Osmo_Fungi-Ccin_6683_OG111
1.9979
Osmo_Fungi-Ccin_3441_OG111
2.1825
Osmo_Fungi-Ccin_11819_OG111
2.3656
Osmo_Fungi-Umay_2534_OG111
1.8681
2.6746
Osmo_Fungi-Gpro_2450_OG111
Osmo_Fungi-Rirr_26336_OG111
Other_Diaphoretickes-Hspp_8671_OG111
2.8014
2.3202
Osmo_Teretosporea-Apar_14102_OG111
2.4897
Pp_Diaphoretickes-Cglo_6839_OG111
2.5725
Osmo_Fungi-Spun_7128_OG111
2.5645
2.6141
Pp_Diaphoretickes-Cpar_21441_OG111
2.645
Cp_Diaphoretickes-Dspp_33302_OG111
Other_Holozoa-Sros_1544_OG111
2.8301
1.8959
2.754
Other_Holozoa-Sros_10880_OG111
Other_Amorphea-Ttra_3577_OG111
2.2085
Other_Amorphea-Ttra_7070_OG111
Osmo_Fungi-Gpro_2976_OG111
2.1592
Osmo_Fungi-Gpro_3688_OG111
3.0134
2.1677
Osmo_Fungi-Gpro_9845_OG111
2.3896
Osmo_Pseudofungi-Hcat_17879_OG111_intClade_4
1.9879
Osmo_Fungi-Ncra_1716_OG111_intClade_4
2.3154
Osmo_Fungi-Spro_4518_OG111
3.0406
Osmo_Fungi-Ncra_9006_OG111
Other_Amorphea-Dgra_19131_OG111
1.3588
Other_Amorphea-Dgra_19130_OG111
2.8599
3.2067
Osmo_Fungi-Umay_6100_OG111
2.9493
Osmo_Fungi-Ncra_7906_OG111
3.002
Osmo_Teretosporea-Apar_5409_OG111
Cp_Diaphoretickes-Ptri_238_OG111

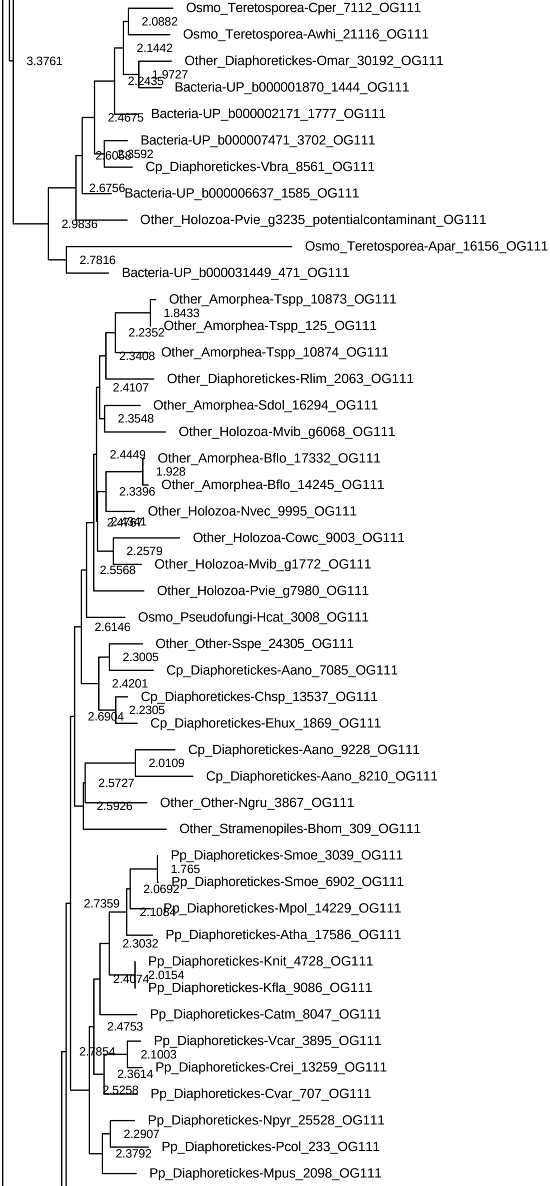
Osmo_Teretosporea-Cper_7112_OG111
2.0882
Osmo_Teretosporea-Awhi_21116_OG111
2.1442
3.3761
Other_Diaphoretickes-Omar_30192_OG111
1.9727
2.2435
Bacteria-UP_b000001870_1444_OG111
2.4675
Bacteria-UP_b000002171_1777_OG111
Bacteria-UP_b000007471_3702_OG111
Cp_Diaphoretickes-Vbra_8561_OG111
2.6756
Bacteria-UP_b000006637_1585_OG111
2.9836
Other_Holozoa-Pvie_g3235_potentialcontaminant_OG111
Osmo_Teretosporea-Apar_16156_OG111
2.7816
Bacteria-UP_b000031449_471_OG111
Other_Amorphea-Tspp_10873_OG111
1.8433
2.2352
Other_Amorphea-Tspp_125_OG111
2.3408
Other_Amorphea-Tspp_10874_OG111
Other_Diaphoretickes-Rlim_2063_OG111
2.4107
Other_Amorphea-Sdol_16294_OG111
2.3548
Other_Holozoa-Mvib_g6068_OG111
2.4449
Other_Amorphea-Bflo_17332_OG111
1.928
2.3396
Other_Amorphea-Bflo_14245_OG111
Other_Holozoa-Nvec_9995_OG111
Other_Holozoa-Cowc_9003_OG111
2.2579
2.5568
Other_Holozoa-Mvib_g1772_OG111
Other_Holozoa-Pvie_g7980_OG111
Osmo_Pseudofungi-Hcat_3008_OG111
2.6146
Other_Other-Sspe_24305_OG111
2.3005
Cp_Diaphoretickes-Aano_7085_OG111
2.4201
Cp_Diaphoretickes-Chsp_13537_OG111
2.2305
2.6904
Cp_Diaphoretickes-Ehux_1869_OG111
Cp_Diaphoretickes-Aano_9228_OG111
2.0109
2.5727
Cp_Diaphoretickes-Aano_8210_OG111
2.5926
Other_Other-Ngru_3867_OG111
Other_Stramenopiles-Bhom_309_OG111
Pp_Diaphoretickes-Smoe_3039_OG111
1.765
2.0692
Pp_Diaphoretickes-Smoe_6902_OG111
2.7359
2.1084
Pp_Diaphoretickes-Mpol_14229_OG111
2.3032
Pp_Diaphoretickes-Atha_17586_OG111
Pp_Diaphoretickes-Knit_4728_OG111
2.0154
2.4074
Pp_Diaphoretickes-Kfla_9086_OG111
Pp_Diaphoretickes-Catm_8047_OG111
2.4753
Pp_Diaphoretickes-Vcar_3895_OG111
2.1003
2.7854
2.3614
Pp_Diaphoretickes-Crei_13259_OG111
2.5258
Pp_Diaphoretickes-Cvar_707_OG111
Pp_Diaphoretickes-Npyr_25528_OG111
2.2907
2.3792
Pp_Diaphoretickes-Pcol_233_OG111
Pp_Diaphoretickes-Mpus_2098_OG111

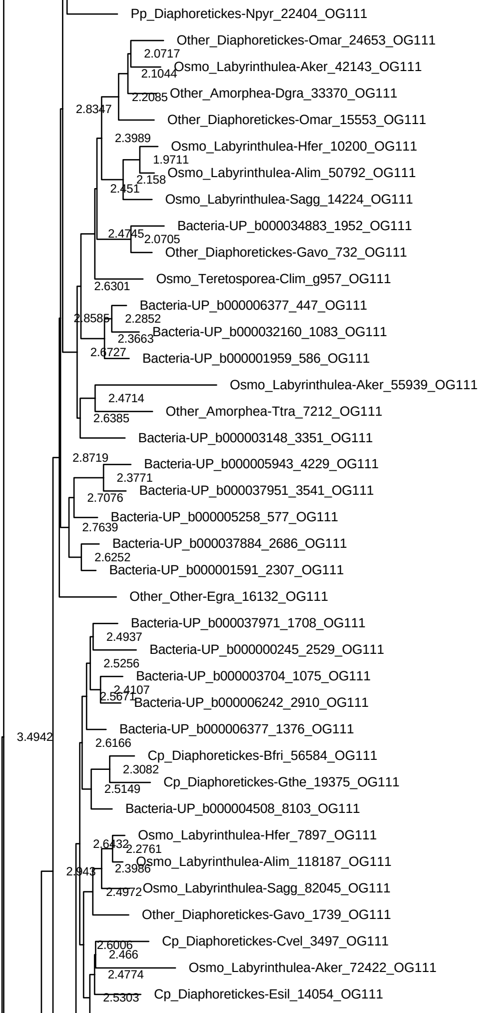
Pp_Diaphoretickes-Npyr_22404_OG111
Other_Diaphoretickes-Omar_24653_OG111
2.0717
Osmo_Labyrinthulea-Aker_42143_OG111
2.1044
Other_Amorphea-Dgra_33370_OG111
2.2085
2.8347
Other_Diaphoretickes-Omar_15553_OG111
2.3989
Osmo_Labyrinthulea-Hfer_10200_OG111
1.9711
Osmo_Labyrinthulea-Alim_50792_OG111
2.158
2.451
Osmo_Labyrinthulea-Sagg_14224_OG111
Bacteria-UP_b000034883_1952_OG111
2.4745
2.0705
Other_Diaphoretickes-Gavo_732_OG111
Osmo_Teretosporea-Clim_g957_OG111
2.6301
Bacteria-UP_b000006377_447_OG111
2.8585
2.2852
Bacteria-UP_b000032160_1083_OG111
2.3663
2.6727
Bacteria-UP_b000001959_586_OG111
Osmo_Labyrinthulea-Aker_55939_OG111
2.4714
Other_Amorphea-Ttra_7212_OG111
2.6385
Bacteria-UP_b000003148_3351_OG111
2.8719
Bacteria-UP_b000005943_4229_OG111
2.3771
Bacteria-UP_b000037951_3541_OG111
2.7076
Bacteria-UP_b000005258_577_OG111
2.7639
Bacteria-UP_b000037884_2686_OG111
2.6252
Bacteria-UP_b000001591_2307_OG111
Other_Other-Egra_16132_OG111
Bacteria-UP_b000037971_1708_OG111
2.4937
Bacteria-UP_b000000245_2529_OG111
2.5256
Bacteria-UP_b000003704_1075_OG111
2.4107
2.5671
Bacteria-UP_b000006242_2910_OG111
Bacteria-UP_b000006377_1376_OG111
3.4942
2.6166
Cp_Diaphoretickes-Bfri_56584_OG111
2.3082
Cp_Diaphoretickes-Gthe_19375_OG111
2.5149
Bacteria-UP_b000004508_8103_OG111
Osmo_Labyrinthulea-Hfer_7897_OG111
2.6432
2.2761
Osmo_Labyrinthulea-Alim_118187_OG111
2.943
2.3986
Osmo_Labyrinthulea-Sagg_82045_OG111
2.4972
Other_Diaphoretickes-Gavo_1739_OG111
Cp_Diaphoretickes-Cvel_3497_OG111
2.6006
2.466
Osmo_Labyrinthulea-Aker_72422_OG111
2.4774
Cp_Diaphoretickes-Esil_14054_OG111
2.5303

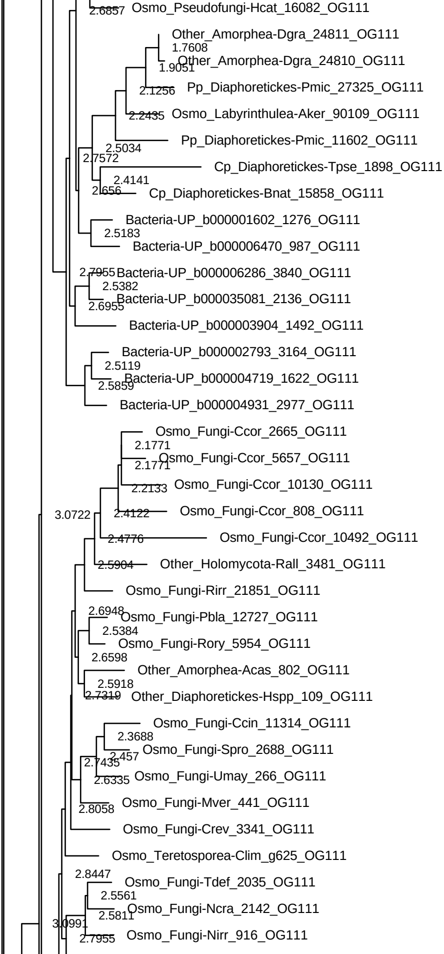

2.6857
Osmo_Pseudofungi-Hcat_16082_OG111
Other_Amorphea-Dgra_24811_OG111
1.7608
Other_Amorphea-Dgra_24810_OG111
1.9051
Pp_Diaphoretickes-Pmic_27325_OG111
2.1256
Osmo_Labyrinthulea-Aker_90109_OG111
2.2435
Pp_Diaphoretickes-Pmic_11602_OG111
2.5034
2.7572
Cp_Diaphoretickes-Tpse_1898_OG111
2.4141
2.656
Cp_Diaphoretickes-Bnat_15858_OG111
Bacteria-UP_b000001602_1276_OG111
2.5183
Bacteria-UP_b000006470_987_OG111
2.7955
Bacteria-UP_b000006286_3840_OG111
2.5382
2.6955
Bacteria-UP_b000035081_2136_OG111
Bacteria-UP_b000003904_1492_OG111
Bacteria-UP_b000002793_3164_OG111
2.5119
2.5859
Bacteria-UP_b000004719_1622_OG111
Bacteria-UP_b000004931_2977_OG111
Osmo_Fungi-Ccor_2665_OG111
2.1771
Osmo_Fungi-Ccor_5657_OG111
2.1771
Osmo_Fungi-Ccor_10130_OG111
2.2133
3.0722
Osmo_Fungi-Ccor_808_OG111
2.4122
Osmo_Fungi-Ccor_10492_OG111
2.4776
Other_Holomycota-Rall_3481_OG111
2.5904
Osmo_Fungi-Rirr_21851_OG111
2.6948
Osmo_Fungi-Pbla_12727_OG111
2.5384
Osmo_Fungi-Rory_5954_OG111
2.6598
Other_Amorphea-Acas_802_OG111
2.5918
2.7319
Other_Diaphoretickes-Hspp_109_OG111
Osmo_Fungi-Ccin_11314_OG111
2.3688
Osmo_Fungi-Spro_2688_OG111
2.457
2.7435
Osmo_Fungi-Umay_266_OG111
2.6335
Osmo_Fungi-Mver_441_OG111
2.8058
Osmo_Fungi-Crev_3341_OG111
Osmo_Teretosporea-Clim_g625_OG111
2.8447
Osmo_Fungi-Tdef_2035_OG111
2.5561
Osmo_Fungi-Ncra_2142_OG111
2.5811
3.0991
Osmo_Fungi-Nirr_916_OG111
2.7955

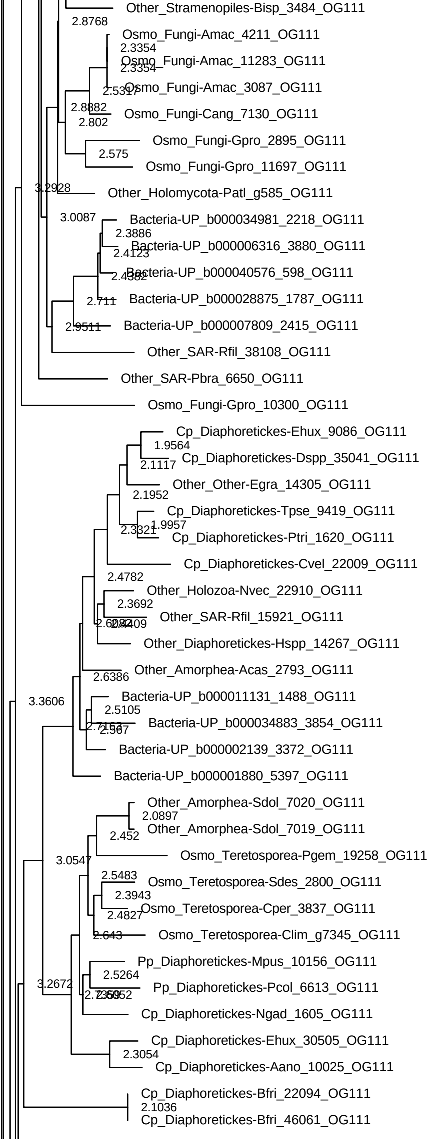

Other_Stramenopiles-Bisp_3484_OG111
2.8768
Osmo_Fungi-Amac_4211_OG111
2.3354
Osmo_Fungi-Amac_11283_OG111
2.3354
Osmo_Fungi-Amac_3087_OG111
2.5317
2.8882
Osmo_Fungi-Cang_7130_OG111
2.802
Osmo_Fungi-Gpro_2895_OG111
2.575
Osmo_Fungi-Gpro_11697_OG111
3.2928
Other_Holomycota-Patl_g585_OG111
3.0087
Bacteria-UP_b000034981_2218_OG111
2.3886
Bacteria-UP_b000006316_3880_OG111
2.4123
Bacteria-UP_b000040576_598_OG111
2.4382
Bacteria-UP_b000028875_1787_OG111
2.711
2.9511
Bacteria-UP_b000007809_2415_OG111
Other_SAR-Rfil_38108_OG111
Other_SAR-Pbra_6650_OG111
Osmo_Fungi-Gpro_10300_OG111
Cp_Diaphoretickes-Ehux_9086_OG111
1.9564
Cp_Diaphoretickes-Dspp_35041_OG111
2.1117
Other_Other-Egra_14305_OG111
2.1952
Cp_Diaphoretickes-Tpse_9419_OG111
2.3321
1.9957
Cp_Diaphoretickes-Ptri_1620_OG111
Cp_Diaphoretickes-Cvel_22009_OG111
2.4782
Other_Holozoa-Nvec_22910_OG111
2.3692
Other_SAR-Rfil_15921_OG111
Other_Diaphoretickes-Hspp_14267_OG111
Other_Amorphea-Acas_2793_OG111
2.6386
3.3606
Bacteria-UP_b000011131_1488_OG111
2.5105
Bacteria-UP_b000034883_3854_OG111
Bacteria-UP_b000002139_3372_OG111
Bacteria-UP_b000001880_5397_OG111
Other_Amorphea-Sdol_7020_OG111
2.0897
Other_Amorphea-Sdol_7019_OG111
2.452
3.0547
Osmo_Teretosporea-Pgem_19258_OG111
2.5483
Osmo_Teretosporea-Sdes_2800_OG111
2.3943
Osmo_Teretosporea-Cper_3837_OG111
2.4827
2.643
Osmo_Teretosporea-Clim_g7345_OG111
Pp_Diaphoretickes-Mpus_10156_OG111
2.5264
3.2672
Pp_Diaphoretickes-Pcol_6613_OG111
Cp_Diaphoretickes-Ngad_1605_OG111
Cp_Diaphoretickes-Ehux_30505_OG111
2.3054
Cp_Diaphoretickes-Aano_10025_OG111
Cp_Diaphoretickes-Bfri_22094_OG111
2.1036
Cp_Diaphoretickes-Bfri_46061_OG111

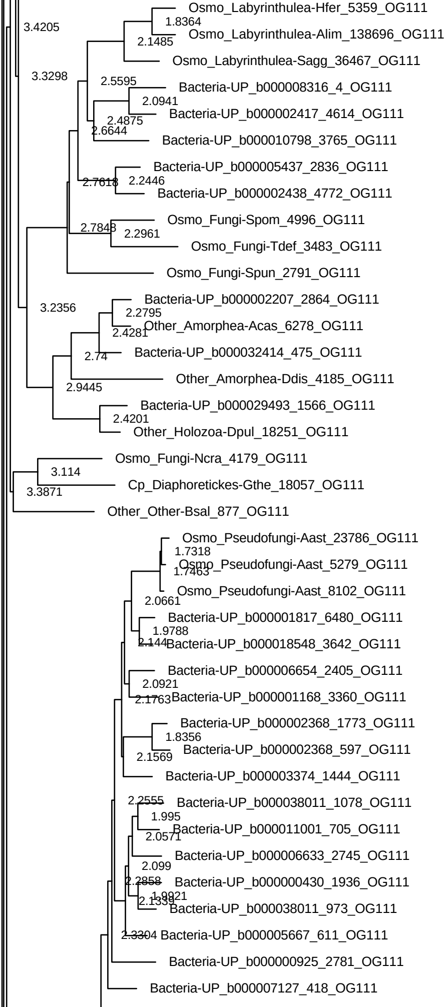

Osmo_Labyrinthulea-Hfer_5359_OG111
1.8364
Osmo_Labyrinthulea-Alim_138696_OG111
2.1485
3.4205
Osmo_Labyrinthulea-Sagg_36467_OG111
3.3298
2.5595
Bacteria-UP_b000008316_4_OG111
2.0941
Bacteria-UP_b000002417_4614_OG111
2.4875
2.6644
Bacteria-UP_b000010798_3765_OG111
Bacteria-UP_b000005437_2836_OG111
2.7618
2.2446
Bacteria-UP_b000002438_4772_OG111
Osmo_Fungi-Spom_4996_OG111
2.7848
2.2961
Osmo_Fungi-Tdef_3483_OG111
Osmo_Fungi-Spun_2791_OG111
Bacteria-UP_b000002207_2864_OG111
3.2356
2.2795
2.4281
Other_Amorphea-Acas_6278_OG111
2.74
Bacteria-UP_b000032414_475_OG111
Other_Amorphea-Ddis_4185_OG111
2.9445
Bacteria-UP_b000029493_1566_OG111
2.4201
Other_Holozoa-Dpul_18251_OG111
Osmo_Fungi-Ncra_4179_OG111
3.114
Cp_Diaphoretickes-Gthe_18057_OG111
3.3871
Other_Other-Bsal_877_OG111
Osmo_Pseudofungi-Aast_23786_OG111
1.7318
Osmo_Pseudofungi-Aast_5279_OG111
1.7463
Osmo_Pseudofungi-Aast_8102_OG111
2.0661
Bacteria-UP_b000001817_6480_OG111
1.9788
2.144
Bacteria-UP_b000018548_3642_OG111
Bacteria-UP_b000006654_2405_OG111
2.0921
2.1763
Bacteria-UP_b000001168_3360_OG111
Bacteria-UP_b000002368_1773_OG111
1.8356
Bacteria-UP_b000002368_597_OG111
2.1569
Bacteria-UP_b000003374_1444_OG111
2.2555
Bacteria-UP_b000038011_1078_OG111
1.995
Bacteria-UP_b000011001_705_OG111
2.0571
Bacteria-UP_b000006633_2745_OG111
2.099
2.2858
Bacteria-UP_b000000430_1936_OG111
1.9921
2.1339
Bacteria-UP_b000038011_973_OG111
2.3304
Bacteria-UP_b000005667_611_OG111
Bacteria-UP_b000000925_2781_OG111
Bacteria-UP_b000007127_418_OG111

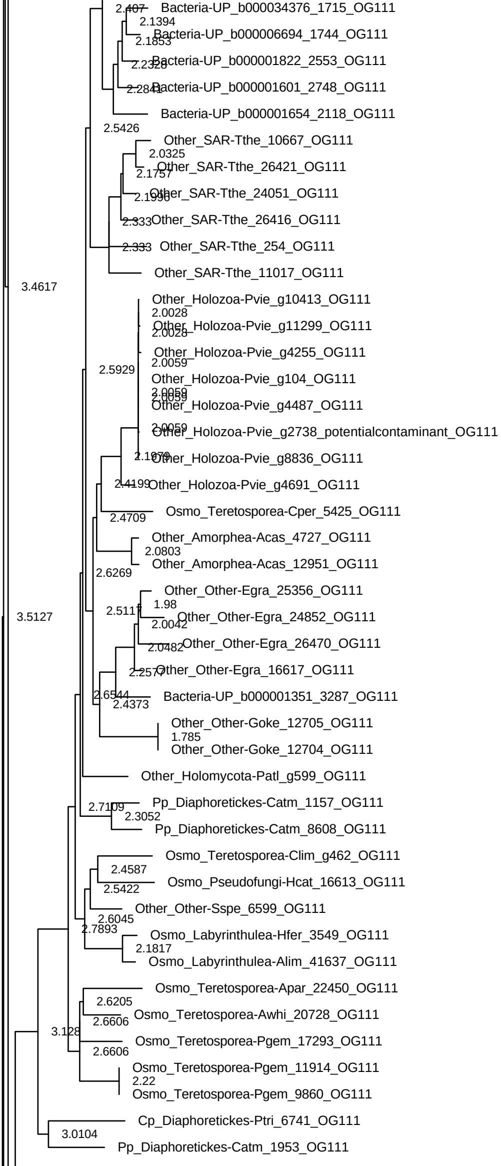
2.407
Bacteria-UP_b000034376_1715_OG111
2.1394
Bacteria-UP_b000006694_1744_OG111
2.1853
Bacteria-UP_b000001822_2553_OG111
2.2328
Bacteria-UP_b000001601_2748_OG111
2.2841
Bacteria-UP_b000001654_2118_OG111
2.5426
Other_SAR-Tthe_10667_OG111
2.0325
Other_SAR-Tthe_26421_OG111
2.1757
Other_SAR-Tthe_24051_OG111
2.1996
Other_SAR-Tthe_26416_OG111
2.333
Other_SAR-Tthe_254_OG111
2.333
Other_SAR-Tthe_11017_OG111
3.4617
Other_Holozoa-Pvie_g10413_OG111
2.0028
Other_Holozoa-Pvie_g11299_OG111
2.0028
Other_Holozoa-Pvie_g4255_OG111
2.0059
2.5929
Other_Holozoa-Pvie_g104_OG111
2.0059
2.0059
Other_Holozoa-Pvie_g4487_OG111
2.0059
Other_Holozoa-Pvie_g2738_potentialcontaminant_OG111
2.1979
Other_Holozoa-Pvie_g8836_OG111
2.4199
Other_Holozoa-Pvie_g4691_OG111
Osmo_Teretosporea-Cper_5425_OG111
2.4709
Other_Amorphea-Acas_4727_OG111
2.0803
Other_Amorphea-Acas_12951_OG111
2.6269
Other_Other-Egra_25356_OG111
1.98
2.5117
Other_Other-Egra_24852_OG111
3.5127
2.0042
Other_Other-Egra_26470_OG111
2.0482
Other_Other-Egra_16617_OG111
2.2577
2.6544
Bacteria-UP_b000001351_3287_OG111
2.4373
Other_Other-Goke_12705_OG111
1.785
Other_Other-Goke_12704_OG111
Other_Holomycota-Patl_g599_OG111
2.7109
Pp_Diaphoretickes-Catm_1157_OG111
2.3052
Pp_Diaphoretickes-Catm_8608_OG111
Osmo_Teretosporea-Clim_g462_OG111
2.4587
Osmo_Pseudofungi-Hcat_16613_OG111
2.5422
Other_Other-Sspe_6599_OG111
2.6045
2.7893
Osmo_Labyrinthulea-Hfer_3549_OG111
2.1817
Osmo_Labyrinthulea-Alim_41637_OG111
Osmo_Teretosporea-Apar_22450_OG111
2.6205
Osmo_Teretosporea-Awhi_20728_OG111
2.6606
3.128
Osmo_Teretosporea-Pgem_17293_OG111
2.6606
Osmo_Teretosporea-Pgem_11914_OG111
2.22
Osmo_Teretosporea-Pgem_9860_OG111
Cp_Diaphoretickes-Ptri_6741_OG111
3.0104
Pp_Diaphoretickes-Catm_1953_OG111

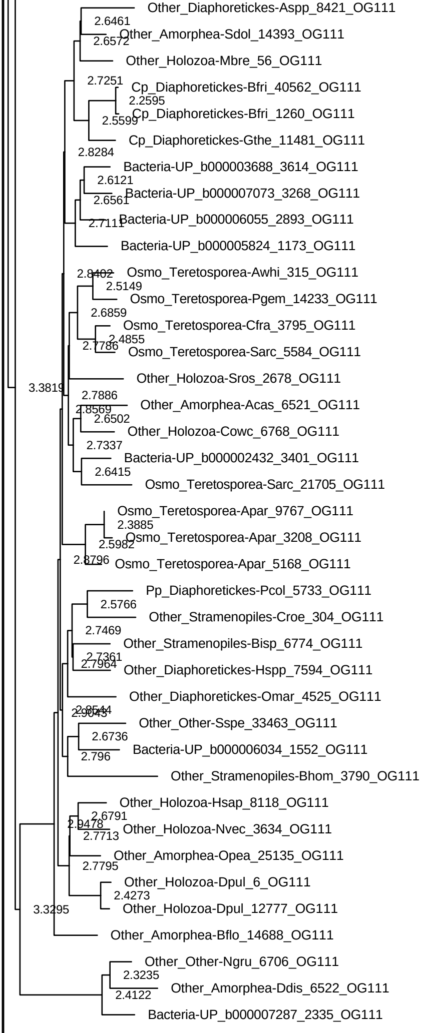
Other_Diaphoretickes-Aspp_8421_OG111
2.6461
Other_Amorphea-Sdol_14393_OG111
2.6572
Other_Holozoa-Mbre_56_OG111
2.7251
Cp_Diaphoretickes-Bfri_40562_OG111
2.2595
Cp_Diaphoretickes-Bfri_1260_OG111
2.5599
Cp_Diaphoretickes-Gthe_11481_OG111
2.8284
Bacteria-UP_b000003688_3614_OG111
2.6121
Bacteria-UP_b000007073_3268_OG111
2.6561
Bacteria-UP_b000006055_2893_OG111
2.7111
Bacteria-UP_b000005824_1173_OG111
2.8402
Osmo_Teretosporea-Awhi_315_OG111
2.5149
Osmo_Teretosporea-Pgem_14233_OG111
2.6859
Osmo_Teretosporea-Cfra_3795_OG111
2.4855
2.7786
Osmo_Teretosporea-Sarc_5584_OG111
Other_Holozoa-Sros_2678_OG111
3.3819
2.7886
Other_Amorphea-Acas_6521_OG111
2.8569
2.6502
Other_Holozoa-Cowc_6768_OG111
2.7337
Bacteria-UP_b000002432_3401_OG111
2.6415
Osmo_Teretosporea-Sarc_21705_OG111
Osmo_Teretosporea-Apar_9767_OG111
2.3885
Osmo_Teretosporea-Apar_3208_OG111
2.5982
2.8796
Osmo_Teretosporea-Apar_5168_OG111
Pp_Diaphoretickes-Pcol_5733_OG111
2.5766
Other_Stramenopiles-Croe_304_OG111
2.7469
Other_Stramenopiles-Bisp_6774_OG111
2.7361
2.7964
Other_Diaphoretickes-Hspp_7594_OG111
Other_Diaphoretickes-Omar_4525_OG111
2.8544
2.9043
Other_Other-Sspe_33463_OG111
2.6736
Bacteria-UP_b000006034_1552_OG111
2.796
Other_Stramenopiles-Bhom_3790_OG111
Other_Holozoa-Hsap_8118_OG111
2.6791
2.9478
Other_Holozoa-Nvec_3634_OG111
2.7713
Other_Amorphea-Opea_25135_OG111
2.7795
Other_Holozoa-Dpul_6_OG111
2.4273
Other_Holozoa-Dpul_12777_OG111
3.3295
Other_Amorphea-Bflo_14688_OG111
Other_Other-Ngru_6706_OG111
2.3235
Other_Amorphea-Ddis_6522_OG111
2.4122
Bacteria-UP_b000007287_2335_OG111

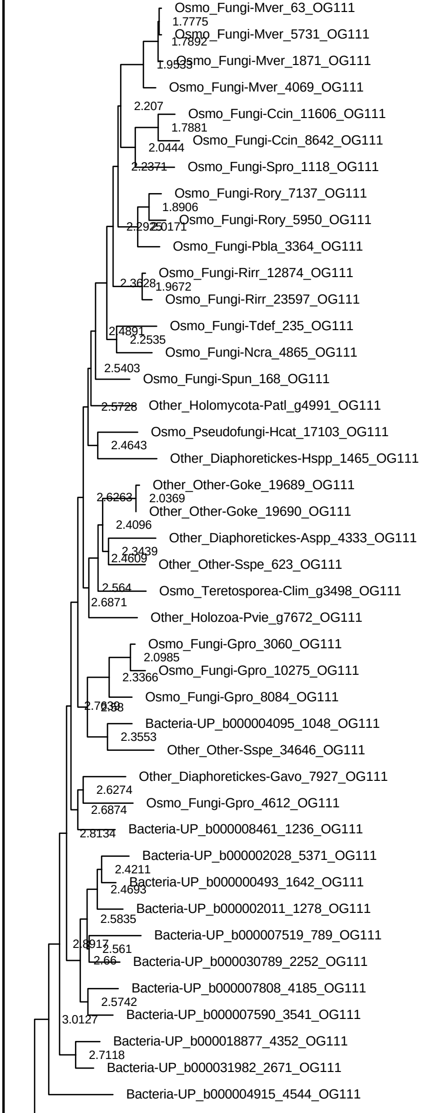
Osmo_Fungi-Mver_63_OG111
1.7775
Osmo_Fungi-Mver_5731_OG111
1.7892
Osmo_Fungi-Mver_1871_OG111
1.9533
Osmo_Fungi-Mver_4069_OG111
2.207
Osmo_Fungi-Ccin_11606_OG111
1.7881
Osmo_Fungi-Ccin_8642_OG111
2.0444
2.2371
Osmo_Fungi-Spro_1118_OG111
Osmo_Fungi-Rory_7137_OG111
1.8906
Osmo_Fungi-Rory_5950_OG111
2.2925
2.0171
Osmo_Fungi-Pbla_3364_OG111
Osmo_Fungi-Rirr_12874_OG111
2.3628
1.9672
Osmo_Fungi-Rirr_23597_OG111
Osmo_Fungi-Tdef_235_OG111
2.4891
2.2535
Osmo_Fungi-Ncra_4865_OG111
2.5403
Osmo_Fungi-Spun_168_OG111
2.5728
Other_Holomycota-Patl_g4991_OG111
Osmo_Pseudofungi-Hcat_17103_OG111
2.4643
Other_Diaphoretickes-Hspp_1465_OG111
Other_Other-Goke_19689_OG111
2.6263
2.0369
Other_Other-Goke_19690_OG111
2.4096
Other_Diaphoretickes-Aspp_4333_OG111
2.3439
2.4609
Other_Other-Sspe_623_OG111
2.564
Osmo_Teretosporea-Clim_g3498_OG111
2.6871
Other_Holozoa-Pvie_g7672_OG111
Osmo_Fungi-Gpro_3060_OG111
2.0985
Osmo_Fungi-Gpro_10275_OG111
2.3366
Osmo_Fungi-Gpro_8084_OG111
2.7639
2.38
Bacteria-UP_b000004095_1048_OG111
2.3553
Other_Other-Sspe_34646_OG111
Other_Diaphoretickes-Gavo_7927_OG111
2.6274
Osmo_Fungi-Gpro_4612_OG111
2.6874
Bacteria-UP_b000008461_1236_OG111
2.8134
Bacteria-UP_b000002028_5371_OG111
2.4211
Bacteria-UP_b000000493_1642_OG111
2.4693
Bacteria-UP_b000002011_1278_OG111
2.5835
Bacteria-UP_b000007519_789_OG111
2.8917
2.561
2.66
Bacteria-UP_b000030789_2252_OG111
Bacteria-UP_b000007808_4185_OG111
2.5742
Bacteria-UP_b000007590_3541_OG111
3.0127
Bacteria-UP_b000018877_4352_OG111
2.7118
Bacteria-UP_b000031982_2671_OG111
Bacteria-UP_b000004915_4544_OG111

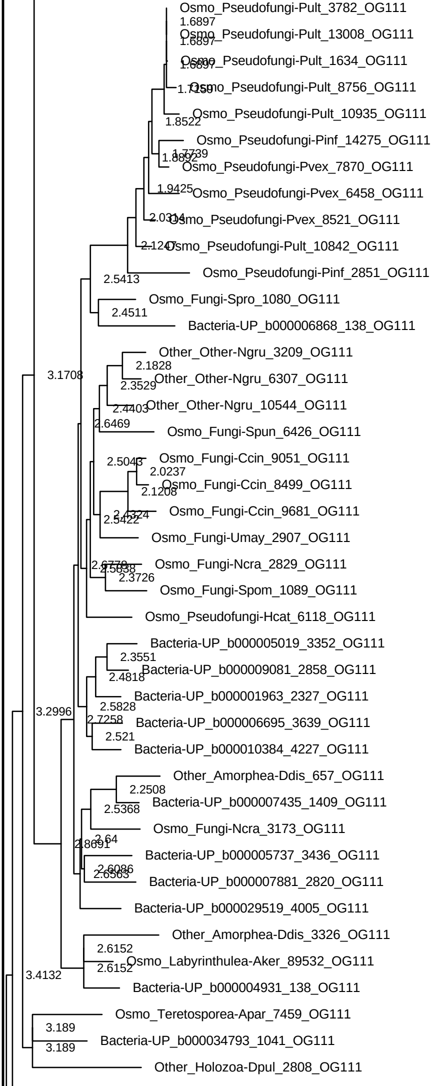

Osmo_Pseudofungi-Pult_3782_OG111
1.6897
Osmo_Pseudofungi-Pult_13008_OG111
1.6897
Osmo_Pseudofungi-Pult_1634_OG111
1.6897
Osmo_Pseudofungi-Pult_8756_OG111
1.7150
Osmo_Pseudofungi-Pult_10935_OG111
1.8522
Osmo_Pseudofungi-Pinf_14275_OG111
1.7739
1.8892
Osmo_Pseudofungi-Pvex_7870_OG111
1.9425
Osmo_Pseudofungi-Pvex_6458_OG111
2.0314
Osmo_Pseudofungi-Pvex_8521_OG111
2.1247
Osmo_Pseudofungi-Pult_10842_OG111
2.5413
Osmo_Pseudofungi-Pinf_2851_OG111
Osmo_Fungi-Spro_1080_OG111
2.4511
Bacteria-UP_b000006868_138_OG111
3.1708
Other_Other-Ngru_3209_OG111
2.1828
Other_Other-Ngru_6307_OG111
2.3529
2.4403
Other_Other-Ngru_10544_OG111
2.6469
Osmo_Fungi-Spun_6426_OG111
2.5043
Osmo_Fungi-Ccin_9051_OG111
2.0237
Osmo_Fungi-Ccin_8499_OG111
2.1208
2.4324
2.5422
Osmo_Fungi-Ccin_9681_OG111
Osmo_Fungi-Umay_2907_OG111
2.6778
2.5638
Osmo_Fungi-Ncra_2829_OG111
2.3726
Osmo_Fungi-Spom_1089_OG111
Osmo_Pseudofungi-Hcat_6118_OG111
Bacteria-UP_b000005019_3352_OG111
2.3551
Bacteria-UP_b000009081_2858_OG111
2.4818
Bacteria-UP_b000001963_2327_OG111
2.5828
3.2996
2.7258
Bacteria-UP_b000006695_3639_OG111
2.521
Bacteria-UP_b000010384_4227_OG111
Other_Amorphea-Ddis_657_OG111
2.2508
Bacteria-UP_b000007435_1409_OG111
2.5368
Osmo_Fungi-Ncra_3173_OG111
2.64
2.8691
Bacteria-UP_b000005737_3436_OG111
2.6086
2.6563
Bacteria-UP_b000007881_2820_OG111
Bacteria-UP_b000029519_4005_OG111
Other_Amorphea-Ddis_3326_OG111
2.6152
Osmo_Labyrinthulea-Aker_89532_OG111
2.6152
3.4132
Bacteria-UP_b000004931_138_OG111
Osmo_Teretosporea-Apar_7459_OG111
3.189
Bacteria-UP_b000034793_1041_OG111
3.189
Other_Holozoa-Dpul_2808_OG111

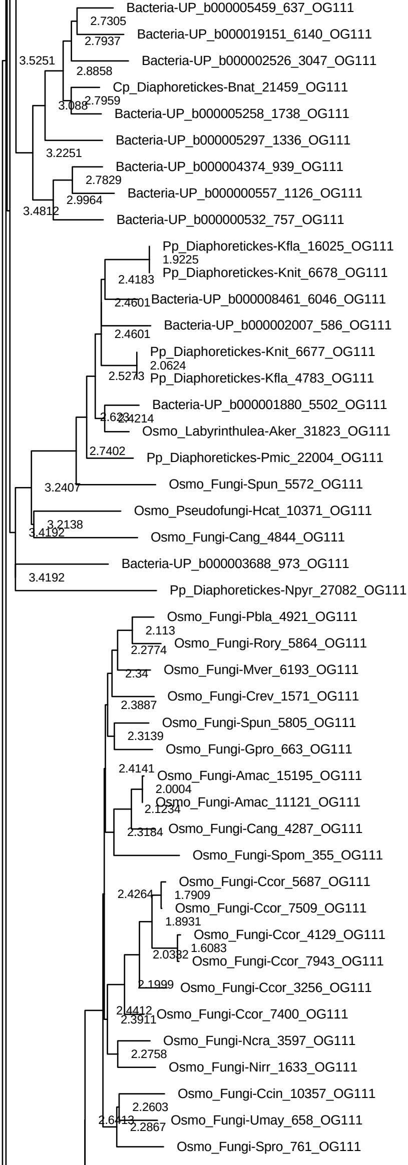
Bacteria-UP_b000005459_637_OG111
2.7305
Bacteria-UP_b000019151_6140_OG111
2.7937
3.5251
Bacteria-UP_b000002526_3047_OG111
2.8858
Cp_Diaphoretickes-Bnat_21459_OG111
2.7959
3.088
Bacteria-UP_b000005258_1738_OG111
Bacteria-UP_b000005297_1336_OG111
3.2251
Bacteria-UP_b000004374_939_OG111
2.7829
Bacteria-UP_b000000557_1126_OG111
2.9964
3.4812
Bacteria-UP_b000000532_757_OG111
Pp_Diaphoretickes-Kfla_16025_OG111
1.9225
Pp_Diaphoretickes-Knit_6678_OG111
2.4183
2.4601
Bacteria-UP_b000008461_6046_OG111
Bacteria-UP_b000002007_586_OG111
2.4601
Pp_Diaphoretickes-Knit_6677_OG111
2.0624
2.5273
Pp_Diaphoretickes-Kfla_4783_OG111
Bacteria-UP_b000001880_5502_OG111
2.623
2.4214
Osmo_Labyrinthulea-Aker_31823_OG111
2.7402
Pp_Diaphoretickes-Pmic_22004_OG111
3.2407
Osmo_Fungi-Spun_5572_OG111
Osmo_Pseudofungi-Hcat_10371_OG111
3.2138
3.4192
Osmo_Fungi-Cang_4844_OG111
Bacteria-UP_b000003688_973_OG111
3.4192
Pp_Diaphoretickes-Npyr_27082_OG111
Osmo_Fungi-Pbla_4921_OG111
2.113
Osmo_Fungi-Rory_5864_OG111
2.2774
2.34
Osmo_Fungi-Mver_6193_OG111
Osmo_Fungi-Crev_1571_OG111
2.3887
Osmo_Fungi-Spun_5805_OG111
2.3139
Osmo_Fungi-Gpro_663_OG111
2.4141
Osmo_Fungi-Amac_15195_OG111
2.0004
Osmo_Fungi-Amac_11121_OG111
2.1234
2.3184
Osmo_Fungi-Cang_4287_OG111
Osmo_Fungi-Spom_355_OG111
Osmo_Fungi-Ccor_5687_OG111
2.4264
1.7909
Osmo_Fungi-Ccor_7509_OG111
1.8931
Osmo_Fungi-Ccor_4129_OG111
1.6083
2.0382
Osmo_Fungi-Ccor_7943_OG111
2.1999
Osmo_Fungi-Ccor_3256_OG111
2.4412
2.3911
Osmo_Fungi-Ccor_7400_OG111
Osmo_Fungi-Ncra_3597_OG111
2.2758
Osmo_Fungi-Nirr_1633_OG111
Osmo_Fungi-Ccin_10357_OG111
2.2603
2.6413
Osmo_Fungi-Umay_658_OG111
2.2867
Osmo_Fungi-Spro_761_OG111

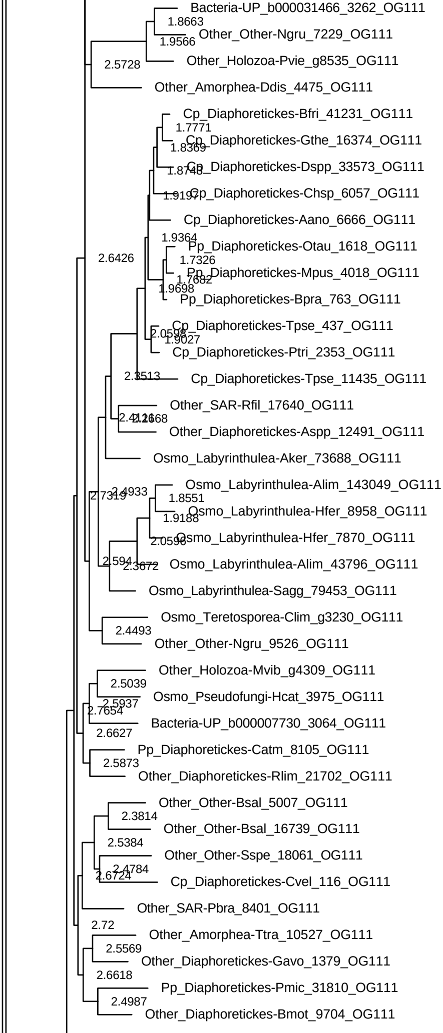
Bacteria-UP_b000031466_3262_OG111
1.8663
Other_Other-Ngru_7229_OG111
1.9566
Other_Holozoa-Pvie_g8535_OG111
2.5728
Other_Amorphea-Ddis_4475_OG111
Cp_Diaphoretickes-Bfri_41231_OG111
1.7771
Cp_Diaphoretickes-Gthe_16374_OG111
1.8369
Cp_Diaphoretickes-Dspp_33573_OG111
1.8748
Cp_Diaphoretickes-Chsp_6057_OG111
1.9197
Cp_Diaphoretickes-Aano_6666_OG111
1.9364
Pp_Diaphoretickes-Otau_1618_OG111
2.6426
1.7326
Pp_Diaphoretickes-Mpus_4018_OG111
1.7682
1.9698
Pp_Diaphoretickes-Bpra_763_OG111
Cp_Diaphoretickes-Tpse_437_OG111
2.0598
1.9027
Cp_Diaphoretickes-Ptri_2353_OG111
2.3513
Cp_Diaphoretickes-Tpse_11435_OG111
Other_SAR-Rfil_17640_OG111
Other_Diaphoretickes-Aspp_12491_OG111
Osmo_Labyrinthulea-Aker_73688_OG111
Osmo_Labyrinthulea-Alim_143049_OG111
2.4933
2.7319
1.8551
Osmo_Labyrinthulea-Hfer_8958_OG111
1.9188
Osmo_Labyrinthulea-Hfer_7870_OG111
2.0596
2.594
2.3672
Osmo_Labyrinthulea-Alim_43796_OG111
Osmo_Labyrinthulea-Sagg_79453_OG111
Osmo_Teretosporea-Clim_g3230_OG111
2.4493
Other_Other-Ngru_9526_OG111
Other_Holozoa-Mvib_g4309_OG111
2.5039
Osmo_Pseudofungi-Hcat_3975_OG111
2.5937
2.7654
Bacteria-UP_b000007730_3064_OG111
2.6627
Pp_Diaphoretickes-Catm_8105_OG111
2.5873
Other_Diaphoretickes-Rlim_21702_OG111
Other_Other-Bsal_5007_OG111
2.3814
Other_Other-Bsal_16739_OG111
2.5384
Other_Other-Sspe_18061_OG111
2.4784
2.6724
Cp_Diaphoretickes-Cvel_116_OG111
Other_SAR-Pbra_8401_OG111
2.72
Other_Amorphea-Ttra_10527_OG111
2.5569
Other_Diaphoretickes-Gavo_1379_OG111
2.6618
Pp_Diaphoretickes-Pmic_31810_OG111
2.4987
Other_Diaphoretickes-Bmot_9704_OG111

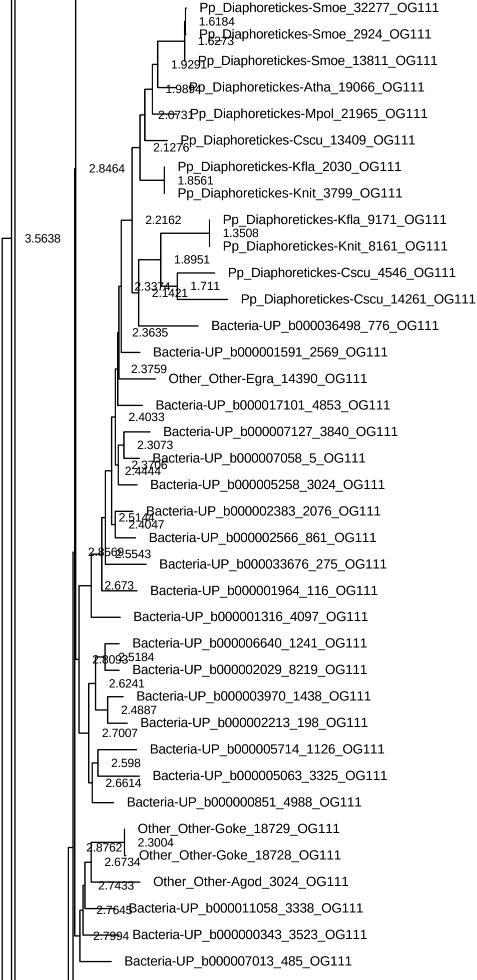
Pp_Diaphoretickes-Smoe_32277_OG111
1.6184
Pp_Diaphoretickes-Smoe_2924_OG111
1.6273
1.9291
Pp_Diaphoretickes-Smoe_13811_OG111
1.9894
Pp_Diaphoretickes-Atha_19066_OG111
2.0731
Pp_Diaphoretickes-Mpol_21965_OG111
2.1276
Pp_Diaphoretickes-Cscu_13409_OG111
2.8464
Pp_Diaphoretickes-Kfla_2030_OG111
1.8561
Pp_Diaphoretickes-Knit_3799_OG111
2.2162
Pp_Diaphoretickes-Kfla_9171_OG111
1.3508
3.5638
Pp_Diaphoretickes-Knit_8161_OG111
1.8951
Pp_Diaphoretickes-Cscu_4546_OG111
2.3374
1.711
2.1421
Pp_Diaphoretickes-Cscu_14261_OG111
Bacteria-UP_b000036498_776_OG111
2.3635
Bacteria-UP_b000001591_2569_OG111
2.3759
Other_Other-Egra_14390_OG111
Bacteria-UP_b000017101_4853_OG111
2.4033
Bacteria-UP_b000007127_3840_OG111
2.3073
Bacteria-UP_b000007058_5_OG111
2.3706
2.4444
Bacteria-UP_b000005258_3024_OG111
Bacteria-UP_b000002383_2076_OG111
2.5144
2.4047
Bacteria-UP_b000002566_861_OG111
2.8569
2.5543
Bacteria-UP_b000033676_275_OG111
2.673
Bacteria-UP_b000001964_116_OG111
Bacteria-UP_b000001316_4097_OG111
Bacteria-UP_b000006640_1241_OG111
2.5184
2.8093
Bacteria-UP_b000002029_8219_OG111
2.6241
Bacteria-UP_b000003970_1438_OG111
2.4887
Bacteria-UP_b000002213_198_OG111
2.7007
Bacteria-UP_b000005714_1126_OG111
2.598
Bacteria-UP_b000005063_3325_OG111
2.6614
Bacteria-UP_b000000851_4988_OG111
Other_Other-Goke_18729_OG111
2.3004
2.8762
Other_Other-Goke_18728_OG111
2.6734
Other_Other-Agod_3024_OG111
2.7433
Bacteria-UP_b000011058_3338_OG111
2.7645
Bacteria-UP_b000000343_3523_OG111
2.7994
Bacteria-UP_b000007013_485_OG111

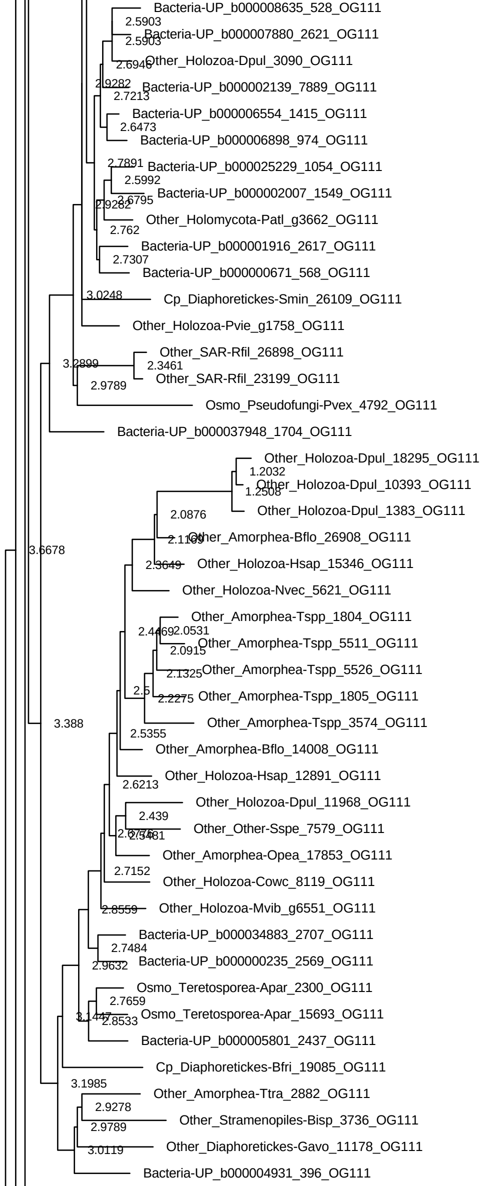

Bacteria-UP_b000008635_528_OG111
2.5903
Bacteria-UP_b000007880_2621_OG111
2.5903
Other_Holozoa-Dpul_3090_OG111
2.6946
2.9282
Bacteria-UP_b000002139_7889_OG111
2.7213
Bacteria-UP_b000006554_1415_OG111
2.6473
Bacteria-UP_b000006898_974_OG111
2.7891
Bacteria-UP_b000025229_1054_OG111
2.5992
Bacteria-UP_b000002007_1549_OG111
2.6795
2.9282
Other_Holomycota-Patl_g3662_OG111
2.762
Bacteria-UP_b000001916_2617_OG111
2.7307
Bacteria-UP_b000000671_568_OG111
3.0248
Cp_Diaphoretickes-Smin_26109_OG111
Other_Holozoa-Pvie_g1758_OG111
Other_SAR-Rfil_26898_OG111
3.2899
2.3461
Other_SAR-Rfil_23199_OG111
2.9789
Osmo_Pseudofungi-Pvex_4792_OG111
Bacteria-UP_b000037948_1704_OG111
Other_Holozoa-Dpul_18295_OG111
1.2032
Other_Holozoa-Dpul_10393_OG111
1.2508
Other_Holozoa-Dpul_1383_OG111
2.0876
Other_Amorphea-Bflo_26908_OG111
2.1169
3.6678
Other_Holozoa-Hsap_15346_OG111
2.3649
Other_Holozoa-Nvec_5621_OG111
Other_Amorphea-Tspp_1804_OG111
2.4469
2.0531
Other_Amorphea-Tspp_5511_OG111
2.0915
Other_Amorphea-Tspp_5526_OG111
2.1325
2.5
Other_Amorphea-Tspp_1805_OG111
2.2275
3.388
Other_Amorphea-Tspp_3574_OG111
2.5355
Other_Amorphea-Bflo_14008_OG111
Other_Holozoa-Hsap_12891_OG111
2.6213
Other_Holozoa-Dpul_11968_OG111
2.439
Other_Other-Sspe_7579_OG111
2.6776
2.5481
Other_Amorphea-Opea_17853_OG111
2.7152
Other_Holozoa-Cowc_8119_OG111
2.8559
Other_Holozoa-Mvib_g6551_OG111
Bacteria-UP_b000034883_2707_OG111
2.7484
2.9632
Bacteria-UP_b000000235_2569_OG111
Osmo_Teretosporea-Apar_2300_OG111
2.7659
3.1447
2.8533
Osmo_Teretosporea-Apar_15693_OG111
Bacteria-UP_b000005801_2437_OG111
Cp_Diaphoretickes-Bfri_19085_OG111
3.1985
Other_Amorphea-Ttra_2882_OG111
2.9278
Other_Stramenopiles-Bisp_3736_OG111
2.9789
Other_Diaphoretickes-Gavo_11178_OG111
3.0119
Bacteria-UP_b000004931_396_OG111

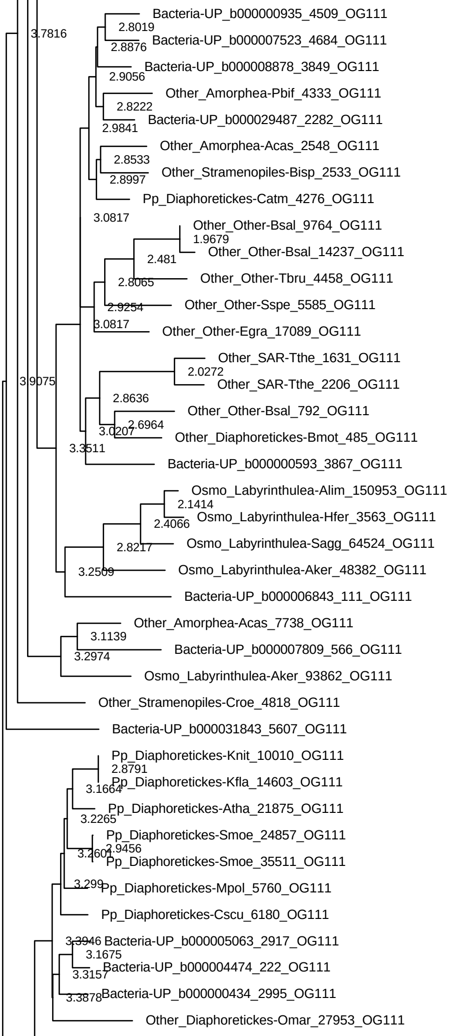
Bacteria-UP_b000000935_4509_OG111
2.8019
Bacteria-UP_b000007523_4684_OG111
2.8876
3.7816
Bacteria-UP_b000008878_3849_OG111
2.9056
Other_Amorphea-Pbif_4333_OG111
2.8222
Bacteria-UP_b000029487_2282_OG111
2.9841
Other_Amorphea-Acas_2548_OG111
2.8533
Other_Stramenopiles-Bisp_2533_OG111
2.8997
Pp_Diaphoretickes-Catm_4276_OG111
3.0817
Other_Other-Bsal_9764_OG111
1.9679
Other_Other-Bsal_14237_OG111
2.481
Other_Other-Tbru_4458_OG111
2.8065
Other_Other-Sspe_5585_OG111
2.9254
3.0817
Other_Other-Egra_17089_OG111
Other_SAR-Tthe_1631_OG111
2.0272
Other_SAR-Tthe_2206_OG111
3.9075
2.8636
Other_Other-Bsal_792_OG111
2.6964
3.0207
Other_Diaphoretickes-Bmot_485_OG111
3.3511
Bacteria-UP_b000000593_3867_OG111
Osmo_Labyrinthulea-Alim_150953_OG111
2.1414
Osmo_Labyrinthulea-Hfer_3563_OG111
2.4066
Osmo_Labyrinthulea-Sagg_64524_OG111
2.8217
3.2509
Osmo_Labyrinthulea-Aker_48382_OG111
Bacteria-UP_b000006843_111_OG111
Other_Amorphea-Acas_7738_OG111
3.1139
Bacteria-UP_b000007809_566_OG111
3.2974
Osmo_Labyrinthulea-Aker_93862_OG111
Other_Stramenopiles-Croe_4818_OG111
Bacteria-UP_b000031843_5607_OG111
Pp_Diaphoretickes-Knit_10010_OG111
2.8791
Pp_Diaphoretickes-Kfla_14603_OG111
3.1664
Pp_Diaphoretickes-Atha_21875_OG111
3.2265
Pp_Diaphoretickes-Smoe_24857_OG111
2.9456
3.2601
Pp_Diaphoretickes-Smoe_35511_OG111
3.299
Pp_Diaphoretickes-Mpol_5760_OG111
Pp_Diaphoretickes-Cscu_6180_OG111
3.3946
Bacteria-UP_b000005063_2917_OG111
3.1675
Bacteria-UP_b000004474_222_OG111
3.3157
Bacteria-UP_b000000434_2995_OG111
3.3878
Other_Diaphoretickes-Omar_27953_OG111

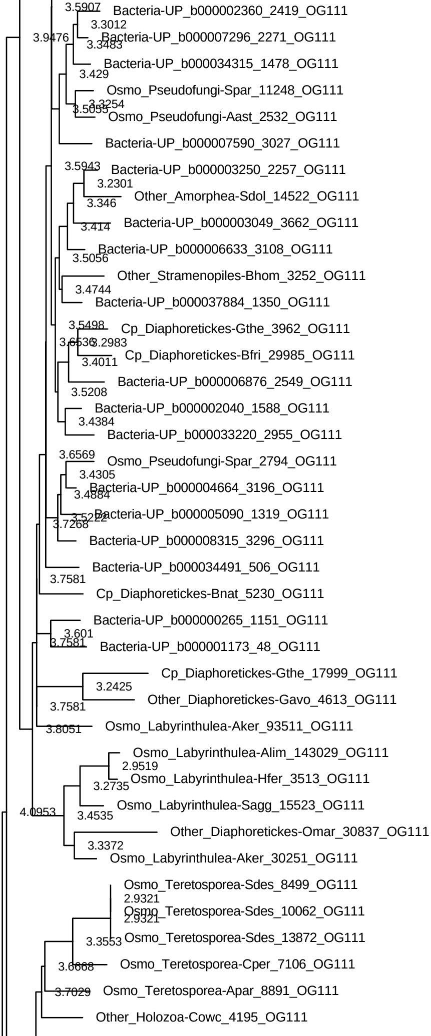

3.5907
Bacteria-UP_b000002360_2419_OG111
3.3012
3.9476
Bacteria-UP_b000007296_2271_OG111
3.3483
Bacteria-UP_b000034315_1478_OG111
3.429
Osmo_Pseudofungi-Spar_11248_OG111
3.3254
3.5055
Osmo_Pseudofungi-Aast_2532_OG111
Bacteria-UP_b000007590_3027_OG111
3.5943
Bacteria-UP_b000003250_2257_OG111
3.2301
Other_Amorphea-Sdol_14522_OG111
3.346
Bacteria-UP_b000003049_3662_OG111
3.414
Bacteria-UP_b000006633_3108_OG111
3.5056
Other_Stramenopiles-Bhom_3252_OG111
3.4744
Bacteria-UP_b000037884_1350_OG111
3.5498
Cp_Diaphoretickes-Gthe_3962_OG111
3.6536
3.2983
Cp_Diaphoretickes-Bfri_29985_OG111
3.4011
Bacteria-UP_b000006876_2549_OG111
3.5208
Bacteria-UP_b000002040_1588_OG111
3.4384
Bacteria-UP_b000033220_2955_OG111
3.6569
Osmo_Pseudofungi-Spar_2794_OG111
3.4305
Bacteria-UP_b000004664_3196_OG111
3.4884
3.5222
Bacteria-UP_b000005090_1319_OG111
3.7268
Bacteria-UP_b000008315_3296_OG111
Bacteria-UP_b000034491_506_OG111
3.7581
Cp_Diaphoretickes-Bnat_5230_OG111
Bacteria-UP_b000000265_1151_OG111
3.601
3.7581
Bacteria-UP_b000001173_48_OG111
Cp_Diaphoretickes-Gthe_17999_OG111
3.2425
Other_Diaphoretickes-Gavo_4613_OG111
3.7581
3.8051
Osmo_Labyrinthulea-Aker_93511_OG111
Osmo_Labyrinthulea-Alim_143029_OG111
2.9519
Osmo_Labyrinthulea-Hfer_3513_OG111
3.2735
Osmo_Labyrinthulea-Sagg_15523_OG111
4.0953
3.4535
Other_Diaphoretickes-Omar_30837_OG111
3.3372
Osmo_Labyrinthulea-Aker_30251_OG111
Osmo_Teretosporea-Sdes_8499_OG111
2.9321
Osmo_Teretosporea-Sdes_10062_OG111
2.9321
Osmo_Teretosporea-Sdes_13872_OG111
3.3553
Osmo_Teretosporea-Cper_7106_OG111
3.6668
3.7029
Osmo_Teretosporea-Apar_8891_OG111
Other_Holozoa-Cowc_4195_OG111

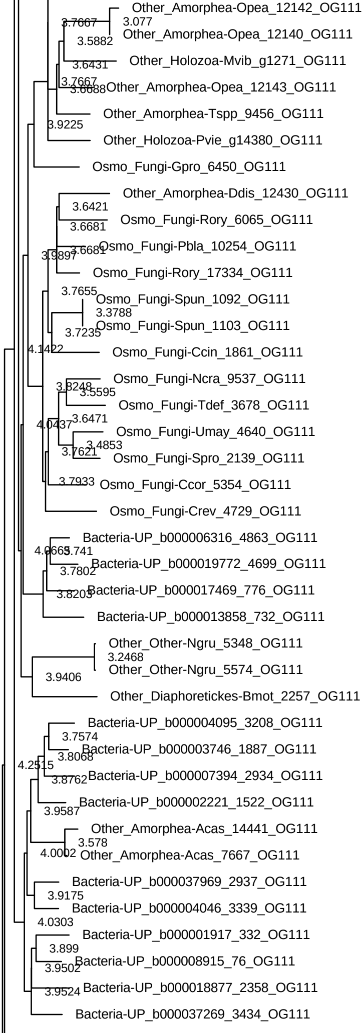

Other_Amorphea-Opea_12142_OG111
3.7667
3.077
3.5882
Other_Amorphea-Opea_12140_OG111
3.6431
Other_Holozoa-Mvib_g1271_OG111
3.7667
3.6688
Other_Amorphea-Opea_12143_OG111
Other_Amorphea-Tspp_9456_OG111
3.9225
Other_Holozoa-Pvie_g14380_OG111
Osmo_Fungi-Gpro_6450_OG111
Other_Amorphea-Ddis_12430_OG111
3.6421
Osmo_Fungi-Rory_6065_OG111
3.6681
3.9897
3.6681
Osmo_Fungi-Pbla_10254_OG111
Osmo_Fungi-Rory_17334_OG111
3.7655
Osmo_Fungi-Spun_1092_OG111
3.3788
3.7235
Osmo_Fungi-Spun_1103_OG111
4.1422
Osmo_Fungi-Ccin_1861_OG111
Osmo_Fungi-Ncra_9537_OG111
3.8248
3.5595
Osmo_Fungi-Tdef_3678_OG111
4.0437
3.6471
Osmo_Fungi-Umay_4640_OG111
3.4853
3.7621
Osmo_Fungi-Spro_2139_OG111
3.7933
Osmo_Fungi-Ccor_5354_OG111
Osmo_Fungi-Crev_4729_OG111
Bacteria-UP_b000006316_4863_OG111
4.0665
3.741
Bacteria-UP_b000019772_4699_OG111
3.7802
3.8203
Bacteria-UP_b000017469_776_OG111
Bacteria-UP_b000013858_732_OG111
Other_Other-Ngru_5348_OG111
3.2468
Other_Other-Ngru_5574_OG111
3.9406
Other_Diaphoretickes-Bmot_2257_OG111
Bacteria-UP_b000004095_3208_OG111
3.7574
Bacteria-UP_b000003746_1887_OG111
3.8068
4.2515
3.8762
Bacteria-UP_b000007394_2934_OG111
3.9587
Bacteria-UP_b000002221_1522_OG111
Other_Amorphea-Acas_14441_OG111
3.578
4.0002
Other_Amorphea-Acas_7667_OG111
Bacteria-UP_b000037969_2937_OG111
3.9175
Bacteria-UP_b000004046_3339_OG111
4.0303
Bacteria-UP_b000001917_332_OG111
3.899
Bacteria-UP_b000008915_76_OG111
3.9502
3.9524
Bacteria-UP_b000018877_2358_OG111
Bacteria-UP_b000037269_3434_OG111

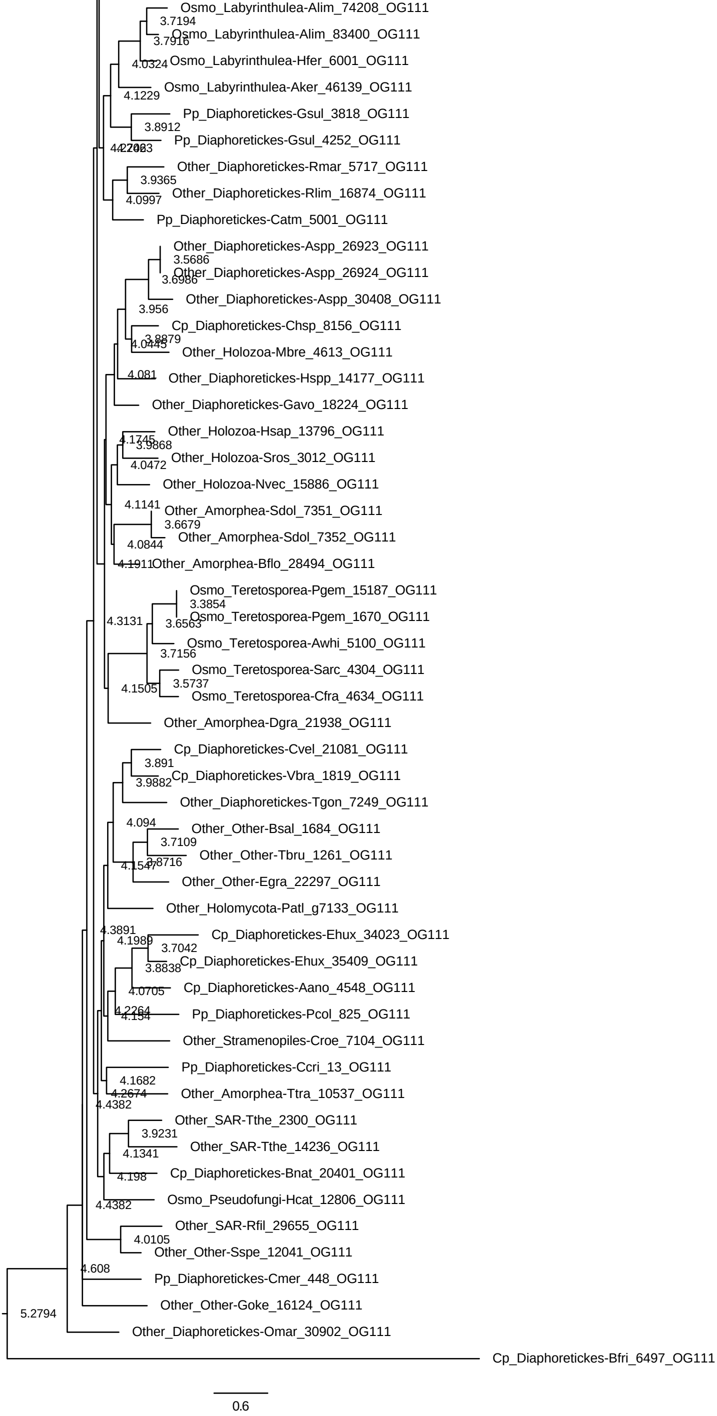
Osmo_Labyrinthulea-Alim_74208_OG111
3.7194
Osmo_Labyrinthulea-Alim_83400_OG111
3.7916
Osmo_Labyrinthulea-Hfer_6001_OG111
4.0324
Osmo_Labyrinthulea-Aker_46139_OG111
4.1229
Pp_Diaphoretickes-Gsul_3818_OG111
3.8912
Pp_Diaphoretickes-Gsul_4252_OG111
Other_Diaphoretickes-Rmar_5717_OG111
3.9365
Other_Diaphoretickes-Rlim_16874_OG111
4.0997
Pp_Diaphoretickes-Catm_5001_OG111
Other_Diaphoretickes-Aspp_26923_OG111
3.5686
Other_Diaphoretickes-Aspp_26924_OG111
3.6986
Other_Diaphoretickes-Aspp_30408_OG111
3.956
Cp_Diaphoretickes-Chsp_8156_OG111
3.8879
4.0445
Other_Holozoa-Mbre_4613_OG111
4.081
Other_Diaphoretickes-Hspp_14177_OG111
Other_Diaphoretickes-Gavo_18224_OG111
Other_Holozoa-Hsap_13796_OG111
4.1745
3.9868
Other_Holozoa-Sros_3012_OG111
4.0472
Other_Holozoa-Nvec_15886_OG111
4.1141
Other_Amorphea-Sdol_7351_OG111
3.6679
Other_Amorphea-Sdol_7352_OG111
4.0844
4.1911
Other_Amorphea-Bflo_28494_OG111
Osmo_Teretosporea-Pgem_15187_OG111
3.3854
4.3131
Osmo_Teretosporea-Pgem_1670_OG111
3.6563
Osmo_Teretosporea-Awhi_5100_OG111
3.7156
Osmo_Teretosporea-Sarc_4304_OG111
3.5737
4.1505
Osmo_Teretosporea-Cfra_4634_OG111
Other_Amorphea-Dgra_21938_OG111
Cp_Diaphoretickes-Cvel_21081_OG111
3.891
Cp_Diaphoretickes-Vbra_1819_OG111
3.9882
Other_Diaphoretickes-Tgon_7249_OG111
4.094
Other_Other-Bsal_1684_OG111
3.7109
Other_Other-Tbru_1261_OG111
4.1547
3.8716
Other_Other-Egra_22297_OG111
Other_Holomycota-Patl_g7133_OG111
4.3891
Cp_Diaphoretickes-Ehux_34023_OG111
4.1989
3.7042
Cp_Diaphoretickes-Ehux_35409_OG111
3.8838
Cp_Diaphoretickes-Aano_4548_OG111
4.0705
4.2264
4.154
Pp_Diaphoretickes-Pcol_825_OG111
Other_Stramenopiles-Croe_7104_OG111
Pp_Diaphoretickes-Ccri_13_OG111
4.1682
4.2674
Other_Amorphea-Ttra_10537_OG111
4.4382
Other_SAR-Tthe_2300_OG111
3.9231
Other_SAR-Tthe_14236_OG111
4.1341
Cp_Diaphoretickes-Bnat_20401_OG111
4.198
Osmo_Pseudofungi-Hcat_12806_OG111
4.4382
Other_SAR-Rfil_29655_OG111
4.0105
Other_Other-Sspe_12041_OG111
4.608
Pp_Diaphoretickes-Cmer_448_OG111
Other_Other-Goke_16124_OG111
5.2794
Other_Diaphoretickes-Omar_30902_OG111
Cp_Diaphoretickes-Bfri_6497_OG111
0.6

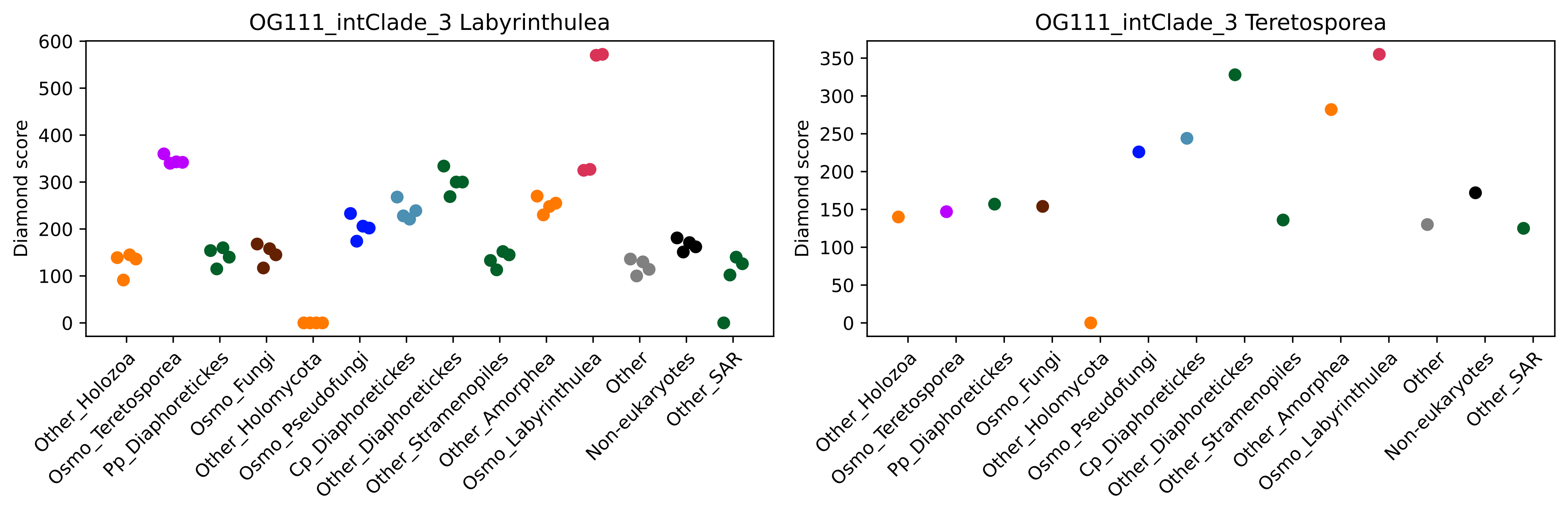
OG111_intClade_3 Labyrinthulea
OG111_intClade_3 Teretosporea
Diamond score
Other_Holozoa
Osmo_Teretosporea
Pp_Diaphoretickes
Osmo_Fungi
Other_Holomycota
Osmo_Pseudofungi
Cp_Diaphoretickes
Other_Diaphoretickes
Other_Stramenopiles
Other_Amorphea
Osmo_Labyrinthulea
Other
Non-eukaryotes
Other_SAR

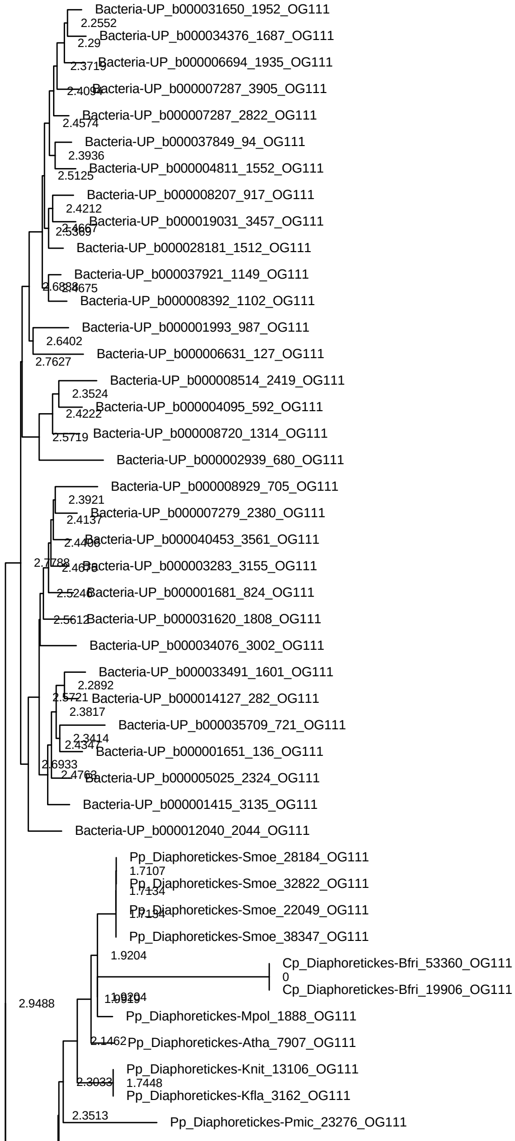

Bacteria-UP_b000031650_1952_OG111
2.2552
Bacteria-UP_b000034376_1687_OG111
2.29
Bacteria-UP_b000006694_1935_OG111
Bacteria-UP_b000007287_3905_OG111
Bacteria-UP_b000007287_2822_OG111
2.4574
Bacteria-UP_b000037849_94_OG111
2.3936
Bacteria-UP_b000004811_1552_OG111
2.5125
Bacteria-UP_b000008207_917_OG111
2.4212
Bacteria-UP_b000019031_3457_OG111
Bacteria-UP_b000028181_1512_OG111
Bacteria-UP_b000037921_1149_OG111
Bacteria-UP_b000008392_1102_OG111
Bacteria-UP_b000001993_987_OG111
2.6402
Bacteria-UP_b000006631_127_OG111
2.7627
Bacteria-UP_b000008514_2419_OG111
2.3524
Bacteria-UP_b000004095_592_OG111
2.4222
Bacteria-UP_b000008720_1314_OG111
2.5719
Bacteria-UP_b000002939_680_OG111
Bacteria-UP_b000008929_705_OG111
2.3921
Bacteria-UP_b000007279_2380_OG111
2.4137
Bacteria-UP_b000040453_3561_OG111
Bacteria-UP_b000003283_3155_OG111
Bacteria-UP_b000001681_824_OG111
Bacteria-UP_b000031620_1808_OG111
Bacteria-UP_b000034076_3002_OG111
Bacteria-UP_b000033491_1601_OG111
2.2892
Bacteria-UP_b000014127_282_OG111
2.3817
Bacteria-UP_b000035709_721_OG111
Bacteria-UP_b000001651_136_OG111
Bacteria-UP_b000005025_2324_OG111
Bacteria-UP_b000001415_3135_OG111
Bacteria-UP_b000012040_2044_OG111
Pp_Diaphoretickes-Smoe_28184_OG111
1.7107
Pp_Diaphoretickes-Smoe_32822_OG111
Pp_Diaphoretickes-Smoe_22049_OG111
Pp_Diaphoretickes-Smoe_38347_OG111
1.9204
Cp_Diaphoretickes-Bfri_53360_OG111
0
Cp_Diaphoretickes-Bfri_19906_OG111
2.9488
Pp_Diaphoretickes-Mpol_1888_OG111
2.1462
Pp_Diaphoretickes-Atha_7907_OG111
Pp_Diaphoretickes-Knit_13106_OG111
1.7448
Pp_Diaphoretickes-Kfla_3162_OG111
2.3513
Pp_Diaphoretickes-Pmic_23276_OG111

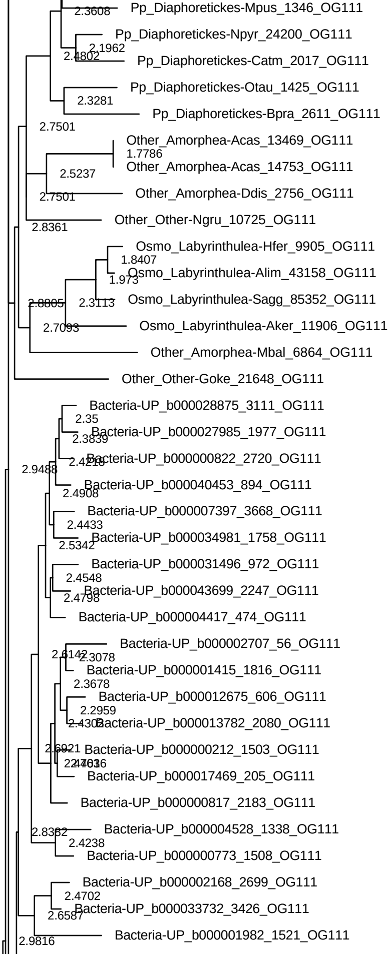
Pp_Diaphoretickes-Mpus_1346_OG111
2.3608
Pp_Diaphoretickes-Npyr_24200_OG111
2.1962
2.4802
Pp_Diaphoretickes-Catm_2017_OG111
Pp_Diaphoretickes-Otau_1425_OG111
2.3281
Pp_Diaphoretickes-Bpra_2611_OG111
2.7501
Other_Amorphea-Acas_13469_OG111
1.7786
Other_Amorphea-Acas_14753_OG111
2.5237
Other_Amorphea-Ddis_2756_OG111
2.7501
Other_Other-Ngru_10725_OG111
2.8361
Osmo_Labyrinthulea-Hfer_9905_OG111
1.8407
Osmo_Labyrinthulea-Alim_43158_OG111
1.973
2.8805
2.3113
Osmo_Labyrinthulea-Sagg_85352_OG111
2.7093
Osmo_Labyrinthulea-Aker_11906_OG111
Other_Amorphea-Mbal_6864_OG111
Other_Other-Goke_21648_OG111
Bacteria-UP_b000028875_3111_OG111
2.35
Bacteria-UP_b000027985_1977_OG111
2.3839
2.4218
Bacteria-UP_b000000822_2720_OG111
2.9488
Bacteria-UP_b000040453_894_OG111
2.4908
Bacteria-UP_b000007397_3668_OG111
2.4433
Bacteria-UP_b000034981_1758_OG111
2.5342
Bacteria-UP_b000031496_972_OG111
2.4548
Bacteria-UP_b000043699_2247_OG111
2.4798
Bacteria-UP_b000004417_474_OG111
Bacteria-UP_b000002707_56_OG111
2.6142
2.3078
Bacteria-UP_b000001415_1816_OG111
2.3678
Bacteria-UP_b000012675_606_OG111
2.2959
2.4302
Bacteria-UP_b000013782_2080_OG111
2.6921
Bacteria-UP_b000000212_1503_OG111
Bacteria-UP_b000017469_205_OG111
Bacteria-UP_b000000817_2183_OG111
2.8382
Bacteria-UP_b000004528_1338_OG111
2.4238
Bacteria-UP_b000000773_1508_OG111
Bacteria-UP_b000002168_2699_OG111
2.4702
Bacteria-UP_b000033732_3426_OG111
2.6587
Bacteria-UP_b000001982_1521_OG111
2.9816

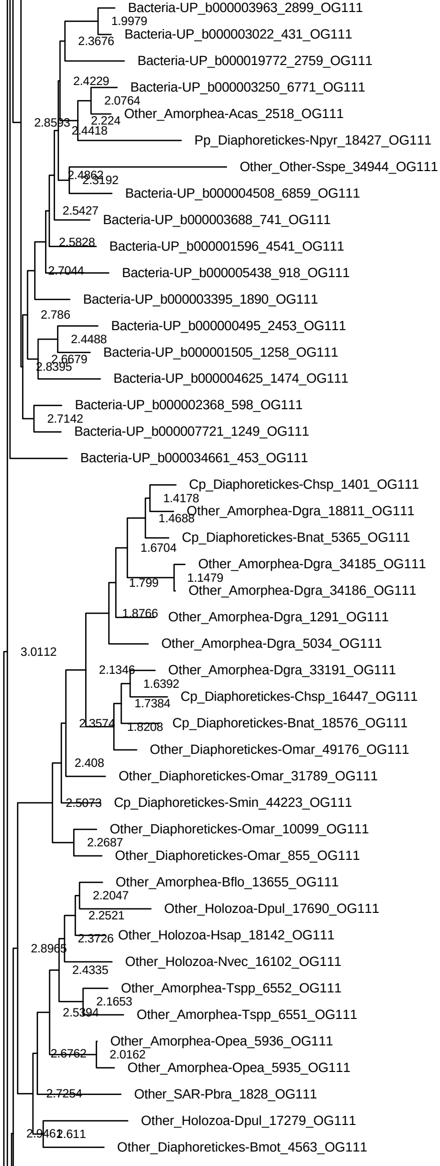
Bacteria-UP_b000003963_2899_OG111
1.9979
Bacteria-UP_b000003022_431_OG111
2.3676
Bacteria-UP_b000019772_2759_OG111
2.4229
Bacteria-UP_b000003250_6771_OG111
2.0764
Other_Amorphea-Acas_2518_OG111
2.224
2.8593
2.4418
Pp_Diaphoretickes-Npyr_18427_OG111
Other_Other-Sspe_34944_OG111
2.4862
2.3192
Bacteria-UP_b000004508_6859_OG111
2.5427
Bacteria-UP_b000003688_741_OG111
2.5828
Bacteria-UP_b000001596_4541_OG111
2.7044
Bacteria-UP_b000005438_918_OG111
Bacteria-UP_b000003395_1890_OG111
2.786
Bacteria-UP_b000000495_2453_OG111
2.4488
Bacteria-UP_b000001505_1258_OG111
2.6679
2.8395
Bacteria-UP_b000004625_1474_OG111
Bacteria-UP_b000002368_598_OG111
2.7142
Bacteria-UP_b000007721_1249_OG111
Bacteria-UP_b000034661_453_OG111
Cp_Diaphoretickes-Chsp_1401_OG111
1.4178
Other_Amorphea-Dgra_18811_OG111
1.4688
Cp_Diaphoretickes-Bnat_5365_OG111
1.6704
Other_Amorphea-Dgra_34185_OG111
1.1479
1.799
Other_Amorphea-Dgra_34186_OG111
1.8766
Other_Amorphea-Dgra_1291_OG111
Other_Amorphea-Dgra_5034_OG111
3.0112
2.1346
Other_Amorphea-Dgra_33191_OG111
1.6392
Cp_Diaphoretickes-Chsp_16447_OG111
1.7384
2.3574
1.8208
Cp_Diaphoretickes-Bnat_18576_OG111
Other_Diaphoretickes-Omar_49176_OG111
2.408
Other_Diaphoretickes-Omar_31789_OG111
2.5073
Cp_Diaphoretickes-Smin_44223_OG111
Other_Diaphoretickes-Omar_10099_OG111
2.2687
Other_Diaphoretickes-Omar_855_OG111
Other_Amorphea-Bflo_13655_OG111
2.2047
Other_Holozoa-Dpul_17690_OG111
2.2521
Other_Holozoa-Hsap_18142_OG111
2.3726
2.8965
Other_Holozoa-Nvec_16102_OG111
2.4335
Other_Amorphea-Tspp_6552_OG111
2.1653
2.5394
Other_Amorphea-Tspp_6551_OG111
Other_Amorphea-Opea_5936_OG111
2.6762
2.0162
Other_Amorphea-Opea_5935_OG111
2.7254
Other_SAR-Pbra_1828_OG111
Other_Holozoa-Dpul_17279_OG111
2.9462
2.611
Other_Diaphoretickes-Bmot_4563_OG111

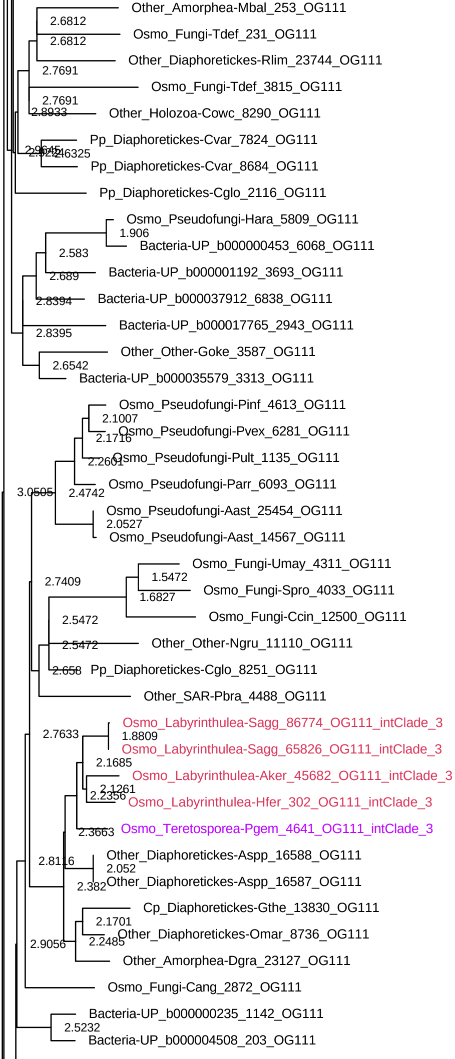

Other_Amorphea-Mbal_253_OG111
2.6812
Osmo_Fungi-Tdef_231_OG111
2.6812
Other_Diaphoretickes-Rlim_23744_OG111
2.7691
Osmo_Fungi-Tdef_3815_OG111
2.7691
2.8933
Other_Holozoa-Cowc_8290_OG111
Pp_Diaphoretickes-Cvar_7824_OG111
Pp_Diaphoretickes-Cvar_8684_OG111
Pp_Diaphoretickes-Cglo_2116_OG111
Osmo_Pseudofungi-Hara_5809_OG111
1.906
Bacteria-UP_b000000453_6068_OG111
2.583
Bacteria-UP_b000001192_3693_OG111
2.689
Bacteria-UP_b000037912_6838_OG111
2.8394
Bacteria-UP_b000017765_2943_OG111
2.8395
Other_Other-Goke_3587_OG111
2.6542
Bacteria-UP_b000035579_3313_OG111
Osmo_Pseudofungi-Pinf_4613_OG111
2.1007
Osmo_Pseudofungi-Pvex_6281_OG111
2.1716
Osmo_Pseudofungi-Pult_1135_OG111
2.2601
Osmo_Pseudofungi-Parr_6093_OG111
3.0505
2.4742
Osmo_Pseudofungi-Aast_25454_OG111
2.0527
Osmo_Pseudofungi-Aast_14567_OG111
Osmo_Fungi-Umay_4311_OG111
2.7409
1.5472
Osmo_Fungi-Spro_4033_OG111
1.6827
Osmo_Fungi-Ccin_12500_OG111
2.5472
Other_Other-Ngru_11110_OG111
2.5472
2.658
Pp_Diaphoretickes-Cglo_8251_OG111
Other_SAR-Pbra_4488_OG111
Osmo_Labyrinthulea-Sagg_86774_OG111_intClade_3
2.7633
1.8809
Osmo_Labyrinthulea-Sagg_65826_OG111_intClade_3
2.1685
Osmo_Labyrinthulea-Aker_45682_OG111_intClade_3
2.1261
2.2356
Osmo_Labyrinthulea-Hfer_302_OG111_intClade_3
2.3663
Osmo_Teretosporea-Pgem_4641_OG111_intClade_3
Other_Diaphoretickes-Aspp_16588_OG111
2.8116
2.052
2.382
Other_Diaphoretickes-Aspp_16587_OG111
Cp_Diaphoretickes-Gthe_13830_OG111
2.1701
Other_Diaphoretickes-Omar_8736_OG111
2.9056
2.2485
Other_Amorphea-Dgra_23127_OG111
Osmo_Fungi-Cang_2872_OG111
Bacteria-UP_b000000235_1142_OG111
2.5232
Bacteria-UP_b000004508_203_OG111

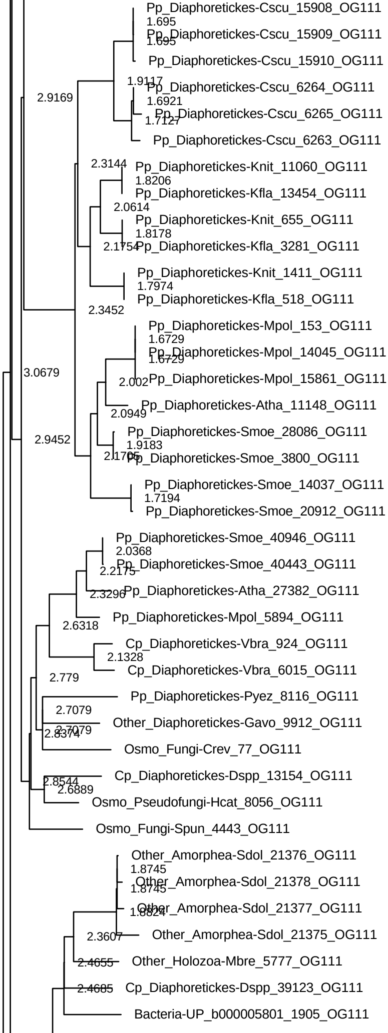
Pp_Diaphoretickes-Cscu_15908_OG111
1.695
Pp_Diaphoretickes-Cscu_15909_OG111
1.695
Pp_Diaphoretickes-Cscu_15910_OG111
1.9117
Pp_Diaphoretickes-Cscu_6264_OG111
2.9169
1.6921
Pp_Diaphoretickes-Cscu_6265_OG111
1.7127
Pp_Diaphoretickes-Cscu_6263_OG111
2.3144
Pp_Diaphoretickes-Knit_11060_OG111
1.8206
Pp_Diaphoretickes-Kfla_13454_OG111
2.0614
Pp_Diaphoretickes-Knit_655_OG111
1.8178
2.1754
Pp_Diaphoretickes-Kfla_3281_OG111
Pp_Diaphoretickes-Knit_1411_OG111
1.7974
Pp_Diaphoretickes-Kfla_518_OG111
2.3452
Pp_Diaphoretickes-Mpol_153_OG111
1.6729
Pp_Diaphoretickes-Mpol_14045_OG111
1.6729
3.0679
2.002
Pp_Diaphoretickes-Mpol_15861_OG111
Pp_Diaphoretickes-Atha_11148_OG111
2.0949
Pp_Diaphoretickes-Smoe_28086_OG111
2.9452
1.9183
2.1705
Pp_Diaphoretickes-Smoe_3800_OG111
Pp_Diaphoretickes-Smoe_14037_OG111
1.7194
Pp_Diaphoretickes-Smoe_20912_OG111
Pp_Diaphoretickes-Smoe_40946_OG111
2.0368
Pp_Diaphoretickes-Smoe_40443_OG111
2.2175
Pp_Diaphoretickes-Atha_27382_OG111
2.3296
Pp_Diaphoretickes-Mpol_5894_OG111
2.6318
Cp_Diaphoretickes-Vbra_924_OG111
2.1328
Cp_Diaphoretickes-Vbra_6015_OG111
2.779
Pp_Diaphoretickes-Pyez_8116_OG111
2.7079
Other_Diaphoretickes-Gavo_9912_OG111
2.7079
2.8374
Osmo_Fungi-Crev_77_OG111
Cp_Diaphoretickes-Dspp_13154_OG111
2.8544
2.6889
Osmo_Pseudofungi-Hcat_8056_OG111
Osmo_Fungi-Spun_4443_OG111
Other_Amorphea-Sdol_21376_OG111
1.8745
Other_Amorphea-Sdol_21378_OG111
1.8745
Other_Amorphea-Sdol_21377_OG111
1.8824
2.3607
Other_Amorphea-Sdol_21375_OG111
2.4655
Other_Holozoa-Mbre_5777_OG111
2.4685
Cp_Diaphoretickes-Dspp_39123_OG111
Bacteria-UP_b000005801_1905_OG111

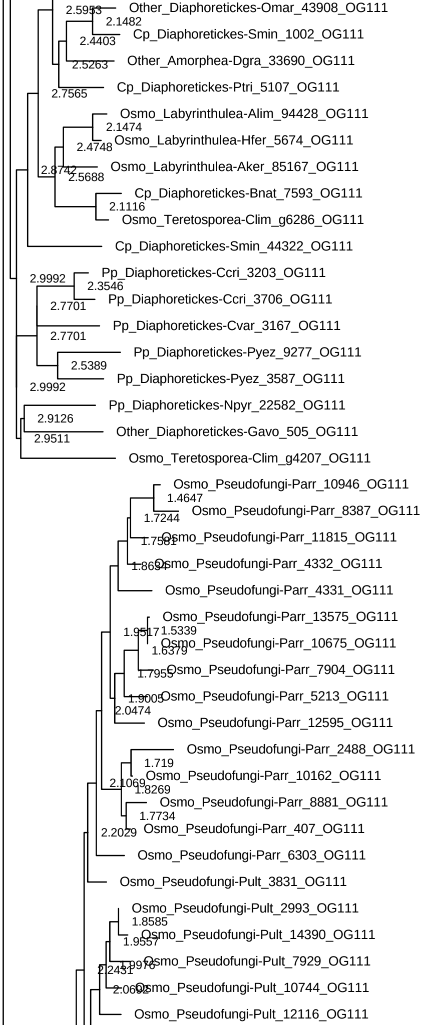
Other_Diaphoretickes-Omar_43908_OG111
2.5953
2.1482
Cp_Diaphoretickes-Smin_1002_OG111
2.4403
Other_Amorphea-Dgra_33690_OG111
2.5263
Cp_Diaphoretickes-Ptri_5107_OG111
2.7565
Osmo_Labyrinthulea-Alim_94428_OG111
2.1474
Osmo_Labyrinthulea-Hfer_5674_OG111
2.4748
Osmo_Labyrinthulea-Aker_85167_OG111
2.8742
2.5688
Cp_Diaphoretickes-Bnat_7593_OG111
2.1116
Osmo_Teretosporea-Clim_g6286_OG111
Cp_Diaphoretickes-Smin_44322_OG111
Pp_Diaphoretickes-Ccri_3203_OG111
2.9992
2.3546
Pp_Diaphoretickes-Ccri_3706_OG111
2.7701
Pp_Diaphoretickes-Cvar_3167_OG111
2.7701
Pp_Diaphoretickes-Pyez_9277_OG111
2.5389
Pp_Diaphoretickes-Pyez_3587_OG111
2.9992
Pp_Diaphoretickes-Npyr_22582_OG111
2.9126
Other_Diaphoretickes-Gavo_505_OG111
2.9511
Osmo_Teretosporea-Clim_g4207_OG111
Osmo_Pseudofungi-Parr_10946_OG111
1.4647
Osmo_Pseudofungi-Parr_8387_OG111
1.7244
Osmo_Pseudofungi-Parr_11815_OG111
1.7581
Osmo_Pseudofungi-Parr_4332_OG111
1.8634
Osmo_Pseudofungi-Parr_4331_OG111
Osmo_Pseudofungi-Parr_13575_OG111
1.9517
1.5339
Osmo_Pseudofungi-Parr_10675_OG111
1.6379
Osmo_Pseudofungi-Parr_7904_OG111
1.7955
Osmo_Pseudofungi-Parr_5213_OG111
1.9005
2.0474
Osmo_Pseudofungi-Parr_12595_OG111
Osmo_Pseudofungi-Parr_2488_OG111
1.719
Osmo_Pseudofungi-Parr_10162_OG111
2.1069
1.8269
Osmo_Pseudofungi-Parr_8881_OG111
1.7734
Osmo_Pseudofungi-Parr_407_OG111
2.2029
Osmo_Pseudofungi-Parr_6303_OG111
Osmo_Pseudofungi-Pult_3831_OG111
Osmo_Pseudofungi-Pult_2993_OG111
1.8585
Osmo_Pseudofungi-Pult_14390_OG111
1.9557
Osmo_Pseudofungi-Pult_7929_OG111
1.9976
2.2431
Osmo_Pseudofungi-Pult_10744_OG111
2.0692
Osmo_Pseudofungi-Pult_12116_OG111

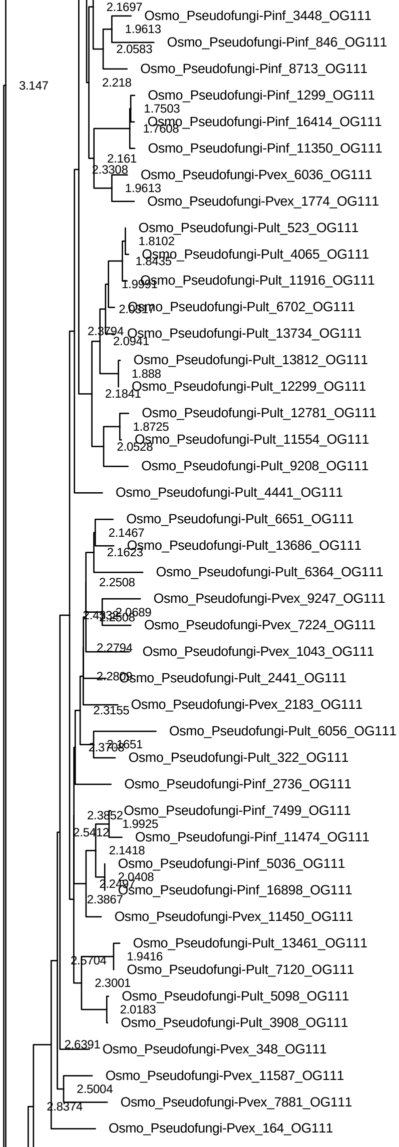

2.1697
Osmo_Pseudofungi-Pinf_3448_OG111
1.9613
Osmo_Pseudofungi-Pinf_846_OG111
2.0583
Osmo_Pseudofungi-Pinf_8713_OG111
3.147
2.218
Osmo_Pseudofungi-Pinf_1299_OG111
1.7503
Osmo_Pseudofungi-Pinf_16414_OG111
1.7608
Osmo_Pseudofungi-Pinf_11350_OG111
2.161
2.3308
Osmo_Pseudofungi-Pvex_6036_OG111
1.9613
Osmo_Pseudofungi-Pvex_1774_OG111
Osmo_Pseudofungi-Pult_523_OG111
1.8102
Osmo_Pseudofungi-Pult_4065_OG111
1.8435
1.9991
Osmo_Pseudofungi-Pult_11916_OG111
2.0317
Osmo_Pseudofungi-Pult_6702_OG111
2.3794
Osmo_Pseudofungi-Pult_13734_OG111
2.0941
Osmo_Pseudofungi-Pult_13812_OG111
1.888
2.1841
Osmo_Pseudofungi-Pult_12299_OG111
Osmo_Pseudofungi-Pult_12781_OG111
1.8725
2.0528
Osmo_Pseudofungi-Pult_11554_OG111
Osmo_Pseudofungi-Pult_9208_OG111
Osmo_Pseudofungi-Pult_4441_OG111
Osmo_Pseudofungi-Pult_6651_OG111
2.1467
2.1623
Osmo_Pseudofungi-Pult_13686_OG111
Osmo_Pseudofungi-Pult_6364_OG111
2.2508
Osmo_Pseudofungi-Pvex_9247_OG111
Osmo_Pseudofungi-Pvex_7224_OG111
2.2794
Osmo_Pseudofungi-Pvex_1043_OG111
2.2809
Osmo_Pseudofungi-Pult_2441_OG111
2.3155
Osmo_Pseudofungi-Pvex_2183_OG111
Osmo_Pseudofungi-Pult_6056_OG111
2.1651
2.3708
Osmo_Pseudofungi-Pult_322_OG111
Osmo_Pseudofungi-Pinf_2736_OG111
2.3852
Osmo_Pseudofungi-Pinf_7499_OG111
1.9925
2.5412
Osmo_Pseudofungi-Pinf_11474_OG111
2.1418
Osmo_Pseudofungi-Pinf_5036_OG111
2.0408
2.2497
Osmo_Pseudofungi-Pinf_16898_OG111
2.3867
Osmo_Pseudofungi-Pvex_11450_OG111
Osmo_Pseudofungi-Pult_13461_OG111
2.5704
1.9416
Osmo_Pseudofungi-Pult_7120_OG111
2.3001
Osmo_Pseudofungi-Pult_5098_OG111
2.0183
Osmo_Pseudofungi-Pult_3908_OG111
2.6391
Osmo_Pseudofungi-Pvex_348_OG111
Osmo_Pseudofungi-Pvex_11587_OG111
2.5004
2.8374
Osmo_Pseudofungi-Pvex_7881_OG111
Osmo_Pseudofungi-Pvex_164_OG111

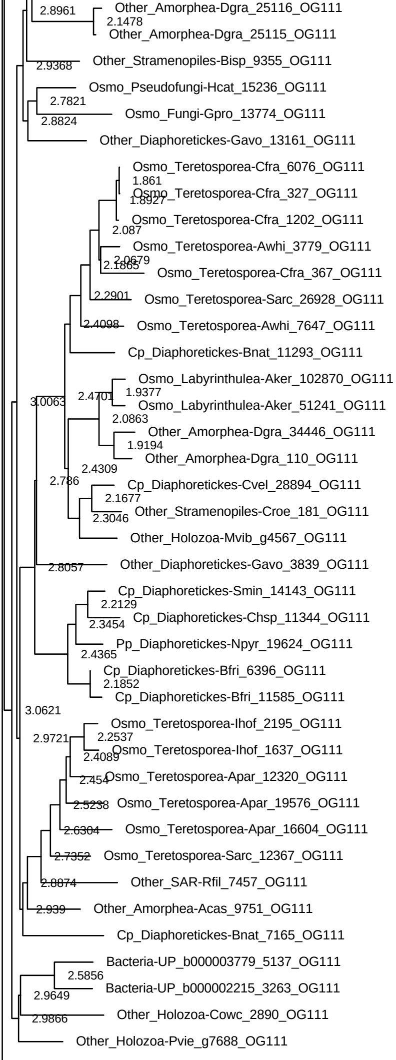
2.8961
Other_Amorphea-Dgra_25116_OG111
2.1478
Other_Amorphea-Dgra_25115_OG111
2.9368
Other_Stramenopiles-Bisp_9355_OG111
Osmo_Pseudofungi-Hcat_15236_OG111
2.7821
Osmo_Fungi-Gpro_13774_OG111
2.8824
Other_Diaphoretickes-Gavo_13161_OG111
Osmo_Teretosporea-Cfra_6076_OG111
1.861
Osmo_Teretosporea-Cfra_327_OG111
1.8927
Osmo_Teretosporea-Cfra_1202_OG111
2.087
Osmo_Teretosporea-Awhi_3779_OG111
2.0679
2.1865
Osmo_Teretosporea-Cfra_367_OG111
2.2901
Osmo_Teretosporea-Sarc_26928_OG111
2.4098
Osmo_Teretosporea-Awhi_7647_OG111
Cp_Diaphoretickes-Bnat_11293_OG111
Osmo_Labyrinthulea-Aker_102870_OG111
1.9377
3.0063
2.4701
Osmo_Labyrinthulea-Aker_51241_OG111
2.0863
Other_Amorphea-Dgra_34446_OG111
1.9194
Other_Amorphea-Dgra_110_OG111
2.4309
2.786
Cp_Diaphoretickes-Cvel_28894_OG111
2.1677
Other_Stramenopiles-Croe_181_OG111
2.3046
Other_Holozoa-Mvib_g4567_OG111
2.8057
Other_Diaphoretickes-Gavo_3839_OG111
Cp_Diaphoretickes-Smin_14143_OG111
2.2129
Cp_Diaphoretickes-Chsp_11344_OG111
2.3454
Pp_Diaphoretickes-Npyr_19624_OG111
2.4365
Cp_Diaphoretickes-Bfri_6396_OG111
2.1852
Cp_Diaphoretickes-Bfri_11585_OG111
3.0621
Osmo_Teretosporea-Ihof_2195_OG111
2.9721
2.2537
Osmo_Teretosporea-Ihof_1637_OG111
2.4089
2.454
Osmo_Teretosporea-Apar_12320_OG111
2.5238
Osmo_Teretosporea-Apar_19576_OG111
2.6304
Osmo_Teretosporea-Apar_16604_OG111
2.7352
Osmo_Teretosporea-Sarc_12367_OG111
2.8874
Other_SAR-Rfil_7457_OG111
2.939
Other_Amorphea-Acas_9751_OG111
Cp_Diaphoretickes-Bnat_7165_OG111
Bacteria-UP_b000003779_5137_OG111
2.5856
Bacteria-UP_b000002215_3263_OG111
2.9649
2.9866
Other_Holozoa-Cowc_2890_OG111
Other_Holozoa-Pvie_g7688_OG111

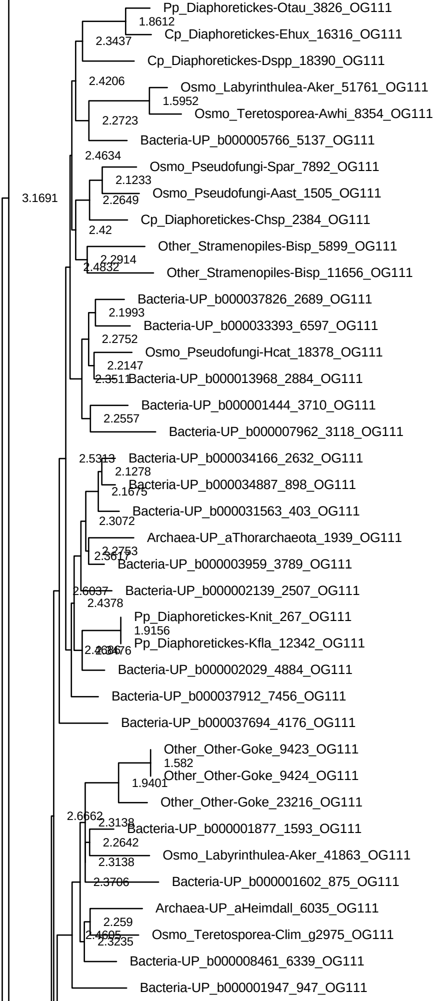
Pp_Diaphoretickes-Otau_3826_OG111
1.8612
Cp_Diaphoretickes-Ehux_16316_OG111
2.3437
Cp_Diaphoretickes-Dspp_18390_OG111
2.4206
Osmo_Labyrinthulea-Aker_51761_OG111
1.5952
Osmo_Teretosporea-Awhi_8354_OG111
2.2723
Bacteria-UP_b000005766_5137_OG111
2.4634
Osmo_Pseudofungi-Spar_7892_OG111
2.1233
Osmo_Pseudofungi-Aast_1505_OG111
2.2649
3.1691
Cp_Diaphoretickes-Chsp_2384_OG111
2.42
Other_Stramenopiles-Bisp_5899_OG111
2.2914
2.4832
Other_Stramenopiles-Bisp_11656_OG111
Bacteria-UP_b000037826_2689_OG111
2.1993
Bacteria-UP_b000033393_6597_OG111
2.2752
Osmo_Pseudofungi-Hcat_18378_OG111
2.2147
2.3511
Bacteria-UP_b000013968_2884_OG111
Bacteria-UP_b000001444_3710_OG111
2.2557
Bacteria-UP_b000007962_3118_OG111
2.5313
Bacteria-UP_b000034166_2632_OG111
2.1278
Bacteria-UP_b000034887_898_OG111
2.1675
Bacteria-UP_b000031563_403_OG111
2.3072
Archaea-UP_aThorarchaeota_1939_OG111
2.2753
2.3617
Bacteria-UP_b000003959_3789_OG111
2.6037
Bacteria-UP_b000002139_2507_OG111
2.4378
Pp_Diaphoretickes-Knit_267_OG111
1.9156
Pp_Diaphoretickes-Kfla_12342_OG111
Bacteria-UP_b000002029_4884_OG111
Bacteria-UP_b000037912_7456_OG111
Bacteria-UP_b000037694_4176_OG111
Other_Other-Goke_9423_OG111
1.582
Other_Other-Goke_9424_OG111
1.9401
Other_Other-Goke_23216_OG111
2.6662
2.3138
Bacteria-UP_b000001877_1593_OG111
2.2642
Osmo_Labyrinthulea-Aker_41863_OG111
2.3138
2.3706
Bacteria-UP_b000001602_875_OG111
Archaea-UP_aHeimdall_6035_OG111
2.259
2.4605
2.3235
Osmo_Teretosporea-Clim_g2975_OG111
Bacteria-UP_b000008461_6339_OG111
Bacteria-UP_b000001947_947_OG111

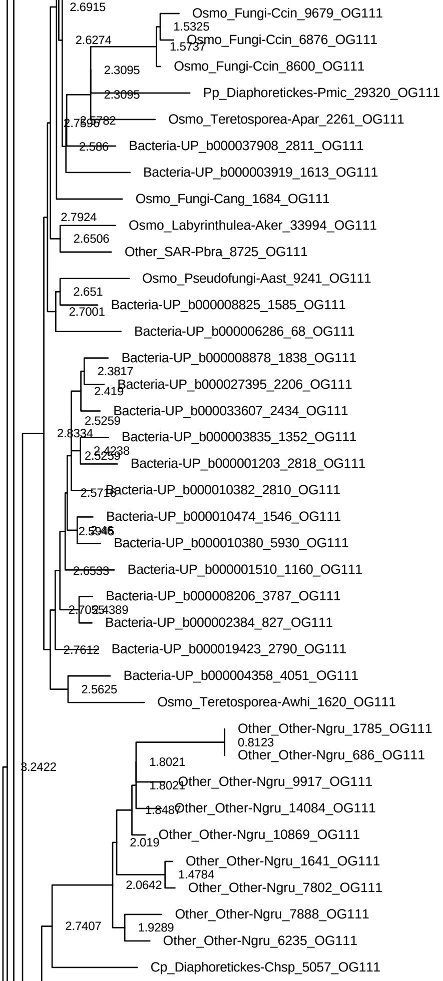
2.6915
Osmo_Fungi-Ccin_9679_OG111
1.5325
Osmo_Fungi-Ccin_6876_OG111
2.6274
1.5737
Osmo_Fungi-Ccin_8600_OG111
2.3095
Pp_Diaphoretickes-Pmic_29320_OG111
2.3095
Osmo_Teretosporea-Apar_2261_OG111
2.5782
Bacteria-UP_b000037908_2811_OG111
2.586
Bacteria-UP_b000003919_1613_OG111
Osmo_Fungi-Cang_1684_OG111
2.7924
Osmo_Labyrinthulea-Aker_33994_OG111
2.6506
Other_SAR-Pbra_8725_OG111
Osmo_Pseudofungi-Aast_9241_OG111
2.651
Bacteria-UP_b000008825_1585_OG111
2.7001
Bacteria-UP_b000006286_68_OG111
Bacteria-UP_b000008878_1838_OG111
2.3817
Bacteria-UP_b000027395_2206_OG111
2.419
Bacteria-UP_b000033607_2434_OG111
2.5259
2.8334
Bacteria-UP_b000003835_1352_OG111
2.4238
2.5259
Bacteria-UP_b000001203_2818_OG111
Bacteria-UP_b000010382_2810_OG111
Bacteria-UP_b000010474_1546_OG111
Bacteria-UP_b000010380_5930_OG111
2.6533
Bacteria-UP_b000001510_1160_OG111
Bacteria-UP_b000008206_3787_OG111
Bacteria-UP_b000002384_827_OG111
2.7612
Bacteria-UP_b000019423_2790_OG111
Bacteria-UP_b000004358_4051_OG111
2.5625
Osmo_Teretosporea-Awhi_1620_OG111
Other_Other-Ngru_1785_OG111
0.8123
Other_Other-Ngru_686_OG111
1.8021
3.2422
Other_Other-Ngru_9917_OG111
1.8021
Other_Other-Ngru_14084_OG111
1.8487
Other_Other-Ngru_10869_OG111
2.019
Other_Other-Ngru_1641_OG111
1.4784
2.0642
Other_Other-Ngru_7802_OG111
Other_Other-Ngru_7888_OG111
2.7407
1.9289
Other_Other-Ngru_6235_OG111
Cp_Diaphoretickes-Chsp_5057_OG111

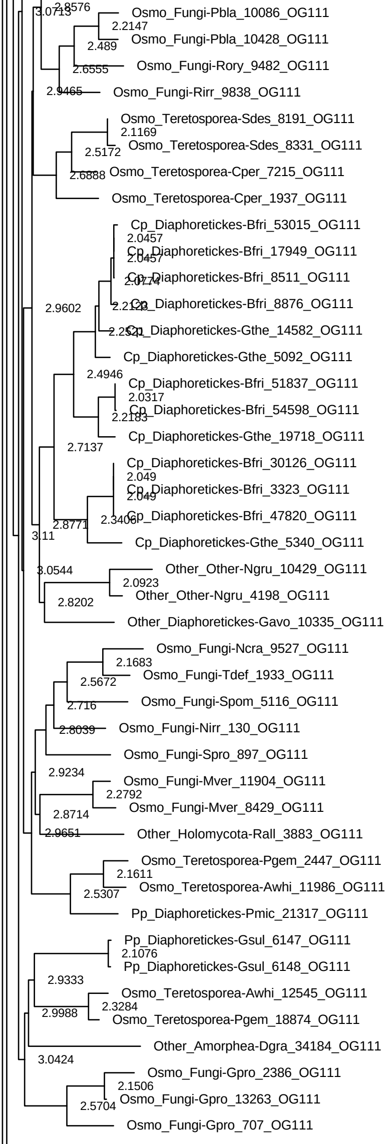
3.0713
2.8576
Osmo_Fungi-Pbla_10086_OG111
2.2147
Osmo_Fungi-Pbla_10428_OG111
2.489
Osmo_Fungi-Rory_9482_OG111
2.6555
2.9465
Osmo_Fungi-Rirr_9838_OG111
Osmo_Teretosporea-Sdes_8191_OG111
2.1169
Osmo_Teretosporea-Sdes_8331_OG111
2.5172
Osmo_Teretosporea-Cper_7215_OG111
2.6888
Osmo_Teretosporea-Cper_1937_OG111
Cp_Diaphoretickes-Bfri_53015_OG111
2.0457
Cp_Diaphoretickes-Bfri_17949_OG111
2.0457
Cp_Diaphoretickes-Bfri_8511_OG111
2.0774
Cp_Diaphoretickes-Bfri_8876_OG111
2.2123
2.9602
Cp_Diaphoretickes-Gthe_14582_OG111
2.2521
Cp_Diaphoretickes-Gthe_5092_OG111
2.4946
Cp_Diaphoretickes-Bfri_51837_OG111
2.0317
Cp_Diaphoretickes-Bfri_54598_OG111
2.2183
Cp_Diaphoretickes-Gthe_19718_OG111
2.7137
Cp_Diaphoretickes-Bfri_30126_OG111
2.049
Cp_Diaphoretickes-Bfri_3323_OG111
2.049
Cp_Diaphoretickes-Bfri_47820_OG111
2.3406
2.8771
3.11
Cp_Diaphoretickes-Gthe_5340_OG111
3.0544
Other_Other-Ngru_10429_OG111
2.0923
Other_Other-Ngru_4198_OG111
2.8202
Other_Diaphoretickes-Gavo_10335_OG111
Osmo_Fungi-Ncra_9527_OG111
2.1683
Osmo_Fungi-Tdef_1933_OG111
2.5672
Osmo_Fungi-Spom_5116_OG111
2.716
Osmo_Fungi-Nirr_130_OG111
2.8039
Osmo_Fungi-Spro_897_OG111
2.9234
Osmo_Fungi-Mver_11904_OG111
2.2792
Osmo_Fungi-Mver_8429_OG111
2.8714
2.9651
Other_Holomycota-Rall_3883_OG111
Osmo_Teretosporea-Pgem_2447_OG111
2.1611
Osmo_Teretosporea-Awhi_11986_OG111
2.5307
Pp_Diaphoretickes-Pmic_21317_OG111
Pp_Diaphoretickes-Gsul_6147_OG111
2.1076
Pp_Diaphoretickes-Gsul_6148_OG111
2.9333
Osmo_Teretosporea-Awhi_12545_OG111
2.3284
2.9988
Osmo_Teretosporea-Pgem_18874_OG111
Other_Amorphea-Dgra_34184_OG111
3.0424
Osmo_Fungi-Gpro_2386_OG111
2.1506
Osmo_Fungi-Gpro_13263_OG111
2.5704
Osmo_Fungi-Gpro_707_OG111

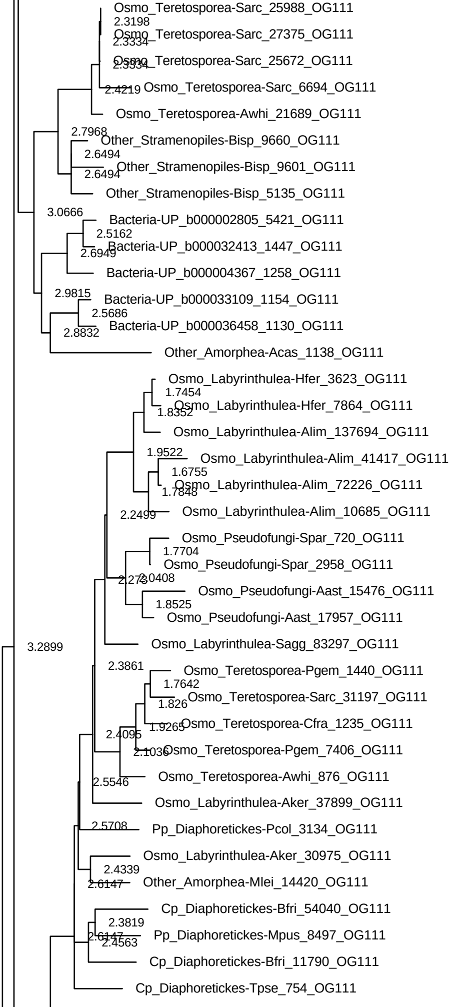

Osmo_Teretosporea-Sarc_25988_OG111
2.3198
Osmo_Teretosporea-Sarc_27375_OG111
2.3334
Osmo_Teretosporea-Sarc_25672_OG111
2.3334
2.4219
Osmo_Teretosporea-Sarc_6694_OG111
Osmo_Teretosporea-Awhi_21689_OG111
2.7968
Other_Stramenopiles-Bisp_9660_OG111
2.6494
Other_Stramenopiles-Bisp_9601_OG111
2.6494
Other_Stramenopiles-Bisp_5135_OG111
3.0666
Bacteria-UP_b000002805_5421_OG111
2.5162
Bacteria-UP_b000032413_1447_OG111
2.6949
Bacteria-UP_b000004367_1258_OG111
2.9815
Bacteria-UP_b000033109_1154_OG111
2.5686
Bacteria-UP_b000036458_1130_OG111
2.8832
Other_Amorphea-Acas_1138_OG111
Osmo_Labyrinthulea-Hfer_3623_OG111
1.7454
Osmo_Labyrinthulea-Hfer_7864_OG111
1.8352
Osmo_Labyrinthulea-Alim_137694_OG111
1.9522
Osmo_Labyrinthulea-Alim_41417_OG111
1.6755
Osmo_Labyrinthulea-Alim_72226_OG111
1.7848
2.2499
Osmo_Labyrinthulea-Alim_10685_OG111
Osmo_Pseudofungi-Spar_720_OG111
1.7704
Osmo_Pseudofungi-Spar_2958_OG111
2.273
2.0408
Osmo_Pseudofungi-Aast_15476_OG111
1.8525
Osmo_Pseudofungi-Aast_17957_OG111
3.2899
Osmo_Labyrinthulea-Sagg_83297_OG111
2.3861
Osmo_Teretosporea-Pgem_1440_OG111
1.7642
Osmo_Teretosporea-Sarc_31197_OG111
1.826
Osmo_Teretosporea-Cfra_1235_OG111
1.9265
2.4095
Osmo_Teretosporea-Pgem_7406_OG111
2.1036
Osmo_Teretosporea-Awhi_876_OG111
2.5546
Osmo_Labyrinthulea-Aker_37899_OG111
2.5708
Pp_Diaphoretickes-Pcol_3134_OG111
Osmo_Labyrinthulea-Aker_30975_OG111
2.4339
2.6147
Other_Amorphea-Mlei_14420_OG111
Cp_Diaphoretickes-Bfri_54040_OG111
2.3819
2.6147
Pp_Diaphoretickes-Mpus_8497_OG111
2.4563
Cp_Diaphoretickes-Bfri_11790_OG111
Cp_Diaphoretickes-Tpse_754_OG111

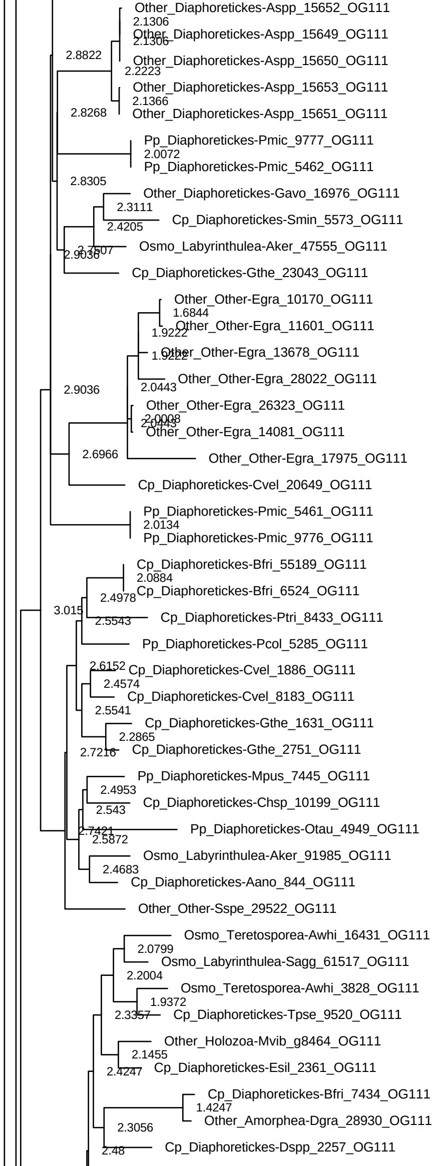

Other_Diaphoretickes-Aspp_15652_OG111
2.1306
Other_Diaphoretickes-Aspp_15649_OG111
2.1306
2.8822
Other_Diaphoretickes-Aspp_15650_OG111
2.2223
Other_Diaphoretickes-Aspp_15653_OG111
2.1366
2.8268
Other_Diaphoretickes-Aspp_15651_OG111
Pp_Diaphoretickes-Pmic_9777_OG111
2.0072
Pp_Diaphoretickes-Pmic_5462_OG111
2.8305
Other_Diaphoretickes-Gavo_16976_OG111
2.3111
Cp_Diaphoretickes-Smin_5573_OG111
2.4205
2.7507
2.9036
Osmo_Labyrinthulea-Aker_47555_OG111
Cp_Diaphoretickes-Gthe_23043_OG111
Other_Other-Egra_10170_OG111
1.6844
Other_Other-Egra_11601_OG111
1.9222
Other_Other-Egra_13678_OG111
1.9222
Other_Other-Egra_28022_OG111
2.0443
2.9036
Other_Other-Egra_26323_OG111
2.0008
2.0443
Other_Other-Egra_14081_OG111
2.6966
Other_Other-Egra_17975_OG111
Cp_Diaphoretickes-Cvel_20649_OG111
Pp_Diaphoretickes-Pmic_5461_OG111
2.0134
Pp_Diaphoretickes-Pmic_9776_OG111
Cp_Diaphoretickes-Bfri_55189_OG111
2.0884
2.4978
Cp_Diaphoretickes-Bfri_6524_OG111
3.015
Cp_Diaphoretickes-Ptri_8433_OG111
2.5543
Pp_Diaphoretickes-Pcol_5285_OG111
2.6152
Cp_Diaphoretickes-Cvel_1886_OG111
2.4574
Cp_Diaphoretickes-Cvel_8183_OG111
2.5541
Cp_Diaphoretickes-Gthe_1631_OG111
2.2865
2.7216
Cp_Diaphoretickes-Gthe_2751_OG111
Pp_Diaphoretickes-Mpus_7445_OG111
2.4953
Cp_Diaphoretickes-Chsp_10199_OG111
2.543
2.7421
Pp_Diaphoretickes-Otau_4949_OG111
2.5872
Osmo_Labyrinthulea-Aker_91985_OG111
2.4683
Cp_Diaphoretickes-Aano_844_OG111
Other_Other-Sspe_29522_OG111
Osmo_Teretosporea-Awhi_16431_OG111
2.0799
Osmo_Labyrinthulea-Sagg_61517_OG111
2.2004
Osmo_Teretosporea-Awhi_3828_OG111
1.9372
2.3357
Cp_Diaphoretickes-Tpse_9520_OG111
Other_Holozoa-Mvib_g8464_OG111
2.1455
2.4247
Cp_Diaphoretickes-Esil_2361_OG111
Cp_Diaphoretickes-Bfri_7434_OG111
1.4247
Other_Amorphea-Dgra_28930_OG111
2.3056
Cp_Diaphoretickes-Dspp_2257_OG111
2.48

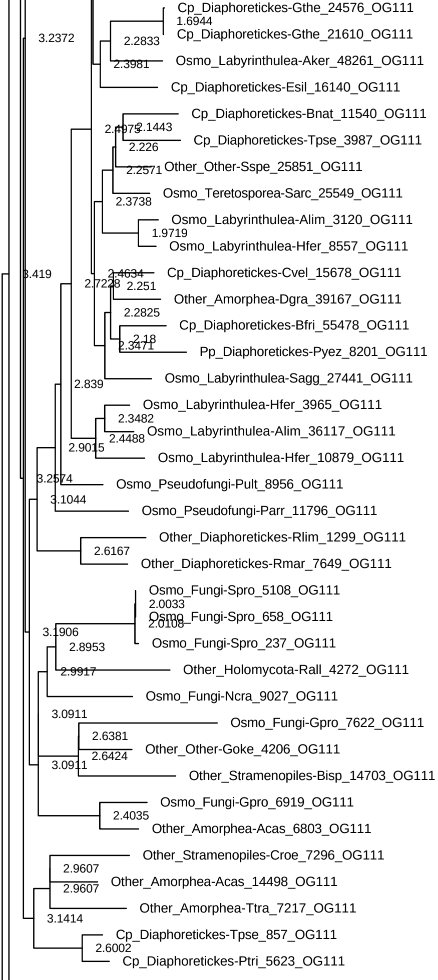

Cp_Diaphoretickes-Gthe_24576_OG111
1.6944
Cp_Diaphoretickes-Gthe_21610_OG111
3.2372
2.2833
Osmo_Labyrinthulea-Aker_48261_OG111
2.3981
Cp_Diaphoretickes-Esil_16140_OG111
Cp_Diaphoretickes-Bnat_11540_OG111
2.4975
2.1443
Cp_Diaphoretickes-Tpse_3987_OG111
2.226
Other_Other-Sspe_25851_OG111
2.2571
Osmo_Teretosporea-Sarc_25549_OG111
2.3738
Osmo_Labyrinthulea-Alim_3120_OG111
1.9719
Osmo_Labyrinthulea-Hfer_8557_OG111
3.419
2.4634
Cp_Diaphoretickes-Cvel_15678_OG111
2.7228
2.251
Other_Amorphea-Dgra_39167_OG111
2.2825
Cp_Diaphoretickes-Bfri_55478_OG111
2.18
2.3471
Pp_Diaphoretickes-Pyez_8201_OG111
2.839
Osmo_Labyrinthulea-Sagg_27441_OG111
Osmo_Labyrinthulea-Hfer_3965_OG111
2.3482
Osmo_Labyrinthulea-Alim_36117_OG111
2.4488
2.9015
Osmo_Labyrinthulea-Hfer_10879_OG111
3.2574
Osmo_Pseudofungi-Pult_8956_OG111
3.1044
Osmo_Pseudofungi-Parr_11796_OG111
Other_Diaphoretickes-Rlim_1299_OG111
2.6167
Other_Diaphoretickes-Rmar_7649_OG111
Osmo_Fungi-Spro_5108_OG111
2.0033
Osmo_Fungi-Spro_658_OG111
2.0108
3.1906
2.8953
Osmo_Fungi-Spro_237_OG111
2.9917
Other_Holomycota-Rall_4272_OG111
Osmo_Fungi-Ncra_9027_OG111
3.0911
Osmo_Fungi-Gpro_7622_OG111
2.6381
Other_Other-Goke_4206_OG111
2.6424
3.0911
Other_Stramenopiles-Bisp_14703_OG111
Osmo_Fungi-Gpro_6919_OG111
2.4035
Other_Amorphea-Acas_6803_OG111
Other_Stramenopiles-Croe_7296_OG111
2.9607
Other_Amorphea-Acas_14498_OG111
2.9607
Other_Amorphea-Ttra_7217_OG111
3.1414
Cp_Diaphoretickes-Tpse_857_OG111
2.6002
Cp_Diaphoretickes-Ptri_5623_OG111

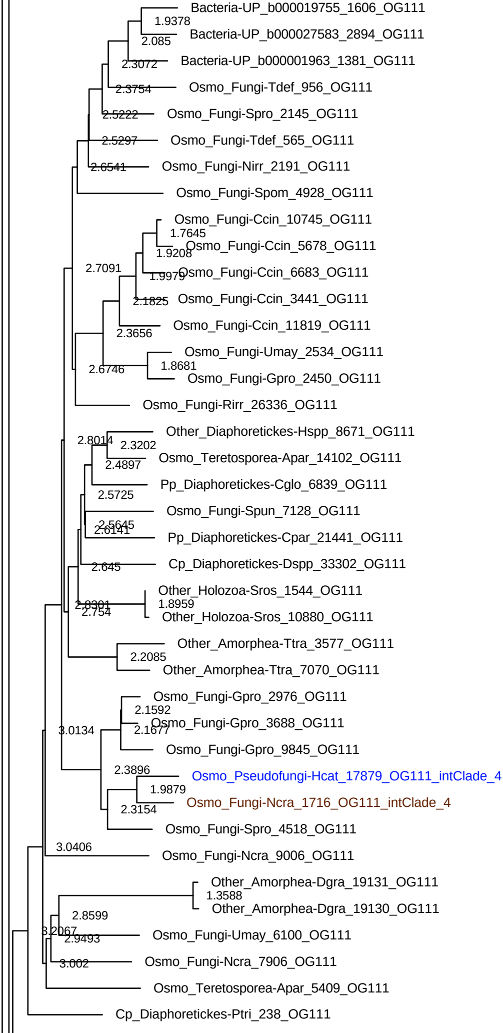
Bacteria-UP_b000019755_1606_OG111
1.9378
Bacteria-UP_b000027583_2894_OG111
2.085
Bacteria-UP_b000001963_1381_OG111
2.3072
Osmo_Fungi-Tdef_956_OG111
2.3754
Osmo_Fungi-Spro_2145_OG111
2.5222
Osmo_Fungi-Tdef_565_OG111
2.5297
Osmo_Fungi-Nirr_2191_OG111
2.6541
Osmo_Fungi-Spom_4928_OG111
Osmo_Fungi-Ccin_10745_OG111
1.7645
Osmo_Fungi-Ccin_5678_OG111
1.9208
2.7091
Osmo_Fungi-Ccin_6683_OG111
1.9979
Osmo_Fungi-Ccin_3441_OG111
2.1825
Osmo_Fungi-Ccin_11819_OG111
2.3656
Osmo_Fungi-Umay_2534_OG111
1.8681
2.6746
Osmo_Fungi-Gpro_2450_OG111
Osmo_Fungi-Rirr_26336_OG111
Other_Diaphoretickes-Hspp_8671_OG111
2.8014
2.3202
Osmo_Teretosporea-Apar_14102_OG111
2.4897
Pp_Diaphoretickes-Cglo_6839_OG111
2.5725
Osmo_Fungi-Spun_7128_OG111
2.5645
2.6141
Pp_Diaphoretickes-Cpar_21441_OG111
Cp_Diaphoretickes-Dspp_33302_OG111
2.645
Other_Holozoa-Sros_1544_OG111
2.8301
1.8959
2.754
Other_Holozoa-Sros_10880_OG111
Other_Amorphea-Ttra_3577_OG111
2.2085
Other_Amorphea-Ttra_7070_OG111
Osmo_Fungi-Gpro_2976_OG111
2.1592
Osmo_Fungi-Gpro_3688_OG111
3.0134
2.1677
Osmo_Fungi-Gpro_9845_OG111
2.3896
Osmo_Pseudofungi-Hcat_17879_OG111_intClade_4
1.9879
Osmo_Fungi-Ncra_1716_OG111_intClade_4
2.3154
Osmo_Fungi-Spro_4518_OG111
3.0406
Osmo_Fungi-Ncra_9006_OG111
Other_Amorphea-Dgra_19131_OG111
1.3588
Other_Amorphea-Dgra_19130_OG111
2.8599
3.2067
Osmo_Fungi-Umay_6100_OG111
2.9493
Osmo_Fungi-Ncra_7906_OG111
3.002
Osmo_Teretosporea-Apar_5409_OG111
Cp_Diaphoretickes-Ptri_238_OG111

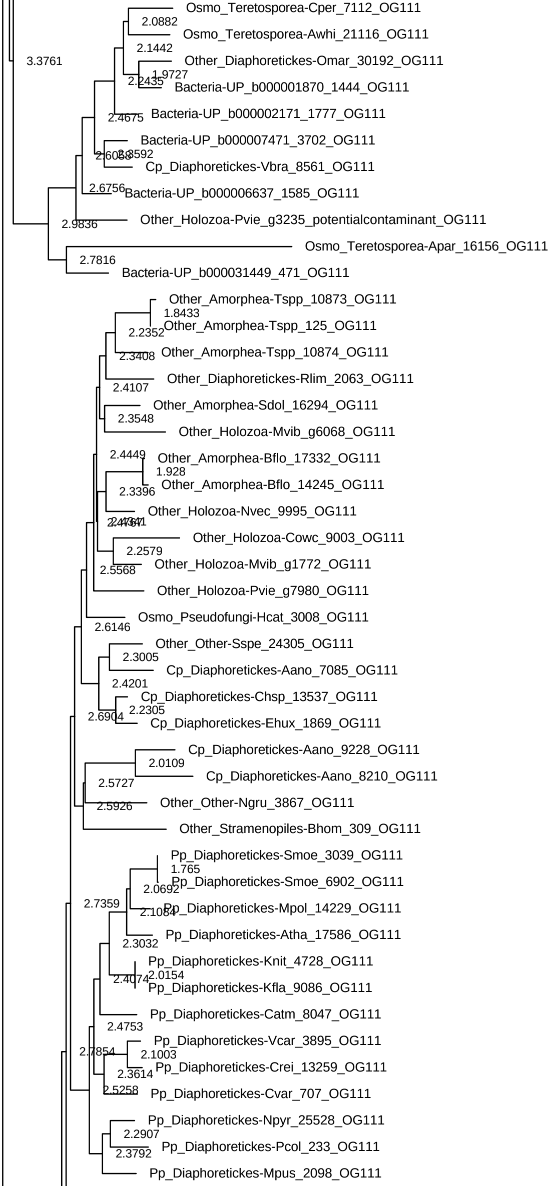
Osmo_Teretosporea-Cper_7112_OG111
2.0882
Osmo_Teretosporea-Awhi_21116_OG111
2.1442
3.3761
Other_Diaphoretickes-Omar_30192_OG111
1.9727
2.2435
Bacteria-UP_b000001870_1444_OG111
2.4675
Bacteria-UP_b000002171_1777_OG111
Bacteria-UP_b000007471_3702_OG111
Cp_Diaphoretickes-Vbra_8561_OG111
2.6756
Bacteria-UP_b000006637_1585_OG111
2.9836
Other_Holozoa-Pvie_g3235_potentialcontaminant_OG111
Osmo_Teretosporea-Apar_16156_OG111
2.7816
Bacteria-UP_b000031449_471_OG111
Other_Amorphea-Tspp_10873_OG111
1.8433
2.2352
Other_Amorphea-Tspp_125_OG111
2.3408
Other_Amorphea-Tspp_10874_OG111
Other_Diaphoretickes-Rlim_2063_OG111
2.4107
Other_Amorphea-Sdol_16294_OG111
2.3548
Other_Holozoa-Mvib_g6068_OG111
2.4449
Other_Amorphea-Bflo_17332_OG111
1.928
2.3396
Other_Amorphea-Bflo_14245_OG111
Other_Holozoa-Nvec_9995_OG111
Other_Holozoa-Cowc_9003_OG111
2.2579
2.5568
Other_Holozoa-Mvib_g1772_OG111
Other_Holozoa-Pvie_g7980_OG111
Osmo_Pseudofungi-Hcat_3008_OG111
2.6146
Other_Other-Sspe_24305_OG111
2.3005
Cp_Diaphoretickes-Aano_7085_OG111
2.4201
Cp_Diaphoretickes-Chsp_13537_OG111
2.2305
2.6904
Cp_Diaphoretickes-Ehux_1869_OG111
Cp_Diaphoretickes-Aano_9228_OG111
2.0109
Cp_Diaphoretickes-Aano_8210_OG111
2.5727
2.5926
Other_Other-Ngru_3867_OG111
Other_Stramenopiles-Bhom_309_OG111
Pp_Diaphoretickes-Smoe_3039_OG111
1.765
2.0692
Pp_Diaphoretickes-Smoe_6902_OG111
2.7359
2.1084
Pp_Diaphoretickes-Mpol_14229_OG111
2.3032
Pp_Diaphoretickes-Atha_17586_OG111
Pp_Diaphoretickes-Knit_4728_OG111
2.0154
2.4074
Pp_Diaphoretickes-Kfla_9086_OG111
Pp_Diaphoretickes-Catm_8047_OG111
2.4753
Pp_Diaphoretickes-Vcar_3895_OG111
2.7854
2.1003
2.3614
Pp_Diaphoretickes-Crei_13259_OG111
2.5258
Pp_Diaphoretickes-Cvar_707_OG111
Pp_Diaphoretickes-Npyr_25528_OG111
2.2907
2.3792
Pp_Diaphoretickes-Pcol_233_OG111
Pp_Diaphoretickes-Mpus_2098_OG111

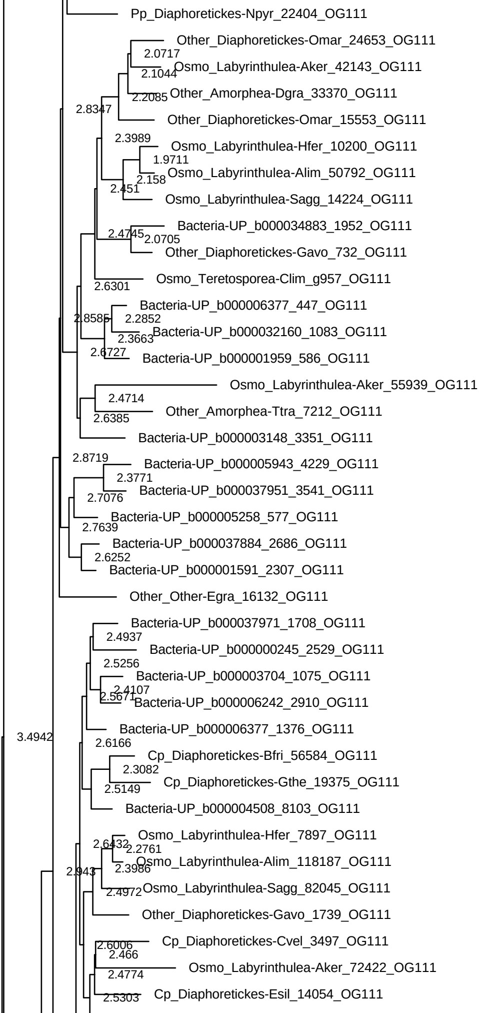
Pp_Diaphoretickes-Npyr_22404_OG111
Other_Diaphoretickes-Omar_24653_OG111
2.0717
Osmo_Labyrinthulea-Aker_42143_OG111
2.1044
Other_Amorphea-Dgra_33370_OG111
2.2085
2.8347
Other_Diaphoretickes-Omar_15553_OG111
2.3989
Osmo_Labyrinthulea-Hfer_10200_OG111
1.9711
Osmo_Labyrinthulea-Alim_50792_OG111
2.158
2.451
Osmo_Labyrinthulea-Sagg_14224_OG111
Bacteria-UP_b000034883_1952_OG111
2.4745
2.0705
Other_Diaphoretickes-Gavo_732_OG111
Osmo_Teretosporea-Clim_g957_OG111
2.6301
Bacteria-UP_b000006377_447_OG111
2.8585
2.2852
Bacteria-UP_b000032160_1083_OG111
2.3663
2.6727
Bacteria-UP_b000001959_586_OG111
Osmo_Labyrinthulea-Aker_55939_OG111
2.4714
Other_Amorphea-Ttra_7212_OG111
2.6385
Bacteria-UP_b000003148_3351_OG111
2.8719
Bacteria-UP_b000005943_4229_OG111
2.3771
Bacteria-UP_b000037951_3541_OG111
2.7076
Bacteria-UP_b000005258_577_OG111
2.7639
Bacteria-UP_b000037884_2686_OG111
2.6252
Bacteria-UP_b000001591_2307_OG111
Other_Other-Egra_16132_OG111
Bacteria-UP_b000037971_1708_OG111
2.4937
Bacteria-UP_b000000245_2529_OG111
2.5256
Bacteria-UP_b000003704_1075_OG111
2.4107
2.5671
Bacteria-UP_b000006242_2910_OG111
3.4942
Bacteria-UP_b000006377_1376_OG111
2.6166
Cp_Diaphoretickes-Bfri_56584_OG111
2.3082
Cp_Diaphoretickes-Gthe_19375_OG111
2.5149
Bacteria-UP_b000004508_8103_OG111
Osmo_Labyrinthulea-Hfer_7897_OG111
2.6432
2.2761
Osmo_Labyrinthulea-Alim_118187_OG111
2.943
2.3986
Osmo_Labyrinthulea-Sagg_82045_OG111
2.4972
Other_Diaphoretickes-Gavo_1739_OG111
Cp_Diaphoretickes-Cvel_3497_OG111
2.6006
2.466
Osmo_Labyrinthulea-Aker_72422_OG111
2.4774
Cp_Diaphoretickes-Esil_14054_OG111
2.5303

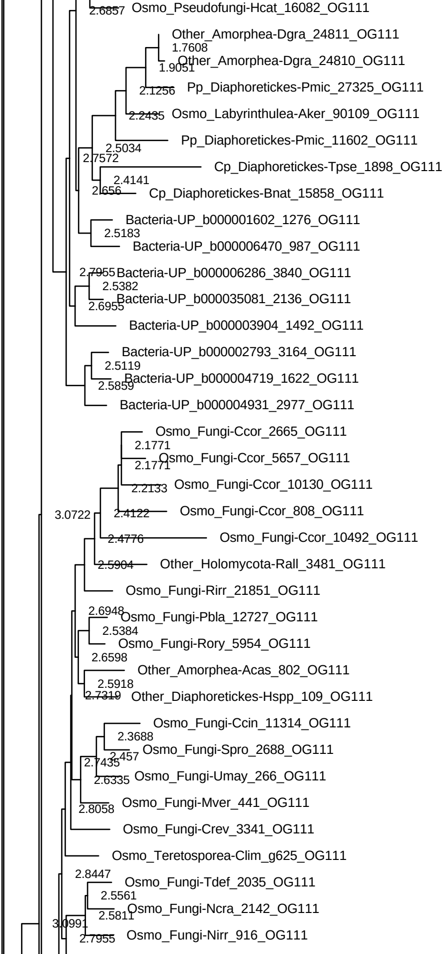

2.6857 Osmo_Pseudofungi-Hcat_16082_OG111
Other_Amorphea-Dgra_24811_OG111
1.7608
Other_Amorphea-Dgra_24810_OG111
1.9051
Pp_Diaphoretickes-Pmic_27325_OG111
2.1256
Osmo_Labyrinthulea-Aker_90109_OG111
2.2435
Pp_Diaphoretickes-Pmic_11602_OG111
2.5034
2.7572
Cp_Diaphoretickes-Tpse_1898_OG111
2.4141
2.656
Cp_Diaphoretickes-Bnat_15858_OG111
Bacteria-UP_b000001602_1276_OG111
2.5183
Bacteria-UP_b000006470_987_OG111
2.7955
Bacteria-UP_b000006286_3840_OG111
2.5382
2.6955
Bacteria-UP_b000035081_2136_OG111
Bacteria-UP_b000003904_1492_OG111
Bacteria-UP_b000002793_3164_OG111
2.5119
2.5859
Bacteria-UP_b000004719_1622_OG111
Bacteria-UP_b000004931_2977_OG111
Osmo_Fungi-Ccor_2665_OG111
2.1771
Osmo_Fungi-Ccor_5657_OG111
2.1771
Osmo_Fungi-Ccor_10130_OG111
2.2133
3.0722
2.4122
Osmo_Fungi-Ccor_808_OG111
2.4776
Osmo_Fungi-Ccor_10492_OG111
2.5904
Other_Holomycota-Rall_3481_OG111
Osmo_Fungi-Rirr_21851_OG111
2.6948
Osmo_Fungi-Pbla_12727_OG111
2.5384
Osmo_Fungi-Rory_5954_OG111
2.6598
Other_Amorphea-Acas_802_OG111
2.5918
2.7319
Other_Diaphoretickes-Hspp_109_OG111
Osmo_Fungi-Ccin_11314_OG111
2.3688
Osmo_Fungi-Spro_2688_OG111
2.457
2.7435
2.6335
Osmo_Fungi-Umay_266_OG111
2.8058
Osmo_Fungi-Mver_441_OG111
Osmo_Fungi-Crev_3341_OG111
Osmo_Teretosporea-Clim_g625_OG111
2.8447
Osmo_Fungi-Tdef_2035_OG111
2.5561
Osmo_Fungi-Ncra_2142_OG111
2.5811
3.0991
2.7955
Osmo_Fungi-Nirr_916_OG111

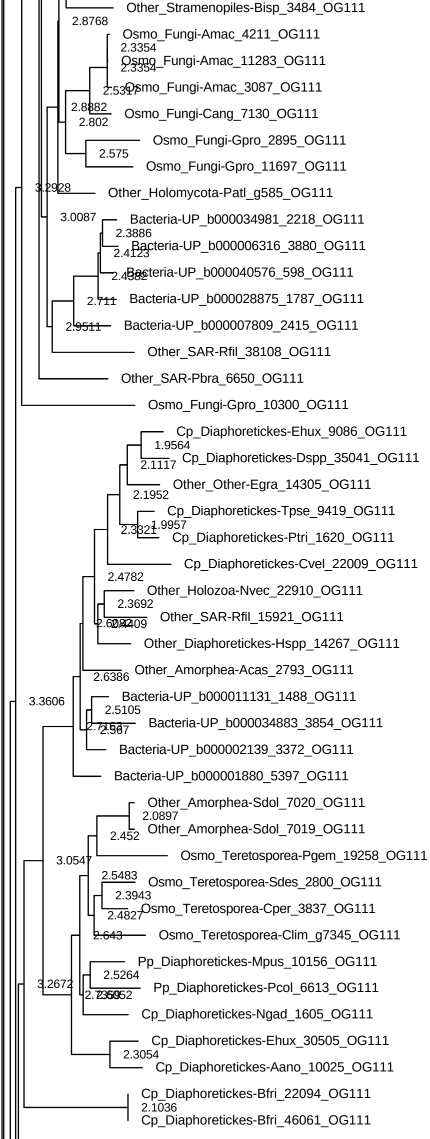

Other_Stramenopiles-Bisp_3484_OG111
2.8768
Osmo_Fungi-Amac_4211_OG111
2.3354
Osmo_Fungi-Amac_11283_OG111
2.3354
Osmo_Fungi-Amac_3087_OG111
2.8882
Osmo_Fungi-Cang_7130_OG111
2.802
Osmo_Fungi-Gpro_2895_OG111
2.575
Osmo_Fungi-Gpro_11697_OG111
3.2928
Other_Holomycota-Patl_g585_OG111
3.0087
Bacteria-UP_b000034981_2218_OG111
2.3886
Bacteria-UP_b000006316_3880_OG111
2.4123
Bacteria-UP_b000040576_598_OG111
2.4382
Bacteria-UP_b000028875_1787_OG111
2.711
Bacteria-UP_b000007809_2415_OG111
2.9511
Other_SAR-Rfil_38108_OG111
Other_SAR-Pbra_6650_OG111
Osmo_Fungi-Gpro_10300_OG111
Cp_Diaphoretickes-Ehux_9086_OG111
1.9564
Cp_Diaphoretickes-Dspp_35041_OG111
2.1117
Other_Other-Egra_14305_OG111
2.1952
Cp_Diaphoretickes-Tpse_9419_OG111
1.9957
2.3321
Cp_Diaphoretickes-Ptri_1620_OG111
Cp_Diaphoretickes-Cvel_22009_OG111
2.4782
Other_Holozoa-Nvec_22910_OG111
2.3692
Other_SAR-Rfil_15921_OG111
Other_Diaphoretickes-Hspp_14267_OG111
Other_Amorphea-Acas_2793_OG111
2.6386
3.3606
Bacteria-UP_b000011131_1488_OG111
2.5105
Bacteria-UP_b000034883_3854_OG111
Bacteria-UP_b000002139_3372_OG111
Bacteria-UP_b000001880_5397_OG111
Other_Amorphea-Sdol_7020_OG111
2.0897
Other_Amorphea-Sdol_7019_OG111
2.452
3.0547
Osmo_Teretosporea-Pgem_19258_OG111
2.5483
Osmo_Teretosporea-Sdes_2800_OG111
2.3943
Osmo_Teretosporea-Cper_3837_OG111
2.4827
2.643
Osmo_Teretosporea-Clim_g7345_OG111
Pp_Diaphoretickes-Mpus_10156_OG111
2.5264
3.2672
Pp_Diaphoretickes-Pcol_6613_OG111
Cp_Diaphoretickes-Ngad_1605_OG111
Cp_Diaphoretickes-Ehux_30505_OG111
2.3054
Cp_Diaphoretickes-Aano_10025_OG111
Cp_Diaphoretickes-Bfri_22094_OG111
2.1036
Cp_Diaphoretickes-Bfri_46061_OG111

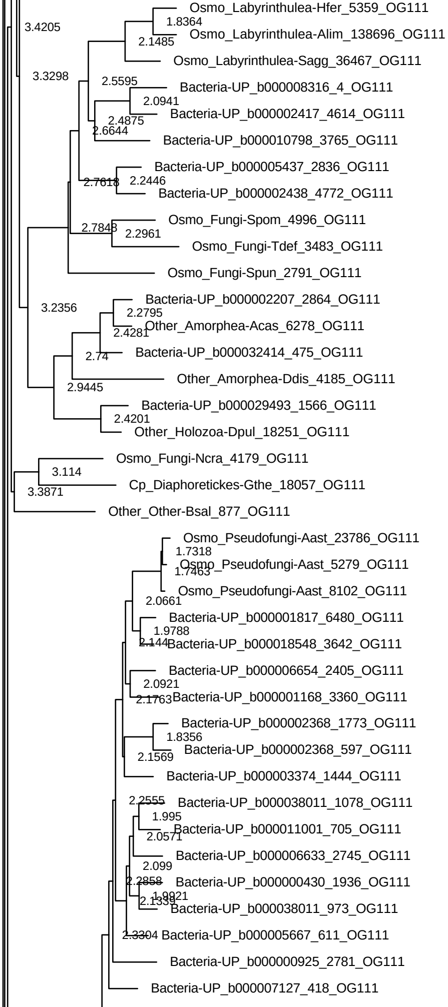
3.4205
Osmo_Labyrinthulea-Hfer_5359_OG111
1.8364
Osmo_Labyrinthulea-Alim_138696_OG111
2.1485
Osmo_Labyrinthulea-Sagg_36467_OG111
3.3298
2.5595
Bacteria-UP_b000008316_4_OG111
2.0941
Bacteria-UP_b000002417_4614_OG111
2.4875
2.6644
Bacteria-UP_b000010798_3765_OG111
Bacteria-UP_b000005437_2836_OG111
2.7618
2.2446
Bacteria-UP_b000002438_4772_OG111
Osmo_Fungi-Spom_4996_OG111
2.7843
2.2961
Osmo_Fungi-Tdef_3483_OG111
Osmo_Fungi-Spun_2791_OG111
Bacteria-UP_b000002207_2864_OG111
3.2356
2.2795
2.4281
Other_Amorphea-Acas_6278_OG111
2.74
Bacteria-UP_b000032414_475_OG111
Other_Amorphea-Ddis_4185_OG111
2.9445
Bacteria-UP_b000029493_1566_OG111
2.4201
Other_Holozoa-Dpul_18251_OG111
Osmo_Fungi-Ncra_4179_OG111
3.114
Cp_Diaphoretickes-Gthe_18057_OG111
3.3871
Other_Other-Bsal_877_OG111
Osmo_Pseudofungi-Aast_23786_OG111
1.7318
Osmo_Pseudofungi-Aast_5279_OG111
1.7463
Osmo_Pseudofungi-Aast_8102_OG111
2.0661
Bacteria-UP_b000001817_6480_OG111
1.9788
2.144
Bacteria-UP_b000018548_3642_OG111
Bacteria-UP_b000006654_2405_OG111
2.0921
2.1763
Bacteria-UP_b000001168_3360_OG111
Bacteria-UP_b000002368_1773_OG111
1.8356
Bacteria-UP_b000002368_597_OG111
2.1569
Bacteria-UP_b000003374_1444_OG111
2.2555
Bacteria-UP_b000038011_1078_OG111
1.995
2.0571
Bacteria-UP_b000011001_705_OG111
Bacteria-UP_b000006633_2745_OG111
2.099
2.2858
Bacteria-UP_b000000430_1936_OG111
1.9921
2.1339
Bacteria-UP_b000038011_973_OG111
2.3304
Bacteria-UP_b000005667_611_OG111
Bacteria-UP_b000000925_2781_OG111
Bacteria-UP_b000007127_418_OG111

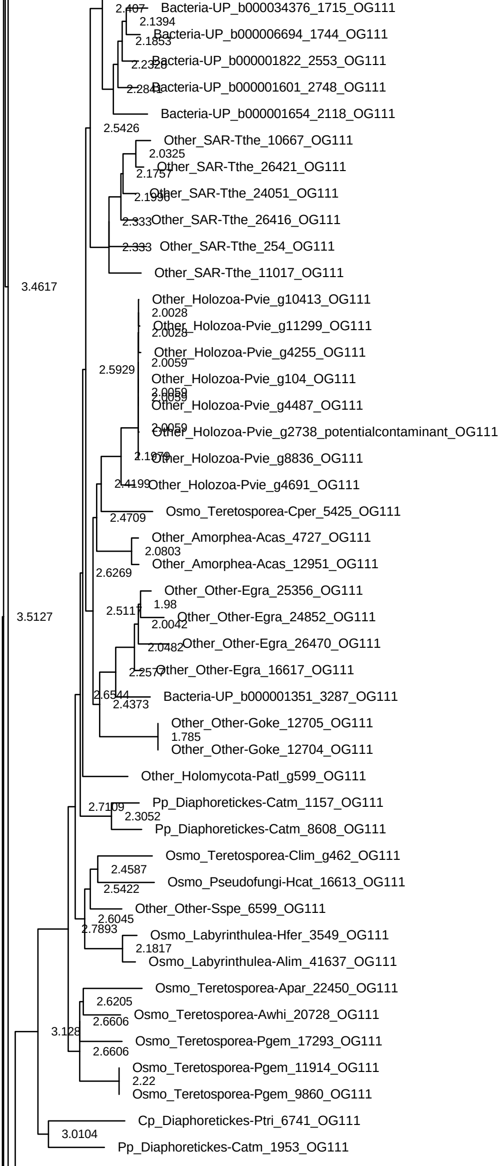
Bacteria-UP_b000034376_1715_OG111
2.407
2.1394
Bacteria-UP_b000006694_1744_OG111
2.1853
Bacteria-UP_b000001822_2553_OG111
2.2328
Bacteria-UP_b000001601_2748_OG111
2.2841
Bacteria-UP_b000001654_2118_OG111
2.5426
Other_SAR-Tthe_10667_OG111
2.0325
Other_SAR-Tthe_26421_OG111
2.1757
Other_SAR-Tthe_24051_OG111
2.1996
Other_SAR-Tthe_26416_OG111
2.333
Other_SAR-Tthe_254_OG111
2.333
Other_SAR-Tthe_11017_OG111
3.4617
Other_Holozoa-Pvie_g10413_OG111
2.0028
Other_Holozoa-Pvie_g11299_OG111
2.0028
Other_Holozoa-Pvie_g4255_OG111
2.0059
2.5929
Other_Holozoa-Pvie_g104_OG111
2.0059
2.0059
Other_Holozoa-Pvie_g4487_OG111
2.0059
Other_Holozoa-Pvie_g2738_potentialcontaminant_OG111
2.1979
Other_Holozoa-Pvie_g8836_OG111
2.4199
Other_Holozoa-Pvie_g4691_OG111
Osmo_Teretosporea-Cper_5425_OG111
2.4709
Other_Amorphea-Acas_4727_OG111
2.0803
Other_Amorphea-Acas_12951_OG111
2.6269
Other_Other-Egra_25356_OG111
1.98
2.5117
3.5127
Other_Other-Egra_24852_OG111
2.0042
Other_Other-Egra_26470_OG111
2.0482
Other_Other-Egra_16617_OG111
2.2577
2.6544
Bacteria-UP_b000001351_3287_OG111
2.4373
Other_Other-Goke_12705_OG111
1.785
Other_Other-Goke_12704_OG111
Other_Holomycota-Patl_g599_OG111
Pp_Diaphoretickes-Catm_1157_OG111
2.7109
2.3052
Pp_Diaphoretickes-Catm_8608_OG111
Osmo_Teretosporea-Clim_g462_OG111
2.4587
Osmo_Pseudofungi-Hcat_16613_OG111
2.5422
Other_Other-Sspe_6599_OG111
2.6045
2.7893
Osmo_Labyrinthulea-Hfer_3549_OG111
2.1817
Osmo_Labyrinthulea-Alim_41637_OG111
Osmo_Teretosporea-Apar_22450_OG111
2.6205
Osmo_Teretosporea-Awhi_20728_OG111
2.6606
3.128
Osmo_Teretosporea-Pgem_17293_OG111
2.6606
Osmo_Teretosporea-Pgem_11914_OG111
2.22
Osmo_Teretosporea-Pgem_9860_OG111
Cp_Diaphoretickes-Ptri_6741_OG111
3.0104
Pp_Diaphoretickes-Catm_1953_OG111

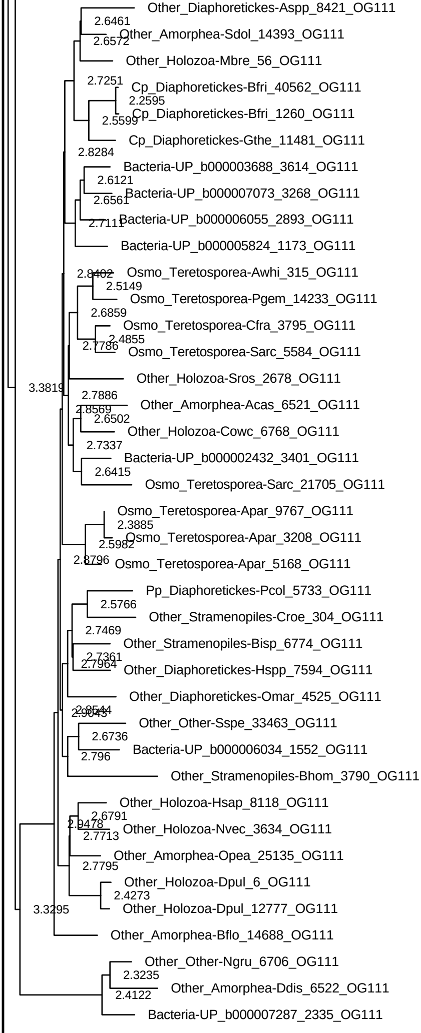

Other_Diaphoretickes-Aspp_8421_OG111
2.6461
Other_Amorphea-Sdol_14393_OG111
2.6572
Other_Holozoa-Mbre_56_OG111
2.7251
Cp_Diaphoretickes-Bfri_40562_OG111
2.2595
Cp_Diaphoretickes-Bfri_1260_OG111
2.5599
Cp_Diaphoretickes-Gthe_11481_OG111
2.8284
Bacteria-UP_b000003688_3614_OG111
2.6121
Bacteria-UP_b000007073_3268_OG111
2.6561
Bacteria-UP_b000006055_2893_OG111
2.7111
Bacteria-UP_b000005824_1173_OG111
2.8402
Osmo_Teretosporea-Awhi_315_OG111
2.5149
Osmo_Teretosporea-Pgem_14233_OG111
2.6859
Osmo_Teretosporea-Cfra_3795_OG111
2.4855
2.7786
Osmo_Teretosporea-Sarc_5584_OG111
Other_Holozoa-Sros_2678_OG111
3.3819
2.7886
Other_Amorphea-Acas_6521_OG111
2.8569
2.6502
Other_Holozoa-Cowc_6768_OG111
2.7337
Bacteria-UP_b000002432_3401_OG111
2.6415
Osmo_Teretosporea-Sarc_21705_OG111
Osmo_Teretosporea-Apar_9767_OG111
2.3885
Osmo_Teretosporea-Apar_3208_OG111
2.5982
2.8796
Osmo_Teretosporea-Apar_5168_OG111
Pp_Diaphoretickes-Pcol_5733_OG111
2.5766
Other_Stramenopiles-Croe_304_OG111
2.7469
Other_Stramenopiles-Bisp_6774_OG111
2.7361
2.7964
Other_Diaphoretickes-Hspp_7594_OG111
Other_Diaphoretickes-Omar_4525_OG111
2.8544
2.9043
Other_Other-Sspe_33463_OG111
2.6736
Bacteria-UP_b000006034_1552_OG111
2.796
Other_Stramenopiles-Bhom_3790_OG111
Other_Holozoa-Hsap_8118_OG111
2.6791
2.9478
Other_Holozoa-Nvec_3634_OG111
2.7713
Other_Amorphea-Opea_25135_OG111
2.7795
Other_Holozoa-Dpul_6_OG111
2.4273
3.3295
Other_Holozoa-Dpul_12777_OG111
Other_Amorphea-Bflo_14688_OG111
Other_Other-Ngru_6706_OG111
2.3235
Other_Amorphea-Ddis_6522_OG111
2.4122
Bacteria-UP_b000007287_2335_OG111

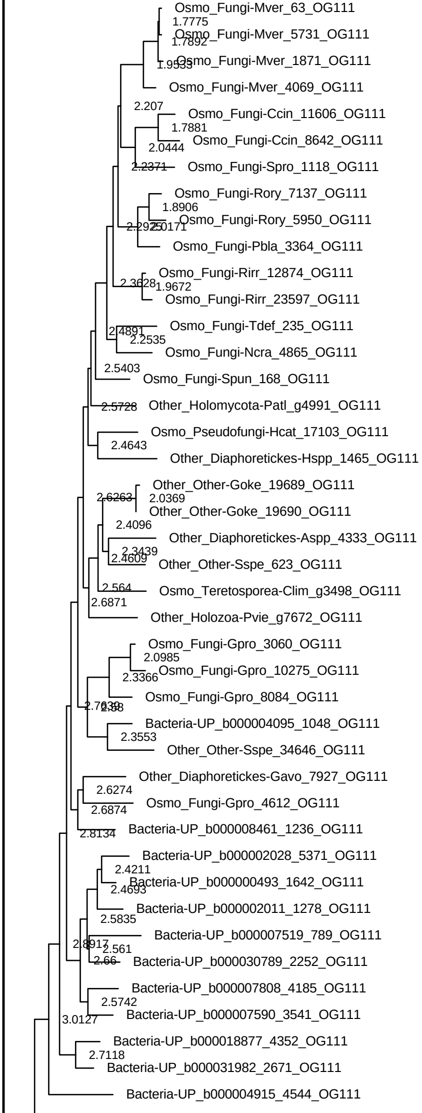
Osmo_Fungi-Mver_63_OG111
1.7775
Osmo_Fungi-Mver_5731_OG111
1.7892
Osmo_Fungi-Mver_1871_OG111
1.9533
Osmo_Fungi-Mver_4069_OG111
2.207
Osmo_Fungi-Ccin_11606_OG111
1.7881
Osmo_Fungi-Ccin_8642_OG111
2.0444
2.2371
Osmo_Fungi-Spro_1118_OG111
Osmo_Fungi-Rory_7137_OG111
1.8906
Osmo_Fungi-Rory_5950_OG111
2.0171
Osmo_Fungi-Pbla_3364_OG111
Osmo_Fungi-Rirr_12874_OG111
2.3628
1.9672
Osmo_Fungi-Rirr_23597_OG111
Osmo_Fungi-Tdef_235_OG111
2.4891
2.2535
Osmo_Fungi-Ncra_4865_OG111
2.5403
Osmo_Fungi-Spun_168_OG111
2.5728
Other_Holomycota-Patl_g4991_OG111
Osmo_Pseudofungi-Hcat_17103_OG111
2.4643
Other_Diaphoretickes-Hspp_1465_OG111
Other_Other-Goke_19689_OG111
2.6263
2.0369
Other_Other-Goke_19690_OG111
2.4096
Other_Diaphoretickes-Aspp_4333_OG111
2.3439
2.4609
Other_Other-Sspe_623_OG111
2.564
Osmo_Teretosporea-Clim_g3498_OG111
2.6871
Other_Holozoa-Pvie_g7672_OG111
Osmo_Fungi-Gpro_3060_OG111
2.0985
Osmo_Fungi-Gpro_10275_OG111
2.3366
Osmo_Fungi-Gpro_8084_OG111
2.38
Bacteria-UP_b000004095_1048_OG111
2.3553
Other_Other-Sspe_34646_OG111
Other_Diaphoretickes-Gavo_7927_OG111
2.6274
Osmo_Fungi-Gpro_4612_OG111
2.6874
2.8134
Bacteria-UP_b000008461_1236_OG111
Bacteria-UP_b000002028_5371_OG111
2.4211
Bacteria-UP_b000000493_1642_OG111
2.4693
Bacteria-UP_b000002011_1278_OG111
2.5835
Bacteria-UP_b000007519_789_OG111
2.8917
2.561
2.66
Bacteria-UP_b000030789_2252_OG111
Bacteria-UP_b000007808_4185_OG111
2.5742
Bacteria-UP_b000007590_3541_OG111
3.0127
Bacteria-UP_b000018877_4352_OG111
2.7118
Bacteria-UP_b000031982_2671_OG111
Bacteria-UP_b000004915_4544_OG111

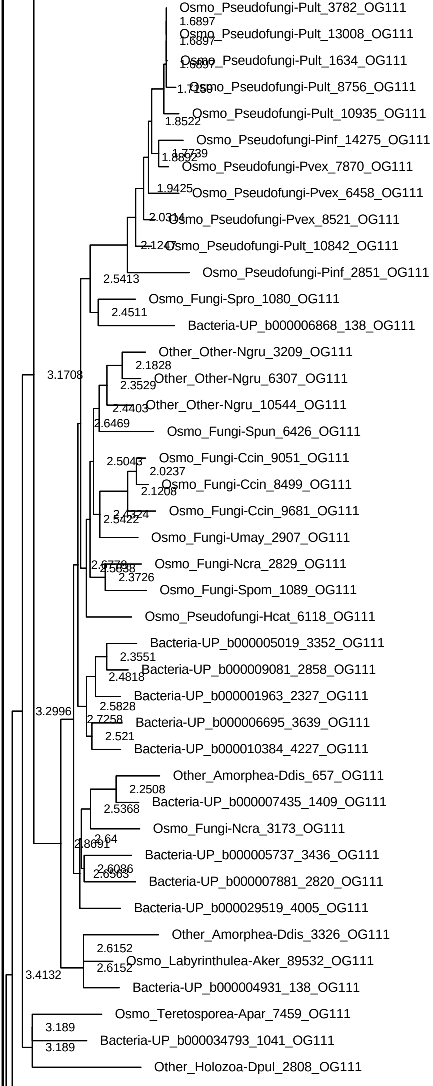
Osmo_Pseudofungi-Pult_3782_OG111
1.6897
Osmo_Pseudofungi-Pult_13008_OG111
1.6897
Osmo_Pseudofungi-Pult_1634_OG111
1.6897
1.7150
Osmo_Pseudofungi-Pult_8756_OG111
Osmo_Pseudofungi-Pult_10935_OG111
1.8522
Osmo_Pseudofungi-Pinf_14275_OG111
1.7739
1.8892
Osmo_Pseudofungi-Pvex_7870_OG111
1.9425
Osmo_Pseudofungi-Pvex_6458_OG111
2.0314
Osmo_Pseudofungi-Pvex_8521_OG111
2.1247
Osmo_Pseudofungi-Pult_10842_OG111
Osmo_Pseudofungi-Pinf_2851_OG111
2.5413
Osmo_Fungi-Spro_1080_OG111
2.4511
Bacteria-UP_b000006868_138_OG111
Other_Other-Ngru_3209_OG111
2.1828
3.1708
Other_Other-Ngru_6307_OG111
2.3529
Other_Other-Ngru_10544_OG111
2.4403
2.6469
Osmo_Fungi-Spun_6426_OG111
Osmo_Fungi-Ccin_9051_OG111
2.5043
2.0237
Osmo_Fungi-Ccin_8499_OG111
2.1208
Osmo_Fungi-Ccin_9681_OG111
2.4324
2.5422
Osmo_Fungi-Umay_2907_OG111
Osmo_Fungi-Ncra_2829_OG111
2.6778
2.5638
2.3726
Osmo_Fungi-Spom_1089_OG111
Osmo_Pseudofungi-Hcat_6118_OG111
Bacteria-UP_b000005019_3352_OG111
2.3551
Bacteria-UP_b000009081_2858_OG111
2.4818
Bacteria-UP_b000001963_2327_OG111
2.5828
3.2996
2.7258
Bacteria-UP_b000006695_3639_OG111
2.521
Bacteria-UP_b000010384_4227_OG111
Other_Amorphea-Ddis_657_OG111
2.2508
Bacteria-UP_b000007435_1409_OG111
2.5368
Osmo_Fungi-Ncra_3173_OG111
2.64
2.8691
Bacteria-UP_b000005737_3436_OG111
2.6086
2.6563
Bacteria-UP_b000007881_2820_OG111
Bacteria-UP_b000029519_4005_OG111
Other_Amorphea-Ddis_3326_OG111
2.6152
Osmo_Labyrinthulea-Aker_89532_OG111
2.6152
3.4132
Bacteria-UP_b000004931_138_OG111
Osmo_Teretosporea-Apar_7459_OG111
3.189
Bacteria-UP_b000034793_1041_OG111
3.189
Other_Holozoa-Dpul_2808_OG111

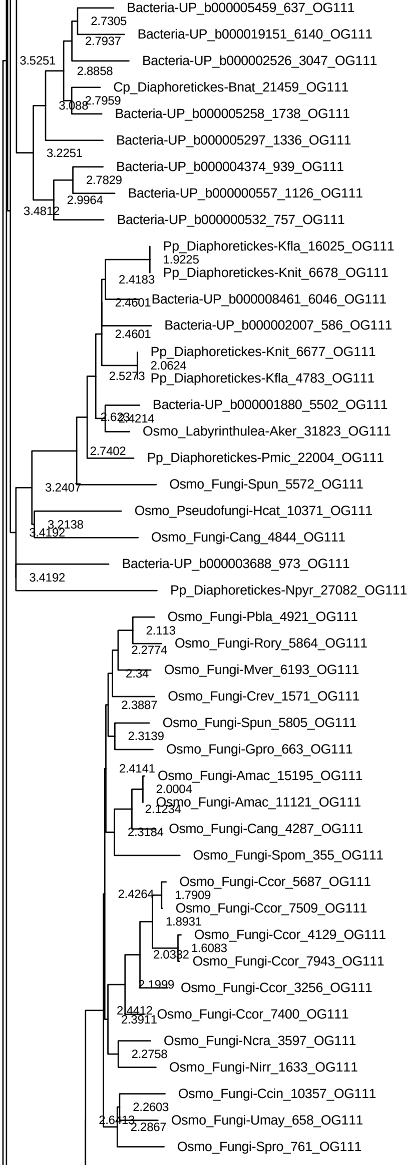
Bacteria-UP_b000005459_637_OG111
2.7305
2.7937
Bacteria-UP_b000019151_6140_OG111
3.5251
Bacteria-UP_b000002526_3047_OG111
2.8858
Cp_Diaphoretickes-Bnat_21459_OG111
2.7959
3.088
Bacteria-UP_b000005258_1738_OG111
Bacteria-UP_b000005297_1336_OG111
3.2251
Bacteria-UP_b000004374_939_OG111
2.7829
Bacteria-UP_b000000557_1126_OG111
2.9964
3.4812
Bacteria-UP_b000000532_757_OG111
Pp_Diaphoretickes-Kfla_16025_OG111
1.9225
Pp_Diaphoretickes-Knit_6678_OG111
2.4183
2.4601
Bacteria-UP_b000008461_6046_OG111
Bacteria-UP_b000002007_586_OG111
2.4601
Pp_Diaphoretickes-Knit_6677_OG111
2.0624
2.5273
Pp_Diaphoretickes-Kfla_4783_OG111
Bacteria-UP_b000001880_5502_OG111
2.623
2.4214
Osmo_Labyrinthulea-Aker_31823_OG111
2.7402
Pp_Diaphoretickes-Pmic_22004_OG111
3.2407
Osmo_Fungi-Spun_5572_OG111
Osmo_Pseudofungi-Hcat_10371_OG111
3.2138
3.4192
Osmo_Fungi-Cang_4844_OG111
Bacteria-UP_b000003688_973_OG111
3.4192
Pp_Diaphoretickes-Npyr_27082_OG111
Osmo_Fungi-Pbla_4921_OG111
2.113
Osmo_Fungi-Rory_5864_OG111
2.2774
2.34
Osmo_Fungi-Mver_6193_OG111
Osmo_Fungi-Crev_1571_OG111
2.3887
Osmo_Fungi-Spun_5805_OG111
2.3139
Osmo_Fungi-Gpro_663_OG111
2.4141
Osmo_Fungi-Amac_15195_OG111
2.0004
Osmo_Fungi-Amac_11121_OG111
2.1234
2.3184
Osmo_Fungi-Cang_4287_OG111
Osmo_Fungi-Spom_355_OG111
Osmo_Fungi-Ccor_5687_OG111
2.4264
1.7909
Osmo_Fungi-Ccor_7509_OG111
1.8931
Osmo_Fungi-Ccor_4129_OG111
1.6083
2.0382
Osmo_Fungi-Ccor_7943_OG111
2.1999
Osmo_Fungi-Ccor_3256_OG111
2.4412
2.3911
Osmo_Fungi-Ccor_7400_OG111
Osmo_Fungi-Ncra_3597_OG111
2.2758
Osmo_Fungi-Nirr_1633_OG111
Osmo_Fungi-Ccin_10357_OG111
2.2603
2.6413
Osmo_Fungi-Umay_658_OG111
2.2867
Osmo_Fungi-Spro_761_OG111

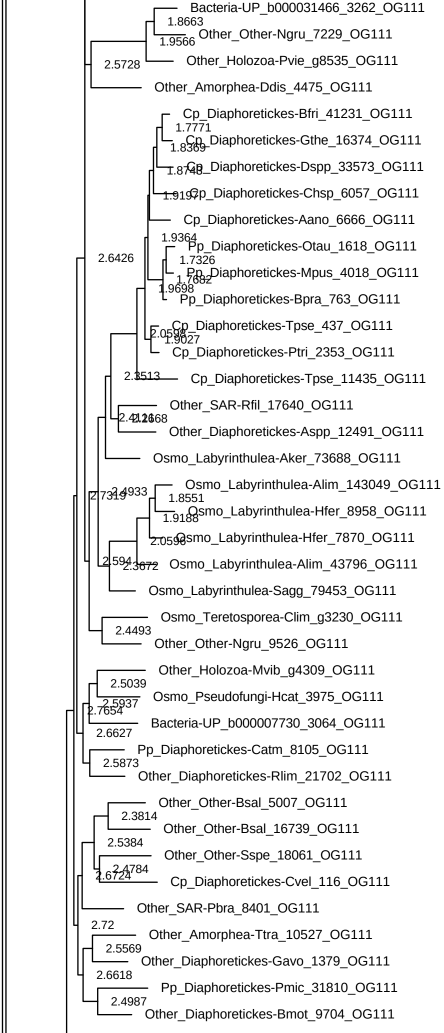

Bacteria-UP_b000031466_3262_OG111
1.8663
Other_Other-Ngru_7229_OG111
1.9566
Other_Holozoa-Pvie_g8535_OG111
2.5728
Other_Amorphea-Ddis_4475_OG111
Cp_Diaphoretickes-Bfri_41231_OG111
1.7771
Cp_Diaphoretickes-Gthe_16374_OG111
1.8369
Cp_Diaphoretickes-Dspp_33573_OG111
1.8748
Cp_Diaphoretickes-Chsp_6057_OG111
1.9197
Cp_Diaphoretickes-Aano_6666_OG111
1.9364
Pp_Diaphoretickes-Otau_1618_OG111
2.6426
1.7326
Pp_Diaphoretickes-Mpus_4018_OG111
1.7682
1.9698
Pp_Diaphoretickes-Bpra_763_OG111
Cp_Diaphoretickes-Tpse_437_OG111
2.0598
1.9027
Cp_Diaphoretickes-Ptri_2353_OG111
2.3513
Cp_Diaphoretickes-Tpse_11435_OG111
Other_SAR-Rfil_17640_OG111
Other_Diaphoretickes-Aspp_12491_OG111
Osmo_Labyrinthulea-Aker_73688_OG111
Osmo_Labyrinthulea-Alim_143049_OG111
2.7319
2.4933
1.8551
Osmo_Labyrinthulea-Hfer_8958_OG111
1.9188
Osmo_Labyrinthulea-Hfer_7870_OG111
2.0596
2.594
2.3672
Osmo_Labyrinthulea-Alim_43796_OG111
Osmo_Labyrinthulea-Sagg_79453_OG111
Osmo_Teretosporea-Clim_g3230_OG111
2.4493
Other_Other-Ngru_9526_OG111
Other_Holozoa-Mvib_g4309_OG111
2.5039
Osmo_Pseudofungi-Hcat_3975_OG111
2.5937
2.7654
Bacteria-UP_b000007730_3064_OG111
2.6627
Pp_Diaphoretickes-Catm_8105_OG111
2.5873
Other_Diaphoretickes-Rlim_21702_OG111
Other_Other-Bsal_5007_OG111
2.3814
Other_Other-Bsal_16739_OG111
2.5384
Other_Other-Sspe_18061_OG111
2.4784
2.6724
Cp_Diaphoretickes-Cvel_116_OG111
Other_SAR-Pbra_8401_OG111
2.72
Other_Amorphea-Ttra_10527_OG111
2.5569
Other_Diaphoretickes-Gavo_1379_OG111
2.6618
Pp_Diaphoretickes-Pmic_31810_OG111
2.4987
Other_Diaphoretickes-Bmot_9704_OG111

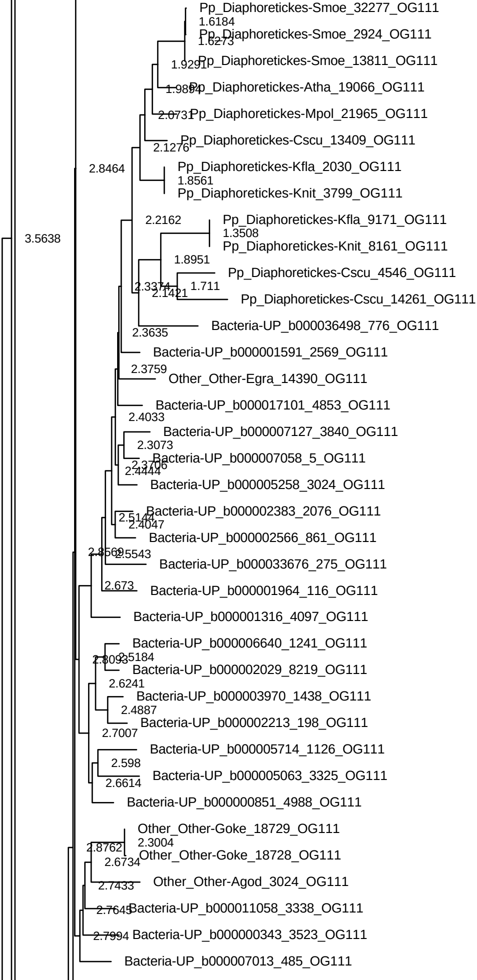

Pp_Diaphoretickes-Smoe_32277_OG111
1.6184
Pp_Diaphoretickes-Smoe_2924_OG111
1.6273
1.9291
Pp_Diaphoretickes-Smoe_13811_OG111
1.9894
Pp_Diaphoretickes-Atha_19066_OG111
2.0731
Pp_Diaphoretickes-Mpol_21965_OG111
2.1276
Pp_Diaphoretickes-Cscu_13409_OG111
2.8464
Pp_Diaphoretickes-Kfla_2030_OG111
1.8561
Pp_Diaphoretickes-Knit_3799_OG111
2.2162
Pp_Diaphoretickes-Kfla_9171_OG111
1.3508
3.5638
Pp_Diaphoretickes-Knit_8161_OG111
1.8951
Pp_Diaphoretickes-Cscu_4546_OG111
2.3374
1.711
2.1421
Pp_Diaphoretickes-Cscu_14261_OG111
Bacteria-UP_b000036498_776_OG111
2.3635
Bacteria-UP_b000001591_2569_OG111
2.3759
Other_Other-Egra_14390_OG111
Bacteria-UP_b000017101_4853_OG111
2.4033
Bacteria-UP_b000007127_3840_OG111
2.3073
Bacteria-UP_b000007058_5_OG111
2.3706
2.4444
Bacteria-UP_b000005258_3024_OG111
Bacteria-UP_b000002383_2076_OG111
2.5144
2.4047
Bacteria-UP_b000002566_861_OG111
2.8569
2.5543
Bacteria-UP_b000033676_275_OG111
2.673
Bacteria-UP_b000001964_116_OG111
Bacteria-UP_b000001316_4097_OG111
Bacteria-UP_b000006640_1241_OG111
2.5184
2.8093
Bacteria-UP_b000002029_8219_OG111
2.6241
Bacteria-UP_b000003970_1438_OG111
2.4887
Bacteria-UP_b000002213_198_OG111
2.7007
Bacteria-UP_b000005714_1126_OG111
2.598
Bacteria-UP_b000005063_3325_OG111
2.6614
Bacteria-UP_b000000851_4988_OG111
Other_Other-Goke_18729_OG111
2.3004
2.8762
Other_Other-Goke_18728_OG111
2.6734
Other_Other-Agod_3024_OG111
2.7433
2.7645
Bacteria-UP_b000011058_3338_OG111
2.7994
Bacteria-UP_b000000343_3523_OG111
Bacteria-UP_b000007013_485_OG111

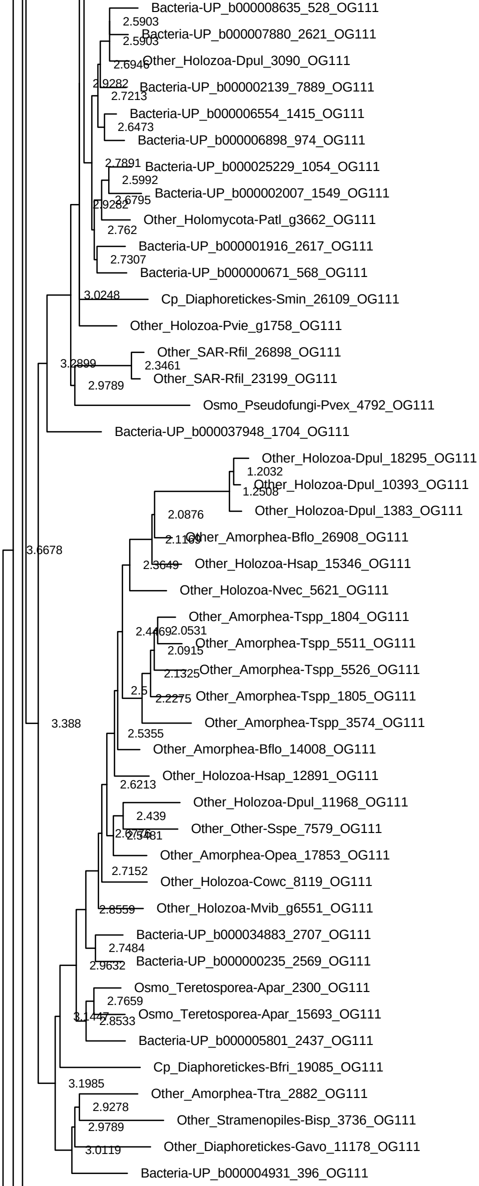
Bacteria-UP_b000008635_528_OG111
2.5903
Bacteria-UP_b000007880_2621_OG111
2.5903
Other_Holozoa-Dpul_3090_OG111
2.6946
2.9282
Bacteria-UP_b000002139_7889_OG111
2.7213
Bacteria-UP_b000006554_1415_OG111
2.6473
Bacteria-UP_b000006898_974_OG111
2.7891
Bacteria-UP_b000025229_1054_OG111
2.5992
Bacteria-UP_b000002007_1549_OG111
2.6795
2.9282
Other_Holomycota-Patl_g3662_OG111
2.762
Bacteria-UP_b000001916_2617_OG111
2.7307
Bacteria-UP_b000000671_568_OG111
3.0248
Cp_Diaphoretickes-Smin_26109_OG111
Other_Holozoa-Pvie_g1758_OG111
Other_SAR-Rfil_26898_OG111
3.2899
2.3461
Other_SAR-Rfil_23199_OG111
2.9789
Osmo_Pseudofungi-Pvex_4792_OG111
Bacteria-UP_b000037948_1704_OG111
Other_Holozoa-Dpul_18295_OG111
1.2032
Other_Holozoa-Dpul_10393_OG111
1.2508
Other_Holozoa-Dpul_1383_OG111
2.0876
Other_Amorphea-Bflo_26908_OG111
2.1169
3.6678
Other_Holozoa-Hsap_15346_OG111
2.3649
Other_Holozoa-Nvec_5621_OG111
Other_Amorphea-Tspp_1804_OG111
2.4469
2.0531
Other_Amorphea-Tspp_5511_OG111
2.0915
Other_Amorphea-Tspp_5526_OG111
2.1325
2.5
Other_Amorphea-Tspp_1805_OG111
2.2275
3.388
Other_Amorphea-Tspp_3574_OG111
2.5355
Other_Amorphea-Bflo_14008_OG111
Other_Holozoa-Hsap_12891_OG111
2.6213
Other_Holozoa-Dpul_11968_OG111
2.439
Other_Other-Sspe_7579_OG111
2.6776
2.5481
Other_Amorphea-Opea_17853_OG111
2.7152
Other_Holozoa-Cowc_8119_OG111
2.8559
Other_Holozoa-Mvib_g6551_OG111
Bacteria-UP_b000034883_2707_OG111
2.7484
Bacteria-UP_b000000235_2569_OG111
2.9632
Osmo_Teretosporea-Apar_2300_OG111
2.7659
3.1447
2.8533
Osmo_Teretosporea-Apar_15693_OG111
Bacteria-UP_b000005801_2437_OG111
Cp_Diaphoretickes-Bfri_19085_OG111
3.1985
Other_Amorphea-Ttra_2882_OG111
2.9278
Other_Stramenopiles-Bisp_3736_OG111
2.9789
Other_Diaphoretickes-Gavo_11178_OG111
3.0119
Bacteria-UP_b000004931_396_OG111

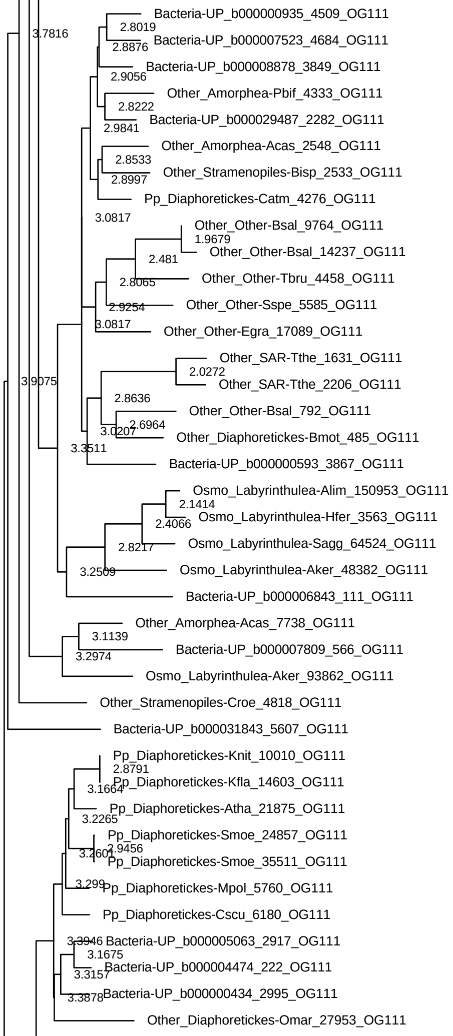
Bacteria-UP_b000000935_4509_OG111
2.8019
Bacteria-UP_b000007523_4684_OG111
2.8876
3.7816
Bacteria-UP_b000008878_3849_OG111
2.9056
Other_Amorphea-Pbif_4333_OG111
2.8222
Bacteria-UP_b000029487_2282_OG111
2.9841
Other_Amorphea-Acas_2548_OG111
2.8533
Other_Stramenopiles-Bisp_2533_OG111
2.8997
Pp_Diaphoretickes-Catm_4276_OG111
3.0817
Other_Other-Bsal_9764_OG111
1.9679
Other_Other-Bsal_14237_OG111
2.481
Other_Other-Tbru_4458_OG111
2.8065
Other_Other-Sspe_5585_OG111
2.9254
3.0817
Other_Other-Egra_17089_OG111
Other_SAR-Tthe_1631_OG111
2.0272
Other_SAR-Tthe_2206_OG111
3.9075
2.8636
Other_Other-Bsal_792_OG111
2.6964
3.0207
Other_Diaphoretickes-Bmot_485_OG111
3.3511
Bacteria-UP_b000000593_3867_OG111
Osmo_Labyrinthulea-Alim_150953_OG111
2.1414
Osmo_Labyrinthulea-Hfer_3563_OG111
2.4066
Osmo_Labyrinthulea-Sagg_64524_OG111
2.8217
3.2509
Osmo_Labyrinthulea-Aker_48382_OG111
Bacteria-UP_b000006843_111_OG111
Other_Amorphea-Acas_7738_OG111
3.1139
Bacteria-UP_b000007809_566_OG111
3.2974
Osmo_Labyrinthulea-Aker_93862_OG111
Other_Stramenopiles-Croe_4818_OG111
Bacteria-UP_b000031843_5607_OG111
Pp_Diaphoretickes-Knit_10010_OG111
2.8791
Pp_Diaphoretickes-Kfla_14603_OG111
3.1664
Pp_Diaphoretickes-Atha_21875_OG111
3.2265
Pp_Diaphoretickes-Smoe_24857_OG111
2.9456
3.2601
Pp_Diaphoretickes-Smoe_35511_OG111
3.299
Pp_Diaphoretickes-Mpol_5760_OG111
Pp_Diaphoretickes-Cscu_6180_OG111
3.3946
Bacteria-UP_b000005063_2917_OG111
3.1675
Bacteria-UP_b000004474_222_OG111
3.3157
Bacteria-UP_b000000434_2995_OG111
3.3878
Other_Diaphoretickes-Omar_27953_OG111

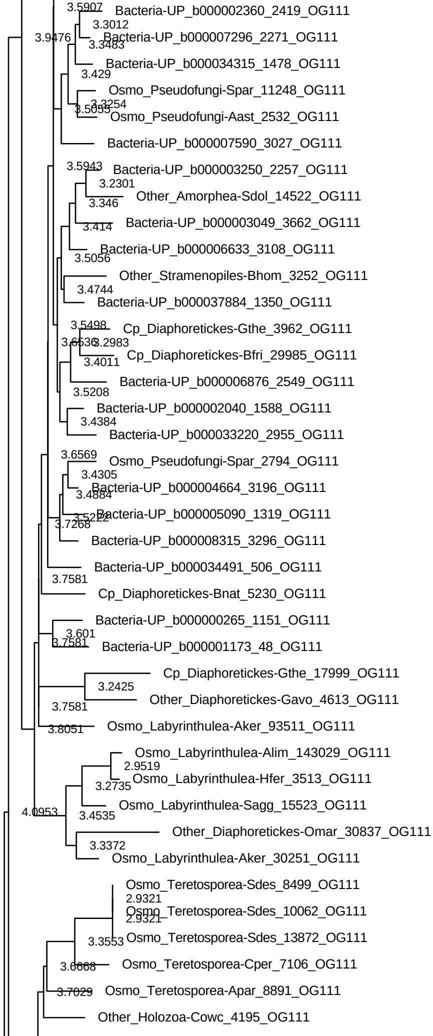
3.5907
Bacteria-UP_b000002360_2419_OG111
3.3012
3.9476
Bacteria-UP_b000007296_2271_OG111
3.3483
Bacteria-UP_b000034315_1478_OG111
3.429
Osmo_Pseudofungi-Spar_11248_OG111
3.3254
3.5055
Osmo_Pseudofungi-Aast_2532_OG111
Bacteria-UP_b000007590_3027_OG111
3.5943
Bacteria-UP_b000003250_2257_OG111
3.2301
Other_Amorphea-Sdol_14522_OG111
3.346
Bacteria-UP_b000003049_3662_OG111
3.414
Bacteria-UP_b000006633_3108_OG111
3.5056
Other_Stramenopiles-Bhom_3252_OG111
3.4744
Bacteria-UP_b000037884_1350_OG111
3.5498
Cp_Diaphoretickes-Gthe_3962_OG111
3.6536
3.2983
Cp_Diaphoretickes-Bfri_29985_OG111
3.4011
Bacteria-UP_b000006876_2549_OG111
3.5208
Bacteria-UP_b000002040_1588_OG111
3.4384
Bacteria-UP_b000033220_2955_OG111
3.6569
Osmo_Pseudofungi-Spar_2794_OG111
3.4305
Bacteria-UP_b000004664_3196_OG111
3.4884
Bacteria-UP_b000005090_1319_OG111
3.5222
3.7268
Bacteria-UP_b000008315_3296_OG111
Bacteria-UP_b000034491_506_OG111
3.7581
Cp_Diaphoretickes-Bnat_5230_OG111
Bacteria-UP_b000000265_1151_OG111
3.601
3.7581
Bacteria-UP_b000001173_48_OG111
Cp_Diaphoretickes-Gthe_17999_OG111
3.2425
Other_Diaphoretickes-Gavo_4613_OG111
3.7581
3.8051
Osmo_Labyrinthulea-Aker_93511_OG111
Osmo_Labyrinthulea-Alim_143029_OG111
2.9519
Osmo_Labyrinthulea-Hfer_3513_OG111
3.2735
Osmo_Labyrinthulea-Sagg_15523_OG111
4.0953
3.4535
Other_Diaphoretickes-Omar_30837_OG111
3.3372
Osmo_Labyrinthulea-Aker_30251_OG111
Osmo_Teretosporea-Sdes_8499_OG111
2.9321
Osmo_Teretosporea-Sdes_10062_OG111
2.9321
Osmo_Teretosporea-Sdes_13872_OG111
3.3553
Osmo_Teretosporea-Cper_7106_OG111
3.6668
3.7029
Osmo_Teretosporea-Apar_8891_OG111
Other_Holozoa-Cowc_4195_OG111

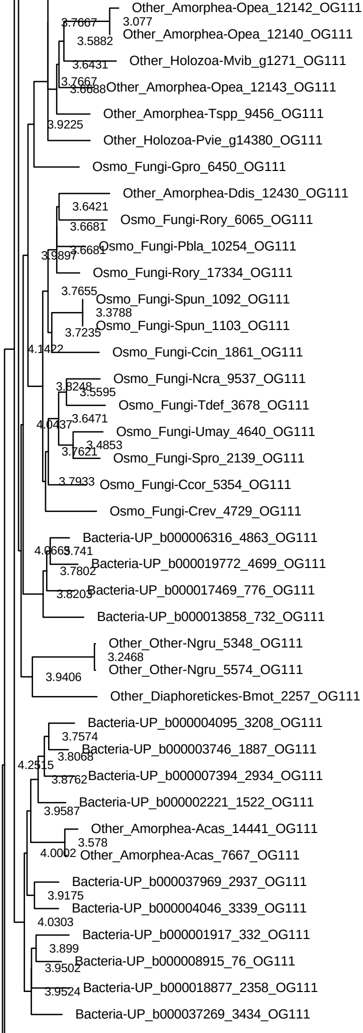

Other_Amorphea-Opea_12142_OG111
3.7667
3.077
Other_Amorphea-Opea_12140_OG111
3.5882
Other_Holozoa-Mvib_g1271_OG111
3.6431
3.7667
3.6688
Other_Amorphea-Opea_12143_OG111
Other_Amorphea-Tspp_9456_OG111
3.9225
Other_Holozoa-Pvie_g14380_OG111
Osmo_Fungi-Gpro_6450_OG111
Other_Amorphea-Ddis_12430_OG111
3.6421
Osmo_Fungi-Rory_6065_OG111
3.6681
Osmo_Fungi-Pbla_10254_OG111
3.6681
3.9897
Osmo_Fungi-Rory_17334_OG111
3.7655
Osmo_Fungi-Spun_1092_OG111
3.3788
Osmo_Fungi-Spun_1103_OG111
3.7235
4.1422
Osmo_Fungi-Ccin_1861_OG111
Osmo_Fungi-Ncra_9537_OG111
3.8248
3.5595
Osmo_Fungi-Tdef_3678_OG111
3.6471
4.0437
Osmo_Fungi-Umay_4640_OG111
3.4853
3.7621
Osmo_Fungi-Spro_2139_OG111
3.7933
Osmo_Fungi-Ccor_5354_OG111
Osmo_Fungi-Crev_4729_OG111
Bacteria-UP_b000006316_4863_OG111
4.0665
3.741
Bacteria-UP_b000019772_4699_OG111
3.7802
Bacteria-UP_b000017469_776_OG111
3.8203
Bacteria-UP_b000013858_732_OG111
Other_Other-Ngru_5348_OG111
3.2468
Other_Other-Ngru_5574_OG111
3.9406
Other_Diaphoretickes-Bmot_2257_OG111
Bacteria-UP_b000004095_3208_OG111
3.7574
Bacteria-UP_b000003746_1887_OG111
3.8068
4.2515
Bacteria-UP_b000007394_2934_OG111
3.8762
Bacteria-UP_b000002221_1522_OG111
3.9587
Other_Amorphea-Acas_14441_OG111
3.578
4.0002
Other_Amorphea-Acas_7667_OG111
Bacteria-UP_b000037969_2937_OG111
3.9175
Bacteria-UP_b000004046_3339_OG111
4.0303
Bacteria-UP_b000001917_332_OG111
3.899
Bacteria-UP_b000008915_76_OG111
3.9502
Bacteria-UP_b000018877_2358_OG111
3.9524
Bacteria-UP_b000037269_3434_OG111

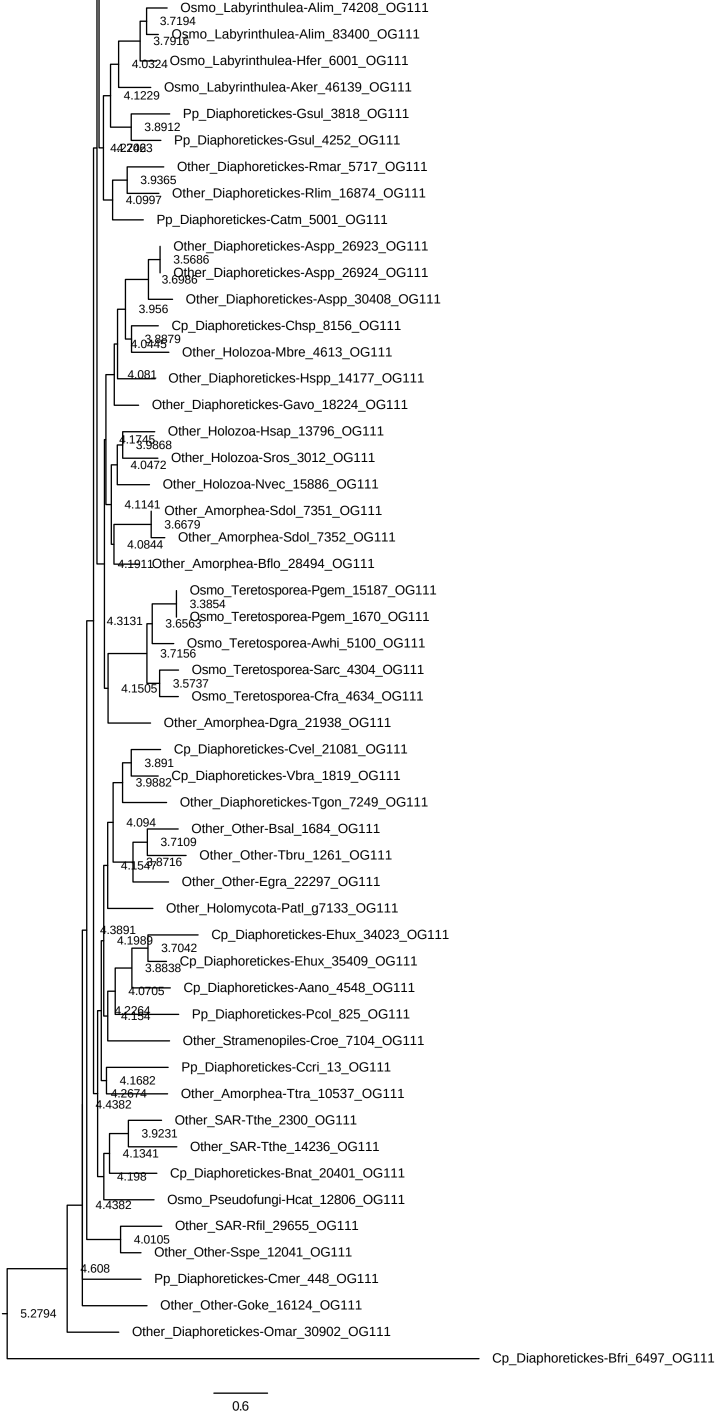
Osmo_Labyrinthulea-Alim_74208_OG111
3.7194
Osmo_Labyrinthulea-Alim_83400_OG111
3.7916
4.0324
Osmo_Labyrinthulea-Hfer_6001_OG111
4.1229
Osmo_Labyrinthulea-Aker_46139_OG111
Pp_Diaphoretickes-Gsul_3818_OG111
3.8912
Pp_Diaphoretickes-Gsul_4252_OG111
Other_Diaphoretickes-Rmar_5717_OG111
3.9365
Other_Diaphoretickes-Rlim_16874_OG111
4.0997
Pp_Diaphoretickes-Catm_5001_OG111
Other_Diaphoretickes-Aspp_26923_OG111
3.5686
Other_Diaphoretickes-Aspp_26924_OG111
3.6986
Other_Diaphoretickes-Aspp_30408_OG111
3.956
Cp_Diaphoretickes-Chsp_8156_OG111
3.8879
4.0445
Other_Holozoa-Mbre_4613_OG111
4.081
Other_Diaphoretickes-Hspp_14177_OG111
Other_Diaphoretickes-Gavo_18224_OG111
Other_Holozoa-Hsap_13796_OG111
4.1745
3.9868
Other_Holozoa-Sros_3012_OG111
4.0472
Other_Holozoa-Nvec_15886_OG111
4.1141
Other_Amorphea-Sdol_7351_OG111
3.6679
Other_Amorphea-Sdol_7352_OG111
4.0844
4.1911
Other_Amorphea-Bflo_28494_OG111
Osmo_Teretosporea-Pgem_15187_OG111
3.3854
Osmo_Teretosporea-Pgem_1670_OG111
3.6563
4.3131
Osmo_Teretosporea-Awhi_5100_OG111
3.7156
Osmo_Teretosporea-Sarc_4304_OG111
3.5737
4.1505
Osmo_Teretosporea-Cfra_4634_OG111
Other_Amorphea-Dgra_21938_OG111
Cp_Diaphoretickes-Cvel_21081_OG111
3.891
Cp_Diaphoretickes-Vbra_1819_OG111
3.9882
Other_Diaphoretickes-Tgon_7249_OG111
4.094
Other_Other-Bsal_1684_OG111
3.7109
Other_Other-Tbru_1261_OG111
4.1547
3.8716
Other_Other-Egra_22297_OG111
Other_Holomycota-Patl_g7133_OG111
4.3891
Cp_Diaphoretickes-Ehux_34023_OG111
4.1989
3.7042
Cp_Diaphoretickes-Ehux_35409_OG111
3.8838
Cp_Diaphoretickes-Aano_4548_OG111
4.0705
4.2264
4.154
Pp_Diaphoretickes-Pcol_825_OG111
Other_Stramenopiles-Croe_7104_OG111
Pp_Diaphoretickes-Ccri_13_OG111
4.1682
4.2674
Other_Amorphea-Ttra_10537_OG111
4.4382
Other_SAR-Tthe_2300_OG111
3.9231
Other_SAR-Tthe_14236_OG111
4.1341
Cp_Diaphoretickes-Bnat_20401_OG111
4.198
Osmo_Pseudofungi-Hcat_12806_OG111
4.4382
Other_SAR-Rfil_29655_OG111
4.0105
Other_Other-Sspe_12041_OG111
4.608
Pp_Diaphoretickes-Cmer_448_OG111
Other_Other-Goke_16124_OG111
5.2794
Other_Diaphoretickes-Omar_30902_OG111
Cp_Diaphoretickes-Bfri_6497_OG111
0.6

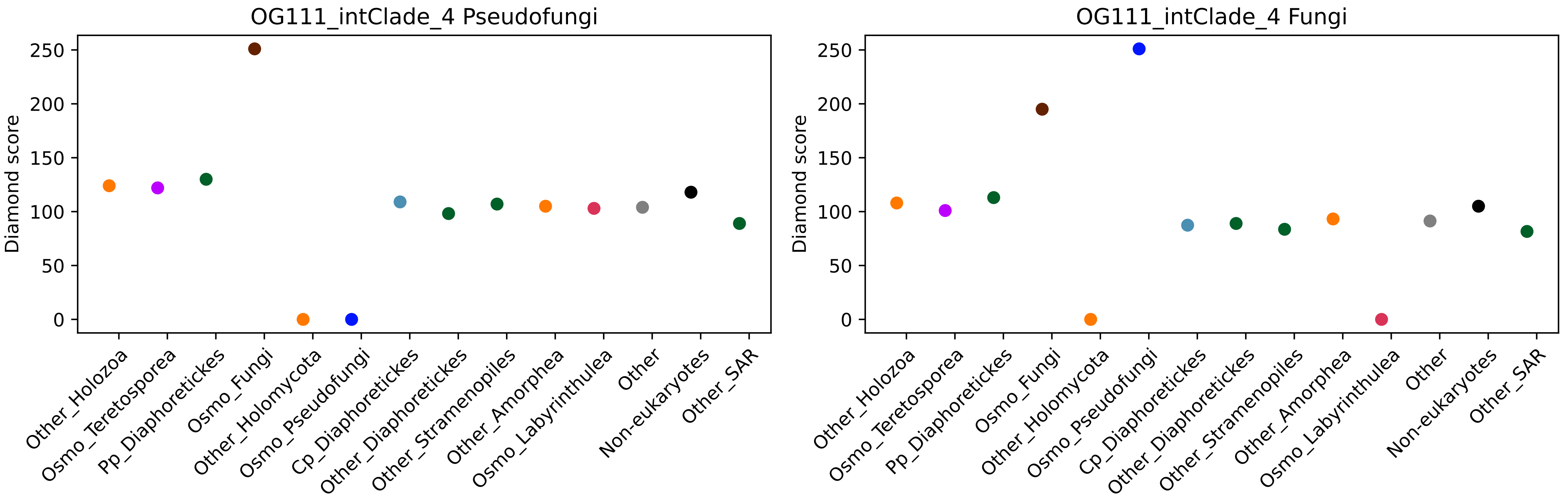

OG111_intClade_4 Pseudofungi
OG111_intClade_4 Fungi
Diamond score
0
50
100
150
200
250
Other_Holozoa
Osmo_Teretosporea
Pp_Diaphoretickes
Osmo_Fungi
Other_Holomycota
Osmo_Pseudofungi
Cp_Diaphoretickes
Other_Diaphoretickes
Other_Stramenopiles
Other_Amorphea
Osmo_Labyrinthulea
Other
Non-eukaryotes
Other_SAR

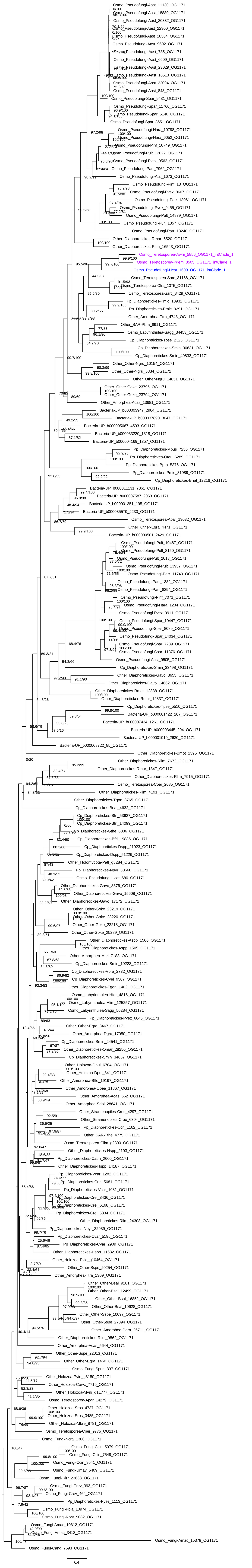

Osmo_Pseudofungi-Aast_11130_OG1171
Osmo_Pseudofungi-Aast_18880_OG1171
Osmo_Pseudofungi-Aast_20332_OG1171
Osmo_Pseudofungi-Aast_22300_OG1171
Osmo_Pseudofungi-Aast_20584_OG1171
Osmo_Pseudofungi-Aast_9602_OG1171
Osmo_Pseudofungi-Aast_735_OG1171
Osmo_Pseudofungi-Aast_6609_OG1171
Osmo_Pseudofungi-Aast_23029_OG1171
Osmo_Pseudofungi-Aast_16513_OG1171
Osmo_Pseudofungi-Aast_22094_OG1171
Osmo_Pseudofungi-Aast_848_OG1171
Osmo_Pseudofungi-Spar_9431_OG1171
Osmo_Pseudofungi-Spar_11760_OG1171
Osmo_Pseudofungi-Spar_5146_OG1171
Osmo_Pseudofungi-Spar_3651_OG1171
Osmo_Pseudofungi-Hara_10798_OG1171
Osmo_Pseudofungi-Hara_6052_OG1171
Osmo_Pseudofungi-Pinf_10749_OG1171
Osmo_Pseudofungi-Pult_12022_OG1171
Osmo_Pseudofungi-Pvex_9562_OG1171
Osmo_Pseudofungi-Parr_7962_OG1171
Osmo_Pseudofungi-Alai_1673_OG1171
Osmo_Pseudofungi-Pinf_18_OG1171
Osmo_Pseudofungi-Pvex_8607_OG1171
Osmo_Pseudofungi-Parr_13061_OG1171
Osmo_Pseudofungi-Pvex_9455_OG1171
Osmo_Pseudofungi-Pult_14839_OG1171
Osmo_Pseudofungi-Pult_1357_OG1171
Osmo_Pseudofungi-Parr_13240_OG1171
Other_Diaphoretickes-Rmar_6520_OG1171
Other_Diaphoretickes-Rlim_16543_OG1171
Osmo_Teretosporea-Awhi_5856_OG1171_intClade_1
Osmo_Teretosporea-Pgem_8505_OG1171_intClade_1
Osmo_Pseudofungi-Hcat_1609_OG1171_intClade_1
Osmo_Teretosporea-Sarc_31166_OG1171
Osmo_Teretosporea-Cfra_1075_OG1171
Osmo_Teretosporea-Sarc_8429_OG1171
Pp_Diaphoretickes-Pmic_18931_OG1171
Pp_Diaphoretickes-Pmic_9291_OG1171
Other_Amorphea-Ttra_4743_OG1171
Other_SAR-Pbra_8911_OG1171
Osmo_Labyrinthulea-Sagg_34453_OG1171
Cp_Diaphoretickes-Tpse_2325_OG1171
Cp_Diaphoretickes-Smin_30631_OG1171
Cp_Diaphoretickes-Smin_40833_OG1171
Other_Other-Ngru_10154_OG1171
Other_Other-Ngru_5834_OG1171
Other_Other-Ngru_14851_OG1171
Other_Other-Goke_23795_OG1171
Other_Other-Goke_23794_OG1171
Other_Amorphea-Acas_13681_OG1171
Bacteria-UP_b000003947_2964_OG1171
Bacteria-UP_b000037890_3647_OG1171
Bacteria-UP_b000005667_4593_OG1171
Bacteria-UP_b000033220_1318_OG1171
Bacteria-UP_b000004169_1357_OG1171
Pp_Diaphoretickes-Mpus_7256_OG1171
Pp_Diaphoretickes-Otau_6289_OG1171
Pp_Diaphoretickes-Bpra_5376_OG1171
Pp_Diaphoretickes-Pmic_31989_OG1171
Cp_Diaphoretickes-Bnat_12216_OG1171
Bacteria-UP_b000011131_7061_OG1171
Bacteria-UP_b000057587_2063_OG1171
Bacteria-UP_b000001351_195_OG1171
Bacteria-UP_b000035579_2230_OG1171
Osmo_Teretosporea-Apar_13032_OG1171
Other_Other-Egra_4471_OG1171
Bacteria-UP_b000000501_2429_OG1171
Osmo_Pseudofungi-Pult_10467_OG1171
Osmo_Pseudofungi-Pult_8150_OG1171
Osmo_Pseudofungi-Pult_2018_OG1171
Osmo_Pseudofungi-Pult_13957_OG1171
Osmo_Pseudofungi-Parr_11740_OG1171
Osmo_Pseudofungi-Parr_1382_OG1171
Osmo_Pseudofungi-Parr_8294_OG1171
Osmo_Pseudofungi-Pinf_7071_OG1171
Osmo_Pseudofungi-Hara_1234_OG1171
Osmo_Pseudofungi-Pvex_9911_OG1171
Osmo_Pseudofungi-Spar_10447_OG1171
Osmo_Pseudofungi-Spar_8089_OG1171
Osmo_Pseudofungi-Spar_14034_OG1171
Osmo_Pseudofungi-Spar_7289_OG1171
Osmo_Pseudofungi-Spar_11376_OG1171
Osmo_Pseudofungi-Aast_9505_OG1171
Cp_Diaphoretickes-Smin_33498_OG1171
Other_Diaphoretickes-Gavo_3655_OG1171
Other_Diaphoretickes-Gavo_14662_OG1171
Other_Diaphoretickes-Rmar_12838_OG1171
Other_Diaphoretickes-Rmar_12837_OG1171
Cp_Diaphoretickes-Tpse_5510_OG1171
Bacteria-UP_b000001422_207_OG1171
Bacteria-UP_b000007434_1261_OG1171
Bacteria-UP_b000003445_204_OG1171
Bacteria-UP_b000001919_2630_OG1171
Bacteria-UP_b000008722_85_OG1171
Other_Diaphoretickes-Bmot_1395_OG1171
Other_Diaphoretickes-Rlim_7672_OG1171
Other_Diaphoretickes-Rmar_1347_OG1171
Other_Diaphoretickes-Rlim_7915_OG1171
Osmo_Teretosporea-Cper_2085_OG1171
Other_Diaphoretickes-Rlim_4191_OG1171
Other_Diaphoretickes-Tgon_3765_OG1171
Cp_Diaphoretickes-Bnat_4632_OG1171
Cp_Diaphoretickes-Bfri_53627_OG1171
Cp_Diaphoretickes-Bfri_14099_OG1171
Cp_Diaphoretickes-Gthe_6006_OG1171
Cp_Diaphoretickes-Bfri_19885_OG1171
Cp_Diaphoretickes-Dspp_21023_OG1171
Cp_Diaphoretickes-Dspp_51226_OG1171
Other_Holomycota-Patl_g8284_OG1171
Pp_Diaphoretickes-Npyr_30660_OG1171
Osmo_Pseudofungi-Hcat_680_OG1171
Other_Diaphoretickes-Gavo_8376_OG1171
Other_Diaphoretickes-Gavo_15608_OG1171
Other_Diaphoretickes-Gavo_17172_OG1171
Other_Other-Goke_23219_OG1171
Other_Other-Goke_23220_OG1171
Other_Other-Goke_23218_OG1171
Other_Other-Goke_25289_OG1171
Other_Diaphoretickes-Aspp_1506_OG1171
Other_Diaphoretickes-Aspp_1505_OG1171
Other_Amorphea-Mlei_7188_OG1171
Cp_Diaphoretickes-Smin_19223_OG1171
Cp_Diaphoretickes-Vbra_2732_OG1171
Cp_Diaphoretickes-Cvel_9507_OG1171
Other_Diaphoretickes-Tgon_1402_OG1171
Osmo_Labyrinthulea-Hfer_4815_OG1171
Osmo_Labyrinthulea-Alim_125257_OG1171
Osmo_Labyrinthulea-Sagg_56284_OG1171
Pp_Diaphoretickes-Pyez_6645_OG1171
Other_Other-Egra_3467_OG1171
Other_Amorphea-Dgra_17950_OG1171
Cp_Diaphoretickes-Smin_24541_OG1171
Other_Diaphoretickes-Omar_28250_OG1171
Cp_Diaphoretickes-Smin_34657_OG1171
Other_Holozoa-Opul_6704_OG1171
Other_Holozoa-Opul_841_OG1171
Other_Amorphea-Bflo_19197_OG1171
Other_Amorphea-Opea_11867_OG1171
Other_Amorphea-Acas_662_OG1171
Other_Amorphea-Sdol_28641_OG1171
Other_Stramenopiles-Croe_4297_OG1171
Other_Stramenopiles-Croe_6304_OG1171
Other_Diaphoretickes-Ccri_1162_OG1171
Other_SAR-Tthe_4775_OG1171
Osmo_Teretosporea-Clim_g2390_OG1171
Other_Diaphoretickes-Hspp_2193_OG1171
Other_Diaphoretickes-Hspp_14187_OG1171
Pp_Diaphoretickes-Catm_2660_OG1171
Pp_Diaphoretickes-Ppyr_1855_OG1171
Pp_Diaphoretickes-Vcar_1282_OG1171
Pp_Diaphoretickes-Crei_5681_OG1171
Pp_Diaphoretickes-Vcar_1081_OG1171
Pp_Diaphoretickes-Crei_3436_OG1171
Pp_Diaphoretickes-Crei_6168_OG1171
Pp_Diaphoretickes-Crei_5334_OG1171
Other_Diaphoretickes-Rlim_24308_OG1171
Pp_Diaphoretickes-Npyr_22939_OG1171
Pp_Diaphoretickes-Cvar_5195_OG1171
Pp_Diaphoretickes-Cvar_2909_OG1171
Other_Diaphoretickes-Hspp_11682_OG1171
Other_Holozoa-Pvie_g10464_OG1171
Other_Other-Sspe_20254_OG1171
Other_Amorphea-Ttra_1309_OG1171
Other_Other-Bsal_9281_OG1171
Other_Other-Bsal_12499_OG1171
Other_Other-Bsal_16852_OG1171
Other_Other-Bsal_10628_OG1171
Other_Other-Sspe_10097_OG1171
Other_Other-Sspe_27394_OG1171
Other_Amorphea-Dgra_26711_OG1171
Other_Diaphoretickes-Rlim_9862_OG1171
Other_Amorphea-Acas_5644_OG1171
Other_Other-Sspe_22013_OG1171
Other_Other-Egra_1460_OG1171
Osmo_Fungi-Spun_837_OG1171
Other_Holozoa-Pvie_g8180_OG1171
Other_Holozoa-Cowc_7719_OG1171
Other_Holozoa-Mvib_g11777_OG1171
Osmo_Teretosporea-Apar_14279_OG1171
Other_Holozoa-Sros_4737_OG1171
Other_Holozoa-Sros_3485_OG1171
Other_Holozoa-Mbre_8781_OG1171
Osmo_Teretosporea-Cper_9775_OG1171
Osmo_Fungi-Ncra_1306_OG1171
Osmo_Fungi-Ccin_5079_OG1171
Osmo_Fungi-Ccin_7549_OG1171
Osmo_Fungi-Ccin_9541_OG1171
Osmo_Fungi-Umay_5409_OG1171
Osmo_Fungi-Rirr_23638_OG1171
Osmo_Fungi-Crev_393_OG1171
Osmo_Fungi-Crev_464_OG1171
Pp_Diaphoretickes-Pyez_1113_OG1171
Osmo_Fungi-Pbla_10974_OG1171
Osmo_Fungi-Rory_9082_OG1171
Osmo_Fungi-Amac_10812_OG1171
Osmo_Fungi-Amac_3413_OG1171
Osmo_Fungi-Amac_15379_OG1171
Osmo_Fungi-Cang_7693_OG1171
0.4

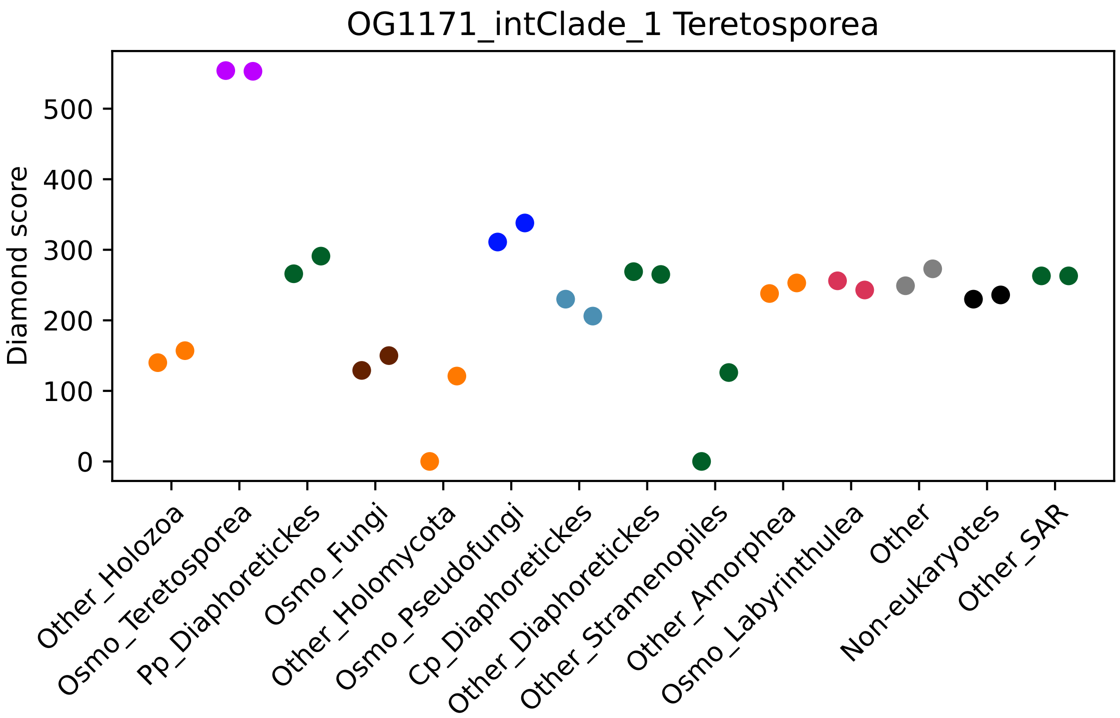
OG1171_intClade_1 Teretosporea
Diamond score
0
100
200
300
400
500
Other_Holozoa
Osmo_Teretosporea
Pp_Diaphoretickes
Osmo_Fungi
Other_Holomycota
Osmo_Pseudofungi
Cp_Diaphoretickes
Other_Diaphoretickes
Other_Stramenopiles
Other_Amorphea
Osmo_Labyrinthulea
Other
Non-eukaryotes
Other_SAR

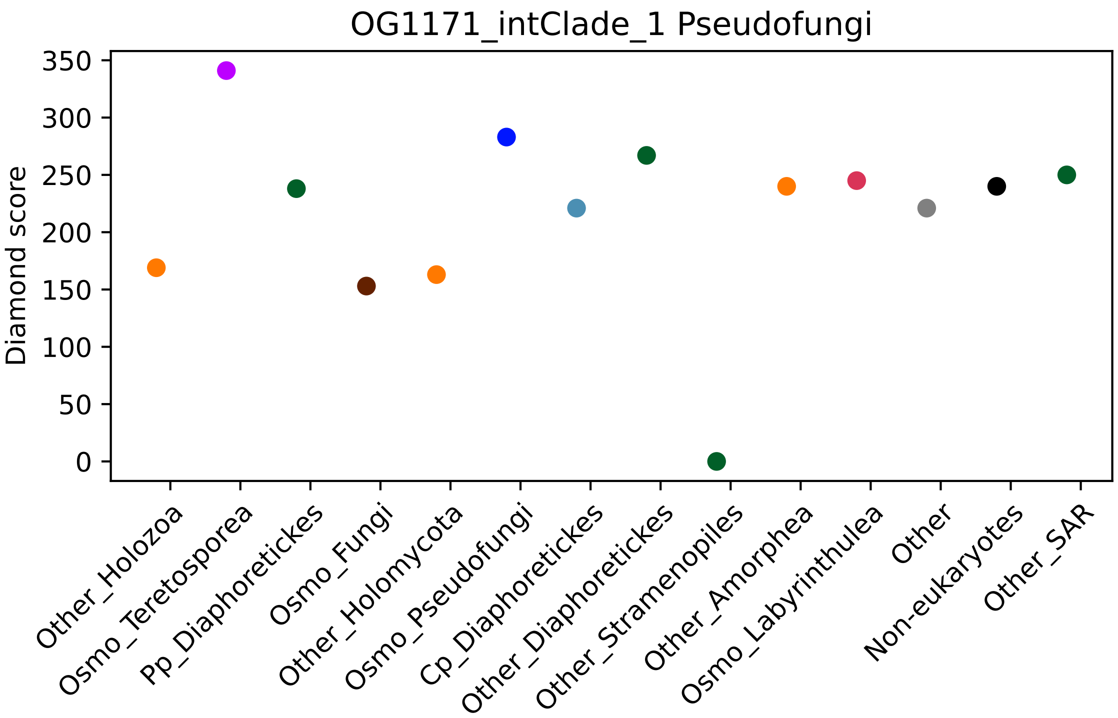
OG1171_intClade_1 Pseudofungi
Diamond score
0
50
100
150
200
250
300
350
Other_Holozoa
Osmo_Teretosporea
Pp_Diaphoretickes
Osmo_Fungi
Other_Holomycota
Osmo_Pseudofungi
Cp_Diaphoretickes
Other_Diaphoretickes
Other_Stramenopiles
Other_Amorphea
Osmo_Labyrinthulea
Other
Non-eukaryotes
Other_SAR

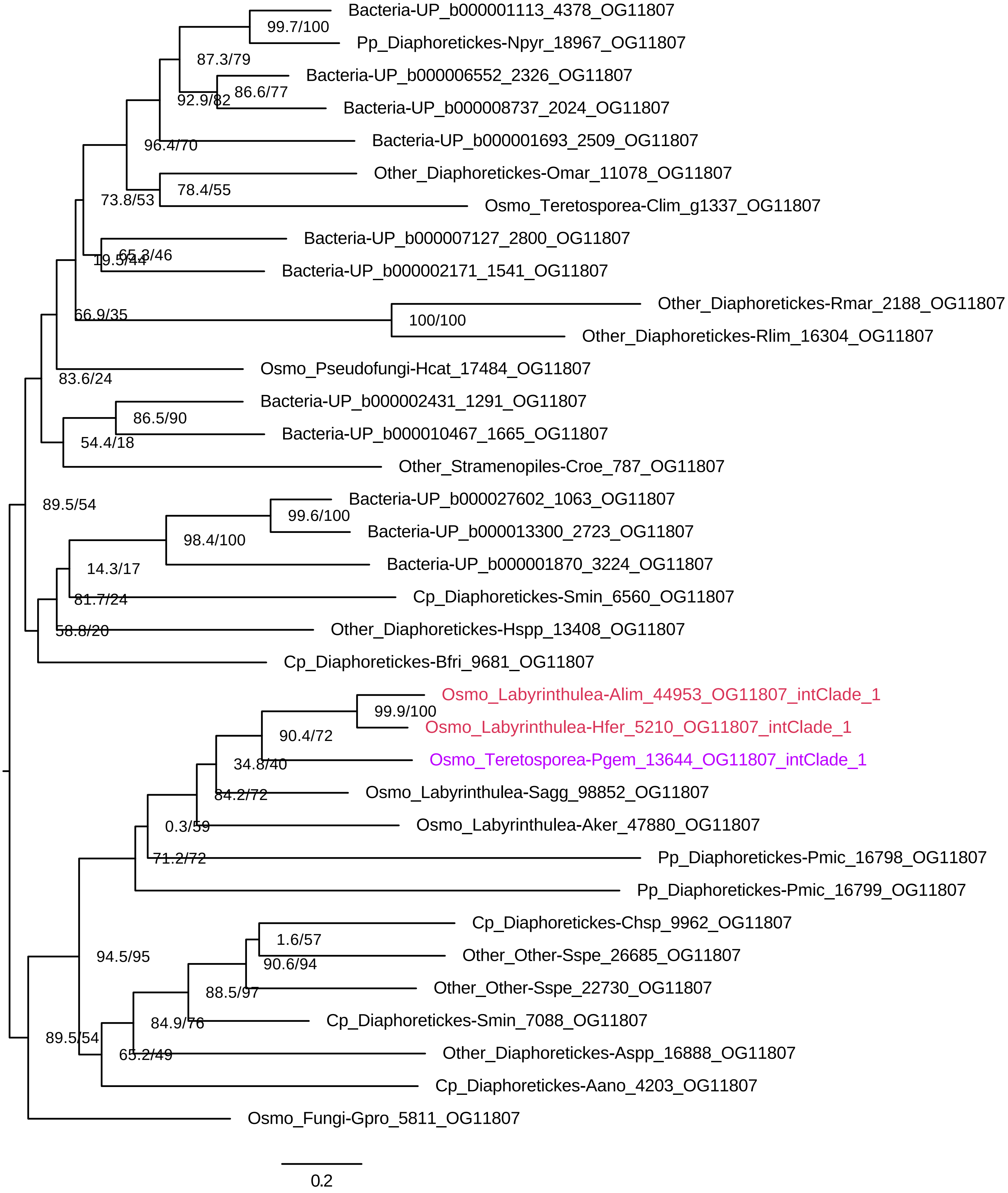
Bacteria-UP_b000001113_4378_OG11807
99.7/100
Pp_Diaphoretickes-Npyr_18967_OG11807
87.3/79
Bacteria-UP_b000006552_2326_OG11807
92.9/82
86.6/77
Bacteria-UP_b000008737_2024_OG11807
Bacteria-UP_b000001693_2509_OG11807
96.4/70
Other_Diaphoretickes-Omar_11078_OG11807
78.4/55
73.8/53
Osmo_Teretosporea-Clim_g1337_OG11807
Bacteria-UP_b000007127_2800_OG11807
65.3/46
19.5/44
Bacteria-UP_b000002171_1541_OG11807
Other_Diaphoretickes-Rmar_2188_OG11807
66.9/35
100/100
Other_Diaphoretickes-Rlim_16304_OG11807
Osmo_Pseudofungi-Hcat_17484_OG11807
83.6/24
Bacteria-UP_b000002431_1291_OG11807
86.5/90
54.4/18
Bacteria-UP_b000010467_1665_OG11807
Other_Stramenopiles-Croe_787_OG11807
89.5/54
Bacteria-UP_b000027602_1063_OG11807
99.6/100
Bacteria-UP_b000013300_2723_OG11807
98.4/100
Bacteria-UP_b000001870_3224_OG11807
14.3/17
81.7/24
Cp_Diaphoretickes-Smin_6560_OG11807
58.8/20
Other_Diaphoretickes-Hspp_13408_OG11807
Cp_Diaphoretickes-Bfri_9681_OG11807
Osmo_Labyrinthulea-Alim_44953_OG11807_intClade_1
99.9/100
Osmo_Labyrinthulea-Hfer_5210_OG11807_intClade_1
90.4/72
Osmo_Teretosporea-Pgem_13644_OG11807_intClade_1
34.8/40
84.2/72
Osmo_Labyrinthulea-Sagg_98852_OG11807
0.3/59
Osmo_Labyrinthulea-Aker_47880_OG11807
71.2/72
Pp_Diaphoretickes-Pmic_16798_OG11807
Pp_Diaphoretickes-Pmic_16799_OG11807
Cp_Diaphoretickes-Chsp_9962_OG11807
1.6/57
94.5/95
Other_Other-Sspe_26685_OG11807
90.6/94
Other_Other-Sspe_22730_OG11807
88.5/97
84.9/76
Cp_Diaphoretickes-Smin_7088_OG11807
89.5/54
65.2/49
Other_Diaphoretickes-Aspp_16888_OG11807
Cp_Diaphoretickes-Aano_4203_OG11807
Osmo_Fungi-Gpro_5811_OG11807
0.2

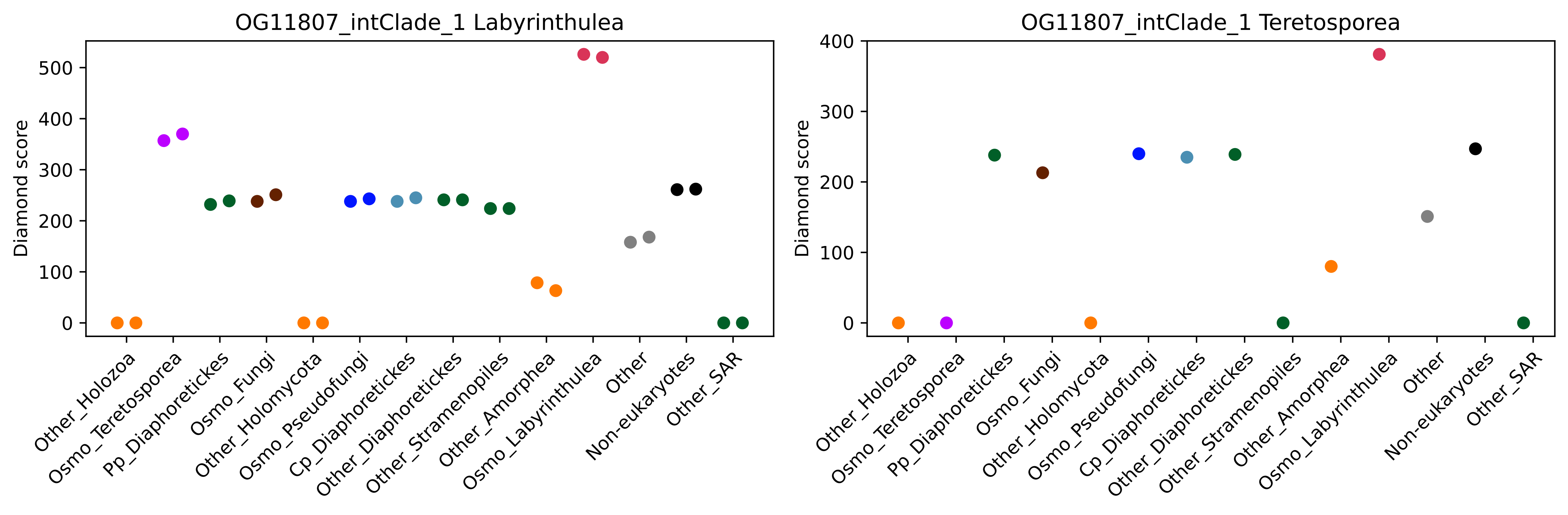

OG11807_intClade_1 Labyrinthulea
Diamond score
0
100
200
300
400
500
Other_Holozoa
Osmo_Teretosporea
Pp_Diaphoretickes
Osmo_Fungi
Other_Holomycota
Osmo_Pseudofungi
Cp_Diaphoretickes
Other_Diaphoretickes
Other_Stramenopiles
Other_Amorphea
Osmo_Labyrinthulea
Other
Non-eukaryotes
Other_SAR
OG11807_intClade_1 Teretosporea
Diamond score
0
100
200
300
400
Other_Holozoa
Osmo_Teretosporea
Pp_Diaphoretickes
Osmo_Fungi
Other_Holomycota
Osmo_Pseudofungi
Cp_Diaphoretickes
Other_Diaphoretickes
Other_Stramenopiles
Other_Amorphea
Osmo_Labyrinthulea
Other
Non-eukaryotes
Other_SAR

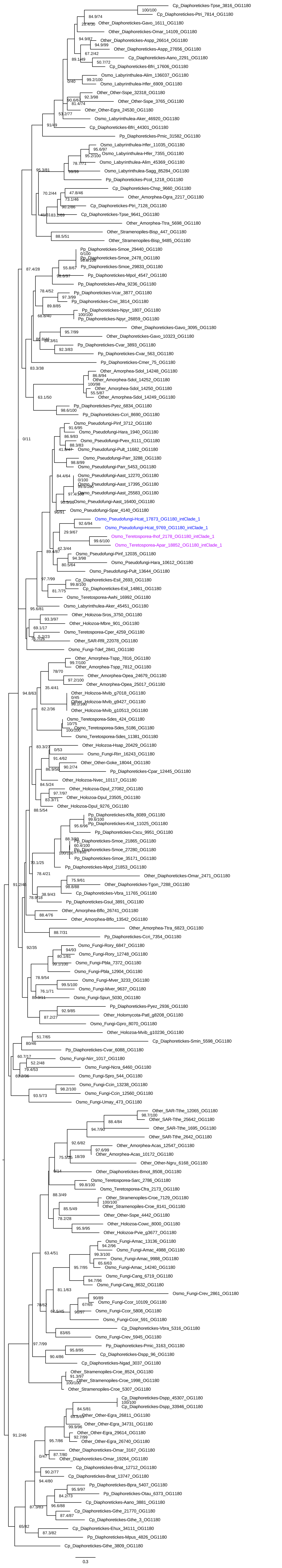

Cp_Diaphoretickes-Tpse_3816_OG1180
Cp_Diaphoretickes-Ptri_7814_OG1180
Other_Diaphoretickes-Gavo_1611_OG1180
Other_Diaphoretickes-Omar_14109_OG1180
Other_Diaphoretickes-Aspp_26614_OG1180
Other_Diaphoretickes-Aspp_27656_OG1180
Cp_Diaphoretickes-Aano_2291_OG1180
Cp_Diaphoretickes-Bfri_17606_OG1180
Osmo_Labyrinthulea-Alim_136037_OG1180
Osmo_Labyrinthulea-Hfer_6909_OG1180
Other_Other-Sspe_32318_OG1180
Other_Other-Sspe_3765_OG1180
Other_Other-Egra_24530_OG1180
Osmo_Labyrinthulea-Aker_46920_OG1180
Cp_Diaphoretickes-Bfri_44301_OG1180
Pp_Diaphoretickes-Pmic_31582_OG1180
Osmo_Labyrinthulea-Hfer_11035_OG1180
Osmo_Labyrinthulea-Hfer_7355_OG1180
Osmo_Labyrinthulea-Alim_45369_OG1180
Osmo_Labyrinthulea-Sagg_85284_OG1180
Pp_Diaphoretickes-Pcol_1218_OG1180
Cp_Diaphoretickes-Chsp_9660_OG1180
Other_Amorphea-Dgra_2217_OG1180
Cp_Diaphoretickes-Ptri_7128_OG1180
Cp_Diaphoretickes-Tpse_9641_OG1180
Other_Amorphea-Ttra_5698_OG1180
Other_Stramenopiles-Bisp_447_OG1180
Other_Stramenopiles-Bisp_9485_OG1180
Pp_Diaphoretickes-Smoe_29440_OG1180
Pp_Diaphoretickes-Smoe_2478_OG1180
Pp_Diaphoretickes-Smoe_29833_OG1180
Pp_Diaphoretickes-Mpol_4547_OG1180
Pp_Diaphoretickes-Atha_9236_OG1180
Cp_Diaphoretickes-Vcar_3877_OG1180
Pp_Diaphoretickes-Crei_3814_OG1180
Pp_Diaphoretickes-Npyr_1807_OG1180
Pp_Diaphoretickes-Npyr_26859_OG1180
Other_Diaphoretickes-Gavo_3095_OG1180
Other_Diaphoretickes-Gavo_10323_OG1180
Pp_Diaphoretickes-Cvar_3893_OG1180
Pp_Diaphoretickes-Cvar_563_OG1180
Pp_Diaphoretickes-Cmer_75_OG1180
Other_Amorphea-Sdol_14248_OG1180
Other_Amorphea-Sdol_14252_OG1180
Other_Amorphea-Sdol_14250_OG1180
Other_Amorphea-Sdol_14249_OG1180
Pp_Diaphoretickes-Pyez_6834_OG1180
Pp_Diaphoretickes-Ccri_8690_OG1180
Osmo_Pseudofungi-Pinf_3712_OG1180
Osmo_Pseudofungi-Hara_1940_OG1180
Osmo_Pseudofungi-Pvex_6111_OG1180
Osmo_Pseudofungi-Pult_11682_OG1180
Osmo_Pseudofungi-Parr_3288_OG1180
Osmo_Pseudofungi-Parr_5453_OG1180
Osmo_Pseudofungi-Aast_12270_OG1180
Osmo_Pseudofungi-Aast_17395_OG1180
Osmo_Pseudofungi-Aast_25583_OG1180
Osmo_Pseudofungi-Aast_16400_OG1180
Osmo_Pseudofungi-Spar_4140_OG1180
Osmo_Pseudofungi-Hcat_17873_OG1180_intClade_1
Osmo_Pseudofungi-Hcat_9769_OG1180_intClade_1
Osmo_Teretosporea-Ihof_2178_OG1180_intClade_1
Osmo_Teretosporea-Apar_18852_OG1180_intClade_1
Osmo_Pseudofungi-Pinf_12035_OG1180
Osmo_Pseudofungi-Hara_10612_OG1180
Osmo_Pseudofungi-Pult_13644_OG1180
Cp_Diaphoretickes-Esil_2693_OG1180
Cp_Diaphoretickes-Esil_14861_OG1180
Osmo_Teretosporea-Awhi_16992_OG1180
Osmo_Labyrinthulea-Aker_45451_OG1180
Other_Holozoa-Sros_3750_OG1180
Other_Holozoa-Mbre_901_OG1180
Osmo_Teretosporea-Cper_4259_OG1180
Other_SAR-Rfil_22078_OG1180
Osmo_Fungi-Tdef_2841_OG1180
Other_Amorphea-Tspp_7816_OG1180
Other_Amorphea-Tspp_7812_OG1180
Other_Amorphea-Opea_24679_OG1180
Other_Amorphea-Opea_25017_OG1180
Other_Holozoa-Mvib_g7018_OG1180
Other_Holozoa-Mvib_g9427_OG1180
Other_Holozoa-Mvib_g10513_OG1180
Osmo_Teretosporea-Sdes_424_OG1180
Osmo_Teretosporea-Sdes_5186_OG1180
Osmo_Teretosporea-Sdes_11381_OG1180
Other_Holozoa-Hsap_20429_OG1180
Osmo_Fungi-Rirr_16243_OG1180
Other_Other-Goke_18044_OG1180
Pp_Diaphoretickes-Cpar_12445_OG1180
Other_Holozoa-Nvec_10117_OG1180
Other_Holozoa-Dpul_27082_OG1180
Other_Holozoa-Dpul_23505_OG1180
Other_Holozoa-Dpul_9276_OG1180
Pp_Diaphoretickes-Kfla_8089_OG1180
Pp_Diaphoretickes-Knit_11025_OG1180
Pp_Diaphoretickes-Cscu_9951_OG1180
Pp_Diaphoretickes-Smoe_21865_OG1180
Pp_Diaphoretickes-Smoe_27280_OG1180
Pp_Diaphoretickes-Smoe_35171_OG1180
Pp_Diaphoretickes-Mpol_21853_OG1180
Other_Diaphoretickes-Omar_2471_OG1180
Other_Diaphoretickes-Tgon_7288_OG1180
Cp_Diaphoretickes-Vbra_11765_OG1180
Pp_Diaphoretickes-Gsul_3891_OG1180
Other_Amorphea-Bflo_26741_OG1180
Other_Amorphea-Bflo_13542_OG1180
Other_Amorphea-Ttra_6823_OG1180
Pp_Diaphoretickes-Ccri_7354_OG1180
Osmo_Fungi-Rory_6847_OG1180
Osmo_Fungi-Rory_12748_OG1180
Osmo_Fungi-Pbla_7372_OG1180
Osmo_Fungi-Pbla_12904_OG1180
Osmo_Fungi-Mver_3233_OG1180
Osmo_Fungi-Mver_9637_OG1180
Osmo_Fungi-Spun_5030_OG1180
Pp_Diaphoretickes-Pyez_2936_OG1180
Other_Holomycota-Patl_g8208_OG1180
Osmo_Fungi-Gpro_8070_OG1180
Other_Holozoa-Mvib_g10236_OG1180
Cp_Diaphoretickes-Smin_5598_OG1180
Pp_Diaphoretickes-Cvar_6088_OG1180
Osmo_Fungi-Nirr_1017_OG1180
Osmo_Fungi-Ncra_6460_OG1180
Osmo_Fungi-Spro_544_OG1180
Osmo_Fungi-Ccin_13238_OG1180
Osmo_Fungi-Ccin_12560_OG1180
Osmo_Fungi-Umay_473_OG1180
Other_SAR-Tthe_12065_OG1180
Other_SAR-Tthe_25642_OG1180
Other_SAR-Tthe_1695_OG1180
Other_SAR-Tthe_2642_OG1180
Other_Amorphea-Acas_12547_OG1180
Other_Amorphea-Acas_10172_OG1180
Other_Other-Ngru_6168_OG1180
Other_Diaphoretickes-Bmot_8508_OG1180
Osmo_Teretosporea-Sarc_2786_OG1180
Osmo_Teretosporea-Cfra_2173_OG1180
Other_Stramenopiles-Croe_7129_OG1180
Other_Stramenopiles-Croe_8141_OG1180
Other_Other-Sspe_4442_OG1180
Other_Holozoa-Cowc_8000_OG1180
Other_Holozoa-Pvie_g3677_OG1180
Osmo_Fungi-Amac_13136_OG1180
Osmo_Fungi-Amac_4988_OG1180
Osmo_Fungi-Amac_9988_OG1180
Osmo_Fungi-Amac_14240_OG1180
Osmo_Fungi-Cang_6719_OG1180
Osmo_Fungi-Cang_8632_OG1180
Osmo_Fungi-Crev_2861_OG1180
Osmo_Fungi-Ccor_10109_OG1180
Osmo_Fungi-Ccor_5808_OG1180
Osmo_Fungi-Ccor_591_OG1180
Cp_Diaphoretickes-Vbra_5316_OG1180
Osmo_Fungi-Crev_5945_OG1180
Pp_Diaphoretickes-Pmic_3163_OG1180
Cp_Diaphoretickes-Dspp_96_OG1180
Cp_Diaphoretickes-Ngad_3037_OG1180
Other_Stramenopiles-Croe_8524_OG1180
Other_Stramenopiles-Croe_1998_OG1180
Other_Stramenopiles-Croe_5307_OG1180
Cp_Diaphoretickes-Dspp_45307_OG1180
Cp_Diaphoretickes-Dspp_33946_OG1180
Other_Other-Egra_26811_OG1180
Other_Other-Egra_34731_OG1180
Other_Other-Egra_29614_OG1180
Other_Other-Egra_26740_OG1180
Other_Diaphoretickes-Omar_3167_OG1180
Other_Diaphoretickes-Omar_19264_OG1180
Cp_Diaphoretickes-Bnat_12712_OG1180
Cp_Diaphoretickes-Bnat_13747_OG1180
Pp_Diaphoretickes-Bpra_5407_OG1180
Pp_Diaphoretickes-Otau_6373_OG1180
Cp_Diaphoretickes-Aano_3881_OG1180
Cp_Diaphoretickes-Gthe_21770_OG1180
Cp_Diaphoretickes-Gthe_3_OG1180
Cp_Diaphoretickes-Ehux_34111_OG1180
Pp_Diaphoretickes-Mpus_4826_OG1180
Cp_Diaphoretickes-Gthe_3809_OG1180
0.3

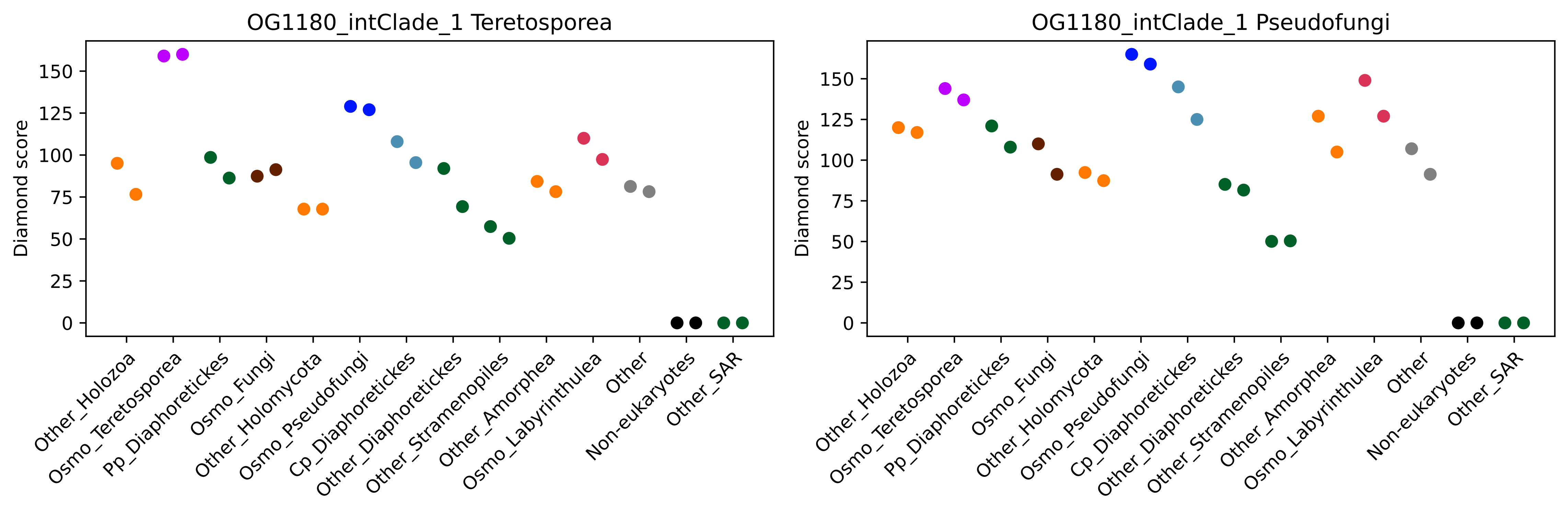
OG1180_intClade_1 Teretosporea
Diamond score
0
25
50
75
100
125
150
Other_Holozoa
Osmo_Teretosporea
Pp_Diaphoretickes
Osmo_Fungi
Other_Holomycota
Osmo_Pseudofungi
Cp_Diaphoretickes
Other_Diaphoretickes
Other_Stramenopiles
Other_Amorphea
Osmo_Labyrinthulea
Other
Non-eukaryotes
Other_SAR
OG1180_intClade_1 Pseudofungi
Diamond score
0
25
50
75
100
125
150
Other_Holozoa
Osmo_Teretosporea
Pp_Diaphoretickes
Osmo_Fungi
Other_Holomycota
Osmo_Pseudofungi
Cp_Diaphoretickes
Other_Diaphoretickes
Other_Stramenopiles
Other_Amorphea
Osmo_Labyrinthulea
Other
Non-eukaryotes
Other_SAR

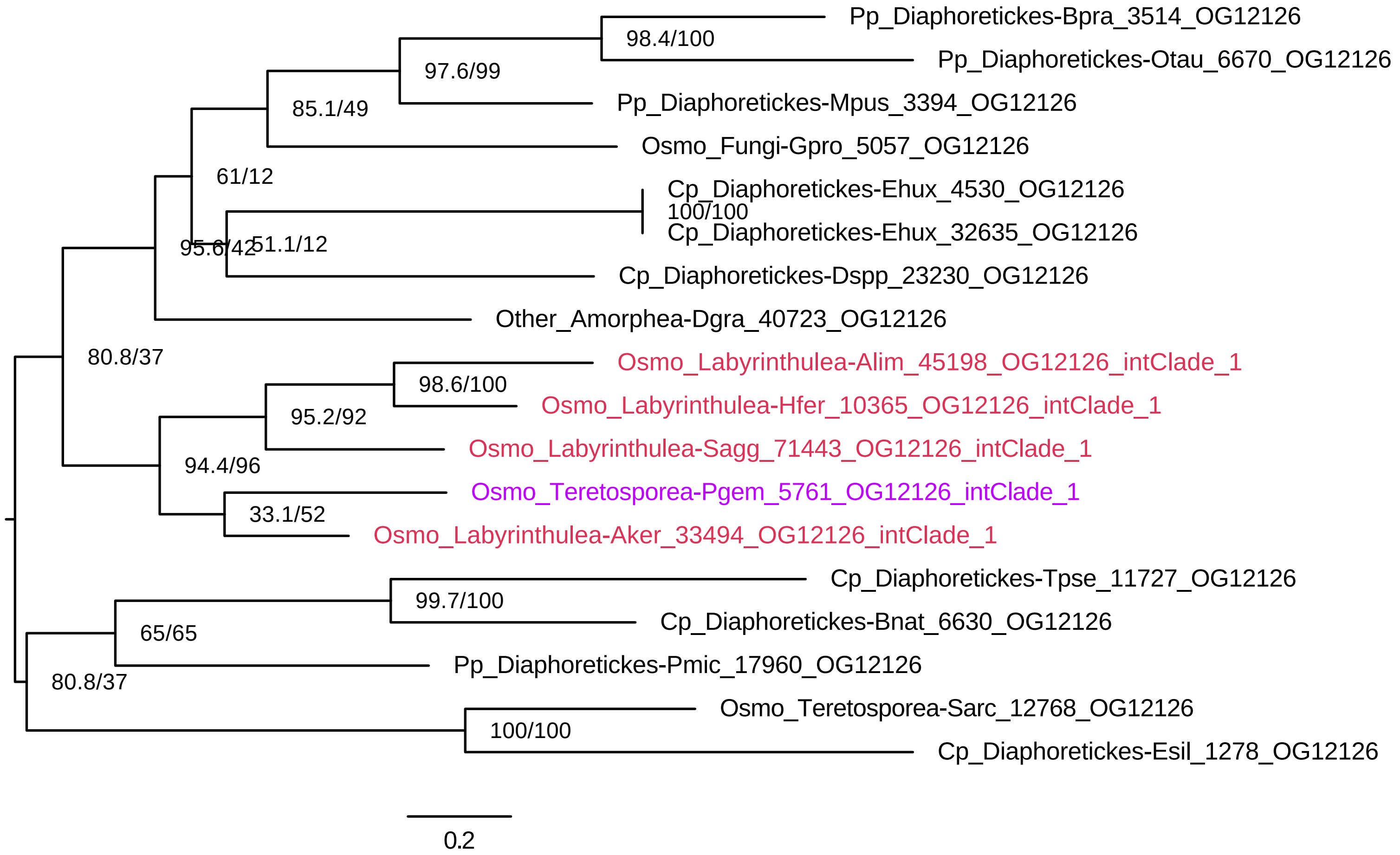
Pp_Diaphoretickes-Bpra_3514_OG12126
98.4/100
Pp_Diaphoretickes-Otau_6670_OG12126
97.6/99
Pp_Diaphoretickes-Mpus_3394_OG12126
85.1/49
Osmo_Fungi-Gpro_5057_OG12126
61/12
Cp_Diaphoretickes-Ehux_4530_OG12126
100/100
Cp_Diaphoretickes-Ehux_32635_OG12126
95.6/42
51.1/12
Cp_Diaphoretickes-Dspp_23230_OG12126
Other_Amorphea-Dgra_40723_OG12126
80.8/37
Osmo_Labyrinthulea-Alim_45198_OG12126_intClade_1
98.6/100
Osmo_Labyrinthulea-Hfer_10365_OG12126_intClade_1
95.2/92
Osmo_Labyrinthulea-Sagg_71443_OG12126_intClade_1
94.4/96
Osmo_Teretosporea-Pgem_5761_OG12126_intClade_1
33.1/52
Osmo_Labyrinthulea-Aker_33494_OG12126_intClade_1
Cp_Diaphoretickes-Tpse_11727_OG12126
99.7/100
Cp_Diaphoretickes-Bnat_6630_OG12126
65/65
Pp_Diaphoretickes-Pmic_17960_OG12126
80.8/37
Osmo_Teretosporea-Sarc_12768_OG12126
100/100
Cp_Diaphoretickes-Esil_1278_OG12126
0.2

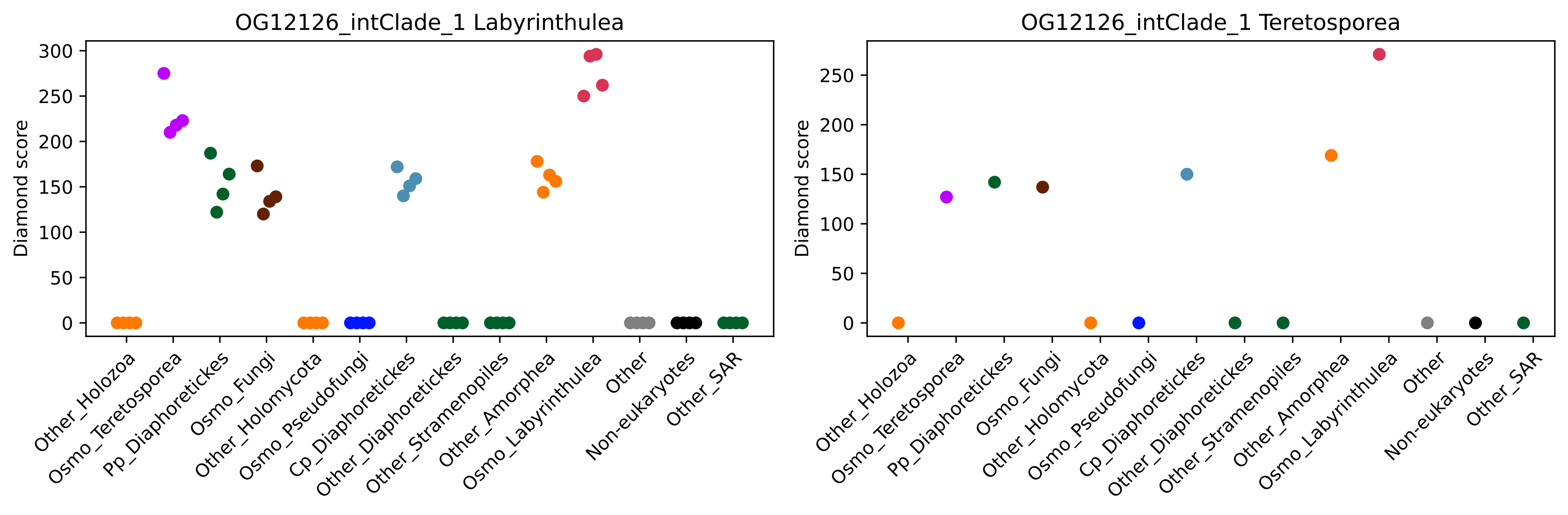

OG12126_intClade_1 Labyrinthulea
Diamond score
300
250
200
150
100
50
0
Other_Holozoa
Osmo_Teretosporea
Pp_Diaphoretickes
Osmo_Fungi
Other_Holomycota
Osmo_Pseudofungi
Cp_Diaphoretickes
Other_Diaphoretickes
Other_Stramenopiles
Other_Amorphea
Osmo_Labyrinthulea
Other
Non-eukaryotes
Other_SAR
OG12126_intClade_1 Teretosporea
Diamond score
250
200
150
100
50
0
Other_Holozoa
Osmo_Teretosporea
Pp_Diaphoretickes
Osmo_Fungi
Other_Holomycota
Osmo_Pseudofungi
Cp_Diaphoretickes
Other_Diaphoretickes
Other_Stramenopiles
Other_Amorphea
Osmo_Labyrinthulea
Other
Non-eukaryotes
Other_SAR

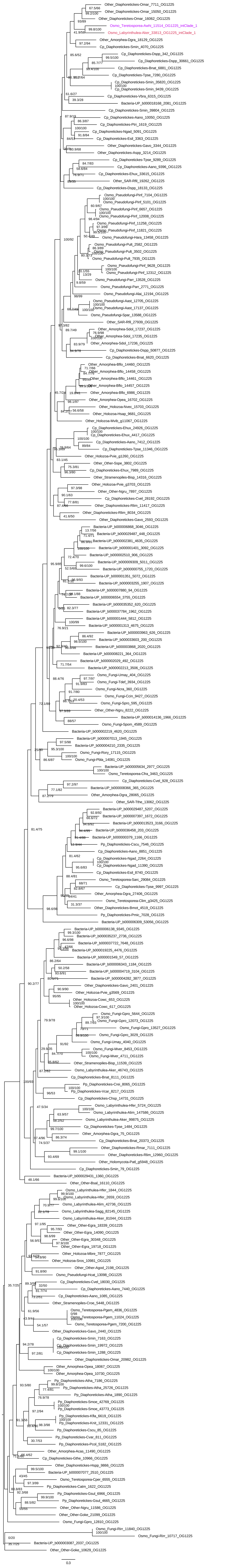

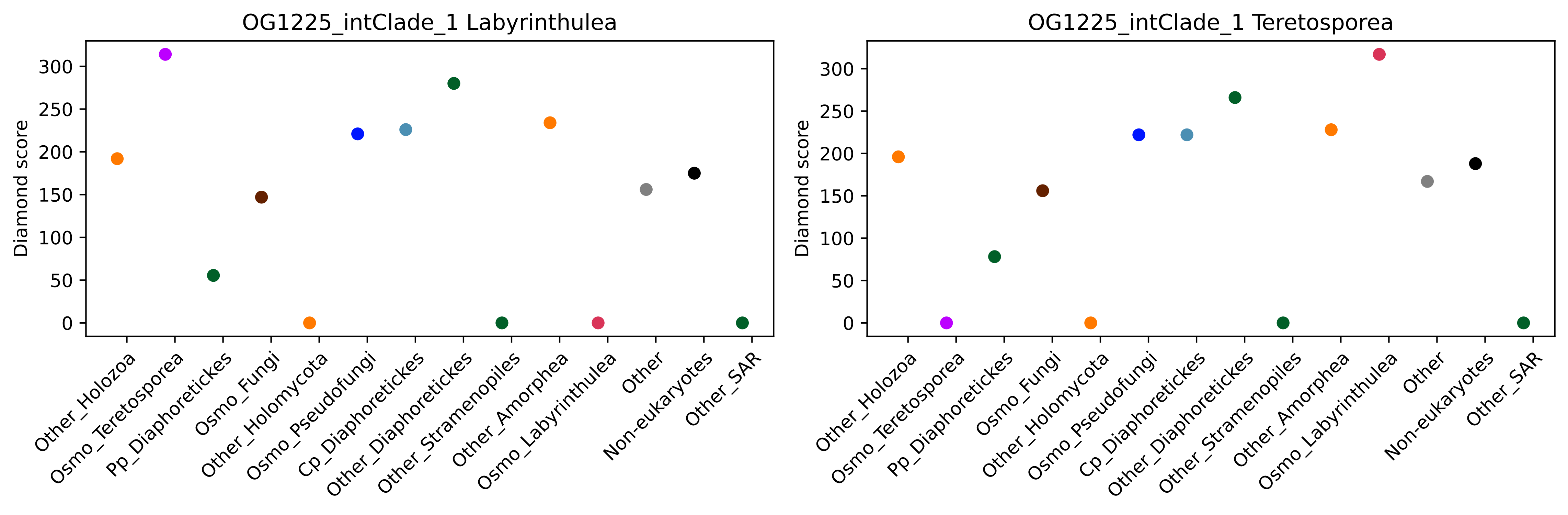

OG1225_intClade_1 Labyrinthulea
OG1225_intClade_1 Teretosporea
Diamond score
0
50
100
150
200
250
300
Other_Holozoa
Osmo_Teretosporea
Pp_Diaphoretickes
Osmo_Fungi
Other_Holomycota
Osmo_Pseudofungi
Cp_Diaphoretickes
Other_Diaphoretickes
Other_Stramenopiles
Other_Amorphea
Osmo_Labyrinthulea
Other
Non-eukaryotes
Other_SAR

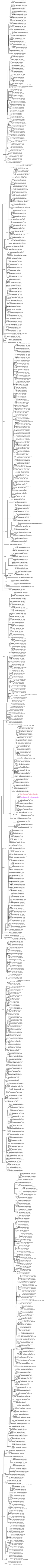

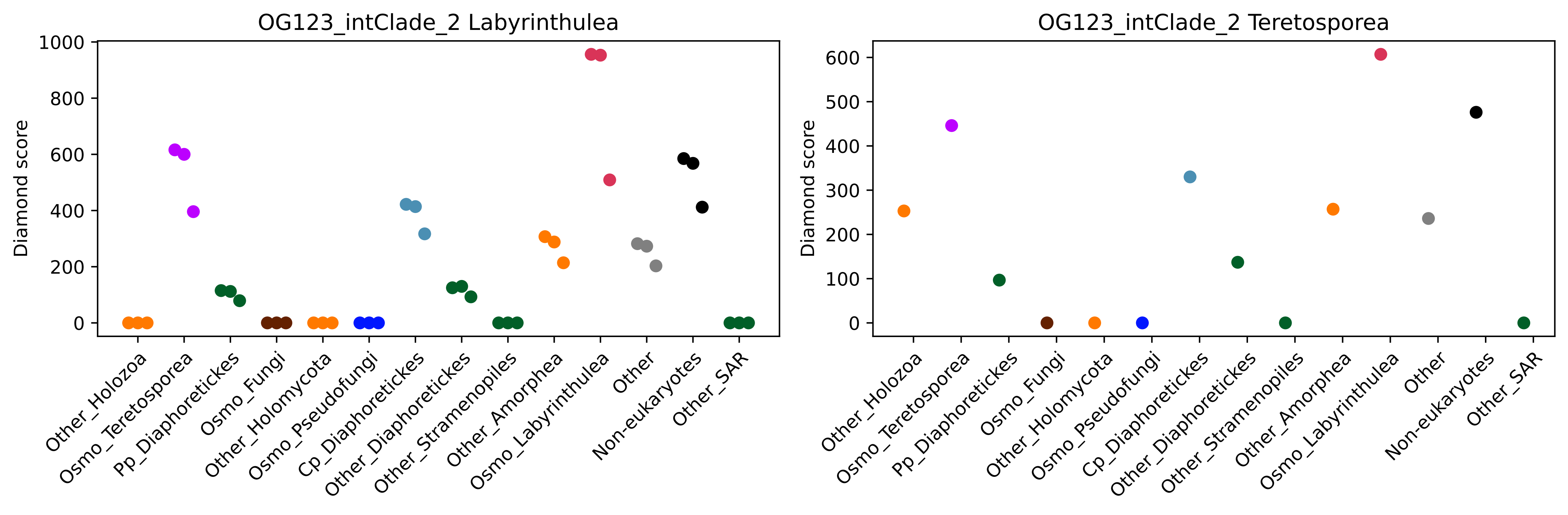

OG123_intClade_2 Labyrinthulea
OG123_intClade_2 Teretosporea
Diamond score
Diamond score
0
200
400
600
800
1000
0
100
200
300
400
500
600
Other_Holozoa
Osmo_Teretosporea
Pp_Diaphoretickes
Osmo_Fungi
Other_Holomycota
Osmo_Pseudofungi
Cp_Diaphoretickes
Other_Diaphoretickes
Other_Stramenopiles
Other_Amorphea
Osmo_Labyrinthulea
Other
Non-eukaryotes
Other_SAR

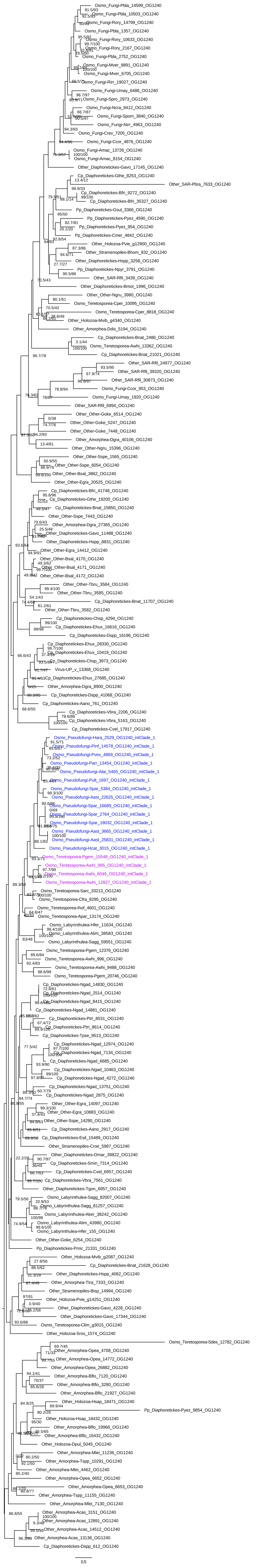

Osmo_Fungi-Pbla_14599_OG1240
Osmo_Fungi-Pbla_10503_OG1240
Osmo_Fungi-Rory_14799_OG1240
Osmo_Fungi-Pbla_1357_OG1240
Osmo_Fungi-Rory_10633_OG1240
Osmo_Fungi-Rory_2167_OG1240
Osmo_Fungi-Pbla_2752_OG1240
Osmo_Fungi-Mver_9891_OG1240
Osmo_Fungi-Mver_6705_OG1240
Osmo_Fungi-Rirr_19027_OG1240
Osmo_Fungi-Umay_6486_OG1240
Osmo_Fungi-Spro_2973_OG1240
Osmo_Fungi-Ncra_9412_OG1240
Osmo_Fungi-Spom_3840_OG1240
Osmo_Fungi-Nirr_4963_OG1240
Osmo_Fungi-Crev_7205_OG1240
Osmo_Fungi-Ccor_4876_OG1240
Osmo_Fungi-Amac_13726_OG1240
Osmo_Fungi-Amac_8154_OG1240
Other_Diaphoretickes-Gavo_17145_OG1240
Cp_Diaphoretickes-Gthe_8253_OG1240
Other_SAR-Pbra_7633_OG1240
Cp_Diaphoretickes-Bfri_9272_OG1240
Cp_Diaphoretickes-Bfri_35327_OG1240
Pp_Diaphoretickes-Gsul_3366_OG1240
Pp_Diaphoretickes-Pyez_4590_OG1240
Pp_Diaphoretickes-Pyez_954_OG1240
Pp_Diaphoretickes-Cmer_4842_OG1240
Other_Holozoa-Pvie_g12900_OG1240
Other_Stramenopiles-Bhom_932_OG1240
Other_Diaphoretickes-Hspp_3258_OG1240
Pp_Diaphoretickes-Npyr_3791_OG1240
Other_SAR-Rfil_3439_OG1240
Other_Diaphoretickes-Bmot_1996_OG1240
Other_Other-Ngru_3980_OG1240
Osmo_Teretosporea-Cper_10095_OG1240
Osmo_Teretosporea-Cper_8818_OG1240
Other_Holozoa-Mvib_g4340_OG1240
Other_Amorphea-Ddis_5194_OG1240
Cp_Diaphoretickes-Bnat_2486_OG1240
Osmo_Teretosporea-Awhi_13362_OG1240
Cp_Diaphoretickes-Bnat_21021_OG1240
Other_SAR-Rfil_24977_OG1240
Other_SAR-Rfil_39320_OG1240
Other_SAR-Rfil_30873_OG1240
Osmo_Fungi-Ccor_953_OG1240
Osmo_Fungi-Umay_1920_OG1240
Other_SAR-Rfil_6994_OG1240
Other_Other-Goke_6514_OG1240
Other_Other-Goke_5247_OG1240
Other_Other-Goke_7448_OG1240
Other_Amorphea-Dgra_40106_OG1240
Other_Other-Ngru_15396_OG1240
Other_Other-Sspe_1565_OG1240
Other_Other-Sspe_6054_OG1240
Other_Other-Bsal_3862_OG1240
Other_Other-Egra_20525_OG1240
Cp_Diaphoretickes-Bfri_41748_OG1240
Cp_Diaphoretickes-Gthe_19200_OG1240
Cp_Diaphoretickes-Bnat_15850_OG1240
Other_Other-Sspe_7443_OG1240
Other_Amorphea-Dgra_27365_OG1240
Other_Diaphoretickes-Gavo_11488_OG1240
Other_Diaphoretickes-Hspp_8831_OG1240
Other_Other-Egra_14412_OG1240
Other_Other-Bsal_4170_OG1240
Other_Other-Bsal_4171_OG1240
Other_Other-Bsal_4172_OG1240
Other_Other-Tbru_3584_OG1240
Other_Other-Tbru_3585_OG1240
Cp_Diaphoretickes-Bnat_11707_OG1240
Other_Other-Tbru_3582_OG1240
Cp_Diaphoretickes-Chsp_4294_OG1240
Cp_Diaphoretickes-Ehux_16616_OG1240
Cp_Diaphoretickes-Dspp_16196_OG1240
Cp_Diaphoretickes-Ehux_28330_OG1240
Cp_Diaphoretickes-Ehux_10419_OG1240
Cp_Diaphoretickes-Chsp_3973_OG1240
Virus-UP_v_13368_OG1240
Cp_Diaphoretickes-Ehux_27685_OG1240
Other_Amorphea-Dgra_8900_OG1240
Cp_Diaphoretickes-Dspp_41068_OG1240
Cp_Diaphoretickes-Aano_761_OG1240
Cp_Diaphoretickes-Vbra_2206_OG1240
Cp_Diaphoretickes-Vbra_5163_OG1240
Cp_Diaphoretickes-Cvel_17917_OG1240
Osmo_Pseudofungi-Hara_2529_OG1240_intClade_1
Osmo_Pseudofungi-Pinf_14578_OG1240_intClade_1
Osmo_Pseudofungi-Pvex_4869_OG1240_intClade_1
Osmo_Pseudofungi-Parr_13454_OG1240_intClade_1
Osmo_Pseudofungi-Alai_5465_OG1240_intClade_1
Osmo_Pseudofungi-Pult_1697_OG1240_intClade_1
Osmo_Pseudofungi-Spar_5384_OG1240_intClade_1
Osmo_Pseudofungi-Aast_22625_OG1240_intClade_1
Osmo_Pseudofungi-Spar_16689_OG1240_intClade_1
Osmo_Pseudofungi-Spar_2764_OG1240_intClade_1
Osmo_Pseudofungi-Spar_18032_OG1240_intClade_1
Osmo_Pseudofungi-Aast_3665_OG1240_intClade_1
Osmo_Pseudofungi-Aast_25831_OG1240_intClade_1
Osmo_Pseudofungi-Hcat_3015_OG1240_intClade_1
Osmo_Teretosporea-Pgem_15548_OG1240_intClade_1
Osmo_Teretosporea-Awhi_995_OG1240_intClade_1
Osmo_Teretosporea-Awhi_6049_OG1240_intClade_1
Osmo_Teretosporea-Awhi_12827_OG1240_intClade_1
Osmo_Teretosporea-Sarc_33213_OG1240
Osmo_Teretosporea-Cfra_8295_OG1240
Osmo_Teretosporea-Ihof_4601_OG1240
Osmo_Teretosporea-Apar_13174_OG1240
Osmo_Labyrinthulea-Hfer_11634_OG1240
Osmo_Labyrinthulea-Alim_38583_OG1240
Osmo_Labyrinthulea-Sagg_59551_OG1240
Osmo_Teretosporea-Pgem_12376_OG1240
Osmo_Teretosporea-Awhi_9488_OG1240
Osmo_Teretosporea-Pgem_20746_OG1240
Cp_Diaphoretickes-Ngad_14830_OG1240
Cp_Diaphoretickes-Ngad_2514_OG1240
Cp_Diaphoretickes-Ngad_8415_OG1240
Cp_Diaphoretickes-Ngad_14881_OG1240
Cp_Diaphoretickes-Ptri_8531_OG1240
Cp_Diaphoretickes-Ptri_8614_OG1240
Cp_Diaphoretickes-Tpse_9513_OG1240
Cp_Diaphoretickes-Ngad_12974_OG1240
Cp_Diaphoretickes-Ngad_7134_OG1240
Cp_Diaphoretickes-Ngad_6685_OG1240
Cp_Diaphoretickes-Ngad_10463_OG1240
Cp_Diaphoretickes-Ngad_4272_OG1240
Cp_Diaphoretickes-Ngad_13751_OG1240
Cp_Diaphoretickes-Ngad_2875_OG1240
Other_Other-Egra_14097_OG1240
Other_Other-Egra_10883_OG1240
Other_Other-Sspe_14290_OG1240
Cp_Diaphoretickes-Aano_2917_OG1240
Cp_Diaphoretickes-Esil_15489_OG1240
Other_Stramenopiles-Croe_5987_OG1240
Other_Diaphoretickes-Omar_39822_OG1240
Cp_Diaphoretickes-Smin_7314_OG1240
Cp_Diaphoretickes-Cvel_6957_OG1240
Cp_Diaphoretickes-Vbra_7561_OG1240
Other_Diaphoretickes-Tgon_6957_OG1240
Osmo_Labyrinthulea-Sagg_82007_OG1240
Osmo_Labyrinthulea-Sagg_81257_OG1240
Osmo_Labyrinthulea-Aker_38242_OG1240
Osmo_Labyrinthulea-Alim_43980_OG1240
Osmo_Labyrinthulea-Hfer_155_OG1240
Other_Other-Goke_6254_OG1240
Pp_Diaphoretickes-Pmic_21331_OG1240
Other_Holozoa-Mvib_g2087_OG1240
Cp_Diaphoretickes-Bnat_21628_OG1240
Other_Diaphoretickes-Hspp_4062_OG1240
Other_Amorphea-Ttra_7333_OG1240
Other_Stramenopiles-Bisp_14994_OG1240
Other_Holozoa-Pvie_g14251_OG1240
Other_Diaphoretickes-Gavo_4228_OG1240
Other_Diaphoretickes-Gavo_17344_OG1240
Osmo_Teretosporea-Clim_g3015_OG1240
Other_Holozoa-Sros_1574_OG1240
Osmo_Teretosporea-Sdes_12782_OG1240
Other_Amorphea-Opea_4708_OG1240
Other_Amorphea-Opea_14772_OG1240
Other_Amorphea-Opea_26882_OG1240
Other_Amorphea-Bflo_7120_OG1240
Other_Amorphea-Bflo_3280_OG1240
Other_Amorphea-Bflo_21927_OG1240
Other_Holozoa-Hsap_18471_OG1240
Pp_Diaphoretickes-Pyez_9854_OG1240
Other_Holozoa-Hsap_18432_OG1240
Other_Amorphea-Bflo_19966_OG1240
Other_Amorphea-Bflo_16432_OG1240
Other_Holozoa-Dpul_5045_OG1240
Other_Amorphea-Mlei_11236_OG1240
Other_Amorphea-Tspp_10291_OG1240
Other_Amorphea-Mlei_4462_OG1240
Other_Amorphea-Opea_6652_OG1240
Other_Amorphea-Opea_6653_OG1240
Other_Amorphea-Tspp_11155_OG1240
Other_Amorphea-Mlei_7130_OG1240
Other_Amorphea-Acas_3151_OG1240
Other_Amorphea-Acas_12891_OG1240
Other_Amorphea-Acas_14512_OG1240
Other_Amorphea-Acas_13136_OG1240
Cp_Diaphoretickes-Dspp_612_OG1240
0.5

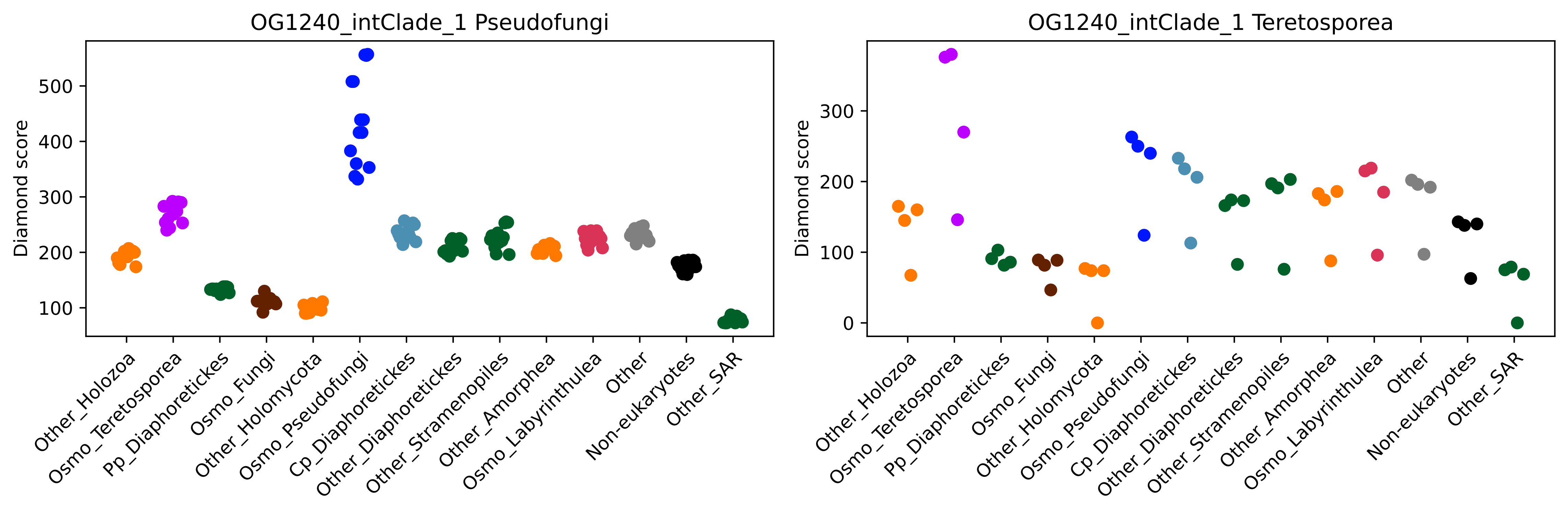
OG1240_intClade_1 Pseudofungi
Diamond score
100
200
300
400
500
Other_Holozoa
Osmo_Teretosporea
Pp_Diaphoretickes
Osmo_Fungi
Other_Holomycota
Osmo_Pseudofungi
Cp_Diaphoretickes
Other_Diaphoretickes
Other_Stramenopiles
Other_Amorphea
Osmo_Labyrinthulea
Other
Non-eukaryotes
Other_SAR
OG1240_intClade_1 Teretosporea
Diamond score
0
100
200
300
Other_Holozoa
Osmo_Teretosporea
Pp_Diaphoretickes
Osmo_Fungi
Other_Holomycota
Osmo_Pseudofungi
Cp_Diaphoretickes
Other_Diaphoretickes
Other_Stramenopiles
Other_Amorphea
Osmo_Labyrinthulea
Other
Non-eukaryotes
Other_SAR

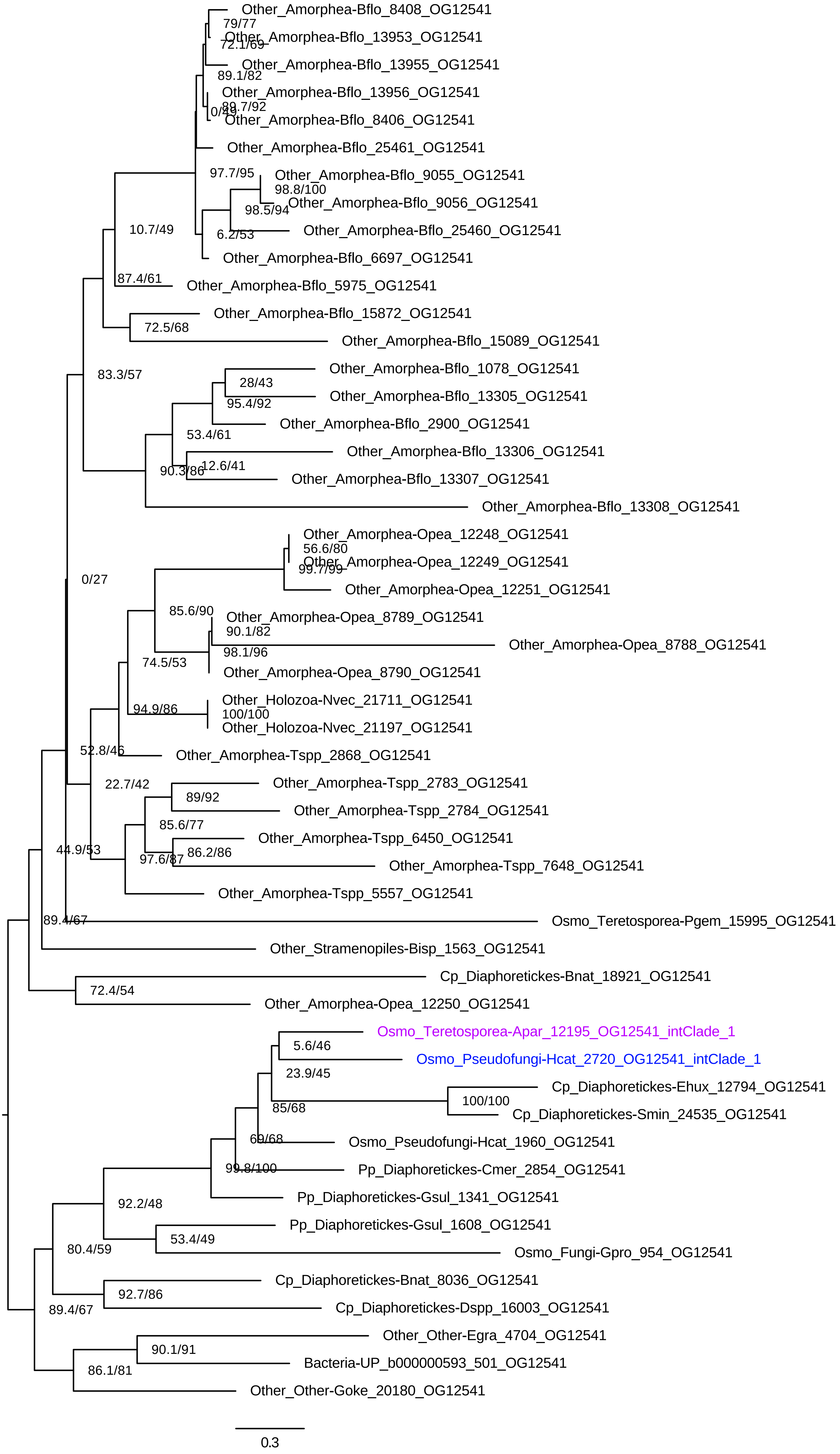

Other_Amorphea-Bflo_8408_OG12541
79/77
Other_Amorphea-Bflo_13953_OG12541
72.1/69
Other_Amorphea-Bflo_13955_OG12541
89.1/82
Other_Amorphea-Bflo_13956_OG12541
89.7/92
0/49
Other_Amorphea-Bflo_8406_OG12541
Other_Amorphea-Bflo_25461_OG12541
97.7/95
Other_Amorphea-Bflo_9055_OG12541
98.8/100
Other_Amorphea-Bflo_9056_OG12541
98.5/94
Other_Amorphea-Bflo_25460_OG12541
10.7/49
6.2/53
Other_Amorphea-Bflo_6697_OG12541
87.4/61
Other_Amorphea-Bflo_5975_OG12541
Other_Amorphea-Bflo_15872_OG12541
72.5/68
Other_Amorphea-Bflo_15089_OG12541
83.3/57
Other_Amorphea-Bflo_1078_OG12541
28/43
Other_Amorphea-Bflo_13305_OG12541
95.4/92
Other_Amorphea-Bflo_2900_OG12541
53.4/61
Other_Amorphea-Bflo_13306_OG12541
12.6/41
90.3/86
Other_Amorphea-Bflo_13307_OG12541
Other_Amorphea-Bflo_13308_OG12541
Other_Amorphea-Opea_12248_OG12541
56.6/80
Other_Amorphea-Opea_12249_OG12541
99.7/99
0/27
Other_Amorphea-Opea_12251_OG12541
85.6/90
Other_Amorphea-Opea_8789_OG12541
90.1/82
Other_Amorphea-Opea_8788_OG12541
98.1/96
74.5/53
Other_Amorphea-Opea_8790_OG12541
Other_Holozoa-Nvec_21711_OG12541
94.9/86
100/100
Other_Holozoa-Nvec_21197_OG12541
52.8/46
Other_Amorphea-Tspp_2868_OG12541
22.7/42
Other_Amorphea-Tspp_2783_OG12541
89/92
Other_Amorphea-Tspp_2784_OG12541
85.6/77
Other_Amorphea-Tspp_6450_OG12541
44.9/53
86.2/86
97.6/87
Other_Amorphea-Tspp_7648_OG12541
Other_Amorphea-Tspp_5557_OG12541
89.4/67
Osmo_Teretosporea-Pgem_15995_OG12541
Other_Stramenopiles-Bisp_1563_OG12541
Cp_Diaphoretickes-Bnat_18921_OG12541
72.4/54
Other_Amorphea-Opea_12250_OG12541
Osmo_Teretosporea-Apar_12195_OG12541_intClade_1
5.6/46
Osmo_Pseudofungi-Hcat_2720_OG12541_intClade_1
23.9/45
Cp_Diaphoretickes-Ehux_12794_OG12541
100/100
85/68
Cp_Diaphoretickes-Smin_24535_OG12541
69/68
Osmo_Pseudofungi-Hcat_1960_OG12541
99.8/100
Pp_Diaphoretickes-Cmer_2854_OG12541
Pp_Diaphoretickes-Gsul_1341_OG12541
92.2/48
Pp_Diaphoretickes-Gsul_1608_OG12541
53.4/49
80.4/59
Osmo_Fungi-Gpro_954_OG12541
Cp_Diaphoretickes-Bnat_8036_OG12541
92.7/86
89.4/67
Cp_Diaphoretickes-Dspp_16003_OG12541
Other_Other-Egra_4704_OG12541
90.1/91
Bacteria-UP_b000000593_501_OG12541
86.1/81
Other_Other-Goke_20180_OG12541
0.3

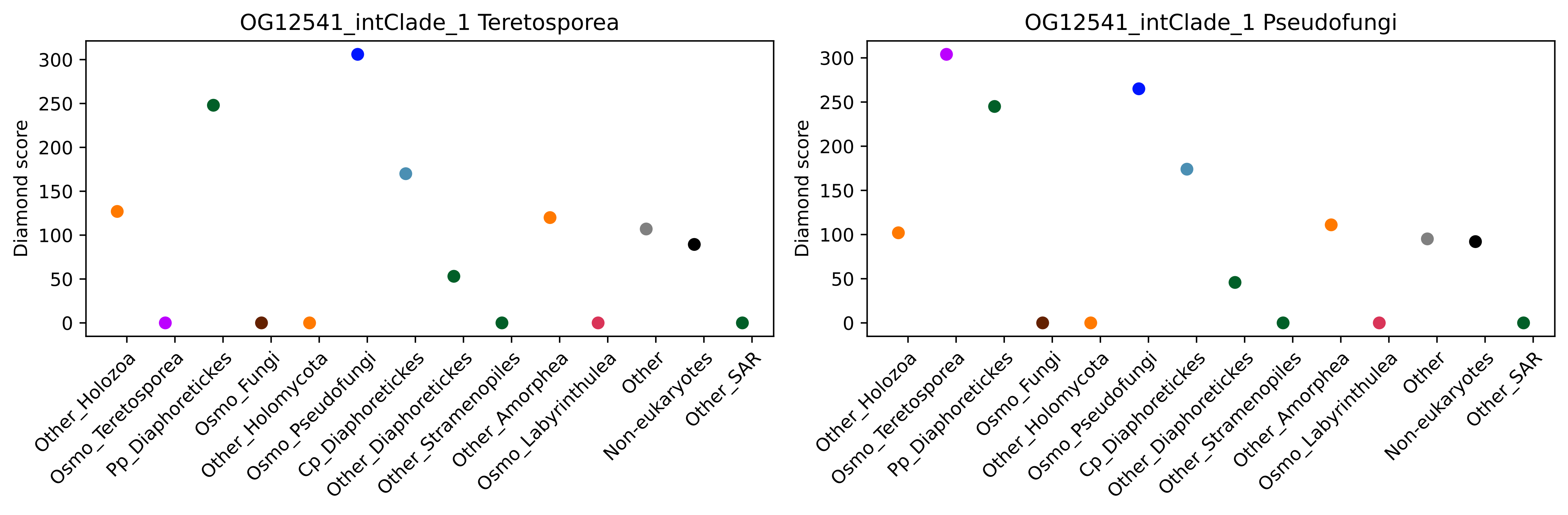

OG12541_intClade_1 Teretosporea
Diamond score
0
50
100
150
200
250
300
Other_Holozoa
Osmo_Teretosporea
Pp_Diaphoretickes
Osmo_Fungi
Other_Holomycota
Osmo_Pseudofungi
Cp_Diaphoretickes
Other_Diaphoretickes
Other_Stramenopiles
Other_Amorphea
Osmo_Labyrinthulea
Other
Non-eukaryotes
Other_SAR
OG12541_intClade_1 Pseudofungi
Diamond score
0
50
100
150
200
250
300
Other_Holozoa
Osmo_Teretosporea
Pp_Diaphoretickes
Osmo_Fungi
Other_Holomycota
Osmo_Pseudofungi
Cp_Diaphoretickes
Other_Diaphoretickes
Other_Stramenopiles
Other_Amorphea
Osmo_Labyrinthulea
Other
Non-eukaryotes
Other_SAR

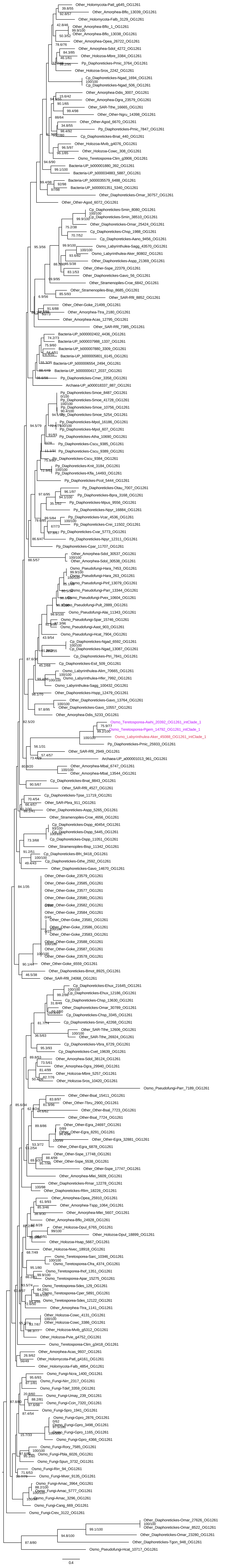

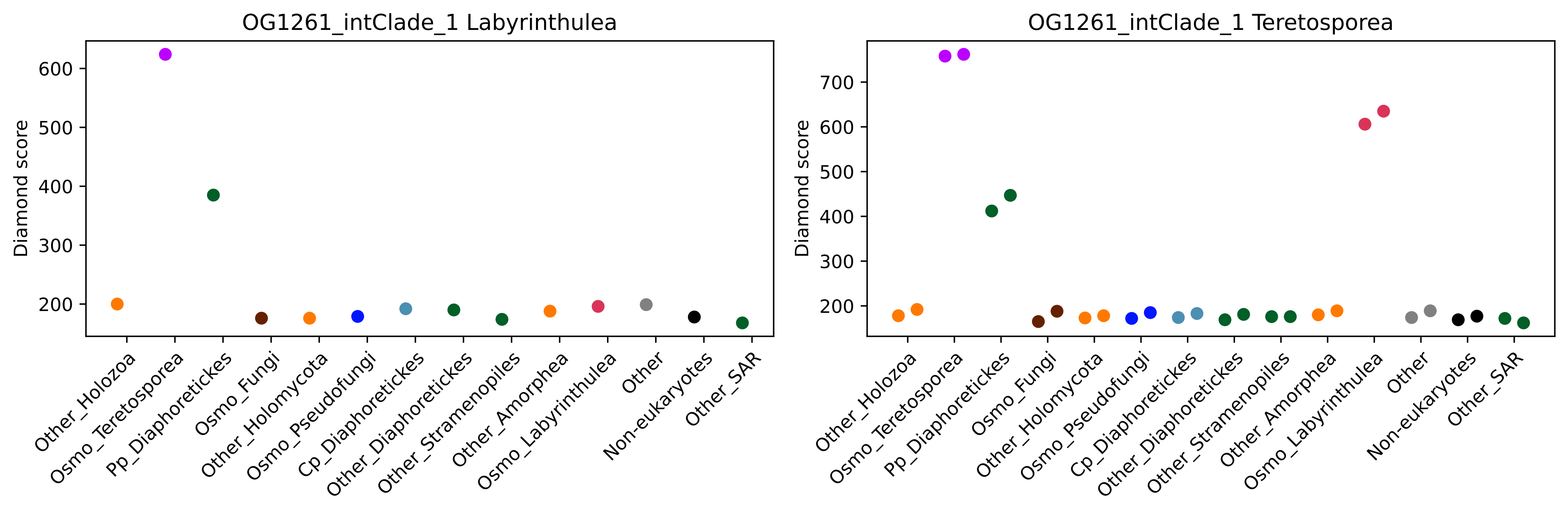
OG1261_intClade_1 Labyrinthulea
OG1261_intClade_1 Teretosporea
Diamond score
200
300
400
500
600
700
Other_Holozoa
Osmo_Teretosporea
Pp_Diaphoretickes
Osmo_Fungi
Other_Holomycota
Osmo_Pseudofungi
Cp_Diaphoretickes
Other_Diaphoretickes
Other_Stramenopiles
Other_Amorphea
Osmo_Labyrinthulea
Other
Non-eukaryotes
Other_SAR

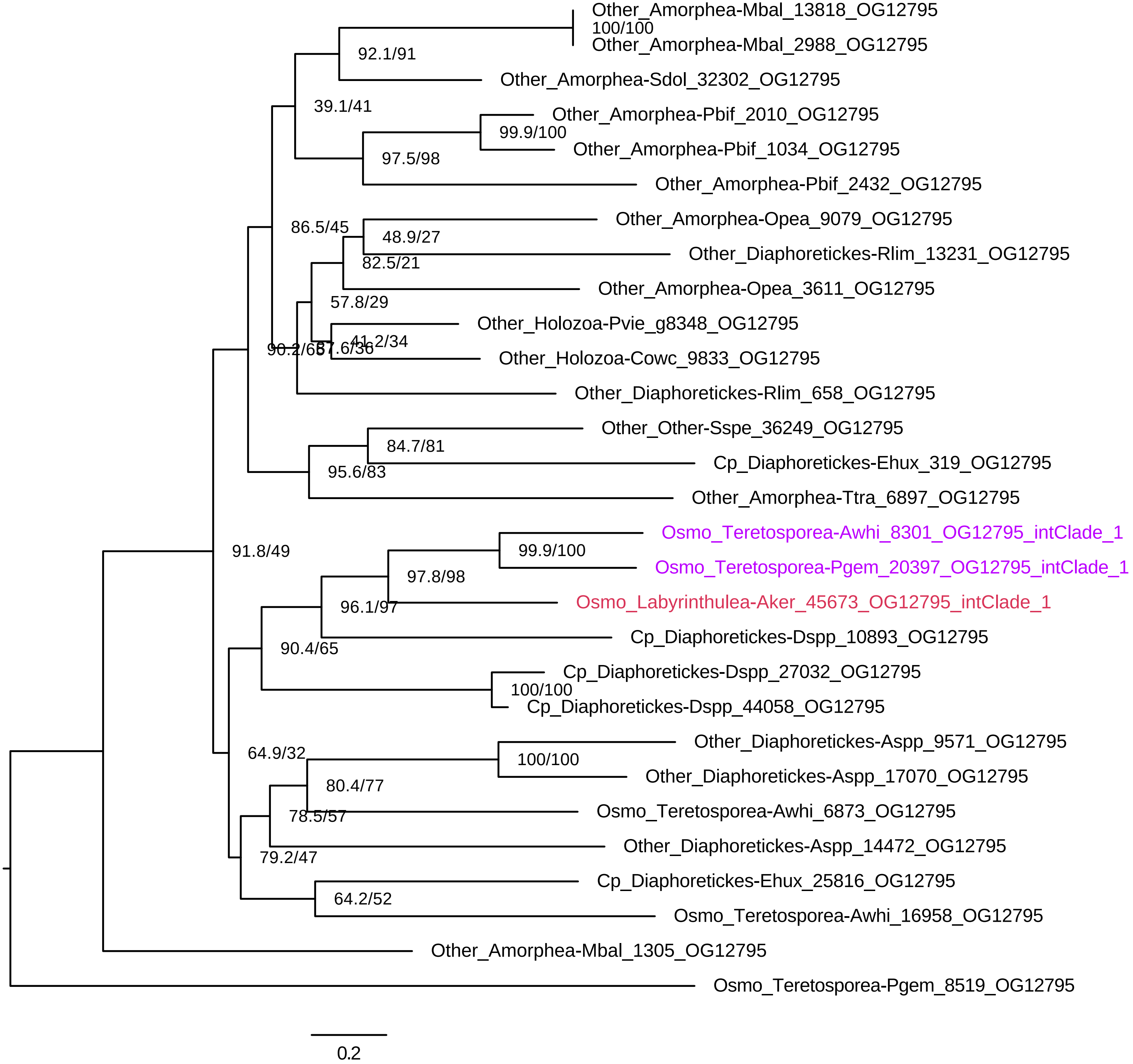
Other_Amorphea-Mbal_13818_OG12795
100/100
Other_Amorphea-Mbal_2988_OG12795
92.1/91
Other_Amorphea-Sdol_32302_OG12795
39.1/41
Other_Amorphea-Pbif_2010_OG12795
99.9/100
Other_Amorphea-Pbif_1034_OG12795
97.5/98
Other_Amorphea-Pbif_2432_OG12795
86.5/45
Other_Amorphea-Opea_9079_OG12795
48.9/27
Other_Diaphoretickes-Rlim_13231_OG12795
82.5/21
Other_Amorphea-Opea_3611_OG12795
57.8/29
Other_Holozoa-Pvie_g8348_OG12795
41.2/34
Other_Holozoa-Cowc_9833_OG12795
Other_Diaphoretickes-Rlim_658_OG12795
Other_Other-Sspe_36249_OG12795
84.7/81
Cp_Diaphoretickes-Ehux_319_OG12795
95.6/83
Other_Amorphea-Ttra_6897_OG12795
91.8/49
Osmo_Teretosporea-Awhi_8301_OG12795_intClade_1
99.9/100
Osmo_Teretosporea-Pgem_20397_OG12795_intClade_1
97.8/98
Osmo_Labyrinthulea-Aker_45673_OG12795_intClade_1
96.1/97
Cp_Diaphoretickes-Dspp_10893_OG12795
90.4/65
Cp_Diaphoretickes-Dspp_27032_OG12795
100/100
Cp_Diaphoretickes-Dspp_44058_OG12795
64.9/32
Other_Diaphoretickes-Aspp_9571_OG12795
100/100
Other_Diaphoretickes-Aspp_17070_OG12795
80.4/77
Osmo_Teretosporea-Awhi_6873_OG12795
78.5/57
Other_Diaphoretickes-Aspp_14472_OG12795
79.2/47
Cp_Diaphoretickes-Ehux_25816_OG12795
64.2/52
Osmo_Teretosporea-Awhi_16958_OG12795
Other_Amorphea-Mbal_1305_OG12795
Osmo_Teretosporea-Pgem_8519_OG12795
0.2

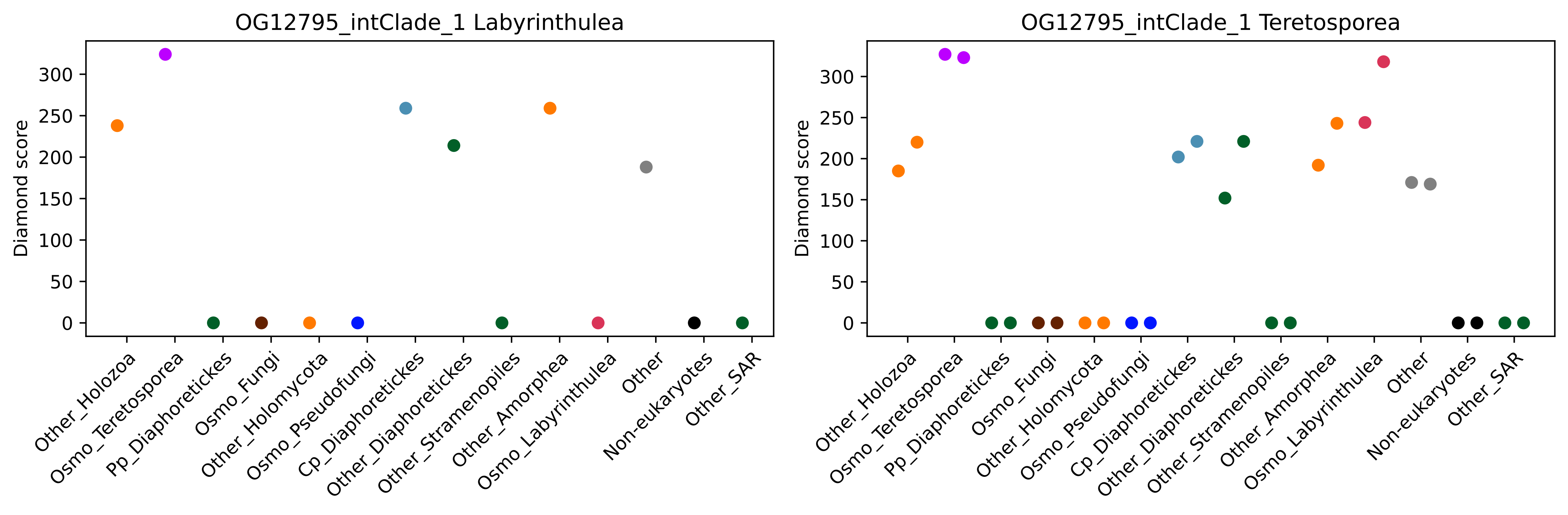

OG12795_intClade_1 Labyrinthulea
OG12795_intClade_1 Teretosporea
Diamond score
0
50
100
150
200
250
300
Other_Holozoa
Osmo_Teretosporea
Pp_Diaphoretickes
Osmo_Fungi
Other_Holomycota
Osmo_Pseudofungi
Cp_Diaphoretickes
Other_Diaphoretickes
Other_Stramenopiles
Other_Amorphea
Osmo_Labyrinthulea
Other
Non-eukaryotes
Other_SAR

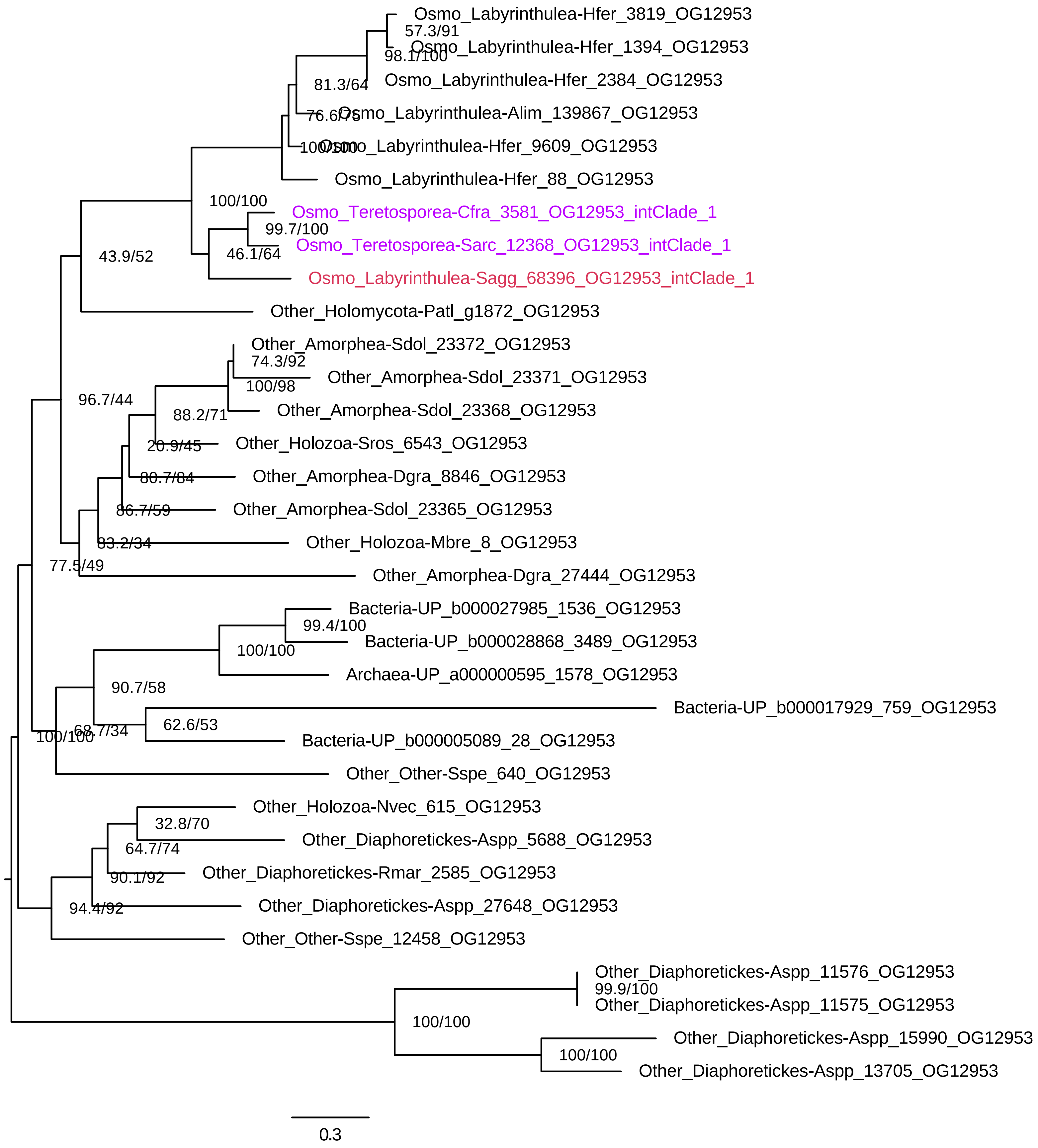

Osmo_Labyrinthulea-Hfer_3819_OG12953
57.3/91
Osmo_Labyrinthulea-Hfer_1394_OG12953
98.1/100
81.3/64
Osmo_Labyrinthulea-Hfer_2384_OG12953
76.6/75
Osmo_Labyrinthulea-Alim_139867_OG12953
100/100
Osmo_Labyrinthulea-Hfer_9609_OG12953
Osmo_Labyrinthulea-Hfer_88_OG12953
100/100
Osmo_Teretosporea-Cfra_3581_OG12953_intClade_1
99.7/100
Osmo_Teretosporea-Sarc_12368_OG12953_intClade_1
43.9/52
46.1/64
Osmo_Labyrinthulea-Sagg_68396_OG12953_intClade_1
Other_Holomycota-Patl_g1872_OG12953
Other_Amorphea-Sdol_23372_OG12953
74.3/92
Other_Amorphea-Sdol_23371_OG12953
100/98
96.7/44
Other_Amorphea-Sdol_23368_OG12953
88.2/71
20.9/45
Other_Holozoa-Sros_6543_OG12953
80.7/84
Other_Amorphea-Dgra_8846_OG12953
86.7/59
Other_Amorphea-Sdol_23365_OG12953
83.2/34
Other_Holozoa-Mbre_8_OG12953
77.5/49
Other_Amorphea-Dgra_27444_OG12953
Bacteria-UP_b000027985_1536_OG12953
99.4/100
Bacteria-UP_b000028868_3489_OG12953
100/100
Archaea-UP_a000000595_1578_OG12953
90.7/58
Bacteria-UP_b000017929_759_OG12953
62.6/53
68.7/34
100/100
Bacteria-UP_b000005089_28_OG12953
Other_Other-Sspe_640_OG12953
Other_Holozoa-Nvec_615_OG12953
32.8/70
Other_Diaphoretickes-Aspp_5688_OG12953
64.7/74
90.1/92
Other_Diaphoretickes-Rmar_2585_OG12953
94.4/92
Other_Diaphoretickes-Aspp_27648_OG12953
Other_Other-Sspe_12458_OG12953
Other_Diaphoretickes-Aspp_11576_OG12953
99.9/100
Other_Diaphoretickes-Aspp_11575_OG12953
100/100
Other_Diaphoretickes-Aspp_15990_OG12953
100/100
Other_Diaphoretickes-Aspp_13705_OG12953
0.3

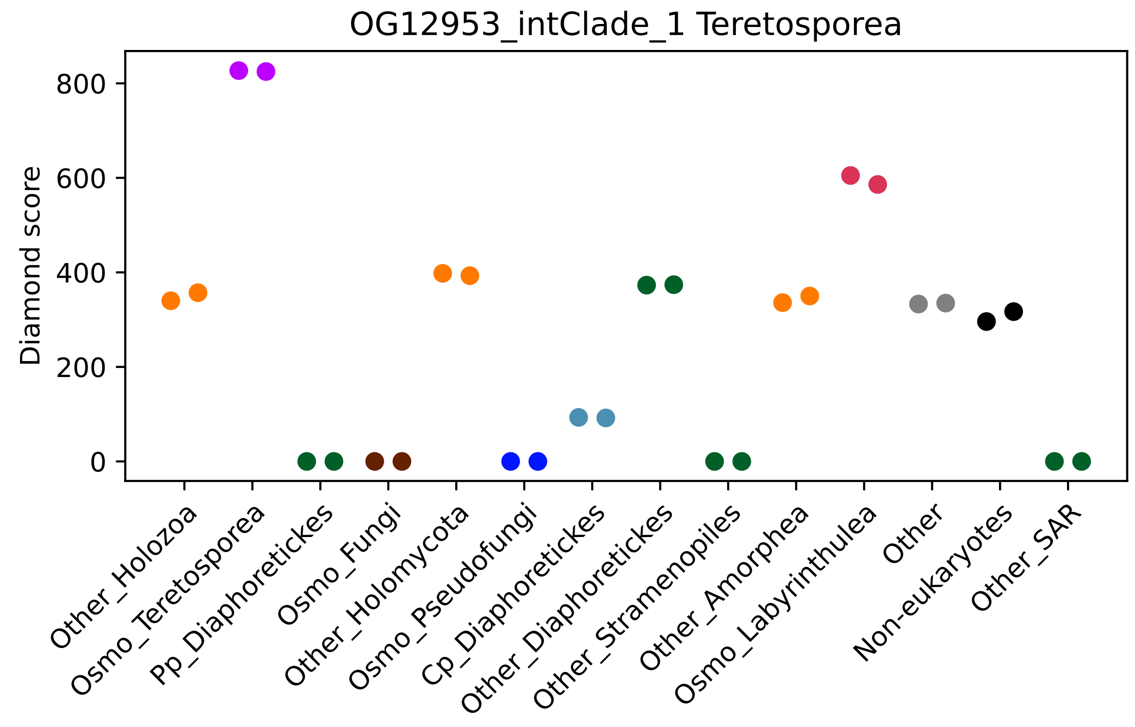
OG12953_intClade_1 Teretosporea
Diamond score
0
200
400
600
800
Other_Holozoa
Osmo_Teretosporea
Pp_Diaphoretickes
Osmo_Fungi
Other_Holomycota
Osmo_Pseudofungi
Cp_Diaphoretickes
Other_Diaphoretickes
Other_Stramenopiles
Other_Amorphea
Osmo_Labyrinthulea
Other
Non-eukaryotes
Other_SAR

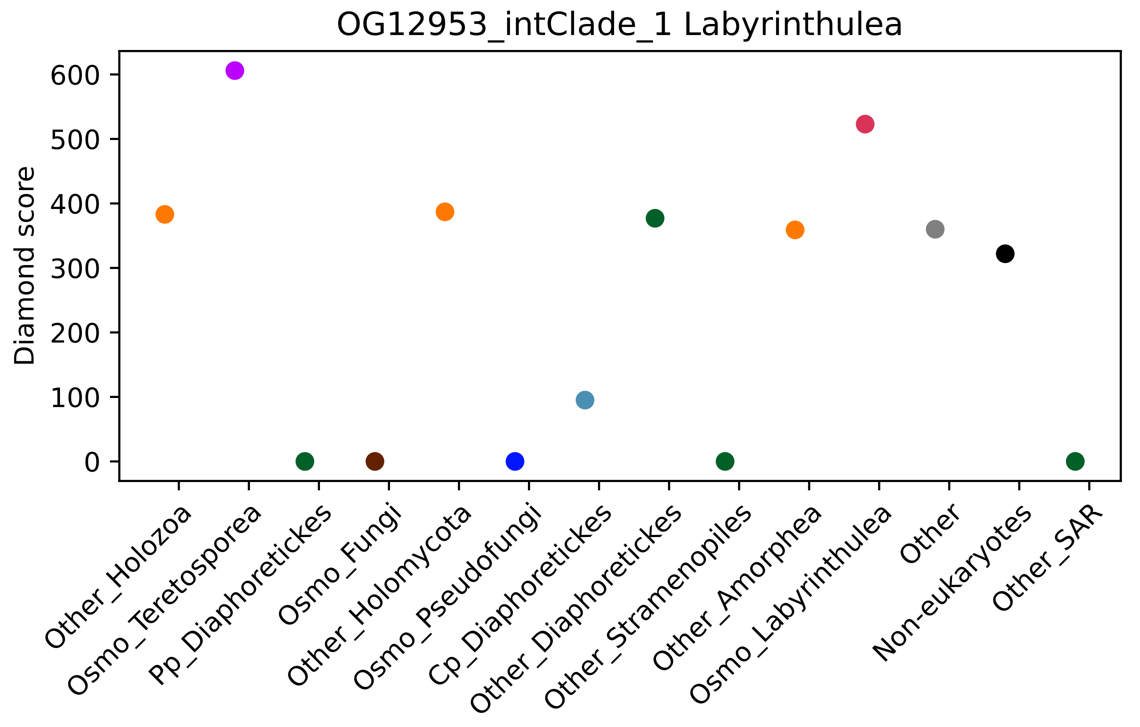
OG12953_intClade_1 Labyrinthulea
Diamond score
0
100
200
300
400
500
600
Other_Holozoa
Osmo_Teretosporea
Pp_Diaphoretickes
Osmo_Fungi
Other_Holomycota
Osmo_Pseudofungi
Cp_Diaphoretickes
Other_Diaphoretickes
Other_Stramenopiles
Other_Amorphea
Osmo_Labyrinthulea
Other
Non-eukaryotes
Other_SAR

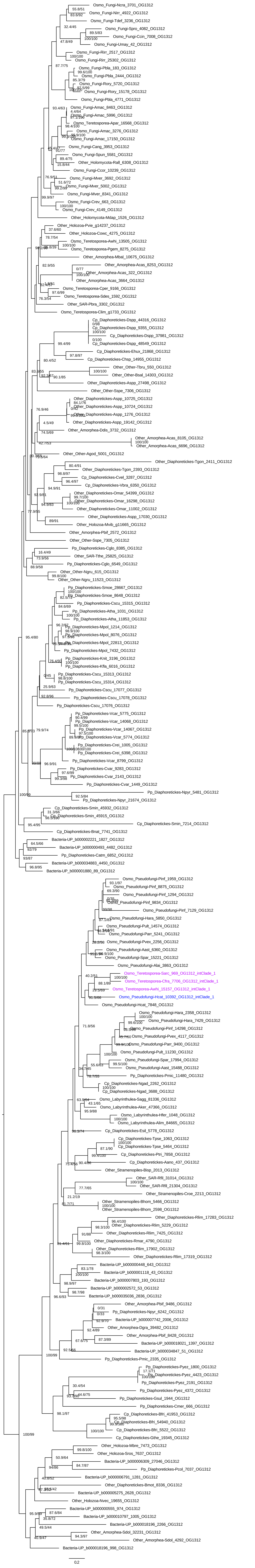

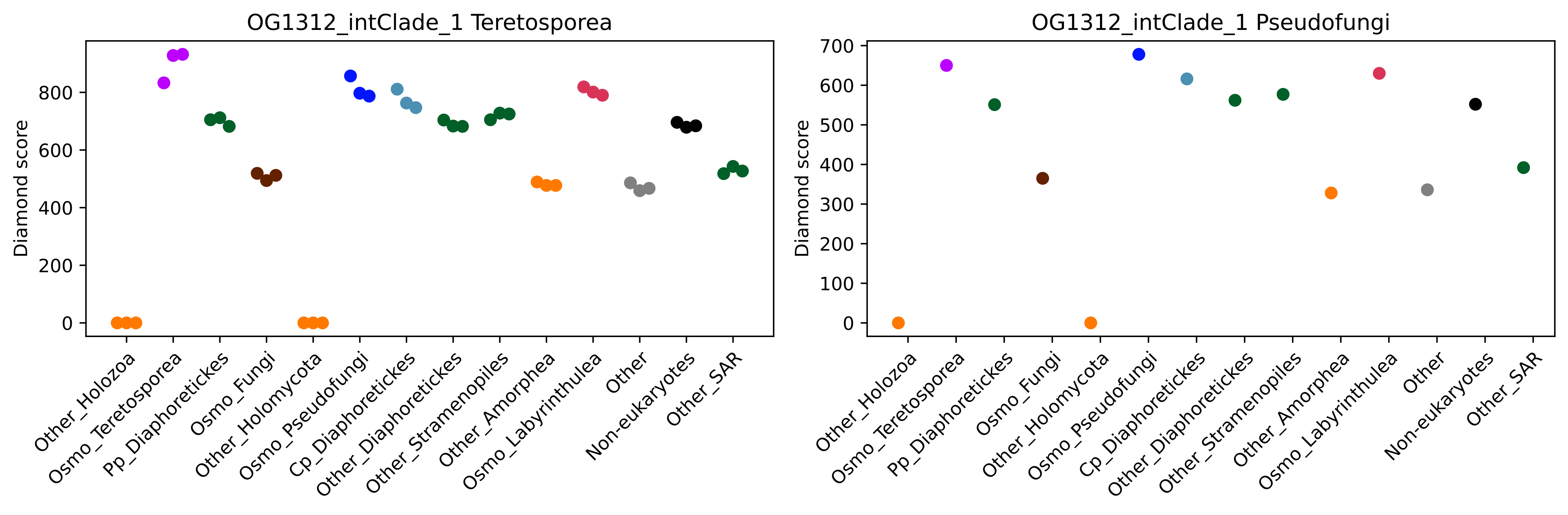
OG1312_intClade_1 Teretosporea
OG1312_intClade_1 Pseudofungi
Diamond score
0
200
400
600
800
0
100
200
300
400
500
600
700
Other_Holozoa
Osmo_Teretosporea
Pp_Diaphoretickes
Osmo_Fungi
Other_Holomycota
Osmo_Pseudofungi
Cp_Diaphoretickes
Other_Diaphoretickes
Other_Stramenopiles
Other_Amorphea
Osmo_Labyrinthulea
Other
Non-eukaryotes
Other_SAR

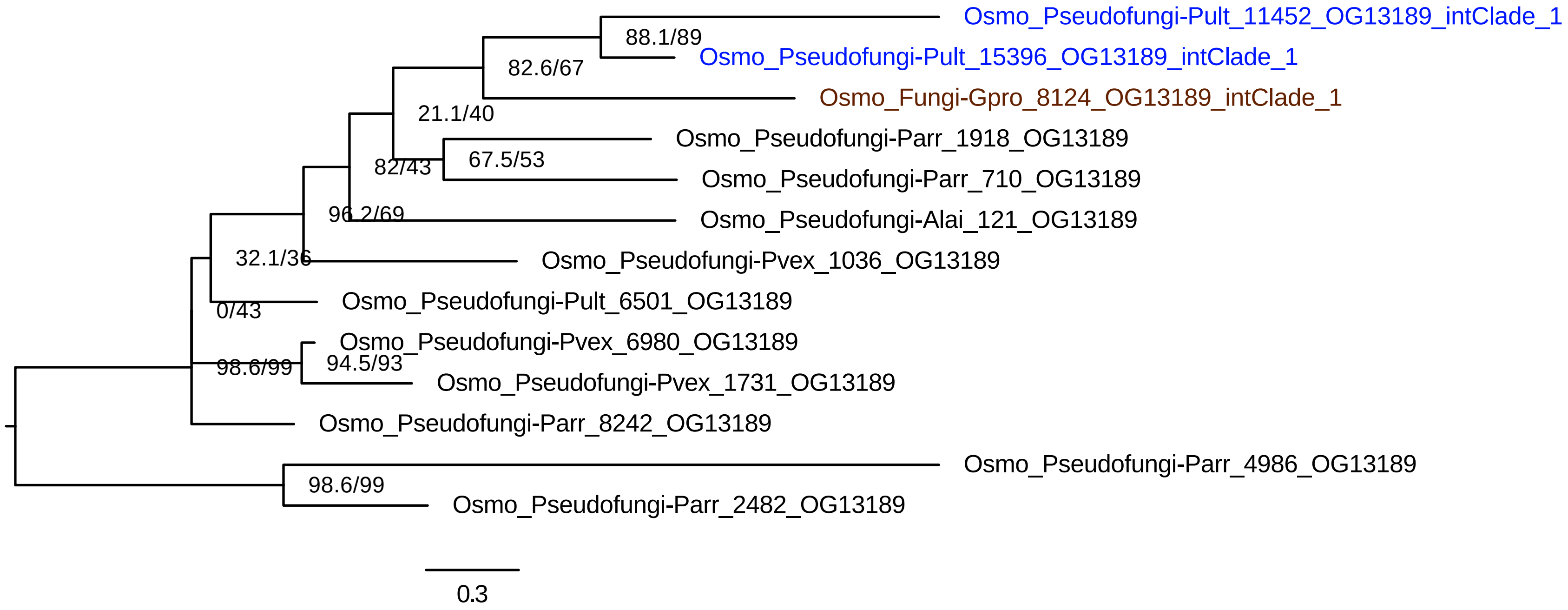

Osmo_Pseudofungi-Pult_11452_OG13189_intClade_1
88.1/89
Osmo_Pseudofungi-Pult_15396_OG13189_intClade_1
82.6/67
Osmo_Fungi-Gpro_8124_OG13189_intClade_1
21.1/40
Osmo_Pseudofungi-Parr_1918_OG13189
67.5/53
82/43
Osmo_Pseudofungi-Parr_710_OG13189
96.2/69
Osmo_Pseudofungi-Alai_121_OG13189
32.1/36
Osmo_Pseudofungi-Pvex_1036_OG13189
Osmo_Pseudofungi-Pult_6501_OG13189
0/43
Osmo_Pseudofungi-Pvex_6980_OG13189
98.6/99
94.5/93
Osmo_Pseudofungi-Pvex_1731_OG13189
Osmo_Pseudofungi-Parr_8242_OG13189
Osmo_Pseudofungi-Parr_4986_OG13189
98.6/99
Osmo_Pseudofungi-Parr_2482_OG13189
0.3

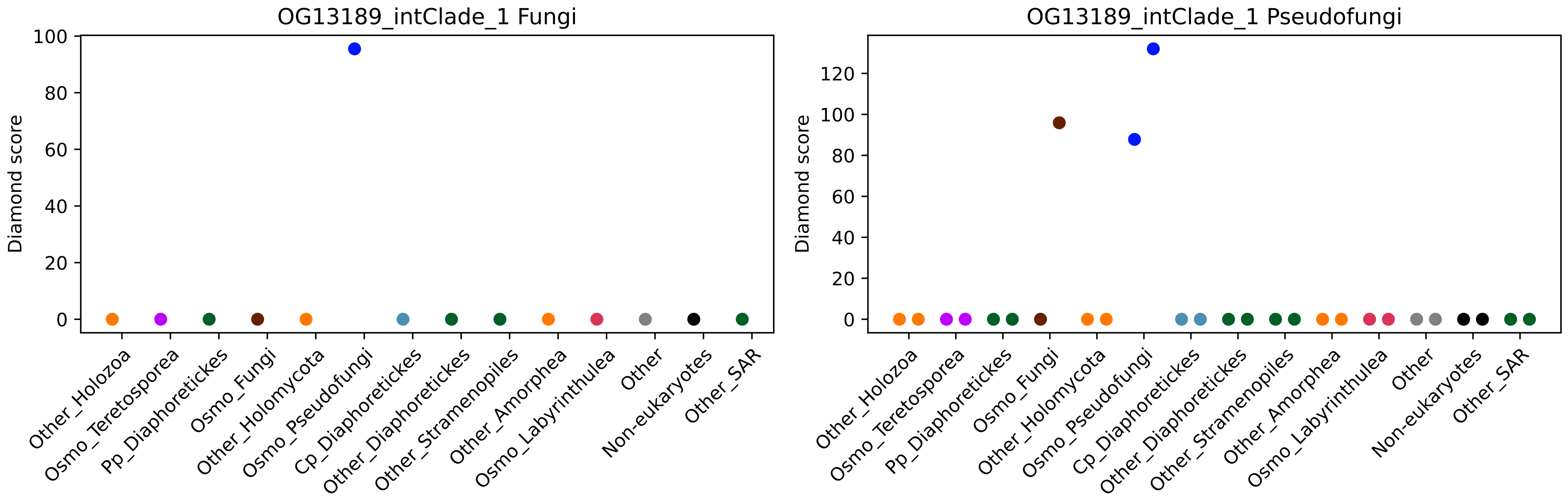
OG13189_intClade_1 Fungi
Diamond score
0
20
40
60
80
100
Other_Holozoa
Osmo_Teretosporea
Pp_Diaphoretickes
Osmo_Fungi
Other_Holomycota
Osmo_Pseudofungi
Cp_Diaphoretickes
Other_Diaphoretickes
Other_Stramenopiles
Other_Amorphea
Osmo_Labyrinthulea
Other
Non-eukaryotes
Other_SAR
OG13189_intClade_1 Pseudofungi
Diamond score
0
20
40
60
80
100
120
Other_Holozoa
Osmo_Teretosporea
Pp_Diaphoretickes
Osmo_Fungi
Other_Holomycota
Osmo_Pseudofungi
Cp_Diaphoretickes
Other_Diaphoretickes
Other_Stramenopiles
Other_Amorphea
Osmo_Labyrinthulea
Other
Non-eukaryotes
Other_SAR

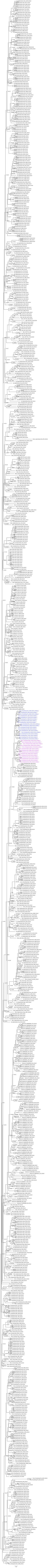

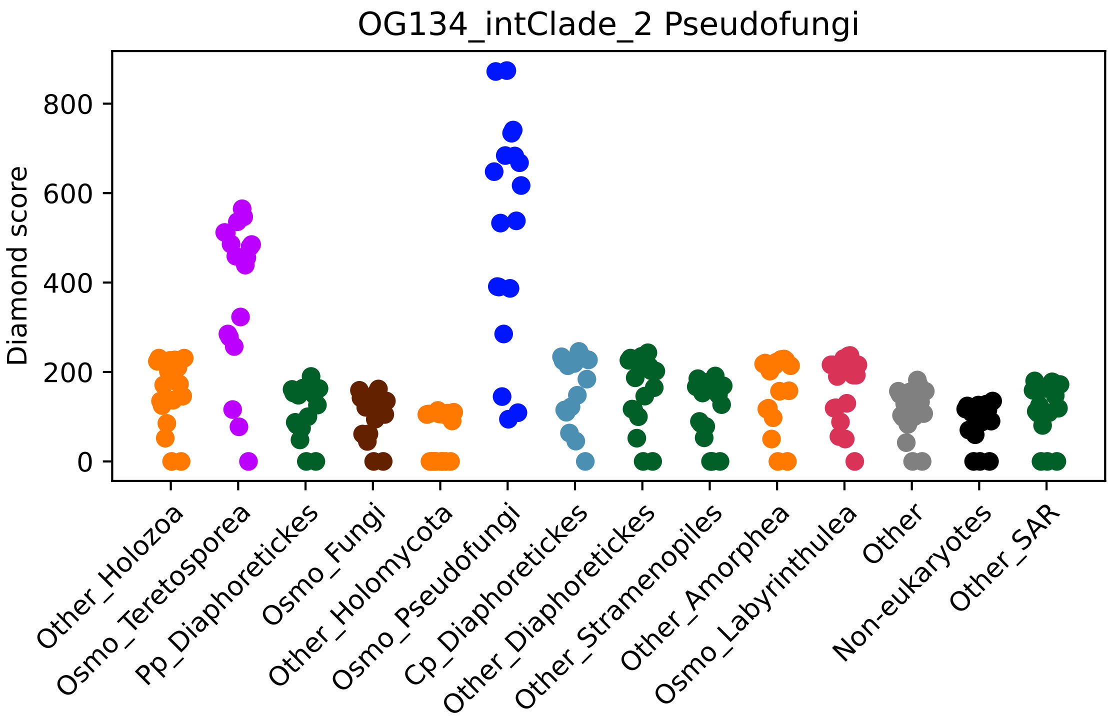

OG134_intClade_2 Pseudofungi
Diamond score
0
200
400
600
800
Other_Holozoa
Osmo_Teretosporea
Pp_Diaphoretickes
Osmo_Fungi
Other_Holomycota
Osmo_Pseudofungi
Cp_Diaphoretickes
Other_Diaphoretickes
Other_Stramenopiles
Other_Amorphea
Osmo_Labyrinthulea
Other
Non-eukaryotes
Other_SAR

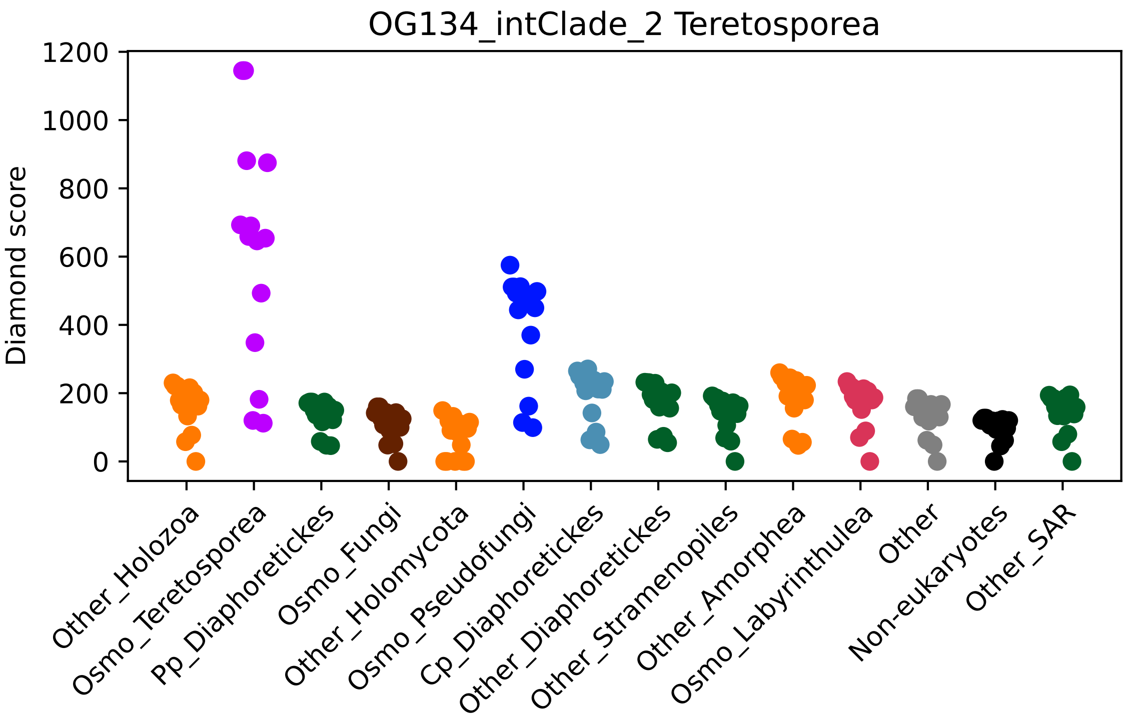

OG134_intClade_2 Teretosporea
Diamond score
0
200
400
600
800
1000
1200
Other_Holozoa
Osmo_Teretosporea
Pp_Diaphoretickes
Osmo_Fungi
Other_Holomycota
Osmo_Pseudofungi
Cp_Diaphoretickes
Other_Diaphoretickes
Other_Stramenopiles
Other_Amorphea
Osmo_Labyrinthulea
Other
Non-eukaryotes
Other_SAR

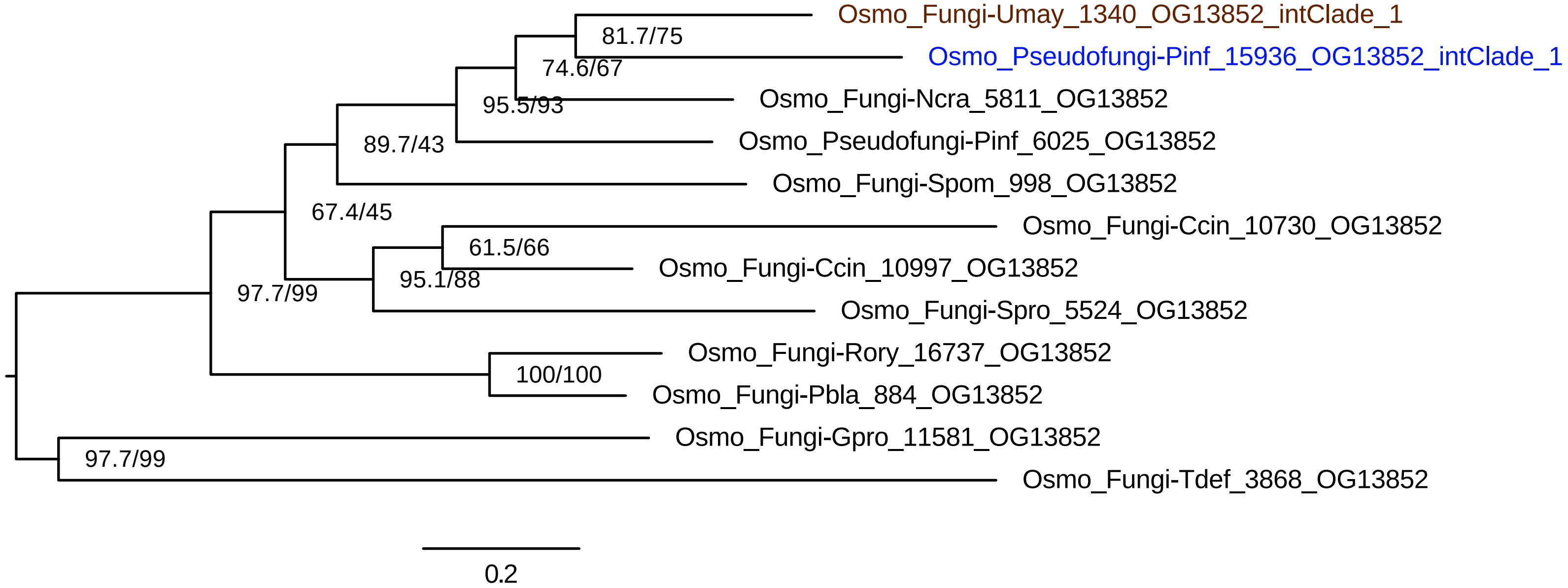

Osmo_Fungi-Umay_1340_OG13852_intClade_1
Osmo_Pseudofungi-Pinf_15936_OG13852_intClade_1
Osmo_Fungi-Ncra_5811_OG13852
Osmo_Pseudofungi-Pinf_6025_OG13852
Osmo_Fungi-Spom_998_OG13852
Osmo_Fungi-Ccin_10730_OG13852
Osmo_Fungi-Ccin_10997_OG13852
Osmo_Fungi-Spro_5524_OG13852
Osmo_Fungi-Rory_16737_OG13852
Osmo_Fungi-Pbla_884_OG13852
Osmo_Fungi-Gpro_11581_OG13852
Osmo_Fungi-Tdef_3868_OG13852
81.7/75
74.6/67
95.5/93
89.7/43
67.4/45
61.5/66
95.1/88
97.7/99
100/100
97.7/99
0.2

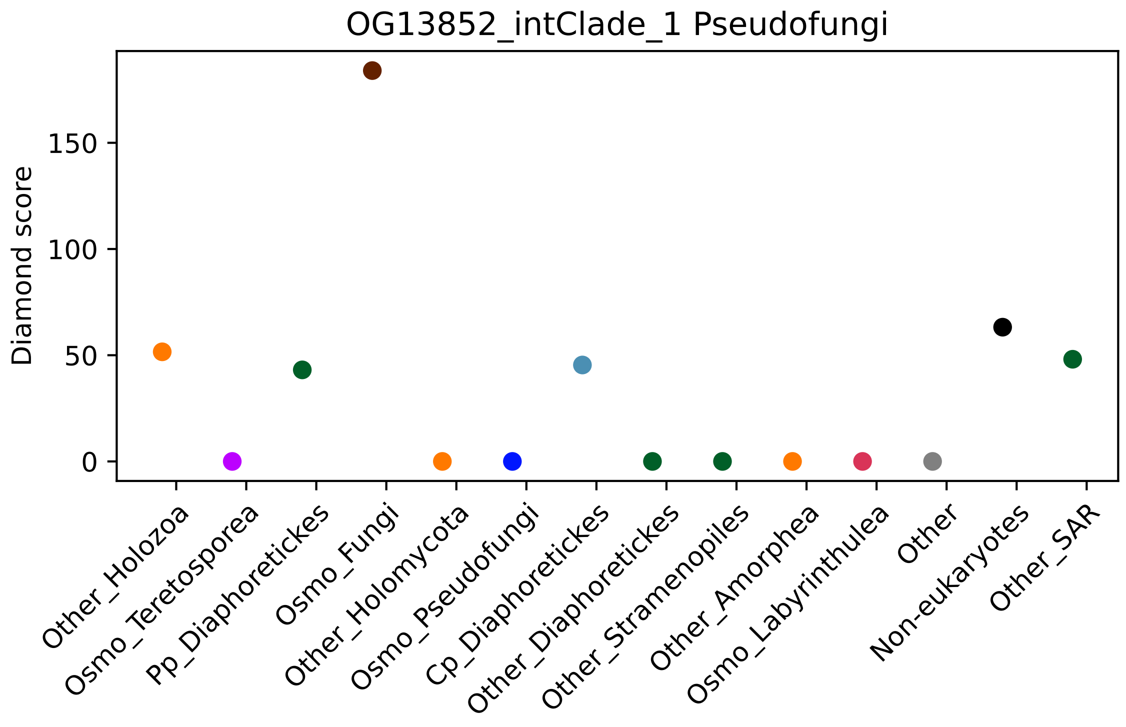
OG13852_intClade_1 Pseudofungi
Diamond score
0
50
100
150
Other_Holozoa
Osmo_Teretosporea
Pp_Diaphoretickes
Osmo_Fungi
Other_Holomycota
Osmo_Pseudofungi
Cp_Diaphoretickes
Other_Diaphoretickes
Other_Stramenopiles
Other_Amorphea
Osmo_Labyrinthulea
Other
Non-eukaryotes
Other_SAR

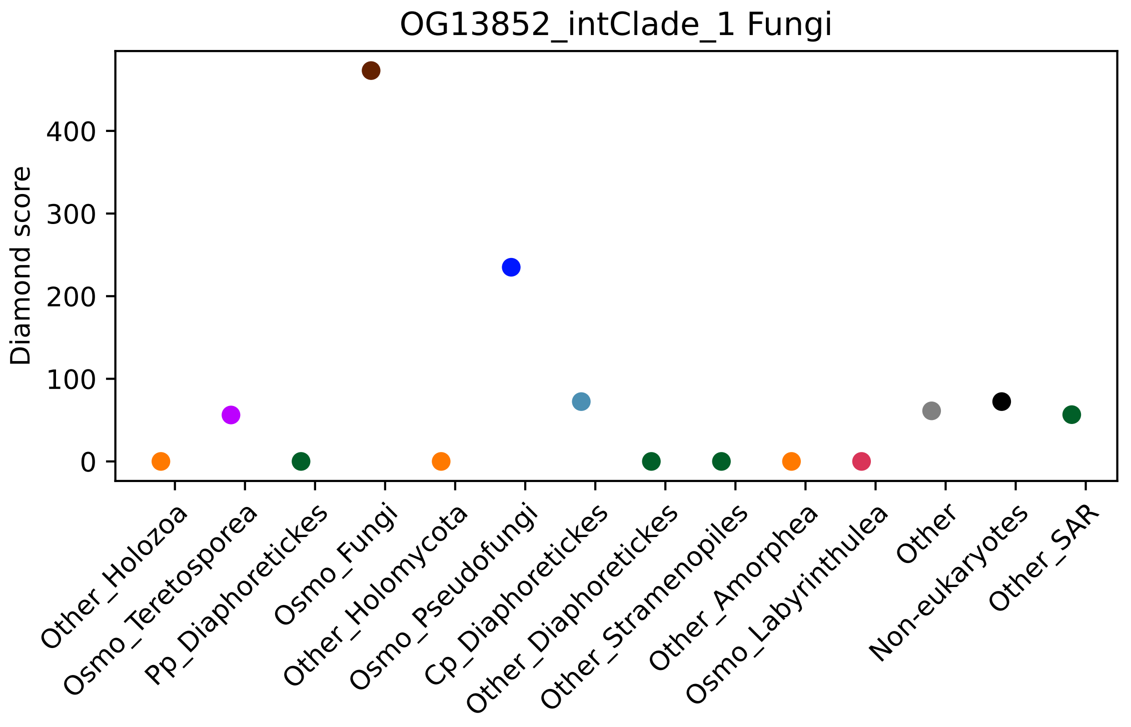
OG13852_intClade_1 Fungi
Diamond score
0
100
200
300
400
Other_Holozoa
Osmo_Teretosporea
Pp_Diaphoretickes
Osmo_Fungi
Other_Holomycota
Osmo_Pseudofungi
Cp_Diaphoretickes
Other_Diaphoretickes
Other_Stramenopiles
Other_Amorphea
Osmo_Labyrinthulea
Other
Non-eukaryotes
Other_SAR

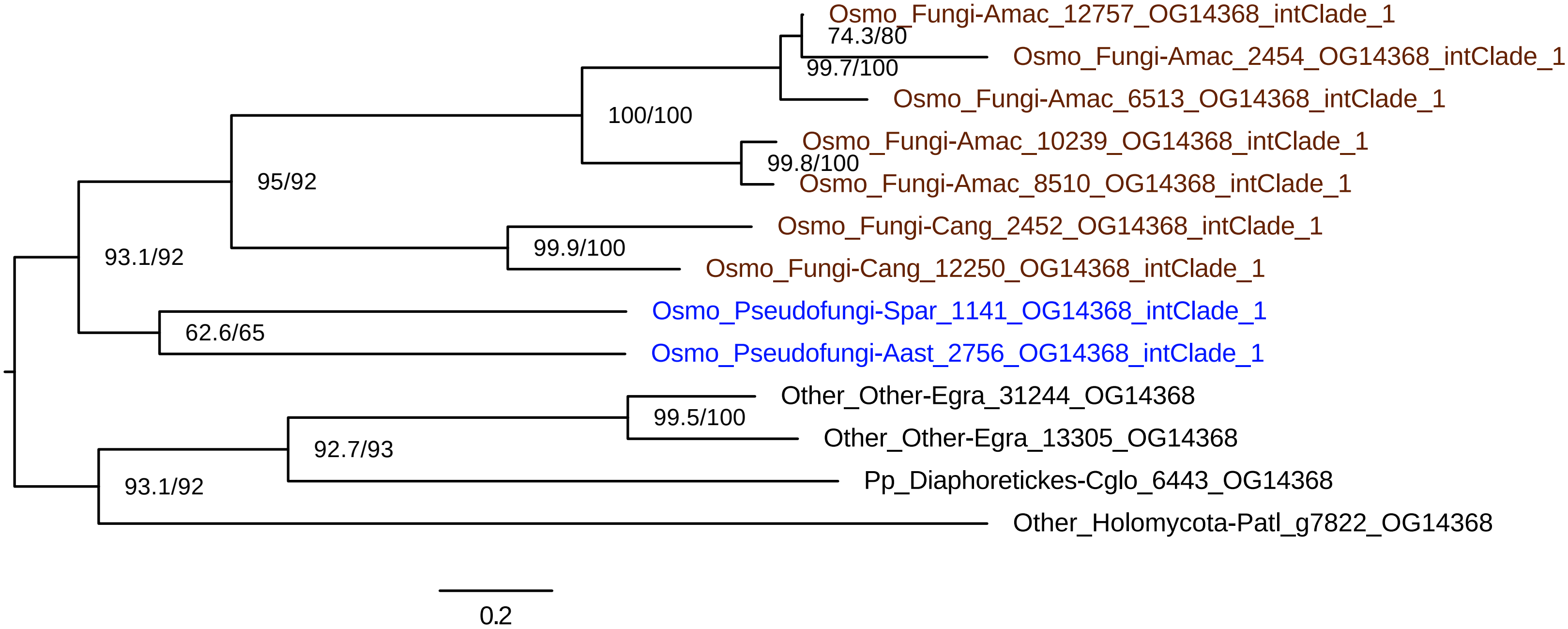
Osmo_Fungi-Amac_12757_OG14368_intClade_1
74.3/80
Osmo_Fungi-Amac_2454_OG14368_intClade_1
99.7/100
Osmo_Fungi-Amac_6513_OG14368_intClade_1
100/100
Osmo_Fungi-Amac_10239_OG14368_intClade_1
99.8/100
Osmo_Fungi-Amac_8510_OG14368_intClade_1
95/92
Osmo_Fungi-Cang_2452_OG14368_intClade_1
99.9/100
Osmo_Fungi-Cang_12250_OG14368_intClade_1
93.1/92
Osmo_Pseudofungi-Spar_1141_OG14368_intClade_1
62.6/65
Osmo_Pseudofungi-Aast_2756_OG14368_intClade_1
Other_Other-Egra_31244_OG14368
99.5/100
Other_Other-Egra_13305_OG14368
92.7/93
Pp_Diaphoretickes-Cglo_6443_OG14368
93.1/92
Other_Holomycota-Patl_g7822_OG14368
0.2

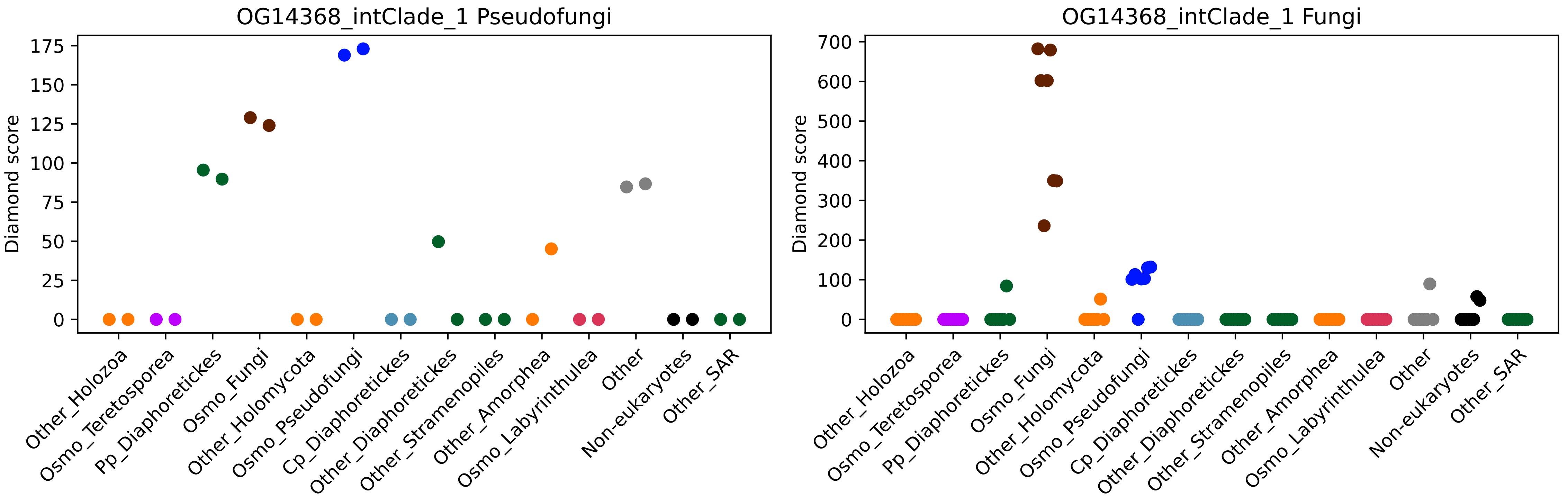
OG14368_intClade_1 Pseudofungi
OG14368_intClade_1 Fungi
Diamond score
Other_Holozoa
Osmo_Teretosporea
Pp_Diaphoretickes
Osmo_Fungi
Other_Holomycota
Osmo_Pseudofungi
Cp_Diaphoretickes
Other_Diaphoretickes
Other_Stramenopiles
Other_Amorphea
Osmo_Labyrinthulea
Other
Non-eukaryotes
Other_SAR

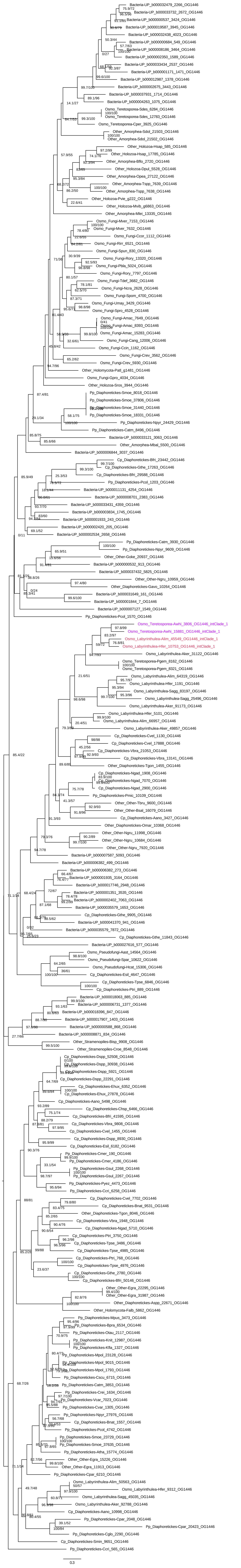

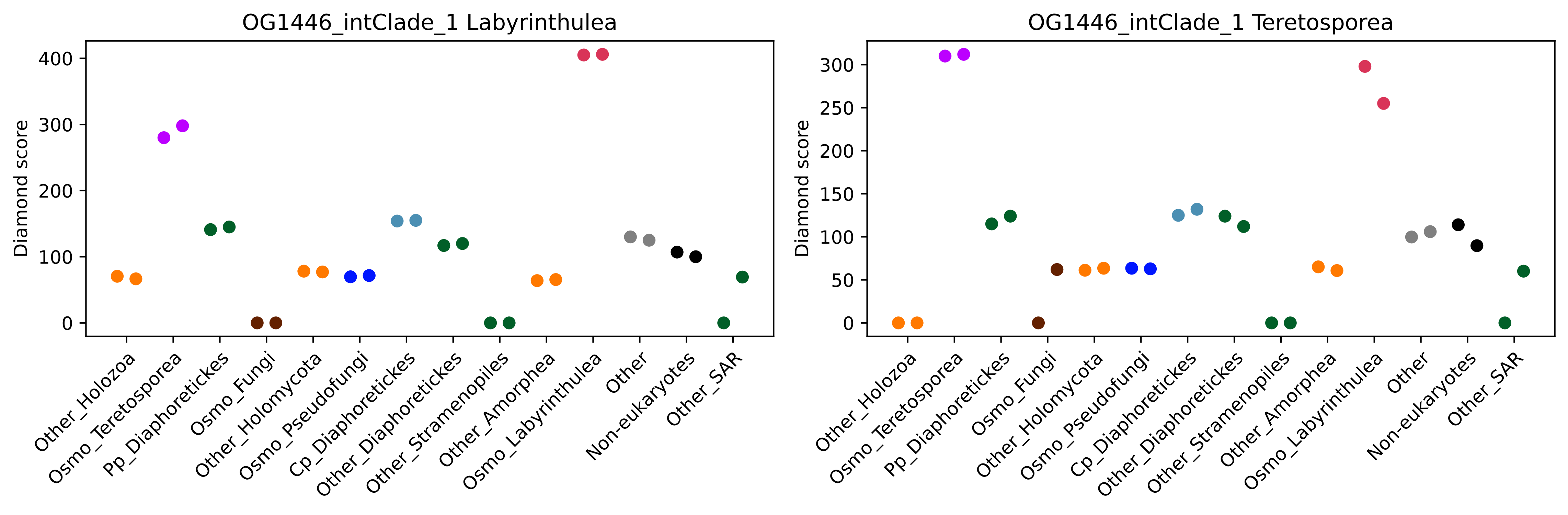

OG1446_intClade_1 Labyrinthulea
Diamond score
0
100
200
300
400
Other_Holozoa
Osmo_Teretosporea
Pp_Diaphoretickes
Osmo_Fungi
Other_Holomycota
Osmo_Pseudofungi
Cp_Diaphoretickes
Other_Diaphoretickes
Other_Stramenopiles
Other_Amorphea
Osmo_Labyrinthulea
Other
Non-eukaryotes
Other_SAR
OG1446_intClade_1 Teretosporea
Diamond score
0
50
100
150
200
250
300
Other_Holozoa
Osmo_Teretosporea
Pp_Diaphoretickes
Osmo_Fungi
Other_Holomycota
Osmo_Pseudofungi
Cp_Diaphoretickes
Other_Diaphoretickes
Other_Stramenopiles
Other_Amorphea
Osmo_Labyrinthulea
Other
Non-eukaryotes
Other_SAR

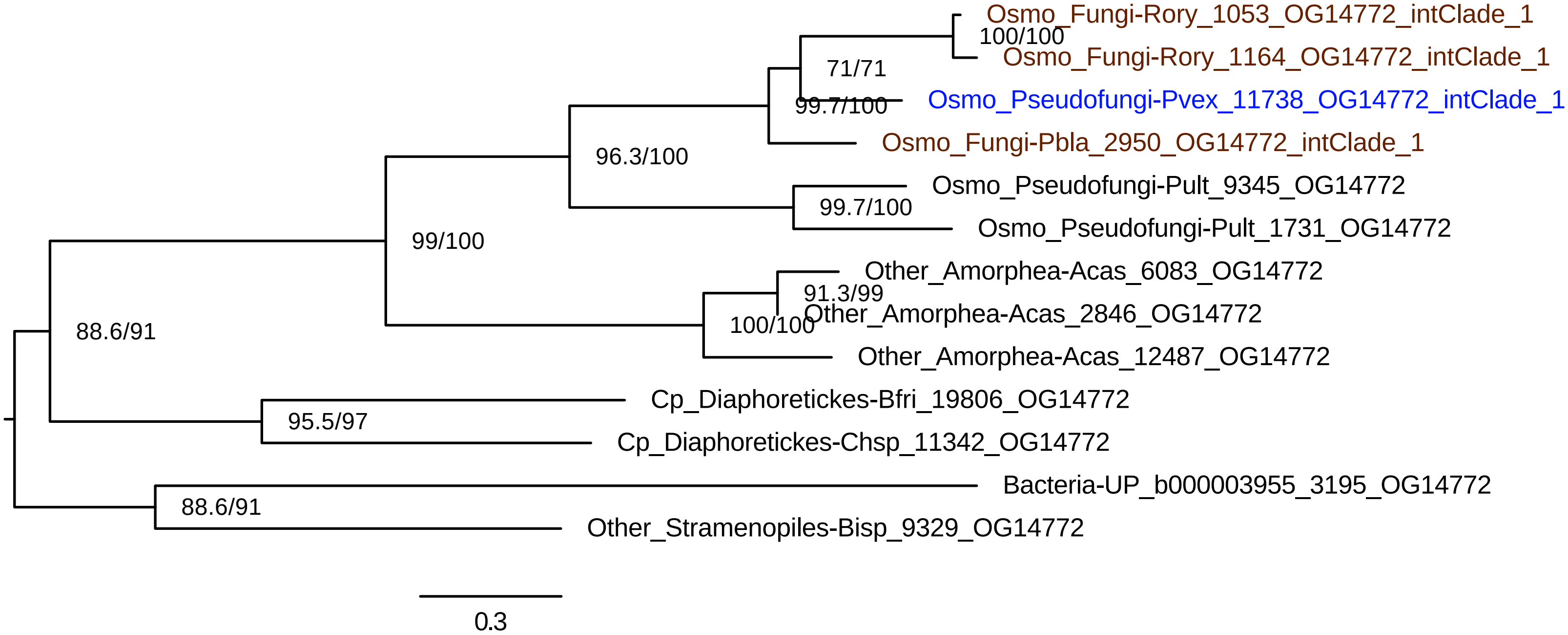
Osmo_Fungi-Rory_1053_OG14772_intClade_1
100/100
Osmo_Fungi-Rory_1164_OG14772_intClade_1
71/71
Osmo_Pseudofungi-Pvex_11738_OG14772_intClade_1
99.7/100
Osmo_Fungi-Pbla_2950_OG14772_intClade_1
96.3/100
Osmo_Pseudofungi-Pult_9345_OG14772
99.7/100
Osmo_Pseudofungi-Pult_1731_OG14772
99/100
Other_Amorphea-Acas_6083_OG14772
91.3/99
Other_Amorphea-Acas_2846_OG14772
100/100
Other_Amorphea-Acas_12487_OG14772
88.6/91
Cp_Diaphoretickes-Bfri_19806_OG14772
95.5/97
Cp_Diaphoretickes-Chsp_11342_OG14772
Bacteria-UP_b000003955_3195_OG14772
88.6/91
Other_Stramenopiles-Bisp_9329_OG14772
0.3

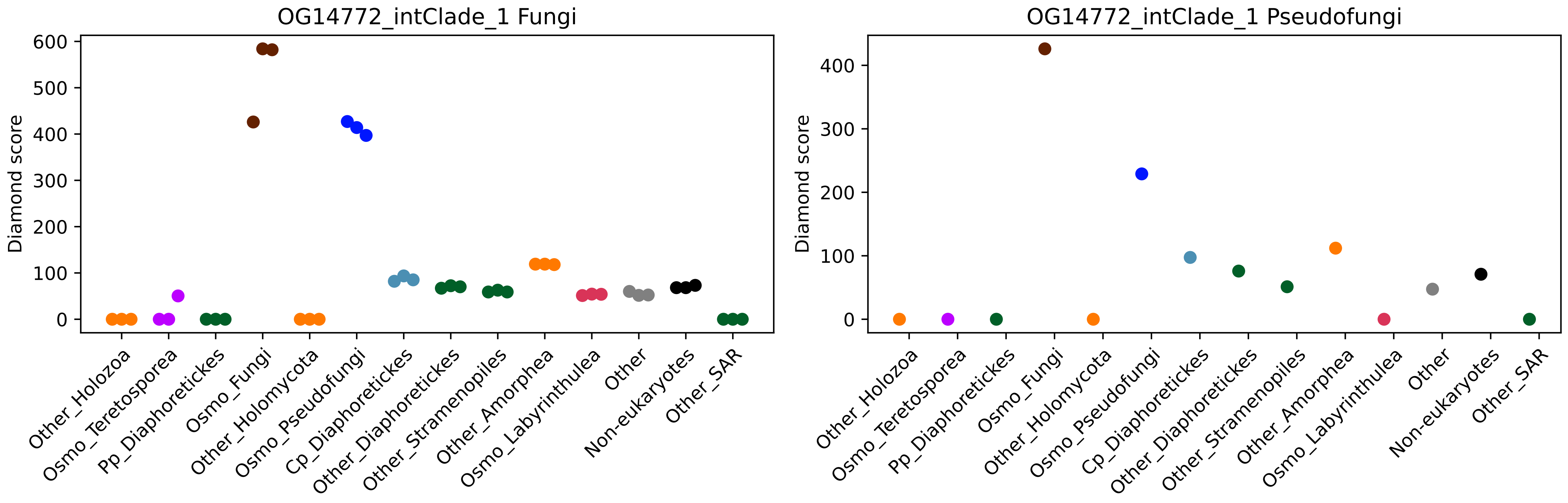
OG14772_intClade_1 Fungi
OG14772_intClade_1 Pseudofungi
Diamond score
0
100
200
300
400
500
600
Other_Holozoa
Osmo_Teretosporea
Pp_Diaphoretickes
Osmo_Fungi
Other_Holomycota
Osmo_Pseudofungi
Cp_Diaphoretickes
Other_Diaphoretickes
Other_Stramenopiles
Other_Amorphea
Osmo_Labyrinthulea
Other
Non-eukaryotes
Other_SAR

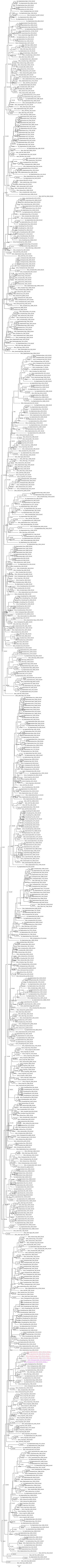

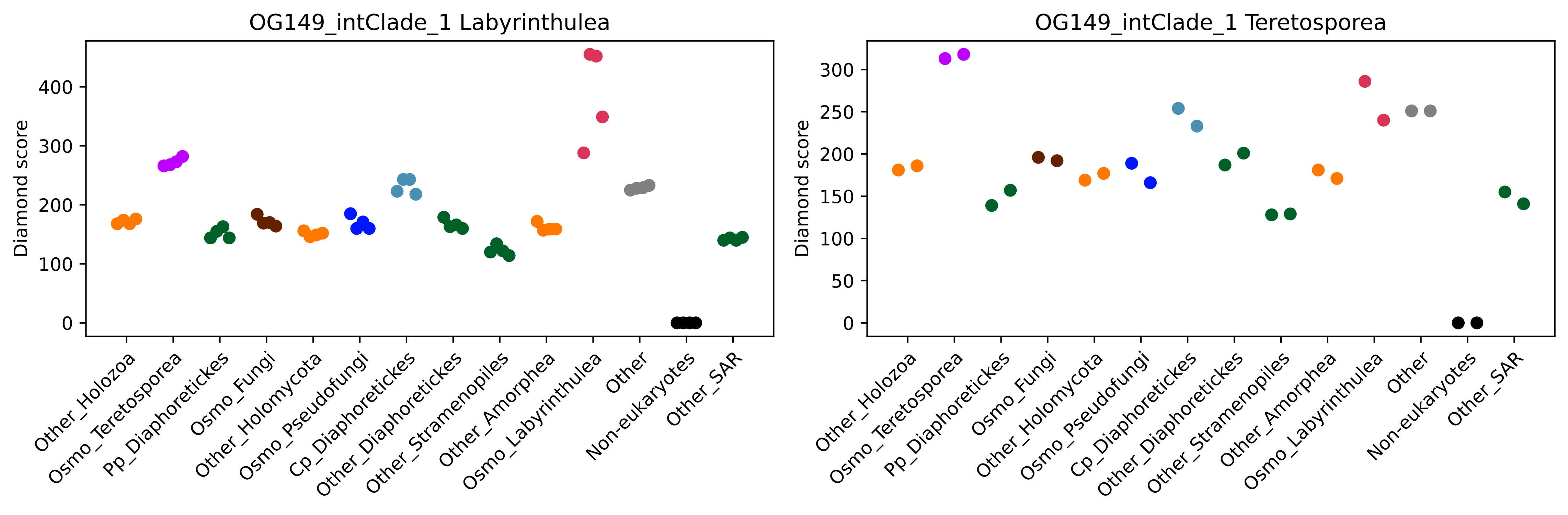

OG149_intClade_1 Labyrinthulea
Diamond score
0
100
200
300
400
Other_Holozoa
Osmo_Teretosporea
Pp_Diaphoretickes
Osmo_Fungi
Other_Holomycota
Osmo_Pseudofungi
Cp_Diaphoretickes
Other_Diaphoretickes
Other_Stramenopiles
Other_Amorphea
Osmo_Labyrinthulea
Other
Non-eukaryotes
Other_SAR
OG149_intClade_1 Teretosporea
Diamond score
0
50
100
150
200
250
300
Other_Holozoa
Osmo_Teretosporea
Pp_Diaphoretickes
Osmo_Fungi
Other_Holomycota
Osmo_Pseudofungi
Cp_Diaphoretickes
Other_Diaphoretickes
Other_Stramenopiles
Other_Amorphea
Osmo_Labyrinthulea
Other
Non-eukaryotes
Other_SAR

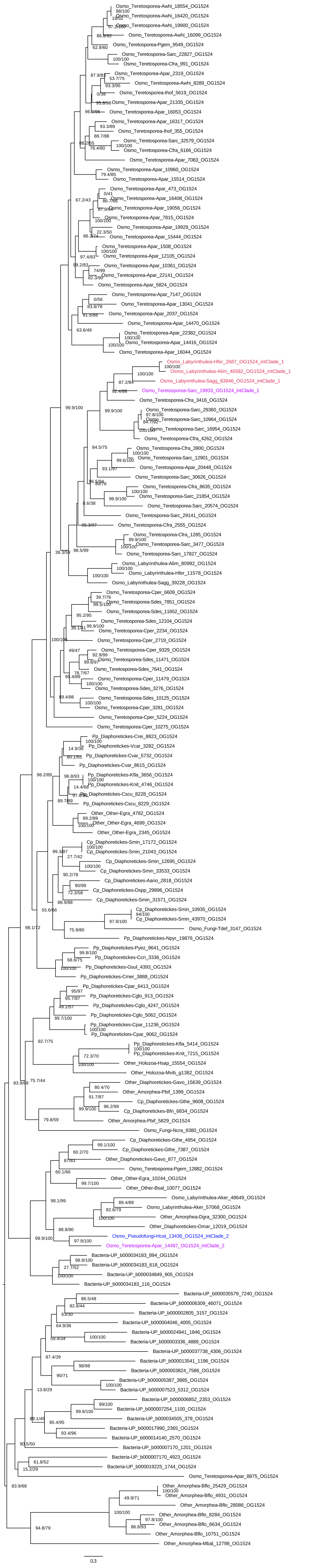
Osmo_Teretosporea-Awhi_18554_OG1524
Osmo_Teretosporea-Awhi_16420_OG1524
Osmo_Teretosporea-Awhi_19900_OG1524
Osmo_Teretosporea-Awhi_16099_OG1524
Osmo_Teretosporea-Pgem_9549_OG1524
Osmo_Teretosporea-Sarc_22827_OG1524
Osmo_Teretosporea-Cfra_991_OG1524
Osmo_Teretosporea-Apar_2319_OG1524
Osmo_Teretosporea-Awhi_8289_OG1524
Osmo_Teretosporea-Ihof_5619_OG1524
Osmo_Teretosporea-Apar_21335_OG1524
Osmo_Teretosporea-Apar_16053_OG1524
Osmo_Teretosporea-Apar_16317_OG1524
Osmo_Teretosporea-Ihof_355_OG1524
Osmo_Teretosporea-Sarc_32579_OG1524
Osmo_Teretosporea-Cfra_6166_OG1524
Osmo_Teretosporea-Apar_7083_OG1524
Osmo_Teretosporea-Apar_10960_OG1524
Osmo_Teretosporea-Apar_15514_OG1524
Osmo_Teretosporea-Apar_473_OG1524
Osmo_Teretosporea-Apar_16408_OG1524
Osmo_Teretosporea-Apar_19056_OG1524
Osmo_Teretosporea-Apar_7815_OG1524
Osmo_Teretosporea-Apar_19929_OG1524
Osmo_Teretosporea-Apar_15444_OG1524
Osmo_Teretosporea-Apar_1508_OG1524
Osmo_Teretosporea-Apar_12105_OG1524
Osmo_Teretosporea-Apar_10361_OG1524
Osmo_Teretosporea-Apar_22141_OG1524
Osmo_Teretosporea-Apar_5824_OG1524
Osmo_Teretosporea-Apar_7147_OG1524
Osmo_Teretosporea-Apar_13041_OG1524
Osmo_Teretosporea-Apar_2037_OG1524
Osmo_Teretosporea-Apar_14470_OG1524
Osmo_Teretosporea-Apar_22382_OG1524
Osmo_Teretosporea-Apar_14416_OG1524
Osmo_Teretosporea-Apar_16044_OG1524
Osmo_Labyrinthulea-Hfer_2687_OG1524_intClade_1
Osmo_Labyrinthulea-Alim_46582_OG1524_intClade_1
Osmo_Labyrinthulea-Sagg_83846_OG1524_intClade_1
Osmo_Teretosporea-Sarc_19933_OG1524_intClade_1
Osmo_Teretosporea-Cfra_3416_OG1524
Osmo_Teretosporea-Sarc_29360_OG1524
Osmo_Teretosporea-Sarc_10964_OG1524
Osmo_Teretosporea-Sarc_16954_OG1524
Osmo_Teretosporea-Cfra_4262_OG1524
Osmo_Teretosporea-Cfra_3900_OG1524
Osmo_Teretosporea-Sarc_12901_OG1524
Osmo_Teretosporea-Apar_20448_OG1524
Osmo_Teretosporea-Sarc_30626_OG1524
Osmo_Teretosporea-Cfra_8635_OG1524
Osmo_Teretosporea-Sarc_21854_OG1524
Osmo_Teretosporea-Sarc_20574_OG1524
Osmo_Teretosporea-Sarc_29141_OG1524
Osmo_Teretosporea-Cfra_2555_OG1524
Osmo_Teretosporea-Cfra_1285_OG1524
Osmo_Teretosporea-Sarc_3477_OG1524
Osmo_Teretosporea-Sarc_17827_OG1524
Osmo_Labyrinthulea-Alim_80982_OG1524
Osmo_Labyrinthulea-Hfer_11578_OG1524
Osmo_Labyrinthulea-Sagg_39228_OG1524
Osmo_Teretosporea-Cper_6609_OG1524
Osmo_Teretosporea-Sdes_7851_OG1524
Osmo_Teretosporea-Sdes_11652_OG1524
Osmo_Teretosporea-Sdes_12104_OG1524
Osmo_Teretosporea-Cper_2234_OG1524
Osmo_Teretosporea-Cper_2719_OG1524
Osmo_Teretosporea-Cper_9329_OG1524
Osmo_Teretosporea-Sdes_11471_OG1524
Osmo_Teretosporea-Sdes_7641_OG1524
Osmo_Teretosporea-Cper_11479_OG1524
Osmo_Teretosporea-Sdes_3276_OG1524
Osmo_Teretosporea-Sdes_10125_OG1524
Osmo_Teretosporea-Cper_3281_OG1524
Osmo_Teretosporea-Cper_5224_OG1524
Osmo_Teretosporea-Cper_10275_OG1524
Pp_Diaphoretickes-Crei_8823_OG1524
Pp_Diaphoretickes-Vcar_3282_OG1524
Pp_Diaphoretickes-Cvar_5732_OG1524
Pp_Diaphoretickes-Cvar_8615_OG1524
Pp_Diaphoretickes-Kfla_3656_OG1524
Pp_Diaphoretickes-Knit_4746_OG1524
Pp_Diaphoretickes-Cscu_8228_OG1524
Pp_Diaphoretickes-Cscu_8229_OG1524
Other_Other-Egra_4782_OG1524
Other_Other-Egra_4699_OG1524
Other_Other-Egra_2345_OG1524
Cp_Diaphoretickes-Smin_17172_OG1524
Cp_Diaphoretickes-Smin_21043_OG1524
Cp_Diaphoretickes-Smin_12695_OG1524
Cp_Diaphoretickes-Smin_33533_OG1524
Cp_Diaphoretickes-Aano_2818_OG1524
Cp_Diaphoretickes-Dspp_29896_OG1524
Cp_Diaphoretickes-Smin_31571_OG1524
Cp_Diaphoretickes-Smin_10935_OG1524
Cp_Diaphoretickes-Smin_43970_OG1524
Osmo_Fungi-Tdef_3147_OG1524
Pp_Diaphoretickes-Npyr_19876_OG1524
Pp_Diaphoretickes-Pyez_9641_OG1524
Pp_Diaphoretickes-Ccri_3336_OG1524
Pp_Diaphoretickes-Gsul_4393_OG1524
Pp_Diaphoretickes-Cmer_3888_OG1524
Pp_Diaphoretickes-Cpar_6413_OG1524
Pp_Diaphoretickes-Cglo_913_OG1524
Pp_Diaphoretickes-Cglo_4247_OG1524
Pp_Diaphoretickes-Cglo_5062_OG1524
Pp_Diaphoretickes-Cpar_11236_OG1524
Pp_Diaphoretickes-Cpar_9062_OG1524
Pp_Diaphoretickes-Kfla_5414_OG1524
Pp_Diaphoretickes-Knit_7215_OG1524
Other_Holozoa-Hsap_15554_OG1524
Other_Holozoa-Mvib_g1382_OG1524
Other_Diaphoretickes-Gavo_15639_OG1524
Other_Amorphea-Pbif_1399_OG1524
Cp_Diaphoretickes-Gthe_9608_OG1524
Cp_Diaphoretickes-Bfri_6834_OG1524
Other_Amorphea-Pbif_5829_OG1524
Osmo_Fungi-Ncra_9380_OG1524
Cp_Diaphoretickes-Gthe_4954_OG1524
Cp_Diaphoretickes-Gthe_7387_OG1524
Other_Diaphoretickes-Gavo_877_OG1524
Osmo_Teretosporea-Pgem_12882_OG1524
Other_Other-Egra_10244_OG1524
Other_Other-Bsal_10077_OG1524
Osmo_Labyrinthulea-Aker_48649_OG1524
Osmo_Labyrinthulea-Aker_57068_OG1524
Other_Amorphea-Dgra_32300_OG1524
Other_Diaphoretickes-Omar_12019_OG1524
Osmo_Pseudofungi-Hcat_13436_OG1524_intClade_2
Osmo_Teretosporea-Apar_14497_OG1524_intClade_2
Bacteria-UP_b000034183_894_OG1524
Bacteria-UP_b000034183_618_OG1524
Bacteria-UP_b000034849_905_OG1524
Bacteria-UP_b000034183_116_OG1524
Bacteria-UP_b000035579_7240_OG1524
Bacteria-UP_b000006309_46071_OG1524
Bacteria-UP_b000002805_3157_OG1524
Bacteria-UP_b000004046_4005_OG1524
Bacteria-UP_b000024941_1846_OG1524
Bacteria-UP_b000003336_4889_OG1524
Bacteria-UP_b000037738_4306_OG1524
Bacteria-UP_b000013541_1196_OG1524
Bacteria-UP_b000003824_7586_OG1524
Bacteria-UP_b000005387_3885_OG1524
Bacteria-UP_b000007523_5312_OG1524
Bacteria-UP_b000006852_2353_OG1524
Bacteria-UP_b000007254_1100_OG1524
Bacteria-UP_b000034505_379_OG1524
Bacteria-UP_b000017990_2365_OG1524
Bacteria-UP_b000014140_2570_OG1524
Bacteria-UP_b000007170_1201_OG1524
Bacteria-UP_b000007170_4923_OG1524
Bacteria-UP_b000019225_1744_OG1524
Osmo_Teretosporea-Apar_8875_OG1524
Other_Amorphea-Bflo_25429_OG1524
Other_Amorphea-Bflo_4931_OG1524
Other_Amorphea-Bflo_28086_OG1524
Other_Amorphea-Bflo_8284_OG1524
Other_Amorphea-Bflo_6634_OG1524
Other_Amorphea-Bflo_10751_OG1524
Other_Amorphea-Mbal_12798_OG1524
0.3

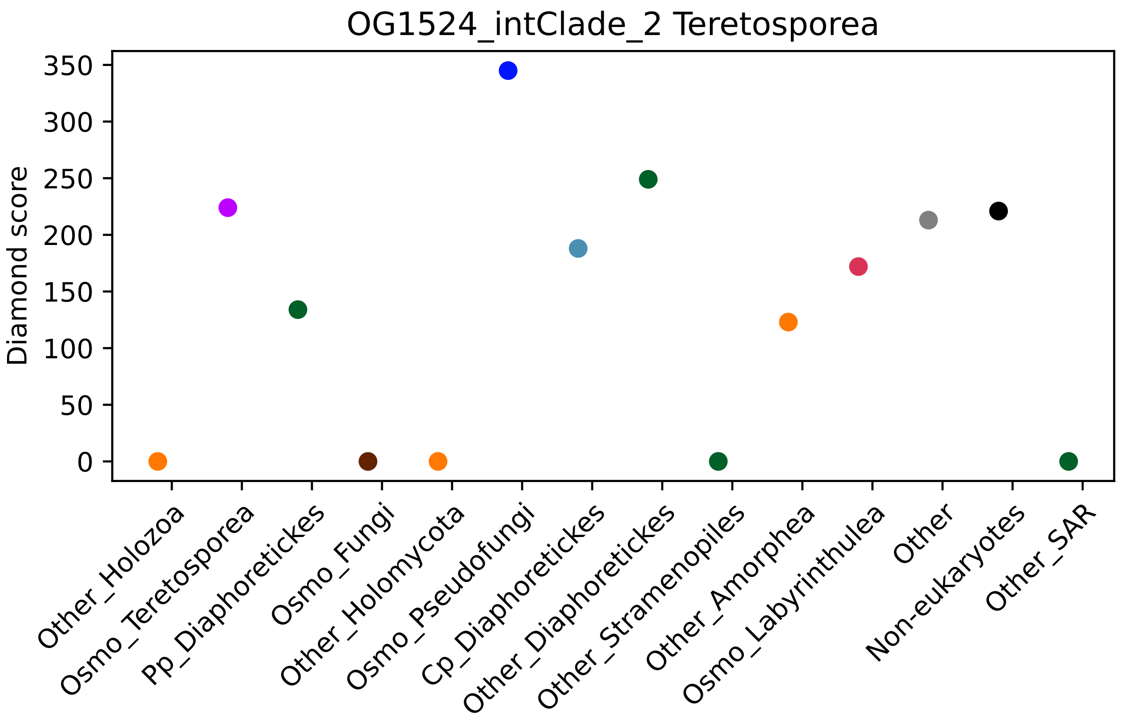
OG1524_intClade_2 Teretosporea
Diamond score
0
50
100
150
200
250
300
350
Other_Holozoa
Osmo_Teretosporea
Pp_Diaphoretickes
Osmo_Fungi
Other_Holomycota
Osmo_Pseudofungi
Cp_Diaphoretickes
Other_Diaphoretickes
Other_Stramenopiles
Other_Amorphea
Osmo_Labyrinthulea
Other
Non-eukaryotes
Other_SAR
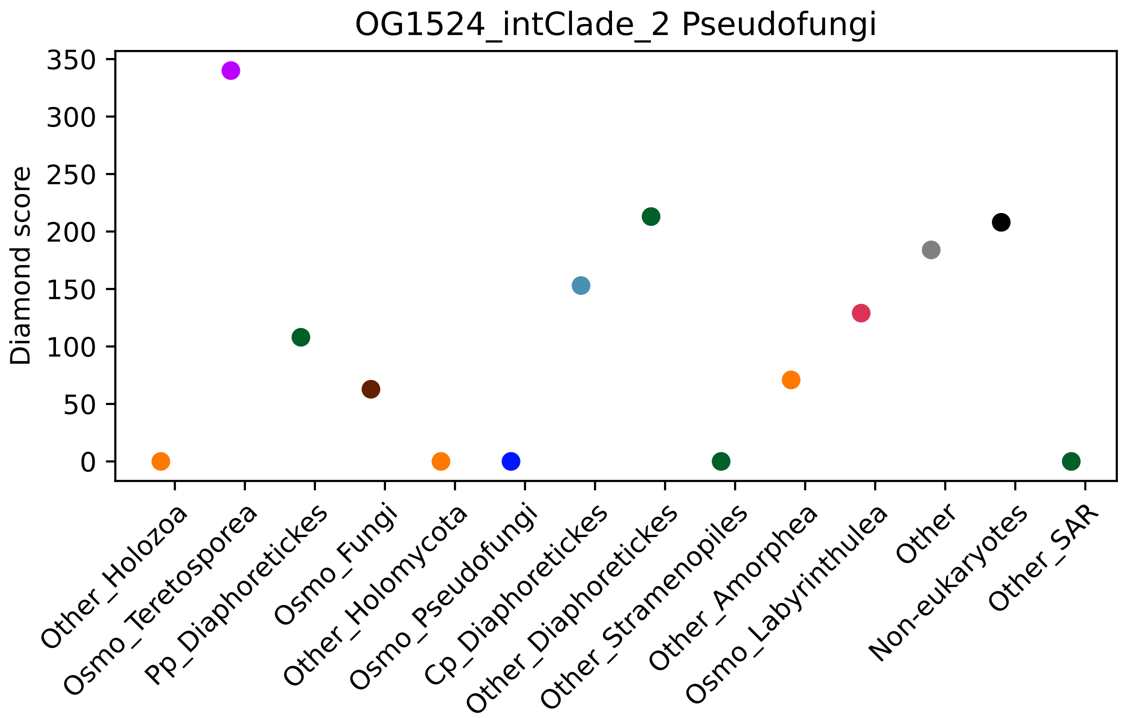
OG1524_intClade_2 Pseudofungi
Diamond score
0
50
100
150
200
250
300
350
Other_Holozoa
Osmo_Teretosporea
Pp_Diaphoretickes
Osmo_Fungi
Other_Holomycota
Osmo_Pseudofungi
Cp_Diaphoretickes
Other_Diaphoretickes
Other_Stramenopiles
Other_Amorphea
Osmo_Labyrinthulea
Other
Non-eukaryotes
Other_SAR

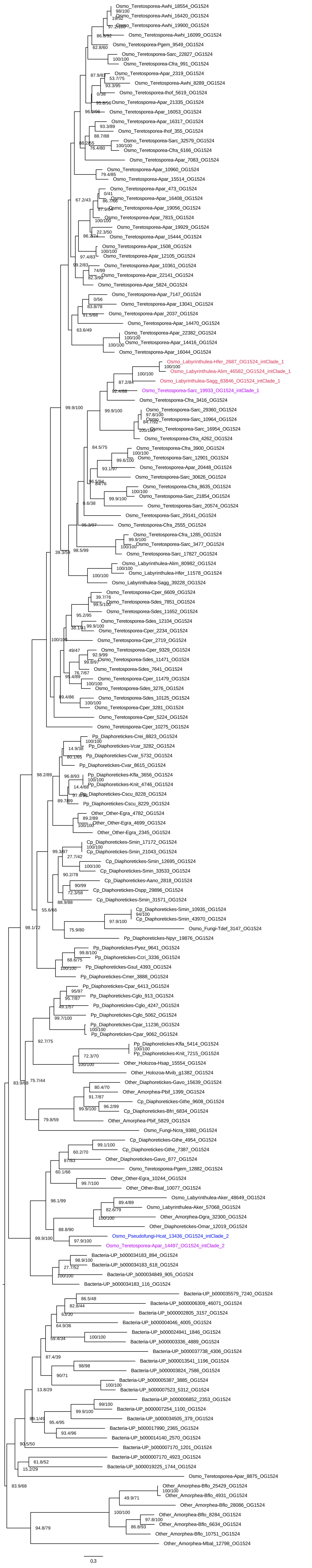

Osmo_Teretosporea-Awhi_18554_OG1524
Osmo_Teretosporea-Awhi_16420_OG1524
Osmo_Teretosporea-Awhi_19900_OG1524
Osmo_Teretosporea-Awhi_16099_OG1524
Osmo_Teretosporea-Pgem_9549_OG1524
Osmo_Teretosporea-Sarc_22827_OG1524
Osmo_Teretosporea-Cfra_991_OG1524
Osmo_Teretosporea-Apar_2319_OG1524
Osmo_Teretosporea-Awhi_8289_OG1524
Osmo_Teretosporea-Ihof_5619_OG1524
Osmo_Teretosporea-Apar_21335_OG1524
Osmo_Teretosporea-Apar_16053_OG1524
Osmo_Teretosporea-Apar_16317_OG1524
Osmo_Teretosporea-Ihof_355_OG1524
Osmo_Teretosporea-Sarc_32579_OG1524
Osmo_Teretosporea-Cfra_6166_OG1524
Osmo_Teretosporea-Apar_7083_OG1524
Osmo_Teretosporea-Apar_10960_OG1524
Osmo_Teretosporea-Apar_15514_OG1524
Osmo_Teretosporea-Apar_473_OG1524
Osmo_Teretosporea-Apar_16408_OG1524
Osmo_Teretosporea-Apar_19056_OG1524
Osmo_Teretosporea-Apar_7815_OG1524
Osmo_Teretosporea-Apar_19929_OG1524
Osmo_Teretosporea-Apar_15444_OG1524
Osmo_Teretosporea-Apar_1508_OG1524
Osmo_Teretosporea-Apar_12105_OG1524
Osmo_Teretosporea-Apar_10361_OG1524
Osmo_Teretosporea-Apar_22141_OG1524
Osmo_Teretosporea-Apar_5824_OG1524
Osmo_Teretosporea-Apar_7147_OG1524
Osmo_Teretosporea-Apar_13041_OG1524
Osmo_Teretosporea-Apar_2037_OG1524
Osmo_Teretosporea-Apar_14470_OG1524
Osmo_Teretosporea-Apar_22382_OG1524
Osmo_Teretosporea-Apar_14416_OG1524
Osmo_Teretosporea-Apar_16044_OG1524
Osmo_Labyrinthulea-Hfer_2687_OG1524_intClade_1
Osmo_Labyrinthulea-Alim_46582_OG1524_intClade_1
Osmo_Labyrinthulea-Sagg_83846_OG1524_intClade_1
Osmo_Teretosporea-Sarc_19933_OG1524_intClade_1
Osmo_Teretosporea-Cfra_3416_OG1524
Osmo_Teretosporea-Sarc_29360_OG1524
Osmo_Teretosporea-Sarc_10964_OG1524
Osmo_Teretosporea-Sarc_16954_OG1524
Osmo_Teretosporea-Cfra_4262_OG1524
Osmo_Teretosporea-Cfra_3900_OG1524
Osmo_Teretosporea-Sarc_12901_OG1524
Osmo_Teretosporea-Apar_20448_OG1524
Osmo_Teretosporea-Sarc_30626_OG1524
Osmo_Teretosporea-Cfra_8635_OG1524
Osmo_Teretosporea-Sarc_21854_OG1524
Osmo_Teretosporea-Sarc_20574_OG1524
Osmo_Teretosporea-Sarc_29141_OG1524
Osmo_Teretosporea-Cfra_2555_OG1524
Osmo_Teretosporea-Cfra_1285_OG1524
Osmo_Teretosporea-Sarc_3477_OG1524
Osmo_Teretosporea-Sarc_17827_OG1524
Osmo_Labyrinthulea-Alim_80982_OG1524
Osmo_Labyrinthulea-Hfer_11578_OG1524
Osmo_Labyrinthulea-Sagg_39228_OG1524
Osmo_Teretosporea-Cper_6609_OG1524
Osmo_Teretosporea-Sdes_7851_OG1524
Osmo_Teretosporea-Sdes_11652_OG1524
Osmo_Teretosporea-Sdes_12104_OG1524
Osmo_Teretosporea-Cper_2234_OG1524
Osmo_Teretosporea-Cper_2719_OG1524
Osmo_Teretosporea-Cper_9329_OG1524
Osmo_Teretosporea-Sdes_11471_OG1524
Osmo_Teretosporea-Sdes_7641_OG1524
Osmo_Teretosporea-Cper_11479_OG1524
Osmo_Teretosporea-Sdes_3276_OG1524
Osmo_Teretosporea-Sdes_10125_OG1524
Osmo_Teretosporea-Cper_3281_OG1524
Osmo_Teretosporea-Cper_5224_OG1524
Osmo_Teretosporea-Cper_10275_OG1524
Pp_Diaphoretickes-Crei_8823_OG1524
Pp_Diaphoretickes-Vcar_3282_OG1524
Pp_Diaphoretickes-Cvar_5732_OG1524
Pp_Diaphoretickes-Cvar_8615_OG1524
Pp_Diaphoretickes-Kfla_3656_OG1524
Pp_Diaphoretickes-Knit_4746_OG1524
Pp_Diaphoretickes-Cscu_8228_OG1524
Pp_Diaphoretickes-Cscu_8229_OG1524
Other_Other-Egra_4782_OG1524
Other_Other-Egra_4699_OG1524
Other_Other-Egra_2345_OG1524
Cp_Diaphoretickes-Smin_17172_OG1524
Cp_Diaphoretickes-Smin_21043_OG1524
Cp_Diaphoretickes-Smin_12695_OG1524
Cp_Diaphoretickes-Smin_33533_OG1524
Cp_Diaphoretickes-Aano_2818_OG1524
Cp_Diaphoretickes-Dspp_29896_OG1524
Cp_Diaphoretickes-Smin_31571_OG1524
Cp_Diaphoretickes-Smin_10935_OG1524
Cp_Diaphoretickes-Smin_43970_OG1524
Osmo_Fungi-Tdef_3147_OG1524
Pp_Diaphoretickes-Npyr_19876_OG1524
Pp_Diaphoretickes-Pyez_9641_OG1524
Pp_Diaphoretickes-Ccri_3336_OG1524
Pp_Diaphoretickes-Gsul_4393_OG1524
Pp_Diaphoretickes-Cmer_3888_OG1524
Pp_Diaphoretickes-Cpar_6413_OG1524
Pp_Diaphoretickes-Cglo_913_OG1524
Pp_Diaphoretickes-Cglo_4247_OG1524
Pp_Diaphoretickes-Cglo_5062_OG1524
Pp_Diaphoretickes-Cpar_11236_OG1524
Pp_Diaphoretickes-Cpar_9062_OG1524
Pp_Diaphoretickes-Kfla_5414_OG1524
Pp_Diaphoretickes-Knit_7215_OG1524
Other_Holozoa-Hsap_15554_OG1524
Other_Holozoa-Mvib_g1382_OG1524
Other_Diaphoretickes-Gavo_15639_OG1524
Other_Amorphea-Pbif_1399_OG1524
Cp_Diaphoretickes-Gthe_9608_OG1524
Cp_Diaphoretickes-Bfri_6834_OG1524
Other_Amorphea-Pbif_5829_OG1524
Osmo_Fungi-Ncra_9380_OG1524
Cp_Diaphoretickes-Gthe_4954_OG1524
Cp_Diaphoretickes-Gthe_7387_OG1524
Other_Diaphoretickes-Gavo_877_OG1524
Osmo_Teretosporea-Pgem_12882_OG1524
Other_Other-Egra_10244_OG1524
Other_Other-Bsal_10077_OG1524
Osmo_Labyrinthulea-Aker_48649_OG1524
Osmo_Labyrinthulea-Aker_57068_OG1524
Other_Amorphea-Dgra_32300_OG1524
Other_Diaphoretickes-Omar_12019_OG1524
Osmo_Pseudofungi-Hcat_13436_OG1524_intClade_2
Osmo_Teretosporea-Apar_14497_OG1524_intClade_2
Bacteria-UP_b000034183_894_OG1524
Bacteria-UP_b000034183_618_OG1524
Bacteria-UP_b000034849_905_OG1524
Bacteria-UP_b000034183_116_OG1524
Bacteria-UP_b000035579_7240_OG1524
Bacteria-UP_b000006309_46071_OG1524
Bacteria-UP_b000002805_3157_OG1524
Bacteria-UP_b000004046_4005_OG1524
Bacteria-UP_b000024941_1846_OG1524
Bacteria-UP_b000003336_4889_OG1524
Bacteria-UP_b000037738_4306_OG1524
Bacteria-UP_b000013541_1196_OG1524
Bacteria-UP_b000003824_7586_OG1524
Bacteria-UP_b000005387_3885_OG1524
Bacteria-UP_b000007523_5312_OG1524
Bacteria-UP_b000006852_2353_OG1524
Bacteria-UP_b000007254_1100_OG1524
Bacteria-UP_b000034505_379_OG1524
Bacteria-UP_b000017990_2365_OG1524
Bacteria-UP_b000014140_2570_OG1524
Bacteria-UP_b000007170_1201_OG1524
Bacteria-UP_b000007170_4923_OG1524
Bacteria-UP_b000019225_1744_OG1524
Osmo_Teretosporea-Apar_8875_OG1524
Other_Amorphea-Bflo_25429_OG1524
Other_Amorphea-Bflo_4931_OG1524
Other_Amorphea-Bflo_28086_OG1524
Other_Amorphea-Bflo_8284_OG1524
Other_Amorphea-Bflo_6634_OG1524
Other_Amorphea-Bflo_10751_OG1524
Other_Amorphea-Mbal_12798_OG1524
0.3

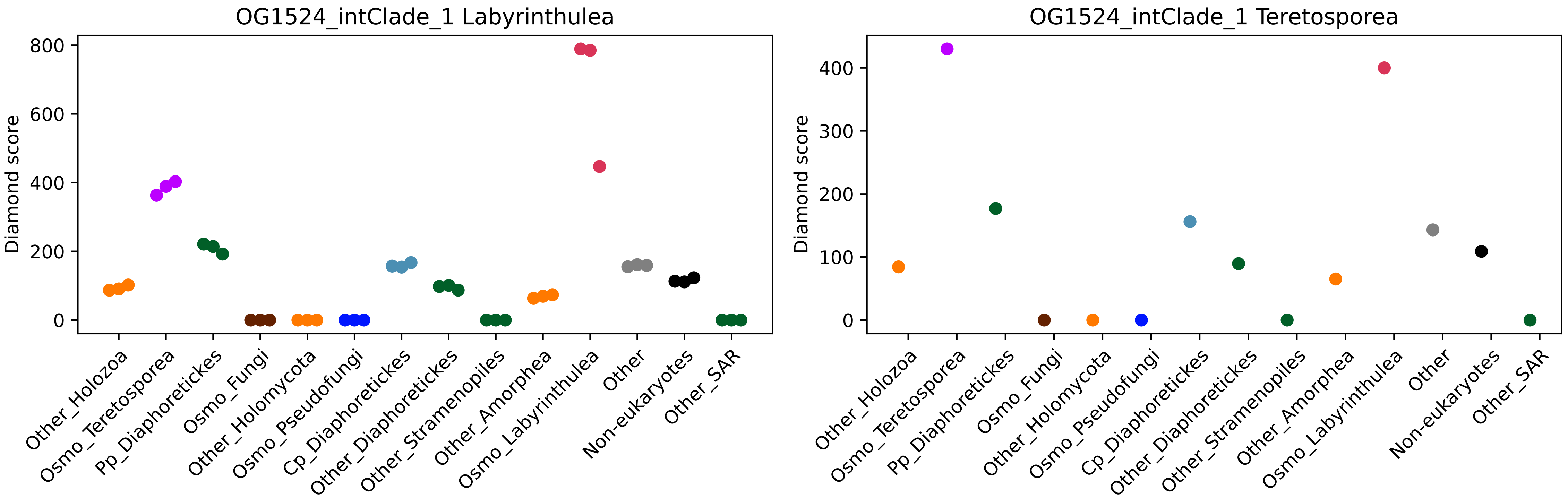
OG1524_intClade_1 Labyrinthulea
OG1524_intClade_1 Teretosporea
Diamond score
0
200
400
600
800
100
300
Other_Holozoa
Osmo_Teretosporea
Pp_Diaphoretickes
Osmo_Fungi
Other_Holomycota
Osmo_Pseudofungi
Cp_Diaphoretickes
Other_Diaphoretickes
Other_Stramenopiles
Other_Amorphea
Osmo_Labyrinthulea
Other
Non-eukaryotes
Other_SAR

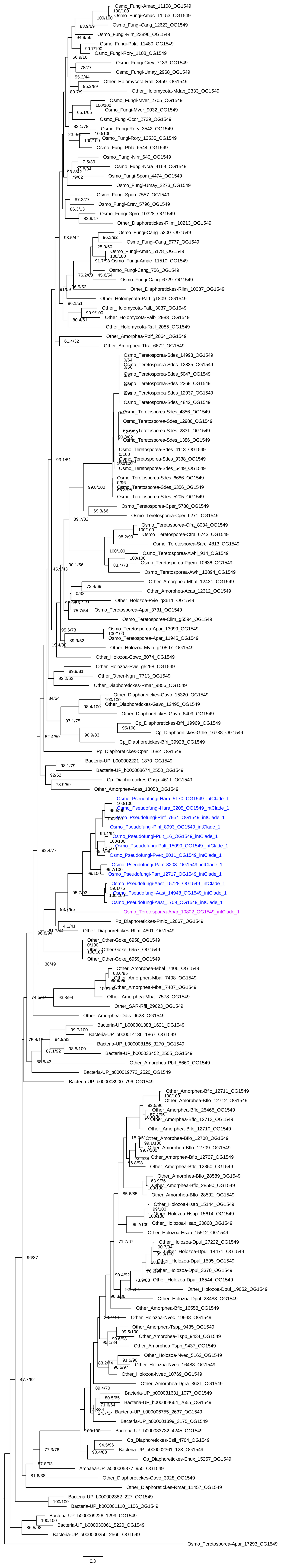

Osmo_Fungi-Amac_11108_OG1549
Osmo_Fungi-Amac_11153_OG1549
Osmo_Fungi-Cang_12623_OG1549
Osmo_Fungi-Rirr_23896_OG1549
Osmo_Fungi-Pbla_11480_OG1549
Osmo_Fungi-Rory_1108_OG1549
Osmo_Fungi-Crev_7133_OG1549
Osmo_Fungi-Umay_2968_OG1549
Other_Holomycota-Rall_3459_OG1549
Other_Holomycota-Mdap_2333_OG1549
Osmo_Fungi-Mver_2705_OG1549
Osmo_Fungi-Mver_9032_OG1549
Osmo_Fungi-Ccor_2739_OG1549
Osmo_Fungi-Rory_3542_OG1549
Osmo_Fungi-Rory_12535_OG1549
Osmo_Fungi-Pbla_6544_OG1549
Osmo_Fungi-Nirr_640_OG1549
Osmo_Fungi-Ncra_4169_OG1549
Osmo_Fungi-Spom_4474_OG1549
Osmo_Fungi-Umay_2273_OG1549
Osmo_Fungi-Spun_7557_OG1549
Osmo_Fungi-Crev_5796_OG1549
Osmo_Fungi-Gpro_10328_OG1549
Other_Diaphoretickes-Rlim_10213_OG1549
Osmo_Fungi-Cang_5300_OG1549
Osmo_Fungi-Cang_5777_OG1549
Osmo_Fungi-Amac_5178_OG1549
Osmo_Fungi-Amac_11510_OG1549
Osmo_Fungi-Cang_756_OG1549
Osmo_Fungi-Cang_6729_OG1549
Other_Diaphoretickes-Rlim_10037_OG1549
Other_Holomycota-Patl_g1809_OG1549
Other_Holomycota-Falb_3037_OG1549
Other_Holomycota-Falb_2983_OG1549
Other_Holomycota-Rall_2085_OG1549
Other_Amorphea-Pbif_2064_OG1549
Other_Amorphea-Ttra_6672_OG1549
Osmo_Teretosporea-Sdes_14993_OG1549
Osmo_Teretosporea-Sdes_12835_OG1549
Osmo_Teretosporea-Sdes_5047_OG1549
Osmo_Teretosporea-Sdes_2269_OG1549
Osmo_Teretosporea-Sdes_12937_OG1549
Osmo_Teretosporea-Sdes_4842_OG1549
Osmo_Teretosporea-Sdes_4356_OG1549
Osmo_Teretosporea-Sdes_12986_OG1549
Osmo_Teretosporea-Sdes_2831_OG1549
Osmo_Teretosporea-Sdes_1386_OG1549
Osmo_Teretosporea-Sdes_4113_OG1549
Osmo_Teretosporea-Sdes_9338_OG1549
Osmo_Teretosporea-Sdes_6449_OG1549
Osmo_Teretosporea-Sdes_6686_OG1549
Osmo_Teretosporea-Sdes_6356_OG1549
Osmo_Teretosporea-Sdes_5205_OG1549
Osmo_Teretosporea-Cper_5780_OG1549
Osmo_Teretosporea-Cper_6271_OG1549
Osmo_Teretosporea-Cfra_8034_OG1549
Osmo_Teretosporea-Cfra_6743_OG1549
Osmo_Teretosporea-Sarc_4813_OG1549
Osmo_Teretosporea-Awhi_914_OG1549
Osmo_Teretosporea-Pgem_10636_OG1549
Osmo_Teretosporea-Awhi_13894_OG1549
Other_Amorphea-Mbal_12431_OG1549
Other_Amorphea-Acas_12312_OG1549
Other_Holozoa-Pvie_g3611_OG1549
Osmo_Teretosporea-Apar_3731_OG1549
Osmo_Teretosporea-Clim_g5594_OG1549
Osmo_Teretosporea-Apar_13099_OG1549
Osmo_Teretosporea-Apar_11945_OG1549
Other_Holozoa-Mvib_g10597_OG1549
Other_Holozoa-Cowc_8074_OG1549
Other_Holozoa-Pvie_g5298_OG1549
Other_Other-Ngru_7713_OG1549
Other_Diaphoretickes-Rmar_9856_OG1549
Other_Diaphoretickes-Gavo_15320_OG1549
Other_Diaphoretickes-Gavo_12495_OG1549
Other_Diaphoretickes-Gavo_6409_OG1549
Cp_Diaphoretickes-Bfri_19969_OG1549
Cp_Diaphoretickes-Gthe_16738_OG1549
Cp_Diaphoretickes-Bfri_39928_OG1549
Pp_Diaphoretickes-Cpar_1682_OG1549
Bacteria-UP_b000002221_1870_OG1549
Bacteria-UP_b000008674_2550_OG1549
Cp_Diaphoretickes-Chsp_4611_OG1549
Other_Amorphea-Acas_13053_OG1549
Osmo_Pseudofungi-Hara_5170_OG1549_intClade_1
Osmo_Pseudofungi-Hara_3205_OG1549_intClade_1
Osmo_Pseudofungi-Pinf_7954_OG1549_intClade_1
Osmo_Pseudofungi-Pinf_8993_OG1549_intClade_1
Osmo_Pseudofungi-Pult_16_OG1549_intClade_1
Osmo_Pseudofungi-Pult_15099_OG1549_intClade_1
Osmo_Pseudofungi-Pvex_8011_OG1549_intClade_1
Osmo_Pseudofungi-Parr_8208_OG1549_intClade_1
Osmo_Pseudofungi-Parr_12717_OG1549_intClade_1
Osmo_Pseudofungi-Aast_15728_OG1549_intClade_1
Osmo_Pseudofungi-Aast_14948_OG1549_intClade_1
Osmo_Pseudofungi-Aast_1709_OG1549_intClade_1
Osmo_Teretosporea-Apar_10802_OG1549_intClade_1
Pp_Diaphoretickes-Pmic_12067_OG1549
Other_Diaphoretickes-Rlim_4801_OG1549
Other_Other-Goke_6958_OG1549
Other_Other-Goke_6957_OG1549
Other_Other-Goke_6959_OG1549
Other_Amorphea-Mbal_7406_OG1549
Other_Amorphea-Mbal_7408_OG1549
Other_Amorphea-Mbal_7407_OG1549
Other_Amorphea-Mbal_7578_OG1549
Other_SAR-Rfil_29623_OG1549
Other_Amorphea-Ddis_9628_OG1549
Bacteria-UP_b000001383_1621_OG1549
Bacteria-UP_b000014136_1867_OG1549
Bacteria-UP_b000008186_3270_OG1549
Bacteria-UP_b000033452_2505_OG1549
Other_Amorphea-Pbif_8660_OG1549
Bacteria-UP_b000019772_2520_OG1549
Bacteria-UP_b000003900_796_OG1549
Other_Amorphea-Bflo_12711_OG1549
Other_Amorphea-Bflo_12712_OG1549
Other_Holozoa-Bflo_25465_OG1549
Other_Amorphea-Bflo_12713_OG1549
Other_Amorphea-Bflo_12710_OG1549
Other_Amorphea-Bflo_12708_OG1549
Other_Amorphea-Bflo_12709_OG1549
Other_Amorphea-Bflo_12707_OG1549
Other_Amorphea-Bflo_12850_OG1549
Other_Amorphea-Bflo_28589_OG1549
Other_Amorphea-Bflo_28590_OG1549
Other_Amorphea-Bflo_28592_OG1549
Other_Holozoa-Hsap_15144_OG1549
Other_Holozoa-Hsap_15614_OG1549
Other_Holozoa-Hsap_20868_OG1549
Other_Holozoa-Hsap_15512_OG1549
Other_Holozoa-Dpul_27222_OG1549
Other_Holozoa-Dpul_14471_OG1549
Other_Holozoa-Dpul_1595_OG1549
Other_Holozoa-Dpul_3370_OG1549
Other_Holozoa-Dpul_16544_OG1549
Other_Holozoa-Dpul_19052_OG1549
Other_Holozoa-Dpul_23483_OG1549
Other_Amorphea-Bflo_16558_OG1549
Other_Holozoa-Nvec_19948_OG1549
Other_Amorphea-Tspp_9435_OG1549
Other_Amorphea-Tspp_9434_OG1549
Other_Amorphea-Tspp_9437_OG1549
Other_Holozoa-Nvec_5162_OG1549
Other_Holozoa-Nvec_16483_OG1549
Other_Holozoa-Nvec_10769_OG1549
Other_Amorphea-Dgra_3621_OG1549
Bacteria-UP_b000031631_1077_OG1549
Bacteria-UP_b000004664_2655_OG1549
Bacteria-UP_b000006755_2637_OG1549
Bacteria-UP_b000001399_3175_OG1549
Bacteria-UP_b000033732_4245_OG1549
Cp_Diaphoretickes-Esil_4704_OG1549
Bacteria-UP_b000002361_123_OG1549
Cp_Diaphoretickes-Ehux_15257_OG1549
Archaea-UP_a000005877_950_OG1549
Other_Diaphoretickes-Gavo_3928_OG1549
Other_Diaphoretickes-Rmar_11457_OG1549
Bacteria-UP_b000002382_227_OG1549
Bacteria-UP_b000001110_1106_OG1549
Bacteria-UP_b000009226_1299_OG1549
Bacteria-UP_b000030061_5220_OG1549
Bacteria-UP_b000000256_2566_OG1549
Osmo_Teretosporea-Apar_17293_OG1549
0.3

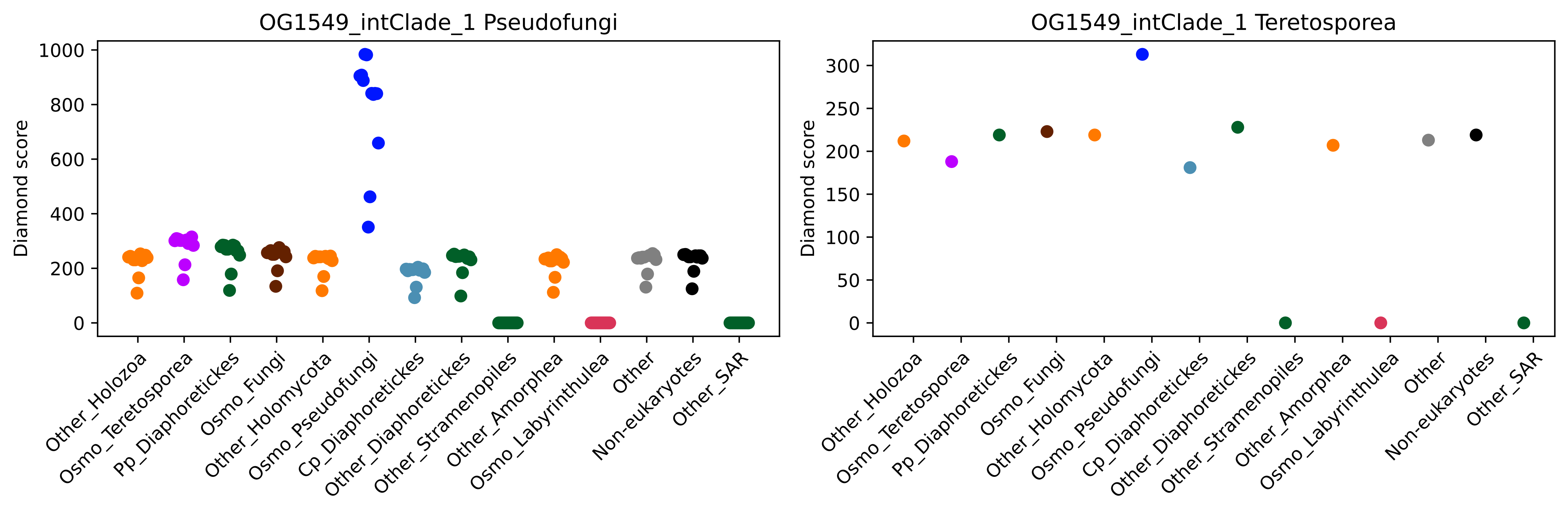

OG1549_intClade_1 Pseudofungi
OG1549_intClade_1 Teretosporea
Diamond score
Other_Holozoa
Osmo_Teretosporea
Pp_Diaphoretickes
Osmo_Fungi
Other_Holomycota
Osmo_Pseudofungi
Cp_Diaphoretickes
Other_Diaphoretickes
Other_Stramenopiles
Other_Amorphea
Osmo_Labyrinthulea
Other
Non-eukaryotes
Other_SAR

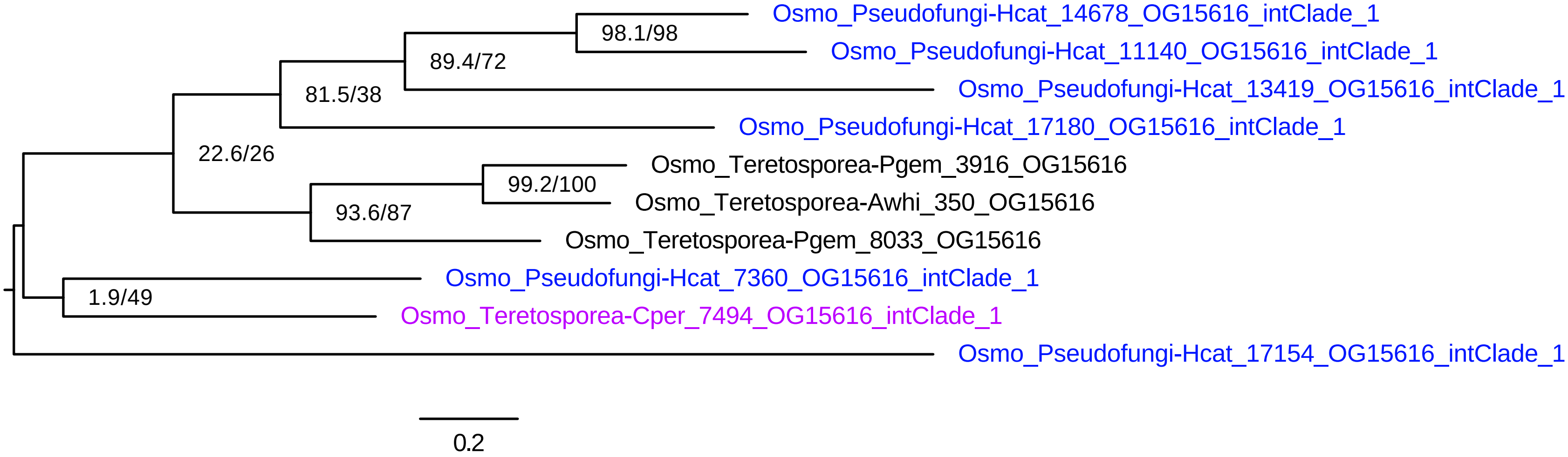
Osmo_Pseudofungi-Hcat_14678_OG15616_intClade_1
98.1/98
Osmo_Pseudofungi-Hcat_11140_OG15616_intClade_1
89.4/72
Osmo_Pseudofungi-Hcat_13419_OG15616_intClade_1
81.5/38
Osmo_Pseudofungi-Hcat_17180_OG15616_intClade_1
22.6/26
Osmo_Teretosporea-Pgem_3916_OG15616
99.2/100
Osmo_Teretosporea-Awhi_350_OG15616
93.6/87
Osmo_Teretosporea-Pgem_8033_OG15616
Osmo_Pseudofungi-Hcat_7360_OG15616_intClade_1
1.9/49
Osmo_Teretosporea-Cper_7494_OG15616_intClade_1
Osmo_Pseudofungi-Hcat_17154_OG15616_intClade_1
0.2

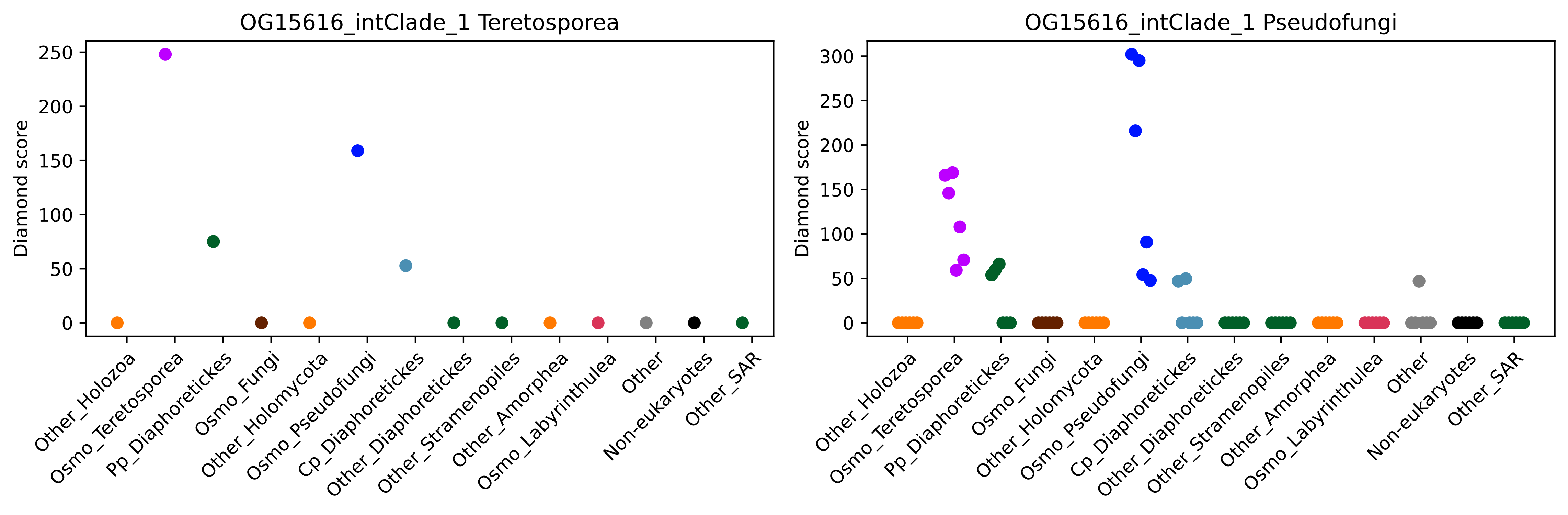

OG15616_intClade_1 Teretosporea
OG15616_intClade_1 Pseudofungi
Diamond score
0
50
100
150
200
250
300
Other_Holozoa
Osmo_Teretosporea
Pp_Diaphoretickes
Osmo_Fungi
Other_Holomycota
Osmo_Pseudofungi
Cp_Diaphoretickes
Other_Diaphoretickes
Other_Stramenopiles
Other_Amorphea
Osmo_Labyrinthulea
Other
Non-eukaryotes
Other_SAR

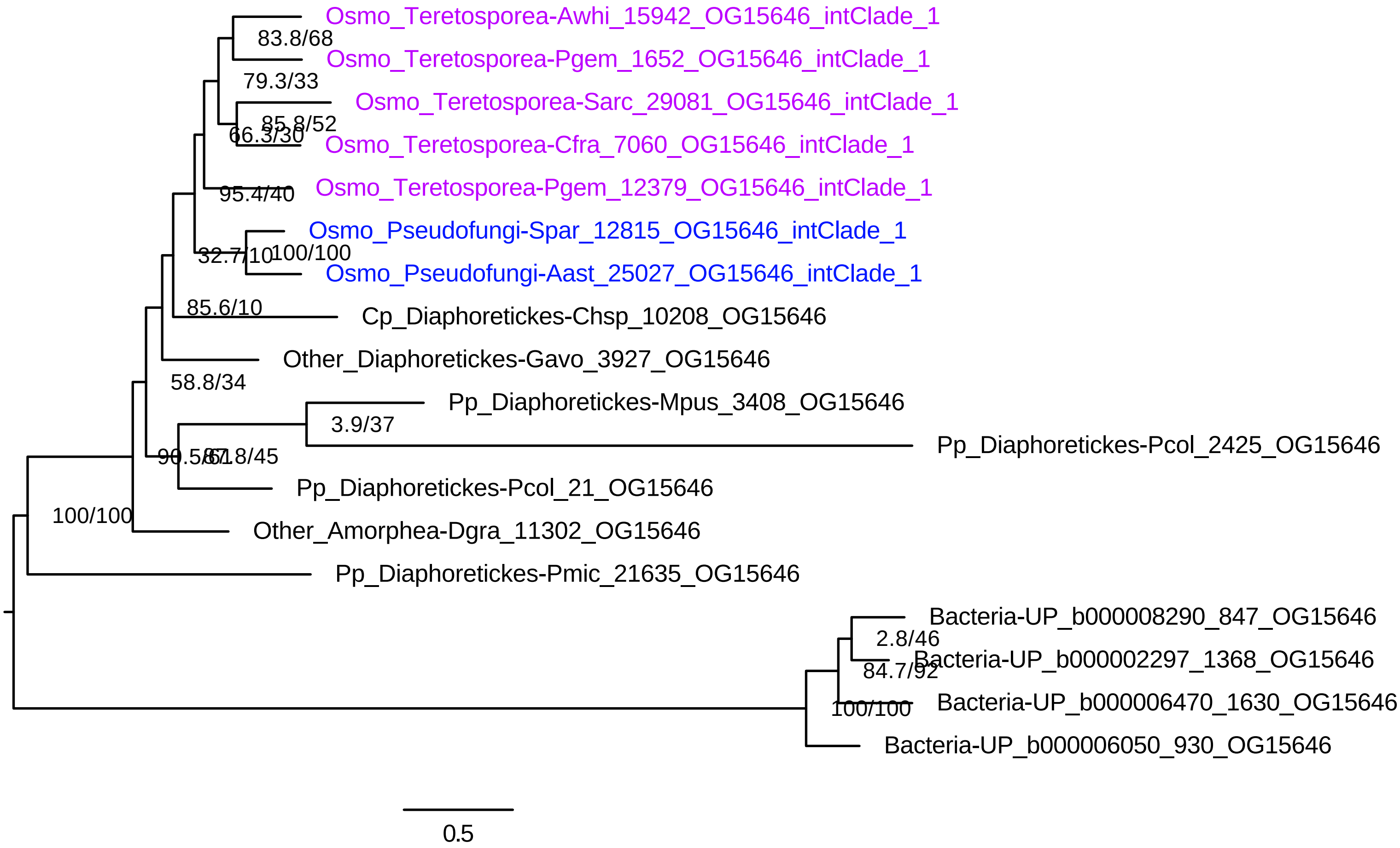

Osmo_Teretosporea-Awhi_15942_OG15646_intClade_1
83.8/68
Osmo_Teretosporea-Pgem_1652_OG15646_intClade_1
79.3/33
Osmo_Teretosporea-Sarc_29081_OG15646_intClade_1
85.8/52
66.3/30
Osmo_Teretosporea-Cfra_7060_OG15646_intClade_1
Osmo_Teretosporea-Pgem_12379_OG15646_intClade_1
95.4/40
Osmo_Pseudofungi-Spar_12815_OG15646_intClade_1
32.7/10
100/100
Osmo_Pseudofungi-Aast_25027_OG15646_intClade_1
85.6/10
Cp_Diaphoretickes-Chsp_10208_OG15646
Other_Diaphoretickes-Gavo_3927_OG15646
58.8/34
Pp_Diaphoretickes-Mpus_3408_OG15646
3.9/37
Pp_Diaphoretickes-Pcol_2425_OG15646
90.5/87
61.8/45
Pp_Diaphoretickes-Pcol_21_OG15646
100/100
Other_Amorphea-Dgra_11302_OG15646
Pp_Diaphoretickes-Pmic_21635_OG15646
Bacteria-UP_b000008290_847_OG15646
2.8/46
Bacteria-UP_b000002297_1368_OG15646
84.7/92
Bacteria-UP_b000006470_1630_OG15646
100/100
Bacteria-UP_b000006050_930_OG15646
0.5

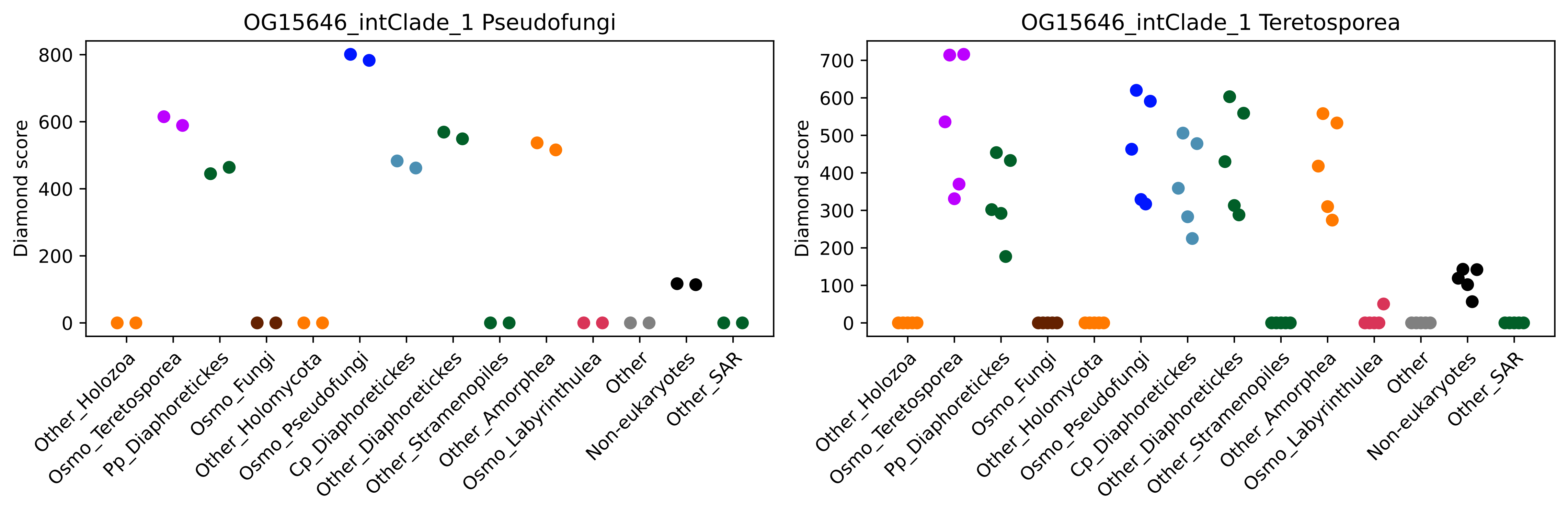
OG15646_intClade_1 Pseudofungi
OG15646_intClade_1 Teretosporea
Diamond score
Other_Holozoa
Osmo_Teretosporea
Pp_Diaphoretickes
Osmo_Fungi
Other_Holomycota
Osmo_Pseudofungi
Cp_Diaphoretickes
Other_Diaphoretickes
Other_Stramenopiles
Other_Amorphea
Osmo_Labyrinthulea
Other
Non-eukaryotes
Other_SAR

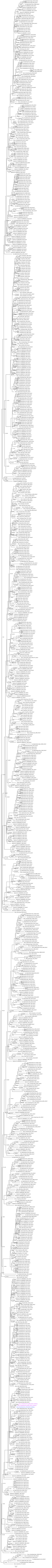

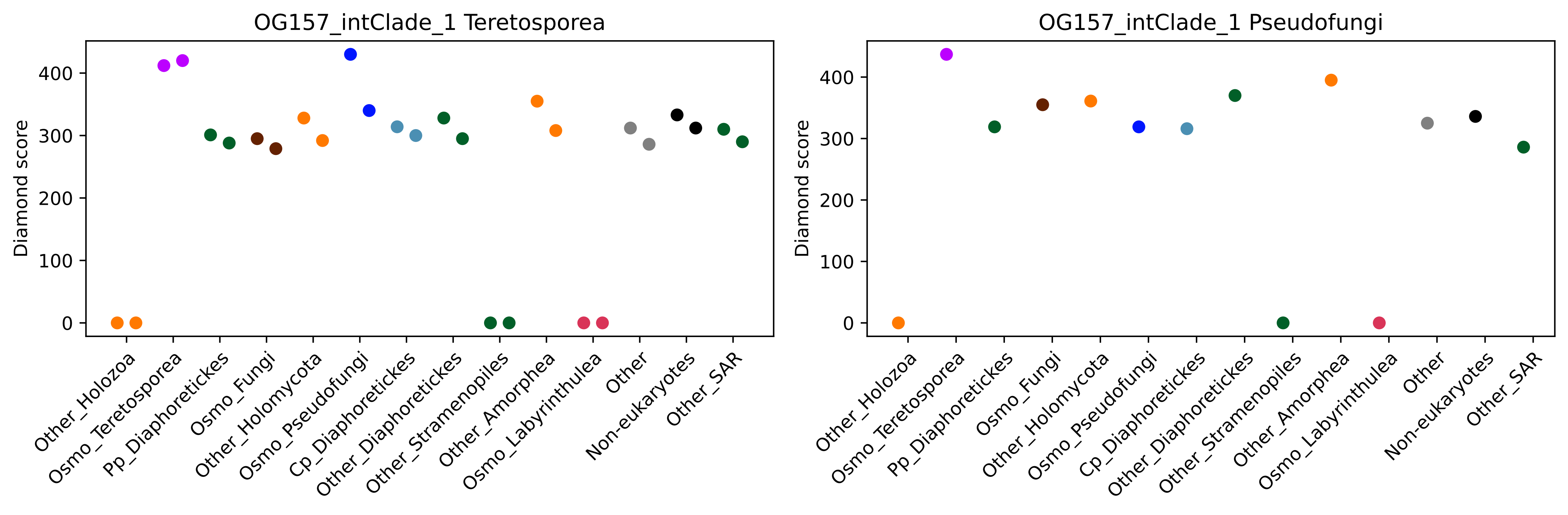

OG157_intClade_1 Teretosporea
OG157_intClade_1 Pseudofungi
Diamond score
0
100
200
300
400
Other_Holozoa
Osmo_Teretosporea
Pp_Diaphoretickes
Osmo_Fungi
Other_Holomycota
Osmo_Pseudofungi
Cp_Diaphoretickes
Other_Diaphoretickes
Other_Stramenopiles
Other_Amorphea
Osmo_Labyrinthulea
Other
Non-eukaryotes
Other_SAR

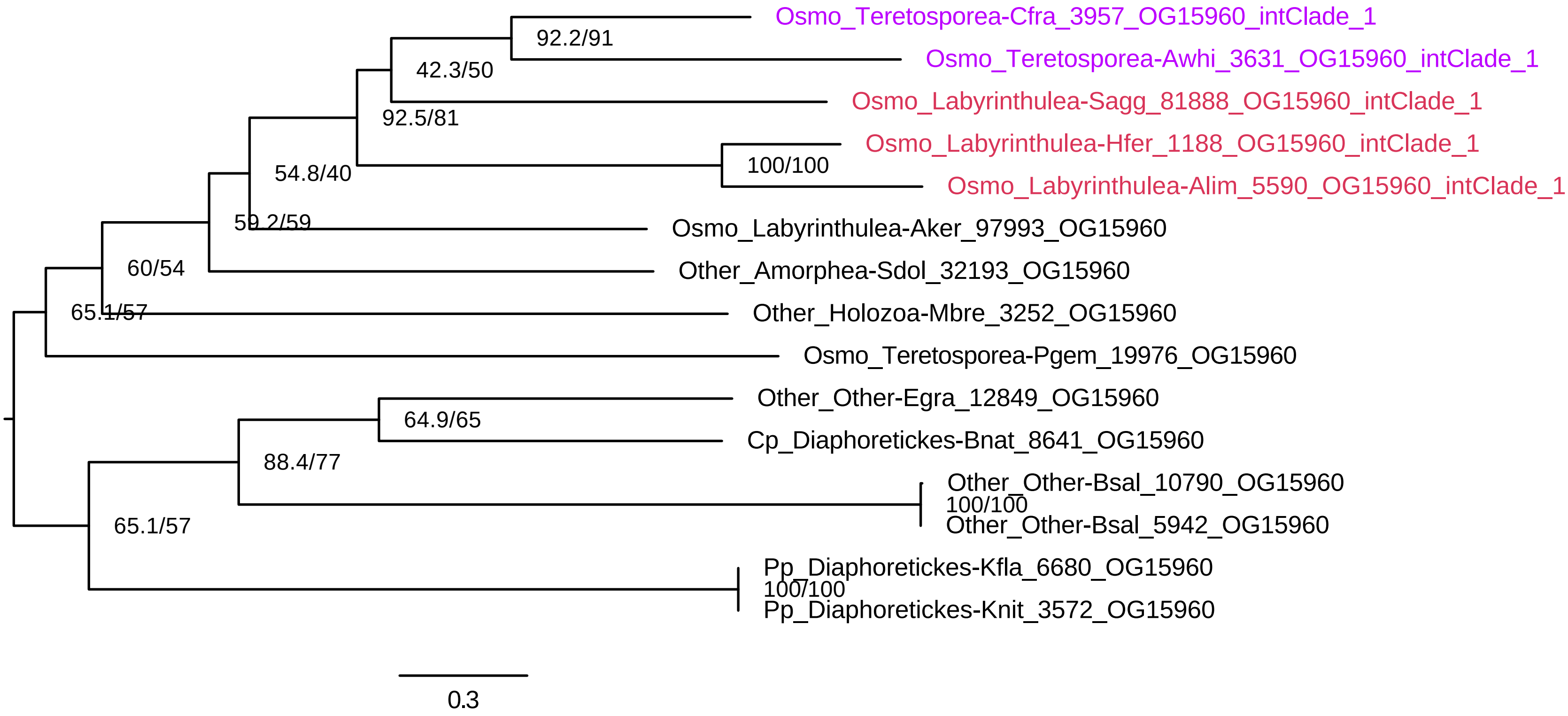

Osmo_Teretosporea-Cfra_3957_OG15960_intClade_1
92.2/91
Osmo_Teretosporea-Awhi_3631_OG15960_intClade_1
42.3/50
Osmo_Labyrinthulea-Sagg_81888_OG15960_intClade_1
92.5/81
Osmo_Labyrinthulea-Hfer_1188_OG15960_intClade_1
100/100
Osmo_Labyrinthulea-Alim_5590_OG15960_intClade_1
54.8/40
59.2/59
Osmo_Labyrinthulea-Aker_97993_OG15960
60/54
Other_Amorphea-Sdol_32193_OG15960
65.1/57
Other_Holozoa-Mbre_3252_OG15960
Osmo_Teretosporea-Pgem_19976_OG15960
Other_Other-Egra_12849_OG15960
64.9/65
Cp_Diaphoretickes-Bnat_8641_OG15960
88.4/77
Other_Other-Bsal_10790_OG15960
100/100
Other_Other-Bsal_5942_OG15960
65.1/57
Pp_Diaphoretickes-Kfla_6680_OG15960
100/100
Pp_Diaphoretickes-Knit_3572_OG15960
0.3

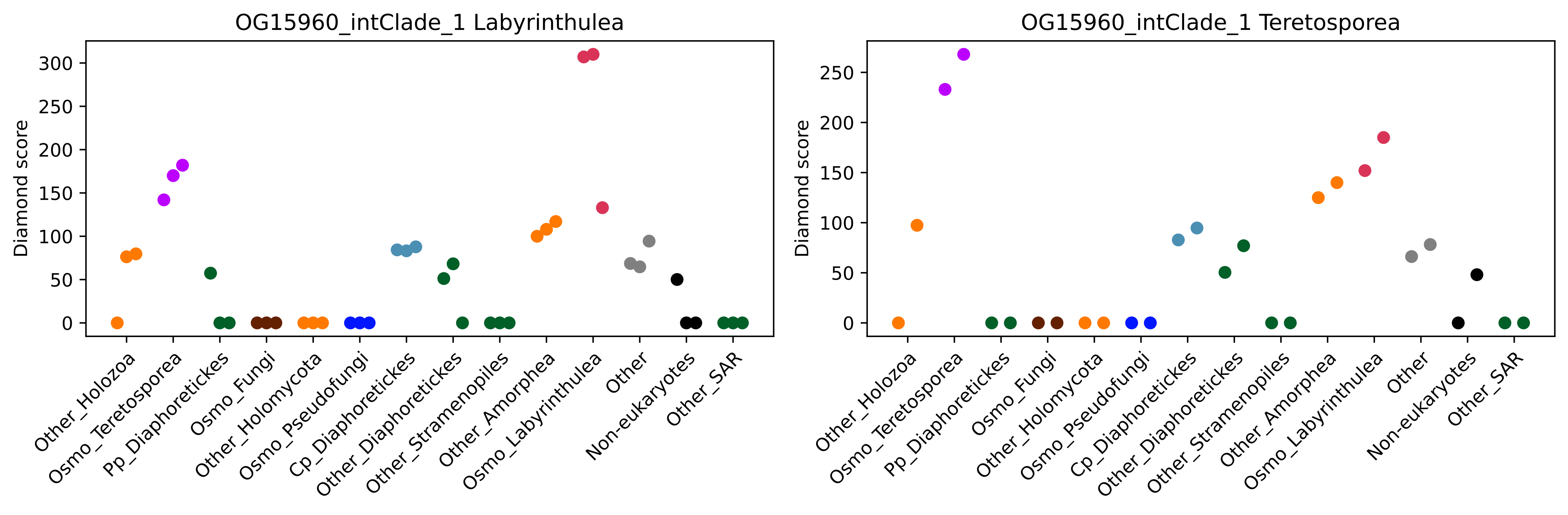
OG15960_intClade_1 Labyrinthulea
Diamond score
0
50
100
150
200
250
300
Other_Holozoa
Osmo_Teretosporea
Pp_Diaphoretickes
Osmo_Fungi
Other_Holomycota
Osmo_Pseudofungi
Cp_Diaphoretickes
Other_Diaphoretickes
Other_Stramenopiles
Other_Amorphea
Osmo_Labyrinthulea
Other
Non-eukaryotes
Other_SAR
OG15960_intClade_1 Teretosporea
Diamond score
0
50
100
150
200
250
Other_Holozoa
Osmo_Teretosporea
Pp_Diaphoretickes
Osmo_Fungi
Other_Holomycota
Osmo_Pseudofungi
Cp_Diaphoretickes
Other_Diaphoretickes
Other_Stramenopiles
Other_Amorphea
Osmo_Labyrinthulea
Other
Non-eukaryotes
Other_SAR

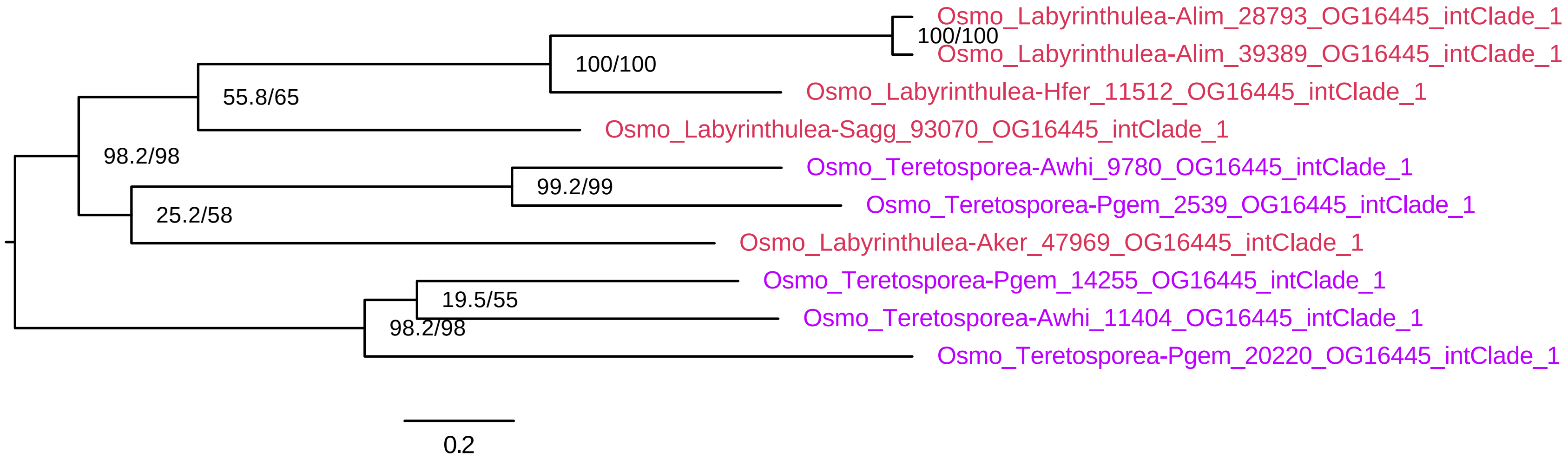
Osmo_Labyrinthulea-Alim_28793_OG16445_intClade_1
100/100
Osmo_Labyrinthulea-Alim_39389_OG16445_intClade_1
100/100
Osmo_Labyrinthulea-Hfer_11512_OG16445_intClade_1
55.8/65
Osmo_Labyrinthulea-Sagg_93070_OG16445_intClade_1
98.2/98
Osmo_Teretosporea-Awhi_9780_OG16445_intClade_1
99.2/99
Osmo_Teretosporea-Pgem_2539_OG16445_intClade_1
25.2/58
Osmo_Labyrinthulea-Aker_47969_OG16445_intClade_1
Osmo_Teretosporea-Pgem_14255_OG16445_intClade_1
19.5/55
Osmo_Teretosporea-Awhi_11404_OG16445_intClade_1
98.2/98
Osmo_Teretosporea-Pgem_20220_OG16445_intClade_1
0.2

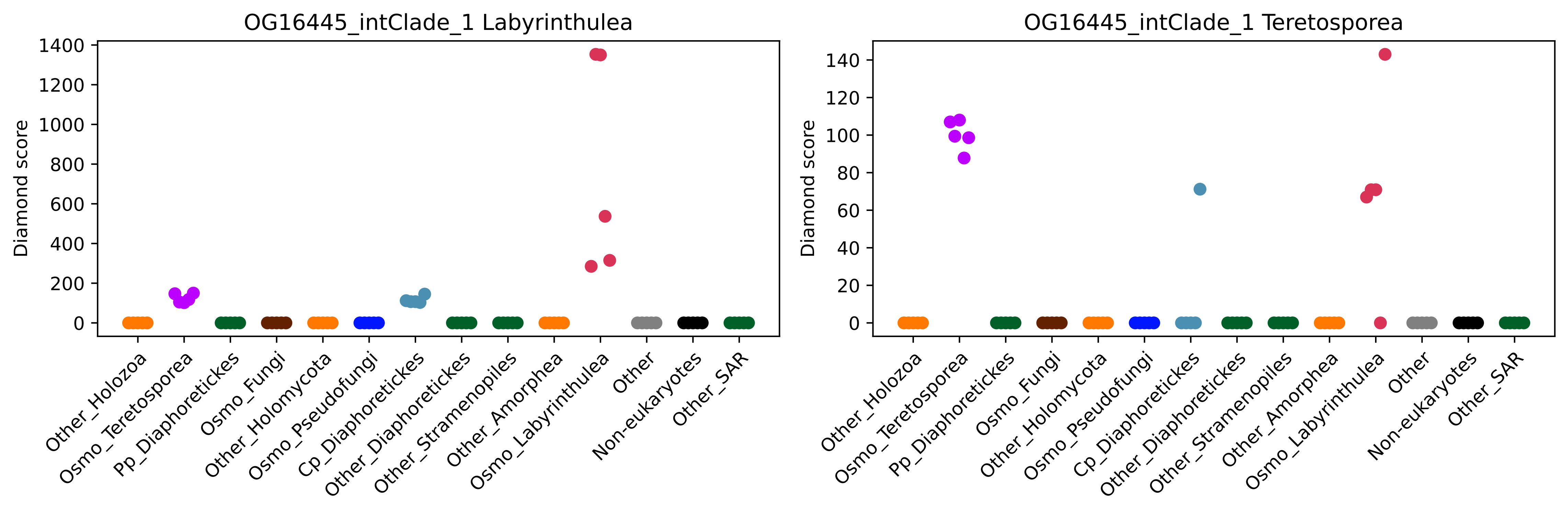
OG16445_intClade_1 Labyrinthulea
OG16445_intClade_1 Teretosporea
Diamond score
0
200
400
600
800
1000
1200
1400
20
40
60
80
100
120
140
Other_Holozoa
Osmo_Teretosporea
Pp_Diaphoretickes
Osmo_Fungi
Other_Holomycota
Osmo_Pseudofungi
Cp_Diaphoretickes
Other_Diaphoretickes
Other_Stramenopiles
Other_Amorphea
Osmo_Labyrinthulea
Other
Non-eukaryotes
Other_SAR

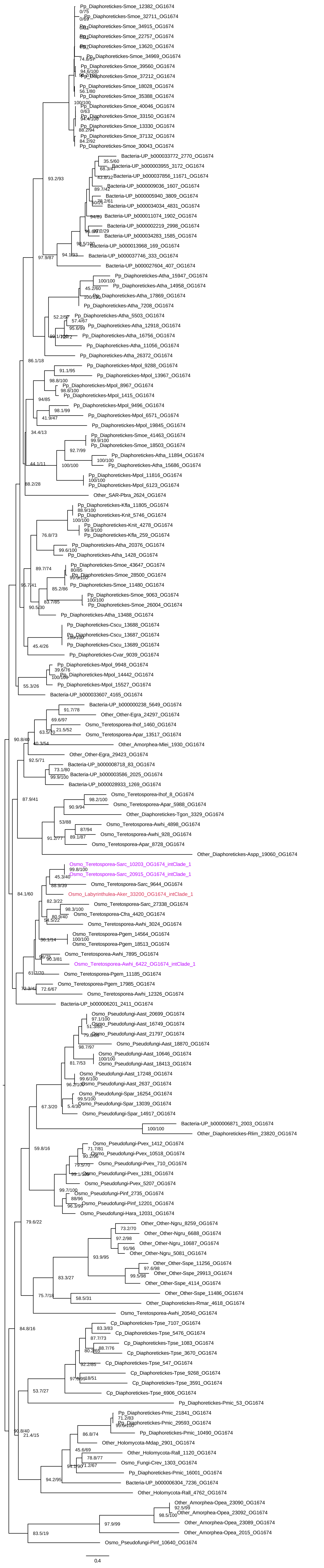
Pp_Diaphoretickes-Smoe_12382_OG1674
Pp_Diaphoretickes-Smoe_32711_OG1674
Pp_Diaphoretickes-Smoe_34915_OG1674
Pp_Diaphoretickes-Smoe_22757_OG1674
Pp_Diaphoretickes-Smoe_13620_OG1674
Pp_Diaphoretickes-Smoe_34969_OG1674
Pp_Diaphoretickes-Smoe_39560_OG1674
Pp_Diaphoretickes-Smoe_37212_OG1674
Pp_Diaphoretickes-Smoe_18028_OG1674
Pp_Diaphoretickes-Smoe_35388_OG1674
Pp_Diaphoretickes-Smoe_40046_OG1674
Pp_Diaphoretickes-Smoe_33150_OG1674
Pp_Diaphoretickes-Smoe_13330_OG1674
Pp_Diaphoretickes-Smoe_37132_OG1674
Pp_Diaphoretickes-Smoe_30043_OG1674
Bacteria-UP_b000033772_2770_OG1674
Bacteria-UP_b000003955_3172_OG1674
Bacteria-UP_b000037856_11671_OG1674
Bacteria-UP_b000009036_1607_OG1674
Bacteria-UP_b000005940_3809_OG1674
Bacteria-UP_b000034034_4831_OG1674
Bacteria-UP_b000011074_1902_OG1674
Bacteria-UP_b000002219_2998_OG1674
Bacteria-UP_b000034283_1585_OG1674
Bacteria-UP_b000013968_169_OG1674
Bacteria-UP_b000037746_333_OG1674
Bacteria-UP_b000027604_407_OG1674
Pp_Diaphoretickes-Atha_15947_OG1674
Pp_Diaphoretickes-Atha_14958_OG1674
Pp_Diaphoretickes-Atha_17869_OG1674
Pp_Diaphoretickes-Atha_7208_OG1674
Pp_Diaphoretickes-Atha_5503_OG1674
Pp_Diaphoretickes-Atha_12918_OG1674
Pp_Diaphoretickes-Atha_16756_OG1674
Pp_Diaphoretickes-Atha_11056_OG1674
Pp_Diaphoretickes-Atha_26372_OG1674
Pp_Diaphoretickes-Mpol_9288_OG1674
Pp_Diaphoretickes-Mpol_13967_OG1674
Pp_Diaphoretickes-Mpol_8967_OG1674
Pp_Diaphoretickes-Mpol_1415_OG1674
Pp_Diaphoretickes-Mpol_9496_OG1674
Pp_Diaphoretickes-Mpol_6571_OG1674
Pp_Diaphoretickes-Mpol_19845_OG1674
Pp_Diaphoretickes-Smoe_41463_OG1674
Pp_Diaphoretickes-Smoe_18503_OG1674
Pp_Diaphoretickes-Atha_11894_OG1674
Pp_Diaphoretickes-Atha_15686_OG1674
Pp_Diaphoretickes-Mpol_11816_OG1674
Pp_Diaphoretickes-Mpol_6123_OG1674
Other_SAR-Pbra_2624_OG1674
Pp_Diaphoretickes-Kfla_11805_OG1674
Pp_Diaphoretickes-Knit_5746_OG1674
Pp_Diaphoretickes-Knit_4278_OG1674
Pp_Diaphoretickes-Kfla_259_OG1674
Pp_Diaphoretickes-Atha_20376_OG1674
Pp_Diaphoretickes-Atha_1428_OG1674
Pp_Diaphoretickes-Smoe_43647_OG1674
Pp_Diaphoretickes-Smoe_28500_OG1674
Pp_Diaphoretickes-Smoe_11480_OG1674
Pp_Diaphoretickes-Smoe_9063_OG1674
Pp_Diaphoretickes-Smoe_26004_OG1674
Pp_Diaphoretickes-Atha_13488_OG1674
Pp_Diaphoretickes-Cscu_13688_OG1674
Pp_Diaphoretickes-Cscu_13687_OG1674
Pp_Diaphoretickes-Cscu_13689_OG1674
Pp_Diaphoretickes-Cvar_9039_OG1674
Pp_Diaphoretickes-Mpol_9948_OG1674
Pp_Diaphoretickes-Mpol_14442_OG1674
Pp_Diaphoretickes-Mpol_15527_OG1674
Bacteria-UP_b000033607_4165_OG1674
Bacteria-UP_b000000238_5649_OG1674
Other_Other-Egra_24297_OG1674
Osmo_Teretosporea-Ihof_1460_OG1674
Osmo_Teretosporea-Apar_13517_OG1674
Other_Amorphea-Mlei_1930_OG1674
Other_Other-Egra_29423_OG1674
Bacteria-UP_b000008718_83_OG1674
Bacteria-UP_b000003586_2025_OG1674
Bacteria-UP_b000028933_1269_OG1674
Osmo_Teretosporea-Ihof_8_OG1674
Osmo_Teretosporea-Apar_5988_OG1674
Other_Diaphoretickes-Tgon_3329_OG1674
Osmo_Teretosporea-Awhi_4898_OG1674
Osmo_Teretosporea-Awhi_928_OG1674
Osmo_Teretosporea-Apar_8728_OG1674
Other_Diaphoretickes-Aspp_19060_OG1674
Osmo_Teretosporea-Sarc_10203_OG1674_intClade_1
Osmo_Teretosporea-Sarc_20915_OG1674_intClade_1
Osmo_Teretosporea-Sarc_9644_OG1674
Osmo_Labyrinthulea-Aker_33200_OG1674_intClade_1
Osmo_Teretosporea-Sarc_27338_OG1674
Osmo_Teretosporea-Cfra_4420_OG1674
Osmo_Teretosporea-Awhi_3024_OG1674
Osmo_Teretosporea-Pgem_14564_OG1674
Osmo_Teretosporea-Pgem_18513_OG1674
Osmo_Teretosporea-Awhi_7895_OG1674
Osmo_Teretosporea-Awhi_6422_OG1674_intClade_1
Osmo_Teretosporea-Pgem_11185_OG1674
Osmo_Teretosporea-Pgem_17985_OG1674
Osmo_Teretosporea-Awhi_12326_OG1674
Bacteria-UP_b000006201_2411_OG1674
Osmo_Pseudofungi-Aast_20699_OG1674
Osmo_Pseudofungi-Aast_16749_OG1674
Osmo_Pseudofungi-Aast_21797_OG1674
Osmo_Pseudofungi-Aast_18870_OG1674
Osmo_Pseudofungi-Aast_10646_OG1674
Osmo_Pseudofungi-Aast_18413_OG1674
Osmo_Pseudofungi-Aast_17248_OG1674
Osmo_Pseudofungi-Aast_2637_OG1674
Osmo_Pseudofungi-Spar_16254_OG1674
Osmo_Pseudofungi-Spar_13039_OG1674
Osmo_Pseudofungi-Spar_14917_OG1674
Bacteria-UP_b000006871_2003_OG1674
Other_Diaphoretickes-Rlim_23820_OG1674
Osmo_Pseudofungi-Pvex_1412_OG1674
Osmo_Pseudofungi-Pvex_10518_OG1674
Osmo_Pseudofungi-Pvex_710_OG1674
Osmo_Pseudofungi-Pvex_1281_OG1674
Osmo_Pseudofungi-Pvex_5207_OG1674
Osmo_Pseudofungi-Pinf_2735_OG1674
Osmo_Pseudofungi-Pinf_12201_OG1674
Osmo_Pseudofungi-Hara_12031_OG1674
Other_Other-Ngru_8259_OG1674
Other_Other-Ngru_6688_OG1674
Other_Other-Ngru_10687_OG1674
Other_Other-Ngru_5081_OG1674
Other_Other-Sspe_11256_OG1674
Other_Other-Sspe_29913_OG1674
Other_Other-Sspe_4114_OG1674
Other_Other-Sspe_11486_OG1674
Other_Diaphoretickes-Rmar_4618_OG1674
Osmo_Teretosporea-Awhi_20540_OG1674
Cp_Diaphoretickes-Tpse_7107_OG1674
Cp_Diaphoretickes-Tpse_5476_OG1674
Cp_Diaphoretickes-Tpse_1083_OG1674
Cp_Diaphoretickes-Tpse_3670_OG1674
Cp_Diaphoretickes-Tpse_547_OG1674
Cp_Diaphoretickes-Tpse_9268_OG1674
Cp_Diaphoretickes-Tpse_3591_OG1674
Cp_Diaphoretickes-Tpse_6906_OG1674
Pp_Diaphoretickes-Pmic_53_OG1674
Pp_Diaphoretickes-Pmic_21841_OG1674
Pp_Diaphoretickes-Pmic_29593_OG1674
Pp_Diaphoretickes-Pmic_10490_OG1674
Other_Holomycota-Mdap_2901_OG1674
Other_Holomycota-Rall_1120_OG1674
Osmo_Fungi-Crev_1303_OG1674
Pp_Diaphoretickes-Pmic_16001_OG1674
Bacteria-UP_b000006304_7236_OG1674
Other_Holomycota-Rall_4762_OG1674
Other_Amorphea-Opea_23090_OG1674
Other_Amorphea-Opea_23092_OG1674
Other_Amorphea-Opea_23089_OG1674
Other_Amorphea-Opea_2015_OG1674
Osmo_Pseudofungi-Pinf_10640_OG1674
0.4

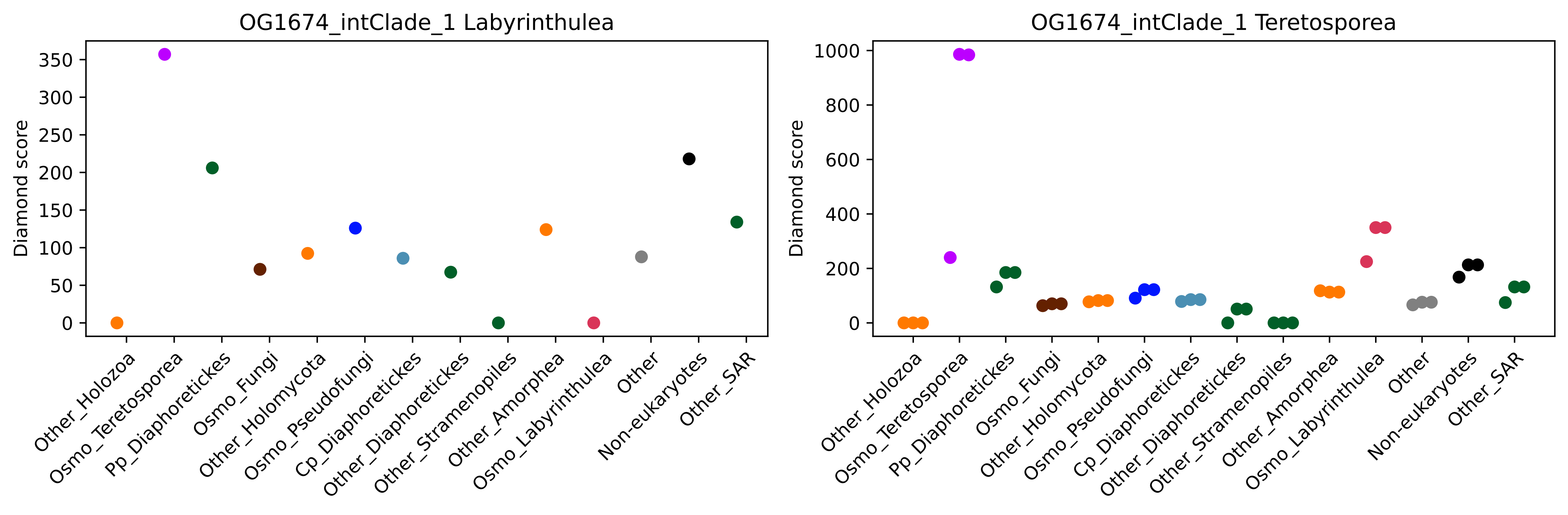

OG1674_intClade_1 Labyrinthulea
Diamond score
0
50
100
150
200
250
300
350
Other_Holozoa
Osmo_Teretosporea
Pp_Diaphoretickes
Osmo_Fungi
Other_Holomycota
Osmo_Pseudofungi
Cp_Diaphoretickes
Other_Diaphoretickes
Other_Stramenopiles
Other_Amorphea
Osmo_Labyrinthulea
Other
Non-eukaryotes
Other_SAR
OG1674_intClade_1 Teretosporea
Diamond score
0
200
400
600
800
1000
Other_Holozoa
Osmo_Teretosporea
Pp_Diaphoretickes
Osmo_Fungi
Other_Holomycota
Osmo_Pseudofungi
Cp_Diaphoretickes
Other_Diaphoretickes
Other_Stramenopiles
Other_Amorphea
Osmo_Labyrinthulea
Other
Non-eukaryotes
Other_SAR

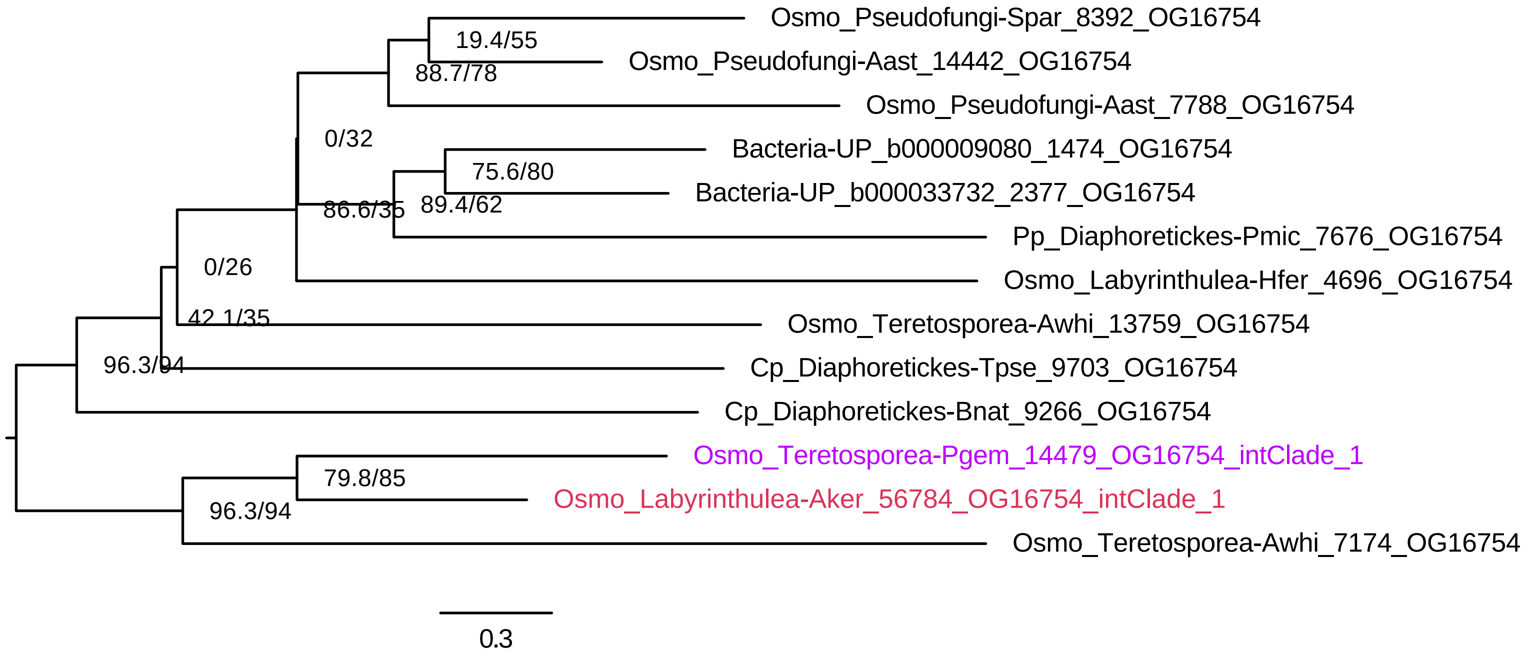
Osmo_Pseudofungi-Spar_8392_OG16754
19.4/55
Osmo_Pseudofungi-Aast_14442_OG16754
88.7/78
Osmo_Pseudofungi-Aast_7788_OG16754
0/32
Bacteria-UP_b000009080_1474_OG16754
75.6/80
Bacteria-UP_b000033732_2377_OG16754
86.6/35
89.4/62
Pp_Diaphoretickes-Pmic_7676_OG16754
0/26
Osmo_Labyrinthulea-Hfer_4696_OG16754
42.1/35
Osmo_Teretosporea-Awhi_13759_OG16754
96.3/94
Cp_Diaphoretickes-Tpse_9703_OG16754
Cp_Diaphoretickes-Bnat_9266_OG16754
Osmo_Teretosporea-Pgem_14479_OG16754_intClade_1
79.8/85
Osmo_Labyrinthulea-Aker_56784_OG16754_intClade_1
96.3/94
Osmo_Teretosporea-Awhi_7174_OG16754
0.3

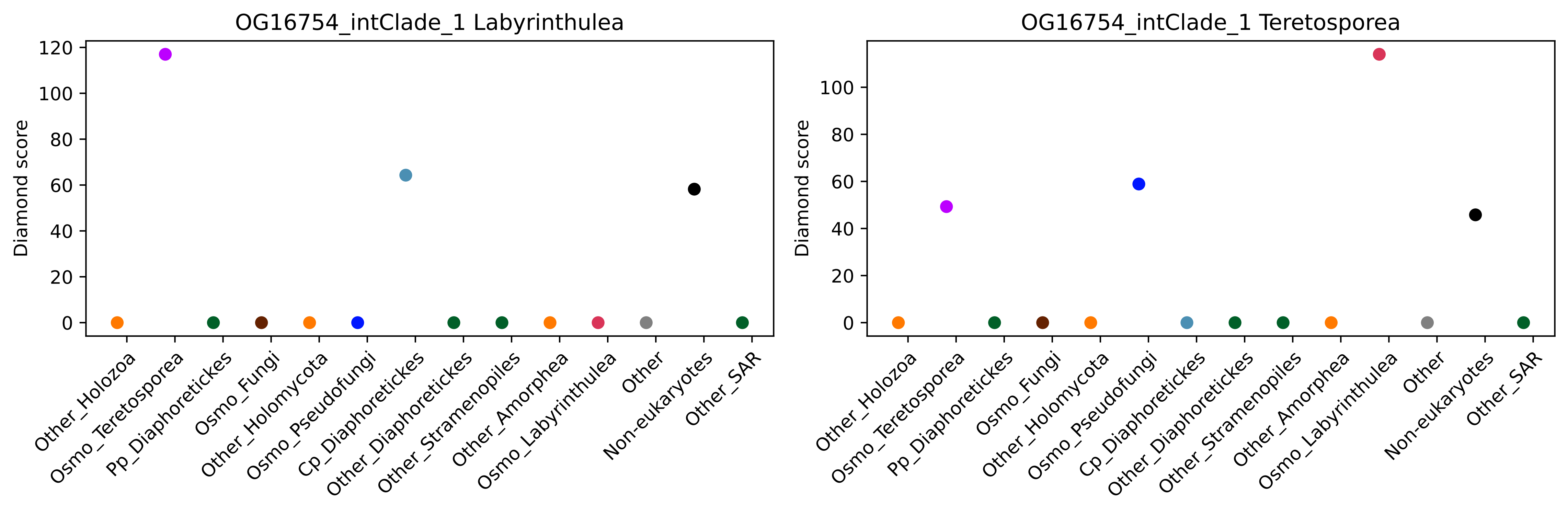

OG16754_intClade_1 Labyrinthulea
OG16754_intClade_1 Teretosporea
Diamond score
0
20
40
60
80
100
120
Other_Holozoa
Osmo_Teretosporea
Pp_Diaphoretickes
Osmo_Fungi
Other_Holomycota
Osmo_Pseudofungi
Cp_Diaphoretickes
Other_Diaphoretickes
Other_Stramenopiles
Other_Amorphea
Osmo_Labyrinthulea
Other
Non-eukaryotes
Other_SAR

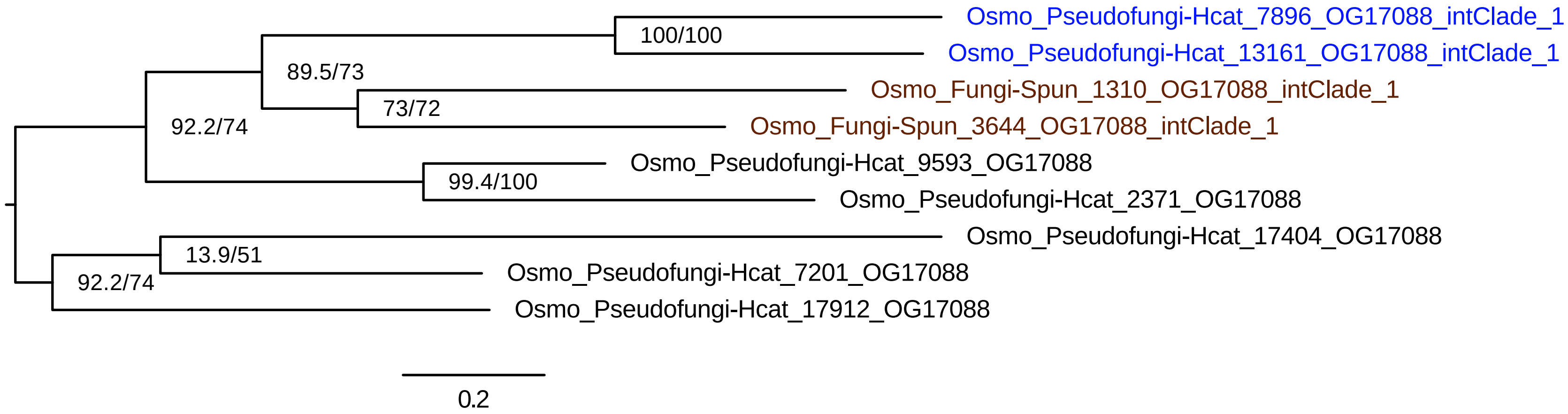

Osmo_Pseudofungi-Hcat_7896_OG17088_intClade_1
100/100
Osmo_Pseudofungi-Hcat_13161_OG17088_intClade_1
89.5/73
Osmo_Fungi-Spun_1310_OG17088_intClade_1
73/72
Osmo_Fungi-Spun_3644_OG17088_intClade_1
92.2/74
Osmo_Pseudofungi-Hcat_9593_OG17088
99.4/100
Osmo_Pseudofungi-Hcat_2371_OG17088
Osmo_Pseudofungi-Hcat_17404_OG17088
13.9/51
Osmo_Pseudofungi-Hcat_7201_OG17088
92.2/74
Osmo_Pseudofungi-Hcat_17912_OG17088
0.2

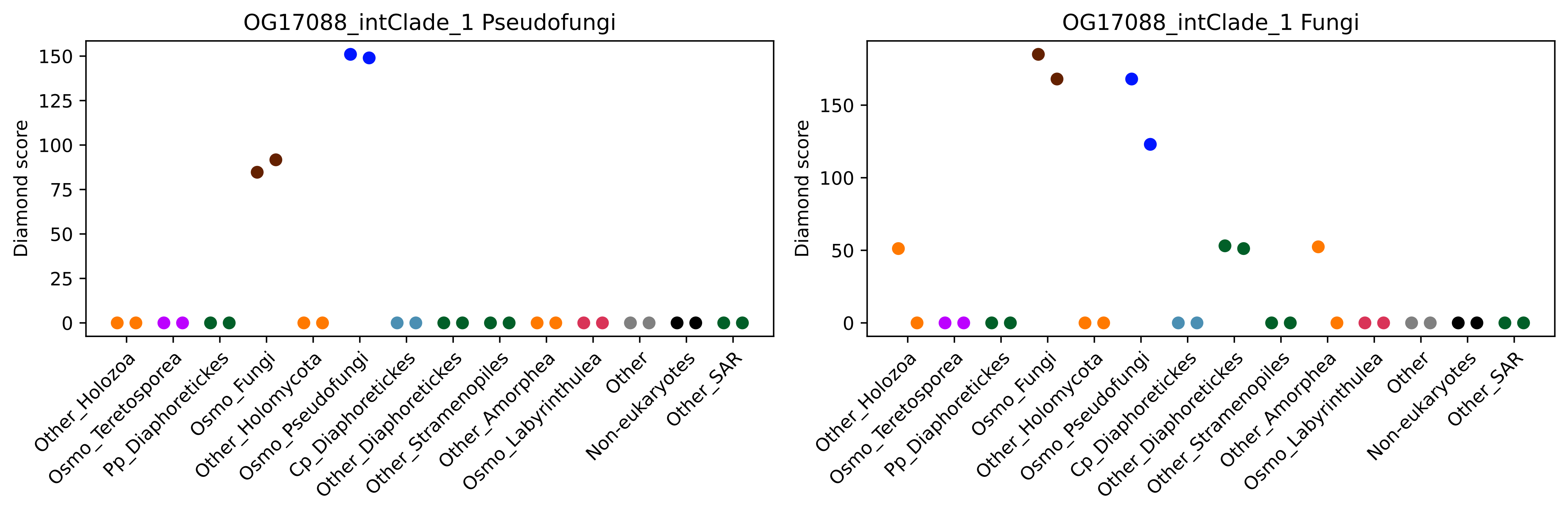

OG17088_intClade_1 Pseudofungi
OG17088_intClade_1 Fungi
Diamond score
0
25
50
75
100
125
150
Other_Holozoa
Osmo_Teretosporea
Pp_Diaphoretickes
Osmo_Fungi
Other_Holomycota
Osmo_Pseudofungi
Cp_Diaphoretickes
Other_Diaphoretickes
Other_Stramenopiles
Other_Amorphea
Osmo_Labyrinthulea
Other
Non-eukaryotes
Other_SAR

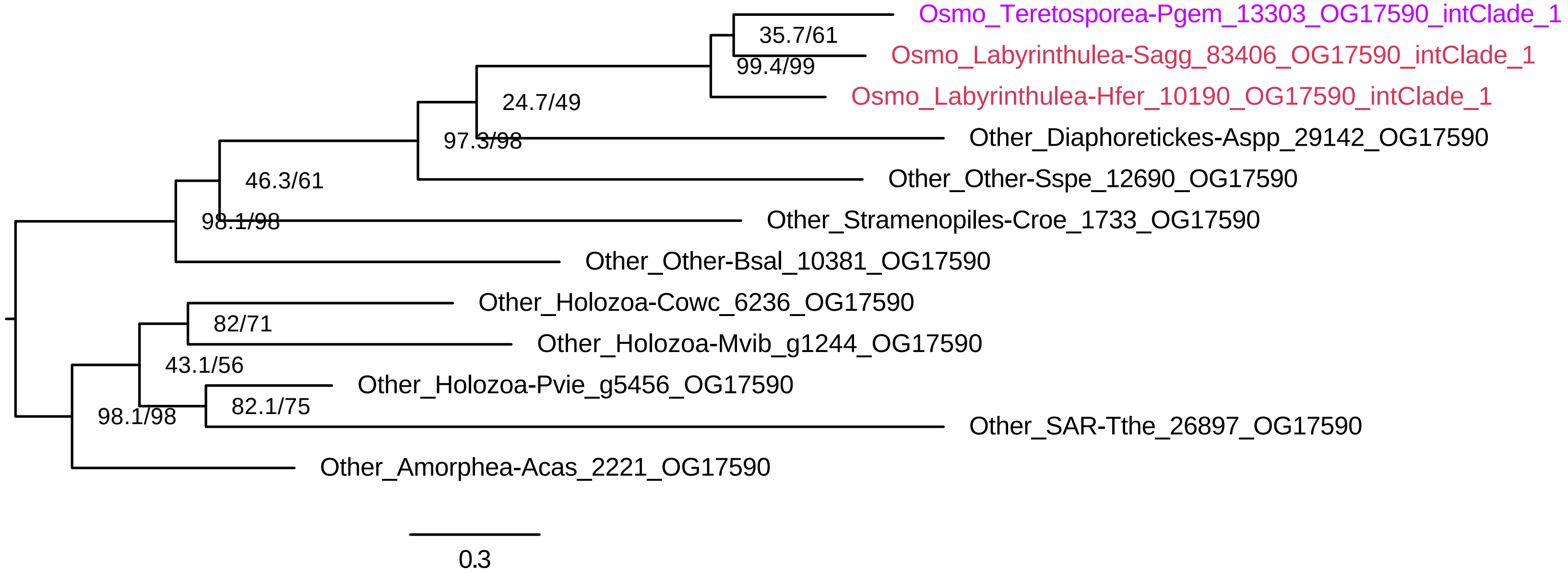
Osmo_Teretosporea-Pgem_13303_OG17590_intClade_1
35.7/61
Osmo_Labyrinthulea-Sagg_83406_OG17590_intClade_1
99.4/99
Osmo_Labyrinthulea-Hfer_10190_OG17590_intClade_1
24.7/49
Other_Diaphoretickes-Aspp_29142_OG17590
97.3/98
Other_Other-Sspe_12690_OG17590
46.3/61
Other_Stramenopiles-Croe_1733_OG17590
98.1/98
Other_Other-Bsal_10381_OG17590
Other_Holozoa-Cowc_6236_OG17590
82/71
Other_Holozoa-Mvib_g1244_OG17590
43.1/56
Other_Holozoa-Pvie_g5456_OG17590
82.1/75
98.1/98
Other_SAR-Tthe_26897_OG17590
Other_Amorphea-Acas_2221_OG17590
0.3

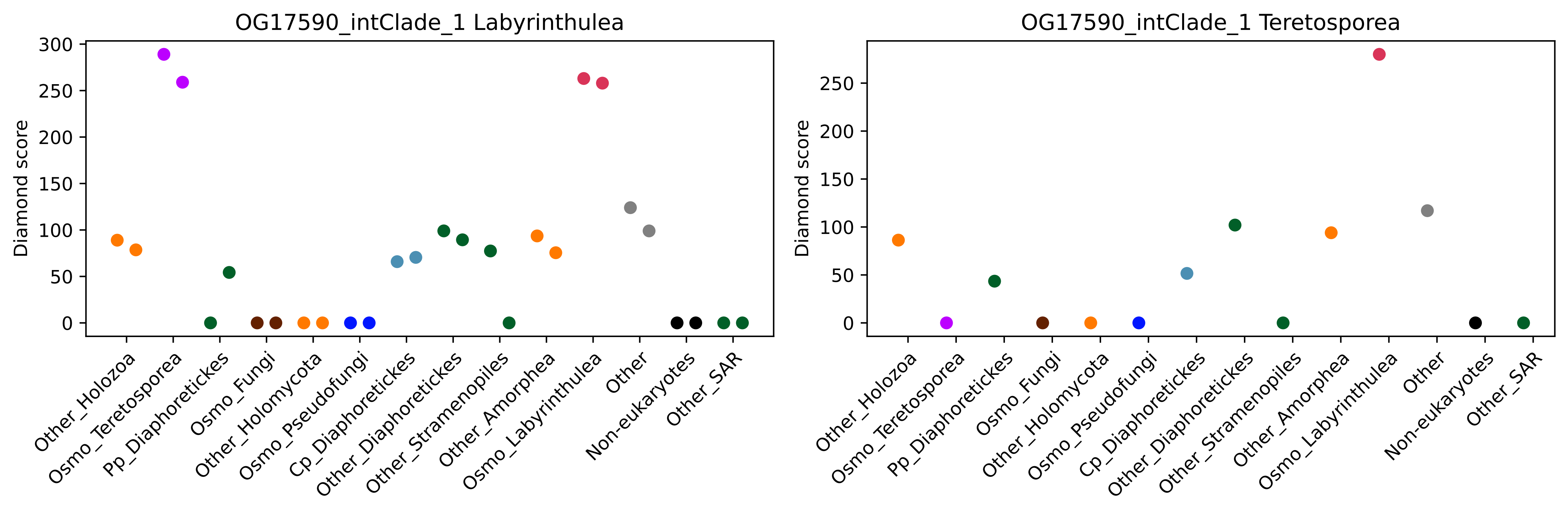

OG17590_intClade_1 Labyrinthulea
OG17590_intClade_1 Teretosporea
Diamond score
0
50
100
150
200
250
300
Other_Holozoa
Osmo_Teretosporea
Pp_Diaphoretickes
Osmo_Fungi
Other_Holomycota
Osmo_Pseudofungi
Cp_Diaphoretickes
Other_Diaphoretickes
Other_Stramenopiles
Other_Amorphea
Osmo_Labyrinthulea
Other
Non-eukaryotes
Other_SAR

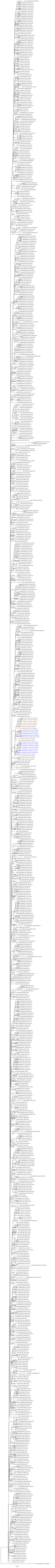

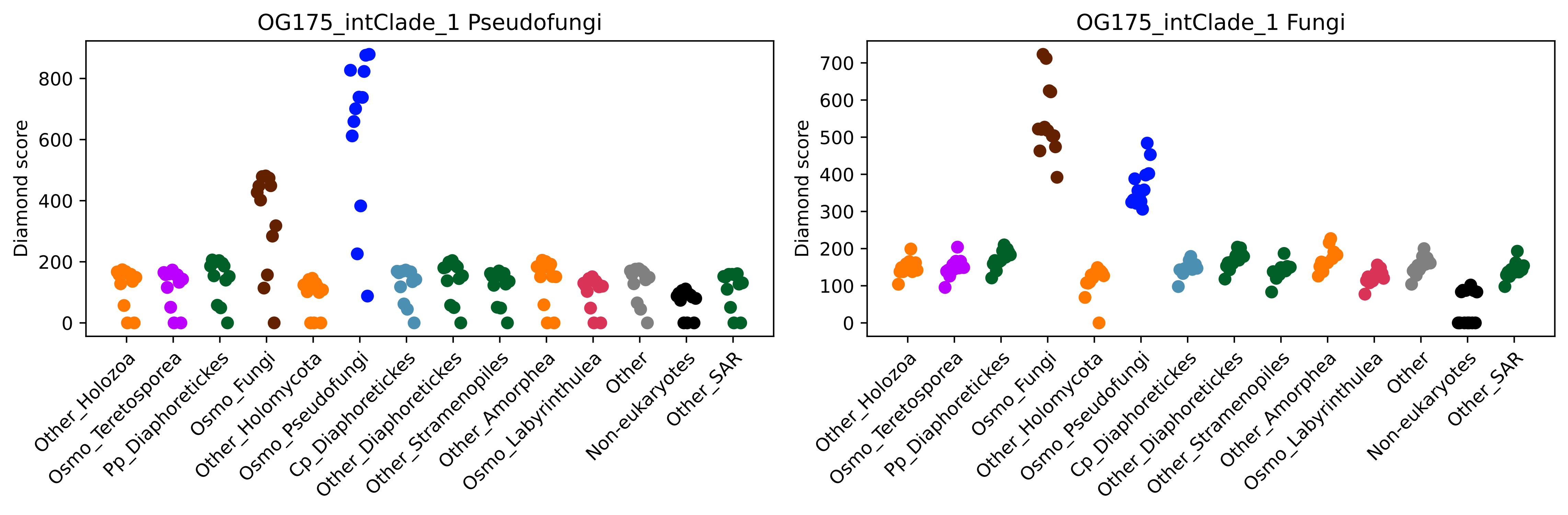

OG175_intClade_1 Pseudofungi
OG175_intClade_1 Fungi
Diamond score
Other_Holozoa
Osmo_Teretosporea
Pp_Diaphoretickes
Osmo_Fungi
Other_Holomycota
Osmo_Pseudofungi
Cp_Diaphoretickes
Other_Diaphoretickes
Other_Stramenopiles
Other_Amorphea
Osmo_Labyrinthulea
Other
Non-eukaryotes
Other_SAR

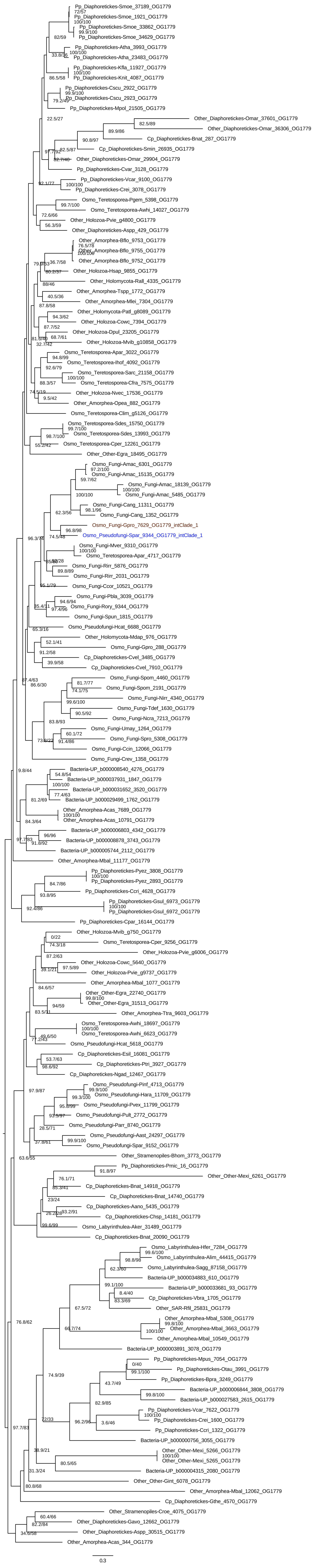

Pp_Diaphoretickes-Smoe_37189_OG1779
Pp_Diaphoretickes-Smoe_1921_OG1779
Pp_Diaphoretickes-Smoe_33862_OG1779
Pp_Diaphoretickes-Smoe_34629_OG1779
Pp_Diaphoretickes-Atha_3993_OG1779
Pp_Diaphoretickes-Atha_23483_OG1779
Pp_Diaphoretickes-Kfla_11927_OG1779
Pp_Diaphoretickes-Knit_4087_OG1779
Pp_Diaphoretickes-Cscu_2922_OG1779
Pp_Diaphoretickes-Cscu_2923_OG1779
Pp_Diaphoretickes-Mpol_21505_OG1779
Other_Diaphoretickes-Omar_37601_OG1779
Other_Diaphoretickes-Omar_36306_OG1779
Cp_Diaphoretickes-Bnat_287_OG1779
Cp_Diaphoretickes-Smin_26935_OG1779
Other_Diaphoretickes-Omar_29904_OG1779
Pp_Diaphoretickes-Cvar_3128_OG1779
Pp_Diaphoretickes-Vcar_9100_OG1779
Pp_Diaphoretickes-Crei_3078_OG1779
Osmo_Teretosporea-Pgem_5398_OG1779
Osmo_Teretosporea-Awhi_14027_OG1779
Other_Holozoa-Pvie_g4800_OG1779
Other_Diaphoretickes-Aspp_429_OG1779
Other_Amorphea-Bflo_9753_OG1779
Other_Amorphea-Bflo_9755_OG1779
Other_Amorphea-Bflo_9752_OG1779
Other_Holozoa-Hsap_9855_OG1779
Other_Holomycota-Rall_4335_OG1779
Other_Amorphea-Tspp_1772_OG1779
Other_Amorphea-Mlei_7304_OG1779
Other_Holomycota-Patl_g8089_OG1779
Other_Holozoa-Cowc_7394_OG1779
Other_Holozoa-Dpul_23205_OG1779
Other_Holozoa-Mvib_g10858_OG1779
Osmo_Teretosporea-Apar_3022_OG1779
Osmo_Teretosporea-Ihof_4092_OG1779
Osmo_Teretosporea-Sarc_21158_OG1779
Osmo_Teretosporea-Cfra_7575_OG1779
Other_Holozoa-Nvec_17536_OG1779
Other_Amorphea-Opea_882_OG1779
Osmo_Teretosporea-Clim_g5126_OG1779
Osmo_Teretosporea-Sdes_15750_OG1779
Osmo_Teretosporea-Sdes_13993_OG1779
Osmo_Teretosporea-Cper_12261_OG1779
Other_Other-Egra_18495_OG1779
Osmo_Fungi-Amac_6301_OG1779
Osmo_Fungi-Amac_15135_OG1779
Osmo_Fungi-Amac_18139_OG1779
Osmo_Fungi-Amac_5485_OG1779
Osmo_Fungi-Cang_11311_OG1779
Osmo_Fungi-Cang_1352_OG1779
Osmo_Fungi-Gpro_7629_OG1779_intClade_1
Osmo_Pseudofungi-Spar_9344_OG1779_intClade_1
Osmo_Fungi-Mver_9310_OG1779
Osmo_Teretosporea-Apar_4717_OG1779
Osmo_Fungi-Rirr_5876_OG1779
Osmo_Fungi-Rirr_2031_OG1779
Osmo_Fungi-Ccor_10521_OG1779
Osmo_Fungi-Pbla_3039_OG1779
Osmo_Fungi-Rory_9344_OG1779
Osmo_Fungi-Spun_1815_OG1779
Osmo_Pseudofungi-Hcat_6688_OG1779
Other_Holomycota-Mdap_976_OG1779
Osmo_Fungi-Gpro_288_OG1779
Cp_Diaphoretickes-Cvel_3485_OG1779
Cp_Diaphoretickes-Cvel_7910_OG1779
Osmo_Fungi-Spom_4460_OG1779
Osmo_Fungi-Spom_2191_OG1779
Osmo_Fungi-Nirr_4340_OG1779
Osmo_Fungi-Tdef_1630_OG1779
Osmo_Fungi-Ncra_7213_OG1779
Osmo_Fungi-Umay_1264_OG1779
Osmo_Fungi-Spro_5308_OG1779
Osmo_Fungi-Ccin_12066_OG1779
Osmo_Fungi-Crev_1358_OG1779
Bacteria-UP_b000008540_4276_OG1779
Bacteria-UP_b000037931_1847_OG1779
Bacteria-UP_b000031652_3520_OG1779
Bacteria-UP_b000029499_1762_OG1779
Other_Amorphea-Acas_7689_OG1779
Other_Amorphea-Acas_10791_OG1779
Bacteria-UP_b000006803_4342_OG1779
Bacteria-UP_b000008878_3743_OG1779
Bacteria-UP_b000005744_2112_OG1779
Other_Amorphea-Mbal_11177_OG1779
Pp_Diaphoretickes-Pyez_3808_OG1779
Pp_Diaphoretickes-Pyez_2893_OG1779
Pp_Diaphoretickes-Ccri_4628_OG1779
Pp_Diaphoretickes-Gsul_6973_OG1779
Pp_Diaphoretickes-Gsul_6972_OG1779
Pp_Diaphoretickes-Cpar_16144_OG1779
Other_Holozoa-Mvib_g750_OG1779
Osmo_Teretosporea-Cper_9256_OG1779
Other_Holozoa-Pvie_g6006_OG1779
Other_Holozoa-Cowc_5640_OG1779
Other_Holozoa-Pvie_g9737_OG1779
Other_Amorphea-Mbal_1077_OG1779
Other_Other-Egra_22740_OG1779
Other_Other-Egra_31513_OG1779
Other_Amorphea-Ttra_9603_OG1779
Osmo_Teretosporea-Awhi_18697_OG1779
Osmo_Teretosporea-Awhi_6623_OG1779
Osmo_Pseudofungi-Hcat_5618_OG1779
Cp_Diaphoretickes-Esil_16081_OG1779
Cp_Diaphoretickes-Ptri_3927_OG1779
Cp_Diaphoretickes-Ngad_12467_OG1779
Osmo_Pseudofungi-Pinf_4713_OG1779
Osmo_Pseudofungi-Hara_11709_OG1779
Osmo_Pseudofungi-Pvex_11799_OG1779
Osmo_Pseudofungi-Pult_2772_OG1779
Osmo_Pseudofungi-Parr_8740_OG1779
Osmo_Pseudofungi-Aast_24297_OG1779
Osmo_Pseudofungi-Spar_9152_OG1779
Other_Stramenopiles-Bhom_3773_OG1779
Pp_Diaphoretickes-Pmic_16_OG1779
Other_Other-Mexi_6261_OG1779
Cp_Diaphoretickes-Bnat_14918_OG1779
Cp_Diaphoretickes-Bnat_14740_OG1779
Cp_Diaphoretickes-Aano_5435_OG1779
Cp_Diaphoretickes-Chsp_14181_OG1779
Osmo_Labyrinthulea-Aker_31489_OG1779
Cp_Diaphoretickes-Bnat_20090_OG1779
Osmo_Labyrinthulea-Hfer_7284_OG1779
Osmo_Labyrinthulea-Alim_44415_OG1779
Osmo_Labyrinthulea-Sagg_87158_OG1779
Bacteria-UP_b000034883_610_OG1779
Bacteria-UP_b000033681_93_OG1779
Cp_Diaphoretickes-Vbra_1705_OG1779
Other_SAR-Rfil_25831_OG1779
Other_Amorphea-Mbal_5308_OG1779
Other_Amorphea-Mbal_3663_OG1779
Other_Amorphea-Mbal_10549_OG1779
Bacteria-UP_b000003891_3078_OG1779
Pp_Diaphoretickes-Mpus_7054_OG1779
Pp_Diaphoretickes-Otau_3991_OG1779
Pp_Diaphoretickes-Bpra_3249_OG1779
Bacteria-UP_b000006844_3808_OG1779
Bacteria-UP_b000027583_2615_OG1779
Pp_Diaphoretickes-Vcar_7622_OG1779
Pp_Diaphoretickes-Crei_1600_OG1779
Pp_Diaphoretickes-Ccri_1322_OG1779
Bacteria-UP_b000000756_3055_OG1779
Other_Other-Mexi_5266_OG1779
Other_Other-Mexi_5265_OG1779
Bacteria-UP_b000004315_2080_OG1779
Other_Other-Gint_6078_OG1779
Other_Amorphea-Mbal_12062_OG1779
Cp_Diaphoretickes-Gthe_4570_OG1779
Other_Stramenopiles-Croe_4075_OG1779
Other_Diaphoretickes-Gavo_12662_OG1779
Other_Diaphoretickes-Aspp_30515_OG1779
Other_Amorphea-Acas_344_OG1779
0.3

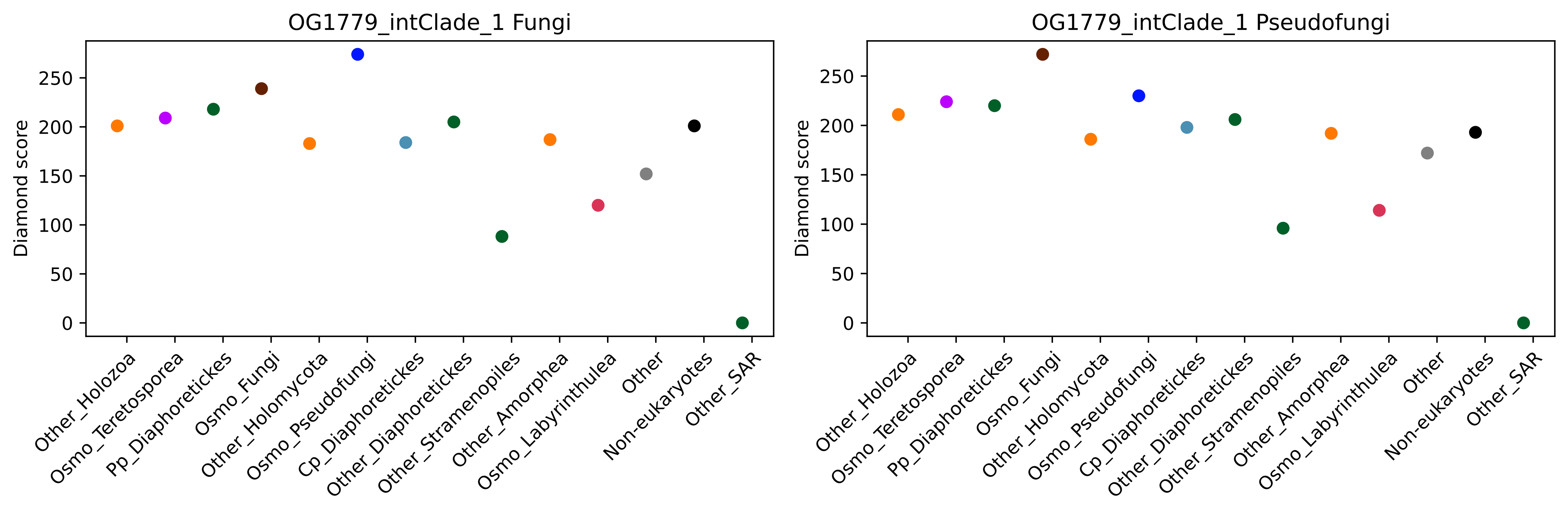

OG1779_intClade_1 Fungi
OG1779_intClade_1 Pseudofungi
Diamond score
0
50
100
150
200
250
Other_Holozoa
Osmo_Teretosporea
Pp_Diaphoretickes
Osmo_Fungi
Other_Holomycota
Osmo_Pseudofungi
Cp_Diaphoretickes
Other_Diaphoretickes
Other_Stramenopiles
Other_Amorphea
Osmo_Labyrinthulea
Other
Non-eukaryotes
Other_SAR

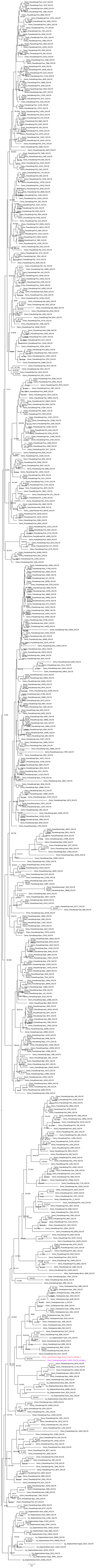

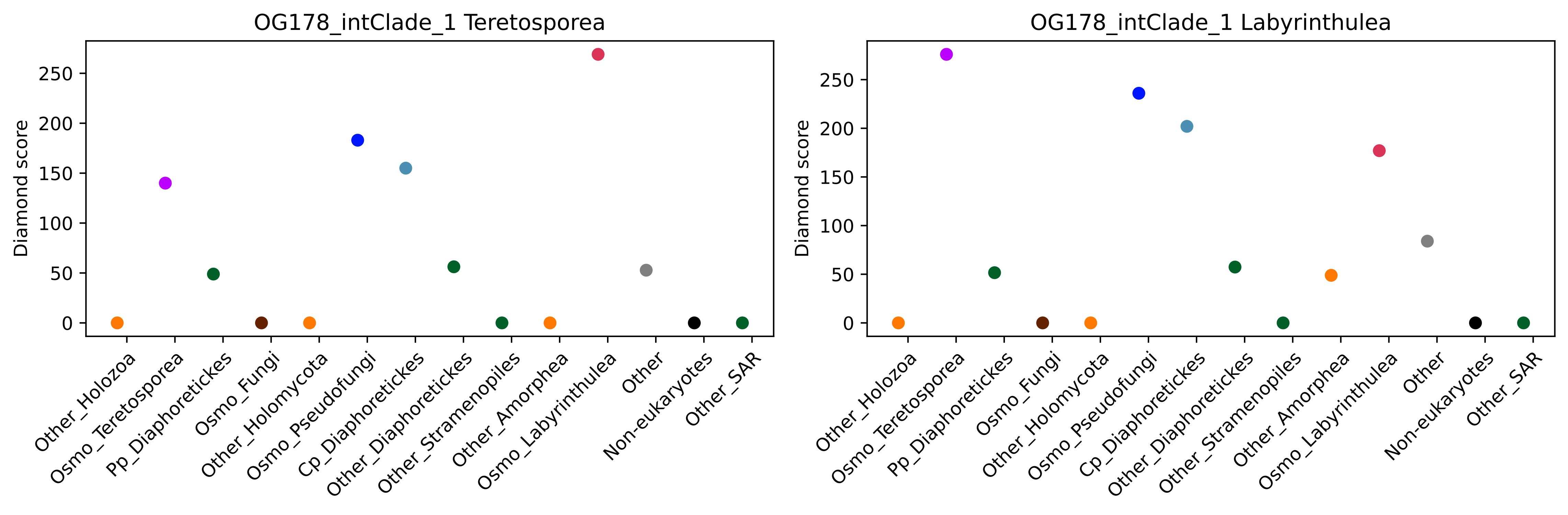
OG178_intClade_1 Teretosporea
Diamond score
0
50
100
150
200
250
Other_Holozoa
Osmo_Teretosporea
Pp_Diaphoretickes
Osmo_Fungi
Other_Holomycota
Osmo_Pseudofungi
Cp_Diaphoretickes
Other_Diaphoretickes
Other_Stramenopiles
Other_Amorphea
Osmo_Labyrinthulea
Other
Non-eukaryotes
Other_SAR
OG178_intClade_1 Labyrinthulea
Diamond score
0
50
100
150
200
250
Other_Holozoa
Osmo_Teretosporea
Pp_Diaphoretickes
Osmo_Fungi
Other_Holomycota
Osmo_Pseudofungi
Cp_Diaphoretickes
Other_Diaphoretickes
Other_Stramenopiles
Other_Amorphea
Osmo_Labyrinthulea
Other
Non-eukaryotes
Other_SAR

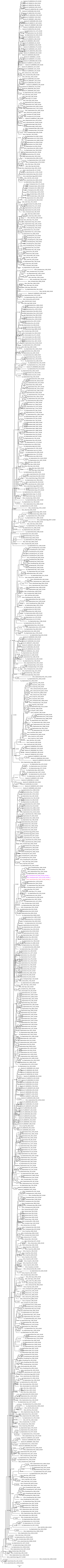

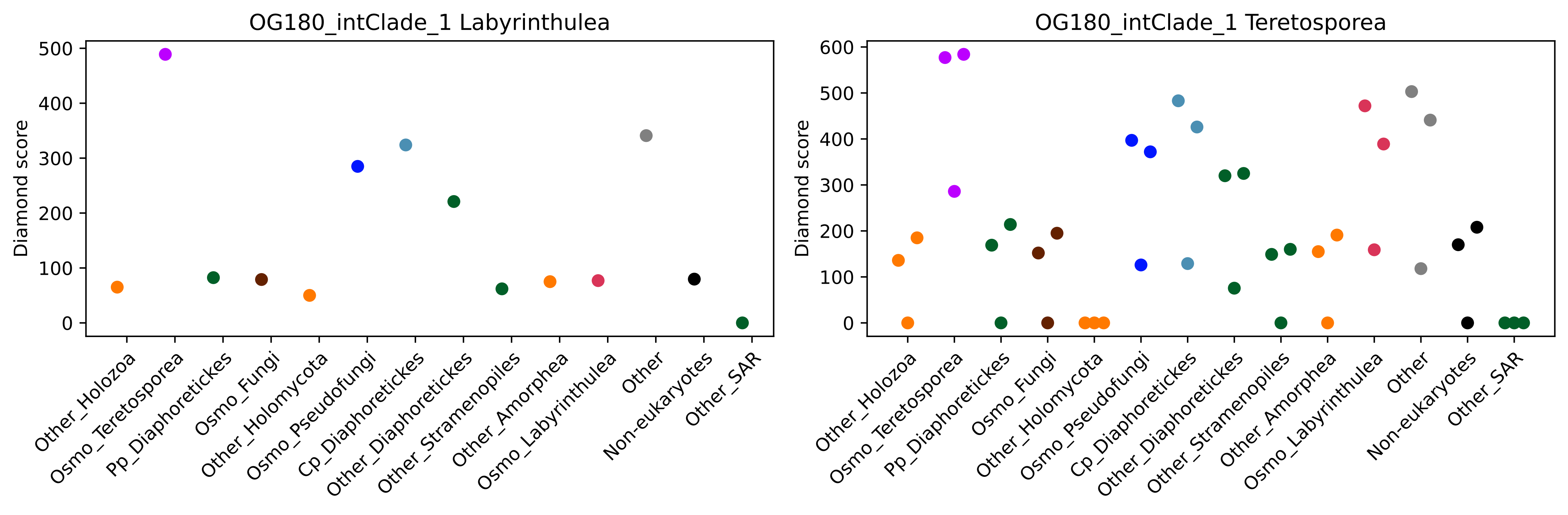

OG180_intClade_1 Labyrinthulea
Diamond score
0
100
200
300
400
500
Other_Holozoa
Osmo_Teretosporea
Pp_Diaphoretickes
Osmo_Fungi
Other_Holomycota
Osmo_Pseudofungi
Cp_Diaphoretickes
Other_Diaphoretickes
Other_Stramenopiles
Other_Amorphea
Osmo_Labyrinthulea
Other
Non-eukaryotes
Other_SAR
OG180_intClade_1 Teretosporea
Diamond score
0
100
200
300
400
500
600
Other_Holozoa
Osmo_Teretosporea
Pp_Diaphoretickes
Osmo_Fungi
Other_Holomycota
Osmo_Pseudofungi
Cp_Diaphoretickes
Other_Diaphoretickes
Other_Stramenopiles
Other_Amorphea
Osmo_Labyrinthulea
Other
Non-eukaryotes
Other_SAR

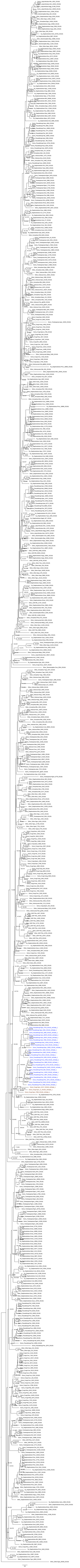

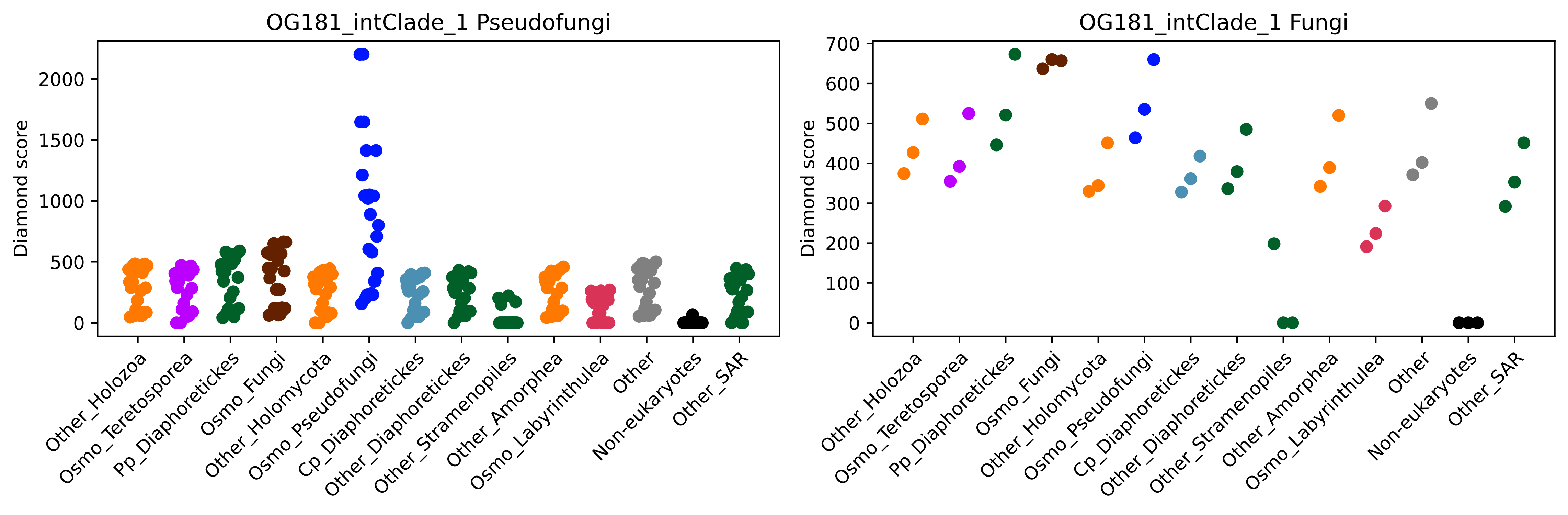
OG181_intClade_1 Pseudofungi
OG181_intClade_1 Fungi
Diamond score
0
500
1000
1500
2000
100
200
300
400
500
600
700
Other_Holozoa
Osmo_Teretosporea
Pp_Diaphoretickes
Osmo_Fungi
Other_Holomycota
Osmo_Pseudofungi
Cp_Diaphoretickes
Other_Diaphoretickes
Other_Stramenopiles
Other_Amorphea
Osmo_Labyrinthulea
Other
Non-eukaryotes
Other_SAR

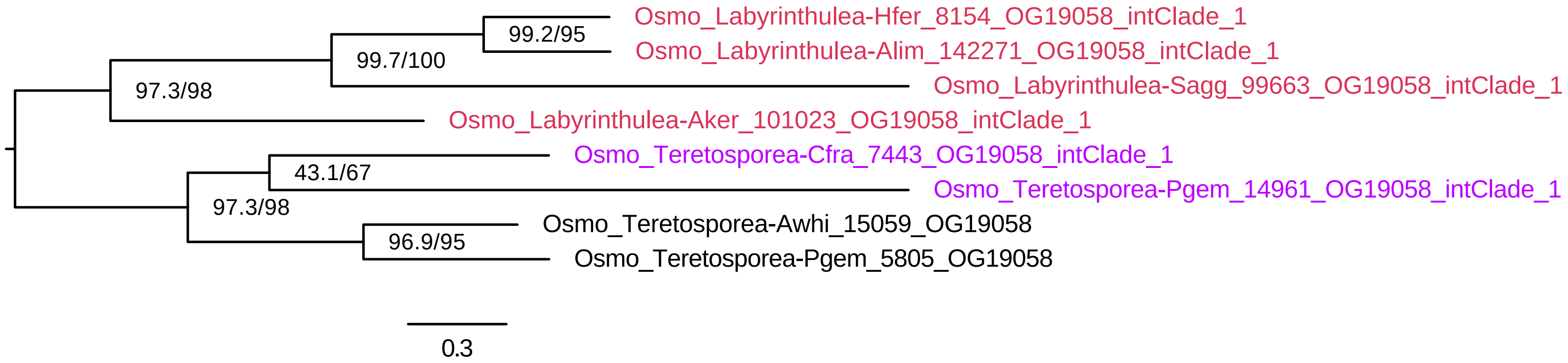

Osmo_Labyrinthulea-Hfer_8154_OG19058_intClade_1
99.2/95
Osmo_Labyrinthulea-Alim_142271_OG19058_intClade_1
99.7/100
Osmo_Labyrinthulea-Sagg_99663_OG19058_intClade_1
97.3/98
Osmo_Labyrinthulea-Aker_101023_OG19058_intClade_1
Osmo_Teretosporea-Cfra_7443_OG19058_intClade_1
43.1/67
Osmo_Teretosporea-Pgem_14961_OG19058_intClade_1
97.3/98
Osmo_Teretosporea-Awhi_15059_OG19058
96.9/95
Osmo_Teretosporea-Pgem_5805_OG19058
0.3

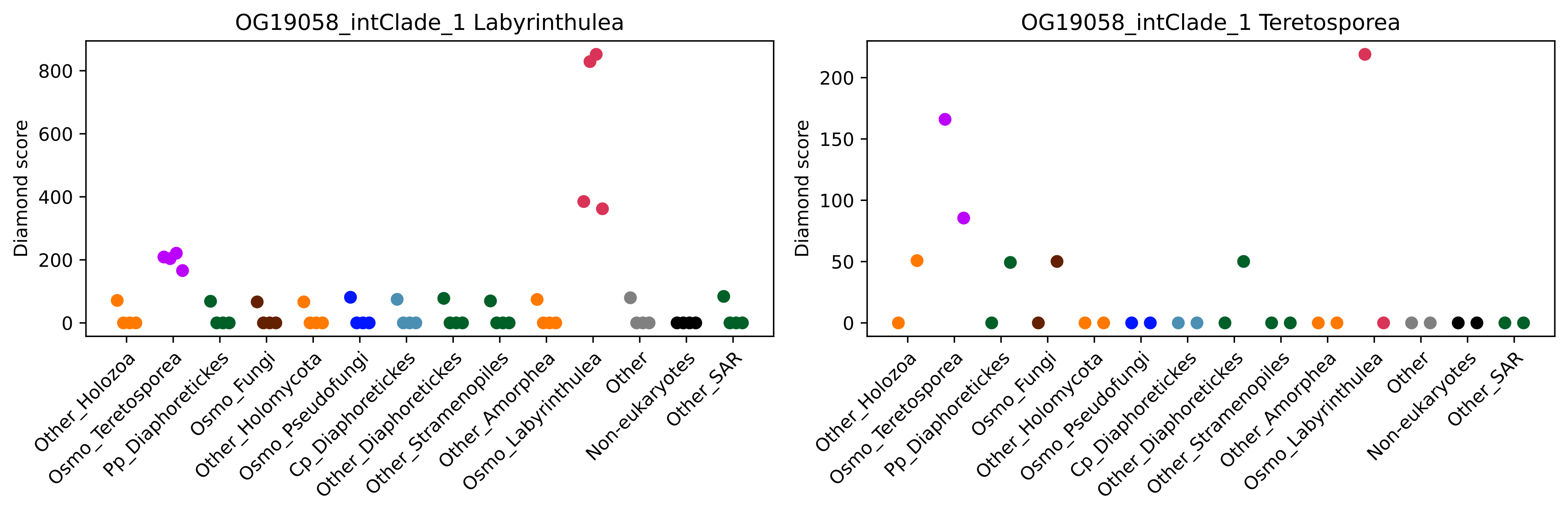

OG19058_intClade_1 Labyrinthulea
OG19058_intClade_1 Teretosporea
Diamond score
800
600
400
200
0
150
100
50
Other_Holozoa
Osmo_Teretosporea
Pp_Diaphoretickes
Osmo_Fungi
Other_Holomycota
Osmo_Pseudofungi
Cp_Diaphoretickes
Other_Diaphoretickes
Other_Stramenopiles
Other_Amorphea
Osmo_Labyrinthulea
Other
Non-eukaryotes
Other_SAR

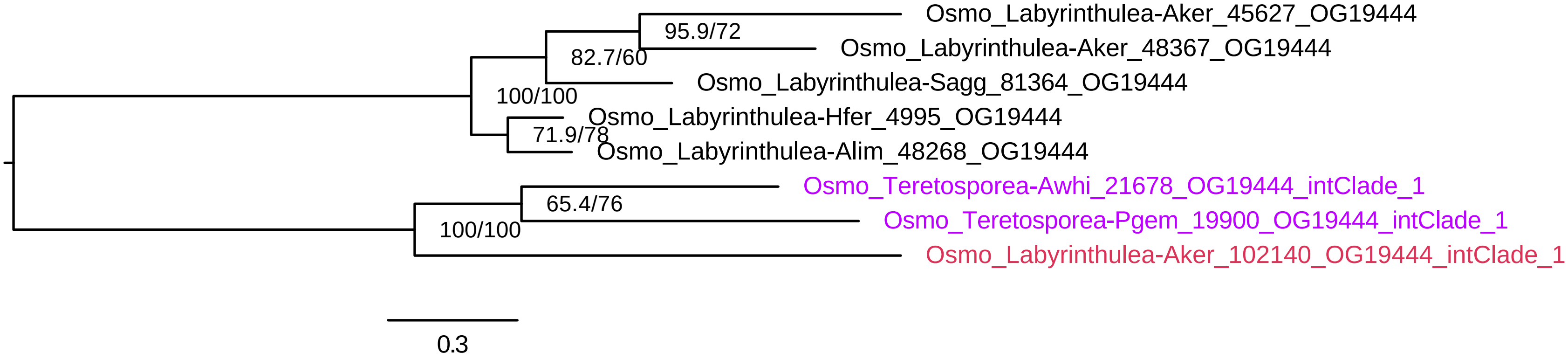
Osmo_Labyrinthulea-Aker_45627_OG19444
95.9/72
Osmo_Labyrinthulea-Aker_48367_OG19444
82.7/60
Osmo_Labyrinthulea-Sagg_81364_OG19444
100/100
Osmo_Labyrinthulea-Hfer_4995_OG19444
71.9/78
Osmo_Labyrinthulea-Alim_48268_OG19444
Osmo_Teretosporea-Awhi_21678_OG19444_intClade_1
65.4/76
Osmo_Teretosporea-Pgem_19900_OG19444_intClade_1
100/100
Osmo_Labyrinthulea-Aker_102140_OG19444_intClade_1
0.3

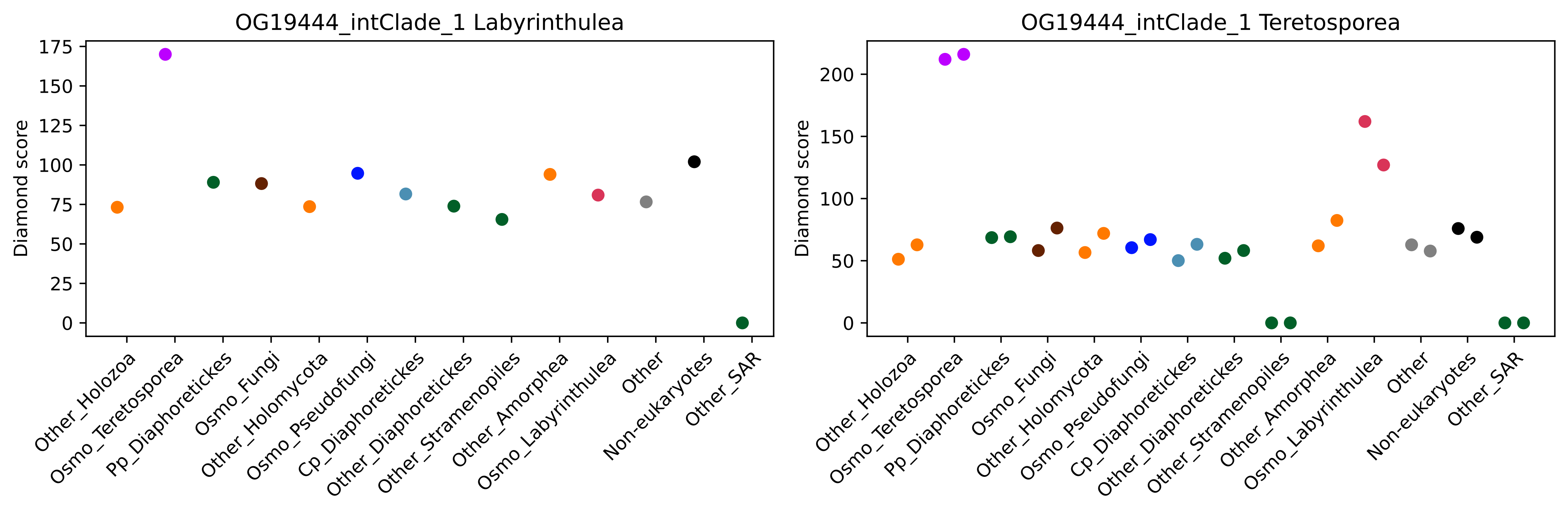

OG19444_intClade_1 Labyrinthulea
Diamond score
0
25
50
75
100
125
150
175
Other_Holozoa
Osmo_Teretosporea
Pp_Diaphoretickes
Osmo_Fungi
Other_Holomycota
Osmo_Pseudofungi
Cp_Diaphoretickes
Other_Diaphoretickes
Other_Stramenopiles
Other_Amorphea
Osmo_Labyrinthulea
Other
Non-eukaryotes
Other_SAR
OG19444_intClade_1 Teretosporea
Diamond score
0
50
100
150
200
Other_Holozoa
Osmo_Teretosporea
Pp_Diaphoretickes
Osmo_Fungi
Other_Holomycota
Osmo_Pseudofungi
Cp_Diaphoretickes
Other_Diaphoretickes
Other_Stramenopiles
Other_Amorphea
Osmo_Labyrinthulea
Other
Non-eukaryotes
Other_SAR

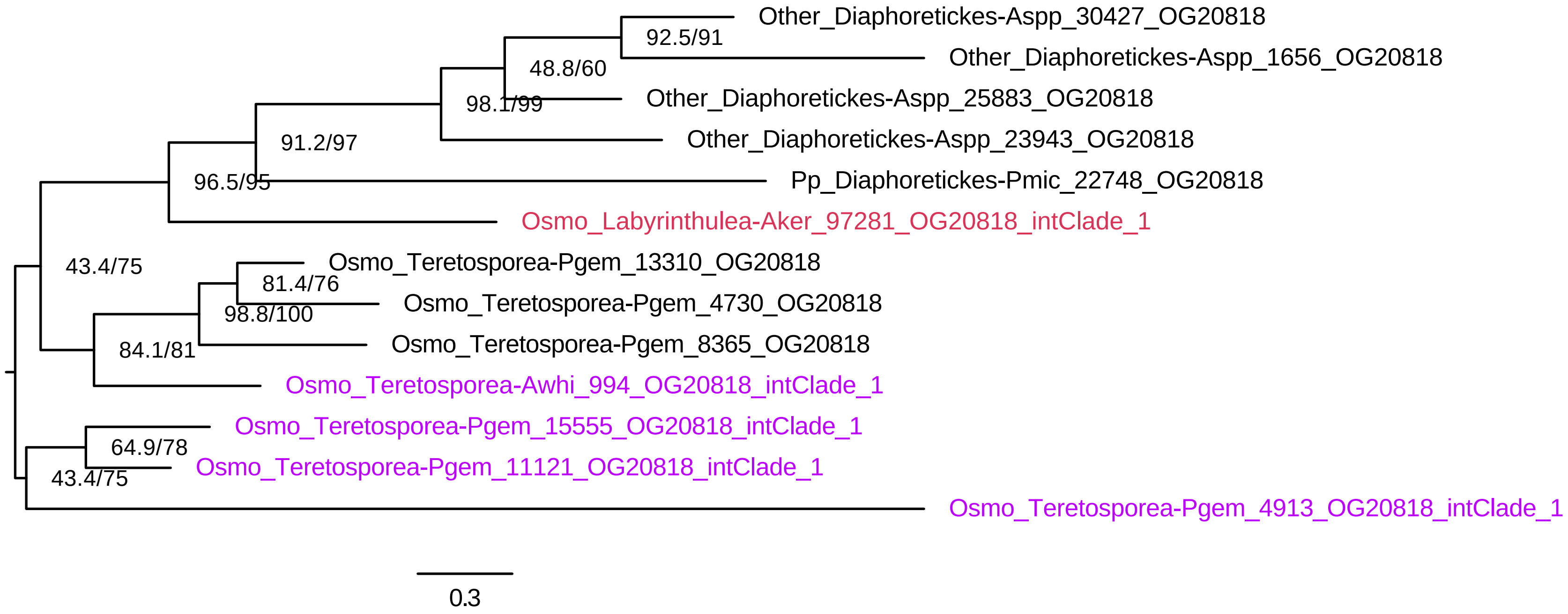
Other_Diaphoretickes-Aspp_30427_OG20818
92.5/91
Other_Diaphoretickes-Aspp_1656_OG20818
48.8/60
Other_Diaphoretickes-Aspp_25883_OG20818
98.1/99
Other_Diaphoretickes-Aspp_23943_OG20818
91.2/97
Pp_Diaphoretickes-Pmic_22748_OG20818
96.5/95
Osmo_Labyrinthulea-Aker_97281_OG20818_intClade_1
43.4/75
Osmo_Teretosporea-Pgem_13310_OG20818
81.4/76
Osmo_Teretosporea-Pgem_4730_OG20818
98.8/100
Osmo_Teretosporea-Pgem_8365_OG20818
84.1/81
Osmo_Teretosporea-Awhi_994_OG20818_intClade_1
Osmo_Teretosporea-Pgem_15555_OG20818_intClade_1
64.9/78
Osmo_Teretosporea-Pgem_11121_OG20818_intClade_1
43.4/75
Osmo_Teretosporea-Pgem_4913_OG20818_intClade_1
0.3

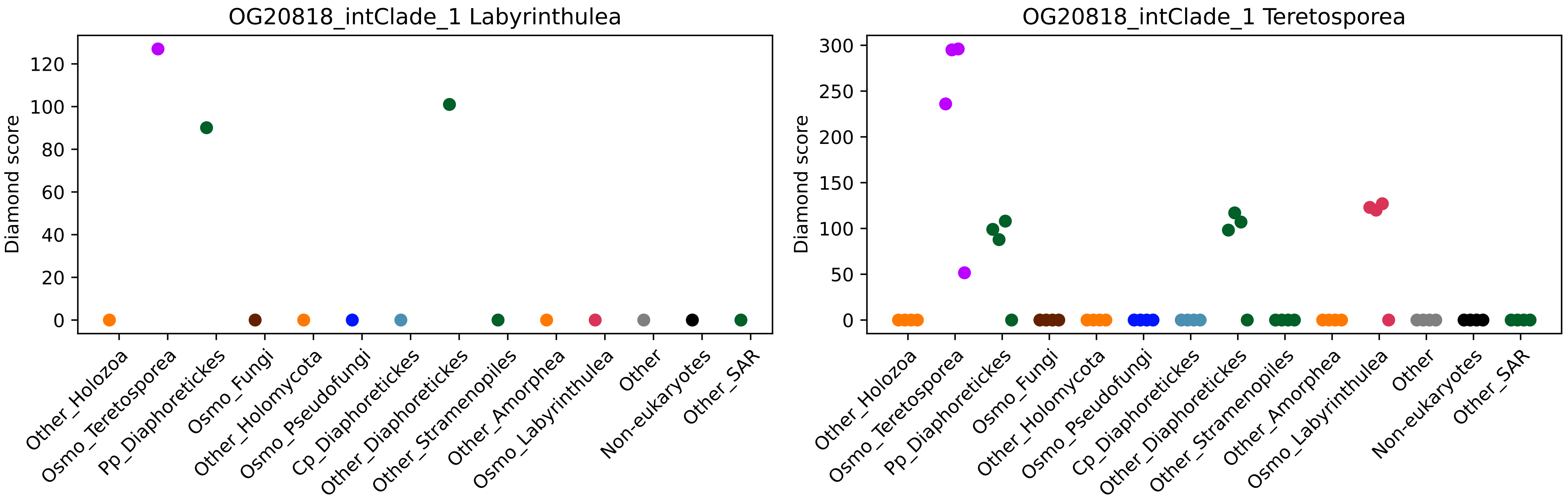
OG20818_intClade_1 Labyrinthulea
Diamond score
0
20
40
60
80
100
120
Other_Holozoa
Osmo_Teretosporea
Pp_Diaphoretickes
Osmo_Fungi
Other_Holomycota
Osmo_Pseudofungi
Cp_Diaphoretickes
Other_Diaphoretickes
Other_Stramenopiles
Other_Amorphea
Osmo_Labyrinthulea
Other
Non-eukaryotes
Other_SAR
OG20818_intClade_1 Teretosporea
Diamond score
0
50
100
150
200
250
300
Other_Holozoa
Osmo_Teretosporea
Pp_Diaphoretickes
Osmo_Fungi
Other_Holomycota
Osmo_Pseudofungi
Cp_Diaphoretickes
Other_Diaphoretickes
Other_Stramenopiles
Other_Amorphea
Osmo_Labyrinthulea
Other
Non-eukaryotes
Other_SAR

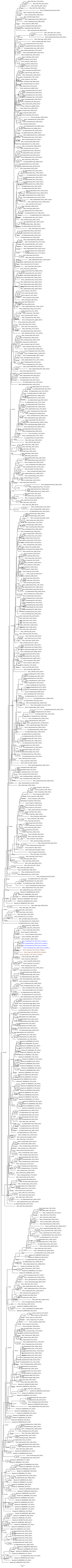

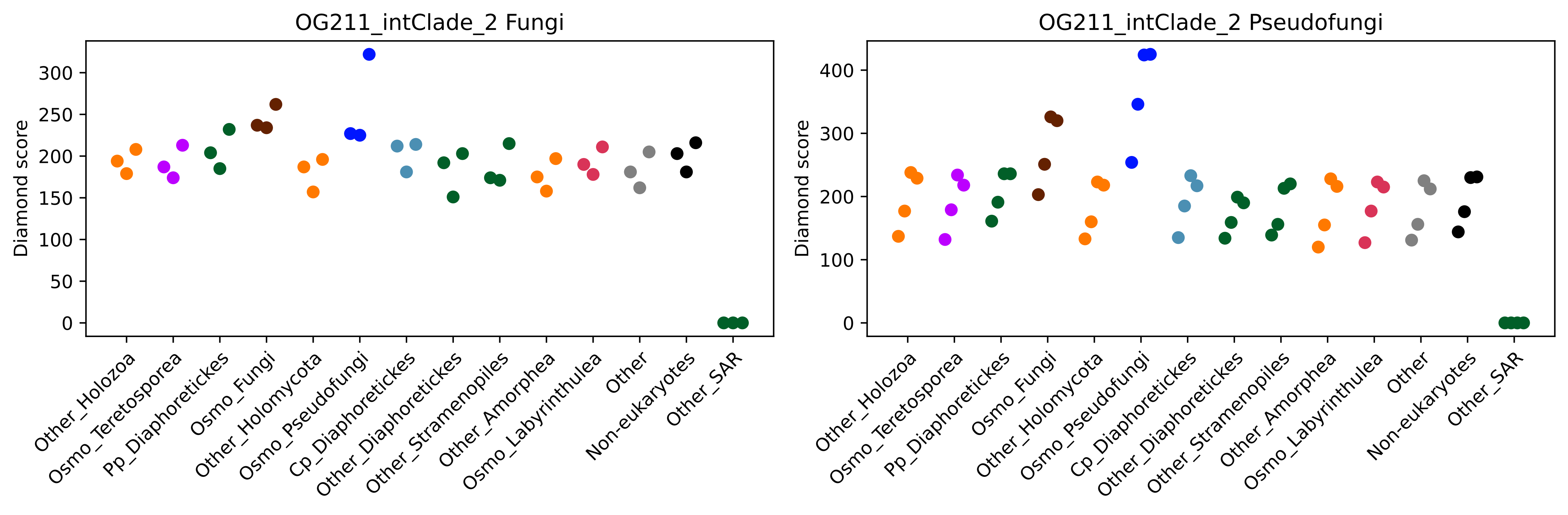
OG211_intClade_2 Fungi
OG211_intClade_2 Pseudofungi
Diamond score
0
50
100
150
200
250
300
400
Other_Holozoa
Osmo_Teretosporea
Pp_Diaphoretickes
Osmo_Fungi
Other_Holomycota
Osmo_Pseudofungi
Cp_Diaphoretickes
Other_Diaphoretickes
Other_Stramenopiles
Other_Amorphea
Osmo_Labyrinthulea
Other
Non-eukaryotes
Other_SAR

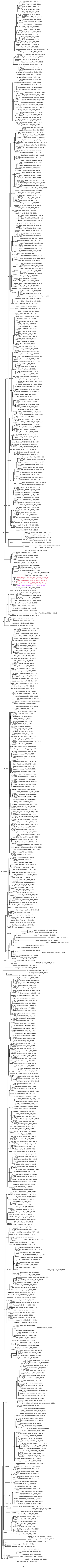

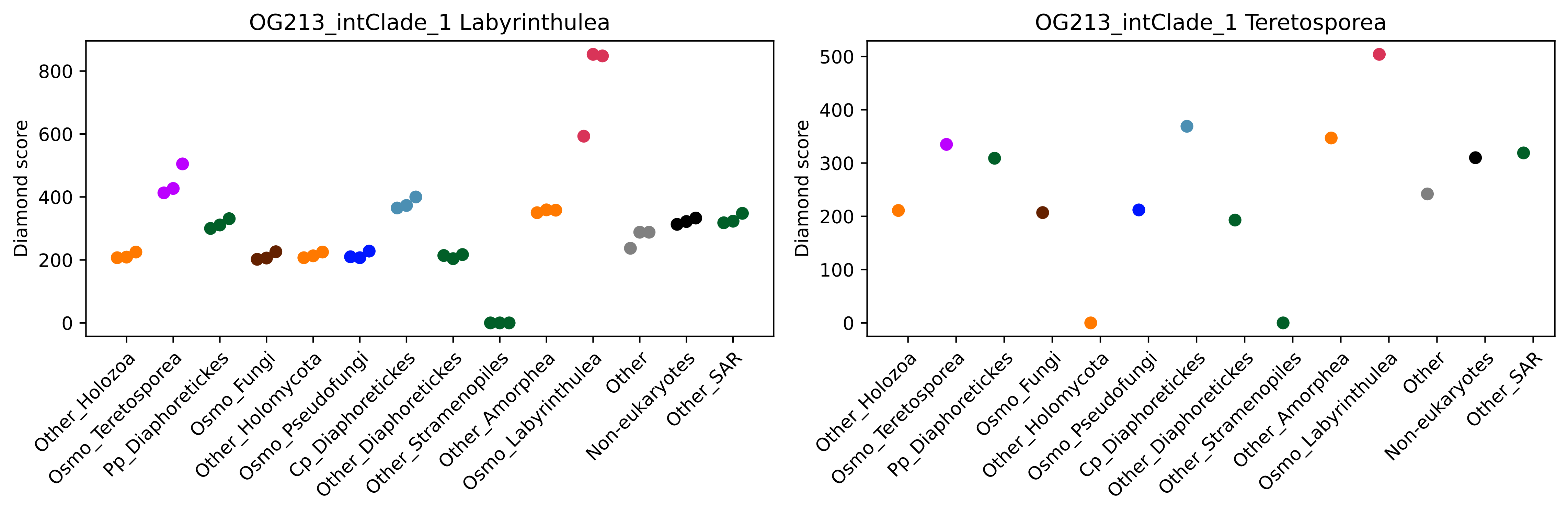
OG213_intClade_1 Labyrinthulea
OG213_intClade_1 Teretosporea
Diamond score
Other_Holozoa
Osmo_Teretosporea
Pp_Diaphoretickes
Osmo_Fungi
Other_Holomycota
Osmo_Pseudofungi
Cp_Diaphoretickes
Other_Diaphoretickes
Other_Stramenopiles
Other_Amorphea
Osmo_Labyrinthulea
Other
Non-eukaryotes
Other_SAR

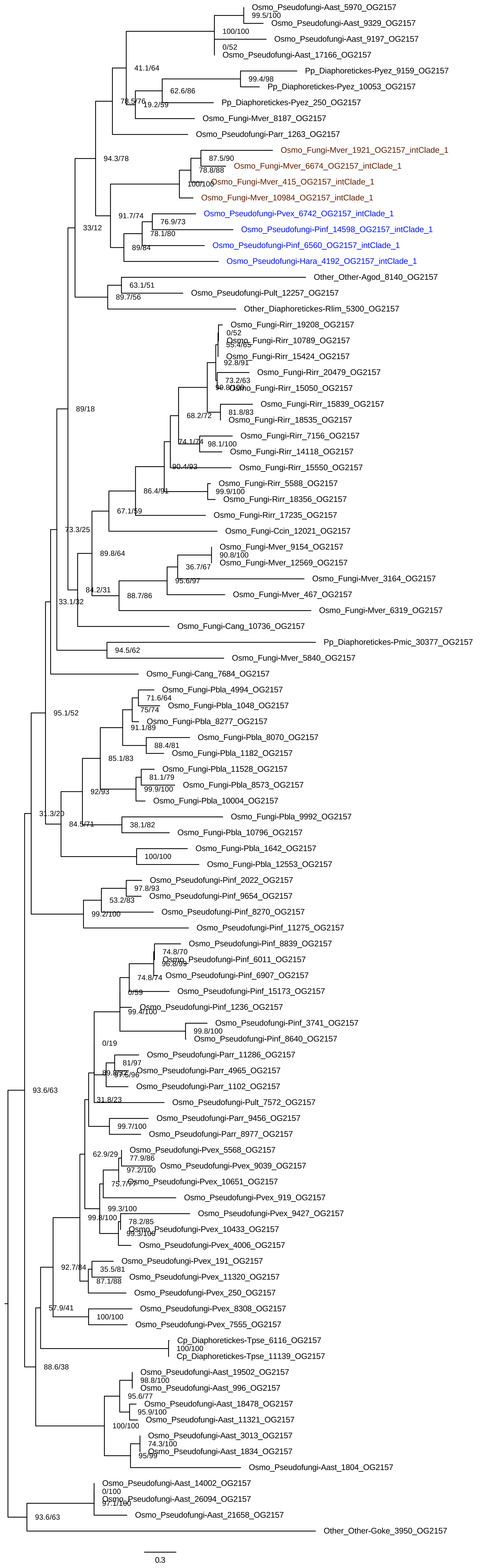

Osmo_Pseudofungi-Aast_5970_OG2157
99.5/100
Osmo_Pseudofungi-Aast_9329_OG2157
100/100
Osmo_Pseudofungi-Aast_9197_OG2157
0/52
Osmo_Pseudofungi-Aast_17166_OG2157
41.1/64
Pp_Diaphoretickes-Pyez_9159_OG2157
99.4/98
Pp_Diaphoretickes-Pyez_10053_OG2157
62.6/86
78.5/76
19.2/59
Pp_Diaphoretickes-Pyez_250_OG2157
Osmo_Fungi-Mver_8187_OG2157
Osmo_Pseudofungi-Parr_1263_OG2157
94.3/78
Osmo_Fungi-Mver_1921_OG2157_intClade_1
87.5/90
Osmo_Fungi-Mver_6674_OG2157_intClade_1
78.8/88
100/100
Osmo_Fungi-Mver_415_OG2157_intClade_1
Osmo_Fungi-Mver_10984_OG2157_intClade_1
91.7/74
Osmo_Pseudofungi-Pvex_6742_OG2157_intClade_1
76.9/73
33/12
Osmo_Pseudofungi-Pinf_14598_OG2157_intClade_1
78.1/80
Osmo_Pseudofungi-Pinf_6560_OG2157_intClade_1
89/84
Osmo_Pseudofungi-Hara_4192_OG2157_intClade_1
Other_Other-Agod_8140_OG2157
63.1/51
Osmo_Pseudofungi-Pult_12257_OG2157
89.7/56
Other_Diaphoretickes-Rlim_5300_OG2157
Osmo_Fungi-Rirr_19208_OG2157
0/52
Osmo_Fungi-Rirr_10789_OG2157
55.4/65
Osmo_Fungi-Rirr_15424_OG2157
92.8/91
Osmo_Fungi-Rirr_20479_OG2157
73.2/63
99.8/100
Osmo_Fungi-Rirr_15050_OG2157
89/18
Osmo_Fungi-Rirr_15839_OG2157
68.2/72
81.8/83
Osmo_Fungi-Rirr_18535_OG2157
Osmo_Fungi-Rirr_7156_OG2157
74.1/74
98.1/100
Osmo_Fungi-Rirr_14118_OG2157
90.4/93
Osmo_Fungi-Rirr_15550_OG2157
Osmo_Fungi-Rirr_5588_OG2157
86.4/91
99.9/100
Osmo_Fungi-Rirr_18356_OG2157
67.1/59
Osmo_Fungi-Rirr_17235_OG2157
73.3/25
Osmo_Fungi-Ccin_12021_OG2157
Osmo_Fungi-Mver_9154_OG2157
89.8/64
90.8/100
Osmo_Fungi-Mver_12569_OG2157
36.7/67
Osmo_Fungi-Mver_3164_OG2157
95.6/97
84.2/31
Osmo_Fungi-Mver_467_OG2157
88.7/86
33.1/32
Osmo_Fungi-Mver_6319_OG2157
Osmo_Fungi-Cang_10736_OG2157
Pp_Diaphoretickes-Pmic_30377_OG2157
94.5/62
Osmo_Fungi-Mver_5840_OG2157
Osmo_Fungi-Cang_7684_OG2157
Osmo_Fungi-Pbla_4994_OG2157
71.6/64
95.1/52
Osmo_Fungi-Pbla_1048_OG2157
75/74
Osmo_Fungi-Pbla_8277_OG2157
91.1/89
Osmo_Fungi-Pbla_8070_OG2157
88.4/81
Osmo_Fungi-Pbla_1182_OG2157
85.1/83
Osmo_Fungi-Pbla_11528_OG2157
81.1/79
Osmo_Fungi-Pbla_8573_OG2157
92/93
99.9/100
Osmo_Fungi-Pbla_10004_OG2157
31.3/20
Osmo_Fungi-Pbla_9992_OG2157
84.5/71
38.1/82
Osmo_Fungi-Pbla_10796_OG2157
Osmo_Fungi-Pbla_1642_OG2157
100/100
Osmo_Fungi-Pbla_12553_OG2157
Osmo_Pseudofungi-Pinf_2022_OG2157
97.8/93
Osmo_Pseudofungi-Pinf_9654_OG2157
53.2/83
99.2/100
Osmo_Pseudofungi-Pinf_8270_OG2157
Osmo_Pseudofungi-Pinf_11275_OG2157
Osmo_Pseudofungi-Pinf_8839_OG2157
74.8/70
Osmo_Pseudofungi-Pinf_6011_OG2157
96.8/99
74.8/74
Osmo_Pseudofungi-Pinf_6907_OG2157
0/59
Osmo_Pseudofungi-Pinf_15173_OG2157
99.4/100
Osmo_Pseudofungi-Pinf_1236_OG2157
Osmo_Pseudofungi-Pinf_3741_OG2157
99.8/100
Osmo_Pseudofungi-Pinf_8640_OG2157
0/19
Osmo_Pseudofungi-Parr_11286_OG2157
81/97
89.8/37
97.5/96
Osmo_Pseudofungi-Parr_4965_OG2157
93.6/63
Osmo_Pseudofungi-Parr_1102_OG2157
31.8/23
Osmo_Pseudofungi-Pult_7572_OG2157
Osmo_Pseudofungi-Parr_9456_OG2157
99.7/100
Osmo_Pseudofungi-Parr_8977_OG2157
62.9/29
Osmo_Pseudofungi-Pvex_5568_OG2157
77.9/86
Osmo_Pseudofungi-Pvex_9039_OG2157
97.2/100
75.7/77
Osmo_Pseudofungi-Pvex_10651_OG2157
Osmo_Pseudofungi-Pvex_919_OG2157
99.3/100
Osmo_Pseudofungi-Pvex_9427_OG2157
99.8/100
78.2/85
Osmo_Pseudofungi-Pvex_10433_OG2157
99.3/100
Osmo_Pseudofungi-Pvex_4006_OG2157
Osmo_Pseudofungi-Pvex_191_OG2157
92.7/84
35.5/81
Osmo_Pseudofungi-Pvex_11320_OG2157
87.1/88
Osmo_Pseudofungi-Pvex_250_OG2157
57.9/41
Osmo_Pseudofungi-Pvex_8308_OG2157
100/100
Osmo_Pseudofungi-Pvex_7555_OG2157
Cp_Diaphoretickes-Tpse_6116_OG2157
100/100
Cp_Diaphoretickes-Tpse_11139_OG2157
88.6/38
Osmo_Pseudofungi-Aast_19502_OG2157
98.8/100
Osmo_Pseudofungi-Aast_996_OG2157
95.6/77
Osmo_Pseudofungi-Aast_18478_OG2157
95.9/100
100/100
Osmo_Pseudofungi-Aast_11321_OG2157
Osmo_Pseudofungi-Aast_3013_OG2157
74.3/100
Osmo_Pseudofungi-Aast_1834_OG2157
95/99
Osmo_Pseudofungi-Aast_1804_OG2157
Osmo_Pseudofungi-Aast_14002_OG2157
0/100
Osmo_Pseudofungi-Aast_26094_OG2157
97.1/100
93.6/63
Osmo_Pseudofungi-Aast_21658_OG2157
Other_Other-Goke_3950_OG2157
0.3

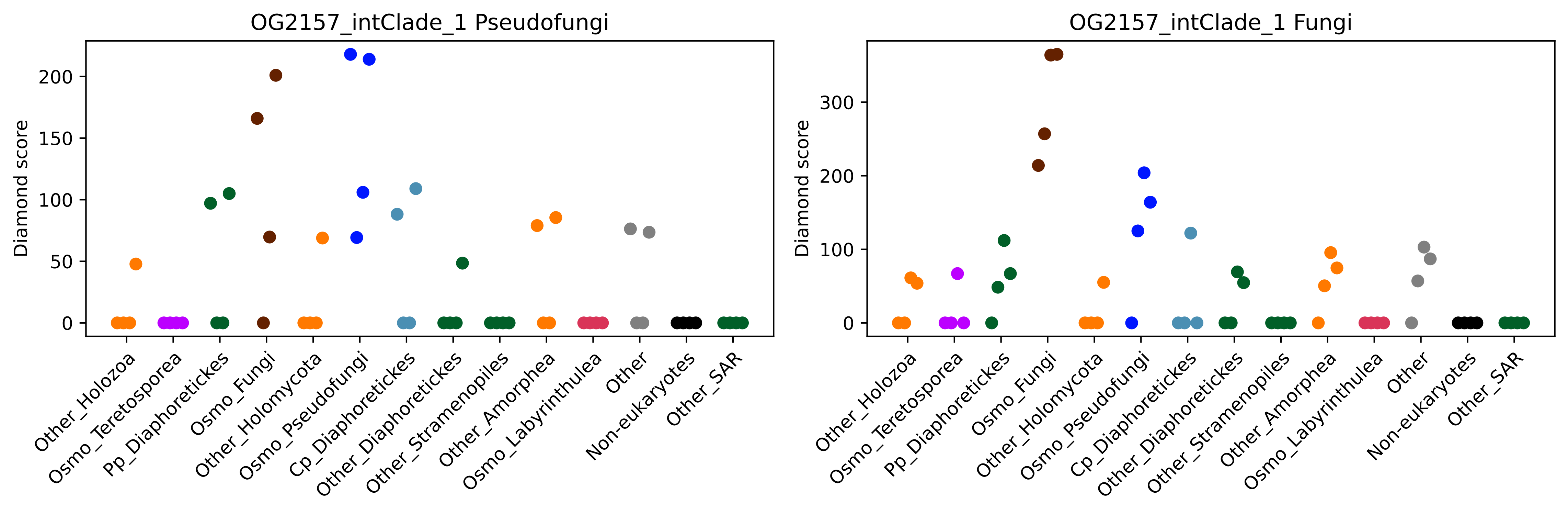
OG2157_intClade_1 Pseudofungi
OG2157_intClade_1 Fungi
Diamond score
0
50
100
150
200
300
Other_Holozoa
Osmo_Teretosporea
Pp_Diaphoretickes
Osmo_Fungi
Other_Holomycota
Osmo_Pseudofungi
Cp_Diaphoretickes
Other_Diaphoretickes
Other_Stramenopiles
Other_Amorphea
Osmo_Labyrinthulea
Other
Non-eukaryotes
Other_SAR

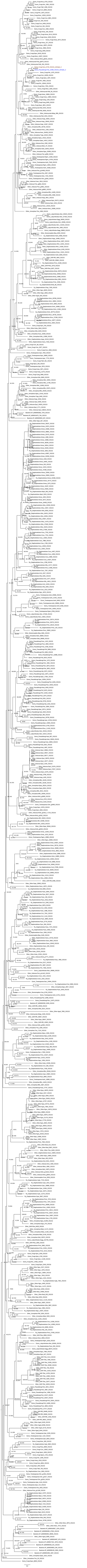

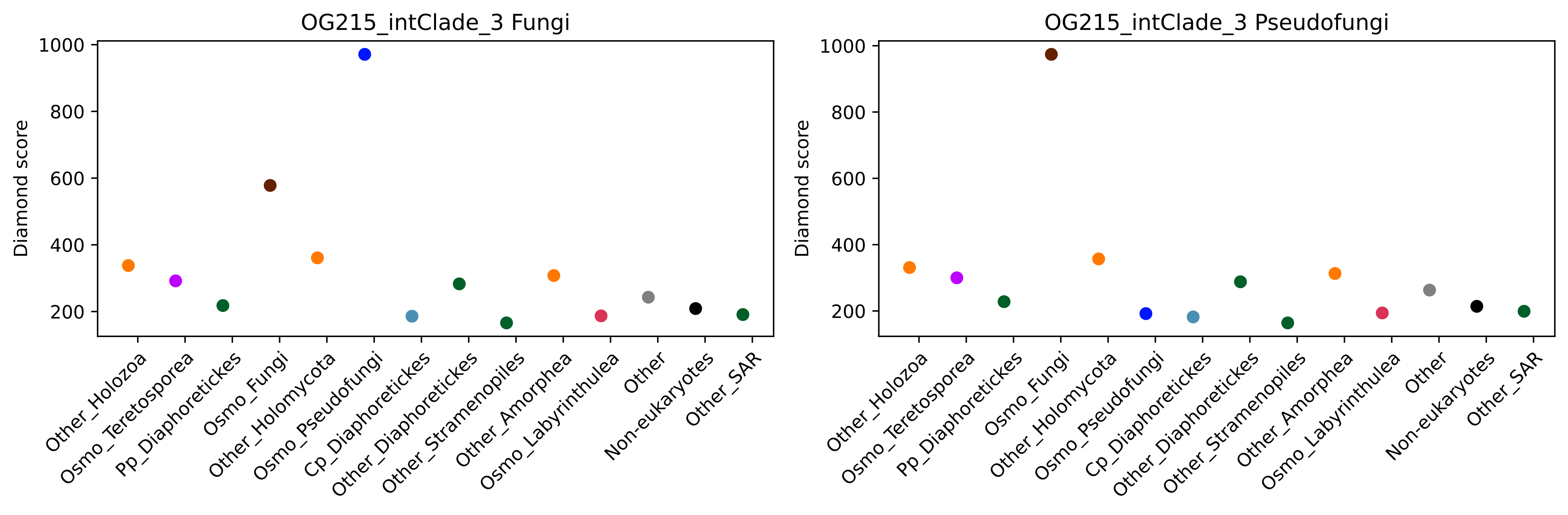
OG215_intClade_3 Fungi
OG215_intClade_3 Pseudofungi
Diamond score
200
400
600
800
1000
Other_Holozoa
Osmo_Teretosporea
Pp_Diaphoretickes
Osmo_Fungi
Other_Holomycota
Osmo_Pseudofungi
Cp_Diaphoretickes
Other_Diaphoretickes
Other_Stramenopiles
Other_Amorphea
Osmo_Labyrinthulea
Other
Non-eukaryotes
Other_SAR

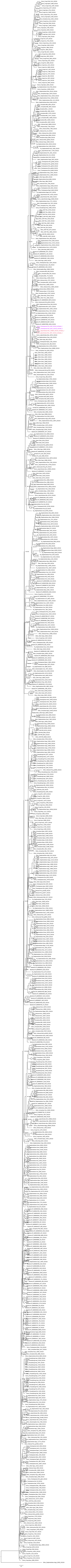

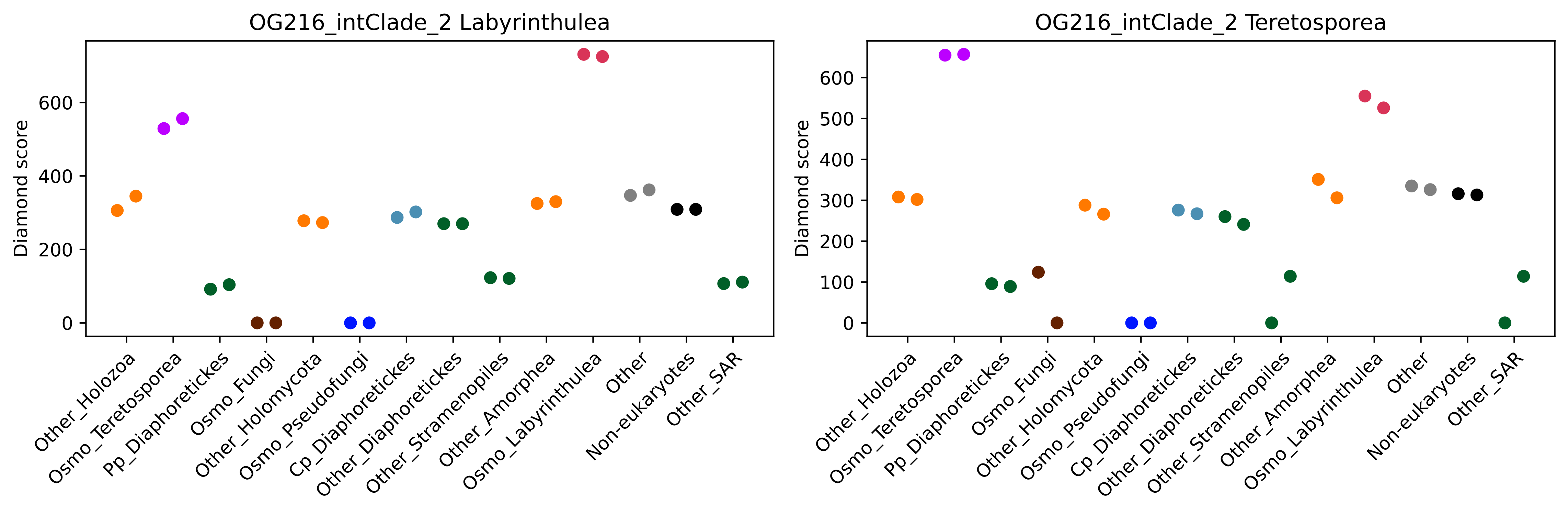

OG216_intClade_2 Labyrinthulea
Diamond score
0
200
400
600
Other_Holozoa
Osmo_Teretosporea
Pp_Diaphoretickes
Osmo_Fungi
Other_Holomycota
Osmo_Pseudofungi
Cp_Diaphoretickes
Other_Diaphoretickes
Other_Stramenopiles
Other_Amorphea
Osmo_Labyrinthulea
Other
Non-eukaryotes
Other_SAR
OG216_intClade_2 Teretosporea
Diamond score
0
100
200
300
400
500
600
Other_Holozoa
Osmo_Teretosporea
Pp_Diaphoretickes
Osmo_Fungi
Other_Holomycota
Osmo_Pseudofungi
Cp_Diaphoretickes
Other_Diaphoretickes
Other_Stramenopiles
Other_Amorphea
Osmo_Labyrinthulea
Other
Non-eukaryotes
Other_SAR

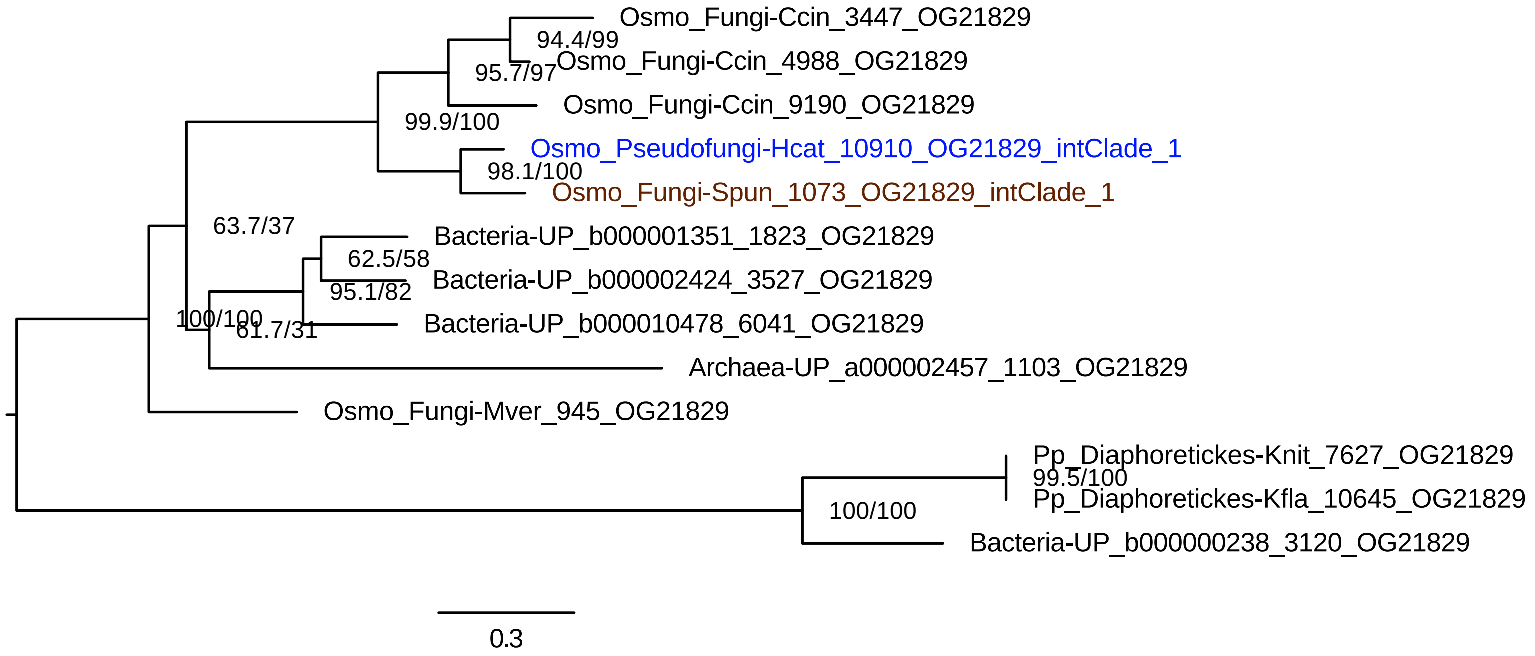
Osmo_Fungi-Ccin_3447_OG21829
94.4/99
Osmo_Fungi-Ccin_4988_OG21829
95.7/97
Osmo_Fungi-Ccin_9190_OG21829
99.9/100
Osmo_Pseudofungi-Hcat_10910_OG21829_intClade_1
98.1/100
Osmo_Fungi-Spun_1073_OG21829_intClade_1
63.7/37
Bacteria-UP_b000001351_1823_OG21829
62.5/58
Bacteria-UP_b000002424_3527_OG21829
95.1/82
100/100
61.7/31
Bacteria-UP_b000010478_6041_OG21829
Archaea-UP_a000002457_1103_OG21829
Osmo_Fungi-Mver_945_OG21829
Pp_Diaphoretickes-Knit_7627_OG21829
99.5/100
Pp_Diaphoretickes-Kfla_10645_OG21829
100/100
Bacteria-UP_b000000238_3120_OG21829
0.3

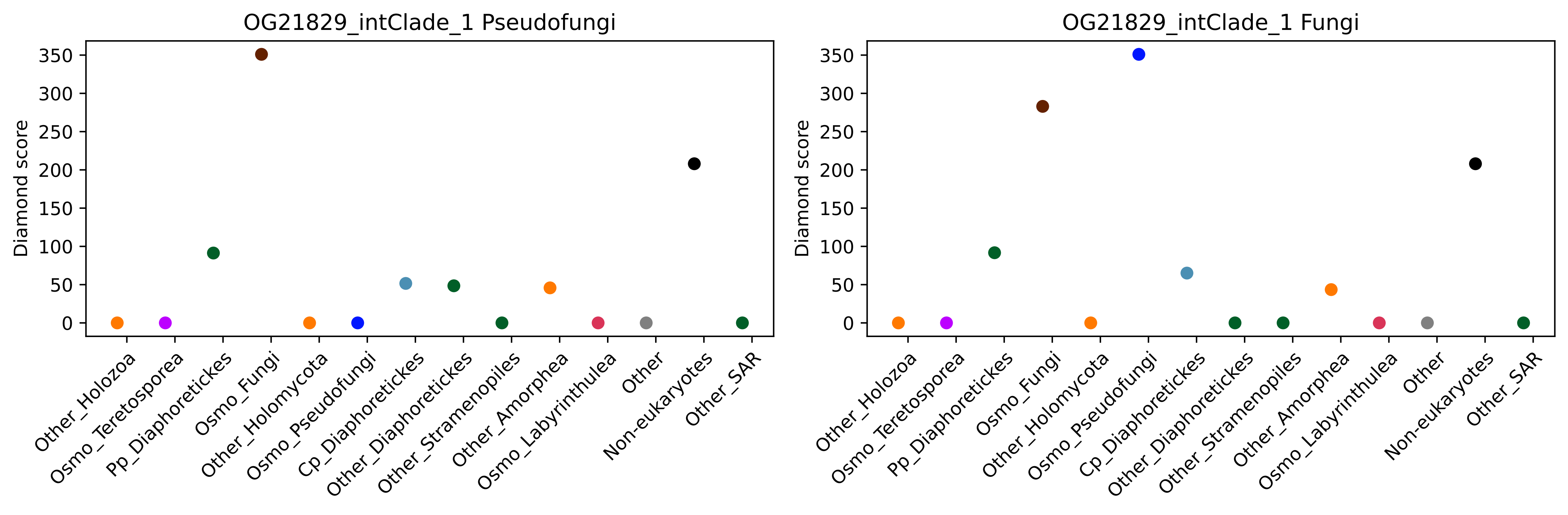

OG21829_intClade_1 Pseudofungi
OG21829_intClade_1 Fungi
Diamond score
0
50
100
150
200
250
300
350
Other_Holozoa
Osmo_Teretosporea
Pp_Diaphoretickes
Osmo_Fungi
Other_Holomycota
Osmo_Pseudofungi
Cp_Diaphoretickes
Other_Diaphoretickes
Other_Stramenopiles
Other_Amorphea
Osmo_Labyrinthulea
Other
Non-eukaryotes
Other_SAR

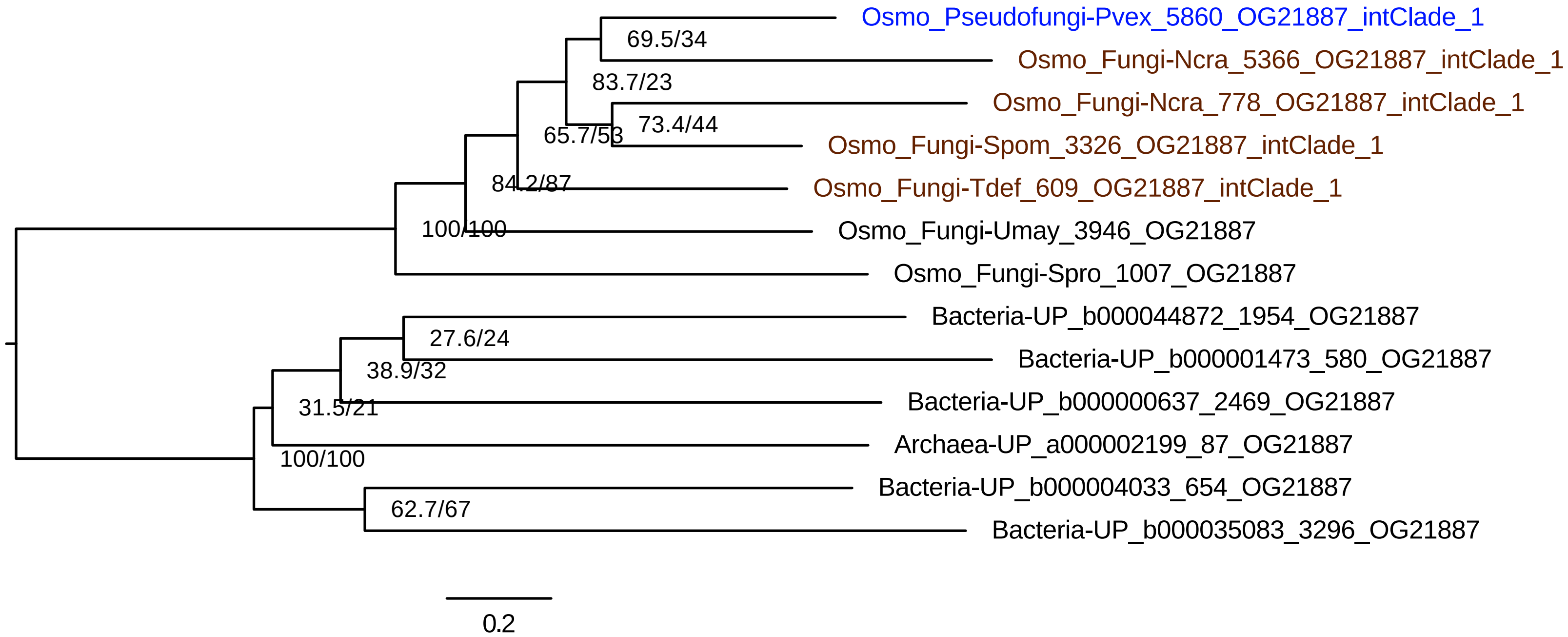
Osmo_Pseudofungi-Pvex_5860_OG21887_intClade_1
Osmo_Fungi-Ncra_5366_OG21887_intClade_1
Osmo_Fungi-Ncra_778_OG21887_intClade_1
Osmo_Fungi-Spom_3326_OG21887_intClade_1
Osmo_Fungi-Tdef_609_OG21887_intClade_1
Osmo_Fungi-Umay_3946_OG21887
Osmo_Fungi-Spro_1007_OG21887
Bacteria-UP_b000044872_1954_OG21887
Bacteria-UP_b000001473_580_OG21887
Bacteria-UP_b000000637_2469_OG21887
Archaea-UP_a000002199_87_OG21887
Bacteria-UP_b000004033_654_OG21887
Bacteria-UP_b000035083_3296_OG21887
69.5/34
83.7/23
73.4/44
65.7/53
84.2/87
100/100
27.6/24
38.9/32
31.5/21
100/100
62.7/67
0.2

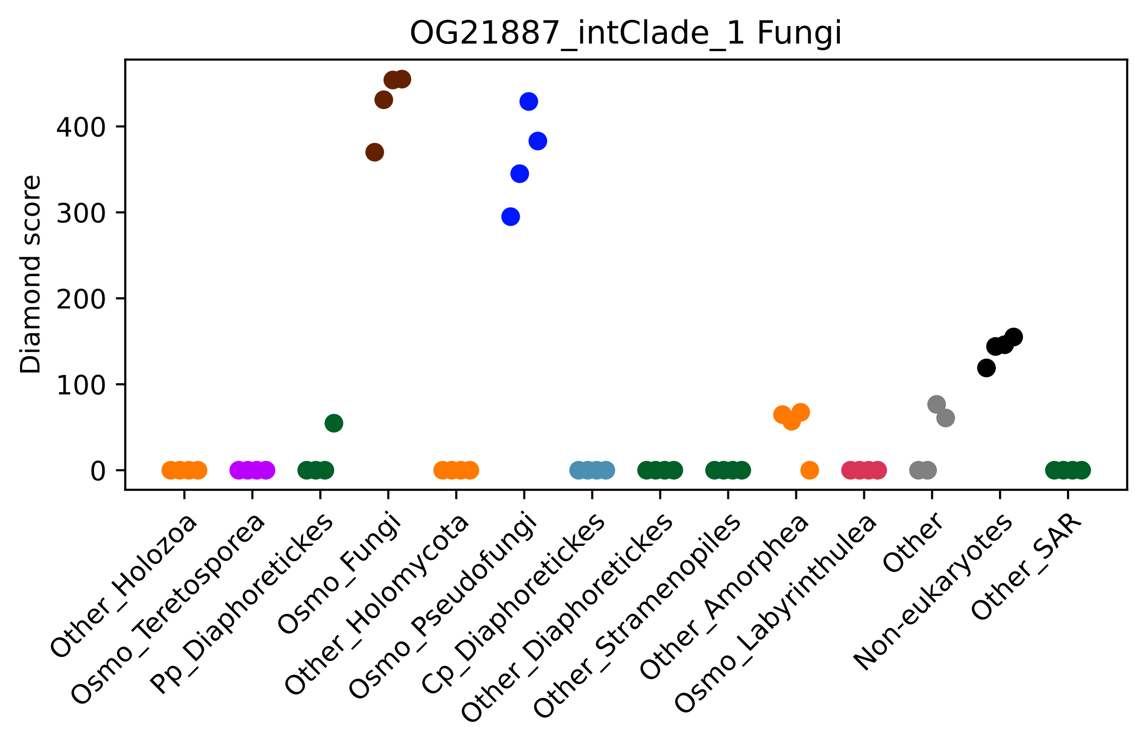
OG21887_intClade_1 Fungi
Diamond score
0
100
200
300
400
Other_Holozoa
Osmo_Teretosporea
Pp_Diaphoretickes
Osmo_Fungi
Other_Holomycota
Osmo_Pseudofungi
Cp_Diaphoretickes
Other_Diaphoretickes
Other_Stramenopiles
Other_Amorphea
Osmo_Labyrinthulea
Other
Non-eukaryotes
Other_SAR

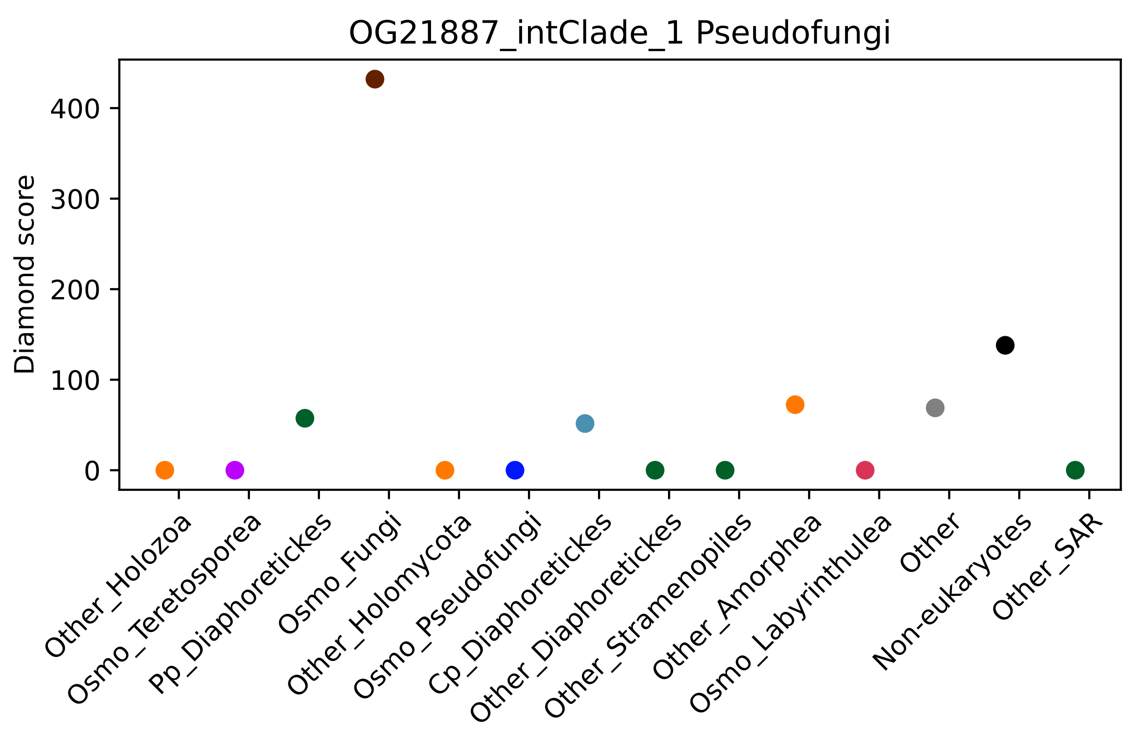
OG21887_intClade_1 Pseudofungi
Diamond score
0
100
200
300
400
Other_Holozoa
Osmo_Teretosporea
Pp_Diaphoretickes
Osmo_Fungi
Other_Holomycota
Osmo_Pseudofungi
Cp_Diaphoretickes
Other_Diaphoretickes
Other_Stramenopiles
Other_Amorphea
Osmo_Labyrinthulea
Other
Non-eukaryotes
Other_SAR

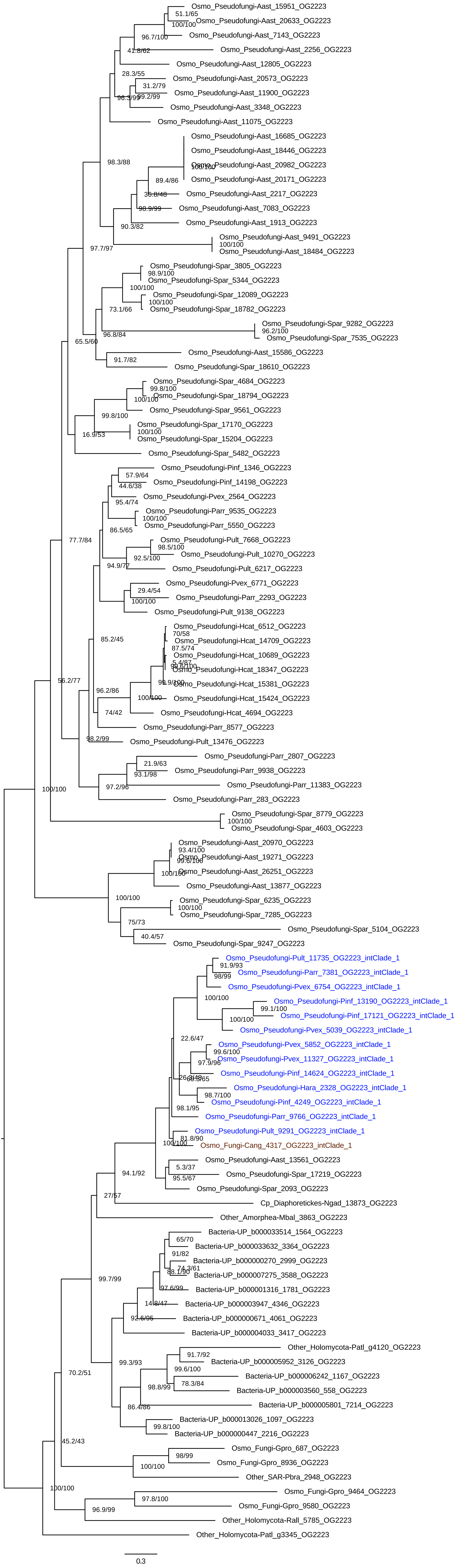
Osmo_Pseudofungi-Aast_15951_OG2223
Osmo_Pseudofungi-Aast_20633_OG2223
Osmo_Pseudofungi-Aast_7143_OG2223
Osmo_Pseudofungi-Aast_2256_OG2223
Osmo_Pseudofungi-Aast_12805_OG2223
Osmo_Pseudofungi-Aast_20573_OG2223
Osmo_Pseudofungi-Aast_11900_OG2223
Osmo_Pseudofungi-Aast_3348_OG2223
Osmo_Pseudofungi-Aast_11075_OG2223
Osmo_Pseudofungi-Aast_16685_OG2223
Osmo_Pseudofungi-Aast_18446_OG2223
Osmo_Pseudofungi-Aast_20982_OG2223
Osmo_Pseudofungi-Aast_20171_OG2223
Osmo_Pseudofungi-Aast_2217_OG2223
Osmo_Pseudofungi-Aast_7083_OG2223
Osmo_Pseudofungi-Aast_1913_OG2223
Osmo_Pseudofungi-Aast_9491_OG2223
Osmo_Pseudofungi-Aast_18484_OG2223
Osmo_Pseudofungi-Spar_3805_OG2223
Osmo_Pseudofungi-Spar_5344_OG2223
Osmo_Pseudofungi-Spar_12089_OG2223
Osmo_Pseudofungi-Spar_18782_OG2223
Osmo_Pseudofungi-Spar_9282_OG2223
Osmo_Pseudofungi-Spar_7535_OG2223
Osmo_Pseudofungi-Aast_15586_OG2223
Osmo_Pseudofungi-Spar_18610_OG2223
Osmo_Pseudofungi-Spar_4684_OG2223
Osmo_Pseudofungi-Spar_18794_OG2223
Osmo_Pseudofungi-Spar_9561_OG2223
Osmo_Pseudofungi-Spar_17170_OG2223
Osmo_Pseudofungi-Spar_15204_OG2223
Osmo_Pseudofungi-Spar_5482_OG2223
Osmo_Pseudofungi-Pinf_1346_OG2223
Osmo_Pseudofungi-Pinf_14198_OG2223
Osmo_Pseudofungi-Pvex_2564_OG2223
Osmo_Pseudofungi-Parr_9535_OG2223
Osmo_Pseudofungi-Parr_5550_OG2223
Osmo_Pseudofungi-Pult_7668_OG2223
Osmo_Pseudofungi-Pult_10270_OG2223
Osmo_Pseudofungi-Pult_6217_OG2223
Osmo_Pseudofungi-Pvex_6771_OG2223
Osmo_Pseudofungi-Parr_2293_OG2223
Osmo_Pseudofungi-Pult_9138_OG2223
Osmo_Pseudofungi-Hcat_6512_OG2223
Osmo_Pseudofungi-Hcat_14709_OG2223
Osmo_Pseudofungi-Hcat_10689_OG2223
Osmo_Pseudofungi-Hcat_18347_OG2223
Osmo_Pseudofungi-Hcat_15381_OG2223
Osmo_Pseudofungi-Hcat_15424_OG2223
Osmo_Pseudofungi-Hcat_4694_OG2223
Osmo_Pseudofungi-Parr_8577_OG2223
Osmo_Pseudofungi-Pult_13476_OG2223
Osmo_Pseudofungi-Parr_2807_OG2223
Osmo_Pseudofungi-Parr_9938_OG2223
Osmo_Pseudofungi-Parr_11383_OG2223
Osmo_Pseudofungi-Parr_283_OG2223
Osmo_Pseudofungi-Spar_8779_OG2223
Osmo_Pseudofungi-Spar_4603_OG2223
Osmo_Pseudofungi-Aast_20970_OG2223
Osmo_Pseudofungi-Aast_19271_OG2223
Osmo_Pseudofungi-Aast_26251_OG2223
Osmo_Pseudofungi-Aast_13877_OG2223
Osmo_Pseudofungi-Spar_6235_OG2223
Osmo_Pseudofungi-Spar_7285_OG2223
Osmo_Pseudofungi-Spar_5104_OG2223
Osmo_Pseudofungi-Spar_9247_OG2223
Osmo_Pseudofungi-Pult_11735_OG2223_intClade_1
Osmo_Pseudofungi-Parr_7381_OG2223_intClade_1
Osmo_Pseudofungi-Pvex_6754_OG2223_intClade_1
Osmo_Pseudofungi-Pinf_13190_OG2223_intClade_1
Osmo_Pseudofungi-Pinf_17121_OG2223_intClade_1
Osmo_Pseudofungi-Pvex_5039_OG2223_intClade_1
Osmo_Pseudofungi-Pvex_5852_OG2223_intClade_1
Osmo_Pseudofungi-Pvex_11327_OG2223_intClade_1
Osmo_Pseudofungi-Pinf_14624_OG2223_intClade_1
Osmo_Pseudofungi-Hara_2328_OG2223_intClade_1
Osmo_Pseudofungi-Pinf_4249_OG2223_intClade_1
Osmo_Pseudofungi-Parr_9766_OG2223_intClade_1
Osmo_Pseudofungi-Pult_9291_OG2223_intClade_1
Osmo_Fungi-Cang_4317_OG2223_intClade_1
Osmo_Pseudofungi-Aast_13561_OG2223
Osmo_Pseudofungi-Spar_17219_OG2223
Osmo_Pseudofungi-Spar_2093_OG2223
Cp_Diaphoretickes-Ngad_13873_OG2223
Other_Amorphea-Mbal_3863_OG2223
Bacteria-UP_b000033514_1564_OG2223
Bacteria-UP_b000033632_3364_OG2223
Bacteria-UP_b000000270_2999_OG2223
Bacteria-UP_b000007275_3588_OG2223
Bacteria-UP_b000001316_1781_OG2223
Bacteria-UP_b000003947_4346_OG2223
Bacteria-UP_b000000671_4061_OG2223
Bacteria-UP_b000004033_3417_OG2223
Other_Holomycota-Patl_g4120_OG2223
Bacteria-UP_b000005952_3126_OG2223
Bacteria-UP_b000006242_1167_OG2223
Bacteria-UP_b000003560_558_OG2223
Bacteria-UP_b000005801_7214_OG2223
Bacteria-UP_b000013026_1097_OG2223
Bacteria-UP_b000000447_2216_OG2223
Osmo_Fungi-Gpro_687_OG2223
Osmo_Fungi-Gpro_8936_OG2223
Other_SAR-Pbra_2948_OG2223
Osmo_Fungi-Gpro_9464_OG2223
Osmo_Fungi-Gpro_9580_OG2223
Other_Holomycota-Rall_5785_OG2223
Other_Holomycota-Patl_g3345_OG2223
0.3

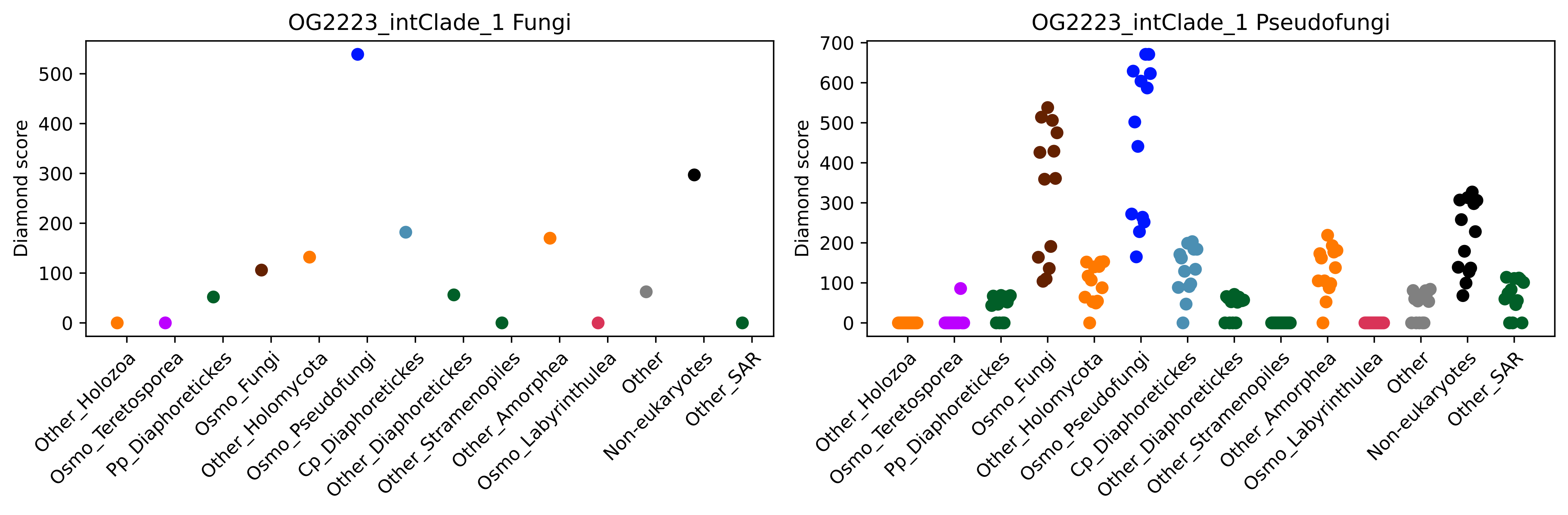
OG2223_intClade_1 Fungi
OG2223_intClade_1 Pseudofungi
Diamond score
0
100
200
300
400
500
600
700
Other_Holozoa
Osmo_Teretosporea
Pp_Diaphoretickes
Osmo_Fungi
Other_Holomycota
Osmo_Pseudofungi
Cp_Diaphoretickes
Other_Diaphoretickes
Other_Stramenopiles
Other_Amorphea
Osmo_Labyrinthulea
Other
Non-eukaryotes
Other_SAR

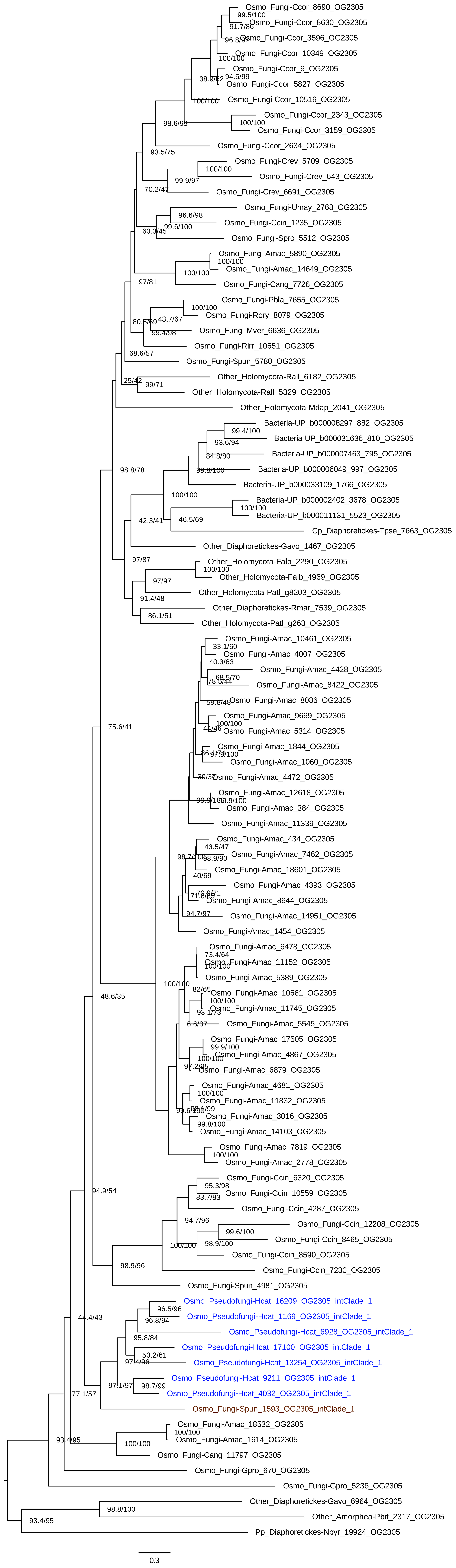

Osmo_Fungi-Ccor_8690_OG2305
Osmo_Fungi-Ccor_8630_OG2305
Osmo_Fungi-Ccor_3596_OG2305
Osmo_Fungi-Ccor_10349_OG2305
Osmo_Fungi-Ccor_9_OG2305
Osmo_Fungi-Ccor_5827_OG2305
Osmo_Fungi-Ccor_10516_OG2305
Osmo_Fungi-Ccor_2343_OG2305
Osmo_Fungi-Ccor_3159_OG2305
Osmo_Fungi-Ccor_2634_OG2305
Osmo_Fungi-Crev_5709_OG2305
Osmo_Fungi-Crev_643_OG2305
Osmo_Fungi-Crev_6691_OG2305
Osmo_Fungi-Umay_2768_OG2305
Osmo_Fungi-Ccin_1235_OG2305
Osmo_Fungi-Spro_5512_OG2305
Osmo_Fungi-Amac_5890_OG2305
Osmo_Fungi-Amac_14649_OG2305
Osmo_Fungi-Cang_7726_OG2305
Osmo_Fungi-Pbla_7655_OG2305
Osmo_Fungi-Rory_8079_OG2305
Osmo_Fungi-Mver_6636_OG2305
Osmo_Fungi-Rirr_10651_OG2305
Osmo_Fungi-Spun_5780_OG2305
Other_Holomycota-Rall_6182_OG2305
Other_Holomycota-Rall_5329_OG2305
Other_Holomycota-Mdap_2041_OG2305
Bacteria-UP_b000008297_882_OG2305
Bacteria-UP_b000031636_810_OG2305
Bacteria-UP_b000007463_795_OG2305
Bacteria-UP_b000006049_997_OG2305
Bacteria-UP_b000033109_1766_OG2305
Bacteria-UP_b000002402_3678_OG2305
Bacteria-UP_b000011131_5523_OG2305
Cp_Diaphoretickes-Tpse_7663_OG2305
Other_Diaphoretickes-Gavo_1467_OG2305
Other_Holomycota-Falb_2290_OG2305
Other_Holomycota-Falb_4969_OG2305
Other_Holomycota-Patl_g8203_OG2305
Other_Diaphoretickes-Rmar_7539_OG2305
Other_Holomycota-Patl_g263_OG2305
Osmo_Fungi-Amac_10461_OG2305
Osmo_Fungi-Amac_4007_OG2305
Osmo_Fungi-Amac_4428_OG2305
Osmo_Fungi-Amac_8422_OG2305
Osmo_Fungi-Amac_8086_OG2305
Osmo_Fungi-Amac_9699_OG2305
Osmo_Fungi-Amac_5314_OG2305
Osmo_Fungi-Amac_1844_OG2305
Osmo_Fungi-Amac_1060_OG2305
Osmo_Fungi-Amac_4472_OG2305
Osmo_Fungi-Amac_12618_OG2305
Osmo_Fungi-Amac_384_OG2305
Osmo_Fungi-Amac_11339_OG2305
Osmo_Fungi-Amac_434_OG2305
Osmo_Fungi-Amac_7462_OG2305
Osmo_Fungi-Amac_18601_OG2305
Osmo_Fungi-Amac_4393_OG2305
Osmo_Fungi-Amac_8644_OG2305
Osmo_Fungi-Amac_14951_OG2305
Osmo_Fungi-Amac_1454_OG2305
Osmo_Fungi-Amac_6478_OG2305
Osmo_Fungi-Amac_11152_OG2305
Osmo_Fungi-Amac_5389_OG2305
Osmo_Fungi-Amac_10661_OG2305
Osmo_Fungi-Amac_11745_OG2305
Osmo_Fungi-Amac_5545_OG2305
Osmo_Fungi-Amac_17505_OG2305
Osmo_Fungi-Amac_4867_OG2305
Osmo_Fungi-Amac_6879_OG2305
Osmo_Fungi-Amac_4681_OG2305
Osmo_Fungi-Amac_11832_OG2305
Osmo_Fungi-Amac_3016_OG2305
Osmo_Fungi-Amac_14103_OG2305
Osmo_Fungi-Amac_7819_OG2305
Osmo_Fungi-Amac_2778_OG2305
Osmo_Fungi-Ccin_6320_OG2305
Osmo_Fungi-Ccin_10559_OG2305
Osmo_Fungi-Ccin_4287_OG2305
Osmo_Fungi-Ccin_12208_OG2305
Osmo_Fungi-Ccin_8465_OG2305
Osmo_Fungi-Ccin_8590_OG2305
Osmo_Fungi-Ccin_7230_OG2305
Osmo_Fungi-Spun_4981_OG2305
Osmo_Pseudofungi-Hcat_16209_OG2305_intClade_1
Osmo_Pseudofungi-Hcat_1169_OG2305_intClade_1
Osmo_Pseudofungi-Hcat_6928_OG2305_intClade_1
Osmo_Pseudofungi-Hcat_17100_OG2305_intClade_1
Osmo_Pseudofungi-Hcat_13254_OG2305_intClade_1
Osmo_Pseudofungi-Hcat_9211_OG2305_intClade_1
Osmo_Pseudofungi-Hcat_4032_OG2305_intClade_1
Osmo_Fungi-Spun_1593_OG2305_intClade_1
Osmo_Fungi-Amac_18532_OG2305
Osmo_Fungi-Amac_1614_OG2305
Osmo_Fungi-Cang_11797_OG2305
Osmo_Fungi-Gpro_670_OG2305
Osmo_Fungi-Gpro_5236_OG2305
Other_Diaphoretickes-Gavo_6964_OG2305
Other_Amorphea-Pbif_2317_OG2305
Pp_Diaphoretickes-Npyr_19924_OG2305
0.3

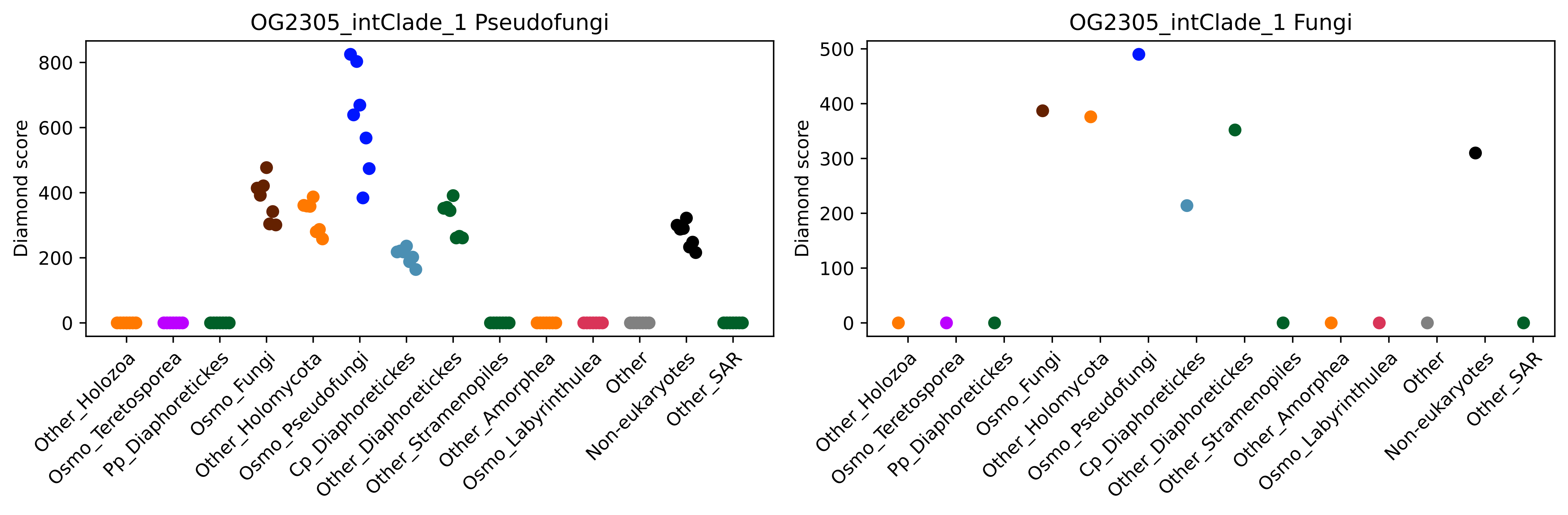
OG2305_intClade_1 Pseudofungi
Diamond score
0
200
400
600
800
Other_Holozoa
Osmo_Teretosporea
Pp_Diaphoretickes
Osmo_Fungi
Other_Holomycota
Osmo_Pseudofungi
Cp_Diaphoretickes
Other_Diaphoretickes
Other_Stramenopiles
Other_Amorphea
Osmo_Labyrinthulea
Other
Non-eukaryotes
Other_SAR
OG2305_intClade_1 Fungi
Diamond score
0
100
200
300
400
500
Other_Holozoa
Osmo_Teretosporea
Pp_Diaphoretickes
Osmo_Fungi
Other_Holomycota
Osmo_Pseudofungi
Cp_Diaphoretickes
Other_Diaphoretickes
Other_Stramenopiles
Other_Amorphea
Osmo_Labyrinthulea
Other
Non-eukaryotes
Other_SAR

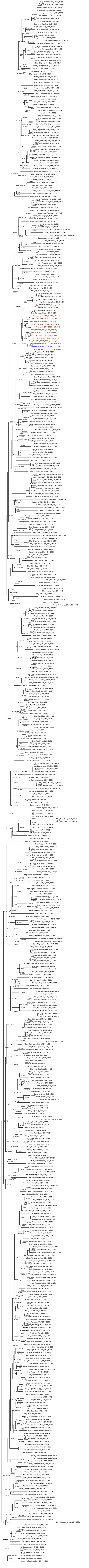

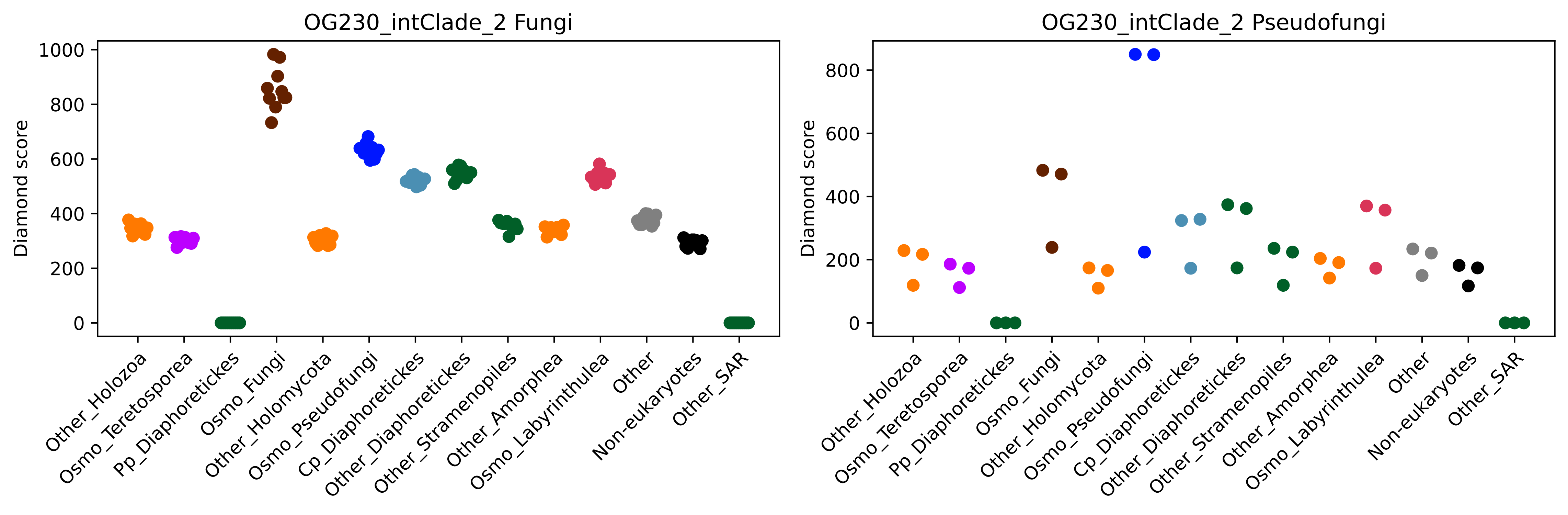
OG230_intClade_2 Fungi
Diamond score
0
200
400
600
800
1000
Other_Holozoa
Osmo_Teretosporea
Pp_Diaphoretickes
Osmo_Fungi
Other_Holomycota
Osmo_Pseudofungi
Cp_Diaphoretickes
Other_Diaphoretickes
Other_Stramenopiles
Other_Amorphea
Osmo_Labyrinthulea
Other
Non-eukaryotes
Other_SAR
OG230_intClade_2 Pseudofungi
Diamond score
0
200
400
600
800
Other_Holozoa
Osmo_Teretosporea
Pp_Diaphoretickes
Osmo_Fungi
Other_Holomycota
Osmo_Pseudofungi
Cp_Diaphoretickes
Other_Diaphoretickes
Other_Stramenopiles
Other_Amorphea
Osmo_Labyrinthulea
Other
Non-eukaryotes
Other_SAR

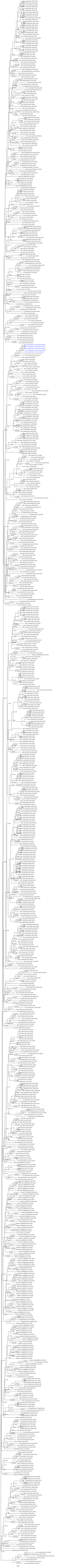

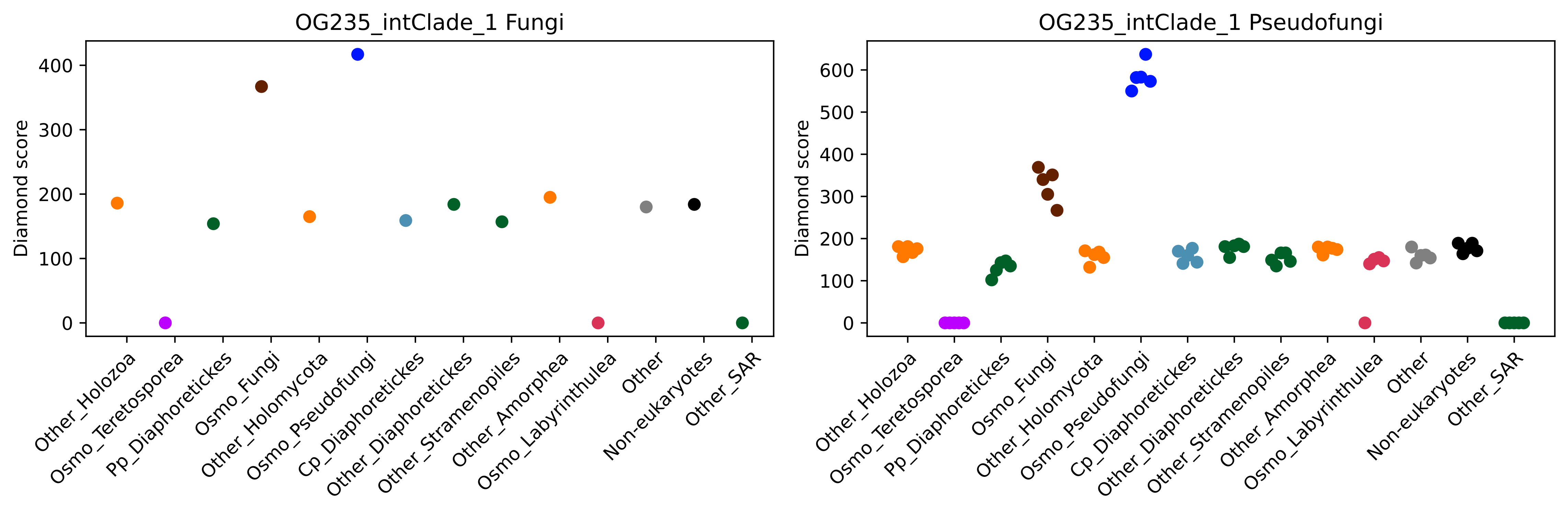

OG235_intClade_1 Fungi
OG235_intClade_1 Pseudofungi
Diamond score
0
100
200
300
400
500
600
Other_Holozoa
Osmo_Teretosporea
Pp_Diaphoretickes
Osmo_Fungi
Other_Holomycota
Osmo_Pseudofungi
Cp_Diaphoretickes
Other_Diaphoretickes
Other_Stramenopiles
Other_Amorphea
Osmo_Labyrinthulea
Other
Non-eukaryotes
Other_SAR

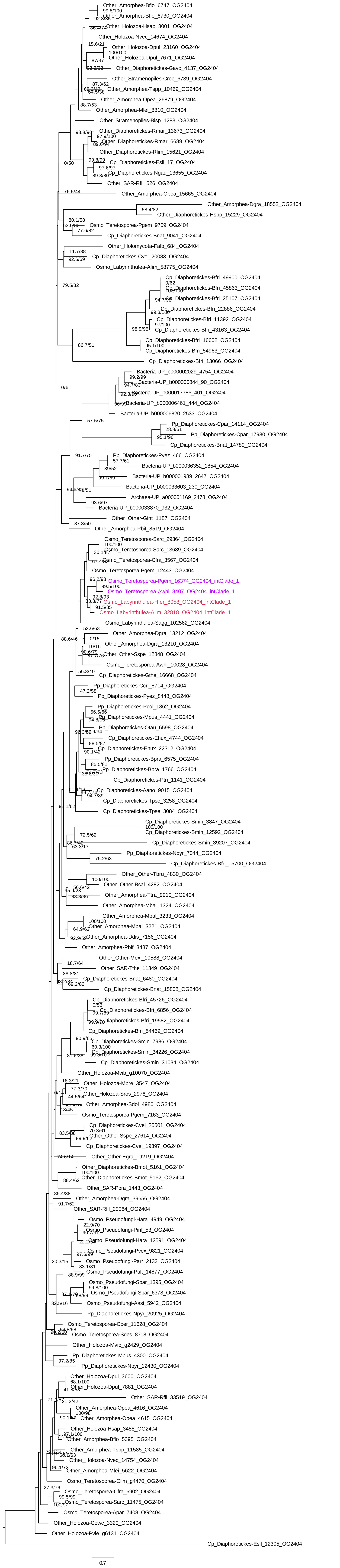
Other_Amorphea-Bflo_6747_OG2404
Other_Amorphea-Bflo_6730_OG2404
Other_Holozoa-Hsap_8001_OG2404
Other_Holozoa-Nvec_14674_OG2404
Other_Holozoa-Dpul_23160_OG2404
Other_Holozoa-Dpul_7671_OG2404
Other_Diaphoretickes-Gavo_4137_OG2404
Other_Stramenopiles-Croe_6739_OG2404
Other_Amorphea-Tspp_10469_OG2404
Other_Amorphea-Opea_26879_OG2404
Other_Amorphea-Mlei_8810_OG2404
Other_Stramenopiles-Bisp_1283_OG2404
Other_Diaphoretickes-Rmar_13673_OG2404
Other_Diaphoretickes-Rmar_6689_OG2404
Other_Diaphoretickes-Rlim_15621_OG2404
Cp_Diaphoretickes-Esil_17_OG2404
Cp_Diaphoretickes-Ngad_13655_OG2404
Other_SAR-Rfil_526_OG2404
Other_Amorphea-Opea_15665_OG2404
Other_Amorphea-Dgra_18552_OG2404
Other_Diaphoretickes-Hspp_15229_OG2404
Osmo_Teretosporea-Pgem_9709_OG2404
Cp_Diaphoretickes-Bnat_9041_OG2404
Other_Holomycota-Falb_684_OG2404
Cp_Diaphoretickes-Cvel_20083_OG2404
Osmo_Labyrinthulea-Alim_58775_OG2404
Cp_Diaphoretickes-Bfri_49900_OG2404
Cp_Diaphoretickes-Bfri_45863_OG2404
Cp_Diaphoretickes-Bfri_25107_OG2404
Cp_Diaphoretickes-Bfri_22886_OG2404
Cp_Diaphoretickes-Bfri_11392_OG2404
Cp_Diaphoretickes-Bfri_43163_OG2404
Cp_Diaphoretickes-Bfri_16602_OG2404
Cp_Diaphoretickes-Bfri_54963_OG2404
Cp_Diaphoretickes-Bfri_13066_OG2404
Bacteria-UP_b000002029_4754_OG2404
Bacteria-UP_b000000844_90_OG2404
Bacteria-UP_b000017786_401_OG2404
Bacteria-UP_b000006461_444_OG2404
Bacteria-UP_b000006820_2533_OG2404
Pp_Diaphoretickes-Cpar_14114_OG2404
Pp_Diaphoretickes-Cpar_17930_OG2404
Cp_Diaphoretickes-Bnat_14789_OG2404
Pp_Diaphoretickes-Pyez_466_OG2404
Bacteria-UP_b000036352_1854_OG2404
Bacteria-UP_b000001989_2647_OG2404
Bacteria-UP_b000033603_230_OG2404
Archaea-UP_a000001169_2478_OG2404
Bacteria-UP_b000033870_932_OG2404
Other_Other-Gint_1187_OG2404
Other_Amorphea-Pbif_8519_OG2404
Osmo_Teretosporea-Sarc_29364_OG2404
Osmo_Teretosporea-Sarc_13639_OG2404
Osmo_Teretosporea-Cfra_3567_OG2404
Osmo_Teretosporea-Pgem_12443_OG2404
Osmo_Teretosporea-Pgem_16374_OG2404_intClade_1
Osmo_Teretosporea-Awhi_8407_OG2404_intClade_1
Osmo_Labyrinthulea-Hfer_8058_OG2404_intClade_1
Osmo_Labyrinthulea-Alim_32818_OG2404_intClade_1
Osmo_Labyrinthulea-Sagg_102562_OG2404
Other_Amorphea-Dgra_13212_OG2404
Other_Amorphea-Dgra_13210_OG2404
Other_Other-Sspe_12848_OG2404
Osmo_Teretosporea-Awhi_10028_OG2404
Cp_Diaphoretickes-Gthe_16668_OG2404
Pp_Diaphoretickes-Ccri_8714_OG2404
Pp_Diaphoretickes-Pyez_8448_OG2404
Pp_Diaphoretickes-Pcol_1862_OG2404
Pp_Diaphoretickes-Mpus_4441_OG2404
Pp_Diaphoretickes-Otau_6598_OG2404
Cp_Diaphoretickes-Ehux_4744_OG2404
Cp_Diaphoretickes-Ehux_22312_OG2404
Pp_Diaphoretickes-Bpra_6575_OG2404
Pp_Diaphoretickes-Bpra_1766_OG2404
Cp_Diaphoretickes-Ptri_1141_OG2404
Cp_Diaphoretickes-Aano_9015_OG2404
Cp_Diaphoretickes-Tpse_3258_OG2404
Cp_Diaphoretickes-Tpse_3084_OG2404
Cp_Diaphoretickes-Smin_3847_OG2404
Cp_Diaphoretickes-Smin_12592_OG2404
Cp_Diaphoretickes-Smin_39207_OG2404
Pp_Diaphoretickes-Npyr_7044_OG2404
Cp_Diaphoretickes-Bfri_15700_OG2404
Other_Other-Tbru_4830_OG2404
Other_Other-Bsal_4282_OG2404
Other_Amorphea-Ttra_9910_OG2404
Other_Amorphea-Mbal_1324_OG2404
Other_Amorphea-Mbal_3233_OG2404
Other_Amorphea-Mbal_3221_OG2404
Other_Amorphea-Ddis_7156_OG2404
Other_Amorphea-Pbif_3487_OG2404
Other_Other-Mexi_10588_OG2404
Other_SAR-Tthe_11349_OG2404
Cp_Diaphoretickes-Bnat_6480_OG2404
Cp_Diaphoretickes-Bnat_15808_OG2404
Cp_Diaphoretickes-Bfri_45726_OG2404
Cp_Diaphoretickes-Bfri_6856_OG2404
Cp_Diaphoretickes-Bfri_19582_OG2404
Cp_Diaphoretickes-Bfri_54469_OG2404
Cp_Diaphoretickes-Smin_7986_OG2404
Cp_Diaphoretickes-Smin_34226_OG2404
Cp_Diaphoretickes-Smin_31034_OG2404
Other_Holozoa-Mvib_g10070_OG2404
Other_Holozoa-Mbre_3547_OG2404
Other_Holozoa-Sros_2976_OG2404
Other_Amorphea-Sdol_4980_OG2404
Osmo_Teretosporea-Pgem_7163_OG2404
Cp_Diaphoretickes-Cvel_25501_OG2404
Other_Other-Sspe_27614_OG2404
Cp_Diaphoretickes-Cvel_19397_OG2404
Other_Other-Egra_19219_OG2404
Other_Diaphoretickes-Bmot_5161_OG2404
Other_Diaphoretickes-Bmot_5162_OG2404
Other_SAR-Pbra_1443_OG2404
Other_Amorphea-Dgra_39656_OG2404
Other_SAR-Rfil_29064_OG2404
Osmo_Pseudofungi-Hara_4949_OG2404
Osmo_Pseudofungi-Pinf_53_OG2404
Osmo_Pseudofungi-Hara_12591_OG2404
Osmo_Pseudofungi-Pvex_9821_OG2404
Osmo_Pseudofungi-Parr_2133_OG2404
Osmo_Pseudofungi-Pult_14877_OG2404
Osmo_Pseudofungi-Spar_1395_OG2404
Osmo_Pseudofungi-Spar_6378_OG2404
Osmo_Pseudofungi-Aast_5942_OG2404
Pp_Diaphoretickes-Npyr_20925_OG2404
Osmo_Teretosporea-Cper_11628_OG2404
Osmo_Teretosporea-Sdes_8718_OG2404
Other_Holozoa-Mvib_g2429_OG2404
Pp_Diaphoretickes-Mpus_4300_OG2404
Pp_Diaphoretickes-Npyr_12430_OG2404
Other_Holozoa-Dpul_3600_OG2404
Other_Holozoa-Dpul_7881_OG2404
Other_SAR-Rfil_33519_OG2404
Other_Amorphea-Opea_4616_OG2404
Other_Amorphea-Opea_4615_OG2404
Other_Holozoa-Hsap_3458_OG2404
Other_Amorphea-Bflo_5395_OG2404
Other_Amorphea-Tspp_11585_OG2404
Other_Holozoa-Nvec_14754_OG2404
Other_Amorphea-Mlei_5622_OG2404
Osmo_Teretosporea-Clim_g4470_OG2404
Osmo_Teretosporea-Cfra_5902_OG2404
Osmo_Teretosporea-Sarc_11475_OG2404
Osmo_Teretosporea-Apar_7408_OG2404
Other_Holozoa-Cowc_3320_OG2404
Other_Holozoa-Pvie_g6131_OG2404
Cp_Diaphoretickes-Esil_12305_OG2404
0.7

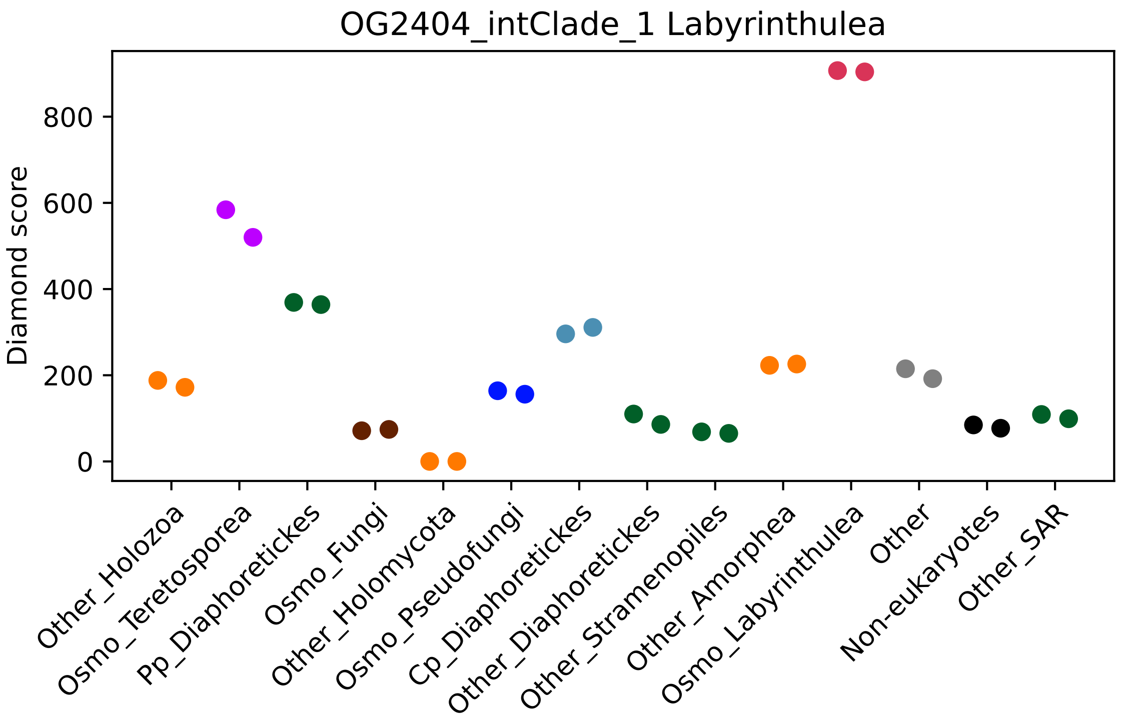

OG2404_intClade_1 Labyrinthulea
Diamond score
0
200
400
600
800
Other_Holozoa
Osmo_Teretosporea
Pp_Diaphoretickes
Osmo_Fungi
Other_Holomycota
Osmo_Pseudofungi
Cp_Diaphoretickes
Other_Diaphoretickes
Other_Stramenopiles
Other_Amorphea
Osmo_Labyrinthulea
Other
Non-eukaryotes
Other_SAR

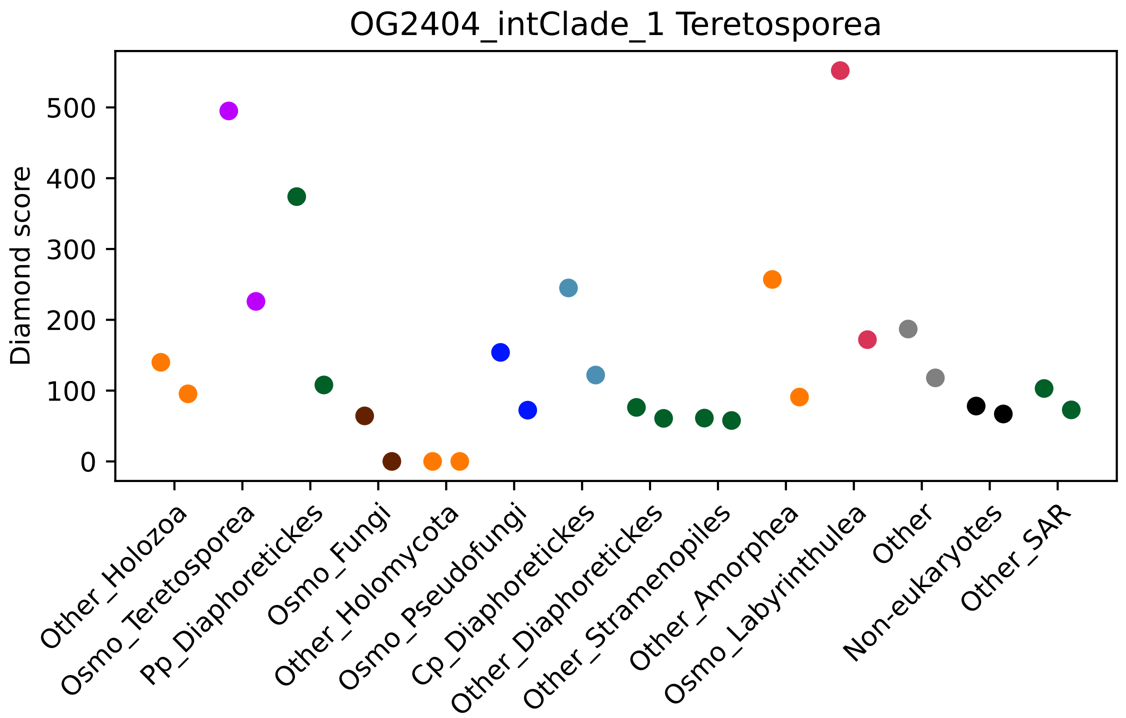

OG2404_intClade_1 Teretosporea
Diamond score
0
100
200
300
400
500
Other_Holozoa
Osmo_Teretosporea
Pp_Diaphoretickes
Osmo_Fungi
Other_Holomycota
Osmo_Pseudofungi
Cp_Diaphoretickes
Other_Diaphoretickes
Other_Stramenopiles
Other_Amorphea
Osmo_Labyrinthulea
Other
Non-eukaryotes
Other_SAR

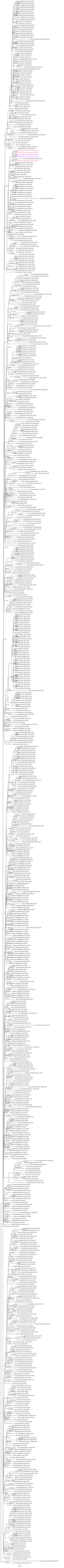

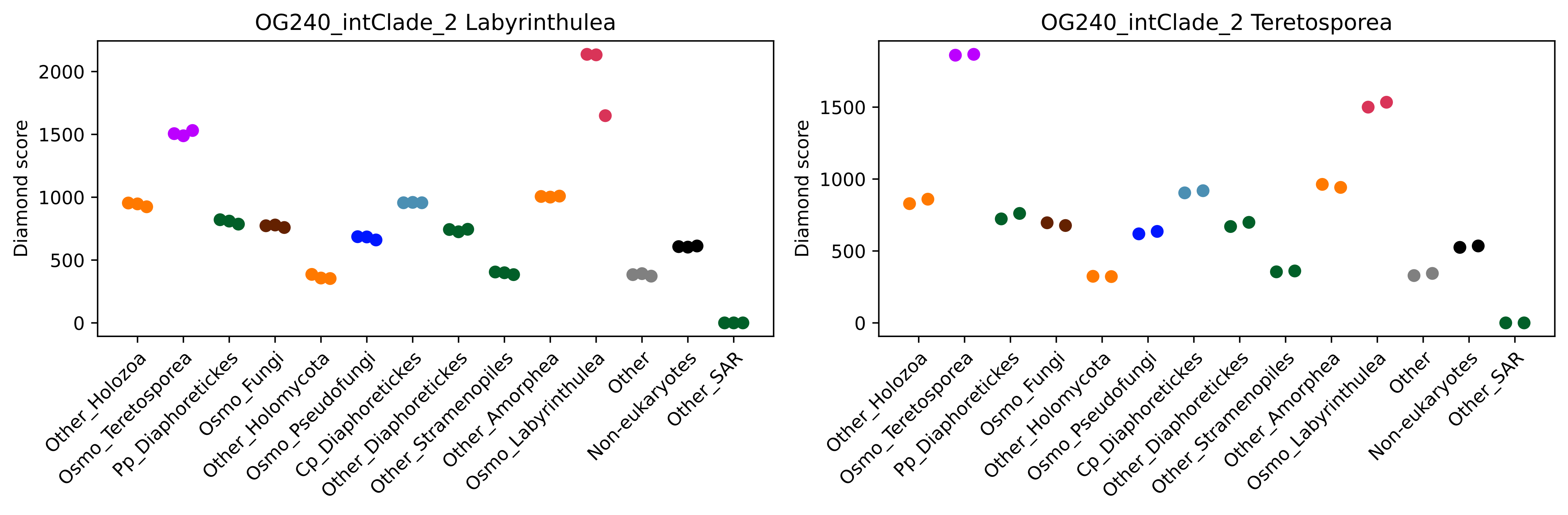
OG240_intClade_2 Labyrinthulea
OG240_intClade_2 Teretosporea
Diamond score
0
500
1000
1500
2000
Other_Holozoa
Osmo_Teretosporea
Pp_Diaphoretickes
Osmo_Fungi
Other_Holomycota
Osmo_Pseudofungi
Cp_Diaphoretickes
Other_Diaphoretickes
Other_Stramenopiles
Other_Amorphea
Osmo_Labyrinthulea
Other
Non-eukaryotes
Other_SAR

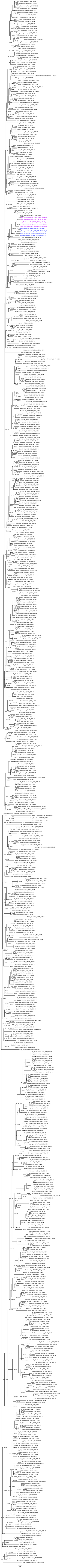

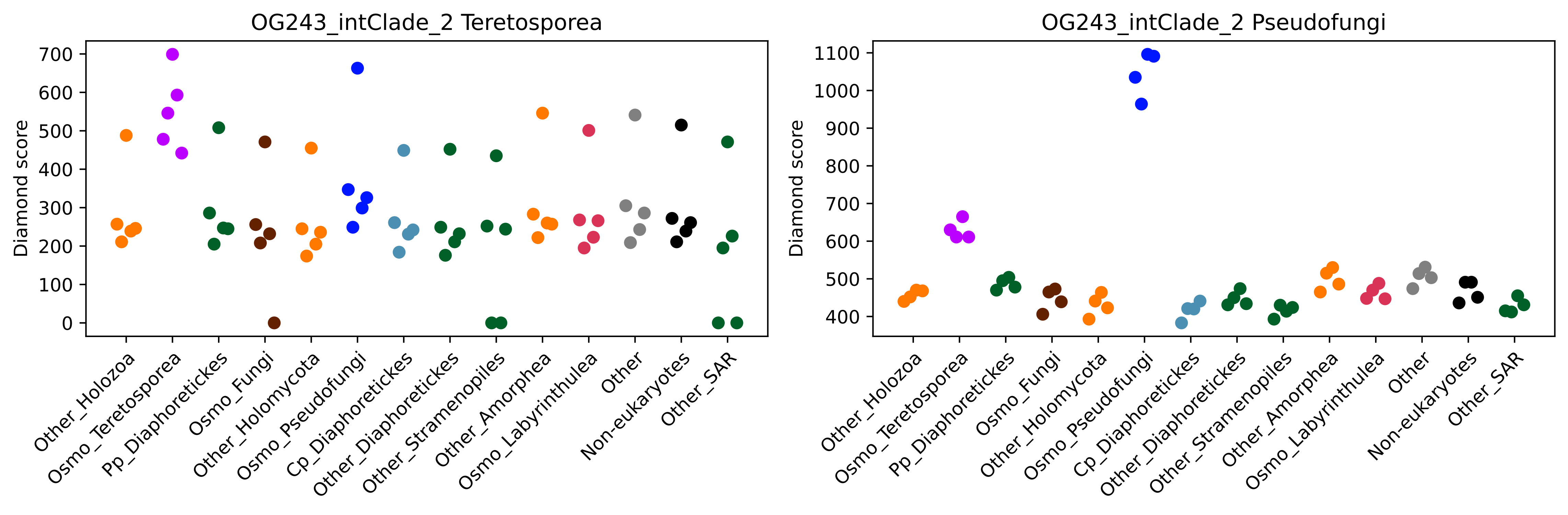
OG243_intClade_2 Teretosporea
OG243_intClade_2 Pseudofungi
Diamond score
Other_Holozoa
Osmo_Teretosporea
Pp_Diaphoretickes
Osmo_Fungi
Other_Holomycota
Osmo_Pseudofungi
Cp_Diaphoretickes
Other_Diaphoretickes
Other_Stramenopiles
Other_Amorphea
Osmo_Labyrinthulea
Other
Non-eukaryotes
Other_SAR

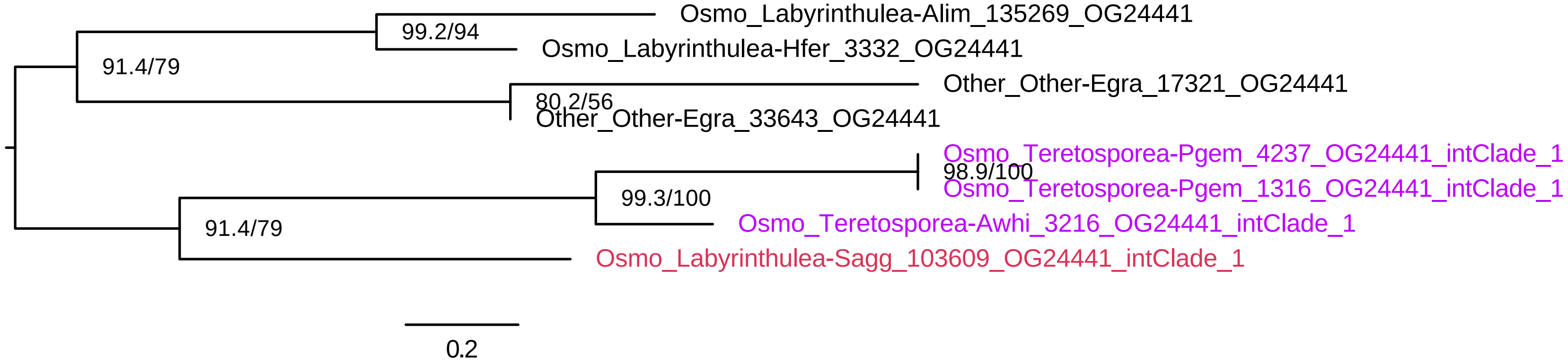
Osmo_Labyrinthulea-Alim_135269_OG24441
99.2/94
Osmo_Labyrinthulea-Hfer_3332_OG24441
91.4/79
Other_Other-Egra_17321_OG24441
80.2/56
Other_Other-Egra_33643_OG24441
Osmo_Teretosporea-Pgem_4237_OG24441_intClade_1
98.9/100
Osmo_Teretosporea-Pgem_1316_OG24441_intClade_1
99.3/100
Osmo_Teretosporea-Awhi_3216_OG24441_intClade_1
91.4/79
Osmo_Labyrinthulea-Sagg_103609_OG24441_intClade_1
0.2

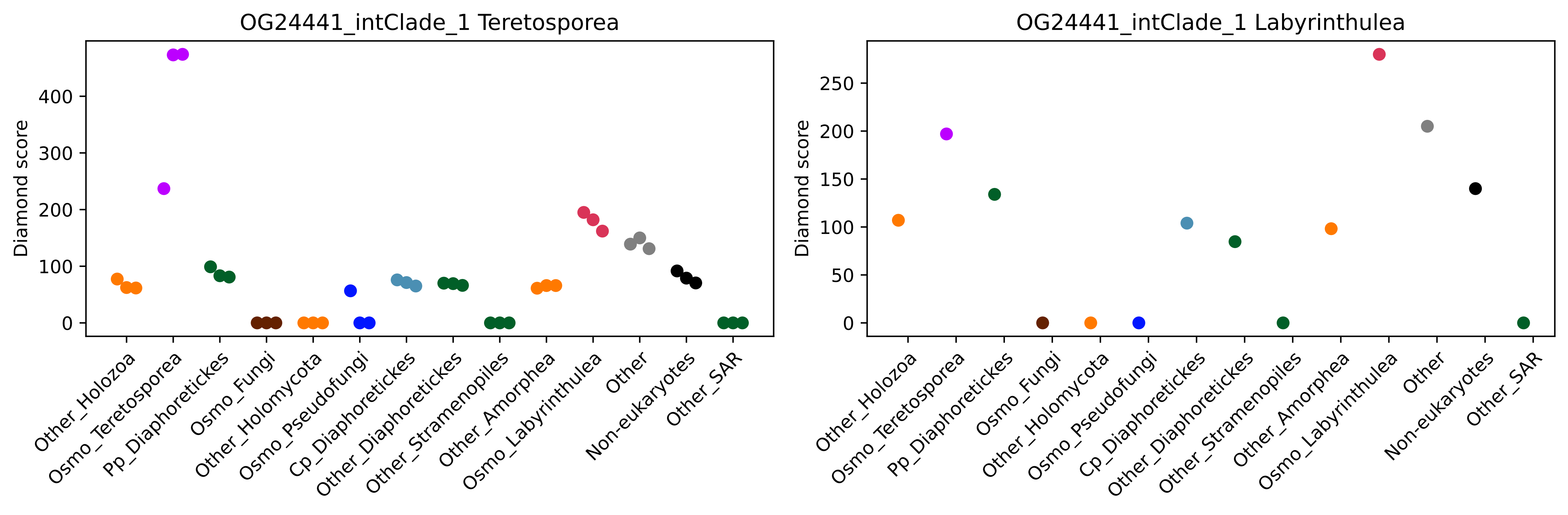

OG24441_intClade_1 Teretosporea
Diamond score
0
100
200
300
400
Other_Holozoa
Osmo_Teretosporea
Pp_Diaphoretickes
Osmo_Fungi
Other_Holomycota
Osmo_Pseudofungi
Cp_Diaphoretickes
Other_Diaphoretickes
Other_Stramenopiles
Other_Amorphea
Osmo_Labyrinthulea
Other
Non-eukaryotes
Other_SAR
OG24441_intClade_1 Labyrinthulea
Diamond score
0
50
100
150
200
250
Other_Holozoa
Osmo_Teretosporea
Pp_Diaphoretickes
Osmo_Fungi
Other_Holomycota
Osmo_Pseudofungi
Cp_Diaphoretickes
Other_Diaphoretickes
Other_Stramenopiles
Other_Amorphea
Osmo_Labyrinthulea
Other
Non-eukaryotes
Other_SAR

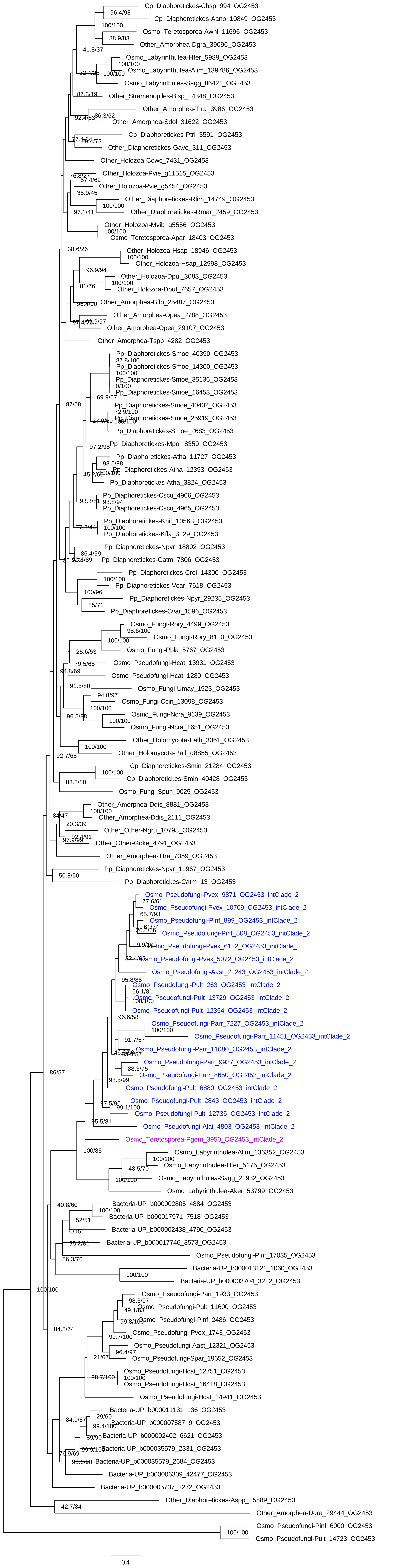

Cp_Diaphoretickes-Chsp_994_OG2453
96.4/98
Cp_Diaphoretickes-Aano_10849_OG2453
100/100
Osmo_Teretosporea-Awhi_11696_OG2453
88.9/83
Other_Amorphea-Dgra_39096_OG2453
41.8/37
Osmo_Labyrinthulea-Hfer_5989_OG2453
100/100
32.4/25
100/100
Osmo_Labyrinthulea-Alim_139786_OG2453
Osmo_Labyrinthulea-Sagg_86421_OG2453
87.3/19
Other_Stramenopiles-Bisp_14348_OG2453
Other_Amorphea-Ttra_3986_OG2453
86.3/62
92.4/63
Other_Amorphea-Sdol_31622_OG2453
Cp_Diaphoretickes-Ptri_3591_OG2453
77.4/34
89.4/73
Other_Diaphoretickes-Gavo_311_OG2453
Other_Holozoa-Cowc_7431_OG2453
76.8/27
Other_Holozoa-Pvie_g11515_OG2453
57.4/62
Other_Holozoa-Pvie_g5454_OG2453
35.9/45
Other_Diaphoretickes-Rlim_14749_OG2453
100/100
97.1/41
Other_Diaphoretickes-Rmar_2459_OG2453
Other_Holozoa-Mvib_g5556_OG2453
100/100
Osmo_Teretosporea-Apar_18403_OG2453
38.6/26
Other_Holozoa-Hsap_18946_OG2453
100/100
Other_Holozoa-Hsap_12998_OG2453
96.9/94
Other_Holozoa-Dpul_3083_OG2453
100/100
81/76
Other_Holozoa-Dpul_7657_OG2453
96.4/90
Other_Amorphea-Bflo_25487_OG2453
Other_Amorphea-Opea_2788_OG2453
97.4/78
99.9/97
Other_Amorphea-Opea_29107_OG2453
Other_Amorphea-Tspp_4282_OG2453
Pp_Diaphoretickes-Smoe_40390_OG2453
87.8/100
Pp_Diaphoretickes-Smoe_14300_OG2453
100/100
Pp_Diaphoretickes-Smoe_35136_OG2453
0/100
Pp_Diaphoretickes-Smoe_16453_OG2453
87/68
69.9/67
Pp_Diaphoretickes-Smoe_40402_OG2453
72.9/100
27.9/50
Pp_Diaphoretickes-Smoe_25919_OG2453
100/100
Pp_Diaphoretickes-Smoe_2683_OG2453
97.2/98
Pp_Diaphoretickes-Mpol_8359_OG2453
Pp_Diaphoretickes-Atha_11727_OG2453
98.5/98
100/100
Pp_Diaphoretickes-Atha_12393_OG2453
45.2/66
Pp_Diaphoretickes-Atha_3824_OG2453
Pp_Diaphoretickes-Cscu_4966_OG2453
93.3/81
93.8/94
Pp_Diaphoretickes-Cscu_4965_OG2453
Pp_Diaphoretickes-Knit_10563_OG2453
77.2/44
100/100
Pp_Diaphoretickes-Kfla_3129_OG2453
86.4/59
Pp_Diaphoretickes-Npyr_18892_OG2453
85.2/74
30.6/89
Pp_Diaphoretickes-Catm_7806_OG2453
Pp_Diaphoretickes-Crei_14300_OG2453
100/100
Pp_Diaphoretickes-Vcar_7618_OG2453
100/96
Pp_Diaphoretickes-Npyr_29235_OG2453
85/71
Pp_Diaphoretickes-Cvar_1596_OG2453
Osmo_Fungi-Rory_4499_OG2453
98.6/100
Osmo_Fungi-Rory_8110_OG2453
100/100
25.6/53
Osmo_Fungi-Pbla_5767_OG2453
79.5/65
Osmo_Pseudofungi-Hcat_13931_OG2453
94.8/69
Osmo_Pseudofungi-Hcat_1280_OG2453
91.5/80
Osmo_Fungi-Umay_1923_OG2453
94.8/97
Osmo_Fungi-Ccin_13098_OG2453
100/100
96.5/88
Osmo_Fungi-Ncra_9139_OG2453
100/100
Osmo_Fungi-Ncra_1651_OG2453
Other_Holomycota-Falb_3061_OG2453
100/100
92.7/68
Other_Holomycota-Patl_g8855_OG2453
Cp_Diaphoretickes-Smin_21284_OG2453
100/100
Cp_Diaphoretickes-Smin_40428_OG2453
83.5/80
Osmo_Fungi-Spun_9025_OG2453
Other_Amorphea-Ddis_8881_OG2453
100/100
84/47
Other_Amorphea-Ddis_2111_OG2453
20.3/39
Other_Other-Ngru_10798_OG2453
92.4/91
97.8/99
Other_Other-Goke_4791_OG2453
Other_Amorphea-Ttra_7359_OG2453
Pp_Diaphoretickes-Npyr_11967_OG2453
50.8/50
Pp_Diaphoretickes-Catm_13_OG2453
Osmo_Pseudofungi-Pvex_9871_OG2453_intClade_2
77.6/61
Osmo_Pseudofungi-Pvex_10709_OG2453_intClade_2
65.7/93
Osmo_Pseudofungi-Pinf_899_OG2453_intClade_2
61/74
26.6/66
Osmo_Pseudofungi-Pinf_508_OG2453_intClade_2
99.9/100
Osmo_Pseudofungi-Pvex_6122_OG2453_intClade_2
32.4/65
Osmo_Pseudofungi-Pvex_5072_OG2453_intClade_2
Osmo_Pseudofungi-Aast_21243_OG2453_intClade_2
95.8/88
Osmo_Pseudofungi-Pult_263_OG2453_intClade_2
66.1/81
Osmo_Pseudofungi-Pult_13729_OG2453_intClade_2
100/100
Osmo_Pseudofungi-Pult_12354_OG2453_intClade_2
96.6/58
Osmo_Pseudofungi-Parr_7227_OG2453_intClade_2
100/100
Osmo_Pseudofungi-Parr_11451_OG2453_intClade_2
91.7/57
46.6/50
63.4/57
Osmo_Pseudofungi-Parr_11080_OG2453_intClade_2
Osmo_Pseudofungi-Parr_9937_OG2453_intClade_2
88.3/75
Osmo_Pseudofungi-Parr_8650_OG2453_intClade_2
98.5/99
Osmo_Pseudofungi-Pult_6880_OG2453_intClade_2
97.5/96
Osmo_Pseudofungi-Pult_2843_OG2453_intClade_2
99.1/100
Osmo_Pseudofungi-Pult_12735_OG2453_intClade_2
95.5/81
Osmo_Pseudofungi-Alai_4803_OG2453_intClade_2
86/57
Osmo_Teretosporea-Pgem_3950_OG2453_intClade_2
100/85
Osmo_Labyrinthulea-Alim_136352_OG2453
100/100
Osmo_Labyrinthulea-Hfer_5175_OG2453
48.5/70
Osmo_Labyrinthulea-Sagg_21932_OG2453
100/100
Osmo_Labyrinthulea-Aker_53799_OG2453
40.8/60
Bacteria-UP_b000002805_4884_OG2453
100/100
52/51
Bacteria-UP_b000017971_7518_OG2453
0/15
Bacteria-UP_b000002438_4790_OG2453
95.2/81
Bacteria-UP_b000017746_3573_OG2453
86.3/70
Osmo_Pseudofungi-Pinf_17035_OG2453
Bacteria-UP_b000013121_1060_OG2453
100/100
Bacteria-UP_b000003704_3212_OG2453
100/100
Osmo_Pseudofungi-Parr_1933_OG2453
98.3/97
Osmo_Pseudofungi-Pult_11600_OG2453
49.1/63
99.8/100
Osmo_Pseudofungi-Pinf_2486_OG2453
84.5/74
99.7/100
Osmo_Pseudofungi-Pvex_1743_OG2453
Osmo_Pseudofungi-Aast_12321_OG2453
96.4/97
21/67
Osmo_Pseudofungi-Spar_19652_OG2453
Osmo_Pseudofungi-Hcat_12751_OG2453
98.7/100
100/100
Osmo_Pseudofungi-Hcat_16418_OG2453
Osmo_Pseudofungi-Hcat_14941_OG2453
Bacteria-UP_b000011131_136_OG2453
84.9/87
29/60
Bacteria-UP_b000007587_9_OG2453
99.4/100
89/90
Bacteria-UP_b000002402_6621_OG2453
99.9/100
Bacteria-UP_b000035579_2331_OG2453
76.9/69
93.6/90
Bacteria-UP_b000035579_2684_OG2453
Bacteria-UP_b000006309_42477_OG2453
Bacteria-UP_b000005737_2272_OG2453
Other_Diaphoretickes-Aspp_15889_OG2453
42.7/84
Other_Amorphea-Dgra_29444_OG2453
Osmo_Pseudofungi-Pinf_6000_OG2453
100/100
Osmo_Pseudofungi-Pult_14723_OG2453
0.4

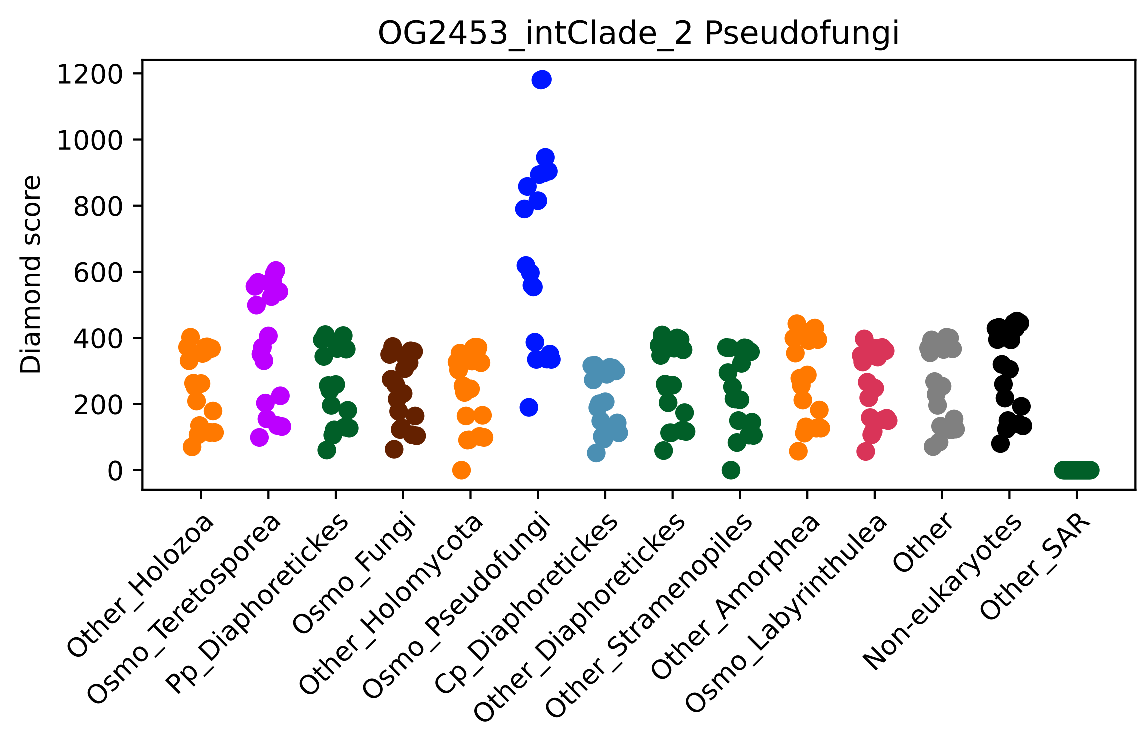
OG2453_intClade_2 Pseudofungi
Diamond score
1200
1000
800
600
400
200
0
Other_Holozoa
Osmo_Teretosporea
Pp_Diaphoretickes
Osmo_Fungi
Other_Holomycota
Osmo_Pseudofungi
Cp_Diaphoretickes
Other_Diaphoretickes
Other_Stramenopiles
Other_Amorphea
Osmo_Labyrinthulea
Other
Non-eukaryotes
Other_SAR

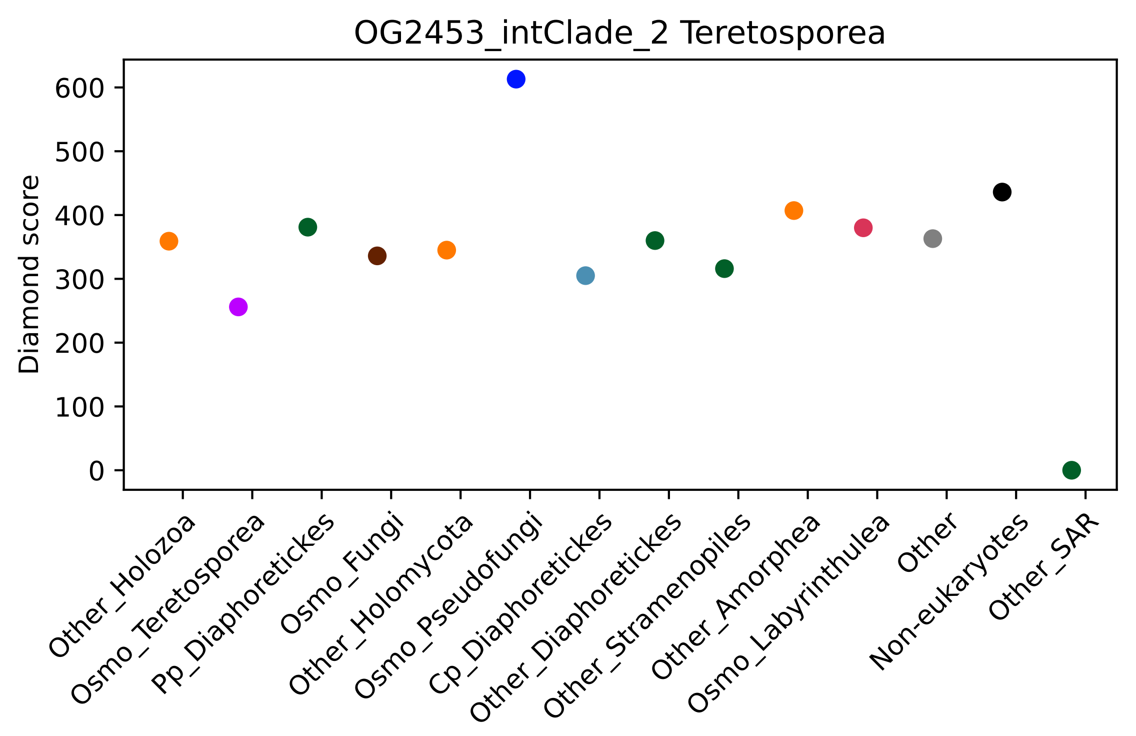
OG2453_intClade_2 Teretosporea
Diamond score
600
500
400
300
200
100
0
Other_Holozoa
Osmo_Teretosporea
Pp_Diaphoretickes
Osmo_Fungi
Other_Holomycota
Osmo_Pseudofungi
Cp_Diaphoretickes
Other_Diaphoretickes
Other_Stramenopiles
Other_Amorphea
Osmo_Labyrinthulea
Other
Non-eukaryotes
Other_SAR

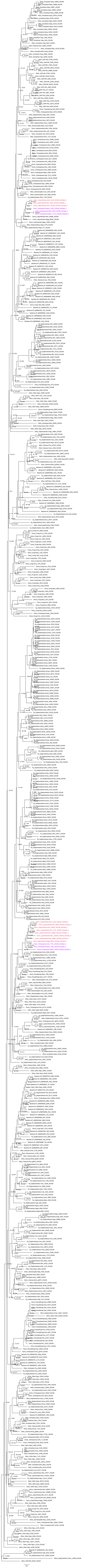

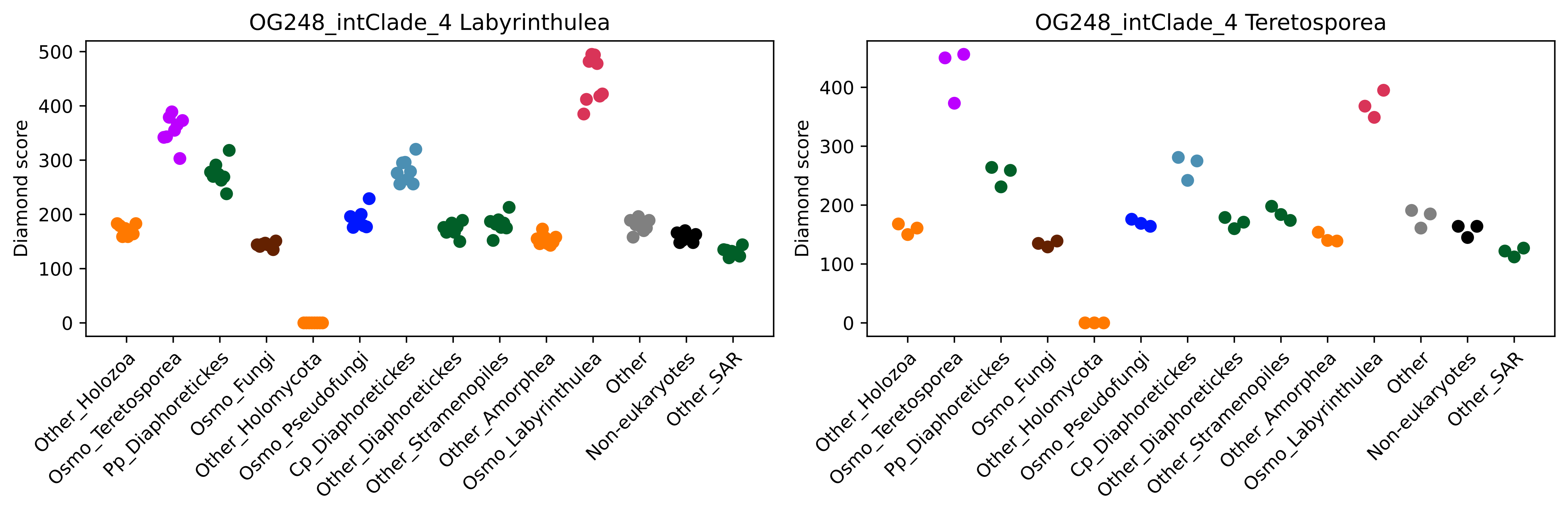

OG248_intClade_4 Labyrinthulea
Diamond score
0
100
200
300
400
500
Other_Holozoa
Osmo_Teretosporea
Pp_Diaphoretickes
Osmo_Fungi
Other_Holomycota
Osmo_Pseudofungi
Cp_Diaphoretickes
Other_Diaphoretickes
Other_Stramenopiles
Other_Amorphea
Osmo_Labyrinthulea
Other
Non-eukaryotes
Other_SAR
OG248_intClade_4 Teretosporea
Diamond score
0
100
200
300
400
Other_Holozoa
Osmo_Teretosporea
Pp_Diaphoretickes
Osmo_Fungi
Other_Holomycota
Osmo_Pseudofungi
Cp_Diaphoretickes
Other_Diaphoretickes
Other_Stramenopiles
Other_Amorphea
Osmo_Labyrinthulea
Other
Non-eukaryotes
Other_SAR

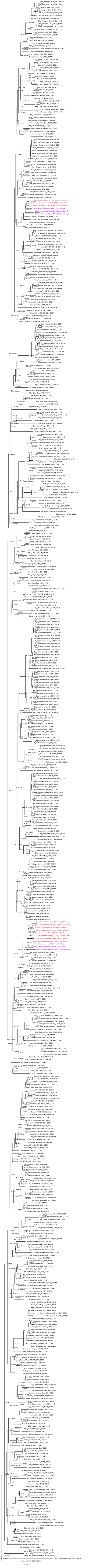

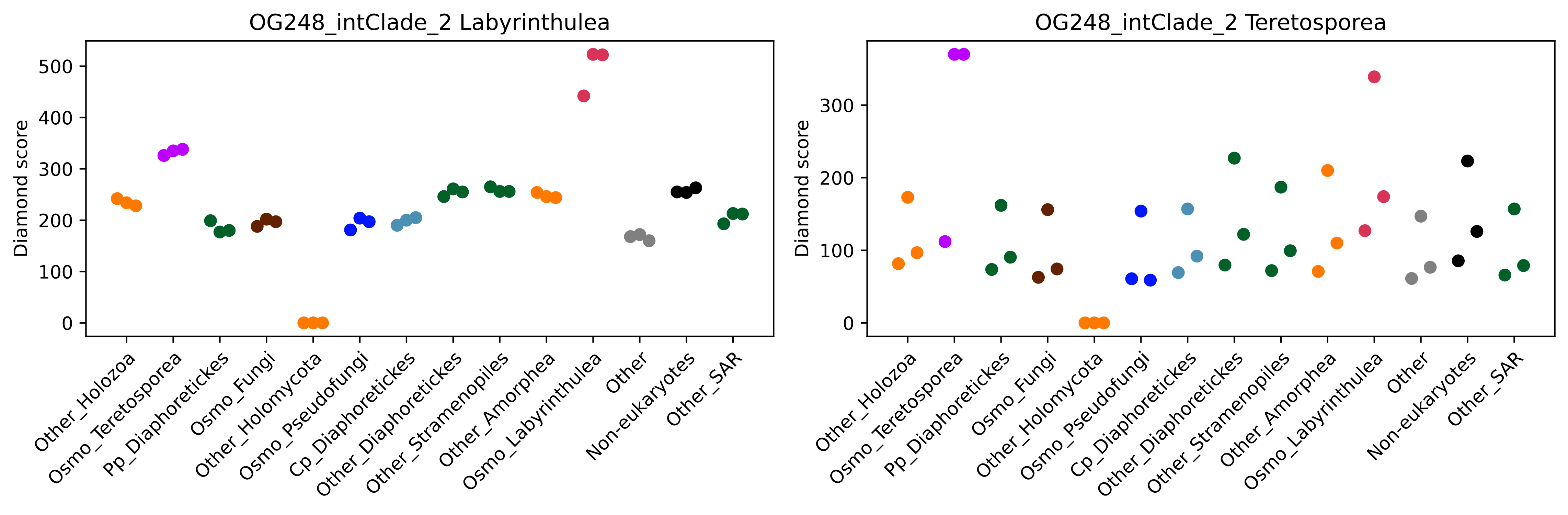
OG248_intClade_2 Labyrinthulea
OG248_intClade_2 Teretosporea
Diamond score
0
100
200
300
400
500
Other_Holozoa
Osmo_Teretosporea
Pp_Diaphoretickes
Osmo_Fungi
Other_Holomycota
Osmo_Pseudofungi
Cp_Diaphoretickes
Other_Diaphoretickes
Other_Stramenopiles
Other_Amorphea
Osmo_Labyrinthulea
Other
Non-eukaryotes
Other_SAR

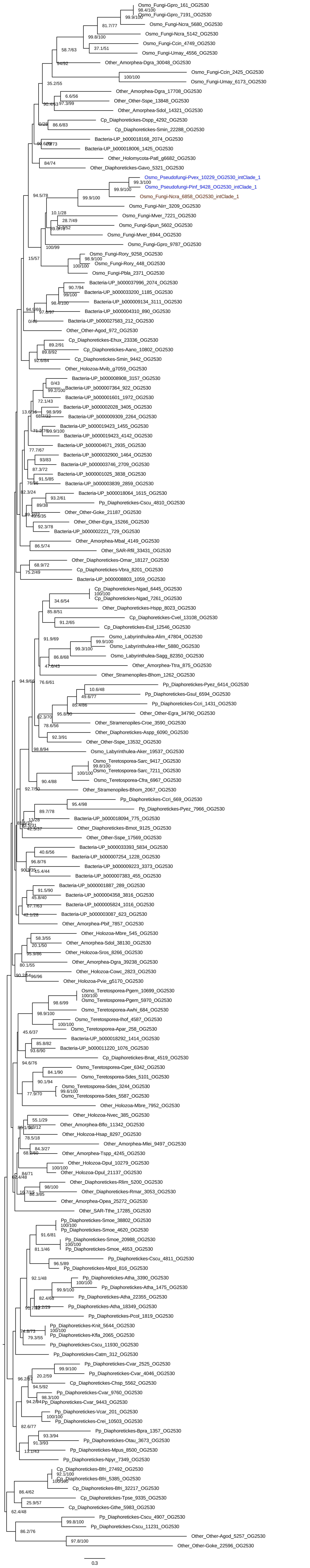
Osmo_Fungi-Gpro_161_OG2530
Osmo_Fungi-Gpro_7191_OG2530
Osmo_Fungi-Ncra_5680_OG2530
Osmo_Fungi-Ncra_5142_OG2530
Osmo_Fungi-Ccin_4749_OG2530
Osmo_Fungi-Umay_4556_OG2530
Other_Amorphea-Dgra_30048_OG2530
Osmo_Fungi-Ccin_2425_OG2530
Osmo_Fungi-Umay_6173_OG2530
Other_Amorphea-Dgra_17708_OG2530
Other_Other-Sspe_13848_OG2530
Other_Amorphea-Sdol_14321_OG2530
Cp_Diaphoretickes-Dspp_4292_OG2530
Cp_Diaphoretickes-Smin_22288_OG2530
Bacteria-UP_b000018168_2074_OG2530
Bacteria-UP_b000018006_1425_OG2530
Other_Holomycota-Patl_g6682_OG2530
Other_Diaphoretickes-Gavo_5321_OG2530
Osmo_Pseudofungi-Pvex_10229_OG2530_intClade_1
Osmo_Pseudofungi-Pinf_9428_OG2530_intClade_1
Osmo_Fungi-Ncra_6858_OG2530_intClade_1
Osmo_Fungi-Nirr_3209_OG2530
Osmo_Fungi-Mver_7221_OG2530
Osmo_Fungi-Spun_5602_OG2530
Osmo_Fungi-Mver_6944_OG2530
Osmo_Fungi-Gpro_9787_OG2530
Osmo_Fungi-Rory_9258_OG2530
Osmo_Fungi-Rory_448_OG2530
Osmo_Fungi-Pbla_2371_OG2530
Bacteria-UP_b000037996_2074_OG2530
Bacteria-UP_b000033200_1185_OG2530
Bacteria-UP_b000009134_3111_OG2530
Bacteria-UP_b000004310_890_OG2530
Bacteria-UP_b000027583_212_OG2530
Other_Other-Agod_972_OG2530
Cp_Diaphoretickes-Ehux_23336_OG2530
Cp_Diaphoretickes-Aano_10802_OG2530
Cp_Diaphoretickes-Smin_9442_OG2530
Other_Holozoa-Mvib_g7059_OG2530
Bacteria-UP_b000008908_3157_OG2530
Bacteria-UP_b000007364_922_OG2530
Bacteria-UP_b000001601_1972_OG2530
Bacteria-UP_b000002028_3405_OG2530
Bacteria-UP_b000009309_2264_OG2530
Bacteria-UP_b000019423_1455_OG2530
Bacteria-UP_b000019423_4142_OG2530
Bacteria-UP_b000004671_2935_OG2530
Bacteria-UP_b000032900_1464_OG2530
Bacteria-UP_b000003746_2709_OG2530
Bacteria-UP_b000001025_3838_OG2530
Bacteria-UP_b000003839_2859_OG2530
Bacteria-UP_b000018064_1615_OG2530
Pp_Diaphoretickes-Cscu_4810_OG2530
Other_Other-Goke_21187_OG2530
Other_Other-Egra_15266_OG2530
Bacteria-UP_b000002221_729_OG2530
Other_Amorphea-Mbal_4149_OG2530
Other_SAR-Rfil_33431_OG2530
Other_Diaphoretickes-Omar_18127_OG2530
Cp_Diaphoretickes-Vbra_8201_OG2530
Bacteria-UP_b000008803_1059_OG2530
Cp_Diaphoretickes-Ngad_6445_OG2530
Cp_Diaphoretickes-Ngad_7261_OG2530
Other_Diaphoretickes-Hspp_8023_OG2530
Cp_Diaphoretickes-Cvel_13108_OG2530
Cp_Diaphoretickes-Esil_12546_OG2530
Osmo_Labyrinthulea-Alim_47804_OG2530
Osmo_Labyrinthulea-Hfer_5880_OG2530
Osmo_Labyrinthulea-Sagg_82350_OG2530
Other_Amorphea-Ttra_875_OG2530
Other_Stramenopiles-Bhom_1262_OG2530
Pp_Diaphoretickes-Pyez_6414_OG2530
Pp_Diaphoretickes-Gsul_6594_OG2530
Pp_Diaphoretickes-Ccri_1431_OG2530
Other_Other-Egra_34790_OG2530
Other_Stramenopiles-Croe_3590_OG2530
Other_Diaphoretickes-Aspp_6090_OG2530
Other_Other-Sspe_13532_OG2530
Osmo_Labyrinthulea-Aker_19537_OG2530
Osmo_Teretosporea-Sarc_9417_OG2530
Osmo_Teretosporea-Sarc_7211_OG2530
Osmo_Teretosporea-Cfra_6967_OG2530
Other_Stramenopiles-Bhom_2067_OG2530
Pp_Diaphoretickes-Ccri_669_OG2530
Pp_Diaphoretickes-Pyez_7966_OG2530
Bacteria-UP_b000018094_775_OG2530
Other_Diaphoretickes-Bmot_9125_OG2530
Other_Other-Sspe_17569_OG2530
Bacteria-UP_b000033393_5834_OG2530
Bacteria-UP_b000007254_1228_OG2530
Bacteria-UP_b000009223_3373_OG2530
Bacteria-UP_b000007383_455_OG2530
Bacteria-UP_b000001887_289_OG2530
Bacteria-UP_b000004358_3816_OG2530
Bacteria-UP_b000005824_1016_OG2530
Bacteria-UP_b000003087_623_OG2530
Other_Amorphea-Pbif_7857_OG2530
Other_Holozoa-Mbre_545_OG2530
Other_Amorphea-Sdol_38130_OG2530
Other_Holozoa-Sros_8266_OG2530
Other_Amorphea-Dgra_39238_OG2530
Other_Holozoa-Cowc_2823_OG2530
Other_Holozoa-Pvie_g5170_OG2530
Osmo_Teretosporea-Pgem_10699_OG2530
Osmo_Teretosporea-Pgem_5970_OG2530
Osmo_Teretosporea-Awhi_684_OG2530
Osmo_Teretosporea-Ihof_4587_OG2530
Osmo_Teretosporea-Apar_258_OG2530
Bacteria-UP_b000018292_1414_OG2530
Bacteria-UP_b000011220_1076_OG2530
Cp_Diaphoretickes-Bnat_4519_OG2530
Osmo_Teretosporea-Cper_6342_OG2530
Osmo_Teretosporea-Sdes_5101_OG2530
Osmo_Teretosporea-Sdes_3244_OG2530
Osmo_Teretosporea-Sdes_5587_OG2530
Other_Holozoa-Mbre_7952_OG2530
Other_Holozoa-Nvec_385_OG2530
Other_Amorphea-Bflo_11342_OG2530
Other_Holozoa-Hsap_8297_OG2530
Other_Amorphea-Mlei_9497_OG2530
Other_Amorphea-Tspp_4245_OG2530
Other_Holozoa-Dpul_10279_OG2530
Other_Holozoa-Dpul_21137_OG2530
Other_Diaphoretickes-Rlim_5200_OG2530
Other_Diaphoretickes-Rmar_3053_OG2530
Other_Amorphea-Opea_25272_OG2530
Other_SAR-Tthe_17285_OG2530
Pp_Diaphoretickes-Smoe_38802_OG2530
Pp_Diaphoretickes-Smoe_4620_OG2530
Pp_Diaphoretickes-Smoe_20988_OG2530
Pp_Diaphoretickes-Smoe_4653_OG2530
Pp_Diaphoretickes-Cscu_4811_OG2530
Pp_Diaphoretickes-Mpol_816_OG2530
Pp_Diaphoretickes-Atha_3390_OG2530
Pp_Diaphoretickes-Atha_1475_OG2530
Pp_Diaphoretickes-Atha_22355_OG2530
Pp_Diaphoretickes-Atha_18349_OG2530
Pp_Diaphoretickes-Pcol_1819_OG2530
Pp_Diaphoretickes-Knit_5644_OG2530
Pp_Diaphoretickes-Kfla_2065_OG2530
Pp_Diaphoretickes-Cscu_11930_OG2530
Pp_Diaphoretickes-Catm_312_OG2530
Pp_Diaphoretickes-Cvar_2525_OG2530
Pp_Diaphoretickes-Cvar_4046_OG2530
Cp_Diaphoretickes-Chsp_5562_OG2530
Pp_Diaphoretickes-Cvar_9760_OG2530
Pp_Diaphoretickes-Cvar_9443_OG2530
Pp_Diaphoretickes-Vcar_201_OG2530
Pp_Diaphoretickes-Crei_10503_OG2530
Pp_Diaphoretickes-Bpra_1357_OG2530
Pp_Diaphoretickes-Otau_3673_OG2530
Pp_Diaphoretickes-Mpus_8500_OG2530
Pp_Diaphoretickes-Npyr_7349_OG2530
Cp_Diaphoretickes-Bfri_27492_OG2530
Cp_Diaphoretickes-Bfri_5385_OG2530
Cp_Diaphoretickes-Bfri_32217_OG2530
Cp_Diaphoretickes-Tpse_9335_OG2530
Cp_Diaphoretickes-Gthe_5983_OG2530
Pp_Diaphoretickes-Cscu_4907_OG2530
Pp_Diaphoretickes-Cscu_11231_OG2530
Other_Other-Agod_5257_OG2530
Other_Other-Goke_22596_OG2530
0.3

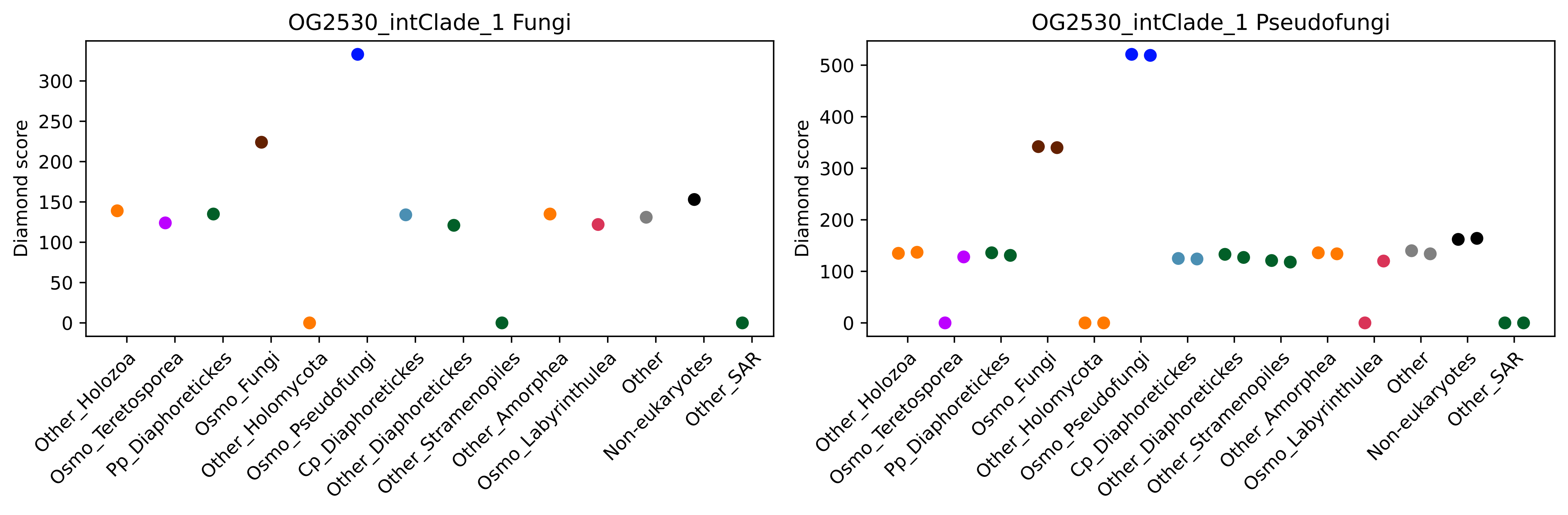
OG2530_intClade_1 Fungi
OG2530_intClade_1 Pseudofungi
Diamond score
Other_Holozoa
Osmo_Teretosporea
Pp_Diaphoretickes
Osmo_Fungi
Other_Holomycota
Osmo_Pseudofungi
Cp_Diaphoretickes
Other_Diaphoretickes
Other_Stramenopiles
Other_Amorphea
Osmo_Labyrinthulea
Other
Non-eukaryotes
Other_SAR

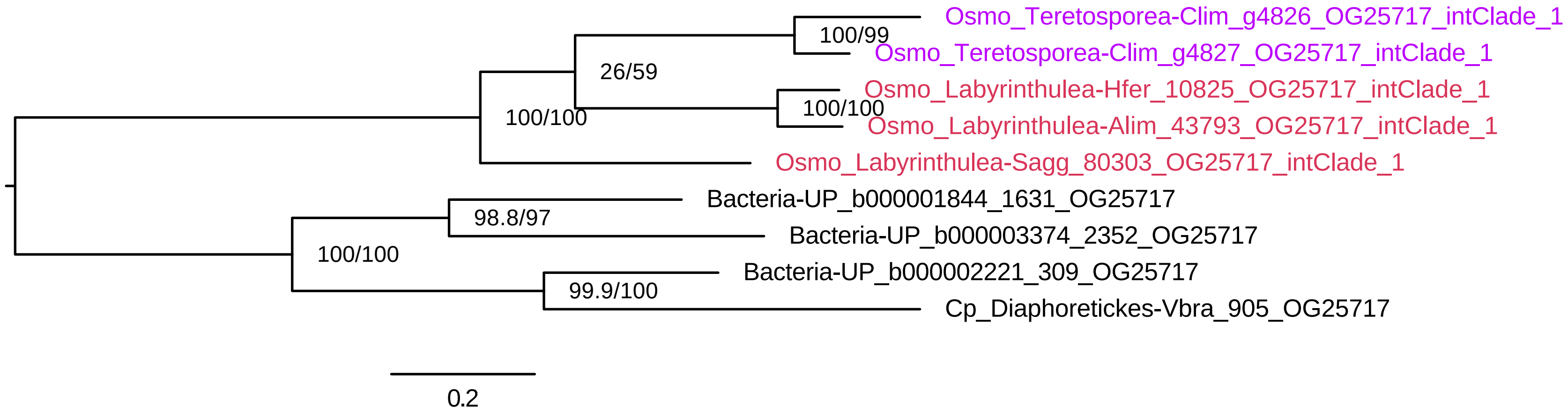

Osmo_Teretosporea-Clim_g4826_OG25717_intClade_1
100/99
Osmo_Teretosporea-Clim_g4827_OG25717_intClade_1
26/59
Osmo_Labyrinthulea-Hfer_10825_OG25717_intClade_1
100/100
Osmo_Labyrinthulea-Alim_43793_OG25717_intClade_1
100/100
Osmo_Labyrinthulea-Sagg_80303_OG25717_intClade_1
Bacteria-UP_b000001844_1631_OG25717
98.8/97
Bacteria-UP_b000003374_2352_OG25717
100/100
Bacteria-UP_b000002221_309_OG25717
99.9/100
Cp_Diaphoretickes-Vbra_905_OG25717
0.2

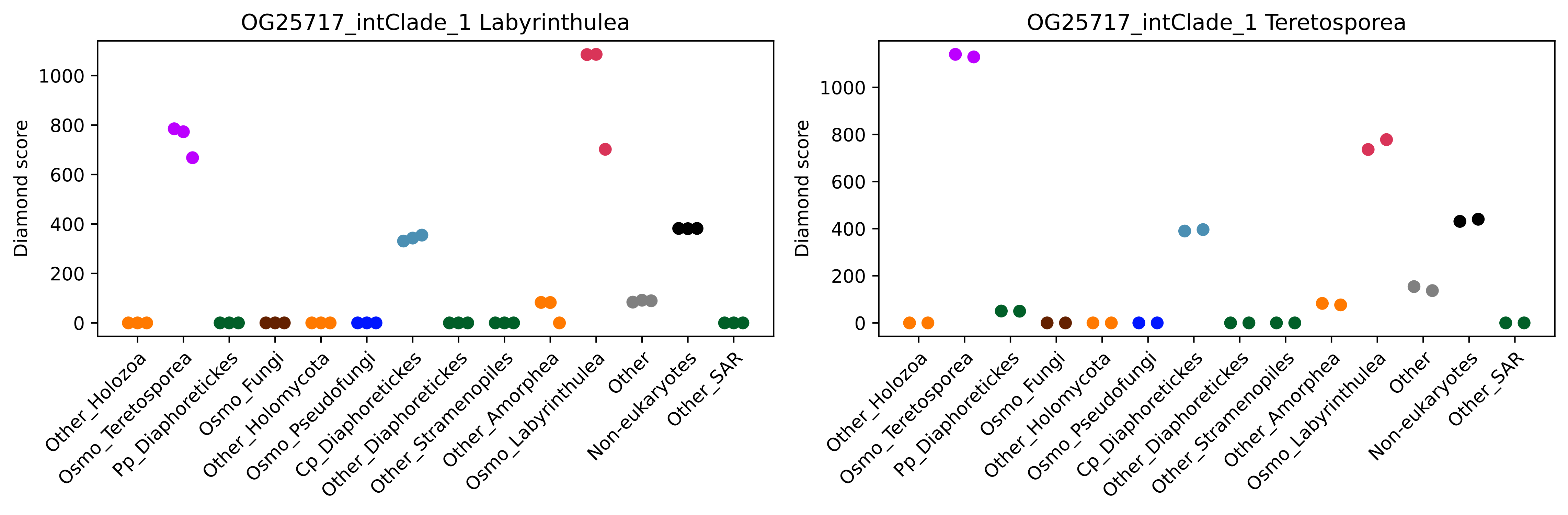
OG25717_intClade_1 Labyrinthulea
Diamond score
0
200
400
600
800
1000
Other_Holozoa
Osmo_Teretosporea
Pp_Diaphoretickes
Osmo_Fungi
Other_Holomycota
Osmo_Pseudofungi
Cp_Diaphoretickes
Other_Diaphoretickes
Other_Stramenopiles
Other_Amorphea
Osmo_Labyrinthulea
Other
Non-eukaryotes
Other_SAR
OG25717_intClade_1 Teretosporea
Diamond score
0
200
400
600
800
1000
Other_Holozoa
Osmo_Teretosporea
Pp_Diaphoretickes
Osmo_Fungi
Other_Holomycota
Osmo_Pseudofungi
Cp_Diaphoretickes
Other_Diaphoretickes
Other_Stramenopiles
Other_Amorphea
Osmo_Labyrinthulea
Other
Non-eukaryotes
Other_SAR

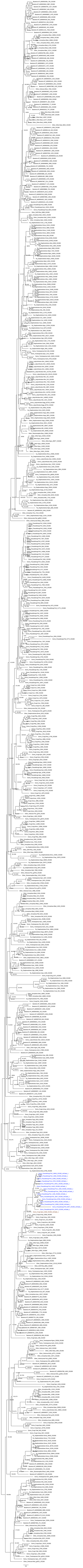

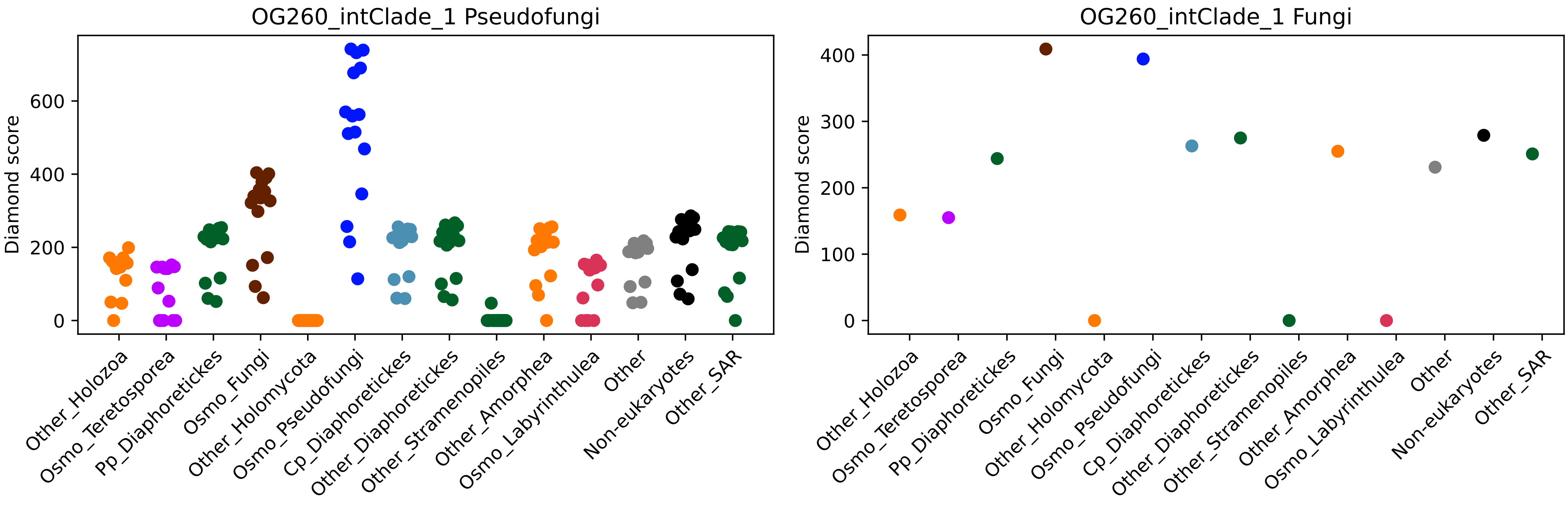

OG260_intClade_1 Pseudofungi
OG260_intClade_1 Fungi
Diamond score
0
200
400
600
100
300
Other_Holozoa
Osmo_Teretosporea
Pp_Diaphoretickes
Osmo_Fungi
Other_Holomycota
Osmo_Pseudofungi
Cp_Diaphoretickes
Other_Diaphoretickes
Other_Stramenopiles
Other_Amorphea
Osmo_Labyrinthulea
Other
Non-eukaryotes
Other_SAR

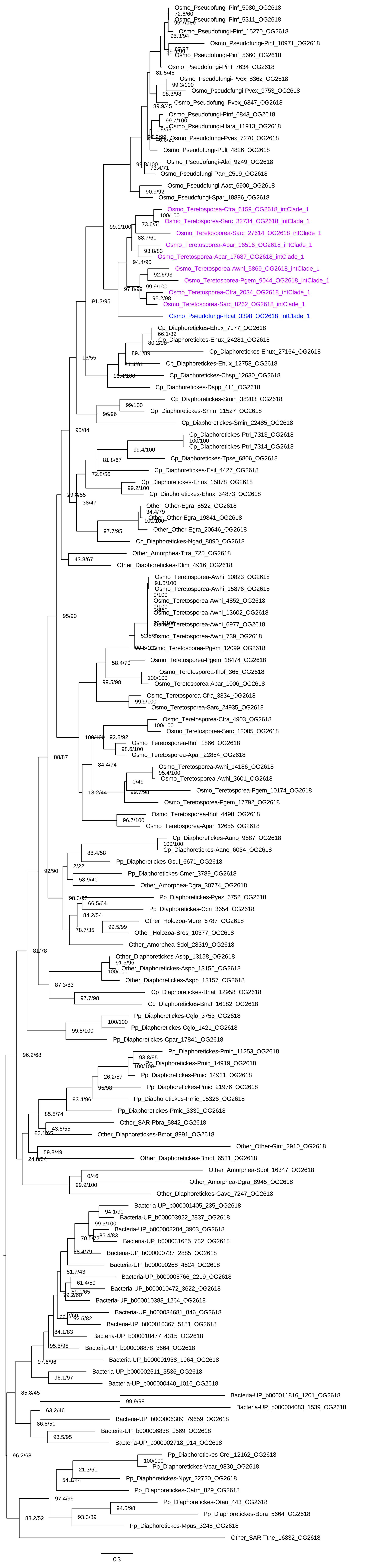
Osmo_Pseudofungi-Pinf_5980_OG2618
Osmo_Pseudofungi-Pinf_5311_OG2618
Osmo_Pseudofungi-Pinf_15270_OG2618
Osmo_Pseudofungi-Pinf_10971_OG2618
Osmo_Pseudofungi-Pinf_5660_OG2618
Osmo_Pseudofungi-Pinf_7634_OG2618
Osmo_Pseudofungi-Pvex_8362_OG2618
Osmo_Pseudofungi-Pvex_9753_OG2618
Osmo_Pseudofungi-Pvex_6347_OG2618
Osmo_Pseudofungi-Pinf_6843_OG2618
Osmo_Pseudofungi-Hara_11913_OG2618
Osmo_Pseudofungi-Pvex_7270_OG2618
Osmo_Pseudofungi-Pult_4826_OG2618
Osmo_Pseudofungi-Alai_9249_OG2618
Osmo_Pseudofungi-Parr_2519_OG2618
Osmo_Pseudofungi-Aast_6900_OG2618
Osmo_Pseudofungi-Spar_18896_OG2618
Osmo_Teretosporea-Cfra_6159_OG2618_intClade_1
Osmo_Teretosporea-Sarc_32734_OG2618_intClade_1
Osmo_Teretosporea-Sarc_27614_OG2618_intClade_1
Osmo_Teretosporea-Apar_16516_OG2618_intClade_1
Osmo_Teretosporea-Apar_17687_OG2618_intClade_1
Osmo_Teretosporea-Awhi_5869_OG2618_intClade_1
Osmo_Teretosporea-Pgem_9044_OG2618_intClade_1
Osmo_Teretosporea-Cfra_2034_OG2618_intClade_1
Osmo_Teretosporea-Sarc_8262_OG2618_intClade_1
Osmo_Pseudofungi-Hcat_3398_OG2618_intClade_1
Cp_Diaphoretickes-Ehux_7177_OG2618
Cp_Diaphoretickes-Ehux_24281_OG2618
Cp_Diaphoretickes-Ehux_27164_OG2618
Cp_Diaphoretickes-Ehux_12758_OG2618
Cp_Diaphoretickes-Chsp_12630_OG2618
Cp_Diaphoretickes-Dspp_411_OG2618
Cp_Diaphoretickes-Smin_38203_OG2618
Cp_Diaphoretickes-Smin_11527_OG2618
Cp_Diaphoretickes-Smin_22485_OG2618
Cp_Diaphoretickes-Ptri_7313_OG2618
Cp_Diaphoretickes-Ptri_7314_OG2618
Cp_Diaphoretickes-Tpse_6806_OG2618
Cp_Diaphoretickes-Esil_4427_OG2618
Cp_Diaphoretickes-Ehux_15878_OG2618
Cp_Diaphoretickes-Ehux_34873_OG2618
Other_Other-Egra_8522_OG2618
Other_Other-Egra_19841_OG2618
Other_Other-Egra_20646_OG2618
Cp_Diaphoretickes-Ngad_8090_OG2618
Other_Amorphea-Ttra_725_OG2618
Other_Diaphoretickes-Rlim_4916_OG2618
Osmo_Teretosporea-Awhi_10823_OG2618
Osmo_Teretosporea-Awhi_15876_OG2618
Osmo_Teretosporea-Awhi_4852_OG2618
Osmo_Teretosporea-Awhi_13602_OG2618
Osmo_Teretosporea-Awhi_6977_OG2618
Osmo_Teretosporea-Awhi_739_OG2618
Osmo_Teretosporea-Pgem_12099_OG2618
Osmo_Teretosporea-Pgem_18474_OG2618
Osmo_Teretosporea-Ihof_366_OG2618
Osmo_Teretosporea-Apar_1006_OG2618
Osmo_Teretosporea-Cfra_3334_OG2618
Osmo_Teretosporea-Sarc_24935_OG2618
Osmo_Teretosporea-Cfra_4903_OG2618
Osmo_Teretosporea-Sarc_12005_OG2618
Osmo_Teretosporea-Ihof_1866_OG2618
Osmo_Teretosporea-Apar_22854_OG2618
Osmo_Teretosporea-Awhi_14186_OG2618
Osmo_Teretosporea-Awhi_3601_OG2618
Osmo_Teretosporea-Pgem_10174_OG2618
Osmo_Teretosporea-Pgem_17792_OG2618
Osmo_Teretosporea-Ihof_4498_OG2618
Osmo_Teretosporea-Apar_12655_OG2618
Cp_Diaphoretickes-Aano_9687_OG2618
Cp_Diaphoretickes-Aano_6034_OG2618
Pp_Diaphoretickes-Gsul_6671_OG2618
Pp_Diaphoretickes-Cmer_3789_OG2618
Other_Amorphea-Dgra_30774_OG2618
Pp_Diaphoretickes-Pyez_6752_OG2618
Pp_Diaphoretickes-Ccri_3654_OG2618
Other_Holozoa-Mbre_6787_OG2618
Other_Holozoa-Sros_10377_OG2618
Other_Amorphea-Sdol_28319_OG2618
Other_Diaphoretickes-Aspp_13158_OG2618
Other_Diaphoretickes-Aspp_13156_OG2618
Other_Diaphoretickes-Aspp_13157_OG2618
Cp_Diaphoretickes-Bnat_12958_OG2618
Cp_Diaphoretickes-Bnat_16182_OG2618
Pp_Diaphoretickes-Cglo_3753_OG2618
Pp_Diaphoretickes-Cglo_1421_OG2618
Pp_Diaphoretickes-Cpar_17841_OG2618
Pp_Diaphoretickes-Pmic_11253_OG2618
Pp_Diaphoretickes-Pmic_14919_OG2618
Pp_Diaphoretickes-Pmic_14921_OG2618
Pp_Diaphoretickes-Pmic_21976_OG2618
Pp_Diaphoretickes-Pmic_15326_OG2618
Pp_Diaphoretickes-Pmic_3339_OG2618
Other_SAR-Pbra_5842_OG2618
Other_Diaphoretickes-Bmot_8991_OG2618
Other_Other-Gint_2910_OG2618
Other_Diaphoretickes-Bmot_6531_OG2618
Other_Amorphea-Sdol_16347_OG2618
Other_Amorphea-Dgra_8945_OG2618
Other_Diaphoretickes-Gavo_7247_OG2618
Bacteria-UP_b000001405_235_OG2618
Bacteria-UP_b000003922_2837_OG2618
Bacteria-UP_b000008204_3903_OG2618
Bacteria-UP_b000031625_732_OG2618
Bacteria-UP_b000000737_2885_OG2618
Bacteria-UP_b000000268_4624_OG2618
Bacteria-UP_b000005766_2219_OG2618
Bacteria-UP_b000010472_3622_OG2618
Bacteria-UP_b000010383_1264_OG2618
Bacteria-UP_b000034681_846_OG2618
Bacteria-UP_b000010367_5181_OG2618
Bacteria-UP_b000010477_4315_OG2618
Bacteria-UP_b000008878_3664_OG2618
Bacteria-UP_b000001938_1964_OG2618
Bacteria-UP_b000002511_3536_OG2618
Bacteria-UP_b000000440_1016_OG2618
Bacteria-UP_b000011816_1201_OG2618
Bacteria-UP_b000004083_1539_OG2618
Bacteria-UP_b000006309_79659_OG2618
Bacteria-UP_b000006838_1669_OG2618
Bacteria-UP_b000002718_914_OG2618
Pp_Diaphoretickes-Crei_12162_OG2618
Pp_Diaphoretickes-Vcar_9830_OG2618
Pp_Diaphoretickes-Npyr_22720_OG2618
Pp_Diaphoretickes-Catm_829_OG2618
Pp_Diaphoretickes-Otau_443_OG2618
Pp_Diaphoretickes-Bpra_5664_OG2618
Pp_Diaphoretickes-Mpus_3248_OG2618
Other_SAR-Tthe_16832_OG2618
0.3

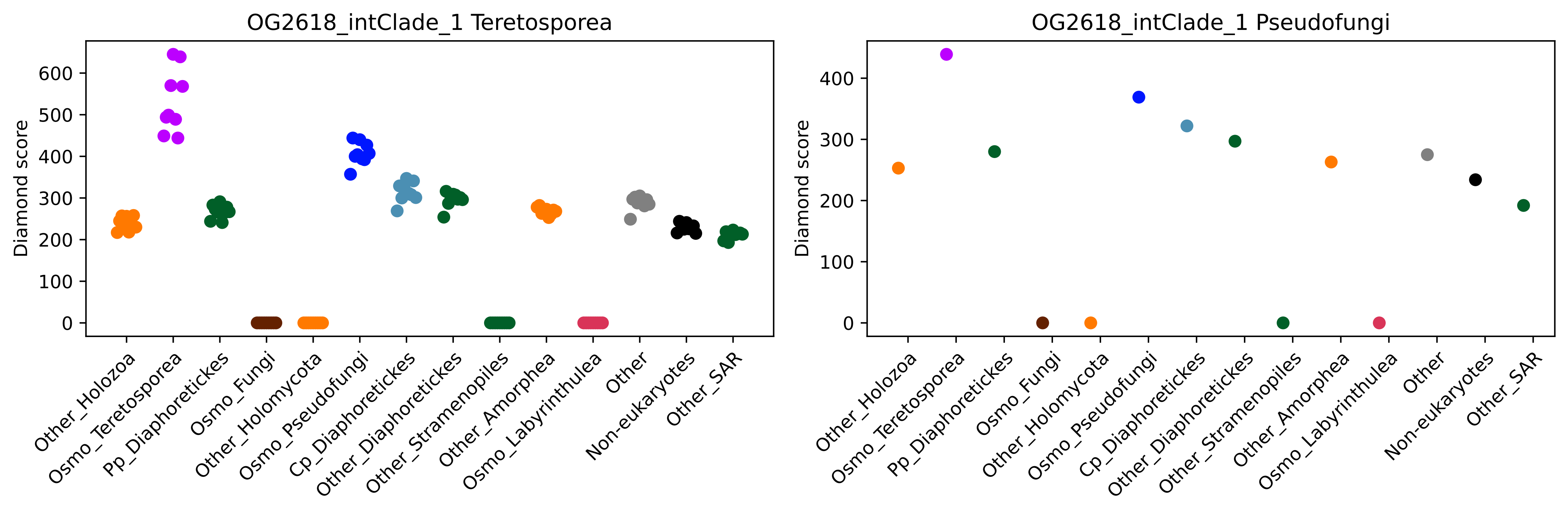

OG2618_intClade_1 Teretosporea
OG2618_intClade_1 Pseudofungi
Diamond score
Other_Holozoa
Osmo_Teretosporea
Pp_Diaphoretickes
Osmo_Fungi
Other_Holomycota
Osmo_Pseudofungi
Cp_Diaphoretickes
Other_Diaphoretickes
Other_Stramenopiles
Other_Amorphea
Osmo_Labyrinthulea
Other
Non-eukaryotes
Other_SAR

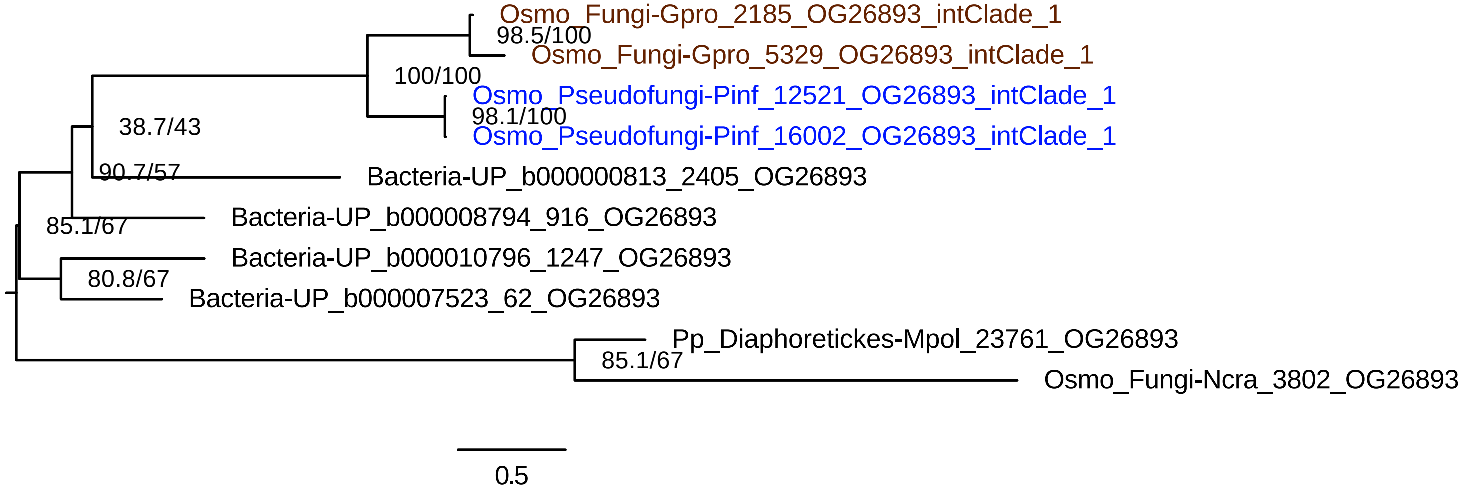
Osmo_Fungi-Gpro_2185_OG26893_intClade_1
98.5/100
Osmo_Fungi-Gpro_5329_OG26893_intClade_1
100/100
Osmo_Pseudofungi-Pinf_12521_OG26893_intClade_1
98.1/100
Osmo_Pseudofungi-Pinf_16002_OG26893_intClade_1
38.7/43
90.7/57
Bacteria-UP_b000000813_2405_OG26893
Bacteria-UP_b000008794_916_OG26893
85.1/67
Bacteria-UP_b000010796_1247_OG26893
80.8/67
Bacteria-UP_b000007523_62_OG26893
Pp_Diaphoretickes-Mpol_23761_OG26893
85.1/67
Osmo_Fungi-Ncra_3802_OG26893
0.5

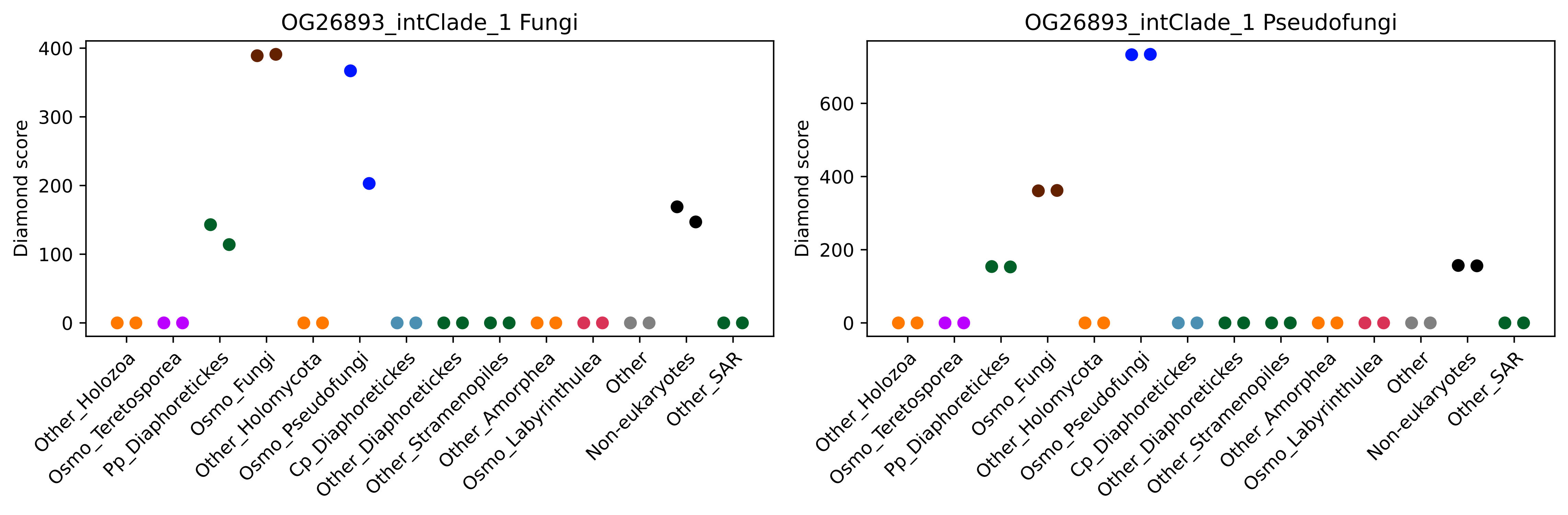

OG26893_intClade_1 Fungi
OG26893_intClade_1 Pseudofungi
Diamond score
0
100
200
300
400
600
Other_Holozoa
Osmo_Teretosporea
Pp_Diaphoretickes
Osmo_Fungi
Other_Holomycota
Osmo_Pseudofungi
Cp_Diaphoretickes
Other_Diaphoretickes
Other_Stramenopiles
Other_Amorphea
Osmo_Labyrinthulea
Other
Non-eukaryotes
Other_SAR

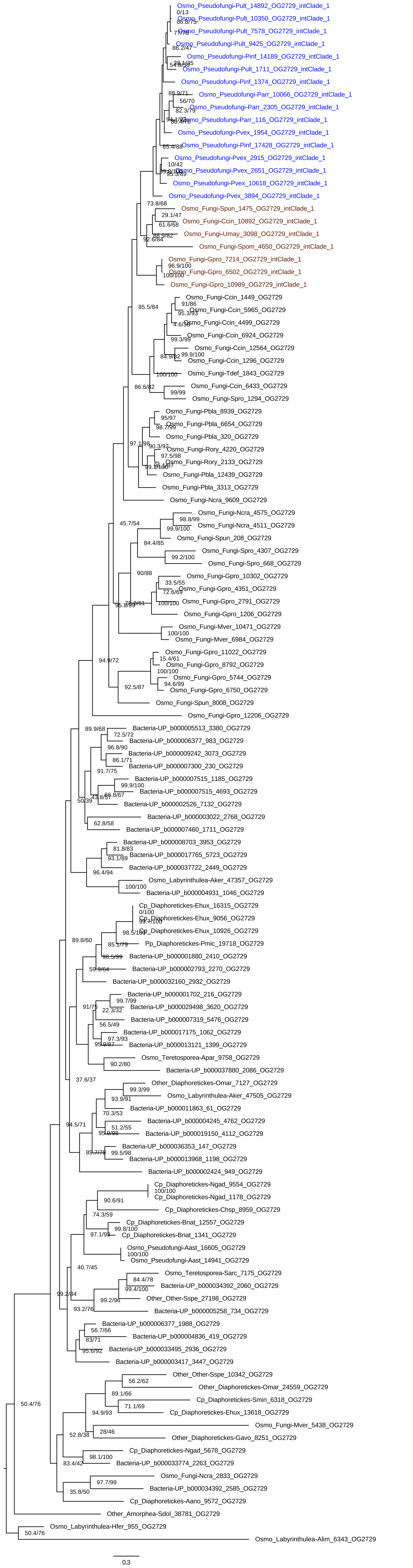

Osmo_Pseudofungi-Pult_14892_OG2729_intClade_1
Osmo_Pseudofungi-Pult_10350_OG2729_intClade_1
Osmo_Pseudofungi-Pult_7578_OG2729_intClade_1
Osmo_Pseudofungi-Pult_9425_OG2729_intClade_1
Osmo_Pseudofungi-Pinf_14189_OG2729_intClade_1
Osmo_Pseudofungi-Pult_1711_OG2729_intClade_1
Osmo_Pseudofungi-Pinf_1374_OG2729_intClade_1
Osmo_Pseudofungi-Parr_10066_OG2729_intClade_1
Osmo_Pseudofungi-Parr_2305_OG2729_intClade_1
Osmo_Pseudofungi-Parr_116_OG2729_intClade_1
Osmo_Pseudofungi-Pvex_1954_OG2729_intClade_1
Osmo_Pseudofungi-Pinf_17428_OG2729_intClade_1
Osmo_Pseudofungi-Pvex_2915_OG2729_intClade_1
Osmo_Pseudofungi-Pvex_2651_OG2729_intClade_1
Osmo_Pseudofungi-Pvex_10618_OG2729_intClade_1
Osmo_Pseudofungi-Pvex_3894_OG2729_intClade_1
Osmo_Fungi-Spun_1475_OG2729_intClade_1
Osmo_Fungi-Ccin_10892_OG2729_intClade_1
Osmo_Fungi-Umay_3098_OG2729_intClade_1
Osmo_Fungi-Spom_4650_OG2729_intClade_1
Osmo_Fungi-Gpro_7214_OG2729_intClade_1
Osmo_Fungi-Gpro_6502_OG2729_intClade_1
Osmo_Fungi-Gpro_10989_OG2729_intClade_1
Osmo_Fungi-Ccin_1449_OG2729
Osmo_Fungi-Ccin_5965_OG2729
Osmo_Fungi-Ccin_4499_OG2729
Osmo_Fungi-Ccin_6924_OG2729
Osmo_Fungi-Ccin_12564_OG2729
Osmo_Fungi-Ccin_1296_OG2729
Osmo_Fungi-Tdef_1843_OG2729
Osmo_Fungi-Ccin_6433_OG2729
Osmo_Fungi-Spro_1294_OG2729
Osmo_Fungi-Pbla_8939_OG2729
Osmo_Fungi-Pbla_6654_OG2729
Osmo_Fungi-Pbla_320_OG2729
Osmo_Fungi-Rory_4220_OG2729
Osmo_Fungi-Rory_2133_OG2729
Osmo_Fungi-Pbla_12439_OG2729
Osmo_Fungi-Pbla_3313_OG2729
Osmo_Fungi-Ncra_9609_OG2729
Osmo_Fungi-Ncra_4575_OG2729
Osmo_Fungi-Ncra_4511_OG2729
Osmo_Fungi-Spun_208_OG2729
Osmo_Fungi-Spro_4307_OG2729
Osmo_Fungi-Spro_668_OG2729
Osmo_Fungi-Gpro_10302_OG2729
Osmo_Fungi-Gpro_4351_OG2729
Osmo_Fungi-Gpro_2791_OG2729
Osmo_Fungi-Gpro_1206_OG2729
Osmo_Fungi-Mver_10471_OG2729
Osmo_Fungi-Mver_6984_OG2729
Osmo_Fungi-Gpro_11022_OG2729
Osmo_Fungi-Gpro_8792_OG2729
Osmo_Fungi-Gpro_5744_OG2729
Osmo_Fungi-Gpro_6750_OG2729
Osmo_Fungi-Spun_8008_OG2729
Osmo_Fungi-Gpro_12206_OG2729
Bacteria-UP_b000005513_3380_OG2729
Bacteria-UP_b000006377_983_OG2729
Bacteria-UP_b000009242_3073_OG2729
Bacteria-UP_b000007300_230_OG2729
Bacteria-UP_b000007515_1185_OG2729
Bacteria-UP_b000007515_4693_OG2729
Bacteria-UP_b000002526_7132_OG2729
Bacteria-UP_b000003022_2768_OG2729
Bacteria-UP_b000007460_1711_OG2729
Bacteria-UP_b000008703_3953_OG2729
Bacteria-UP_b000017765_5723_OG2729
Bacteria-UP_b000037722_2449_OG2729
Osmo_Labyrinthulea-Aker_47357_OG2729
Bacteria-UP_b000004931_1046_OG2729
Cp_Diaphoretickes-Ehux_16315_OG2729
Cp_Diaphoretickes-Ehux_9056_OG2729
Cp_Diaphoretickes-Ehux_10926_OG2729
Pp_Diaphoretickes-Pmic_19718_OG2729
Bacteria-UP_b000001880_2410_OG2729
Bacteria-UP_b000002793_2270_OG2729
Bacteria-UP_b000032160_2932_OG2729
Bacteria-UP_b000001702_216_OG2729
Bacteria-UP_b000029498_3620_OG2729
Bacteria-UP_b000007319_5476_OG2729
Bacteria-UP_b000017175_1062_OG2729
Bacteria-UP_b000013121_1399_OG2729
Osmo_Teretosporea-Apar_9758_OG2729
Bacteria-UP_b000037880_2086_OG2729
Other_Diaphoretickes-Omar_7127_OG2729
Osmo_Labyrinthulea-Aker_47505_OG2729
Bacteria-UP_b000011863_61_OG2729
Bacteria-UP_b000004245_4762_OG2729
Bacteria-UP_b000019150_4112_OG2729
Bacteria-UP_b000036353_147_OG2729
Bacteria-UP_b000013968_1198_OG2729
Bacteria-UP_b000002424_949_OG2729
Cp_Diaphoretickes-Ngad_9554_OG2729
Cp_Diaphoretickes-Ngad_1178_OG2729
Cp_Diaphoretickes-Chsp_8959_OG2729
Cp_Diaphoretickes-Bnat_12557_OG2729
Cp_Diaphoretickes-Bnat_1341_OG2729
Osmo_Pseudofungi-Aast_16605_OG2729
Osmo_Pseudofungi-Aast_14941_OG2729
Osmo_Teretosporea-Sarc_7175_OG2729
Bacteria-UP_b000034392_2060_OG2729
Other_Other-Sspe_27198_OG2729
Bacteria-UP_b000005258_734_OG2729
Bacteria-UP_b000006377_1988_OG2729
Bacteria-UP_b000004836_419_OG2729
Bacteria-UP_b000033495_2936_OG2729
Bacteria-UP_b000003417_3447_OG2729
Other_Other-Sspe_10342_OG2729
Other_Diaphoretickes-Omar_24559_OG2729
Cp_Diaphoretickes-Smin_6318_OG2729
Cp_Diaphoretickes-Ehux_13618_OG2729
Osmo_Fungi-Mver_5438_OG2729
Other_Diaphoretickes-Gavo_8251_OG2729
Cp_Diaphoretickes-Ngad_5678_OG2729
Bacteria-UP_b000033774_2263_OG2729
Osmo_Fungi-Ncra_2833_OG2729
Bacteria-UP_b000034392_2585_OG2729
Cp_Diaphoretickes-Aano_9572_OG2729
Other_Amorphea-Sdol_38781_OG2729
Osmo_Labyrinthulea-Hfer_955_OG2729
Osmo_Labyrinthulea-Alim_6343_OG2729
0.3

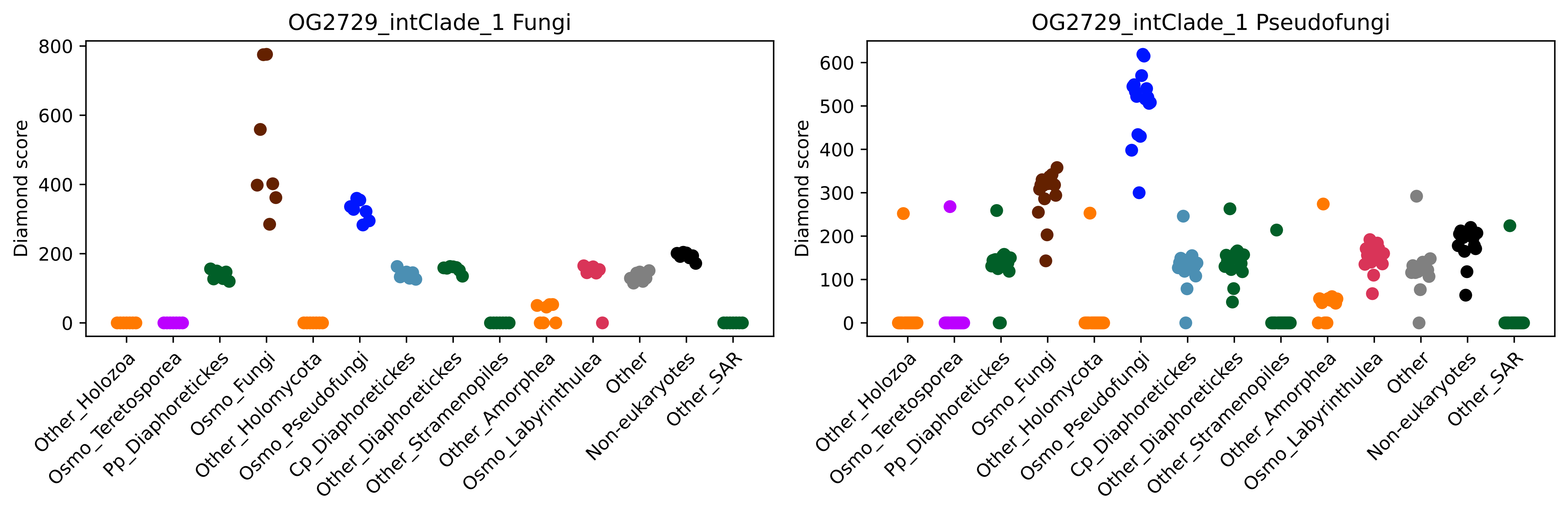
OG2729_intClade_1 Fungi
OG2729_intClade_1 Pseudofungi
Diamond score
0
200
400
600
800
100
300
500
Other_Holozoa
Osmo_Teretosporea
Pp_Diaphoretickes
Osmo_Fungi
Other_Holomycota
Osmo_Pseudofungi
Cp_Diaphoretickes
Other_Diaphoretickes
Other_Stramenopiles
Other_Amorphea
Osmo_Labyrinthulea
Other
Non-eukaryotes
Other_SAR

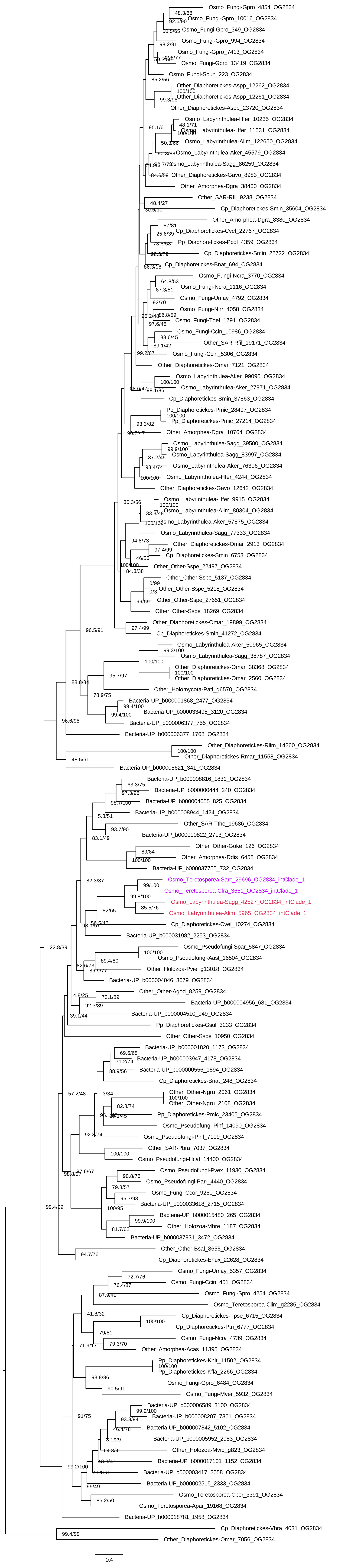
Osmo_Fungi-Gpro_4854_OG2834
Osmo_Fungi-Gpro_10016_OG2834
Osmo_Fungi-Gpro_349_OG2834
Osmo_Fungi-Gpro_994_OG2834
Osmo_Fungi-Gpro_7413_OG2834
Osmo_Fungi-Gpro_13419_OG2834
Osmo_Fungi-Spun_223_OG2834
Other_Diaphoretickes-Aspp_12262_OG2834
Other_Diaphoretickes-Aspp_12261_OG2834
Other_Diaphoretickes-Aspp_23720_OG2834
Osmo_Labyrinthulea-Hfer_10235_OG2834
Osmo_Labyrinthulea-Hfer_11531_OG2834
Osmo_Labyrinthulea-Alim_122650_OG2834
Osmo_Labyrinthulea-Aker_45579_OG2834
Osmo_Labyrinthulea-Sagg_86259_OG2834
Other_Diaphoretickes-Gavo_8983_OG2834
Other_Amorphea-Dgra_38400_OG2834
Other_SAR-Rfil_9238_OG2834
Cp_Diaphoretickes-Smin_35604_OG2834
Other_Amorphea-Dgra_8380_OG2834
Cp_Diaphoretickes-Cvel_22767_OG2834
Pp_Diaphoretickes-Pcol_4359_OG2834
Cp_Diaphoretickes-Smin_22722_OG2834
Cp_Diaphoretickes-Bnat_694_OG2834
Osmo_Fungi-Ncra_3770_OG2834
Osmo_Fungi-Ncra_1116_OG2834
Osmo_Fungi-Umay_4792_OG2834
Osmo_Fungi-Nirr_4058_OG2834
Osmo_Fungi-Tdef_1791_OG2834
Osmo_Fungi-Ccin_10986_OG2834
Other_SAR-Rfil_19171_OG2834
Osmo_Fungi-Ccin_5306_OG2834
Other_Diaphoretickes-Omar_7121_OG2834
Osmo_Labyrinthulea-Aker_99090_OG2834
Osmo_Labyrinthulea-Aker_27971_OG2834
Cp_Diaphoretickes-Smin_37863_OG2834
Pp_Diaphoretickes-Pmic_28497_OG2834
Pp_Diaphoretickes-Pmic_27214_OG2834
Other_Amorphea-Dgra_10764_OG2834
Osmo_Labyrinthulea-Sagg_39500_OG2834
Osmo_Labyrinthulea-Sagg_83997_OG2834
Osmo_Labyrinthulea-Aker_76306_OG2834
Osmo_Labyrinthulea-Hfer_4244_OG2834
Other_Diaphoretickes-Gavo_12642_OG2834
Osmo_Labyrinthulea-Hfer_9915_OG2834
Osmo_Labyrinthulea-Alim_80304_OG2834
Osmo_Labyrinthulea-Aker_57875_OG2834
Osmo_Labyrinthulea-Sagg_77333_OG2834
Other_Diaphoretickes-Omar_2913_OG2834
Cp_Diaphoretickes-Smin_6753_OG2834
Other_Other-Sspe_22497_OG2834
Other_Other-Sspe_5137_OG2834
Other_Other-Sspe_5218_OG2834
Other_Other-Sspe_27651_OG2834
Other_Other-Sspe_18269_OG2834
Other_Diaphoretickes-Omar_19899_OG2834
Cp_Diaphoretickes-Smin_41272_OG2834
Osmo_Labyrinthulea-Aker_50965_OG2834
Osmo_Labyrinthulea-Sagg_38787_OG2834
Other_Diaphoretickes-Omar_38368_OG2834
Other_Diaphoretickes-Omar_2560_OG2834
Other_Holomycota-Patl_g6570_OG2834
Bacteria-UP_b000001868_2477_OG2834
Bacteria-UP_b000033495_3120_OG2834
Bacteria-UP_b000006377_755_OG2834
Bacteria-UP_b000006377_1768_OG2834
Other_Diaphoretickes-Rlim_14260_OG2834
Other_Diaphoretickes-Rmar_11558_OG2834
Bacteria-UP_b000005621_341_OG2834
Bacteria-UP_b000008816_1831_OG2834
Bacteria-UP_b000000444_240_OG2834
Bacteria-UP_b000004055_825_OG2834
Bacteria-UP_b000008944_1424_OG2834
Other_SAR-Tthe_19686_OG2834
Bacteria-UP_b000000822_2713_OG2834
Other_Other-Goke_126_OG2834
Other_Amorphea-Ddis_6458_OG2834
Bacteria-UP_b000037755_732_OG2834
Osmo_Teretosporea-Sarc_29696_OG2834_intClade_1
Osmo_Teretosporea-Cfra_3651_OG2834_intClade_1
Osmo_Labyrinthulea-Sagg_42527_OG2834_intClade_1
Osmo_Labyrinthulea-Alim_5965_OG2834_intClade_1
Cp_Diaphoretickes-Cvel_10274_OG2834
Bacteria-UP_b000031982_2253_OG2834
Osmo_Pseudofungi-Spar_5847_OG2834
Osmo_Pseudofungi-Aast_16504_OG2834
Other_Holozoa-Pvie_g13018_OG2834
Bacteria-UP_b000004046_3679_OG2834
Other_Other-Agod_8259_OG2834
Bacteria-UP_b000004956_681_OG2834
Bacteria-UP_b000004510_949_OG2834
Pp_Diaphoretickes-Gsul_3233_OG2834
Other_Other-Sspe_10950_OG2834
Bacteria-UP_b000001820_1173_OG2834
Bacteria-UP_b000003947_4178_OG2834
Bacteria-UP_b000000556_1594_OG2834
Cp_Diaphoretickes-Bnat_248_OG2834
Other_Other-Ngru_2061_OG2834
Other_Other-Ngru_2108_OG2834
Pp_Diaphoretickes-Pmic_23405_OG2834
Osmo_Pseudofungi-Pinf_14090_OG2834
Osmo_Pseudofungi-Pinf_7109_OG2834
Other_SAR-Pbra_7037_OG2834
Osmo_Pseudofungi-Hcat_14400_OG2834
Osmo_Pseudofungi-Pvex_11930_OG2834
Osmo_Pseudofungi-Parr_4440_OG2834
Osmo_Fungi-Ccor_9260_OG2834
Bacteria-UP_b000033618_2715_OG2834
Bacteria-UP_b000015480_265_OG2834
Other_Holozoa-Mbre_1187_OG2834
Bacteria-UP_b000037931_3472_OG2834
Other_Other-Bsal_8655_OG2834
Cp_Diaphoretickes-Ehux_22628_OG2834
Osmo_Fungi-Umay_5357_OG2834
Osmo_Fungi-Ccin_451_OG2834
Osmo_Fungi-Spro_4254_OG2834
Osmo_Teretosporea-Clim_g2285_OG2834
Cp_Diaphoretickes-Tpse_6715_OG2834
Cp_Diaphoretickes-Ptri_6777_OG2834
Osmo_Fungi-Ncra_4739_OG2834
Other_Amorphea-Acas_11395_OG2834
Pp_Diaphoretickes-Knit_11502_OG2834
Pp_Diaphoretickes-Kfla_2266_OG2834
Osmo_Fungi-Gpro_6484_OG2834
Osmo_Fungi-Mver_5932_OG2834
Bacteria-UP_b000006589_3100_OG2834
Bacteria-UP_b000008207_7361_OG2834
Bacteria-UP_b000007842_5102_OG2834
Bacteria-UP_b000005952_2983_OG2834
Other_Holozoa-Mvib_g823_OG2834
Bacteria-UP_b000017101_1152_OG2834
Bacteria-UP_b000003417_2058_OG2834
Bacteria-UP_b000002515_2333_OG2834
Osmo_Teretosporea-Cper_3391_OG2834
Osmo_Teretosporea-Apar_19168_OG2834
Bacteria-UP_b000018781_1958_OG2834
Cp_Diaphoretickes-Vbra_4031_OG2834
Other_Diaphoretickes-Omar_7056_OG2834
0.4

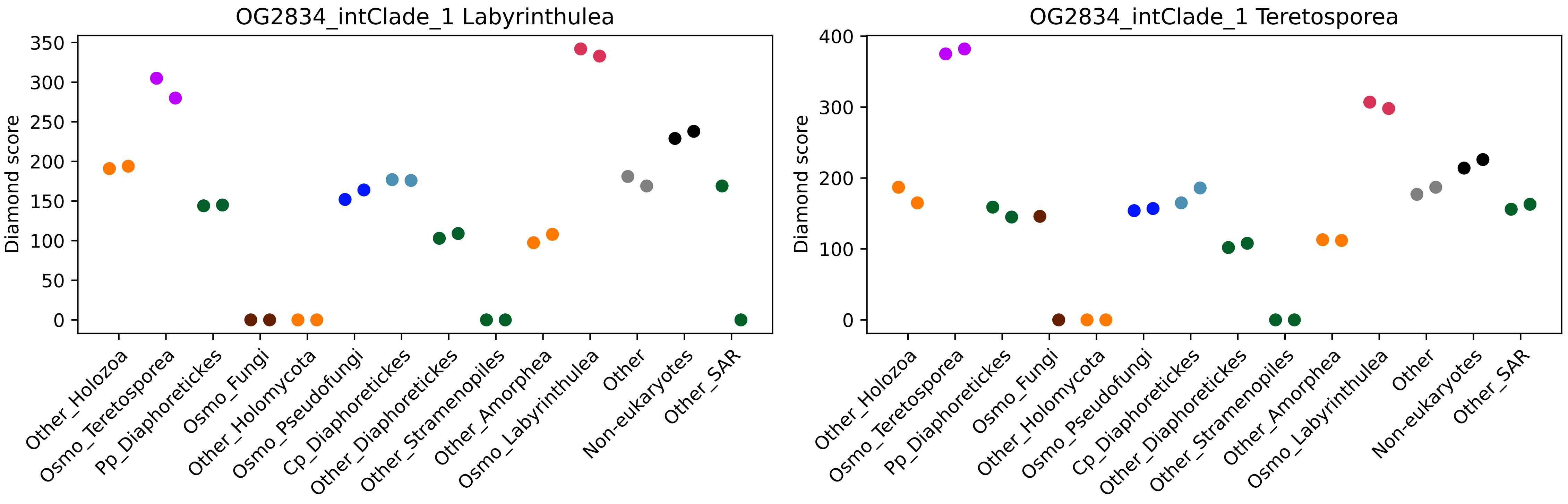

OG2834_intClade_1 Labyrinthulea
Diamond score
0
50
100
150
200
250
300
350
Other_Holozoa
Osmo_Teretosporea
Pp_Diaphoretickes
Osmo_Fungi
Other_Holomycota
Osmo_Pseudofungi
Cp_Diaphoretickes
Other_Diaphoretickes
Other_Stramenopiles
Other_Amorphea
Osmo_Labyrinthulea
Other
Non-eukaryotes
Other_SAR
OG2834_intClade_1 Teretosporea
Diamond score
0
100
200
300
400
Other_Holozoa
Osmo_Teretosporea
Pp_Diaphoretickes
Osmo_Fungi
Other_Holomycota
Osmo_Pseudofungi
Cp_Diaphoretickes
Other_Diaphoretickes
Other_Stramenopiles
Other_Amorphea
Osmo_Labyrinthulea
Other
Non-eukaryotes
Other_SAR

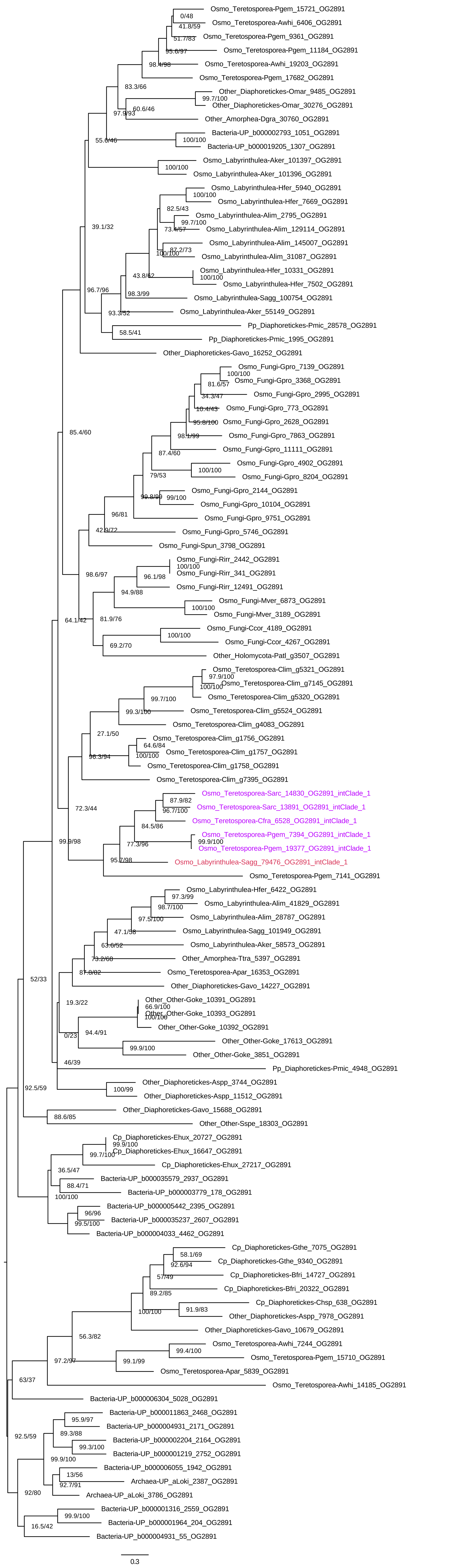

Osmo_Teretosporea-Pgem_15721_OG2891
0/48
Osmo_Teretosporea-Awhi_6406_OG2891
41.8/59
Osmo_Teretosporea-Pgem_9361_OG2891
51.7/83
Osmo_Teretosporea-Pgem_11184_OG2891
95.6/97
Osmo_Teretosporea-Awhi_19203_OG2891
98.4/98
Osmo_Teretosporea-Pgem_17682_OG2891
83.3/66
Other_Diaphoretickes-Omar_9485_OG2891
99.7/100
Other_Diaphoretickes-Omar_30276_OG2891
60.6/46
97.9/93
Other_Amorphea-Dgra_30760_OG2891
Bacteria-UP_b000002793_1051_OG2891
55.6/46
100/100
Bacteria-UP_b000019205_1307_OG2891
Osmo_Labyrinthulea-Aker_101397_OG2891
100/100
Osmo_Labyrinthulea-Aker_101396_OG2891
Osmo_Labyrinthulea-Hfer_5940_OG2891
100/100
Osmo_Labyrinthulea-Hfer_7669_OG2891
82.5/43
Osmo_Labyrinthulea-Alim_2795_OG2891
39.1/32
99.7/100
73.4/57
Osmo_Labyrinthulea-Alim_129114_OG2891
Osmo_Labyrinthulea-Alim_145007_OG2891
87.2/73
100/100
Osmo_Labyrinthulea-Alim_31087_OG2891
Osmo_Labyrinthulea-Hfer_10331_OG2891
43.8/62
100/100
Osmo_Labyrinthulea-Hfer_7502_OG2891
96.7/96
98.3/99
Osmo_Labyrinthulea-Sagg_100754_OG2891
93.3/52
Osmo_Labyrinthulea-Aker_55149_OG2891
Pp_Diaphoretickes-Pmic_28578_OG2891
58.5/41
Pp_Diaphoretickes-Pmic_1995_OG2891
Other_Diaphoretickes-Gavo_16252_OG2891
Osmo_Fungi-Gpro_7139_OG2891
100/100
Osmo_Fungi-Gpro_3368_OG2891
81.6/57
Osmo_Fungi-Gpro_2995_OG2891
34.3/47
Osmo_Fungi-Gpro_773_OG2891
10.4/43
Osmo_Fungi-Gpro_2628_OG2891
95.8/100
85.4/60
98.1/99
Osmo_Fungi-Gpro_7863_OG2891
87.4/60
Osmo_Fungi-Gpro_11111_OG2891
Osmo_Fungi-Gpro_4902_OG2891
100/100
79/53
Osmo_Fungi-Gpro_8204_OG2891
Osmo_Fungi-Gpro_2144_OG2891
99.8/99
99/100
Osmo_Fungi-Gpro_10104_OG2891
96/81
Osmo_Fungi-Gpro_9751_OG2891
42.9/72
Osmo_Fungi-Gpro_5746_OG2891
Osmo_Fungi-Spun_3798_OG2891
Osmo_Fungi-Rirr_2442_OG2891
100/100
Osmo_Fungi-Rirr_341_OG2891
98.6/97
96.1/98
Osmo_Fungi-Rirr_12491_OG2891
94.9/88
Osmo_Fungi-Mver_6873_OG2891
100/100
Osmo_Fungi-Mver_3189_OG2891
81.9/76
64.1/42
Osmo_Fungi-Ccor_4189_OG2891
100/100
Osmo_Fungi-Ccor_4267_OG2891
69.2/70
Other_Holomycota-Patl_g3507_OG2891
Osmo_Teretosporea-Clim_g5321_OG2891
97.9/100
Osmo_Teretosporea-Clim_g7145_OG2891
100/100
99.7/100
Osmo_Teretosporea-Clim_g5320_OG2891
99.3/100
Osmo_Teretosporea-Clim_g5524_OG2891
Osmo_Teretosporea-Clim_g4083_OG2891
27.1/50
Osmo_Teretosporea-Clim_g1756_OG2891
64.6/84
Osmo_Teretosporea-Clim_g1757_OG2891
100/100
98.3/94
Osmo_Teretosporea-Clim_g1758_OG2891
Osmo_Teretosporea-Clim_g7395_OG2891
Osmo_Teretosporea-Sarc_14830_OG2891_intClade_1
87.9/82
Osmo_Teretosporea-Sarc_13891_OG2891_intClade_1
72.3/44
96.7/100
Osmo_Teretosporea-Cfra_6528_OG2891_intClade_1
84.5/86
Osmo_Teretosporea-Pgem_7394_OG2891_intClade_1
99.9/98
99.9/100
77.3/96
Osmo_Teretosporea-Pgem_19377_OG2891_intClade_1
95.7/98
Osmo_Labyrinthulea-Sagg_79476_OG2891_intClade_1
Osmo_Teretosporea-Pgem_7141_OG2891
Osmo_Labyrinthulea-Hfer_6422_OG2891
97.3/99
Osmo_Labyrinthulea-Alim_41829_OG2891
98.7/100
97.5/100
Osmo_Labyrinthulea-Alim_28787_OG2891
47.1/58
Osmo_Labyrinthulea-Sagg_101949_OG2891
63.6/52
Osmo_Labyrinthulea-Aker_58573_OG2891
73.2/68
Other_Amorphea-Ttra_5397_OG2891
87.8/82
Osmo_Teretosporea-Apar_16353_OG2891
52/33
Other_Diaphoretickes-Gavo_14227_OG2891
Other_Other-Goke_10391_OG2891
19.3/22
66.9/100
Other_Other-Goke_10393_OG2891
100/100
Other_Other-Goke_10392_OG2891
0/23
94.4/91
Other_Other-Goke_17613_OG2891
99.9/100
Other_Other-Goke_3851_OG2891
46/39
Pp_Diaphoretickes-Pmic_4948_OG2891
Other_Diaphoretickes-Aspp_3744_OG2891
92.5/59
100/99
Other_Diaphoretickes-Aspp_11512_OG2891
Other_Diaphoretickes-Gavo_15688_OG2891
88.6/85
Other_Other-Sspe_18303_OG2891
Cp_Diaphoretickes-Ehux_20727_OG2891
99.9/100
99.7/100
Cp_Diaphoretickes-Ehux_16647_OG2891
36.5/47
Cp_Diaphoretickes-Ehux_27217_OG2891
Bacteria-UP_b000035579_2937_OG2891
88.4/71
100/100
Bacteria-UP_b000003779_178_OG2891
Bacteria-UP_b000005442_2395_OG2891
96/96
99.5/100
Bacteria-UP_b000035237_2607_OG2891
Bacteria-UP_b000004033_4462_OG2891
Cp_Diaphoretickes-Gthe_7075_OG2891
58.1/69
Cp_Diaphoretickes-Gthe_9340_OG2891
92.6/94
57/49
Cp_Diaphoretickes-Bfri_14727_OG2891
89.2/85
Cp_Diaphoretickes-Bfri_20322_OG2891
100/100
Cp_Diaphoretickes-Chsp_638_OG2891
91.9/83
Other_Diaphoretickes-Aspp_7978_OG2891
Other_Diaphoretickes-Gavo_10679_OG2891
56.3/82
Osmo_Teretosporea-Awhi_7244_OG2891
99.4/100
97.2/97
99.1/99
Osmo_Teretosporea-Pgem_15710_OG2891
63/37
Osmo_Teretosporea-Apar_5839_OG2891
Osmo_Teretosporea-Awhi_14185_OG2891
Bacteria-UP_b000006304_5028_OG2891
Bacteria-UP_b000011863_2468_OG2891
95.9/97
Bacteria-UP_b000004931_2171_OG2891
92.5/59
89.3/88
Bacteria-UP_b000002204_2164_OG2891
99.3/100
Bacteria-UP_b000001219_2752_OG2891
99.9/100
Bacteria-UP_b000006055_1942_OG2891
13/56
Archaea-UP_aLoki_2387_OG2891
92.7/91
92/80
Archaea-UP_aLoki_3786_OG2891
Bacteria-UP_b000001316_2559_OG2891
99.9/100
Bacteria-UP_b000001964_204_OG2891
16.5/42
Bacteria-UP_b000004931_55_OG2891
0.3

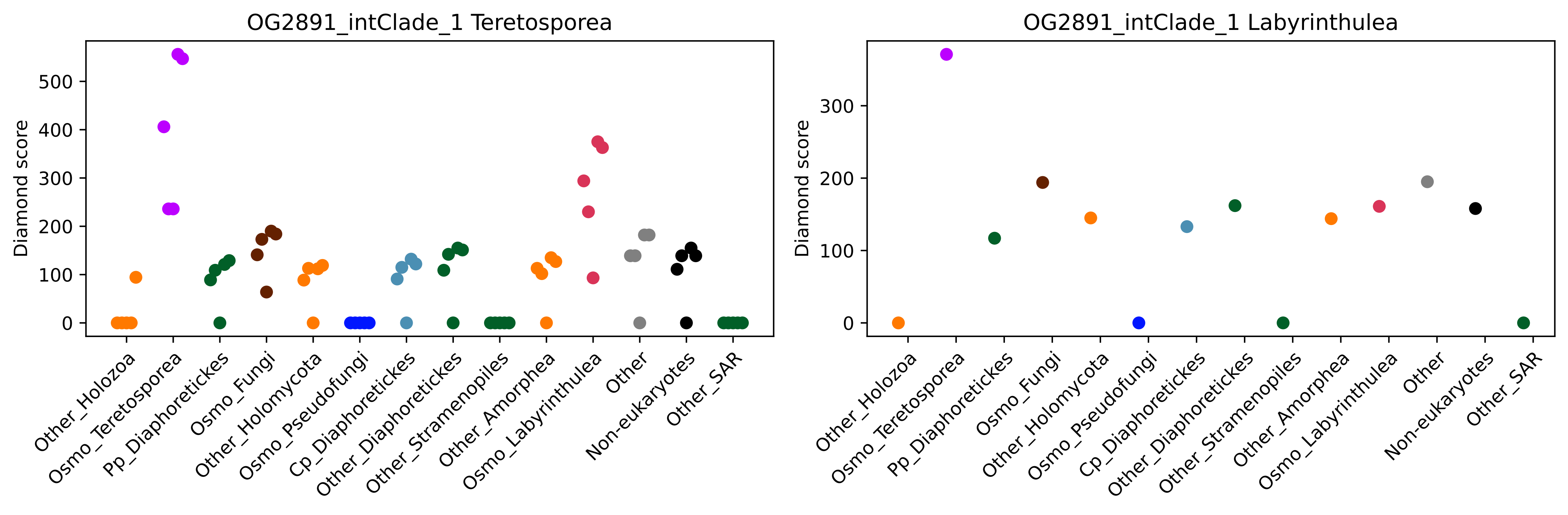
OG2891_intClade_1 Teretosporea
OG2891_intClade_1 Labyrinthulea
Diamond score
0
100
200
300
400
500
Other_Holozoa
Osmo_Teretosporea
Pp_Diaphoretickes
Osmo_Fungi
Other_Holomycota
Osmo_Pseudofungi
Cp_Diaphoretickes
Other_Diaphoretickes
Other_Stramenopiles
Other_Amorphea
Osmo_Labyrinthulea
Other
Non-eukaryotes
Other_SAR

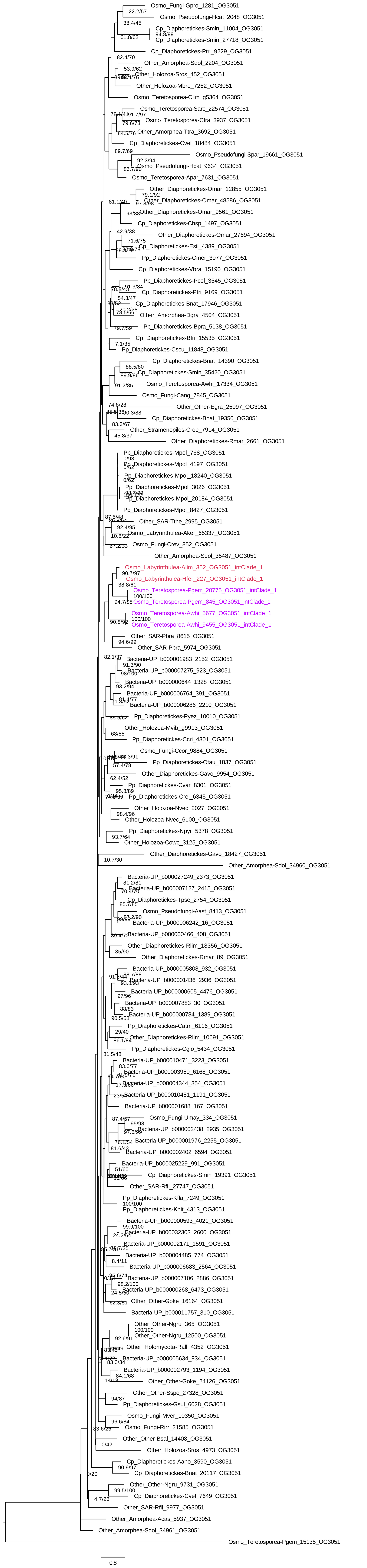
Osmo_Fungi-Gpro_1281_OG3051
Osmo_Pseudofungi-Hcat_2048_OG3051
Cp_Diaphoretickes-Smin_11004_OG3051
Cp_Diaphoretickes-Smin_27718_OG3051
Cp_Diaphoretickes-Ptri_9229_OG3051
Other_Amorphea-Sdol_2204_OG3051
Other_Holozoa-Sros_452_OG3051
Other_Holozoa-Mbre_7262_OG3051
Osmo_Teretosporea-Clim_g5364_OG3051
Osmo_Teretosporea-Sarc_22574_OG3051
Osmo_Teretosporea-Cfra_3937_OG3051
Other_Amorphea-Ttra_3692_OG3051
Cp_Diaphoretickes-Cvel_18484_OG3051
Osmo_Pseudofungi-Spar_19661_OG3051
Osmo_Pseudofungi-Hcat_9634_OG3051
Osmo_Teretosporea-Apar_7631_OG3051
Other_Diaphoretickes-Omar_12855_OG3051
Other_Diaphoretickes-Omar_48586_OG3051
Other_Diaphoretickes-Omar_9561_OG3051
Cp_Diaphoretickes-Chsp_1497_OG3051
Other_Diaphoretickes-Omar_27694_OG3051
Cp_Diaphoretickes-Esil_4389_OG3051
Pp_Diaphoretickes-Cmer_3977_OG3051
Cp_Diaphoretickes-Vbra_15190_OG3051
Pp_Diaphoretickes-Pcol_3545_OG3051
Cp_Diaphoretickes-Ptri_9169_OG3051
Cp_Diaphoretickes-Bnat_17946_OG3051
Other_Amorphea-Dgra_4504_OG3051
Pp_Diaphoretickes-Bpra_5138_OG3051
Cp_Diaphoretickes-Bfri_15535_OG3051
Pp_Diaphoretickes-Cscu_11848_OG3051
Cp_Diaphoretickes-Bnat_14390_OG3051
Cp_Diaphoretickes-Smin_35420_OG3051
Osmo_Teretosporea-Awhi_17334_OG3051
Osmo_Fungi-Cang_7845_OG3051
Other_Other-Egra_25097_OG3051
Cp_Diaphoretickes-Bnat_19350_OG3051
Other_Stramenopiles-Croe_7914_OG3051
Other_Diaphoretickes-Rmar_2661_OG3051
Pp_Diaphoretickes-Mpol_768_OG3051
Pp_Diaphoretickes-Mpol_4197_OG3051
Pp_Diaphoretickes-Mpol_18240_OG3051
Pp_Diaphoretickes-Mpol_3026_OG3051
Pp_Diaphoretickes-Mpol_20184_OG3051
Pp_Diaphoretickes-Mpol_8427_OG3051
Other_SAR-Tthe_2995_OG3051
Osmo_Labyrinthulea-Aker_65337_OG3051
Osmo_Fungi-Crev_852_OG3051
Other_Amorphea-Sdol_35487_OG3051
Osmo_Labyrinthulea-Alim_352_OG3051_intClade_1
Osmo_Labyrinthulea-Hfer_227_OG3051_intClade_1
Osmo_Teretosporea-Pgem_20775_OG3051_intClade_1
Osmo_Teretosporea-Pgem_845_OG3051_intClade_1
Osmo_Teretosporea-Awhi_5677_OG3051_intClade_1
Osmo_Teretosporea-Awhi_9455_OG3051_intClade_1
Other_SAR-Pbra_8615_OG3051
Other_SAR-Pbra_5974_OG3051
Bacteria-UP_b000001983_2152_OG3051
Bacteria-UP_b000007275_923_OG3051
Bacteria-UP_b000000644_1328_OG3051
Bacteria-UP_b000006764_391_OG3051
Bacteria-UP_b000006286_2210_OG3051
Pp_Diaphoretickes-Pyez_10010_OG3051
Other_Holozoa-Mvib_g9913_OG3051
Pp_Diaphoretickes-Ccri_4301_OG3051
Osmo_Fungi-Ccor_9884_OG3051
Pp_Diaphoretickes-Otau_1837_OG3051
Other_Diaphoretickes-Gavo_9954_OG3051
Pp_Diaphoretickes-Cvar_8301_OG3051
Pp_Diaphoretickes-Crei_6345_OG3051
Other_Holozoa-Nvec_2027_OG3051
Other_Holozoa-Nvec_6100_OG3051
Pp_Diaphoretickes-Npyr_5378_OG3051
Other_Holozoa-Cowc_3125_OG3051
Other_Diaphoretickes-Gavo_18427_OG3051
Other_Amorphea-Sdol_34960_OG3051
Bacteria-UP_b000027249_2373_OG3051
Bacteria-UP_b000007127_2415_OG3051
Cp_Diaphoretickes-Tpse_2754_OG3051
Osmo_Pseudofungi-Aast_8413_OG3051
Bacteria-UP_b000006242_16_OG3051
Bacteria-UP_b000000466_408_OG3051
Other_Diaphoretickes-Rlim_18356_OG3051
Other_Diaphoretickes-Rmar_89_OG3051
Bacteria-UP_b000005808_932_OG3051
Bacteria-UP_b000001436_2936_OG3051
Bacteria-UP_b000000605_4476_OG3051
Bacteria-UP_b000007883_30_OG3051
Bacteria-UP_b000000784_1389_OG3051
Pp_Diaphoretickes-Catm_6116_OG3051
Other_Diaphoretickes-Rlim_10691_OG3051
Pp_Diaphoretickes-Cglo_5434_OG3051
Bacteria-UP_b000010471_3223_OG3051
Bacteria-UP_b000003959_6168_OG3051
Bacteria-UP_b000004344_354_OG3051
Bacteria-UP_b000010481_1191_OG3051
Bacteria-UP_b000001688_167_OG3051
Osmo_Fungi-Umay_334_OG3051
Bacteria-UP_b000002438_2935_OG3051
Bacteria-UP_b000001976_2255_OG3051
Bacteria-UP_b000002402_6594_OG3051
Bacteria-UP_b000025229_991_OG3051
Cp_Diaphoretickes-Smin_19391_OG3051
Other_SAR-Rfil_27747_OG3051
Pp_Diaphoretickes-Kfla_7249_OG3051
Pp_Diaphoretickes-Knit_4313_OG3051
Bacteria-UP_b000000593_4021_OG3051
Bacteria-UP_b000032303_2600_OG3051
Bacteria-UP_b000002171_1591_OG3051
Bacteria-UP_b000004485_774_OG3051
Bacteria-UP_b000006683_2564_OG3051
Bacteria-UP_b000007106_2886_OG3051
Bacteria-UP_b000000268_6473_OG3051
Other_Other-Goke_16164_OG3051
Bacteria-UP_b000011757_310_OG3051
Other_Other-Ngru_365_OG3051
Other_Other-Ngru_12500_OG3051
Other_Holomycota-Rall_4352_OG3051
Bacteria-UP_b000005634_934_OG3051
Bacteria-UP_b000002793_1194_OG3051
Other_Other-Goke_24126_OG3051
Other_Other-Sspe_27328_OG3051
Pp_Diaphoretickes-Gsul_6028_OG3051
Osmo_Fungi-Mver_10350_OG3051
Osmo_Fungi-Rirr_21585_OG3051
Other_Other-Bsal_14408_OG3051
Other_Holozoa-Sros_4973_OG3051
Cp_Diaphoretickes-Aano_3590_OG3051
Cp_Diaphoretickes-Bnat_20117_OG3051
Other_Other-Ngru_9731_OG3051
Cp_Diaphoretickes-Cvel_7649_OG3051
Other_SAR-Rfil_9977_OG3051
Other_Amorphea-Acas_5937_OG3051
Other_Amorphea-Sdol_34961_OG3051
Osmo_Teretosporea-Pgem_15135_OG3051
0.8

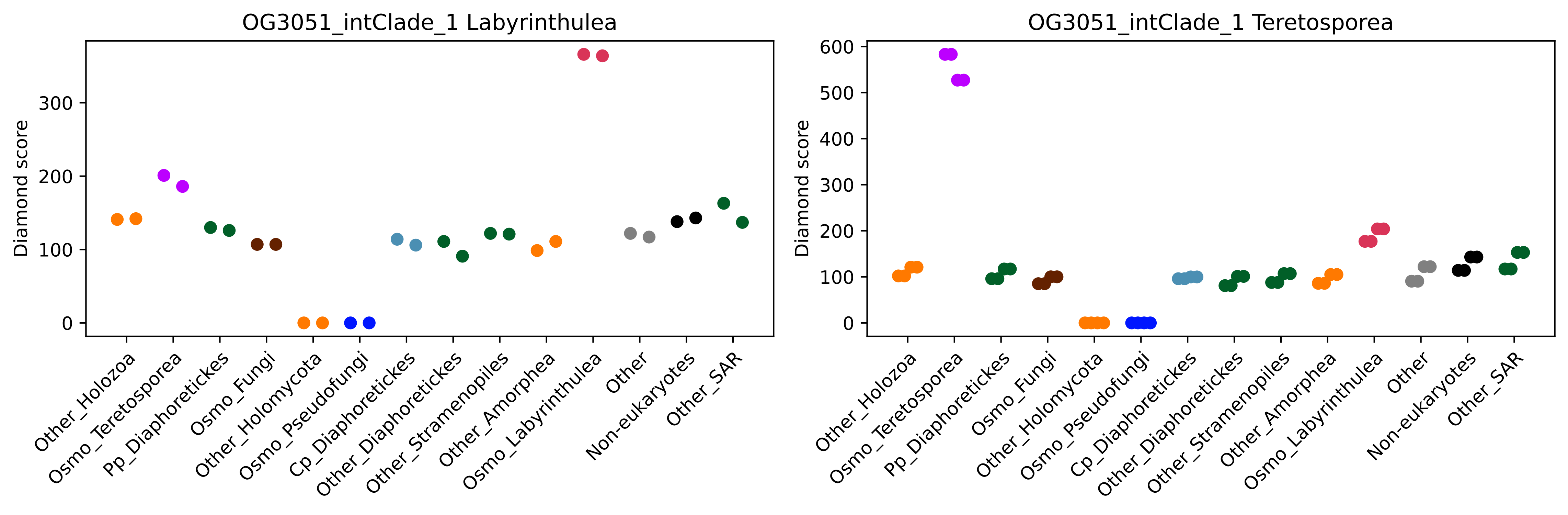

OG3051_intClade_1 Labyrinthulea
OG3051_intClade_1 Teretosporea
Diamond score
0
100
200
300
400
500
600
Other_Holozoa
Osmo_Teretosporea
Pp_Diaphoretickes
Osmo_Fungi
Other_Holomycota
Osmo_Pseudofungi
Cp_Diaphoretickes
Other_Diaphoretickes
Other_Stramenopiles
Other_Amorphea
Osmo_Labyrinthulea
Other
Non-eukaryotes
Other_SAR

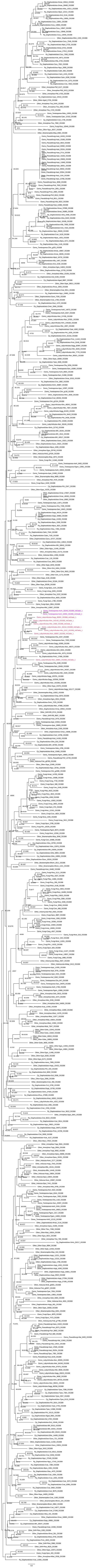

Pp_Diaphoretickes-Bpra_2494_OG308
Pp_Diaphoretickes-Otau_4475_OG308
Pp_Diaphoretickes-Cvar_1554_OG308
Pp_Diaphoretickes-Cvar_1555_OG308
Pp_Diaphoretickes-Cvar_3372_OG308
Pp_Diaphoretickes-Cvar_9396_OG308
Pp_Diaphoretickes-Cvar_9464_OG308
Pp_Diaphoretickes-Cvar_9679_OG308
Other_Other-Sspe_14826_OG308
Other_Stramenopiles-Croe_1189_OG308
Other_Stramenopiles-Bhom_104_OG308
Other_Stramenopiles-Bhom_5964_OG308
Cp_Diaphoretickes-Cvel_14877_OG308
Cp_Diaphoretickes-Cvel_27348_OG308
Cp_Diaphoretickes-Vbra_191_OG308
Other_Diaphoretickes-Omar_34883_OG308
Cp_Diaphoretickes-Dspp_49981_OG308
Cp_Diaphoretickes-Bfri_30547_OG308
Osmo_Pseudofungi-Hcat_12336_OG308
Other_SAR-Pbra_1283_OG308
Other_Diaphoretickes-Bmot_351_OG308
Cp_Diaphoretickes-Bnat_3740_OG308
Pp_Diaphoretickes-Pmic_4820_OG308
Other_SAR-Pbra_2988_OG308
Other_SAR-Pbra_8525_OG308
Other_Other-Egra_12973_OG308
Osmo_Fungi-Gpro_453_OG308
Other_Amorphea-Mbal_2038_OG308
Pp_Diaphoretickes-Vcar_13361_OG308
0.3

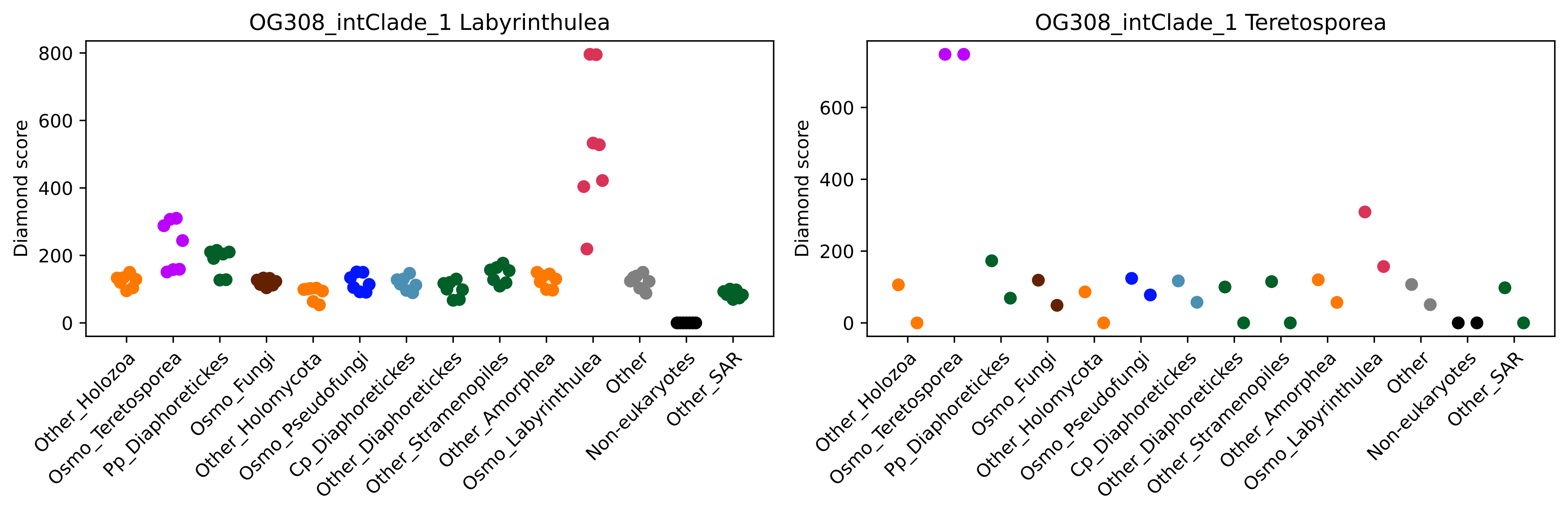
OG308_intClade_1 Labyrinthulea
Diamond score
0
200
400
600
800
Other_Holozoa
Osmo_Teretosporea
Pp_Diaphoretickes
Osmo_Fungi
Other_Holomycota
Osmo_Pseudofungi
Cp_Diaphoretickes
Other_Diaphoretickes
Other_Stramenopiles
Other_Amorphea
Osmo_Labyrinthulea
Other
Non-eukaryotes
Other_SAR
OG308_intClade_1 Teretosporea
Diamond score
0
200
400
600
Other_Holozoa
Osmo_Teretosporea
Pp_Diaphoretickes
Osmo_Fungi
Other_Holomycota
Osmo_Pseudofungi
Cp_Diaphoretickes
Other_Diaphoretickes
Other_Stramenopiles
Other_Amorphea
Osmo_Labyrinthulea
Other
Non-eukaryotes
Other_SAR

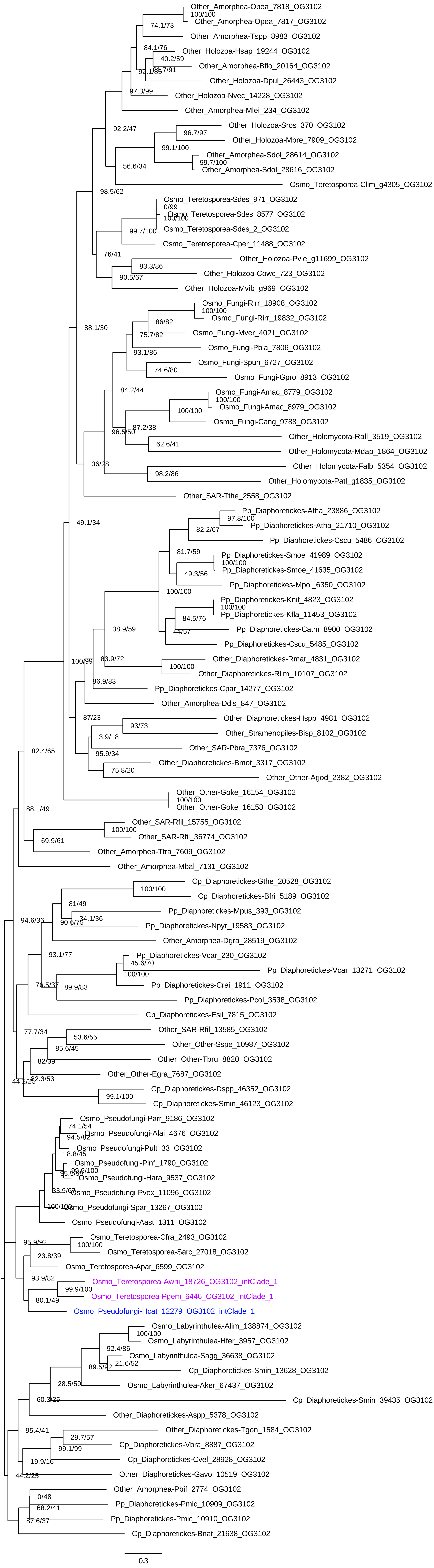

Other_Amorphea-Opea_7818_OG3102
100/100
Other_Amorphea-Opea_7817_OG3102
74.1/73
Other_Amorphea-Tspp_8983_OG3102
84.1/76
Other_Holozoa-Hsap_19244_OG3102
40.2/59
Other_Amorphea-Bflo_20164_OG3102
92.1/85
81.7/91
Other_Holozoa-Dpul_26443_OG3102
97.3/99
Other_Holozoa-Nvec_14228_OG3102
Other_Amorphea-Mlei_234_OG3102
92.2/47
Other_Holozoa-Sros_370_OG3102
96.7/97
Other_Holozoa-Mbre_7909_OG3102
99.1/100
Other_Amorphea-Sdol_28614_OG3102
99.7/100
Other_Amorphea-Sdol_28616_OG3102
56.6/34
Osmo_Teretosporea-Clim_g4305_OG3102
98.5/62
Osmo_Teretosporea-Sdes_971_OG3102
0/99
Osmo_Teretosporea-Sdes_8577_OG3102
100/100
99.7/100
Osmo_Teretosporea-Sdes_2_OG3102
Osmo_Teretosporea-Cper_11488_OG3102
76/41
Other_Holozoa-Pvie_g11699_OG3102
83.3/86
Other_Holozoa-Cowc_723_OG3102
90.5/67
Other_Holozoa-Mvib_g969_OG3102
Osmo_Fungi-Rirr_18908_OG3102
100/100
Osmo_Fungi-Rirr_19832_OG3102
88.1/30
86/82
75.7/82
Osmo_Fungi-Mver_4021_OG3102
93.1/86
Osmo_Fungi-Pbla_7806_OG3102
Osmo_Fungi-Spun_6727_OG3102
74.6/80
Osmo_Fungi-Gpro_8913_OG3102
84.2/44
Osmo_Fungi-Amac_8779_OG3102
100/100
Osmo_Fungi-Amac_8979_OG3102
100/100
Osmo_Fungi-Cang_9788_OG3102
87.2/38
96.5/50
Other_Holomycota-Rall_3519_OG3102
62.6/41
Other_Holomycota-Mdap_1864_OG3102
36/28
Other_Holomycota-Falb_5354_OG3102
98.2/86
Other_Holomycota-Patl_g1835_OG3102
Other_SAR-Tthe_2558_OG3102
49.1/34
Pp_Diaphoretickes-Atha_23886_OG3102
97.8/100
Pp_Diaphoretickes-Atha_21710_OG3102
82.2/67
Pp_Diaphoretickes-Cscu_5486_OG3102
81.7/59
Pp_Diaphoretickes-Smoe_41989_OG3102
100/100
Pp_Diaphoretickes-Smoe_41635_OG3102
49.3/56
Pp_Diaphoretickes-Mpol_6350_OG3102
100/100
Pp_Diaphoretickes-Knit_4823_OG3102
100/100
Pp_Diaphoretickes-Kfla_11453_OG3102
84.5/76
44/57
Pp_Diaphoretickes-Catm_8900_OG3102
38.9/59
Pp_Diaphoretickes-Cscu_5485_OG3102
83.9/72
Other_Diaphoretickes-Rmar_4831_OG3102
100/99
100/100
Other_Diaphoretickes-Rlim_10107_OG3102
86.9/83
Pp_Diaphoretickes-Cpar_14277_OG3102
Other_Amorphea-Ddis_847_OG3102
87/23
Other_Diaphoretickes-Hspp_4981_OG3102
93/73
Other_Stramenopiles-Bisp_8102_OG3102
3.9/18
Other_SAR-Pbra_7376_OG3102
95.9/34
Other_Diaphoretickes-Bmot_3317_OG3102
75.8/20
Other_Other-Agod_2382_OG3102
82.4/65
Other_Other-Goke_16154_OG3102
100/100
Other_Other-Goke_16153_OG3102
88.1/49
Other_SAR-Rfil_15755_OG3102
100/100
Other_SAR-Rfil_36774_OG3102
69.9/61
Other_Amorphea-Ttra_7609_OG3102
Other_Amorphea-Mbal_7131_OG3102
Cp_Diaphoretickes-Gthe_20528_OG3102
100/100
Cp_Diaphoretickes-Bfri_5189_OG3102
81/49
Pp_Diaphoretickes-Mpus_393_OG3102
94.6/36
34.1/36
90.6/75
Pp_Diaphoretickes-Npyr_19583_OG3102
Other_Amorphea-Dgra_28519_OG3102
93.1/77
Pp_Diaphoretickes-Vcar_230_OG3102
45.6/70
Pp_Diaphoretickes-Vcar_13271_OG3102
100/100
76.5/37
89.9/83
Pp_Diaphoretickes-Crei_1911_OG3102
Pp_Diaphoretickes-Pcol_3538_OG3102
Cp_Diaphoretickes-Esil_7815_OG3102
77.7/34
Other_SAR-Rfil_13585_OG3102
53.6/55
Other_Other-Sspe_10987_OG3102
85.6/45
82/39
Other_Other-Tbru_8820_OG3102
44.2/25
82.3/53
Other_Other-Egra_7687_OG3102
Cp_Diaphoretickes-Dspp_46352_OG3102
99.1/100
Cp_Diaphoretickes-Smin_46123_OG3102
Osmo_Pseudofungi-Parr_9186_OG3102
74.1/54
94.5/82
Osmo_Pseudofungi-Alai_4676_OG3102
18.8/45
Osmo_Pseudofungi-Pult_33_OG3102
99.9/100
Osmo_Pseudofungi-Pinf_1790_OG3102
95.5/89
Osmo_Pseudofungi-Hara_9537_OG3102
33.9/67
Osmo_Pseudofungi-Pvex_11096_OG3102
100/100
Osmo_Pseudofungi-Spar_13267_OG3102
Osmo_Pseudofungi-Aast_1311_OG3102
95.9/92
Osmo_Teretosporea-Cfra_2493_OG3102
100/100
Osmo_Teretosporea-Sarc_27018_OG3102
23.8/39
Osmo_Teretosporea-Apar_6599_OG3102
93.9/82
Osmo_Teretosporea-Awhi_18726_OG3102_intClade_1
99.9/100
Osmo_Teretosporea-Pgem_6446_OG3102_intClade_1
80.1/49
Osmo_Pseudofungi-Hcat_12279_OG3102_intClade_1
Osmo_Labyrinthulea-Alim_138874_OG3102
100/100
Osmo_Labyrinthulea-Hfer_3957_OG3102
92.4/86
Osmo_Labyrinthulea-Sagg_36638_OG3102
21.6/52
89.5/92
Cp_Diaphoretickes-Smin_13628_OG3102
28.5/59
Osmo_Labyrinthulea-Aker_67437_OG3102
60.3/25
Cp_Diaphoretickes-Smin_39435_OG3102
Other_Diaphoretickes-Aspp_5378_OG3102
95.4/41
Other_Diaphoretickes-Tgon_1584_OG3102
29.7/57
Cp_Diaphoretickes-Vbra_8887_OG3102
99.1/99
19.9/16
Cp_Diaphoretickes-Cvel_28928_OG3102
44.2/25
Other_Diaphoretickes-Gavo_10519_OG3102
Other_Amorphea-Pbif_2774_OG3102
0/48
Pp_Diaphoretickes-Pmic_10909_OG3102
68.2/41
Pp_Diaphoretickes-Pmic_10910_OG3102
87.6/37
Cp_Diaphoretickes-Bnat_21638_OG3102
0.3

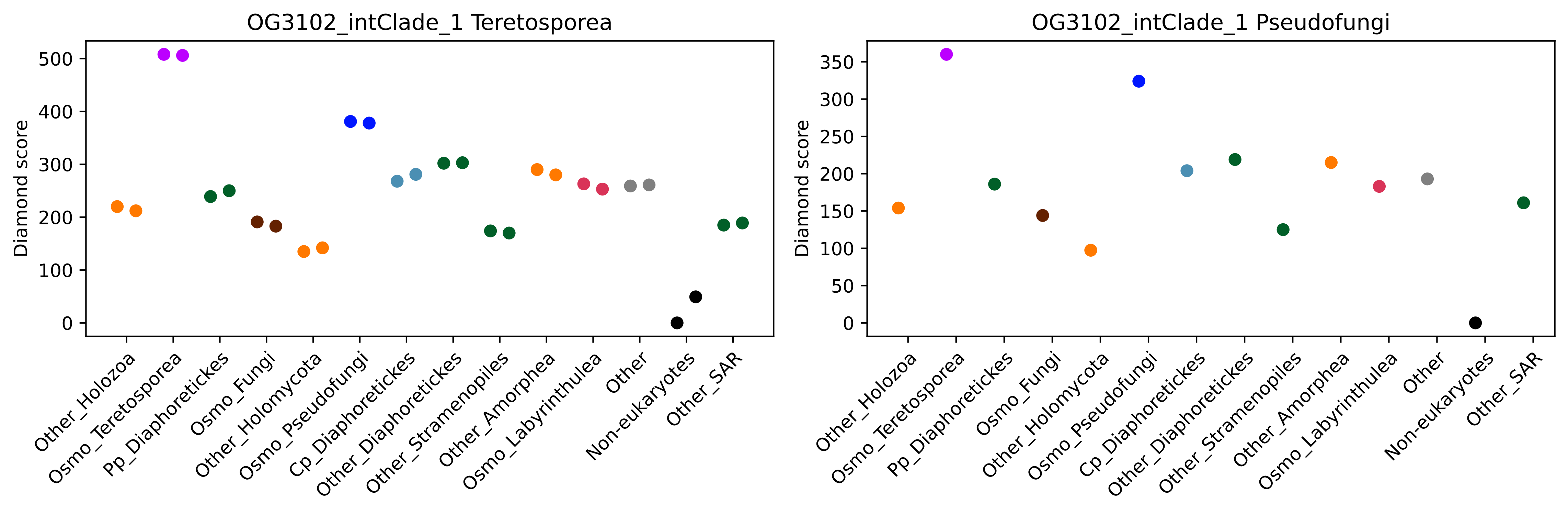
OG3102_intClade_1 Teretosporea
Diamond score
0
100
200
300
400
500
Other_Holozoa
Osmo_Teretosporea
Pp_Diaphoretickes
Osmo_Fungi
Other_Holomycota
Osmo_Pseudofungi
Cp_Diaphoretickes
Other_Diaphoretickes
Other_Stramenopiles
Other_Amorphea
Osmo_Labyrinthulea
Other
Non-eukaryotes
Other_SAR
OG3102_intClade_1 Pseudofungi
Diamond score
0
50
100
150
200
250
300
350
Other_Holozoa
Osmo_Teretosporea
Pp_Diaphoretickes
Osmo_Fungi
Other_Holomycota
Osmo_Pseudofungi
Cp_Diaphoretickes
Other_Diaphoretickes
Other_Stramenopiles
Other_Amorphea
Osmo_Labyrinthulea
Other
Non-eukaryotes
Other_SAR

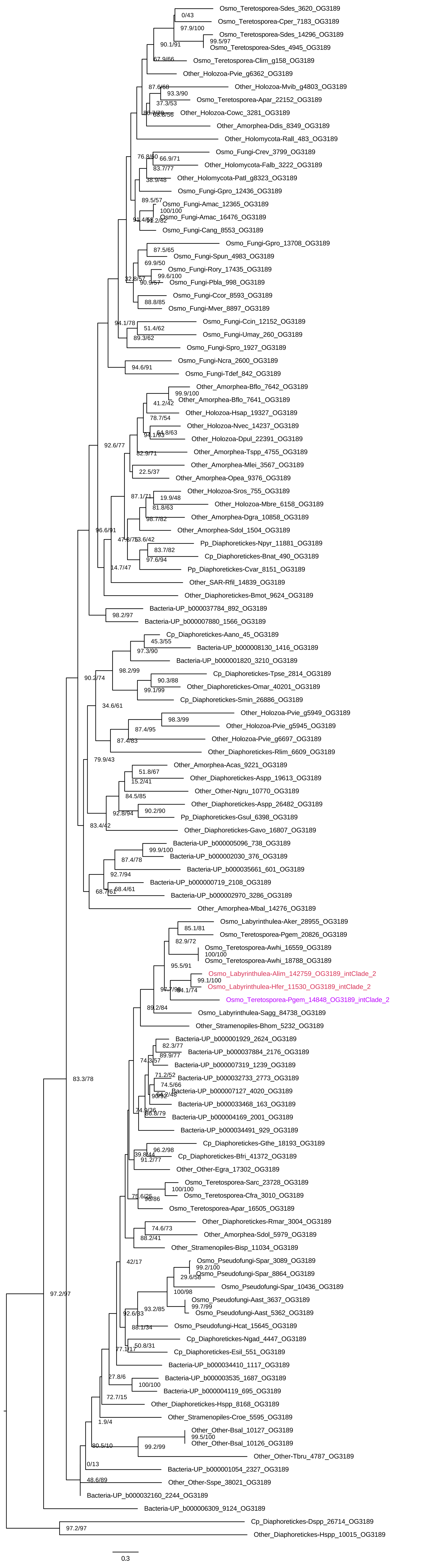
Osmo_Teretosporea-Sdes_3620_OG3189
Osmo_Teretosporea-Cper_7183_OG3189
Osmo_Teretosporea-Sdes_14296_OG3189
Osmo_Teretosporea-Sdes_4945_OG3189
Osmo_Teretosporea-Clim_g158_OG3189
Other_Holozoa-Pvie_g6362_OG3189
Other_Holozoa-Mvib_g4803_OG3189
Osmo_Teretosporea-Apar_22152_OG3189
Other_Holozoa-Cowc_3281_OG3189
Other_Amorphea-Ddis_8349_OG3189
Other_Holomycota-Rall_483_OG3189
Osmo_Fungi-Crev_3799_OG3189
Other_Holomycota-Falb_3222_OG3189
Other_Holomycota-Patl_g8323_OG3189
Osmo_Fungi-Gpro_12436_OG3189
Osmo_Fungi-Amac_12365_OG3189
Osmo_Fungi-Amac_16476_OG3189
Osmo_Fungi-Cang_8553_OG3189
Osmo_Fungi-Gpro_13708_OG3189
Osmo_Fungi-Spun_4983_OG3189
Osmo_Fungi-Rory_17435_OG3189
Osmo_Fungi-Pbla_998_OG3189
Osmo_Fungi-Ccor_8593_OG3189
Osmo_Fungi-Mver_8897_OG3189
Osmo_Fungi-Ccin_12152_OG3189
Osmo_Fungi-Umay_260_OG3189
Osmo_Fungi-Spro_1927_OG3189
Osmo_Fungi-Ncra_2600_OG3189
Osmo_Fungi-Tdef_842_OG3189
Other_Amorphea-Bflo_7642_OG3189
Other_Amorphea-Bflo_7641_OG3189
Other_Holozoa-Hsap_19327_OG3189
Other_Holozoa-Nvec_14237_OG3189
Other_Holozoa-Dpul_22391_OG3189
Other_Amorphea-Tspp_4755_OG3189
Other_Amorphea-Mlei_3567_OG3189
Other_Amorphea-Opea_9376_OG3189
Other_Holozoa-Sros_755_OG3189
Other_Holozoa-Mbre_6158_OG3189
Other_Amorphea-Dgra_10858_OG3189
Other_Amorphea-Sdol_1504_OG3189
Pp_Diaphoretickes-Npyr_11881_OG3189
Cp_Diaphoretickes-Bnat_490_OG3189
Pp_Diaphoretickes-Cvar_8151_OG3189
Other_SAR-Rfil_14839_OG3189
Other_Diaphoretickes-Bmot_9624_OG3189
Bacteria-UP_b000037784_892_OG3189
Bacteria-UP_b000007880_1566_OG3189
Cp_Diaphoretickes-Aano_45_OG3189
Bacteria-UP_b000008130_1416_OG3189
Bacteria-UP_b000001820_3210_OG3189
Cp_Diaphoretickes-Tpse_2814_OG3189
Other_Diaphoretickes-Omar_40201_OG3189
Cp_Diaphoretickes-Smin_26886_OG3189
Other_Holozoa-Pvie_g5949_OG3189
Other_Holozoa-Pvie_g5945_OG3189
Other_Holozoa-Pvie_g6697_OG3189
Other_Diaphoretickes-Rlim_6609_OG3189
Other_Amorphea-Acas_9221_OG3189
Other_Diaphoretickes-Aspp_19613_OG3189
Other_Other-Ngru_10770_OG3189
Other_Diaphoretickes-Aspp_26482_OG3189
Pp_Diaphoretickes-Gsul_6398_OG3189
Other_Diaphoretickes-Gavo_16807_OG3189
Bacteria-UP_b000005096_738_OG3189
Bacteria-UP_b000002030_376_OG3189
Bacteria-UP_b000035661_601_OG3189
Bacteria-UP_b000000719_2108_OG3189
Bacteria-UP_b000002970_3286_OG3189
Other_Amorphea-Mbal_14276_OG3189
Osmo_Labyrinthulea-Aker_28955_OG3189
Osmo_Teretosporea-Pgem_20826_OG3189
Osmo_Teretosporea-Awhi_16559_OG3189
Osmo_Teretosporea-Awhi_18788_OG3189
Osmo_Labyrinthulea-Alim_142759_OG3189_intClade_2
Osmo_Labyrinthulea-Hfer_11530_OG3189_intClade_2
Osmo_Teretosporea-Pgem_14848_OG3189_intClade_2
Osmo_Labyrinthulea-Sagg_84738_OG3189
Other_Stramenopiles-Bhom_5232_OG3189
Bacteria-UP_b000001929_2624_OG3189
Bacteria-UP_b000037884_2176_OG3189
Bacteria-UP_b000007319_1239_OG3189
Bacteria-UP_b000032733_2773_OG3189
Bacteria-UP_b000007127_4020_OG3189
Bacteria-UP_b000033468_163_OG3189
Bacteria-UP_b000004169_2001_OG3189
Bacteria-UP_b000034491_929_OG3189
Cp_Diaphoretickes-Gthe_18193_OG3189
Cp_Diaphoretickes-Bfri_41372_OG3189
Other_Other-Egra_17302_OG3189
Osmo_Teretosporea-Sarc_23728_OG3189
Osmo_Teretosporea-Cfra_3010_OG3189
Osmo_Teretosporea-Apar_16505_OG3189
Other_Diaphoretickes-Rmar_3004_OG3189
Other_Amorphea-Sdol_5979_OG3189
Other_Stramenopiles-Bisp_11034_OG3189
Osmo_Pseudofungi-Spar_3089_OG3189
Osmo_Pseudofungi-Spar_8864_OG3189
Osmo_Pseudofungi-Spar_10436_OG3189
Osmo_Pseudofungi-Aast_3637_OG3189
Osmo_Pseudofungi-Aast_5362_OG3189
Osmo_Pseudofungi-Hcat_15645_OG3189
Cp_Diaphoretickes-Ngad_4447_OG3189
Cp_Diaphoretickes-Esil_551_OG3189
Bacteria-UP_b000034410_1117_OG3189
Bacteria-UP_b000003535_1687_OG3189
Bacteria-UP_b000004119_695_OG3189
Other_Diaphoretickes-Hspp_8168_OG3189
Other_Stramenopiles-Croe_5595_OG3189
Other_Other-Bsal_10127_OG3189
Other_Other-Bsal_10126_OG3189
Other_Other-Tbru_4787_OG3189
Bacteria-UP_b000001054_2327_OG3189
Other_Other-Sspe_38021_OG3189
Bacteria-UP_b000032160_2244_OG3189
Bacteria-UP_b000006309_9124_OG3189
Cp_Diaphoretickes-Dspp_26714_OG3189
Other_Diaphoretickes-Hspp_10015_OG3189
0.3

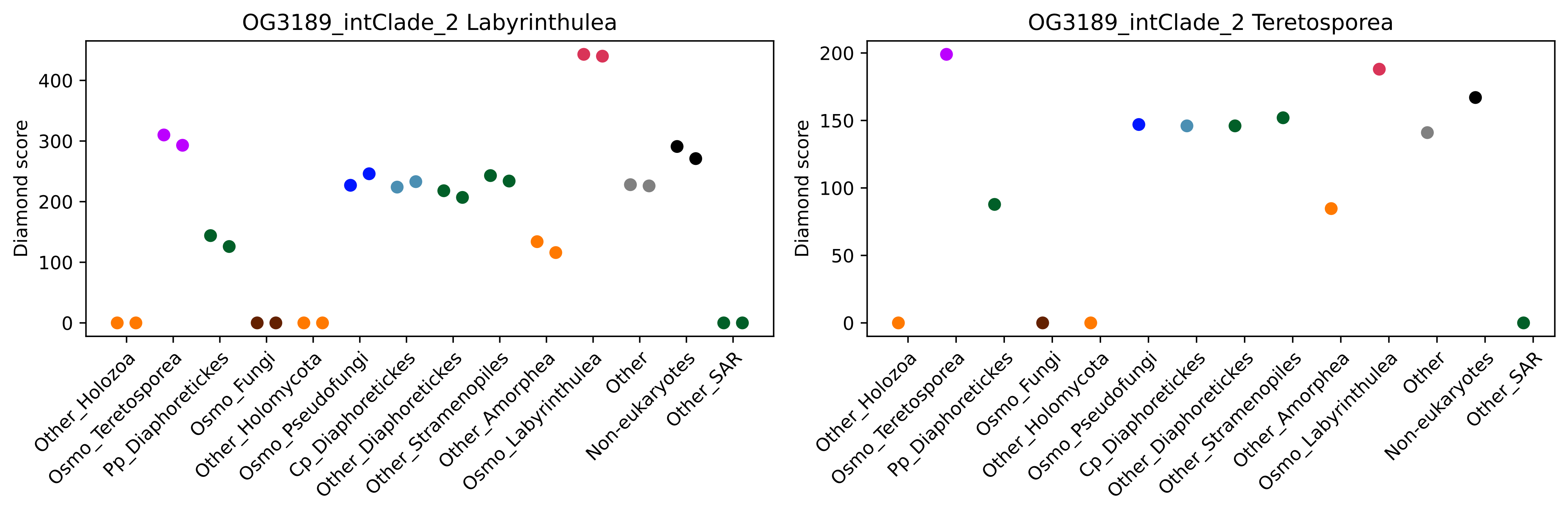
OG3189_intClade_2 Labyrinthulea
Diamond score
0
100
200
300
400
Other_Holozoa
Osmo_Teretosporea
Pp_Diaphoretickes
Osmo_Fungi
Other_Holomycota
Osmo_Pseudofungi
Cp_Diaphoretickes
Other_Diaphoretickes
Other_Stramenopiles
Other_Amorphea
Osmo_Labyrinthulea
Other
Non-eukaryotes
Other_SAR
OG3189_intClade_2 Teretosporea
Diamond score
0
50
100
150
200
Other_Holozoa
Osmo_Teretosporea
Pp_Diaphoretickes
Osmo_Fungi
Other_Holomycota
Osmo_Pseudofungi
Cp_Diaphoretickes
Other_Diaphoretickes
Other_Stramenopiles
Other_Amorphea
Osmo_Labyrinthulea
Other
Non-eukaryotes
Other_SAR

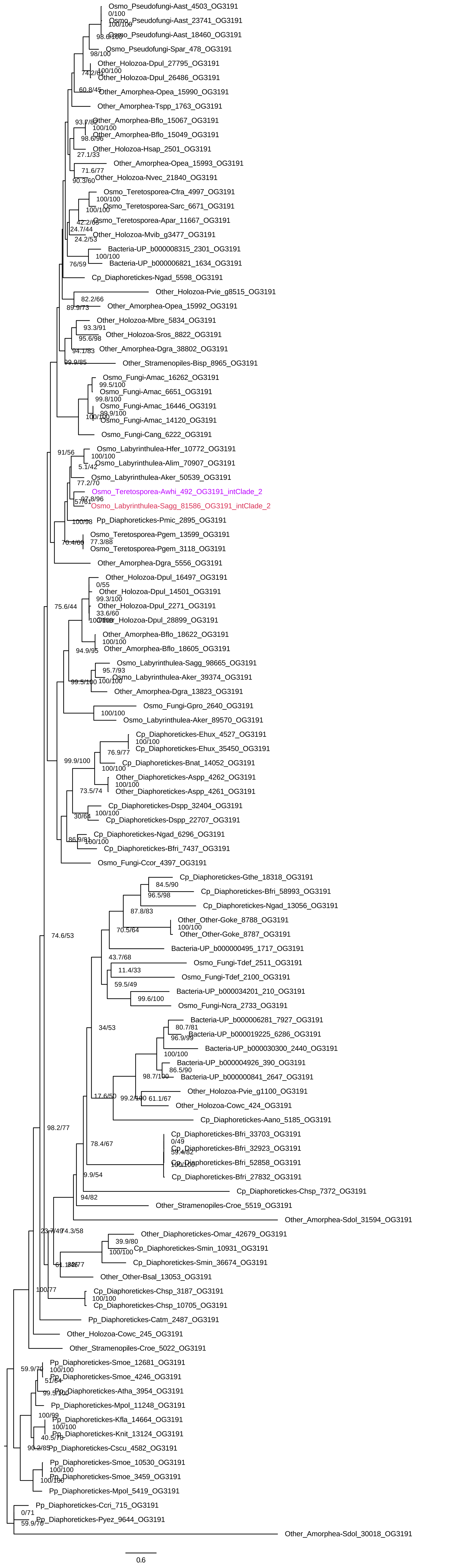
Osmo_Pseudofungi-Aast_4503_OG3191
Osmo_Pseudofungi-Aast_23741_OG3191
Osmo_Pseudofungi-Aast_18460_OG3191
Osmo_Pseudofungi-Spar_478_OG3191
Other_Holozoa-Dpul_27795_OG3191
Other_Holozoa-Dpul_26486_OG3191
Other_Amorphea-Opea_15990_OG3191
Other_Amorphea-Tspp_1763_OG3191
Other_Amorphea-Bflo_15067_OG3191
Other_Amorphea-Bflo_15049_OG3191
Other_Holozoa-Hsap_2501_OG3191
Other_Amorphea-Opea_15993_OG3191
Other_Holozoa-Nvec_21840_OG3191
Osmo_Teretosporea-Cfra_4997_OG3191
Osmo_Teretosporea-Sarc_6671_OG3191
Osmo_Teretosporea-Apar_11667_OG3191
Other_Holozoa-Mvib_g3477_OG3191
Bacteria-UP_b000008315_2301_OG3191
Bacteria-UP_b000006821_1634_OG3191
Cp_Diaphoretickes-Ngad_5598_OG3191
Other_Holozoa-Pvie_g8515_OG3191
Other_Amorphea-Opea_15992_OG3191
Other_Holozoa-Mbre_5834_OG3191
Other_Holozoa-Sros_8822_OG3191
Other_Amorphea-Dgra_38802_OG3191
Other_Stramenopiles-Bisp_8965_OG3191
Osmo_Fungi-Amac_16262_OG3191
Osmo_Fungi-Amac_6651_OG3191
Osmo_Fungi-Amac_16446_OG3191
Osmo_Fungi-Amac_14120_OG3191
Osmo_Fungi-Cang_6222_OG3191
Osmo_Labyrinthulea-Hfer_10772_OG3191
Osmo_Labyrinthulea-Alim_70907_OG3191
Osmo_Labyrinthulea-Aker_50539_OG3191
Osmo_Teretosporea-Awhi_492_OG3191_intClade_2
Osmo_Labyrinthulea-Sagg_81586_OG3191_intClade_2
Pp_Diaphoretickes-Pmic_2895_OG3191
Osmo_Teretosporea-Pgem_13599_OG3191
Osmo_Teretosporea-Pgem_3118_OG3191
Other_Amorphea-Dgra_5556_OG3191
Other_Holozoa-Dpul_16497_OG3191
Other_Holozoa-Dpul_14501_OG3191
Other_Holozoa-Dpul_2271_OG3191
Other_Holozoa-Dpul_28899_OG3191
Other_Amorphea-Bflo_18622_OG3191
Other_Amorphea-Bflo_18605_OG3191
Osmo_Labyrinthulea-Sagg_98665_OG3191
Osmo_Labyrinthulea-Aker_39374_OG3191
Other_Amorphea-Dgra_13823_OG3191
Osmo_Fungi-Gpro_2640_OG3191
Osmo_Labyrinthulea-Aker_89570_OG3191
Cp_Diaphoretickes-Ehux_4527_OG3191
Cp_Diaphoretickes-Ehux_35450_OG3191
Cp_Diaphoretickes-Bnat_14052_OG3191
Other_Diaphoretickes-Aspp_4262_OG3191
Other_Diaphoretickes-Aspp_4261_OG3191
Cp_Diaphoretickes-Dspp_32404_OG3191
Cp_Diaphoretickes-Dspp_22707_OG3191
Cp_Diaphoretickes-Ngad_6296_OG3191
Cp_Diaphoretickes-Bfri_7437_OG3191
Osmo_Fungi-Ccor_4397_OG3191
Cp_Diaphoretickes-Gthe_18318_OG3191
Cp_Diaphoretickes-Bfri_58993_OG3191
Cp_Diaphoretickes-Ngad_13056_OG3191
Other_Other-Goke_8788_OG3191
Other_Other-Goke_8787_OG3191
Bacteria-UP_b000000495_1717_OG3191
Osmo_Fungi-Tdef_2511_OG3191
Osmo_Fungi-Tdef_2100_OG3191
Bacteria-UP_b000034201_210_OG3191
Osmo_Fungi-Ncra_2733_OG3191
Bacteria-UP_b000006281_7927_OG3191
Bacteria-UP_b000019225_6286_OG3191
Bacteria-UP_b000030300_2440_OG3191
Bacteria-UP_b000004926_390_OG3191
Bacteria-UP_b000000841_2647_OG3191
Other_Holozoa-Pvie_g1100_OG3191
Other_Holozoa-Cowc_424_OG3191
Cp_Diaphoretickes-Aano_5185_OG3191
Cp_Diaphoretickes-Bfri_33703_OG3191
Cp_Diaphoretickes-Bfri_32923_OG3191
Cp_Diaphoretickes-Bfri_52858_OG3191
Cp_Diaphoretickes-Bfri_27832_OG3191
Cp_Diaphoretickes-Chsp_7372_OG3191
Other_Stramenopiles-Croe_5519_OG3191
Other_Amorphea-Sdol_31594_OG3191
Other_Diaphoretickes-Omar_42679_OG3191
Cp_Diaphoretickes-Smin_10931_OG3191
Cp_Diaphoretickes-Smin_36674_OG3191
Other_Other-Bsal_13053_OG3191
Cp_Diaphoretickes-Chsp_3187_OG3191
Cp_Diaphoretickes-Chsp_10705_OG3191
Pp_Diaphoretickes-Catm_2487_OG3191
Other_Holozoa-Cowc_245_OG3191
Other_Stramenopiles-Croe_5022_OG3191
Pp_Diaphoretickes-Smoe_12681_OG3191
Pp_Diaphoretickes-Smoe_4246_OG3191
Pp_Diaphoretickes-Atha_3954_OG3191
Pp_Diaphoretickes-Mpol_11248_OG3191
Pp_Diaphoretickes-Kfla_14664_OG3191
Pp_Diaphoretickes-Knit_13124_OG3191
Pp_Diaphoretickes-Cscu_4582_OG3191
Pp_Diaphoretickes-Smoe_10530_OG3191
Pp_Diaphoretickes-Smoe_3459_OG3191
Pp_Diaphoretickes-Mpol_5419_OG3191
Pp_Diaphoretickes-Ccri_715_OG3191
Pp_Diaphoretickes-Pyez_9644_OG3191
Other_Amorphea-Sdol_30018_OG3191
0.6

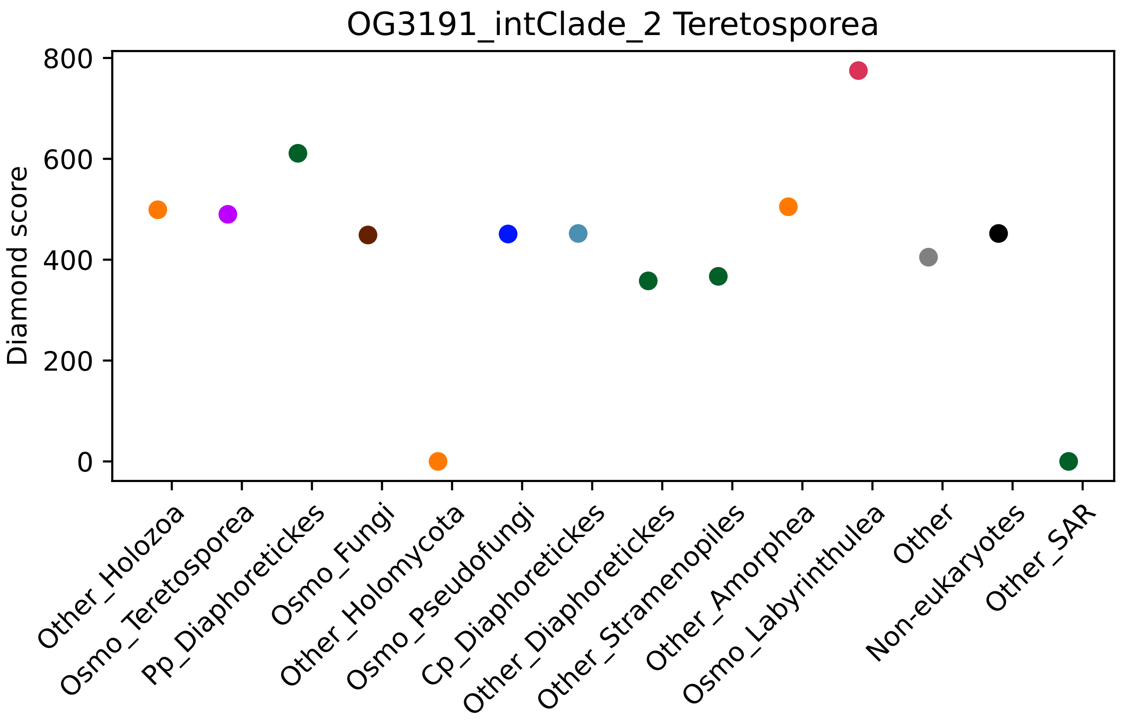
OG3191_intClade_2 Teretosporea
Diamond score
0
200
400
600
800
Other_Holozoa
Osmo_Teretosporea
Pp_Diaphoretickes
Osmo_Fungi
Other_Holomycota
Osmo_Pseudofungi
Cp_Diaphoretickes
Other_Diaphoretickes
Other_Stramenopiles
Other_Amorphea
Osmo_Labyrinthulea
Other
Non-eukaryotes
Other_SAR
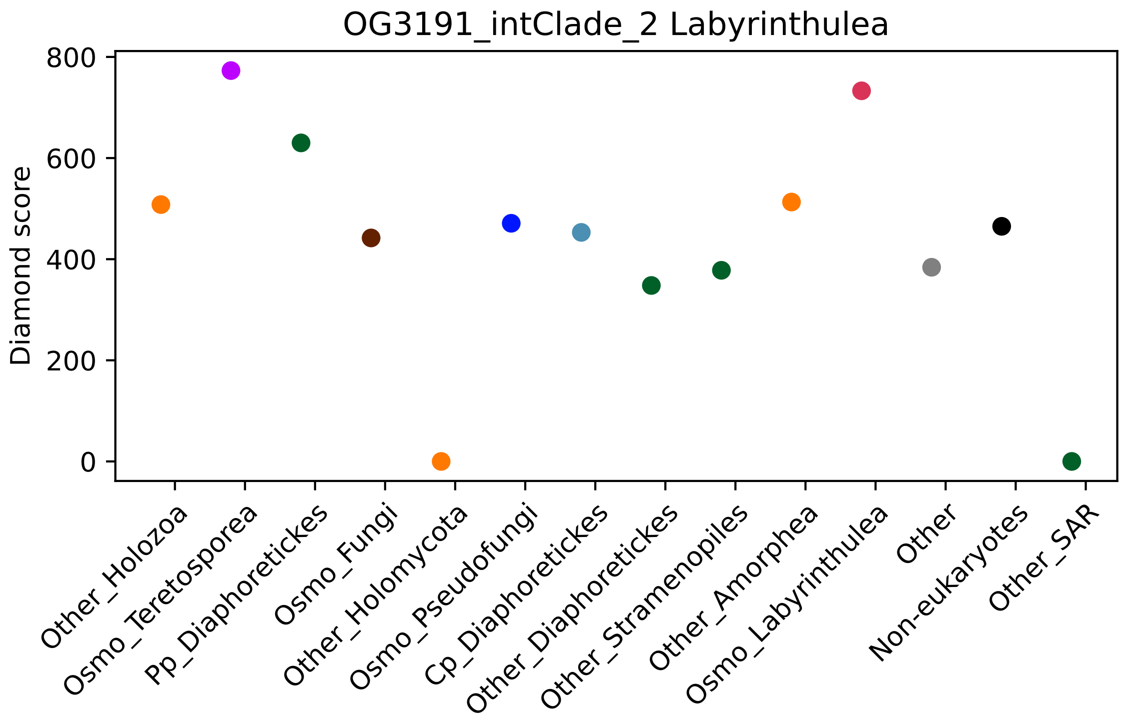
OG3191_intClade_2 Labyrinthulea
Diamond score
0
200
400
600
800
Other_Holozoa
Osmo_Teretosporea
Pp_Diaphoretickes
Osmo_Fungi
Other_Holomycota
Osmo_Pseudofungi
Cp_Diaphoretickes
Other_Diaphoretickes
Other_Stramenopiles
Other_Amorphea
Osmo_Labyrinthulea
Other
Non-eukaryotes
Other_SAR

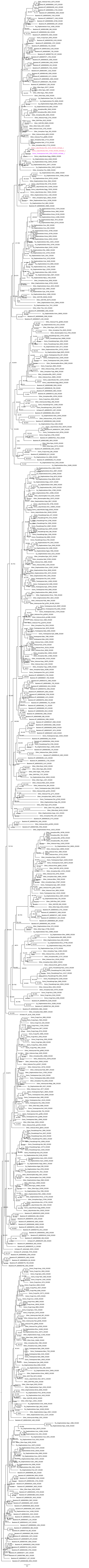

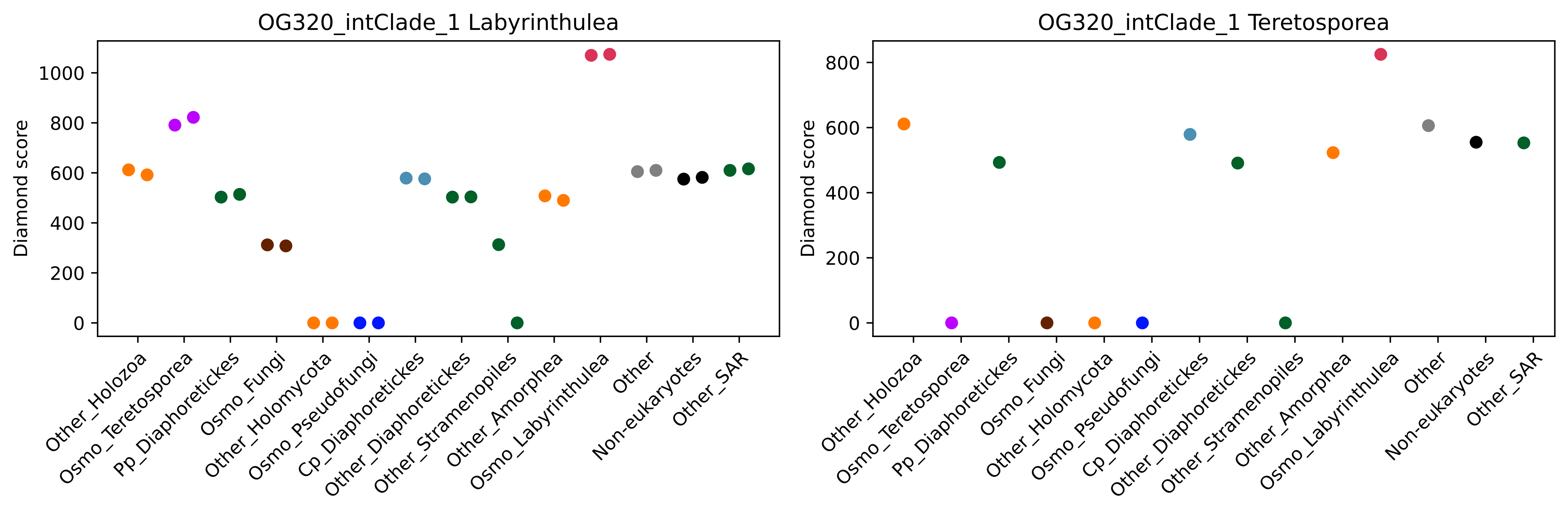

OG320_intClade_1 Labyrinthulea
Diamond score
0
200
400
600
800
1000
Other_Holozoa
Osmo_Teretosporea
Pp_Diaphoretickes
Osmo_Fungi
Other_Holomycota
Osmo_Pseudofungi
Cp_Diaphoretickes
Other_Diaphoretickes
Other_Stramenopiles
Other_Amorphea
Osmo_Labyrinthulea
Other
Non-eukaryotes
Other_SAR
OG320_intClade_1 Teretosporea
Diamond score
0
200
400
600
800
Other_Holozoa
Osmo_Teretosporea
Pp_Diaphoretickes
Osmo_Fungi
Other_Holomycota
Osmo_Pseudofungi
Cp_Diaphoretickes
Other_Diaphoretickes
Other_Stramenopiles
Other_Amorphea
Osmo_Labyrinthulea
Other
Non-eukaryotes
Other_SAR

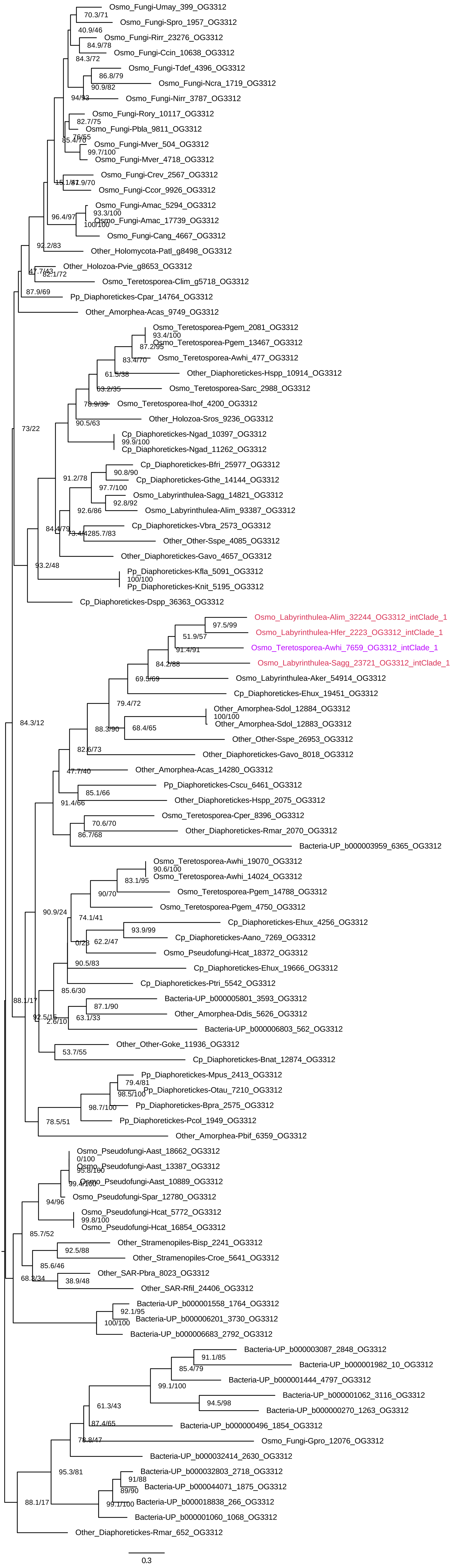

Osmo_Fungi-Umay_399_OG3312
70.3/71
Osmo_Fungi-Spro_1957_OG3312
40.9/46
Osmo_Fungi-Rirr_23276_OG3312
84.9/78
Osmo_Fungi-Ccin_10638_OG3312
84.3/72
Osmo_Fungi-Tdef_4396_OG3312
86.8/79
Osmo_Fungi-Ncra_1719_OG3312
90.9/82
Osmo_Fungi-Nirr_3787_OG3312
94/93
Osmo_Fungi-Rory_10117_OG3312
82.7/75
Osmo_Fungi-Pbla_9811_OG3312
76/55
85.4/70
Osmo_Fungi-Mver_504_OG3312
99.7/100
Osmo_Fungi-Mver_4718_OG3312
Osmo_Fungi-Crev_2567_OG3312
15.1/81.9/70
Osmo_Fungi-Ccor_9926_OG3312
Osmo_Fungi-Amac_5294_OG3312
93.3/100
96.4/97
Osmo_Fungi-Amac_17739_OG3312
100/100
Osmo_Fungi-Cang_4667_OG3312
92.2/83
Other_Holomycota-Patl_g8498_OG3312
Other_Holozoa-Pvie_g8653_OG3312
47.7/43
82.1/72
Osmo_Teretosporea-Clim_g5718_OG3312
87.9/69
Pp_Diaphoretickes-Cpar_14764_OG3312
Other_Amorphea-Acas_9749_OG3312
Osmo_Teretosporea-Pgem_2081_OG3312
93.4/100
Osmo_Teretosporea-Pgem_13467_OG3312
87.2/95
83.4/70
Osmo_Teretosporea-Awhi_477_OG3312
61.5/38
Other_Diaphoretickes-Hspp_10914_OG3312
63.2/35
Osmo_Teretosporea-Sarc_2988_OG3312
78.9/39
Osmo_Teretosporea-Ihof_4200_OG3312
90.5/63
Other_Holozoa-Sros_9236_OG3312
73/22
Cp_Diaphoretickes-Ngad_10397_OG3312
99.9/100
Cp_Diaphoretickes-Ngad_11262_OG3312
Cp_Diaphoretickes-Bfri_25977_OG3312
90.8/90
91.2/78
Cp_Diaphoretickes-Gthe_14144_OG3312
97.7/100
Osmo_Labyrinthulea-Sagg_14821_OG3312
92.8/92
92.6/86
Osmo_Labyrinthulea-Alim_93387_OG3312
Cp_Diaphoretickes-Vbra_2573_OG3312
84.4/79
73.4/4285.7/83
Other_Other-Sspe_4085_OG3312
Other_Diaphoretickes-Gavo_4657_OG3312
93.2/48
Pp_Diaphoretickes-Kfla_5091_OG3312
100/100
Pp_Diaphoretickes-Knit_5195_OG3312
Cp_Diaphoretickes-Dspp_36363_OG3312
Osmo_Labyrinthulea-Alim_32244_OG3312_intClade_1
97.5/99
Osmo_Labyrinthulea-Hfer_2223_OG3312_intClade_1
51.9/57
Osmo_Teretosporea-Awhi_7659_OG3312_intClade_1
91.4/91
84.2/88
Osmo_Labyrinthulea-Sagg_23721_OG3312_intClade_1
69.5/69
Osmo_Labyrinthulea-Aker_54914_OG3312
Cp_Diaphoretickes-Ehux_19451_OG3312
79.4/72
Other_Amorphea-Sdol_12884_OG3312
100/100
84.3/12
88.3/90
68.4/65
Other_Amorphea-Sdol_12883_OG3312
Other_Other-Sspe_26953_OG3312
82.6/73
Other_Diaphoretickes-Gavo_8018_OG3312
47.7/40
Other_Amorphea-Acas_14280_OG3312
Pp_Diaphoretickes-Cscu_6461_OG3312
85.1/66
91.4/66
Other_Diaphoretickes-Hspp_2075_OG3312
Osmo_Teretosporea-Cper_8396_OG3312
70.6/70
86.7/68
Other_Diaphoretickes-Rmar_2070_OG3312
Bacteria-UP_b000003959_6365_OG3312
Osmo_Teretosporea-Awhi_19070_OG3312
90.6/100
83.1/95
Osmo_Teretosporea-Awhi_14024_OG3312
90/70
Osmo_Teretosporea-Pgem_14788_OG3312
90.9/24
Osmo_Teretosporea-Pgem_4750_OG3312
74.1/41
Cp_Diaphoretickes-Ehux_4256_OG3312
93.9/99
0/23
62.2/47
Cp_Diaphoretickes-Aano_7269_OG3312
Osmo_Pseudofungi-Hcat_18372_OG3312
90.5/83
Cp_Diaphoretickes-Ehux_19666_OG3312
Cp_Diaphoretickes-Ptri_5542_OG3312
85.6/30
88.1/17
Bacteria-UP_b000005801_3593_OG3312
87.1/90
92.5/16
2.6/10
63.1/33
Other_Amorphea-Ddis_5626_OG3312
Bacteria-UP_b000006803_562_OG3312
Other_Other-Goke_11936_OG3312
53.7/55
Cp_Diaphoretickes-Bnat_12874_OG3312
Pp_Diaphoretickes-Mpus_2413_OG3312
79.4/81
98.5/100
Pp_Diaphoretickes-Otau_7210_OG3312
98.7/100
Pp_Diaphoretickes-Bpra_2575_OG3312
78.5/51
Pp_Diaphoretickes-Pcol_1949_OG3312
Other_Amorphea-Pbif_6359_OG3312
Osmo_Pseudofungi-Aast_18662_OG3312
0/100
Osmo_Pseudofungi-Aast_13387_OG3312
95.8/100
99.4/100
Osmo_Pseudofungi-Aast_10889_OG3312
94/96
Osmo_Pseudofungi-Spar_12780_OG3312
Osmo_Pseudofungi-Hcat_5772_OG3312
99.8/100
Osmo_Pseudofungi-Hcat_16854_OG3312
85.7/52
Other_Stramenopiles-Bisp_2241_OG3312
92.5/88
Other_Stramenopiles-Croe_5641_OG3312
85.6/46
Other_SAR-Pbra_8023_OG3312
68.8/34
38.9/48
Other_SAR-Rfil_24406_OG3312
Bacteria-UP_b000001558_1764_OG3312
92.1/95
100/100
Bacteria-UP_b000006201_3730_OG3312
Bacteria-UP_b000006683_2792_OG3312
Bacteria-UP_b000003087_2848_OG3312
91.1/85
Bacteria-UP_b000001982_10_OG3312
85.4/79
Bacteria-UP_b000001444_4797_OG3312
99.1/100
Bacteria-UP_b000001062_3116_OG3312
61.3/43
94.5/98
Bacteria-UP_b000000270_1263_OG3312
87.4/65
Bacteria-UP_b000000496_1854_OG3312
78.8/47
Osmo_Fungi-Gpro_12076_OG3312
Bacteria-UP_b000032414_2630_OG3312
95.3/81
Bacteria-UP_b000032803_2718_OG3312
91/88
Bacteria-UP_b000044071_1875_OG3312
89/90
88.1/17
99.1/100
Bacteria-UP_b000018838_266_OG3312
Bacteria-UP_b000001060_1068_OG3312
Other_Diaphoretickes-Rmar_652_OG3312
0.3

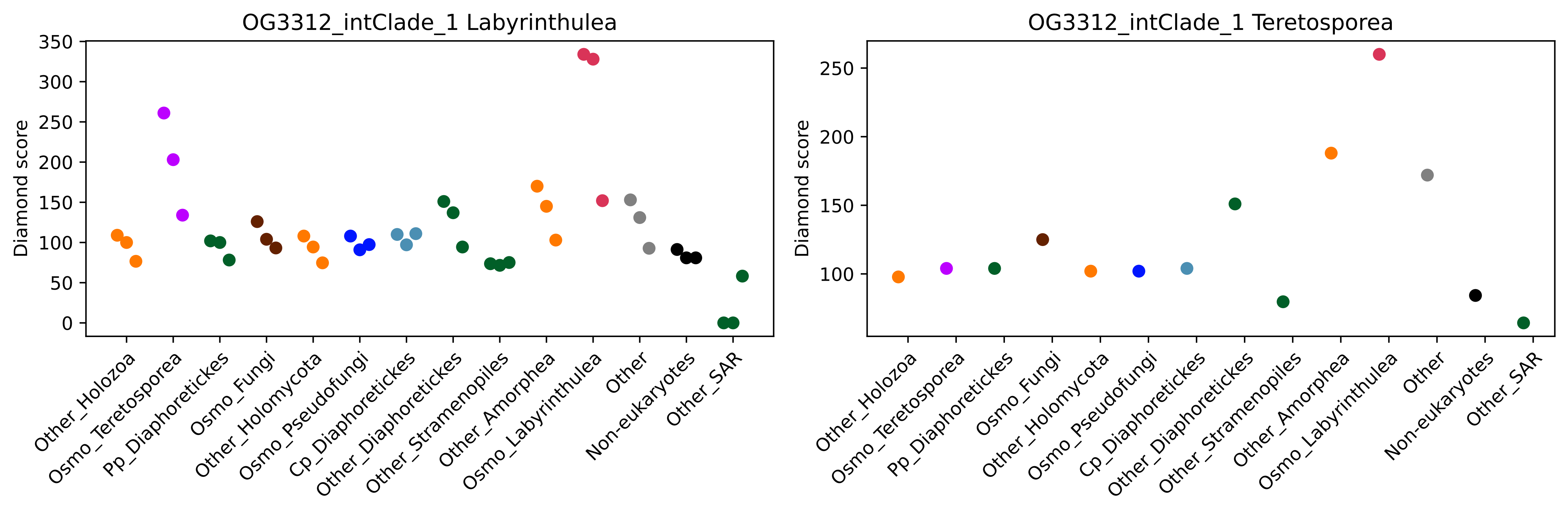
OG3312_intClade_1 Labyrinthulea
OG3312_intClade_1 Teretosporea
Diamond score
Other_Holozoa
Osmo_Teretosporea
Pp_Diaphoretickes
Osmo_Fungi
Other_Holomycota
Osmo_Pseudofungi
Cp_Diaphoretickes
Other_Diaphoretickes
Other_Stramenopiles
Other_Amorphea
Osmo_Labyrinthulea
Other
Non-eukaryotes
Other_SAR

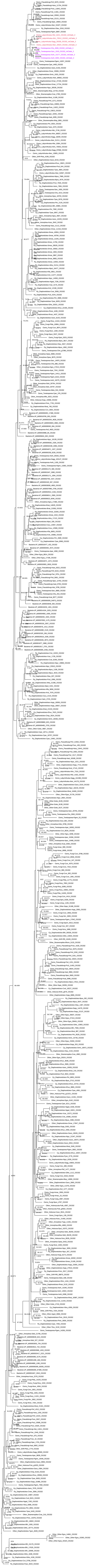

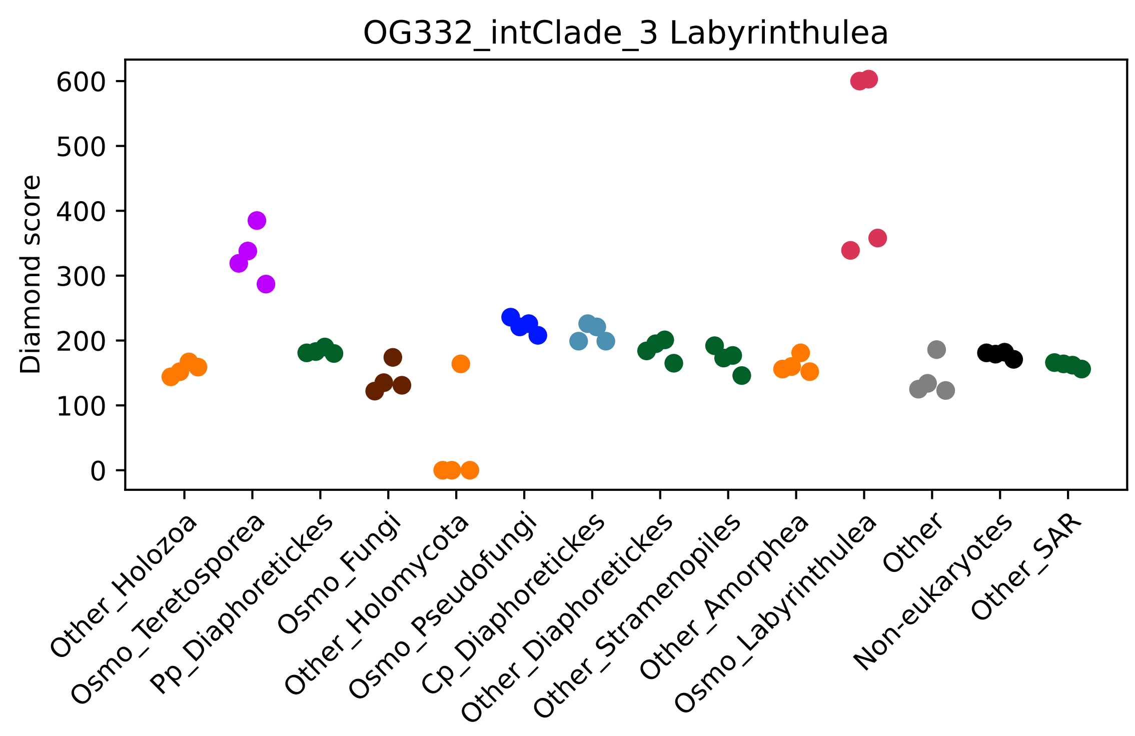

OG332_intClade_3 Labyrinthulea
Diamond score
0
100
200
300
400
500
600
Other_Holozoa
Osmo_Teretosporea
Pp_Diaphoretickes
Osmo_Fungi
Other_Holomycota
Osmo_Pseudofungi
Cp_Diaphoretickes
Other_Diaphoretickes
Other_Stramenopiles
Other_Amorphea
Osmo_Labyrinthulea
Other
Non-eukaryotes
Other_SAR

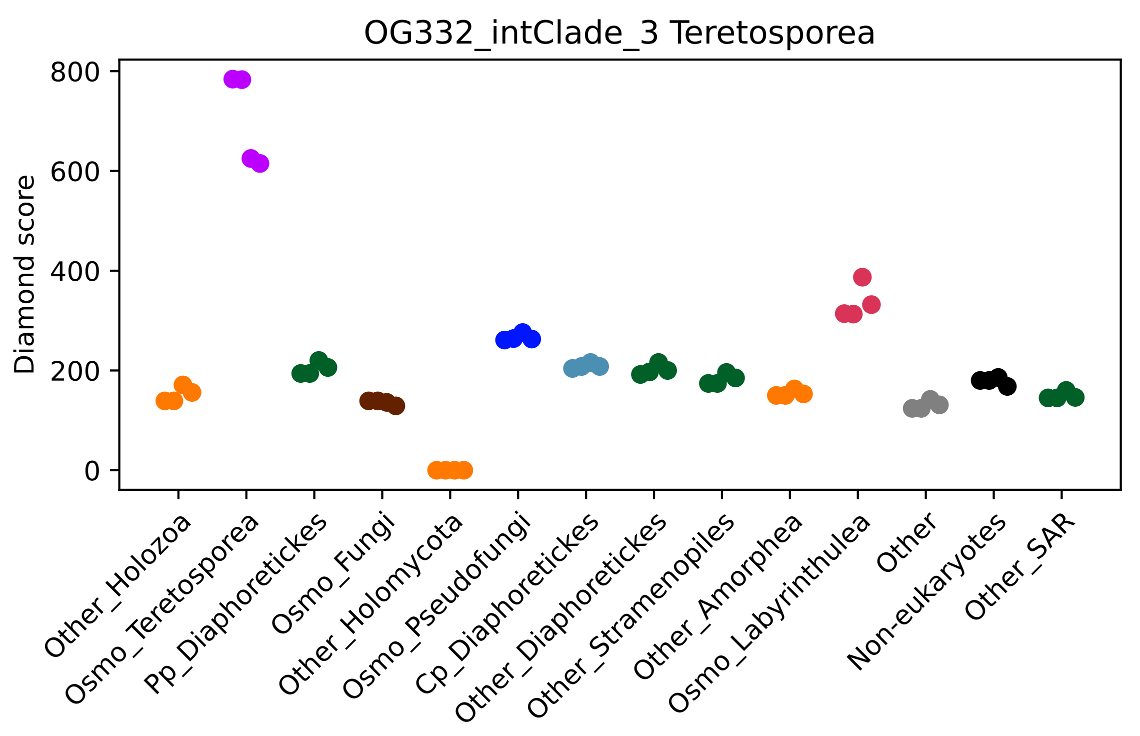

OG332_intClade_3 Teretosporea
Diamond score
0
200
400
600
800
Other_Holozoa
Osmo_Teretosporea
Pp_Diaphoretickes
Osmo_Fungi
Other_Holomycota
Osmo_Pseudofungi
Cp_Diaphoretickes
Other_Diaphoretickes
Other_Stramenopiles
Other_Amorphea
Osmo_Labyrinthulea
Other
Non-eukaryotes
Other_SAR

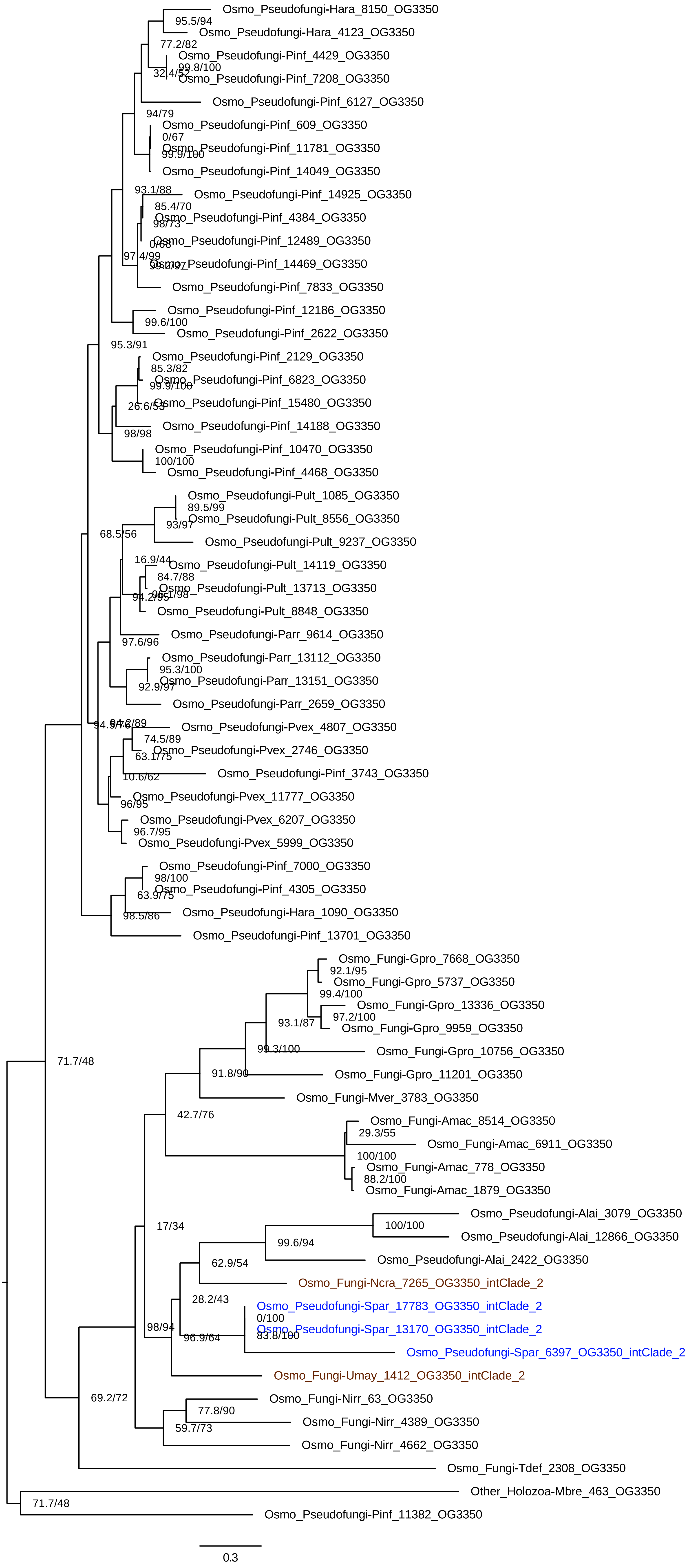

Osmo_Pseudofungi-Hara_8150_OG3350
95.5/94
Osmo_Pseudofungi-Hara_4123_OG3350
77.2/82
Osmo_Pseudofungi-Pinf_4429_OG3350
99.8/100
32.4/52
Osmo_Pseudofungi-Pinf_7208_OG3350
Osmo_Pseudofungi-Pinf_6127_OG3350
94/79
Osmo_Pseudofungi-Pinf_609_OG3350
0/67
Osmo_Pseudofungi-Pinf_11781_OG3350
99.9/100
Osmo_Pseudofungi-Pinf_14049_OG3350
93.1/88
Osmo_Pseudofungi-Pinf_14925_OG3350
85.4/70
Osmo_Pseudofungi-Pinf_4384_OG3350
98/73
Osmo_Pseudofungi-Pinf_12489_OG3350
0/68
97.4/99
Osmo_Pseudofungi-Pinf_14469_OG3350
Osmo_Pseudofungi-Pinf_7833_OG3350
Osmo_Pseudofungi-Pinf_12186_OG3350
99.6/100
Osmo_Pseudofungi-Pinf_2622_OG3350
95.3/91
Osmo_Pseudofungi-Pinf_2129_OG3350
85.3/82
Osmo_Pseudofungi-Pinf_6823_OG3350
99.9/100
26.6/53
Osmo_Pseudofungi-Pinf_15480_OG3350
Osmo_Pseudofungi-Pinf_14188_OG3350
98/98
Osmo_Pseudofungi-Pinf_10470_OG3350
100/100
Osmo_Pseudofungi-Pinf_4468_OG3350
Osmo_Pseudofungi-Pult_1085_OG3350
89.5/99
Osmo_Pseudofungi-Pult_8556_OG3350
93/97
68.5/56
Osmo_Pseudofungi-Pult_9237_OG3350
16.9/44
Osmo_Pseudofungi-Pult_14119_OG3350
84.7/88
Osmo_Pseudofungi-Pult_13713_OG3350
94.2/95
Osmo_Pseudofungi-Pult_8848_OG3350
97.6/96
Osmo_Pseudofungi-Parr_9614_OG3350
Osmo_Pseudofungi-Parr_13112_OG3350
95.3/100
Osmo_Pseudofungi-Parr_13151_OG3350
92.9/97
Osmo_Pseudofungi-Parr_2659_OG3350
Osmo_Pseudofungi-Pvex_4807_OG3350
74.5/89
Osmo_Pseudofungi-Pvex_2746_OG3350
63.1/75
Osmo_Pseudofungi-Pinf_3743_OG3350
10.6/62
Osmo_Pseudofungi-Pvex_11777_OG3350
96/95
Osmo_Pseudofungi-Pvex_6207_OG3350
96.7/95
Osmo_Pseudofungi-Pvex_5999_OG3350
Osmo_Pseudofungi-Pinf_7000_OG3350
98/100
Osmo_Pseudofungi-Pinf_4305_OG3350
63.9/75
98.5/86
Osmo_Pseudofungi-Hara_1090_OG3350
Osmo_Pseudofungi-Pinf_13701_OG3350
Osmo_Fungi-Gpro_7668_OG3350
92.1/95
Osmo_Fungi-Gpro_5737_OG3350
99.4/100
Osmo_Fungi-Gpro_13336_OG3350
97.2/100
93.1/87
Osmo_Fungi-Gpro_9959_OG3350
99.3/100
Osmo_Fungi-Gpro_10756_OG3350
91.8/90
Osmo_Fungi-Gpro_11201_OG3350
Osmo_Fungi-Mver_3783_OG3350
71.7/48
42.7/76
Osmo_Fungi-Amac_8514_OG3350
29.3/55
Osmo_Fungi-Amac_6911_OG3350
100/100
Osmo_Fungi-Amac_778_OG3350
88.2/100
Osmo_Fungi-Amac_1879_OG3350
Osmo_Pseudofungi-Alai_3079_OG3350
17/34
100/100
Osmo_Pseudofungi-Alai_12866_OG3350
99.6/94
Osmo_Pseudofungi-Alai_2422_OG3350
62.9/54
Osmo_Fungi-Ncra_7265_OG3350_intClade_2
28.2/43
Osmo_Pseudofungi-Spar_17783_OG3350_intClade_2
0/100
98/94
Osmo_Pseudofungi-Spar_13170_OG3350_intClade_2
96.9/64
83.8/100
Osmo_Pseudofungi-Spar_6397_OG3350_intClade_2
Osmo_Fungi-Umay_1412_OG3350_intClade_2
69.2/72
Osmo_Fungi-Nirr_63_OG3350
77.8/90
Osmo_Fungi-Nirr_4389_OG3350
59.7/73
Osmo_Fungi-Nirr_4662_OG3350
Osmo_Fungi-Tdef_2308_OG3350
Other_Holozoa-Mbre_463_OG3350
71.7/48
Osmo_Pseudofungi-Pinf_11382_OG3350
0.3

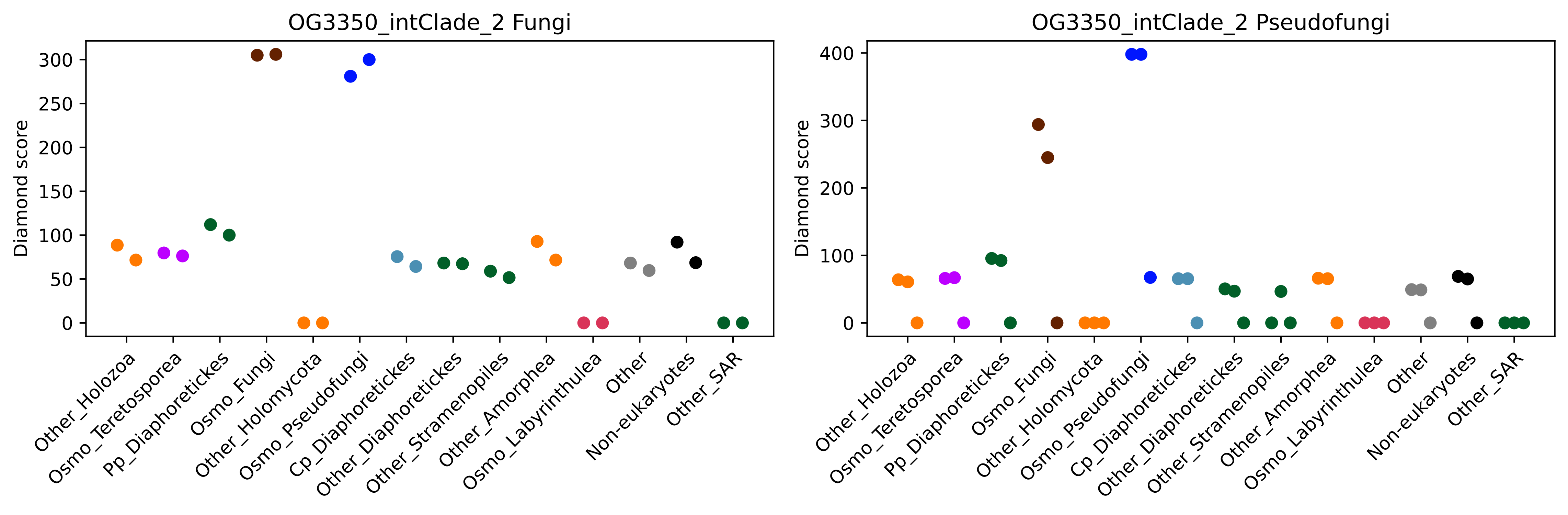
OG3350_intClade_2 Fungi
OG3350_intClade_2 Pseudofungi
Diamond score
0
50
100
150
200
250
300
400
Other_Holozoa
Osmo_Teretosporea
Pp_Diaphoretickes
Osmo_Fungi
Other_Holomycota
Osmo_Pseudofungi
Cp_Diaphoretickes
Other_Diaphoretickes
Other_Stramenopiles
Other_Amorphea
Osmo_Labyrinthulea
Other
Non-eukaryotes
Other_SAR

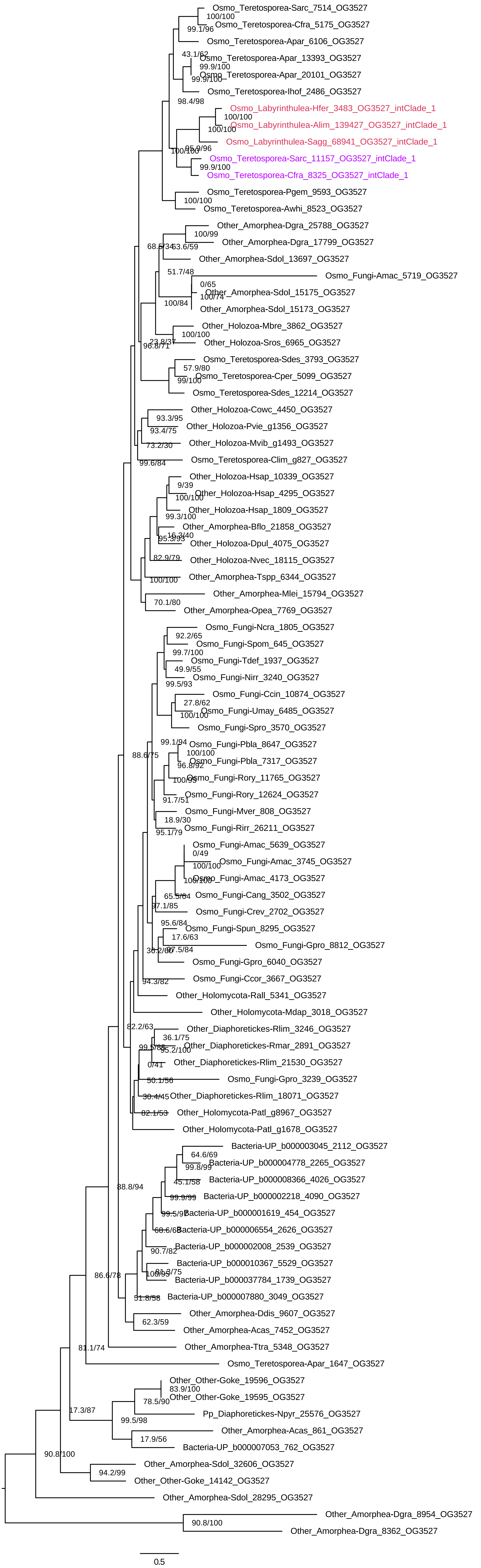

Osmo_Teretosporea-Sarc_7514_OG3527
Osmo_Teretosporea-Cfra_5175_OG3527
Osmo_Teretosporea-Apar_6106_OG3527
Osmo_Teretosporea-Apar_13393_OG3527
Osmo_Teretosporea-Apar_20101_OG3527
Osmo_Teretosporea-Ihof_2486_OG3527
Osmo_Labyrinthulea-Hfer_3483_OG3527_intClade_1
Osmo_Labyrinthulea-Alim_139427_OG3527_intClade_1
Osmo_Labyrinthulea-Sagg_68941_OG3527_intClade_1
Osmo_Teretosporea-Sarc_11157_OG3527_intClade_1
Osmo_Teretosporea-Cfra_8325_OG3527_intClade_1
Osmo_Teretosporea-Pgem_9593_OG3527
Osmo_Teretosporea-Awhi_8523_OG3527
Other_Amorphea-Dgra_25788_OG3527
Other_Amorphea-Dgra_17799_OG3527
Other_Amorphea-Sdol_13697_OG3527
Osmo_Fungi-Amac_5719_OG3527
Other_Amorphea-Sdol_15175_OG3527
Other_Amorphea-Sdol_15173_OG3527
Other_Holozoa-Mbre_3862_OG3527
Other_Holozoa-Sros_6965_OG3527
Osmo_Teretosporea-Sdes_3793_OG3527
Osmo_Teretosporea-Cper_5099_OG3527
Osmo_Teretosporea-Sdes_12214_OG3527
Other_Holozoa-Cowc_4450_OG3527
Other_Holozoa-Pvie_g1356_OG3527
Other_Holozoa-Mvib_g1493_OG3527
Osmo_Teretosporea-Clim_g827_OG3527
Other_Holozoa-Hsap_10339_OG3527
Other_Holozoa-Hsap_4295_OG3527
Other_Holozoa-Hsap_1809_OG3527
Other_Amorphea-Bflo_21858_OG3527
Other_Holozoa-Dpul_4075_OG3527
Other_Holozoa-Nvec_18115_OG3527
Other_Amorphea-Tspp_6344_OG3527
Other_Amorphea-Mlei_15794_OG3527
Other_Amorphea-Opea_7769_OG3527
Osmo_Fungi-Ncra_1805_OG3527
Osmo_Fungi-Spom_645_OG3527
Osmo_Fungi-Tdef_1937_OG3527
Osmo_Fungi-Nirr_3240_OG3527
Osmo_Fungi-Ccin_10874_OG3527
Osmo_Fungi-Umay_6485_OG3527
Osmo_Fungi-Spro_3570_OG3527
Osmo_Fungi-Pbla_8647_OG3527
Osmo_Fungi-Pbla_7317_OG3527
Osmo_Fungi-Rory_11765_OG3527
Osmo_Fungi-Rory_12624_OG3527
Osmo_Fungi-Mver_808_OG3527
Osmo_Fungi-Rirr_26211_OG3527
Osmo_Fungi-Amac_5639_OG3527
Osmo_Fungi-Amac_3745_OG3527
Osmo_Fungi-Amac_4173_OG3527
Osmo_Fungi-Cang_3502_OG3527
Osmo_Fungi-Crev_2702_OG3527
Osmo_Fungi-Spun_8295_OG3527
Osmo_Fungi-Gpro_8812_OG3527
Osmo_Fungi-Gpro_6040_OG3527
Osmo_Fungi-Ccor_3667_OG3527
Other_Holomycota-Rall_5341_OG3527
Other_Holomycota-Mdap_3018_OG3527
Other_Diaphoretickes-Rlim_3246_OG3527
Other_Diaphoretickes-Rmar_2891_OG3527
Other_Diaphoretickes-Rlim_21530_OG3527
Osmo_Fungi-Gpro_3239_OG3527
Other_Diaphoretickes-Rlim_18071_OG3527
Other_Holomycota-Patl_g8967_OG3527
Other_Holomycota-Patl_g1678_OG3527
Bacteria-UP_b000003045_2112_OG3527
Bacteria-UP_b000004778_2265_OG3527
Bacteria-UP_b000008366_4026_OG3527
Bacteria-UP_b000002218_4090_OG3527
Bacteria-UP_b000001619_454_OG3527
Bacteria-UP_b000006554_2626_OG3527
Bacteria-UP_b000002008_2539_OG3527
Bacteria-UP_b000010367_5529_OG3527
Bacteria-UP_b000037784_1739_OG3527
Bacteria-UP_b000007880_3049_OG3527
Other_Amorphea-Ddis_9607_OG3527
Other_Amorphea-Acas_7452_OG3527
Other_Amorphea-Ttra_5348_OG3527
Osmo_Teretosporea-Apar_1647_OG3527
Other_Other-Goke_19596_OG3527
Other_Other-Goke_19595_OG3527
Pp_Diaphoretickes-Npyr_25576_OG3527
Other_Amorphea-Acas_861_OG3527
Bacteria-UP_b000007053_762_OG3527
Other_Amorphea-Sdol_32606_OG3527
Other_Other-Goke_14142_OG3527
Other_Amorphea-Sdol_28295_OG3527
Other_Amorphea-Dgra_8954_OG3527
Other_Amorphea-Dgra_8362_OG3527
0.5

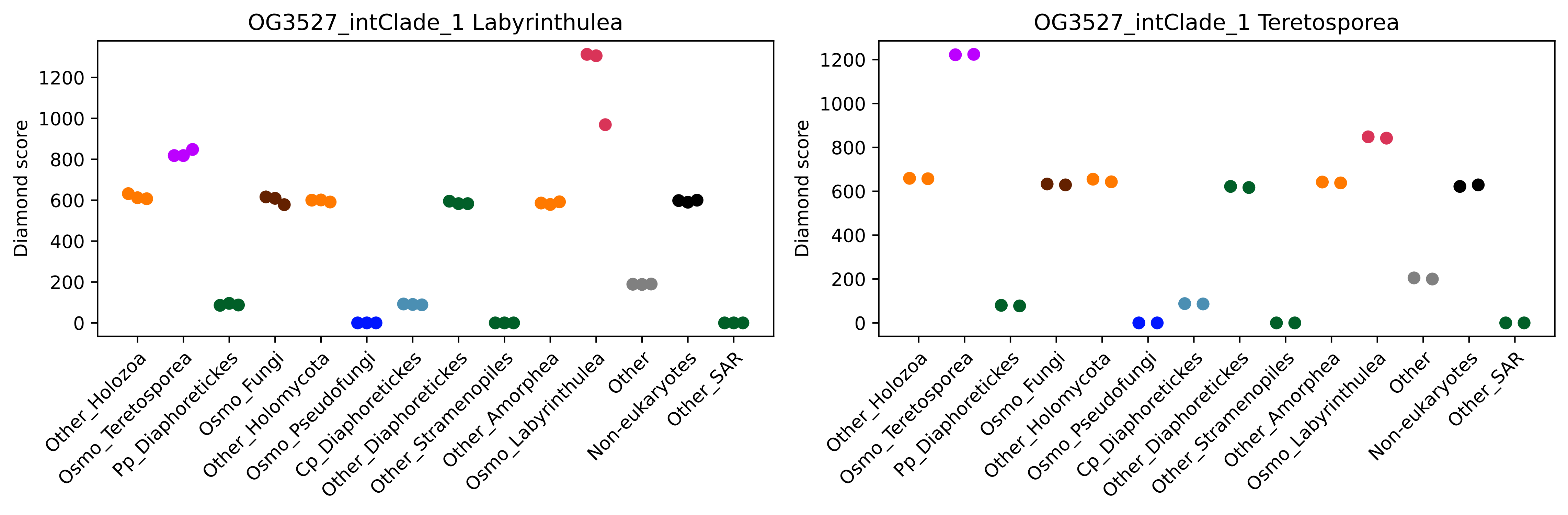

OG3527_intClade_1 Labyrinthulea
OG3527_intClade_1 Teretosporea
Diamond score
0
200
400
600
800
1000
1200
Other_Holozoa
Osmo_Teretosporea
Pp_Diaphoretickes
Osmo_Fungi
Other_Holomycota
Osmo_Pseudofungi
Cp_Diaphoretickes
Other_Diaphoretickes
Other_Stramenopiles
Other_Amorphea
Osmo_Labyrinthulea
Other
Non-eukaryotes
Other_SAR

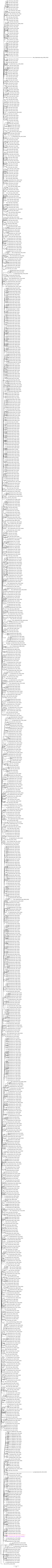

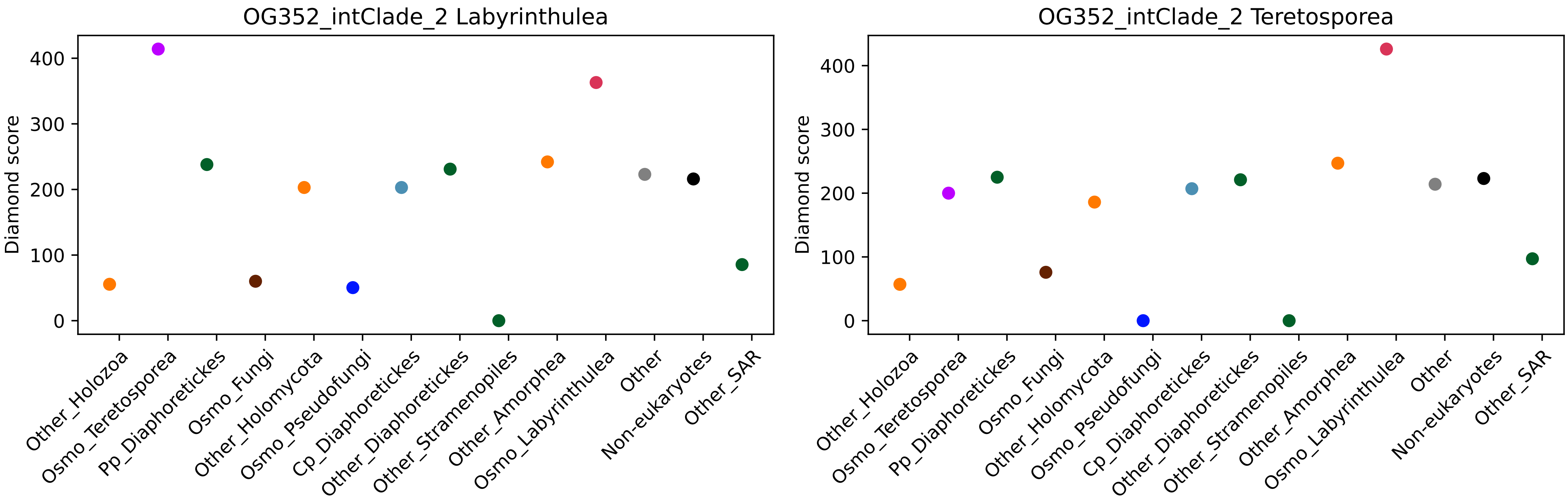

OG352_intClade_2 Labyrinthulea
Diamond score
400
300
200
100
0
Other_Holozoa
Osmo_Teretosporea
Pp_Diaphoretickes
Osmo_Fungi
Other_Holomycota
Osmo_Pseudofungi
Cp_Diaphoretickes
Other_Diaphoretickes
Other_Stramenopiles
Other_Amorphea
Osmo_Labyrinthulea
Other
Non-eukaryotes
Other_SAR
OG352_intClade_2 Teretosporea
Diamond score
400
300
200
100
0
Other_Holozoa
Osmo_Teretosporea
Pp_Diaphoretickes
Osmo_Fungi
Other_Holomycota
Osmo_Pseudofungi
Cp_Diaphoretickes
Other_Diaphoretickes
Other_Stramenopiles
Other_Amorphea
Osmo_Labyrinthulea
Other
Non-eukaryotes
Other_SAR

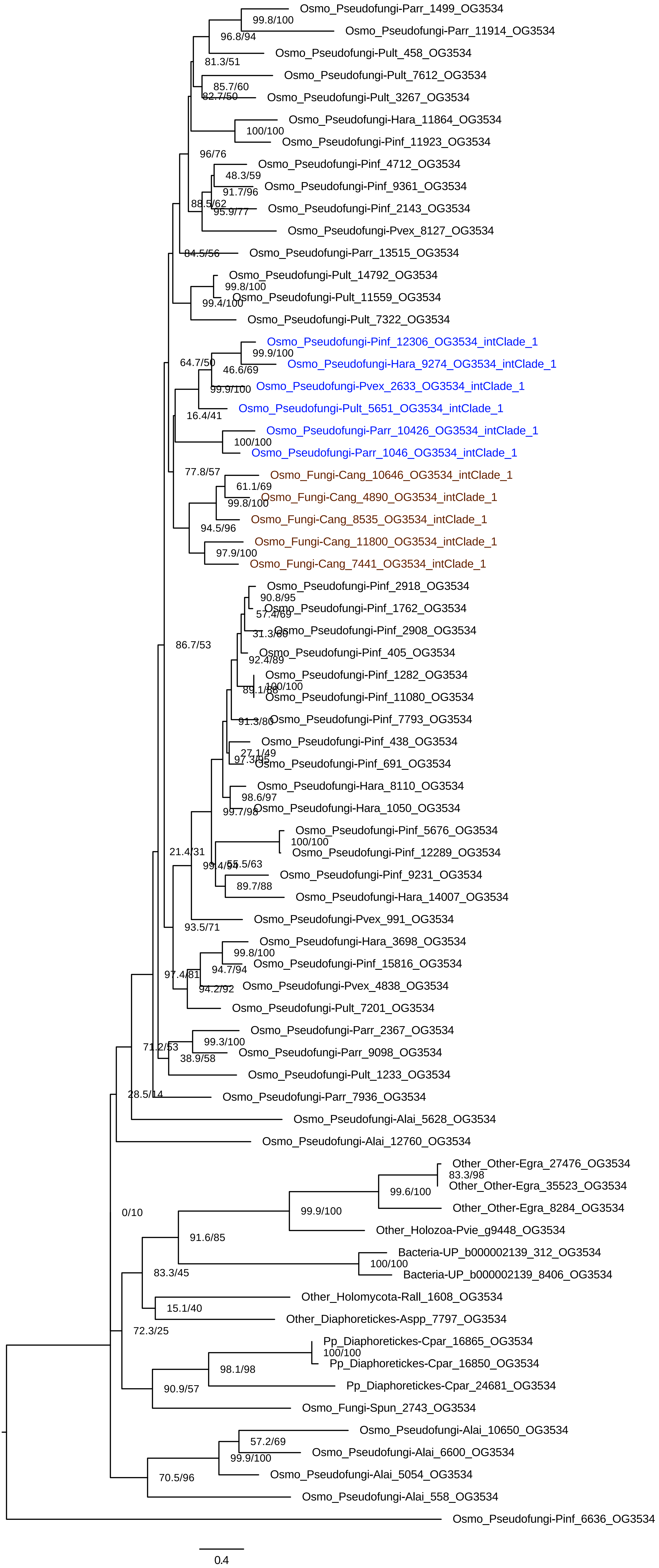

Osmo_Pseudofungi-Parr_1499_OG3534
Osmo_Pseudofungi-Parr_11914_OG3534
Osmo_Pseudofungi-Pult_458_OG3534
Osmo_Pseudofungi-Pult_7612_OG3534
Osmo_Pseudofungi-Pult_3267_OG3534
Osmo_Pseudofungi-Hara_11864_OG3534
Osmo_Pseudofungi-Pinf_11923_OG3534
Osmo_Pseudofungi-Pinf_4712_OG3534
Osmo_Pseudofungi-Pinf_9361_OG3534
Osmo_Pseudofungi-Pinf_2143_OG3534
Osmo_Pseudofungi-Pvex_8127_OG3534
Osmo_Pseudofungi-Parr_13515_OG3534
Osmo_Pseudofungi-Pult_14792_OG3534
Osmo_Pseudofungi-Pult_11559_OG3534
Osmo_Pseudofungi-Pult_7322_OG3534
Osmo_Pseudofungi-Pinf_12306_OG3534_intClade_1
Osmo_Pseudofungi-Hara_9274_OG3534_intClade_1
Osmo_Pseudofungi-Pvex_2633_OG3534_intClade_1
Osmo_Pseudofungi-Pult_5651_OG3534_intClade_1
Osmo_Pseudofungi-Parr_10426_OG3534_intClade_1
Osmo_Pseudofungi-Parr_1046_OG3534_intClade_1
Osmo_Fungi-Cang_10646_OG3534_intClade_1
Osmo_Fungi-Cang_4890_OG3534_intClade_1
Osmo_Fungi-Cang_8535_OG3534_intClade_1
Osmo_Fungi-Cang_11800_OG3534_intClade_1
Osmo_Fungi-Cang_7441_OG3534_intClade_1
Osmo_Pseudofungi-Pinf_2918_OG3534
Osmo_Pseudofungi-Pinf_1762_OG3534
Osmo_Pseudofungi-Pinf_2908_OG3534
Osmo_Pseudofungi-Pinf_405_OG3534
Osmo_Pseudofungi-Pinf_1282_OG3534
Osmo_Pseudofungi-Pinf_11080_OG3534
Osmo_Pseudofungi-Pinf_7793_OG3534
Osmo_Pseudofungi-Pinf_438_OG3534
Osmo_Pseudofungi-Pinf_691_OG3534
Osmo_Pseudofungi-Hara_8110_OG3534
Osmo_Pseudofungi-Hara_1050_OG3534
Osmo_Pseudofungi-Pinf_5676_OG3534
Osmo_Pseudofungi-Pinf_12289_OG3534
Osmo_Pseudofungi-Pinf_9231_OG3534
Osmo_Pseudofungi-Hara_14007_OG3534
Osmo_Pseudofungi-Pvex_991_OG3534
Osmo_Pseudofungi-Hara_3698_OG3534
Osmo_Pseudofungi-Pinf_15816_OG3534
Osmo_Pseudofungi-Pvex_4838_OG3534
Osmo_Pseudofungi-Pult_7201_OG3534
Osmo_Pseudofungi-Parr_2367_OG3534
Osmo_Pseudofungi-Parr_9098_OG3534
Osmo_Pseudofungi-Pult_1233_OG3534
Osmo_Pseudofungi-Parr_7936_OG3534
Osmo_Pseudofungi-Alai_5628_OG3534
Osmo_Pseudofungi-Alai_12760_OG3534
Other_Other-Egra_27476_OG3534
Other_Other-Egra_35523_OG3534
Other_Other-Egra_8284_OG3534
Other_Holozoa-Pvie_g9448_OG3534
Bacteria-UP_b000002139_312_OG3534
Bacteria-UP_b000002139_8406_OG3534
Other_Holomycota-Rall_1608_OG3534
Other_Diaphoretickes-Aspp_7797_OG3534
Pp_Diaphoretickes-Cpar_16865_OG3534
Pp_Diaphoretickes-Cpar_16850_OG3534
Pp_Diaphoretickes-Cpar_24681_OG3534
Osmo_Fungi-Spun_2743_OG3534
Osmo_Pseudofungi-Alai_10650_OG3534
Osmo_Pseudofungi-Alai_6600_OG3534
Osmo_Pseudofungi-Alai_5054_OG3534
Osmo_Pseudofungi-Alai_558_OG3534
Osmo_Pseudofungi-Pinf_6636_OG3534
99.8/100
96.8/94
81.3/51
85.7/60
82.7/50
100/100
96/76
48.3/59
91.7/96
88.5/62
95.9/77
84.5/56
99.8/100
99.4/100
99.9/100
64.7/50
46.6/69
99.9/100
16.4/41
100/100
77.8/57
61.1/69
99.8/100
94.5/96
97.9/100
90.8/95
57.4/69
31.3/60
86.7/53
92.4/89
100/100
89.1/88
91.3/80
27.1/49
97.3/95
98.6/97
99.7/98
100/100
21.4/31
99.4/94
55.5/63
89.7/88
93.5/71
99.8/100
94.7/94
97.4/81
94.2/92
99.3/100
71.2/53
38.9/58
28.5/14
83.3/98
99.6/100
99.9/100
0/10
91.6/85
100/100
83.3/45
15.1/40
72.3/25
100/100
98.1/98
90.9/57
57.2/69
99.9/100
70.5/96
0.4

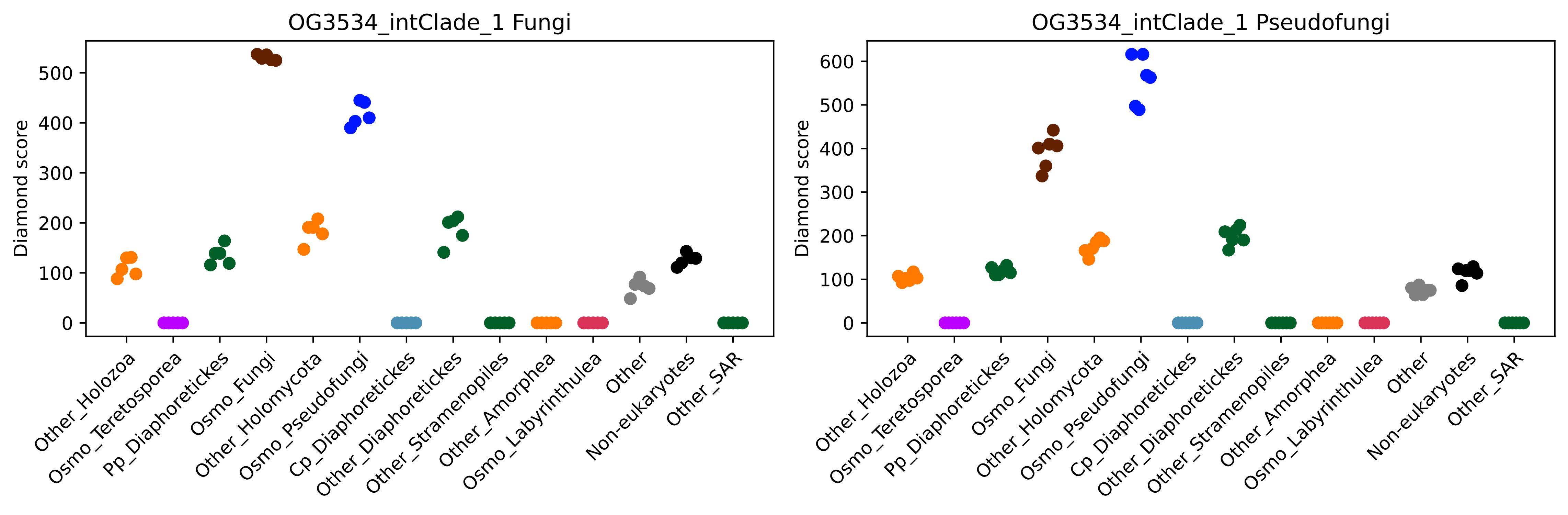

OG3534_intClade_1 Fungi
OG3534_intClade_1 Pseudofungi
Diamond score
0
100
200
300
400
500
600
Other_Holozoa
Osmo_Teretosporea
Pp_Diaphoretickes
Osmo_Fungi
Other_Holomycota
Osmo_Pseudofungi
Cp_Diaphoretickes
Other_Diaphoretickes
Other_Stramenopiles
Other_Amorphea
Osmo_Labyrinthulea
Other
Non-eukaryotes
Other_SAR

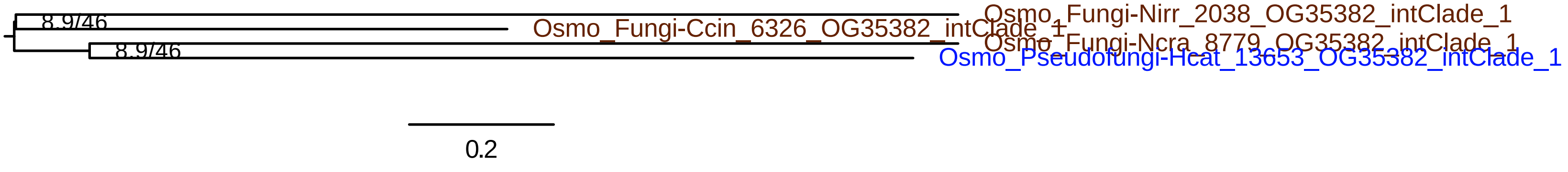
Osmo_Fungi-Nirr_2038_OG35382_intClade_1
8.9/46
Osmo_Fungi-Ccin_6326_OG35382_intClade_1
Osmo_Fungi-Ncra_8779_OG35382_intClade_1
8.9/46
Osmo_Pseudofungi-Hcat_13653_OG35382_intClade_1
0.2

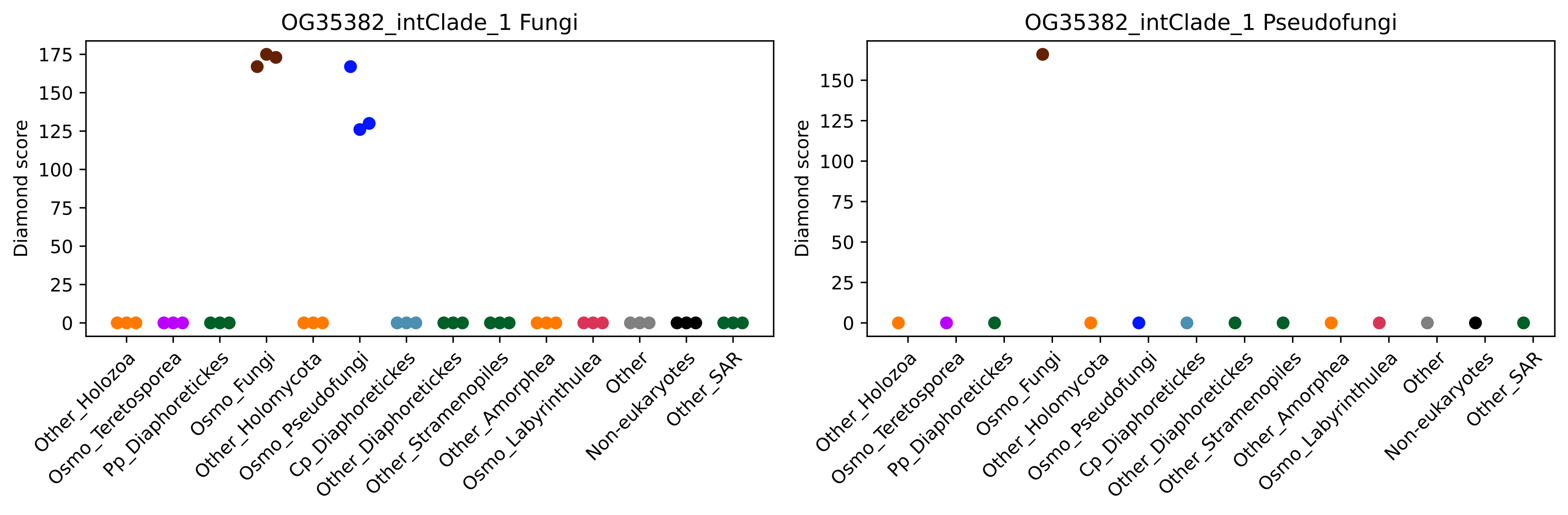
OG35382_intClade_1 Fungi
Diamond score
0
25
50
75
100
125
150
175
Other_Holozoa
Osmo_Teretosporea
Pp_Diaphoretickes
Osmo_Fungi
Other_Holomycota
Osmo_Pseudofungi
Cp_Diaphoretickes
Other_Diaphoretickes
Other_Stramenopiles
Other_Amorphea
Osmo_Labyrinthulea
Other
Non-eukaryotes
Other_SAR
OG35382_intClade_1 Pseudofungi
Diamond score
0
25
50
75
100
125
150
Other_Holozoa
Osmo_Teretosporea
Pp_Diaphoretickes
Osmo_Fungi
Other_Holomycota
Osmo_Pseudofungi
Cp_Diaphoretickes
Other_Diaphoretickes
Other_Stramenopiles
Other_Amorphea
Osmo_Labyrinthulea
Other
Non-eukaryotes
Other_SAR

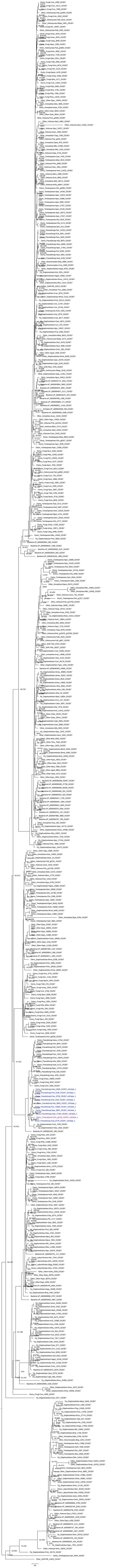

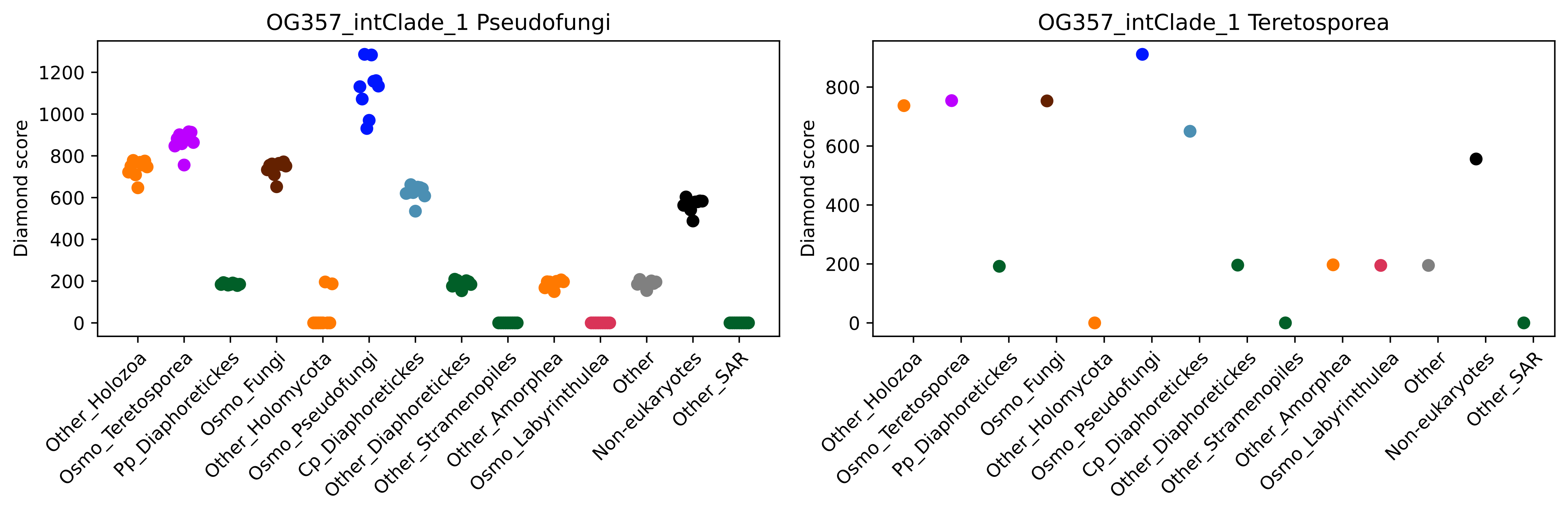

OG357_intClade_1 Pseudofungi
OG357_intClade_1 Teretosporea
Diamond score
0
200
400
600
800
1000
1200
Other_Holozoa
Osmo_Teretosporea
Pp_Diaphoretickes
Osmo_Fungi
Other_Holomycota
Osmo_Pseudofungi
Cp_Diaphoretickes
Other_Diaphoretickes
Other_Stramenopiles
Other_Amorphea
Osmo_Labyrinthulea
Other
Non-eukaryotes
Other_SAR

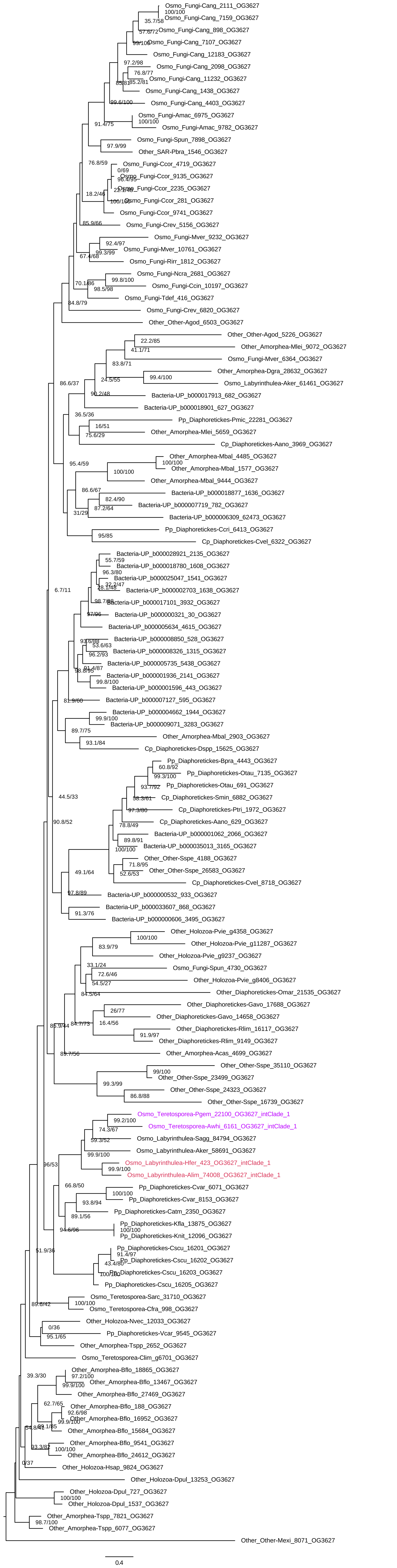

Osmo_Fungi-Cang_2111_OG3627
Osmo_Fungi-Cang_7159_OG3627
Osmo_Fungi-Cang_898_OG3627
Osmo_Fungi-Cang_7107_OG3627
Osmo_Fungi-Cang_12183_OG3627
Osmo_Fungi-Cang_2098_OG3627
Osmo_Fungi-Cang_11232_OG3627
Osmo_Fungi-Cang_1438_OG3627
Osmo_Fungi-Cang_4403_OG3627
Osmo_Fungi-Amac_6975_OG3627
Osmo_Fungi-Amac_9782_OG3627
Osmo_Fungi-Spun_7898_OG3627
Other_SAR-Pbra_1546_OG3627
Osmo_Fungi-Ccor_4719_OG3627
Osmo_Fungi-Ccor_9135_OG3627
Osmo_Fungi-Ccor_2235_OG3627
Osmo_Fungi-Ccor_281_OG3627
Osmo_Fungi-Ccor_9741_OG3627
Osmo_Fungi-Crev_5156_OG3627
Osmo_Fungi-Mver_9232_OG3627
Osmo_Fungi-Mver_10761_OG3627
Osmo_Fungi-Rirr_1812_OG3627
Osmo_Fungi-Ncra_2681_OG3627
Osmo_Fungi-Ccin_10197_OG3627
Osmo_Fungi-Tdef_416_OG3627
Osmo_Fungi-Crev_6820_OG3627
Other_Other-Agod_6503_OG3627
Other_Other-Agod_5226_OG3627
Other_Amorphea-Mlei_9072_OG3627
Osmo_Fungi-Mver_6364_OG3627
Other_Amorphea-Dgra_28632_OG3627
Osmo_Labyrinthulea-Aker_61461_OG3627
Bacteria-UP_b000017913_682_OG3627
Bacteria-UP_b000018901_627_OG3627
Pp_Diaphoretickes-Pmic_22281_OG3627
Other_Amorphea-Mlei_5659_OG3627
Cp_Diaphoretickes-Aano_3969_OG3627
Other_Amorphea-Mbal_4485_OG3627
Other_Amorphea-Mbal_1577_OG3627
Other_Amorphea-Mbal_9444_OG3627
Bacteria-UP_b000018877_1636_OG3627
Bacteria-UP_b000007719_782_OG3627
Bacteria-UP_b000006309_62473_OG3627
Pp_Diaphoretickes-Ccri_6413_OG3627
Cp_Diaphoretickes-Cvel_6322_OG3627
Bacteria-UP_b000028921_2135_OG3627
Bacteria-UP_b000018780_1608_OG3627
Bacteria-UP_b000025047_1541_OG3627
Bacteria-UP_b000002703_1638_OG3627
Bacteria-UP_b000017101_3932_OG3627
Bacteria-UP_b000000321_30_OG3627
Bacteria-UP_b000005634_4615_OG3627
Bacteria-UP_b000008850_528_OG3627
Bacteria-UP_b000008326_1315_OG3627
Bacteria-UP_b000005735_5438_OG3627
Bacteria-UP_b000001936_2141_OG3627
Bacteria-UP_b000001596_443_OG3627
Bacteria-UP_b000007127_595_OG3627
Bacteria-UP_b000004662_1944_OG3627
Bacteria-UP_b000009071_3283_OG3627
Other_Amorphea-Mbal_2903_OG3627
Cp_Diaphoretickes-Dspp_15625_OG3627
Pp_Diaphoretickes-Bpra_4443_OG3627
Pp_Diaphoretickes-Otau_7135_OG3627
Pp_Diaphoretickes-Otau_691_OG3627
Cp_Diaphoretickes-Smin_6882_OG3627
Cp_Diaphoretickes-Ptri_1972_OG3627
Cp_Diaphoretickes-Aano_629_OG3627
Bacteria-UP_b000001062_2066_OG3627
Bacteria-UP_b000035013_3165_OG3627
Other_Other-Sspe_4188_OG3627
Other_Other-Sspe_26583_OG3627
Cp_Diaphoretickes-Cvel_8718_OG3627
Bacteria-UP_b000000532_933_OG3627
Bacteria-UP_b000033607_868_OG3627
Bacteria-UP_b000000606_3495_OG3627
Other_Holozoa-Pvie_g4358_OG3627
Other_Holozoa-Pvie_g11287_OG3627
Other_Holozoa-Pvie_g9237_OG3627
Osmo_Fungi-Spun_4730_OG3627
Other_Holozoa-Pvie_g8406_OG3627
Other_Diaphoretickes-Omar_21535_OG3627
Other_Diaphoretickes-Gavo_17688_OG3627
Other_Diaphoretickes-Gavo_14658_OG3627
Other_Diaphoretickes-Rlim_16117_OG3627
Other_Diaphoretickes-Rlim_9149_OG3627
Other_Amorphea-Acas_4699_OG3627
Other_Other-Sspe_35110_OG3627
Other_Other-Sspe_23499_OG3627
Other_Other-Sspe_24323_OG3627
Other_Other-Sspe_16739_OG3627
Osmo_Teretosporea-Pgem_22100_OG3627_intClade_1
Osmo_Teretosporea-Awhi_6161_OG3627_intClade_1
Osmo_Labyrinthulea-Sagg_84794_OG3627
Osmo_Labyrinthulea-Aker_58691_OG3627
Osmo_Labyrinthulea-Hfer_423_OG3627_intClade_1
Osmo_Labyrinthulea-Alim_74008_OG3627_intClade_1
Pp_Diaphoretickes-Cvar_6071_OG3627
Pp_Diaphoretickes-Cvar_8153_OG3627
Pp_Diaphoretickes-Catm_2350_OG3627
Pp_Diaphoretickes-Kfla_13875_OG3627
Pp_Diaphoretickes-Knit_12096_OG3627
Pp_Diaphoretickes-Cscu_16201_OG3627
Pp_Diaphoretickes-Cscu_16202_OG3627
Pp_Diaphoretickes-Cscu_16203_OG3627
Pp_Diaphoretickes-Cscu_16205_OG3627
Osmo_Teretosporea-Sarc_31710_OG3627
Osmo_Teretosporea-Cfra_998_OG3627
Other_Holozoa-Nvec_12033_OG3627
Pp_Diaphoretickes-Vcar_9545_OG3627
Other_Amorphea-Tspp_2652_OG3627
Osmo_Teretosporea-Clim_g6701_OG3627
Other_Amorphea-Bflo_18865_OG3627
Other_Amorphea-Bflo_13467_OG3627
Other_Amorphea-Bflo_27469_OG3627
Other_Amorphea-Bflo_188_OG3627
Other_Amorphea-Bflo_16952_OG3627
Other_Amorphea-Bflo_15684_OG3627
Other_Amorphea-Bflo_9541_OG3627
Other_Amorphea-Bflo_24612_OG3627
Other_Holozoa-Hsap_9824_OG3627
Other_Holozoa-Dpul_13253_OG3627
Other_Holozoa-Dpul_727_OG3627
Other_Holozoa-Dpul_1537_OG3627
Other_Amorphea-Tspp_7821_OG3627
Other_Amorphea-Tspp_6077_OG3627
Other_Other-Mexi_8071_OG3627
0.4

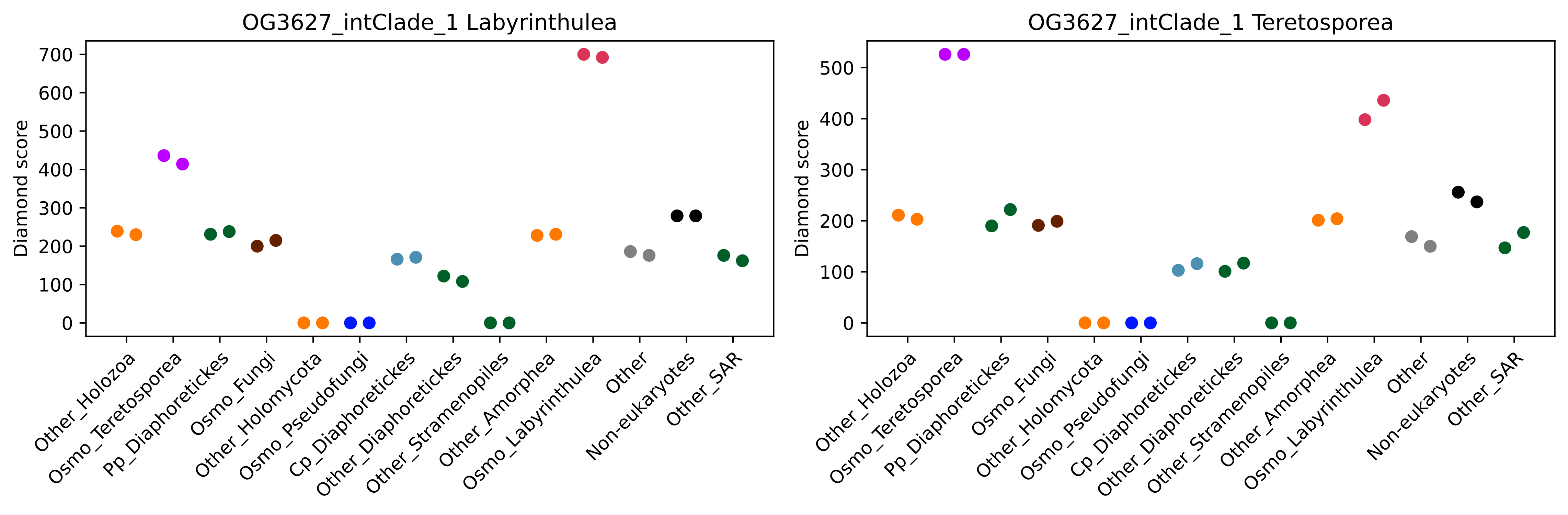

OG3627_intClade_1 Labyrinthulea
OG3627_intClade_1 Teretosporea
Diamond score
Other_Holozoa
Osmo_Teretosporea
Pp_Diaphoretickes
Osmo_Fungi
Other_Holomycota
Osmo_Pseudofungi
Cp_Diaphoretickes
Other_Diaphoretickes
Other_Stramenopiles
Other_Amorphea
Osmo_Labyrinthulea
Other
Non-eukaryotes
Other_SAR

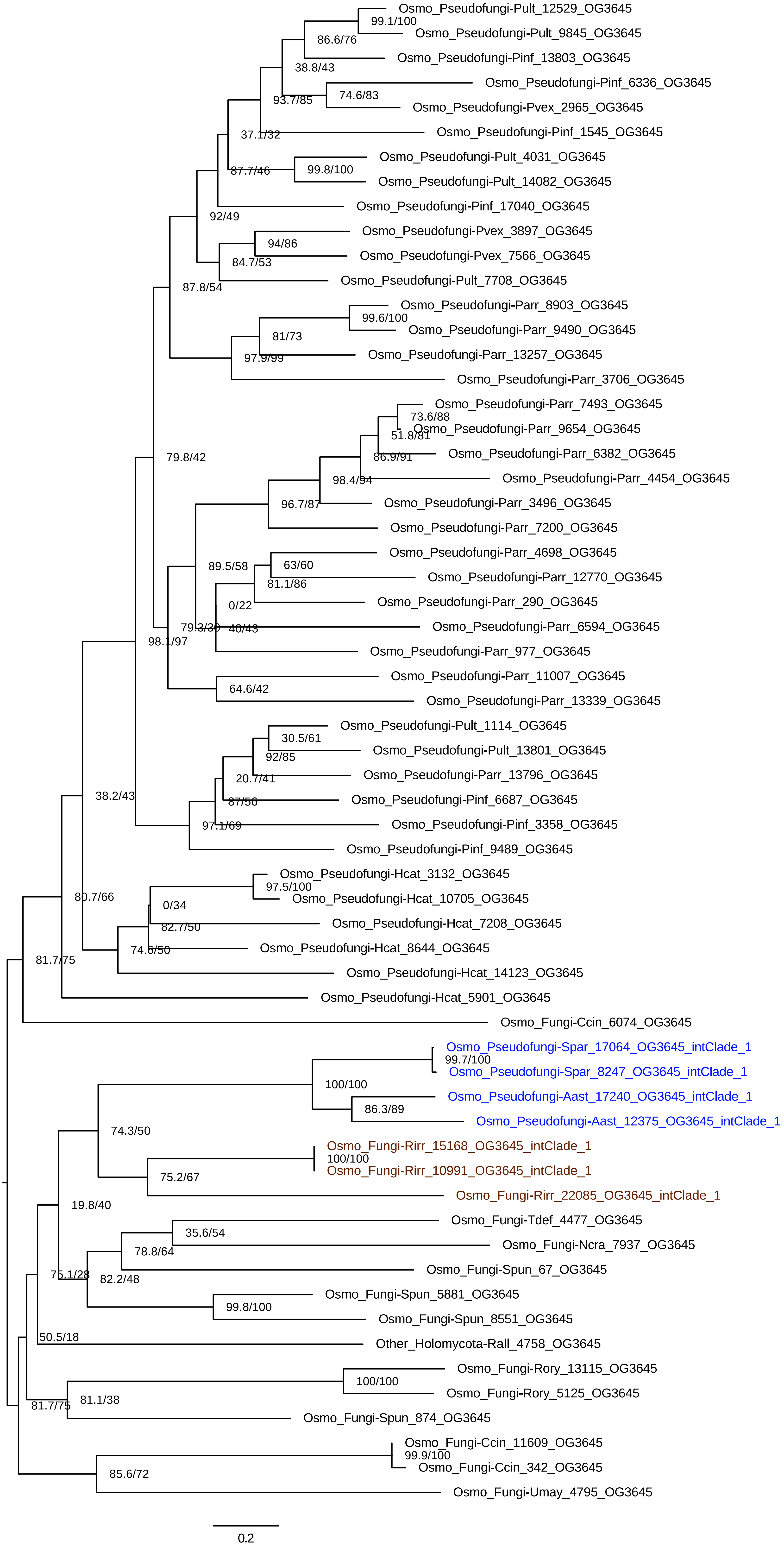

Osmo_Pseudofungi-Pult_12529_OG3645
Osmo_Pseudofungi-Pult_9845_OG3645
Osmo_Pseudofungi-Pinf_13803_OG3645
Osmo_Pseudofungi-Pinf_6336_OG3645
Osmo_Pseudofungi-Pvex_2965_OG3645
Osmo_Pseudofungi-Pinf_1545_OG3645
Osmo_Pseudofungi-Pult_4031_OG3645
Osmo_Pseudofungi-Pult_14082_OG3645
Osmo_Pseudofungi-Pinf_17040_OG3645
Osmo_Pseudofungi-Pvex_3897_OG3645
Osmo_Pseudofungi-Pvex_7566_OG3645
Osmo_Pseudofungi-Pult_7708_OG3645
Osmo_Pseudofungi-Parr_8903_OG3645
Osmo_Pseudofungi-Parr_9490_OG3645
Osmo_Pseudofungi-Parr_13257_OG3645
Osmo_Pseudofungi-Parr_3706_OG3645
Osmo_Pseudofungi-Parr_7493_OG3645
Osmo_Pseudofungi-Parr_9654_OG3645
Osmo_Pseudofungi-Parr_6382_OG3645
Osmo_Pseudofungi-Parr_4454_OG3645
Osmo_Pseudofungi-Parr_3496_OG3645
Osmo_Pseudofungi-Parr_7200_OG3645
Osmo_Pseudofungi-Parr_4698_OG3645
Osmo_Pseudofungi-Parr_12770_OG3645
Osmo_Pseudofungi-Parr_290_OG3645
Osmo_Pseudofungi-Parr_6594_OG3645
Osmo_Pseudofungi-Parr_977_OG3645
Osmo_Pseudofungi-Parr_11007_OG3645
Osmo_Pseudofungi-Parr_13339_OG3645
Osmo_Pseudofungi-Pult_1114_OG3645
Osmo_Pseudofungi-Pult_13801_OG3645
Osmo_Pseudofungi-Parr_13796_OG3645
Osmo_Pseudofungi-Pinf_6687_OG3645
Osmo_Pseudofungi-Pinf_3358_OG3645
Osmo_Pseudofungi-Pinf_9489_OG3645
Osmo_Pseudofungi-Hcat_3132_OG3645
Osmo_Pseudofungi-Hcat_10705_OG3645
Osmo_Pseudofungi-Hcat_7208_OG3645
Osmo_Pseudofungi-Hcat_8644_OG3645
Osmo_Pseudofungi-Hcat_14123_OG3645
Osmo_Pseudofungi-Hcat_5901_OG3645
Osmo_Fungi-Ccin_6074_OG3645
Osmo_Pseudofungi-Spar_17064_OG3645_intClade_1
Osmo_Pseudofungi-Spar_8247_OG3645_intClade_1
Osmo_Pseudofungi-Aast_17240_OG3645_intClade_1
Osmo_Pseudofungi-Aast_12375_OG3645_intClade_1
Osmo_Fungi-Rirr_15168_OG3645_intClade_1
Osmo_Fungi-Rirr_10991_OG3645_intClade_1
Osmo_Fungi-Rirr_22085_OG3645_intClade_1
Osmo_Fungi-Tdef_4477_OG3645
Osmo_Fungi-Ncra_7937_OG3645
Osmo_Fungi-Spun_67_OG3645
Osmo_Fungi-Spun_5881_OG3645
Osmo_Fungi-Spun_8551_OG3645
Other_Holomycota-Rall_4758_OG3645
Osmo_Fungi-Rory_13115_OG3645
Osmo_Fungi-Rory_5125_OG3645
Osmo_Fungi-Spun_874_OG3645
Osmo_Fungi-Ccin_11609_OG3645
Osmo_Fungi-Ccin_342_OG3645
Osmo_Fungi-Umay_4795_OG3645
0.2

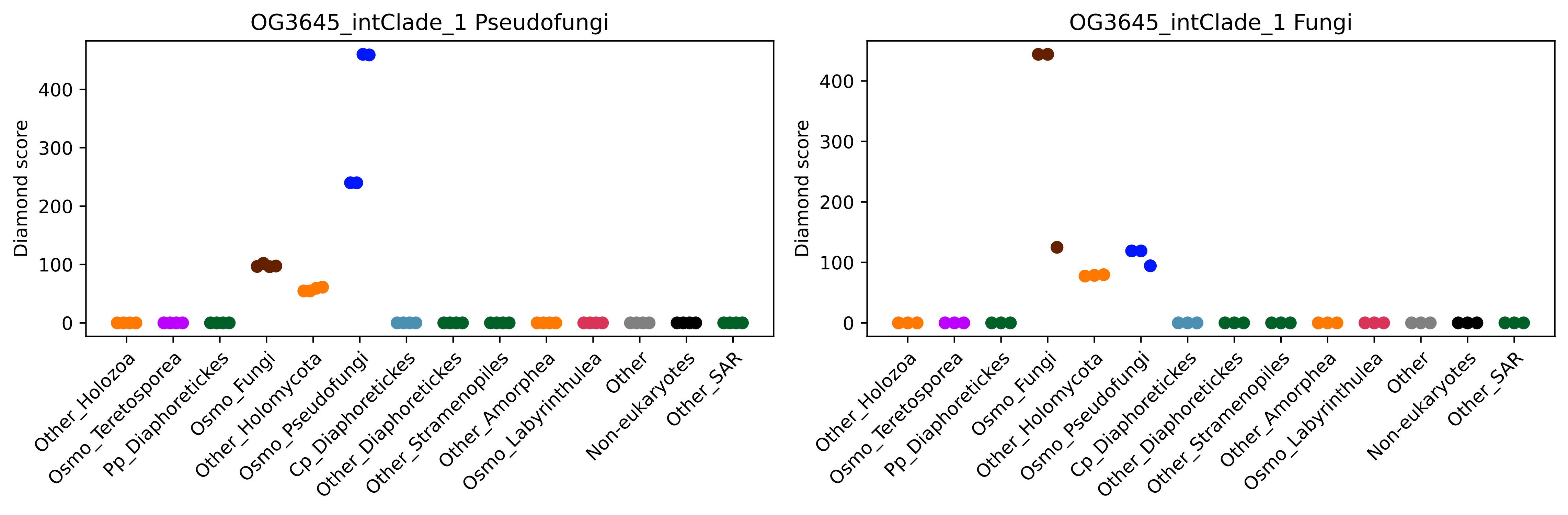
OG3645_intClade_1 Pseudofungi
OG3645_intClade_1 Fungi
Diamond score
0
100
200
300
400
Other_Holozoa
Osmo_Teretosporea
Pp_Diaphoretickes
Osmo_Fungi
Other_Holomycota
Osmo_Pseudofungi
Cp_Diaphoretickes
Other_Diaphoretickes
Other_Stramenopiles
Other_Amorphea
Osmo_Labyrinthulea
Other
Non-eukaryotes
Other_SAR

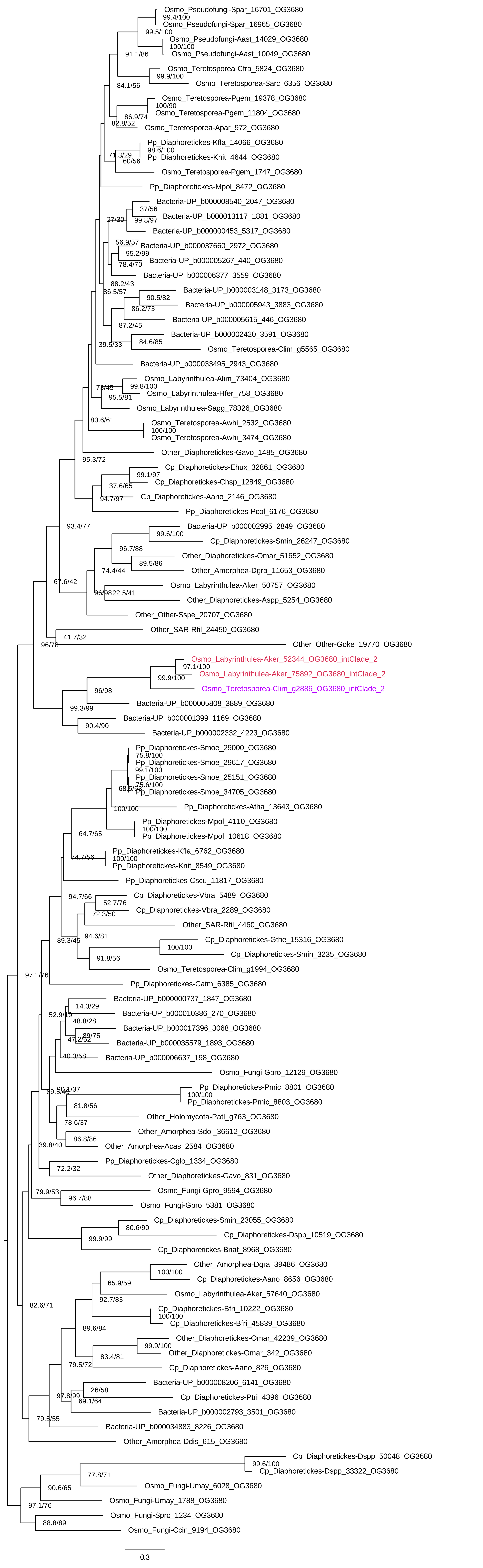
Osmo_Pseudofungi-Spar_16701_OG3680
Osmo_Pseudofungi-Spar_16965_OG3680
Osmo_Pseudofungi-Aast_14029_OG3680
Osmo_Pseudofungi-Aast_10049_OG3680
Osmo_Teretosporea-Cfra_5824_OG3680
Osmo_Teretosporea-Sarc_6356_OG3680
Osmo_Teretosporea-Pgem_19378_OG3680
Osmo_Teretosporea-Pgem_11804_OG3680
Osmo_Teretosporea-Apar_972_OG3680
Pp_Diaphoretickes-Kfla_14066_OG3680
Pp_Diaphoretickes-Knit_4644_OG3680
Osmo_Teretosporea-Pgem_1747_OG3680
Pp_Diaphoretickes-Mpol_8472_OG3680
Bacteria-UP_b000008540_2047_OG3680
Bacteria-UP_b000013117_1881_OG3680
Bacteria-UP_b000000453_5317_OG3680
Bacteria-UP_b000037660_2972_OG3680
Bacteria-UP_b000005267_440_OG3680
Bacteria-UP_b000006377_3559_OG3680
Bacteria-UP_b000003148_3173_OG3680
Bacteria-UP_b000005943_3883_OG3680
Bacteria-UP_b000005615_446_OG3680
Bacteria-UP_b000002420_3591_OG3680
Osmo_Teretosporea-Clim_g5565_OG3680
Bacteria-UP_b000033495_2943_OG3680
Osmo_Labyrinthulea-Alim_73404_OG3680
Osmo_Labyrinthulea-Hfer_758_OG3680
Osmo_Labyrinthulea-Sagg_78326_OG3680
Osmo_Teretosporea-Awhi_2532_OG3680
Osmo_Teretosporea-Awhi_3474_OG3680
Other_Diaphoretickes-Gavo_1485_OG3680
Cp_Diaphoretickes-Ehux_32861_OG3680
Cp_Diaphoretickes-Chsp_12849_OG3680
Cp_Diaphoretickes-Aano_2146_OG3680
Pp_Diaphoretickes-Pcol_6176_OG3680
Bacteria-UP_b000002995_2849_OG3680
Cp_Diaphoretickes-Smin_26247_OG3680
Other_Diaphoretickes-Omar_51652_OG3680
Other_Amorphea-Dgra_11653_OG3680
Osmo_Labyrinthulea-Aker_50757_OG3680
Other_Diaphoretickes-Aspp_5254_OG3680
Other_Other-Sspe_20707_OG3680
Other_SAR-Rfil_24450_OG3680
Other_Other-Goke_19770_OG3680
Osmo_Labyrinthulea-Aker_52344_OG3680_intClade_2
Osmo_Labyrinthulea-Aker_75892_OG3680_intClade_2
Osmo_Teretosporea-Clim_g2886_OG3680_intClade_2
Bacteria-UP_b000005808_3889_OG3680
Bacteria-UP_b000001399_1169_OG3680
Bacteria-UP_b000002332_4223_OG3680
Pp_Diaphoretickes-Smoe_29000_OG3680
Pp_Diaphoretickes-Smoe_29617_OG3680
Pp_Diaphoretickes-Smoe_25151_OG3680
Pp_Diaphoretickes-Smoe_34705_OG3680
Pp_Diaphoretickes-Atha_13643_OG3680
Pp_Diaphoretickes-Mpol_4110_OG3680
Pp_Diaphoretickes-Mpol_10618_OG3680
Pp_Diaphoretickes-Kfla_6762_OG3680
Pp_Diaphoretickes-Knit_8549_OG3680
Pp_Diaphoretickes-Cscu_11817_OG3680
Cp_Diaphoretickes-Vbra_5489_OG3680
Cp_Diaphoretickes-Vbra_2289_OG3680
Other_SAR-Rfil_4460_OG3680
Cp_Diaphoretickes-Gthe_15316_OG3680
Cp_Diaphoretickes-Smin_3235_OG3680
Osmo_Teretosporea-Clim_g1994_OG3680
Pp_Diaphoretickes-Catm_6385_OG3680
Bacteria-UP_b000000737_1847_OG3680
Bacteria-UP_b000010386_270_OG3680
Bacteria-UP_b000017396_3068_OG3680
Bacteria-UP_b000035579_1893_OG3680
Bacteria-UP_b000006637_198_OG3680
Osmo_Fungi-Gpro_12129_OG3680
Pp_Diaphoretickes-Pmic_8801_OG3680
Pp_Diaphoretickes-Pmic_8803_OG3680
Other_Holomycota-Patl_g763_OG3680
Other_Amorphea-Sdol_36612_OG3680
Other_Amorphea-Acas_2584_OG3680
Pp_Diaphoretickes-Cglo_1334_OG3680
Other_Diaphoretickes-Gavo_831_OG3680
Osmo_Fungi-Gpro_9594_OG3680
Osmo_Fungi-Gpro_5381_OG3680
Cp_Diaphoretickes-Smin_23055_OG3680
Cp_Diaphoretickes-Dspp_10519_OG3680
Cp_Diaphoretickes-Bnat_8968_OG3680
Other_Amorphea-Dgra_39486_OG3680
Cp_Diaphoretickes-Aano_8656_OG3680
Osmo_Labyrinthulea-Aker_57640_OG3680
Cp_Diaphoretickes-Bfri_10222_OG3680
Cp_Diaphoretickes-Bfri_45839_OG3680
Other_Diaphoretickes-Omar_42239_OG3680
Other_Diaphoretickes-Omar_342_OG3680
Cp_Diaphoretickes-Aano_826_OG3680
Bacteria-UP_b000008206_6141_OG3680
Cp_Diaphoretickes-Ptri_4396_OG3680
Bacteria-UP_b000002793_3501_OG3680
Bacteria-UP_b000034883_8226_OG3680
Other_Amorphea-Ddis_615_OG3680
Cp_Diaphoretickes-Dspp_50048_OG3680
Cp_Diaphoretickes-Dspp_33322_OG3680
Osmo_Fungi-Umay_6028_OG3680
Osmo_Fungi-Umay_1788_OG3680
Osmo_Fungi-Spro_1234_OG3680
Osmo_Fungi-Ccin_9194_OG3680
0.3

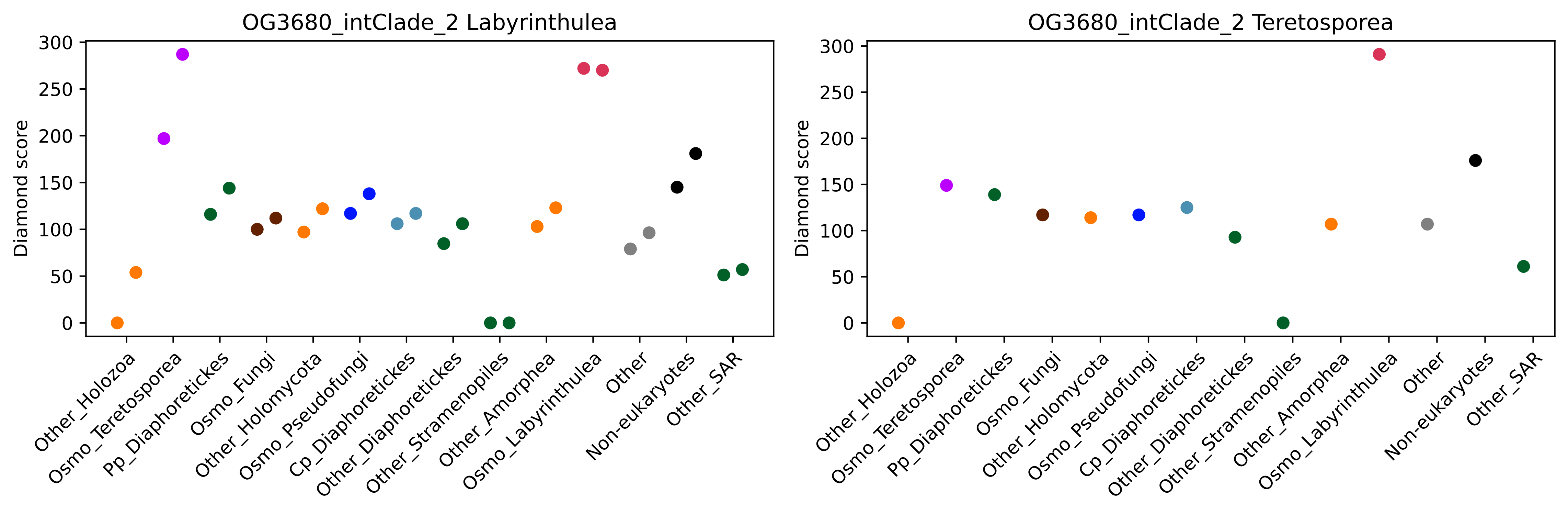

OG3680_intClade_2 Labyrinthulea
Diamond score
0
50
100
150
200
250
300
Other_Holozoa
Osmo_Teretosporea
Pp_Diaphoretickes
Osmo_Fungi
Other_Holomycota
Osmo_Pseudofungi
Cp_Diaphoretickes
Other_Diaphoretickes
Other_Stramenopiles
Other_Amorphea
Osmo_Labyrinthulea
Other
Non-eukaryotes
Other_SAR
OG3680_intClade_2 Teretosporea
Diamond score
0
50
100
150
200
250
300
Other_Holozoa
Osmo_Teretosporea
Pp_Diaphoretickes
Osmo_Fungi
Other_Holomycota
Osmo_Pseudofungi
Cp_Diaphoretickes
Other_Diaphoretickes
Other_Stramenopiles
Other_Amorphea
Osmo_Labyrinthulea
Other
Non-eukaryotes
Other_SAR

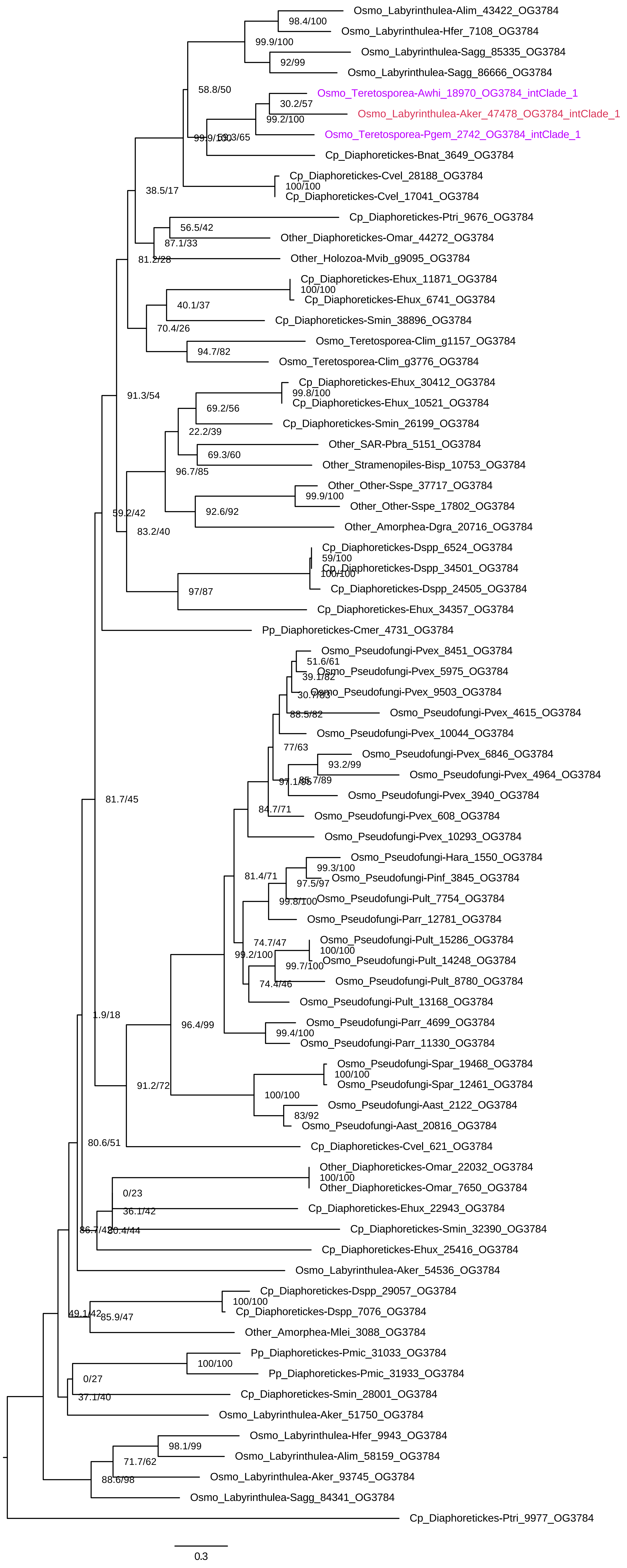

Osmo_Labyrinthulea-Alim_43422_OG3784
98.4/100
Osmo_Labyrinthulea-Hfer_7108_OG3784
99.9/100
Osmo_Labyrinthulea-Sagg_85335_OG3784
92/99
Osmo_Labyrinthulea-Sagg_86666_OG3784
58.8/50
Osmo_Teretosporea-Awhi_18970_OG3784_intClade_1
30.2/57
Osmo_Labyrinthulea-Aker_47478_OG3784_intClade_1
99.2/100
99.9/100
50.3/65
Osmo_Teretosporea-Pgem_2742_OG3784_intClade_1
Cp_Diaphoretickes-Bnat_3649_OG3784
Cp_Diaphoretickes-Cvel_28188_OG3784
100/100
Cp_Diaphoretickes-Cvel_17041_OG3784
38.5/17
Cp_Diaphoretickes-Ptri_9676_OG3784
56.5/42
Other_Diaphoretickes-Omar_44272_OG3784
87.1/33
81.2/28
Other_Holozoa-Mvib_g9095_OG3784
Cp_Diaphoretickes-Ehux_11871_OG3784
100/100
Cp_Diaphoretickes-Ehux_6741_OG3784
40.1/37
Cp_Diaphoretickes-Smin_38896_OG3784
70.4/26
Osmo_Teretosporea-Clim_g1157_OG3784
94.7/82
Osmo_Teretosporea-Clim_g3776_OG3784
91.3/54
Cp_Diaphoretickes-Ehux_30412_OG3784
99.8/100
Cp_Diaphoretickes-Ehux_10521_OG3784
69.2/56
Cp_Diaphoretickes-Smin_26199_OG3784
22.2/39
Other_SAR-Pbra_5151_OG3784
69.3/60
Other_Stramenopiles-Bisp_10753_OG3784
96.7/85
Other_Other-Sspe_37717_OG3784
99.9/100
Other_Other-Sspe_17802_OG3784
92.6/92
59.2/42
Other_Amorphea-Dgra_20716_OG3784
83.2/40
Cp_Diaphoretickes-Dspp_6524_OG3784
59/100
Cp_Diaphoretickes-Dspp_34501_OG3784
100/100
Cp_Diaphoretickes-Dspp_24505_OG3784
97/87
Cp_Diaphoretickes-Ehux_34357_OG3784
Pp_Diaphoretickes-Cmer_4731_OG3784
Osmo_Pseudofungi-Pvex_8451_OG3784
51.6/61
Osmo_Pseudofungi-Pvex_5975_OG3784
39.1/82
Osmo_Pseudofungi-Pvex_9503_OG3784
30.7/83
Osmo_Pseudofungi-Pvex_4615_OG3784
88.5/82
Osmo_Pseudofungi-Pvex_10044_OG3784
77/63
Osmo_Pseudofungi-Pvex_6846_OG3784
93.2/99
Osmo_Pseudofungi-Pvex_4964_OG3784
97.1/85
85.7/89
Osmo_Pseudofungi-Pvex_3940_OG3784
81.7/45
84.7/71
Osmo_Pseudofungi-Pvex_608_OG3784
Osmo_Pseudofungi-Pvex_10293_OG3784
Osmo_Pseudofungi-Hara_1550_OG3784
99.3/100
81.4/71
Osmo_Pseudofungi-Pinf_3845_OG3784
97.5/97
99.8/100
Osmo_Pseudofungi-Pult_7754_OG3784
Osmo_Pseudofungi-Parr_12781_OG3784
74.7/47
Osmo_Pseudofungi-Pult_15286_OG3784
100/100
99.2/100
Osmo_Pseudofungi-Pult_14248_OG3784
99.7/100
74.4/46
Osmo_Pseudofungi-Pult_8780_OG3784
Osmo_Pseudofungi-Pult_13168_OG3784
1.9/18
96.4/99
Osmo_Pseudofungi-Parr_4699_OG3784
99.4/100
Osmo_Pseudofungi-Parr_11330_OG3784
Osmo_Pseudofungi-Spar_19468_OG3784
100/100
91.2/72
Osmo_Pseudofungi-Spar_12461_OG3784
100/100
Osmo_Pseudofungi-Aast_2122_OG3784
83/92
Osmo_Pseudofungi-Aast_20816_OG3784
80.6/51
Cp_Diaphoretickes-Cvel_621_OG3784
Other_Diaphoretickes-Omar_22032_OG3784
100/100
0/23
Other_Diaphoretickes-Omar_7650_OG3784
36.1/42
Cp_Diaphoretickes-Ehux_22943_OG3784
86.7/42
30.4/44
Cp_Diaphoretickes-Smin_32390_OG3784
Cp_Diaphoretickes-Ehux_25416_OG3784
Osmo_Labyrinthulea-Aker_54536_OG3784
Cp_Diaphoretickes-Dspp_29057_OG3784
100/100
49.1/42
Cp_Diaphoretickes-Dspp_7076_OG3784
85.9/47
Other_Amorphea-Mlei_3088_OG3784
Pp_Diaphoretickes-Pmic_31033_OG3784
100/100
Pp_Diaphoretickes-Pmic_31933_OG3784
0/27
Cp_Diaphoretickes-Smin_28001_OG3784
37.1/40
Osmo_Labyrinthulea-Aker_51750_OG3784
Osmo_Labyrinthulea-Hfer_9943_OG3784
98.1/99
Osmo_Labyrinthulea-Alim_58159_OG3784
71.7/62
Osmo_Labyrinthulea-Aker_93745_OG3784
88.6/98
Osmo_Labyrinthulea-Sagg_84341_OG3784
Cp_Diaphoretickes-Ptri_9977_OG3784
0.3

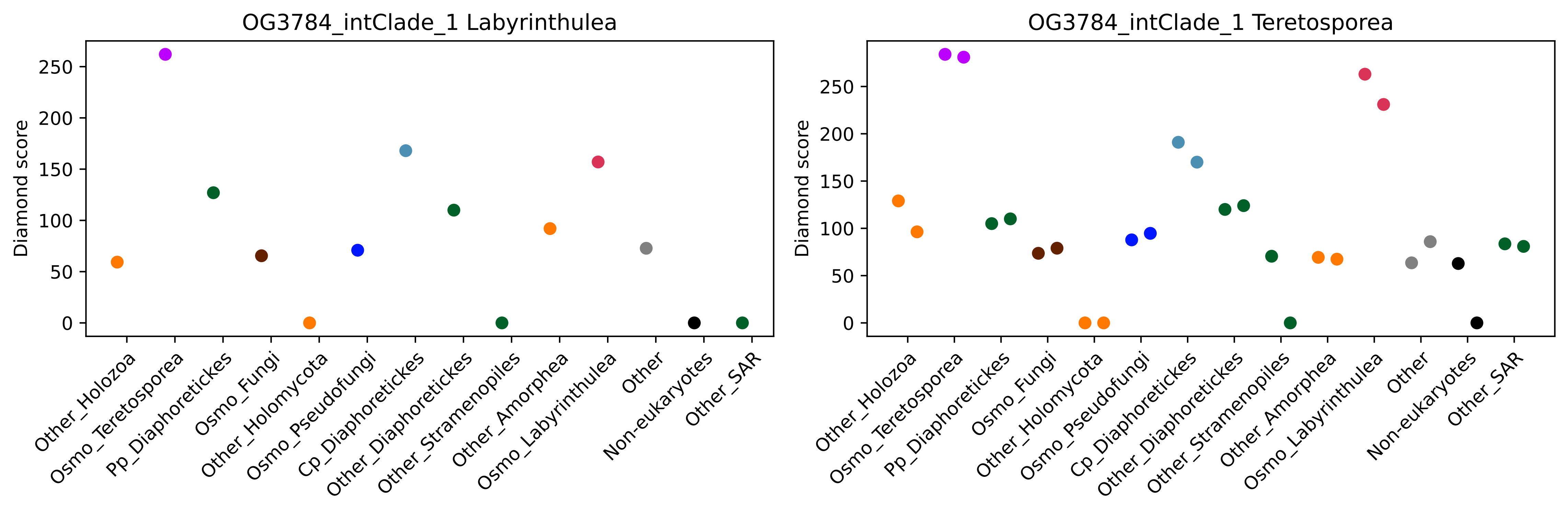
OG3784_intClade_1 Labyrinthulea
OG3784_intClade_1 Teretosporea
Diamond score
0
50
100
150
200
250
Other_Holozoa
Osmo_Teretosporea
Pp_Diaphoretickes
Osmo_Fungi
Other_Holomycota
Osmo_Pseudofungi
Cp_Diaphoretickes
Other_Diaphoretickes
Other_Stramenopiles
Other_Amorphea
Osmo_Labyrinthulea
Other
Non-eukaryotes
Other_SAR

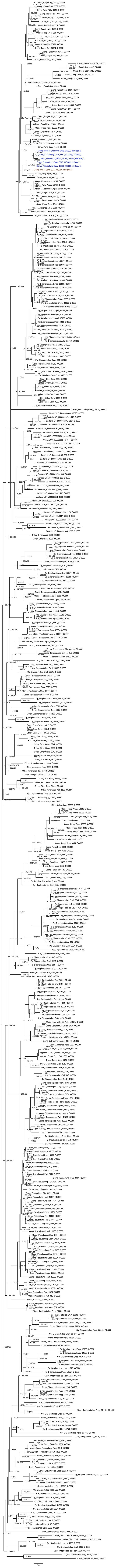

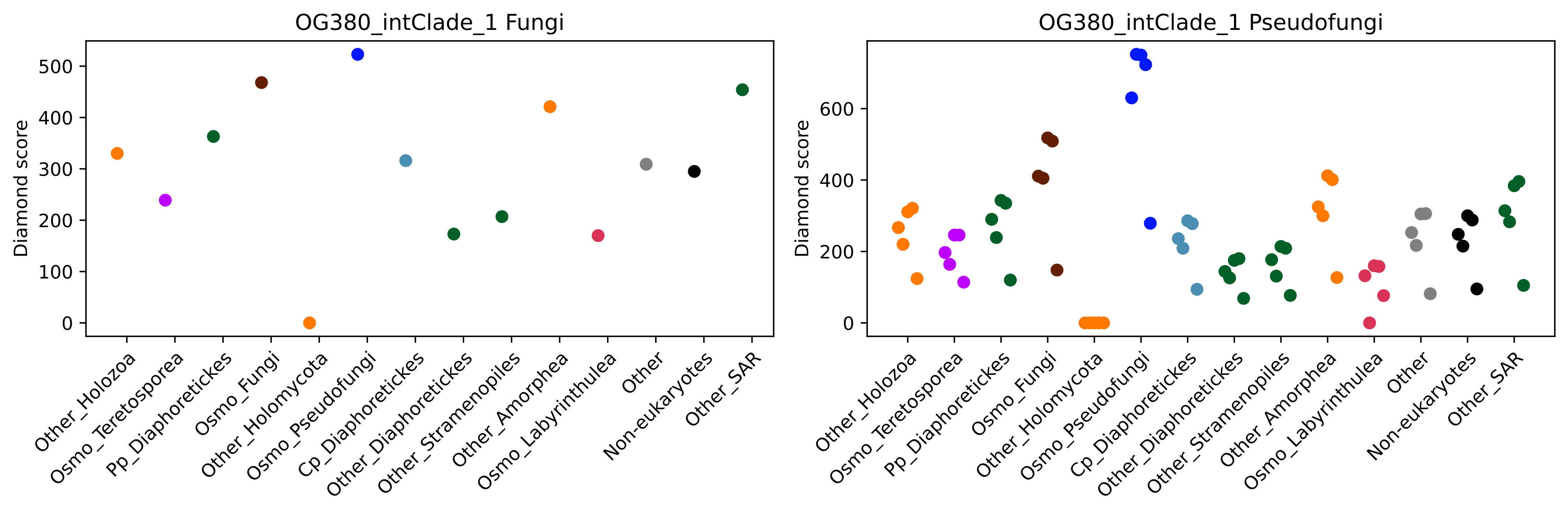

OG380_intClade_1 Fungi
Diamond score
0
100
200
300
400
500
Other_Holozoa
Osmo_Teretosporea
Pp_Diaphoretickes
Osmo_Fungi
Other_Holomycota
Osmo_Pseudofungi
Cp_Diaphoretickes
Other_Diaphoretickes
Other_Stramenopiles
Other_Amorphea
Osmo_Labyrinthulea
Other
Non-eukaryotes
Other_SAR
OG380_intClade_1 Pseudofungi
Diamond score
0
200
400
600
Other_Holozoa
Osmo_Teretosporea
Pp_Diaphoretickes
Osmo_Fungi
Other_Holomycota
Osmo_Pseudofungi
Cp_Diaphoretickes
Other_Diaphoretickes
Other_Stramenopiles
Other_Amorphea
Osmo_Labyrinthulea
Other
Non-eukaryotes
Other_SAR

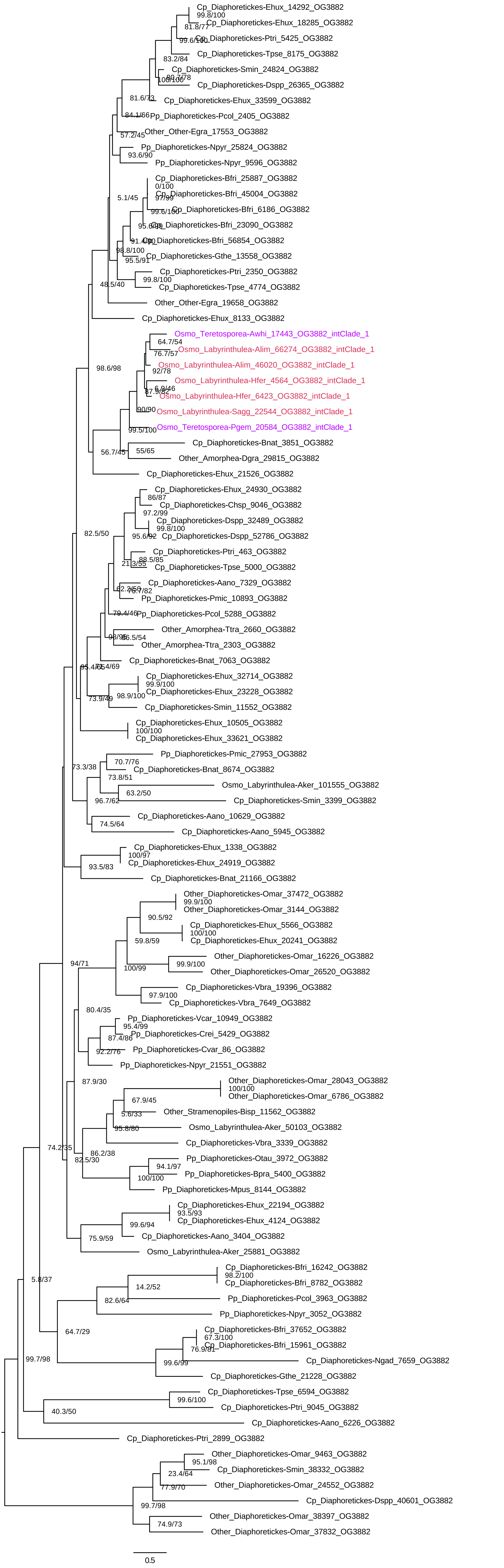

Cp_Diaphoretickes-Ehux_14292_OG3882
Cp_Diaphoretickes-Ehux_18285_OG3882
Cp_Diaphoretickes-Ptri_5425_OG3882
Cp_Diaphoretickes-Tpse_8175_OG3882
Cp_Diaphoretickes-Smin_24824_OG3882
Cp_Diaphoretickes-Dspp_26365_OG3882
Cp_Diaphoretickes-Ehux_33599_OG3882
Pp_Diaphoretickes-Pcol_2405_OG3882
Other_Other-Egra_17553_OG3882
Pp_Diaphoretickes-Npyr_25824_OG3882
Pp_Diaphoretickes-Npyr_9596_OG3882
Cp_Diaphoretickes-Bfri_25887_OG3882
Cp_Diaphoretickes-Bfri_45004_OG3882
Cp_Diaphoretickes-Bfri_6186_OG3882
Cp_Diaphoretickes-Bfri_23090_OG3882
Cp_Diaphoretickes-Bfri_56854_OG3882
Cp_Diaphoretickes-Gthe_13558_OG3882
Cp_Diaphoretickes-Ptri_2350_OG3882
Cp_Diaphoretickes-Tpse_4774_OG3882
Other_Other-Egra_19658_OG3882
Cp_Diaphoretickes-Ehux_8133_OG3882
Osmo_Teretosporea-Awhi_17443_OG3882_intClade_1
Osmo_Labyrinthulea-Alim_66274_OG3882_intClade_1
Osmo_Labyrinthulea-Alim_46020_OG3882_intClade_1
Osmo_Labyrinthulea-Hfer_4564_OG3882_intClade_1
Osmo_Labyrinthulea-Hfer_6423_OG3882_intClade_1
Osmo_Labyrinthulea-Sagg_22544_OG3882_intClade_1
Osmo_Teretosporea-Pgem_20584_OG3882_intClade_1
Cp_Diaphoretickes-Bnat_3851_OG3882
Other_Amorphea-Dgra_29815_OG3882
Cp_Diaphoretickes-Ehux_21526_OG3882
Cp_Diaphoretickes-Ehux_24930_OG3882
Cp_Diaphoretickes-Chsp_9046_OG3882
Cp_Diaphoretickes-Dspp_32489_OG3882
Cp_Diaphoretickes-Dspp_52786_OG3882
Cp_Diaphoretickes-Ptri_463_OG3882
Cp_Diaphoretickes-Tpse_5000_OG3882
Cp_Diaphoretickes-Aano_7329_OG3882
Pp_Diaphoretickes-Pmic_10893_OG3882
Pp_Diaphoretickes-Pcol_5288_OG3882
Other_Amorphea-Ttra_2660_OG3882
Other_Amorphea-Ttra_2303_OG3882
Cp_Diaphoretickes-Bnat_7063_OG3882
Cp_Diaphoretickes-Ehux_32714_OG3882
Cp_Diaphoretickes-Ehux_23228_OG3882
Cp_Diaphoretickes-Smin_11552_OG3882
Cp_Diaphoretickes-Ehux_10505_OG3882
Cp_Diaphoretickes-Ehux_33621_OG3882
Pp_Diaphoretickes-Pmic_27953_OG3882
Cp_Diaphoretickes-Bnat_8674_OG3882
Osmo_Labyrinthulea-Aker_101555_OG3882
Cp_Diaphoretickes-Smin_3399_OG3882
Cp_Diaphoretickes-Aano_10629_OG3882
Cp_Diaphoretickes-Aano_5945_OG3882
Cp_Diaphoretickes-Ehux_1338_OG3882
Cp_Diaphoretickes-Ehux_24919_OG3882
Cp_Diaphoretickes-Bnat_21166_OG3882
Other_Diaphoretickes-Omar_37472_OG3882
Other_Diaphoretickes-Omar_3144_OG3882
Cp_Diaphoretickes-Ehux_5566_OG3882
Cp_Diaphoretickes-Ehux_20241_OG3882
Other_Diaphoretickes-Omar_16226_OG3882
Other_Diaphoretickes-Omar_26520_OG3882
Cp_Diaphoretickes-Vbra_19396_OG3882
Cp_Diaphoretickes-Vbra_7649_OG3882
Pp_Diaphoretickes-Vcar_10949_OG3882
Pp_Diaphoretickes-Crei_5429_OG3882
Pp_Diaphoretickes-Cvar_86_OG3882
Pp_Diaphoretickes-Npyr_21551_OG3882
Other_Diaphoretickes-Omar_28043_OG3882
Other_Diaphoretickes-Omar_6786_OG3882
Other_Stramenopiles-Bisp_11562_OG3882
Osmo_Labyrinthulea-Aker_50103_OG3882
Cp_Diaphoretickes-Vbra_3339_OG3882
Pp_Diaphoretickes-Otau_3972_OG3882
Pp_Diaphoretickes-Bpra_5400_OG3882
Pp_Diaphoretickes-Mpus_8144_OG3882
Cp_Diaphoretickes-Ehux_22194_OG3882
Cp_Diaphoretickes-Ehux_4124_OG3882
Cp_Diaphoretickes-Aano_3404_OG3882
Osmo_Labyrinthulea-Aker_25881_OG3882
Cp_Diaphoretickes-Bfri_16242_OG3882
Cp_Diaphoretickes-Bfri_8782_OG3882
Pp_Diaphoretickes-Pcol_3963_OG3882
Pp_Diaphoretickes-Npyr_3052_OG3882
Cp_Diaphoretickes-Bfri_37652_OG3882
Cp_Diaphoretickes-Bfri_15961_OG3882
Cp_Diaphoretickes-Ngad_7659_OG3882
Cp_Diaphoretickes-Gthe_21228_OG3882
Cp_Diaphoretickes-Tpse_6594_OG3882
Cp_Diaphoretickes-Ptri_9045_OG3882
Cp_Diaphoretickes-Aano_6226_OG3882
Cp_Diaphoretickes-Ptri_2899_OG3882
Other_Diaphoretickes-Omar_9463_OG3882
Cp_Diaphoretickes-Smin_38332_OG3882
Other_Diaphoretickes-Omar_24552_OG3882
Cp_Diaphoretickes-Dspp_40601_OG3882
Other_Diaphoretickes-Omar_38397_OG3882
Other_Diaphoretickes-Omar_37832_OG3882
0.5

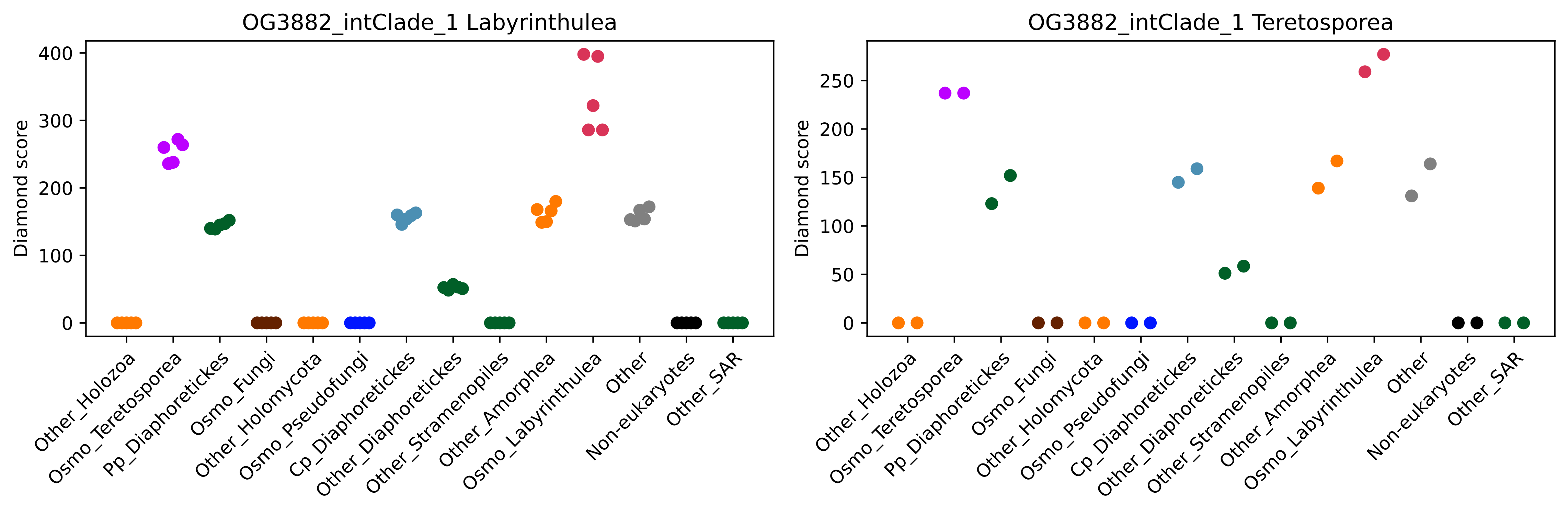
OG3882_intClade_1 Labyrinthulea
Diamond score
400
300
200
100
0
Other_Holozoa
Osmo_Teretosporea
Pp_Diaphoretickes
Osmo_Fungi
Other_Holomycota
Osmo_Pseudofungi
Cp_Diaphoretickes
Other_Diaphoretickes
Other_Stramenopiles
Other_Amorphea
Osmo_Labyrinthulea
Other
Non-eukaryotes
Other_SAR
OG3882_intClade_1 Teretosporea
Diamond score
250
200
150
100
50
0
Other_Holozoa
Osmo_Teretosporea
Pp_Diaphoretickes
Osmo_Fungi
Other_Holomycota
Osmo_Pseudofungi
Cp_Diaphoretickes
Other_Diaphoretickes
Other_Stramenopiles
Other_Amorphea
Osmo_Labyrinthulea
Other
Non-eukaryotes
Other_SAR

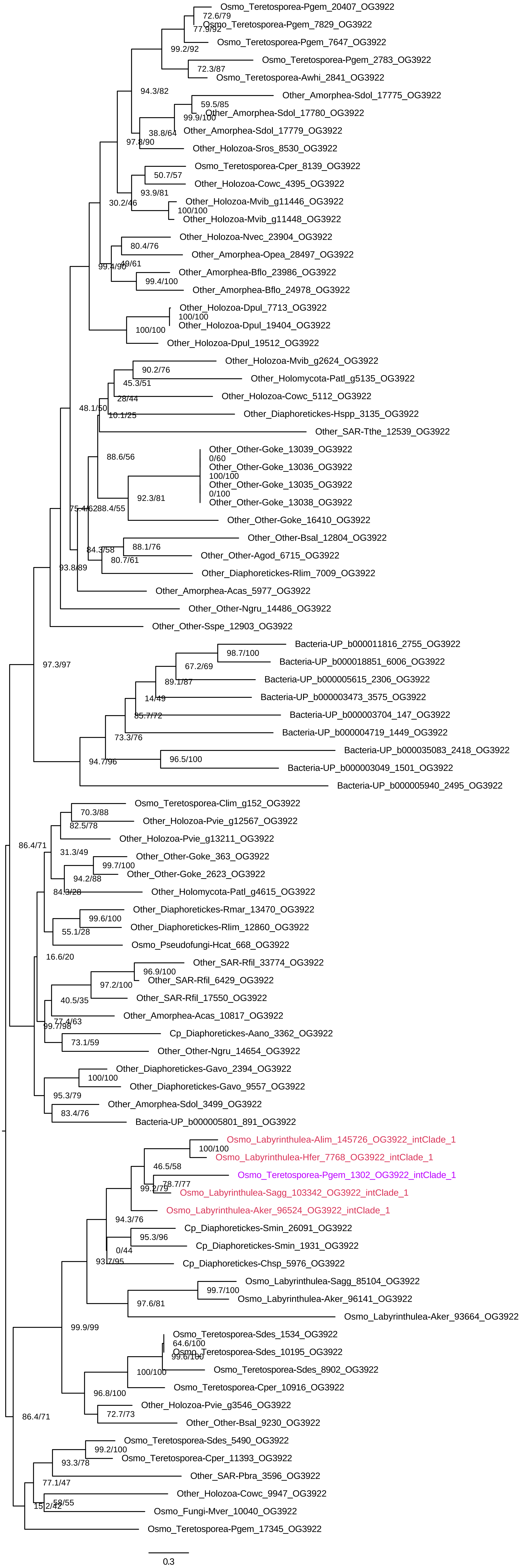
Osmo_Teretosporea-Pgem_20407_OG3922
Osmo_Teretosporea-Pgem_7829_OG3922
Osmo_Teretosporea-Pgem_7647_OG3922
Osmo_Teretosporea-Pgem_2783_OG3922
Osmo_Teretosporea-Awhi_2841_OG3922
Other_Amorphea-Sdol_17775_OG3922
Other_Amorphea-Sdol_17780_OG3922
Other_Amorphea-Sdol_17779_OG3922
Other_Holozoa-Sros_8530_OG3922
Osmo_Teretosporea-Cper_8139_OG3922
Other_Holozoa-Cowc_4395_OG3922
Other_Holozoa-Mvib_g11446_OG3922
Other_Holozoa-Mvib_g11448_OG3922
Other_Holozoa-Nvec_23904_OG3922
Other_Amorphea-Opea_28497_OG3922
Other_Amorphea-Bflo_23986_OG3922
Other_Amorphea-Bflo_24978_OG3922
Other_Holozoa-Dpul_7713_OG3922
Other_Holozoa-Dpul_19404_OG3922
Other_Holozoa-Dpul_19512_OG3922
Other_Holozoa-Mvib_g2624_OG3922
Other_Holomycota-Patl_g5135_OG3922
Other_Holozoa-Cowc_5112_OG3922
Other_Diaphoretickes-Hspp_3135_OG3922
Other_SAR-Tthe_12539_OG3922
Other_Other-Goke_13039_OG3922
Other_Other-Goke_13036_OG3922
Other_Other-Goke_13035_OG3922
Other_Other-Goke_13038_OG3922
Other_Other-Goke_16410_OG3922
Other_Other-Bsal_12804_OG3922
Other_Other-Agod_6715_OG3922
Other_Diaphoretickes-Rlim_7009_OG3922
Other_Amorphea-Acas_5977_OG3922
Other_Other-Ngru_14486_OG3922
Other_Other-Sspe_12903_OG3922
Bacteria-UP_b000011816_2755_OG3922
Bacteria-UP_b000018851_6006_OG3922
Bacteria-UP_b000005615_2306_OG3922
Bacteria-UP_b000003473_3575_OG3922
Bacteria-UP_b000003704_147_OG3922
Bacteria-UP_b000004719_1449_OG3922
Bacteria-UP_b000035083_2418_OG3922
Bacteria-UP_b000003049_1501_OG3922
Bacteria-UP_b000005940_2495_OG3922
Osmo_Teretosporea-Clim_g152_OG3922
Other_Holozoa-Pvie_g12567_OG3922
Other_Holozoa-Pvie_g13211_OG3922
Other_Other-Goke_363_OG3922
Other_Other-Goke_2623_OG3922
Other_Holomycota-Patl_g4615_OG3922
Other_Diaphoretickes-Rmar_13470_OG3922
Other_Diaphoretickes-Rlim_12860_OG3922
Osmo_Pseudofungi-Hcat_668_OG3922
Other_SAR-Rfil_33774_OG3922
Other_SAR-Rfil_6429_OG3922
Other_SAR-Rfil_17550_OG3922
Other_Amorphea-Acas_10817_OG3922
Cp_Diaphoretickes-Aano_3362_OG3922
Other_Other-Ngru_14654_OG3922
Other_Diaphoretickes-Gavo_2394_OG3922
Other_Diaphoretickes-Gavo_9557_OG3922
Other_Amorphea-Sdol_3499_OG3922
Bacteria-UP_b000005801_891_OG3922
Osmo_Labyrinthulea-Alim_145726_OG3922_intClade_1
Osmo_Labyrinthulea-Hfer_7768_OG3922_intClade_1
Osmo_Teretosporea-Pgem_1302_OG3922_intClade_1
Osmo_Labyrinthulea-Sagg_103342_OG3922_intClade_1
Osmo_Labyrinthulea-Aker_96524_OG3922_intClade_1
Cp_Diaphoretickes-Smin_26091_OG3922
Cp_Diaphoretickes-Smin_1931_OG3922
Cp_Diaphoretickes-Chsp_5976_OG3922
Osmo_Labyrinthulea-Sagg_85104_OG3922
Osmo_Labyrinthulea-Aker_96141_OG3922
Osmo_Labyrinthulea-Aker_93664_OG3922
Osmo_Teretosporea-Sdes_1534_OG3922
Osmo_Teretosporea-Sdes_10195_OG3922
Osmo_Teretosporea-Sdes_8902_OG3922
Osmo_Teretosporea-Cper_10916_OG3922
Other_Holozoa-Pvie_g3546_OG3922
Other_Other-Bsal_9230_OG3922
Osmo_Teretosporea-Sdes_5490_OG3922
Osmo_Teretosporea-Cper_11393_OG3922
Other_SAR-Pbra_3596_OG3922
Other_Holozoa-Cowc_9947_OG3922
Osmo_Fungi-Mver_10040_OG3922
Osmo_Teretosporea-Pgem_17345_OG3922
0.3

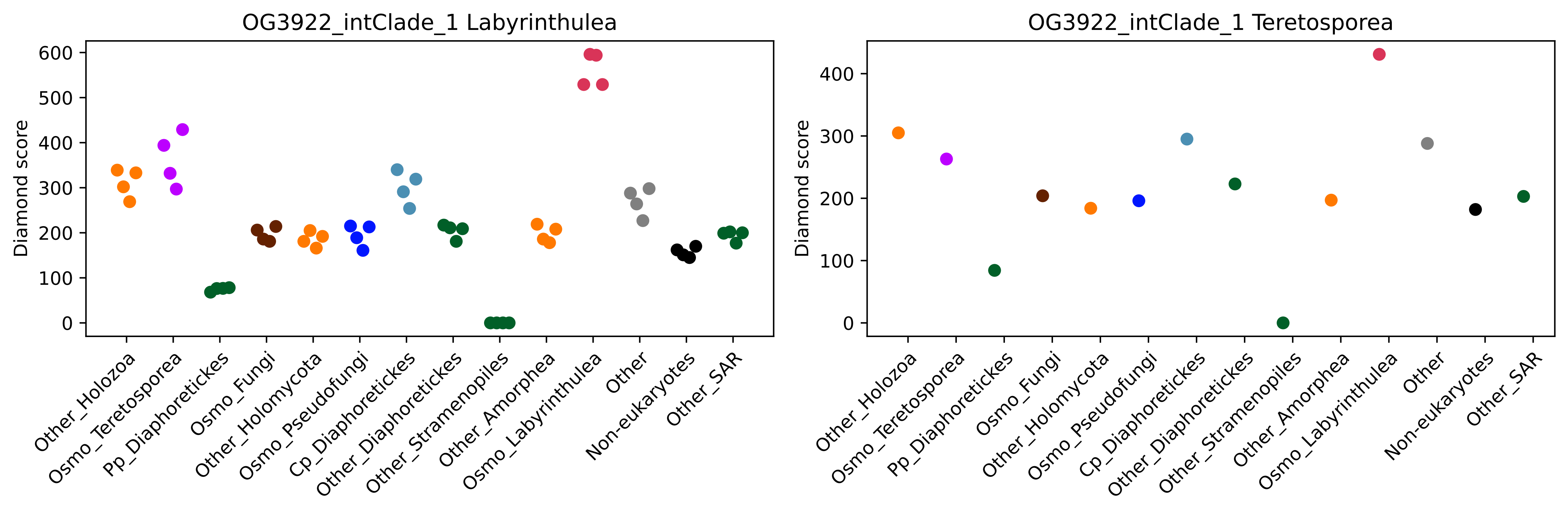
OG3922_intClade_1 Labyrinthulea
OG3922_intClade_1 Teretosporea
Diamond score
Other_Holozoa
Osmo_Teretosporea
Pp_Diaphoretickes
Osmo_Fungi
Other_Holomycota
Osmo_Pseudofungi
Cp_Diaphoretickes
Other_Diaphoretickes
Other_Stramenopiles
Other_Amorphea
Osmo_Labyrinthulea
Other
Non-eukaryotes
Other_SAR

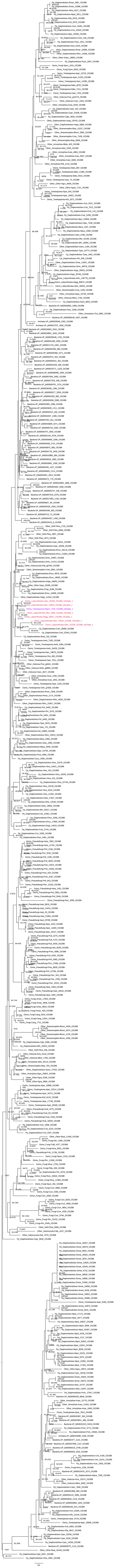

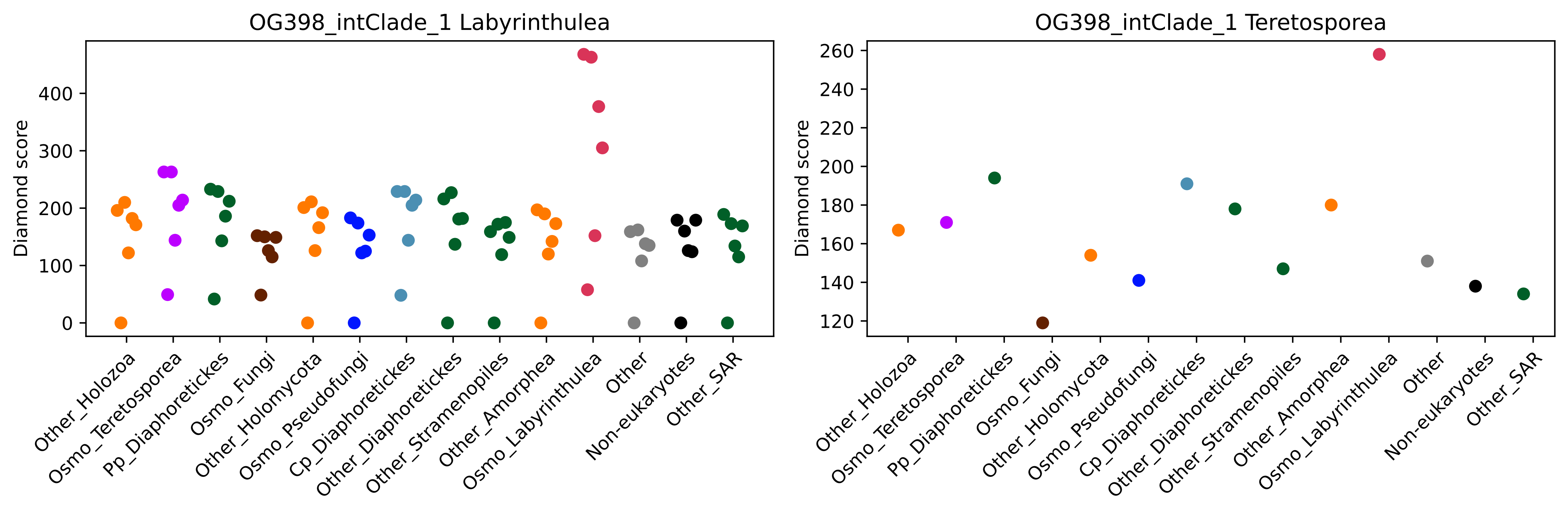

OG398_intClade_1 Labyrinthulea
Diamond score
0
100
200
300
400
Other_Holozoa
Osmo_Teretosporea
Pp_Diaphoretickes
Osmo_Fungi
Other_Holomycota
Osmo_Pseudofungi
Cp_Diaphoretickes
Other_Diaphoretickes
Other_Stramenopiles
Other_Amorphea
Osmo_Labyrinthulea
Other
Non-eukaryotes
Other_SAR
OG398_intClade_1 Teretosporea
Diamond score
120
140
160
180
200
220
240
260
Other_Holozoa
Osmo_Teretosporea
Pp_Diaphoretickes
Osmo_Fungi
Other_Holomycota
Osmo_Pseudofungi
Cp_Diaphoretickes
Other_Diaphoretickes
Other_Stramenopiles
Other_Amorphea
Osmo_Labyrinthulea
Other
Non-eukaryotes
Other_SAR

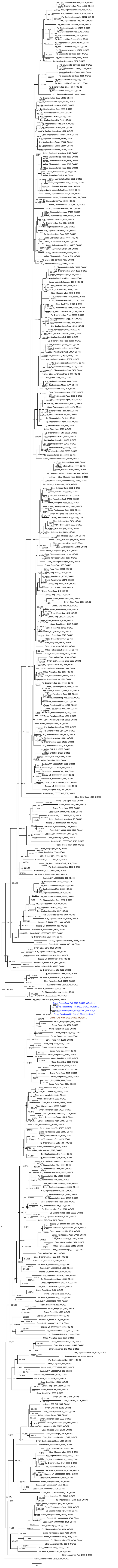

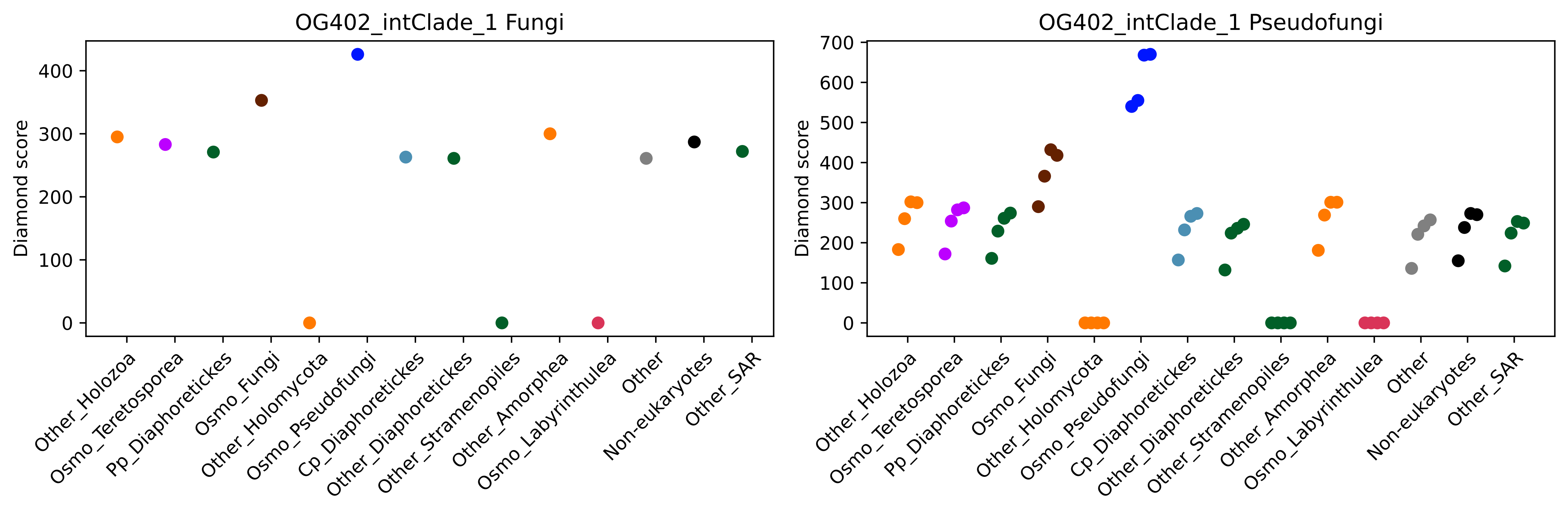

OG402_intClade_1 Fungi
OG402_intClade_1 Pseudofungi
Diamond score
0
100
200
300
400
500
600
700
Other_Holozoa
Osmo_Teretosporea
Pp_Diaphoretickes
Osmo_Fungi
Other_Holomycota
Osmo_Pseudofungi
Cp_Diaphoretickes
Other_Diaphoretickes
Other_Stramenopiles
Other_Amorphea
Osmo_Labyrinthulea
Other
Non-eukaryotes
Other_SAR

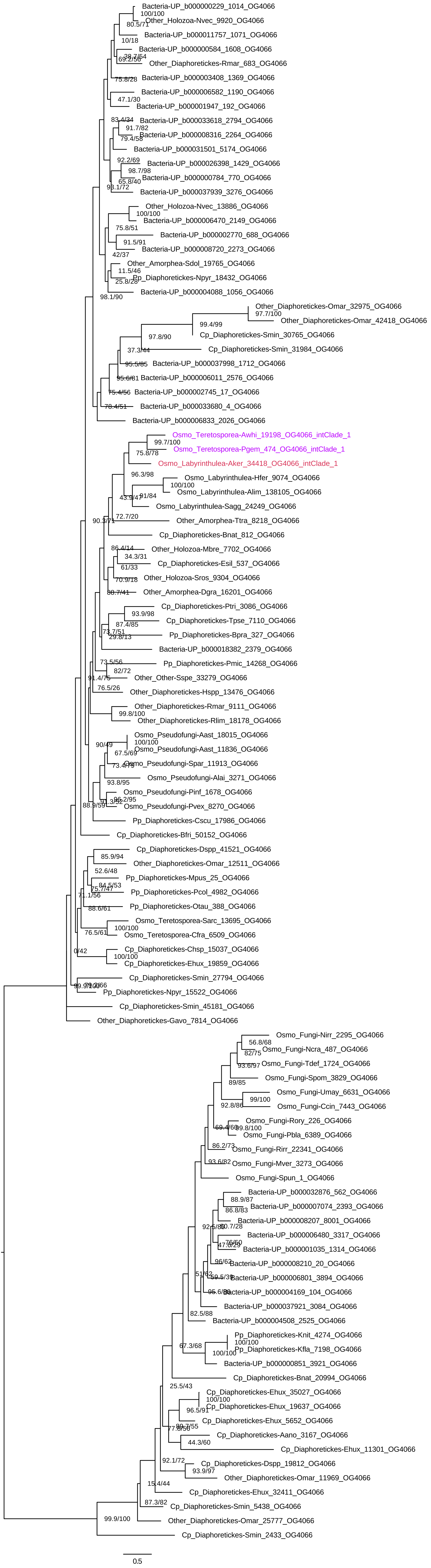

Bacteria-UP_b000000229_1014_OG4066
100/100
Other_Holozoa-Nvec_9920_OG4066
80.5/71
Bacteria-UP_b000011757_1071_OG4066
10/18
Bacteria-UP_b000000584_1608_OG4066
38.7/54
69.2/56
Other_Diaphoretickes-Rmar_683_OG4066
75.8/28
Bacteria-UP_b000003408_1369_OG4066
Bacteria-UP_b000006582_1190_OG4066
47.1/30
Bacteria-UP_b000001947_192_OG4066
83.4/34
Bacteria-UP_b000033618_2794_OG4066
91.7/82
Bacteria-UP_b000008316_2264_OG4066
79.4/56
Bacteria-UP_b000031501_5174_OG4066
92.2/69
Bacteria-UP_b000026398_1429_OG4066
98.7/98
Bacteria-UP_b000000784_770_OG4066
65.8/40
93.1/72
Bacteria-UP_b000037939_3276_OG4066
Other_Holozoa-Nvec_13886_OG4066
100/100
Bacteria-UP_b000006470_2149_OG4066
75.8/51
Bacteria-UP_b000002770_688_OG4066
91.5/91
Bacteria-UP_b000008720_2273_OG4066
42/37
Other_Amorphea-Sdol_19765_OG4066
11.5/46
Pp_Diaphoretickes-Npyr_18432_OG4066
25.8/28
Bacteria-UP_b000004088_1056_OG4066
98.1/90
Other_Diaphoretickes-Omar_32975_OG4066
97.7/100
Other_Diaphoretickes-Omar_42418_OG4066
99.4/99
Cp_Diaphoretickes-Smin_30765_OG4066
97.8/90
37.3/44
Cp_Diaphoretickes-Smin_31984_OG4066
95.5/85
Bacteria-UP_b000037998_1712_OG4066
95.6/81
Bacteria-UP_b000006011_2576_OG4066
75.4/56
Bacteria-UP_b000002745_17_OG4066
78.4/51
Bacteria-UP_b000033680_4_OG4066
Bacteria-UP_b000006833_2026_OG4066
Osmo_Teretosporea-Awhi_19198_OG4066_intClade_1
99.7/100
Osmo_Teretosporea-Pgem_474_OG4066_intClade_1
75.8/78
Osmo_Labyrinthulea-Aker_34418_OG4066_intClade_1
96.3/98
Osmo_Labyrinthulea-Hfer_9074_OG4066
100/100
Osmo_Labyrinthulea-Alim_138105_OG4066
43.9/47
91/84
Osmo_Labyrinthulea-Sagg_24249_OG4066
72.7/20
90.3/71
Other_Amorphea-Ttra_8218_OG4066
Cp_Diaphoretickes-Bnat_812_OG4066
86.4/14
Other_Holozoa-Mbre_7702_OG4066
34.3/31
Cp_Diaphoretickes-Esil_537_OG4066
61/33
70.9/18
Other_Holozoa-Sros_9304_OG4066
88.7/41
Other_Amorphea-Dgra_16201_OG4066
Cp_Diaphoretickes-Ptri_3086_OG4066
93.9/98
87.4/85
Cp_Diaphoretickes-Tpse_7110_OG4066
73.7/51
29.8/13
Pp_Diaphoretickes-Bpra_327_OG4066
Bacteria-UP_b000018382_2379_OG4066
73.5/56
Pp_Diaphoretickes-Pmic_14268_OG4066
82/72
91.4/75
Other_Other-Sspe_33279_OG4066
76.5/26
Other_Diaphoretickes-Hspp_13476_OG4066
Other_Diaphoretickes-Rmar_9111_OG4066
99.8/100
Other_Diaphoretickes-Rlim_18178_OG4066
Osmo_Pseudofungi-Aast_18015_OG4066
90/49
100/100
Osmo_Pseudofungi-Aast_11836_OG4066
67.5/69
73.4/73
Osmo_Pseudofungi-Spar_11913_OG4066
93.8/95
Osmo_Pseudofungi-Alai_3271_OG4066
Osmo_Pseudofungi-Pinf_1678_OG4066
96.2/95
91.3/92
88.9/59
Osmo_Pseudofungi-Pvex_8270_OG4066
Pp_Diaphoretickes-Cscu_17986_OG4066
Cp_Diaphoretickes-Bfri_50152_OG4066
Cp_Diaphoretickes-Dspp_41521_OG4066
85.9/94
Other_Diaphoretickes-Omar_12511_OG4066
52.6/48
Pp_Diaphoretickes-Mpus_25_OG4066
84.5/53
75.7/47
71.1/56
Pp_Diaphoretickes-Pcol_4982_OG4066
88.6/61
Pp_Diaphoretickes-Otau_388_OG4066
Osmo_Teretosporea-Sarc_13695_OG4066
100/100
76.5/61
Osmo_Teretosporea-Cfra_6509_OG4066
0/42
Cp_Diaphoretickes-Chsp_15037_OG4066
100/100
Cp_Diaphoretickes-Ehux_19859_OG4066
Cp_Diaphoretickes-Smin_27794_OG4066
99.9/100
Pp_Diaphoretickes-Npyr_15522_OG4066
Cp_Diaphoretickes-Smin_45181_OG4066
Other_Diaphoretickes-Gavo_7814_OG4066
Osmo_Fungi-Nirr_2295_OG4066
56.8/68
82/75
Osmo_Fungi-Ncra_487_OG4066
93.6/97
Osmo_Fungi-Tdef_1724_OG4066
Osmo_Fungi-Spom_3829_OG4066
89/85
Osmo_Fungi-Umay_6631_OG4066
99/100
92.8/86
Osmo_Fungi-Ccin_7443_OG4066
Osmo_Fungi-Rory_226_OG4066
69.4/60
99.8/100
Osmo_Fungi-Pbla_6389_OG4066
86.2/73
Osmo_Fungi-Rirr_22341_OG4066
93.6/82
Osmo_Fungi-Mver_3273_OG4066
Osmo_Fungi-Spun_1_OG4066
Bacteria-UP_b000032876_562_OG4066
88.9/87
86.8/83
Bacteria-UP_b000007074_2393_OG4066
92.6/85
60.7/28
Bacteria-UP_b000008207_8001_OG4066
Bacteria-UP_b000006480_3317_OG4066
76/60
47.6/29
Bacteria-UP_b000001035_1314_OG4066
96/62
Bacteria-UP_b000008210_20_OG4066
51/62
59.5/39
Bacteria-UP_b000006801_3894_OG4066
95.6/86
Bacteria-UP_b000004169_104_OG4066
82.5/88
Bacteria-UP_b000037921_3084_OG4066
Bacteria-UP_b000004508_2525_OG4066
Pp_Diaphoretickes-Knit_4274_OG4066
100/100
67.3/68
Pp_Diaphoretickes-Kfla_7198_OG4066
100/100
Bacteria-UP_b000000851_3921_OG4066
Cp_Diaphoretickes-Bnat_20994_OG4066
25.5/43
Cp_Diaphoretickes-Ehux_35027_OG4066
100/100
96.5/91
Cp_Diaphoretickes-Ehux_19637_OG4066
89.7/55
77.8/56
Cp_Diaphoretickes-Ehux_5652_OG4066
Cp_Diaphoretickes-Aano_3167_OG4066
44.3/60
Cp_Diaphoretickes-Ehux_11301_OG4066
92.1/72
Cp_Diaphoretickes-Dspp_19812_OG4066
93.9/97
Other_Diaphoretickes-Omar_11969_OG4066
15.4/44
Cp_Diaphoretickes-Ehux_32411_OG4066
87.3/82
Cp_Diaphoretickes-Smin_5438_OG4066
Other_Diaphoretickes-Omar_25777_OG4066
99.9/100
Cp_Diaphoretickes-Smin_2433_OG4066
0.5

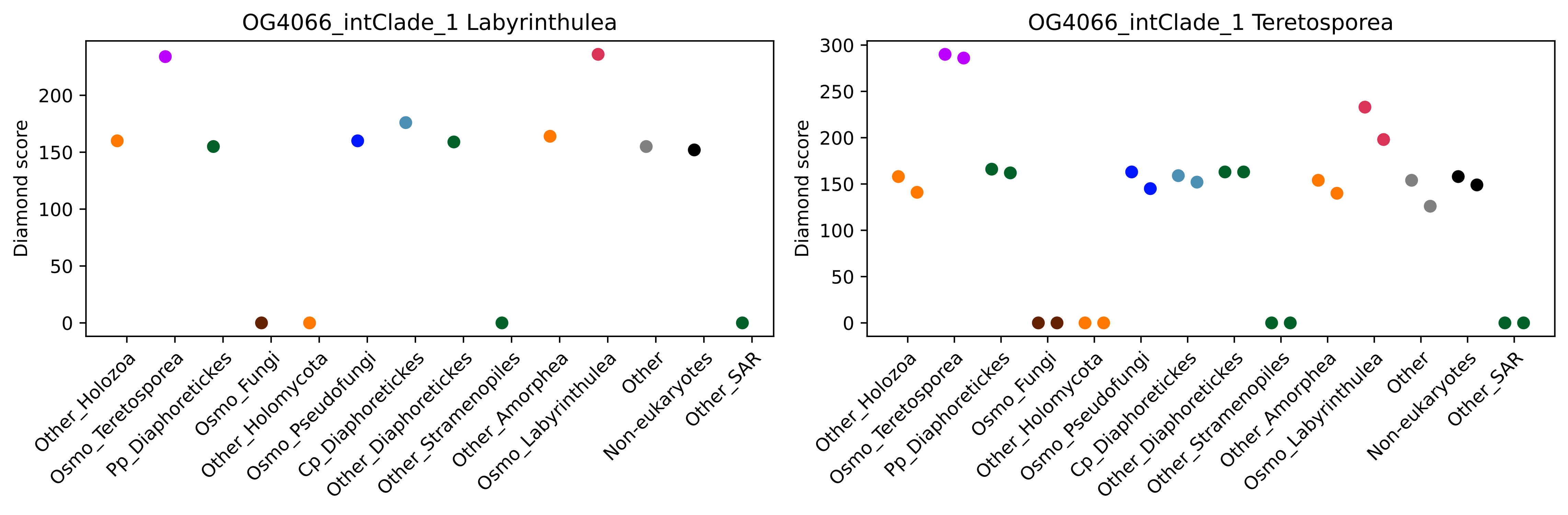

OG4066_intClade_1 Labyrinthulea
OG4066_intClade_1 Teretosporea
Diamond score
0
50
100
150
200
250
300
Other_Holozoa
Osmo_Teretosporea
Pp_Diaphoretickes
Osmo_Fungi
Other_Holomycota
Osmo_Pseudofungi
Cp_Diaphoretickes
Other_Diaphoretickes
Other_Stramenopiles
Other_Amorphea
Osmo_Labyrinthulea
Other
Non-eukaryotes
Other_SAR

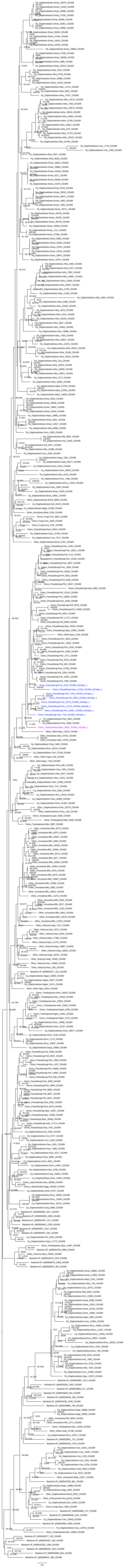

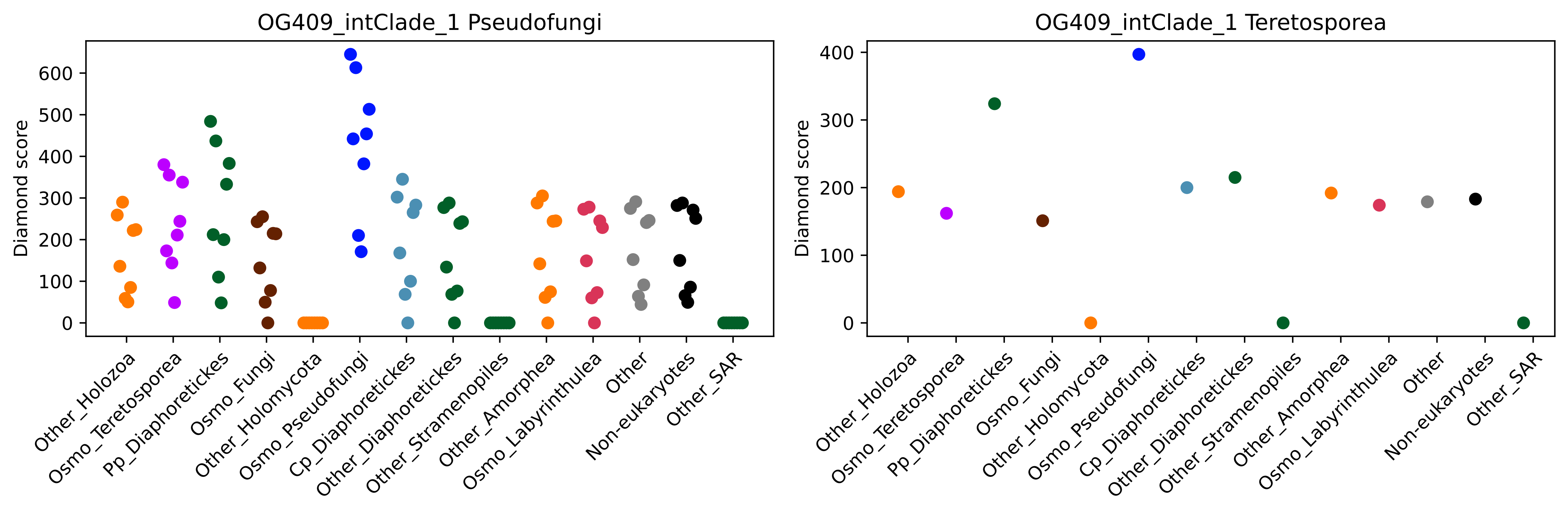
OG409_intClade_1 Pseudofungi
OG409_intClade_1 Teretosporea
Diamond score
0
100
200
300
400
500
600
Other_Holozoa
Osmo_Teretosporea
Pp_Diaphoretickes
Osmo_Fungi
Other_Holomycota
Osmo_Pseudofungi
Cp_Diaphoretickes
Other_Diaphoretickes
Other_Stramenopiles
Other_Amorphea
Osmo_Labyrinthulea
Other
Non-eukaryotes
Other_SAR

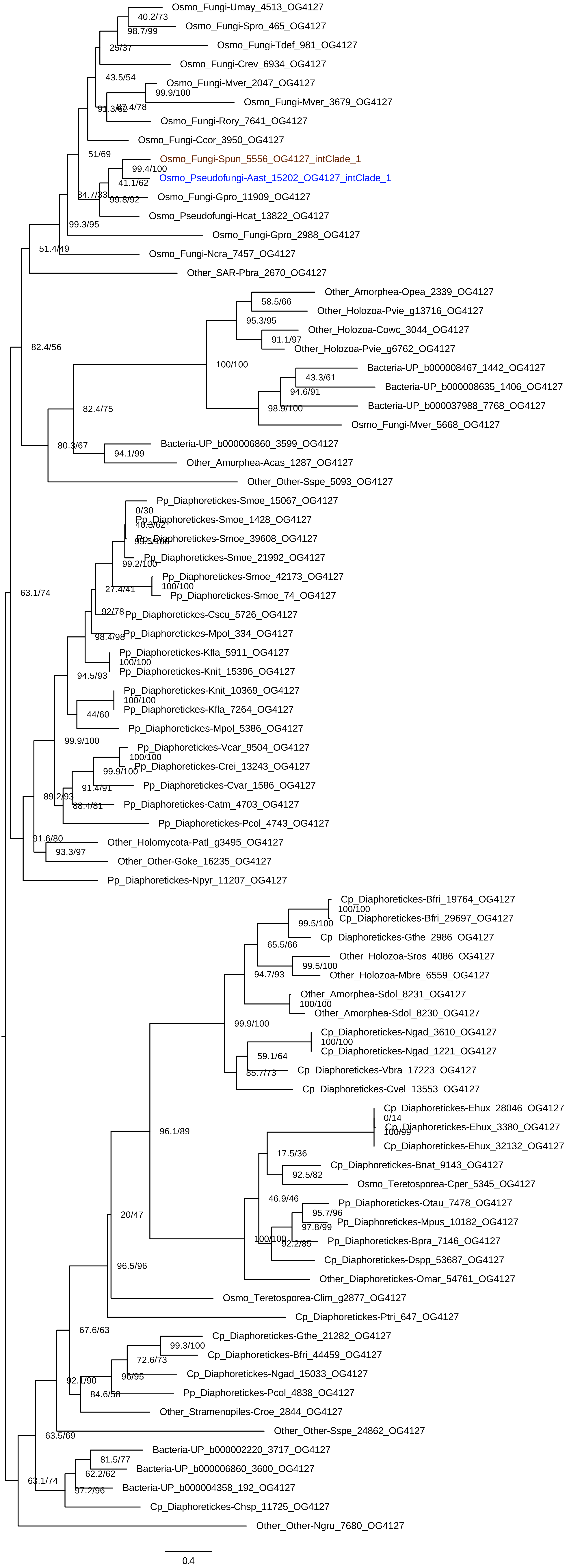
Osmo_Fungi-Umay_4513_OG4127
40.2/73
Osmo_Fungi-Spro_465_OG4127
98.7/99
Osmo_Fungi-Tdef_981_OG4127
25/37
Osmo_Fungi-Crev_6934_OG4127
43.5/54
Osmo_Fungi-Mver_2047_OG4127
99.9/100
Osmo_Fungi-Mver_3679_OG4127
91.3/62
87.4/78
Osmo_Fungi-Rory_7641_OG4127
Osmo_Fungi-Ccor_3950_OG4127
51/69
Osmo_Fungi-Spun_5556_OG4127_intClade_1
99.4/100
Osmo_Pseudofungi-Aast_15202_OG4127_intClade_1
41.1/62
84.7/33
Osmo_Fungi-Gpro_11909_OG4127
99.8/92
Osmo_Pseudofungi-Hcat_13822_OG4127
99.3/95
Osmo_Fungi-Gpro_2988_OG4127
51.4/49
Osmo_Fungi-Ncra_7457_OG4127
Other_SAR-Pbra_2670_OG4127
Other_Amorphea-Opea_2339_OG4127
58.5/66
Other_Holozoa-Pvie_g13716_OG4127
95.3/95
Other_Holozoa-Cowc_3044_OG4127
91.1/97
Other_Holozoa-Pvie_g6762_OG4127
82.4/56
100/100
Bacteria-UP_b000008467_1442_OG4127
43.3/61
Bacteria-UP_b000008635_1406_OG4127
94.6/91
Bacteria-UP_b000037988_7768_OG4127
82.4/75
98.9/100
Osmo_Fungi-Mver_5668_OG4127
Bacteria-UP_b000006860_3599_OG4127
80.3/67
94.1/99
Other_Amorphea-Acas_1287_OG4127
Other_Other-Sspe_5093_OG4127
Pp_Diaphoretickes-Smoe_15067_OG4127
0/30
Pp_Diaphoretickes-Smoe_1428_OG4127
40.3/62
Pp_Diaphoretickes-Smoe_39608_OG4127
99.5/100
Pp_Diaphoretickes-Smoe_21992_OG4127
99.2/100
Pp_Diaphoretickes-Smoe_42173_OG4127
100/100
27.4/41
Pp_Diaphoretickes-Smoe_74_OG4127
63.1/74
92/78
Pp_Diaphoretickes-Cscu_5726_OG4127
98.4/98
Pp_Diaphoretickes-Mpol_334_OG4127
Pp_Diaphoretickes-Kfla_5911_OG4127
100/100
94.5/93
Pp_Diaphoretickes-Knit_15396_OG4127
Pp_Diaphoretickes-Knit_10369_OG4127
100/100
Pp_Diaphoretickes-Kfla_7264_OG4127
44/60
Pp_Diaphoretickes-Mpol_5386_OG4127
99.9/100
Pp_Diaphoretickes-Vcar_9504_OG4127
100/100
Pp_Diaphoretickes-Crei_13243_OG4127
99.9/100
91.4/91
Pp_Diaphoretickes-Cvar_1586_OG4127
89.2/93
88.4/81
Pp_Diaphoretickes-Catm_4703_OG4127
Pp_Diaphoretickes-Pcol_4743_OG4127
91.6/80
Other_Holomycota-Patl_g3495_OG4127
93.3/97
Other_Other-Goke_16235_OG4127
Pp_Diaphoretickes-Npyr_11207_OG4127
Cp_Diaphoretickes-Bfri_19764_OG4127
100/100
Cp_Diaphoretickes-Bfri_29697_OG4127
99.5/100
Cp_Diaphoretickes-Gthe_2986_OG4127
65.5/66
Other_Holozoa-Sros_4086_OG4127
99.5/100
94.7/93
Other_Holozoa-Mbre_6559_OG4127
Other_Amorphea-Sdol_8231_OG4127
100/100
Other_Amorphea-Sdol_8230_OG4127
99.9/100
Cp_Diaphoretickes-Ngad_3610_OG4127
100/100
59.1/64
Cp_Diaphoretickes-Ngad_1221_OG4127
85.7/73
Cp_Diaphoretickes-Vbra_17223_OG4127
Cp_Diaphoretickes-Cvel_13553_OG4127
Cp_Diaphoretickes-Ehux_28046_OG4127
0/14
Cp_Diaphoretickes-Ehux_3380_OG4127
96.1/89
100/99
Cp_Diaphoretickes-Ehux_32132_OG4127
17.5/36
Cp_Diaphoretickes-Bnat_9143_OG4127
92.5/82
Osmo_Teretosporea-Cper_5345_OG4127
20/47
46.9/46
Pp_Diaphoretickes-Otau_7478_OG4127
95.7/96
Pp_Diaphoretickes-Mpus_10182_OG4127
97.8/99
100/100
92.2/85
Pp_Diaphoretickes-Bpra_7146_OG4127
Cp_Diaphoretickes-Dspp_53687_OG4127
96.5/96
Other_Diaphoretickes-Omar_54761_OG4127
Osmo_Teretosporea-Clim_g2877_OG4127
Cp_Diaphoretickes-Ptri_647_OG4127
67.6/63
Cp_Diaphoretickes-Gthe_21282_OG4127
99.3/100
Cp_Diaphoretickes-Bfri_44459_OG4127
72.6/73
92.1/90
96/95
Cp_Diaphoretickes-Ngad_15033_OG4127
84.6/58
Pp_Diaphoretickes-Pcol_4838_OG4127
Other_Stramenopiles-Croe_2844_OG4127
63.5/69
Other_Other-Sspe_24862_OG4127
Bacteria-UP_b000002220_3717_OG4127
81.5/77
Bacteria-UP_b000006860_3600_OG4127
63.1/74
62.2/62
97.2/96
Bacteria-UP_b000004358_192_OG4127
Cp_Diaphoretickes-Chsp_11725_OG4127
Other_Other-Ngru_7680_OG4127
0.4

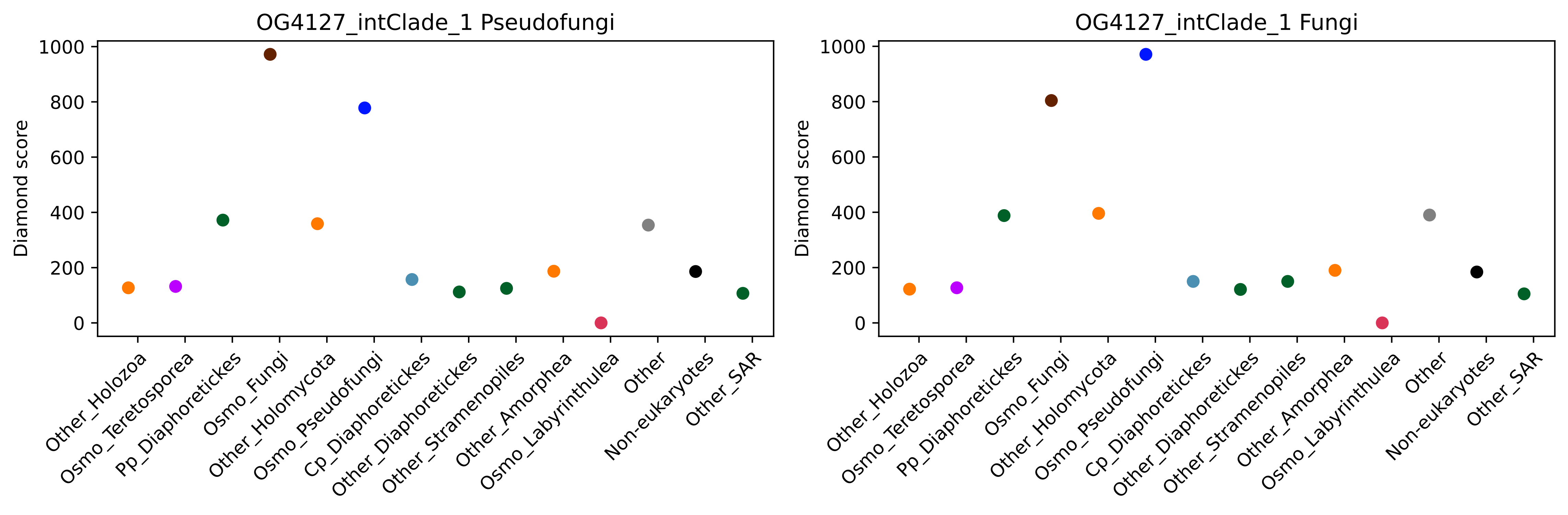

OG4127_intClade_1 Pseudofungi
OG4127_intClade_1 Fungi
Diamond score
0
200
400
600
800
1000
Other_Holozoa
Osmo_Teretosporea
Pp_Diaphoretickes
Osmo_Fungi
Other_Holomycota
Osmo_Pseudofungi
Cp_Diaphoretickes
Other_Diaphoretickes
Other_Stramenopiles
Other_Amorphea
Osmo_Labyrinthulea
Other
Non-eukaryotes
Other_SAR

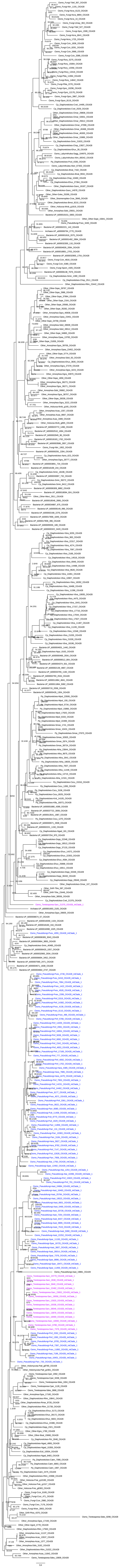

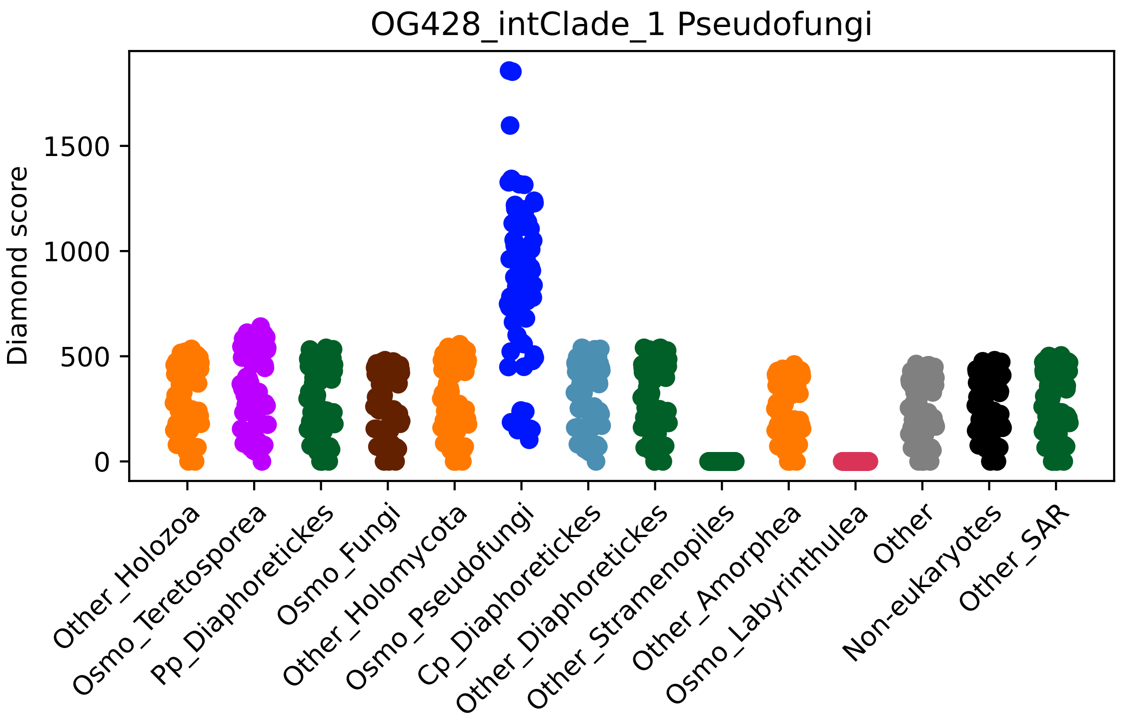
OG428_intClade_1 Pseudofungi
Diamond score
0
500
1000
1500
Other_Holozoa
Osmo_Teretosporea
Pp_Diaphoretickes
Osmo_Fungi
Other_Holomycota
Osmo_Pseudofungi
Cp_Diaphoretickes
Other_Diaphoretickes
Other_Stramenopiles
Other_Amorphea
Osmo_Labyrinthulea
Other
Non-eukaryotes
Other_SAR

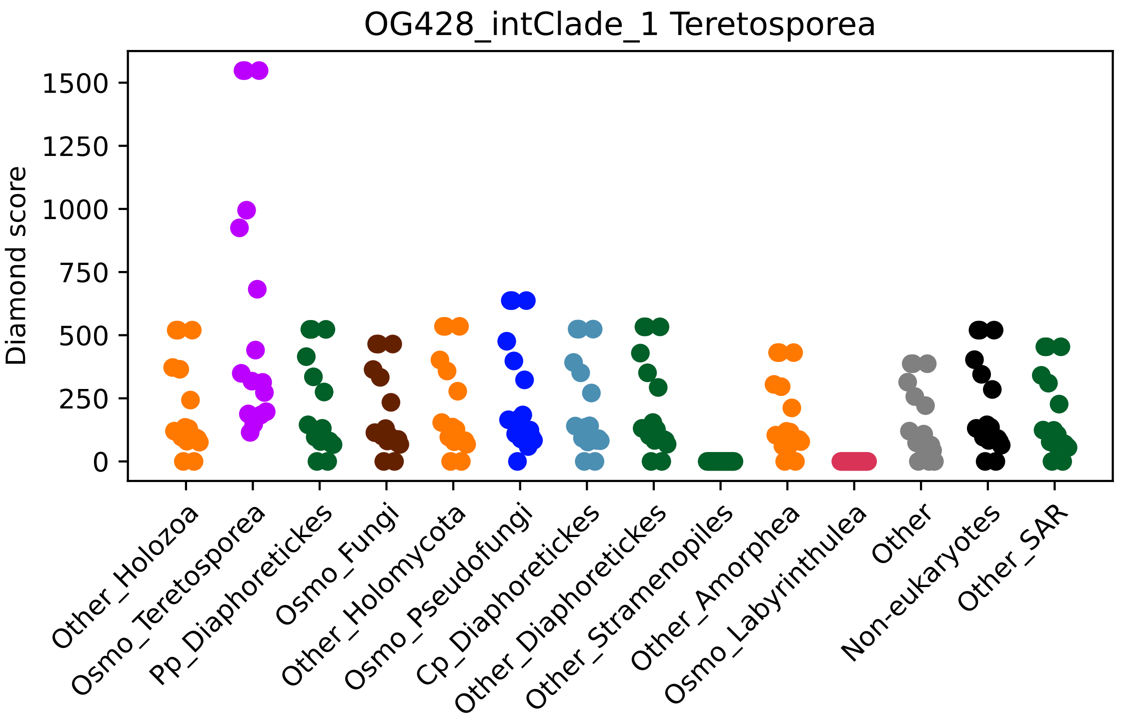
OG428_intClade_1 Teretosporea
Diamond score
0
250
500
750
1000
1250
1500
Other_Holozoa
Osmo_Teretosporea
Pp_Diaphoretickes
Osmo_Fungi
Other_Holomycota
Osmo_Pseudofungi
Cp_Diaphoretickes
Other_Diaphoretickes
Other_Stramenopiles
Other_Amorphea
Osmo_Labyrinthulea
Other
Non-eukaryotes
Other_SAR

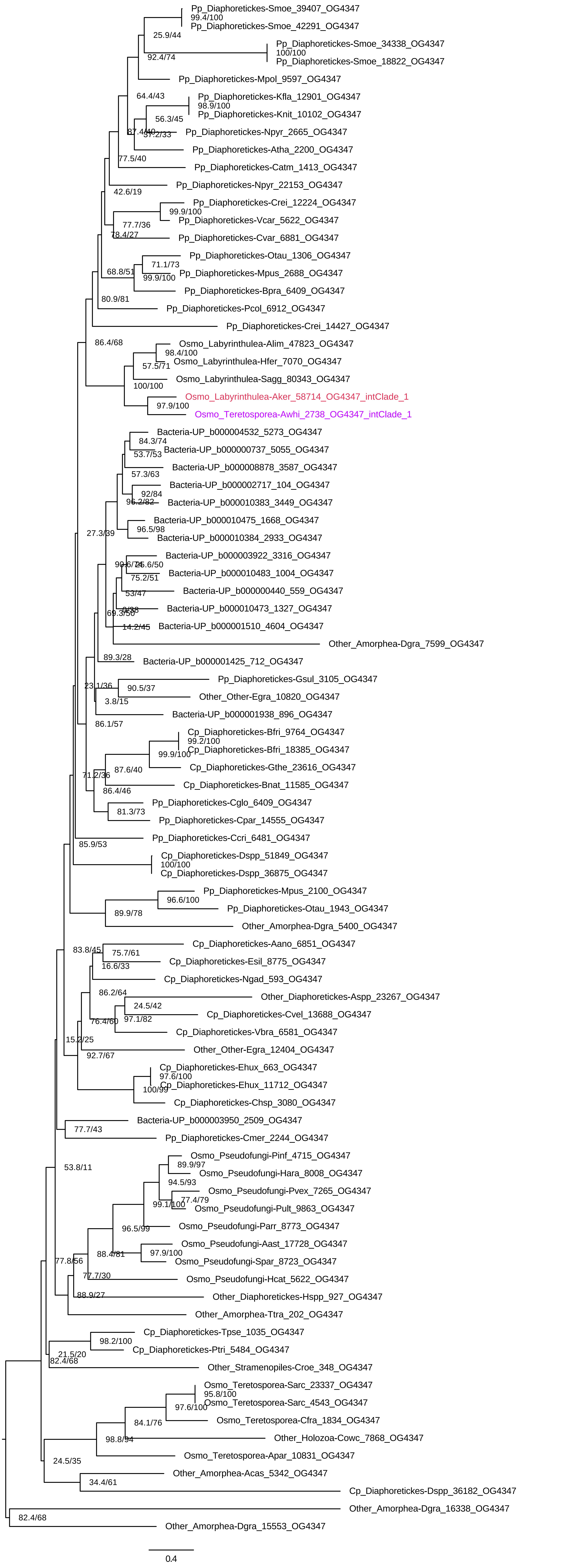

Pp_Diaphoretickes-Smoe_39407_OG4347
99.4/100
Pp_Diaphoretickes-Smoe_42291_OG4347
25.9/44
Pp_Diaphoretickes-Smoe_34338_OG4347
100/100
Pp_Diaphoretickes-Smoe_18822_OG4347
92.4/74
Pp_Diaphoretickes-Mpol_9597_OG4347
64.4/43
Pp_Diaphoretickes-Kfla_12901_OG4347
98.9/100
Pp_Diaphoretickes-Knit_10102_OG4347
56.3/45
87.4/40
37.2/33
Pp_Diaphoretickes-Npyr_2665_OG4347
Pp_Diaphoretickes-Atha_2200_OG4347
77.5/40
Pp_Diaphoretickes-Catm_1413_OG4347
Pp_Diaphoretickes-Npyr_22153_OG4347
42.6/19
Pp_Diaphoretickes-Crei_12224_OG4347
99.9/100
77.7/36
Pp_Diaphoretickes-Vcar_5622_OG4347
78.4/27
Pp_Diaphoretickes-Cvar_6881_OG4347
Pp_Diaphoretickes-Otau_1306_OG4347
71.1/73
68.8/51
Pp_Diaphoretickes-Mpus_2688_OG4347
99.9/100
Pp_Diaphoretickes-Bpra_6409_OG4347
80.9/81
Pp_Diaphoretickes-Pcol_6912_OG4347
Pp_Diaphoretickes-Crei_14427_OG4347
86.4/68
Osmo_Labyrinthulea-Alim_47823_OG4347
98.4/100
57.5/71
Osmo_Labyrinthulea-Hfer_7070_OG4347
Osmo_Labyrinthulea-Sagg_80343_OG4347
100/100
Osmo_Labyrinthulea-Aker_58714_OG4347_intClade_1
97.9/100
Osmo_Teretosporea-Awhi_2738_OG4347_intClade_1
Bacteria-UP_b000004532_5273_OG4347
84.3/74
53.7/53
Bacteria-UP_b000000737_5055_OG4347
57.3/63
Bacteria-UP_b000008878_3587_OG4347
Bacteria-UP_b000002717_104_OG4347
92/84
96.2/82
Bacteria-UP_b000010383_3449_OG4347
Bacteria-UP_b000010475_1668_OG4347
96.5/98
27.3/39
Bacteria-UP_b000010384_2933_OG4347
Bacteria-UP_b000003922_3316_OG4347
90.6/74
26.6/50
Bacteria-UP_b000010483_1004_OG4347
75.2/51
53/47
Bacteria-UP_b000000440_559_OG4347
0/38
69.3/50
Bacteria-UP_b000010473_1327_OG4347
14.2/45
Bacteria-UP_b000001510_4604_OG4347
Other_Amorphea-Dgra_7599_OG4347
89.3/28
Bacteria-UP_b000001425_712_OG4347
Pp_Diaphoretickes-Gsul_3105_OG4347
23.1/36
90.5/37
Other_Other-Egra_10820_OG4347
3.8/15
Bacteria-UP_b000001938_896_OG4347
86.1/57
Cp_Diaphoretickes-Bfri_9764_OG4347
99.2/100
Cp_Diaphoretickes-Bfri_18385_OG4347
99.9/100
Cp_Diaphoretickes-Gthe_23616_OG4347
87.6/40
71.2/36
Cp_Diaphoretickes-Bnat_11585_OG4347
86.4/46
Pp_Diaphoretickes-Cglo_6409_OG4347
81.3/73
Pp_Diaphoretickes-Cpar_14555_OG4347
Pp_Diaphoretickes-Ccri_6481_OG4347
85.9/53
Cp_Diaphoretickes-Dspp_51849_OG4347
100/100
Cp_Diaphoretickes-Dspp_36875_OG4347
Pp_Diaphoretickes-Mpus_2100_OG4347
96.6/100
Pp_Diaphoretickes-Otau_1943_OG4347
89.9/78
Other_Amorphea-Dgra_5400_OG4347
83.8/45
Cp_Diaphoretickes-Aano_6851_OG4347
75.7/61
Cp_Diaphoretickes-Esil_8775_OG4347
16.6/33
Cp_Diaphoretickes-Ngad_593_OG4347
86.2/64
Other_Diaphoretickes-Aspp_23267_OG4347
24.5/42
Cp_Diaphoretickes-Cvel_13688_OG4347
76.4/60
97.1/82
Cp_Diaphoretickes-Vbra_6581_OG4347
15.2/25
92.7/67
Other_Other-Egra_12404_OG4347
Cp_Diaphoretickes-Ehux_663_OG4347
97.6/100
Cp_Diaphoretickes-Ehux_11712_OG4347
100/99
Cp_Diaphoretickes-Chsp_3080_OG4347
Bacteria-UP_b000003950_2509_OG4347
77.7/43
Pp_Diaphoretickes-Cmer_2244_OG4347
Osmo_Pseudofungi-Pinf_4715_OG4347
53.8/11
89.9/97
Osmo_Pseudofungi-Hara_8008_OG4347
94.5/93
Osmo_Pseudofungi-Pvex_7265_OG4347
77.4/79
99.1/100
Osmo_Pseudofungi-Pult_9863_OG4347
Osmo_Pseudofungi-Parr_8773_OG4347
96.5/99
Osmo_Pseudofungi-Aast_17728_OG4347
88.4/81
97.9/100
Osmo_Pseudofungi-Spar_8723_OG4347
77.8/56
77.7/30
Osmo_Pseudofungi-Hcat_5622_OG4347
88.9/27
Other_Diaphoretickes-Hspp_927_OG4347
Other_Amorphea-Ttra_202_OG4347
Cp_Diaphoretickes-Tpse_1035_OG4347
98.2/100
Cp_Diaphoretickes-Ptri_5484_OG4347
21.5/20
82.4/68
Other_Stramenopiles-Croe_348_OG4347
Osmo_Teretosporea-Sarc_23337_OG4347
95.8/100
Osmo_Teretosporea-Sarc_4543_OG4347
97.6/100
Osmo_Teretosporea-Cfra_1834_OG4347
84.1/76
Other_Holozoa-Cowc_7868_OG4347
98.8/94
Osmo_Teretosporea-Apar_10831_OG4347
24.5/35
Other_Amorphea-Acas_5342_OG4347
34.4/61
Cp_Diaphoretickes-Dspp_36182_OG4347
Other_Amorphea-Dgra_16338_OG4347
82.4/68
Other_Amorphea-Dgra_15553_OG4347
0.4

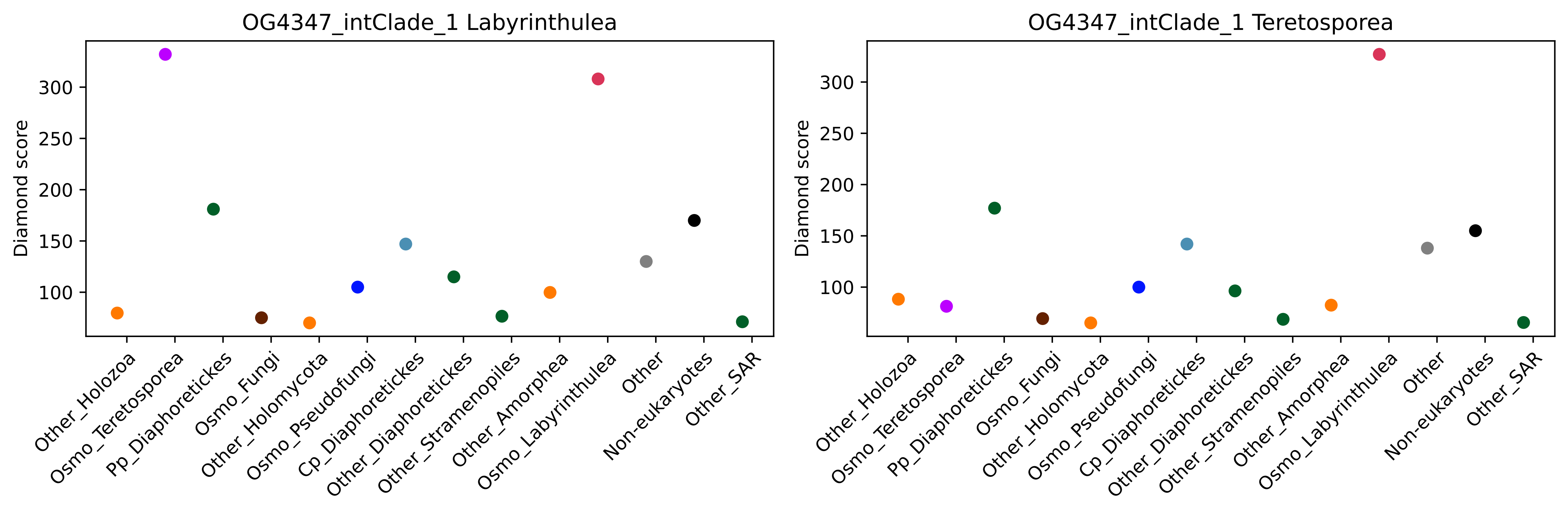

OG4347_intClade_1 Labyrinthulea
Diamond score
100
150
200
250
300
Other_Holozoa
Osmo_Teretosporea
Pp_Diaphoretickes
Osmo_Fungi
Other_Holomycota
Osmo_Pseudofungi
Cp_Diaphoretickes
Other_Diaphoretickes
Other_Stramenopiles
Other_Amorphea
Osmo_Labyrinthulea
Other
Non-eukaryotes
Other_SAR
OG4347_intClade_1 Teretosporea
Diamond score
100
150
200
250
300
Other_Holozoa
Osmo_Teretosporea
Pp_Diaphoretickes
Osmo_Fungi
Other_Holomycota
Osmo_Pseudofungi
Cp_Diaphoretickes
Other_Diaphoretickes
Other_Stramenopiles
Other_Amorphea
Osmo_Labyrinthulea
Other
Non-eukaryotes
Other_SAR

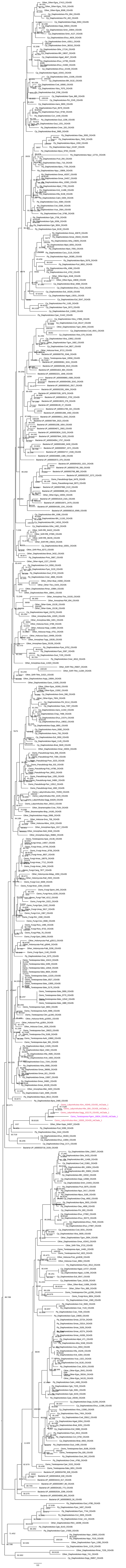

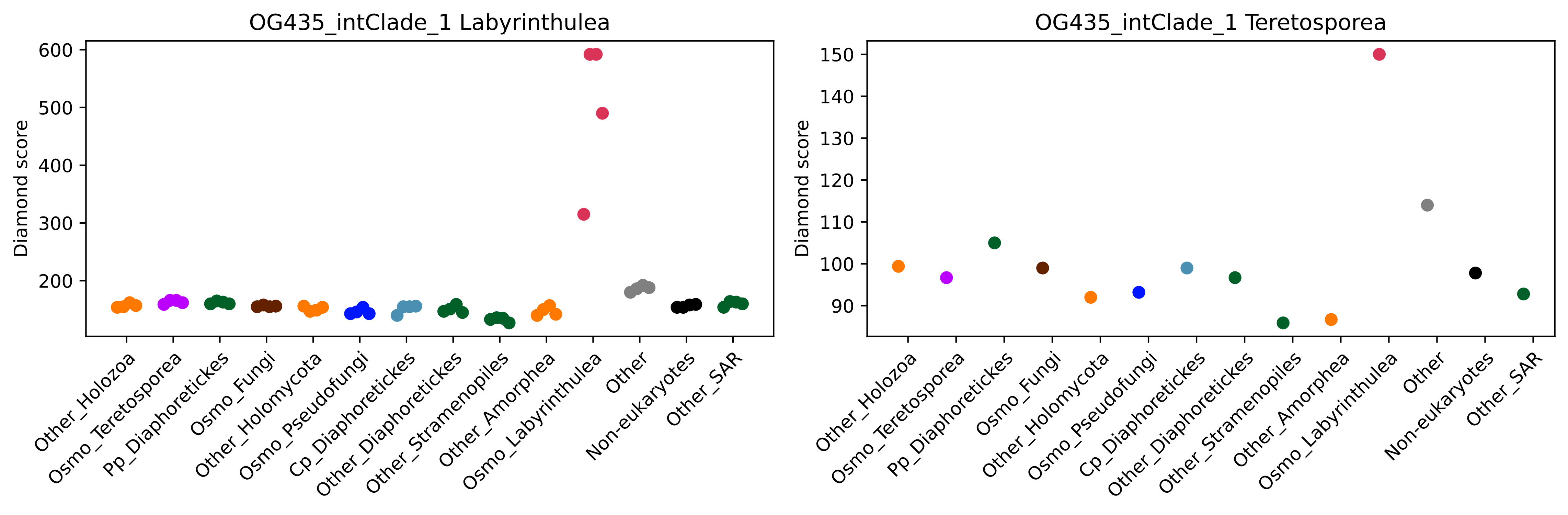
OG435_intClade_1 Labyrinthulea
Diamond score
600
500
400
300
200
Other_Holozoa
Osmo_Teretosporea
Pp_Diaphoretickes
Osmo_Fungi
Other_Holomycota
Osmo_Pseudofungi
Cp_Diaphoretickes
Other_Diaphoretickes
Other_Stramenopiles
Other_Amorphea
Osmo_Labyrinthulea
Other
Non-eukaryotes
Other_SAR
OG435_intClade_1 Teretosporea
Diamond score
150
140
130
120
110
100
90

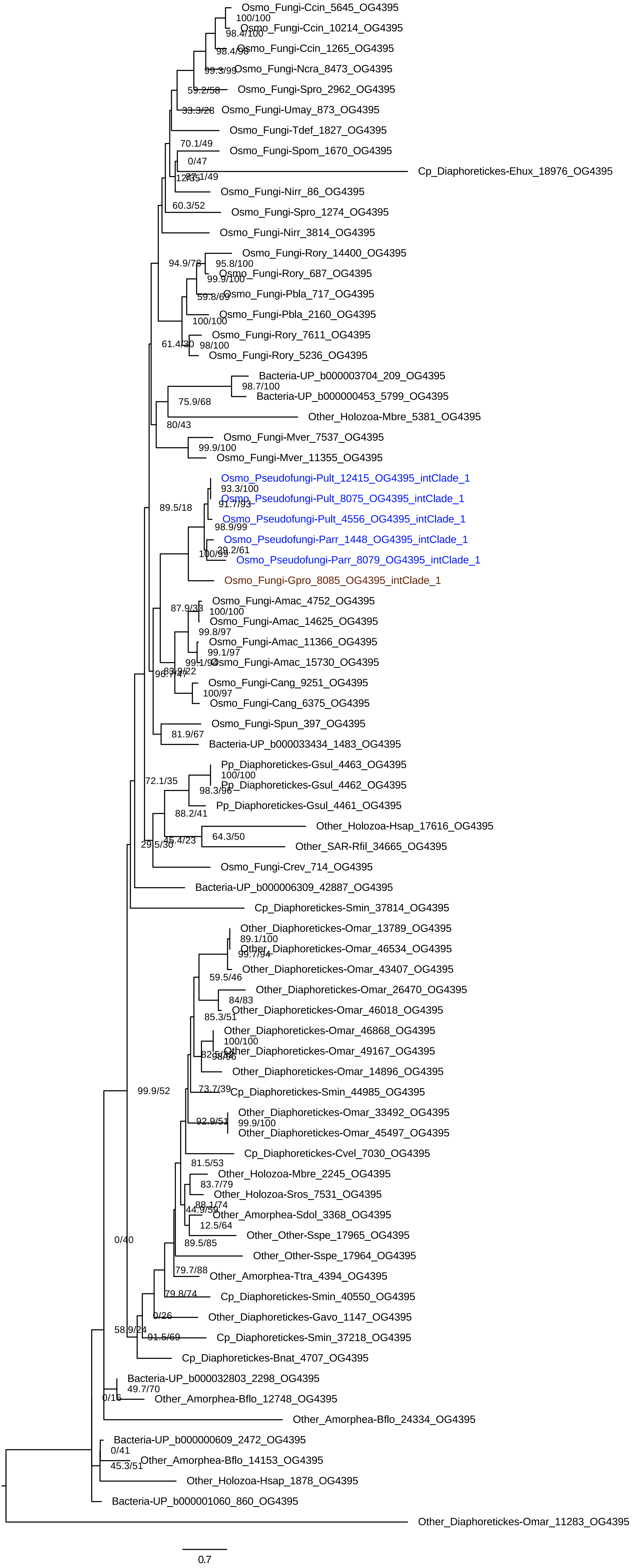

Osmo_Fungi-Ccin_5645_OG4395
Osmo_Fungi-Ccin_10214_OG4395
Osmo_Fungi-Ccin_1265_OG4395
Osmo_Fungi-Ncra_8473_OG4395
Osmo_Fungi-Spro_2962_OG4395
Osmo_Fungi-Umay_873_OG4395
Osmo_Fungi-Tdef_1827_OG4395
Osmo_Fungi-Spom_1670_OG4395
Cp_Diaphoretickes-Ehux_18976_OG4395
Osmo_Fungi-Nirr_86_OG4395
Osmo_Fungi-Spro_1274_OG4395
Osmo_Fungi-Nirr_3814_OG4395
Osmo_Fungi-Rory_14400_OG4395
Osmo_Fungi-Rory_687_OG4395
Osmo_Fungi-Pbla_717_OG4395
Osmo_Fungi-Pbla_2160_OG4395
Osmo_Fungi-Rory_7611_OG4395
Osmo_Fungi-Rory_5236_OG4395
Bacteria-UP_b000003704_209_OG4395
Bacteria-UP_b000000453_5799_OG4395
Other_Holozoa-Mbre_5381_OG4395
Osmo_Fungi-Mver_7537_OG4395
Osmo_Fungi-Mver_11355_OG4395
Osmo_Pseudofungi-Pult_12415_OG4395_intClade_1
Osmo_Pseudofungi-Pult_8075_OG4395_intClade_1
Osmo_Pseudofungi-Pult_4556_OG4395_intClade_1
Osmo_Pseudofungi-Parr_1448_OG4395_intClade_1
Osmo_Pseudofungi-Parr_8079_OG4395_intClade_1
Osmo_Fungi-Gpro_8085_OG4395_intClade_1
Osmo_Fungi-Amac_4752_OG4395
Osmo_Fungi-Amac_14625_OG4395
Osmo_Fungi-Amac_11366_OG4395
Osmo_Fungi-Amac_15730_OG4395
Osmo_Fungi-Cang_9251_OG4395
Osmo_Fungi-Cang_6375_OG4395
Osmo_Fungi-Spun_397_OG4395
Bacteria-UP_b000033434_1483_OG4395
Pp_Diaphoretickes-Gsul_4463_OG4395
Pp_Diaphoretickes-Gsul_4462_OG4395
Pp_Diaphoretickes-Gsul_4461_OG4395
Other_Holozoa-Hsap_17616_OG4395
Other_SAR-Rfil_34665_OG4395
Osmo_Fungi-Crev_714_OG4395
Bacteria-UP_b000006309_42887_OG4395
Cp_Diaphoretickes-Smin_37814_OG4395
Other_Diaphoretickes-Omar_13789_OG4395
Other_Diaphoretickes-Omar_46534_OG4395
Other_Diaphoretickes-Omar_43407_OG4395
Other_Diaphoretickes-Omar_26470_OG4395
Other_Diaphoretickes-Omar_46018_OG4395
Other_Diaphoretickes-Omar_46868_OG4395
Other_Diaphoretickes-Omar_49167_OG4395
Other_Diaphoretickes-Omar_14896_OG4395
Cp_Diaphoretickes-Smin_44985_OG4395
Other_Diaphoretickes-Omar_33492_OG4395
Other_Diaphoretickes-Omar_45497_OG4395
Cp_Diaphoretickes-Cvel_7030_OG4395
Other_Holozoa-Mbre_2245_OG4395
Other_Holozoa-Sros_7531_OG4395
Other_Amorphea-Sdol_3368_OG4395
Other_Other-Sspe_17965_OG4395
Other_Other-Sspe_17964_OG4395
Other_Amorphea-Ttra_4394_OG4395
Cp_Diaphoretickes-Smin_40550_OG4395
Other_Diaphoretickes-Gavo_1147_OG4395
Cp_Diaphoretickes-Smin_37218_OG4395
Cp_Diaphoretickes-Bnat_4707_OG4395
Bacteria-UP_b000032803_2298_OG4395
Other_Amorphea-Bflo_12748_OG4395
Other_Amorphea-Bflo_24334_OG4395
Bacteria-UP_b000000609_2472_OG4395
Other_Amorphea-Bflo_14153_OG4395
Other_Holozoa-Hsap_1878_OG4395
Bacteria-UP_b000001060_860_OG4395
Other_Diaphoretickes-Omar_11283_OG4395
0.7

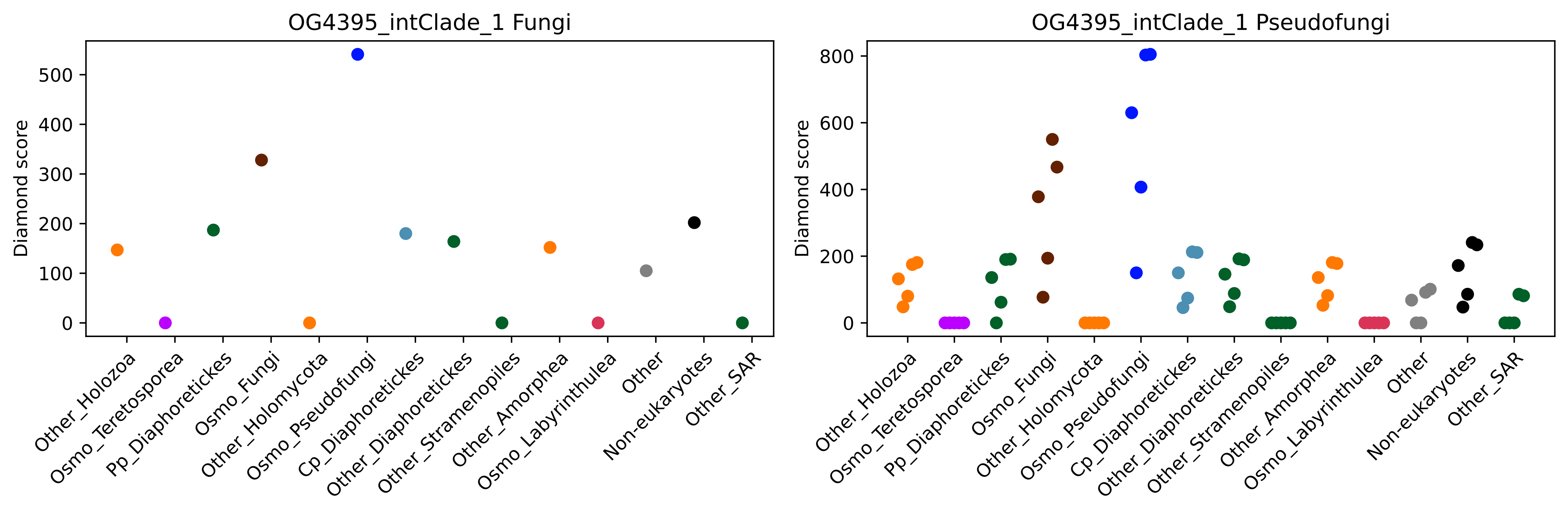
OG4395_intClade_1 Fungi
OG4395_intClade_1 Pseudofungi
Diamond score
0
100
200
300
400
500
600
800
Other_Holozoa
Osmo_Teretosporea
Pp_Diaphoretickes
Osmo_Fungi
Other_Holomycota
Osmo_Pseudofungi
Cp_Diaphoretickes
Other_Diaphoretickes
Other_Stramenopiles
Other_Amorphea
Osmo_Labyrinthulea
Other
Non-eukaryotes
Other_SAR

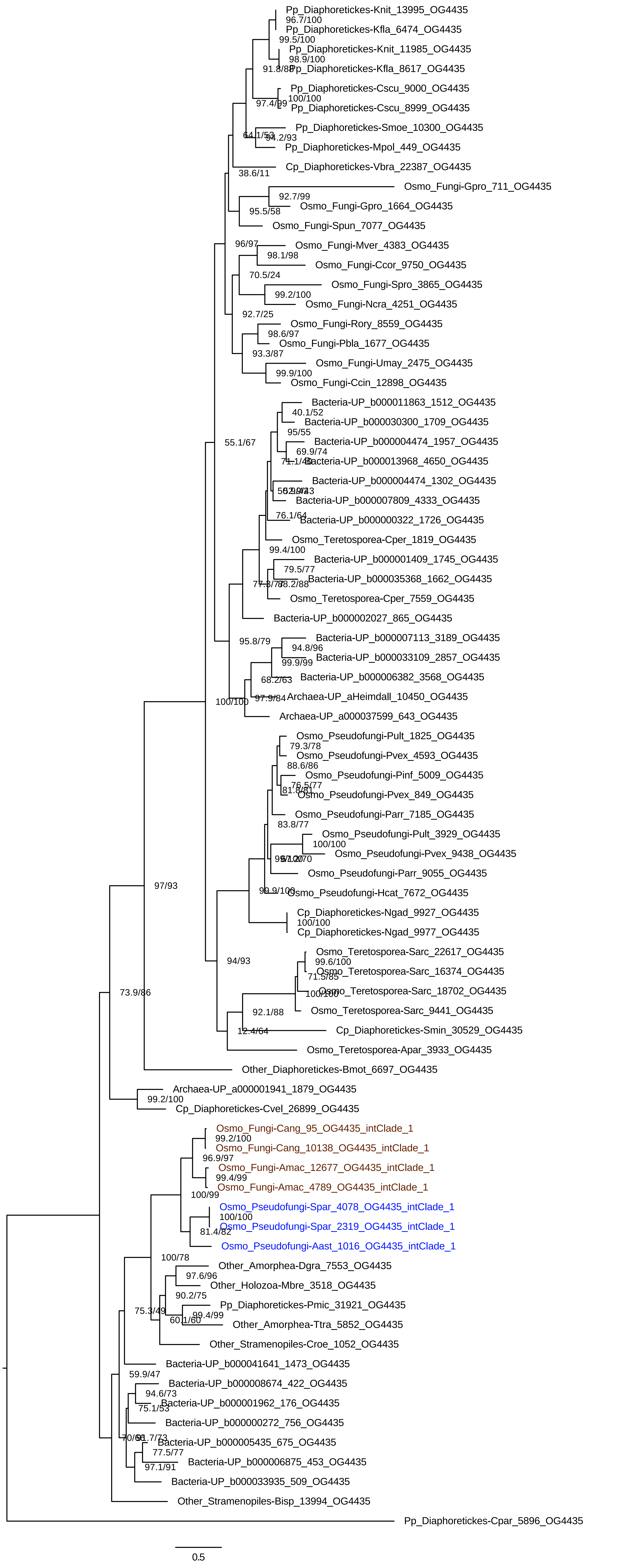

Pp_Diaphoretickes-Knit_13995_OG4435
96.7/100
Pp_Diaphoretickes-Kfla_6474_OG4435
99.5/100
Pp_Diaphoretickes-Knit_11985_OG4435
98.9/100
91.8/88
Pp_Diaphoretickes-Kfla_8617_OG4435
Pp_Diaphoretickes-Cscu_9000_OG4435
100/100
97.4/99
Pp_Diaphoretickes-Cscu_8999_OG4435
Pp_Diaphoretickes-Smoe_10300_OG4435
64.1/53
94.2/93
Pp_Diaphoretickes-Mpol_449_OG4435
Cp_Diaphoretickes-Vbra_22387_OG4435
38.6/11
Osmo_Fungi-Gpro_711_OG4435
92.7/99
Osmo_Fungi-Gpro_1664_OG4435
95.5/58
Osmo_Fungi-Spun_7077_OG4435
96/97
Osmo_Fungi-Mver_4383_OG4435
98.1/98
Osmo_Fungi-Ccor_9750_OG4435
70.5/24
Osmo_Fungi-Spro_3865_OG4435
99.2/100
Osmo_Fungi-Ncra_4251_OG4435
92.7/25
Osmo_Fungi-Rory_8559_OG4435
98.6/97
Osmo_Fungi-Pbla_1677_OG4435
93.3/87
Osmo_Fungi-Umay_2475_OG4435
99.9/100
Osmo_Fungi-Ccin_12898_OG4435
Bacteria-UP_b000011863_1512_OG4435
40.1/52
Bacteria-UP_b000030300_1709_OG4435
95/55
55.1/67
Bacteria-UP_b000004474_1957_OG4435
69.9/74
Bacteria-UP_b000013968_4650_OG4435
Bacteria-UP_b000004474_1302_OG4435
Bacteria-UP_b000007809_4333_OG4435
76.1/64
Bacteria-UP_b000000322_1726_OG4435
Osmo_Teretosporea-Cper_1819_OG4435
99.4/100
Bacteria-UP_b000001409_1745_OG4435
79.5/77
Bacteria-UP_b000035368_1662_OG4435
Osmo_Teretosporea-Cper_7559_OG4435
Bacteria-UP_b000002027_865_OG4435
95.8/79
Bacteria-UP_b000007113_3189_OG4435
94.8/96
Bacteria-UP_b000033109_2857_OG4435
99.9/99
68.2/63
Bacteria-UP_b000006382_3568_OG4435
100/100
97.9/84
Archaea-UP_aHeimdall_10450_OG4435
Archaea-UP_a000037599_643_OG4435
Osmo_Pseudofungi-Pult_1825_OG4435
79.3/78
Osmo_Pseudofungi-Pvex_4593_OG4435
88.6/86
Osmo_Pseudofungi-Pinf_5009_OG4435
76.5/77
Osmo_Pseudofungi-Pvex_849_OG4435
Osmo_Pseudofungi-Parr_7185_OG4435
83.8/77
Osmo_Pseudofungi-Pult_3929_OG4435
100/100
Osmo_Pseudofungi-Pvex_9438_OG4435
Osmo_Pseudofungi-Parr_9055_OG4435
97/93
99.9/100
Osmo_Pseudofungi-Hcat_7672_OG4435
Cp_Diaphoretickes-Ngad_9927_OG4435
100/100
Cp_Diaphoretickes-Ngad_9977_OG4435
94/93
Osmo_Teretosporea-Sarc_22617_OG4435
99.6/100
Osmo_Teretosporea-Sarc_16374_OG4435
71.5/85
73.9/86
Osmo_Teretosporea-Sarc_18702_OG4435
100/100
Osmo_Teretosporea-Sarc_9441_OG4435
92.1/88
12.4/64
Cp_Diaphoretickes-Smin_30529_OG4435
Osmo_Teretosporea-Apar_3933_OG4435
Other_Diaphoretickes-Bmot_6697_OG4435
Archaea-UP_a000001941_1879_OG4435
99.2/100
Cp_Diaphoretickes-Cvel_26899_OG4435
Osmo_Fungi-Cang_95_OG4435_intClade_1
99.2/100
Osmo_Fungi-Cang_10138_OG4435_intClade_1
96.9/97
Osmo_Fungi-Amac_12677_OG4435_intClade_1
99.4/99
Osmo_Fungi-Amac_4789_OG4435_intClade_1
100/99
Osmo_Pseudofungi-Spar_4078_OG4435_intClade_1
100/100
Osmo_Pseudofungi-Spar_2319_OG4435_intClade_1
81.4/82
Osmo_Pseudofungi-Aast_1016_OG4435_intClade_1
100/78
Other_Amorphea-Dgra_7553_OG4435
97.6/96
Other_Holozoa-Mbre_3518_OG4435
90.2/75
75.3/49
Pp_Diaphoretickes-Pmic_31921_OG4435
99.4/99
60.1/60
Other_Amorphea-Ttra_5852_OG4435
Other_Stramenopiles-Croe_1052_OG4435
Bacteria-UP_b000041641_1473_OG4435
59.9/47
Bacteria-UP_b000008674_422_OG4435
94.6/73
Bacteria-UP_b000001962_176_OG4435
75.1/53
Bacteria-UP_b000000272_756_OG4435
Bacteria-UP_b000005435_675_OG4435
77.5/77
Bacteria-UP_b000006875_453_OG4435
97.1/91
Bacteria-UP_b000033935_509_OG4435
Other_Stramenopiles-Bisp_13994_OG4435
Pp_Diaphoretickes-Cpar_5896_OG4435
0.5

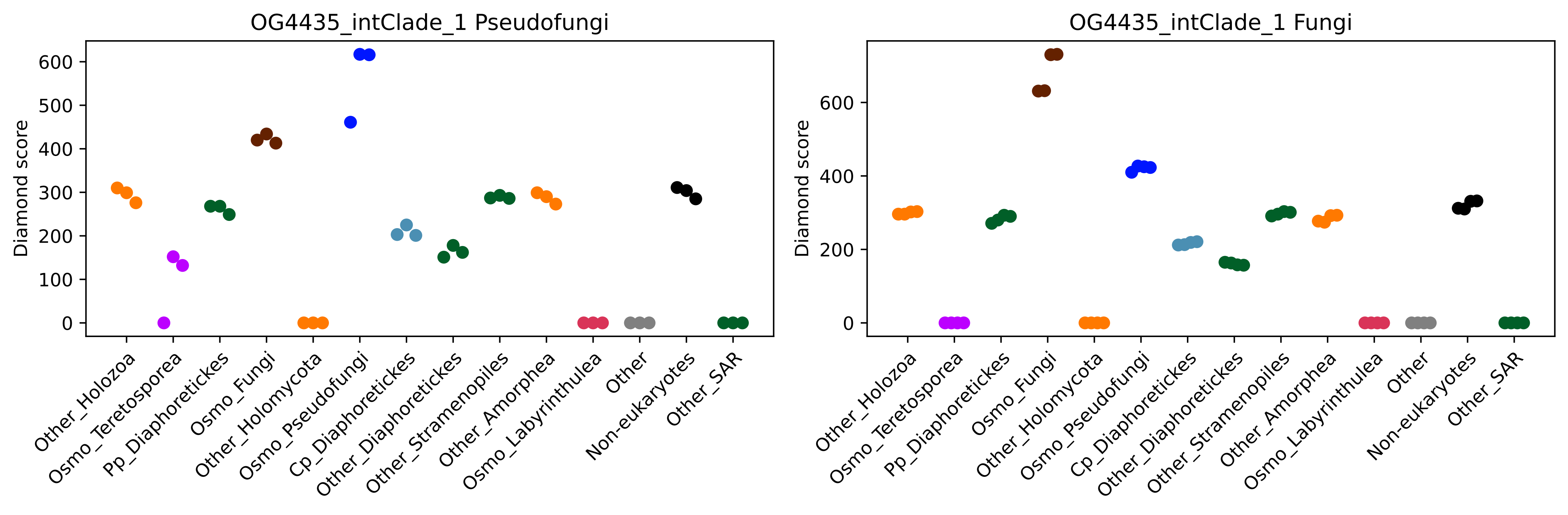

OG4435_intClade_1 Pseudofungi
OG4435_intClade_1 Fungi
Diamond score
0
100
200
300
400
500
600
Other_Holozoa
Osmo_Teretosporea
Pp_Diaphoretickes
Osmo_Fungi
Other_Holomycota
Osmo_Pseudofungi
Cp_Diaphoretickes
Other_Diaphoretickes
Other_Stramenopiles
Other_Amorphea
Osmo_Labyrinthulea
Other
Non-eukaryotes
Other_SAR

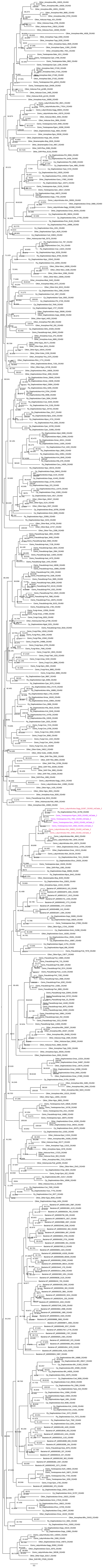

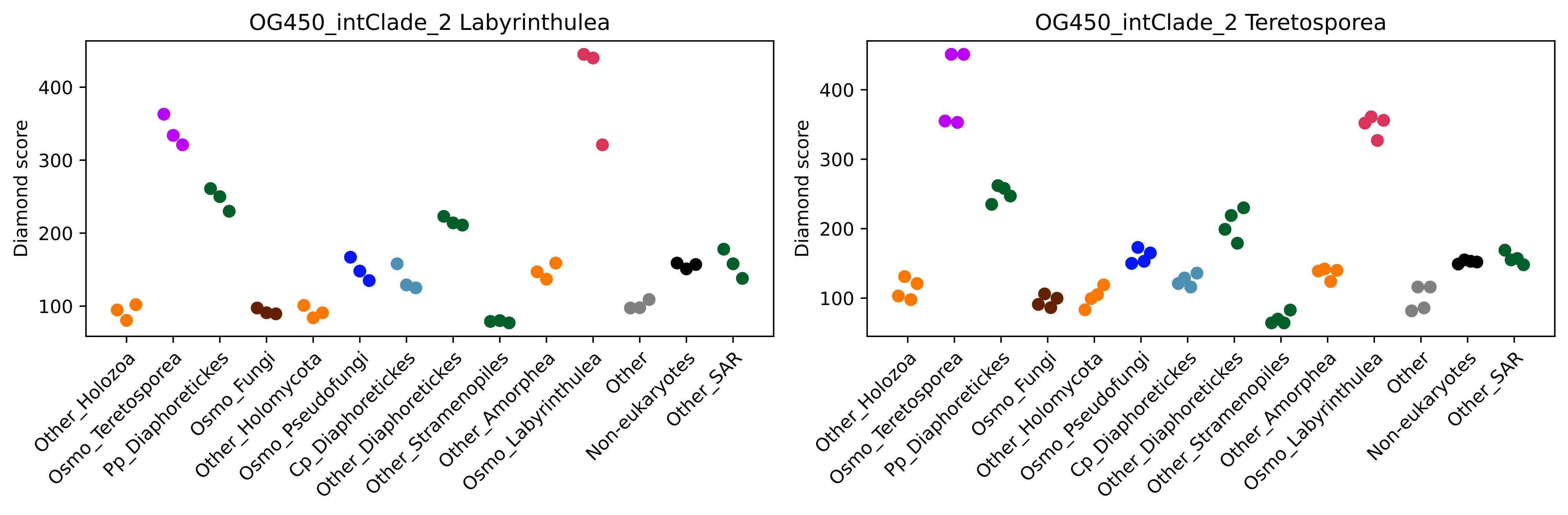
OG450_intClade_2 Labyrinthulea
Diamond score
400
300
200
100
Other_Holozoa
Osmo_Teretosporea
Pp_Diaphoretickes
Osmo_Fungi
Other_Holomycota
Osmo_Pseudofungi
Cp_Diaphoretickes
Other_Diaphoretickes
Other_Stramenopiles
Other_Amorphea
Osmo_Labyrinthulea
Other
Non-eukaryotes
Other_SAR
OG450_intClade_2 Teretosporea
Diamond score
400
300
200
100
Other_Holozoa
Osmo_Teretosporea
Pp_Diaphoretickes
Osmo_Fungi
Other_Holomycota
Osmo_Pseudofungi
Cp_Diaphoretickes
Other_Diaphoretickes
Other_Stramenopiles
Other_Amorphea
Osmo_Labyrinthulea
Other
Non-eukaryotes
Other_SAR

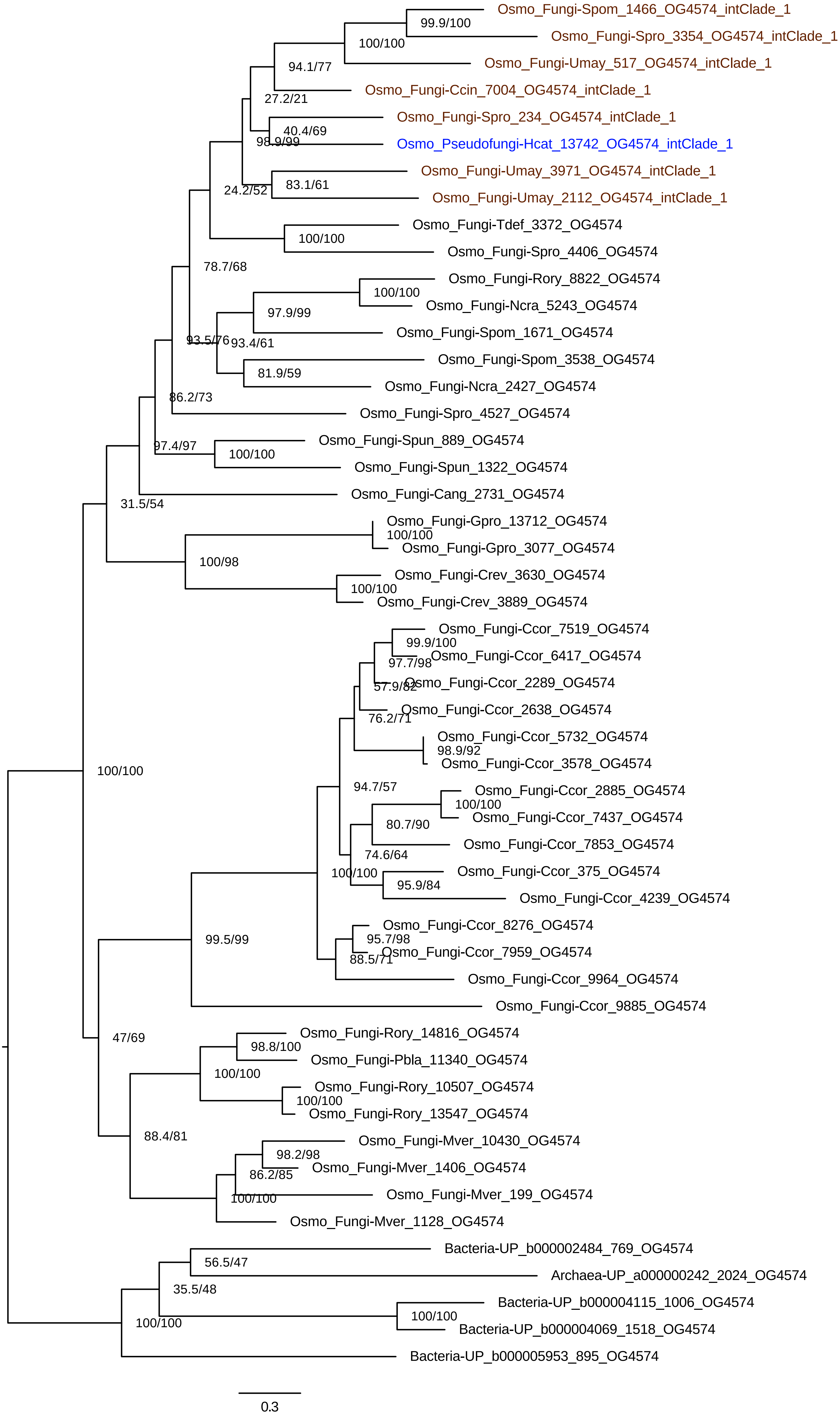
Osmo_Fungi-Spom_1466_OG4574_intClade_1
99.9/100
Osmo_Fungi-Spro_3354_OG4574_intClade_1
100/100
Osmo_Fungi-Umay_517_OG4574_intClade_1
94.1/77
Osmo_Fungi-Ccin_7004_OG4574_intClade_1
27.2/21
Osmo_Fungi-Spro_234_OG4574_intClade_1
40.4/69
98.9/99
Osmo_Pseudofungi-Hcat_13742_OG4574_intClade_1
Osmo_Fungi-Umay_3971_OG4574_intClade_1
83.1/61
24.2/52
Osmo_Fungi-Umay_2112_OG4574_intClade_1
Osmo_Fungi-Tdef_3372_OG4574
100/100
Osmo_Fungi-Spro_4406_OG4574
78.7/68
Osmo_Fungi-Rory_8822_OG4574
100/100
Osmo_Fungi-Ncra_5243_OG4574
97.9/99
Osmo_Fungi-Spom_1671_OG4574
93.5/76
93.4/61
Osmo_Fungi-Spom_3538_OG4574
81.9/59
Osmo_Fungi-Ncra_2427_OG4574
86.2/73
Osmo_Fungi-Spro_4527_OG4574
97.4/97
Osmo_Fungi-Spun_889_OG4574
100/100
Osmo_Fungi-Spun_1322_OG4574
Osmo_Fungi-Cang_2731_OG4574
31.5/54
Osmo_Fungi-Gpro_13712_OG4574
100/100
Osmo_Fungi-Gpro_3077_OG4574
100/98
Osmo_Fungi-Crev_3630_OG4574
100/100
Osmo_Fungi-Crev_3889_OG4574
Osmo_Fungi-Ccor_7519_OG4574
99.9/100
Osmo_Fungi-Ccor_6417_OG4574
97.7/98
57.9/82
Osmo_Fungi-Ccor_2289_OG4574
Osmo_Fungi-Ccor_2638_OG4574
76.2/71
Osmo_Fungi-Ccor_5732_OG4574
98.9/92
Osmo_Fungi-Ccor_3578_OG4574
100/100
94.7/57
Osmo_Fungi-Ccor_2885_OG4574
100/100
Osmo_Fungi-Ccor_7437_OG4574
80.7/90
Osmo_Fungi-Ccor_7853_OG4574
74.6/64
Osmo_Fungi-Ccor_375_OG4574
100/100
95.9/84
Osmo_Fungi-Ccor_4239_OG4574
Osmo_Fungi-Ccor_8276_OG4574
95.7/98
99.5/99
Osmo_Fungi-Ccor_7959_OG4574
88.5/71
Osmo_Fungi-Ccor_9964_OG4574
Osmo_Fungi-Ccor_9885_OG4574
47/69
Osmo_Fungi-Rory_14816_OG4574
98.8/100
Osmo_Fungi-Pbla_11340_OG4574
100/100
Osmo_Fungi-Rory_10507_OG4574
100/100
Osmo_Fungi-Rory_13547_OG4574
88.4/81
Osmo_Fungi-Mver_10430_OG4574
98.2/98
Osmo_Fungi-Mver_1406_OG4574
86.2/85
100/100
Osmo_Fungi-Mver_199_OG4574
Osmo_Fungi-Mver_1128_OG4574
Bacteria-UP_b000002484_769_OG4574
56.5/47
Archaea-UP_a000000242_2024_OG4574
35.5/48
Bacteria-UP_b000004115_1006_OG4574
100/100
100/100
Bacteria-UP_b000004069_1518_OG4574
Bacteria-UP_b000005953_895_OG4574
0.3

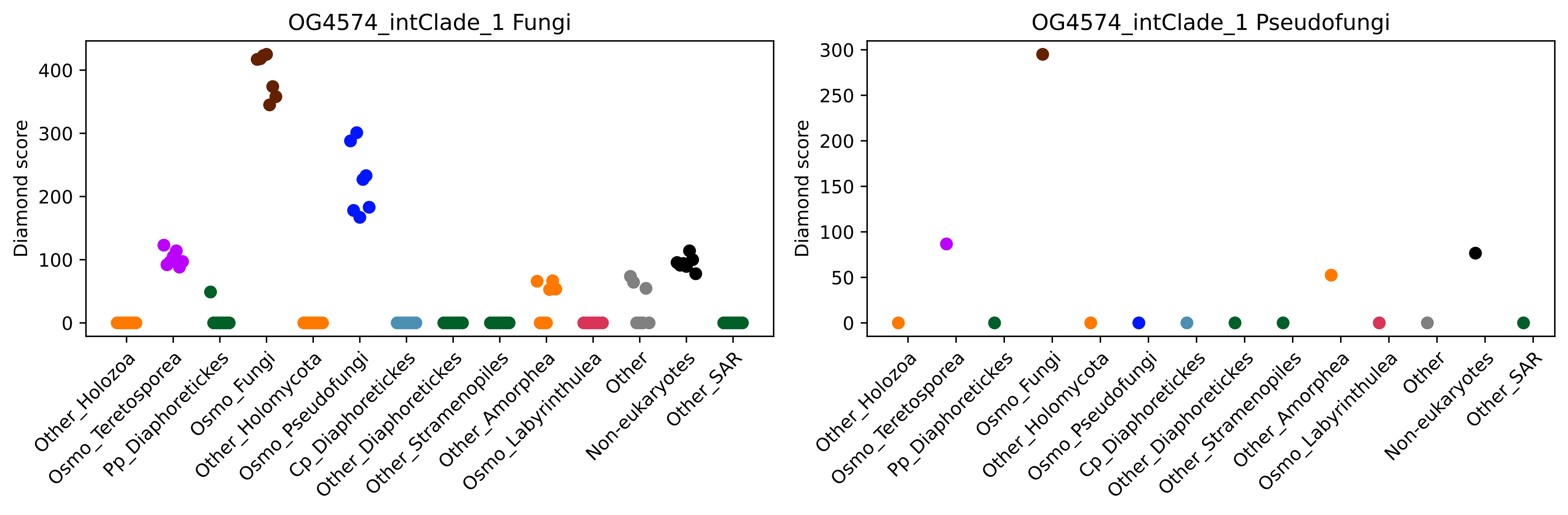

OG4574_intClade_1 Fungi
OG4574_intClade_1 Pseudofungi
Diamond score
Other_Holozoa
Osmo_Teretosporea
Pp_Diaphoretickes
Osmo_Fungi
Other_Holomycota
Osmo_Pseudofungi
Cp_Diaphoretickes
Other_Diaphoretickes
Other_Stramenopiles
Other_Amorphea
Osmo_Labyrinthulea
Other
Non-eukaryotes
Other_SAR

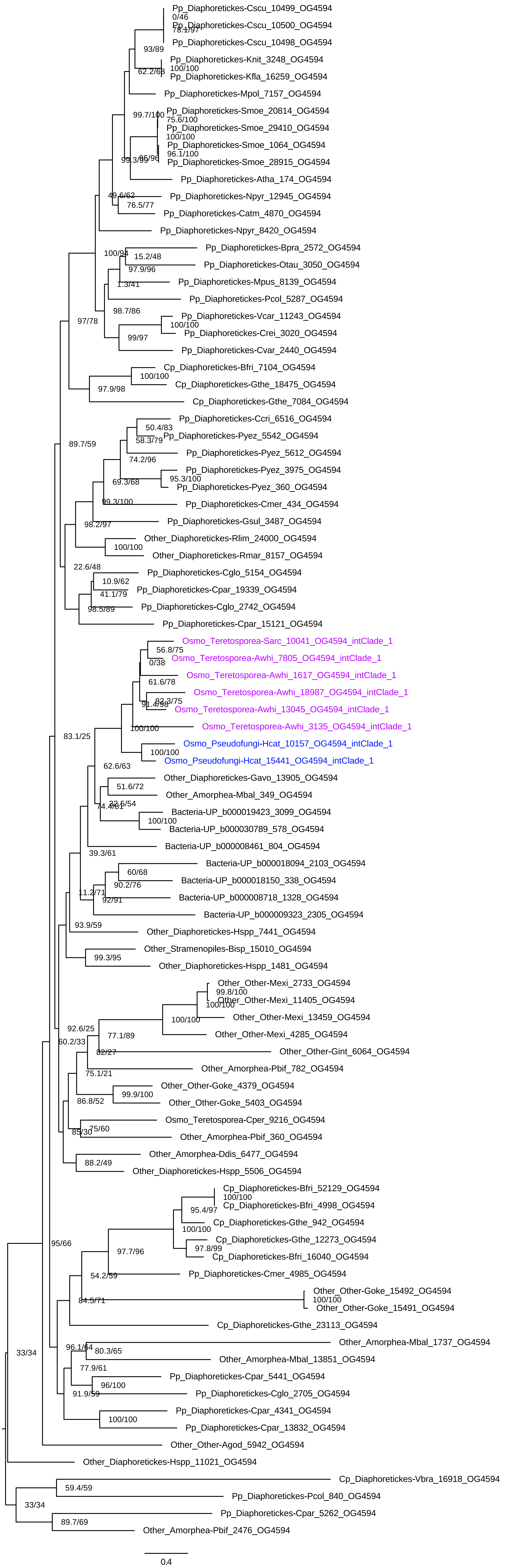

Pp_Diaphoretickes-Cscu_10499_OG4594
Pp_Diaphoretickes-Cscu_10500_OG4594
Pp_Diaphoretickes-Cscu_10498_OG4594
Pp_Diaphoretickes-Knit_3248_OG4594
Pp_Diaphoretickes-Kfla_16259_OG4594
Pp_Diaphoretickes-Mpol_7157_OG4594
Pp_Diaphoretickes-Smoe_20814_OG4594
Pp_Diaphoretickes-Smoe_29410_OG4594
Pp_Diaphoretickes-Smoe_1064_OG4594
Pp_Diaphoretickes-Smoe_28915_OG4594
Pp_Diaphoretickes-Atha_174_OG4594
Pp_Diaphoretickes-Npyr_12945_OG4594
Pp_Diaphoretickes-Catm_4870_OG4594
Pp_Diaphoretickes-Npyr_8420_OG4594
Pp_Diaphoretickes-Bpra_2572_OG4594
Pp_Diaphoretickes-Otau_3050_OG4594
Pp_Diaphoretickes-Mpus_8139_OG4594
Pp_Diaphoretickes-Pcol_5287_OG4594
Pp_Diaphoretickes-Vcar_11243_OG4594
Pp_Diaphoretickes-Crei_3020_OG4594
Pp_Diaphoretickes-Cvar_2440_OG4594
Cp_Diaphoretickes-Bfri_7104_OG4594
Cp_Diaphoretickes-Gthe_18475_OG4594
Cp_Diaphoretickes-Gthe_7084_OG4594
Pp_Diaphoretickes-Ccri_6516_OG4594
Pp_Diaphoretickes-Pyez_5542_OG4594
Pp_Diaphoretickes-Pyez_5612_OG4594
Pp_Diaphoretickes-Pyez_3975_OG4594
Pp_Diaphoretickes-Pyez_360_OG4594
Pp_Diaphoretickes-Cmer_434_OG4594
Pp_Diaphoretickes-Gsul_3487_OG4594
Other_Diaphoretickes-Rlim_24000_OG4594
Other_Diaphoretickes-Rmar_8157_OG4594
Pp_Diaphoretickes-Cglo_5154_OG4594
Pp_Diaphoretickes-Cpar_19339_OG4594
Pp_Diaphoretickes-Cglo_2742_OG4594
Pp_Diaphoretickes-Cpar_15121_OG4594
Osmo_Teretosporea-Sarc_10041_OG4594_intClade_1
Osmo_Teretosporea-Awhi_7805_OG4594_intClade_1
Osmo_Teretosporea-Awhi_1617_OG4594_intClade_1
Osmo_Teretosporea-Awhi_18987_OG4594_intClade_1
Osmo_Teretosporea-Awhi_13045_OG4594_intClade_1
Osmo_Teretosporea-Awhi_3135_OG4594_intClade_1
Osmo_Pseudofungi-Hcat_10157_OG4594_intClade_1
Osmo_Pseudofungi-Hcat_15441_OG4594_intClade_1
Other_Diaphoretickes-Gavo_13905_OG4594
Other_Amorphea-Mbal_349_OG4594
Bacteria-UP_b000019423_3099_OG4594
Bacteria-UP_b000030789_578_OG4594
Bacteria-UP_b000008461_804_OG4594
Bacteria-UP_b000018094_2103_OG4594
Bacteria-UP_b000018150_338_OG4594
Bacteria-UP_b000008718_1328_OG4594
Bacteria-UP_b000009323_2305_OG4594
Other_Diaphoretickes-Hspp_7441_OG4594
Other_Stramenopiles-Bisp_15010_OG4594
Other_Diaphoretickes-Hspp_1481_OG4594
Other_Other-Mexi_2733_OG4594
Other_Other-Mexi_11405_OG4594
Other_Other-Mexi_13459_OG4594
Other_Other-Mexi_4285_OG4594
Other_Other-Gint_6064_OG4594
Other_Amorphea-Pbif_782_OG4594
Other_Other-Goke_4379_OG4594
Other_Other-Goke_5403_OG4594
Osmo_Teretosporea-Cper_9216_OG4594
Other_Amorphea-Pbif_360_OG4594
Other_Amorphea-Ddis_6477_OG4594
Other_Diaphoretickes-Hspp_5506_OG4594
Cp_Diaphoretickes-Bfri_52129_OG4594
Cp_Diaphoretickes-Bfri_4998_OG4594
Cp_Diaphoretickes-Gthe_942_OG4594
Cp_Diaphoretickes-Gthe_12273_OG4594
Cp_Diaphoretickes-Bfri_16040_OG4594
Pp_Diaphoretickes-Cmer_4985_OG4594
Other_Other-Goke_15492_OG4594
Other_Other-Goke_15491_OG4594
Cp_Diaphoretickes-Gthe_23113_OG4594
Other_Amorphea-Mbal_1737_OG4594
Other_Amorphea-Mbal_13851_OG4594
Pp_Diaphoretickes-Cpar_5441_OG4594
Pp_Diaphoretickes-Cglo_2705_OG4594
Pp_Diaphoretickes-Cpar_4341_OG4594
Pp_Diaphoretickes-Cpar_13832_OG4594
Other_Other-Agod_5942_OG4594
Other_Diaphoretickes-Hspp_11021_OG4594
Cp_Diaphoretickes-Vbra_16918_OG4594
Pp_Diaphoretickes-Pcol_840_OG4594
Pp_Diaphoretickes-Cpar_5262_OG4594
Other_Amorphea-Pbif_2476_OG4594
0.4

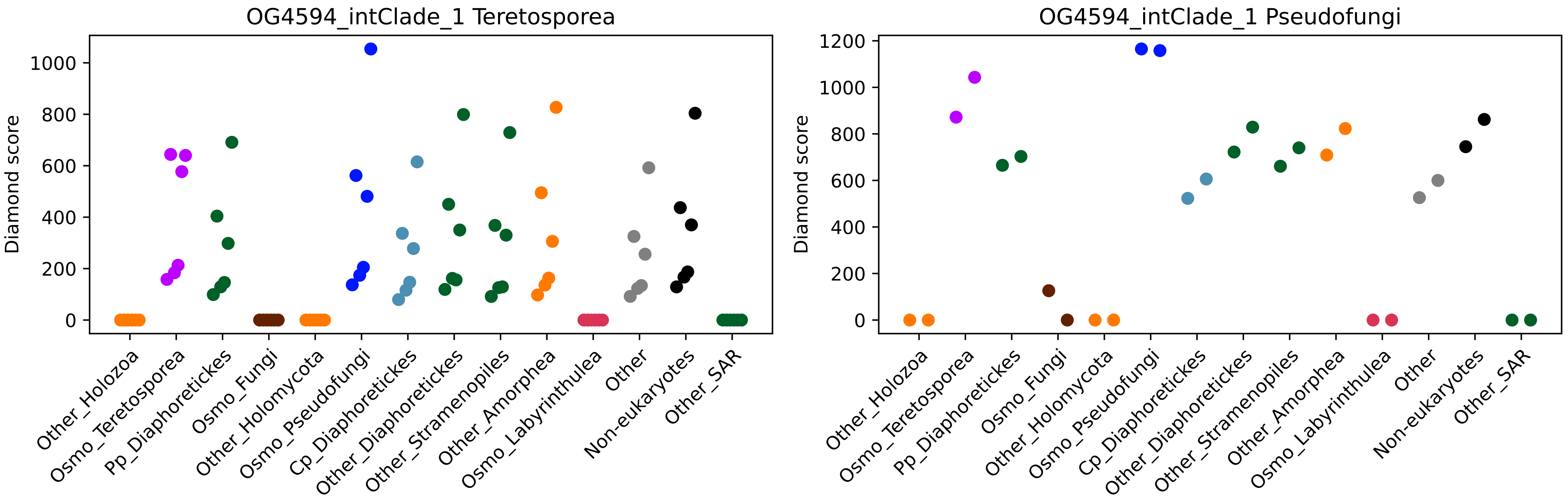

OG4594_intClade_1 Teretosporea
OG4594_intClade_1 Pseudofungi
Diamond score
0
200
400
600
800
1000
1200
Other_Holozoa
Osmo_Teretosporea
Pp_Diaphoretickes
Osmo_Fungi
Other_Holomycota
Osmo_Pseudofungi
Cp_Diaphoretickes
Other_Diaphoretickes
Other_Stramenopiles
Other_Amorphea
Osmo_Labyrinthulea
Other
Non-eukaryotes
Other_SAR

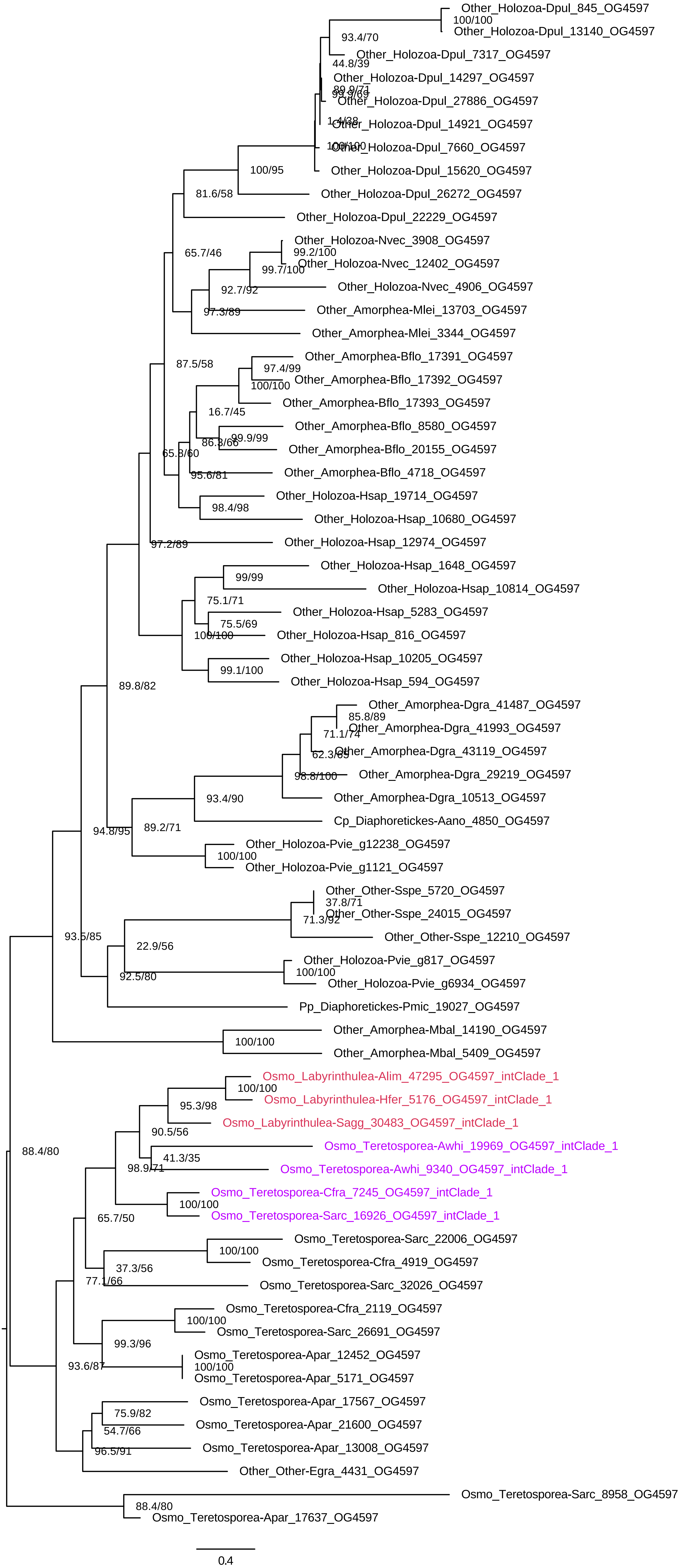

Other_Holozoa-Dpul_845_OG4597
100/100
Other_Holozoa-Dpul_13140_OG4597
93.4/70
Other_Holozoa-Dpul_7317_OG4597
44.8/39
Other_Holozoa-Dpul_14297_OG4597
Other_Holozoa-Dpul_27886_OG4597
Other_Holozoa-Dpul_14921_OG4597
Other_Holozoa-Dpul_7660_OG4597
100/95
Other_Holozoa-Dpul_15620_OG4597
81.6/58
Other_Holozoa-Dpul_26272_OG4597
Other_Holozoa-Dpul_22229_OG4597
Other_Holozoa-Nvec_3908_OG4597
99.2/100
65.7/46
99.7/100
Other_Holozoa-Nvec_12402_OG4597
92.7/92
Other_Holozoa-Nvec_4906_OG4597
97.3/89
Other_Amorphea-Mlei_13703_OG4597
Other_Amorphea-Mlei_3344_OG4597
87.5/58
Other_Amorphea-Bflo_17391_OG4597
97.4/99
100/100
Other_Amorphea-Bflo_17392_OG4597
Other_Amorphea-Bflo_17393_OG4597
16.7/45
Other_Amorphea-Bflo_8580_OG4597
99.9/99
Other_Amorphea-Bflo_20155_OG4597
95.6/81
Other_Amorphea-Bflo_4718_OG4597
Other_Holozoa-Hsap_19714_OG4597
98.4/98
Other_Holozoa-Hsap_10680_OG4597
97.2/89
Other_Holozoa-Hsap_12974_OG4597
Other_Holozoa-Hsap_1648_OG4597
99/99
Other_Holozoa-Hsap_10814_OG4597
75.1/71
Other_Holozoa-Hsap_5283_OG4597
75.5/69
Other_Holozoa-Hsap_816_OG4597
Other_Holozoa-Hsap_10205_OG4597
99.1/100
89.8/82
Other_Holozoa-Hsap_594_OG4597
Other_Amorphea-Dgra_41487_OG4597
85.8/89
Other_Amorphea-Dgra_41993_OG4597
71.1/74
Other_Amorphea-Dgra_43119_OG4597
Other_Amorphea-Dgra_29219_OG4597
93.4/90
Other_Amorphea-Dgra_10513_OG4597
Cp_Diaphoretickes-Aano_4850_OG4597
94.8/95
89.2/71
Other_Holozoa-Pvie_g12238_OG4597
100/100
Other_Holozoa-Pvie_g1121_OG4597
Other_Other-Sspe_5720_OG4597
37.8/71
Other_Other-Sspe_24015_OG4597
71.3/92
93.5/85
Other_Other-Sspe_12210_OG4597
22.9/56
Other_Holozoa-Pvie_g817_OG4597
100/100
92.5/80
Other_Holozoa-Pvie_g6934_OG4597
Pp_Diaphoretickes-Pmic_19027_OG4597
Other_Amorphea-Mbal_14190_OG4597
100/100
Other_Amorphea-Mbal_5409_OG4597
Osmo_Labyrinthulea-Alim_47295_OG4597_intClade_1
100/100
Osmo_Labyrinthulea-Hfer_5176_OG4597_intClade_1
95.3/98
Osmo_Labyrinthulea-Sagg_30483_OG4597_intClade_1
90.5/56
Osmo_Teretosporea-Awhi_19969_OG4597_intClade_1
88.4/80
41.3/35
98.9/71
Osmo_Teretosporea-Awhi_9340_OG4597_intClade_1
Osmo_Teretosporea-Cfra_7245_OG4597_intClade_1
100/100
Osmo_Teretosporea-Sarc_16926_OG4597_intClade_1
65.7/50
Osmo_Teretosporea-Sarc_22006_OG4597
100/100
Osmo_Teretosporea-Cfra_4919_OG4597
37.3/56
77.1/66
Osmo_Teretosporea-Sarc_32026_OG4597
Osmo_Teretosporea-Cfra_2119_OG4597
100/100
Osmo_Teretosporea-Sarc_26691_OG4597
99.3/96
Osmo_Teretosporea-Apar_12452_OG4597
93.6/87
100/100
Osmo_Teretosporea-Apar_5171_OG4597
Osmo_Teretosporea-Apar_17567_OG4597
75.9/82
Osmo_Teretosporea-Apar_21600_OG4597
54.7/66
Osmo_Teretosporea-Apar_13008_OG4597
96.5/91
Other_Other-Egra_4431_OG4597
Osmo_Teretosporea-Sarc_8958_OG4597
88.4/80
Osmo_Teretosporea-Apar_17637_OG4597
0.4

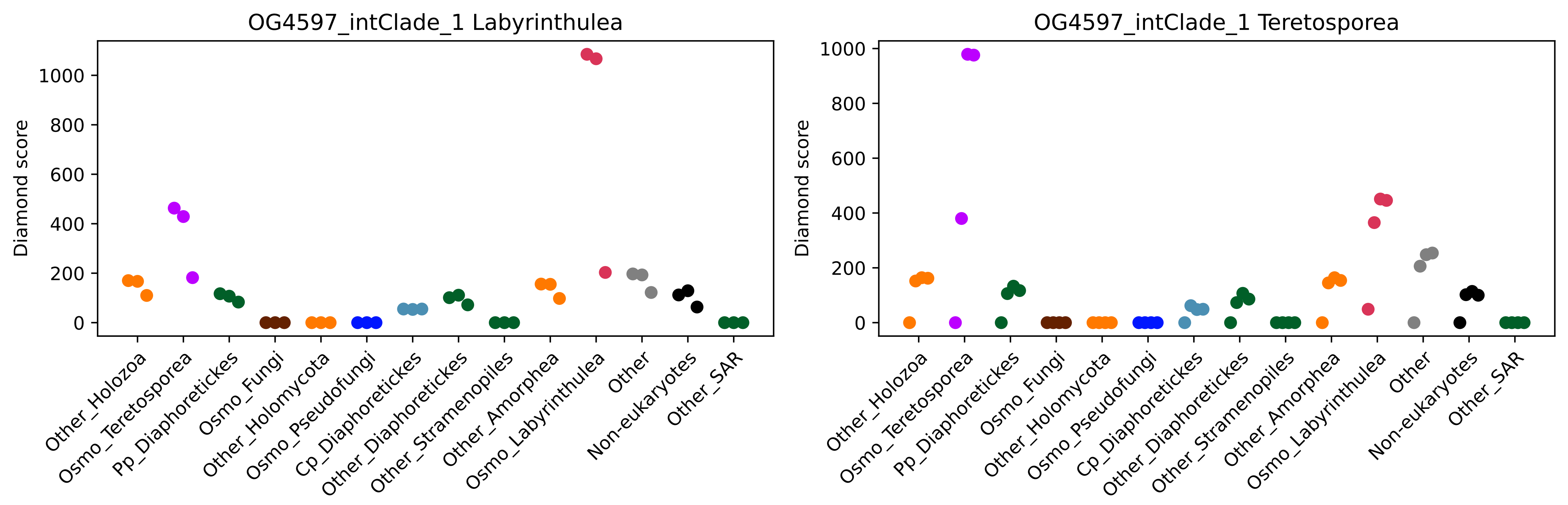

OG4597_intClade_1 Labyrinthulea
OG4597_intClade_1 Teretosporea
Diamond score
0
200
400
600
800
1000
Other_Holozoa
Osmo_Teretosporea
Pp_Diaphoretickes
Osmo_Fungi
Other_Holomycota
Osmo_Pseudofungi
Cp_Diaphoretickes
Other_Diaphoretickes
Other_Stramenopiles
Other_Amorphea
Osmo_Labyrinthulea
Other
Non-eukaryotes
Other_SAR

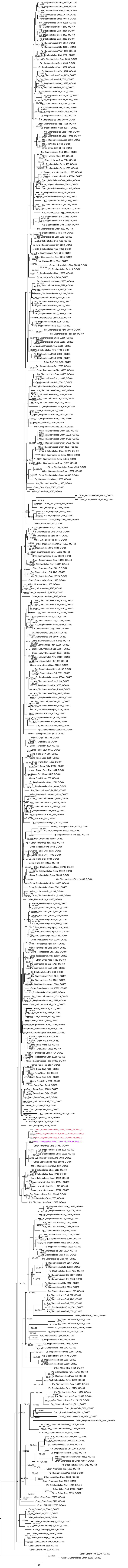

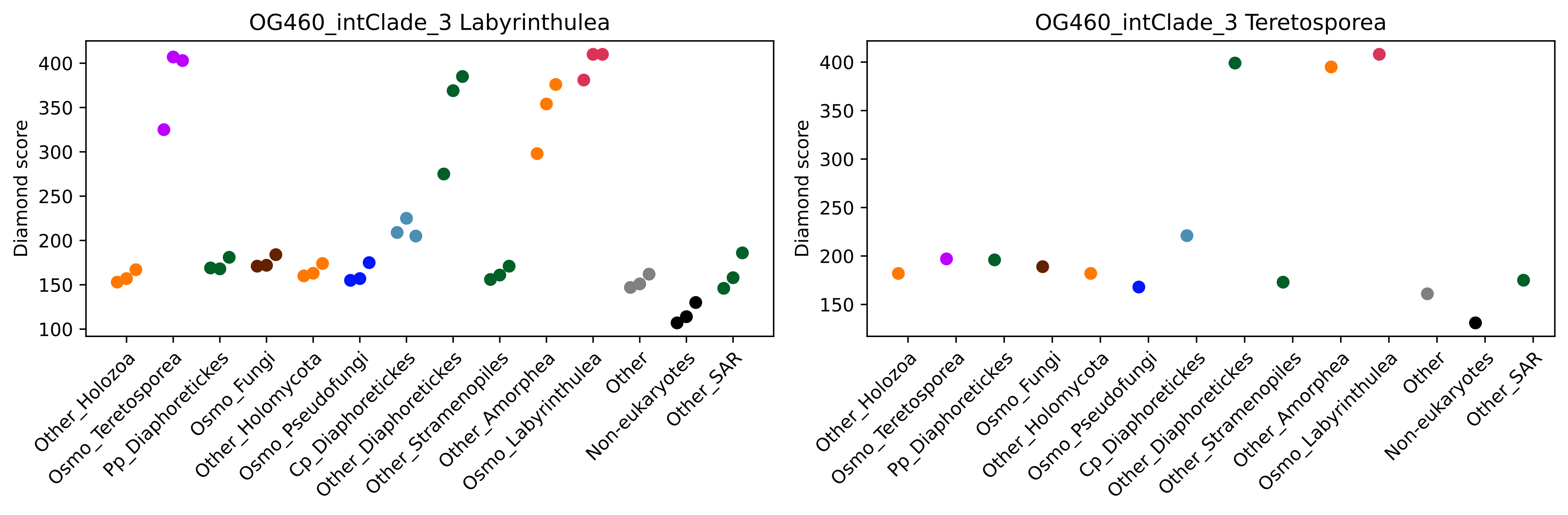

OG460_intClade_3 Labyrinthulea
OG460_intClade_3 Teretosporea
Diamond score
Other_Holozoa
Osmo_Teretosporea
Pp_Diaphoretickes
Osmo_Fungi
Other_Holomycota
Osmo_Pseudofungi
Cp_Diaphoretickes
Other_Diaphoretickes
Other_Stramenopiles
Other_Amorphea
Osmo_Labyrinthulea
Other
Non-eukaryotes
Other_SAR

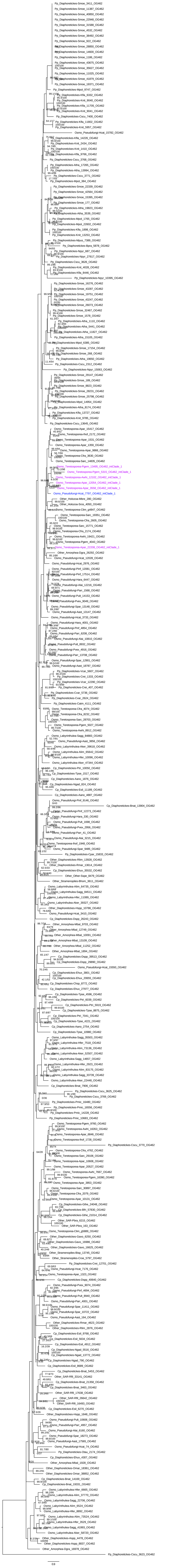

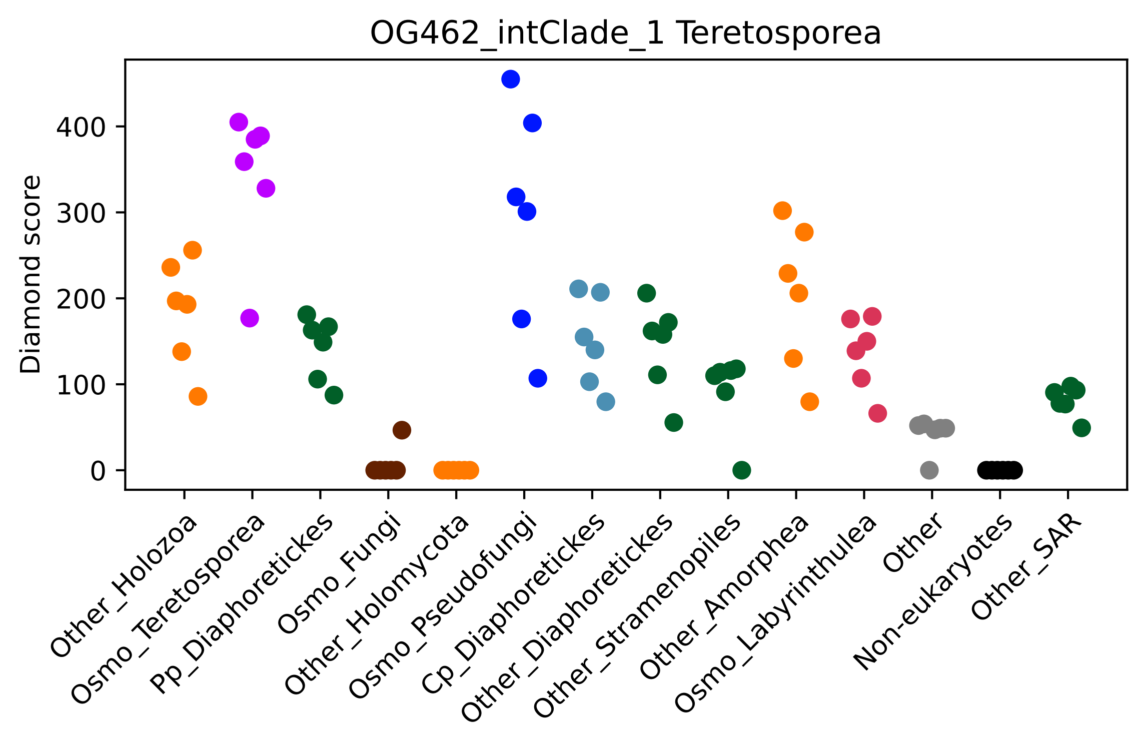

OG462_intClade_1 Teretosporea
Diamond score
0
100
200
300
400
Other_Holozoa
Osmo_Teretosporea
Pp_Diaphoretickes
Osmo_Fungi
Other_Holomycota
Osmo_Pseudofungi
Cp_Diaphoretickes
Other_Diaphoretickes
Other_Stramenopiles
Other_Amorphea
Osmo_Labyrinthulea
Other
Non-eukaryotes
Other_SAR

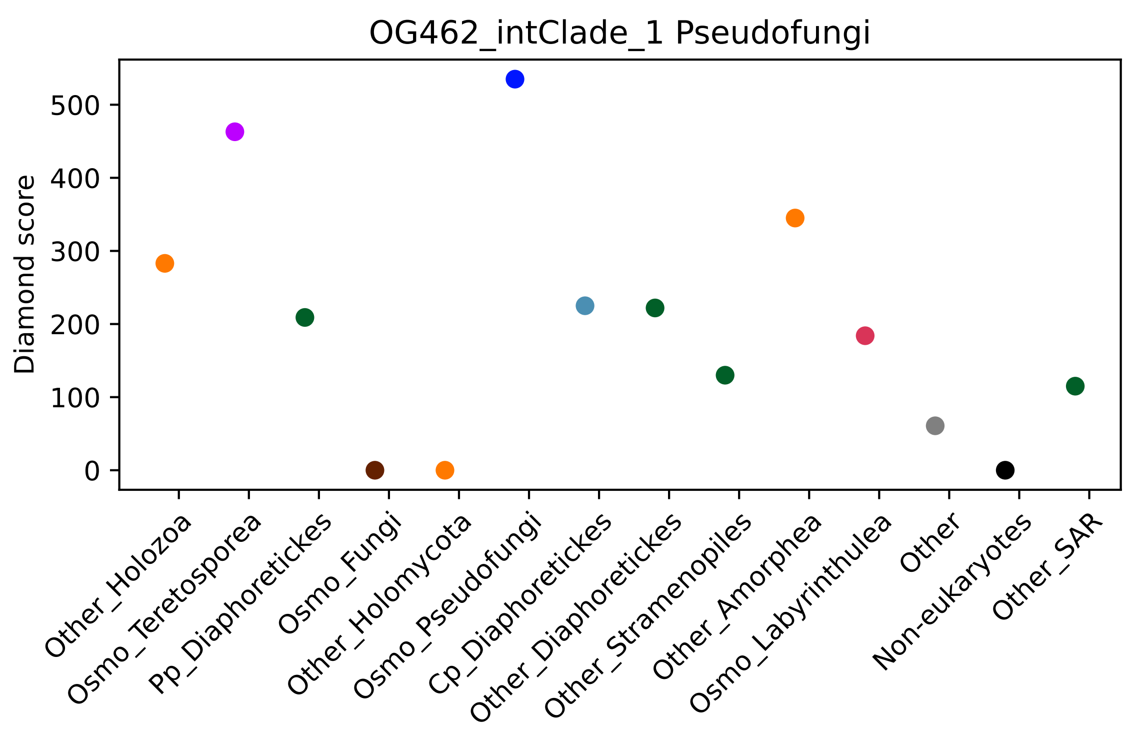

OG462_intClade_1 Pseudofungi
Diamond score
0
100
200
300
400
500
Other_Holozoa
Osmo_Teretosporea
Pp_Diaphoretickes
Osmo_Fungi
Other_Holomycota
Osmo_Pseudofungi
Cp_Diaphoretickes
Other_Diaphoretickes
Other_Stramenopiles
Other_Amorphea
Osmo_Labyrinthulea
Other
Non-eukaryotes
Other_SAR

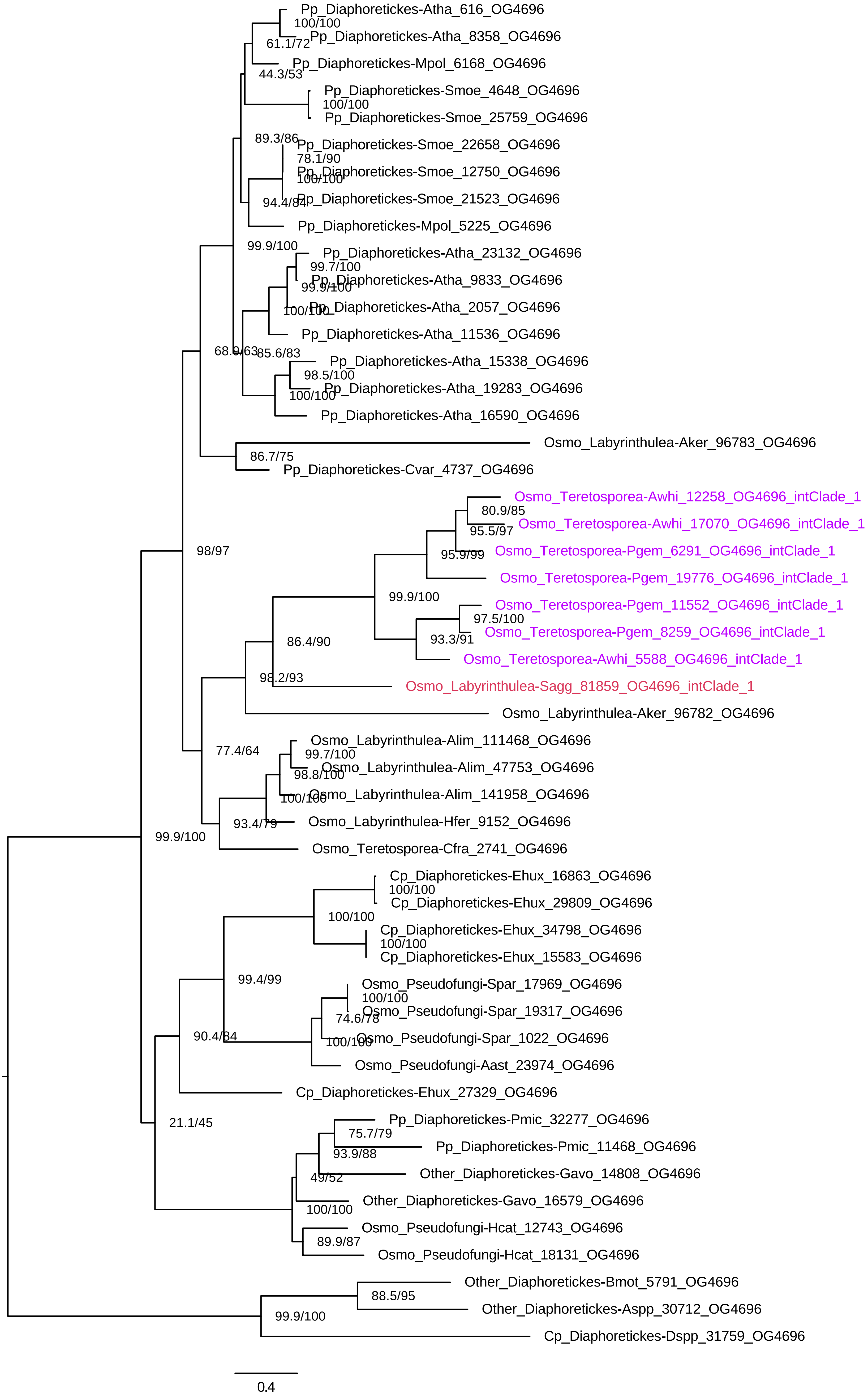

Pp_Diaphoretickes-Atha_616_OG4696
Pp_Diaphoretickes-Atha_8358_OG4696
Pp_Diaphoretickes-Mpol_6168_OG4696
Pp_Diaphoretickes-Smoe_4648_OG4696
Pp_Diaphoretickes-Smoe_25759_OG4696
Pp_Diaphoretickes-Smoe_22658_OG4696
Pp_Diaphoretickes-Smoe_12750_OG4696
Pp_Diaphoretickes-Smoe_21523_OG4696
Pp_Diaphoretickes-Mpol_5225_OG4696
Pp_Diaphoretickes-Atha_23132_OG4696
Pp_Diaphoretickes-Atha_9833_OG4696
Pp_Diaphoretickes-Atha_2057_OG4696
Pp_Diaphoretickes-Atha_11536_OG4696
Pp_Diaphoretickes-Atha_15338_OG4696
Pp_Diaphoretickes-Atha_19283_OG4696
Pp_Diaphoretickes-Atha_16590_OG4696
Osmo_Labyrinthulea-Aker_96783_OG4696
Pp_Diaphoretickes-Cvar_4737_OG4696
Osmo_Teretosporea-Awhi_12258_OG4696_intClade_1
Osmo_Teretosporea-Awhi_17070_OG4696_intClade_1
Osmo_Teretosporea-Pgem_6291_OG4696_intClade_1
Osmo_Teretosporea-Pgem_19776_OG4696_intClade_1
Osmo_Teretosporea-Pgem_11552_OG4696_intClade_1
Osmo_Teretosporea-Pgem_8259_OG4696_intClade_1
Osmo_Teretosporea-Awhi_5588_OG4696_intClade_1
Osmo_Labyrinthulea-Sagg_81859_OG4696_intClade_1
Osmo_Labyrinthulea-Aker_96782_OG4696
Osmo_Labyrinthulea-Alim_111468_OG4696
Osmo_Labyrinthulea-Alim_47753_OG4696
Osmo_Labyrinthulea-Alim_141958_OG4696
Osmo_Labyrinthulea-Hfer_9152_OG4696
Osmo_Teretosporea-Cfra_2741_OG4696
Cp_Diaphoretickes-Ehux_16863_OG4696
Cp_Diaphoretickes-Ehux_29809_OG4696
Cp_Diaphoretickes-Ehux_34798_OG4696
Cp_Diaphoretickes-Ehux_15583_OG4696
Osmo_Pseudofungi-Spar_17969_OG4696
Osmo_Pseudofungi-Spar_19317_OG4696
Osmo_Pseudofungi-Spar_1022_OG4696
Osmo_Pseudofungi-Aast_23974_OG4696
Cp_Diaphoretickes-Ehux_27329_OG4696
Pp_Diaphoretickes-Pmic_32277_OG4696
Pp_Diaphoretickes-Pmic_11468_OG4696
Other_Diaphoretickes-Gavo_14808_OG4696
Other_Diaphoretickes-Gavo_16579_OG4696
Osmo_Pseudofungi-Hcat_12743_OG4696
Osmo_Pseudofungi-Hcat_18131_OG4696
Other_Diaphoretickes-Bmot_5791_OG4696
Other_Diaphoretickes-Aspp_30712_OG4696
Cp_Diaphoretickes-Dspp_31759_OG4696
100/100
61.1/72
44.3/53
100/100
89.3/86
78.1/90
100/100
94.4/84
99.9/100
99.7/100
99.9/100
100/100
68.9/63
85.6/83
98.5/100
100/100
86.7/75
80.9/85
95.5/97
95.9/99
98/97
99.9/100
97.5/100
93.3/91
86.4/90
98.2/93
77.4/64
99.7/100
98.8/100
100/100
93.4/79
99.9/100
100/100
100/100
100/100
99.4/99
100/100
74.6/78
100/100
90.4/84
21.1/45
75.7/79
93.9/88
49/52
100/100
89.9/87
88.5/95
99.9/100
0.4

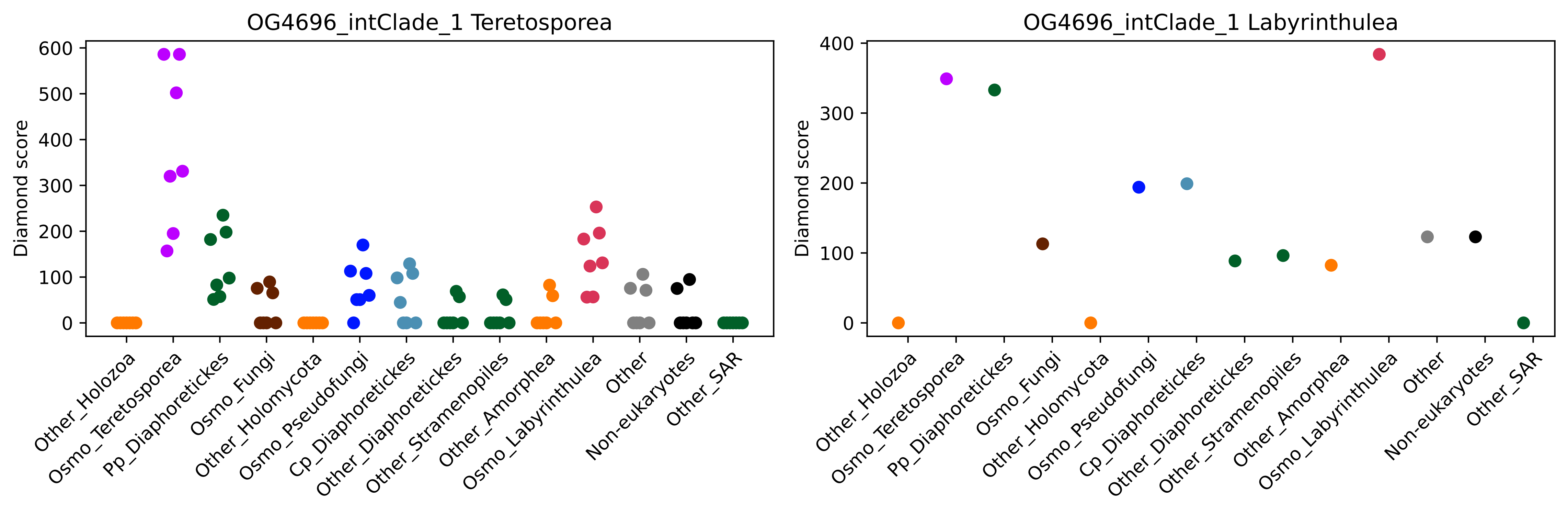
OG4696_intClade_1 Teretosporea
OG4696_intClade_1 Labyrinthulea
Diamond score
Other_Holozoa
Osmo_Teretosporea
Pp_Diaphoretickes
Osmo_Fungi
Other_Holomycota
Osmo_Pseudofungi
Cp_Diaphoretickes
Other_Diaphoretickes
Other_Stramenopiles
Other_Amorphea
Osmo_Labyrinthulea
Other
Non-eukaryotes
Other_SAR

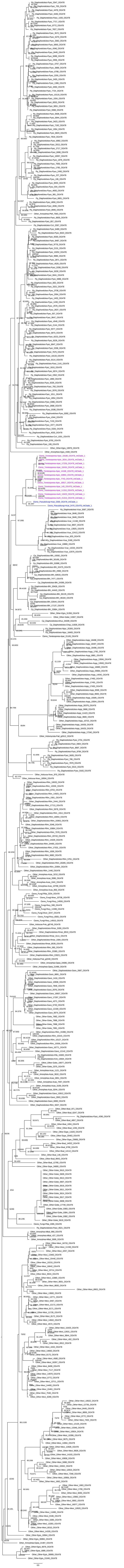

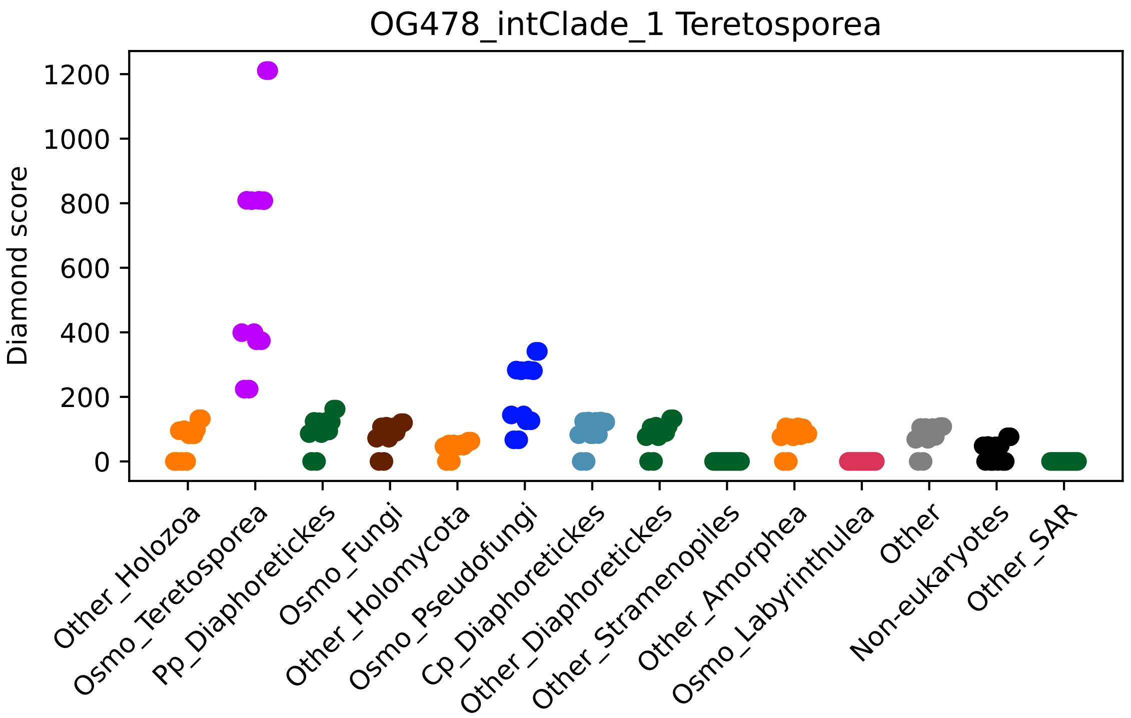

OG478_intClade_1 Teretosporea
Diamond score
0
200
400
600
800
1000
1200
Other_Holozoa
Osmo_Teretosporea
Pp_Diaphoretickes
Osmo_Fungi
Other_Holomycota
Osmo_Pseudofungi
Cp_Diaphoretickes
Other_Diaphoretickes
Other_Stramenopiles
Other_Amorphea
Osmo_Labyrinthulea
Other
Non-eukaryotes
Other_SAR

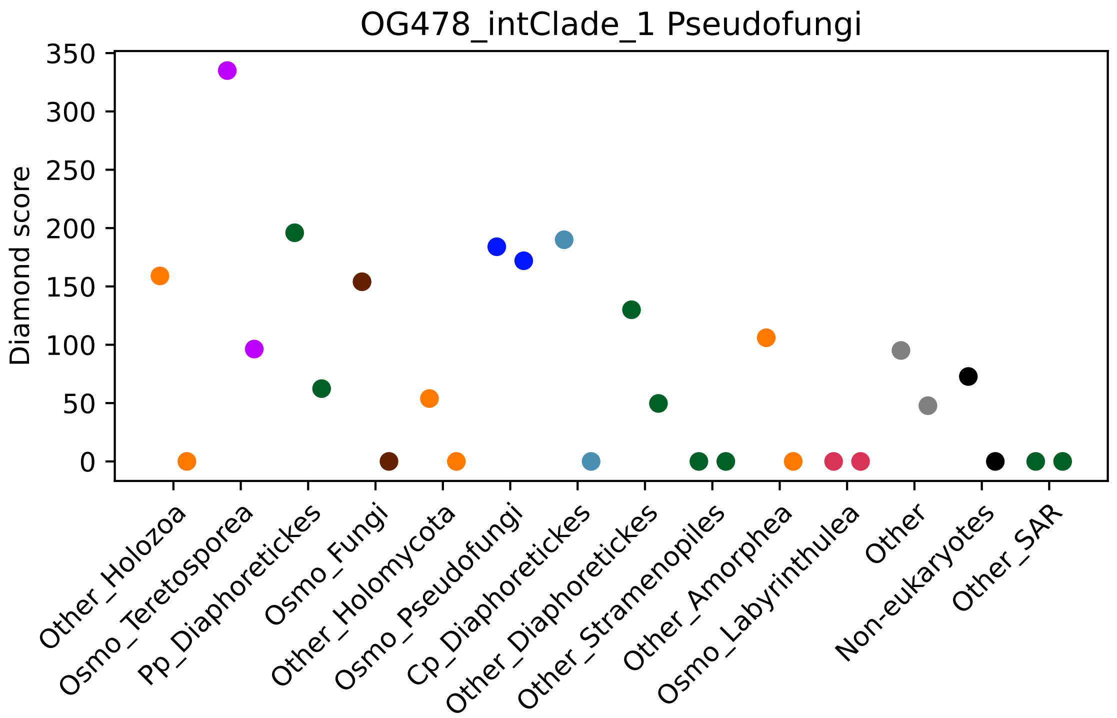

OG478_intClade_1 Pseudofungi
Diamond score
0
50
100
150
200
250
300
350
Other_Holozoa
Osmo_Teretosporea
Pp_Diaphoretickes
Osmo_Fungi
Other_Holomycota
Osmo_Pseudofungi
Cp_Diaphoretickes
Other_Diaphoretickes
Other_Stramenopiles
Other_Amorphea
Osmo_Labyrinthulea
Other
Non-eukaryotes
Other_SAR

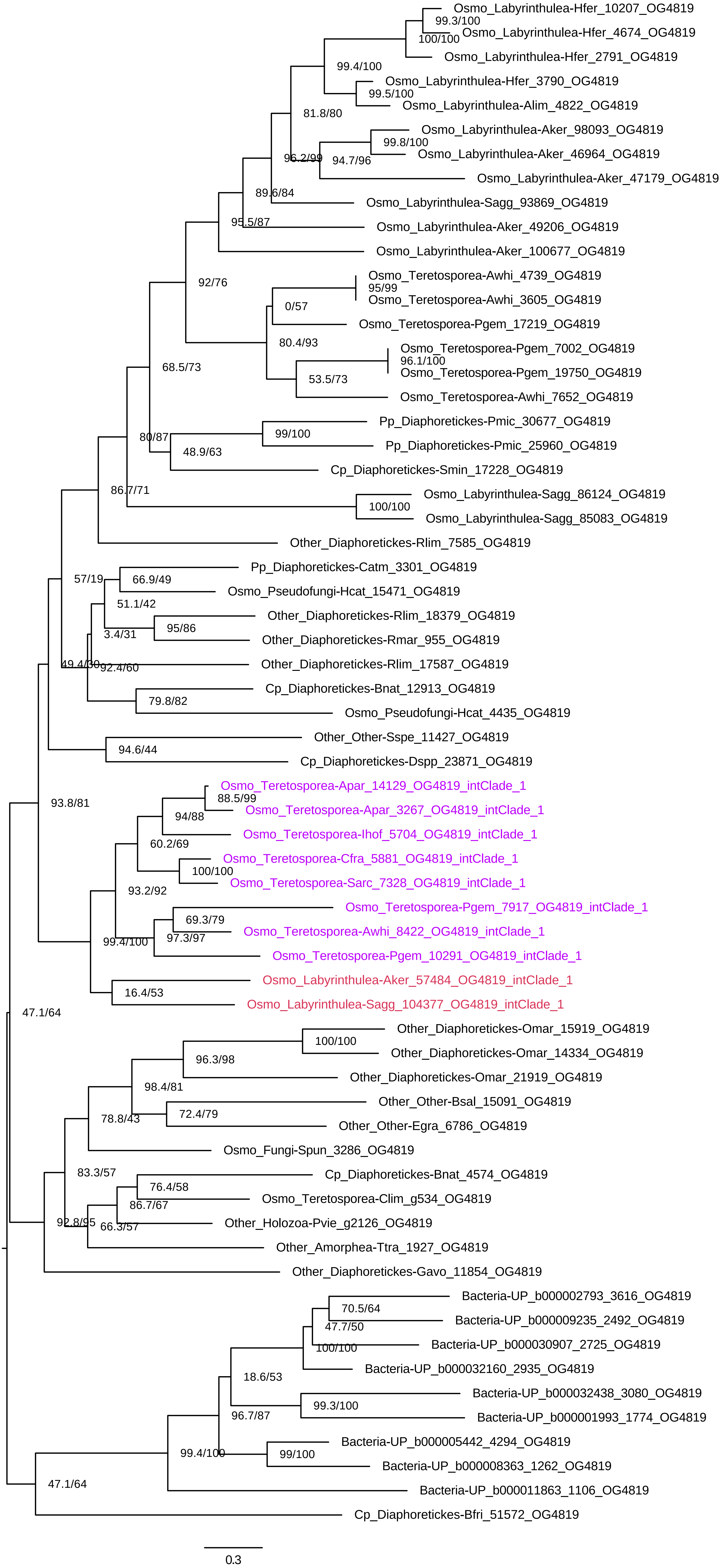

Osmo_Labyrinthulea-Hfer_10207_OG4819
99.3/100
100/100
Osmo_Labyrinthulea-Hfer_4674_OG4819
Osmo_Labyrinthulea-Hfer_2791_OG4819
99.4/100
Osmo_Labyrinthulea-Hfer_3790_OG4819
99.5/100
Osmo_Labyrinthulea-Alim_4822_OG4819
81.8/80
Osmo_Labyrinthulea-Aker_98093_OG4819
99.8/100
96.2/99
94.7/96
Osmo_Labyrinthulea-Aker_46964_OG4819
Osmo_Labyrinthulea-Aker_47179_OG4819
89.6/84
Osmo_Labyrinthulea-Sagg_93869_OG4819
95.5/87
Osmo_Labyrinthulea-Aker_49206_OG4819
Osmo_Labyrinthulea-Aker_100677_OG4819
Osmo_Teretosporea-Awhi_4739_OG4819
92/76
95/99
Osmo_Teretosporea-Awhi_3605_OG4819
0/57
Osmo_Teretosporea-Pgem_17219_OG4819
80.4/93
Osmo_Teretosporea-Pgem_7002_OG4819
96.1/100
68.5/73
Osmo_Teretosporea-Pgem_19750_OG4819
53.5/73
Osmo_Teretosporea-Awhi_7652_OG4819
Pp_Diaphoretickes-Pmic_30677_OG4819
80/87
99/100
Pp_Diaphoretickes-Pmic_25960_OG4819
48.9/63
Cp_Diaphoretickes-Smin_17228_OG4819
86.7/71
Osmo_Labyrinthulea-Sagg_86124_OG4819
100/100
Osmo_Labyrinthulea-Sagg_85083_OG4819
Other_Diaphoretickes-Rlim_7585_OG4819
Pp_Diaphoretickes-Catm_3301_OG4819
57/19
66.9/49
Osmo_Pseudofungi-Hcat_15471_OG4819
51.1/42
Other_Diaphoretickes-Rlim_18379_OG4819
95/86
3.4/31
Other_Diaphoretickes-Rmar_955_OG4819
49.4/30
92.4/60
Other_Diaphoretickes-Rlim_17587_OG4819
Cp_Diaphoretickes-Bnat_12913_OG4819
79.8/82
Osmo_Pseudofungi-Hcat_4435_OG4819
Other_Other-Sspe_11427_OG4819
94.6/44
Cp_Diaphoretickes-Dspp_23871_OG4819
Osmo_Teretosporea-Apar_14129_OG4819_intClade_1
88.5/99
93.8/81
94/88
Osmo_Teretosporea-Apar_3267_OG4819_intClade_1
Osmo_Teretosporea-Ihof_5704_OG4819_intClade_1
60.2/69
Osmo_Teretosporea-Cfra_5881_OG4819_intClade_1
100/100
Osmo_Teretosporea-Sarc_7328_OG4819_intClade_1
93.2/92
Osmo_Teretosporea-Pgem_7917_OG4819_intClade_1
69.3/79
Osmo_Teretosporea-Awhi_8422_OG4819_intClade_1
99.4/100
97.3/97
Osmo_Teretosporea-Pgem_10291_OG4819_intClade_1
Osmo_Labyrinthulea-Aker_57484_OG4819_intClade_1
16.4/53
Osmo_Labyrinthulea-Sagg_104377_OG4819_intClade_1
47.1/64
Other_Diaphoretickes-Omar_15919_OG4819
100/100
Other_Diaphoretickes-Omar_14334_OG4819
96.3/98
Other_Diaphoretickes-Omar_21919_OG4819
98.4/81
Other_Other-Bsal_15091_OG4819
72.4/79
78.8/43
Other_Other-Egra_6786_OG4819
Osmo_Fungi-Spun_3286_OG4819
83.3/57
Cp_Diaphoretickes-Bnat_4574_OG4819
76.4/58
Osmo_Teretosporea-Clim_g534_OG4819
86.7/67
92.8/95
66.3/57
Other_Holozoa-Pvie_g2126_OG4819
Other_Amorphea-Ttra_1927_OG4819
Other_Diaphoretickes-Gavo_11854_OG4819
Bacteria-UP_b000002793_3616_OG4819
70.5/64
Bacteria-UP_b000009235_2492_OG4819
47.7/50
100/100
Bacteria-UP_b000030907_2725_OG4819
Bacteria-UP_b000032160_2935_OG4819
18.6/53
Bacteria-UP_b000032438_3080_OG4819
99.3/100
96.7/87
Bacteria-UP_b000001993_1774_OG4819
Bacteria-UP_b000005442_4294_OG4819
99.4/100
99/100
Bacteria-UP_b000008363_1262_OG4819
47.1/64
Bacteria-UP_b000011863_1106_OG4819
Cp_Diaphoretickes-Bfri_51572_OG4819
0.3

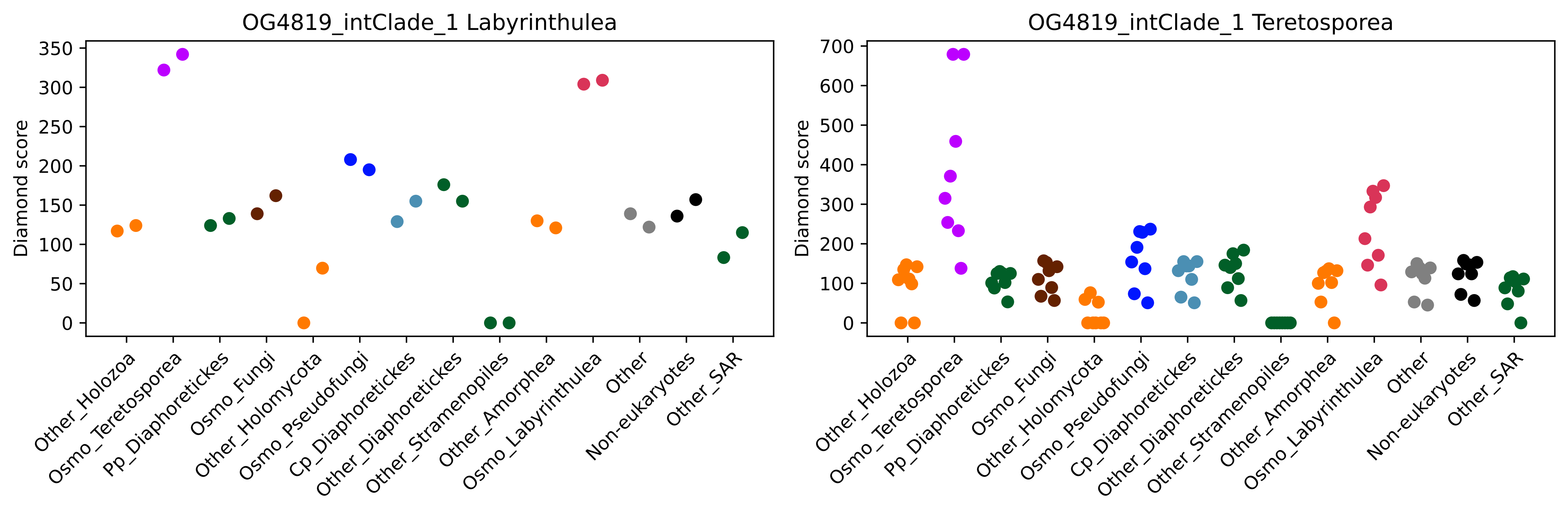
OG4819_intClade_1 Labyrinthulea
OG4819_intClade_1 Teretosporea
Diamond score
0
50
100
150
200
250
300
350
400
500
600
700
Other_Holozoa
Osmo_Teretosporea
Pp_Diaphoretickes
Osmo_Fungi
Other_Holomycota
Osmo_Pseudofungi
Cp_Diaphoretickes
Other_Diaphoretickes
Other_Stramenopiles
Other_Amorphea
Osmo_Labyrinthulea
Other
Non-eukaryotes
Other_SAR

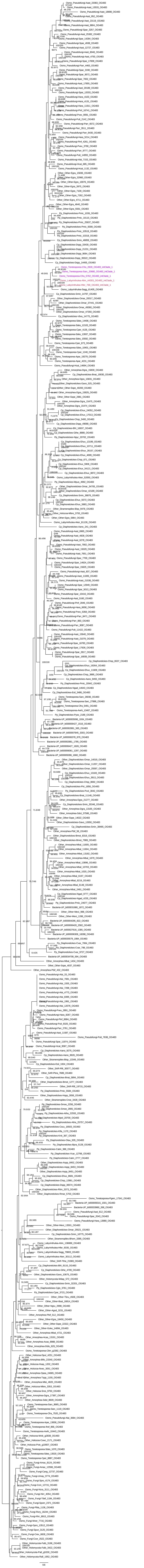

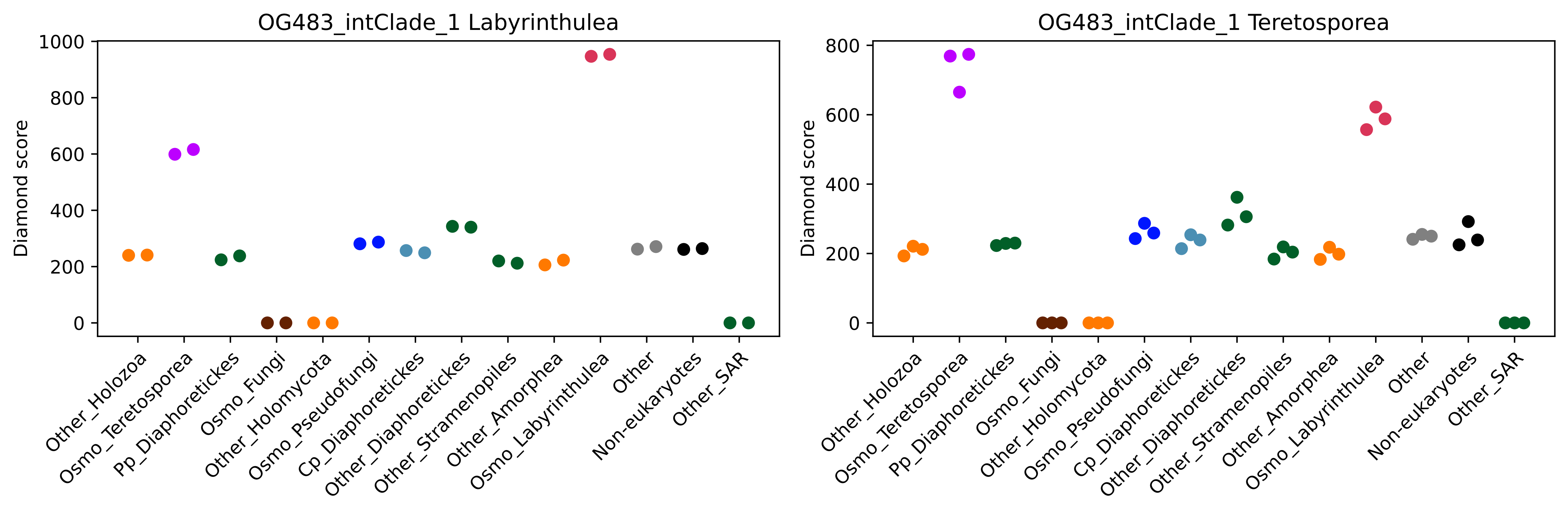
OG483_intClade_1 Labyrinthulea
Diamond score
1000
800
600
400
200
0
Other_Holozoa
Osmo_Teretosporea
Pp_Diaphoretickes
Osmo_Fungi
Other_Holomycota
Osmo_Pseudofungi
Cp_Diaphoretickes
Other_Diaphoretickes
Other_Stramenopiles
Other_Amorphea
Osmo_Labyrinthulea
Other
Non-eukaryotes
Other_SAR
OG483_intClade_1 Teretosporea
Diamond score
800
600
400
200
0
Other_Holozoa
Osmo_Teretosporea
Pp_Diaphoretickes
Osmo_Fungi
Other_Holomycota
Osmo_Pseudofungi
Cp_Diaphoretickes
Other_Diaphoretickes
Other_Stramenopiles
Other_Amorphea
Osmo_Labyrinthulea
Other
Non-eukaryotes
Other_SAR

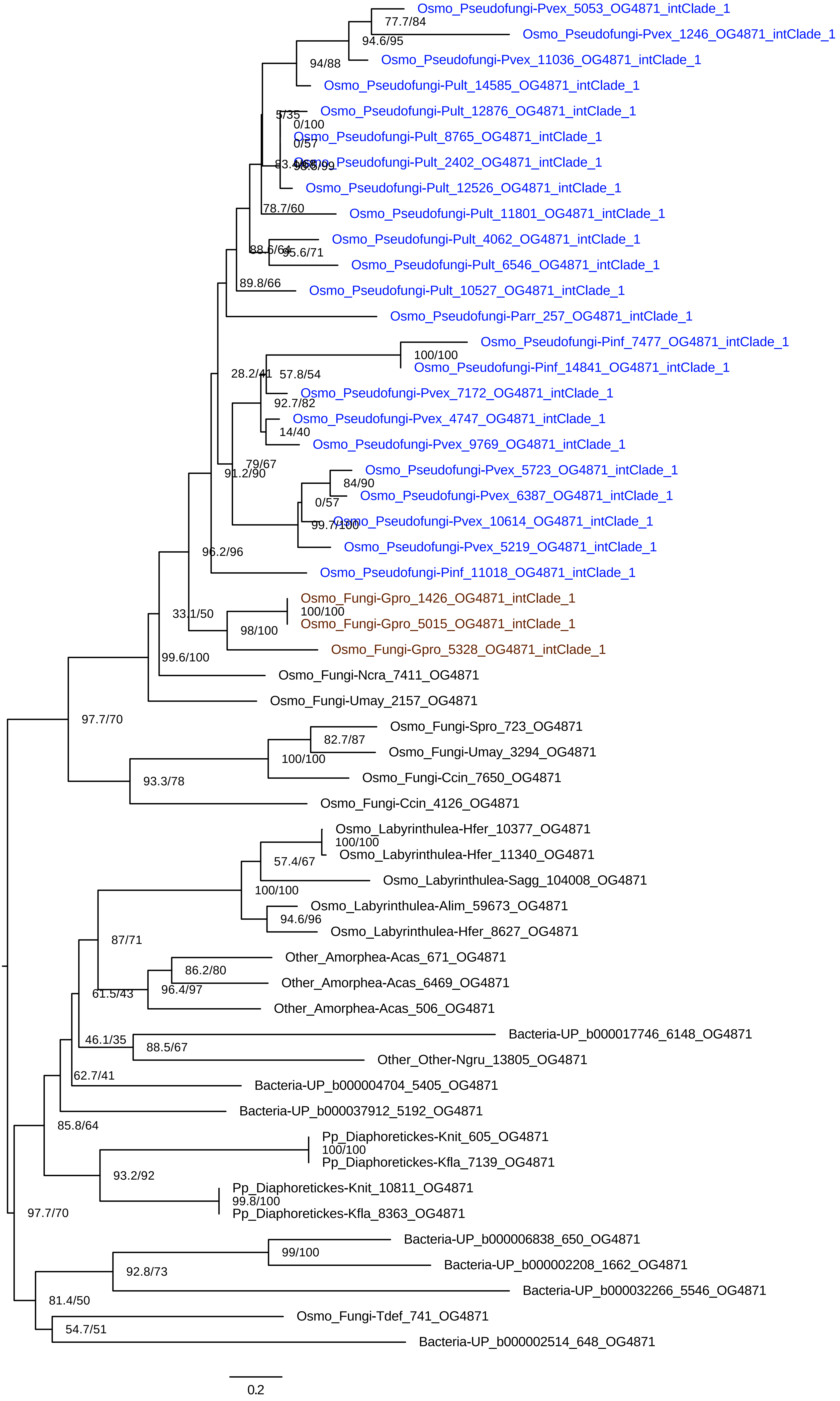

Osmo_Pseudofungi-Pvex_5053_OG4871_intClade_1
77.7/84
Osmo_Pseudofungi-Pvex_1246_OG4871_intClade_1
94.6/95
Osmo_Pseudofungi-Pvex_11036_OG4871_intClade_1
94/88
Osmo_Pseudofungi-Pult_14585_OG4871_intClade_1
5/35
Osmo_Pseudofungi-Pult_12876_OG4871_intClade_1
0/100
Osmo_Pseudofungi-Pult_8765_OG4871_intClade_1
0/57
Osmo_Pseudofungi-Pult_2402_OG4871_intClade_1
98.3/99
Osmo_Pseudofungi-Pult_12526_OG4871_intClade_1
78.7/60
Osmo_Pseudofungi-Pult_11801_OG4871_intClade_1
Osmo_Pseudofungi-Pult_4062_OG4871_intClade_1
95.6/71
Osmo_Pseudofungi-Pult_6546_OG4871_intClade_1
89.8/66
Osmo_Pseudofungi-Pult_10527_OG4871_intClade_1
Osmo_Pseudofungi-Parr_257_OG4871_intClade_1
Osmo_Pseudofungi-Pinf_7477_OG4871_intClade_1
100/100
Osmo_Pseudofungi-Pinf_14841_OG4871_intClade_1
28.2/41
57.8/54
Osmo_Pseudofungi-Pvex_7172_OG4871_intClade_1
92.7/82
Osmo_Pseudofungi-Pvex_4747_OG4871_intClade_1
14/40
Osmo_Pseudofungi-Pvex_9769_OG4871_intClade_1
79/67
91.2/90
Osmo_Pseudofungi-Pvex_5723_OG4871_intClade_1
84/90
Osmo_Pseudofungi-Pvex_6387_OG4871_intClade_1
0/57
Osmo_Pseudofungi-Pvex_10614_OG4871_intClade_1
99.7/100
Osmo_Pseudofungi-Pvex_5219_OG4871_intClade_1
96.2/96
Osmo_Pseudofungi-Pinf_11018_OG4871_intClade_1
Osmo_Fungi-Gpro_1426_OG4871_intClade_1
100/100
33.1/50
Osmo_Fungi-Gpro_5015_OG4871_intClade_1
98/100
Osmo_Fungi-Gpro_5328_OG4871_intClade_1
99.6/100
Osmo_Fungi-Ncra_7411_OG4871
Osmo_Fungi-Umay_2157_OG4871
97.7/70
Osmo_Fungi-Spro_723_OG4871
82.7/87
Osmo_Fungi-Umay_3294_OG4871
100/100
Osmo_Fungi-Ccin_7650_OG4871
93.3/78
Osmo_Fungi-Ccin_4126_OG4871
Osmo_Labyrinthulea-Hfer_10377_OG4871
100/100
Osmo_Labyrinthulea-Hfer_11340_OG4871
57.4/67
Osmo_Labyrinthulea-Sagg_104008_OG4871
100/100
Osmo_Labyrinthulea-Alim_59673_OG4871
94.6/96
Osmo_Labyrinthulea-Hfer_8627_OG4871
87/71
Other_Amorphea-Acas_671_OG4871
86.2/80
Other_Amorphea-Acas_6469_OG4871
96.4/97
61.5/43
Other_Amorphea-Acas_506_OG4871
Bacteria-UP_b000017746_6148_OG4871
46.1/35
88.5/67
Other_Other-Ngru_13805_OG4871
62.7/41
Bacteria-UP_b000004704_5405_OG4871
Bacteria-UP_b000037912_5192_OG4871
85.8/64
Pp_Diaphoretickes-Knit_605_OG4871
100/100
Pp_Diaphoretickes-Kfla_7139_OG4871
93.2/92
Pp_Diaphoretickes-Knit_10811_OG4871
99.8/100
97.7/70
Pp_Diaphoretickes-Kfla_8363_OG4871
Bacteria-UP_b000006838_650_OG4871
99/100
Bacteria-UP_b000002208_1662_OG4871
92.8/73
Bacteria-UP_b000032266_5546_OG4871
81.4/50
Osmo_Fungi-Tdef_741_OG4871
54.7/51
Bacteria-UP_b000002514_648_OG4871
0.2

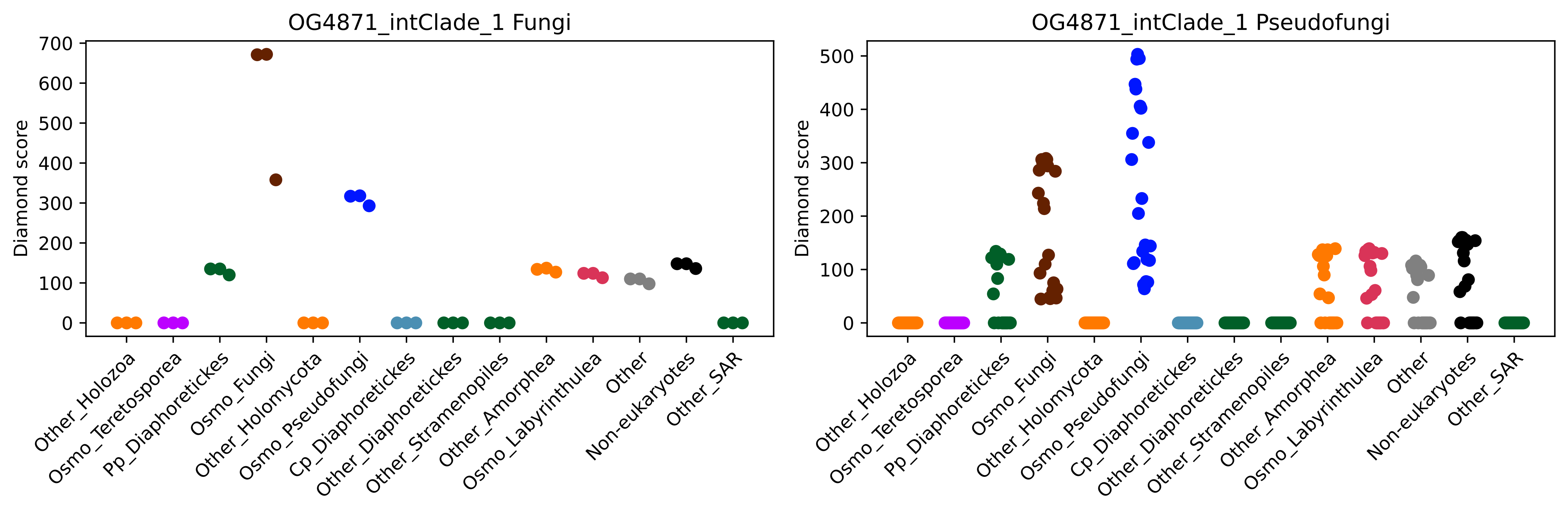
OG4871_intClade_1 Fungi
OG4871_intClade_1 Pseudofungi
Diamond score
0
100
200
300
400
500
600
700
Other_Holozoa
Osmo_Teretosporea
Pp_Diaphoretickes
Osmo_Fungi
Other_Holomycota
Osmo_Pseudofungi
Cp_Diaphoretickes
Other_Diaphoretickes
Other_Stramenopiles
Other_Amorphea
Osmo_Labyrinthulea
Other
Non-eukaryotes
Other_SAR

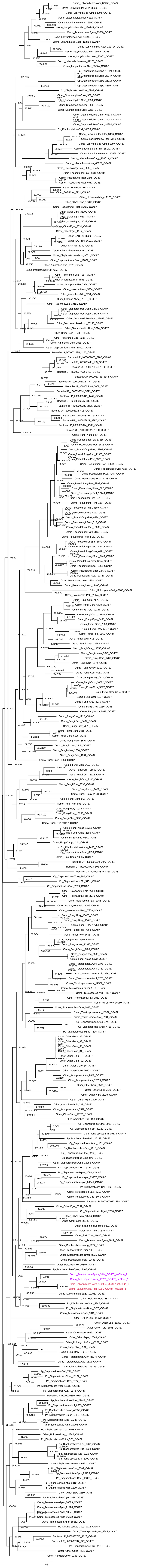

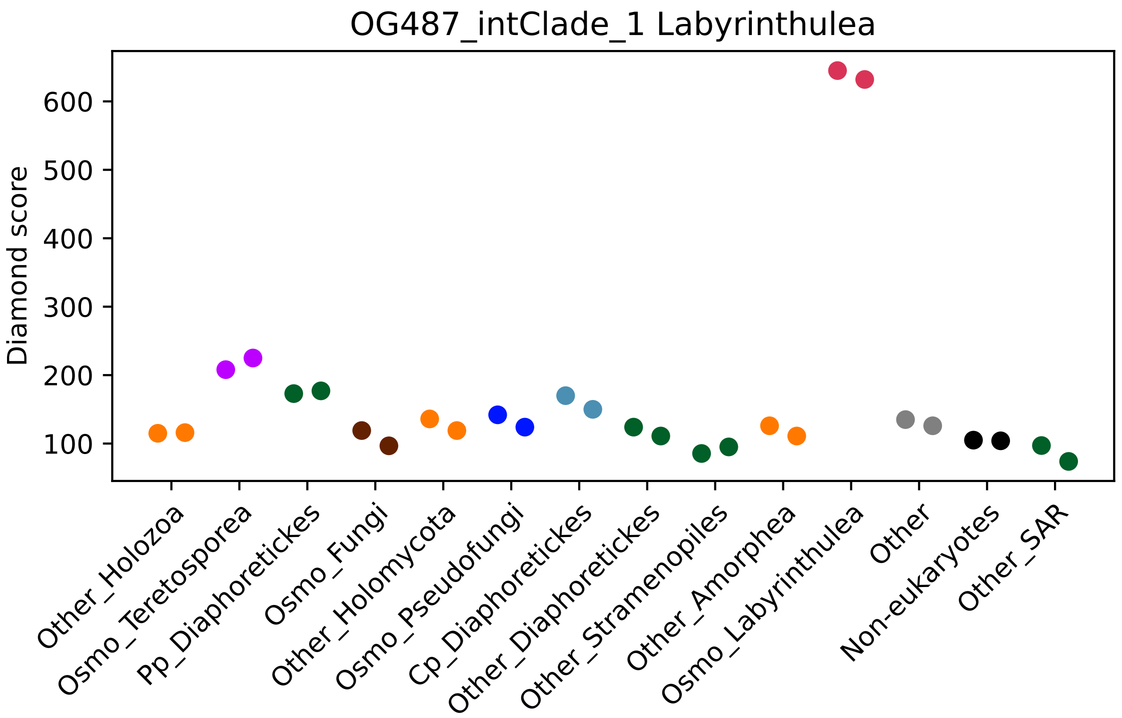
OG487_intClade_1 Labyrinthulea
Diamond score
100
200
300
400
500
600
Other_Holozoa
Osmo_Teretosporea
Pp_Diaphoretickes
Osmo_Fungi
Other_Holomycota
Osmo_Pseudofungi
Cp_Diaphoretickes
Other_Diaphoretickes
Other_Stramenopiles
Other_Amorphea
Osmo_Labyrinthulea
Other
Non-eukaryotes
Other_SAR

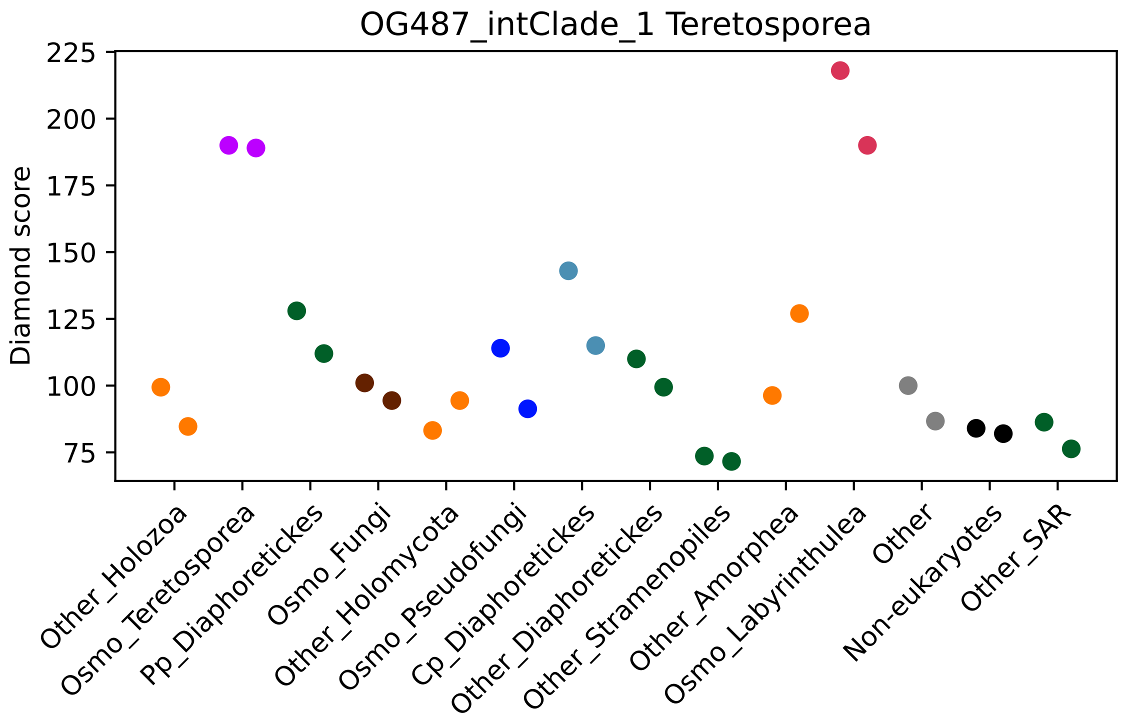
OG487_intClade_1 Teretosporea
Diamond score
75
100
125
150
175
200
225
Other_Holozoa
Osmo_Teretosporea
Pp_Diaphoretickes
Osmo_Fungi
Other_Holomycota
Osmo_Pseudofungi
Cp_Diaphoretickes
Other_Diaphoretickes
Other_Stramenopiles
Other_Amorphea
Osmo_Labyrinthulea
Other
Non-eukaryotes
Other_SAR

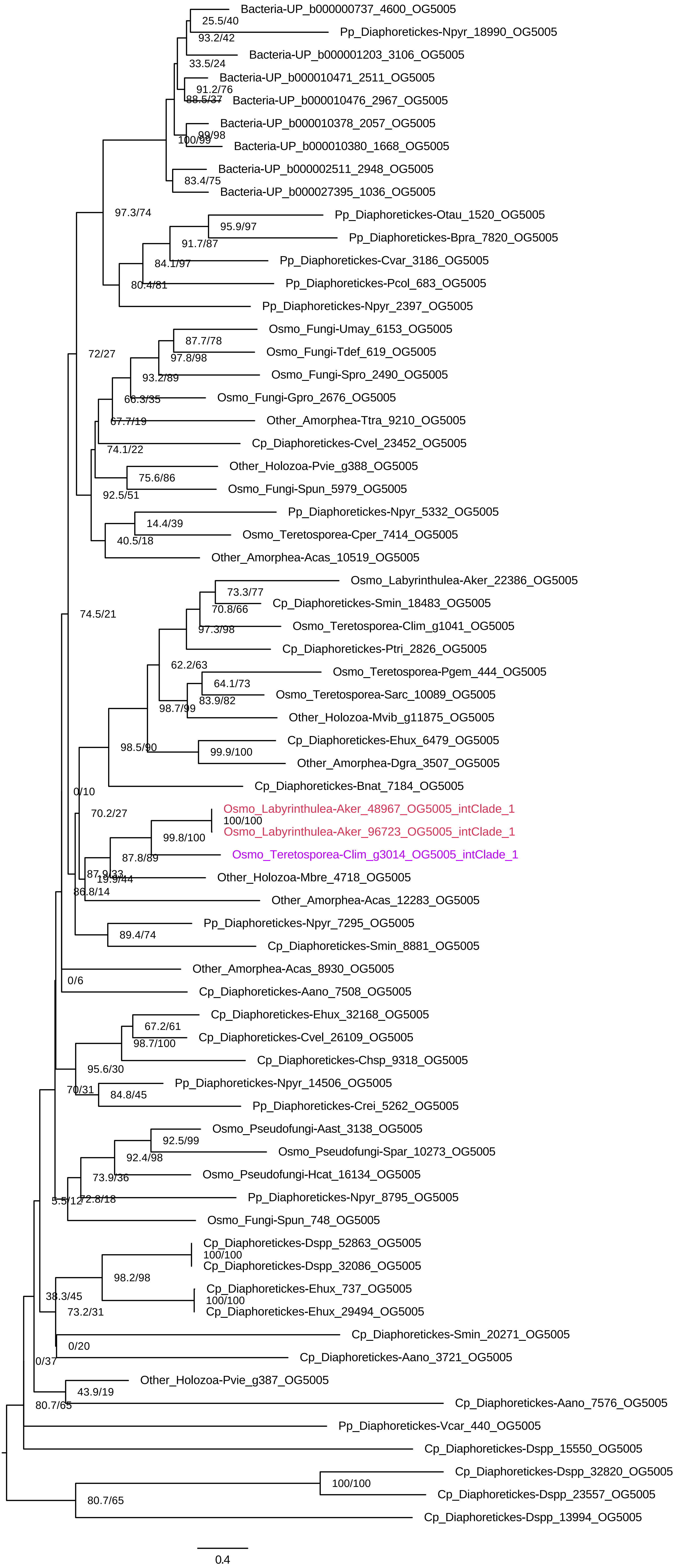

Bacteria-UP_b000000737_4600_OG5005
25.5/40
Pp_Diaphoretickes-Npyr_18990_OG5005
93.2/42
Bacteria-UP_b000001203_3106_OG5005
33.5/24
Bacteria-UP_b000010471_2511_OG5005
91.2/76
88.5/37
Bacteria-UP_b000010476_2967_OG5005
Bacteria-UP_b000010378_2057_OG5005
99/98
100/99
Bacteria-UP_b000010380_1668_OG5005
Bacteria-UP_b000002511_2948_OG5005
83.4/75
Bacteria-UP_b000027395_1036_OG5005
97.3/74
Pp_Diaphoretickes-Otau_1520_OG5005
95.9/97
Pp_Diaphoretickes-Bpra_7820_OG5005
91.7/87
Pp_Diaphoretickes-Cvar_3186_OG5005
84.1/97
Pp_Diaphoretickes-Pcol_683_OG5005
80.4/81
Pp_Diaphoretickes-Npyr_2397_OG5005
Osmo_Fungi-Umay_6153_OG5005
87.7/78
72/27
Osmo_Fungi-Tdef_619_OG5005
97.8/98
Osmo_Fungi-Spro_2490_OG5005
93.2/89
Osmo_Fungi-Gpro_2676_OG5005
66.3/35
Other_Amorphea-Ttra_9210_OG5005
67.7/19
Cp_Diaphoretickes-Cvel_23452_OG5005
74.1/22
Other_Holozoa-Pvie_g388_OG5005
75.6/86
Osmo_Fungi-Spun_5979_OG5005
92.5/51
Pp_Diaphoretickes-Npyr_5332_OG5005
14.4/39
Osmo_Teretosporea-Cper_7414_OG5005
40.5/18
Other_Amorphea-Acas_10519_OG5005
Osmo_Labyrinthulea-Aker_22386_OG5005
73.3/77
Cp_Diaphoretickes-Smin_18483_OG5005
70.8/66
74.5/21
Osmo_Teretosporea-Clim_g1041_OG5005
97.3/98
Cp_Diaphoretickes-Ptri_2826_OG5005
62.2/63
Osmo_Teretosporea-Pgem_444_OG5005
64.1/73
Osmo_Teretosporea-Sarc_10089_OG5005
83.9/82
98.7/99
Other_Holozoa-Mvib_g11875_OG5005
Cp_Diaphoretickes-Ehux_6479_OG5005
98.5/90
99.9/100
Other_Amorphea-Dgra_3507_OG5005
Cp_Diaphoretickes-Bnat_7184_OG5005
0/10
Osmo_Labyrinthulea-Aker_48967_OG5005_intClade_1
70.2/27
100/100
Osmo_Labyrinthulea-Aker_96723_OG5005_intClade_1
99.8/100
Osmo_Teretosporea-Clim_g3014_OG5005_intClade_1
87.8/89
87.9/33
19.9/44
Other_Holozoa-Mbre_4718_OG5005
86.8/14
Other_Amorphea-Acas_12283_OG5005
Pp_Diaphoretickes-Npyr_7295_OG5005
89.4/74
Cp_Diaphoretickes-Smin_8881_OG5005
Other_Amorphea-Acas_8930_OG5005
0/6
Cp_Diaphoretickes-Aano_7508_OG5005
Cp_Diaphoretickes-Ehux_32168_OG5005
67.2/61
Cp_Diaphoretickes-Cvel_26109_OG5005
98.7/100
Cp_Diaphoretickes-Chsp_9318_OG5005
95.6/30
Pp_Diaphoretickes-Npyr_14506_OG5005
70/31
84.8/45
Pp_Diaphoretickes-Crei_5262_OG5005
Osmo_Pseudofungi-Aast_3138_OG5005
92.5/99
Osmo_Pseudofungi-Spar_10273_OG5005
92.4/98
Osmo_Pseudofungi-Hcat_16134_OG5005
73.9/36
5.5/12
72.8/18
Pp_Diaphoretickes-Npyr_8795_OG5005
Osmo_Fungi-Spun_748_OG5005
Cp_Diaphoretickes-Dspp_52863_OG5005
100/100
Cp_Diaphoretickes-Dspp_32086_OG5005
98.2/98
Cp_Diaphoretickes-Ehux_737_OG5005
38.3/45
100/100
73.2/31
Cp_Diaphoretickes-Ehux_29494_OG5005
Cp_Diaphoretickes-Smin_20271_OG5005
0/20
0/37
Cp_Diaphoretickes-Aano_3721_OG5005
Other_Holozoa-Pvie_g387_OG5005
43.9/19
80.7/65
Cp_Diaphoretickes-Aano_7576_OG5005
Pp_Diaphoretickes-Vcar_440_OG5005
Cp_Diaphoretickes-Dspp_15550_OG5005
Cp_Diaphoretickes-Dspp_32820_OG5005
100/100
80.7/65
Cp_Diaphoretickes-Dspp_23557_OG5005
Cp_Diaphoretickes-Dspp_13994_OG5005
0.4

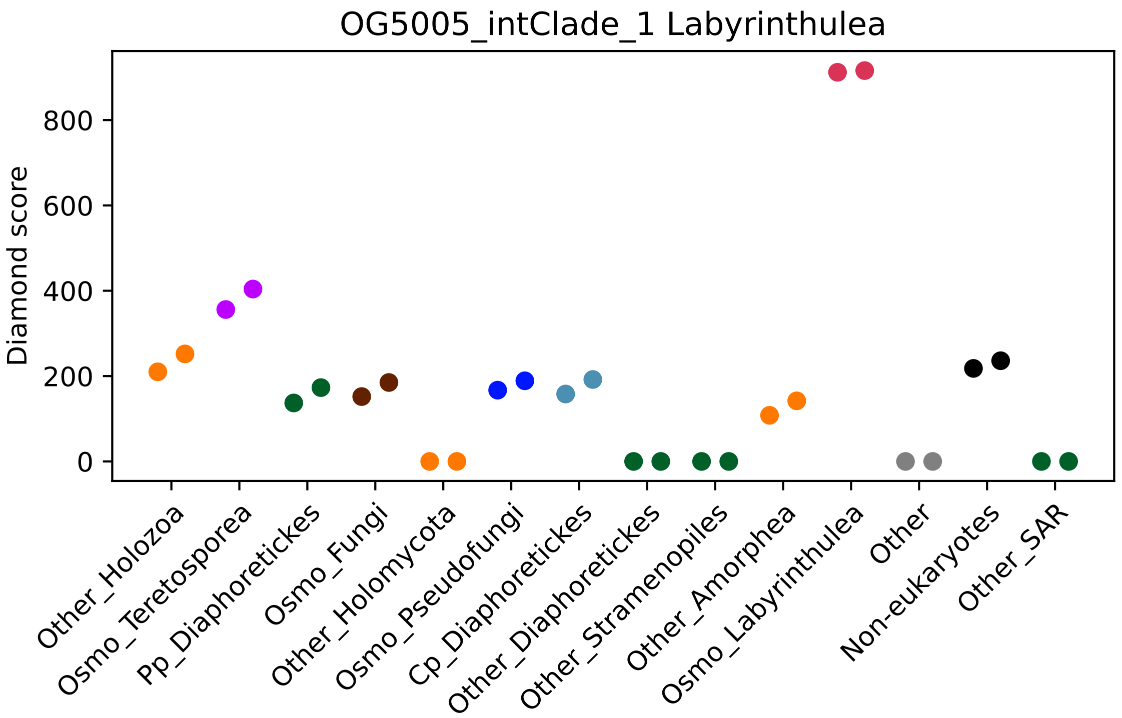

OG5005_intClade_1 Labyrinthulea
Diamond score
0
200
400
600
800
Other_Holozoa
Osmo_Teretosporea
Pp_Diaphoretickes
Osmo_Fungi
Other_Holomycota
Osmo_Pseudofungi
Cp_Diaphoretickes
Other_Diaphoretickes
Other_Stramenopiles
Other_Amorphea
Osmo_Labyrinthulea
Other
Non-eukaryotes
Other_SAR

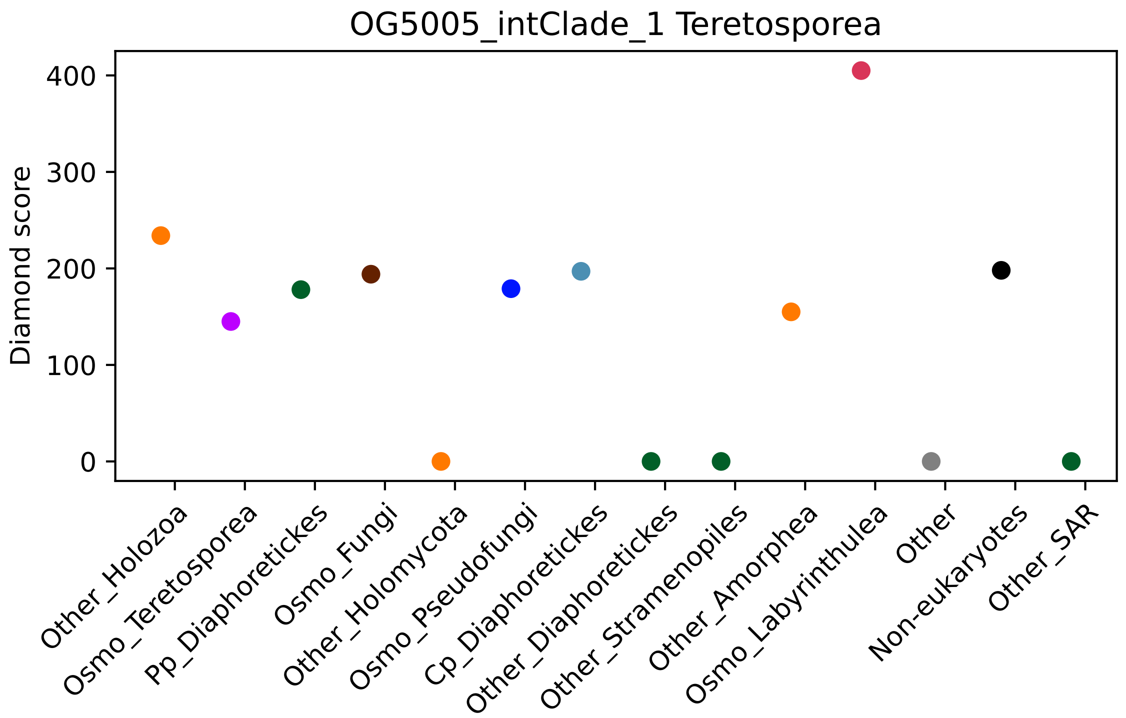

OG5005_intClade_1 Teretosporea
Diamond score
0
100
200
300
400
Other_Holozoa
Osmo_Teretosporea
Pp_Diaphoretickes
Osmo_Fungi
Other_Holomycota
Osmo_Pseudofungi
Cp_Diaphoretickes
Other_Diaphoretickes
Other_Stramenopiles
Other_Amorphea
Osmo_Labyrinthulea
Other
Non-eukaryotes
Other_SAR

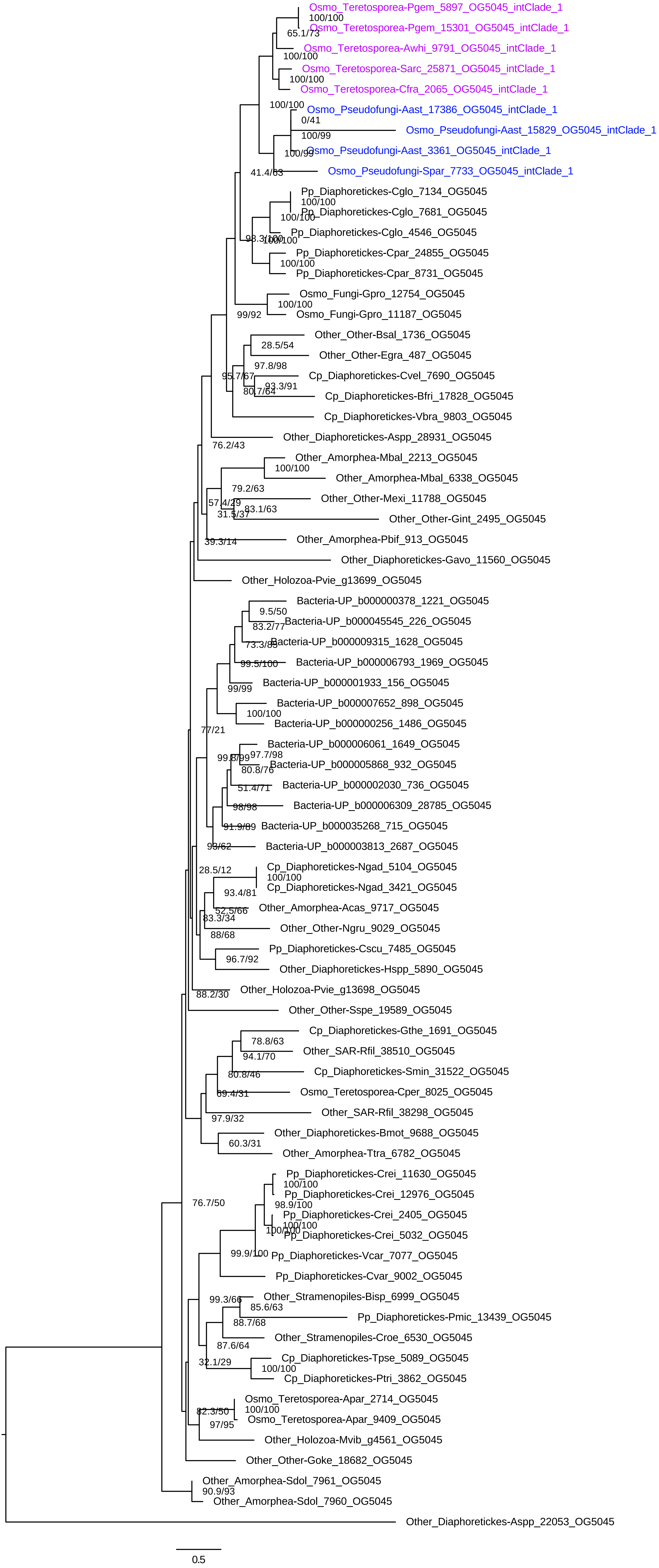
Osmo_Teretosporea-Pgem_5897_OG5045_intClade_1
100/100
65.1/73
Osmo_Teretosporea-Pgem_15301_OG5045_intClade_1
Osmo_Teretosporea-Awhi_9791_OG5045_intClade_1
100/100
Osmo_Teretosporea-Sarc_25871_OG5045_intClade_1
100/100
Osmo_Teretosporea-Cfra_2065_OG5045_intClade_1
100/100
Osmo_Pseudofungi-Aast_17386_OG5045_intClade_1
0/41
Osmo_Pseudofungi-Aast_15829_OG5045_intClade_1
100/99
Osmo_Pseudofungi-Aast_3361_OG5045_intClade_1
100/99
41.4/63
Osmo_Pseudofungi-Spar_7733_OG5045_intClade_1
Pp_Diaphoretickes-Cglo_7134_OG5045
100/100
Pp_Diaphoretickes-Cglo_7681_OG5045
100/100
Pp_Diaphoretickes-Cglo_4546_OG5045
98.3/100
100/100
Pp_Diaphoretickes-Cpar_24855_OG5045
100/100
Pp_Diaphoretickes-Cpar_8731_OG5045
Osmo_Fungi-Gpro_12754_OG5045
100/100
99/92
Osmo_Fungi-Gpro_11187_OG5045
Other_Other-Bsal_1736_OG5045
28.5/54
Other_Other-Egra_487_OG5045
97.8/98
Cp_Diaphoretickes-Cvel_7690_OG5045
95.7/67
93.3/91
80.7/64
Cp_Diaphoretickes-Bfri_17828_OG5045
Cp_Diaphoretickes-Vbra_9803_OG5045
Other_Diaphoretickes-Aspp_28931_OG5045
76.2/43
Other_Amorphea-Mbal_2213_OG5045
100/100
Other_Amorphea-Mbal_6338_OG5045
79.2/63
Other_Other-Mexi_11788_OG5045
57.4/29
83.1/63
31.5/37
Other_Other-Gint_2495_OG5045
Other_Amorphea-Pbif_913_OG5045
39.3/14
Other_Diaphoretickes-Gavo_11560_OG5045
Other_Holozoa-Pvie_g13699_OG5045
Bacteria-UP_b000000378_1221_OG5045
9.5/50
Bacteria-UP_b000045545_226_OG5045
83.2/77
Bacteria-UP_b000009315_1628_OG5045
73.3/85
Bacteria-UP_b000006793_1969_OG5045
99.5/100
Bacteria-UP_b000001933_156_OG5045
99/99
Bacteria-UP_b000007652_898_OG5045
100/100
Bacteria-UP_b000000256_1486_OG5045
77/21
Bacteria-UP_b000006061_1649_OG5045
99.8/99
97.7/98
Bacteria-UP_b000005868_932_OG5045
80.8/76
Bacteria-UP_b000002030_736_OG5045
51.4/71
Bacteria-UP_b000006309_28785_OG5045
98/98
Bacteria-UP_b000035268_715_OG5045
91.9/89
Bacteria-UP_b000003813_2687_OG5045
93/62
Cp_Diaphoretickes-Ngad_5104_OG5045
28.5/12
100/100
Cp_Diaphoretickes-Ngad_3421_OG5045
93.4/81
Other_Amorphea-Acas_9717_OG5045
52.5/66
83.3/34
Other_Other-Ngru_9029_OG5045
88/68
Pp_Diaphoretickes-Cscu_7485_OG5045
96.7/92
Other_Diaphoretickes-Hspp_5890_OG5045
Other_Holozoa-Pvie_g13698_OG5045
88.2/30
Other_Other-Sspe_19589_OG5045
Cp_Diaphoretickes-Gthe_1691_OG5045
78.8/63
Other_SAR-Rfil_38510_OG5045
94.1/70
Cp_Diaphoretickes-Smin_31522_OG5045
80.8/46
Osmo_Teretosporea-Cper_8025_OG5045
69.4/31
Other_SAR-Rfil_38298_OG5045
97.9/32
Other_Diaphoretickes-Bmot_9688_OG5045
60.3/31
Other_Amorphea-Ttra_6782_OG5045
Pp_Diaphoretickes-Crei_11630_OG5045
100/100
Pp_Diaphoretickes-Crei_12976_OG5045
76.7/50
98.9/100
Pp_Diaphoretickes-Crei_2405_OG5045
100/100
100/100
Pp_Diaphoretickes-Crei_5032_OG5045
99.9/100
Pp_Diaphoretickes-Vcar_7077_OG5045
Pp_Diaphoretickes-Cvar_9002_OG5045
Other_Stramenopiles-Bisp_6999_OG5045
99.3/66
85.6/63
Pp_Diaphoretickes-Pmic_13439_OG5045
88.7/68
Other_Stramenopiles-Croe_6530_OG5045
87.6/64
Cp_Diaphoretickes-Tpse_5089_OG5045
32.1/29
100/100
Cp_Diaphoretickes-Ptri_3862_OG5045
Osmo_Teretosporea-Apar_2714_OG5045
82.3/50
100/100
Osmo_Teretosporea-Apar_9409_OG5045
97/95
Other_Holozoa-Mvib_g4561_OG5045
Other_Other-Goke_18682_OG5045
Other_Amorphea-Sdol_7961_OG5045
90.9/93
Other_Amorphea-Sdol_7960_OG5045
Other_Diaphoretickes-Aspp_22053_OG5045
0.5

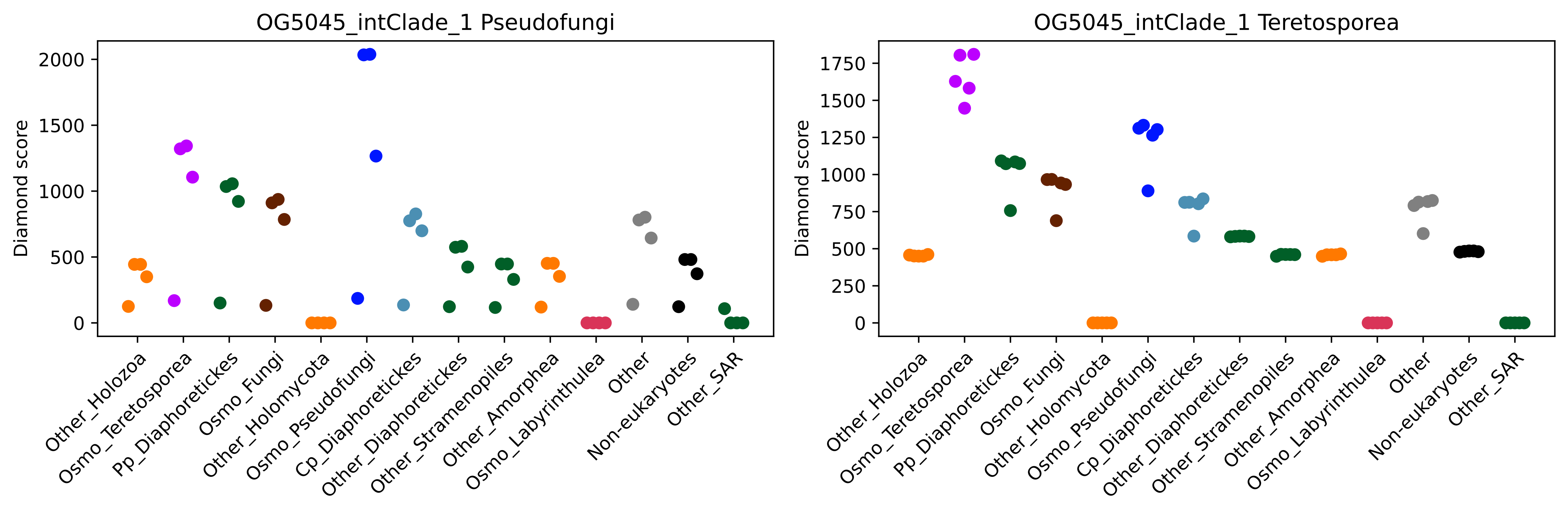
OG5045_intClade_1 Pseudofungi
OG5045_intClade_1 Teretosporea
Diamond score
Other_Holozoa
Osmo_Teretosporea
Pp_Diaphoretickes
Osmo_Fungi
Other_Holomycota
Osmo_Pseudofungi
Cp_Diaphoretickes
Other_Diaphoretickes
Other_Stramenopiles
Other_Amorphea
Osmo_Labyrinthulea
Other
Non-eukaryotes
Other_SAR

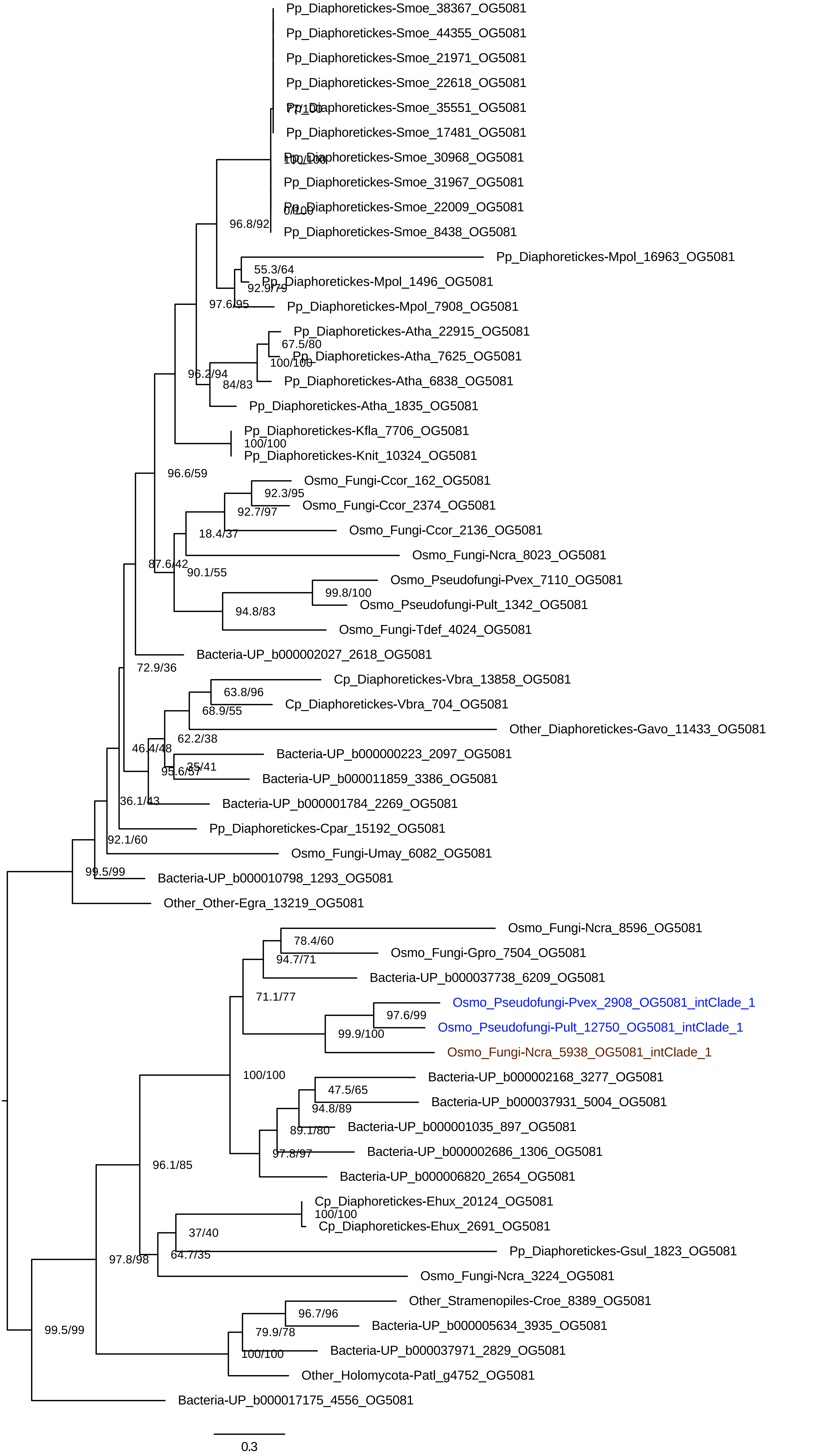
Pp_Diaphoretickes-Smoe_38367_OG5081
Pp_Diaphoretickes-Smoe_44355_OG5081
Pp_Diaphoretickes-Smoe_21971_OG5081
Pp_Diaphoretickes-Smoe_22618_OG5081
Pp_Diaphoretickes-Smoe_35551_OG5081
77/100
Pp_Diaphoretickes-Smoe_17481_OG5081
Pp_Diaphoretickes-Smoe_30968_OG5081
100/100
Pp_Diaphoretickes-Smoe_31967_OG5081
Pp_Diaphoretickes-Smoe_22009_OG5081
0/100
96.8/92
Pp_Diaphoretickes-Smoe_8438_OG5081
Pp_Diaphoretickes-Mpol_16963_OG5081
55.3/64
Pp_Diaphoretickes-Mpol_1496_OG5081
92.9/79
97.6/95
Pp_Diaphoretickes-Mpol_7908_OG5081
Pp_Diaphoretickes-Atha_22915_OG5081
67.5/80
Pp_Diaphoretickes-Atha_7625_OG5081
100/100
96.2/94
Pp_Diaphoretickes-Atha_6838_OG5081
84/83
Pp_Diaphoretickes-Atha_1835_OG5081
Pp_Diaphoretickes-Kfla_7706_OG5081
100/100
Pp_Diaphoretickes-Knit_10324_OG5081
96.6/59
Osmo_Fungi-Ccor_162_OG5081
92.3/95
Osmo_Fungi-Ccor_2374_OG5081
92.7/97
Osmo_Fungi-Ccor_2136_OG5081
18.4/37
Osmo_Fungi-Ncra_8023_OG5081
87.6/42
90.1/55
Osmo_Pseudofungi-Pvex_7110_OG5081
99.8/100
Osmo_Pseudofungi-Pult_1342_OG5081
94.8/83
Osmo_Fungi-Tdef_4024_OG5081
Bacteria-UP_b000002027_2618_OG5081
72.9/36
Cp_Diaphoretickes-Vbra_13858_OG5081
63.8/96
Cp_Diaphoretickes-Vbra_704_OG5081
68.9/55
Other_Diaphoretickes-Gavo_11433_OG5081
62.2/38
46.4/48
Bacteria-UP_b000000223_2097_OG5081
35/41
95.6/57
Bacteria-UP_b000011859_3386_OG5081
36.1/43
Bacteria-UP_b000001784_2269_OG5081
Pp_Diaphoretickes-Cpar_15192_OG5081
92.1/60
Osmo_Fungi-Umay_6082_OG5081
99.5/99
Bacteria-UP_b000010798_1293_OG5081
Other_Other-Egra_13219_OG5081
Osmo_Fungi-Ncra_8596_OG5081
78.4/60
Osmo_Fungi-Gpro_7504_OG5081
94.7/71
Bacteria-UP_b000037738_6209_OG5081
71.1/77
Osmo_Pseudofungi-Pvex_2908_OG5081_intClade_1
97.6/99
Osmo_Pseudofungi-Pult_12750_OG5081_intClade_1
99.9/100
Osmo_Fungi-Ncra_5938_OG5081_intClade_1
100/100
Bacteria-UP_b000002168_3277_OG5081
47.5/65
Bacteria-UP_b000037931_5004_OG5081
94.8/89
Bacteria-UP_b000001035_897_OG5081
89.1/80
Bacteria-UP_b000002686_1306_OG5081
97.8/97
Bacteria-UP_b000006820_2654_OG5081
96.1/85
Cp_Diaphoretickes-Ehux_20124_OG5081
100/100
Cp_Diaphoretickes-Ehux_2691_OG5081
37/40
Pp_Diaphoretickes-Gsul_1823_OG5081
97.8/98
64.7/35
Osmo_Fungi-Ncra_3224_OG5081
Other_Stramenopiles-Croe_8389_OG5081
96.7/96
Bacteria-UP_b000005634_3935_OG5081
99.5/99
79.9/78
Bacteria-UP_b000037971_2829_OG5081
100/100
Other_Holomycota-Patl_g4752_OG5081
Bacteria-UP_b000017175_4556_OG5081
0.3

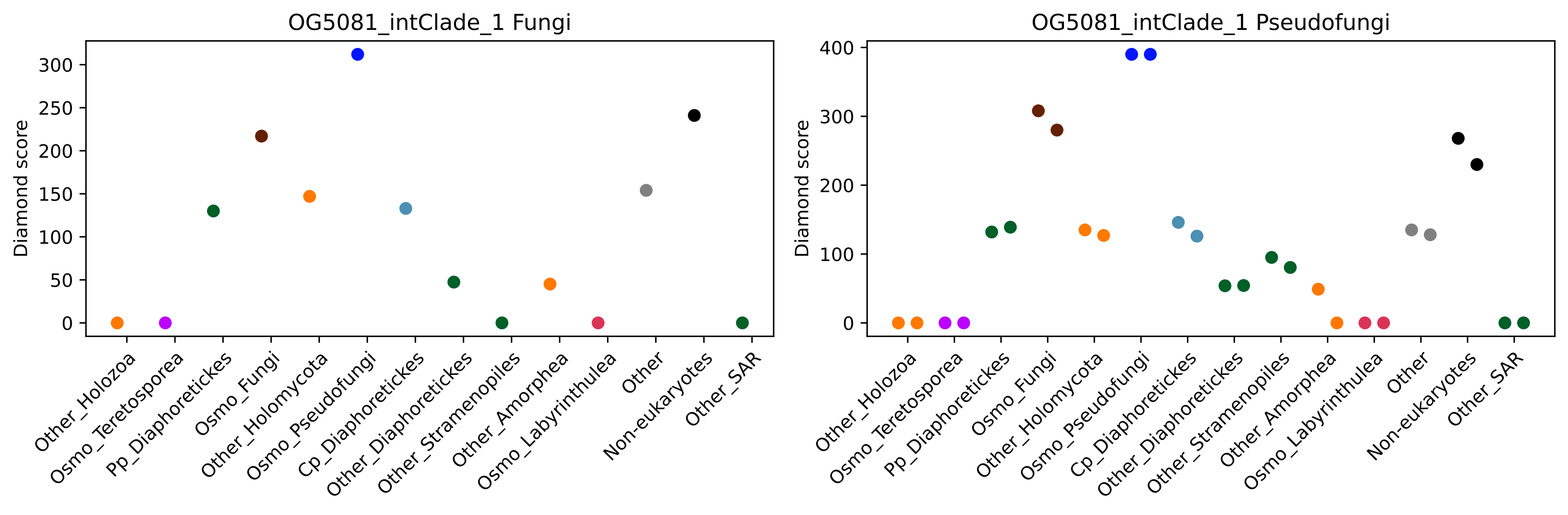

OG5081_intClade_1 Fungi
Diamond score
0
50
100
150
200
250
300
Other_Holozoa
Osmo_Teretosporea
Pp_Diaphoretickes
Osmo_Fungi
Other_Holomycota
Osmo_Pseudofungi
Cp_Diaphoretickes
Other_Diaphoretickes
Other_Stramenopiles
Other_Amorphea
Osmo_Labyrinthulea
Other
Non-eukaryotes
Other_SAR
OG5081_intClade_1 Pseudofungi
Diamond score
0
100
200
300
400
Other_Holozoa
Osmo_Teretosporea
Pp_Diaphoretickes
Osmo_Fungi
Other_Holomycota
Osmo_Pseudofungi
Cp_Diaphoretickes
Other_Diaphoretickes
Other_Stramenopiles
Other_Amorphea
Osmo_Labyrinthulea
Other
Non-eukaryotes
Other_SAR

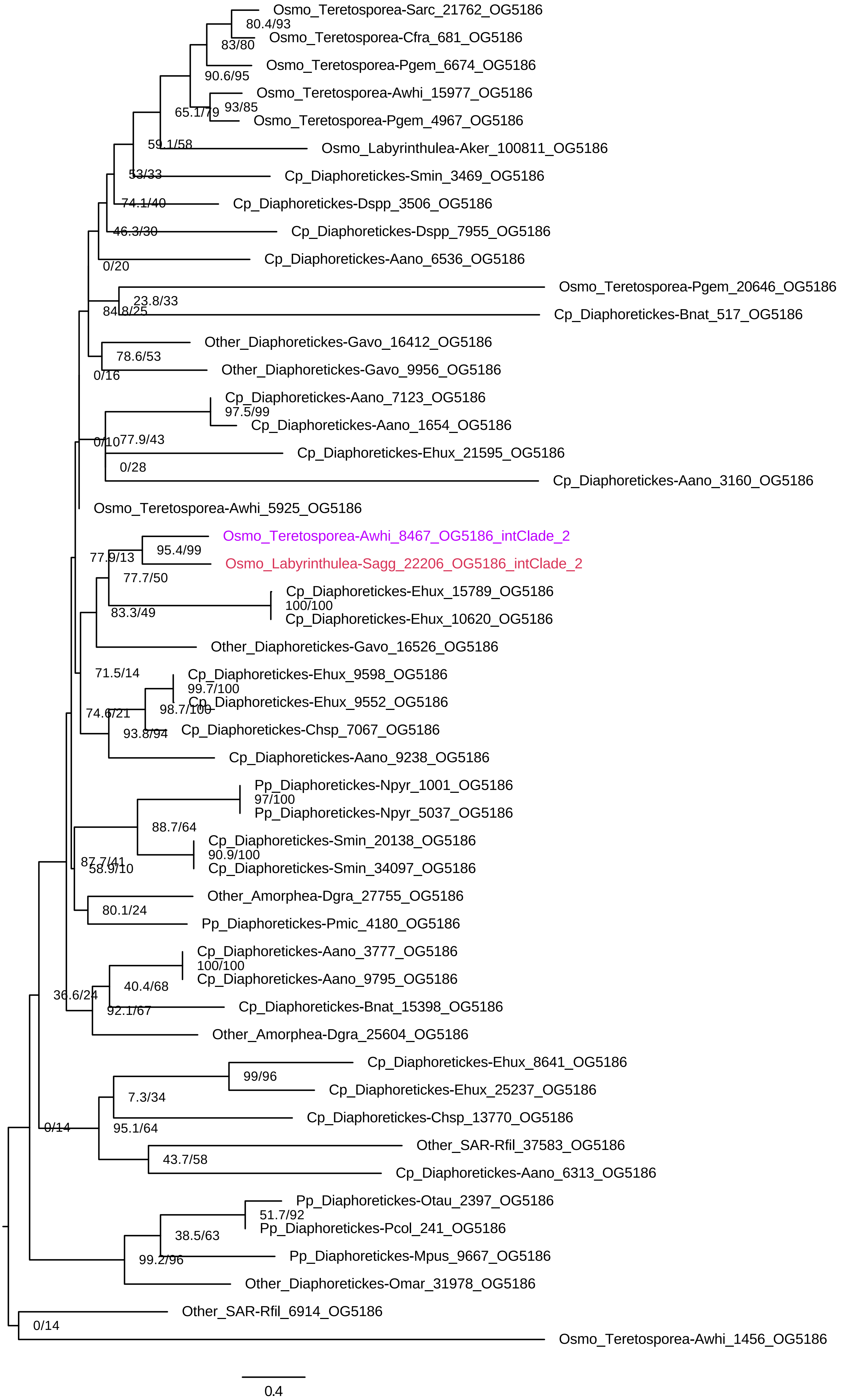
Osmo_Teretosporea-Sarc_21762_OG5186
80.4/93
Osmo_Teretosporea-Cfra_681_OG5186
83/80
Osmo_Teretosporea-Pgem_6674_OG5186
90.6/95
Osmo_Teretosporea-Awhi_15977_OG5186
93/85
65.1/79
Osmo_Teretosporea-Pgem_4967_OG5186
59.1/58
Osmo_Labyrinthulea-Aker_100811_OG5186
53/33
Cp_Diaphoretickes-Smin_3469_OG5186
74.1/40
Cp_Diaphoretickes-Dspp_3506_OG5186
46.3/30
Cp_Diaphoretickes-Dspp_7955_OG5186
Cp_Diaphoretickes-Aano_6536_OG5186
0/20
Osmo_Teretosporea-Pgem_20646_OG5186
23.8/33
84.8/25
Cp_Diaphoretickes-Bnat_517_OG5186
Other_Diaphoretickes-Gavo_16412_OG5186
78.6/53
Other_Diaphoretickes-Gavo_9956_OG5186
0/16
Cp_Diaphoretickes-Aano_7123_OG5186
97.5/99
Cp_Diaphoretickes-Aano_1654_OG5186
0/10
77.9/43
Cp_Diaphoretickes-Ehux_21595_OG5186
0/28
Cp_Diaphoretickes-Aano_3160_OG5186
Osmo_Teretosporea-Awhi_5925_OG5186
Osmo_Teretosporea-Awhi_8467_OG5186_intClade_2
95.4/99
77.9/13
Osmo_Labyrinthulea-Sagg_22206_OG5186_intClade_2
77.7/50
Cp_Diaphoretickes-Ehux_15789_OG5186
100/100
83.3/49
Cp_Diaphoretickes-Ehux_10620_OG5186
Other_Diaphoretickes-Gavo_16526_OG5186
71.5/14
Cp_Diaphoretickes-Ehux_9598_OG5186
99.7/100
Cp_Diaphoretickes-Ehux_9552_OG5186
98.7/100
74.6/21
Cp_Diaphoretickes-Chsp_7067_OG5186
93.8/94
Cp_Diaphoretickes-Aano_9238_OG5186
Pp_Diaphoretickes-Npyr_1001_OG5186
97/100
Pp_Diaphoretickes-Npyr_5037_OG5186
88.7/64
Cp_Diaphoretickes-Smin_20138_OG5186
90.9/100
87.7/41
58.9/10
Cp_Diaphoretickes-Smin_34097_OG5186
Other_Amorphea-Dgra_27755_OG5186
80.1/24
Pp_Diaphoretickes-Pmic_4180_OG5186
Cp_Diaphoretickes-Aano_3777_OG5186
100/100
40.4/68
Cp_Diaphoretickes-Aano_9795_OG5186
36.6/24
Cp_Diaphoretickes-Bnat_15398_OG5186
92.1/67
Other_Amorphea-Dgra_25604_OG5186
Cp_Diaphoretickes-Ehux_8641_OG5186
99/96
Cp_Diaphoretickes-Ehux_25237_OG5186
7.3/34
Cp_Diaphoretickes-Chsp_13770_OG5186
0/14
95.1/64
Other_SAR-Rfil_37583_OG5186
43.7/58
Cp_Diaphoretickes-Aano_6313_OG5186
Pp_Diaphoretickes-Otau_2397_OG5186
51.7/92
Pp_Diaphoretickes-Pcol_241_OG5186
38.5/63
Pp_Diaphoretickes-Mpus_9667_OG5186
99.2/96
Other_Diaphoretickes-Omar_31978_OG5186
Other_SAR-Rfil_6914_OG5186
0/14
Osmo_Teretosporea-Awhi_1456_OG5186
0.4

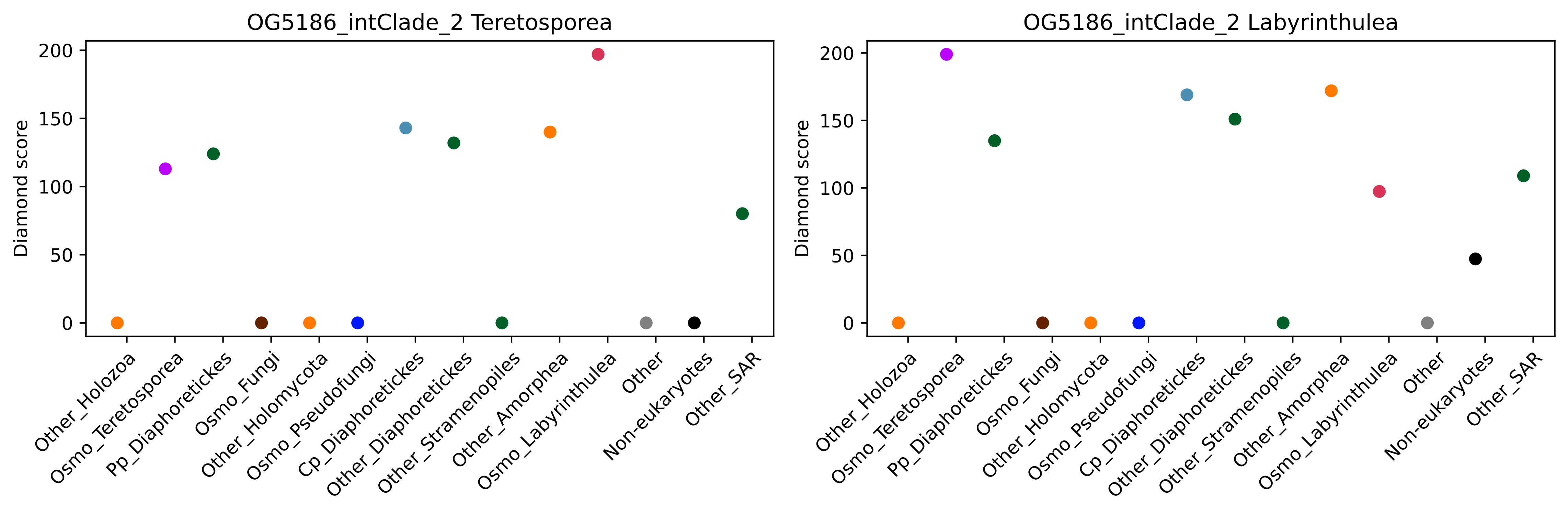

OG5186_intClade_2 Teretosporea
OG5186_intClade_2 Labyrinthulea
Diamond score
0
50
100
150
200
Other_Holozoa
Osmo_Teretosporea
Pp_Diaphoretickes
Osmo_Fungi
Other_Holomycota
Osmo_Pseudofungi
Cp_Diaphoretickes
Other_Diaphoretickes
Other_Stramenopiles
Other_Amorphea
Osmo_Labyrinthulea
Other
Non-eukaryotes
Other_SAR

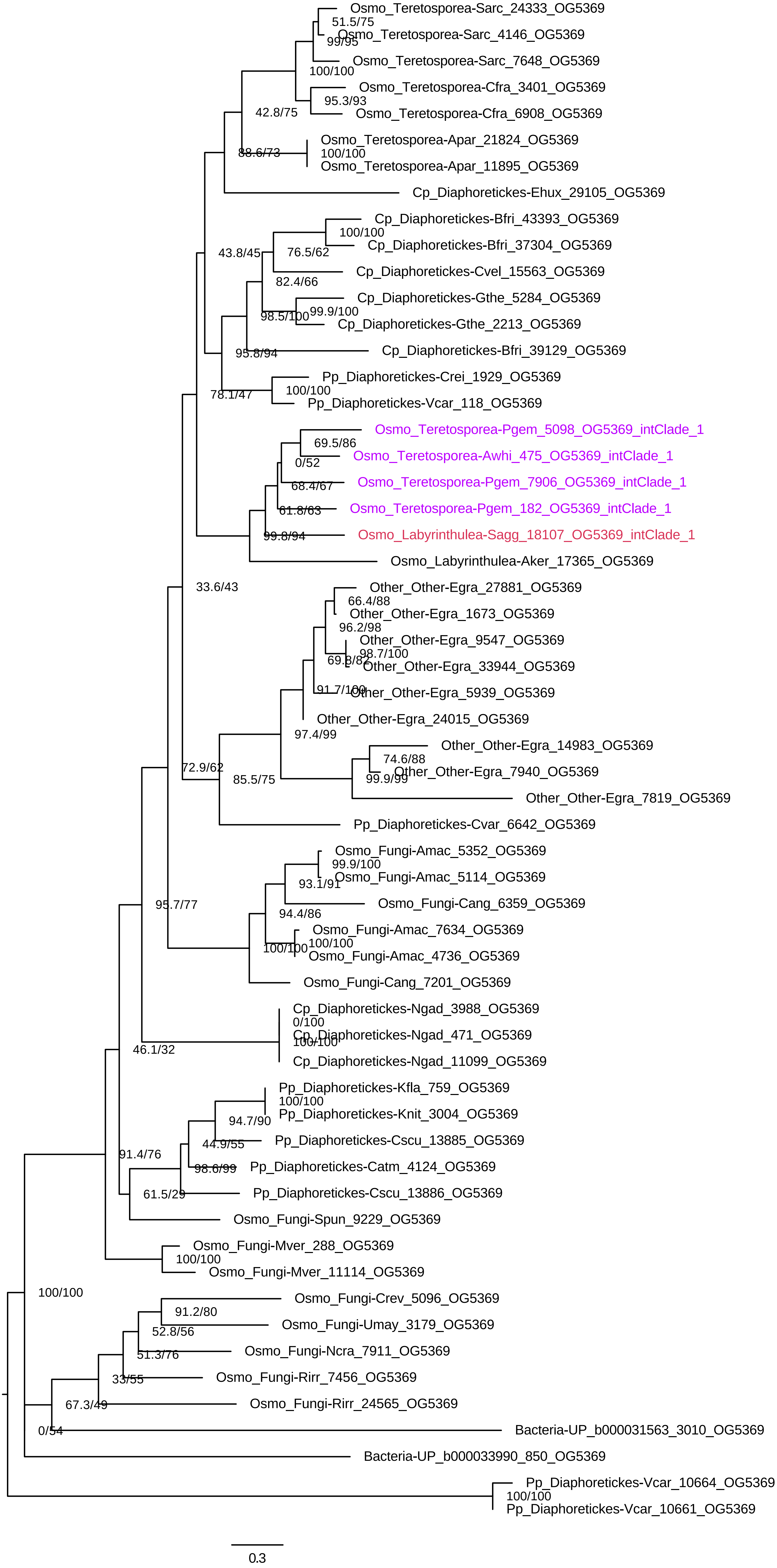

Osmo_Teretosporea-Sarc_24333_OG5369
51.5/75
99/95
Osmo_Teretosporea-Sarc_4146_OG5369
Osmo_Teretosporea-Sarc_7648_OG5369
100/100
Osmo_Teretosporea-Cfra_3401_OG5369
95.3/93
42.8/75
Osmo_Teretosporea-Cfra_6908_OG5369
Osmo_Teretosporea-Apar_21824_OG5369
88.6/73
100/100
Osmo_Teretosporea-Apar_11895_OG5369
Cp_Diaphoretickes-Ehux_29105_OG5369
Cp_Diaphoretickes-Bfri_43393_OG5369
100/100
43.8/45
76.5/62
Cp_Diaphoretickes-Bfri_37304_OG5369
Cp_Diaphoretickes-Cvel_15563_OG5369
82.4/66
Cp_Diaphoretickes-Gthe_5284_OG5369
98.5/100
99.9/100
Cp_Diaphoretickes-Gthe_2213_OG5369
95.8/94
Cp_Diaphoretickes-Bfri_39129_OG5369
Pp_Diaphoretickes-Crei_1929_OG5369
78.1/47
100/100
Pp_Diaphoretickes-Vcar_118_OG5369
Osmo_Teretosporea-Pgem_5098_OG5369_intClade_1
69.5/86
Osmo_Teretosporea-Awhi_475_OG5369_intClade_1
0/52
68.4/67
Osmo_Teretosporea-Pgem_7906_OG5369_intClade_1
61.8/63
Osmo_Teretosporea-Pgem_182_OG5369_intClade_1
99.8/94
Osmo_Labyrinthulea-Sagg_18107_OG5369_intClade_1
Osmo_Labyrinthulea-Aker_17365_OG5369
33.6/43
Other_Other-Egra_27881_OG5369
66.4/88
Other_Other-Egra_1673_OG5369
96.2/98
Other_Other-Egra_9547_OG5369
98.7/100
69.8/82
Other_Other-Egra_33944_OG5369
91.7/100
Other_Other-Egra_5939_OG5369
Other_Other-Egra_24015_OG5369
97.4/99
Other_Other-Egra_14983_OG5369
72.9/62
74.6/88
85.5/75
99.9/99
Other_Other-Egra_7940_OG5369
Other_Other-Egra_7819_OG5369
Pp_Diaphoretickes-Cvar_6642_OG5369
Osmo_Fungi-Amac_5352_OG5369
99.9/100
93.1/91
Osmo_Fungi-Amac_5114_OG5369
95.7/77
Osmo_Fungi-Cang_6359_OG5369
94.4/86
Osmo_Fungi-Amac_7634_OG5369
100/100
100/100
Osmo_Fungi-Amac_4736_OG5369
Osmo_Fungi-Cang_7201_OG5369
Cp_Diaphoretickes-Ngad_3988_OG5369
0/100
Cp_Diaphoretickes-Ngad_471_OG5369
100/100
46.1/32
Cp_Diaphoretickes-Ngad_11099_OG5369
Pp_Diaphoretickes-Kfla_759_OG5369
100/100
Pp_Diaphoretickes-Knit_3004_OG5369
94.7/90
44.9/55
Pp_Diaphoretickes-Cscu_13885_OG5369
91.4/76
98.6/99
Pp_Diaphoretickes-Catm_4124_OG5369
61.5/29
Pp_Diaphoretickes-Cscu_13886_OG5369
Osmo_Fungi-Spun_9229_OG5369
Osmo_Fungi-Mver_288_OG5369
100/100
Osmo_Fungi-Mver_11114_OG5369
100/100
Osmo_Fungi-Crev_5096_OG5369
91.2/80
52.8/56
Osmo_Fungi-Umay_3179_OG5369
51.3/76
Osmo_Fungi-Ncra_7911_OG5369
33/55
Osmo_Fungi-Rirr_7456_OG5369
67.3/49
Osmo_Fungi-Rirr_24565_OG5369
0/54
Bacteria-UP_b000031563_3010_OG5369
Bacteria-UP_b000033990_850_OG5369
Pp_Diaphoretickes-Vcar_10664_OG5369
100/100
Pp_Diaphoretickes-Vcar_10661_OG5369
0.3

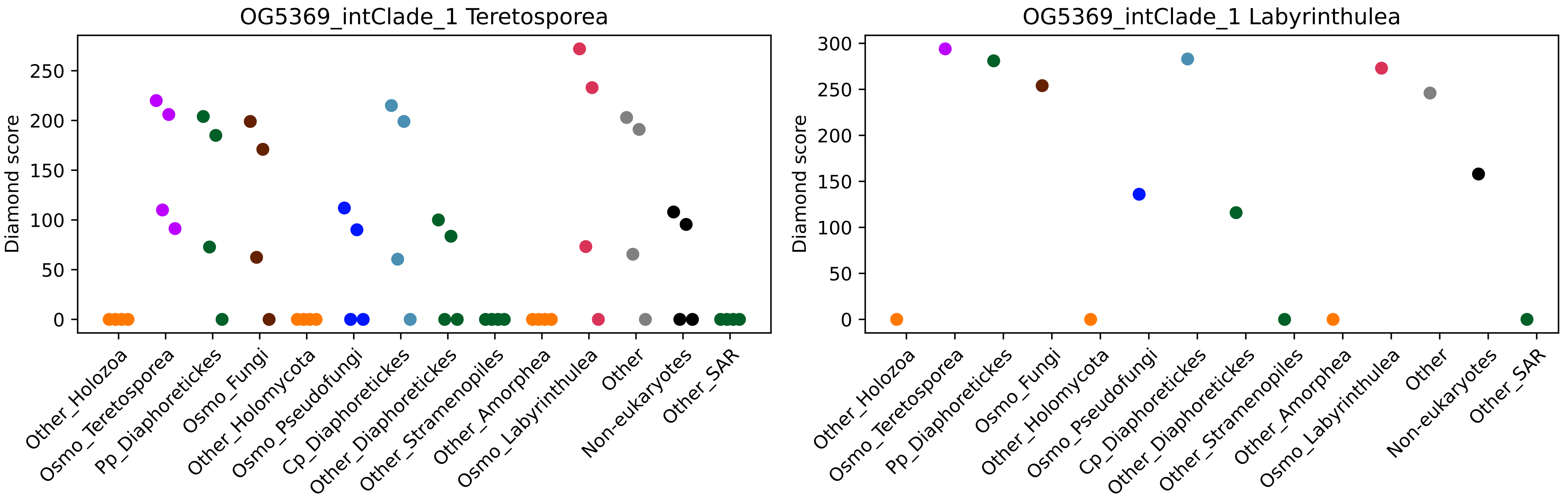

OG5369_intClade_1 Teretosporea
OG5369_intClade_1 Labyrinthulea
Diamond score
0
50
100
150
200
250
300
Other_Holozoa
Osmo_Teretosporea
Pp_Diaphoretickes
Osmo_Fungi
Other_Holomycota
Osmo_Pseudofungi
Cp_Diaphoretickes
Other_Diaphoretickes
Other_Stramenopiles
Other_Amorphea
Osmo_Labyrinthulea
Other
Non-eukaryotes
Other_SAR

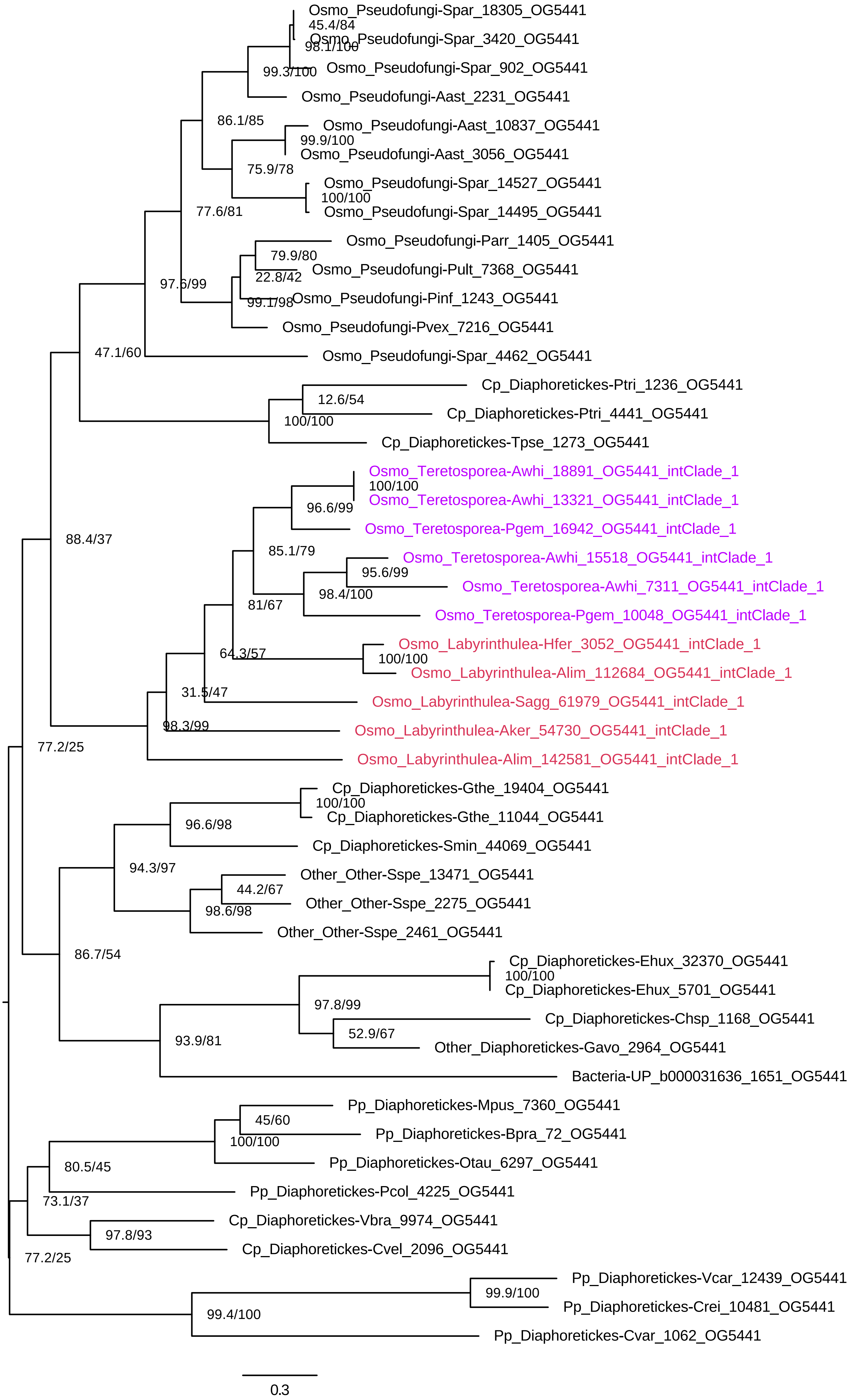
Osmo_Pseudofungi-Spar_18305_OG5441
45.4/84
Osmo_Pseudofungi-Spar_3420_OG5441
98.1/100
99.3/100
Osmo_Pseudofungi-Spar_902_OG5441
Osmo_Pseudofungi-Aast_2231_OG5441
86.1/85
Osmo_Pseudofungi-Aast_10837_OG5441
99.9/100
Osmo_Pseudofungi-Aast_3056_OG5441
75.9/78
Osmo_Pseudofungi-Spar_14527_OG5441
100/100
Osmo_Pseudofungi-Spar_14495_OG5441
77.6/81
Osmo_Pseudofungi-Parr_1405_OG5441
79.9/80
Osmo_Pseudofungi-Pult_7368_OG5441
22.8/42
97.6/99
99.1/98
Osmo_Pseudofungi-Pinf_1243_OG5441
Osmo_Pseudofungi-Pvex_7216_OG5441
47.1/60
Osmo_Pseudofungi-Spar_4462_OG5441
Cp_Diaphoretickes-Ptri_1236_OG5441
12.6/54
Cp_Diaphoretickes-Ptri_4441_OG5441
100/100
Cp_Diaphoretickes-Tpse_1273_OG5441
Osmo_Teretosporea-Awhi_18891_OG5441_intClade_1
100/100
Osmo_Teretosporea-Awhi_13321_OG5441_intClade_1
96.6/99
Osmo_Teretosporea-Pgem_16942_OG5441_intClade_1
88.4/37
85.1/79
Osmo_Teretosporea-Awhi_15518_OG5441_intClade_1
95.6/99
Osmo_Teretosporea-Awhi_7311_OG5441_intClade_1
98.4/100
81/67
Osmo_Teretosporea-Pgem_10048_OG5441_intClade_1
Osmo_Labyrinthulea-Hfer_3052_OG5441_intClade_1
64.3/57
100/100
Osmo_Labyrinthulea-Alim_112684_OG5441_intClade_1
31.5/47
Osmo_Labyrinthulea-Sagg_61979_OG5441_intClade_1
98.3/99
Osmo_Labyrinthulea-Aker_54730_OG5441_intClade_1
77.2/25
Osmo_Labyrinthulea-Alim_142581_OG5441_intClade_1
Cp_Diaphoretickes-Gthe_19404_OG5441
100/100
Cp_Diaphoretickes-Gthe_11044_OG5441
96.6/98
Cp_Diaphoretickes-Smin_44069_OG5441
94.3/97
Other_Other-Sspe_13471_OG5441
44.2/67
Other_Other-Sspe_2275_OG5441
98.6/98
Other_Other-Sspe_2461_OG5441
86.7/54
Cp_Diaphoretickes-Ehux_32370_OG5441
100/100
Cp_Diaphoretickes-Ehux_5701_OG5441
97.8/99
Cp_Diaphoretickes-Chsp_1168_OG5441
52.9/67
93.9/81
Other_Diaphoretickes-Gavo_2964_OG5441
Bacteria-UP_b000031636_1651_OG5441
Pp_Diaphoretickes-Mpus_7360_OG5441
45/60
Pp_Diaphoretickes-Bpra_72_OG5441
100/100
Pp_Diaphoretickes-Otau_6297_OG5441
80.5/45
Pp_Diaphoretickes-Pcol_4225_OG5441
73.1/37
Cp_Diaphoretickes-Vbra_9974_OG5441
97.8/93
Cp_Diaphoretickes-Cvel_2096_OG5441
77.2/25
Pp_Diaphoretickes-Vcar_12439_OG5441
99.9/100
Pp_Diaphoretickes-Crei_10481_OG5441
99.4/100
Pp_Diaphoretickes-Cvar_1062_OG5441
0.3

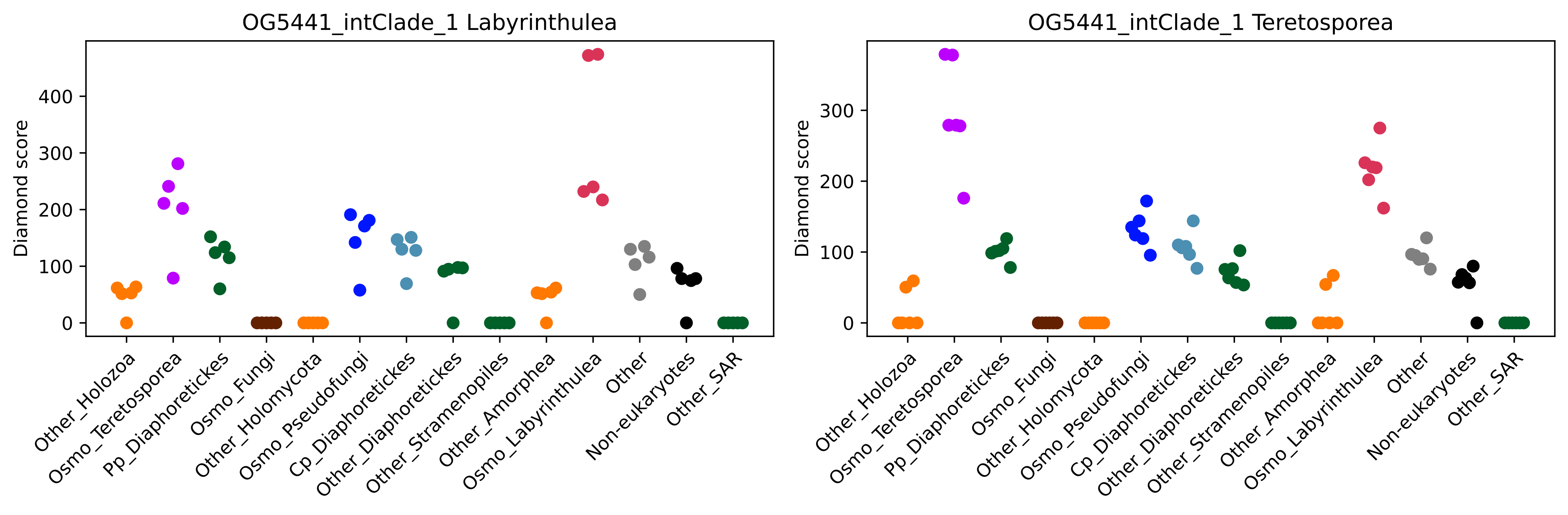

OG5441_intClade_1 Labyrinthulea
OG5441_intClade_1 Teretosporea
Diamond score
0
100
200
300
400
Other_Holozoa
Osmo_Teretosporea
Pp_Diaphoretickes
Osmo_Fungi
Other_Holomycota
Osmo_Pseudofungi
Cp_Diaphoretickes
Other_Diaphoretickes
Other_Stramenopiles
Other_Amorphea
Osmo_Labyrinthulea
Other
Non-eukaryotes
Other_SAR

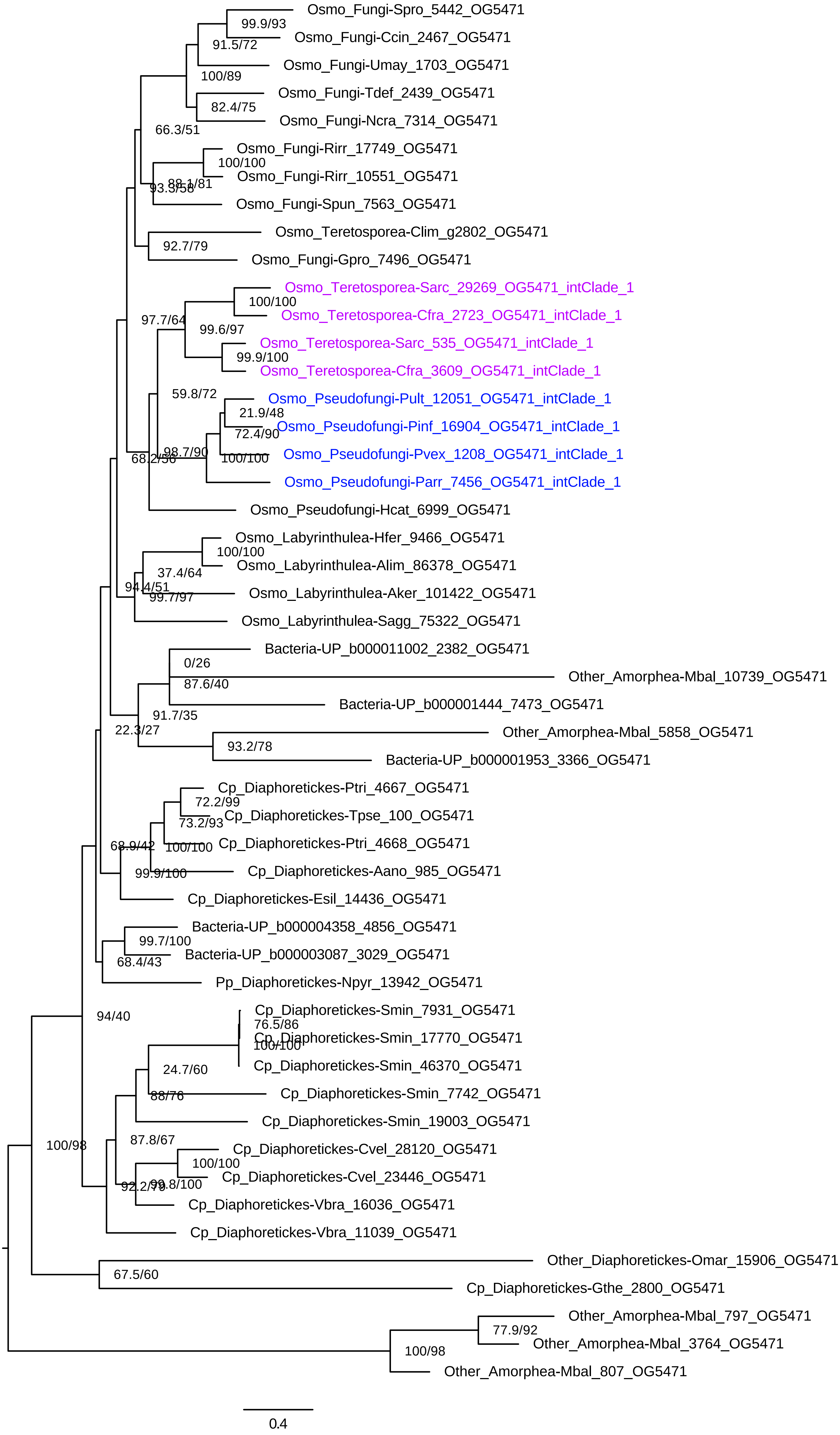

Osmo_Fungi-Spro_5442_OG5471
Osmo_Fungi-Ccin_2467_OG5471
Osmo_Fungi-Umay_1703_OG5471
Osmo_Fungi-Tdef_2439_OG5471
Osmo_Fungi-Ncra_7314_OG5471
Osmo_Fungi-Rirr_17749_OG5471
Osmo_Fungi-Rirr_10551_OG5471
Osmo_Fungi-Spun_7563_OG5471
Osmo_Teretosporea-Clim_g2802_OG5471
Osmo_Fungi-Gpro_7496_OG5471
Osmo_Teretosporea-Sarc_29269_OG5471_intClade_1
Osmo_Teretosporea-Cfra_2723_OG5471_intClade_1
Osmo_Teretosporea-Sarc_535_OG5471_intClade_1
Osmo_Teretosporea-Cfra_3609_OG5471_intClade_1
Osmo_Pseudofungi-Pult_12051_OG5471_intClade_1
Osmo_Pseudofungi-Pinf_16904_OG5471_intClade_1
Osmo_Pseudofungi-Pvex_1208_OG5471_intClade_1
Osmo_Pseudofungi-Parr_7456_OG5471_intClade_1
Osmo_Pseudofungi-Hcat_6999_OG5471
Osmo_Labyrinthulea-Hfer_9466_OG5471
Osmo_Labyrinthulea-Alim_86378_OG5471
Osmo_Labyrinthulea-Aker_101422_OG5471
Osmo_Labyrinthulea-Sagg_75322_OG5471
Bacteria-UP_b000011002_2382_OG5471
Other_Amorphea-Mbal_10739_OG5471
Bacteria-UP_b000001444_7473_OG5471
Other_Amorphea-Mbal_5858_OG5471
Bacteria-UP_b000001953_3366_OG5471
Cp_Diaphoretickes-Ptri_4667_OG5471
Cp_Diaphoretickes-Tpse_100_OG5471
Cp_Diaphoretickes-Ptri_4668_OG5471
Cp_Diaphoretickes-Aano_985_OG5471
Cp_Diaphoretickes-Esil_14436_OG5471
Bacteria-UP_b000004358_4856_OG5471
Bacteria-UP_b000003087_3029_OG5471
Pp_Diaphoretickes-Npyr_13942_OG5471
Cp_Diaphoretickes-Smin_7931_OG5471
Cp_Diaphoretickes-Smin_17770_OG5471
Cp_Diaphoretickes-Smin_46370_OG5471
Cp_Diaphoretickes-Smin_7742_OG5471
Cp_Diaphoretickes-Smin_19003_OG5471
Cp_Diaphoretickes-Cvel_28120_OG5471
Cp_Diaphoretickes-Cvel_23446_OG5471
Cp_Diaphoretickes-Vbra_16036_OG5471
Cp_Diaphoretickes-Vbra_11039_OG5471
Other_Diaphoretickes-Omar_15906_OG5471
Cp_Diaphoretickes-Gthe_2800_OG5471
Other_Amorphea-Mbal_797_OG5471
Other_Amorphea-Mbal_3764_OG5471
Other_Amorphea-Mbal_807_OG5471
0.4

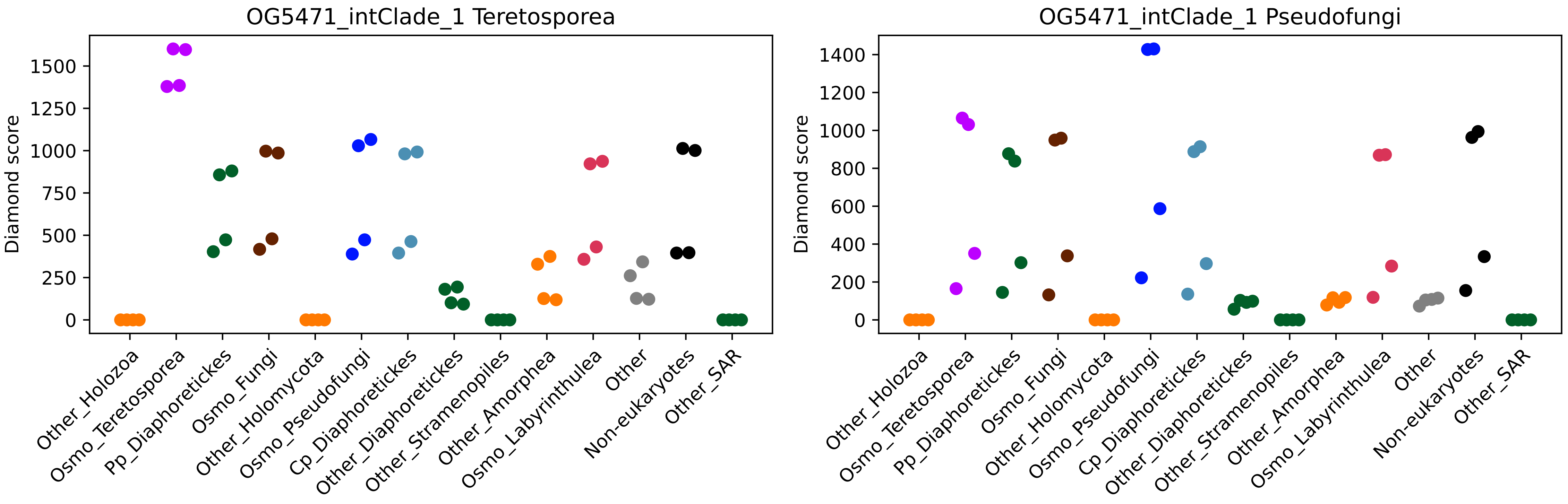
OG5471_intClade_1 Teretosporea
OG5471_intClade_1 Pseudofungi
Diamond score
0
250
500
750
1000
1250
1500
200
400
600
800
1200
1400
Other_Holozoa
Osmo_Teretosporea
Pp_Diaphoretickes
Osmo_Fungi
Other_Holomycota
Osmo_Pseudofungi
Cp_Diaphoretickes
Other_Diaphoretickes
Other_Stramenopiles
Other_Amorphea
Osmo_Labyrinthulea
Other
Non-eukaryotes
Other_SAR

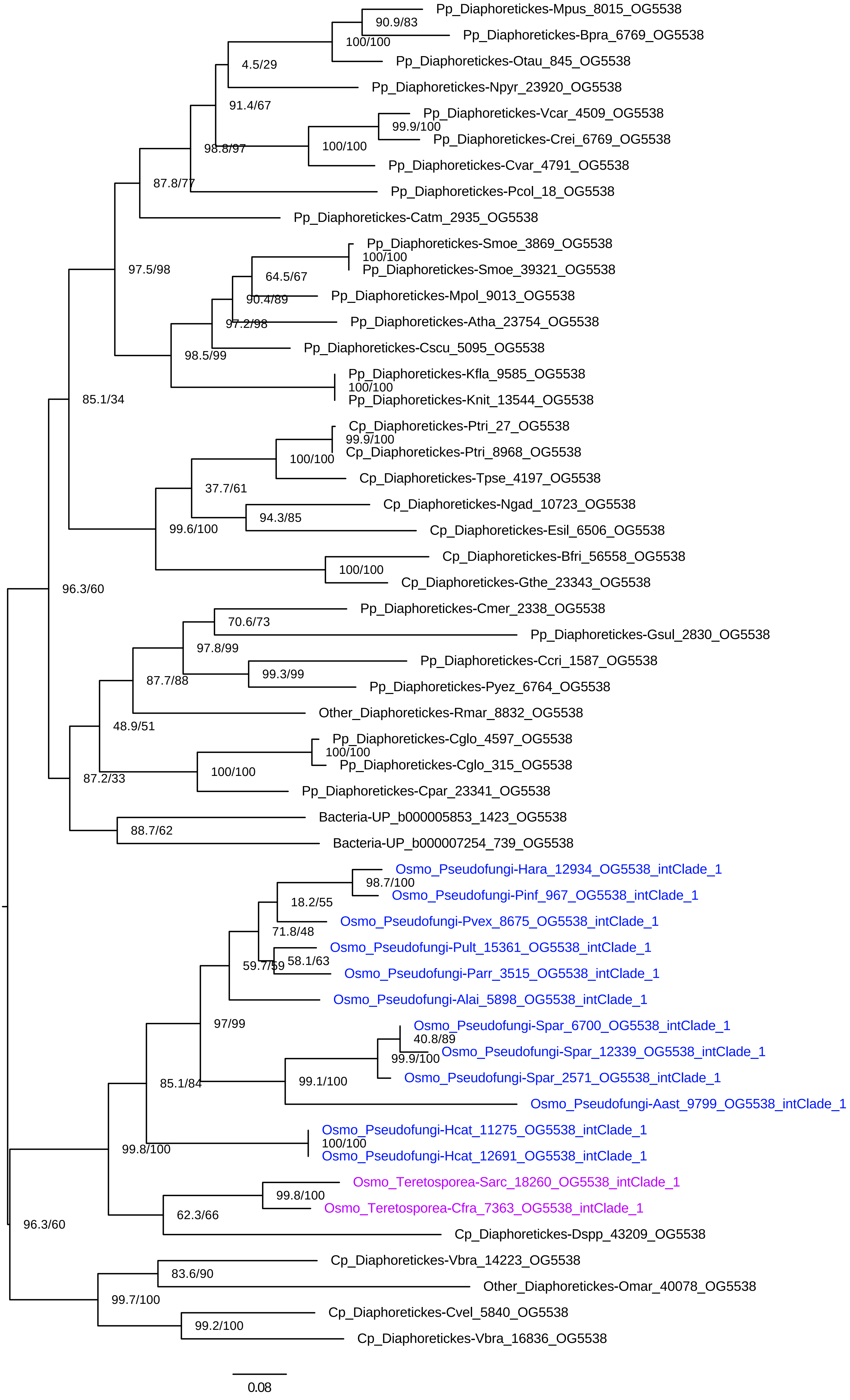
Pp_Diaphoretickes-Mpus_8015_OG5538
90.9/83
Pp_Diaphoretickes-Bpra_6769_OG5538
100/100
Pp_Diaphoretickes-Otau_845_OG5538
4.5/29
Pp_Diaphoretickes-Npyr_23920_OG5538
91.4/67
Pp_Diaphoretickes-Vcar_4509_OG5538
99.9/100
Pp_Diaphoretickes-Crei_6769_OG5538
98.8/97
100/100
Pp_Diaphoretickes-Cvar_4791_OG5538
87.8/77
Pp_Diaphoretickes-Pcol_18_OG5538
Pp_Diaphoretickes-Catm_2935_OG5538
Pp_Diaphoretickes-Smoe_3869_OG5538
100/100
Pp_Diaphoretickes-Smoe_39321_OG5538
97.5/98
64.5/67
Pp_Diaphoretickes-Mpol_9013_OG5538
90.4/89
Pp_Diaphoretickes-Atha_23754_OG5538
97.2/98
Pp_Diaphoretickes-Cscu_5095_OG5538
98.5/99
Pp_Diaphoretickes-Kfla_9585_OG5538
100/100
Pp_Diaphoretickes-Knit_13544_OG5538
85.1/34
Cp_Diaphoretickes-Ptri_27_OG5538
99.9/100
Cp_Diaphoretickes-Ptri_8968_OG5538
100/100
Cp_Diaphoretickes-Tpse_4197_OG5538
37.7/61
Cp_Diaphoretickes-Ngad_10723_OG5538
94.3/85
99.6/100
Cp_Diaphoretickes-Esil_6506_OG5538
Cp_Diaphoretickes-Bfri_56558_OG5538
100/100
Cp_Diaphoretickes-Gthe_23343_OG5538
96.3/60
Pp_Diaphoretickes-Cmer_2338_OG5538
70.6/73
Pp_Diaphoretickes-Gsul_2830_OG5538
97.8/99
Pp_Diaphoretickes-Ccri_1587_OG5538
99.3/99
87.7/88
Pp_Diaphoretickes-Pyez_6764_OG5538
Other_Diaphoretickes-Rmar_8832_OG5538
48.9/51
Pp_Diaphoretickes-Cglo_4597_OG5538
100/100
Pp_Diaphoretickes-Cglo_315_OG5538
100/100
87.2/33
Pp_Diaphoretickes-Cpar_23341_OG5538
Bacteria-UP_b000005853_1423_OG5538
88.7/62
Bacteria-UP_b000007254_739_OG5538
Osmo_Pseudofungi-Hara_12934_OG5538_intClade_1
98.7/100
Osmo_Pseudofungi-Pinf_967_OG5538_intClade_1
18.2/55
Osmo_Pseudofungi-Pvex_8675_OG5538_intClade_1
71.8/48
Osmo_Pseudofungi-Pult_15361_OG5538_intClade_1
58.1/63
59.7/59
Osmo_Pseudofungi-Parr_3515_OG5538_intClade_1
Osmo_Pseudofungi-Alai_5898_OG5538_intClade_1
97/99
Osmo_Pseudofungi-Spar_6700_OG5538_intClade_1
40.8/89
Osmo_Pseudofungi-Spar_12339_OG5538_intClade_1
99.9/100
85.1/84
99.1/100
Osmo_Pseudofungi-Spar_2571_OG5538_intClade_1
Osmo_Pseudofungi-Aast_9799_OG5538_intClade_1
Osmo_Pseudofungi-Hcat_11275_OG5538_intClade_1
100/100
99.8/100
Osmo_Pseudofungi-Hcat_12691_OG5538_intClade_1
Osmo_Teretosporea-Sarc_18260_OG5538_intClade_1
99.8/100
Osmo_Teretosporea-Cfra_7363_OG5538_intClade_1
96.3/60
62.3/66
Cp_Diaphoretickes-Dspp_43209_OG5538
Cp_Diaphoretickes-Vbra_14223_OG5538
83.6/90
Other_Diaphoretickes-Omar_40078_OG5538
99.7/100
Cp_Diaphoretickes-Cvel_5840_OG5538
99.2/100
Cp_Diaphoretickes-Vbra_16836_OG5538
0.08

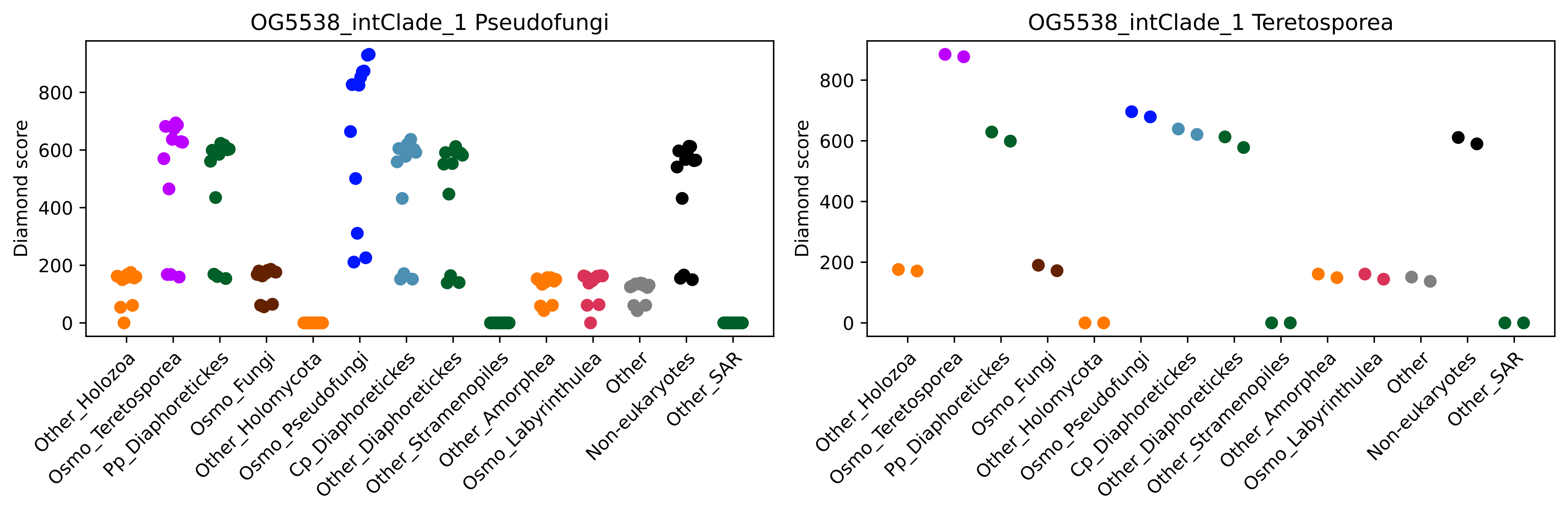

OG5538_intClade_1 Pseudofungi
OG5538_intClade_1 Teretosporea
Diamond score
0
200
400
600
800
Other_Holozoa
Osmo_Teretosporea
Pp_Diaphoretickes
Osmo_Fungi
Other_Holomycota
Osmo_Pseudofungi
Cp_Diaphoretickes
Other_Diaphoretickes
Other_Stramenopiles
Other_Amorphea
Osmo_Labyrinthulea
Other
Non-eukaryotes
Other_SAR

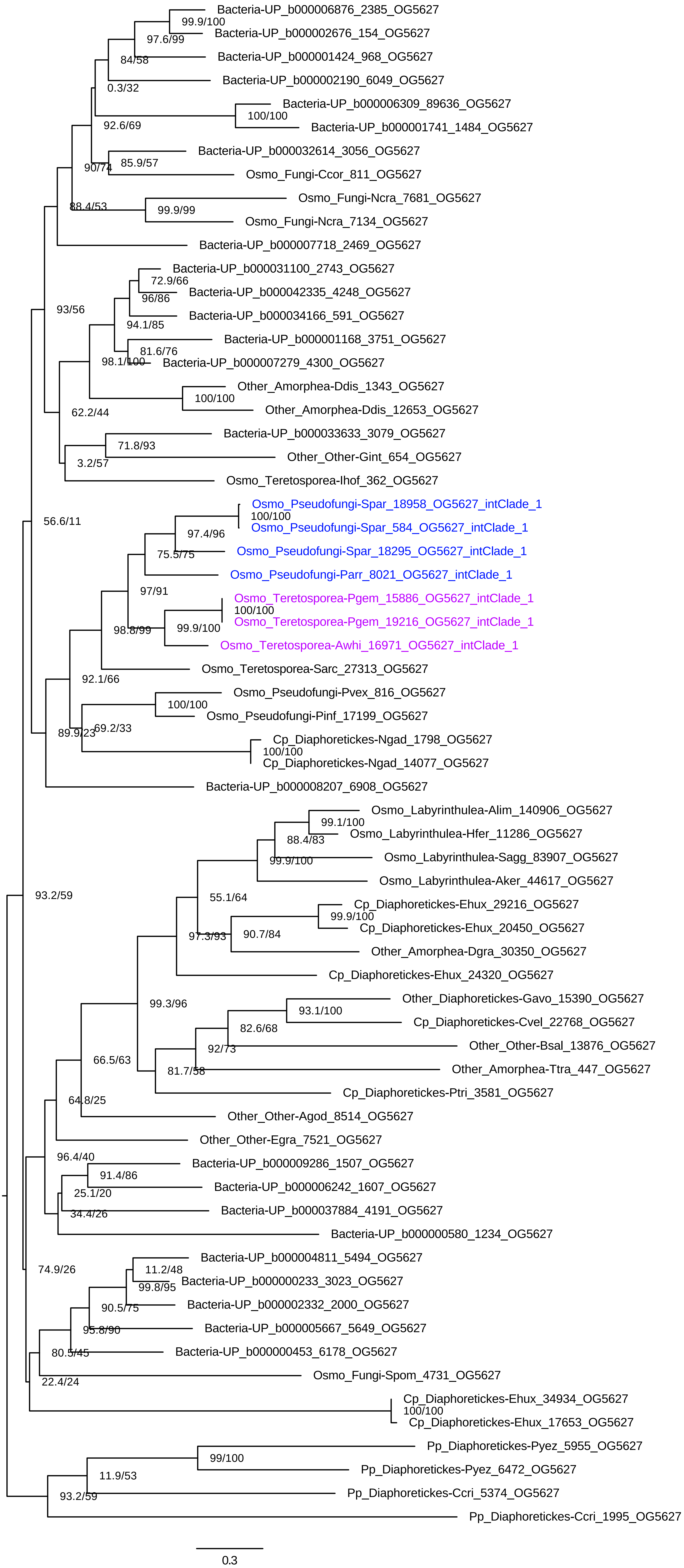

Bacteria-UP_b000006876_2385_OG5627
99.9/100
Bacteria-UP_b000002676_154_OG5627
97.6/99
Bacteria-UP_b000001424_968_OG5627
84/58
Bacteria-UP_b000002190_6049_OG5627
0.3/32
Bacteria-UP_b000006309_89636_OG5627
100/100
Bacteria-UP_b000001741_1484_OG5627
92.6/69
Bacteria-UP_b000032614_3056_OG5627
90/74
85.9/57
Osmo_Fungi-Ccor_811_OG5627
Osmo_Fungi-Ncra_7681_OG5627
88.4/53
99.9/99
Osmo_Fungi-Ncra_7134_OG5627
Bacteria-UP_b000007718_2469_OG5627
Bacteria-UP_b000031100_2743_OG5627
72.9/66
96/86
Bacteria-UP_b000042335_4248_OG5627
93/56
Bacteria-UP_b000034166_591_OG5627
94.1/85
Bacteria-UP_b000001168_3751_OG5627
81.6/76
98.1/100
Bacteria-UP_b000007279_4300_OG5627
Other_Amorphea-Ddis_1343_OG5627
100/100
62.2/44
Other_Amorphea-Ddis_12653_OG5627
Bacteria-UP_b000033633_3079_OG5627
71.8/93
Other_Other-Gint_654_OG5627
3.2/57
Osmo_Teretosporea-Ihof_362_OG5627
Osmo_Pseudofungi-Spar_18958_OG5627_intClade_1
100/100
56.6/11
Osmo_Pseudofungi-Spar_584_OG5627_intClade_1
97.4/96
75.5/75
Osmo_Pseudofungi-Spar_18295_OG5627_intClade_1
Osmo_Pseudofungi-Parr_8021_OG5627_intClade_1
97/91
Osmo_Teretosporea-Pgem_15886_OG5627_intClade_1
100/100
98.8/99
99.9/100
Osmo_Teretosporea-Pgem_19216_OG5627_intClade_1
Osmo_Teretosporea-Awhi_16971_OG5627_intClade_1
Osmo_Teretosporea-Sarc_27313_OG5627
92.1/66
Osmo_Pseudofungi-Pvex_816_OG5627
100/100
Osmo_Pseudofungi-Pinf_17199_OG5627
89.9/23
69.2/33
Cp_Diaphoretickes-Ngad_1798_OG5627
100/100
Cp_Diaphoretickes-Ngad_14077_OG5627
Bacteria-UP_b000008207_6908_OG5627
Osmo_Labyrinthulea-Alim_140906_OG5627
99.1/100
Osmo_Labyrinthulea-Hfer_11286_OG5627
88.4/83
99.9/100
Osmo_Labyrinthulea-Sagg_83907_OG5627
Osmo_Labyrinthulea-Aker_44617_OG5627
93.2/59
55.1/64
Cp_Diaphoretickes-Ehux_29216_OG5627
99.9/100
97.3/93
90.7/84
Cp_Diaphoretickes-Ehux_20450_OG5627
Other_Amorphea-Dgra_30350_OG5627
Cp_Diaphoretickes-Ehux_24320_OG5627
99.3/96
Other_Diaphoretickes-Gavo_15390_OG5627
93.1/100
Cp_Diaphoretickes-Cvel_22768_OG5627
82.6/68
92/73
Other_Other-Bsal_13876_OG5627
66.5/63
81.7/58
Other_Amorphea-Ttra_447_OG5627
Cp_Diaphoretickes-Ptri_3581_OG5627
64.8/25
Other_Other-Agod_8514_OG5627
Other_Other-Egra_7521_OG5627
96.4/40
Bacteria-UP_b000009286_1507_OG5627
91.4/86
25.1/20
Bacteria-UP_b000006242_1607_OG5627
34.4/26
Bacteria-UP_b000037884_4191_OG5627
Bacteria-UP_b000000580_1234_OG5627
Bacteria-UP_b000004811_5494_OG5627
74.9/26
11.2/48
Bacteria-UP_b000000233_3023_OG5627
99.8/95
Bacteria-UP_b000002332_2000_OG5627
90.5/75
95.8/90
Bacteria-UP_b000005667_5649_OG5627
80.5/45
Bacteria-UP_b000000453_6178_OG5627
22.4/24
Osmo_Fungi-Spom_4731_OG5627
Cp_Diaphoretickes-Ehux_34934_OG5627
100/100
Cp_Diaphoretickes-Ehux_17653_OG5627
Pp_Diaphoretickes-Pyez_5955_OG5627
99/100
Pp_Diaphoretickes-Pyez_6472_OG5627
11.9/53
Pp_Diaphoretickes-Ccri_5374_OG5627
93.2/59
Pp_Diaphoretickes-Ccri_1995_OG5627
0.3

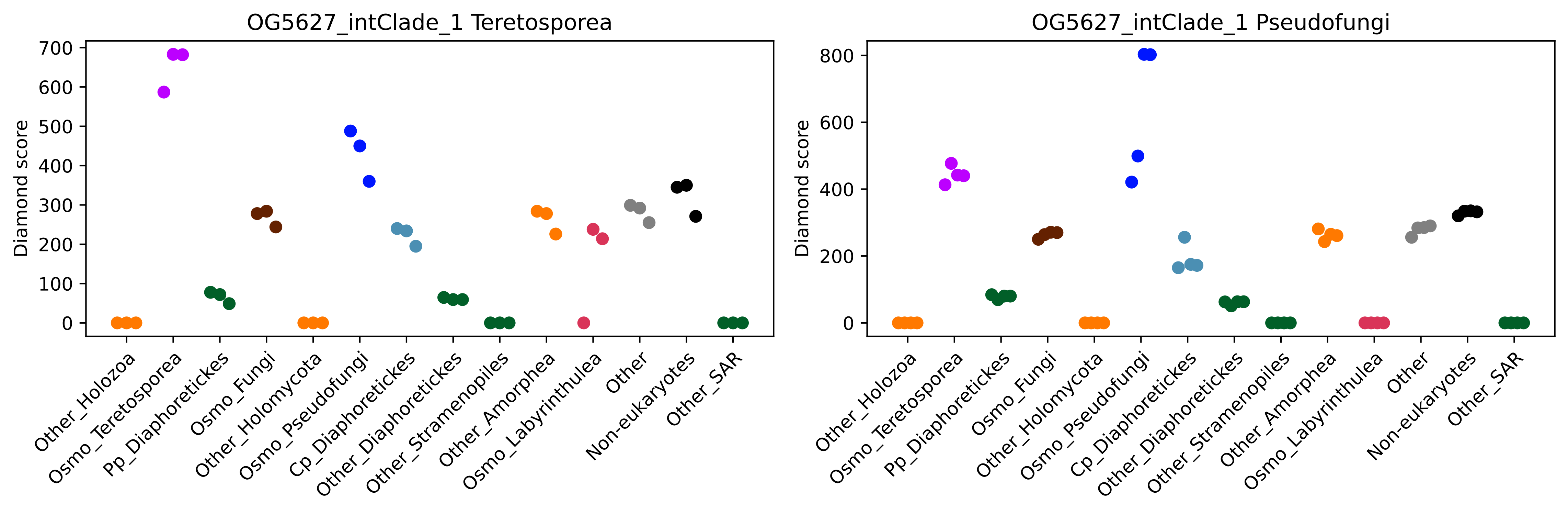
OG5627_intClade_1 Teretosporea
OG5627_intClade_1 Pseudofungi
Diamond score
0
100
200
300
400
500
600
700
800
Other_Holozoa
Osmo_Teretosporea
Pp_Diaphoretickes
Osmo_Fungi
Other_Holomycota
Osmo_Pseudofungi
Cp_Diaphoretickes
Other_Diaphoretickes
Other_Stramenopiles
Other_Amorphea
Osmo_Labyrinthulea
Other
Non-eukaryotes
Other_SAR

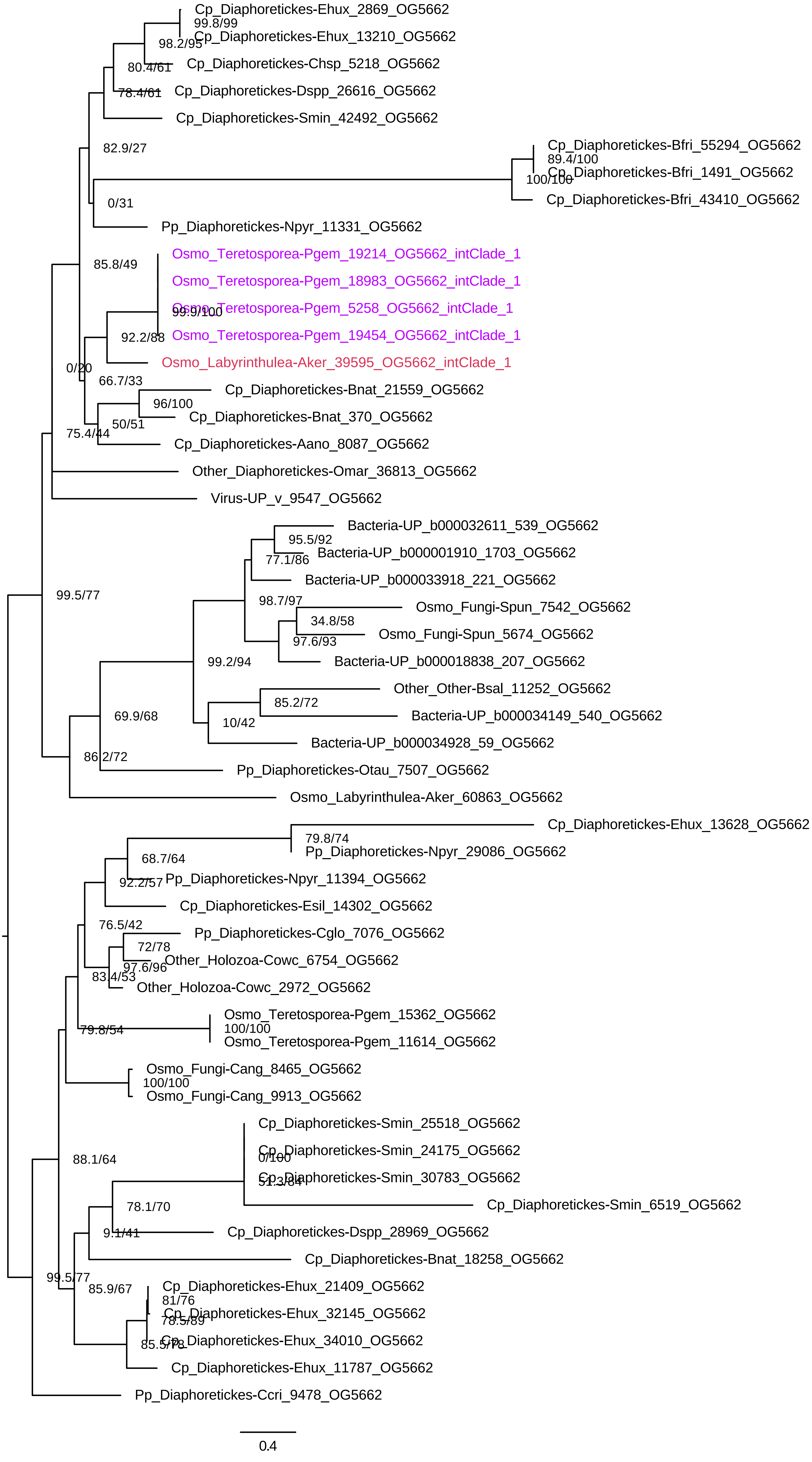
Cp_Diaphoretickes-Ehux_2869_OG5662
99.8/99
Cp_Diaphoretickes-Ehux_13210_OG5662
98.2/95
Cp_Diaphoretickes-Chsp_5218_OG5662
80.4/61
Cp_Diaphoretickes-Dspp_26616_OG5662
78.4/61
Cp_Diaphoretickes-Smin_42492_OG5662
82.9/27
Cp_Diaphoretickes-Bfri_55294_OG5662
89.4/100
Cp_Diaphoretickes-Bfri_1491_OG5662
100/100
Cp_Diaphoretickes-Bfri_43410_OG5662
0/31
Pp_Diaphoretickes-Npyr_11331_OG5662
85.8/49
Osmo_Teretosporea-Pgem_19214_OG5662_intClade_1
Osmo_Teretosporea-Pgem_18983_OG5662_intClade_1
Osmo_Teretosporea-Pgem_5258_OG5662_intClade_1
99.9/100
Osmo_Teretosporea-Pgem_19454_OG5662_intClade_1
92.2/88
Osmo_Labyrinthulea-Aker_39595_OG5662_intClade_1
0/20
66.7/33
Cp_Diaphoretickes-Bnat_21559_OG5662
96/100
Cp_Diaphoretickes-Bnat_370_OG5662
50/51
75.4/44
Cp_Diaphoretickes-Aano_8087_OG5662
Other_Diaphoretickes-Omar_36813_OG5662
Virus-UP_v_9547_OG5662
Bacteria-UP_b000032611_539_OG5662
95.5/92
Bacteria-UP_b000001910_1703_OG5662
77.1/86
Bacteria-UP_b000033918_221_OG5662
99.5/77
98.7/97
Osmo_Fungi-Spun_7542_OG5662
34.8/58
Osmo_Fungi-Spun_5674_OG5662
97.6/93
Bacteria-UP_b000018838_207_OG5662
99.2/94
Other_Other-Bsal_11252_OG5662
85.2/72
Bacteria-UP_b000034149_540_OG5662
69.9/68
10/42
Bacteria-UP_b000034928_59_OG5662
86.2/72
Pp_Diaphoretickes-Otau_7507_OG5662
Osmo_Labyrinthulea-Aker_60863_OG5662
Cp_Diaphoretickes-Ehux_13628_OG5662
79.8/74
Pp_Diaphoretickes-Npyr_29086_OG5662
68.7/64
Pp_Diaphoretickes-Npyr_11394_OG5662
92.2/57
Cp_Diaphoretickes-Esil_14302_OG5662
76.5/42
Pp_Diaphoretickes-Cglo_7076_OG5662
72/78
Other_Holozoa-Cowc_6754_OG5662
97.6/96
83.4/53
Other_Holozoa-Cowc_2972_OG5662
Osmo_Teretosporea-Pgem_15362_OG5662
100/100
79.8/54
Osmo_Teretosporea-Pgem_11614_OG5662
Osmo_Fungi-Cang_8465_OG5662
100/100
Osmo_Fungi-Cang_9913_OG5662
Cp_Diaphoretickes-Smin_25518_OG5662
88.1/64
Cp_Diaphoretickes-Smin_24175_OG5662
0/100
Cp_Diaphoretickes-Smin_30783_OG5662
51.3/84
78.1/70
Cp_Diaphoretickes-Smin_6519_OG5662
9.1/41
Cp_Diaphoretickes-Dspp_28969_OG5662
Cp_Diaphoretickes-Bnat_18258_OG5662
99.5/77
85.9/67
Cp_Diaphoretickes-Ehux_21409_OG5662
81/76
Cp_Diaphoretickes-Ehux_32145_OG5662
78.5/89
85.5/78
Cp_Diaphoretickes-Ehux_34010_OG5662
Cp_Diaphoretickes-Ehux_11787_OG5662
Pp_Diaphoretickes-Ccri_9478_OG5662
0.4

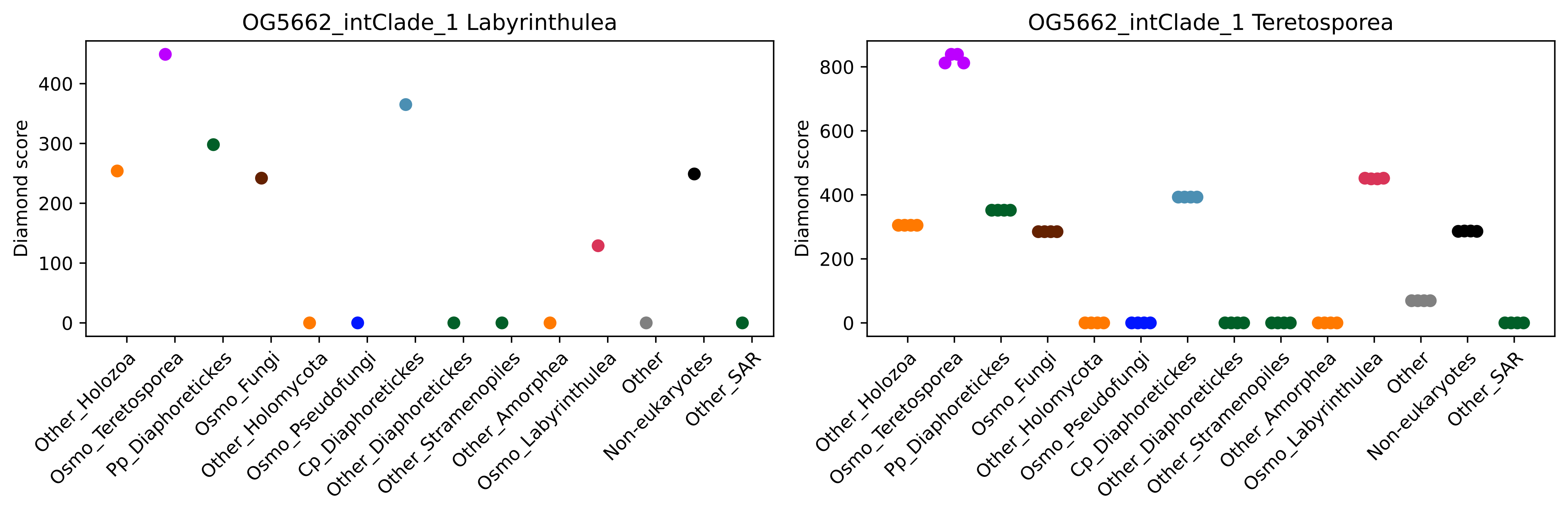

OG5662_intClade_1 Labyrinthulea
Diamond score
0
100
200
300
400
Other_Holozoa
Osmo_Teretosporea
Pp_Diaphoretickes
Osmo_Fungi
Other_Holomycota
Osmo_Pseudofungi
Cp_Diaphoretickes
Other_Diaphoretickes
Other_Stramenopiles
Other_Amorphea
Osmo_Labyrinthulea
Other
Non-eukaryotes
Other_SAR
OG5662_intClade_1 Teretosporea
Diamond score
0
200
400
600
800
Other_Holozoa
Osmo_Teretosporea
Pp_Diaphoretickes
Osmo_Fungi
Other_Holomycota
Osmo_Pseudofungi
Cp_Diaphoretickes
Other_Diaphoretickes
Other_Stramenopiles
Other_Amorphea
Osmo_Labyrinthulea
Other
Non-eukaryotes
Other_SAR

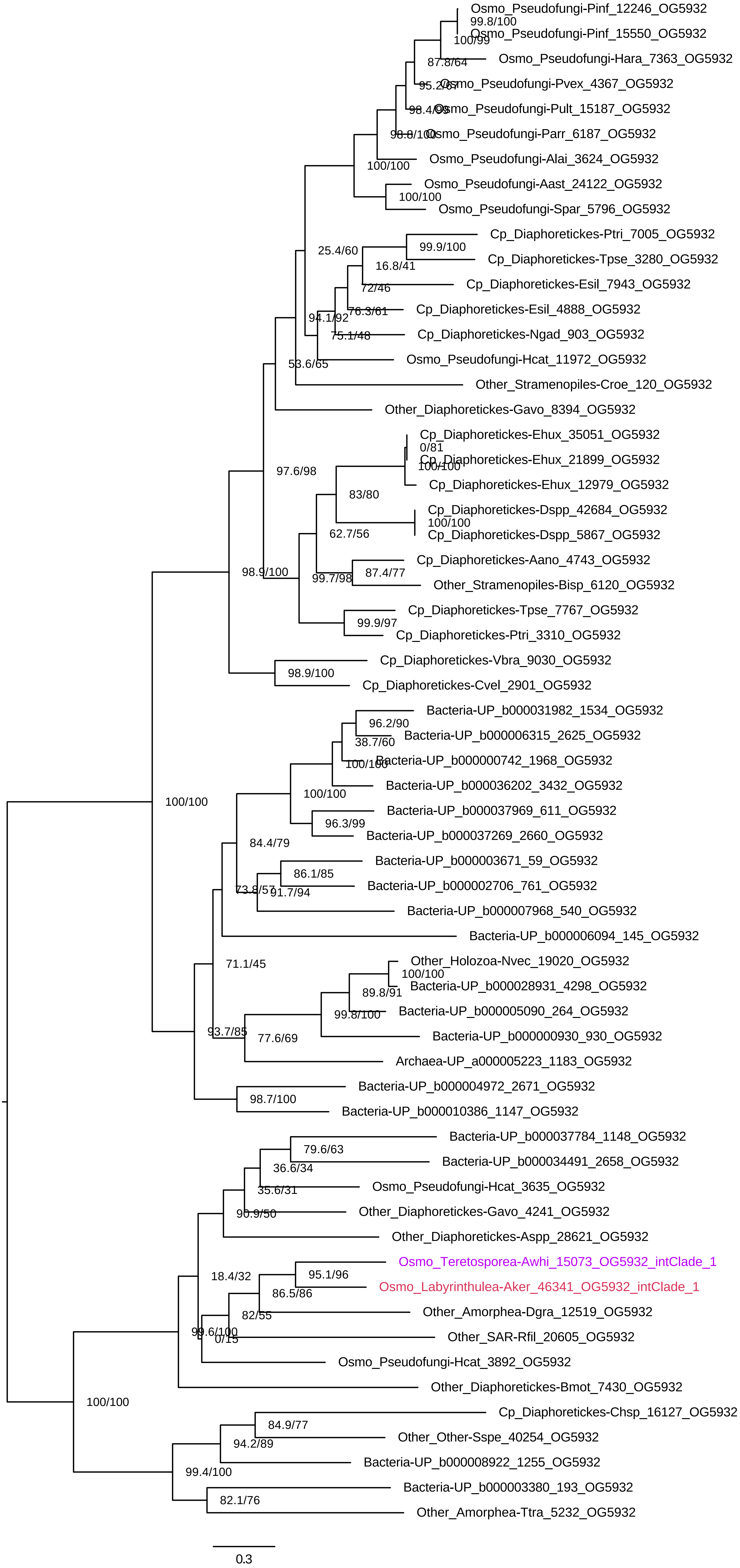
Osmo_Pseudofungi-Pinf_12246_OG5932
99.8/100
Osmo_Pseudofungi-Pinf_15550_OG5932
100/99
Osmo_Pseudofungi-Hara_7363_OG5932
87.8/64
Osmo_Pseudofungi-Pvex_4367_OG5932
95.2/67
Osmo_Pseudofungi-Pult_15187_OG5932
98.4/99
Osmo_Pseudofungi-Parr_6187_OG5932
98.8/100
Osmo_Pseudofungi-Alai_3624_OG5932
100/100
Osmo_Pseudofungi-Aast_24122_OG5932
100/100
Osmo_Pseudofungi-Spar_5796_OG5932
Cp_Diaphoretickes-Ptri_7005_OG5932
99.9/100
25.4/60
Cp_Diaphoretickes-Tpse_3280_OG5932
16.8/41
Cp_Diaphoretickes-Esil_7943_OG5932
72/46
Cp_Diaphoretickes-Esil_4888_OG5932
76.3/61
94.1/92
Cp_Diaphoretickes-Ngad_903_OG5932
75.1/48
Osmo_Pseudofungi-Hcat_11972_OG5932
53.6/65
Other_Stramenopiles-Croe_120_OG5932
Other_Diaphoretickes-Gavo_8394_OG5932
Cp_Diaphoretickes-Ehux_35051_OG5932
0/81
Cp_Diaphoretickes-Ehux_21899_OG5932
100/100
97.6/98
Cp_Diaphoretickes-Ehux_12979_OG5932
83/80
Cp_Diaphoretickes-Dspp_42684_OG5932
100/100
Cp_Diaphoretickes-Dspp_5867_OG5932
62.7/56
Cp_Diaphoretickes-Aano_4743_OG5932
98.9/100
87.4/77
99.7/98
Other_Stramenopiles-Bisp_6120_OG5932
Cp_Diaphoretickes-Tpse_7767_OG5932
99.9/97
Cp_Diaphoretickes-Ptri_3310_OG5932
Cp_Diaphoretickes-Vbra_9030_OG5932
98.9/100
Cp_Diaphoretickes-Cvel_2901_OG5932
Bacteria-UP_b000031982_1534_OG5932
96.2/90
Bacteria-UP_b000006315_2625_OG5932
38.7/60
Bacteria-UP_b000000742_1968_OG5932
100/100
100/100
Bacteria-UP_b000036202_3432_OG5932
100/100
Bacteria-UP_b000037969_611_OG5932
96.3/99
Bacteria-UP_b000037269_2660_OG5932
84.4/79
Bacteria-UP_b000003671_59_OG5932
86.1/85
Bacteria-UP_b000002706_761_OG5932
73.8/57
91.7/94
Bacteria-UP_b000007968_540_OG5932
Bacteria-UP_b000006094_145_OG5932
71.1/45
Other_Holozoa-Nvec_19020_OG5932
100/100
Bacteria-UP_b000028931_4298_OG5932
89.8/91
Bacteria-UP_b000005090_264_OG5932
99.8/100
93.7/85
77.6/69
Bacteria-UP_b000000930_930_OG5932
Archaea-UP_a000005223_1183_OG5932
Bacteria-UP_b000004972_2671_OG5932
98.7/100
Bacteria-UP_b000010386_1147_OG5932
Bacteria-UP_b000037784_1148_OG5932
79.6/63
Bacteria-UP_b000034491_2658_OG5932
36.6/34
Osmo_Pseudofungi-Hcat_3635_OG5932
35.6/31
Other_Diaphoretickes-Gavo_4241_OG5932
90.9/50
Other_Diaphoretickes-Aspp_28621_OG5932
Osmo_Teretosporea-Awhi_15073_OG5932_intClade_1
18.4/32
95.1/96
Osmo_Labyrinthulea-Aker_46341_OG5932_intClade_1
86.5/86
Other_Amorphea-Dgra_12519_OG5932
82/55
99.6/100
0/15
Other_SAR-Rfil_20605_OG5932
Osmo_Pseudofungi-Hcat_3892_OG5932
Other_Diaphoretickes-Bmot_7430_OG5932
100/100
Cp_Diaphoretickes-Chsp_16127_OG5932
84.9/77
Other_Other-Sspe_40254_OG5932
94.2/89
Bacteria-UP_b000008922_1255_OG5932
99.4/100
Bacteria-UP_b000003380_193_OG5932
82.1/76
Other_Amorphea-Ttra_5232_OG5932
0.3

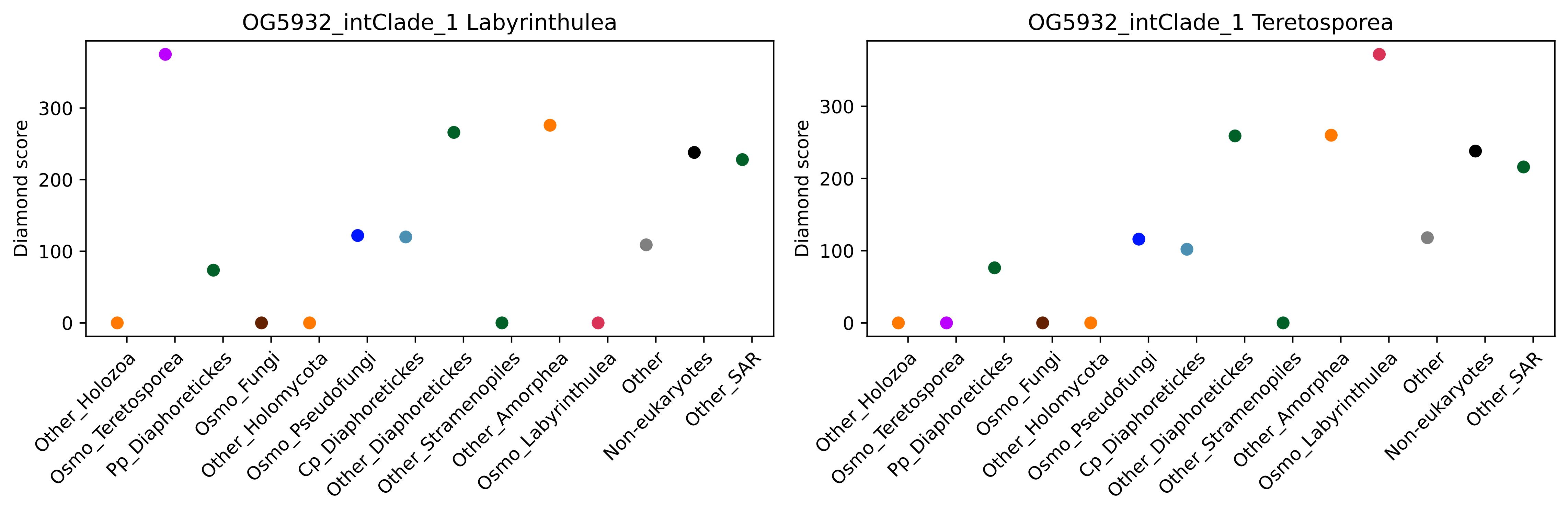
OG5932_intClade_1 Labyrinthulea
Diamond score
0
100
200
300
Other_Holozoa
Osmo_Teretosporea
Pp_Diaphoretickes
Osmo_Fungi
Other_Holomycota
Osmo_Pseudofungi
Cp_Diaphoretickes
Other_Diaphoretickes
Other_Stramenopiles
Other_Amorphea
Osmo_Labyrinthulea
Other
Non-eukaryotes
Other_SAR
OG5932_intClade_1 Teretosporea
Diamond score
0
100
200
300
Other_Holozoa
Osmo_Teretosporea
Pp_Diaphoretickes
Osmo_Fungi
Other_Holomycota
Osmo_Pseudofungi
Cp_Diaphoretickes
Other_Diaphoretickes
Other_Stramenopiles
Other_Amorphea
Osmo_Labyrinthulea
Other
Non-eukaryotes
Other_SAR

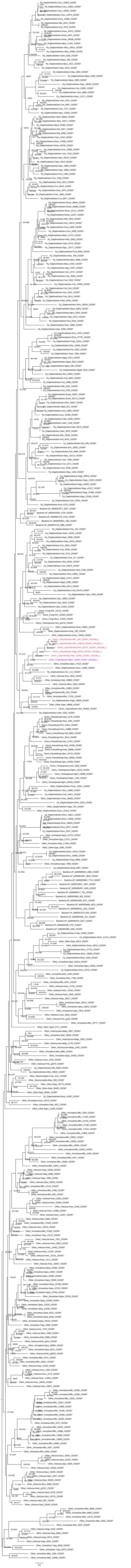

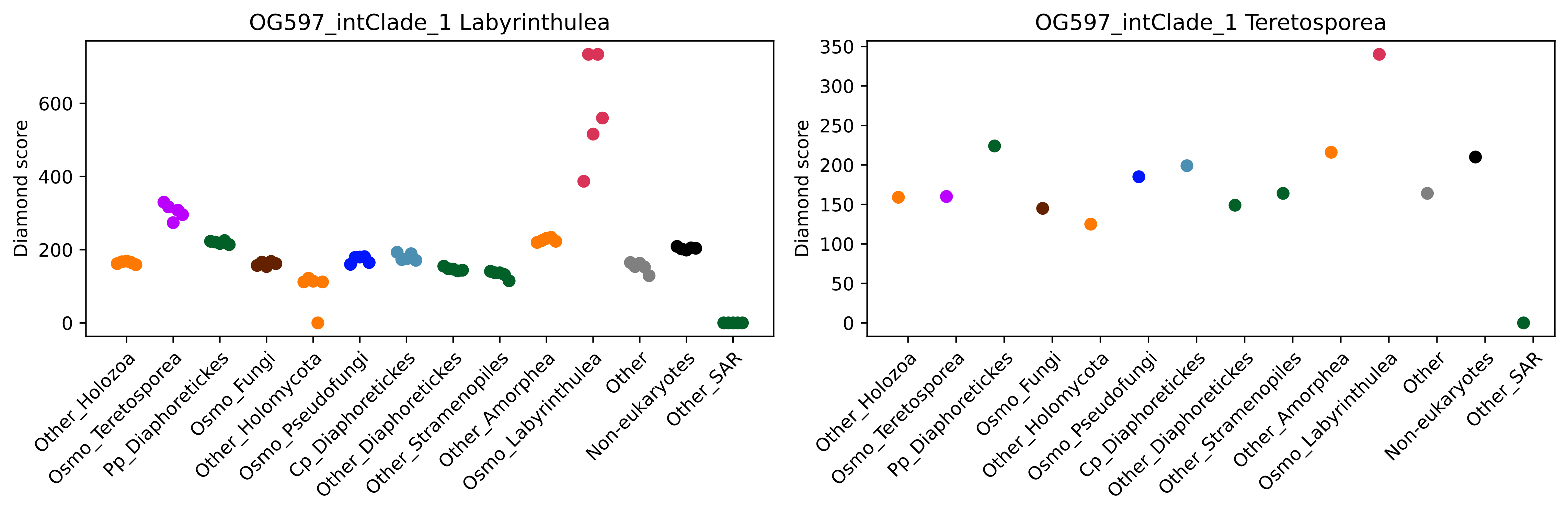
OG597_intClade_1 Labyrinthulea
Diamond score
0
200
400
600
Other_Holozoa
Osmo_Teretosporea
Pp_Diaphoretickes
Osmo_Fungi
Other_Holomycota
Osmo_Pseudofungi
Cp_Diaphoretickes
Other_Diaphoretickes
Other_Stramenopiles
Other_Amorphea
Osmo_Labyrinthulea
Other
Non-eukaryotes
Other_SAR
OG597_intClade_1 Teretosporea
Diamond score
0
50
100
150
200
250
300
350
Other_Holozoa
Osmo_Teretosporea
Pp_Diaphoretickes
Osmo_Fungi
Other_Holomycota
Osmo_Pseudofungi
Cp_Diaphoretickes
Other_Diaphoretickes
Other_Stramenopiles
Other_Amorphea
Osmo_Labyrinthulea
Other
Non-eukaryotes
Other_SAR

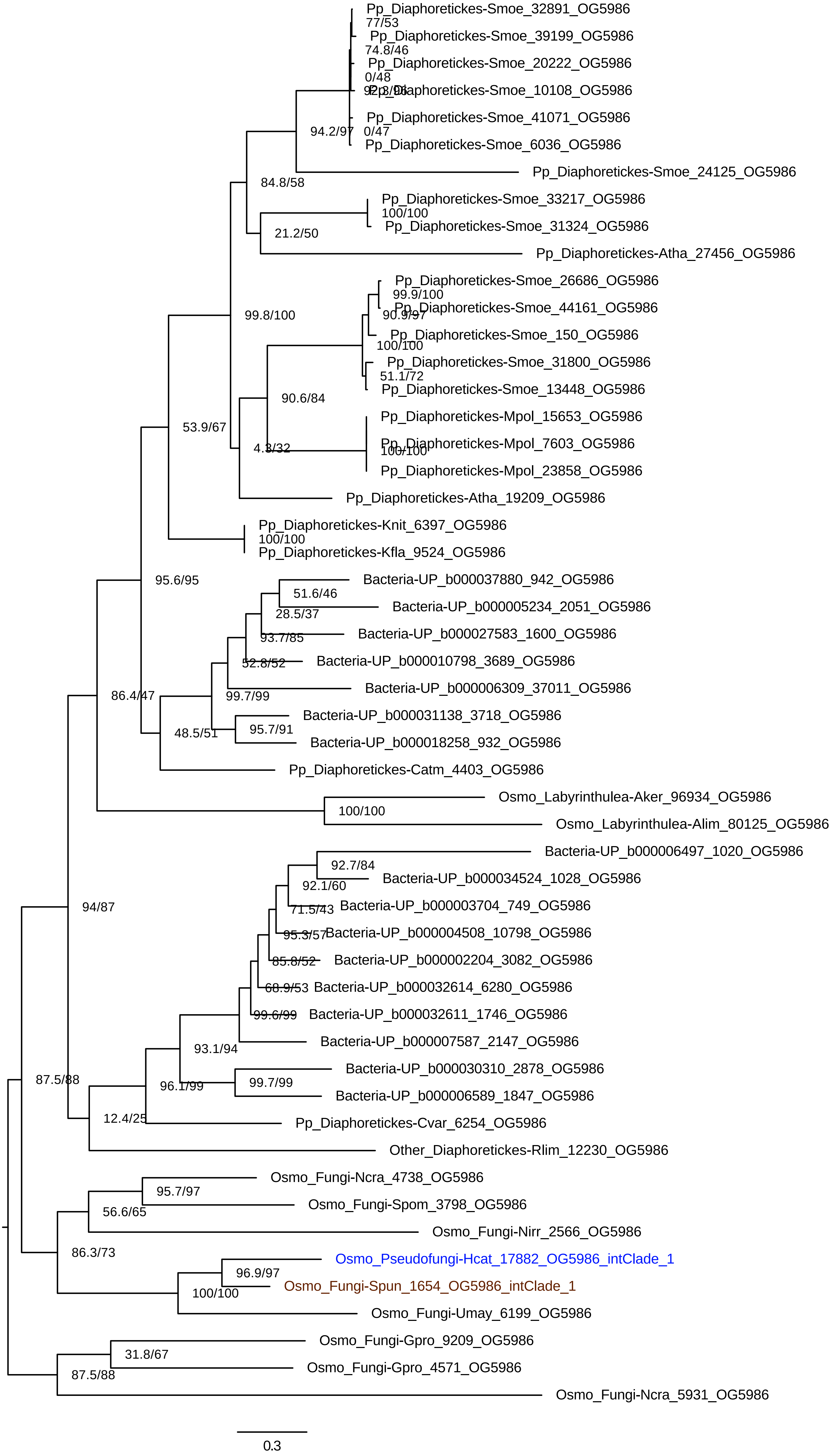

Pp_Diaphoretickes-Smoe_32891_OG5986
77/53
Pp_Diaphoretickes-Smoe_39199_OG5986
74.8/46
Pp_Diaphoretickes-Smoe_20222_OG5986
0/48
Pp_Diaphoretickes-Smoe_10108_OG5986
Pp_Diaphoretickes-Smoe_41071_OG5986
94.2/97
0/47
Pp_Diaphoretickes-Smoe_6036_OG5986
Pp_Diaphoretickes-Smoe_24125_OG5986
84.8/58
Pp_Diaphoretickes-Smoe_33217_OG5986
100/100
Pp_Diaphoretickes-Smoe_31324_OG5986
21.2/50
Pp_Diaphoretickes-Atha_27456_OG5986
Pp_Diaphoretickes-Smoe_26686_OG5986
99.9/100
Pp_Diaphoretickes-Smoe_44161_OG5986
99.8/100
90.9/97
Pp_Diaphoretickes-Smoe_150_OG5986
100/100
Pp_Diaphoretickes-Smoe_31800_OG5986
51.1/72
Pp_Diaphoretickes-Smoe_13448_OG5986
90.6/84
Pp_Diaphoretickes-Mpol_15653_OG5986
53.9/67
Pp_Diaphoretickes-Mpol_7603_OG5986
4.3/32
100/100
Pp_Diaphoretickes-Mpol_23858_OG5986
Pp_Diaphoretickes-Atha_19209_OG5986
Pp_Diaphoretickes-Knit_6397_OG5986
100/100
Pp_Diaphoretickes-Kfla_9524_OG5986
95.6/95
Bacteria-UP_b000037880_942_OG5986
51.6/46
Bacteria-UP_b000005234_2051_OG5986
28.5/37
Bacteria-UP_b000027583_1600_OG5986
93.7/85
Bacteria-UP_b000010798_3689_OG5986
52.8/52
Bacteria-UP_b000006309_37011_OG5986
86.4/47
99.7/99
Bacteria-UP_b000031138_3718_OG5986
95.7/91
48.5/51
Bacteria-UP_b000018258_932_OG5986
Pp_Diaphoretickes-Catm_4403_OG5986
Osmo_Labyrinthulea-Aker_96934_OG5986
100/100
Osmo_Labyrinthulea-Alim_80125_OG5986
Bacteria-UP_b000006497_1020_OG5986
92.7/84
Bacteria-UP_b000034524_1028_OG5986
92.1/60
94/87
Bacteria-UP_b000003704_749_OG5986
71.5/43
Bacteria-UP_b000004508_10798_OG5986
95.3/57
Bacteria-UP_b000002204_3082_OG5986
85.8/52
Bacteria-UP_b000032614_6280_OG5986
68.9/53
Bacteria-UP_b000032611_1746_OG5986
99.6/99
Bacteria-UP_b000007587_2147_OG5986
93.1/94
Bacteria-UP_b000030310_2878_OG5986
87.5/88
99.7/99
96.1/99
Bacteria-UP_b000006589_1847_OG5986
Pp_Diaphoretickes-Cvar_6254_OG5986
12.4/25
Other_Diaphoretickes-Rlim_12230_OG5986
Osmo_Fungi-Ncra_4738_OG5986
95.7/97
Osmo_Fungi-Spom_3798_OG5986
56.6/65
Osmo_Fungi-Nirr_2566_OG5986
86.3/73
Osmo_Pseudofungi-Hcat_17882_OG5986_intClade_1
96.9/97
Osmo_Fungi-Spun_1654_OG5986_intClade_1
100/100
Osmo_Fungi-Umay_6199_OG5986
Osmo_Fungi-Gpro_9209_OG5986
31.8/67
Osmo_Fungi-Gpro_4571_OG5986
87.5/88
Osmo_Fungi-Ncra_5931_OG5986
0.3

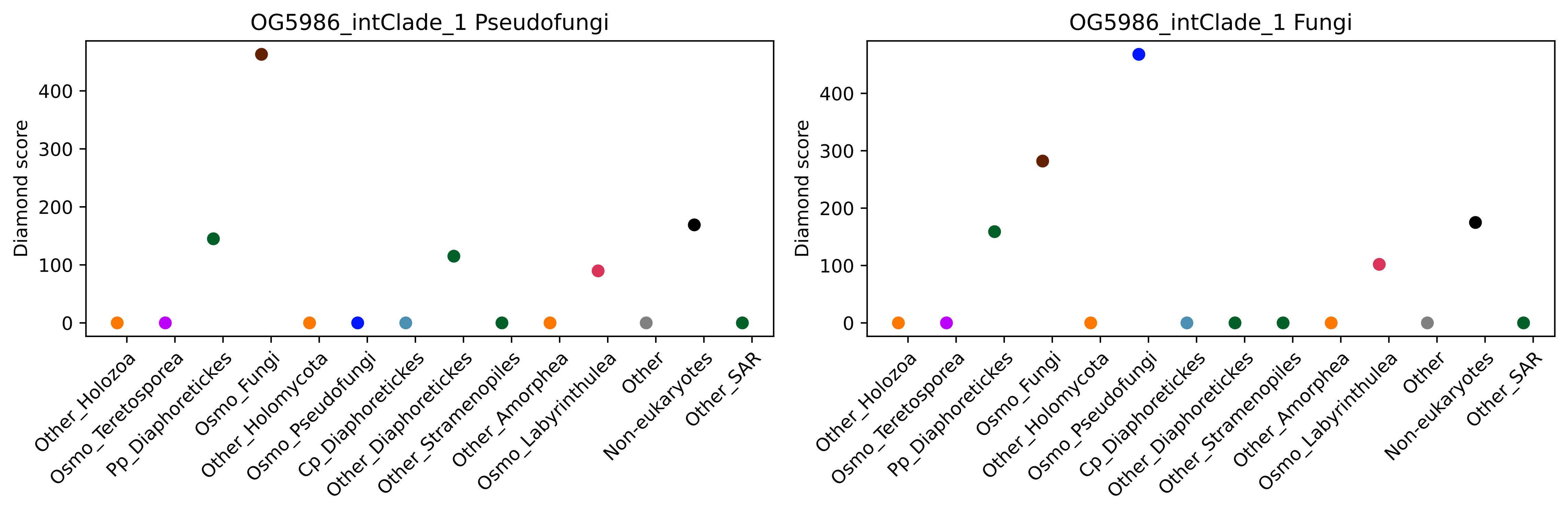

OG5986_intClade_1 Pseudofungi
Diamond score
0
100
200
300
400
Other_Holozoa
Osmo_Teretosporea
Pp_Diaphoretickes
Osmo_Fungi
Other_Holomycota
Osmo_Pseudofungi
Cp_Diaphoretickes
Other_Diaphoretickes
Other_Stramenopiles
Other_Amorphea
Osmo_Labyrinthulea
Other
Non-eukaryotes
Other_SAR
OG5986_intClade_1 Fungi
Diamond score
0
100
200
300
400
Other_Holozoa
Osmo_Teretosporea
Pp_Diaphoretickes
Osmo_Fungi
Other_Holomycota
Osmo_Pseudofungi
Cp_Diaphoretickes
Other_Diaphoretickes
Other_Stramenopiles
Other_Amorphea
Osmo_Labyrinthulea
Other
Non-eukaryotes
Other_SAR

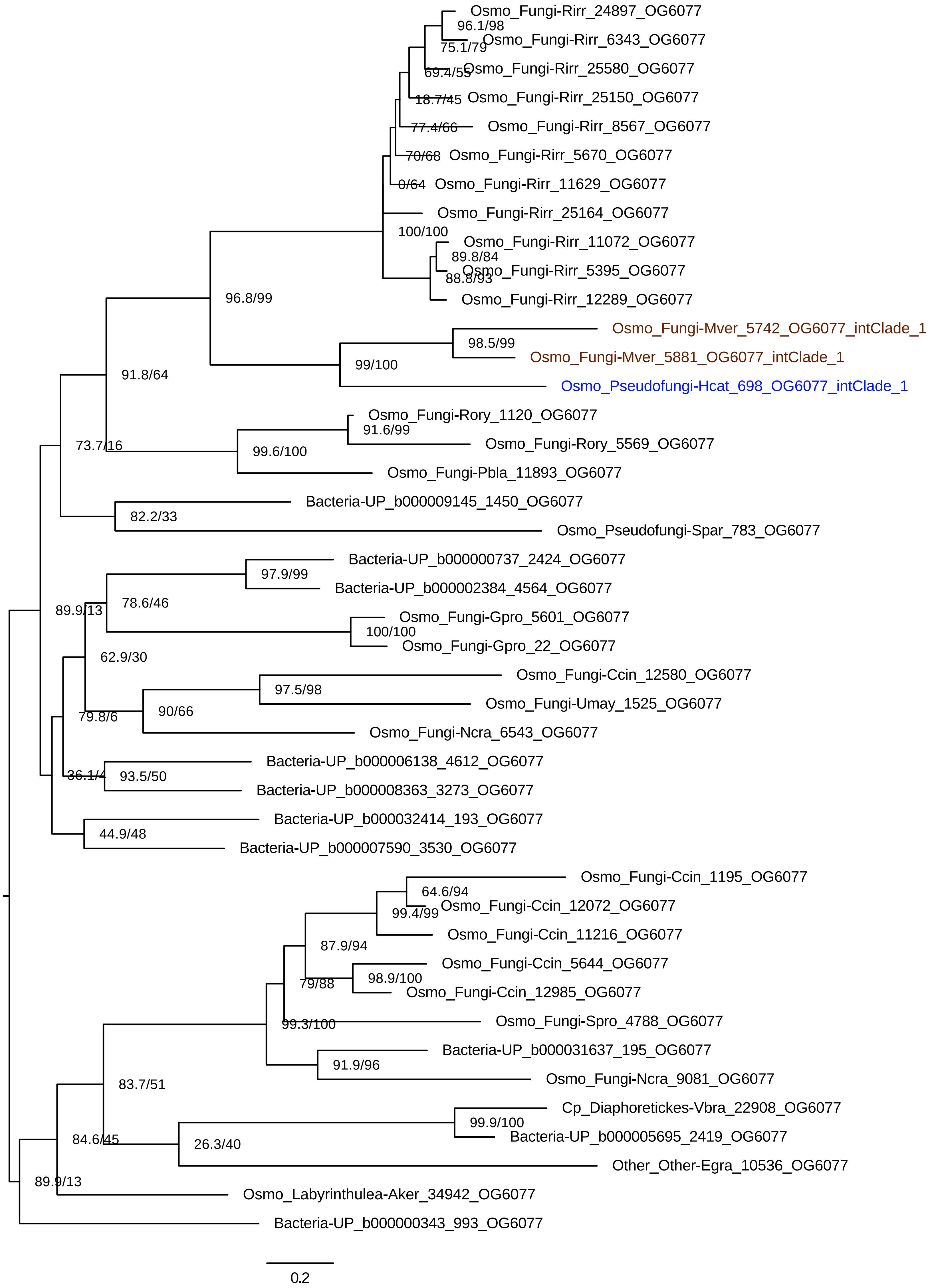

Osmo_Fungi-Rirr_24897_OG6077
96.1/98
Osmo_Fungi-Rirr_6343_OG6077
75.1/79
69.4/55
Osmo_Fungi-Rirr_25580_OG6077
18.7/45
Osmo_Fungi-Rirr_25150_OG6077
77.4/66
Osmo_Fungi-Rirr_8567_OG6077
70/68
Osmo_Fungi-Rirr_5670_OG6077
0/64
Osmo_Fungi-Rirr_11629_OG6077
Osmo_Fungi-Rirr_25164_OG6077
100/100
Osmo_Fungi-Rirr_11072_OG6077
89.8/84
Osmo_Fungi-Rirr_5395_OG6077
88.8/93
Osmo_Fungi-Rirr_12289_OG6077
96.8/99
Osmo_Fungi-Mver_5742_OG6077_intClade_1
98.5/99
Osmo_Fungi-Mver_5881_OG6077_intClade_1
99/100
Osmo_Pseudofungi-Hcat_698_OG6077_intClade_1
91.8/64
Osmo_Fungi-Rory_1120_OG6077
91.6/99
Osmo_Fungi-Rory_5569_OG6077
73.7/16
99.6/100
Osmo_Fungi-Pbla_11893_OG6077
Bacteria-UP_b000009145_1450_OG6077
82.2/33
Osmo_Pseudofungi-Spar_783_OG6077
Bacteria-UP_b000000737_2424_OG6077
97.9/99
Bacteria-UP_b000002384_4564_OG6077
89.9/13
78.6/46
Osmo_Fungi-Gpro_5601_OG6077
100/100
Osmo_Fungi-Gpro_22_OG6077
62.9/30
Osmo_Fungi-Ccin_12580_OG6077
97.5/98
Osmo_Fungi-Umay_1525_OG6077
79.8/6
90/66
Osmo_Fungi-Ncra_6543_OG6077
Bacteria-UP_b000006138_4612_OG6077
36.1/4
93.5/50
Bacteria-UP_b000008363_3273_OG6077
Bacteria-UP_b000032414_193_OG6077
44.9/48
Bacteria-UP_b000007590_3530_OG6077
Osmo_Fungi-Ccin_1195_OG6077
64.6/94
Osmo_Fungi-Ccin_12072_OG6077
99.4/99
Osmo_Fungi-Ccin_11216_OG6077
87.9/94
Osmo_Fungi-Ccin_5644_OG6077
98.9/100
79/88
Osmo_Fungi-Ccin_12985_OG6077
99.3/100
Osmo_Fungi-Spro_4788_OG6077
Bacteria-UP_b000031637_195_OG6077
91.9/96
Osmo_Fungi-Ncra_9081_OG6077
83.7/51
Cp_Diaphoretickes-Vbra_22908_OG6077
99.9/100
84.6/45
Bacteria-UP_b000005695_2419_OG6077
26.3/40
Other_Other-Egra_10536_OG6077
89.9/13
Osmo_Labyrinthulea-Aker_34942_OG6077
Bacteria-UP_b000000343_993_OG6077
0.2

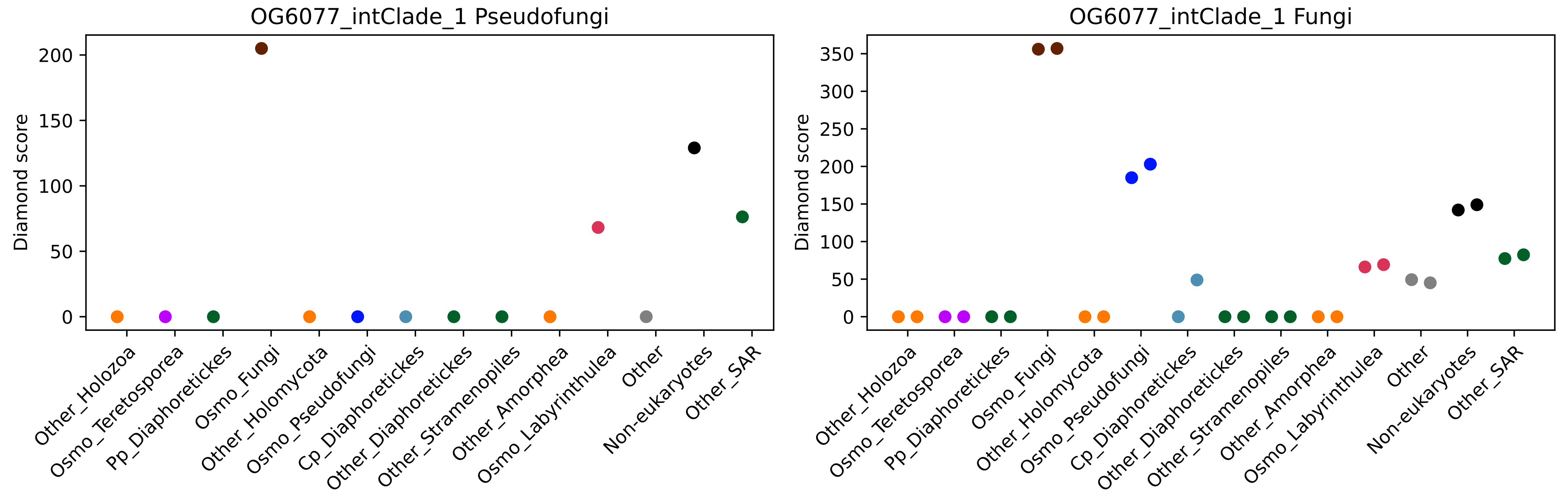
OG6077_intClade_1 Pseudofungi
OG6077_intClade_1 Fungi
Diamond score
0
50
100
150
200
250
300
350
Other_Holozoa
Osmo_Teretosporea
Pp_Diaphoretickes
Osmo_Fungi
Other_Holomycota
Osmo_Pseudofungi
Cp_Diaphoretickes
Other_Diaphoretickes
Other_Stramenopiles
Other_Amorphea
Osmo_Labyrinthulea
Other
Non-eukaryotes
Other_SAR

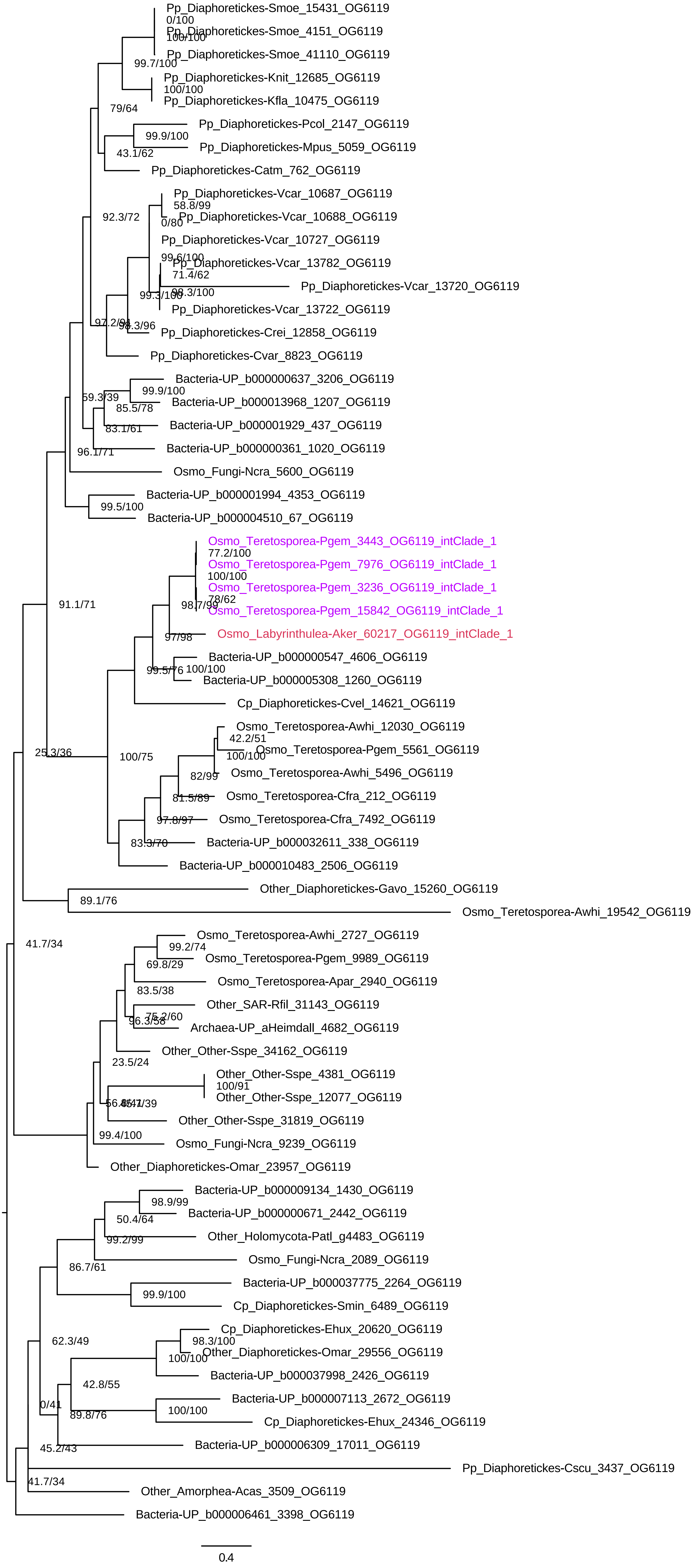
Pp_Diaphoretickes-Smoe_15431_OG6119
0/100
Pp_Diaphoretickes-Smoe_4151_OG6119
100/100
Pp_Diaphoretickes-Smoe_41110_OG6119
99.7/100
Pp_Diaphoretickes-Knit_12685_OG6119
100/100
Pp_Diaphoretickes-Kfla_10475_OG6119
79/64
Pp_Diaphoretickes-Pcol_2147_OG6119
99.9/100
Pp_Diaphoretickes-Mpus_5059_OG6119
43.1/62
Pp_Diaphoretickes-Catm_762_OG6119
Pp_Diaphoretickes-Vcar_10687_OG6119
58.8/99
92.3/72
0/80
Pp_Diaphoretickes-Vcar_10688_OG6119
Pp_Diaphoretickes-Vcar_10727_OG6119
99.6/100
Pp_Diaphoretickes-Vcar_13782_OG6119
71.4/62
Pp_Diaphoretickes-Vcar_13720_OG6119
99.3/100
98.3/100
Pp_Diaphoretickes-Vcar_13722_OG6119
97.2/91
98.3/96
Pp_Diaphoretickes-Crei_12858_OG6119
Pp_Diaphoretickes-Cvar_8823_OG6119
Bacteria-UP_b000000637_3206_OG6119
99.9/100
59.3/39
85.5/78
Bacteria-UP_b000013968_1207_OG6119
Bacteria-UP_b000001929_437_OG6119
83.1/61
Bacteria-UP_b000000361_1020_OG6119
96.1/71
Osmo_Fungi-Ncra_5600_OG6119
Bacteria-UP_b000001994_4353_OG6119
99.5/100
Bacteria-UP_b000004510_67_OG6119
Osmo_Teretosporea-Pgem_3443_OG6119_intClade_1
77.2/100
Osmo_Teretosporea-Pgem_7976_OG6119_intClade_1
100/100
Osmo_Teretosporea-Pgem_3236_OG6119_intClade_1
91.1/71
98.7/99
78/62
Osmo_Teretosporea-Pgem_15842_OG6119_intClade_1
Osmo_Labyrinthulea-Aker_60217_OG6119_intClade_1
97/98
Bacteria-UP_b000000547_4606_OG6119
99.5/76
100/100
Bacteria-UP_b000005308_1260_OG6119
Cp_Diaphoretickes-Cvel_14621_OG6119
Osmo_Teretosporea-Awhi_12030_OG6119
42.2/51
100/100
Osmo_Teretosporea-Pgem_5561_OG6119
25.3/36
100/75
82/99
Osmo_Teretosporea-Awhi_5496_OG6119
81.5/89
Osmo_Teretosporea-Cfra_212_OG6119
97.8/97
Osmo_Teretosporea-Cfra_7492_OG6119
83.3/70
Bacteria-UP_b000032611_338_OG6119
Bacteria-UP_b000010483_2506_OG6119
Other_Diaphoretickes-Gavo_15260_OG6119
89.1/76
Osmo_Teretosporea-Awhi_19542_OG6119
Osmo_Teretosporea-Awhi_2727_OG6119
41.7/34
99.2/74
Osmo_Teretosporea-Pgem_9989_OG6119
69.8/29
Osmo_Teretosporea-Apar_2940_OG6119
83.5/38
Other_SAR-Rfil_31143_OG6119
75.2/60
96.3/38
Archaea-UP_aHeimdall_4682_OG6119
Other_Other-Sspe_34162_OG6119
23.5/24
Other_Other-Sspe_4381_OG6119
100/91
56.8/47
85.1/39
Other_Other-Sspe_12077_OG6119
Other_Other-Sspe_31819_OG6119
99.4/100
Osmo_Fungi-Ncra_9239_OG6119
Other_Diaphoretickes-Omar_23957_OG6119
Bacteria-UP_b000009134_1430_OG6119
98.9/99
50.4/64
Bacteria-UP_b000000671_2442_OG6119
Other_Holomycota-Patl_g4483_OG6119
99.2/99
Osmo_Fungi-Ncra_2089_OG6119
86.7/61
Bacteria-UP_b000037775_2264_OG6119
99.9/100
Cp_Diaphoretickes-Smin_6489_OG6119
Cp_Diaphoretickes-Ehux_20620_OG6119
62.3/49
98.3/100
100/100
Other_Diaphoretickes-Omar_29556_OG6119
Bacteria-UP_b000037998_2426_OG6119
42.8/55
Bacteria-UP_b000007113_2672_OG6119
0/41
89.8/76
100/100
Cp_Diaphoretickes-Ehux_24346_OG6119
45.2/43
Bacteria-UP_b000006309_17011_OG6119
Pp_Diaphoretickes-Cscu_3437_OG6119
41.7/34
Other_Amorphea-Acas_3509_OG6119
Bacteria-UP_b000006461_3398_OG6119
0.4

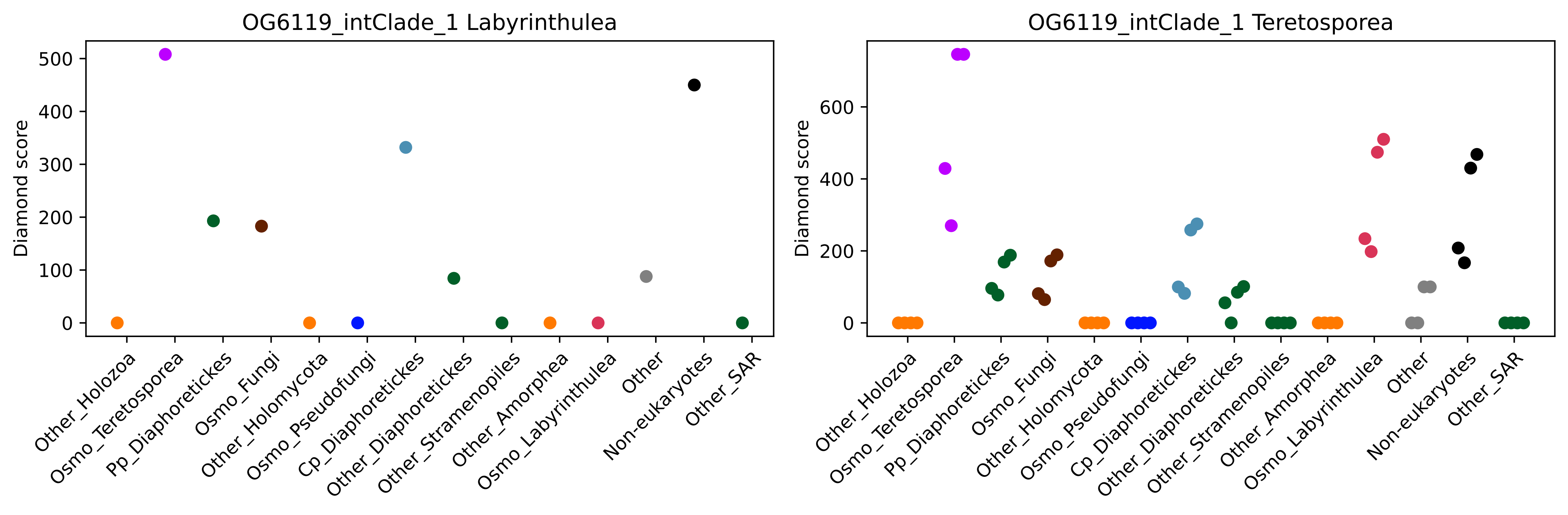

OG6119_intClade_1 Labyrinthulea
Diamond score
0
100
200
300
400
500
Other_Holozoa
Osmo_Teretosporea
Pp_Diaphoretickes
Osmo_Fungi
Other_Holomycota
Osmo_Pseudofungi
Cp_Diaphoretickes
Other_Diaphoretickes
Other_Stramenopiles
Other_Amorphea
Osmo_Labyrinthulea
Other
Non-eukaryotes
Other_SAR
OG6119_intClade_1 Teretosporea
Diamond score
0
200
400
600
Other_Holozoa
Osmo_Teretosporea
Pp_Diaphoretickes
Osmo_Fungi
Other_Holomycota
Osmo_Pseudofungi
Cp_Diaphoretickes
Other_Diaphoretickes
Other_Stramenopiles
Other_Amorphea
Osmo_Labyrinthulea
Other
Non-eukaryotes
Other_SAR

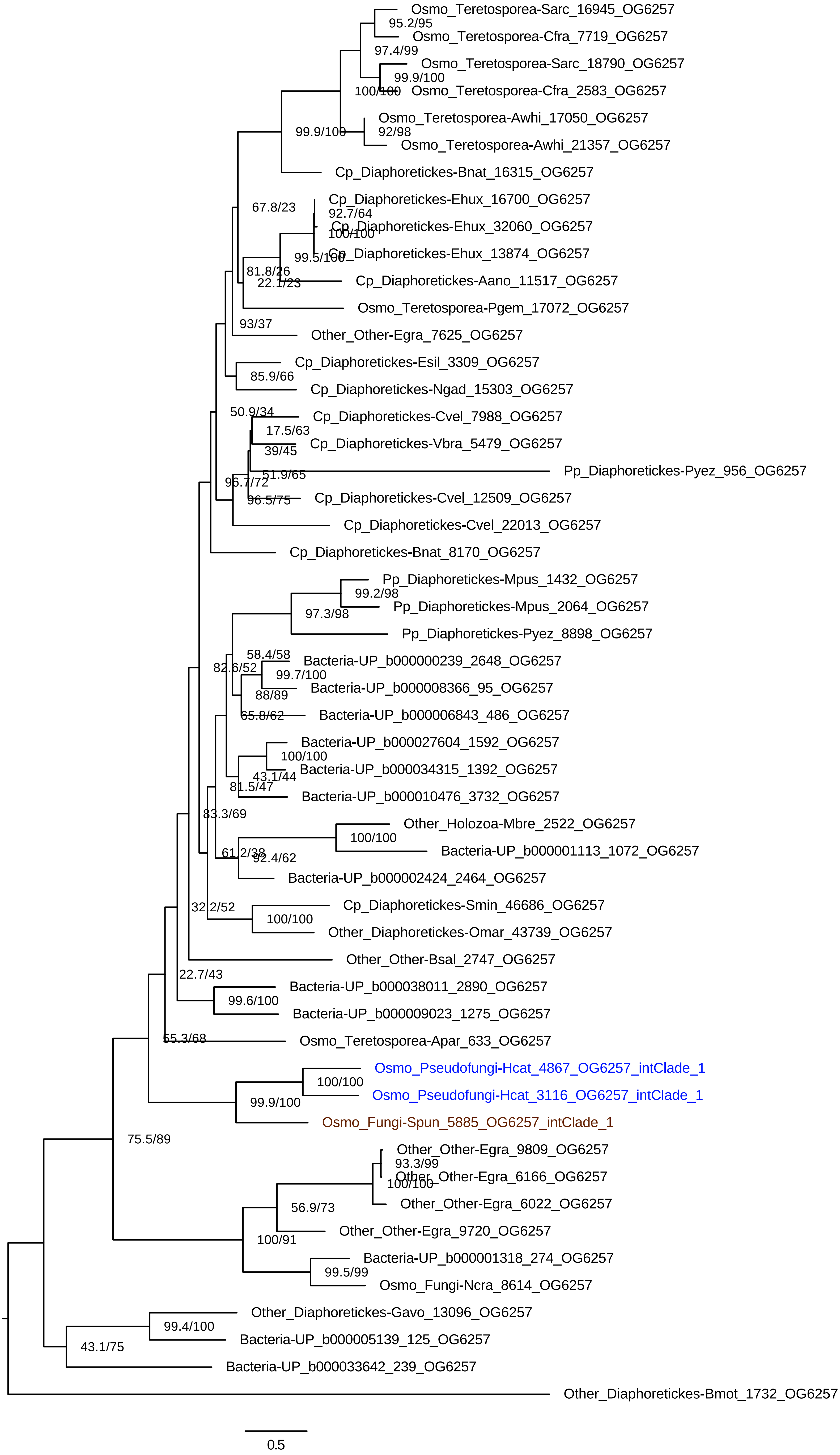
Osmo_Teretosporea-Sarc_16945_OG6257
95.2/95
Osmo_Teretosporea-Cfra_7719_OG6257
97.4/99
Osmo_Teretosporea-Sarc_18790_OG6257
99.9/100
100/100
Osmo_Teretosporea-Cfra_2583_OG6257
Osmo_Teretosporea-Awhi_17050_OG6257
99.9/100
92/98
Osmo_Teretosporea-Awhi_21357_OG6257
Cp_Diaphoretickes-Bnat_16315_OG6257
67.8/23
Cp_Diaphoretickes-Ehux_16700_OG6257
92.7/64
Cp_Diaphoretickes-Ehux_32060_OG6257
100/100
99.5/100
Cp_Diaphoretickes-Ehux_13874_OG6257
81.8/26
22.1/23
Cp_Diaphoretickes-Aano_11517_OG6257
Osmo_Teretosporea-Pgem_17072_OG6257
93/37
Other_Other-Egra_7625_OG6257
Cp_Diaphoretickes-Esil_3309_OG6257
85.9/66
Cp_Diaphoretickes-Ngad_15303_OG6257
50.9/34
Cp_Diaphoretickes-Cvel_7988_OG6257
17.5/63
Cp_Diaphoretickes-Vbra_5479_OG6257
39/45
Pp_Diaphoretickes-Pyez_956_OG6257
51.9/65
96.7/72
96.5/75
Cp_Diaphoretickes-Cvel_12509_OG6257
Cp_Diaphoretickes-Cvel_22013_OG6257
Cp_Diaphoretickes-Bnat_8170_OG6257
Pp_Diaphoretickes-Mpus_1432_OG6257
99.2/98
Pp_Diaphoretickes-Mpus_2064_OG6257
97.3/98
Pp_Diaphoretickes-Pyez_8898_OG6257
58.4/58
Bacteria-UP_b000000239_2648_OG6257
82.6/52
99.7/100
Bacteria-UP_b000008366_95_OG6257
88/89
65.8/62
Bacteria-UP_b000006843_486_OG6257
Bacteria-UP_b000027604_1592_OG6257
100/100
43.1/44
Bacteria-UP_b000034315_1392_OG6257
81.5/47
Bacteria-UP_b000010476_3732_OG6257
83.3/69
Other_Holozoa-Mbre_2522_OG6257
100/100
Bacteria-UP_b000001113_1072_OG6257
61.2/38
92.4/62
Bacteria-UP_b000002424_2464_OG6257
32.2/52
Cp_Diaphoretickes-Smin_46686_OG6257
100/100
Other_Diaphoretickes-Omar_43739_OG6257
Other_Other-Bsal_2747_OG6257
22.7/43
Bacteria-UP_b000038011_2890_OG6257
99.6/100
Bacteria-UP_b000009023_1275_OG6257
55.3/68
Osmo_Teretosporea-Apar_633_OG6257
Osmo_Pseudofungi-Hcat_4867_OG6257_intClade_1
100/100
Osmo_Pseudofungi-Hcat_3116_OG6257_intClade_1
99.9/100
Osmo_Fungi-Spun_5885_OG6257_intClade_1
75.5/89
Other_Other-Egra_9809_OG6257
93.3/99
Other_Other-Egra_6166_OG6257
100/100
Other_Other-Egra_6022_OG6257
56.9/73
Other_Other-Egra_9720_OG6257
100/91
Bacteria-UP_b000001318_274_OG6257
99.5/99
Osmo_Fungi-Ncra_8614_OG6257
Other_Diaphoretickes-Gavo_13096_OG6257
99.4/100
Bacteria-UP_b000005139_125_OG6257
43.1/75
Bacteria-UP_b000033642_239_OG6257
Other_Diaphoretickes-Bmot_1732_OG6257
0.5

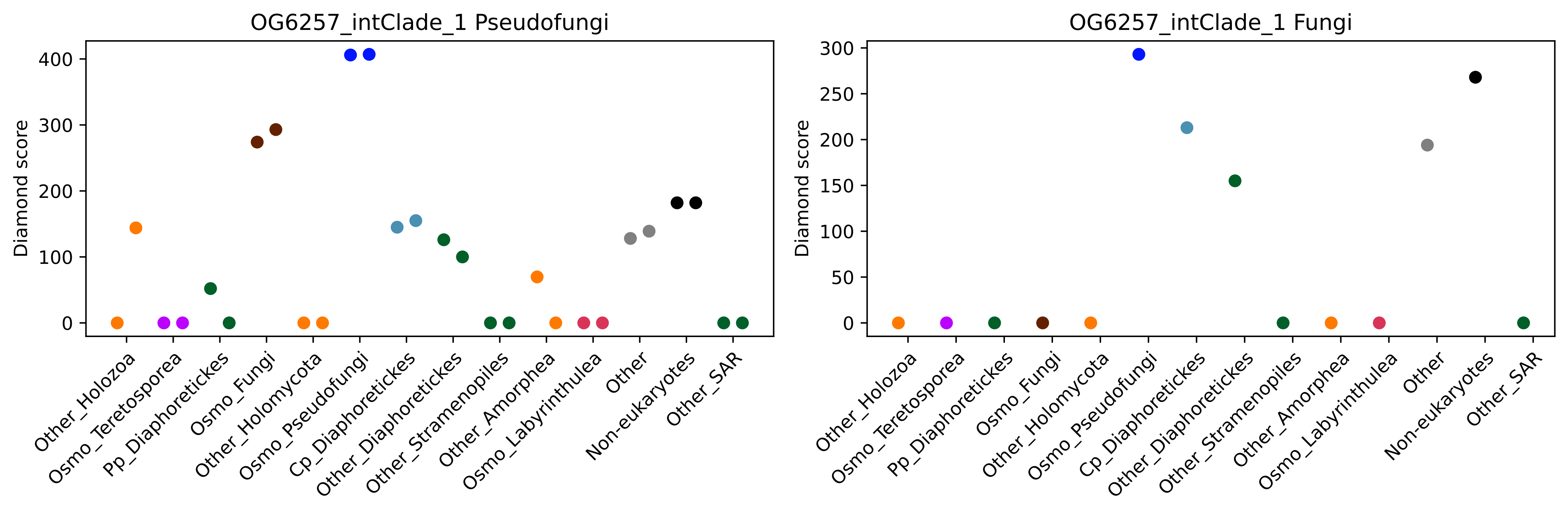
OG6257_intClade_1 Pseudofungi
OG6257_intClade_1 Fungi
Diamond score
Other_Holozoa
Osmo_Teretosporea
Pp_Diaphoretickes
Osmo_Fungi
Other_Holomycota
Osmo_Pseudofungi
Cp_Diaphoretickes
Other_Diaphoretickes
Other_Stramenopiles
Other_Amorphea
Osmo_Labyrinthulea
Other
Non-eukaryotes
Other_SAR

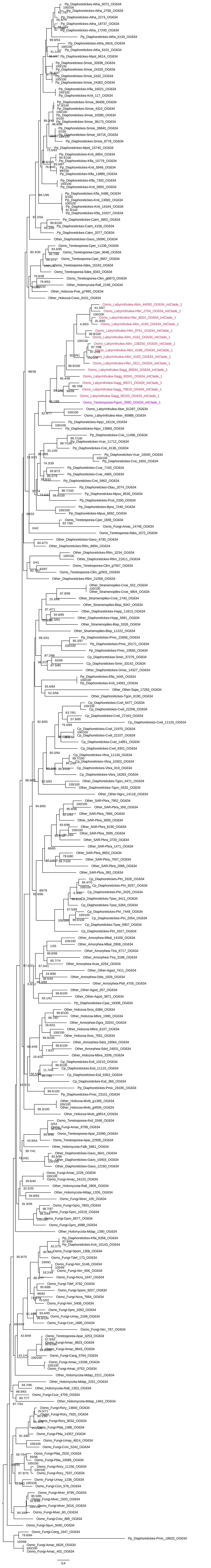

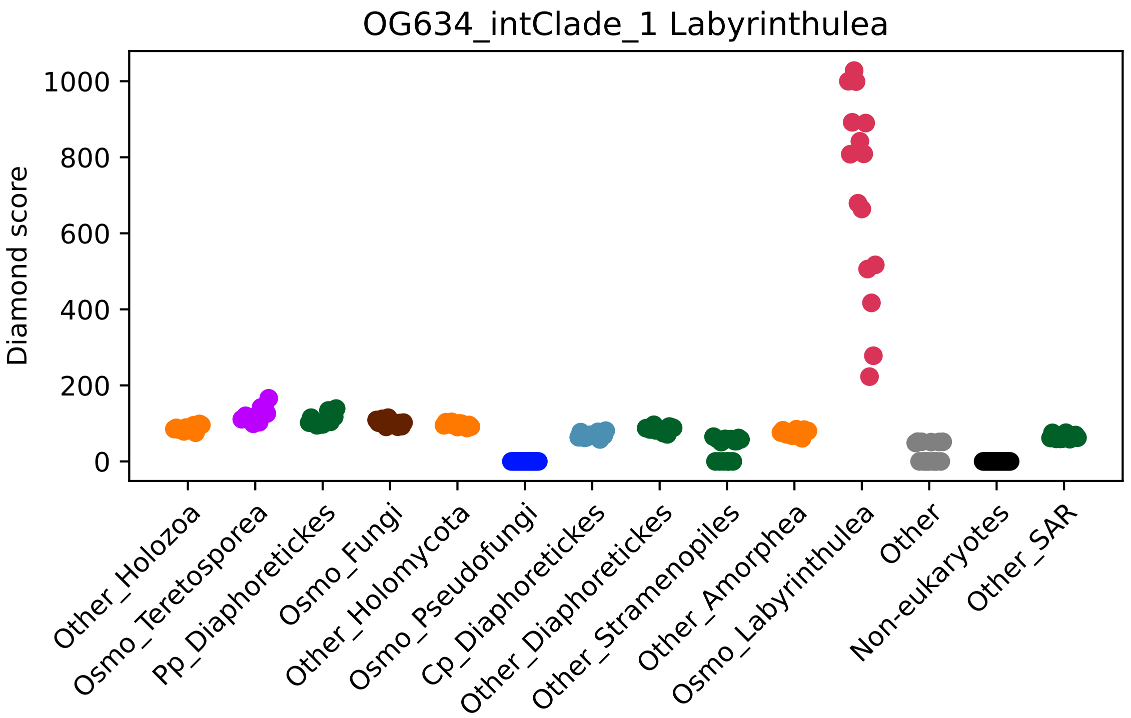

OG634_intClade_1 Labyrinthulea
Diamond score
0
200
400
600
800
1000
Other_Holozoa
Osmo_Teretosporea
Pp_Diaphoretickes
Osmo_Fungi
Other_Holomycota
Osmo_Pseudofungi
Cp_Diaphoretickes
Other_Diaphoretickes
Other_Stramenopiles
Other_Amorphea
Osmo_Labyrinthulea
Other
Non-eukaryotes
Other_SAR

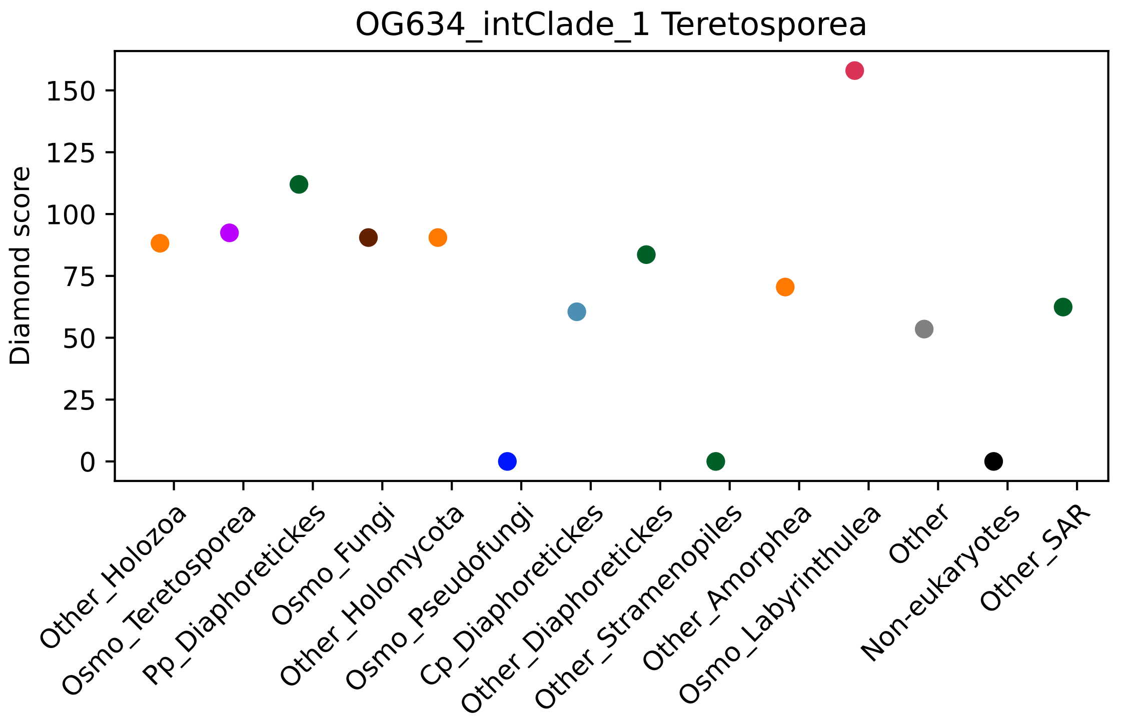

OG634_intClade_1 Teretosporea
Diamond score
0
25
50
75
100
125
150
Other_Holozoa
Osmo_Teretosporea
Pp_Diaphoretickes
Osmo_Fungi
Other_Holomycota
Osmo_Pseudofungi
Cp_Diaphoretickes
Other_Diaphoretickes
Other_Stramenopiles
Other_Amorphea
Osmo_Labyrinthulea
Other
Non-eukaryotes
Other_SAR

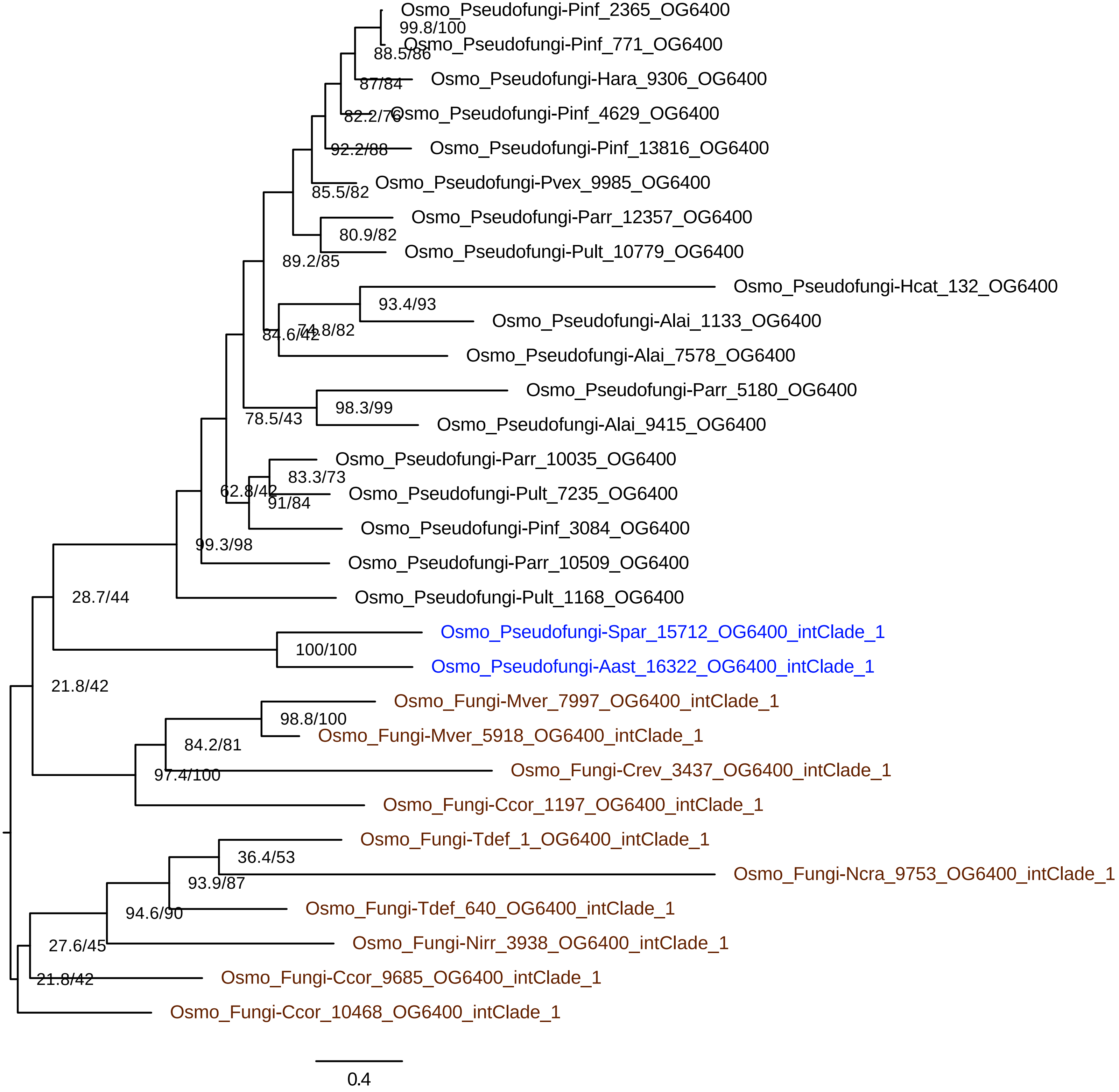

Osmo_Pseudofungi-Pinf_2365_OG6400
99.8/100
Osmo_Pseudofungi-Pinf_771_OG6400
88.5/86
Osmo_Pseudofungi-Hara_9306_OG6400
87/84
Osmo_Pseudofungi-Pinf_4629_OG6400
82.2/76
Osmo_Pseudofungi-Pinf_13816_OG6400
92.2/88
Osmo_Pseudofungi-Pvex_9985_OG6400
85.5/82
Osmo_Pseudofungi-Parr_12357_OG6400
80.9/82
Osmo_Pseudofungi-Pult_10779_OG6400
89.2/85
Osmo_Pseudofungi-Hcat_132_OG6400
93.4/93
Osmo_Pseudofungi-Alai_1133_OG6400
74.8/82
84.6/42
Osmo_Pseudofungi-Alai_7578_OG6400
Osmo_Pseudofungi-Parr_5180_OG6400
98.3/99
Osmo_Pseudofungi-Alai_9415_OG6400
78.5/43
Osmo_Pseudofungi-Parr_10035_OG6400
83.3/73
Osmo_Pseudofungi-Pult_7235_OG6400
62.8/42
91/84
Osmo_Pseudofungi-Pinf_3084_OG6400
99.3/98
Osmo_Pseudofungi-Parr_10509_OG6400
Osmo_Pseudofungi-Pult_1168_OG6400
28.7/44
Osmo_Pseudofungi-Spar_15712_OG6400_intClade_1
100/100
Osmo_Pseudofungi-Aast_16322_OG6400_intClade_1
21.8/42
Osmo_Fungi-Mver_7997_OG6400_intClade_1
98.8/100
Osmo_Fungi-Mver_5918_OG6400_intClade_1
84.2/81
Osmo_Fungi-Crev_3437_OG6400_intClade_1
97.4/100
Osmo_Fungi-Ccor_1197_OG6400_intClade_1
Osmo_Fungi-Tdef_1_OG6400_intClade_1
36.4/53
Osmo_Fungi-Ncra_9753_OG6400_intClade_1
93.9/87
Osmo_Fungi-Tdef_640_OG6400_intClade_1
94.6/90
Osmo_Fungi-Nirr_3938_OG6400_intClade_1
27.6/45
Osmo_Fungi-Ccor_9685_OG6400_intClade_1
21.8/42
Osmo_Fungi-Ccor_10468_OG6400_intClade_1
0.4

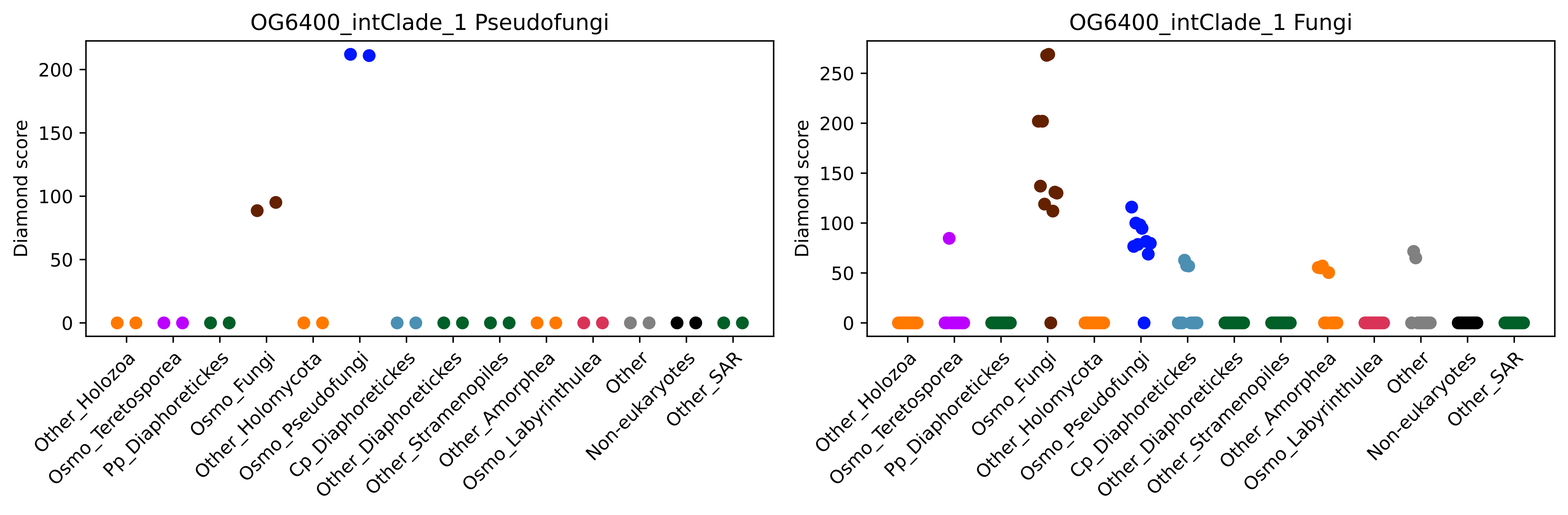
OG6400_intClade_1 Pseudofungi
OG6400_intClade_1 Fungi
Diamond score
0
50
100
150
200
250
Other_Holozoa
Osmo_Teretosporea
Pp_Diaphoretickes
Osmo_Fungi
Other_Holomycota
Osmo_Pseudofungi
Cp_Diaphoretickes
Other_Diaphoretickes
Other_Stramenopiles
Other_Amorphea
Osmo_Labyrinthulea
Other
Non-eukaryotes
Other_SAR

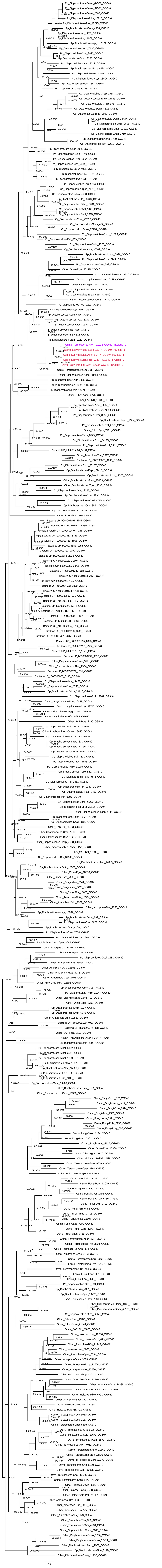

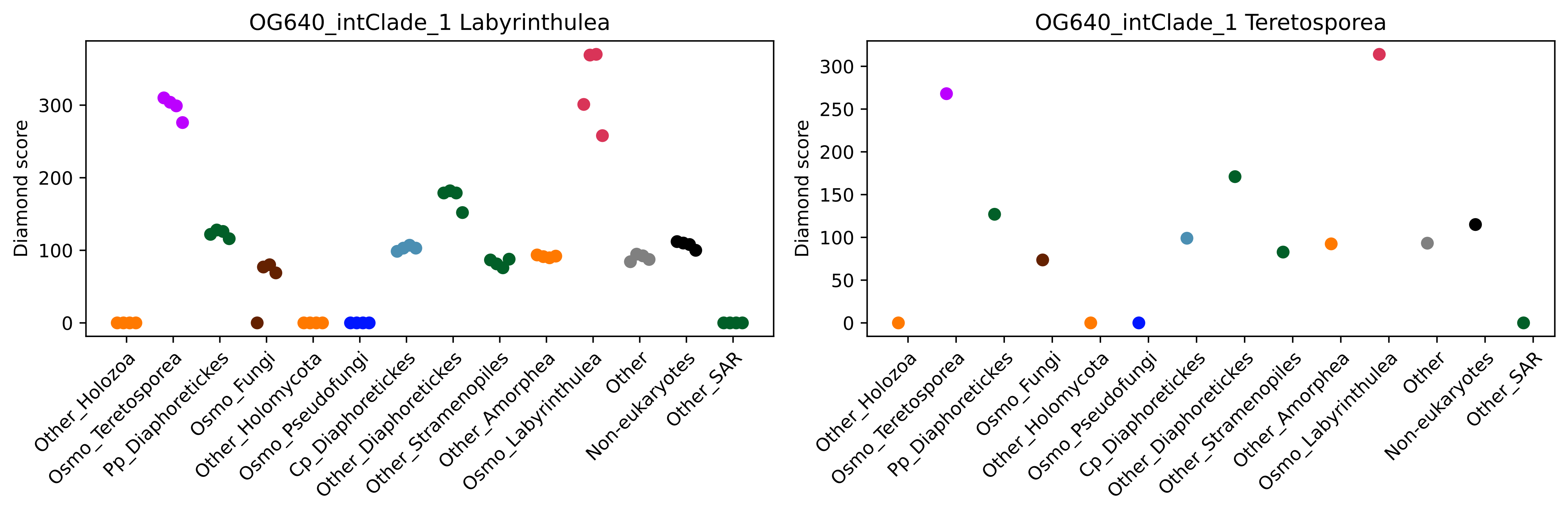
OG640_intClade_1 Labyrinthulea
OG640_intClade_1 Teretosporea
Diamond score
0
100
200
300
0
50
100
150
200
250
300
Other_Holozoa
Osmo_Teretosporea
Pp_Diaphoretickes
Osmo_Fungi
Other_Holomycota
Osmo_Pseudofungi
Cp_Diaphoretickes
Other_Diaphoretickes
Other_Stramenopiles
Other_Amorphea
Osmo_Labyrinthulea
Other
Non-eukaryotes
Other_SAR

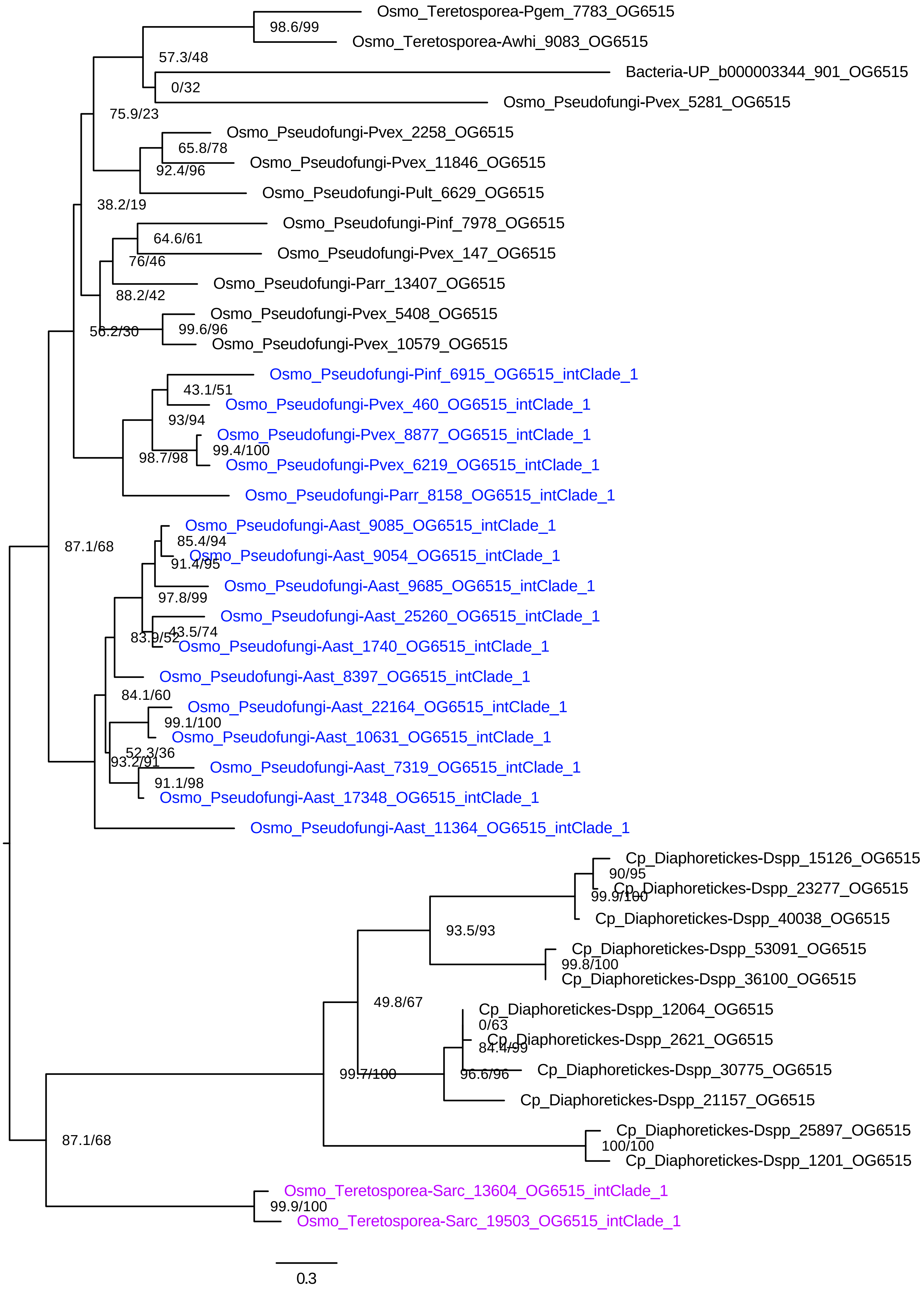

Osmo_Teretosporea-Pgem_7783_OG6515
98.6/99
Osmo_Teretosporea-Awhi_9083_OG6515
57.3/48
Bacteria-UP_b000003344_901_OG6515
0/32
Osmo_Pseudofungi-Pvex_5281_OG6515
75.9/23
Osmo_Pseudofungi-Pvex_2258_OG6515
65.8/78
Osmo_Pseudofungi-Pvex_11846_OG6515
92.4/96
Osmo_Pseudofungi-Pult_6629_OG6515
38.2/19
Osmo_Pseudofungi-Pinf_7978_OG6515
64.6/61
Osmo_Pseudofungi-Pvex_147_OG6515
76/46
Osmo_Pseudofungi-Parr_13407_OG6515
88.2/42
Osmo_Pseudofungi-Pvex_5408_OG6515
56.2/30
99.6/96
Osmo_Pseudofungi-Pvex_10579_OG6515
Osmo_Pseudofungi-Pinf_6915_OG6515_intClade_1
43.1/51
Osmo_Pseudofungi-Pvex_460_OG6515_intClade_1
93/94
Osmo_Pseudofungi-Pvex_8877_OG6515_intClade_1
99.4/100
98.7/98
Osmo_Pseudofungi-Pvex_6219_OG6515_intClade_1
Osmo_Pseudofungi-Parr_8158_OG6515_intClade_1
Osmo_Pseudofungi-Aast_9085_OG6515_intClade_1
85.4/94
87.1/68
Osmo_Pseudofungi-Aast_9054_OG6515_intClade_1
91.4/95
Osmo_Pseudofungi-Aast_9685_OG6515_intClade_1
97.8/99
Osmo_Pseudofungi-Aast_25260_OG6515_intClade_1
43.5/74
83.9/52
Osmo_Pseudofungi-Aast_1740_OG6515_intClade_1
Osmo_Pseudofungi-Aast_8397_OG6515_intClade_1
84.1/60
Osmo_Pseudofungi-Aast_22164_OG6515_intClade_1
99.1/100
Osmo_Pseudofungi-Aast_10631_OG6515_intClade_1
52.3/36
93.2/91
Osmo_Pseudofungi-Aast_7319_OG6515_intClade_1
91.1/98
Osmo_Pseudofungi-Aast_17348_OG6515_intClade_1
Osmo_Pseudofungi-Aast_11364_OG6515_intClade_1
Cp_Diaphoretickes-Dspp_15126_OG6515
90/95
Cp_Diaphoretickes-Dspp_23277_OG6515
99.9/100
Cp_Diaphoretickes-Dspp_40038_OG6515
93.5/93
Cp_Diaphoretickes-Dspp_53091_OG6515
99.8/100
Cp_Diaphoretickes-Dspp_36100_OG6515
49.8/67
Cp_Diaphoretickes-Dspp_12064_OG6515
0/63
Cp_Diaphoretickes-Dspp_2621_OG6515
84.4/99
99.7/100
96.6/96
Cp_Diaphoretickes-Dspp_30775_OG6515
Cp_Diaphoretickes-Dspp_21157_OG6515
87.1/68
Cp_Diaphoretickes-Dspp_25897_OG6515
100/100
Cp_Diaphoretickes-Dspp_1201_OG6515
Osmo_Teretosporea-Sarc_13604_OG6515_intClade_1
99.9/100
Osmo_Teretosporea-Sarc_19503_OG6515_intClade_1
0.3

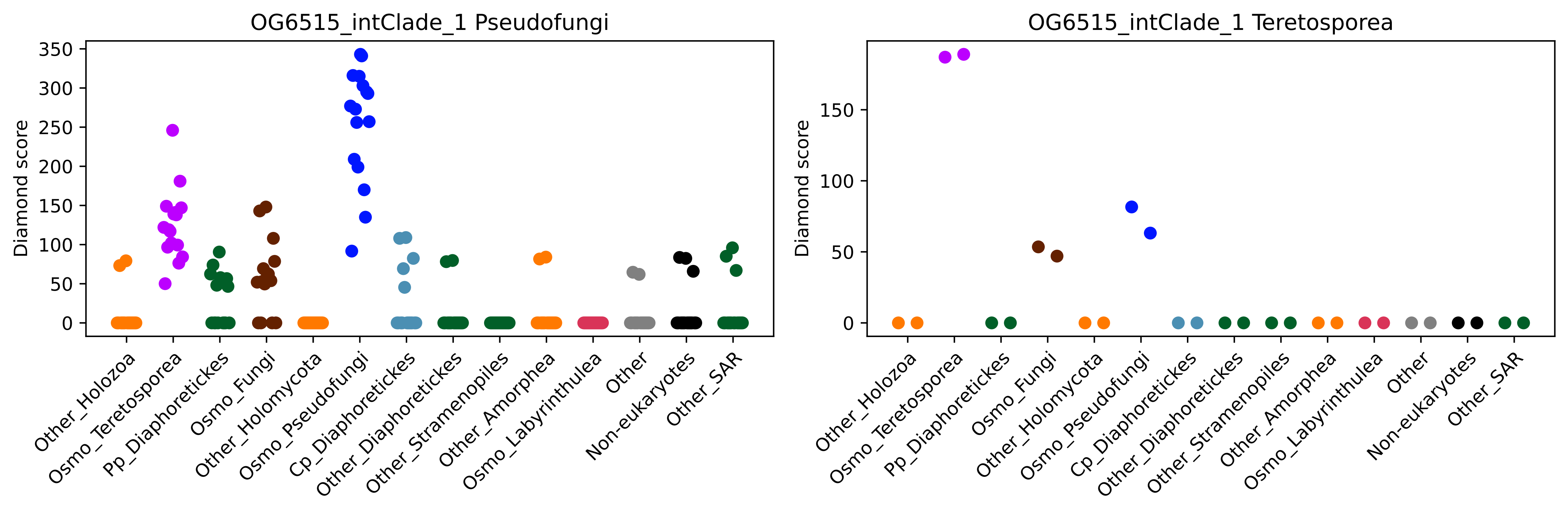

OG6515_intClade_1 Pseudofungi
OG6515_intClade_1 Teretosporea
Diamond score
Other_Holozoa
Osmo_Teretosporea
Pp_Diaphoretickes
Osmo_Fungi
Other_Holomycota
Osmo_Pseudofungi
Cp_Diaphoretickes
Other_Diaphoretickes
Other_Stramenopiles
Other_Amorphea
Osmo_Labyrinthulea
Other
Non-eukaryotes
Other_SAR

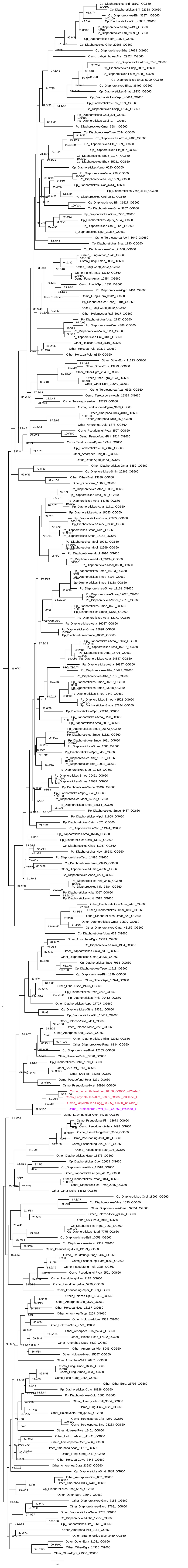
0.3

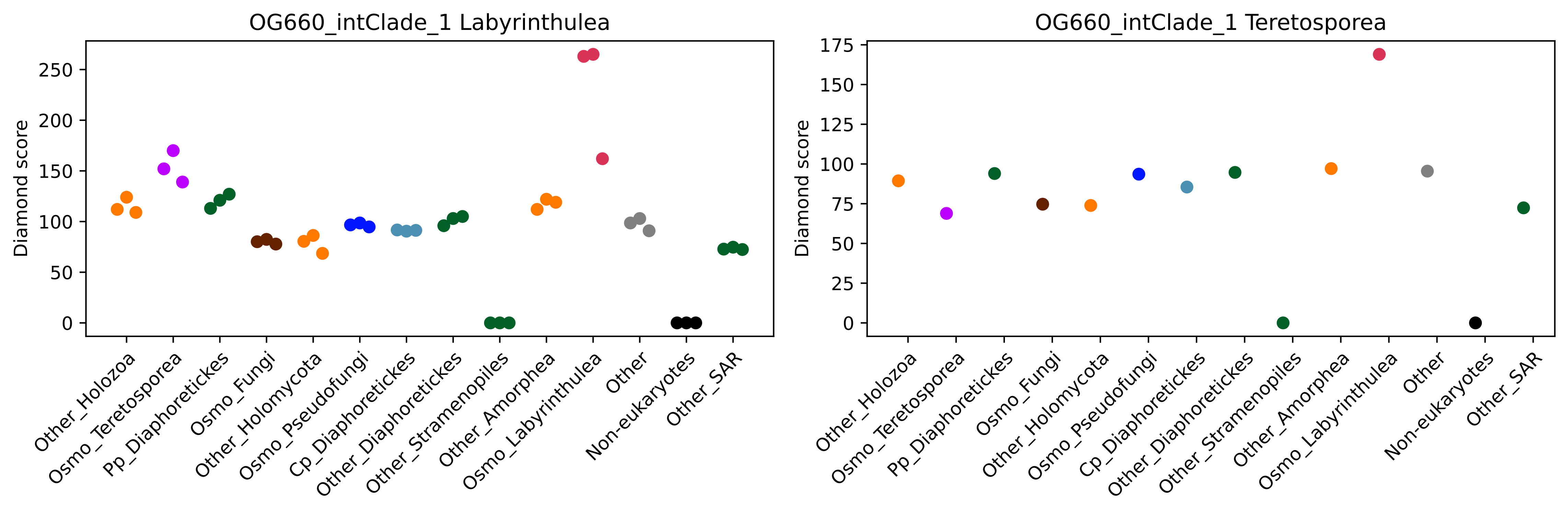
OG660_intClade_1 Labyrinthulea
Diamond score
0
50
100
150
200
250
Other_Holozoa
Osmo_Teretosporea
Pp_Diaphoretickes
Osmo_Fungi
Other_Holomycota
Osmo_Pseudofungi
Cp_Diaphoretickes
Other_Diaphoretickes
Other_Stramenopiles
Other_Amorphea
Osmo_Labyrinthulea
Other
Non-eukaryotes
Other_SAR
OG660_intClade_1 Teretosporea
Diamond score
0
25
50
75
100
125
150
175
Other_Holozoa
Osmo_Teretosporea
Pp_Diaphoretickes
Osmo_Fungi
Other_Holomycota
Osmo_Pseudofungi
Cp_Diaphoretickes
Other_Diaphoretickes
Other_Stramenopiles
Other_Amorphea
Osmo_Labyrinthulea
Other
Non-eukaryotes
Other_SAR

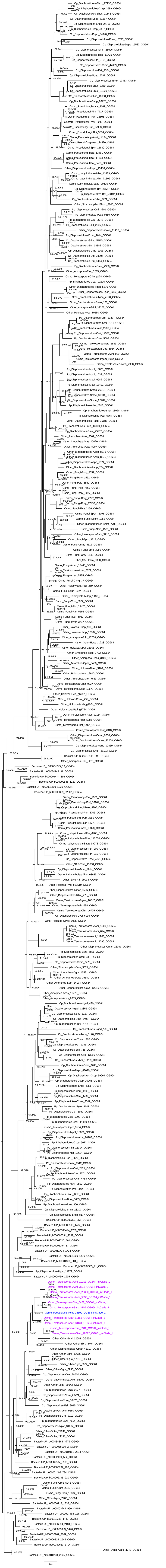

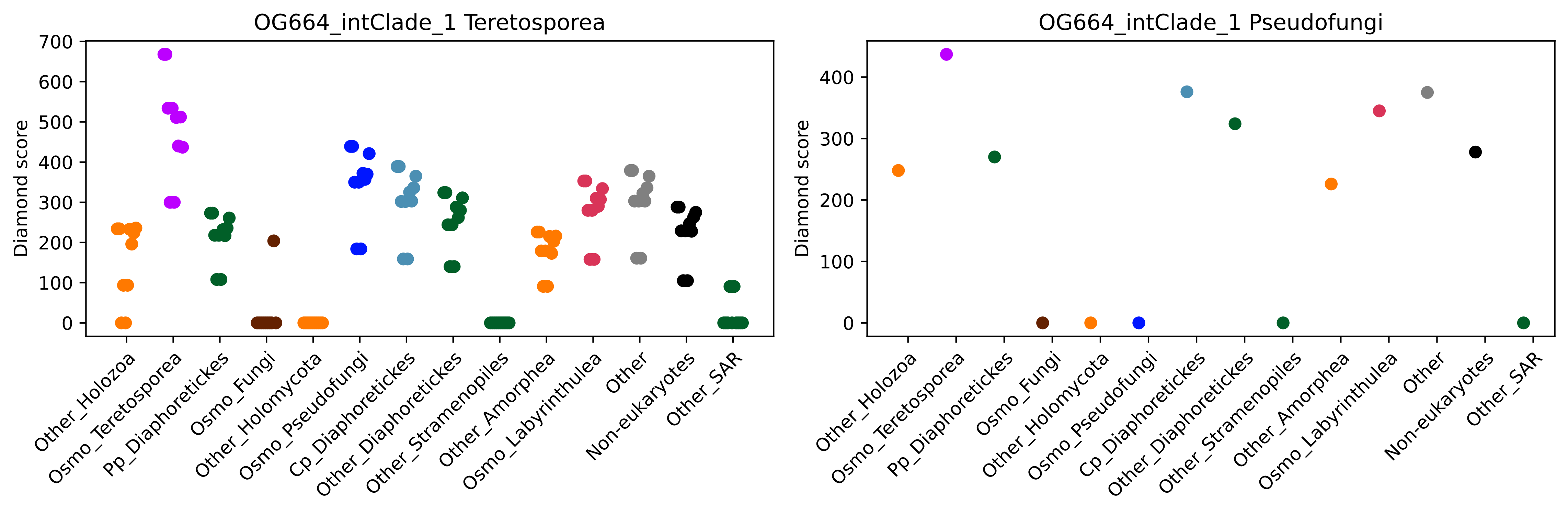
OG664_intClade_1 Teretosporea
OG664_intClade_1 Pseudofungi
Diamond score
0
100
200
300
400
500
600
700
Other_Holozoa
Osmo_Teretosporea
Pp_Diaphoretickes
Osmo_Fungi
Other_Holomycota
Osmo_Pseudofungi
Cp_Diaphoretickes
Other_Diaphoretickes
Other_Stramenopiles
Other_Amorphea
Osmo_Labyrinthulea
Other
Non-eukaryotes
Other_SAR

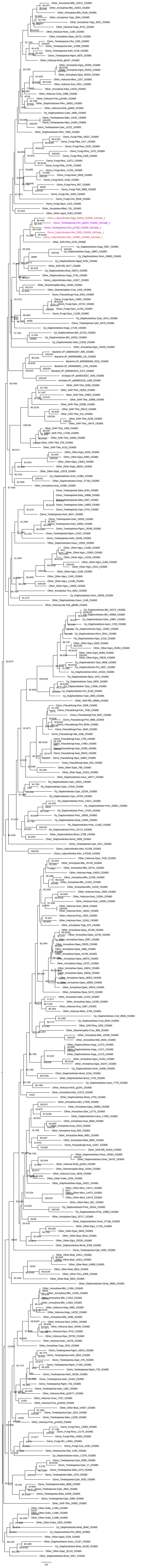

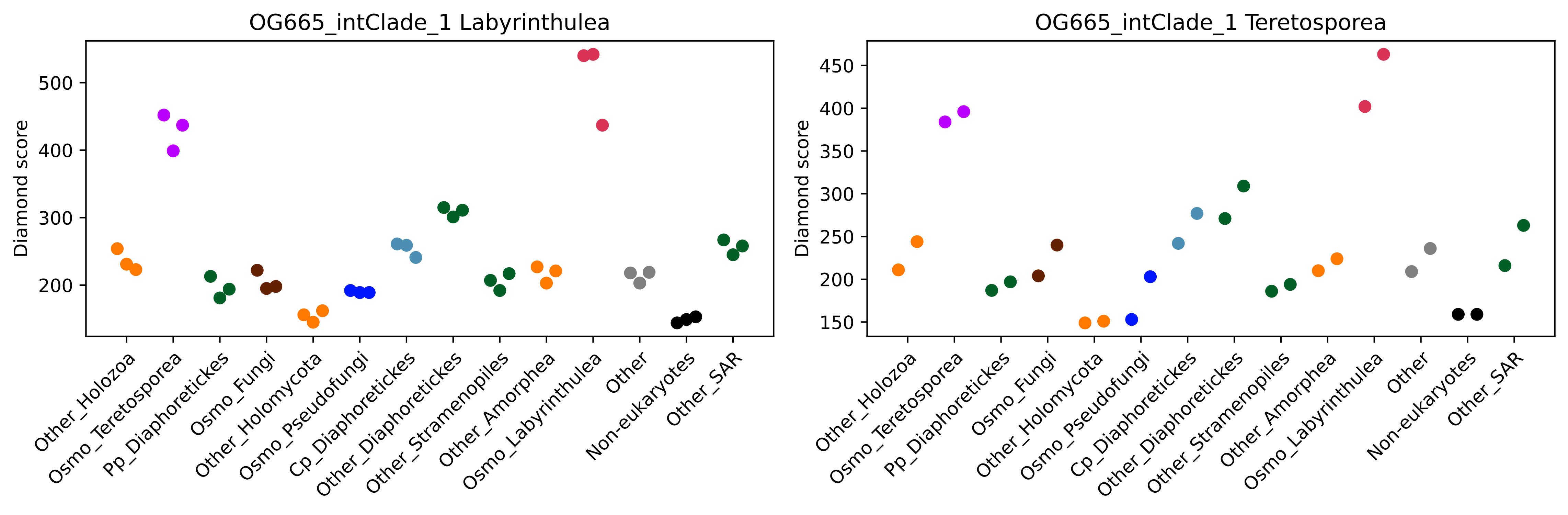
OG665_intClade_1 Labyrinthulea
Diamond score
500
400
300
200
Other_Holozoa
Osmo_Teretosporea
Pp_Diaphoretickes
Osmo_Fungi
Other_Holomycota
Osmo_Pseudofungi
Cp_Diaphoretickes
Other_Diaphoretickes
Other_Stramenopiles
Other_Amorphea
Osmo_Labyrinthulea
Other
Non-eukaryotes
Other_SAR
OG665_intClade_1 Teretosporea
450
400
350
300
250
200
150
Other_Holozoa
Osmo_Teretosporea
Pp_Diaphoretickes
Osmo_Fungi
Other_Holomycota
Osmo_Pseudofungi
Cp_Diaphoretickes
Other_Diaphoretickes
Other_Stramenopiles
Other_Amorphea
Osmo_Labyrinthulea
Other
Non-eukaryotes
Other_SAR

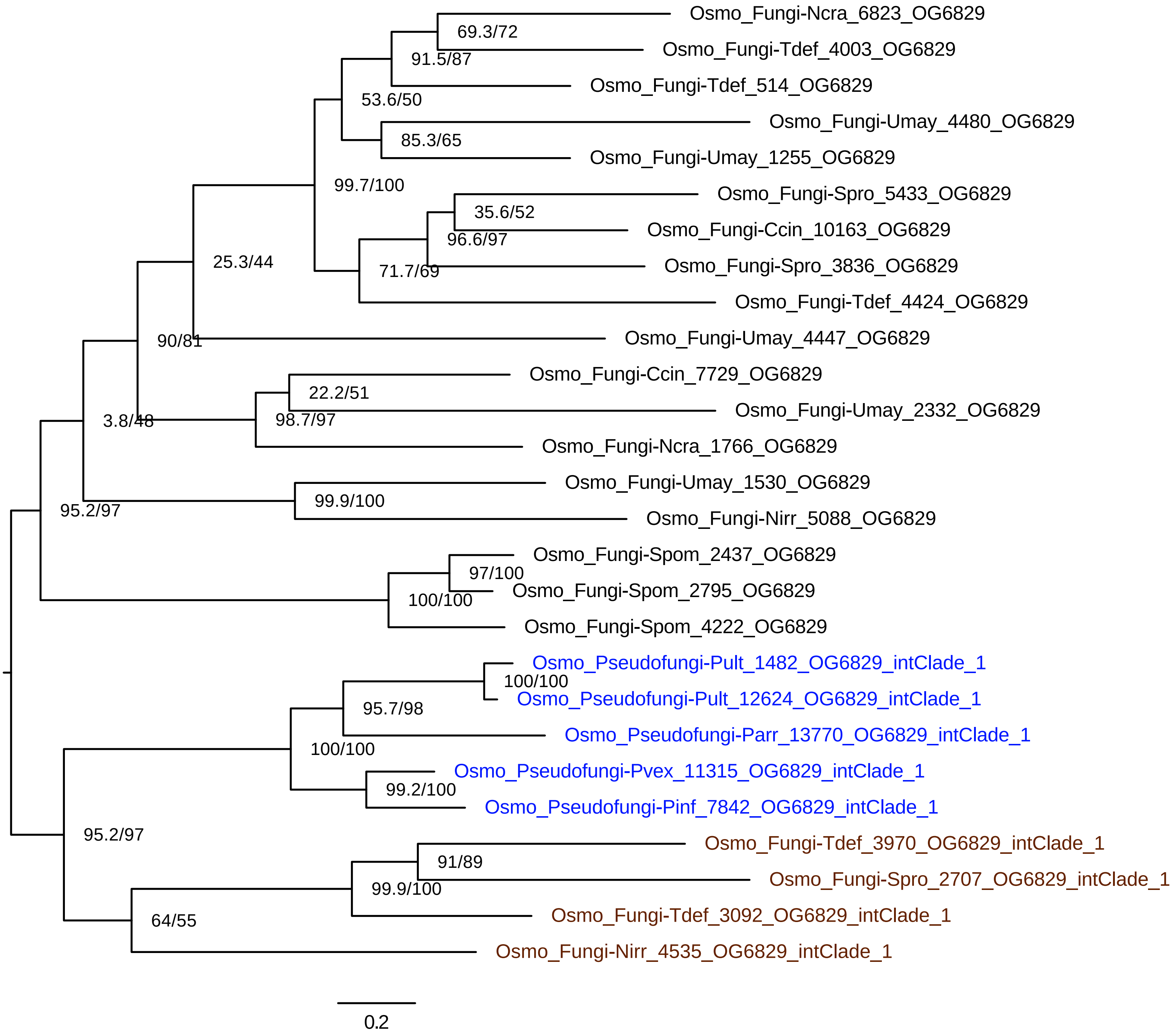
Osmo_Fungi-Ncra_6823_OG6829
69.3/72
Osmo_Fungi-Tdef_4003_OG6829
91.5/87
Osmo_Fungi-Tdef_514_OG6829
53.6/50
Osmo_Fungi-Umay_4480_OG6829
85.3/65
Osmo_Fungi-Umay_1255_OG6829
99.7/100
Osmo_Fungi-Spro_5433_OG6829
35.6/52
Osmo_Fungi-Ccin_10163_OG6829
96.6/97
25.3/44
Osmo_Fungi-Spro_3836_OG6829
71.7/69
Osmo_Fungi-Tdef_4424_OG6829
Osmo_Fungi-Umay_4447_OG6829
90/81
Osmo_Fungi-Ccin_7729_OG6829
22.2/51
Osmo_Fungi-Umay_2332_OG6829
3.8/48
98.7/97
Osmo_Fungi-Ncra_1766_OG6829
Osmo_Fungi-Umay_1530_OG6829
99.9/100
95.2/97
Osmo_Fungi-Nirr_5088_OG6829
Osmo_Fungi-Spom_2437_OG6829
97/100
Osmo_Fungi-Spom_2795_OG6829
100/100
Osmo_Fungi-Spom_4222_OG6829
Osmo_Pseudofungi-Pult_1482_OG6829_intClade_1
100/100
Osmo_Pseudofungi-Pult_12624_OG6829_intClade_1
95.7/98
Osmo_Pseudofungi-Parr_13770_OG6829_intClade_1
100/100
Osmo_Pseudofungi-Pvex_11315_OG6829_intClade_1
99.2/100
Osmo_Pseudofungi-Pinf_7842_OG6829_intClade_1
95.2/97
Osmo_Fungi-Tdef_3970_OG6829_intClade_1
91/89
Osmo_Fungi-Spro_2707_OG6829_intClade_1
99.9/100
Osmo_Fungi-Tdef_3092_OG6829_intClade_1
64/55
Osmo_Fungi-Nirr_4535_OG6829_intClade_1
0.2

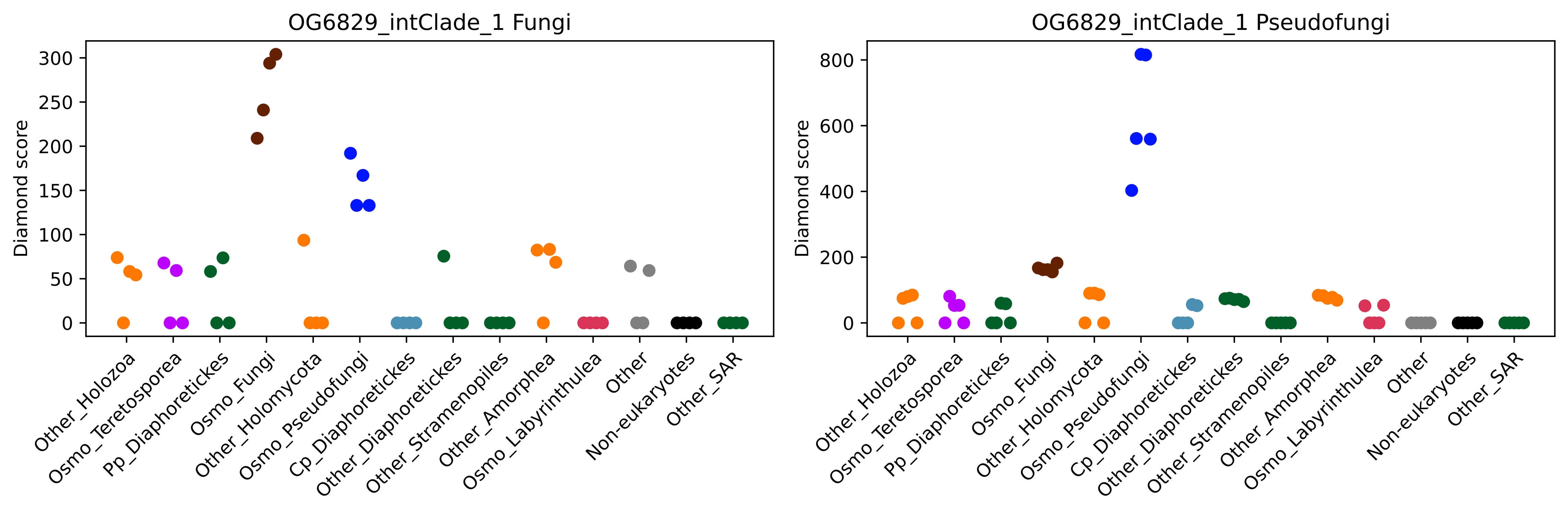
OG6829_intClade_1 Fungi
OG6829_intClade_1 Pseudofungi
Diamond score
0
50
100
150
200
250
300
200
400
600
800
Other_Holozoa
Osmo_Teretosporea
Pp_Diaphoretickes
Osmo_Fungi
Other_Holomycota
Osmo_Pseudofungi
Cp_Diaphoretickes
Other_Diaphoretickes
Other_Stramenopiles
Other_Amorphea
Osmo_Labyrinthulea
Other
Non-eukaryotes
Other_SAR

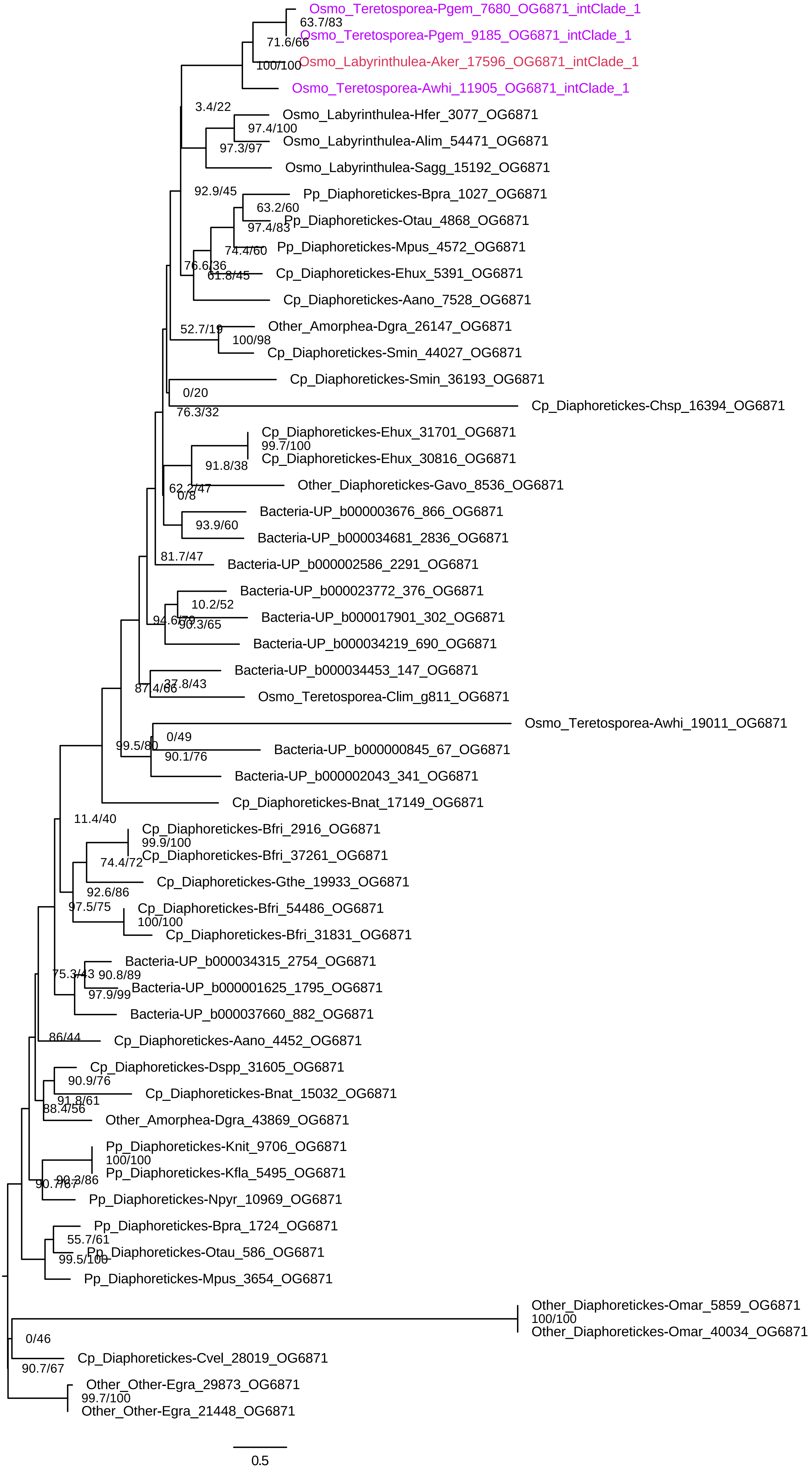

Osmo_Teretosporea-Pgem_7680_OG6871_intClade_1
63.7/83
Osmo_Teretosporea-Pgem_9185_OG6871_intClade_1
71.6/66
Osmo_Labyrinthulea-Aker_17596_OG6871_intClade_1
100/100
Osmo_Teretosporea-Awhi_11905_OG6871_intClade_1
3.4/22
Osmo_Labyrinthulea-Hfer_3077_OG6871
97.4/100
Osmo_Labyrinthulea-Alim_54471_OG6871
97.3/97
Osmo_Labyrinthulea-Sagg_15192_OG6871
92.9/45
Pp_Diaphoretickes-Bpra_1027_OG6871
63.2/60
Pp_Diaphoretickes-Otau_4868_OG6871
97.4/83
Pp_Diaphoretickes-Mpus_4572_OG6871
74.4/60
76.6/36
61.8/45
Cp_Diaphoretickes-Ehux_5391_OG6871
Cp_Diaphoretickes-Aano_7528_OG6871
52.7/19
Other_Amorphea-Dgra_26147_OG6871
100/98
Cp_Diaphoretickes-Smin_44027_OG6871
Cp_Diaphoretickes-Smin_36193_OG6871
0/20
Cp_Diaphoretickes-Chsp_16394_OG6871
76.3/32
Cp_Diaphoretickes-Ehux_31701_OG6871
99.7/100
Cp_Diaphoretickes-Ehux_30816_OG6871
91.8/38
Other_Diaphoretickes-Gavo_8536_OG6871
62.2/47
0/8
Bacteria-UP_b000003676_866_OG6871
93.9/60
Bacteria-UP_b000034681_2836_OG6871
81.7/47
Bacteria-UP_b000002586_2291_OG6871
Bacteria-UP_b000023772_376_OG6871
10.2/52
Bacteria-UP_b000017901_302_OG6871
94.6/79
90.3/65
Bacteria-UP_b000034219_690_OG6871
Bacteria-UP_b000034453_147_OG6871
37.8/43
87.4/66
Osmo_Teretosporea-Clim_g811_OG6871
Osmo_Teretosporea-Awhi_19011_OG6871
0/49
99.5/80
Bacteria-UP_b000000845_67_OG6871
90.1/76
Bacteria-UP_b000002043_341_OG6871
Cp_Diaphoretickes-Bnat_17149_OG6871
11.4/40
Cp_Diaphoretickes-Bfri_2916_OG6871
99.9/100
Cp_Diaphoretickes-Bfri_37261_OG6871
74.4/72
Cp_Diaphoretickes-Gthe_19933_OG6871
92.6/86
97.5/75
Cp_Diaphoretickes-Bfri_54486_OG6871
100/100
Cp_Diaphoretickes-Bfri_31831_OG6871
Bacteria-UP_b000034315_2754_OG6871
75.3/43
90.8/89
Bacteria-UP_b000001625_1795_OG6871
97.9/99
Bacteria-UP_b000037660_882_OG6871
86/44
Cp_Diaphoretickes-Aano_4452_OG6871
Cp_Diaphoretickes-Dspp_31605_OG6871
90.9/76
Cp_Diaphoretickes-Bnat_15032_OG6871
91.8/61
88.4/56
Other_Amorphea-Dgra_43869_OG6871
Pp_Diaphoretickes-Knit_9706_OG6871
100/100
Pp_Diaphoretickes-Kfla_5495_OG6871
90.7/67
90.3/86
Pp_Diaphoretickes-Npyr_10969_OG6871
Pp_Diaphoretickes-Bpra_1724_OG6871
55.7/61
Pp_Diaphoretickes-Otau_586_OG6871
99.5/100
Pp_Diaphoretickes-Mpus_3654_OG6871
Other_Diaphoretickes-Omar_5859_OG6871
100/100
Other_Diaphoretickes-Omar_40034_OG6871
0/46
Cp_Diaphoretickes-Cvel_28019_OG6871
90.7/67
Other_Other-Egra_29873_OG6871
99.7/100
Other_Other-Egra_21448_OG6871
0.5

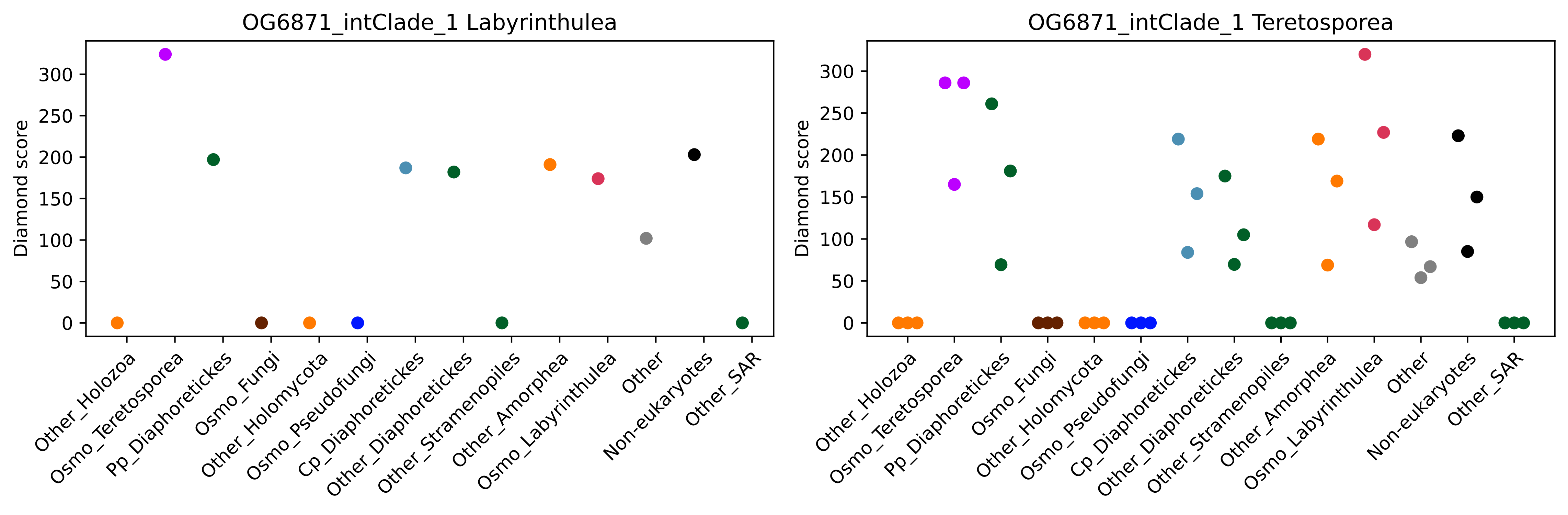
OG6871_intClade_1 Labyrinthulea
OG6871_intClade_1 Teretosporea
Diamond score
0
50
100
150
200
250
300
Other_Holozoa
Osmo_Teretosporea
Pp_Diaphoretickes
Osmo_Fungi
Other_Holomycota
Osmo_Pseudofungi
Cp_Diaphoretickes
Other_Diaphoretickes
Other_Stramenopiles
Other_Amorphea
Osmo_Labyrinthulea
Other
Non-eukaryotes
Other_SAR

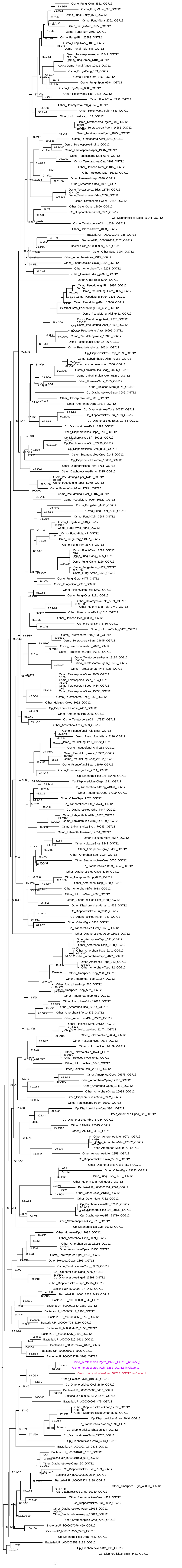
0.3

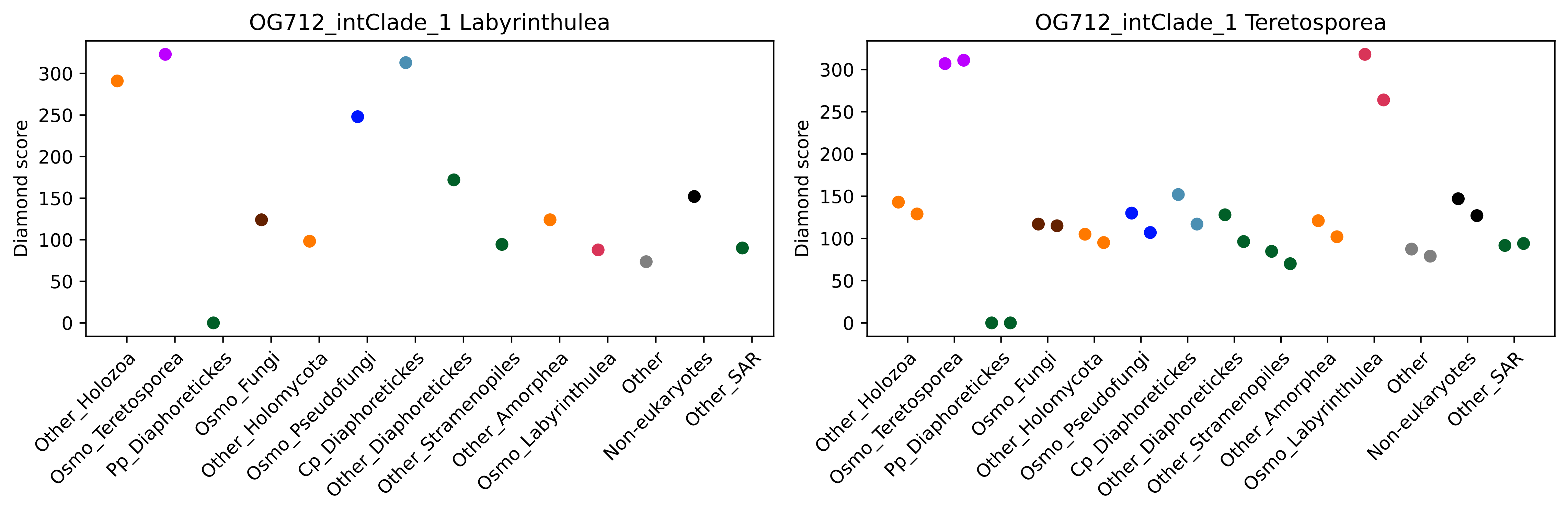

OG712_intClade_1 Labyrinthulea
OG712_intClade_1 Teretosporea
Diamond score
0
50
100
150
200
250
300
Other_Holozoa
Osmo_Teretosporea
Pp_Diaphoretickes
Osmo_Fungi
Other_Holomycota
Osmo_Pseudofungi
Cp_Diaphoretickes
Other_Diaphoretickes
Other_Stramenopiles
Other_Amorphea
Osmo_Labyrinthulea
Other
Non-eukaryotes
Other_SAR

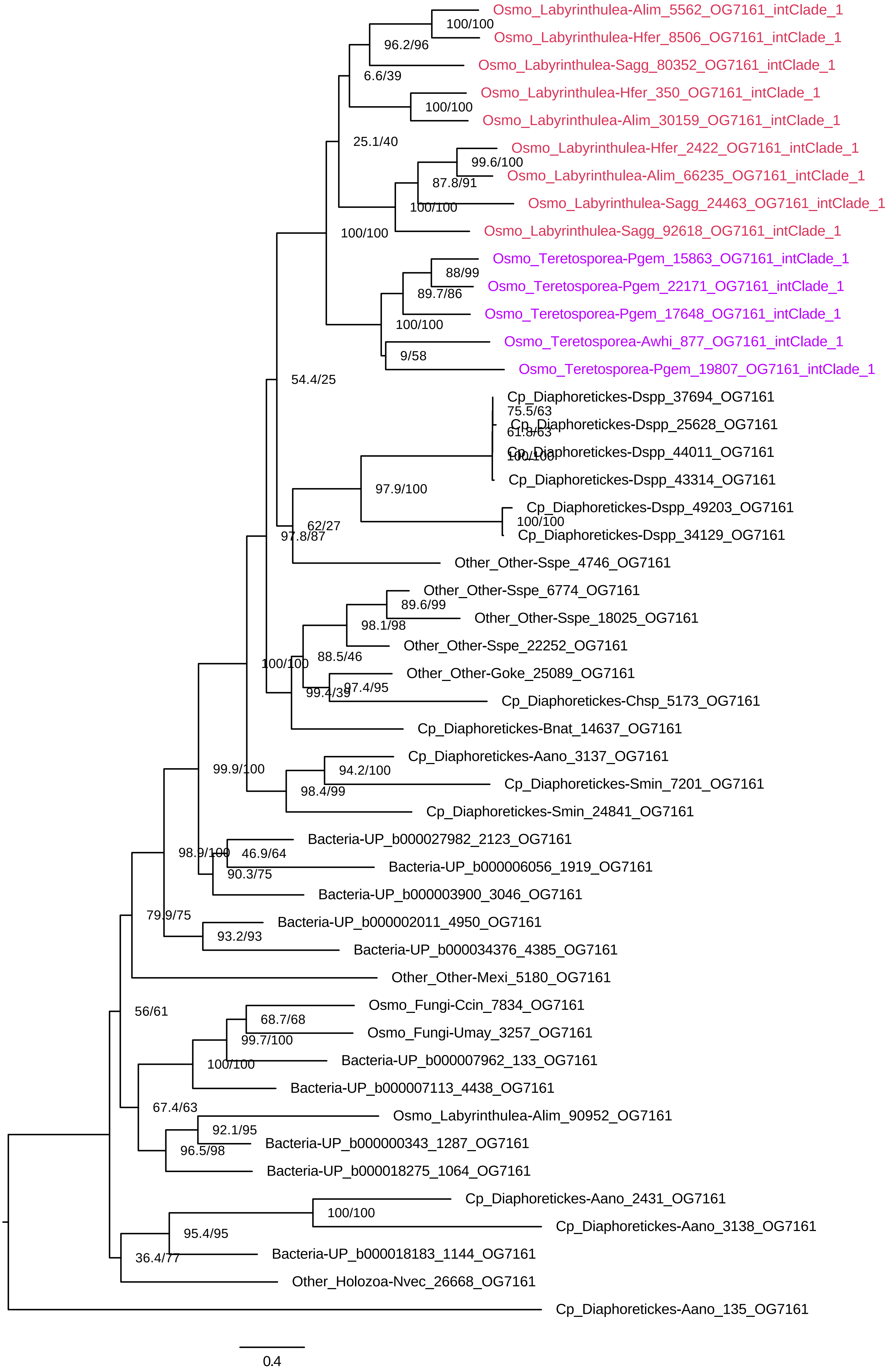

Osmo_Labyrinthulea-Alim_5562_OG7161_intClade_1
100/100
Osmo_Labyrinthulea-Hfer_8506_OG7161_intClade_1
96.2/96
Osmo_Labyrinthulea-Sagg_80352_OG7161_intClade_1
6.6/39
Osmo_Labyrinthulea-Hfer_350_OG7161_intClade_1
100/100
Osmo_Labyrinthulea-Alim_30159_OG7161_intClade_1
25.1/40
Osmo_Labyrinthulea-Hfer_2422_OG7161_intClade_1
99.6/100
Osmo_Labyrinthulea-Alim_66235_OG7161_intClade_1
87.8/91
Osmo_Labyrinthulea-Sagg_24463_OG7161_intClade_1
100/100
100/100
Osmo_Labyrinthulea-Sagg_92618_OG7161_intClade_1
Osmo_Teretosporea-Pgem_15863_OG7161_intClade_1
88/99
Osmo_Teretosporea-Pgem_22171_OG7161_intClade_1
89.7/86
Osmo_Teretosporea-Pgem_17648_OG7161_intClade_1
100/100
Osmo_Teretosporea-Awhi_877_OG7161_intClade_1
9/58
Osmo_Teretosporea-Pgem_19807_OG7161_intClade_1
54.4/25
Cp_Diaphoretickes-Dspp_37694_OG7161
75.5/63
Cp_Diaphoretickes-Dspp_25628_OG7161
61.8/63
Cp_Diaphoretickes-Dspp_44011_OG7161
100/100
Cp_Diaphoretickes-Dspp_43314_OG7161
97.9/100
Cp_Diaphoretickes-Dspp_49203_OG7161
100/100
62/27
Cp_Diaphoretickes-Dspp_34129_OG7161
97.8/87
Other_Other-Sspe_4746_OG7161
Other_Other-Sspe_6774_OG7161
89.6/99
Other_Other-Sspe_18025_OG7161
98.1/98
Other_Other-Sspe_22252_OG7161
88.5/46
100/100
Other_Other-Goke_25089_OG7161
97.4/95
99.4/39
Cp_Diaphoretickes-Chsp_5173_OG7161
Cp_Diaphoretickes-Bnat_14637_OG7161
Cp_Diaphoretickes-Aano_3137_OG7161
94.2/100
99.9/100
Cp_Diaphoretickes-Smin_7201_OG7161
98.4/99
Cp_Diaphoretickes-Smin_24841_OG7161
Bacteria-UP_b000027982_2123_OG7161
98.9/100
46.9/64
Bacteria-UP_b000006056_1919_OG7161
90.3/75
Bacteria-UP_b000003900_3046_OG7161
79.9/75
Bacteria-UP_b000002011_4950_OG7161
93.2/93
Bacteria-UP_b000034376_4385_OG7161
Other_Other-Mexi_5180_OG7161
56/61
Osmo_Fungi-Ccin_7834_OG7161
68.7/68
Osmo_Fungi-Umay_3257_OG7161
99.7/100
Bacteria-UP_b000007962_133_OG7161
100/100
Bacteria-UP_b000007113_4438_OG7161
67.4/63
Osmo_Labyrinthulea-Alim_90952_OG7161
92.1/95
Bacteria-UP_b000000343_1287_OG7161
96.5/98
Bacteria-UP_b000018275_1064_OG7161
Cp_Diaphoretickes-Aano_2431_OG7161
100/100
Cp_Diaphoretickes-Aano_3138_OG7161
95.4/95
Bacteria-UP_b000018183_1144_OG7161
36.4/77
Other_Holozoa-Nvec_26668_OG7161
Cp_Diaphoretickes-Aano_135_OG7161
0.4

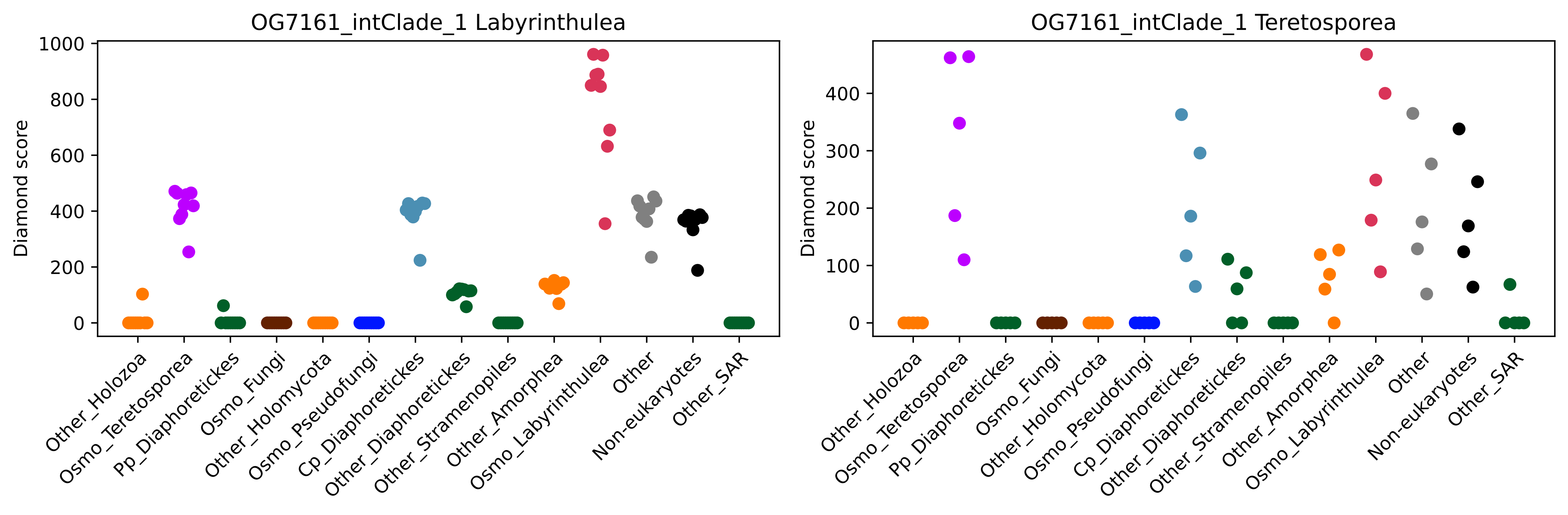

OG7161_intClade_1 Labyrinthulea
OG7161_intClade_1 Teretosporea
Diamond score
0
200
400
600
800
1000
100
300
Other_Holozoa
Osmo_Teretosporea
Pp_Diaphoretickes
Osmo_Fungi
Other_Holomycota
Osmo_Pseudofungi
Cp_Diaphoretickes
Other_Diaphoretickes
Other_Stramenopiles
Other_Amorphea
Osmo_Labyrinthulea
Other
Non-eukaryotes
Other_SAR

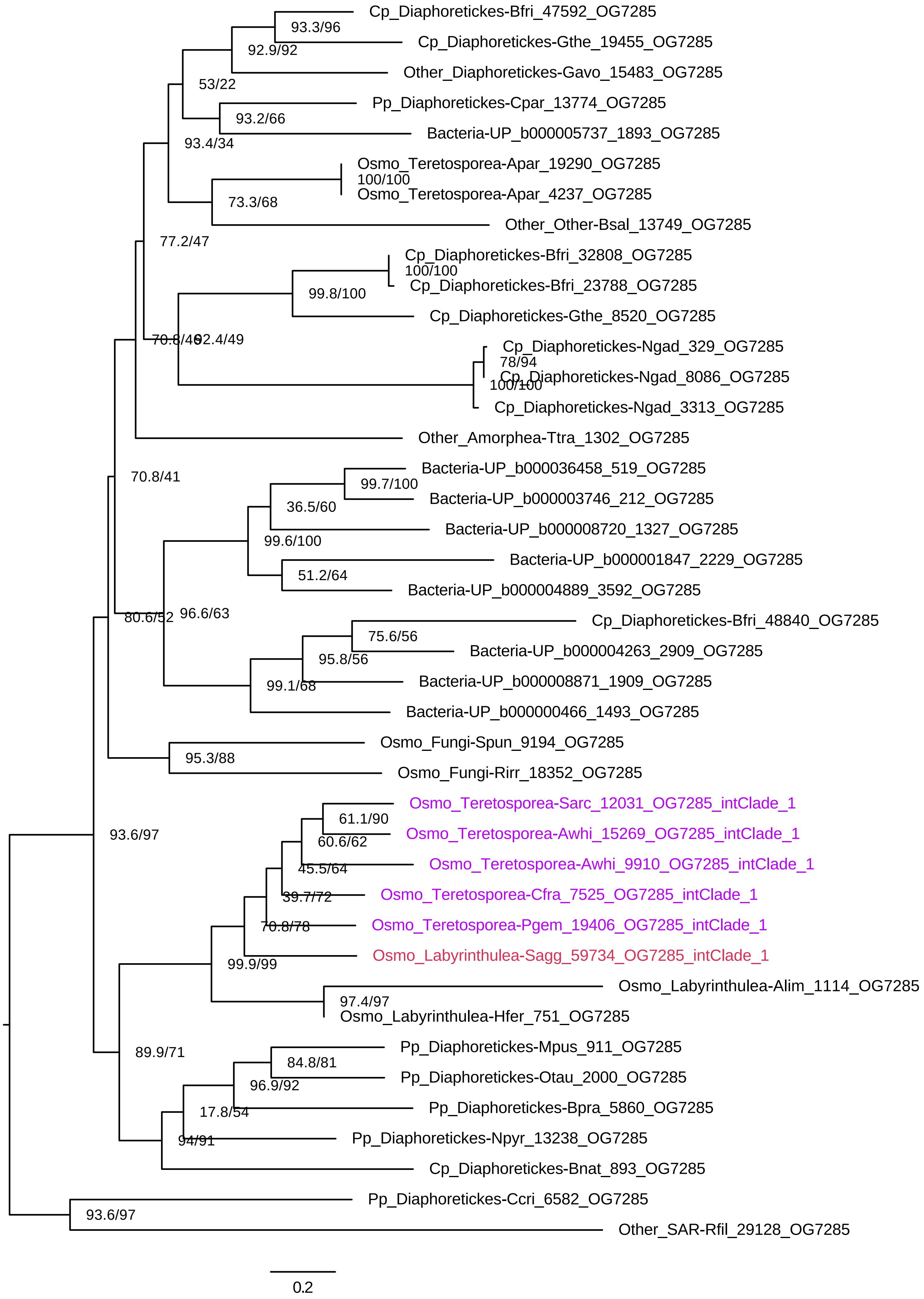
Cp_Diaphoretickes-Bfri_47592_OG7285
93.3/96
Cp_Diaphoretickes-Gthe_19455_OG7285
92.9/92
Other_Diaphoretickes-Gavo_15483_OG7285
53/22
Pp_Diaphoretickes-Cpar_13774_OG7285
93.2/66
Bacteria-UP_b000005737_1893_OG7285
93.4/34
Osmo_Teretosporea-Apar_19290_OG7285
100/100
Osmo_Teretosporea-Apar_4237_OG7285
73.3/68
Other_Other-Bsal_13749_OG7285
77.2/47
Cp_Diaphoretickes-Bfri_32808_OG7285
100/100
Cp_Diaphoretickes-Bfri_23788_OG7285
99.8/100
Cp_Diaphoretickes-Gthe_8520_OG7285
70.8/40
92.4/49
Cp_Diaphoretickes-Ngad_329_OG7285
78/94
Cp_Diaphoretickes-Ngad_8086_OG7285
100/100
Cp_Diaphoretickes-Ngad_3313_OG7285
Other_Amorphea-Ttra_1302_OG7285
70.8/41
Bacteria-UP_b000036458_519_OG7285
99.7/100
Bacteria-UP_b000003746_212_OG7285
36.5/60
Bacteria-UP_b000008720_1327_OG7285
99.6/100
Bacteria-UP_b000001847_2229_OG7285
51.2/64
Bacteria-UP_b000004889_3592_OG7285
80.6/52
96.6/63
Cp_Diaphoretickes-Bfri_48840_OG7285
75.6/56
Bacteria-UP_b000004263_2909_OG7285
95.8/56
Bacteria-UP_b000008871_1909_OG7285
99.1/68
Bacteria-UP_b000000466_1493_OG7285
Osmo_Fungi-Spun_9194_OG7285
95.3/88
Osmo_Fungi-Rirr_18352_OG7285
Osmo_Teretosporea-Sarc_12031_OG7285_intClade_1
61.1/90
Osmo_Teretosporea-Awhi_15269_OG7285_intClade_1
60.6/62
93.6/97
Osmo_Teretosporea-Awhi_9910_OG7285_intClade_1
45.5/64
Osmo_Teretosporea-Cfra_7525_OG7285_intClade_1
39.7/72
Osmo_Teretosporea-Pgem_19406_OG7285_intClade_1
70.8/78
Osmo_Labyrinthulea-Sagg_59734_OG7285_intClade_1
99.9/99
Osmo_Labyrinthulea-Alim_1114_OG7285
97.4/97
Osmo_Labyrinthulea-Hfer_751_OG7285
89.9/71
Pp_Diaphoretickes-Mpus_911_OG7285
84.8/81
Pp_Diaphoretickes-Otau_2000_OG7285
96.9/92
Pp_Diaphoretickes-Bpra_5860_OG7285
17.8/54
Pp_Diaphoretickes-Npyr_13238_OG7285
94/91
Cp_Diaphoretickes-Bnat_893_OG7285
Pp_Diaphoretickes-Ccri_6582_OG7285
93.6/97
Other_SAR-Rfil_29128_OG7285
0.2

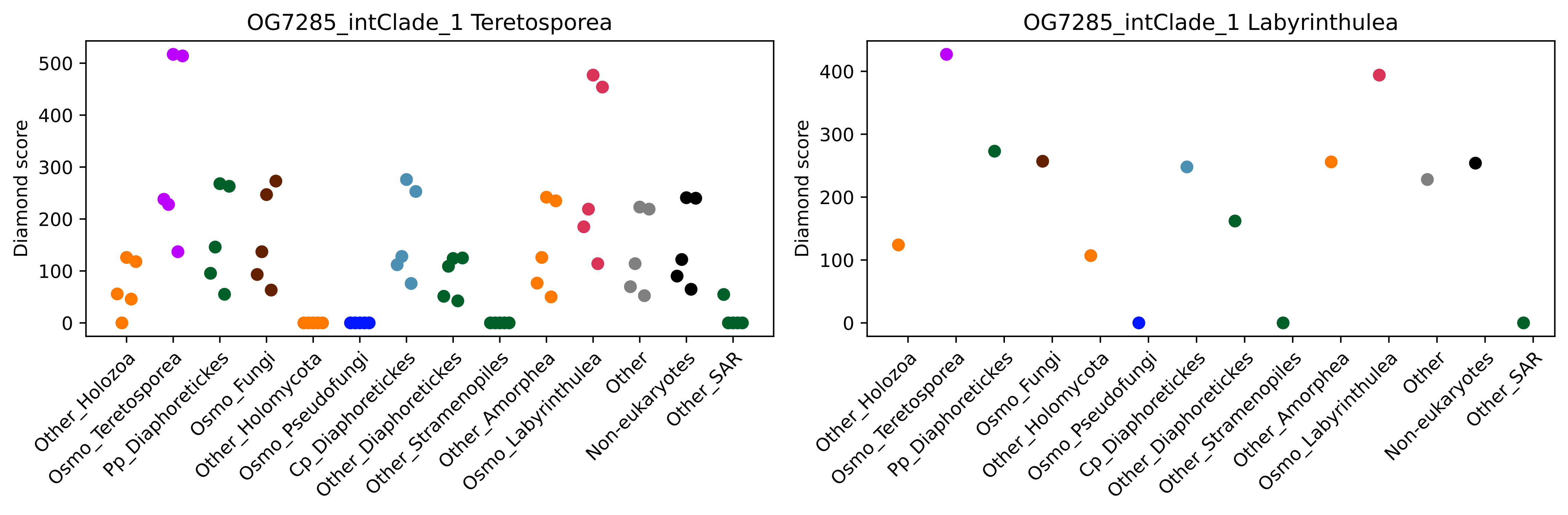

OG7285_intClade_1 Teretosporea
OG7285_intClade_1 Labyrinthulea
Diamond score
0
100
200
300
400
500
Other_Holozoa
Osmo_Teretosporea
Pp_Diaphoretickes
Osmo_Fungi
Other_Holomycota
Osmo_Pseudofungi
Cp_Diaphoretickes
Other_Diaphoretickes
Other_Stramenopiles
Other_Amorphea
Osmo_Labyrinthulea
Other
Non-eukaryotes
Other_SAR

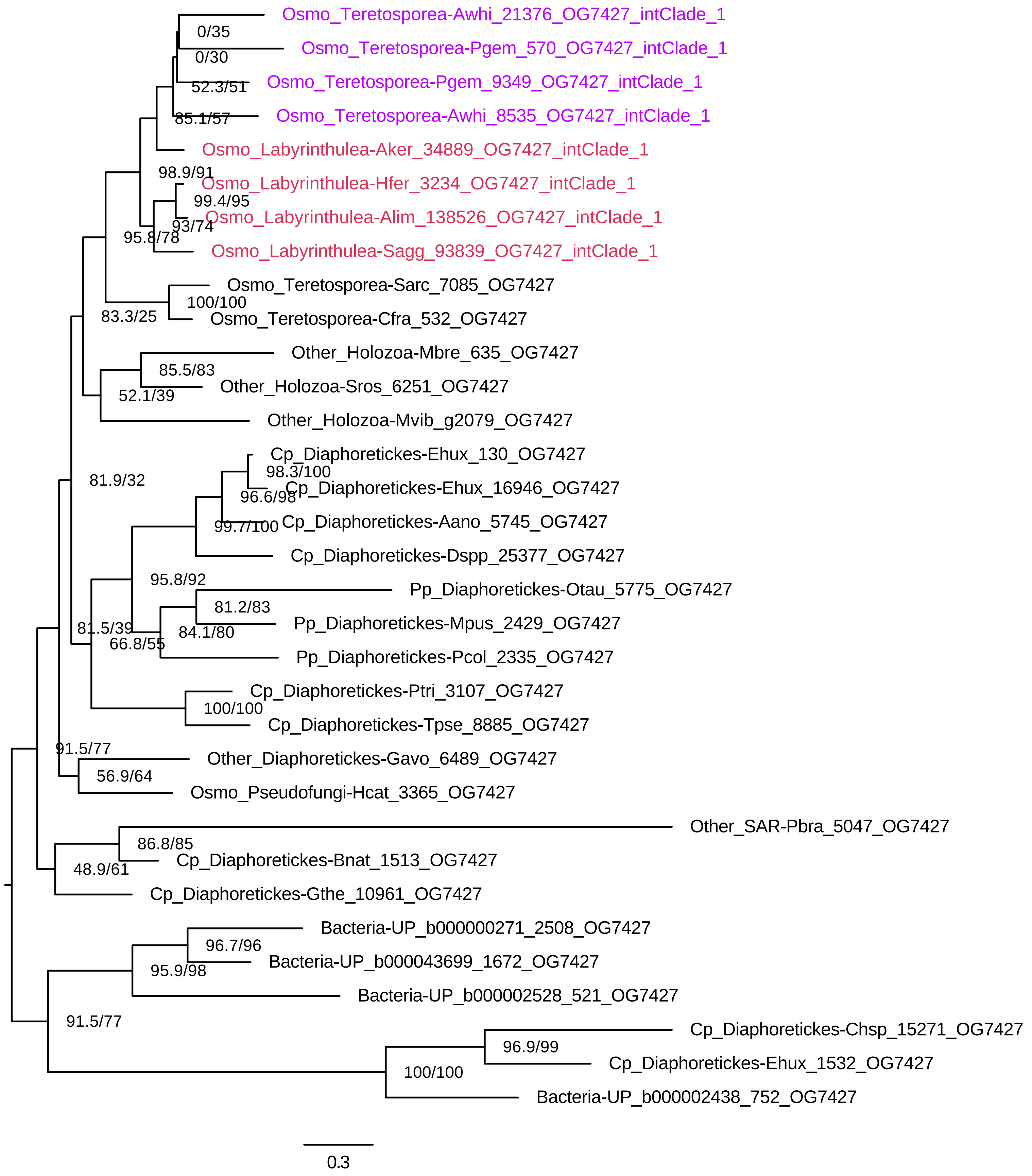

Osmo_Teretosporea-Awhi_21376_OG7427_intClade_1
0/35
Osmo_Teretosporea-Pgem_570_OG7427_intClade_1
0/30
Osmo_Teretosporea-Pgem_9349_OG7427_intClade_1
52.3/51
Osmo_Teretosporea-Awhi_8535_OG7427_intClade_1
85.1/57
Osmo_Labyrinthulea-Aker_34889_OG7427_intClade_1
98.9/91
Osmo_Labyrinthulea-Hfer_3234_OG7427_intClade_1
99.4/95
Osmo_Labyrinthulea-Alim_138526_OG7427_intClade_1
93/74
95.8/78
Osmo_Labyrinthulea-Sagg_93839_OG7427_intClade_1
Osmo_Teretosporea-Sarc_7085_OG7427
100/100
83.3/25
Osmo_Teretosporea-Cfra_532_OG7427
Other_Holozoa-Mbre_635_OG7427
85.5/83
Other_Holozoa-Sros_6251_OG7427
52.1/39
Other_Holozoa-Mvib_g2079_OG7427
Cp_Diaphoretickes-Ehux_130_OG7427
98.3/100
81.9/32
Cp_Diaphoretickes-Ehux_16946_OG7427
96.6/98
Cp_Diaphoretickes-Aano_5745_OG7427
99.7/100
Cp_Diaphoretickes-Dspp_25377_OG7427
95.8/92
Pp_Diaphoretickes-Otau_5775_OG7427
81.2/83
Pp_Diaphoretickes-Mpus_2429_OG7427
81.5/39
84.1/80
66.8/55
Pp_Diaphoretickes-Pcol_2335_OG7427
Cp_Diaphoretickes-Ptri_3107_OG7427
100/100
Cp_Diaphoretickes-Tpse_8885_OG7427
91.5/77
Other_Diaphoretickes-Gavo_6489_OG7427
56.9/64
Osmo_Pseudofungi-Hcat_3365_OG7427
Other_SAR-Pbra_5047_OG7427
86.8/85
Cp_Diaphoretickes-Bnat_1513_OG7427
48.9/61
Cp_Diaphoretickes-Gthe_10961_OG7427
Bacteria-UP_b000000271_2508_OG7427
96.7/96
Bacteria-UP_b000043699_1672_OG7427
95.9/98
Bacteria-UP_b000002528_521_OG7427
91.5/77
Cp_Diaphoretickes-Chsp_15271_OG7427
96.9/99
Cp_Diaphoretickes-Ehux_1532_OG7427
100/100
Bacteria-UP_b000002438_752_OG7427
0.3

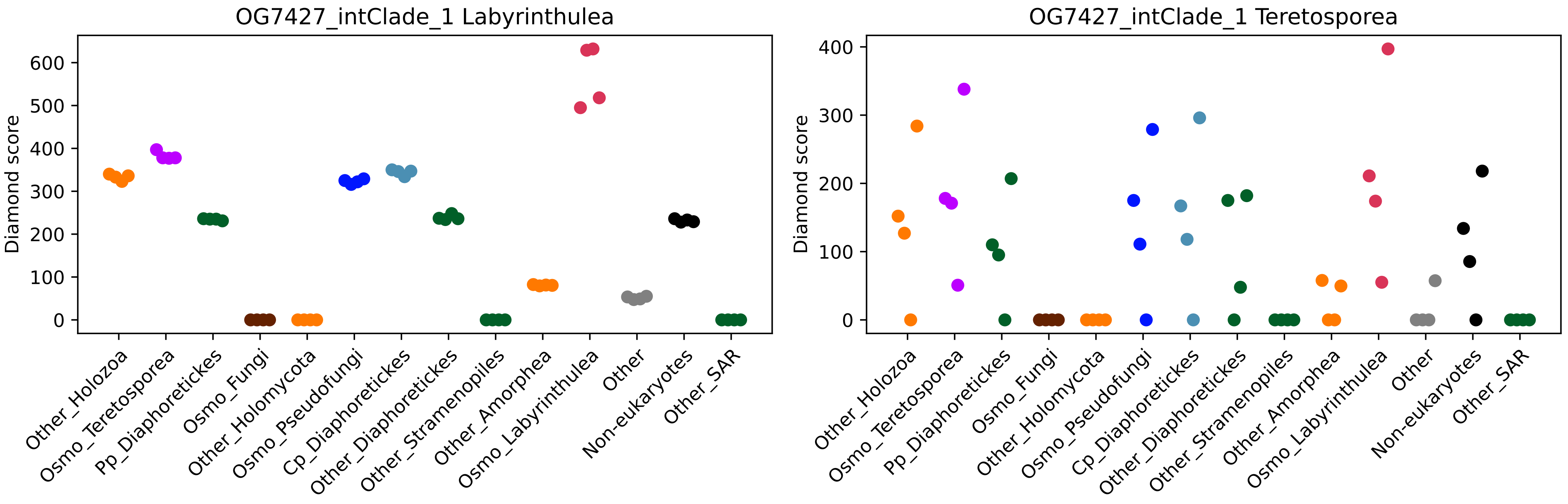

OG7427_intClade_1 Labyrinthulea
OG7427_intClade_1 Teretosporea
Diamond score
0
100
200
300
400
500
600
Other_Holozoa
Osmo_Teretosporea
Pp_Diaphoretickes
Osmo_Fungi
Other_Holomycota
Osmo_Pseudofungi
Cp_Diaphoretickes
Other_Diaphoretickes
Other_Stramenopiles
Other_Amorphea
Osmo_Labyrinthulea
Other
Non-eukaryotes
Other_SAR

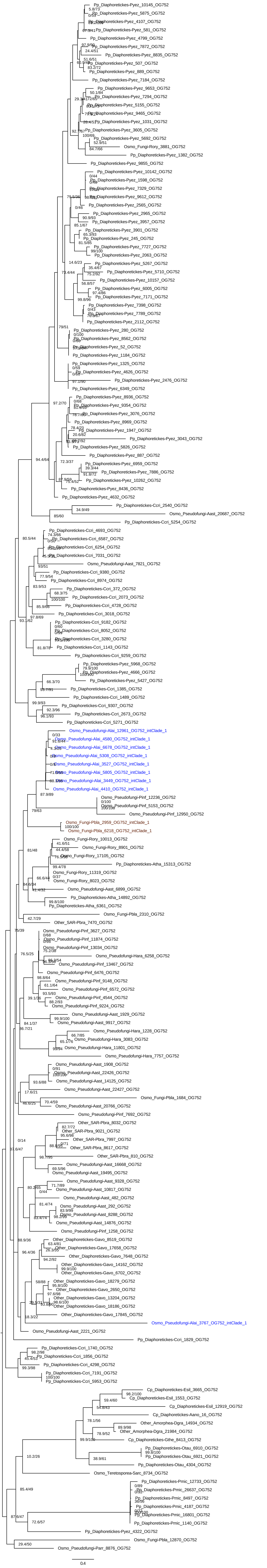
Pp_Diaphoretickes-Pyez_10145_OG752
Pp_Diaphoretickes-Pyez_5875_OG752
Pp_Diaphoretickes-Pyez_4107_OG752
Pp_Diaphoretickes-Pyez_581_OG752
Pp_Diaphoretickes-Pyez_4799_OG752
Pp_Diaphoretickes-Pyez_7872_OG752
Pp_Diaphoretickes-Pyez_8835_OG752
Pp_Diaphoretickes-Pyez_507_OG752
Pp_Diaphoretickes-Pyez_889_OG752
Pp_Diaphoretickes-Pyez_7184_OG752
Pp_Diaphoretickes-Pyez_9653_OG752
Pp_Diaphoretickes-Pyez_7294_OG752
Pp_Diaphoretickes-Pyez_5155_OG752
Pp_Diaphoretickes-Pyez_9465_OG752
Pp_Diaphoretickes-Pyez_1031_OG752
Pp_Diaphoretickes-Pyez_3605_OG752
Pp_Diaphoretickes-Pyez_5692_OG752
Osmo_Fungi-Rory_3881_OG752
Pp_Diaphoretickes-Pyez_1382_OG752
Pp_Diaphoretickes-Pyez_9855_OG752
Pp_Diaphoretickes-Pyez_10142_OG752
Pp_Diaphoretickes-Pyez_1598_OG752
Pp_Diaphoretickes-Pyez_7329_OG752
Pp_Diaphoretickes-Pyez_9612_OG752
Pp_Diaphoretickes-Pyez_2565_OG752
Pp_Diaphoretickes-Pyez_2965_OG752
Pp_Diaphoretickes-Pyez_3957_OG752
Pp_Diaphoretickes-Pyez_3901_OG752
Pp_Diaphoretickes-Pyez_245_OG752
Pp_Diaphoretickes-Pyez_7727_OG752
Pp_Diaphoretickes-Pyez_2063_OG752
Pp_Diaphoretickes-Pyez_5267_OG752
Pp_Diaphoretickes-Pyez_5710_OG752
Pp_Diaphoretickes-Pyez_10157_OG752
Pp_Diaphoretickes-Pyez_6005_OG752
Pp_Diaphoretickes-Pyez_7171_OG752
Pp_Diaphoretickes-Pyez_7398_OG752
Pp_Diaphoretickes-Pyez_7789_OG752
Pp_Diaphoretickes-Pyez_2112_OG752
Pp_Diaphoretickes-Pyez_280_OG752
Pp_Diaphoretickes-Pyez_8562_OG752
Pp_Diaphoretickes-Pyez_52_OG752
Pp_Diaphoretickes-Pyez_1184_OG752
Pp_Diaphoretickes-Pyez_1325_OG752
Pp_Diaphoretickes-Pyez_4626_OG752
Pp_Diaphoretickes-Pyez_2476_OG752
Pp_Diaphoretickes-Pyez_6349_OG752
Pp_Diaphoretickes-Pyez_8936_OG752
Pp_Diaphoretickes-Pyez_9354_OG752
Pp_Diaphoretickes-Pyez_3076_OG752
Pp_Diaphoretickes-Pyez_8969_OG752
Pp_Diaphoretickes-Pyez_1947_OG752
Pp_Diaphoretickes-Pyez_3043_OG752
Pp_Diaphoretickes-Pyez_5826_OG752
Pp_Diaphoretickes-Pyez_887_OG752
Pp_Diaphoretickes-Pyez_6959_OG752
Pp_Diaphoretickes-Pyez_7886_OG752
Pp_Diaphoretickes-Pyez_10262_OG752
Pp_Diaphoretickes-Pyez_8436_OG752
Pp_Diaphoretickes-Pyez_4632_OG752
Pp_Diaphoretickes-Ccri_2540_OG752
Osmo_Pseudofungi-Aast_20687_OG752
Pp_Diaphoretickes-Ccri_5254_OG752
Pp_Diaphoretickes-Ccri_4693_OG752
Pp_Diaphoretickes-Ccri_6587_OG752
Pp_Diaphoretickes-Ccri_6254_OG752
Pp_Diaphoretickes-Ccri_7031_OG752
Osmo_Pseudofungi-Aast_7821_OG752
Pp_Diaphoretickes-Ccri_9380_OG752
Pp_Diaphoretickes-Ccri_8974_OG752
Pp_Diaphoretickes-Ccri_372_OG752
Pp_Diaphoretickes-Ccri_2073_OG752
Pp_Diaphoretickes-Ccri_4728_OG752
Pp_Diaphoretickes-Ccri_3018_OG752
Pp_Diaphoretickes-Ccri_9182_OG752
Pp_Diaphoretickes-Ccri_8052_OG752
Pp_Diaphoretickes-Ccri_3280_OG752
Pp_Diaphoretickes-Ccri_1143_OG752
Pp_Diaphoretickes-Ccri_9259_OG752
Pp_Diaphoretickes-Pyez_5968_OG752
Pp_Diaphoretickes-Pyez_4666_OG752
Pp_Diaphoretickes-Pyez_5427_OG752
Pp_Diaphoretickes-Ccri_1385_OG752
Pp_Diaphoretickes-Ccri_1489_OG752
Pp_Diaphoretickes-Ccri_9307_OG752
Pp_Diaphoretickes-Ccri_2673_OG752
Pp_Diaphoretickes-Ccri_5271_OG752
Osmo_Pseudofungi-Alai_12961_OG752_intClade_1
Osmo_Pseudofungi-Alai_4580_OG752_intClade_1
Osmo_Pseudofungi-Alai_6678_OG752_intClade_1
Osmo_Pseudofungi-Alai_5308_OG752_intClade_1
Osmo_Pseudofungi-Alai_3527_OG752_intClade_1
Osmo_Pseudofungi-Alai_5805_OG752_intClade_1
Osmo_Pseudofungi-Alai_3449_OG752_intClade_1
Osmo_Pseudofungi-Alai_4410_OG752_intClade_1
Osmo_Pseudofungi-Pinf_12236_OG752
Osmo_Pseudofungi-Pinf_5153_OG752
Osmo_Pseudofungi-Pinf_12950_OG752
Osmo_Fungi-Pbla_2959_OG752_intClade_1
Osmo_Fungi-Pbla_6218_OG752_intClade_1
Osmo_Fungi-Rory_10013_OG752
Osmo_Fungi-Rory_8901_OG752
Osmo_Fungi-Rory_17105_OG752
Pp_Diaphoretickes-Atha_15313_OG752
Osmo_Fungi-Rory_11319_OG752
Osmo_Fungi-Rory_8023_OG752
Osmo_Pseudofungi-Aast_6899_OG752
Pp_Diaphoretickes-Atha_14892_OG752
Pp_Diaphoretickes-Atha_6361_OG752
Osmo_Fungi-Pbla_2310_OG752
Other_SAR-Pbra_7470_OG752
Osmo_Pseudofungi-Pinf_3627_OG752
Osmo_Pseudofungi-Pinf_11874_OG752
Osmo_Pseudofungi-Pinf_13034_OG752
Osmo_Pseudofungi-Hara_6258_OG752
Osmo_Pseudofungi-Pinf_13467_OG752
Osmo_Pseudofungi-Pinf_6476_OG752
Osmo_Pseudofungi-Pinf_9148_OG752
Osmo_Pseudofungi-Pinf_6572_OG752
Osmo_Pseudofungi-Pinf_4544_OG752
Osmo_Pseudofungi-Pinf_9224_OG752
Osmo_Pseudofungi-Aast_1929_OG752
Osmo_Pseudofungi-Aast_9917_OG752
Osmo_Pseudofungi-Hara_1228_OG752
Osmo_Pseudofungi-Hara_3083_OG752
Osmo_Pseudofungi-Hara_11801_OG752
Osmo_Pseudofungi-Hara_7757_OG752
Osmo_Pseudofungi-Aast_1908_OG752
Osmo_Pseudofungi-Aast_22426_OG752
Osmo_Pseudofungi-Aast_14125_OG752
Osmo_Pseudofungi-Aast_22427_OG752
Osmo_Fungi-Pbla_1684_OG752
Osmo_Pseudofungi-Aast_20766_OG752
Osmo_Pseudofungi-Pinf_7692_OG752
Other_SAR-Pbra_8032_OG752
Other_SAR-Pbra_9021_OG752
Other_SAR-Pbra_7997_OG752
Other_SAR-Pbra_8617_OG752
Other_SAR-Pbra_810_OG752
Osmo_Pseudofungi-Aast_16668_OG752
Osmo_Pseudofungi-Aast_19495_OG752
Osmo_Pseudofungi-Aast_9328_OG752
Osmo_Pseudofungi-Aast_10817_OG752
Osmo_Pseudofungi-Aast_482_OG752
Osmo_Pseudofungi-Aast_292_OG752
Osmo_Pseudofungi-Aast_8288_OG752
Osmo_Pseudofungi-Aast_14876_OG752
Osmo_Pseudofungi-Pinf_1258_OG752
Other_Diaphoretickes-Gavo_8519_OG752
Other_Diaphoretickes-Gavo_17658_OG752
Other_Diaphoretickes-Gavo_7648_OG752
Other_Diaphoretickes-Gavo_14162_OG752
Other_Diaphoretickes-Gavo_6702_OG752
Other_Diaphoretickes-Gavo_18279_OG752
Other_Diaphoretickes-Gavo_2650_OG752
Other_Diaphoretickes-Gavo_13204_OG752
Other_Diaphoretickes-Gavo_18186_OG752
Other_Diaphoretickes-Gavo_17845_OG752
Osmo_Pseudofungi-Alai_3767_OG752_intClade_1
Osmo_Pseudofungi-Aast_2221_OG752
Pp_Diaphoretickes-Ccri_1829_OG752
Pp_Diaphoretickes-Ccri_1740_OG752
Pp_Diaphoretickes-Ccri_1856_OG752
Pp_Diaphoretickes-Ccri_4298_OG752
Pp_Diaphoretickes-Ccri_7191_OG752
Pp_Diaphoretickes-Ccri_5953_OG752
Cp_Diaphoretickes-Esil_3665_OG752
Cp_Diaphoretickes-Esil_1553_OG752
Cp_Diaphoretickes-Esil_12919_OG752
Cp_Diaphoretickes-Aano_16_OG752
Other_Amorphea-Dgra_14934_OG752
Other_Amorphea-Dgra_21984_OG752
Cp_Diaphoretickes-Ghe_8413_OG752
Pp_Diaphoretickes-Otau_6910_OG752
Pp_Diaphoretickes-Otau_6921_OG752
Pp_Diaphoretickes-Otau_4304_OG752
Osmo_Teretosporea-Sarc_8734_OG752
Pp_Diaphoretickes-Pmic_12733_OG752
Pp_Diaphoretickes-Pmic_26637_OG752
Pp_Diaphoretickes-Pmic_8497_OG752
Pp_Diaphoretickes-Pmic_4187_OG752
Pp_Diaphoretickes-Pmic_16801_OG752
Pp_Diaphoretickes-Pmic_1140_OG752
Pp_Diaphoretickes-Pyez_4322_OG752
Osmo_Fungi-Pbla_12870_OG752
Osmo_Pseudofungi-Parr_8876_OG752
0.4

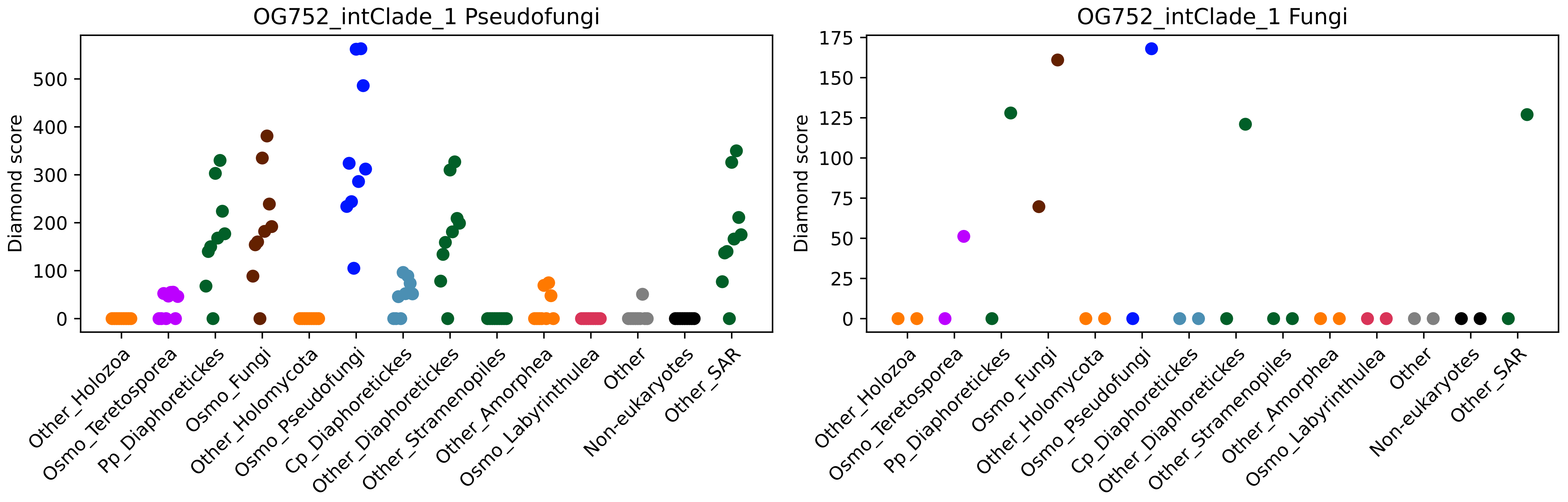

OG752_intClade_1 Pseudofungi
OG752_intClade_1 Fungi
Diamond score
0
100
200
300
400
500
25
50
75
125
150
175
Other_Holozoa
Osmo_Teretosporea
Pp_Diaphoretickes
Osmo_Fungi
Other_Holomycota
Osmo_Pseudofungi
Cp_Diaphoretickes
Other_Diaphoretickes
Other_Stramenopiles
Other_Amorphea
Osmo_Labyrinthulea
Other
Non-eukaryotes
Other_SAR

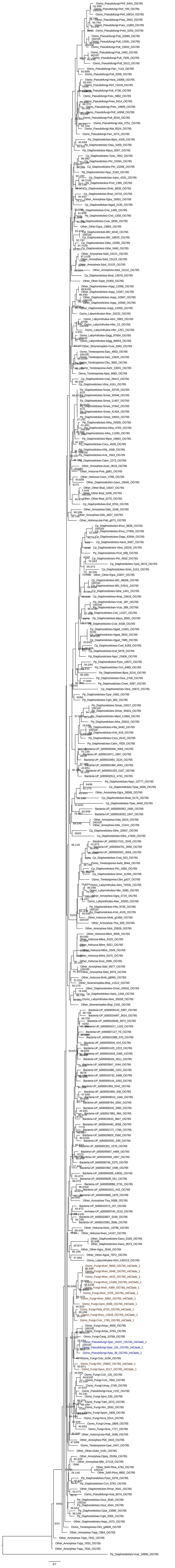

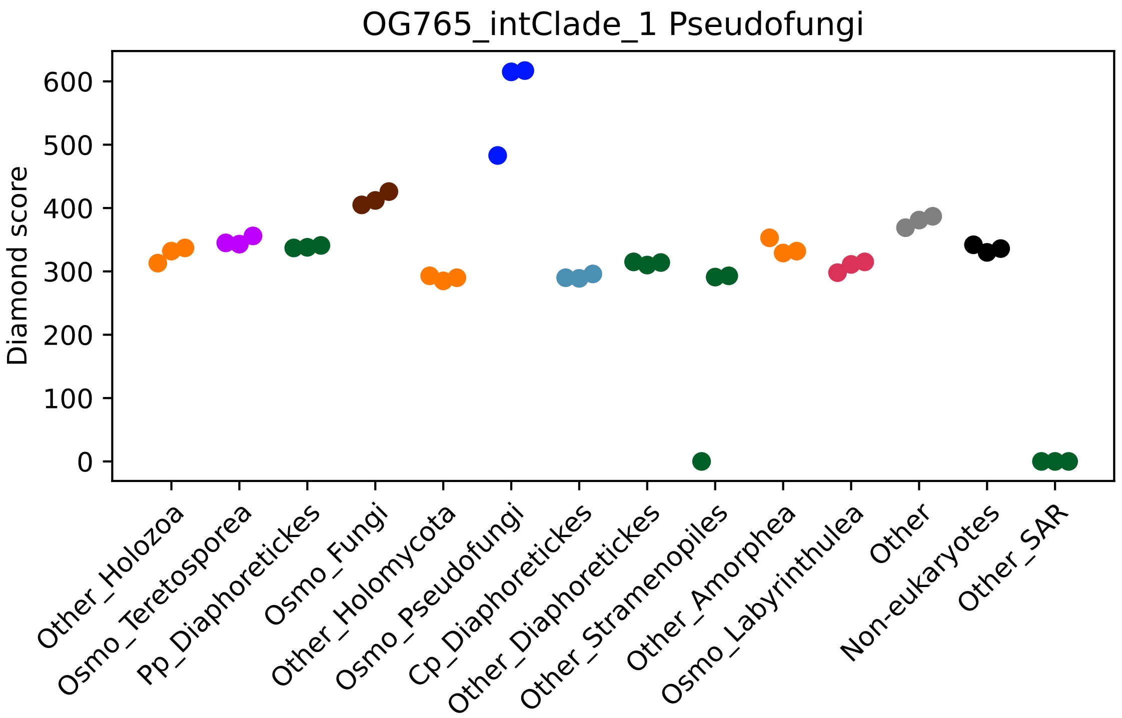

OG765_intClade_1 Pseudofungi
Diamond score
0
100
200
300
400
500
600
Other_Holozoa
Osmo_Teretosporea
Pp_Diaphoretickes
Osmo_Fungi
Other_Holomycota
Osmo_Pseudofungi
Cp_Diaphoretickes
Other_Diaphoretickes
Other_Stramenopiles
Other_Amorphea
Osmo_Labyrinthulea
Other
Non-eukaryotes
Other_SAR

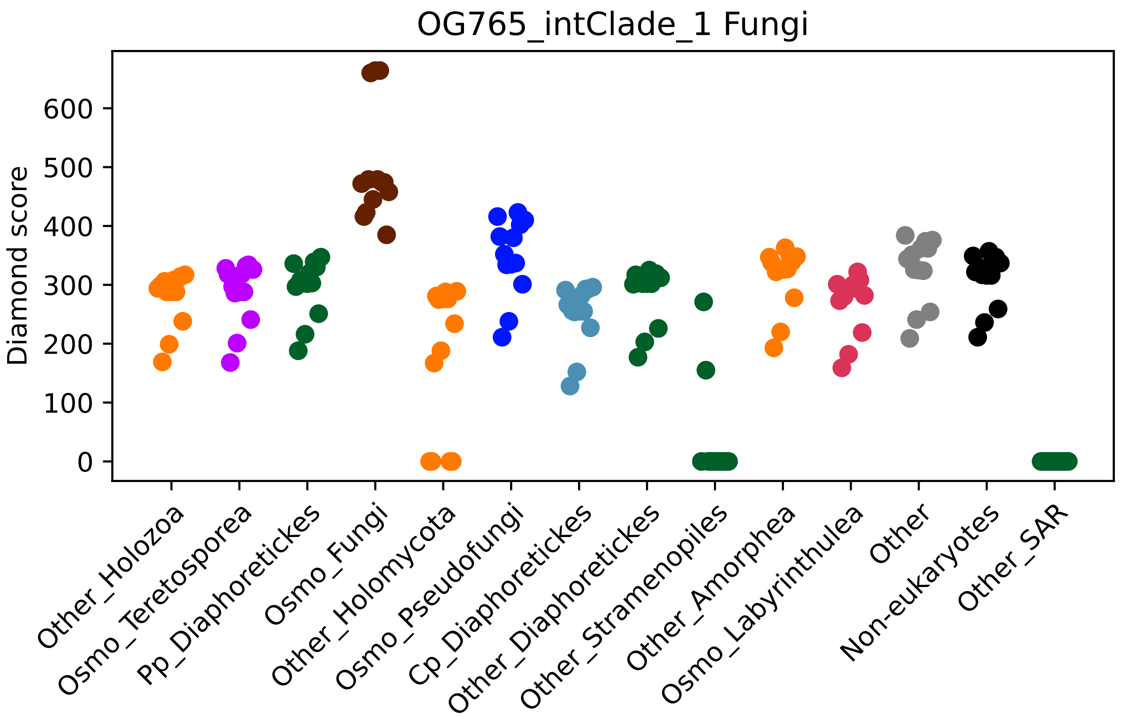

OG765_intClade_1 Fungi
Diamond score
0
100
200
300
400
500
600
Other_Holozoa
Osmo_Teretosporea
Pp_Diaphoretickes
Osmo_Fungi
Other_Holomycota
Osmo_Pseudofungi
Cp_Diaphoretickes
Other_Diaphoretickes
Other_Stramenopiles
Other_Amorphea
Osmo_Labyrinthulea
Other
Non-eukaryotes
Other_SAR

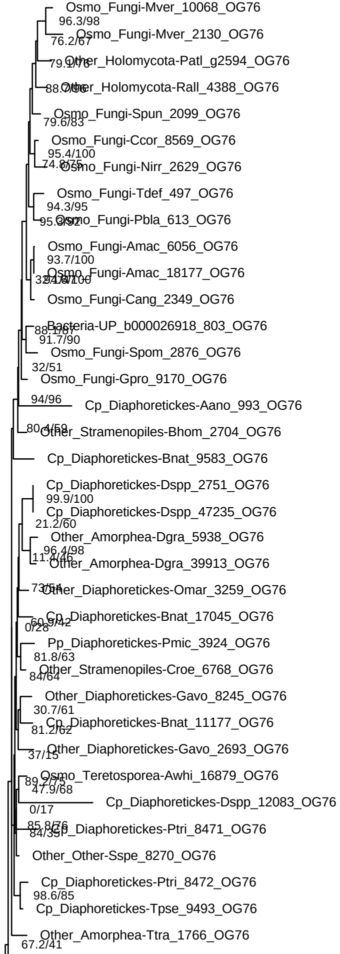
Osmo_Fungi-Mver_10068_OG76
96.3/98
Osmo_Fungi-Mver_2130_OG76
76.2/67
Other_Holomycota-Patl_g2594_OG76
79.1/76
Other_Holomycota-Rall_4388_OG76
88.7/96
Osmo_Fungi-Spun_2099_OG76
79.6/83
Osmo_Fungi-Ccor_8569_OG76
95.4/100
Osmo_Fungi-Nirr_2629_OG76
74.8/75
Osmo_Fungi-Tdef_497_OG76
94.3/95
Osmo_Fungi-Pbla_613_OG76
95.3/92
Osmo_Fungi-Amac_6056_OG76
93.7/100
Osmo_Fungi-Amac_18177_OG76
Osmo_Fungi-Cang_2349_OG76
Bacteria-UP_b000026918_803_OG76
88.1/87
91.7/90
Osmo_Fungi-Spom_2876_OG76
32/51
Osmo_Fungi-Gpro_9170_OG76
94/96
Cp_Diaphoretickes-Aano_993_OG76
80.4/59
Other_Stramenopiles-Bhom_2704_OG76
Cp_Diaphoretickes-Bnat_9583_OG76
Cp_Diaphoretickes-Dspp_2751_OG76
99.9/100
Cp_Diaphoretickes-Dspp_47235_OG76
21.2/60
Other_Amorphea-Dgra_5938_OG76
96.4/98
11.4/46
Other_Amorphea-Dgra_39913_OG76
73/54
Other_Diaphoretickes-Omar_3259_OG76
Cp_Diaphoretickes-Bnat_17045_OG76
60.9/42
0/28
Pp_Diaphoretickes-Pmic_3924_OG76
81.8/63
Other_Stramenopiles-Croe_6768_OG76
84/64
Other_Diaphoretickes-Gavo_8245_OG76
30.7/61
Cp_Diaphoretickes-Bnat_11177_OG76
81.2/62
Other_Diaphoretickes-Gavo_2693_OG76
37/15
Osmo_Teretosporea-Awhi_16879_OG76
89.2/75
47.9/68
Cp_Diaphoretickes-Dspp_12083_OG76
0/17
85.8/76
84/35
Cp_Diaphoretickes-Ptri_8471_OG76
Other_Other-Sspe_8270_OG76
Cp_Diaphoretickes-Ptri_8472_OG76
98.6/85
Cp_Diaphoretickes-Tpse_9493_OG76
Other_Amorphea-Ttra_1766_OG76
67.2/41

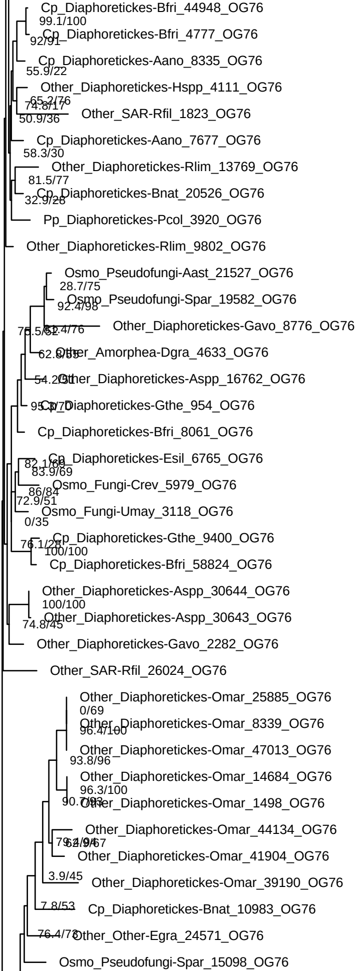
Cp_Diaphoretickes-Bfri_44948_OG76
99.1/100
92/91
Cp_Diaphoretickes-Bfri_4777_OG76
Cp_Diaphoretickes-Aano_8335_OG76
55.9/22
Other_Diaphoretickes-Hspp_4111_OG76
65.2/76
74.8/17
Other_SAR-Rfil_1823_OG76
50.9/36
Cp_Diaphoretickes-Aano_7677_OG76
58.3/30
Other_Diaphoretickes-Rlim_13769_OG76
81.5/77
Cp_Diaphoretickes-Bnat_20526_OG76
32.9/28
Pp_Diaphoretickes-Pcol_3920_OG76
Other_Diaphoretickes-Rlim_9802_OG76
Osmo_Pseudofungi-Aast_21527_OG76
28.7/75
Osmo_Pseudofungi-Spar_19582_OG76
92.4/98
Other_Diaphoretickes-Gavo_8776_OG76
Other_Amorphea-Dgra_4633_OG76
Other_Diaphoretickes-Aspp_16762_OG76
Cp_Diaphoretickes-Gthe_954_OG76
Cp_Diaphoretickes-Bfri_8061_OG76
Cp_Diaphoretickes-Esil_6765_OG76
82.1/69
83.9/69
Osmo_Fungi-Crev_5979_OG76
86/84
72.9/51
Osmo_Fungi-Umay_3118_OG76
0/35
Cp_Diaphoretickes-Gthe_9400_OG76
76.1/28
100/100
Cp_Diaphoretickes-Bfri_58824_OG76
Other_Diaphoretickes-Aspp_30644_OG76
100/100
Other_Diaphoretickes-Aspp_30643_OG76
74.8/45
Other_Diaphoretickes-Gavo_2282_OG76
Other_SAR-Rfil_26024_OG76
Other_Diaphoretickes-Omar_25885_OG76
0/69
Other_Diaphoretickes-Omar_8339_OG76
96.4/100
Other_Diaphoretickes-Omar_47013_OG76
93.8/96
Other_Diaphoretickes-Omar_14684_OG76
96.3/100
Other_Diaphoretickes-Omar_1498_OG76
Other_Diaphoretickes-Omar_44134_OG76
Other_Diaphoretickes-Omar_41904_OG76
3.9/45
Other_Diaphoretickes-Omar_39190_OG76
7.8/53
Cp_Diaphoretickes-Bnat_10983_OG76
76.4/73
Other_Other-Egra_24571_OG76
Osmo_Pseudofungi-Spar_15098_OG76

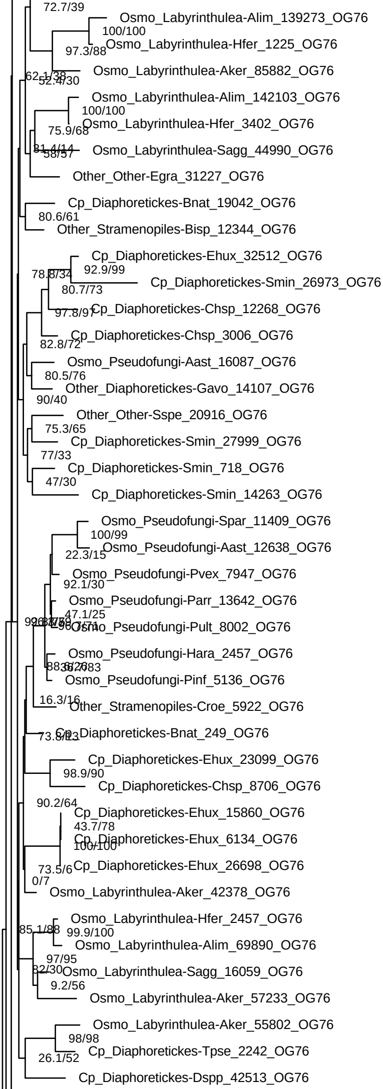
72.7/39
Osmo_Labyrinthulea-Alim_139273_OG76
100/100
Osmo_Labyrinthulea-Hfer_1225_OG76
97.3/88
Osmo_Labyrinthulea-Aker_85882_OG76
Osmo_Labyrinthulea-Alim_142103_OG76
100/100
Osmo_Labyrinthulea-Hfer_3402_OG76
75.9/68
Osmo_Labyrinthulea-Sagg_44990_OG76
58/57
Other_Other-Egra_31227_OG76
Cp_Diaphoretickes-Bnat_19042_OG76
80.6/61
Other_Stramenopiles-Bisp_12344_OG76
Cp_Diaphoretickes-Ehux_32512_OG76
92.9/99
Cp_Diaphoretickes-Smin_26973_OG76
80.7/73
Cp_Diaphoretickes-Chsp_12268_OG76
97.8/97
Cp_Diaphoretickes-Chsp_3006_OG76
82.8/72
Osmo_Pseudofungi-Aast_16087_OG76
80.5/76
Other_Diaphoretickes-Gavo_14107_OG76
90/40
Other_Other-Sspe_20916_OG76
75.3/65
Cp_Diaphoretickes-Smin_27999_OG76
77/33
Cp_Diaphoretickes-Smin_718_OG76
47/30
Cp_Diaphoretickes-Smin_14263_OG76
Osmo_Pseudofungi-Spar_11409_OG76
100/99
Osmo_Pseudofungi-Aast_12638_OG76
22.3/15
Osmo_Pseudofungi-Pvex_7947_OG76
92.1/30
Osmo_Pseudofungi-Parr_13642_OG76
47.1/25
Osmo_Pseudofungi-Pult_8002_OG76
Osmo_Pseudofungi-Hara_2457_OG76
Osmo_Pseudofungi-Pinf_5136_OG76
16.3/16
Other_Stramenopiles-Croe_5922_OG76
Cp_Diaphoretickes-Bnat_249_OG76
Cp_Diaphoretickes-Ehux_23099_OG76
98.9/90
Cp_Diaphoretickes-Chsp_8706_OG76
90.2/64
Cp_Diaphoretickes-Ehux_15860_OG76
43.7/78
Cp_Diaphoretickes-Ehux_6134_OG76
100/100
Cp_Diaphoretickes-Ehux_26698_OG76
73.5/6
0/7
Osmo_Labyrinthulea-Aker_42378_OG76
Osmo_Labyrinthulea-Hfer_2457_OG76
85.1/88
99.9/100
Osmo_Labyrinthulea-Alim_69890_OG76
97/95
82/30
Osmo_Labyrinthulea-Sagg_16059_OG76
9.2/56
Osmo_Labyrinthulea-Aker_57233_OG76
Osmo_Labyrinthulea-Aker_55802_OG76
98/98
Cp_Diaphoretickes-Tpse_2242_OG76
26.1/52
Cp_Diaphoretickes-Dspp_42513_OG76

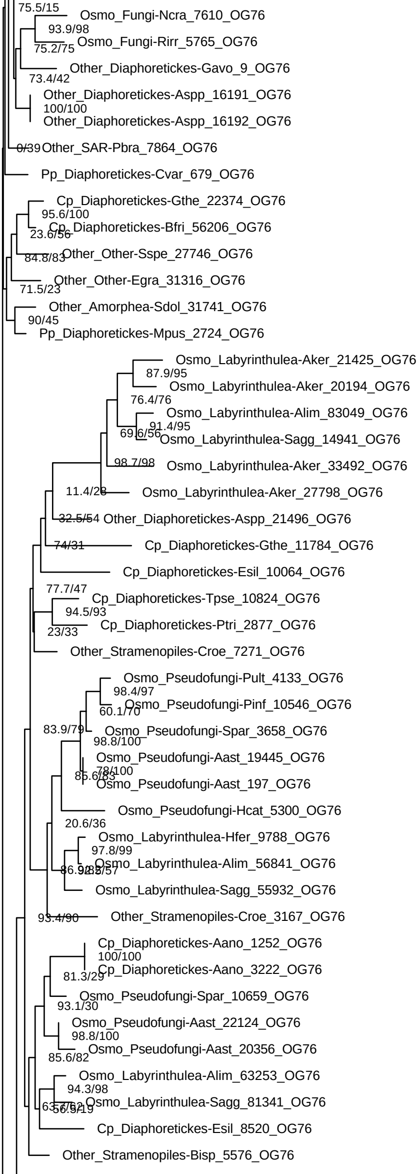

75.5/15
Osmo_Fungi-Ncra_7610_OG76
93.9/98
Osmo_Fungi-Rirr_5765_OG76
75.2/75
Other_Diaphoretickes-Gavo_9_OG76
73.4/42
Other_Diaphoretickes-Aspp_16191_OG76
100/100
Other_Diaphoretickes-Aspp_16192_OG76
0/39
Other_SAR-Pbra_7864_OG76
Pp_Diaphoretickes-Cvar_679_OG76
Cp_Diaphoretickes-Gthe_22374_OG76
95.6/100
Cp_Diaphoretickes-Bfri_56206_OG76
23.6/56
Other_Other-Sspe_27746_OG76
84.8/83
Other_Other-Egra_31316_OG76
71.5/23
Other_Amorphea-Sdol_31741_OG76
90/45
Pp_Diaphoretickes-Mpus_2724_OG76
Osmo_Labyrinthulea-Aker_21425_OG76
87.9/95
Osmo_Labyrinthulea-Aker_20194_OG76
76.4/76
Osmo_Labyrinthulea-Alim_83049_OG76
91.4/95
69.6/56
Osmo_Labyrinthulea-Sagg_14941_OG76
98.7/98
Osmo_Labyrinthulea-Aker_33492_OG76
11.4/28
Osmo_Labyrinthulea-Aker_27798_OG76
32.5/54
Other_Diaphoretickes-Aspp_21496_OG76
74/31
Cp_Diaphoretickes-Gthe_11784_OG76
Cp_Diaphoretickes-Esil_10064_OG76
77.7/47
Cp_Diaphoretickes-Tpse_10824_OG76
94.5/93
Cp_Diaphoretickes-Ptri_2877_OG76
23/33
Other_Stramenopiles-Croe_7271_OG76
Osmo_Pseudofungi-Pult_4133_OG76
98.4/97
Osmo_Pseudofungi-Pinf_10546_OG76
60.1/70
83.9/79
Osmo_Pseudofungi-Spar_3658_OG76
98.8/100
Osmo_Pseudofungi-Aast_19445_OG76
78/100
85.6/83
Osmo_Pseudofungi-Aast_197_OG76
Osmo_Pseudofungi-Hcat_5300_OG76
20.6/36
Osmo_Labyrinthulea-Hfer_9788_OG76
97.8/99
Osmo_Labyrinthulea-Alim_56841_OG76
86.9/88.5/57
Osmo_Labyrinthulea-Sagg_55932_OG76
93.4/90
Other_Stramenopiles-Croe_3167_OG76
Cp_Diaphoretickes-Aano_1252_OG76
100/100
Cp_Diaphoretickes-Aano_3222_OG76
81.3/29
Osmo_Pseudofungi-Spar_10659_OG76
93.1/30
Osmo_Pseudofungi-Aast_22124_OG76
98.8/100
Osmo_Pseudofungi-Aast_20356_OG76
85.6/82
Osmo_Labyrinthulea-Alim_63253_OG76
94.3/98
63.7/62
56.9/19
Osmo_Labyrinthulea-Sagg_81341_OG76
Cp_Diaphoretickes-Esil_8520_OG76
Other_Stramenopiles-Bisp_5576_OG76

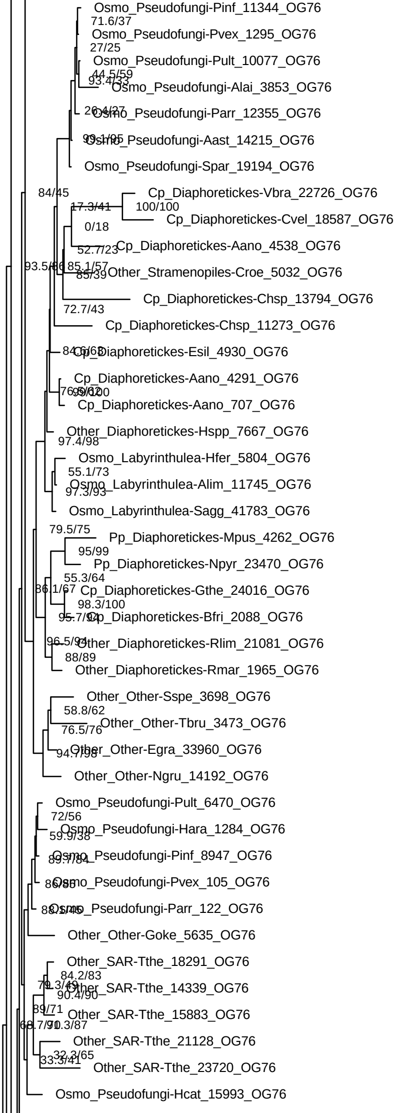

Osmo_Pseudofungi-Pinf_11344_OG76
71.6/37
Osmo_Pseudofungi-Pvex_1295_OG76
27/25
Osmo_Pseudofungi-Pult_10077_OG76
44.5/59
93.4/33
Osmo_Pseudofungi-Alai_3853_OG76
Osmo_Pseudofungi-Parr_12355_OG76
Osmo_Pseudofungi-Aast_14215_OG76
Osmo_Pseudofungi-Spar_19194_OG76
84/45
Cp_Diaphoretickes-Vbra_22726_OG76
17.3/41
100/100
Cp_Diaphoretickes-Cvel_18587_OG76
0/18
Cp_Diaphoretickes-Aano_4538_OG76
52.7/23
93.5/86
85.1/57
85/39
Other_Stramenopiles-Croe_5032_OG76
Cp_Diaphoretickes-Chsp_13794_OG76
72.7/43
Cp_Diaphoretickes-Chsp_11273_OG76
Cp_Diaphoretickes-Esil_4930_OG76
Cp_Diaphoretickes-Aano_4291_OG76
Cp_Diaphoretickes-Aano_707_OG76
Other_Diaphoretickes-Hspp_7667_OG76
97.4/98
Osmo_Labyrinthulea-Hfer_5804_OG76
55.1/73
Osmo_Labyrinthulea-Alim_11745_OG76
97.3/93
Osmo_Labyrinthulea-Sagg_41783_OG76
79.5/75
Pp_Diaphoretickes-Mpus_4262_OG76
95/99
Pp_Diaphoretickes-Npyr_23470_OG76
55.3/64
86.1/67
Cp_Diaphoretickes-Gthe_24016_OG76
98.3/100
Cp_Diaphoretickes-Bfri_2088_OG76
Other_Diaphoretickes-Rlim_21081_OG76
88/89
Other_Diaphoretickes-Rmar_1965_OG76
Other_Other-Sspe_3698_OG76
58.8/62
Other_Other-Tbru_3473_OG76
76.5/76
Other_Other-Egra_33960_OG76
Other_Other-Ngru_14192_OG76
Osmo_Pseudofungi-Pult_6470_OG76
72/56
Osmo_Pseudofungi-Hara_1284_OG76
59.9/38
Osmo_Pseudofungi-Pinf_8947_OG76
Osmo_Pseudofungi-Pvex_105_OG76
Osmo_Pseudofungi-Parr_122_OG76
Other_Other-Goke_5635_OG76
Other_SAR-Tthe_18291_OG76
84.2/83
Other_SAR-Tthe_14339_OG76
90.4/90
Other_SAR-Tthe_15883_OG76
89/71
Other_SAR-Tthe_21128_OG76
32.3/65
33.3/41
Other_SAR-Tthe_23720_OG76
Osmo_Pseudofungi-Hcat_15993_OG76

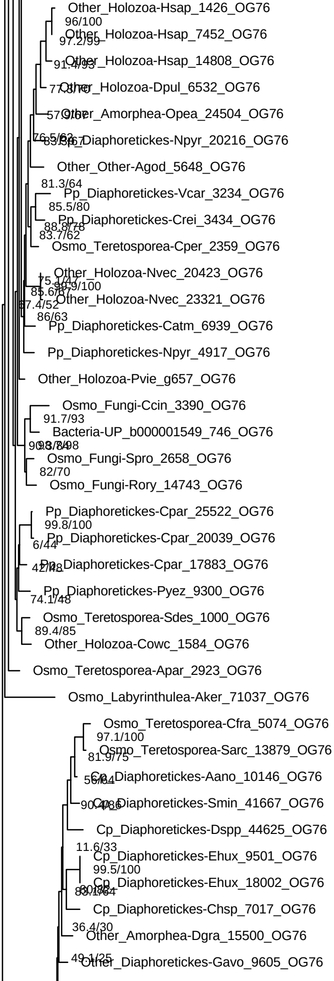
Other_Holozoa-Hsap_1426_OG76
96/100
Other_Holozoa-Hsap_7452_OG76
97.2/99
Other_Holozoa-Hsap_14808_OG76
91.4/93
Other_Holozoa-Dpul_6532_OG76
Other_Amorphea-Opea_24504_OG76
Pp_Diaphoretickes-Npyr_20216_OG76
Other_Other-Agod_5648_OG76
81.3/64
Pp_Diaphoretickes-Vcar_3234_OG76
85.5/80
Pp_Diaphoretickes-Crei_3434_OG76
88.8/78
83.7/62
Osmo_Teretosporea-Cper_2359_OG76
Other_Holozoa-Nvec_20423_OG76
99.9/100
Other_Holozoa-Nvec_23321_OG76
86/63
Pp_Diaphoretickes-Catm_6939_OG76
Pp_Diaphoretickes-Npyr_4917_OG76
Other_Holozoa-Pvie_g657_OG76
Osmo_Fungi-Ccin_3390_OG76
91.7/93
Bacteria-UP_b000001549_746_OG76
Osmo_Fungi-Spro_2658_OG76
82/70
Osmo_Fungi-Rory_14743_OG76
Pp_Diaphoretickes-Cpar_25522_OG76
99.8/100
Pp_Diaphoretickes-Cpar_20039_OG76
6/44
Pp_Diaphoretickes-Cpar_17883_OG76
Pp_Diaphoretickes-Pyez_9300_OG76
74.1/48
Osmo_Teretosporea-Sdes_1000_OG76
89.4/85
Other_Holozoa-Cowc_1584_OG76
Osmo_Teretosporea-Apar_2923_OG76
Osmo_Labyrinthulea-Aker_71037_OG76
Osmo_Teretosporea-Cfra_5074_OG76
97.1/100
Osmo_Teretosporea-Sarc_13879_OG76
81.9/75
Cp_Diaphoretickes-Aano_10146_OG76
Cp_Diaphoretickes-Smin_41667_OG76
Cp_Diaphoretickes-Dspp_44625_OG76
11.6/33
Cp_Diaphoretickes-Ehux_9501_OG76
99.5/100
Cp_Diaphoretickes-Ehux_18002_OG76
Cp_Diaphoretickes-Chsp_7017_OG76
36.4/30
Other_Amorphea-Dgra_15500_OG76
Other_Diaphoretickes-Gavo_9605_OG76

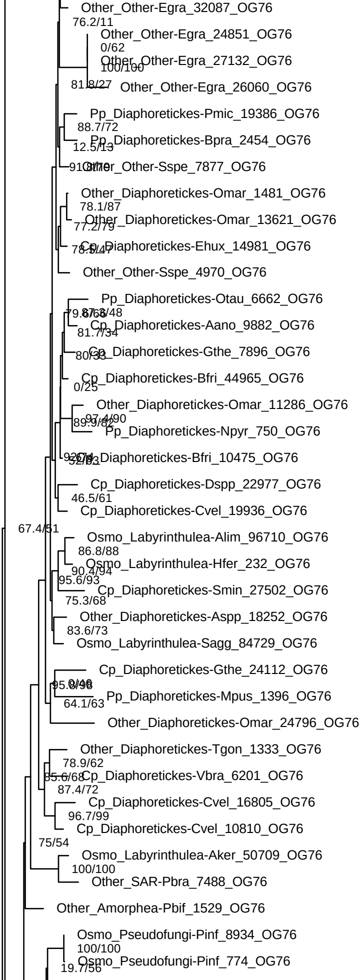

Other_Other-Egra_32087_OG76
76.2/11
Other_Other-Egra_24851_OG76
0/62
Other_Other-Egra_27132_OG76
100/100
81.8/27
Other_Other-Egra_26060_OG76
Pp_Diaphoretickes-Pmic_19386_OG76
88.7/72
Pp_Diaphoretickes-Bpra_2454_OG76
12.5/13
Other_Other-Sspe_7877_OG76
Other_Diaphoretickes-Omar_1481_OG76
78.1/87
Other_Diaphoretickes-Omar_13621_OG76
77.2/79
Cp_Diaphoretickes-Ehux_14981_OG76
Other_Other-Sspe_4970_OG76
Pp_Diaphoretickes-Otau_6662_OG76
Cp_Diaphoretickes-Aano_9882_OG76
81.7/34
Cp_Diaphoretickes-Gthe_7896_OG76
80/33
Cp_Diaphoretickes-Bfri_44965_OG76
0/25
Other_Diaphoretickes-Omar_11286_OG76
97.4/90
89.9/82
Pp_Diaphoretickes-Npyr_750_OG76
Cp_Diaphoretickes-Bfri_10475_OG76
Cp_Diaphoretickes-Dspp_22977_OG76
46.5/61
Cp_Diaphoretickes-Cvel_19936_OG76
67.4/51
Osmo_Labyrinthulea-Alim_96710_OG76
86.8/88
Osmo_Labyrinthulea-Hfer_232_OG76
90.4/94
95.6/93
Cp_Diaphoretickes-Smin_27502_OG76
75.3/68
Other_Diaphoretickes-Aspp_18252_OG76
83.6/73
Osmo_Labyrinthulea-Sagg_84729_OG76
Cp_Diaphoretickes-Gthe_24112_OG76
Pp_Diaphoretickes-Mpus_1396_OG76
64.1/63
Other_Diaphoretickes-Omar_24796_OG76
Other_Diaphoretickes-Tgon_1333_OG76
78.9/62
85.6/68
Cp_Diaphoretickes-Vbra_6201_OG76
87.4/72
Cp_Diaphoretickes-Cvel_16805_OG76
96.7/99
Cp_Diaphoretickes-Cvel_10810_OG76
75/54
Osmo_Labyrinthulea-Aker_50709_OG76
100/100
Other_SAR-Pbra_7488_OG76
Other_Amorphea-Pbif_1529_OG76
Osmo_Pseudofungi-Pinf_8934_OG76
100/100
Osmo_Pseudofungi-Pinf_774_OG76
19.7/56

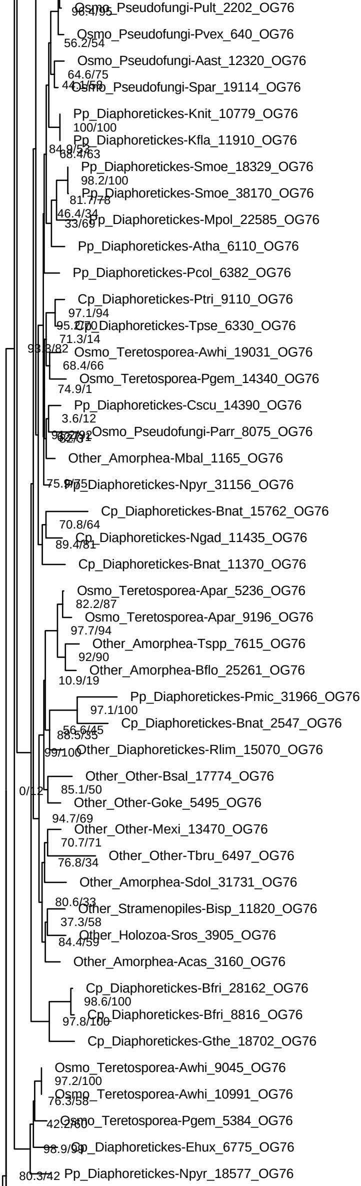

Osmo_Pseudofungi-Pult_2202_OG76
98.4/95
Osmo_Pseudofungi-Pvex_640_OG76
56.2/54
Osmo_Pseudofungi-Aast_12320_OG76
64.6/75
44.1/58
Osmo_Pseudofungi-Spar_19114_OG76
Pp_Diaphoretickes-Knit_10779_OG76
100/100
Pp_Diaphoretickes-Kfla_11910_OG76
84.9/53
68.4/63
Pp_Diaphoretickes-Smoe_18329_OG76
98.2/100
Pp_Diaphoretickes-Smoe_38170_OG76
81.7/78
46.4/34
33/69
Pp_Diaphoretickes-Mpol_22585_OG76
Pp_Diaphoretickes-Atha_6110_OG76
Pp_Diaphoretickes-Pcol_6382_OG76
Cp_Diaphoretickes-Ptri_9110_OG76
97.1/94
95.2/70
Cp_Diaphoretickes-Tpse_6330_OG76
71.3/14
93.3/82
Osmo_Teretosporea-Awhi_19031_OG76
68.4/66
Osmo_Teretosporea-Pgem_14340_OG76
74.9/1
Pp_Diaphoretickes-Cscu_14390_OG76
3.6/12
Osmo_Pseudofungi-Parr_8075_OG76
Other_Amorphea-Mbal_1165_OG76
75.9/75
Pp_Diaphoretickes-Npyr_31156_OG76
Cp_Diaphoretickes-Bnat_15762_OG76
70.8/64
Cp_Diaphoretickes-Ngad_11435_OG76
89.4/81
Cp_Diaphoretickes-Bnat_11370_OG76
Osmo_Teretosporea-Apar_5236_OG76
82.2/87
Osmo_Teretosporea-Apar_9196_OG76
97.7/94
Other_Amorphea-Tspp_7615_OG76
92/90
Other_Amorphea-Bflo_25261_OG76
10.9/19
Pp_Diaphoretickes-Pmic_31966_OG76
97.1/100
Cp_Diaphoretickes-Bnat_2547_OG76
56.6/45
88.5/35
99/100
Other_Diaphoretickes-Rlim_15070_OG76
Other_Other-Bsal_17774_OG76
85.1/50
0/12
Other_Other-Goke_5495_OG76
94.7/69
Other_Other-Mexi_13470_OG76
70.7/71
Other_Other-Tbru_6497_OG76
76.8/34
Other_Amorphea-Sdol_31731_OG76
80.6/33
Other_Stramenopiles-Bisp_11820_OG76
37.3/58
Other_Holozoa-Sros_3905_OG76
84.4/59
Other_Amorphea-Acas_3160_OG76
Cp_Diaphoretickes-Bfri_28162_OG76
98.6/100
Cp_Diaphoretickes-Bfri_8816_OG76
97.8/100
Cp_Diaphoretickes-Gthe_18702_OG76
Osmo_Teretosporea-Awhi_9045_OG76
97.2/100
Osmo_Teretosporea-Awhi_10991_OG76
76.3/58
Osmo_Teretosporea-Pgem_5384_OG76
42.2/60
Cp_Diaphoretickes-Ehux_6775_OG76
98.9/94
Pp_Diaphoretickes-Npyr_18577_OG76
80.3/42

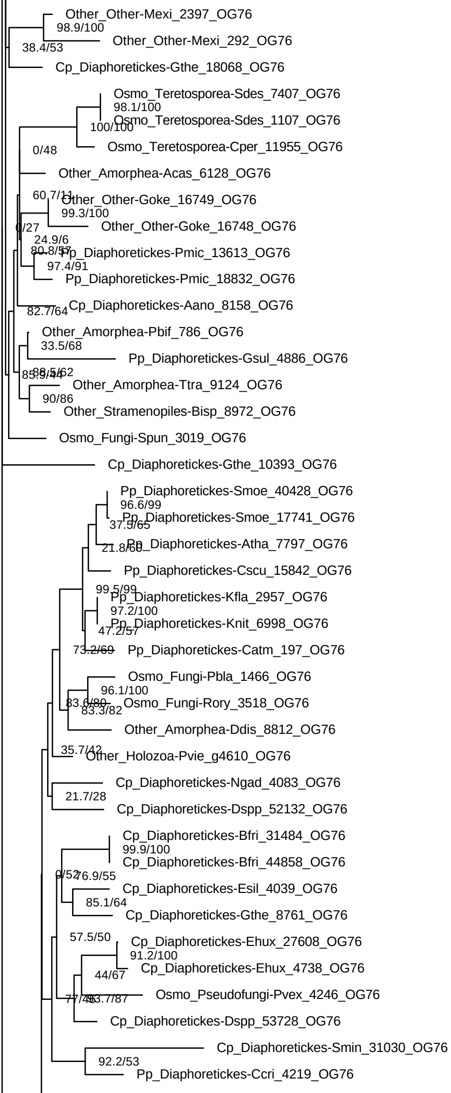

Other_Other-Mexi_2397_OG76
98.9/100
Other_Other-Mexi_292_OG76
38.4/53
Cp_Diaphoretickes-Gthe_18068_OG76
Osmo_Teretosporea-Sdes_7407_OG76
98.1/100
Osmo_Teretosporea-Sdes_1107_OG76
100/100
Osmo_Teretosporea-Cper_11955_OG76
0/48
Other_Amorphea-Acas_6128_OG76
60.7/11
Other_Other-Goke_16749_OG76
99.3/100
Other_Other-Goke_16748_OG76
0/27
24.9/6
80.8/57
Pp_Diaphoretickes-Pmic_13613_OG76
97.4/91
Pp_Diaphoretickes-Pmic_18832_OG76
Cp_Diaphoretickes-Aano_8158_OG76
82.7/64
Other_Amorphea-Pbif_786_OG76
33.5/68
Pp_Diaphoretickes-Gsul_4886_OG76
85.9/44
88.5/62
Other_Amorphea-Ttra_9124_OG76
90/86
Other_Stramenopiles-Bisp_8972_OG76
Osmo_Fungi-Spun_3019_OG76
Cp_Diaphoretickes-Gthe_10393_OG76
Pp_Diaphoretickes-Smoe_40428_OG76
96.6/99
Pp_Diaphoretickes-Smoe_17741_OG76
37.5/65
Pp_Diaphoretickes-Atha_7797_OG76
21.8/60
Pp_Diaphoretickes-Cscu_15842_OG76
99.5/99
Pp_Diaphoretickes-Kfla_2957_OG76
97.2/100
Pp_Diaphoretickes-Knit_6998_OG76
47.2/57
73.2/69
Pp_Diaphoretickes-Catm_197_OG76
Osmo_Fungi-Pbla_1466_OG76
96.1/100
83.6/80
83.3/82
Osmo_Fungi-Rory_3518_OG76
Other_Amorphea-Ddis_8812_OG76
35.7/42
Other_Holozoa-Pvie_g4610_OG76
Cp_Diaphoretickes-Ngad_4083_OG76
21.7/28
Cp_Diaphoretickes-Dspp_52132_OG76
Cp_Diaphoretickes-Bfri_31484_OG76
99.9/100
Cp_Diaphoretickes-Bfri_44858_OG76
0/52
76.9/55
Cp_Diaphoretickes-Esil_4039_OG76
85.1/64
Cp_Diaphoretickes-Gthe_8761_OG76
57.5/50
Cp_Diaphoretickes-Ehux_27608_OG76
91.2/100
Cp_Diaphoretickes-Ehux_4738_OG76
44/67
77/49
63.7/87
Osmo_Pseudofungi-Pvex_4246_OG76
Cp_Diaphoretickes-Dspp_53728_OG76
Cp_Diaphoretickes-Smin_31030_OG76
92.2/53
Pp_Diaphoretickes-Ccri_4219_OG76

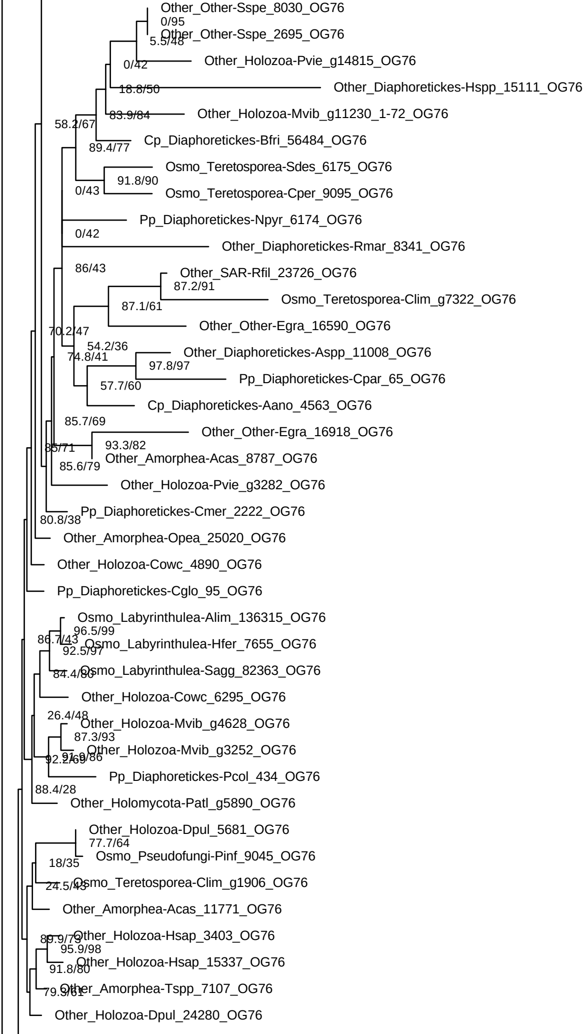

Other_Other-Sspe_8030_OG76
0/95
Other_Other-Sspe_2695_OG76
5.5/48
Other_Holozoa-Pvie_g14815_OG76
0/42
Other_Diaphoretickes-Hspp_15111_OG76
18.8/50
Other_Holozoa-Mvib_g11230_1-72_OG76
83.9/84
58.2/67
Cp_Diaphoretickes-Bfri_56484_OG76
89.4/77
Osmo_Teretosporea-Sdes_6175_OG76
91.8/90
Osmo_Teretosporea-Cper_9095_OG76
0/43
Pp_Diaphoretickes-Npyr_6174_OG76
0/42
Other_Diaphoretickes-Rmar_8341_OG76
86/43
Other_SAR-Rfil_23726_OG76
87.2/91
Osmo_Teretosporea-Clim_g7322_OG76
87.1/61
Other_Other-Egra_16590_OG76
70.2/47
54.2/36
74.8/41
Other_Diaphoretickes-Aspp_11008_OG76
97.8/97
Pp_Diaphoretickes-Cpar_65_OG76
57.7/60
Cp_Diaphoretickes-Aano_4563_OG76
85.7/69
Other_Other-Egra_16918_OG76
93.3/82
85/71
85.6/79
Other_Amorphea-Acas_8787_OG76
Other_Holozoa-Pvie_g3282_OG76
Pp_Diaphoretickes-Cmer_2222_OG76
80.8/38
Other_Amorphea-Opea_25020_OG76
Other_Holozoa-Cowc_4890_OG76
Pp_Diaphoretickes-Cglo_95_OG76
Osmo_Labyrinthulea-Alim_136315_OG76
96.5/99
86.7/43
Osmo_Labyrinthulea-Hfer_7655_OG76
92.5/97
Osmo_Labyrinthulea-Sagg_82363_OG76
84.4/80
Other_Holozoa-Cowc_6295_OG76
26.4/48
Other_Holozoa-Mvib_g4628_OG76
87.3/93
Other_Holozoa-Mvib_g3252_OG76
92.2/69
91.9/86
Pp_Diaphoretickes-Pcol_434_OG76
88.4/28
Other_Holomycota-Patl_g5890_OG76
Other_Holozoa-Dpul_5681_OG76
77.7/64
Osmo_Pseudofungi-Pinf_9045_OG76
18/35
Osmo_Teretosporea-Clim_g1906_OG76
24.5/43
Other_Amorphea-Acas_11771_OG76
89.9/73
Other_Holozoa-Hsap_3403_OG76
95.9/98
Other_Holozoa-Hsap_15337_OG76
91.8/80
Other_Amorphea-Tspp_7107_OG76
79.3/61
Other_Holozoa-Dpul_24280_OG76

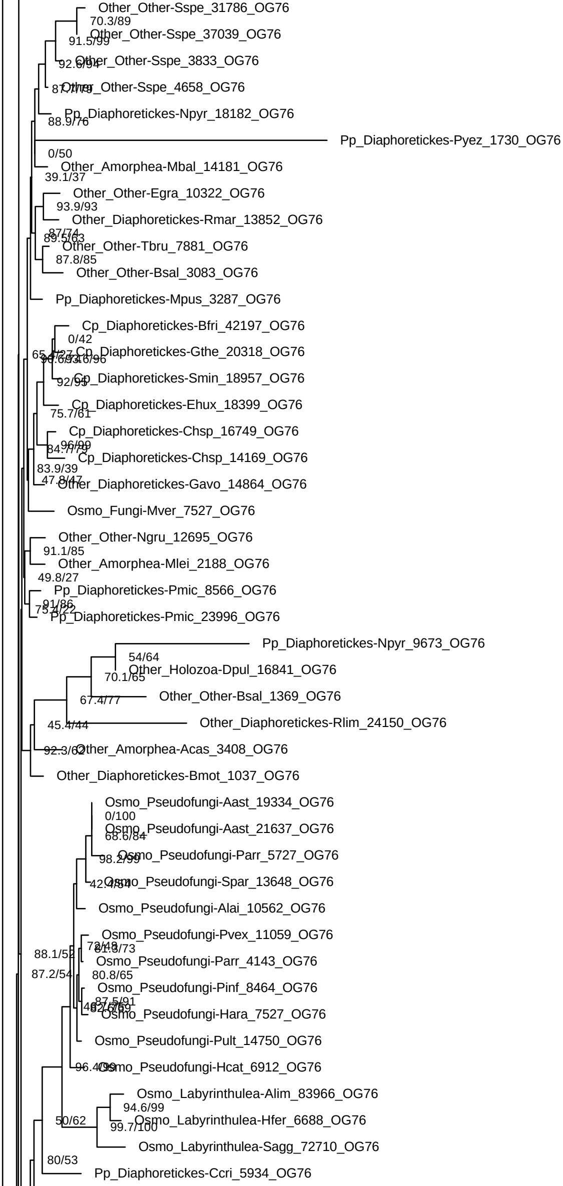
Other_Other-Sspe_31786_OG76
70.3/89
Other_Other-Sspe_37039_OG76
91.5/99
Other_Other-Sspe_3833_OG76
Other_Other-Sspe_4658_OG76
Pp_Diaphoretickes-Npyr_18182_OG76
88.9/76
Pp_Diaphoretickes-Pyez_1730_OG76
0/50
Other_Amorphea-Mbal_14181_OG76
39.1/37
Other_Other-Egra_10322_OG76
93.9/93
Other_Diaphoretickes-Rmar_13852_OG76
Other_Other-Tbru_7881_OG76
87.8/85
Other_Other-Bsal_3083_OG76
Pp_Diaphoretickes-Mpus_3287_OG76
Cp_Diaphoretickes-Bfri_42197_OG76
0/42
Cp_Diaphoretickes-Gthe_20318_OG76
Cp_Diaphoretickes-Smin_18957_OG76
Cp_Diaphoretickes-Ehux_18399_OG76
75.7/61
Cp_Diaphoretickes-Chsp_16749_OG76
Cp_Diaphoretickes-Chsp_14169_OG76
83.9/39
Other_Diaphoretickes-Gavo_14864_OG76
Osmo_Fungi-Mver_7527_OG76
Other_Other-Ngru_12695_OG76
91.1/85
Other_Amorphea-Mlei_2188_OG76
49.8/27
Pp_Diaphoretickes-Pmic_8566_OG76
Pp_Diaphoretickes-Pmic_23996_OG76
Pp_Diaphoretickes-Npyr_9673_OG76
54/64
Other_Holozoa-Dpul_16841_OG76
70.1/65
Other_Other-Bsal_1369_OG76
67.4/77
Other_Diaphoretickes-Rlim_24150_OG76
45.4/44
Other_Amorphea-Acas_3408_OG76
Other_Diaphoretickes-Bmot_1037_OG76
Osmo_Pseudofungi-Aast_19334_OG76
0/100
Osmo_Pseudofungi-Aast_21637_OG76
Osmo_Pseudofungi-Parr_5727_OG76
Osmo_Pseudofungi-Spar_13648_OG76
Osmo_Pseudofungi-Alai_10562_OG76
Osmo_Pseudofungi-Pvex_11059_OG76
Osmo_Pseudofungi-Parr_4143_OG76
88.1/52
87.2/54
80.8/65
Osmo_Pseudofungi-Pinf_8464_OG76
Osmo_Pseudofungi-Hara_7527_OG76
Osmo_Pseudofungi-Pult_14750_OG76
Osmo_Pseudofungi-Hcat_6912_OG76
Osmo_Labyrinthulea-Alim_83966_OG76
94.6/99
Osmo_Labyrinthulea-Hfer_6688_OG76
50/62
99.7/100
Osmo_Labyrinthulea-Sagg_72710_OG76
80/53
Pp_Diaphoretickes-Ccri_5934_OG76

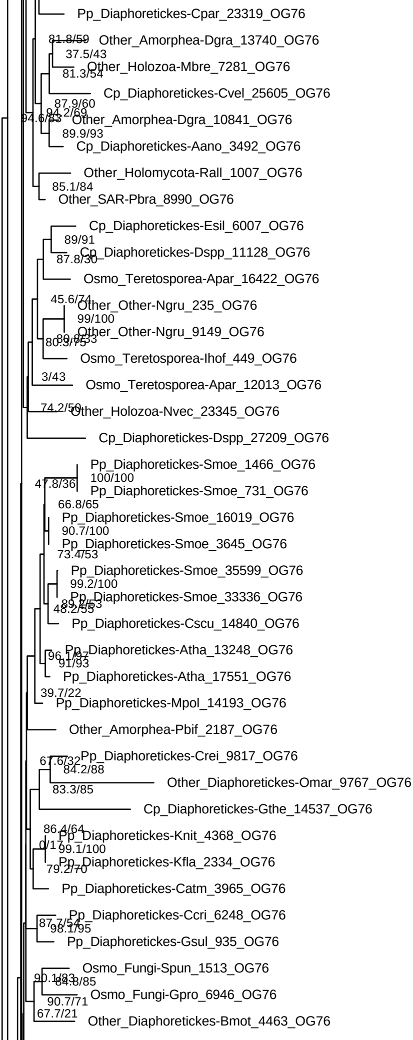

Pp_Diaphoretickes-Cpar_23319_OG76
81.8/59
Other_Amorphea-Dgra_13740_OG76
37.5/43
Other_Holozoa-Mbre_7281_OG76
81.3/54
Cp_Diaphoretickes-Cvel_25605_OG76
87.9/60
94.2/69
94.6/83
Other_Amorphea-Dgra_10841_OG76
89.9/93
Cp_Diaphoretickes-Aano_3492_OG76
Other_Holomycota-Rall_1007_OG76
85.1/84
Other_SAR-Pbra_8990_OG76
Cp_Diaphoretickes-Esil_6007_OG76
89/91
Cp_Diaphoretickes-Dspp_11128_OG76
87.8/30
Osmo_Teretosporea-Apar_16422_OG76
45.6/74
Other_Other-Ngru_235_OG76
99/100
Other_Other-Ngru_9149_OG76
80.3/75
Osmo_Teretosporea-Ihof_449_OG76
3/43
Osmo_Teretosporea-Apar_12013_OG76
74.2/50
Other_Holozoa-Nvec_23345_OG76
Cp_Diaphoretickes-Dspp_27209_OG76
Pp_Diaphoretickes-Smoe_1466_OG76
100/100
47.8/36
Pp_Diaphoretickes-Smoe_731_OG76
66.8/65
Pp_Diaphoretickes-Smoe_16019_OG76
90.7/100
Pp_Diaphoretickes-Smoe_3645_OG76
73.4/53
Pp_Diaphoretickes-Smoe_35599_OG76
99.2/100
Pp_Diaphoretickes-Smoe_33336_OG76
48.2/55
Pp_Diaphoretickes-Cscu_14840_OG76
Pp_Diaphoretickes-Atha_13248_OG76
96.1/97
91/93
Pp_Diaphoretickes-Atha_17551_OG76
39.7/22
Pp_Diaphoretickes-Mpol_14193_OG76
Other_Amorphea-Pbif_2187_OG76
67.6/32
Pp_Diaphoretickes-Crei_9817_OG76
84.2/88
Other_Diaphoretickes-Omar_9767_OG76
83.3/85
Cp_Diaphoretickes-Gthe_14537_OG76
86.4/64
Pp_Diaphoretickes-Knit_4368_OG76
0/17
99.1/100
Pp_Diaphoretickes-Kfla_2334_OG76
79.2/70
Pp_Diaphoretickes-Catm_3965_OG76
Pp_Diaphoretickes-Ccri_6248_OG76
87.7/54
98.1/95
Pp_Diaphoretickes-Gsul_935_OG76
Osmo_Fungi-Spun_1513_OG76
90.1/83
84.8/85
Osmo_Fungi-Gpro_6946_OG76
90.7/71
67.7/21
Other_Diaphoretickes-Bmot_4463_OG76

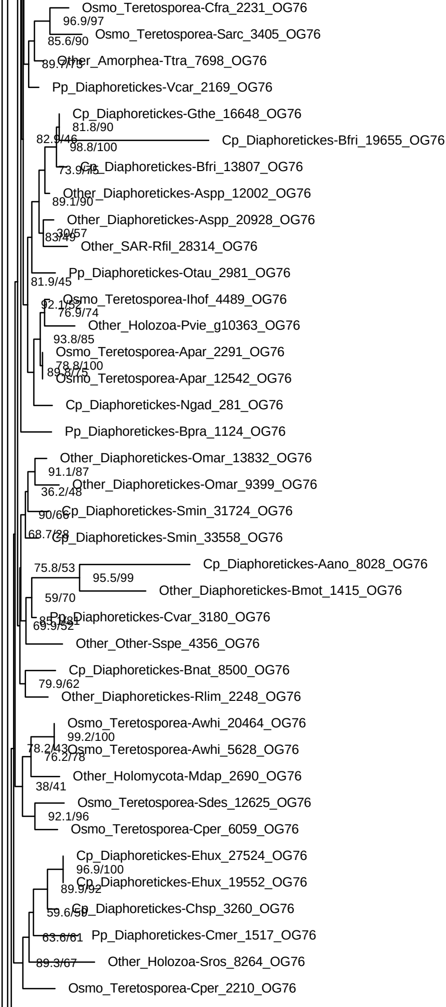
Osmo_Teretosporea-Cfra_2231_OG76
96.9/97
Osmo_Teretosporea-Sarc_3405_OG76
85.6/90
Other_Amorphea-Ttra_7698_OG76
89.7/73
Pp_Diaphoretickes-Vcar_2169_OG76
Cp_Diaphoretickes-Gthe_16648_OG76
81.8/90
82.9/46
Cp_Diaphoretickes-Bfri_19655_OG76
98.8/100
Cp_Diaphoretickes-Bfri_13807_OG76
73.9/75
Other_Diaphoretickes-Aspp_12002_OG76
89.1/90
Other_Diaphoretickes-Aspp_20928_OG76
30/57
83/49
Other_SAR-Rfil_28314_OG76
Pp_Diaphoretickes-Otau_2981_OG76
81.9/45
Osmo_Teretosporea-Ihof_4489_OG76
92.1/52
76.9/74
Other_Holozoa-Pvie_g10363_OG76
93.8/85
Osmo_Teretosporea-Apar_2291_OG76
78.8/100
89.8/75
Osmo_Teretosporea-Apar_12542_OG76
Cp_Diaphoretickes-Ngad_281_OG76
Pp_Diaphoretickes-Bpra_1124_OG76
Other_Diaphoretickes-Omar_13832_OG76
91.1/87
Other_Diaphoretickes-Omar_9399_OG76
36.2/48
Cp_Diaphoretickes-Smin_31724_OG76
90/66
68.7/28
Cp_Diaphoretickes-Smin_33558_OG76
Cp_Diaphoretickes-Aano_8028_OG76
75.8/53
95.5/99
Other_Diaphoretickes-Bmot_1415_OG76
59/70
Pp_Diaphoretickes-Cvar_3180_OG76
85.1/81
69.9/52
Other_Other-Sspe_4356_OG76
Cp_Diaphoretickes-Bnat_8500_OG76
79.9/62
Other_Diaphoretickes-Rlim_2248_OG76
Osmo_Teretosporea-Awhi_20464_OG76
99.2/100
78.2/43
Osmo_Teretosporea-Awhi_5628_OG76
76.2/78
Other_Holomycota-Mdap_2690_OG76
38/41
Osmo_Teretosporea-Sdes_12625_OG76
92.1/96
Osmo_Teretosporea-Cper_6059_OG76
Cp_Diaphoretickes-Ehux_27524_OG76
96.9/100
Cp_Diaphoretickes-Ehux_19552_OG76
89.9/92
Cp_Diaphoretickes-Chsp_3260_OG76
59.6/50
Pp_Diaphoretickes-Cmer_1517_OG76
63.6/61
Other_Holozoa-Sros_8264_OG76
89.3/67
Osmo_Teretosporea-Cper_2210_OG76

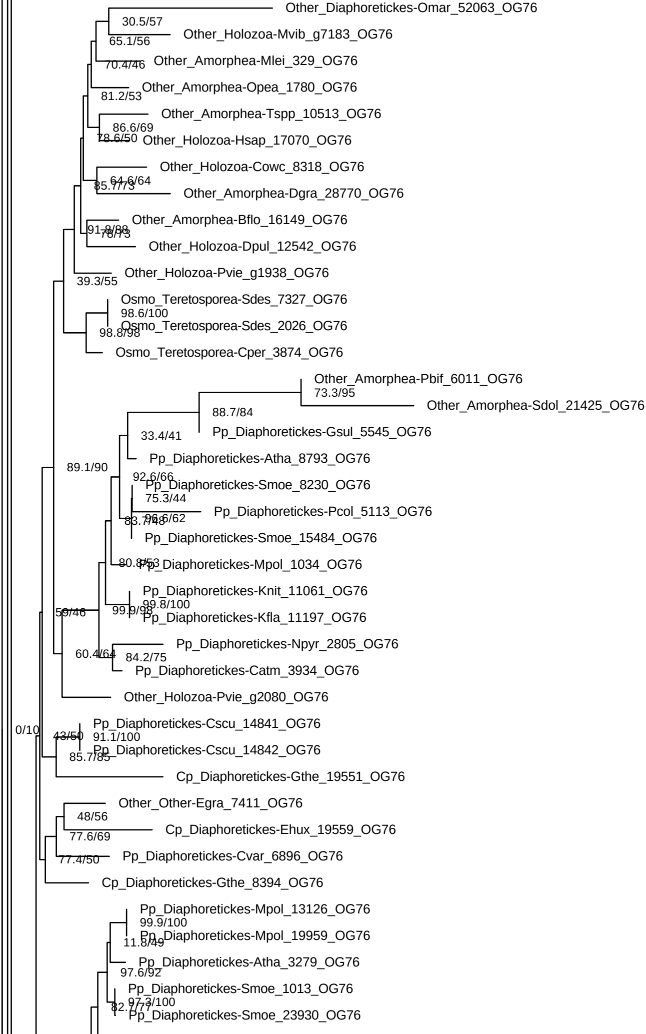
Other_Diaphoretickes-Omar_52063_OG76
30.5/57
Other_Holozoa-Mvib_g7183_OG76
65.1/56
Other_Amorphea-Mlei_329_OG76
70.4/46
Other_Amorphea-Opea_1780_OG76
81.2/53
Other_Amorphea-Tspp_10513_OG76
86.6/69
78.6/50
Other_Holozoa-Hsap_17070_OG76
Other_Holozoa-Cowc_8318_OG76
64.6/64
85.7/73
Other_Amorphea-Dgra_28770_OG76
Other_Amorphea-Bflo_16149_OG76
91.8/88
78/73
Other_Holozoa-Dpul_12542_OG76
Other_Holozoa-Pvie_g1938_OG76
39.3/55
Osmo_Teretosporea-Sdes_7327_OG76
98.6/100
Osmo_Teretosporea-Sdes_2026_OG76
98.8/98
Osmo_Teretosporea-Cper_3874_OG76
Other_Amorphea-Pbif_6011_OG76
73.3/95
Other_Amorphea-Sdol_21425_OG76
88.7/84
Pp_Diaphoretickes-Gsul_5545_OG76
33.4/41
Pp_Diaphoretickes-Atha_8793_OG76
89.1/90
92.6/66
Pp_Diaphoretickes-Smoe_8230_OG76
75.3/44
Pp_Diaphoretickes-Pcol_5113_OG76
96.6/62
83.7/48
Pp_Diaphoretickes-Smoe_15484_OG76
80.8/53
Pp_Diaphoretickes-Mpol_1034_OG76
Pp_Diaphoretickes-Knit_11061_OG76
99.8/100
99.9/98
59/46
Pp_Diaphoretickes-Kfla_11197_OG76
Pp_Diaphoretickes-Npyr_2805_OG76
60.4/64
84.2/75
Pp_Diaphoretickes-Catm_3934_OG76
Other_Holozoa-Pvie_g2080_OG76
Pp_Diaphoretickes-Cscu_14841_OG76
0/10
43/50
91.1/100
Pp_Diaphoretickes-Cscu_14842_OG76
85.7/85
Cp_Diaphoretickes-Gthe_19551_OG76
Other_Other-Egra_7411_OG76
48/56
Cp_Diaphoretickes-Ehux_19559_OG76
77.6/69
Pp_Diaphoretickes-Cvar_6896_OG76
77.4/50
Cp_Diaphoretickes-Gthe_8394_OG76
Pp_Diaphoretickes-Mpol_13126_OG76
99.9/100
Pp_Diaphoretickes-Mpol_19959_OG76
11.8/49
Pp_Diaphoretickes-Atha_3279_OG76
97.6/92
Pp_Diaphoretickes-Smoe_1013_OG76
97.3/100
82.7/77
Pp_Diaphoretickes-Smoe_23930_OG76

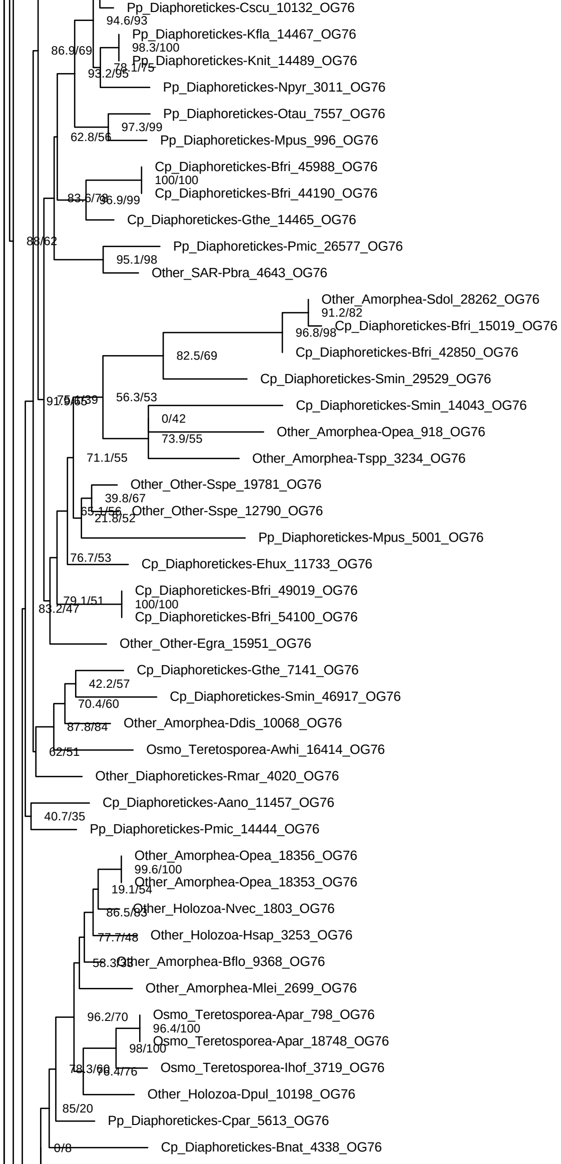
Pp_Diaphoretickes-Cscu_10132_OG76
94.6/93
Pp_Diaphoretickes-Kfla_14467_OG76
86.9/69
98.3/100
Pp_Diaphoretickes-Knit_14489_OG76
78.1/75
93.2/95
Pp_Diaphoretickes-Npyr_3011_OG76
Pp_Diaphoretickes-Otau_7557_OG76
97.3/99
62.8/56
Pp_Diaphoretickes-Mpus_996_OG76
Cp_Diaphoretickes-Bfri_45988_OG76
100/100
Cp_Diaphoretickes-Bfri_44190_OG76
83.6/79
96.9/99
Cp_Diaphoretickes-Gthe_14465_OG76
88/62
Pp_Diaphoretickes-Pmic_26577_OG76
95.1/98
Other_SAR-Pbra_4643_OG76
Other_Amorphea-Sdol_28262_OG76
91.2/82
Cp_Diaphoretickes-Bfri_15019_OG76
96.8/98
82.5/69
Cp_Diaphoretickes-Bfri_42850_OG76
Cp_Diaphoretickes-Smin_29529_OG76
91.9/65
75.1/39
56.3/53
Cp_Diaphoretickes-Smin_14043_OG76
0/42
Other_Amorphea-Opea_918_OG76
73.9/55
71.1/55
Other_Amorphea-Tspp_3234_OG76
Other_Other-Sspe_19781_OG76
39.8/67
65.1/56
21.8/52
Other_Other-Sspe_12790_OG76
Pp_Diaphoretickes-Mpus_5001_OG76
76.7/53
Cp_Diaphoretickes-Ehux_11733_OG76
Cp_Diaphoretickes-Bfri_49019_OG76
79.1/51
83.2/47
100/100
Cp_Diaphoretickes-Bfri_54100_OG76
Other_Other-Egra_15951_OG76
Cp_Diaphoretickes-Gthe_7141_OG76
42.2/57
70.4/60
Cp_Diaphoretickes-Smin_46917_OG76
87.8/84
Other_Amorphea-Ddis_10068_OG76
62/51
Osmo_Teretosporea-Awhi_16414_OG76
Other_Diaphoretickes-Rmar_4020_OG76
Cp_Diaphoretickes-Aano_11457_OG76
40.7/35
Pp_Diaphoretickes-Pmic_14444_OG76
Other_Amorphea-Opea_18356_OG76
99.6/100
19.1/54
Other_Amorphea-Opea_18353_OG76
86.5/83
Other_Holozoa-Nvec_1803_OG76
77.7/48
Other_Holozoa-Hsap_3253_OG76
58.3/35
Other_Amorphea-Bflo_9368_OG76
Other_Amorphea-Mlei_2699_OG76
96.2/70
Osmo_Teretosporea-Apar_798_OG76
96.4/100
98/100
Osmo_Teretosporea-Apar_18748_OG76
78.3/60
76.4/76
Osmo_Teretosporea-Ihof_3719_OG76
Other_Holozoa-Dpul_10198_OG76
85/20
Pp_Diaphoretickes-Cpar_5613_OG76
0/8
Cp_Diaphoretickes-Bnat_4338_OG76

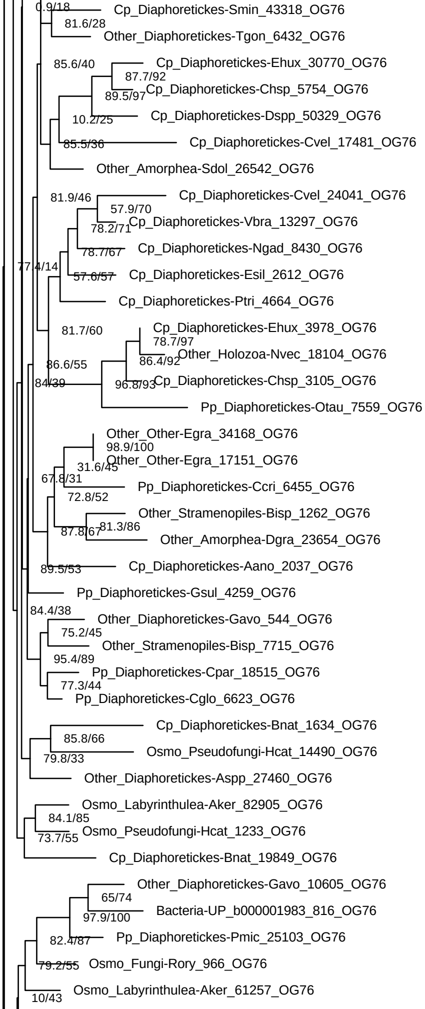
0.9/18
Cp_Diaphoretickes-Smin_43318_OG76
81.6/28
Other_Diaphoretickes-Tgon_6432_OG76
85.6/40
Cp_Diaphoretickes-Ehux_30770_OG76
87.7/92
Cp_Diaphoretickes-Chsp_5754_OG76
89.5/97
Cp_Diaphoretickes-Dspp_50329_OG76
10.2/25
Cp_Diaphoretickes-Cvel_17481_OG76
85.5/36
Other_Amorphea-Sdol_26542_OG76
81.9/46
Cp_Diaphoretickes-Cvel_24041_OG76
57.9/70
Cp_Diaphoretickes-Vbra_13297_OG76
78.2/71
Cp_Diaphoretickes-Ngad_8430_OG76
78.7/67
77.4/14
Cp_Diaphoretickes-Esil_2612_OG76
57.6/57
Cp_Diaphoretickes-Ptri_4664_OG76
81.7/60
Cp_Diaphoretickes-Ehux_3978_OG76
78.7/97
Other_Holozoa-Nvec_18104_OG76
86.6/55
86.4/92
Cp_Diaphoretickes-Chsp_3105_OG76
84/39
96.8/93
Pp_Diaphoretickes-Otau_7559_OG76
Other_Other-Egra_34168_OG76
98.9/100
Other_Other-Egra_17151_OG76
31.6/45
67.8/31
Pp_Diaphoretickes-Ccri_6455_OG76
72.8/52
Other_Stramenopiles-Bisp_1262_OG76
87.8/67
81.3/86
Other_Amorphea-Dgra_23654_OG76
Cp_Diaphoretickes-Aano_2037_OG76
89.5/53
Pp_Diaphoretickes-Gsul_4259_OG76
84.4/38
Other_Diaphoretickes-Gavo_544_OG76
75.2/45
Other_Stramenopiles-Bisp_7715_OG76
95.4/89
Pp_Diaphoretickes-Cpar_18515_OG76
77.3/44
Pp_Diaphoretickes-Cglo_6623_OG76
Cp_Diaphoretickes-Bnat_1634_OG76
85.8/66
Osmo_Pseudofungi-Hcat_14490_OG76
79.8/33
Other_Diaphoretickes-Aspp_27460_OG76
Osmo_Labyrinthulea-Aker_82905_OG76
84.1/85
Osmo_Pseudofungi-Hcat_1233_OG76
73.7/55
Cp_Diaphoretickes-Bnat_19849_OG76
Other_Diaphoretickes-Gavo_10605_OG76
65/74
Bacteria-UP_b000001983_816_OG76
97.9/100
Pp_Diaphoretickes-Pmic_25103_OG76
82.4/87
Osmo_Fungi-Rory_966_OG76
79.2/55
Osmo_Labyrinthulea-Aker_61257_OG76
10/43

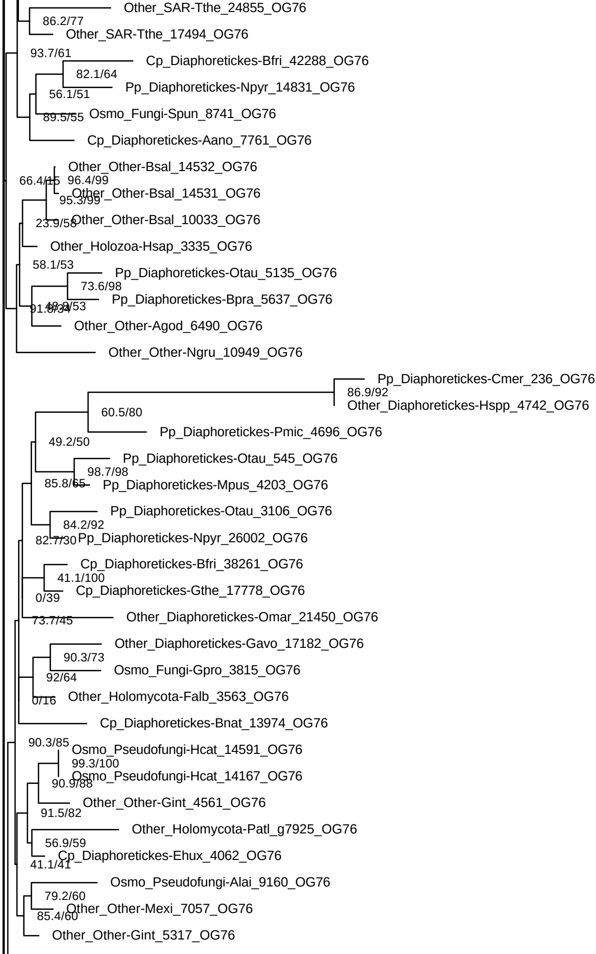
Other_SAR-Tthe_24855_OG76
86.2/77
Other_SAR-Tthe_17494_OG76
93.7/61
Cp_Diaphoretickes-Bfri_42288_OG76
82.1/64
Pp_Diaphoretickes-Npyr_14831_OG76
56.1/51
Osmo_Fungi-Spun_8741_OG76
89.5/55
Cp_Diaphoretickes-Aano_7761_OG76
Other_Other-Bsal_14532_OG76
66.4/15
96.4/99
Other_Other-Bsal_14531_OG76
95.3/99
Other_Other-Bsal_10033_OG76
23.9/58
Other_Holozoa-Hsap_3335_OG76
58.1/53
Pp_Diaphoretickes-Otau_5135_OG76
73.6/98
Pp_Diaphoretickes-Bpra_5637_OG76
91.8/34
48.9/53
Other_Other-Agod_6490_OG76
Other_Other-Ngru_10949_OG76
Pp_Diaphoretickes-Cmer_236_OG76
86.9/92
Other_Diaphoretickes-Hspp_4742_OG76
60.5/80
Pp_Diaphoretickes-Pmic_4696_OG76
49.2/50
Pp_Diaphoretickes-Otau_545_OG76
98.7/98
85.8/65
Pp_Diaphoretickes-Mpus_4203_OG76
Pp_Diaphoretickes-Otau_3106_OG76
84.2/92
82.7/30
Pp_Diaphoretickes-Npyr_26002_OG76
Cp_Diaphoretickes-Bfri_38261_OG76
41.1/100
Cp_Diaphoretickes-Gthe_17778_OG76
0/39
Other_Diaphoretickes-Omar_21450_OG76
73.7/45
Other_Diaphoretickes-Gavo_17182_OG76
90.3/73
Osmo_Fungi-Gpro_3815_OG76
92/64
Other_Holomycota-Falb_3563_OG76
0/16
Cp_Diaphoretickes-Bnat_13974_OG76
90.3/85
Osmo_Pseudofungi-Hcat_14591_OG76
99.3/100
Osmo_Pseudofungi-Hcat_14167_OG76
90.9/88
Other_Other-Gint_4561_OG76
91.5/82
Other_Holomycota-Patl_g7925_OG76
56.9/59
Cp_Diaphoretickes-Ehux_4062_OG76
41.1/41
Osmo_Pseudofungi-Alai_9160_OG76
79.2/60
Other_Other-Mexi_7057_OG76
85.4/60
Other_Other-Gint_5317_OG76

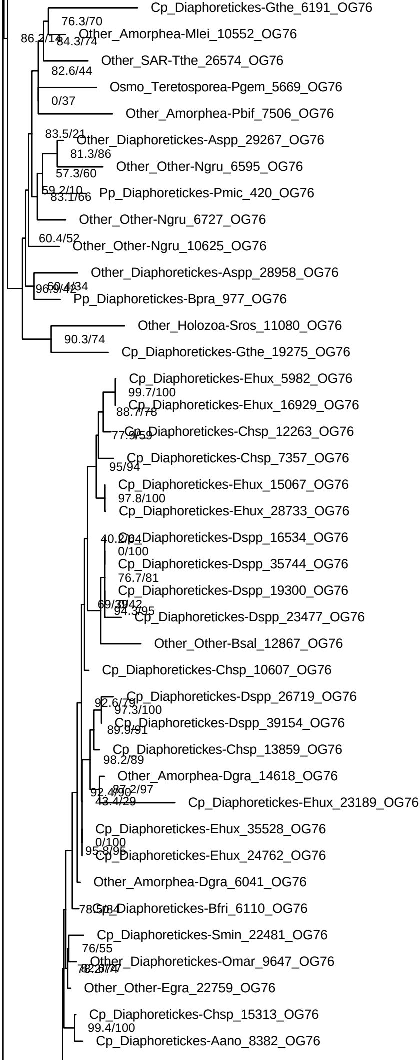

Cp_Diaphoretickes-Gthe_6191_OG76
76.3/70
86.2/14
Other_Amorphea-Mlei_10552_OG76
84.3/74
Other_SAR-Tthe_26574_OG76
82.6/44
Osmo_Teretosporea-Pgem_5669_OG76
0/37
Other_Amorphea-Pbif_7506_OG76
83.5/21
Other_Diaphoretickes-Aspp_29267_OG76
81.3/86
Other_Other-Ngru_6595_OG76
57.3/60
Pp_Diaphoretickes-Pmic_420_OG76
83.1/66
Other_Other-Ngru_6727_OG76
60.4/52
Other_Other-Ngru_10625_OG76
Other_Diaphoretickes-Aspp_28958_OG76
Pp_Diaphoretickes-Bpra_977_OG76
Other_Holozoa-Sros_11080_OG76
90.3/74
Cp_Diaphoretickes-Gthe_19275_OG76
Cp_Diaphoretickes-Ehux_5982_OG76
99.7/100
Cp_Diaphoretickes-Ehux_16929_OG76
88.7/78
Cp_Diaphoretickes-Chsp_12263_OG76
77.9/59
Cp_Diaphoretickes-Chsp_7357_OG76
95/94
Cp_Diaphoretickes-Ehux_15067_OG76
97.8/100
Cp_Diaphoretickes-Ehux_28733_OG76
Cp_Diaphoretickes-Dspp_16534_OG76
0/100
Cp_Diaphoretickes-Dspp_35744_OG76
76.7/81
Cp_Diaphoretickes-Dspp_19300_OG76
94.3/95
Cp_Diaphoretickes-Dspp_23477_OG76
Other_Other-Bsal_12867_OG76
Cp_Diaphoretickes-Chsp_10607_OG76
Cp_Diaphoretickes-Dspp_26719_OG76
92.6/79
97.3/100
Cp_Diaphoretickes-Dspp_39154_OG76
89.9/91
Cp_Diaphoretickes-Chsp_13859_OG76
98.2/89
Other_Amorphea-Dgra_14618_OG76
87.2/97
43.4/29
Cp_Diaphoretickes-Ehux_23189_OG76
Cp_Diaphoretickes-Ehux_35528_OG76
0/100
95.8/95
Cp_Diaphoretickes-Ehux_24762_OG76
Other_Amorphea-Dgra_6041_OG76
Cp_Diaphoretickes-Bfri_6110_OG76
Cp_Diaphoretickes-Smin_22481_OG76
76/55
Other_Diaphoretickes-Omar_9647_OG76
Other_Other-Egra_22759_OG76
Cp_Diaphoretickes-Chsp_15313_OG76
99.4/100
Cp_Diaphoretickes-Aano_8382_OG76

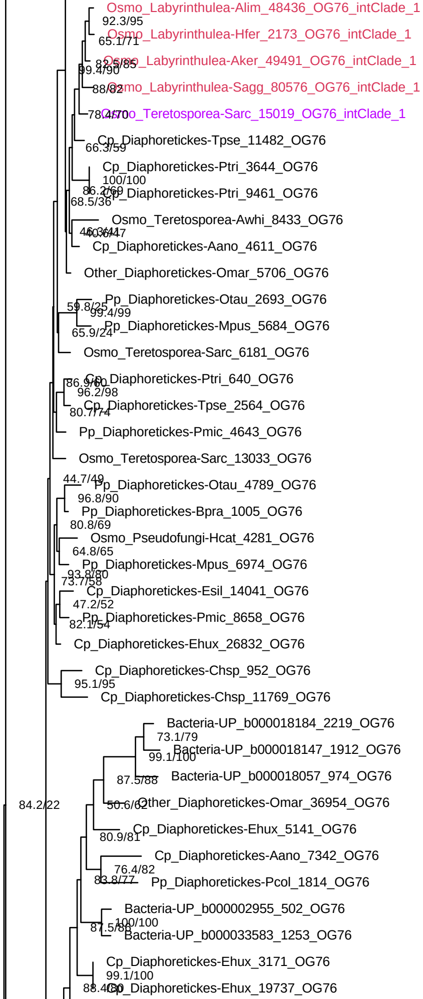

Osmo_Labyrinthulea-Alim_48436_OG76_intClade_1
92.3/95
Osmo_Labyrinthulea-Hfer_2173_OG76_intClade_1
65.1/71
Osmo_Labyrinthulea-Aker_49491_OG76_intClade_1
82.5/85
99.4/90
Osmo_Labyrinthulea-Sagg_80576_OG76_intClade_1
88/82
Osmo_Teretosporea-Sarc_15019_OG76_intClade_1
78.4/70
Cp_Diaphoretickes-Tpse_11482_OG76
66.3/59
Cp_Diaphoretickes-Ptri_3644_OG76
100/100
86.2/69
Cp_Diaphoretickes-Ptri_9461_OG76
68.5/36
Osmo_Teretosporea-Awhi_8433_OG76
46.3/41
40.6/47
Cp_Diaphoretickes-Aano_4611_OG76
Other_Diaphoretickes-Omar_5706_OG76
Pp_Diaphoretickes-Otau_2693_OG76
59.8/25
99.4/99
Pp_Diaphoretickes-Mpus_5684_OG76
65.9/24
Osmo_Teretosporea-Sarc_6181_OG76
Cp_Diaphoretickes-Ptri_640_OG76
86.9/60
96.2/98
Cp_Diaphoretickes-Tpse_2564_OG76
80.7/74
Pp_Diaphoretickes-Pmic_4643_OG76
Osmo_Teretosporea-Sarc_13033_OG76
44.7/49
Pp_Diaphoretickes-Otau_4789_OG76
96.8/90
Pp_Diaphoretickes-Bpra_1005_OG76
80.8/69
Osmo_Pseudofungi-Hcat_4281_OG76
64.8/65
Pp_Diaphoretickes-Mpus_6974_OG76
93.8/80
73.7/58
Cp_Diaphoretickes-Esil_14041_OG76
47.2/52
Pp_Diaphoretickes-Pmic_8658_OG76
82.1/54
Cp_Diaphoretickes-Ehux_26832_OG76
Cp_Diaphoretickes-Chsp_952_OG76
95.1/95
Cp_Diaphoretickes-Chsp_11769_OG76
Bacteria-UP_b000018184_2219_OG76
73.1/79
Bacteria-UP_b000018147_1912_OG76
99.1/100
Bacteria-UP_b000018057_974_OG76
87.5/88
84.2/22
Other_Diaphoretickes-Omar_36954_OG76
50.6/62
Cp_Diaphoretickes-Ehux_5141_OG76
80.9/81
Cp_Diaphoretickes-Aano_7342_OG76
76.4/82
83.8/77
Pp_Diaphoretickes-Pcol_1814_OG76
Bacteria-UP_b000002955_502_OG76
100/100
87.5/86
Bacteria-UP_b000033583_1253_OG76
Cp_Diaphoretickes-Ehux_3171_OG76
99.1/100
88.4/80
Cp_Diaphoretickes-Ehux_19737_OG76

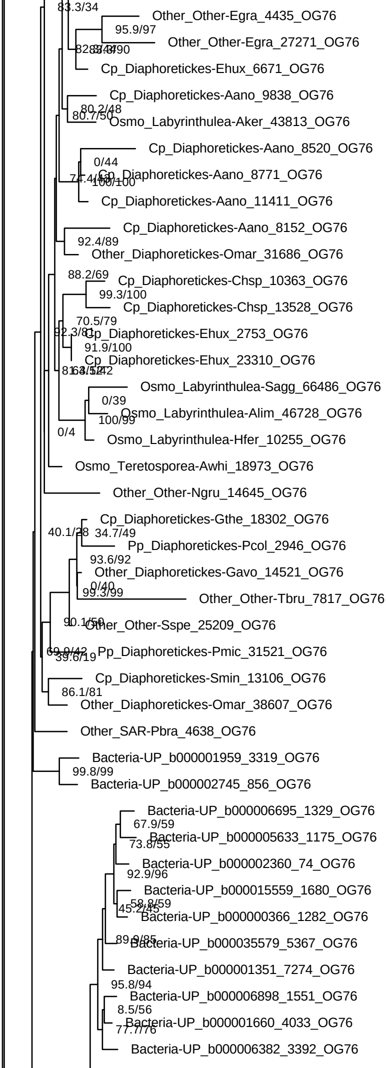
83.3/34
Other_Other-Egra_4435_OG76
95.9/97
Other_Other-Egra_27271_OG76
Cp_Diaphoretickes-Ehux_6671_OG76
Cp_Diaphoretickes-Aano_9838_OG76
80.2/48
80.7/50
Osmo_Labyrinthulea-Aker_43813_OG76
Cp_Diaphoretickes-Aano_8520_OG76
0/44
Cp_Diaphoretickes-Aano_8771_OG76
100/100
Cp_Diaphoretickes-Aano_11411_OG76
Cp_Diaphoretickes-Aano_8152_OG76
92.4/89
Other_Diaphoretickes-Omar_31686_OG76
88.2/69
Cp_Diaphoretickes-Chsp_10363_OG76
99.3/100
Cp_Diaphoretickes-Chsp_13528_OG76
70.5/79
Cp_Diaphoretickes-Ehux_2753_OG76
91.9/100
Cp_Diaphoretickes-Ehux_23310_OG76
Osmo_Labyrinthulea-Sagg_66486_OG76
0/39
Osmo_Labyrinthulea-Alim_46728_OG76
100/99
0/4
Osmo_Labyrinthulea-Hfer_10255_OG76
Osmo_Teretosporea-Awhi_18973_OG76
Other_Other-Ngru_14645_OG76
Cp_Diaphoretickes-Gthe_18302_OG76
40.1/28
34.7/49
Pp_Diaphoretickes-Pcol_2946_OG76
93.6/92
Other_Diaphoretickes-Gavo_14521_OG76
0/40
99.3/99
Other_Other-Tbru_7817_OG76
Other_Other-Sspe_25209_OG76
39.6/19
Pp_Diaphoretickes-Pmic_31521_OG76
Cp_Diaphoretickes-Smin_13106_OG76
86.1/81
Other_Diaphoretickes-Omar_38607_OG76
Other_SAR-Pbra_4638_OG76
Bacteria-UP_b000001959_3319_OG76
99.8/99
Bacteria-UP_b000002745_856_OG76
Bacteria-UP_b000006695_1329_OG76
67.9/59
Bacteria-UP_b000005633_1175_OG76
73.8/55
Bacteria-UP_b000002360_74_OG76
92.9/96
Bacteria-UP_b000015559_1680_OG76
58.8/59
45.2/45
Bacteria-UP_b000000366_1282_OG76
89.9/85
Bacteria-UP_b000035579_5367_OG76
Bacteria-UP_b000001351_7274_OG76
95.8/94
Bacteria-UP_b000006898_1551_OG76
8.5/56
Bacteria-UP_b000001660_4033_OG76
77.7/76
Bacteria-UP_b000006382_3392_OG76

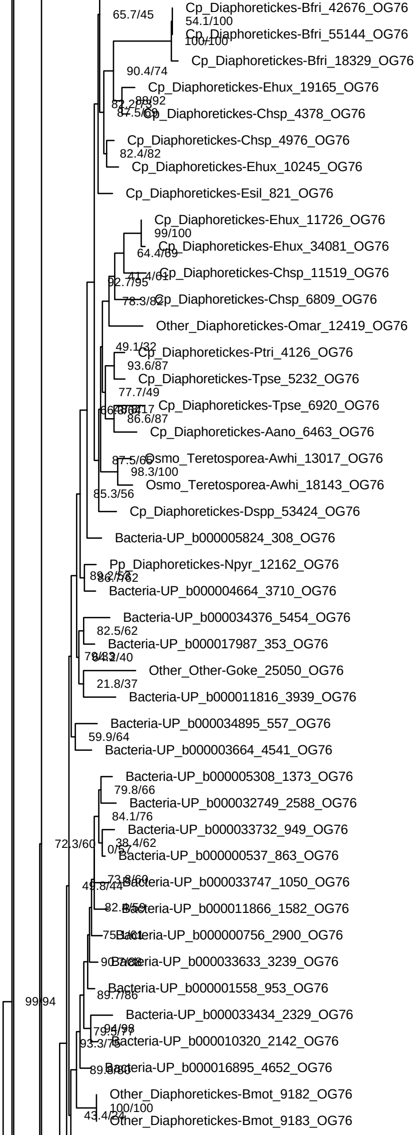
Cp_Diaphoretickes-Bfri_42676_OG76
Cp_Diaphoretickes-Bfri_55144_OG76
Cp_Diaphoretickes-Bfri_18329_OG76
Cp_Diaphoretickes-Ehux_19165_OG76
Cp_Diaphoretickes-Chsp_4378_OG76
Cp_Diaphoretickes-Chsp_4976_OG76
Cp_Diaphoretickes-Ehux_10245_OG76
Cp_Diaphoretickes-Esil_821_OG76
Cp_Diaphoretickes-Ehux_11726_OG76
Cp_Diaphoretickes-Ehux_34081_OG76
Cp_Diaphoretickes-Chsp_11519_OG76
Cp_Diaphoretickes-Chsp_6809_OG76
Other_Diaphoretickes-Omar_12419_OG76
Cp_Diaphoretickes-Ptri_4126_OG76
Cp_Diaphoretickes-Tpse_5232_OG76
Cp_Diaphoretickes-Tpse_6920_OG76
Cp_Diaphoretickes-Aano_6463_OG76
Osmo_Teretosporea-Awhi_13017_OG76
Osmo_Teretosporea-Awhi_18143_OG76
Cp_Diaphoretickes-Dspp_53424_OG76
Bacteria-UP_b000005824_308_OG76
Pp_Diaphoretickes-Npyr_12162_OG76
Bacteria-UP_b000004664_3710_OG76
Bacteria-UP_b000034376_5454_OG76
Bacteria-UP_b000017987_353_OG76
Other_Other-Goke_25050_OG76
Bacteria-UP_b000011816_3939_OG76
Bacteria-UP_b000034895_557_OG76
Bacteria-UP_b000003664_4541_OG76
Bacteria-UP_b000005308_1373_OG76
Bacteria-UP_b000032749_2588_OG76
Bacteria-UP_b000033732_949_OG76
Bacteria-UP_b000000537_863_OG76
Bacteria-UP_b000033747_1050_OG76
Bacteria-UP_b000011866_1582_OG76
Bacteria-UP_b000000756_2900_OG76
Bacteria-UP_b000033633_3239_OG76
Bacteria-UP_b000001558_953_OG76
Bacteria-UP_b000033434_2329_OG76
Bacteria-UP_b000010320_2142_OG76
Bacteria-UP_b000016895_4652_OG76
Other_Diaphoretickes-Bmot_9182_OG76
Other_Diaphoretickes-Bmot_9183_OG76
65.7/45
54.1/100
100/100
90.4/74
82.4/82
99/100
64.4/69
92.7/95
78.3/82
49.1/32
93.6/87
77.7/49
86.6/87
98.3/100
85.3/56
82.5/62
21.8/37
59.9/64
79.8/66
84.1/76
72.3/60
38.4/62
49.8/44
89.7/86
99/94
100/100
43.4/24

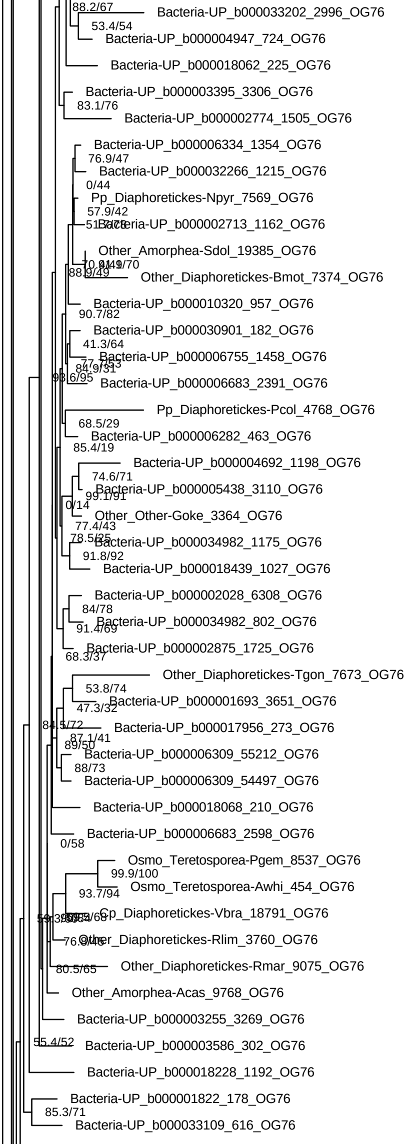

88.2/67
Bacteria-UP_b000033202_2996_OG76
53.4/54
Bacteria-UP_b000004947_724_OG76
Bacteria-UP_b000018062_225_OG76
Bacteria-UP_b000003395_3306_OG76
83.1/76
Bacteria-UP_b000002774_1505_OG76
Bacteria-UP_b000006334_1354_OG76
76.9/47
Bacteria-UP_b000032266_1215_OG76
0/44
Pp_Diaphoretickes-Npyr_7569_OG76
57.9/42
Bacteria-UP_b000002713_1162_OG76
Other_Amorphea-Sdol_19385_OG76
41.9/70
88.9/49
Other_Diaphoretickes-Bmot_7374_OG76
Bacteria-UP_b000010320_957_OG76
90.7/82
Bacteria-UP_b000030901_182_OG76
41.3/64
Bacteria-UP_b000006755_1458_OG76
84.9/31
93.6/95
Bacteria-UP_b000006683_2391_OG76
Pp_Diaphoretickes-Pcol_4768_OG76
68.5/29
Bacteria-UP_b000006282_463_OG76
85.4/19
Bacteria-UP_b000004692_1198_OG76
74.6/71
Bacteria-UP_b000005438_3110_OG76
99.1/91
0/14
Other_Other-Goke_3364_OG76
77.4/43
78.5/25
Bacteria-UP_b000034982_1175_OG76
91.8/92
Bacteria-UP_b000018439_1027_OG76
Bacteria-UP_b000002028_6308_OG76
84/78
Bacteria-UP_b000034982_802_OG76
91.4/69
Bacteria-UP_b000002875_1725_OG76
68.3/37
Other_Diaphoretickes-Tgon_7673_OG76
53.8/74
Bacteria-UP_b000001693_3651_OG76
47.3/32
84.5/72
Bacteria-UP_b000017956_273_OG76
87.1/41
89/50
Bacteria-UP_b000006309_55212_OG76
88/73
Bacteria-UP_b000006309_54497_OG76
Bacteria-UP_b000018068_210_OG76
Bacteria-UP_b000006683_2598_OG76
0/58
Osmo_Teretosporea-Pgem_8537_OG76
99.9/100
Osmo_Teretosporea-Awhi_454_OG76
93.7/94
Cp_Diaphoretickes-Vbra_18791_OG76
Other_Diaphoretickes-Rlim_3760_OG76
Other_Diaphoretickes-Rmar_9075_OG76
80.5/65
Other_Amorphea-Acas_9768_OG76
Bacteria-UP_b000003255_3269_OG76
55.4/52
Bacteria-UP_b000003586_302_OG76
Bacteria-UP_b000018228_1192_OG76
Bacteria-UP_b000001822_178_OG76
85.3/71
Bacteria-UP_b000033109_616_OG76

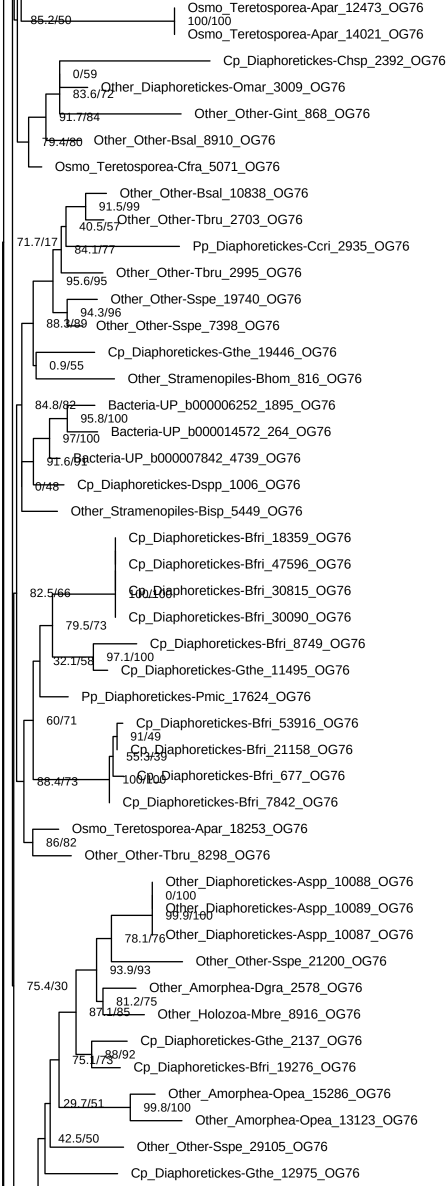

Osmo_Teretosporea-Apar_12473_OG76
85.2/50
100/100
Osmo_Teretosporea-Apar_14021_OG76
Cp_Diaphoretickes-Chsp_2392_OG76
0/59
Other_Diaphoretickes-Omar_3009_OG76
83.6/72
Other_Other-Gint_868_OG76
91.7/84
Other_Other-Bsal_8910_OG76
79.4/80
Osmo_Teretosporea-Cfra_5071_OG76
Other_Other-Bsal_10838_OG76
91.5/99
Other_Other-Tbru_2703_OG76
40.5/57
71.7/17
Pp_Diaphoretickes-Ccri_2935_OG76
84.1/77
Other_Other-Tbru_2995_OG76
95.6/95
Other_Other-Sspe_19740_OG76
94.3/96
88.3/89
Other_Other-Sspe_7398_OG76
Cp_Diaphoretickes-Gthe_19446_OG76
0.9/55
Other_Stramenopiles-Bhom_816_OG76
84.8/82
Bacteria-UP_b000006252_1895_OG76
95.8/100
Bacteria-UP_b000014572_264_OG76
97/100
Bacteria-UP_b000007842_4739_OG76
91.6/91
0/48
Cp_Diaphoretickes-Dspp_1006_OG76
Other_Stramenopiles-Bisp_5449_OG76
Cp_Diaphoretickes-Bfri_18359_OG76
Cp_Diaphoretickes-Bfri_47596_OG76
82.5/66
Cp_Diaphoretickes-Bfri_30815_OG76
100/100
Cp_Diaphoretickes-Bfri_30090_OG76
79.5/73
Cp_Diaphoretickes-Bfri_8749_OG76
97.1/100
32.1/58
Cp_Diaphoretickes-Gthe_11495_OG76
Pp_Diaphoretickes-Pmic_17624_OG76
60/71
Cp_Diaphoretickes-Bfri_53916_OG76
91/49
Cp_Diaphoretickes-Bfri_21158_OG76
55.3/39
88.4/73
100/100
Cp_Diaphoretickes-Bfri_677_OG76
Cp_Diaphoretickes-Bfri_7842_OG76
Osmo_Teretosporea-Apar_18253_OG76
86/82
Other_Other-Tbru_8298_OG76
Other_Diaphoretickes-Aspp_10088_OG76
0/100
Other_Diaphoretickes-Aspp_10089_OG76
99.9/100
78.1/76
Other_Diaphoretickes-Aspp_10087_OG76
Other_Other-Sspe_21200_OG76
93.9/93
75.4/30
Other_Amorphea-Dgra_2578_OG76
81.2/75
87.1/85
Other_Holozoa-Mbre_8916_OG76
Cp_Diaphoretickes-Gthe_2137_OG76
88/92
75.1/73
Cp_Diaphoretickes-Bfri_19276_OG76
Other_Amorphea-Opea_15286_OG76
29.7/51
99.8/100
Other_Amorphea-Opea_13123_OG76
42.5/50
Other_Other-Sspe_29105_OG76
Cp_Diaphoretickes-Gthe_12975_OG76

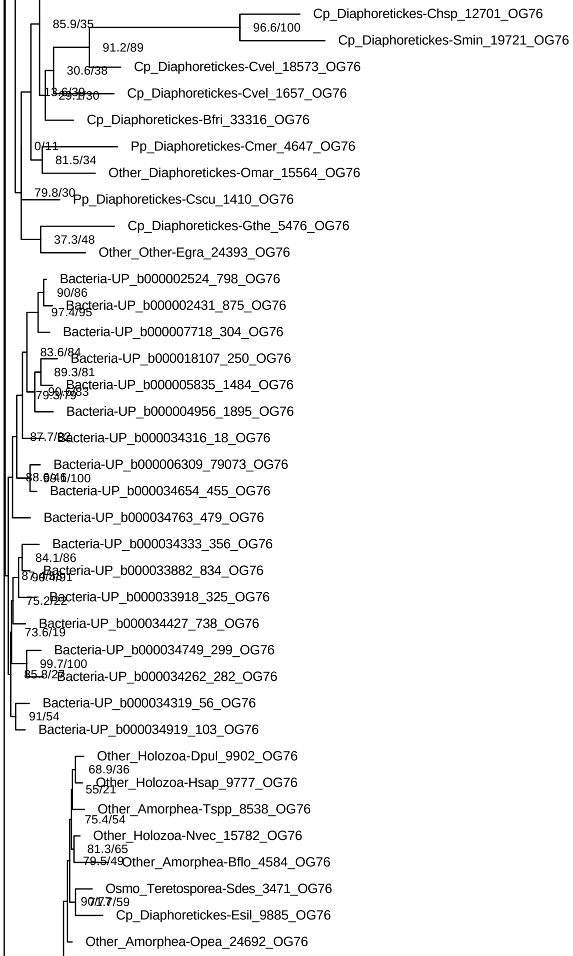

Cp_Diaphoretickes-Chsp_12701_OG76
96.6/100
Cp_Diaphoretickes-Smin_19721_OG76
85.9/35
91.2/89
30.6/38
Cp_Diaphoretickes-Cvel_18573_OG76
13.6/30
29.1/30
Cp_Diaphoretickes-Cvel_1657_OG76
Cp_Diaphoretickes-Bfri_33316_OG76
0/11
Pp_Diaphoretickes-Cmer_4647_OG76
81.5/34
Other_Diaphoretickes-Omar_15564_OG76
79.8/30
Pp_Diaphoretickes-Cscu_1410_OG76
Cp_Diaphoretickes-Gthe_5476_OG76
37.3/48
Other_Other-Egra_24393_OG76
Bacteria-UP_b000002524_798_OG76
90/86
Bacteria-UP_b000002431_875_OG76
97.4/95
Bacteria-UP_b000007718_304_OG76
83.6/84
Bacteria-UP_b000018107_250_OG76
89.3/81
Bacteria-UP_b000005835_1484_OG76
90.5/83
79.3/79
Bacteria-UP_b000004956_1895_OG76
87.7/82
Bacteria-UP_b000034316_18_OG76
Bacteria-UP_b000006309_79073_OG76
88.9/46
99.1/100
Bacteria-UP_b000034654_455_OG76
Bacteria-UP_b000034763_479_OG76
Bacteria-UP_b000034333_356_OG76
84.1/86
Bacteria-UP_b000033882_834_OG76
96.4/91
75.2/22
Bacteria-UP_b000033918_325_OG76
Bacteria-UP_b000034427_738_OG76
73.6/19
Bacteria-UP_b000034749_299_OG76
99.7/100
85.8/27
Bacteria-UP_b000034262_282_OG76
Bacteria-UP_b000034319_56_OG76
91/54
Bacteria-UP_b000034919_103_OG76
Other_Holozoa-Dpul_9902_OG76
68.9/36
Other_Holozoa-Hsap_9777_OG76
55/21
Other_Amorphea-Tspp_8538_OG76
75.4/54
Other_Holozoa-Nvec_15782_OG76
81.3/65
79.5/49
Other_Amorphea-Bflo_4584_OG76
Osmo_Teretosporea-Sdes_3471_OG76
77.7/59
Cp_Diaphoretickes-Esil_9885_OG76
Other_Amorphea-Opea_24692_OG76

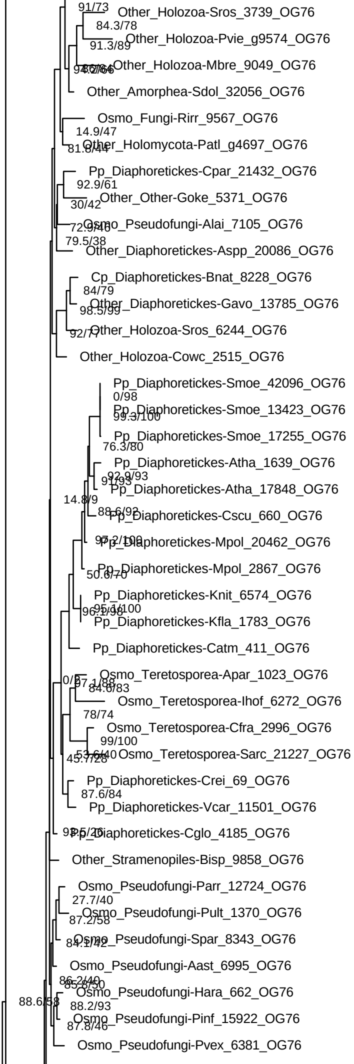

91/73
Other_Holozoa-Sros_3739_OG76
84.3/78
Other_Holozoa-Pvie_g9574_OG76
91.3/89
Other_Holozoa-Mbre_9049_OG76
Other_Amorphea-Sdol_32056_OG76
Osmo_Fungi-Rirr_9567_OG76
14.9/47
Other_Holomycota-Patl_g4697_OG76
81.8/44
Pp_Diaphoretickes-Cpar_21432_OG76
92.9/61
Other_Other-Goke_5371_OG76
30/42
Osmo_Pseudofungi-Alai_7105_OG76
72.9/40
79.5/38
Other_Diaphoretickes-Aspp_20086_OG76
Cp_Diaphoretickes-Bnat_8228_OG76
84/79
Other_Diaphoretickes-Gavo_13785_OG76
98.5/99
Other_Holozoa-Sros_6244_OG76
92/77
Other_Holozoa-Cowc_2515_OG76
Pp_Diaphoretickes-Smoe_42096_OG76
0/98
Pp_Diaphoretickes-Smoe_13423_OG76
99.3/100
Pp_Diaphoretickes-Smoe_17255_OG76
76.3/80
Pp_Diaphoretickes-Atha_1639_OG76
92.9/93
91/93
Pp_Diaphoretickes-Atha_17848_OG76
14.8/9
88.6/92
Pp_Diaphoretickes-Cscu_660_OG76
97.2/100
Pp_Diaphoretickes-Mpol_20462_OG76
Pp_Diaphoretickes-Mpol_2867_OG76
50.6/70
Pp_Diaphoretickes-Knit_6574_OG76
95.1/100
96.1/98
Pp_Diaphoretickes-Kfla_1783_OG76
Pp_Diaphoretickes-Catm_411_OG76
Osmo_Teretosporea-Apar_1023_OG76
0/3
97.1/88
84.6/83
Osmo_Teretosporea-Ihof_6272_OG76
78/74
Osmo_Teretosporea-Cfra_2996_OG76
99/100
53.6/40
45.7/28
Osmo_Teretosporea-Sarc_21227_OG76
Pp_Diaphoretickes-Crei_69_OG76
87.6/84
Pp_Diaphoretickes-Vcar_11501_OG76
Pp_Diaphoretickes-Cglo_4185_OG76
Other_Stramenopiles-Bisp_9858_OG76
Osmo_Pseudofungi-Parr_12724_OG76
27.7/40
Osmo_Pseudofungi-Pult_1370_OG76
87.2/58
Osmo_Pseudofungi-Spar_8343_OG76
84.1/42
Osmo_Pseudofungi-Aast_6995_OG76
86.2/40
85.8/50
Osmo_Pseudofungi-Hara_662_OG76
88.6/58
88.2/93
Osmo_Pseudofungi-Pinf_15922_OG76
87.8/46
Osmo_Pseudofungi-Pvex_6381_OG76

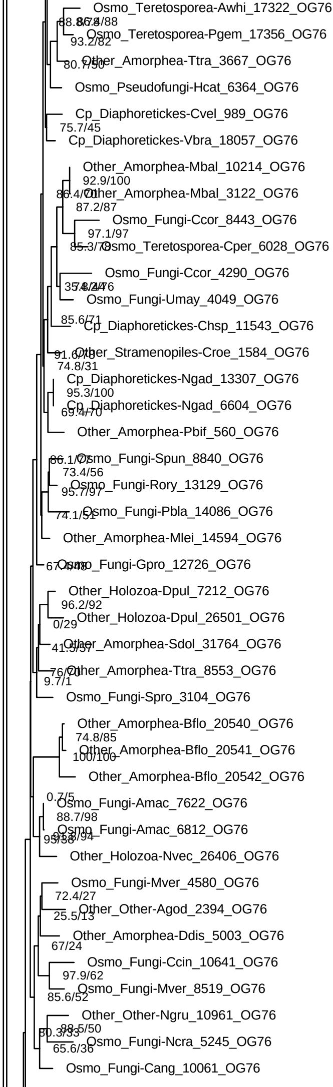
Osmo_Teretosporea-Awhi_17322_OG76
Osmo_Teretosporea-Pgem_17356_OG76
93.2/82
Other_Amorphea-Ttra_3667_OG76
Osmo_Pseudofungi-Hcat_6364_OG76
Cp_Diaphoretickes-Cvel_989_OG76
75.7/45
Cp_Diaphoretickes-Vbra_18057_OG76
Other_Amorphea-Mbal_10214_OG76
92.9/100
Other_Amorphea-Mbal_3122_OG76
87.2/87
Osmo_Fungi-Ccor_8443_OG76
97.1/97
Osmo_Teretosporea-Cper_6028_OG76
Osmo_Fungi-Ccor_4290_OG76
Osmo_Fungi-Umay_4049_OG76
85.6/71
Cp_Diaphoretickes-Chsp_11543_OG76
Other_Stramenopiles-Croe_1584_OG76
74.8/31
Cp_Diaphoretickes-Ngad_13307_OG76
95.3/100
Cp_Diaphoretickes-Ngad_6604_OG76
69.4/70
Other_Amorphea-Pbif_560_OG76
Osmo_Fungi-Spun_8840_OG76
73.4/56
Osmo_Fungi-Rory_13129_OG76
95.7/97
Osmo_Fungi-Pbla_14086_OG76
74.1/51
Other_Amorphea-Mlei_14594_OG76
Osmo_Fungi-Gpro_12726_OG76
Other_Holozoa-Dpul_7212_OG76
96.2/92
0/29
Other_Holozoa-Dpul_26501_OG76
Other_Amorphea-Sdol_31764_OG76
Other_Amorphea-Ttra_8553_OG76
9.7/1
Osmo_Fungi-Spro_3104_OG76
Other_Amorphea-Bflo_20540_OG76
74.8/85
Other_Amorphea-Bflo_20541_OG76
100/100
Other_Amorphea-Bflo_20542_OG76
0.7/5
Osmo_Fungi-Amac_7622_OG76
88.7/98
Osmo_Fungi-Amac_6812_OG76
Other_Holozoa-Nvec_26406_OG76
Osmo_Fungi-Mver_4580_OG76
72.4/27
Other_Other-Agod_2394_OG76
25.5/13
Other_Amorphea-Ddis_5003_OG76
67/24
Osmo_Fungi-Ccin_10641_OG76
97.9/62
Osmo_Fungi-Mver_8519_OG76
85.6/52
Other_Other-Ngru_10961_OG76
88.5/50
Osmo_Fungi-Ncra_5245_OG76
65.6/36
Osmo_Fungi-Cang_10061_OG76

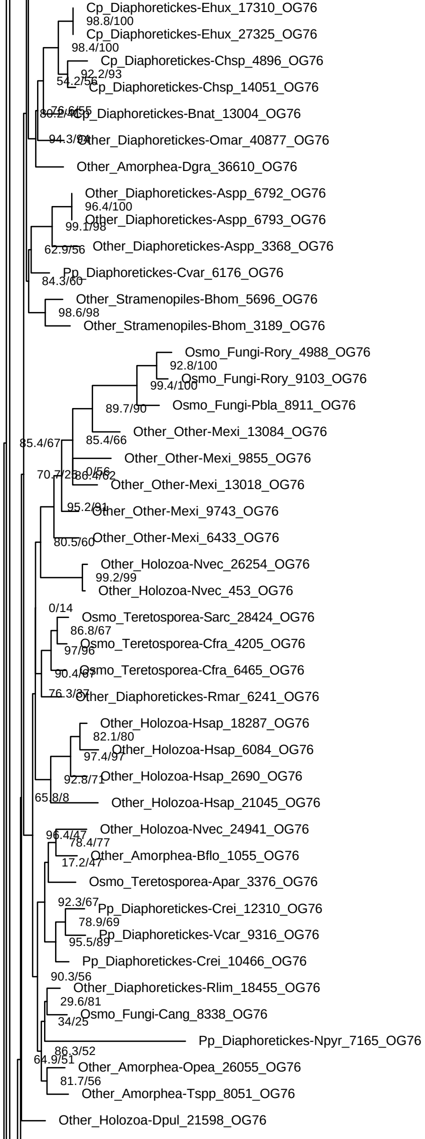

Cp_Diaphoretickes-Ehux_17310_OG76
98.8/100
Cp_Diaphoretickes-Ehux_27325_OG76
98.4/100
Cp_Diaphoretickes-Chsp_4896_OG76
92.2/93
54.2/56
Cp_Diaphoretickes-Chsp_14051_OG76
76.6/55
Cp_Diaphoretickes-Bnat_13004_OG76
94.3/94
Other_Diaphoretickes-Omar_40877_OG76
Other_Amorphea-Dgra_36610_OG76
Other_Diaphoretickes-Aspp_6792_OG76
96.4/100
Other_Diaphoretickes-Aspp_6793_OG76
99.1/98
Other_Diaphoretickes-Aspp_3368_OG76
62.9/56
Pp_Diaphoretickes-Cvar_6176_OG76
84.3/60
Other_Stramenopiles-Bhom_5696_OG76
98.6/98
Other_Stramenopiles-Bhom_3189_OG76
Osmo_Fungi-Rory_4988_OG76
92.8/100
Osmo_Fungi-Rory_9103_OG76
99.4/100
Osmo_Fungi-Pbla_8911_OG76
89.7/90
Other_Other-Mexi_13084_OG76
85.4/66
85.4/67
Other_Other-Mexi_9855_OG76
0/56
86.4/62
Other_Other-Mexi_13018_OG76
95.2/91
Other_Other-Mexi_9743_OG76
80.5/60
Other_Other-Mexi_6433_OG76
Other_Holozoa-Nvec_26254_OG76
99.2/99
Other_Holozoa-Nvec_453_OG76
0/14
Osmo_Teretosporea-Sarc_28424_OG76
86.8/67
Osmo_Teretosporea-Cfra_4205_OG76
97/96
Osmo_Teretosporea-Cfra_6465_OG76
90.4/67
76.3/37
Other_Diaphoretickes-Rmar_6241_OG76
Other_Holozoa-Hsap_18287_OG76
82.1/80
Other_Holozoa-Hsap_6084_OG76
97.4/97
Other_Holozoa-Hsap_2690_OG76
92.8/71
65.8/8
Other_Holozoa-Hsap_21045_OG76
Other_Holozoa-Nvec_24941_OG76
96.4/47
78.4/77
Other_Amorphea-Bflo_1055_OG76
17.2/47
Osmo_Teretosporea-Apar_3376_OG76
92.3/67
Pp_Diaphoretickes-Crei_12310_OG76
78.9/69
Pp_Diaphoretickes-Vcar_9316_OG76
95.5/89
Pp_Diaphoretickes-Crei_10466_OG76
90.3/56
Other_Diaphoretickes-Rlim_18455_OG76
29.6/81
Osmo_Fungi-Cang_8338_OG76
34/25
Pp_Diaphoretickes-Npyr_7165_OG76
86.3/52
64.9/51
Other_Amorphea-Opea_26055_OG76
81.7/56
Other_Amorphea-Tspp_8051_OG76
Other_Holozoa-Dpul_21598_OG76

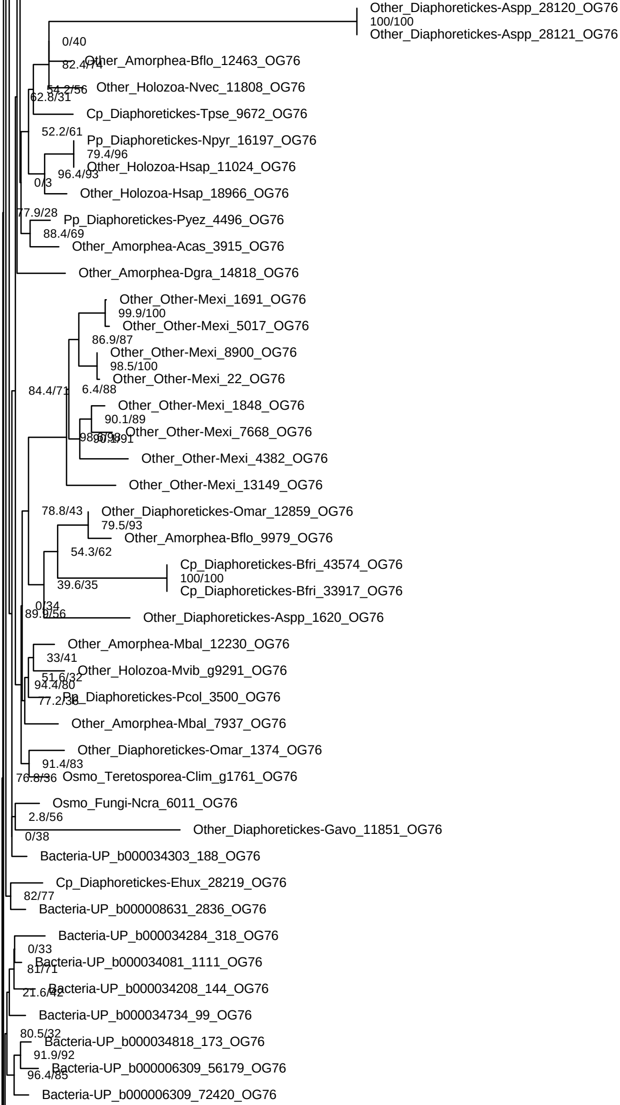
Other_Diaphoretickes-Aspp_28120_OG76
100/100
Other_Diaphoretickes-Aspp_28121_OG76
0/40
Other_Amorphea-Bflo_12463_OG76
82.4/74
54.2/56
Other_Holozoa-Nvec_11808_OG76
62.8/31
Cp_Diaphoretickes-Tpse_9672_OG76
52.2/61
Pp_Diaphoretickes-Npyr_16197_OG76
79.4/96
Other_Holozoa-Hsap_11024_OG76
96.4/93
0/3
Other_Holozoa-Hsap_18966_OG76
77.9/28
Pp_Diaphoretickes-Pyez_4496_OG76
88.4/69
Other_Amorphea-Acas_3915_OG76
Other_Amorphea-Dgra_14818_OG76
Other_Other-Mexi_1691_OG76
99.9/100
Other_Other-Mexi_5017_OG76
86.9/87
Other_Other-Mexi_8900_OG76
98.5/100
Other_Other-Mexi_22_OG76
84.4/71
6.4/88
Other_Other-Mexi_1848_OG76
90.1/89
Other_Other-Mexi_7668_OG76
Other_Other-Mexi_4382_OG76
Other_Other-Mexi_13149_OG76
78.8/43
Other_Diaphoretickes-Omar_12859_OG76
79.5/93
Other_Amorphea-Bflo_9979_OG76
54.3/62
Cp_Diaphoretickes-Bfri_43574_OG76
100/100
Cp_Diaphoretickes-Bfri_33917_OG76
39.6/35
0/34
89.9/56
Other_Diaphoretickes-Aspp_1620_OG76
Other_Amorphea-Mbal_12230_OG76
33/41
51.6/32
Other_Holozoa-Mvib_g9291_OG76
94.4/80
77.2/36
Pp_Diaphoretickes-Pcol_3500_OG76
Other_Amorphea-Mbal_7937_OG76
Other_Diaphoretickes-Omar_1374_OG76
91.4/83
76.8/36
Osmo_Teretosporea-Clim_g1761_OG76
Osmo_Fungi-Ncra_6011_OG76
2.8/56
Other_Diaphoretickes-Gavo_11851_OG76
0/38
Bacteria-UP_b000034303_188_OG76
Cp_Diaphoretickes-Ehux_28219_OG76
82/77
Bacteria-UP_b000008631_2836_OG76
Bacteria-UP_b000034284_318_OG76
0/33
Bacteria-UP_b000034081_1111_OG76
81/71
21.6/42
Bacteria-UP_b000034208_144_OG76
Bacteria-UP_b000034734_99_OG76
80.5/32
Bacteria-UP_b000034818_173_OG76
91.9/92
Bacteria-UP_b000006309_56179_OG76
96.4/85
Bacteria-UP_b000006309_72420_OG76

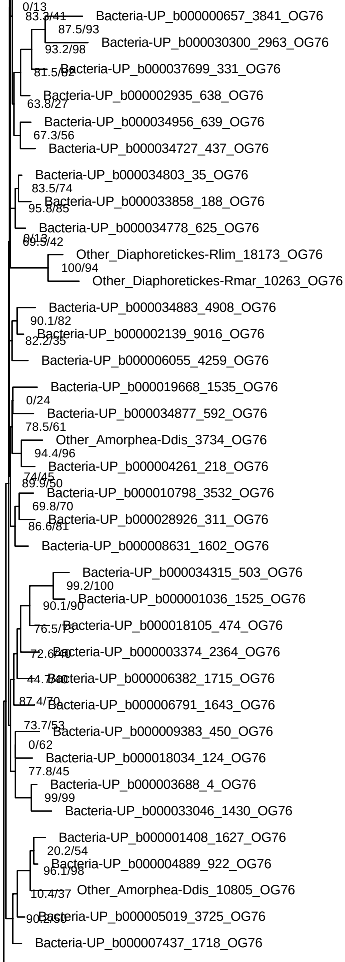
0/13
83.3/41
Bacteria-UP_b000000657_3841_OG76
87.5/93
Bacteria-UP_b000030300_2963_OG76
93.2/98
Bacteria-UP_b000037699_331_OG76
81.5/82
Bacteria-UP_b000002935_638_OG76
63.8/27
Bacteria-UP_b000034956_639_OG76
67.3/56
Bacteria-UP_b000034727_437_OG76
Bacteria-UP_b000034803_35_OG76
83.5/74
Bacteria-UP_b000033858_188_OG76
95.8/85
Bacteria-UP_b000034778_625_OG76
0/13
69.5/42
Other_Diaphoretickes-Rlim_18173_OG76
100/94
Other_Diaphoretickes-Rmar_10263_OG76
Bacteria-UP_b000034883_4908_OG76
90.1/82
Bacteria-UP_b000002139_9016_OG76
82.2/35
Bacteria-UP_b000006055_4259_OG76
Bacteria-UP_b000019668_1535_OG76
0/24
Bacteria-UP_b000034877_592_OG76
78.5/61
Other_Amorphea-Ddis_3734_OG76
94.4/96
Bacteria-UP_b000004261_218_OG76
74/45
89.9/50
Bacteria-UP_b000010798_3532_OG76
69.8/70
Bacteria-UP_b000028926_311_OG76
86.6/81
Bacteria-UP_b000008631_1602_OG76
Bacteria-UP_b000034315_503_OG76
99.2/100
Bacteria-UP_b000001036_1525_OG76
90.1/90
Bacteria-UP_b000018105_474_OG76
76.5/75
Bacteria-UP_b000003374_2364_OG76
Bacteria-UP_b000006382_1715_OG76
87.4/70
Bacteria-UP_b000006791_1643_OG76
73.7/53
Bacteria-UP_b000009383_450_OG76
0/62
Bacteria-UP_b000018034_124_OG76
77.8/45
Bacteria-UP_b000003688_4_OG76
99/99
Bacteria-UP_b000033046_1430_OG76
Bacteria-UP_b000001408_1627_OG76
20.2/54
Bacteria-UP_b000004889_922_OG76
96.1/98
Other_Amorphea-Ddis_10805_OG76
10.4/37
Bacteria-UP_b000005019_3725_OG76
Bacteria-UP_b000007437_1718_OG76

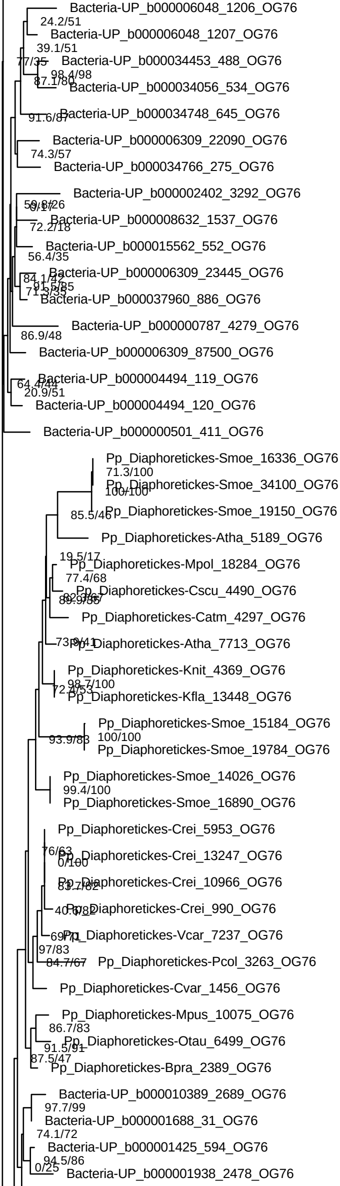

Bacteria-UP_b000006048_1206_OG76
24.2/51
Bacteria-UP_b000006048_1207_OG76
39.1/51
77/35
Bacteria-UP_b000034453_488_OG76
98.4/98
87.1/80
Bacteria-UP_b000034056_534_OG76
91.6/87
Bacteria-UP_b000034748_645_OG76
Bacteria-UP_b000006309_22090_OG76
74.3/57
Bacteria-UP_b000034766_275_OG76
Bacteria-UP_b000002402_3292_OG76
59.8/26
0/17
Bacteria-UP_b000008632_1537_OG76
72.2/18
Bacteria-UP_b000015562_552_OG76
56.4/35
Bacteria-UP_b000006309_23445_OG76
84.1/42
Bacteria-UP_b000037960_886_OG76
Bacteria-UP_b000000787_4279_OG76
86.9/48
Bacteria-UP_b000006309_87500_OG76
Bacteria-UP_b000004494_119_OG76
64.4/44
20.9/51
Bacteria-UP_b000004494_120_OG76
Bacteria-UP_b000000501_411_OG76
Pp_Diaphoretickes-Smoe_16336_OG76
71.3/100
Pp_Diaphoretickes-Smoe_34100_OG76
100/100
Pp_Diaphoretickes-Smoe_19150_OG76
85.5/46
Pp_Diaphoretickes-Atha_5189_OG76
19.5/17
Pp_Diaphoretickes-Mpol_18284_OG76
77.4/68
Pp_Diaphoretickes-Cscu_4490_OG76
Pp_Diaphoretickes-Catm_4297_OG76
Pp_Diaphoretickes-Atha_7713_OG76
Pp_Diaphoretickes-Knit_4369_OG76
98.7/100
72.4/53
Pp_Diaphoretickes-Kfla_13448_OG76
Pp_Diaphoretickes-Smoe_15184_OG76
100/100
93.9/83
Pp_Diaphoretickes-Smoe_19784_OG76
Pp_Diaphoretickes-Smoe_14026_OG76
99.4/100
Pp_Diaphoretickes-Smoe_16890_OG76
Pp_Diaphoretickes-Crei_5953_OG76
76/63
0/100
Pp_Diaphoretickes-Crei_13247_OG76
Pp_Diaphoretickes-Crei_10966_OG76
Pp_Diaphoretickes-Crei_990_OG76
Pp_Diaphoretickes-Vcar_7237_OG76
97/83
84.7/67
Pp_Diaphoretickes-Pcol_3263_OG76
Pp_Diaphoretickes-Cvar_1456_OG76
Pp_Diaphoretickes-Mpus_10075_OG76
86.7/83
Pp_Diaphoretickes-Otau_6499_OG76
91.5/91
87.5/47
Pp_Diaphoretickes-Bpra_2389_OG76
Bacteria-UP_b000010389_2689_OG76
97.7/99
Bacteria-UP_b000001688_31_OG76
74.1/72
Bacteria-UP_b000001425_594_OG76
94.5/86
0/25
Bacteria-UP_b000001938_2478_OG76

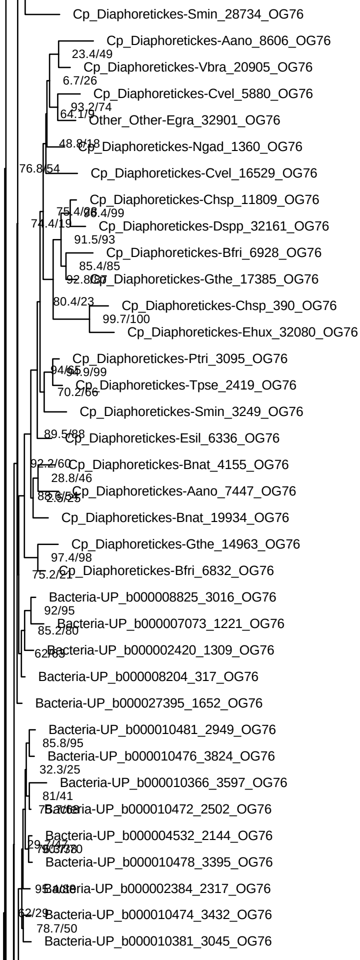
Cp_Diaphoretickes-Smin_28734_OG76
Cp_Diaphoretickes-Aano_8606_OG76
23.4/49
Cp_Diaphoretickes-Vbra_20905_OG76
6.7/26
Cp_Diaphoretickes-Cvel_5880_OG76
93.2/74
64.1/9
Other_Other-Egra_32901_OG76
48.8/18
Cp_Diaphoretickes-Ngad_1360_OG76
76.8/54
Cp_Diaphoretickes-Cvel_16529_OG76
Cp_Diaphoretickes-Chsp_11809_OG76
74.4/19
Cp_Diaphoretickes-Dspp_32161_OG76
91.5/93
Cp_Diaphoretickes-Bfri_6928_OG76
85.4/85
Cp_Diaphoretickes-Gthe_17385_OG76
80.4/23
Cp_Diaphoretickes-Chsp_390_OG76
99.7/100
Cp_Diaphoretickes-Ehux_32080_OG76
Cp_Diaphoretickes-Ptri_3095_OG76
Cp_Diaphoretickes-Tpse_2419_OG76
70.2/66
Cp_Diaphoretickes-Smin_3249_OG76
89.5/88
Cp_Diaphoretickes-Esil_6336_OG76
92.2/60
Cp_Diaphoretickes-Bnat_4155_OG76
28.8/46
Cp_Diaphoretickes-Aano_7447_OG76
Cp_Diaphoretickes-Bnat_19934_OG76
Cp_Diaphoretickes-Gthe_14963_OG76
97.4/98
75.2/21
Cp_Diaphoretickes-Bfri_6832_OG76
Bacteria-UP_b000008825_3016_OG76
92/95
Bacteria-UP_b000007073_1221_OG76
85.2/80
Bacteria-UP_b000002420_1309_OG76
Bacteria-UP_b000008204_317_OG76
Bacteria-UP_b000027395_1652_OG76
Bacteria-UP_b000010481_2949_OG76
85.8/95
Bacteria-UP_b000010476_3824_OG76
32.3/25
Bacteria-UP_b000010366_3597_OG76
81/41
Bacteria-UP_b000010472_2502_OG76
Bacteria-UP_b000004532_2144_OG76
Bacteria-UP_b000010478_3395_OG76
Bacteria-UP_b000002384_2317_OG76
62/29
Bacteria-UP_b000010474_3432_OG76
78.7/50
Bacteria-UP_b000010381_3045_OG76

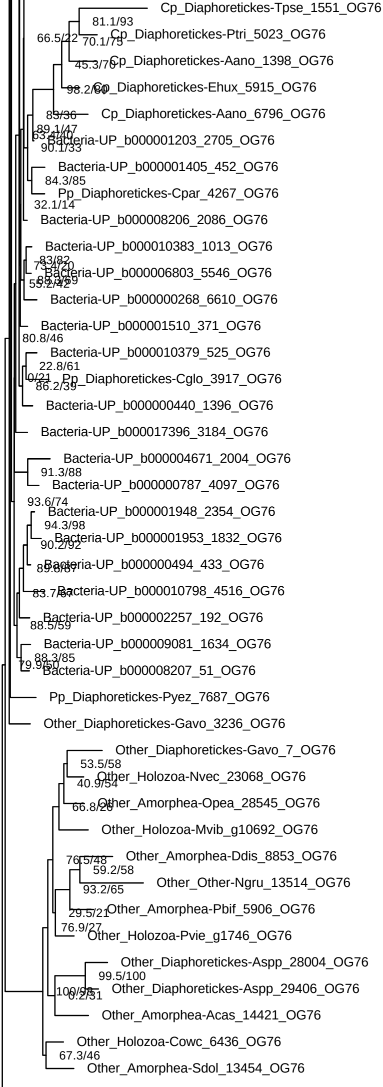
Cp_Diaphoretickes-Tpse_1551_OG76
81.1/93
66.5/22
70.1/75
Cp_Diaphoretickes-Ptri_5023_OG76
45.3/70
Cp_Diaphoretickes-Aano_1398_OG76
98.2/60
Cp_Diaphoretickes-Ehux_5915_OG76
83/36
Cp_Diaphoretickes-Aano_6796_OG76
89.1/47
63.4/40
90.1/33
Bacteria-UP_b000001203_2705_OG76
Bacteria-UP_b000001405_452_OG76
84.3/85
Pp_Diaphoretickes-Cpar_4267_OG76
32.1/14
Bacteria-UP_b000008206_2086_OG76
Bacteria-UP_b000010383_1013_OG76
83/82
73.4/20
Bacteria-UP_b000006803_5546_OG76
88.3/59
55.2/42
Bacteria-UP_b000000268_6610_OG76
Bacteria-UP_b000001510_371_OG76
80.8/46
Bacteria-UP_b000010379_525_OG76
22.8/61
0/21
86.2/39
Pp_Diaphoretickes-Cglo_3917_OG76
Bacteria-UP_b000000440_1396_OG76
Bacteria-UP_b000017396_3184_OG76
Bacteria-UP_b000004671_2004_OG76
91.3/88
Bacteria-UP_b000000787_4097_OG76
93.6/74
Bacteria-UP_b000001948_2354_OG76
94.3/98
Bacteria-UP_b000001953_1832_OG76
90.2/92
Bacteria-UP_b000000494_433_OG76
89.6/87
Bacteria-UP_b000010798_4516_OG76
83.7/67
Bacteria-UP_b000002257_192_OG76
88.5/59
Bacteria-UP_b000009081_1634_OG76
88.3/85
79.9/50
Bacteria-UP_b000008207_51_OG76
Pp_Diaphoretickes-Pyez_7687_OG76
Other_Diaphoretickes-Gavo_3236_OG76
Other_Diaphoretickes-Gavo_7_OG76
53.5/58
Other_Holozoa-Nvec_23068_OG76
40.9/54
Other_Amorphea-Opea_28545_OG76
66.8/26
Other_Holozoa-Mvib_g10692_OG76
Other_Amorphea-Ddis_8853_OG76
76.5/48
59.2/58
Other_Other-Ngru_13514_OG76
93.2/65
Other_Amorphea-Pbif_5906_OG76
29.5/21
76.9/27
Other_Holozoa-Pvie_g1746_OG76
Other_Diaphoretickes-Aspp_28004_OG76
99.5/100
100/98
0.2/31
Other_Diaphoretickes-Aspp_29406_OG76
Other_Amorphea-Acas_14421_OG76
Other_Holozoa-Cowc_6436_OG76
67.3/46
Other_Amorphea-Sdol_13454_OG76

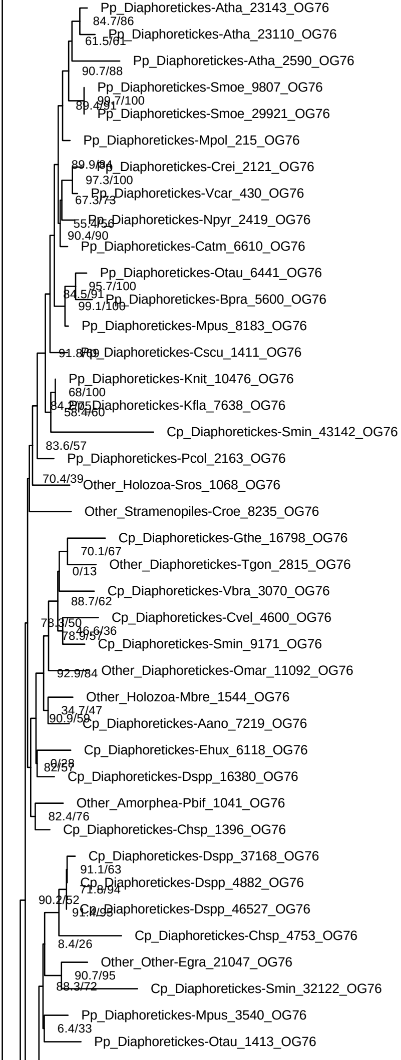
Pp_Diaphoretickes-Atha_23143_OG76
84.7/86
Pp_Diaphoretickes-Atha_23110_OG76
61.5/61
Pp_Diaphoretickes-Atha_2590_OG76
90.7/88
Pp_Diaphoretickes-Smoe_9807_OG76
99.7/100
89.4/91
Pp_Diaphoretickes-Smoe_29921_OG76
Pp_Diaphoretickes-Mpol_215_OG76
89.9/84
Pp_Diaphoretickes-Crei_2121_OG76
97.3/100
Pp_Diaphoretickes-Vcar_430_OG76
67.3/73
Pp_Diaphoretickes-Npyr_2419_OG76
55.4/56
90.4/90
Pp_Diaphoretickes-Catm_6610_OG76
Pp_Diaphoretickes-Otau_6441_OG76
95.7/100
84.5/91
Pp_Diaphoretickes-Bpra_5600_OG76
99.1/100
Pp_Diaphoretickes-Mpus_8183_OG76
91.8/69
Pp_Diaphoretickes-Cscu_1411_OG76
Pp_Diaphoretickes-Knit_10476_OG76
68/100
84.2/75
Pp_Diaphoretickes-Kfla_7638_OG76
58.4/60
Cp_Diaphoretickes-Smin_43142_OG76
83.6/57
Pp_Diaphoretickes-Pcol_2163_OG76
70.4/39
Other_Holozoa-Sros_1068_OG76
Other_Stramenopiles-Croe_8235_OG76
Cp_Diaphoretickes-Gthe_16798_OG76
70.1/67
Other_Diaphoretickes-Tgon_2815_OG76
0/13
Cp_Diaphoretickes-Vbra_3070_OG76
88.7/62
Cp_Diaphoretickes-Cvel_4600_OG76
78.3/50
46.6/36
78.9/57
Cp_Diaphoretickes-Smin_9171_OG76
92.9/84
Other_Diaphoretickes-Omar_11092_OG76
Other_Holozoa-Mbre_1544_OG76
34.7/47
90.9/59
Cp_Diaphoretickes-Aano_7219_OG76
Cp_Diaphoretickes-Ehux_6118_OG76
0/28
82/57
Cp_Diaphoretickes-Dspp_16380_OG76
Other_Amorphea-Pbif_1041_OG76
82.4/76
Cp_Diaphoretickes-Chsp_1396_OG76
Cp_Diaphoretickes-Dspp_37168_OG76
91.1/63
Cp_Diaphoretickes-Dspp_4882_OG76
71.8/94
90.2/52
Cp_Diaphoretickes-Dspp_46527_OG76
91.4/95
Cp_Diaphoretickes-Chsp_4753_OG76
8.4/26
Other_Other-Egra_21047_OG76
90.7/95
88.3/72
Cp_Diaphoretickes-Smin_32122_OG76
Pp_Diaphoretickes-Mpus_3540_OG76
6.4/33
Pp_Diaphoretickes-Otau_1413_OG76

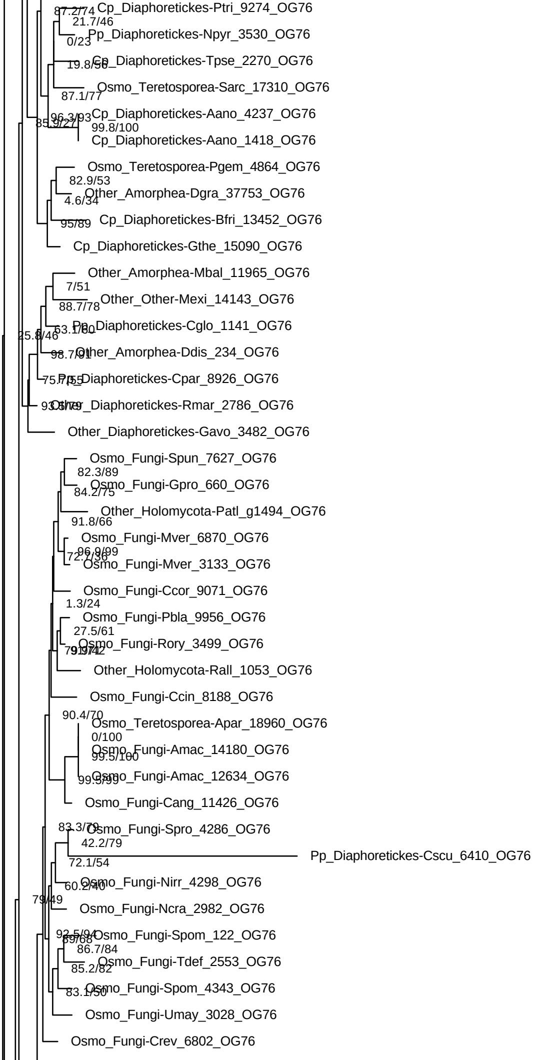

87.2/74
Cp_Diaphoretickes-Ptri_9274_OG76
21.7/46
Pp_Diaphoretickes-Npyr_3530_OG76
0/23
Cp_Diaphoretickes-Tpse_2270_OG76
19.8/56
Osmo_Teretosporea-Sarc_17310_OG76
87.1/77
96.3/93
Cp_Diaphoretickes-Aano_4237_OG76
85.9/27
99.8/100
Cp_Diaphoretickes-Aano_1418_OG76
Osmo_Teretosporea-Pgem_4864_OG76
82.9/53
Other_Amorphea-Dgra_37753_OG76
4.6/34
95/89
Cp_Diaphoretickes-Bfri_13452_OG76
Cp_Diaphoretickes-Gthe_15090_OG76
Other_Amorphea-Mbal_11965_OG76
7/51
88.7/78
Other_Other-Mexi_14143_OG76
Pp_Diaphoretickes-Cglo_1141_OG76
63.1/60
25.8/46
98.7/91
Other_Amorphea-Ddis_234_OG76
75.7/55
Pp_Diaphoretickes-Cpar_8926_OG76
93.8/79
Other_Diaphoretickes-Rmar_2786_OG76
Other_Diaphoretickes-Gavo_3482_OG76
Osmo_Fungi-Spun_7627_OG76
82.3/89
Osmo_Fungi-Gpro_660_OG76
84.2/75
Other_Holomycota-Patl_g1494_OG76
91.8/66
Osmo_Fungi-Mver_6870_OG76
96.9/99
72.7/36
Osmo_Fungi-Mver_3133_OG76
Osmo_Fungi-Ccor_9071_OG76
1.3/24
Osmo_Fungi-Pbla_9956_OG76
27.5/61
Osmo_Fungi-Rory_3499_OG76
Other_Holomycota-Rall_1053_OG76
Osmo_Fungi-Ccin_8188_OG76
90.4/70
Osmo_Teretosporea-Apar_18960_OG76
0/100
Osmo_Fungi-Amac_14180_OG76
99.5/100
Osmo_Fungi-Amac_12634_OG76
99.3/99
Osmo_Fungi-Cang_11426_OG76
83.3/79
Osmo_Fungi-Spro_4286_OG76
42.2/79
Pp_Diaphoretickes-Cscu_6410_OG76
72.1/54
Osmo_Fungi-Nirr_4298_OG76
60.2/40
79/49
Osmo_Fungi-Ncra_2982_OG76
92.5/94
59/68
Osmo_Fungi-Spom_122_OG76
86.7/84
Osmo_Fungi-Tdef_2553_OG76
85.2/82
Osmo_Fungi-Spom_4343_OG76
83.1/50
Osmo_Fungi-Umay_3028_OG76
Osmo_Fungi-Crev_6802_OG76

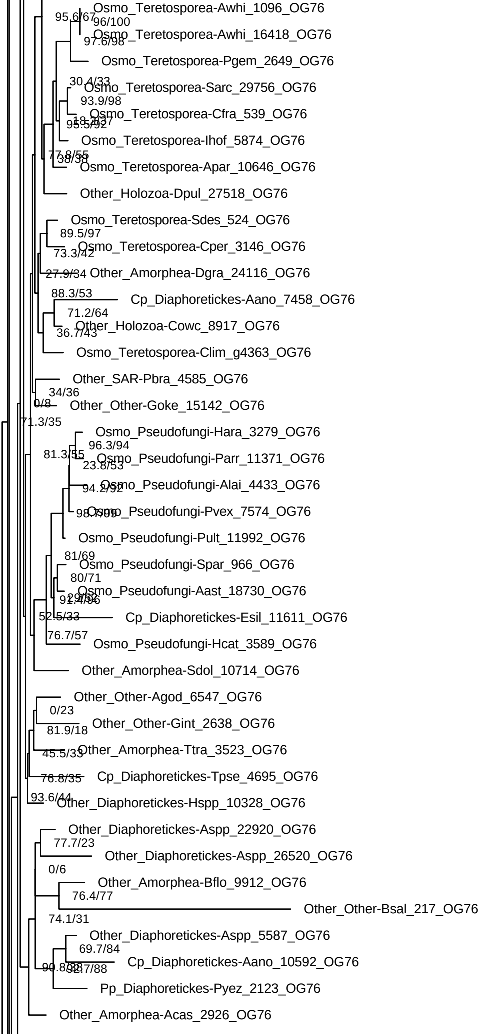
Osmo_Teretosporea-Awhi_1096_OG76
95.6/67
96/100
Osmo_Teretosporea-Awhi_16418_OG76
97.6/98
Osmo_Teretosporea-Pgem_2649_OG76
30.4/33
Osmo_Teretosporea-Sarc_29756_OG76
93.9/98
Osmo_Teretosporea-Cfra_539_OG76
Osmo_Teretosporea-Ihof_5874_OG76
77.8/55
38/38
Osmo_Teretosporea-Apar_10646_OG76
Other_Holozoa-Dpul_27518_OG76
Osmo_Teretosporea-Sdes_524_OG76
89.5/97
Osmo_Teretosporea-Cper_3146_OG76
73.3/42
27.9/34
Other_Amorphea-Dgra_24116_OG76
88.3/53
Cp_Diaphoretickes-Aano_7458_OG76
71.2/64
Other_Holozoa-Cowc_8917_OG76
36.7/43
Osmo_Teretosporea-Clim_g4363_OG76
Other_SAR-Pbra_4585_OG76
34/36
0/8
Other_Other-Goke_15142_OG76
71.3/35
Osmo_Pseudofungi-Hara_3279_OG76
96.3/94
81.3/55
Osmo_Pseudofungi-Parr_11371_OG76
23.8/53
Osmo_Pseudofungi-Alai_4433_OG76
94.2/92
Osmo_Pseudofungi-Pvex_7574_OG76
Osmo_Pseudofungi-Pult_11992_OG76
81/69
Osmo_Pseudofungi-Spar_966_OG76
80/71
Osmo_Pseudofungi-Aast_18730_OG76
52.5/33
Cp_Diaphoretickes-Esil_11611_OG76
76.7/57
Osmo_Pseudofungi-Hcat_3589_OG76
Other_Amorphea-Sdol_10714_OG76
Other_Other-Agod_6547_OG76
0/23
Other_Other-Gint_2638_OG76
81.9/18
Other_Amorphea-Ttra_3523_OG76
45.5/33
76.8/35
Cp_Diaphoretickes-Tpse_4695_OG76
93.6/44
Other_Diaphoretickes-Hspp_10328_OG76
Other_Diaphoretickes-Aspp_22920_OG76
77.7/23
Other_Diaphoretickes-Aspp_26520_OG76
0/6
Other_Amorphea-Bflo_9912_OG76
76.4/77
Other_Other-Bsal_217_OG76
74.1/31
Other_Diaphoretickes-Aspp_5587_OG76
69.7/84
Cp_Diaphoretickes-Aano_10592_OG76
Pp_Diaphoretickes-Pyez_2123_OG76
Other_Amorphea-Acas_2926_OG76

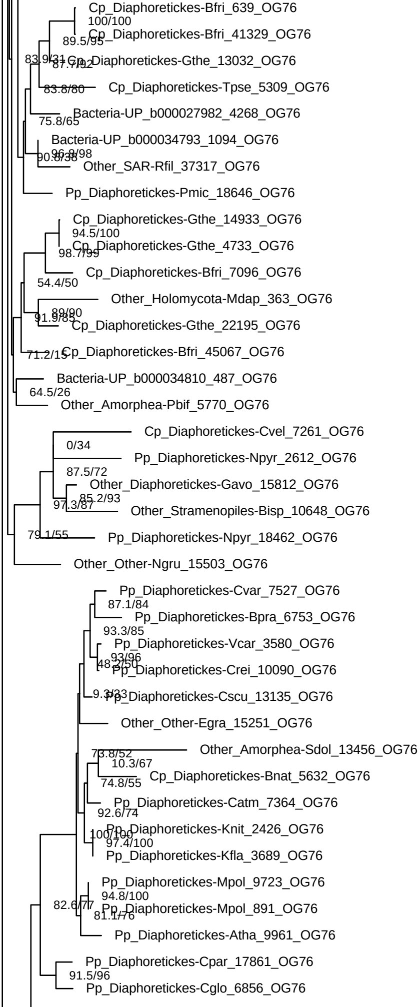
Cp_Diaphoretickes-Bfri_639_OG76
100/100
Cp_Diaphoretickes-Bfri_41329_OG76
89.5/95
83.9/31
87.7/92
Cp_Diaphoretickes-Gthe_13032_OG76
83.8/80
Cp_Diaphoretickes-Tpse_5309_OG76
75.8/65
Bacteria-UP_b000027982_4268_OG76
Bacteria-UP_b000034793_1094_OG76
96.8/98
90.8/38
Other_SAR-Rfil_37317_OG76
Pp_Diaphoretickes-Pmic_18646_OG76
Cp_Diaphoretickes-Gthe_14933_OG76
94.5/100
98.7/99
Cp_Diaphoretickes-Gthe_4733_OG76
Cp_Diaphoretickes-Bfri_7096_OG76
54.4/50
Other_Holomycota-Mdap_363_OG76
89/90
91.9/85
Cp_Diaphoretickes-Gthe_22195_OG76
71.2/15
Cp_Diaphoretickes-Bfri_45067_OG76
Bacteria-UP_b000034810_487_OG76
64.5/26
Other_Amorphea-Pbif_5770_OG76
Cp_Diaphoretickes-Cvel_7261_OG76
0/34
Pp_Diaphoretickes-Npyr_2612_OG76
87.5/72
Other_Diaphoretickes-Gavo_15812_OG76
85.2/93
97.3/87
Other_Stramenopiles-Bisp_10648_OG76
79.1/55
Pp_Diaphoretickes-Npyr_18462_OG76
Other_Other-Ngru_15503_OG76
Pp_Diaphoretickes-Cvar_7527_OG76
87.1/84
Pp_Diaphoretickes-Bpra_6753_OG76
93.3/85
Pp_Diaphoretickes-Vcar_3580_OG76
93/96
48.2/50
Pp_Diaphoretickes-Crei_10090_OG76
9.3/33
Pp_Diaphoretickes-Cscu_13135_OG76
Other_Other-Egra_15251_OG76
73.8/52
Other_Amorphea-Sdol_13456_OG76
10.3/67
74.8/55
Cp_Diaphoretickes-Bnat_5632_OG76
Pp_Diaphoretickes-Catm_7364_OG76
92.6/74
100/100
Pp_Diaphoretickes-Knit_2426_OG76
97.4/100
Pp_Diaphoretickes-Kfla_3689_OG76
Pp_Diaphoretickes-Mpol_9723_OG76
94.8/100
82.6/77
Pp_Diaphoretickes-Mpol_891_OG76
81.1/76
Pp_Diaphoretickes-Atha_9961_OG76
Pp_Diaphoretickes-Cpar_17861_OG76
91.5/96
Pp_Diaphoretickes-Cglo_6856_OG76

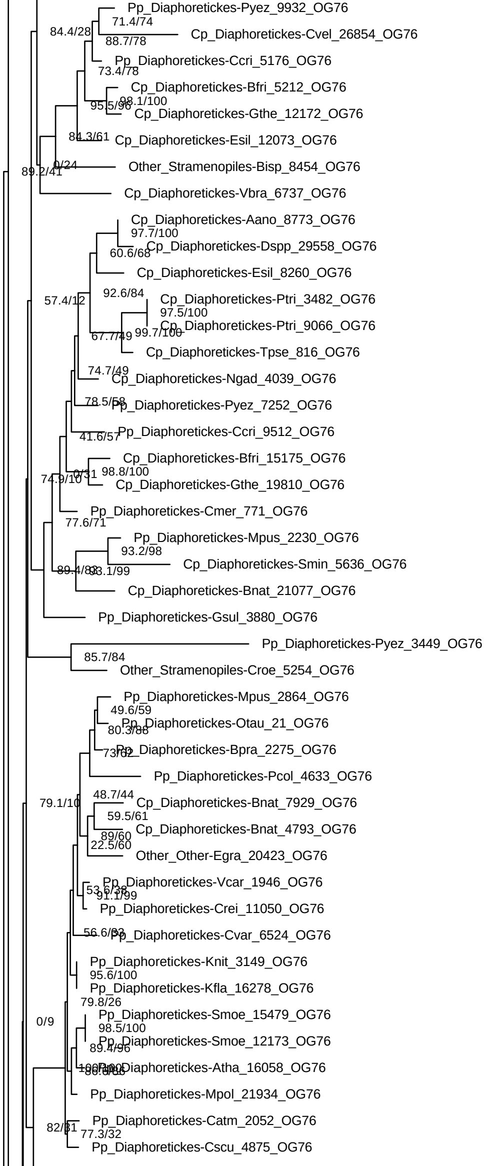

Pp_Diaphoretickes-Pyez_9932_OG76
71.4/74
84.4/28
Cp_Diaphoretickes-Cvel_26854_OG76
88.7/78
Pp_Diaphoretickes-Ccri_5176_OG76
73.4/78
Cp_Diaphoretickes-Bfri_5212_OG76
98.1/100
95.5/96
Cp_Diaphoretickes-Gthe_12172_OG76
84.3/61
Cp_Diaphoretickes-Esil_12073_OG76
0/24
Other_Stramenopiles-Bisp_8454_OG76
89.2/41
Cp_Diaphoretickes-Vbra_6737_OG76
Cp_Diaphoretickes-Aano_8773_OG76
97.7/100
60.6/68
Cp_Diaphoretickes-Dspp_29558_OG76
Cp_Diaphoretickes-Esil_8260_OG76
92.6/84
57.4/12
Cp_Diaphoretickes-Ptri_3482_OG76
97.5/100
Cp_Diaphoretickes-Ptri_9066_OG76
67.7/49
99.7/100
Cp_Diaphoretickes-Tpse_816_OG76
74.7/49
Cp_Diaphoretickes-Ngad_4039_OG76
78.5/58
Pp_Diaphoretickes-Pyez_7252_OG76
41.6/57
Pp_Diaphoretickes-Ccri_9512_OG76
Cp_Diaphoretickes-Bfri_15175_OG76
0/31
98.8/100
74.9/10
Cp_Diaphoretickes-Gthe_19810_OG76
Pp_Diaphoretickes-Cmer_771_OG76
77.6/71
Pp_Diaphoretickes-Mpus_2230_OG76
93.2/98
Cp_Diaphoretickes-Smin_5636_OG76
89.4/83
93.1/99
Cp_Diaphoretickes-Bnat_21077_OG76
Pp_Diaphoretickes-Gsul_3880_OG76
Pp_Diaphoretickes-Pyez_3449_OG76
85.7/84
Other_Stramenopiles-Croe_5254_OG76
Pp_Diaphoretickes-Mpus_2864_OG76
49.6/59
Pp_Diaphoretickes-Otau_21_OG76
80.3/88
73/62
Pp_Diaphoretickes-Bpra_2275_OG76
Pp_Diaphoretickes-Pcol_4633_OG76
48.7/44
79.1/10
Cp_Diaphoretickes-Bnat_7929_OG76
59.5/61
Cp_Diaphoretickes-Bnat_4793_OG76
89/60
22.5/60
Other_Other-Egra_20423_OG76
Pp_Diaphoretickes-Vcar_1946_OG76
53.6/38
91.1/99
Pp_Diaphoretickes-Crei_11050_OG76
56.6/32
Pp_Diaphoretickes-Cvar_6524_OG76
Pp_Diaphoretickes-Knit_3149_OG76
95.6/100
Pp_Diaphoretickes-Kfla_16278_OG76
79.8/26
0/9
Pp_Diaphoretickes-Smoe_15479_OG76
98.5/100
Pp_Diaphoretickes-Smoe_12173_OG76
89.4/96
100/100
86.6/85
Pp_Diaphoretickes-Atha_16058_OG76
Pp_Diaphoretickes-Mpol_21934_OG76
Pp_Diaphoretickes-Catm_2052_OG76
82/31
77.3/32
Pp_Diaphoretickes-Cscu_4875_OG76

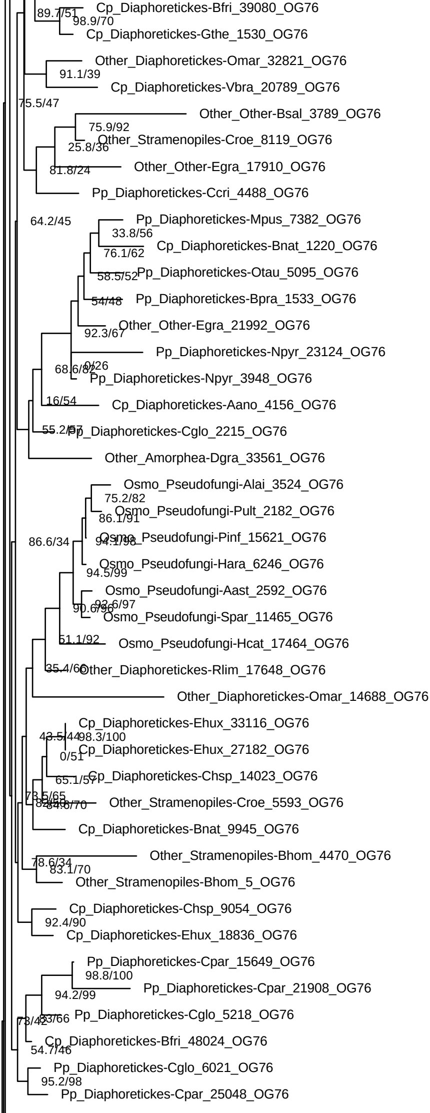

Cp_Diaphoretickes-Bfri_39080_OG76
89.7/51
98.9/70
Cp_Diaphoretickes-Gthe_1530_OG76
Other_Diaphoretickes-Omar_32821_OG76
91.1/39
Cp_Diaphoretickes-Vbra_20789_OG76
75.5/47
Other_Other-Bsal_3789_OG76
75.9/92
Other_Stramenopiles-Croe_8119_OG76
25.8/36
Other_Other-Egra_17910_OG76
81.8/24
Pp_Diaphoretickes-Ccri_4488_OG76
64.2/45
Pp_Diaphoretickes-Mpus_7382_OG76
33.8/56
Cp_Diaphoretickes-Bnat_1220_OG76
76.1/62
Pp_Diaphoretickes-Otau_5095_OG76
58.5/52
Pp_Diaphoretickes-Bpra_1533_OG76
54/48
Other_Other-Egra_21992_OG76
92.3/67
Pp_Diaphoretickes-Npyr_23124_OG76
0/26
68.6/82
Pp_Diaphoretickes-Npyr_3948_OG76
16/54
Cp_Diaphoretickes-Aano_4156_OG76
55.2/57
Pp_Diaphoretickes-Cglo_2215_OG76
Other_Amorphea-Dgra_33561_OG76
Osmo_Pseudofungi-Alai_3524_OG76
75.2/82
Osmo_Pseudofungi-Pult_2182_OG76
86.1/91
86.6/34
Osmo_Pseudofungi-Pinf_15621_OG76
94.1/98
Osmo_Pseudofungi-Hara_6246_OG76
94.5/99
Osmo_Pseudofungi-Aast_2592_OG76
92.6/97
90.6/96
Osmo_Pseudofungi-Spar_11465_OG76
51.1/92
Osmo_Pseudofungi-Hcat_17464_OG76
35.4/66
Other_Diaphoretickes-Rlim_17648_OG76
Other_Diaphoretickes-Omar_14688_OG76
Cp_Diaphoretickes-Ehux_33116_OG76
43.5/44
98.3/100
Cp_Diaphoretickes-Ehux_27182_OG76
0/51
Cp_Diaphoretickes-Chsp_14023_OG76
65.1/57
73.5/65
84.6/70
Other_Stramenopiles-Croe_5593_OG76
Cp_Diaphoretickes-Bnat_9945_OG76
Other_Stramenopiles-Bhom_4470_OG76
78.6/34
83.1/70
Other_Stramenopiles-Bhom_5_OG76
Cp_Diaphoretickes-Chsp_9054_OG76
92.4/90
Cp_Diaphoretickes-Ehux_18836_OG76
Pp_Diaphoretickes-Cpar_15649_OG76
98.8/100
Pp_Diaphoretickes-Cpar_21908_OG76
94.2/99
Pp_Diaphoretickes-Cglo_5218_OG76
73/42
Cp_Diaphoretickes-Bfri_48024_OG76
54.7/46
Pp_Diaphoretickes-Cglo_6021_OG76
95.2/98
Pp_Diaphoretickes-Cpar_25048_OG76

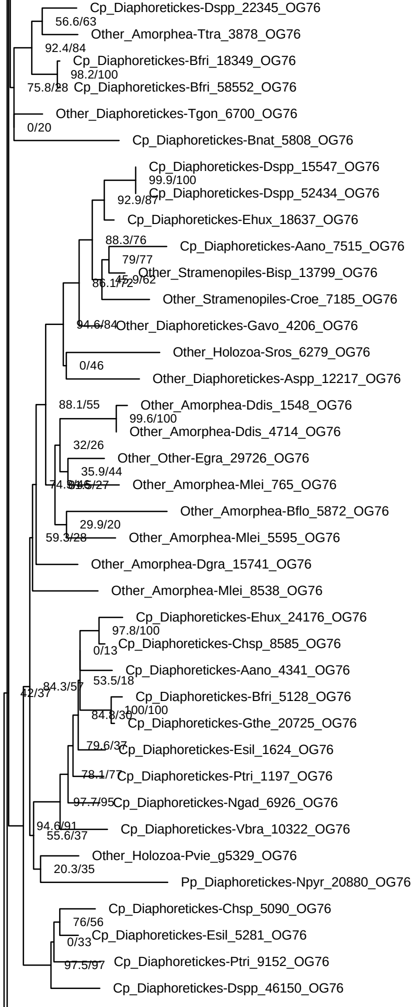
Cp_Diaphoretickes-Dspp_22345_OG76
56.6/63
Other_Amorphea-Ttra_3878_OG76
92.4/84
Cp_Diaphoretickes-Bfri_18349_OG76
98.2/100
75.8/28
Cp_Diaphoretickes-Bfri_58552_OG76
Other_Diaphoretickes-Tgon_6700_OG76
0/20
Cp_Diaphoretickes-Bnat_5808_OG76
Cp_Diaphoretickes-Dspp_15547_OG76
99.9/100
92.9/87
Cp_Diaphoretickes-Dspp_52434_OG76
Cp_Diaphoretickes-Ehux_18637_OG76
88.3/76
Cp_Diaphoretickes-Aano_7515_OG76
79/77
Other_Stramenopiles-Bisp_13799_OG76
45.9/62
86.1/72
Other_Stramenopiles-Croe_7185_OG76
94.6/84
Other_Diaphoretickes-Gavo_4206_OG76
Other_Holozoa-Sros_6279_OG76
0/46
Other_Diaphoretickes-Aspp_12217_OG76
88.1/55
Other_Amorphea-Ddis_1548_OG76
99.6/100
Other_Amorphea-Ddis_4714_OG76
32/26
Other_Other-Egra_29726_OG76
35.9/44
Other_Amorphea-Mlei_765_OG76
Other_Amorphea-Bflo_5872_OG76
29.9/20
59.3/28
Other_Amorphea-Mlei_5595_OG76
Other_Amorphea-Dgra_15741_OG76
Other_Amorphea-Mlei_8538_OG76
Cp_Diaphoretickes-Ehux_24176_OG76
97.8/100
0/13
Cp_Diaphoretickes-Chsp_8585_OG76
Cp_Diaphoretickes-Aano_4341_OG76
53.5/18
84.3/57
42/37
Cp_Diaphoretickes-Bfri_5128_OG76
100/100
84.8/30
Cp_Diaphoretickes-Gthe_20725_OG76
79.6/37
Cp_Diaphoretickes-Esil_1624_OG76
78.1/77
Cp_Diaphoretickes-Ptri_1197_OG76
97.7/95
Cp_Diaphoretickes-Ngad_6926_OG76
94.6/91
55.6/37
Cp_Diaphoretickes-Vbra_10322_OG76
Other_Holozoa-Pvie_g5329_OG76
20.3/35
Pp_Diaphoretickes-Npyr_20880_OG76
Cp_Diaphoretickes-Chsp_5090_OG76
76/56
0/33
Cp_Diaphoretickes-Esil_5281_OG76
97.5/97
Cp_Diaphoretickes-Ptri_9152_OG76
Cp_Diaphoretickes-Dspp_46150_OG76

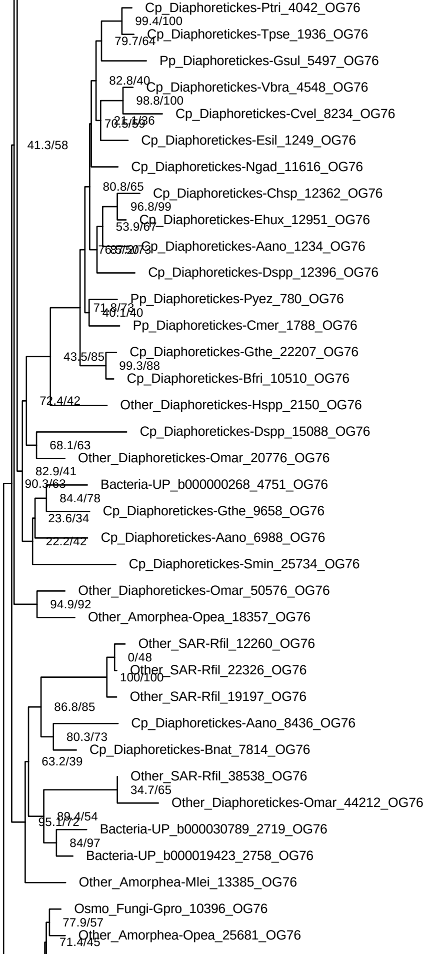
Cp_Diaphoretickes-Ptri_4042_OG76
99.4/100
79.7/64
Cp_Diaphoretickes-Tpse_1936_OG76
Pp_Diaphoretickes-Gsul_5497_OG76
82.8/40
Cp_Diaphoretickes-Vbra_4548_OG76
98.8/100
Cp_Diaphoretickes-Cvel_8234_OG76
Cp_Diaphoretickes-Esil_1249_OG76
41.3/58
Cp_Diaphoretickes-Ngad_11616_OG76
80.8/65
Cp_Diaphoretickes-Chsp_12362_OG76
96.8/99
Cp_Diaphoretickes-Ehux_12951_OG76
53.9/67
Cp_Diaphoretickes-Aano_1234_OG76
Cp_Diaphoretickes-Dspp_12396_OG76
Pp_Diaphoretickes-Pyez_780_OG76
40.1/40
Pp_Diaphoretickes-Cmer_1788_OG76
43.5/85
Cp_Diaphoretickes-Gthe_22207_OG76
99.3/88
Cp_Diaphoretickes-Bfri_10510_OG76
72.4/42
Other_Diaphoretickes-Hspp_2150_OG76
Cp_Diaphoretickes-Dspp_15088_OG76
68.1/63
Other_Diaphoretickes-Omar_20776_OG76
82.9/41
90.3/63
Bacteria-UP_b000000268_4751_OG76
84.4/78
Cp_Diaphoretickes-Gthe_9658_OG76
23.6/34
Cp_Diaphoretickes-Aano_6988_OG76
22.2/42
Cp_Diaphoretickes-Smin_25734_OG76
Other_Diaphoretickes-Omar_50576_OG76
94.9/92
Other_Amorphea-Opea_18357_OG76
Other_SAR-Rfil_12260_OG76
0/48
Other_SAR-Rfil_22326_OG76
100/100
Other_SAR-Rfil_19197_OG76
86.8/85
Cp_Diaphoretickes-Aano_8436_OG76
80.3/73
Cp_Diaphoretickes-Bnat_7814_OG76
63.2/39
Other_SAR-Rfil_38538_OG76
34.7/65
Other_Diaphoretickes-Omar_44212_OG76
89.4/54
95.1/72
Bacteria-UP_b000030789_2719_OG76
84/97
Bacteria-UP_b000019423_2758_OG76
Other_Amorphea-Mlei_13385_OG76
Osmo_Fungi-Gpro_10396_OG76
77.9/57
Other_Amorphea-Opea_25681_OG76
71.4/45

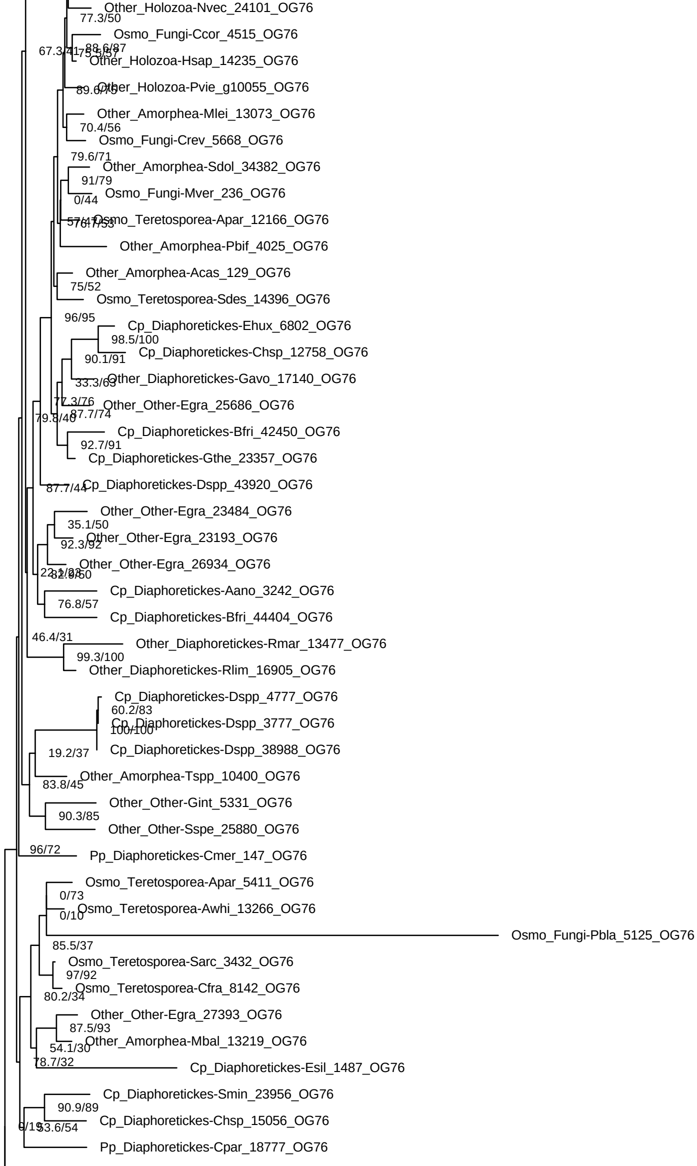
Other_Holozoa-Nvec_24101_OG76
77.3/50
Osmo_Fungi-Ccor_4515_OG76
88.6/87
Other_Holozoa-Hsap_14235_OG76
Other_Holozoa-Pvie_g10055_OG76
Other_Amorphea-Mlei_13073_OG76
70.4/56
Osmo_Fungi-Crev_5668_OG76
79.6/71
Other_Amorphea-Sdol_34382_OG76
91/79
Osmo_Fungi-Mver_236_OG76
0/44
Osmo_Teretosporea-Apar_12166_OG76
Other_Amorphea-Pbif_4025_OG76
Other_Amorphea-Acas_129_OG76
75/52
Osmo_Teretosporea-Sdes_14396_OG76
96/95
Cp_Diaphoretickes-Ehux_6802_OG76
98.5/100
Cp_Diaphoretickes-Chsp_12758_OG76
90.1/91
Other_Diaphoretickes-Gavo_17140_OG76
33.3/63
77.3/76
Other_Other-Egra_25686_OG76
87.7/74
79.8/40
Cp_Diaphoretickes-Bfri_42450_OG76
92.7/91
Cp_Diaphoretickes-Gthe_23357_OG76
87.7/44
Cp_Diaphoretickes-Dspp_43920_OG76
Other_Other-Egra_23484_OG76
35.1/50
Other_Other-Egra_23193_OG76
92.3/92
Other_Other-Egra_26934_OG76
Cp_Diaphoretickes-Aano_3242_OG76
76.8/57
Cp_Diaphoretickes-Bfri_44404_OG76
46.4/31
Other_Diaphoretickes-Rmar_13477_OG76
99.3/100
Other_Diaphoretickes-Rlim_16905_OG76
Cp_Diaphoretickes-Dspp_4777_OG76
60.2/83
Cp_Diaphoretickes-Dspp_3777_OG76
100/100
Cp_Diaphoretickes-Dspp_38988_OG76
19.2/37
Other_Amorphea-Tspp_10400_OG76
83.8/45
Other_Other-Gint_5331_OG76
90.3/85
Other_Other-Sspe_25880_OG76
96/72
Pp_Diaphoretickes-Cmer_147_OG76
Osmo_Teretosporea-Apar_5411_OG76
0/73
Osmo_Teretosporea-Awhi_13266_OG76
0/10
Osmo_Fungi-Pbla_5125_OG76
85.5/37
Osmo_Teretosporea-Sarc_3432_OG76
97/92
Osmo_Teretosporea-Cfra_8142_OG76
80.2/34
Other_Other-Egra_27393_OG76
87.5/93
Other_Amorphea-Mbal_13219_OG76
54.1/30
78.7/32
Cp_Diaphoretickes-Esil_1487_OG76
Cp_Diaphoretickes-Smin_23956_OG76
90.9/89
Cp_Diaphoretickes-Chsp_15056_OG76
0/19
53.6/54
Pp_Diaphoretickes-Cpar_18777_OG76

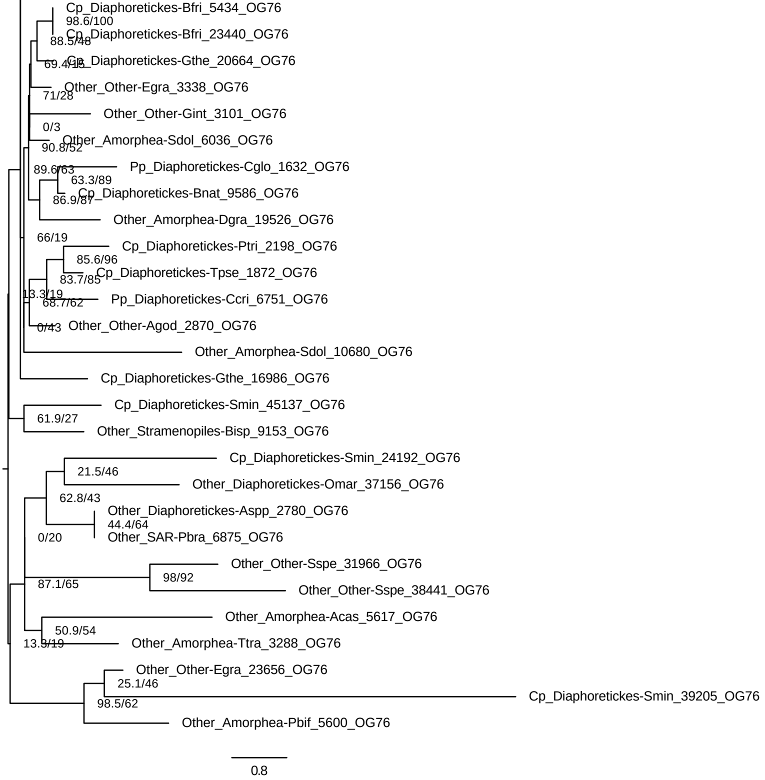
Cp_Diaphoretickes-Bfri_5434_OG76
98.6/100
88.5/48
Cp_Diaphoretickes-Bfri_23440_OG76
69.4/15
Cp_Diaphoretickes-Gthe_20664_OG76
71/28
Other_Other-Egra_3338_OG76
Other_Other-Gint_3101_OG76
0/3
Other_Amorphea-Sdol_6036_OG76
90.8/52
89.6/63
Pp_Diaphoretickes-Cglo_1632_OG76
63.3/89
86.9/87
Cp_Diaphoretickes-Bnat_9586_OG76
Other_Amorphea-Dgra_19526_OG76
66/19
Cp_Diaphoretickes-Ptri_2198_OG76
85.6/96
83.7/85
Cp_Diaphoretickes-Tpse_1872_OG76
13.3/19
68.7/62
Pp_Diaphoretickes-Ccri_6751_OG76
0/43
Other_Other-Agod_2870_OG76
Other_Amorphea-Sdol_10680_OG76
Cp_Diaphoretickes-Gthe_16986_OG76
Cp_Diaphoretickes-Smin_45137_OG76
61.9/27
Other_Stramenopiles-Bisp_9153_OG76
Cp_Diaphoretickes-Smin_24192_OG76
21.5/46
Other_Diaphoretickes-Omar_37156_OG76
62.8/43
Other_Diaphoretickes-Aspp_2780_OG76
44.4/64
0/20
Other_SAR-Pbra_6875_OG76
Other_Other-Sspe_31966_OG76
98/92
87.1/65
Other_Other-Sspe_38441_OG76
Other_Amorphea-Acas_5617_OG76
50.9/54
13.3/19
Other_Amorphea-Ttra_3288_OG76
Other_Other-Egra_23656_OG76
25.1/46
Cp_Diaphoretickes-Smin_39205_OG76
98.5/62
Other_Amorphea-Pbif_5600_OG76
0.8

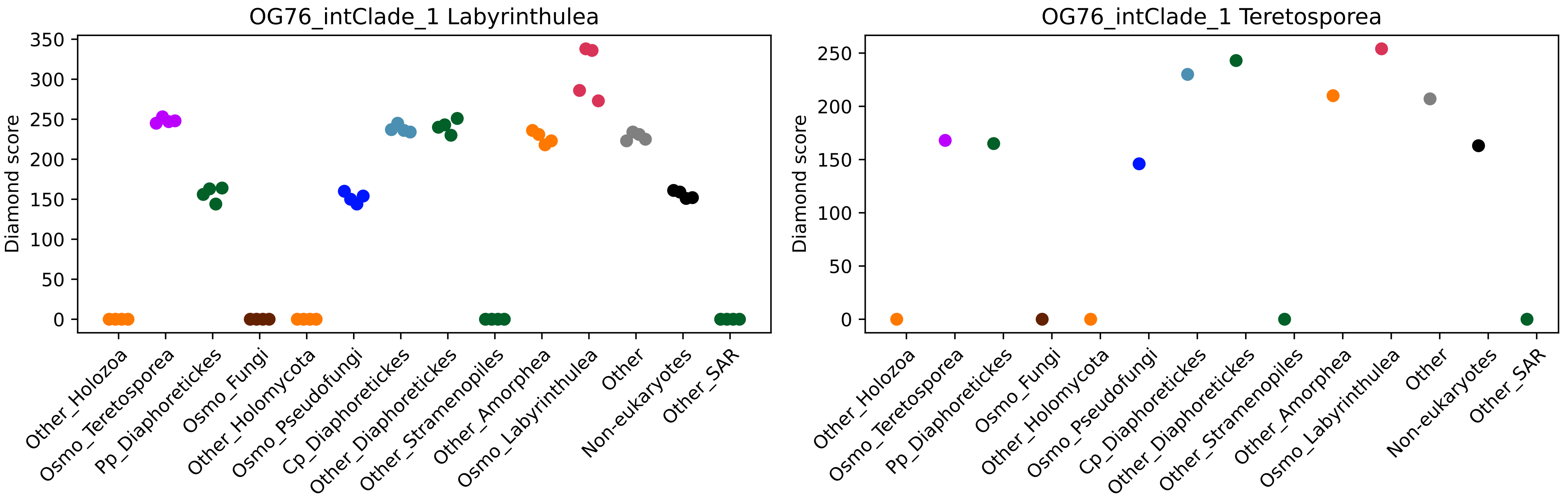
OG76_intClade_1 Labyrinthulea
OG76_intClade_1 Teretosporea
Diamond score
0
50
100
150
200
250
300
350
Other_Holozoa
Osmo_Teretosporea
Pp_Diaphoretickes
Osmo_Fungi
Other_Holomycota
Osmo_Pseudofungi
Cp_Diaphoretickes
Other_Diaphoretickes
Other_Stramenopiles
Other_Amorphea
Osmo_Labyrinthulea
Other
Non-eukaryotes
Other_SAR

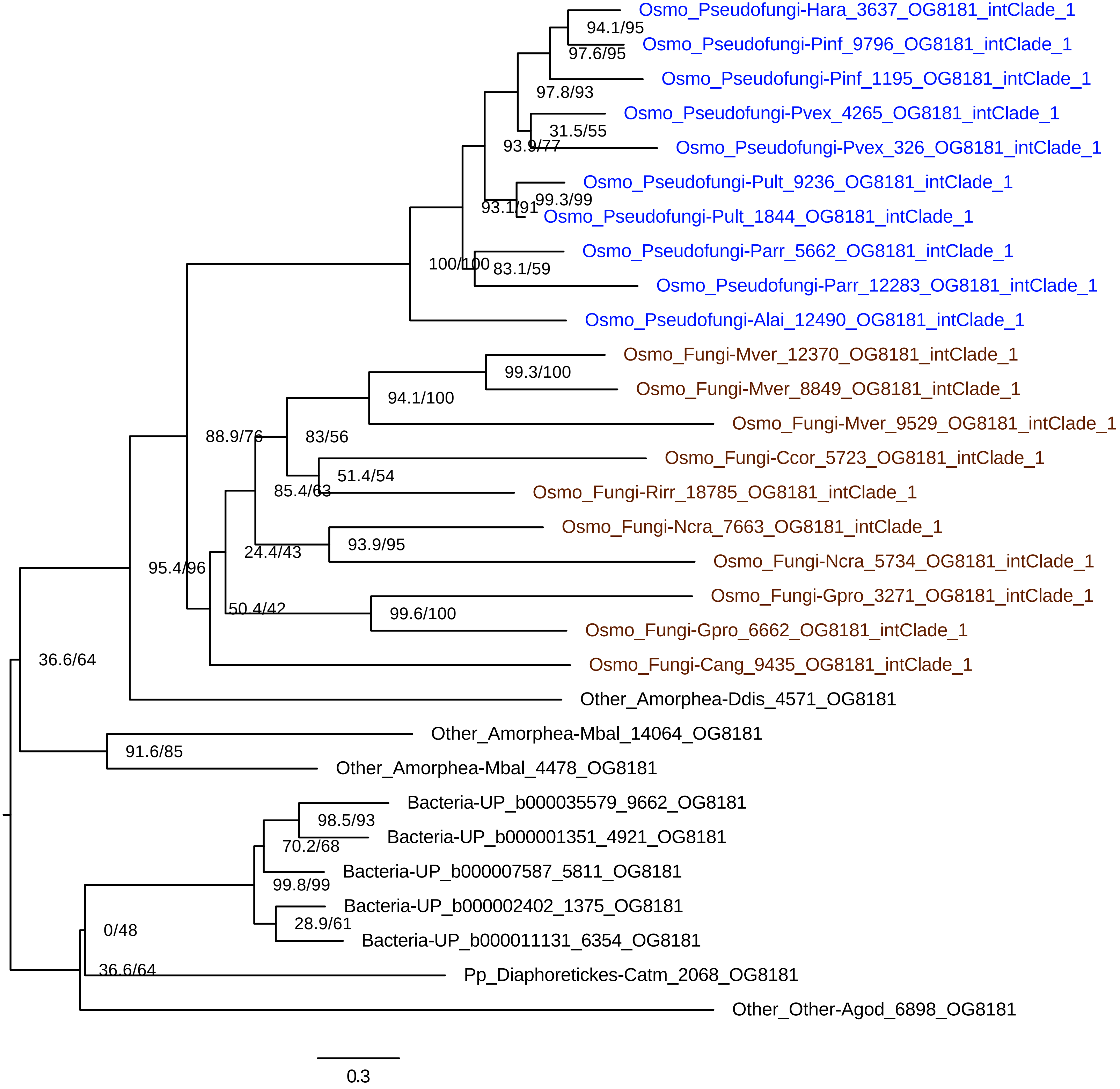
Osmo_Pseudofungi-Hara_3637_OG8181_intClade_1
94.1/95
Osmo_Pseudofungi-Pinf_9796_OG8181_intClade_1
97.6/95
Osmo_Pseudofungi-Pinf_1195_OG8181_intClade_1
97.8/93
Osmo_Pseudofungi-Pvex_4265_OG8181_intClade_1
31.5/55
93.9/77
Osmo_Pseudofungi-Pvex_326_OG8181_intClade_1
Osmo_Pseudofungi-Pult_9236_OG8181_intClade_1
99.3/99
93.1/91
Osmo_Pseudofungi-Pult_1844_OG8181_intClade_1
Osmo_Pseudofungi-Parr_5662_OG8181_intClade_1
100/100
83.1/59
Osmo_Pseudofungi-Parr_12283_OG8181_intClade_1
Osmo_Pseudofungi-Alai_12490_OG8181_intClade_1
Osmo_Fungi-Mver_12370_OG8181_intClade_1
99.3/100
Osmo_Fungi-Mver_8849_OG8181_intClade_1
94.1/100
Osmo_Fungi-Mver_9529_OG8181_intClade_1
88.9/76
83/56
Osmo_Fungi-Ccor_5723_OG8181_intClade_1
51.4/54
85.4/63
Osmo_Fungi-Rirr_18785_OG8181_intClade_1
Osmo_Fungi-Ncra_7663_OG8181_intClade_1
93.9/95
24.4/43
95.4/96
Osmo_Fungi-Ncra_5734_OG8181_intClade_1
Osmo_Fungi-Gpro_3271_OG8181_intClade_1
50.4/42
99.6/100
Osmo_Fungi-Gpro_6662_OG8181_intClade_1
36.6/64
Osmo_Fungi-Cang_9435_OG8181_intClade_1
Other_Amorphea-Ddis_4571_OG8181
Other_Amorphea-Mbal_14064_OG8181
91.6/85
Other_Amorphea-Mbal_4478_OG8181
Bacteria-UP_b000035579_9662_OG8181
98.5/93
Bacteria-UP_b000001351_4921_OG8181
70.2/68
Bacteria-UP_b000007587_5811_OG8181
99.8/99
Bacteria-UP_b000002402_1375_OG8181
28.9/61
0/48
Bacteria-UP_b000011131_6354_OG8181
36.6/64
Pp_Diaphoretickes-Catm_2068_OG8181
Other_Other-Agod_6898_OG8181
0.3

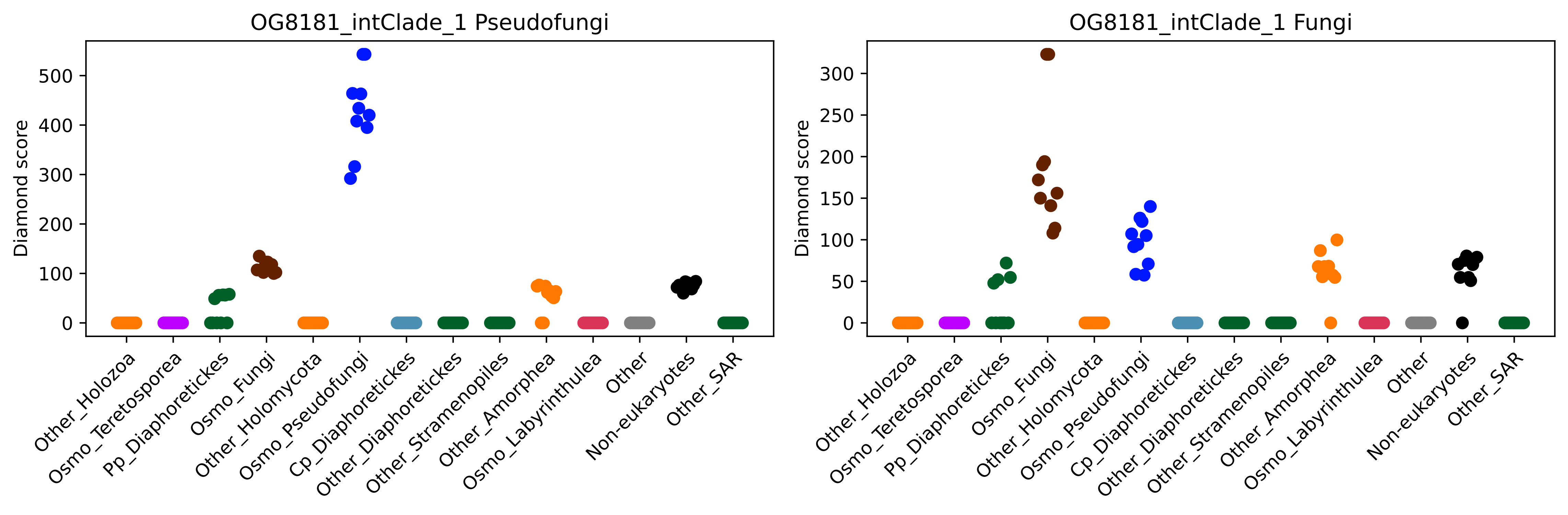
OG8181_intClade_1 Pseudofungi
OG8181_intClade_1 Fungi
Diamond score
0
100
200
300
400
500
50
150
250
Other_Holozoa
Osmo_Teretosporea
Pp_Diaphoretickes
Osmo_Fungi
Other_Holomycota
Osmo_Pseudofungi
Cp_Diaphoretickes
Other_Diaphoretickes
Other_Stramenopiles
Other_Amorphea
Osmo_Labyrinthulea
Other
Non-eukaryotes
Other_SAR

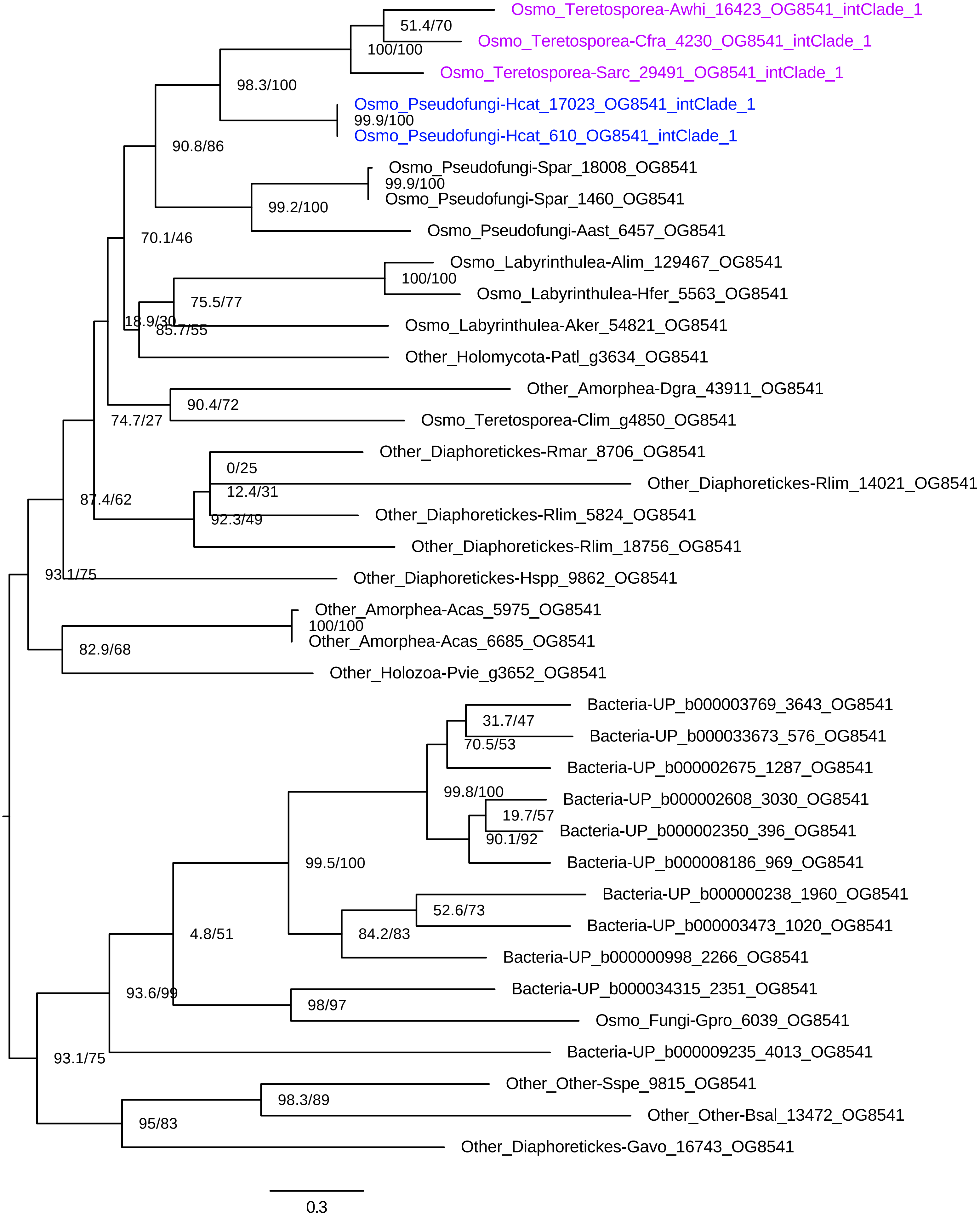
Osmo_Teretosporea-Awhi_16423_OG8541_intClade_1
51.4/70
Osmo_Teretosporea-Cfra_4230_OG8541_intClade_1
100/100
Osmo_Teretosporea-Sarc_29491_OG8541_intClade_1
98.3/100
Osmo_Pseudofungi-Hcat_17023_OG8541_intClade_1
99.9/100
Osmo_Pseudofungi-Hcat_610_OG8541_intClade_1
90.8/86
Osmo_Pseudofungi-Spar_18008_OG8541
99.9/100
Osmo_Pseudofungi-Spar_1460_OG8541
99.2/100
Osmo_Pseudofungi-Aast_6457_OG8541
70.1/46
Osmo_Labyrinthulea-Alim_129467_OG8541
100/100
Osmo_Labyrinthulea-Hfer_5563_OG8541
75.5/77
18.9/30
85.7/55
Osmo_Labyrinthulea-Aker_54821_OG8541
Other_Holomycota-Patl_g3634_OG8541
Other_Amorphea-Dgra_43911_OG8541
90.4/72
74.7/27
Osmo_Teretosporea-Clim_g4850_OG8541
Other_Diaphoretickes-Rmar_8706_OG8541
0/25
Other_Diaphoretickes-Rlim_14021_OG8541
12.4/31
87.4/62
Other_Diaphoretickes-Rlim_5824_OG8541
92.3/49
Other_Diaphoretickes-Rlim_18756_OG8541
93.1/75
Other_Diaphoretickes-Hspp_9862_OG8541
Other_Amorphea-Acas_5975_OG8541
100/100
Other_Amorphea-Acas_6685_OG8541
82.9/68
Other_Holozoa-Pvie_g3652_OG8541
Bacteria-UP_b000003769_3643_OG8541
31.7/47
Bacteria-UP_b000033673_576_OG8541
70.5/53
Bacteria-UP_b000002675_1287_OG8541
99.8/100
Bacteria-UP_b000002608_3030_OG8541
19.7/57
Bacteria-UP_b000002350_396_OG8541
90.1/92
99.5/100
Bacteria-UP_b000008186_969_OG8541
Bacteria-UP_b000000238_1960_OG8541
52.6/73
Bacteria-UP_b000003473_1020_OG8541
4.8/51
84.2/83
Bacteria-UP_b000000998_2266_OG8541
Bacteria-UP_b000034315_2351_OG8541
93.6/99
98/97
Osmo_Fungi-Gpro_6039_OG8541
Bacteria-UP_b000009235_4013_OG8541
93.1/75
Other_Other-Sspe_9815_OG8541
98.3/89
Other_Other-Bsal_13472_OG8541
95/83
Other_Diaphoretickes-Gavo_16743_OG8541
0.3

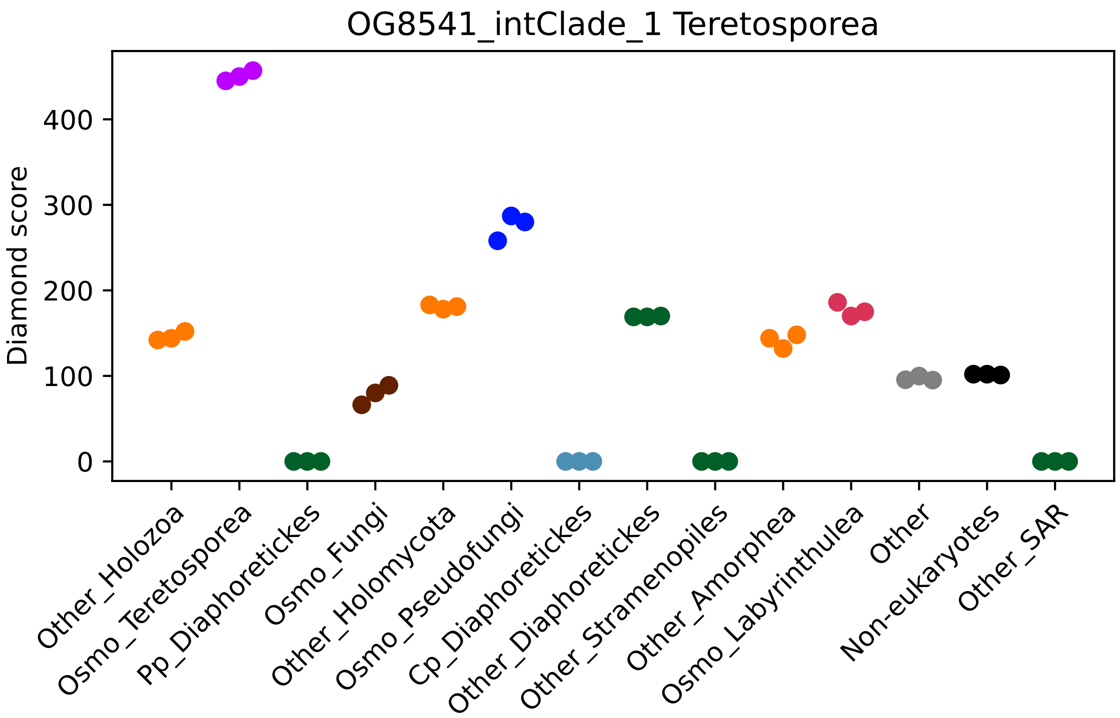
OG8541_intClade_1 Teretosporea
Diamond score
0
100
200
300
400
Other_Holozoa
Osmo_Teretosporea
Pp_Diaphoretickes
Osmo_Fungi
Other_Holomycota
Osmo_Pseudofungi
Cp_Diaphoretickes
Other_Diaphoretickes
Other_Stramenopiles
Other_Amorphea
Osmo_Labyrinthulea
Other
Non-eukaryotes
Other_SAR

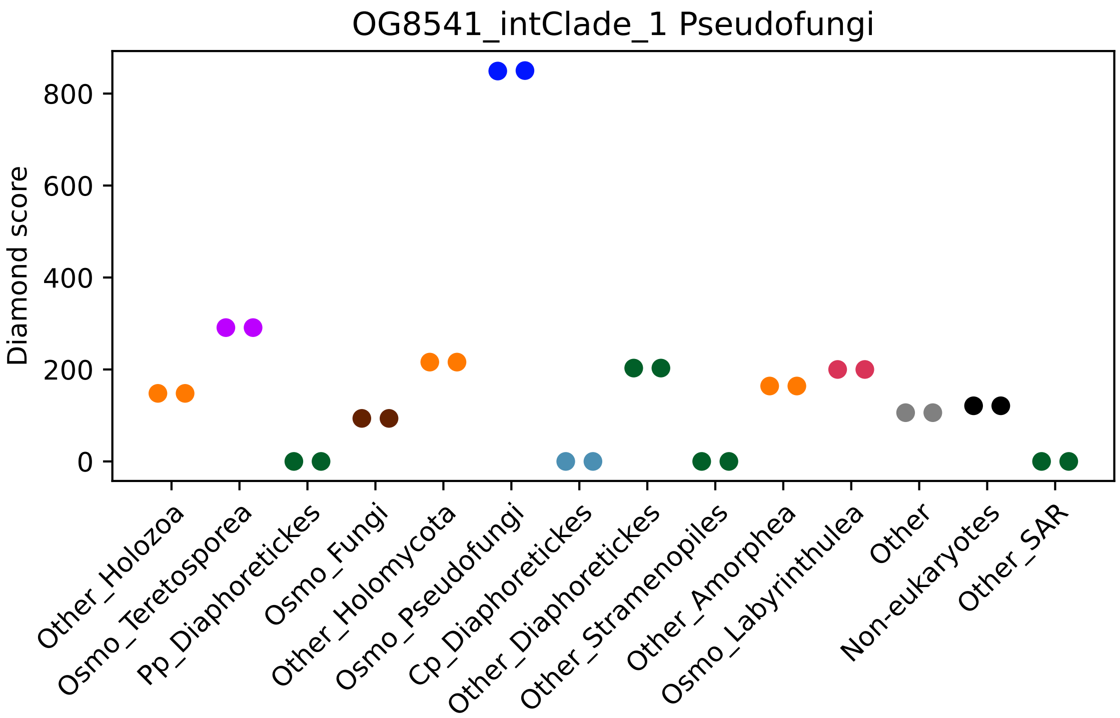
OG8541_intClade_1 Pseudofungi
Diamond score
0
200
400
600
800
Other_Holozoa
Osmo_Teretosporea
Pp_Diaphoretickes
Osmo_Fungi
Other_Holomycota
Osmo_Pseudofungi
Cp_Diaphoretickes
Other_Diaphoretickes
Other_Stramenopiles
Other_Amorphea
Osmo_Labyrinthulea
Other
Non-eukaryotes
Other_SAR

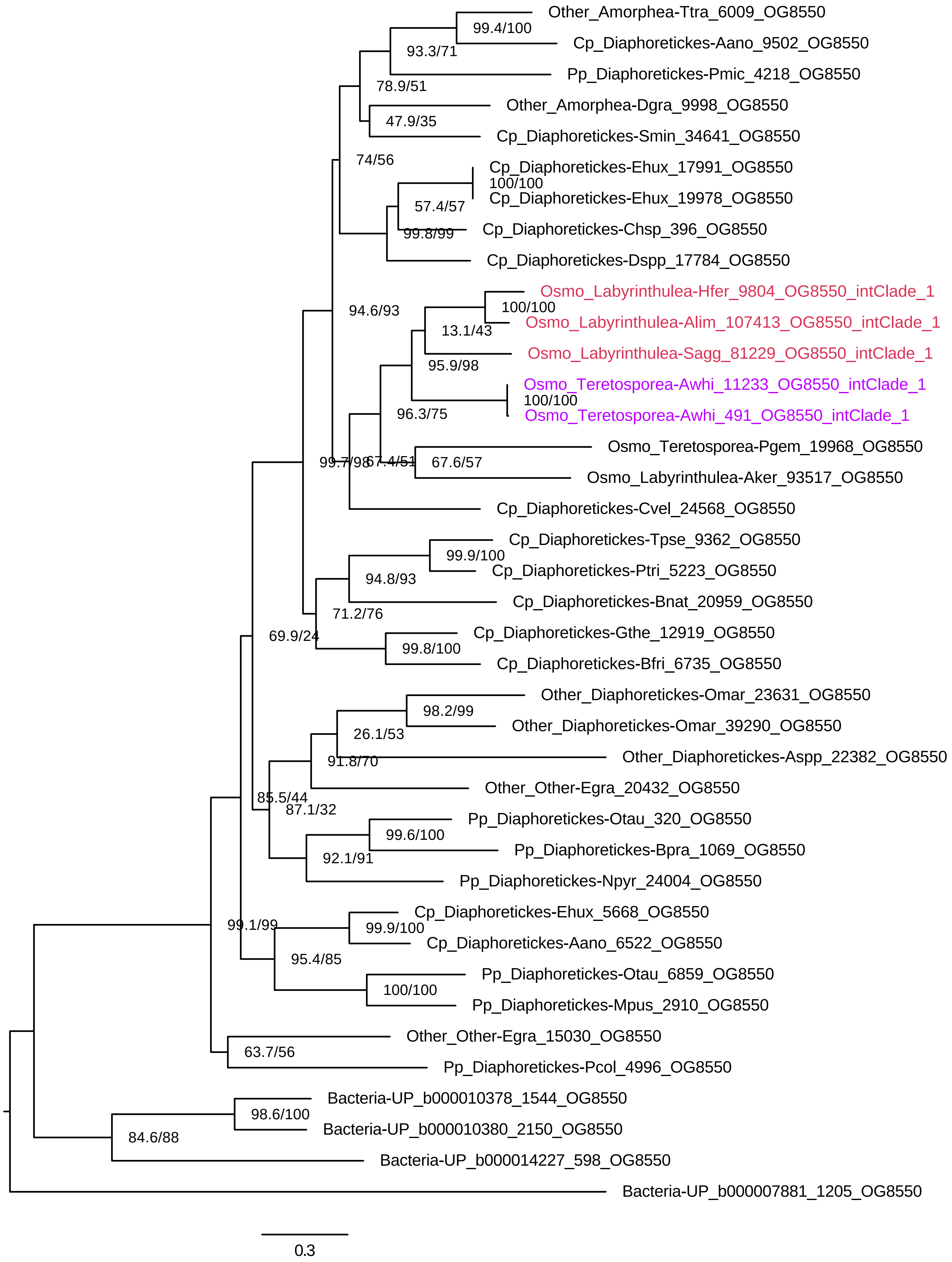

Other_Amorphea-Ttra_6009_OG8550
99.4/100
Cp_Diaphoretickes-Aano_9502_OG8550
93.3/71
Pp_Diaphoretickes-Pmic_4218_OG8550
78.9/51
Other_Amorphea-Dgra_9998_OG8550
47.9/35
Cp_Diaphoretickes-Smin_34641_OG8550
74/56
Cp_Diaphoretickes-Ehux_17991_OG8550
100/100
Cp_Diaphoretickes-Ehux_19978_OG8550
57.4/57
Cp_Diaphoretickes-Chsp_396_OG8550
99.8/99
Cp_Diaphoretickes-Dspp_17784_OG8550
Osmo_Labyrinthulea-Hfer_9804_OG8550_intClade_1
94.6/93
100/100
Osmo_Labyrinthulea-Alim_107413_OG8550_intClade_1
13.1/43
Osmo_Labyrinthulea-Sagg_81229_OG8550_intClade_1
95.9/98
Osmo_Teretosporea-Awhi_11233_OG8550_intClade_1
100/100
Osmo_Teretosporea-Awhi_491_OG8550_intClade_1
96.3/75
Osmo_Teretosporea-Pgem_19968_OG8550
99.7/98
67.4/51
67.6/57
Osmo_Labyrinthulea-Aker_93517_OG8550
Cp_Diaphoretickes-Cvel_24568_OG8550
Cp_Diaphoretickes-Tpse_9362_OG8550
99.9/100
Cp_Diaphoretickes-Ptri_5223_OG8550
94.8/93
Cp_Diaphoretickes-Bnat_20959_OG8550
71.2/76
Cp_Diaphoretickes-Gthe_12919_OG8550
69.9/24
99.8/100
Cp_Diaphoretickes-Bfri_6735_OG8550
Other_Diaphoretickes-Omar_23631_OG8550
98.2/99
Other_Diaphoretickes-Omar_39290_OG8550
26.1/53
Other_Diaphoretickes-Aspp_22382_OG8550
91.8/70
Other_Other-Egra_20432_OG8550
85.5/44
87.1/32
Pp_Diaphoretickes-Otau_320_OG8550
99.6/100
Pp_Diaphoretickes-Bpra_1069_OG8550
92.1/91
Pp_Diaphoretickes-Npyr_24004_OG8550
Cp_Diaphoretickes-Ehux_5668_OG8550
99.1/99
99.9/100
Cp_Diaphoretickes-Aano_6522_OG8550
95.4/85
Pp_Diaphoretickes-Otau_6859_OG8550
100/100
Pp_Diaphoretickes-Mpus_2910_OG8550
Other_Other-Egra_15030_OG8550
63.7/56
Pp_Diaphoretickes-Pcol_4996_OG8550
Bacteria-UP_b000010378_1544_OG8550
98.6/100
Bacteria-UP_b000010380_2150_OG8550
84.6/88
Bacteria-UP_b000014227_598_OG8550
Bacteria-UP_b000007881_1205_OG8550
0.3

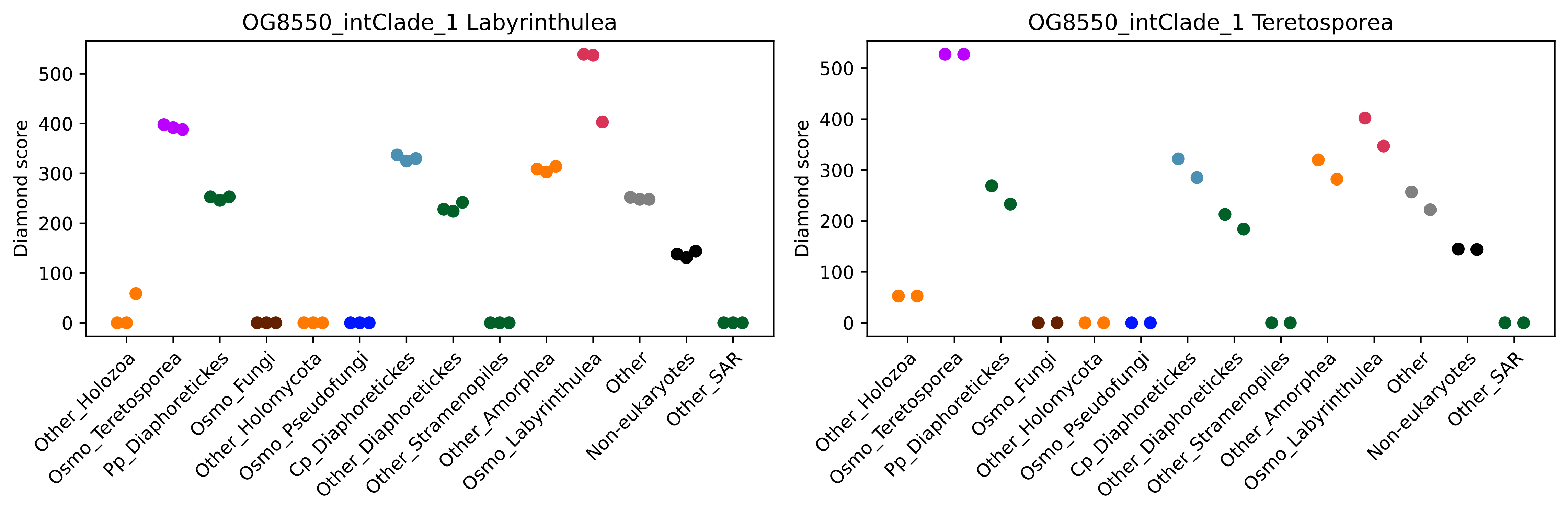

OG8550_intClade_1 Labyrinthulea
OG8550_intClade_1 Teretosporea
Diamond score
0
100
200
300
400
500
Other_Holozoa
Osmo_Teretosporea
Pp_Diaphoretickes
Osmo_Fungi
Other_Holomycota
Osmo_Pseudofungi
Cp_Diaphoretickes
Other_Diaphoretickes
Other_Stramenopiles
Other_Amorphea
Osmo_Labyrinthulea
Other
Non-eukaryotes
Other_SAR

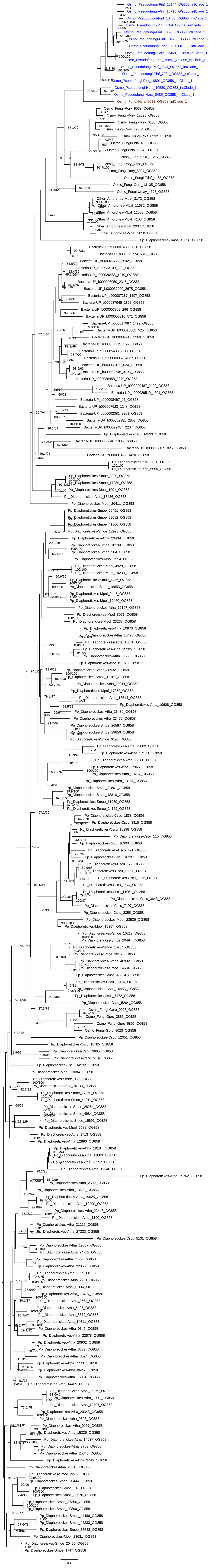

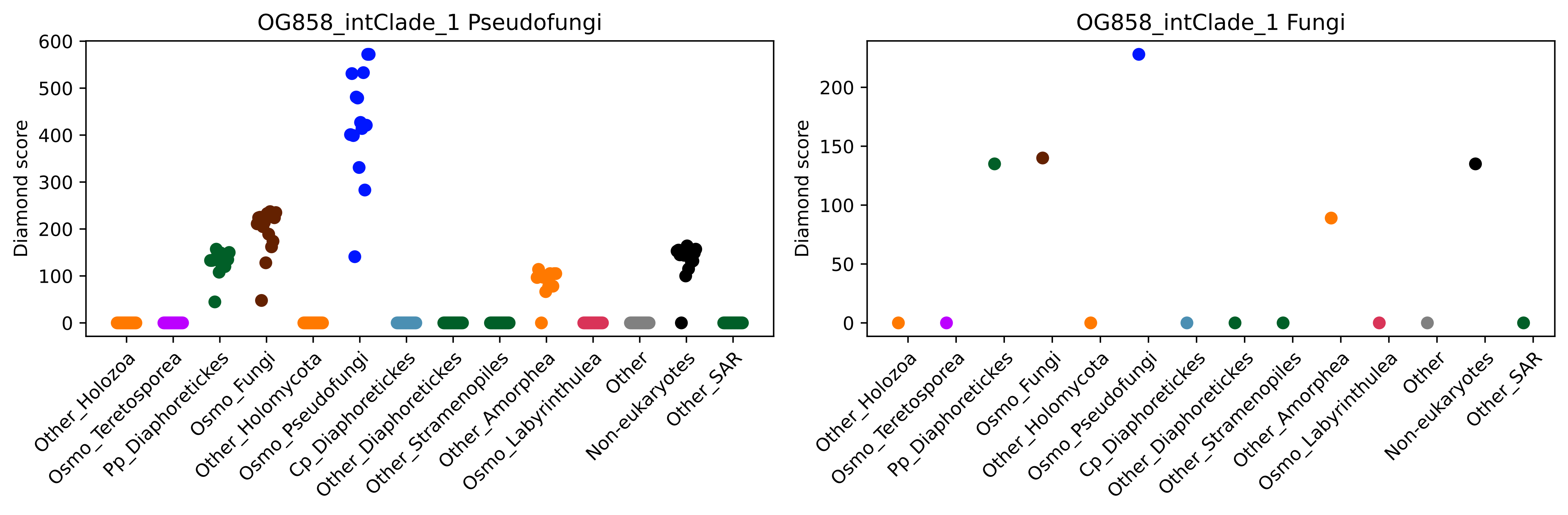

OG858_intClade_1 Pseudofungi
Diamond score
0
100
200
300
400
500
600
Other_Holozoa
Osmo_Teretosporea
Pp_Diaphoretickes
Osmo_Fungi
Other_Holomycota
Osmo_Pseudofungi
Cp_Diaphoretickes
Other_Diaphoretickes
Other_Stramenopiles
Other_Amorphea
Osmo_Labyrinthulea
Other
Non-eukaryotes
Other_SAR
OG858_intClade_1 Fungi
Diamond score
0
50
100
150
200
Other_Holozoa
Osmo_Teretosporea
Pp_Diaphoretickes
Osmo_Fungi
Other_Holomycota
Osmo_Pseudofungi
Cp_Diaphoretickes
Other_Diaphoretickes
Other_Stramenopiles
Other_Amorphea
Osmo_Labyrinthulea
Other
Non-eukaryotes
Other_SAR

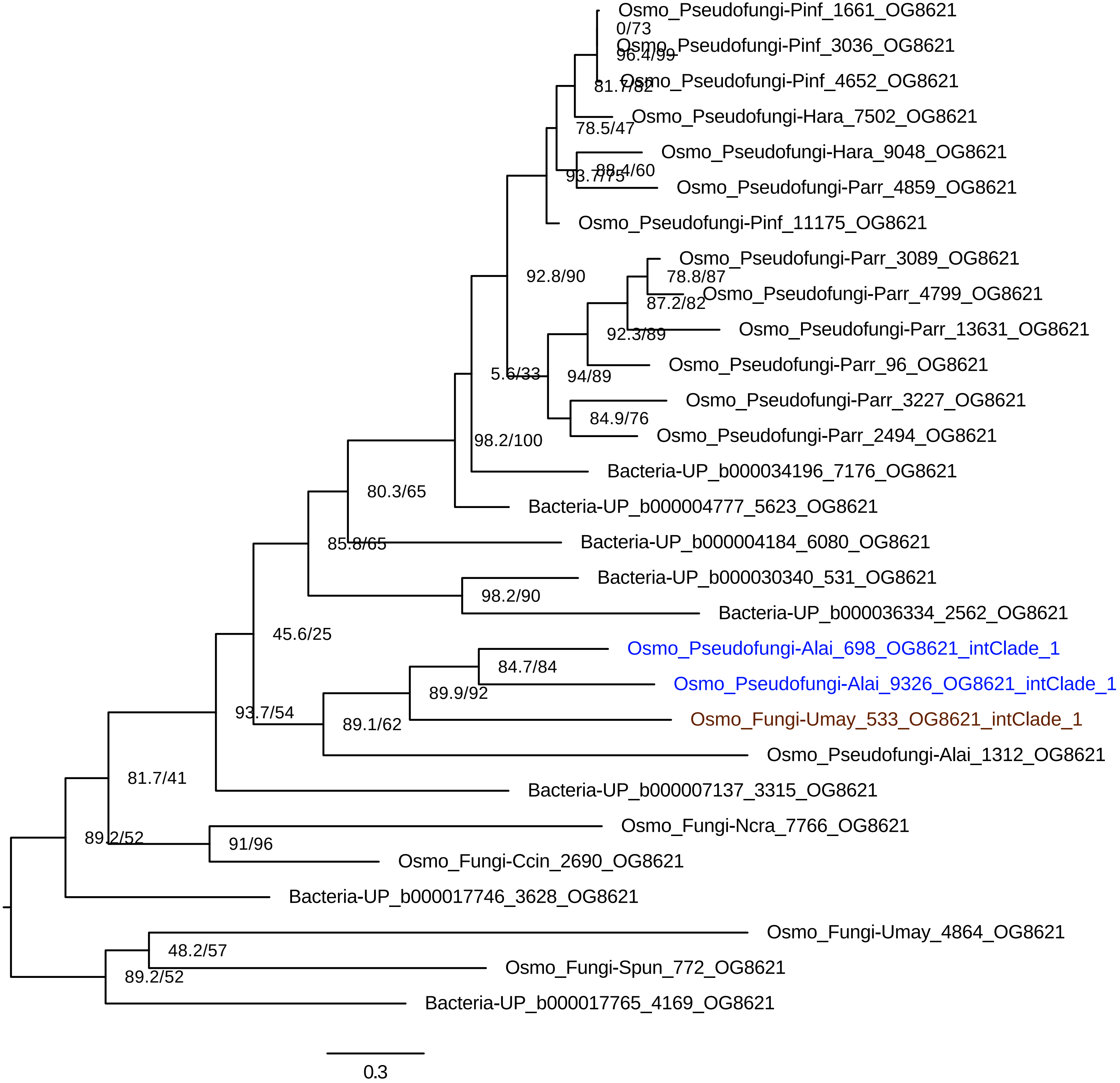

Osmo_Pseudofungi-Pinf_1661_OG8621
0/73
Osmo_Pseudofungi-Pinf_3036_OG8621
96.4/99
Osmo_Pseudofungi-Pinf_4652_OG8621
81.7/82
Osmo_Pseudofungi-Hara_7502_OG8621
78.5/47
Osmo_Pseudofungi-Hara_9048_OG8621
88.4/60
93.7/75
Osmo_Pseudofungi-Parr_4859_OG8621
Osmo_Pseudofungi-Pinf_11175_OG8621
Osmo_Pseudofungi-Parr_3089_OG8621
92.8/90
78.8/87
Osmo_Pseudofungi-Parr_4799_OG8621
87.2/82
Osmo_Pseudofungi-Parr_13631_OG8621
92.3/89
Osmo_Pseudofungi-Parr_96_OG8621
5.6/33
94/89
Osmo_Pseudofungi-Parr_3227_OG8621
84.9/76
Osmo_Pseudofungi-Parr_2494_OG8621
98.2/100
Bacteria-UP_b000034196_7176_OG8621
80.3/65
Bacteria-UP_b000004777_5623_OG8621
85.8/65
Bacteria-UP_b000004184_6080_OG8621
Bacteria-UP_b000030340_531_OG8621
98.2/90
Bacteria-UP_b000036334_2562_OG8621
45.6/25
Osmo_Pseudofungi-Alai_698_OG8621_intClade_1
84.7/84
Osmo_Pseudofungi-Alai_9326_OG8621_intClade_1
89.9/92
93.7/54
Osmo_Fungi-Umay_533_OG8621_intClade_1
89.1/62
Osmo_Pseudofungi-Alai_1312_OG8621
81.7/41
Bacteria-UP_b000007137_3315_OG8621
Osmo_Fungi-Ncra_7766_OG8621
89.2/52
91/96
Osmo_Fungi-Ccin_2690_OG8621
Bacteria-UP_b000017746_3628_OG8621
Osmo_Fungi-Umay_4864_OG8621
48.2/57
Osmo_Fungi-Spun_772_OG8621
89.2/52
Bacteria-UP_b000017765_4169_OG8621
0.3

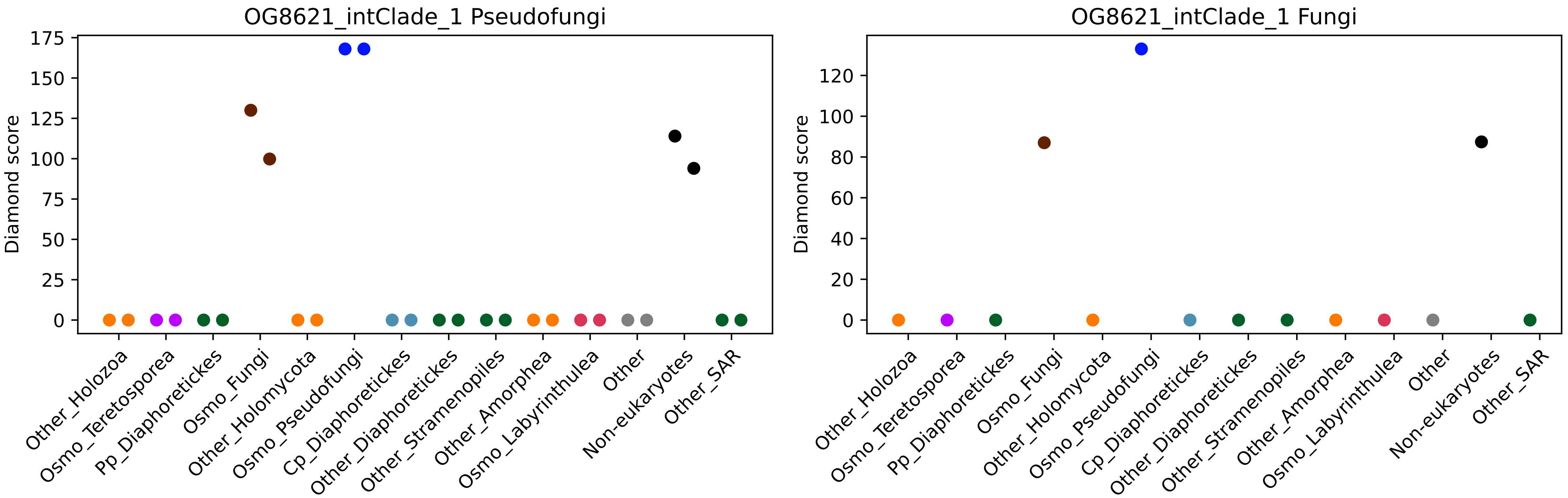
OG8621_intClade_1 Pseudofungi
Diamond score
175
150
125
100
75
50
25
0
Other_Holozoa
Osmo_Teretosporea
Pp_Diaphoretickes
Osmo_Fungi
Other_Holomycota
Osmo_Pseudofungi
Cp_Diaphoretickes
Other_Diaphoretickes
Other_Stramenopiles
Other_Amorphea
Osmo_Labyrinthulea
Other
Non-eukaryotes
Other_SAR
OG8621_intClade_1 Fungi
Diamond score
120
100
80
60
40
20
0
Other_Holozoa
Osmo_Teretosporea
Pp_Diaphoretickes
Osmo_Fungi
Other_Holomycota
Osmo_Pseudofungi
Cp_Diaphoretickes
Other_Diaphoretickes
Other_Stramenopiles
Other_Amorphea
Osmo_Labyrinthulea
Other
Non-eukaryotes
Other_SAR

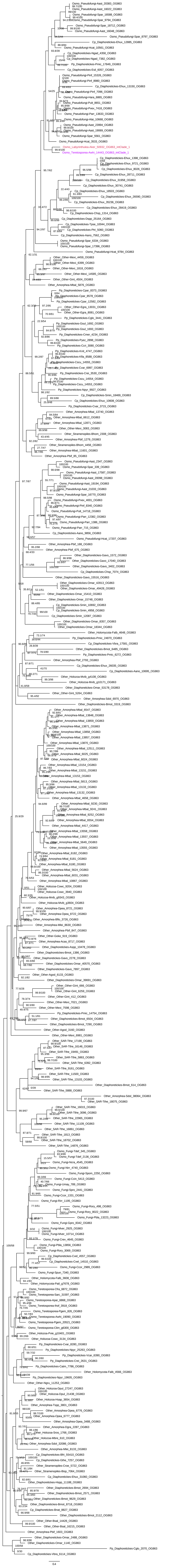

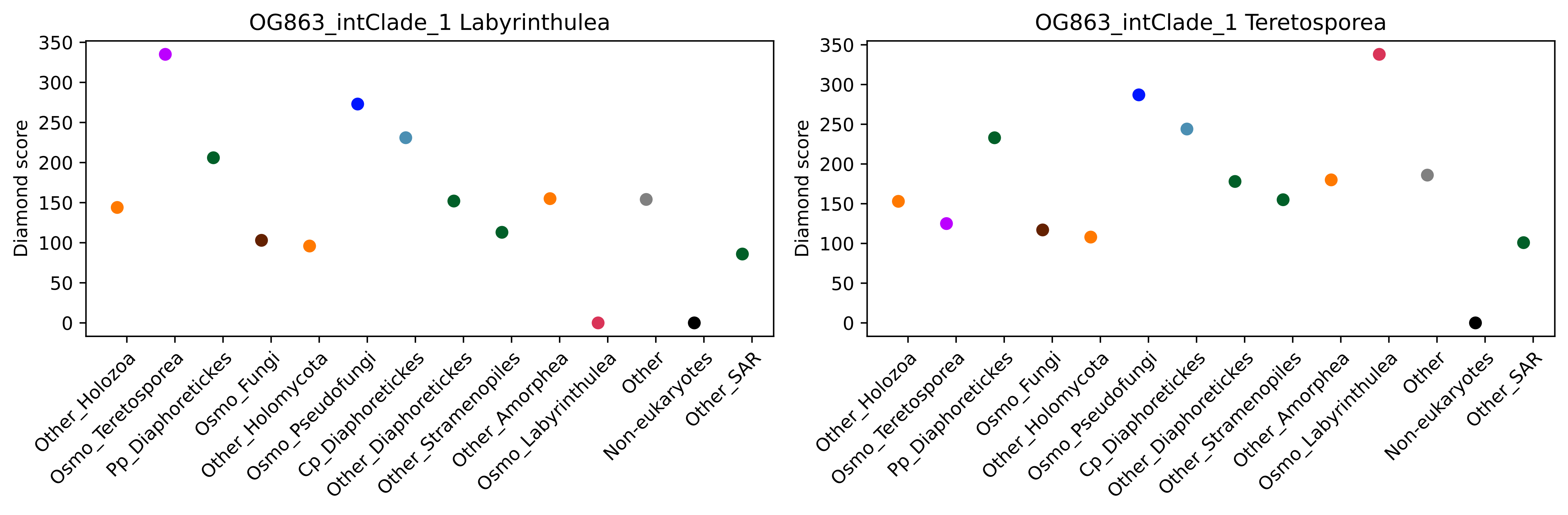

OG863_intClade_1 Labyrinthulea
OG863_intClade_1 Teretosporea
Diamond score
0
50
100
150
200
250
300
350
Other_Holozoa
Osmo_Teretosporea
Pp_Diaphoretickes
Osmo_Fungi
Other_Holomycota
Osmo_Pseudofungi
Cp_Diaphoretickes
Other_Diaphoretickes
Other_Stramenopiles
Other_Amorphea
Osmo_Labyrinthulea
Other
Non-eukaryotes
Other_SAR

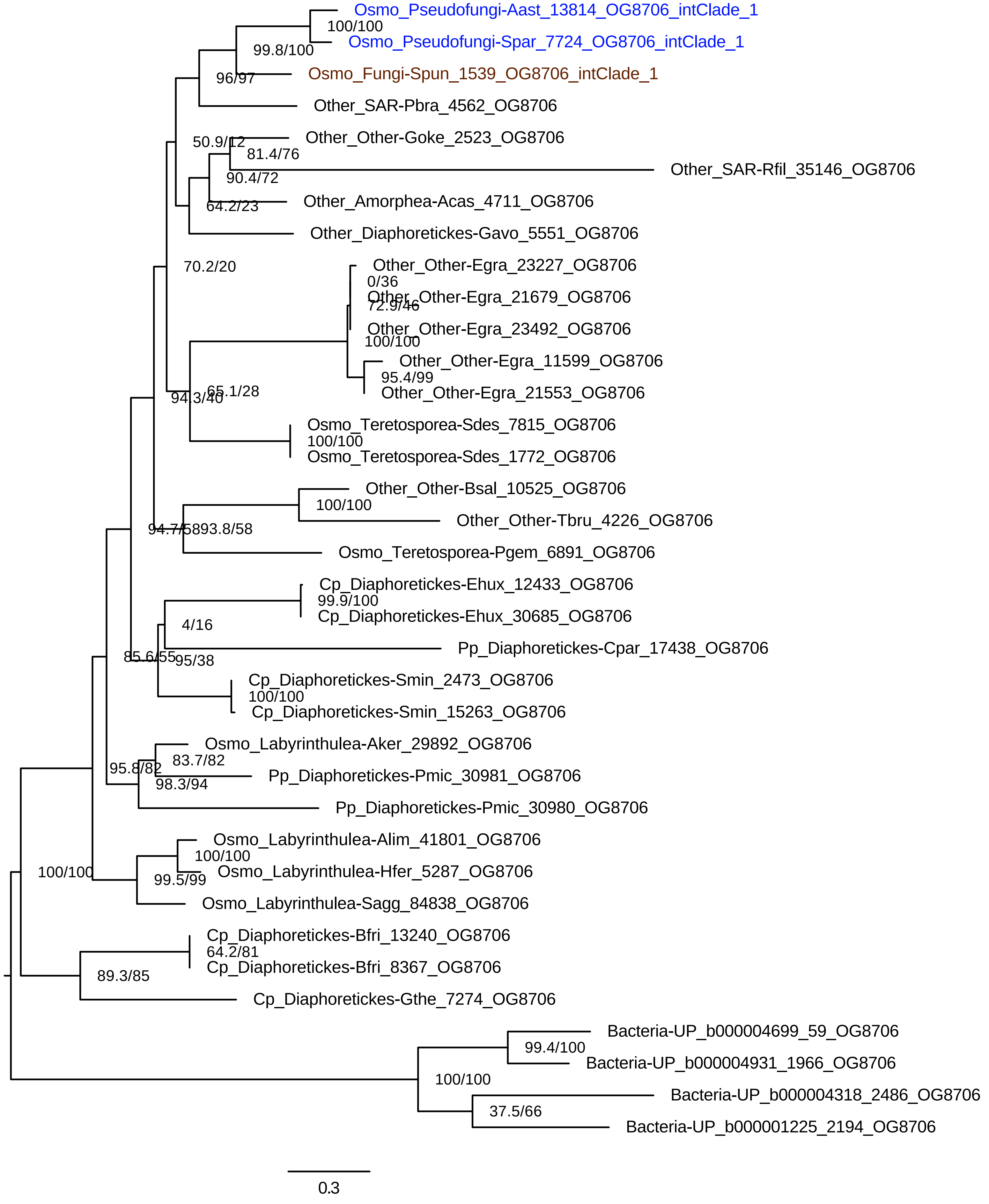

Osmo_Pseudofungi-Aast_13814_OG8706_intClade_1
100/100
Osmo_Pseudofungi-Spar_7724_OG8706_intClade_1
99.8/100
Osmo_Fungi-Spun_1539_OG8706_intClade_1
96/97
Other_SAR-Pbra_4562_OG8706
50.9/12
Other_Other-Goke_2523_OG8706
81.4/76
Other_SAR-Rfil_35146_OG8706
90.4/72
Other_Amorphea-Acas_4711_OG8706
64.2/23
Other_Diaphoretickes-Gavo_5551_OG8706
70.2/20
Other_Other-Egra_23227_OG8706
0/36
Other_Other-Egra_21679_OG8706
72.9/46
Other_Other-Egra_23492_OG8706
100/100
Other_Other-Egra_11599_OG8706
95.4/99
Other_Other-Egra_21553_OG8706
65.1/28
94.3/40
Osmo_Teretosporea-Sdes_7815_OG8706
100/100
Osmo_Teretosporea-Sdes_1772_OG8706
Other_Other-Bsal_10525_OG8706
100/100
Other_Other-Tbru_4226_OG8706
94.7/58
93.8/58
Osmo_Teretosporea-Pgem_6891_OG8706
Cp_Diaphoretickes-Ehux_12433_OG8706
99.9/100
Cp_Diaphoretickes-Ehux_30685_OG8706
4/16
Pp_Diaphoretickes-Cpar_17438_OG8706
85.6/55
95/38
Cp_Diaphoretickes-Smin_2473_OG8706
100/100
Cp_Diaphoretickes-Smin_15263_OG8706
Osmo_Labyrinthulea-Aker_29892_OG8706
83.7/82
95.8/82
Pp_Diaphoretickes-Pmic_30981_OG8706
98.3/94
Pp_Diaphoretickes-Pmic_30980_OG8706
Osmo_Labyrinthulea-Alim_41801_OG8706
100/100
100/100
Osmo_Labyrinthulea-Hfer_5287_OG8706
99.5/99
Osmo_Labyrinthulea-Sagg_84838_OG8706
Cp_Diaphoretickes-Bfri_13240_OG8706
64.2/81
Cp_Diaphoretickes-Bfri_8367_OG8706
89.3/85
Cp_Diaphoretickes-Gthe_7274_OG8706
Bacteria-UP_b000004699_59_OG8706
99.4/100
Bacteria-UP_b000004931_1966_OG8706
100/100
Bacteria-UP_b000004318_2486_OG8706
37.5/66
Bacteria-UP_b000001225_2194_OG8706
0.3

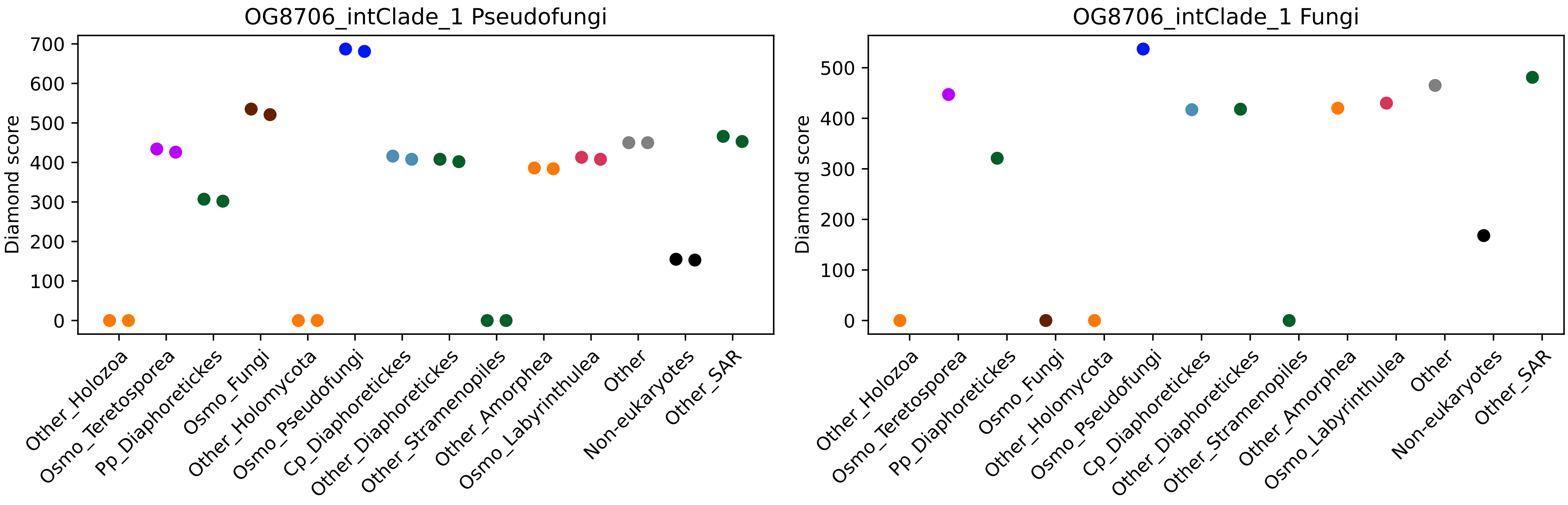
OG8706_intClade_1 Pseudofungi
OG8706_intClade_1 Fungi
Diamond score
0
100
200
300
400
500
600
700
Other_Holozoa
Osmo_Teretosporea
Pp_Diaphoretickes
Osmo_Fungi
Other_Holomycota
Osmo_Pseudofungi
Cp_Diaphoretickes
Other_Diaphoretickes
Other_Stramenopiles
Other_Amorphea
Osmo_Labyrinthulea
Other
Non-eukaryotes
Other_SAR

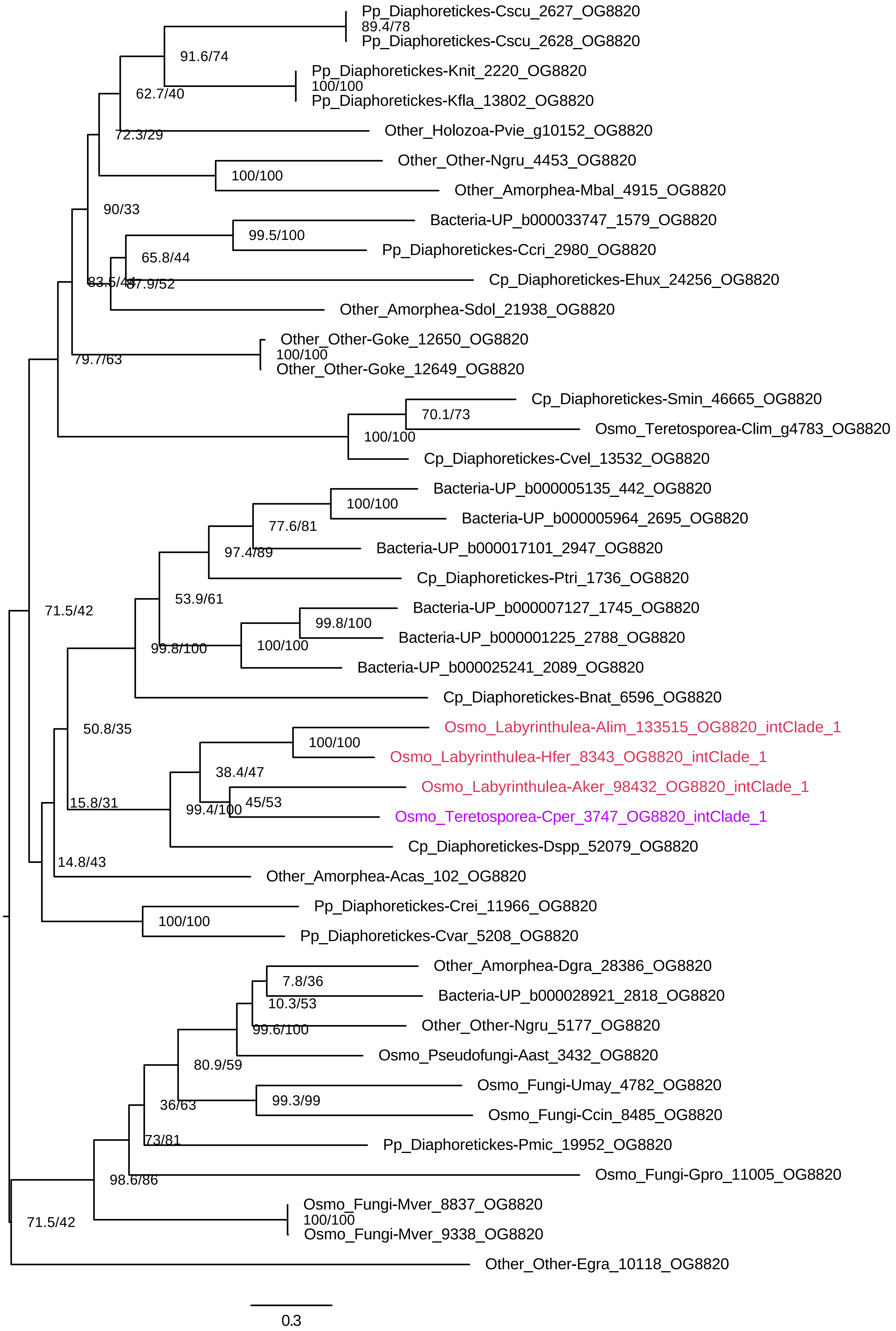
Pp_Diaphoretickes-Cscu_2627_OG8820
89.4/78
Pp_Diaphoretickes-Cscu_2628_OG8820
91.6/74
Pp_Diaphoretickes-Knit_2220_OG8820
100/100
Pp_Diaphoretickes-Kfla_13802_OG8820
62.7/40
Other_Holozoa-Pvie_g10152_OG8820
72.3/29
Other_Other-Ngru_4453_OG8820
100/100
Other_Amorphea-Mbal_4915_OG8820
90/33
Bacteria-UP_b000033747_1579_OG8820
99.5/100
Pp_Diaphoretickes-Ccri_2980_OG8820
65.8/44
Cp_Diaphoretickes-Ehux_24256_OG8820
83.5/44
87.9/52
Other_Amorphea-Sdol_21938_OG8820
Other_Other-Goke_12650_OG8820
100/100
Other_Other-Goke_12649_OG8820
79.7/63
Cp_Diaphoretickes-Smin_46665_OG8820
70.1/73
Osmo_Teretosporea-Clim_g4783_OG8820
100/100
Cp_Diaphoretickes-Cvel_13532_OG8820
Bacteria-UP_b000005135_442_OG8820
100/100
Bacteria-UP_b000005964_2695_OG8820
77.6/81
Bacteria-UP_b000017101_2947_OG8820
97.4/89
Cp_Diaphoretickes-Ptri_1736_OG8820
53.9/61
Bacteria-UP_b000007127_1745_OG8820
99.8/100
Bacteria-UP_b000001225_2788_OG8820
71.5/42
99.8/100
100/100
Bacteria-UP_b000025241_2089_OG8820
Cp_Diaphoretickes-Bnat_6596_OG8820
50.8/35
Osmo_Labyrinthulea-Alim_133515_OG8820_intClade_1
100/100
Osmo_Labyrinthulea-Hfer_8343_OG8820_intClade_1
38.4/47
Osmo_Labyrinthulea-Aker_98432_OG8820_intClade_1
15.8/31
45/53
99.4/100
Osmo_Teretosporea-Cper_3747_OG8820_intClade_1
Cp_Diaphoretickes-Dspp_52079_OG8820
14.8/43
Other_Amorphea-Acas_102_OG8820
Pp_Diaphoretickes-Crei_11966_OG8820
100/100
Pp_Diaphoretickes-Cvar_5208_OG8820
Other_Amorphea-Dgra_28386_OG8820
7.8/36
Bacteria-UP_b000028921_2818_OG8820
10.3/53
Other_Other-Ngru_5177_OG8820
99.6/100
Osmo_Pseudofungi-Aast_3432_OG8820
80.9/59
Osmo_Fungi-Umay_4782_OG8820
99.3/99
36/63
Osmo_Fungi-Ccin_8485_OG8820
73/81
Pp_Diaphoretickes-Pmic_19952_OG8820
98.6/86
Osmo_Fungi-Gpro_11005_OG8820
Osmo_Fungi-Mver_8837_OG8820
71.5/42
100/100
Osmo_Fungi-Mver_9338_OG8820
Other_Other-Egra_10118_OG8820
0.3

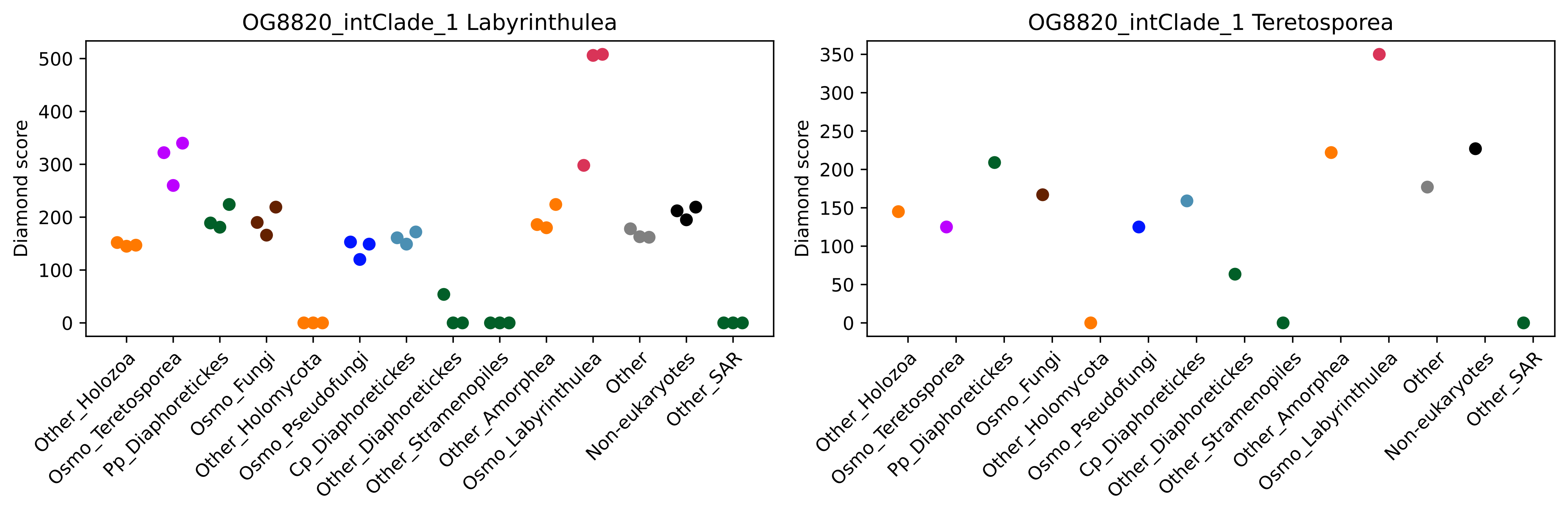

OG8820_intClade_1 Labyrinthulea
OG8820_intClade_1 Teretosporea
Diamond score
0
100
200
300
400
500
0
50
100
150
200
250
300
350
Other_Holozoa
Osmo_Teretosporea
Pp_Diaphoretickes
Osmo_Fungi
Other_Holomycota
Osmo_Pseudofungi
Cp_Diaphoretickes
Other_Diaphoretickes
Other_Stramenopiles
Other_Amorphea
Osmo_Labyrinthulea
Other
Non-eukaryotes
Other_SAR

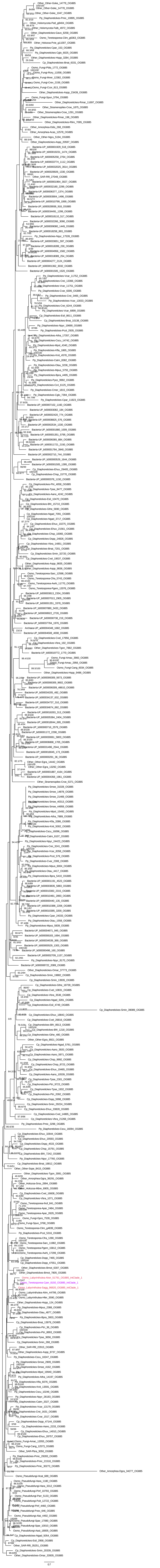

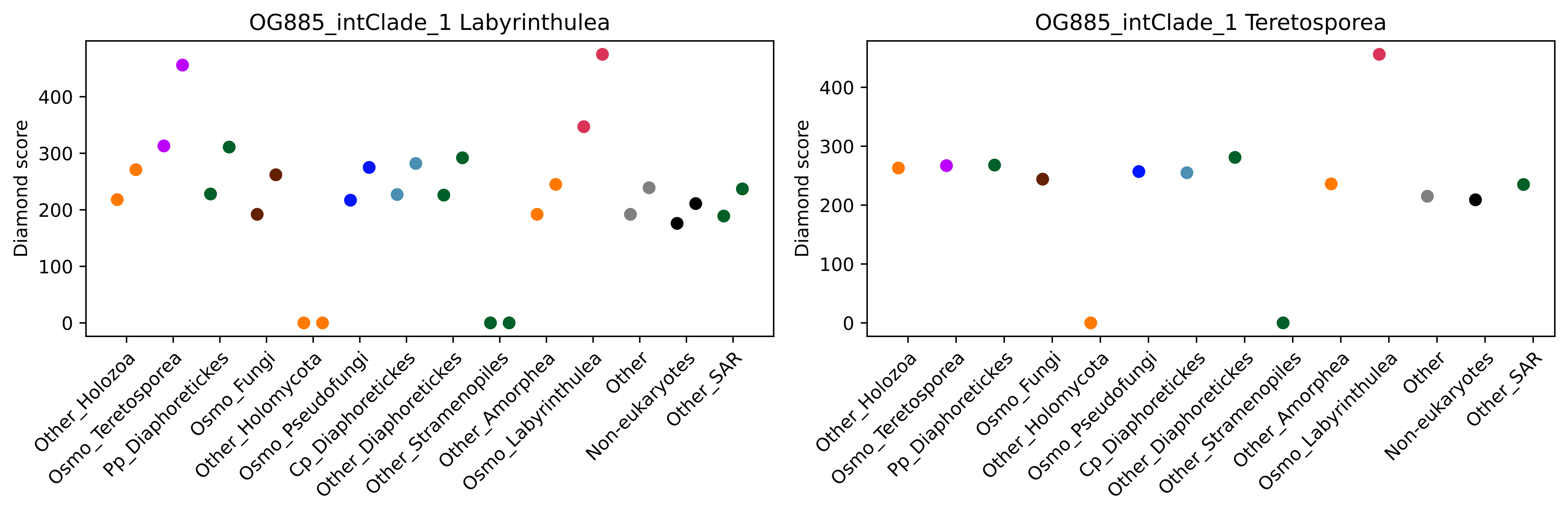

OG885_intClade_1 Labyrinthulea
OG885_intClade_1 Teretosporea
Diamond score
0
100
200
300
400
Other_Holozoa
Osmo_Teretosporea
Pp_Diaphoretickes
Osmo_Fungi
Other_Holomycota
Osmo_Pseudofungi
Cp_Diaphoretickes
Other_Diaphoretickes
Other_Stramenopiles
Other_Amorphea
Osmo_Labyrinthulea
Other
Non-eukaryotes
Other_SAR

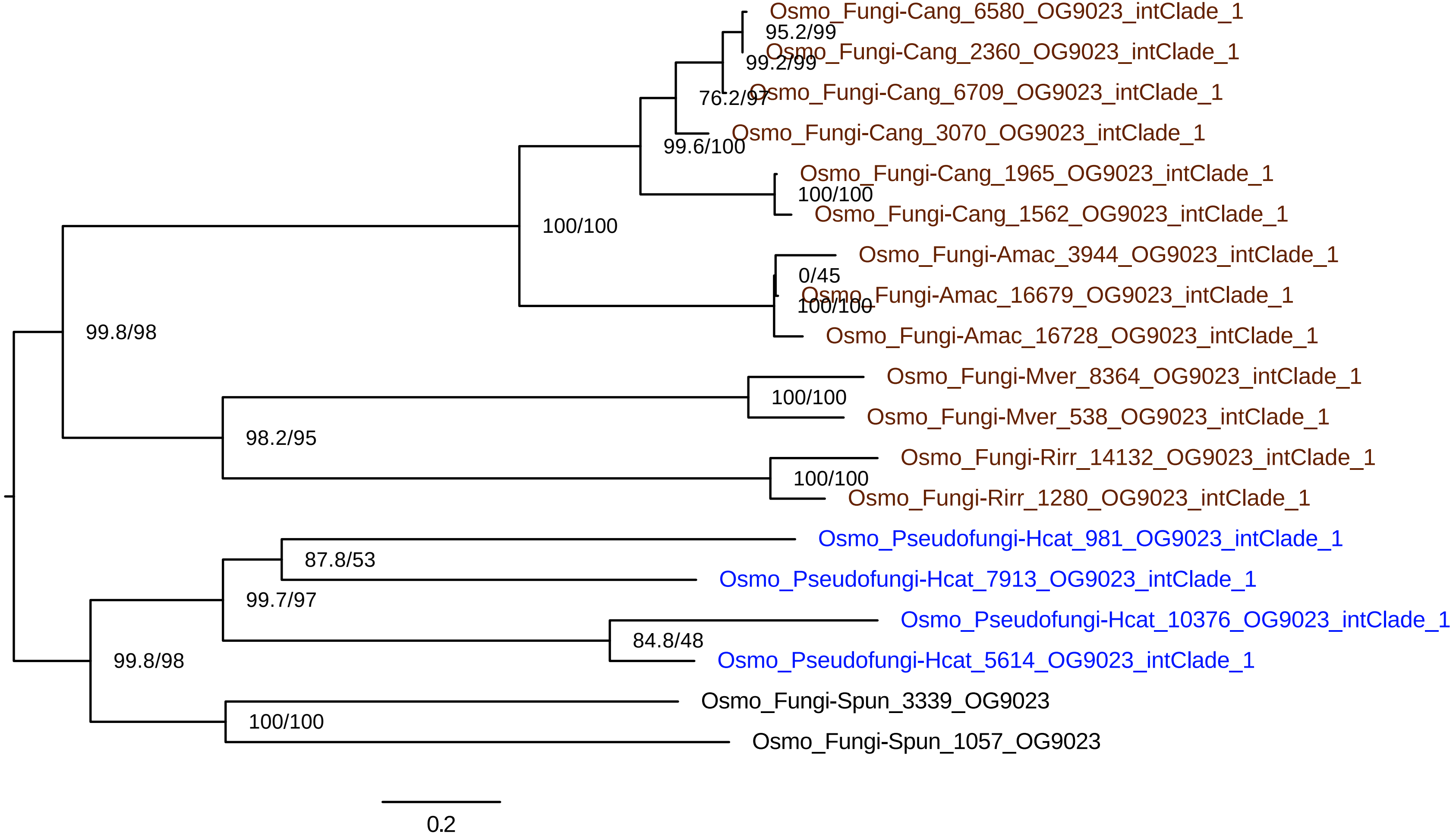
Osmo_Fungi-Cang_6580_OG9023_intClade_1
95.2/99
Osmo_Fungi-Cang_2360_OG9023_intClade_1
99.2/99
Osmo_Fungi-Cang_6709_OG9023_intClade_1
76.2/97
Osmo_Fungi-Cang_3070_OG9023_intClade_1
99.6/100
Osmo_Fungi-Cang_1965_OG9023_intClade_1
100/100
Osmo_Fungi-Cang_1562_OG9023_intClade_1
100/100
Osmo_Fungi-Amac_3944_OG9023_intClade_1
0/45
Osmo_Fungi-Amac_16679_OG9023_intClade_1
100/100
Osmo_Fungi-Amac_16728_OG9023_intClade_1
99.8/98
Osmo_Fungi-Mver_8364_OG9023_intClade_1
100/100
Osmo_Fungi-Mver_538_OG9023_intClade_1
98.2/95
Osmo_Fungi-Rirr_14132_OG9023_intClade_1
100/100
Osmo_Fungi-Rirr_1280_OG9023_intClade_1
Osmo_Pseudofungi-Hcat_981_OG9023_intClade_1
87.8/53
Osmo_Pseudofungi-Hcat_7913_OG9023_intClade_1
99.7/97
Osmo_Pseudofungi-Hcat_10376_OG9023_intClade_1
84.8/48
Osmo_Pseudofungi-Hcat_5614_OG9023_intClade_1
99.8/98
Osmo_Fungi-Spun_3339_OG9023
100/100
Osmo_Fungi-Spun_1057_OG9023
0.2

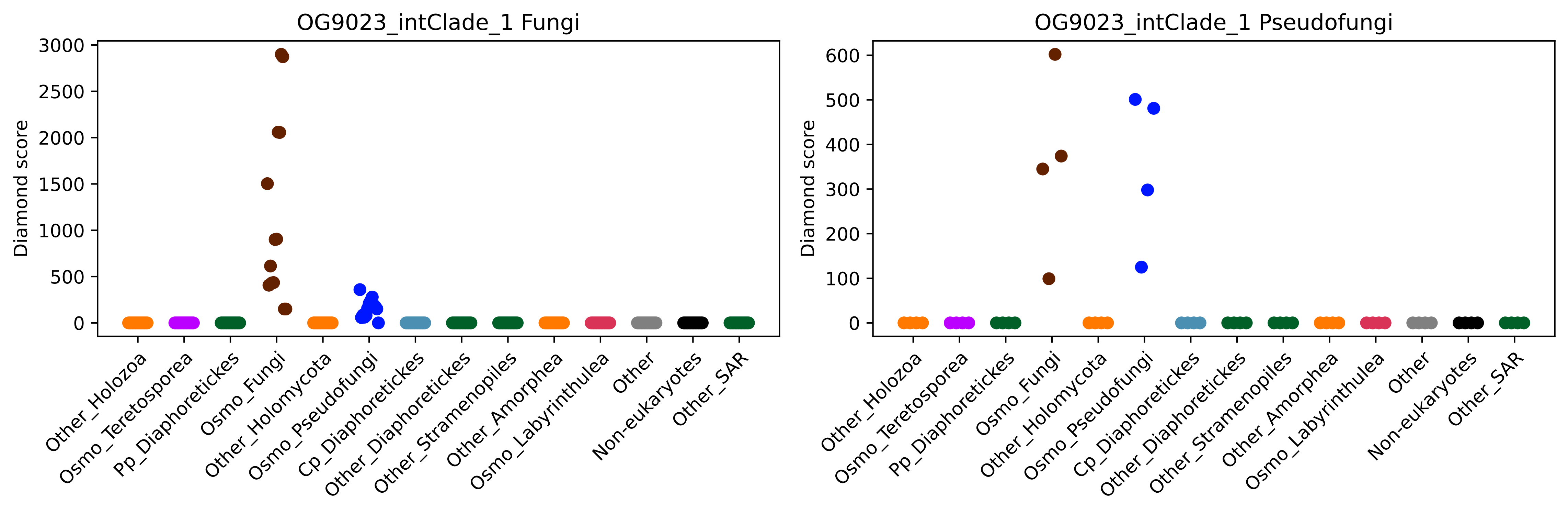

OG9023_intClade_1 Fungi
OG9023_intClade_1 Pseudofungi
Diamond score
0
500
1000
1500
2000
2500
3000
100
200
300
400
500
600
Other_Holozoa
Osmo_Teretosporea
Pp_Diaphoretickes
Osmo_Fungi
Other_Holomycota
Osmo_Pseudofungi
Cp_Diaphoretickes
Other_Diaphoretickes
Other_Stramenopiles
Other_Amorphea
Osmo_Labyrinthulea
Other
Non-eukaryotes
Other_SAR

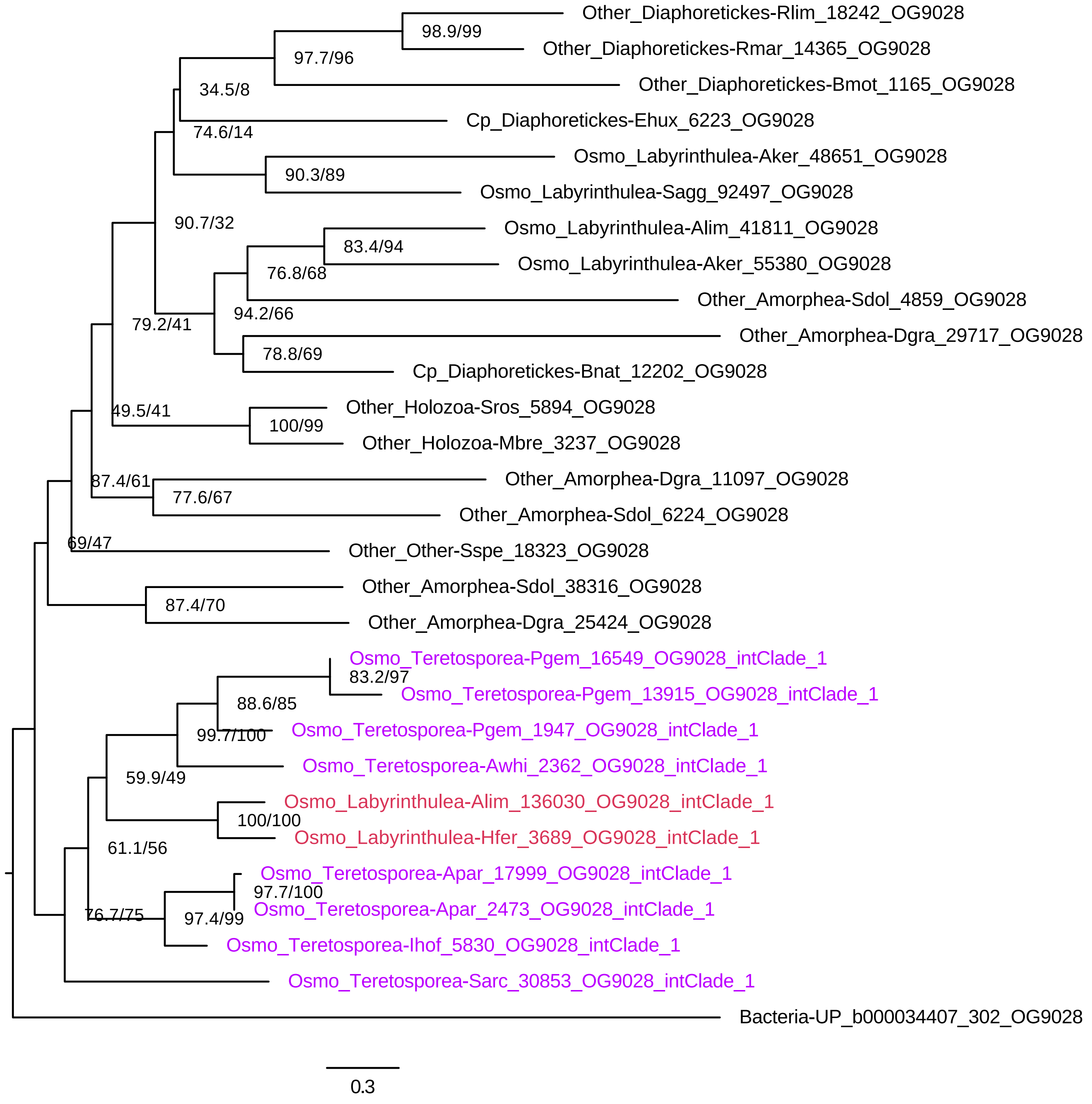
Other_Diaphoretickes-Rlim_18242_OG9028
98.9/99
Other_Diaphoretickes-Rmar_14365_OG9028
97.7/96
Other_Diaphoretickes-Bmot_1165_OG9028
34.5/8
Cp_Diaphoretickes-Ehux_6223_OG9028
74.6/14
Osmo_Labyrinthulea-Aker_48651_OG9028
90.3/89
Osmo_Labyrinthulea-Sagg_92497_OG9028
90.7/32
Osmo_Labyrinthulea-Alim_41811_OG9028
83.4/94
Osmo_Labyrinthulea-Aker_55380_OG9028
76.8/68
Other_Amorphea-Sdol_4859_OG9028
94.2/66
79.2/41
Other_Amorphea-Dgra_29717_OG9028
78.8/69
Cp_Diaphoretickes-Bnat_12202_OG9028
Other_Holozoa-Sros_5894_OG9028
49.5/41
100/99
Other_Holozoa-Mbre_3237_OG9028
Other_Amorphea-Dgra_11097_OG9028
87.4/61
77.6/67
Other_Amorphea-Sdol_6224_OG9028
69/47
Other_Other-Sspe_18323_OG9028
Other_Amorphea-Sdol_38316_OG9028
87.4/70
Other_Amorphea-Dgra_25424_OG9028
Osmo_Teretosporea-Pgem_16549_OG9028_intClade_1
83.2/97
Osmo_Teretosporea-Pgem_13915_OG9028_intClade_1
88.6/85
Osmo_Teretosporea-Pgem_1947_OG9028_intClade_1
99.7/100
Osmo_Teretosporea-Awhi_2362_OG9028_intClade_1
59.9/49
Osmo_Labyrinthulea-Alim_136030_OG9028_intClade_1
100/100
Osmo_Labyrinthulea-Hfer_3689_OG9028_intClade_1
61.1/56
Osmo_Teretosporea-Apar_17999_OG9028_intClade_1
97.7/100
Osmo_Teretosporea-Apar_2473_OG9028_intClade_1
76.7/75
97.4/99
Osmo_Teretosporea-Ihof_5830_OG9028_intClade_1
Osmo_Teretosporea-Sarc_30853_OG9028_intClade_1
Bacteria-UP_b000034407_302_OG9028
0.3

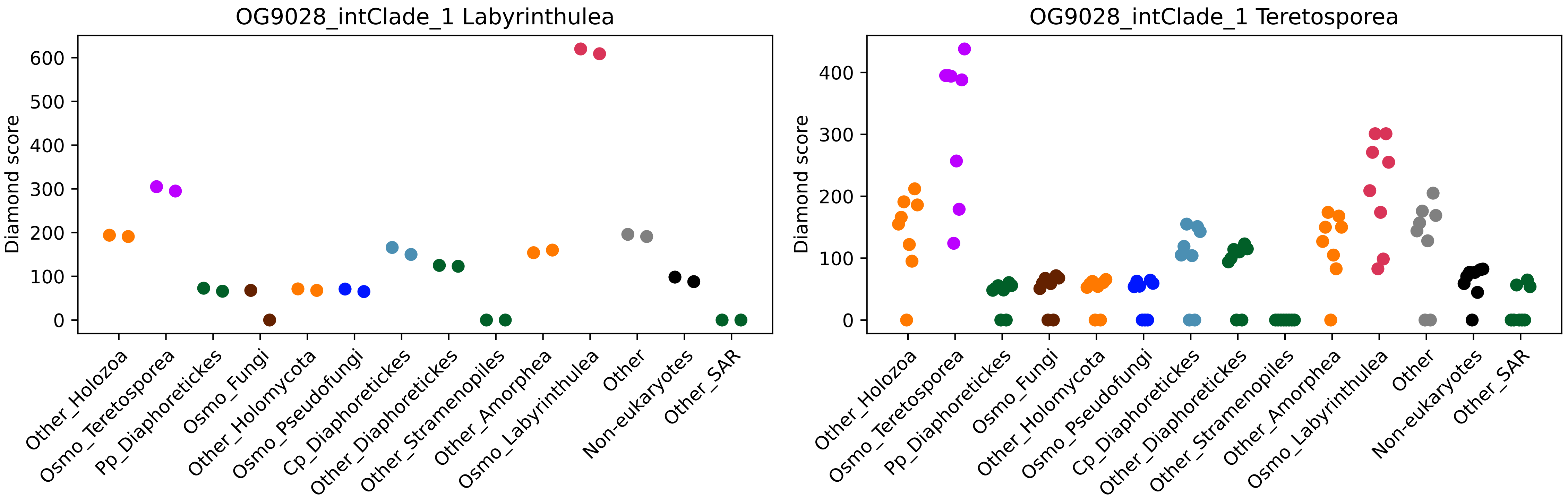

OG9028_intClade_1 Labyrinthulea
OG9028_intClade_1 Teretosporea
Diamond score
0
100
200
300
400
500
600
Other_Holozoa
Osmo_Teretosporea
Pp_Diaphoretickes
Osmo_Fungi
Other_Holomycota
Osmo_Pseudofungi
Cp_Diaphoretickes
Other_Diaphoretickes
Other_Stramenopiles
Other_Amorphea
Osmo_Labyrinthulea
Other
Non-eukaryotes
Other_SAR

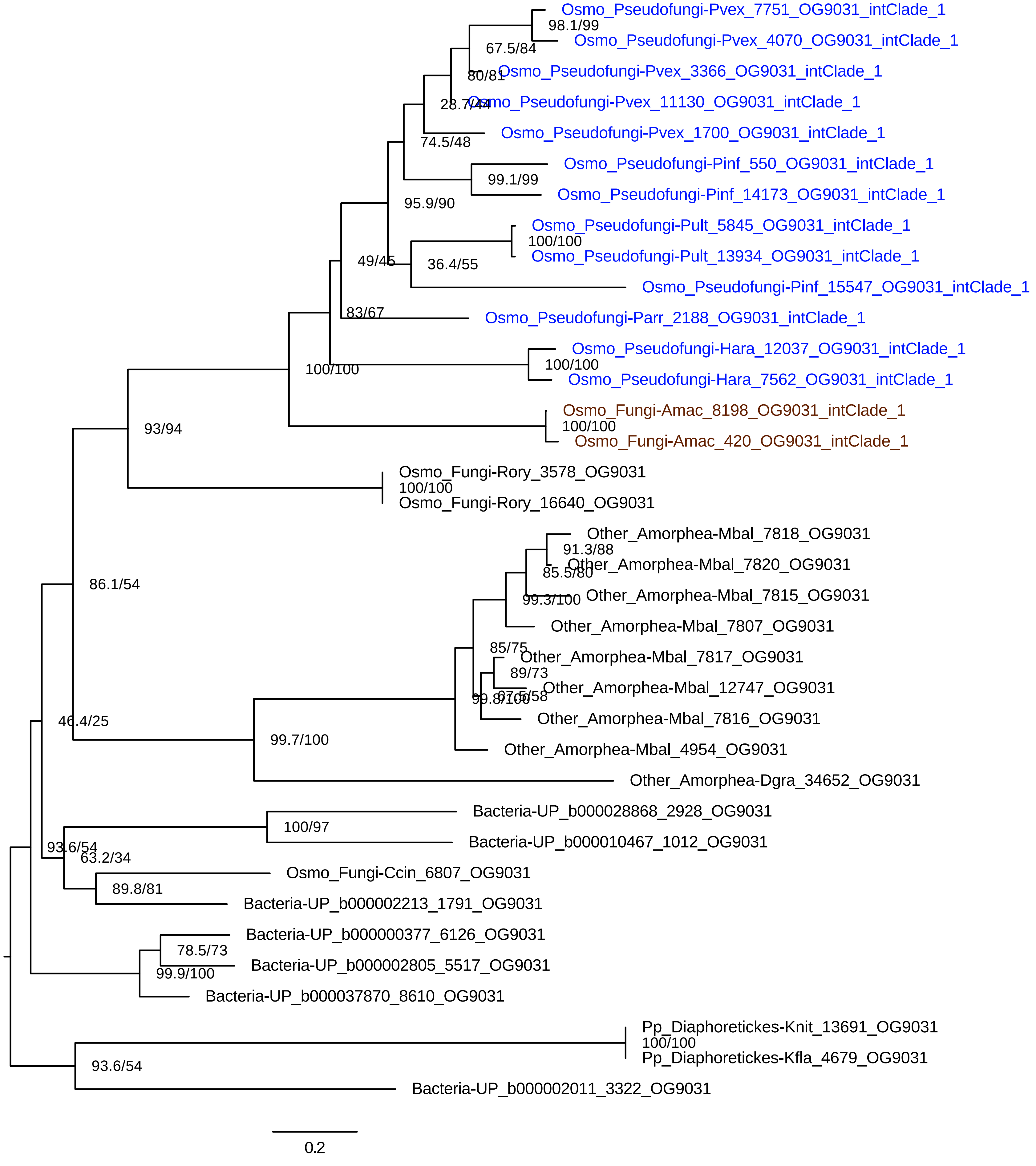
Osmo_Pseudofungi-Pvex_7751_OG9031_intClade_1
98.1/99
Osmo_Pseudofungi-Pvex_4070_OG9031_intClade_1
67.5/84
Osmo_Pseudofungi-Pvex_3366_OG9031_intClade_1
80/81
Osmo_Pseudofungi-Pvex_11130_OG9031_intClade_1
28.7/44
Osmo_Pseudofungi-Pvex_1700_OG9031_intClade_1
74.5/48
Osmo_Pseudofungi-Pinf_550_OG9031_intClade_1
99.1/99
Osmo_Pseudofungi-Pinf_14173_OG9031_intClade_1
95.9/90
Osmo_Pseudofungi-Pult_5845_OG9031_intClade_1
100/100
Osmo_Pseudofungi-Pult_13934_OG9031_intClade_1
49/45
36.4/55
Osmo_Pseudofungi-Pinf_15547_OG9031_intClade_1
83/67
Osmo_Pseudofungi-Parr_2188_OG9031_intClade_1
Osmo_Pseudofungi-Hara_12037_OG9031_intClade_1
100/100
100/100
Osmo_Pseudofungi-Hara_7562_OG9031_intClade_1
Osmo_Fungi-Amac_8198_OG9031_intClade_1
100/100
Osmo_Fungi-Amac_420_OG9031_intClade_1
93/94
Osmo_Fungi-Rory_3578_OG9031
100/100
Osmo_Fungi-Rory_16640_OG9031
Other_Amorphea-Mbal_7818_OG9031
91.3/88
Other_Amorphea-Mbal_7820_OG9031
85.5/80
86.1/54
Other_Amorphea-Mbal_7815_OG9031
99.3/100
Other_Amorphea-Mbal_7807_OG9031
85/75
Other_Amorphea-Mbal_7817_OG9031
89/73
Other_Amorphea-Mbal_12747_OG9031
67.5/58
99.8/100
Other_Amorphea-Mbal_7816_OG9031
46.4/25
99.7/100
Other_Amorphea-Mbal_4954_OG9031
Other_Amorphea-Dgra_34652_OG9031
Bacteria-UP_b000028868_2928_OG9031
100/97
Bacteria-UP_b000010467_1012_OG9031
93.6/54
63.2/34
Osmo_Fungi-Ccin_6807_OG9031
89.8/81
Bacteria-UP_b000002213_1791_OG9031
Bacteria-UP_b000000377_6126_OG9031
78.5/73
Bacteria-UP_b000002805_5517_OG9031
99.9/100
Bacteria-UP_b000037870_8610_OG9031
Pp_Diaphoretickes-Knit_13691_OG9031
100/100
Pp_Diaphoretickes-Kfla_4679_OG9031
93.6/54
Bacteria-UP_b000002011_3322_OG9031
0.2

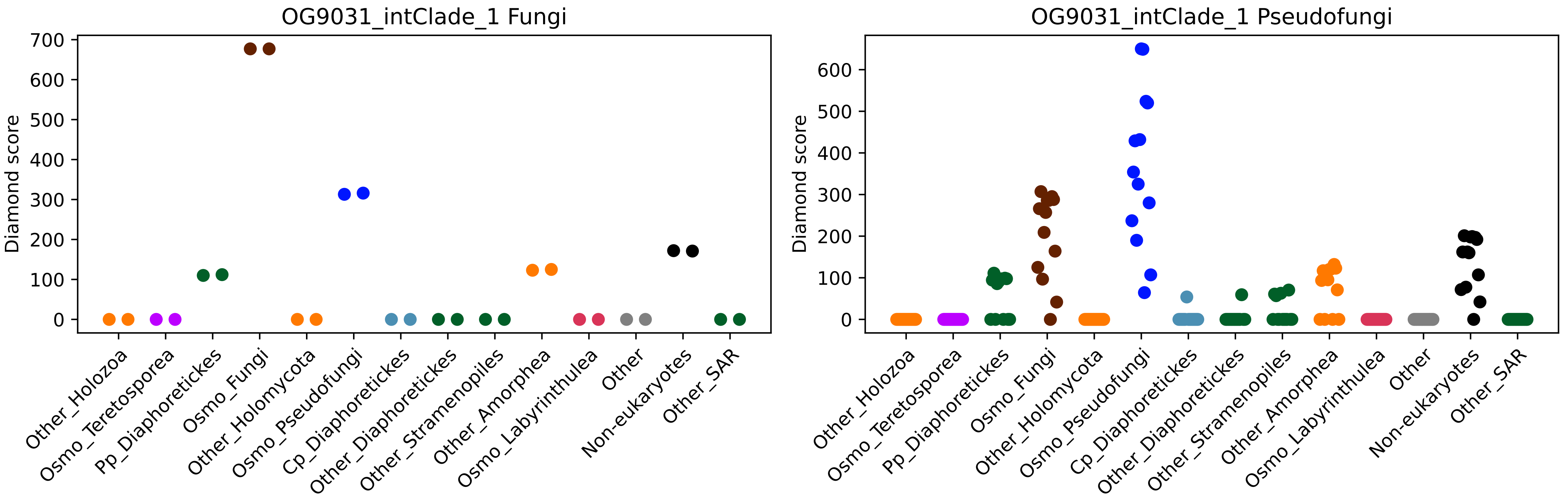
OG9031_intClade_1 Fungi
Diamond score
0
100
200
300
400
500
600
700
Other_Holozoa
Osmo_Teretosporea
Pp_Diaphoretickes
Osmo_Fungi
Other_Holomycota
Osmo_Pseudofungi
Cp_Diaphoretickes
Other_Diaphoretickes
Other_Stramenopiles
Other_Amorphea
Osmo_Labyrinthulea
Other
Non-eukaryotes
Other_SAR
OG9031_intClade_1 Pseudofungi
Diamond score
0
100
200
300
400
500
600
Other_Holozoa
Osmo_Teretosporea
Pp_Diaphoretickes
Osmo_Fungi
Other_Holomycota
Osmo_Pseudofungi
Cp_Diaphoretickes
Other_Diaphoretickes
Other_Stramenopiles
Other_Amorphea
Osmo_Labyrinthulea
Other
Non-eukaryotes
Other_SAR

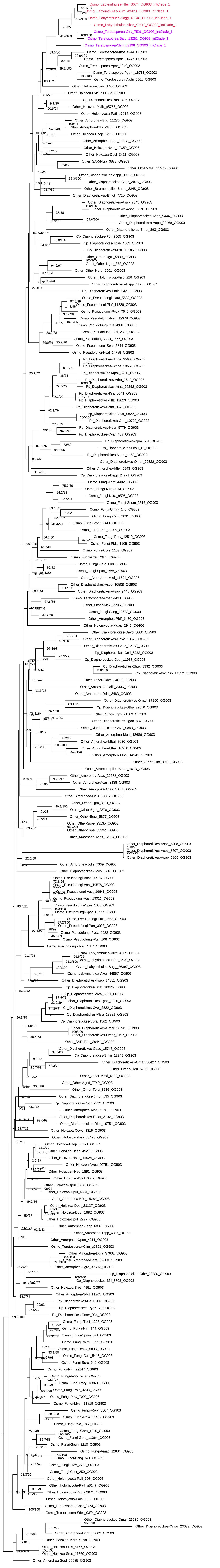
0.4

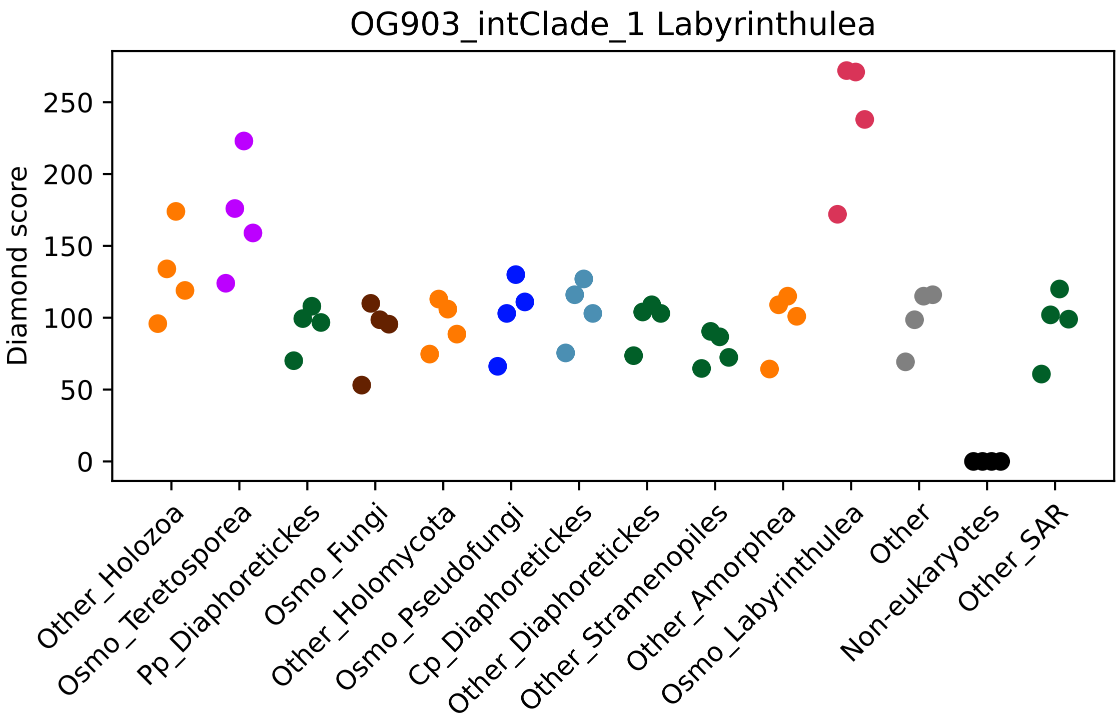
OG903_intClade_1 Labyrinthulea
Diamond score
0
50
100
150
200
250
Other_Holozoa
Osmo_Teretosporea
Pp_Diaphoretickes
Osmo_Fungi
Other_Holomycota
Osmo_Pseudofungi
Cp_Diaphoretickes
Other_Diaphoretickes
Other_Stramenopiles
Other_Amorphea
Osmo_Labyrinthulea
Other
Non-eukaryotes
Other_SAR

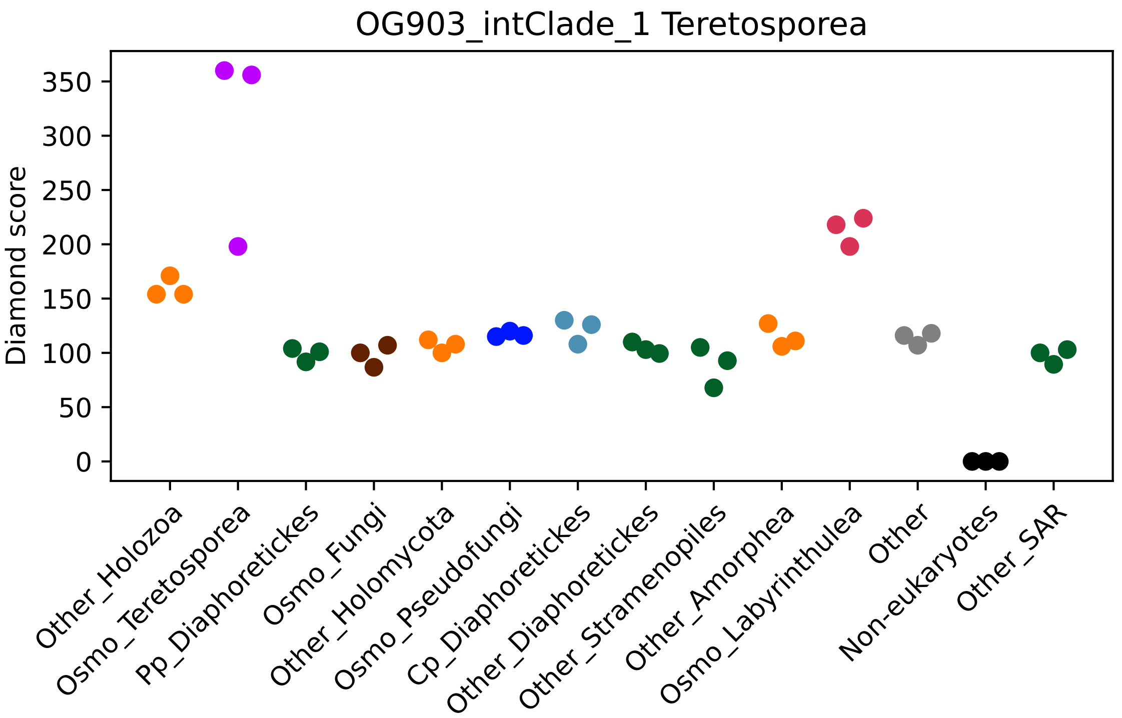
OG903_intClade_1 Teretosporea
Diamond score
0
50
100
150
200
250
300
350
Other_Holozoa
Osmo_Teretosporea
Pp_Diaphoretickes
Osmo_Fungi
Other_Holomycota
Osmo_Pseudofungi
Cp_Diaphoretickes
Other_Diaphoretickes
Other_Stramenopiles
Other_Amorphea
Osmo_Labyrinthulea
Other
Non-eukaryotes
Other_SAR

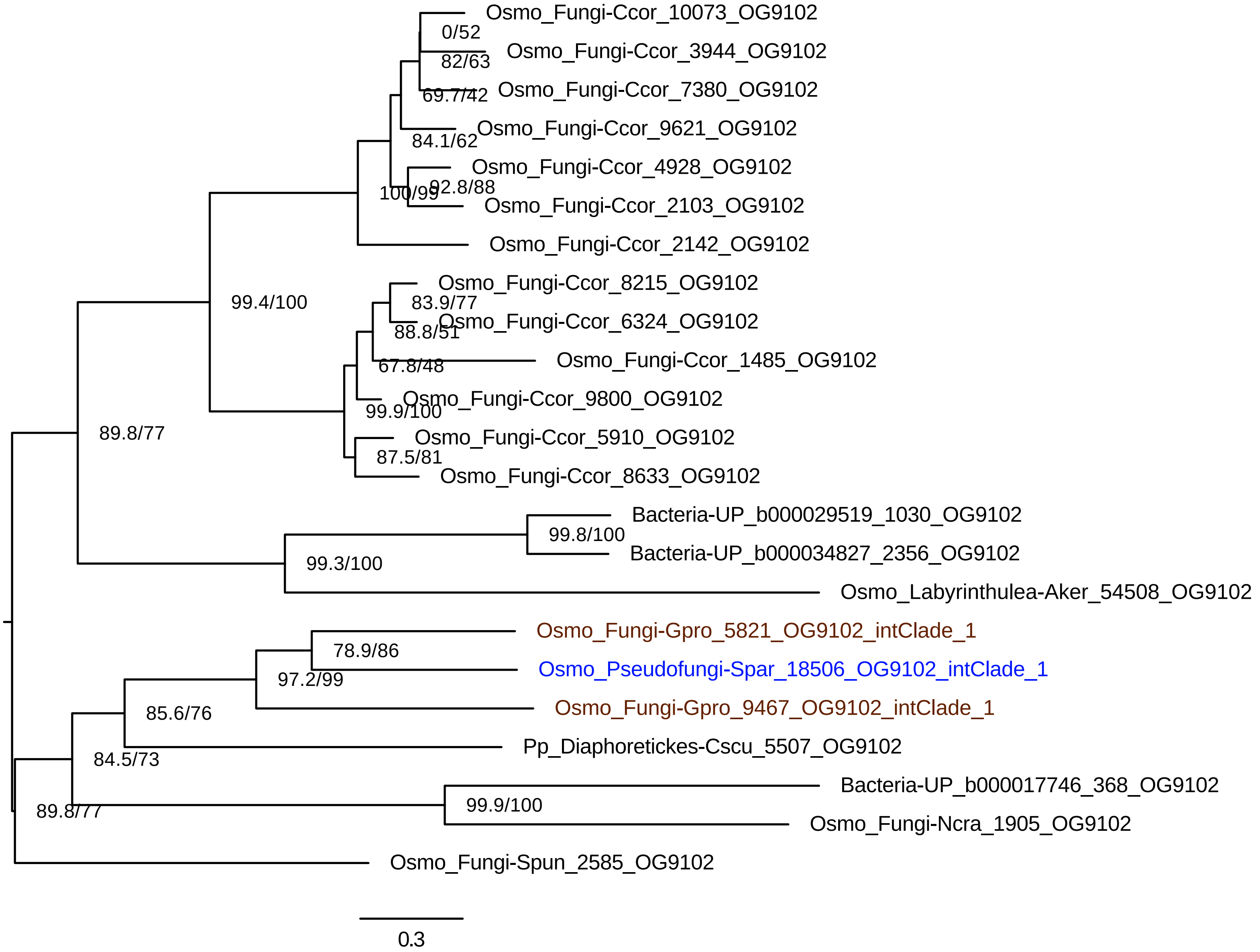
Osmo_Fungi-Ccor_10073_OG9102
0/52
Osmo_Fungi-Ccor_3944_OG9102
82/63
Osmo_Fungi-Ccor_7380_OG9102
69.7/42
Osmo_Fungi-Ccor_9621_OG9102
84.1/62
Osmo_Fungi-Ccor_4928_OG9102
100/99
92.8/88
Osmo_Fungi-Ccor_2103_OG9102
Osmo_Fungi-Ccor_2142_OG9102
99.4/100
Osmo_Fungi-Ccor_8215_OG9102
83.9/77
Osmo_Fungi-Ccor_6324_OG9102
88.8/51
Osmo_Fungi-Ccor_1485_OG9102
67.8/48
Osmo_Fungi-Ccor_9800_OG9102
99.9/100
89.8/77
Osmo_Fungi-Ccor_5910_OG9102
87.5/81
Osmo_Fungi-Ccor_8633_OG9102
Bacteria-UP_b000029519_1030_OG9102
99.8/100
Bacteria-UP_b000034827_2356_OG9102
99.3/100
Osmo_Labyrinthulea-Aker_54508_OG9102
Osmo_Fungi-Gpro_5821_OG9102_intClade_1
78.9/86
Osmo_Pseudofungi-Spar_18506_OG9102_intClade_1
97.2/99
Osmo_Fungi-Gpro_9467_OG9102_intClade_1
85.6/76
Pp_Diaphoretickes-Cscu_5507_OG9102
84.5/73
Bacteria-UP_b000017746_368_OG9102
99.9/100
89.8/77
Osmo_Fungi-Ncra_1905_OG9102
Osmo_Fungi-Spun_2585_OG9102
0.3

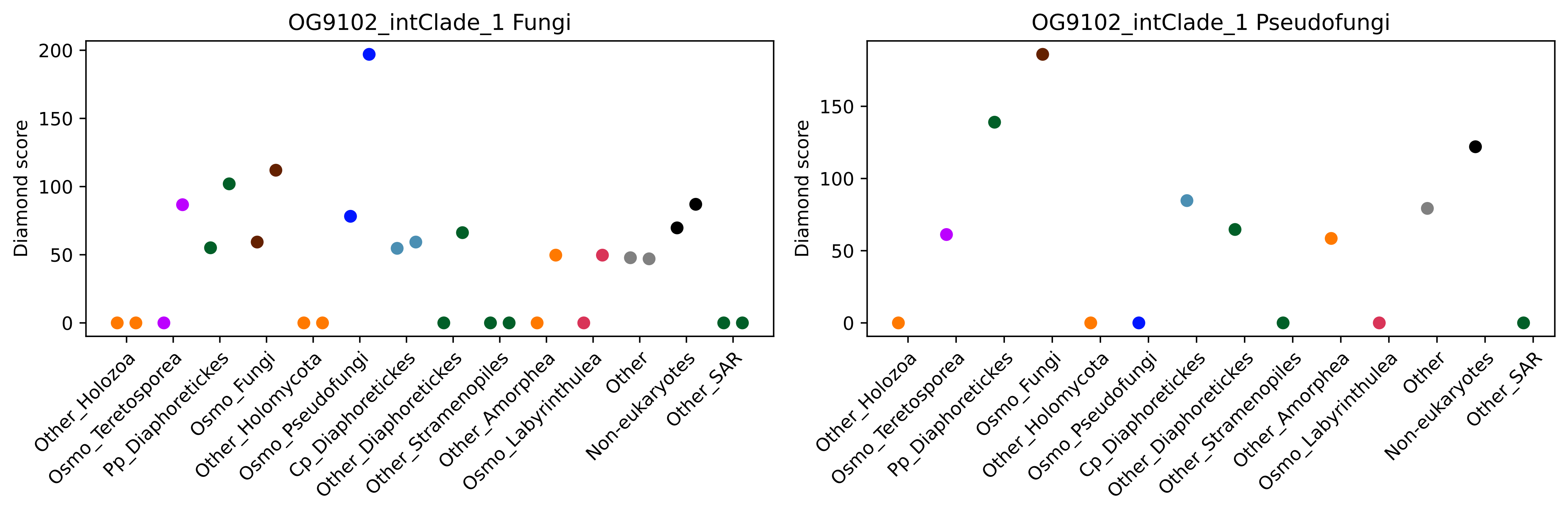

OG9102_intClade_1 Fungi
Diamond score
200
150
100
50
0
Other_Holozoa
Osmo_Teretosporea
Pp_Diaphoretickes
Osmo_Fungi
Other_Holomycota
Osmo_Pseudofungi
Cp_Diaphoretickes
Other_Diaphoretickes
Other_Stramenopiles
Other_Amorphea
Osmo_Labyrinthulea
Other
Non-eukaryotes
Other_SAR
OG9102_intClade_1 Pseudofungi
Diamond score
150
100
50
0
Other_Holozoa
Osmo_Teretosporea
Pp_Diaphoretickes
Osmo_Fungi
Other_Holomycota
Osmo_Pseudofungi
Cp_Diaphoretickes
Other_Diaphoretickes
Other_Stramenopiles
Other_Amorphea
Osmo_Labyrinthulea
Other
Non-eukaryotes
Other_SAR

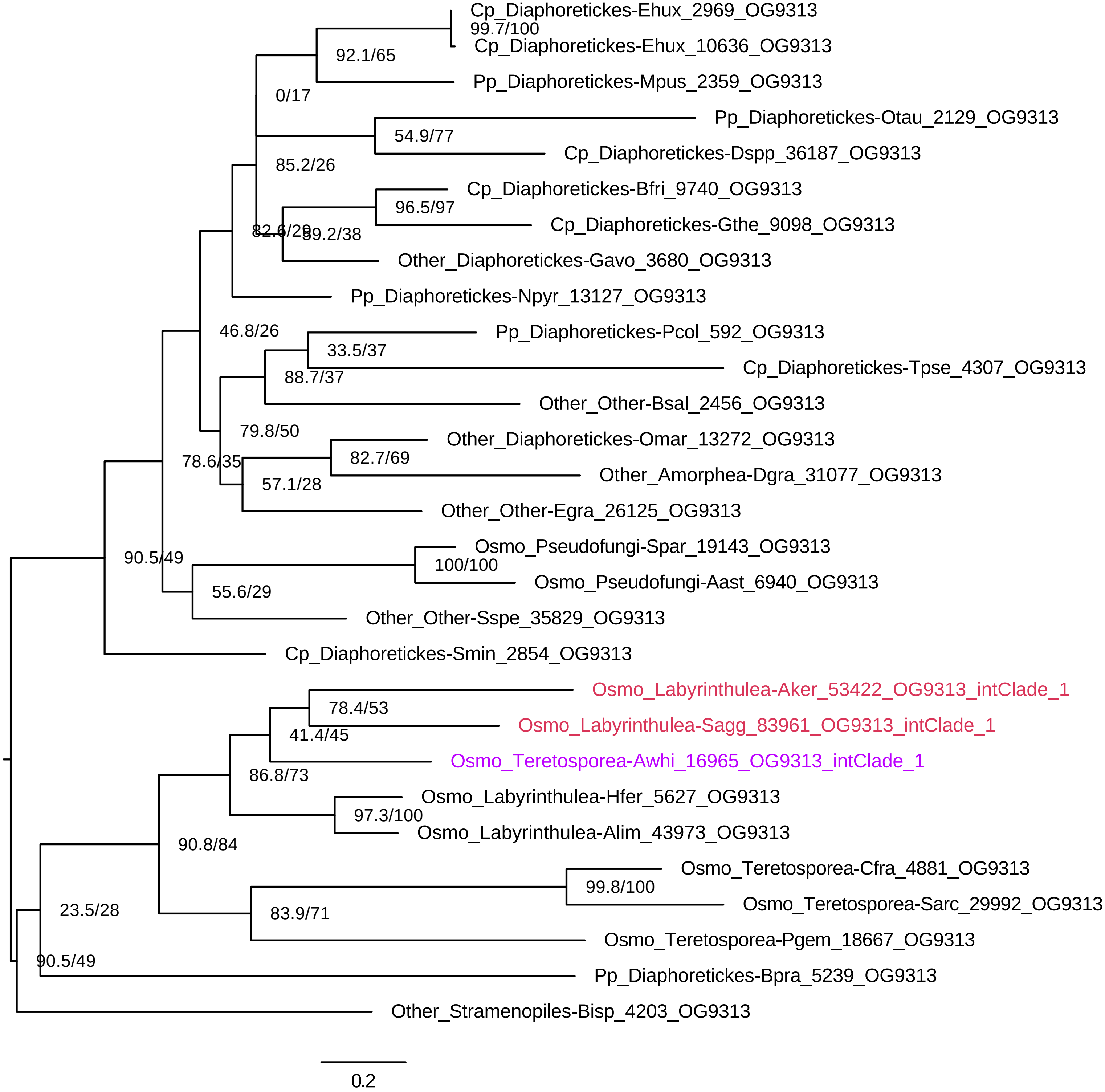
Cp_Diaphoretickes-Ehux_2969_OG9313
99.7/100
Cp_Diaphoretickes-Ehux_10636_OG9313
92.1/65
Pp_Diaphoretickes-Mpus_2359_OG9313
0/17
Pp_Diaphoretickes-Otau_2129_OG9313
54.9/77
Cp_Diaphoretickes-Dspp_36187_OG9313
85.2/26
Cp_Diaphoretickes-Bfri_9740_OG9313
96.5/97
Cp_Diaphoretickes-Gthe_9098_OG9313
82.6/28
99.2/38
Other_Diaphoretickes-Gavo_3680_OG9313
Pp_Diaphoretickes-Npyr_13127_OG9313
46.8/26
Pp_Diaphoretickes-Pcol_592_OG9313
33.5/37
Cp_Diaphoretickes-Tpse_4307_OG9313
88.7/37
Other_Other-Bsal_2456_OG9313
79.8/50
Other_Diaphoretickes-Omar_13272_OG9313
82.7/69
78.6/35
Other_Amorphea-Dgra_31077_OG9313
57.1/28
Other_Other-Egra_26125_OG9313
Osmo_Pseudofungi-Spar_19143_OG9313
90.5/49
100/100
Osmo_Pseudofungi-Aast_6940_OG9313
55.6/29
Other_Other-Sspe_35829_OG9313
Cp_Diaphoretickes-Smin_2854_OG9313
Osmo_Labyrinthulea-Aker_53422_OG9313_intClade_1
78.4/53
Osmo_Labyrinthulea-Sagg_83961_OG9313_intClade_1
41.4/45
Osmo_Teretosporea-Awhi_16965_OG9313_intClade_1
86.8/73
Osmo_Labyrinthulea-Hfer_5627_OG9313
97.3/100
Osmo_Labyrinthulea-Alim_43973_OG9313
90.8/84
Osmo_Teretosporea-Cfra_4881_OG9313
99.8/100
23.5/28
83.9/71
Osmo_Teretosporea-Sarc_29992_OG9313
Osmo_Teretosporea-Pgem_18667_OG9313
90.5/49
Pp_Diaphoretickes-Bpra_5239_OG9313
Other_Stramenopiles-Bisp_4203_OG9313
0.2

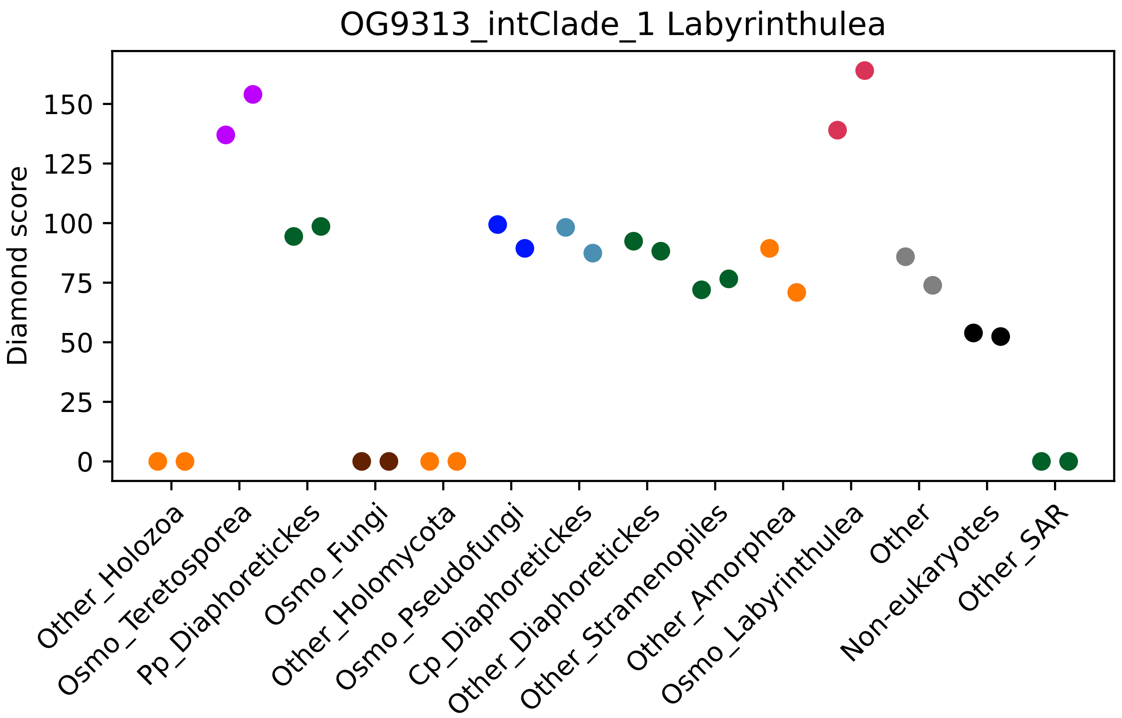
OG9313_intClade_1 Labyrinthulea
Diamond score
0
25
50
75
100
125
150
Other_Holozoa
Osmo_Teretosporea
Pp_Diaphoretickes
Osmo_Fungi
Other_Holomycota
Osmo_Pseudofungi
Cp_Diaphoretickes
Other_Diaphoretickes
Other_Stramenopiles
Other_Amorphea
Osmo_Labyrinthulea
Other
Non-eukaryotes
Other_SAR

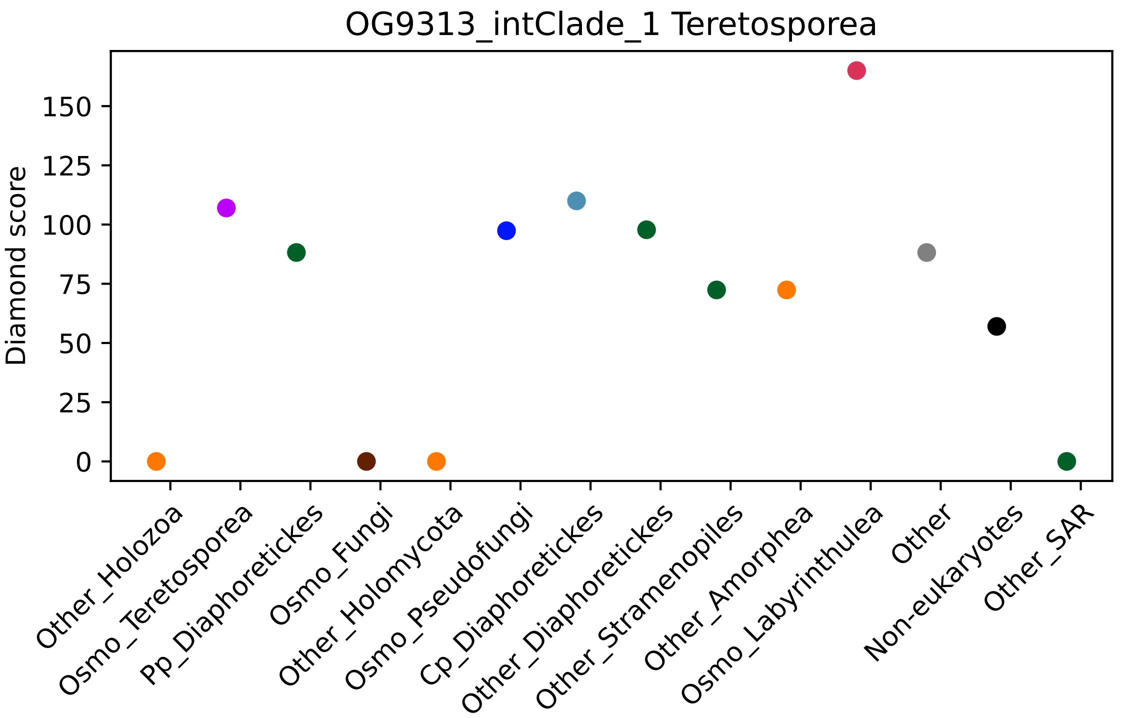
OG9313_intClade_1 Teretosporea
Diamond score
0
25
50
75
100
125
150
Other_Holozoa
Osmo_Teretosporea
Pp_Diaphoretickes
Osmo_Fungi
Other_Holomycota
Osmo_Pseudofungi
Cp_Diaphoretickes
Other_Diaphoretickes
Other_Stramenopiles
Other_Amorphea
Osmo_Labyrinthulea
Other
Non-eukaryotes
Other_SAR

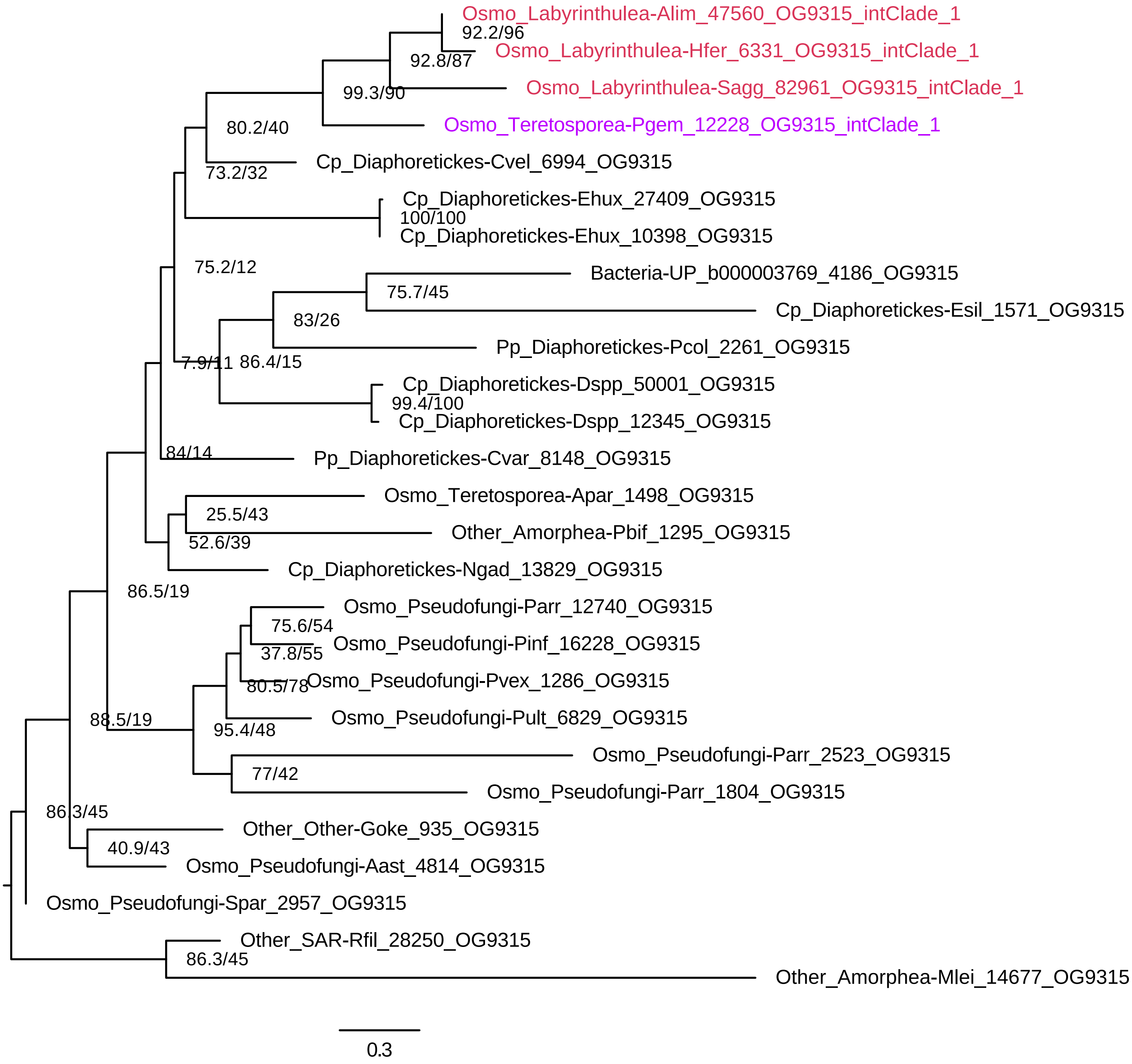
Osmo_Labyrinthulea-Alim_47560_OG9315_intClade_1
92.2/96
Osmo_Labyrinthulea-Hfer_6331_OG9315_intClade_1
92.8/87
Osmo_Labyrinthulea-Sagg_82961_OG9315_intClade_1
99.3/90
Osmo_Teretosporea-Pgem_12228_OG9315_intClade_1
80.2/40
Cp_Diaphoretickes-Cvel_6994_OG9315
73.2/32
Cp_Diaphoretickes-Ehux_27409_OG9315
100/100
Cp_Diaphoretickes-Ehux_10398_OG9315
75.2/12
Bacteria-UP_b000003769_4186_OG9315
75.7/45
Cp_Diaphoretickes-Esil_1571_OG9315
83/26
Pp_Diaphoretickes-Pcol_2261_OG9315
7.9/11
86.4/15
Cp_Diaphoretickes-Dspp_50001_OG9315
99.4/100
Cp_Diaphoretickes-Dspp_12345_OG9315
84/14
Pp_Diaphoretickes-Cvar_8148_OG9315
Osmo_Teretosporea-Apar_1498_OG9315
25.5/43
Other_Amorphea-Pbif_1295_OG9315
52.6/39
Cp_Diaphoretickes-Ngad_13829_OG9315
86.5/19
Osmo_Pseudofungi-Parr_12740_OG9315
75.6/54
Osmo_Pseudofungi-Pinf_16228_OG9315
37.8/55
80.5/78
Osmo_Pseudofungi-Pvex_1286_OG9315
Osmo_Pseudofungi-Pult_6829_OG9315
88.5/19
95.4/48
Osmo_Pseudofungi-Parr_2523_OG9315
77/42
Osmo_Pseudofungi-Parr_1804_OG9315
86.3/45
Other_Other-Goke_935_OG9315
40.9/43
Osmo_Pseudofungi-Aast_4814_OG9315
Osmo_Pseudofungi-Spar_2957_OG9315
Other_SAR-Rfil_28250_OG9315
86.3/45
Other_Amorphea-Mlei_14677_OG9315
0.3

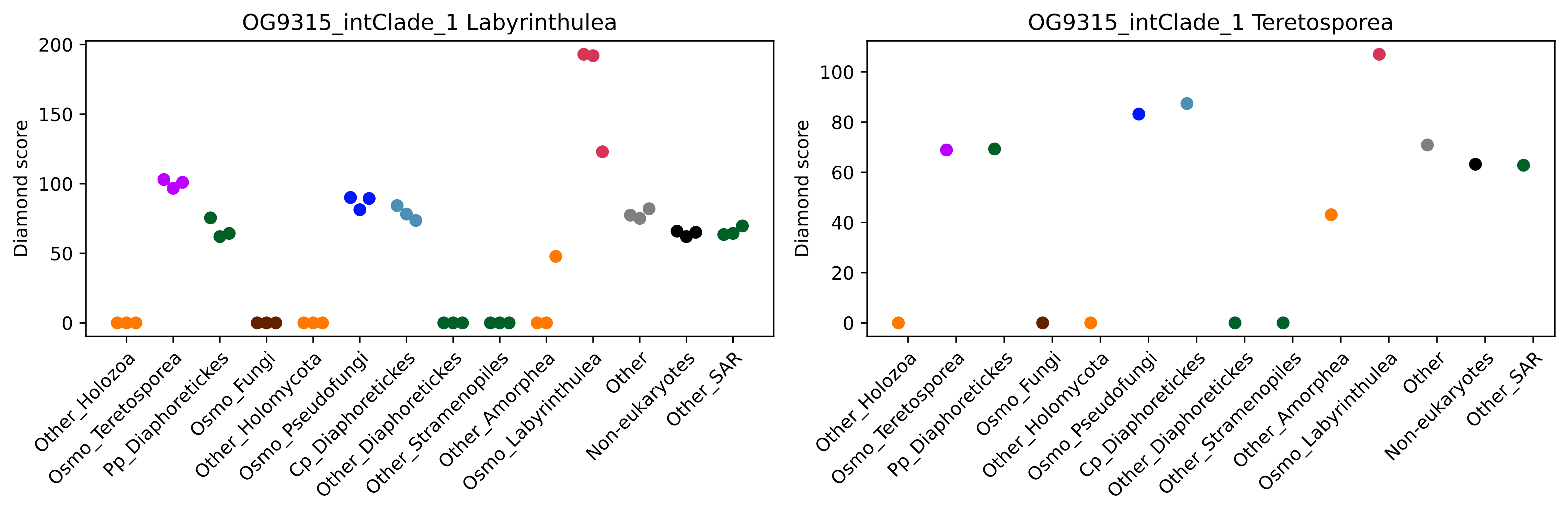

OG9315_intClade_1 Labyrinthulea
OG9315_intClade_1 Teretosporea
Diamond score
0
50
100
150
200
0
20
40
60
80
100
Other_Holozoa
Osmo_Teretosporea
Pp_Diaphoretickes
Osmo_Fungi
Other_Holomycota
Osmo_Pseudofungi
Cp_Diaphoretickes
Other_Diaphoretickes
Other_Stramenopiles
Other_Amorphea
Osmo_Labyrinthulea
Other
Non-eukaryotes
Other_SAR

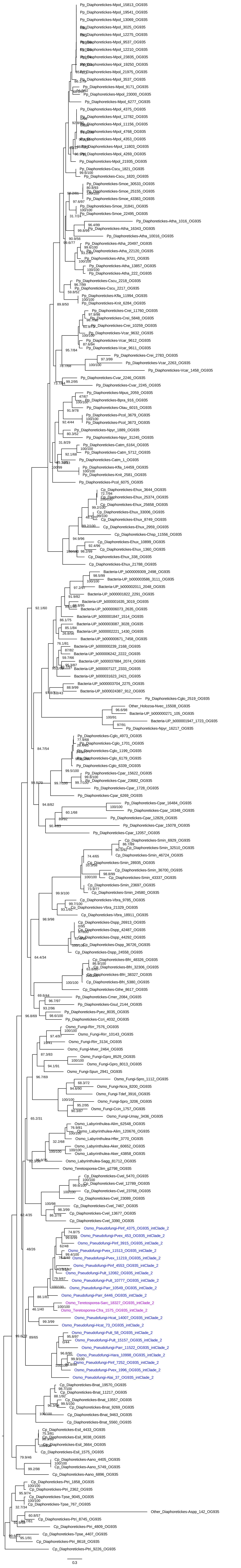

Pp_Diaphoretickes-Mpol_15813_OG935
Pp_Diaphoretickes-Mpol_19541_OG935
Pp_Diaphoretickes-Mpol_13069_OG935
Pp_Diaphoretickes-Mpol_3025_OG935
Pp_Diaphoretickes-Mpol_12275_OG935
Pp_Diaphoretickes-Mpol_9537_OG935
Pp_Diaphoretickes-Mpol_12210_OG935
Pp_Diaphoretickes-Mpol_23835_OG935
Pp_Diaphoretickes-Mpol_19250_OG935
Pp_Diaphoretickes-Mpol_21975_OG935
Pp_Diaphoretickes-Mpol_3537_OG935
Pp_Diaphoretickes-Mpol_9171_OG935
Pp_Diaphoretickes-Mpol_23000_OG935
Pp_Diaphoretickes-Mpol_6277_OG935
Pp_Diaphoretickes-Mpol_4375_OG935
Pp_Diaphoretickes-Mpol_12782_OG935
Pp_Diaphoretickes-Mpol_11156_OG935
Pp_Diaphoretickes-Mpol_4768_OG935
Pp_Diaphoretickes-Mpol_4353_OG935
Pp_Diaphoretickes-Mpol_11803_OG935
Pp_Diaphoretickes-Mpol_4269_OG935
Pp_Diaphoretickes-Mpol_21935_OG935
Pp_Diaphoretickes-Cscu_1821_OG935
Pp_Diaphoretickes-Cscu_1820_OG935
Pp_Diaphoretickes-Smoe_30533_OG935
Pp_Diaphoretickes-Smoe_25155_OG935
Pp_Diaphoretickes-Smoe_43383_OG935
Pp_Diaphoretickes-Smoe_31841_OG935
Pp_Diaphoretickes-Smoe_22495_OG935
Pp_Diaphoretickes-Atha_1016_OG935
Pp_Diaphoretickes-Atha_16343_OG935
Pp_Diaphoretickes-Atha_10016_OG935
Pp_Diaphoretickes-Atha_20497_OG935
Pp_Diaphoretickes-Atha_22120_OG935
Pp_Diaphoretickes-Atha_9721_OG935
Pp_Diaphoretickes-Atha_13857_OG935
Pp_Diaphoretickes-Atha_222_OG935
Pp_Diaphoretickes-Cscu_2218_OG935
Pp_Diaphoretickes-Cscu_2217_OG935
Pp_Diaphoretickes-Kfla_11994_OG935
Pp_Diaphoretickes-Knit_6284_OG935
Pp_Diaphoretickes-Crei_11760_OG935
Pp_Diaphoretickes-Crei_5848_OG935
Pp_Diaphoretickes-Crei_10259_OG935
Pp_Diaphoretickes-Vcar_9632_OG935
Pp_Diaphoretickes-Vcar_9612_OG935
Pp_Diaphoretickes-Vcar_9611_OG935
Pp_Diaphoretickes-Crei_2783_OG935
Pp_Diaphoretickes-Vcar_2263_OG935
Pp_Diaphoretickes-Vcar_1458_OG935
Pp_Diaphoretickes-Cvar_2246_OG935
Pp_Diaphoretickes-Cvar_2245_OG935
Pp_Diaphoretickes-Mpus_2059_OG935
Pp_Diaphoretickes-Bpra_916_OG935
Pp_Diaphoretickes-Otau_6015_OG935
Pp_Diaphoretickes-Pcol_3679_OG935
Pp_Diaphoretickes-Pcol_3673_OG935
Pp_Diaphoretickes-Npyr_1889_OG935
Pp_Diaphoretickes-Npyr_31245_OG935
Pp_Diaphoretickes-Catm_6164_OG935
Pp_Diaphoretickes-Catm_5712_OG935
Pp_Diaphoretickes-Catm_1_OG935
Pp_Diaphoretickes-Kfla_14459_OG935
Pp_Diaphoretickes-Knit_2581_OG935
Pp_Diaphoretickes-Pcol_6075_OG935
Cp_Diaphoretickes-Ehux_3644_OG935
Cp_Diaphoretickes-Ehux_25374_OG935
Cp_Diaphoretickes-Ehux_25658_OG935
Cp_Diaphoretickes-Ehux_33006_OG935
Cp_Diaphoretickes-Ehux_8749_OG935
Cp_Diaphoretickes-Ehux_44292_OG935
Cp_Diaphoretickes-Ehux_2959_OG935
Cp_Diaphoretickes-Chsp_11556_OG935
Cp_Diaphoretickes-Ehux_10899_OG935
Cp_Diaphoretickes-Ehux_1360_OG935
Cp_Diaphoretickes-Ehux_338_OG935
Cp_Diaphoretickes-Ehux_21788_OG935
Bacteria-UP_b000009309_2498_OG935
Bacteria-UP_b000003586_3111_OG935
Bacteria-UP_b000002011_2048_OG935
Bacteria-UP_b000001822_2291_OG935
Bacteria-UP_b000001635_3019_OG935
Bacteria-UP_b000006073_2635_OG935
Bacteria-UP_b000001847_1514_OG935
Bacteria-UP_b000003087_3028_OG935
Bacteria-UP_b000002221_1430_OG935
Bacteria-UP_b000000671_7458_OG935
Bacteria-UP_b000000239_2168_OG935
Bacteria-UP_b000006242_2222_OG935
Bacteria-UP_b000037884_2074_OG935
Bacteria-UP_b000007127_2333_OG935
Bacteria-UP_b000031623_2421_OG935
Bacteria-UP_b000003704_2275_OG935
Bacteria-UP_b000024387_912_OG935
Pp_Diaphoretickes-Cglo_2519_OG935
Other_Holozoa-Nvec_15508_OG935
Bacteria-UP_b000000271_105_OG935
Bacteria-UP_b000001947_1723_OG935
Pp_Diaphoretickes-Npyr_16217_OG935
Pp_Diaphoretickes-Cglo_4973_OG935
Pp_Diaphoretickes-Cglo_1701_OG935
Pp_Diaphoretickes-Cglo_1199_OG935
Pp_Diaphoretickes-Cglo_6179_OG935
Pp_Diaphoretickes-Cglo_6339_OG935
Pp_Diaphoretickes-Cpar_15622_OG935
Pp_Diaphoretickes-Cpar_23682_OG935
Pp_Diaphoretickes-Cpar_1728_OG935
Pp_Diaphoretickes-Cpar_6269_OG935
Pp_Diaphoretickes-Cpar_16484_OG935
Pp_Diaphoretickes-Cpar_16348_OG935
Pp_Diaphoretickes-Cpar_12829_OG935
Pp_Diaphoretickes-Cpar_15078_OG935
Pp_Diaphoretickes-Cpar_12057_OG935
Cp_Diaphoretickes-Smin_6929_OG935
Cp_Diaphoretickes-Smin_32510_OG935
Cp_Diaphoretickes-Smin_46724_OG935
Cp_Diaphoretickes-Smin_28935_OG935
Cp_Diaphoretickes-Smin_36700_OG935
Cp_Diaphoretickes-Smin_43337_OG935
Cp_Diaphoretickes-Smin_23697_OG935
Cp_Diaphoretickes-Smin_24580_OG935
Cp_Diaphoretickes-Vbra_9785_OG935
Cp_Diaphoretickes-Vbra_21329_OG935
Cp_Diaphoretickes-Vbra_18911_OG935
Cp_Diaphoretickes-Dspp_26913_OG935
Cp_Diaphoretickes-Dspp_42487_OG935
Cp_Diaphoretickes-Dspp_44292_OG935
Cp_Diaphoretickes-Dspp_36726_OG935
Cp_Diaphoretickes-Dspp_24558_OG935
Cp_Diaphoretickes-Bfri_48326_OG935
Cp_Diaphoretickes-Bfri_32306_OG935
Cp_Diaphoretickes-Bfri_38327_OG935
Cp_Diaphoretickes-Bfri_5380_OG935
Cp_Diaphoretickes-Gthe_8617_OG935
Pp_Diaphoretickes-Cmer_2084_OG935
Pp_Diaphoretickes-Gsul_2144_OG935
Pp_Diaphoretickes-Pyez_8035_OG935
Pp_Diaphoretickes-Ccri_4032_OG935
Osmo_Fungi-Rirr_7576_OG935
Osmo_Fungi-Rirr_10143_OG935
Osmo_Fungi-Rirr_3134_OG935
Osmo_Fungi-Mver_2464_OG935
Osmo_Fungi-Gpro_8529_OG935
Osmo_Fungi-Gpro_8013_OG935
Osmo_Fungi-Spun_2941_OG935
Osmo_Fungi-Spro_1112_OG935
Osmo_Fungi-Ncra_8200_OG935
Osmo_Fungi-Tdef_3916_OG935
Osmo_Fungi-Spro_3206_OG935
Osmo_Fungi-Ccin_1757_OG935
Osmo_Fungi-Umay_3436_OG935
Osmo_Labyrinthulea-Alim_62548_OG935
Osmo_Labyrinthulea-Alim_120676_OG935
Osmo_Labyrinthulea-Hfer_3770_OG935
Osmo_Labyrinthulea-Aker_60652_OG935
Osmo_Labyrinthulea-Aker_43858_OG935
Osmo_Labyrinthulea-Sagg_81712_OG935
Osmo_Teretosporea-Clim_g2798_OG935
Cp_Diaphoretickes-Cvel_5470_OG935
Cp_Diaphoretickes-Cvel_12789_OG935
Cp_Diaphoretickes-Cvel_23768_OG935
Cp_Diaphoretickes-Cvel_23089_OG935
Cp_Diaphoretickes-Cvel_7467_OG935
Cp_Diaphoretickes-Cvel_13677_OG935
Cp_Diaphoretickes-Cvel_3390_OG935
Osmo_Pseudofungi-Pinf_4375_OG935_intClade_2
Osmo_Pseudofungi-Pvex_453_OG935_intClade_2
Osmo_Pseudofungi-Pinf_3915_OG935_intClade_2
Osmo_Pseudofungi-Pvex_11513_OG935_intClade_2
Osmo_Pseudofungi-Pvex_11219_OG935_intClade_2
Osmo_Pseudofungi-Pinf_4553_OG935_intClade_2
Osmo_Pseudofungi-Pult_12082_OG935_intClade_2
Osmo_Pseudofungi-Pult_10777_OG935_intClade_2
Osmo_Pseudofungi-Parr_10549_OG935_intClade_2
Osmo_Pseudofungi-Parr_6446_OG935_intClade_2
Osmo_Teretosporea-Sarc_18327_OG935_intClade_2
Osmo_Teretosporea-Cfra_1575_OG935_intClade_2
Osmo_Pseudofungi-Hcat_14007_OG935_intClade_2
Osmo_Pseudofungi-Hcat_73_OG935_intClade_2
Osmo_Pseudofungi-Pult_58_OG935_intClade_2
Osmo_Pseudofungi-Pult_15157_OG935_intClade_2
Osmo_Pseudofungi-Parr_11522_OG935_intClade_2
Osmo_Pseudofungi-Hara_10998_OG935_intClade_2
Osmo_Pseudofungi-Pinf_7252_OG935_intClade_2
Osmo_Pseudofungi-Pvex_1996_OG935_intClade_2
Osmo_Pseudofungi-Alai_37_OG935_intClade_2
Cp_Diaphoretickes-Bnat_19570_OG935
Cp_Diaphoretickes-Bnat_11217_OG935
Cp_Diaphoretickes-Bnat_13557_OG935
Cp_Diaphoretickes-Bnat_9269_OG935
Cp_Diaphoretickes-Bnat_9463_OG935
Cp_Diaphoretickes-Bnat_5560_OG935
Cp_Diaphoretickes-Esil_4433_OG935
Cp_Diaphoretickes-Esil_9038_OG935
Cp_Diaphoretickes-Esil_3664_OG935
Cp_Diaphoretickes-Esil_1575_OG935
Cp_Diaphoretickes-Aano_4405_OG935
Cp_Diaphoretickes-Aano_5749_OG935
Cp_Diaphoretickes-Aano_6896_OG935
Cp_Diaphoretickes-Ptri_1858_OG935
Cp_Diaphoretickes-Ptri_2362_OG935
Cp_Diaphoretickes-Tpse_9045_OG935
Cp_Diaphoretickes-Tpse_767_OG935
Other_Diaphoretickes-Aspp_142_OG935
Cp_Diaphoretickes-Ptri_8745_OG935
Cp_Diaphoretickes-Ptri_4809_OG935
Cp_Diaphoretickes-Tpse_4407_OG935
Cp_Diaphoretickes-Ptri_8618_OG935
Cp_Diaphoretickes-Ptri_9226_OG935
0.3

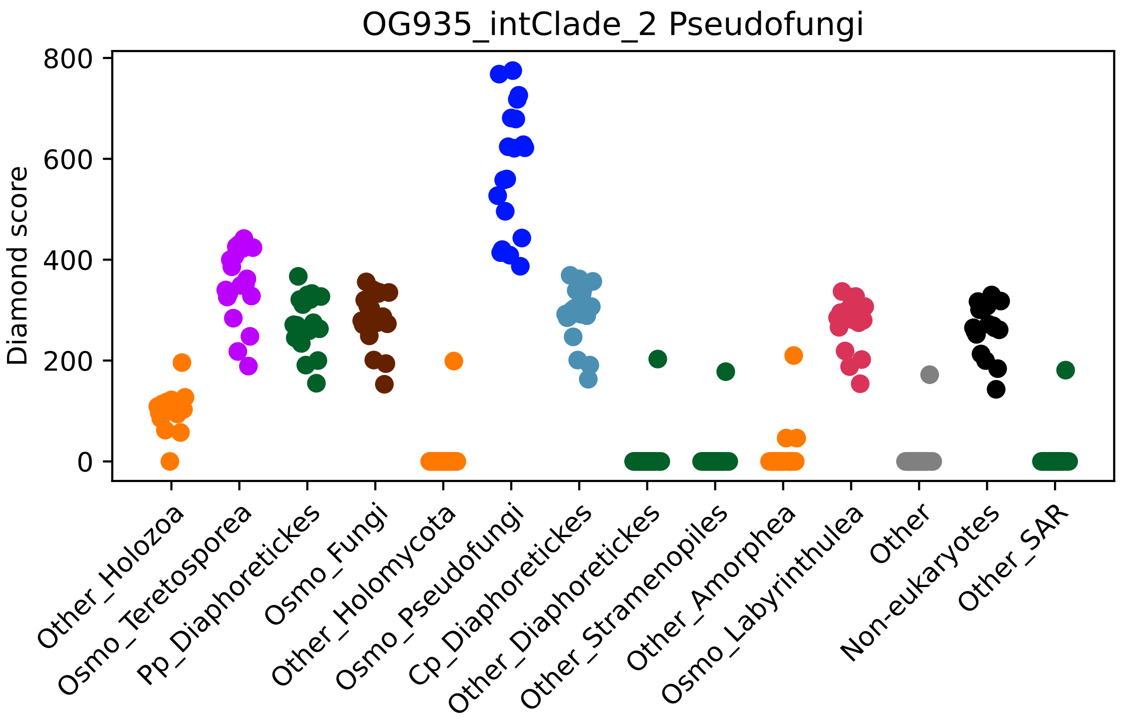

OG935_intClade_2 Pseudofungi
Diamond score
0
200
400
600
800
Other_Holozoa
Osmo_Teretosporea
Pp_Diaphoretickes
Osmo_Fungi
Other_Holomycota
Osmo_Pseudofungi
Cp_Diaphoretickes
Other_Diaphoretickes
Other_Stramenopiles
Other_Amorphea
Osmo_Labyrinthulea
Other
Non-eukaryotes
Other_SAR

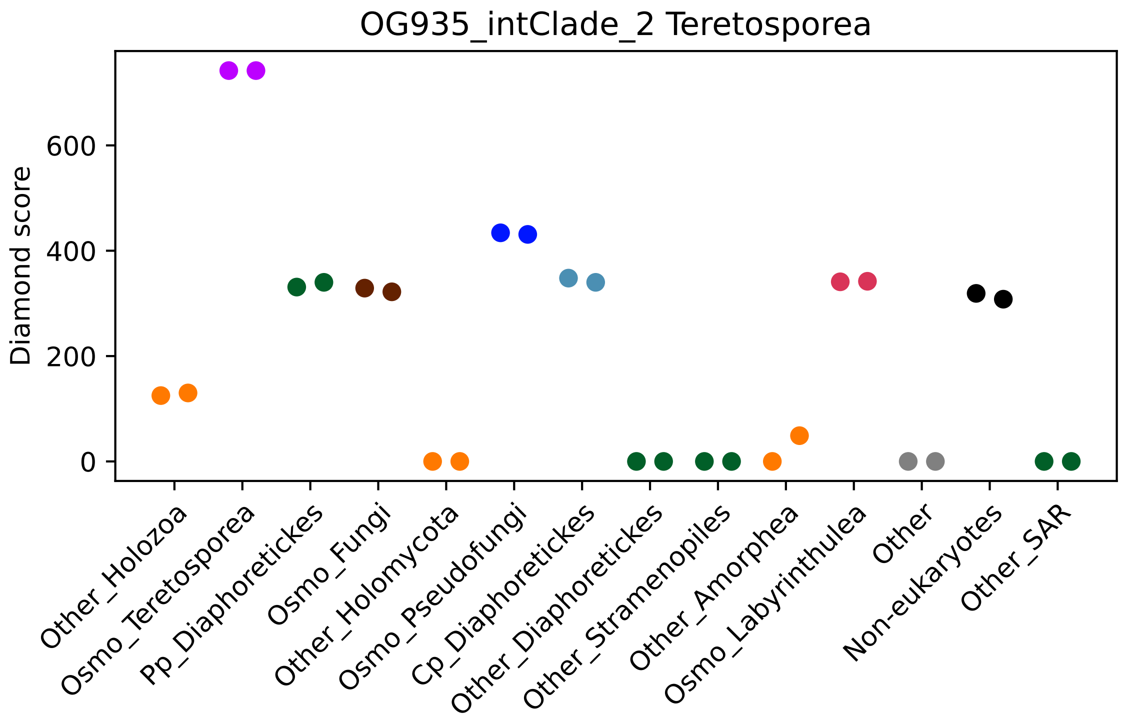

OG935_intClade_2 Teretosporea
Diamond score
0
200
400
600
Other_Holozoa
Osmo_Teretosporea
Pp_Diaphoretickes
Osmo_Fungi
Other_Holomycota
Osmo_Pseudofungi
Cp_Diaphoretickes
Other_Diaphoretickes
Other_Stramenopiles
Other_Amorphea
Osmo_Labyrinthulea
Other
Non-eukaryotes
Other_SAR

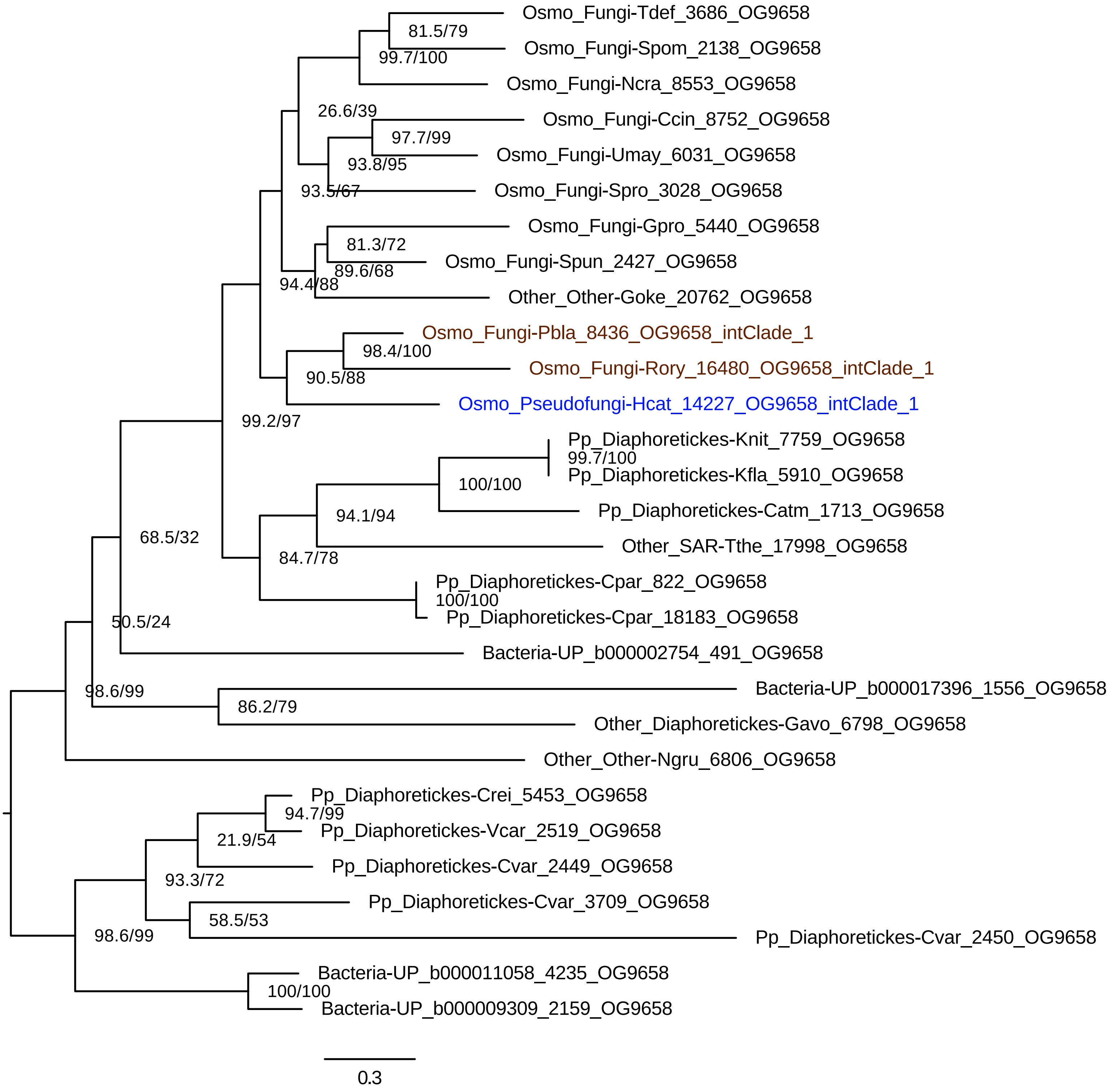

Osmo_Fungi-Tdef_3686_OG9658
81.5/79
Osmo_Fungi-Spom_2138_OG9658
99.7/100
Osmo_Fungi-Ncra_8553_OG9658
26.6/39
Osmo_Fungi-Ccin_8752_OG9658
97.7/99
Osmo_Fungi-Umay_6031_OG9658
93.8/95
93.5/67
Osmo_Fungi-Spro_3028_OG9658
Osmo_Fungi-Gpro_5440_OG9658
81.3/72
Osmo_Fungi-Spun_2427_OG9658
89.6/68
94.4/88
Other_Other-Goke_20762_OG9658
Osmo_Fungi-Pbla_8436_OG9658_intClade_1
98.4/100
Osmo_Fungi-Rory_16480_OG9658_intClade_1
90.5/88
Osmo_Pseudofungi-Hcat_14227_OG9658_intClade_1
99.2/97
Pp_Diaphoretickes-Knit_7759_OG9658
99.7/100
Pp_Diaphoretickes-Kfla_5910_OG9658
100/100
Pp_Diaphoretickes-Catm_1713_OG9658
94.1/94
68.5/32
Other_SAR-Tthe_17998_OG9658
84.7/78
Pp_Diaphoretickes-Cpar_822_OG9658
100/100
Pp_Diaphoretickes-Cpar_18183_OG9658
50.5/24
Bacteria-UP_b000002754_491_OG9658
98.6/99
Bacteria-UP_b000017396_1556_OG9658
86.2/79
Other_Diaphoretickes-Gavo_6798_OG9658
Other_Other-Ngru_6806_OG9658
Pp_Diaphoretickes-Crei_5453_OG9658
94.7/99
Pp_Diaphoretickes-Vcar_2519_OG9658
21.9/54
Pp_Diaphoretickes-Cvar_2449_OG9658
93.3/72
Pp_Diaphoretickes-Cvar_3709_OG9658
58.5/53
98.6/99
Pp_Diaphoretickes-Cvar_2450_OG9658
Bacteria-UP_b000011058_4235_OG9658
100/100
Bacteria-UP_b000009309_2159_OG9658
0.3

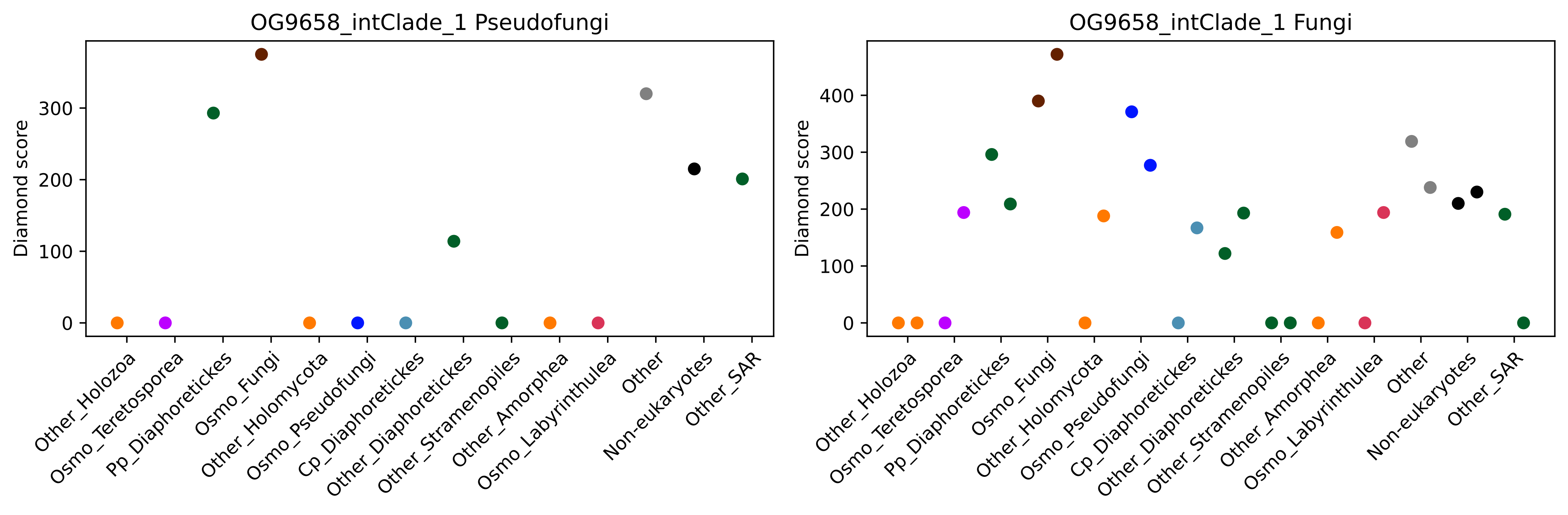
OG9658_intClade_1 Pseudofungi
OG9658_intClade_1 Fungi
Diamond score
0
100
200
300
400
Other_Holozoa
Osmo_Teretosporea
Pp_Diaphoretickes
Osmo_Fungi
Other_Holomycota
Osmo_Pseudofungi
Cp_Diaphoretickes
Other_Diaphoretickes
Other_Stramenopiles
Other_Amorphea
Osmo_Labyrinthulea
Other
Non-eukaryotes
Other_SAR

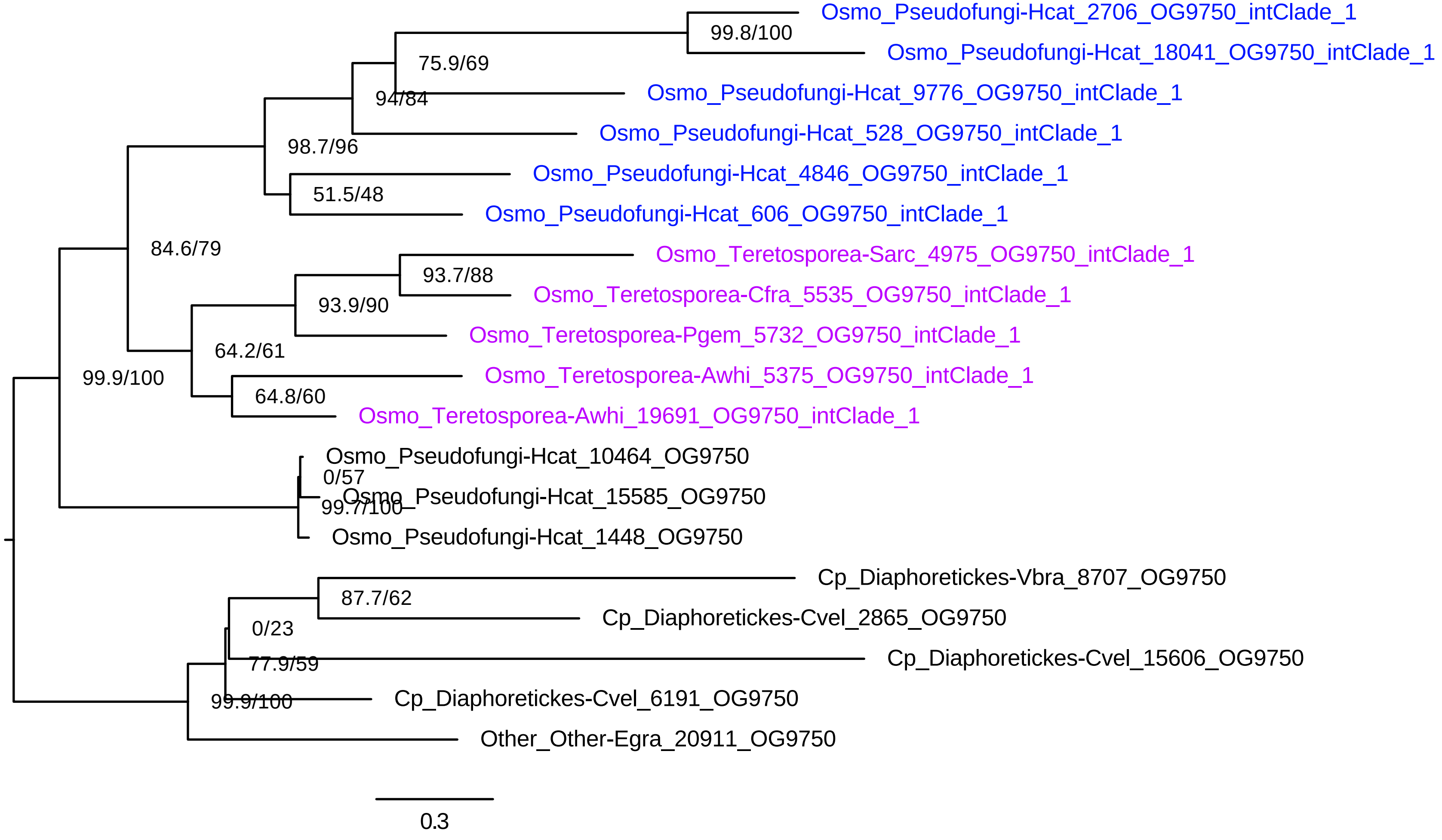
Osmo_Pseudofungi-Hcat_2706_OG9750_intClade_1
99.8/100
Osmo_Pseudofungi-Hcat_18041_OG9750_intClade_1
75.9/69
Osmo_Pseudofungi-Hcat_9776_OG9750_intClade_1
94/84
Osmo_Pseudofungi-Hcat_528_OG9750_intClade_1
98.7/96
Osmo_Pseudofungi-Hcat_4846_OG9750_intClade_1
51.5/48
Osmo_Pseudofungi-Hcat_606_OG9750_intClade_1
84.6/79
Osmo_Teretosporea-Sarc_4975_OG9750_intClade_1
93.7/88
Osmo_Teretosporea-Cfra_5535_OG9750_intClade_1
93.9/90
Osmo_Teretosporea-Pgem_5732_OG9750_intClade_1
64.2/61
Osmo_Teretosporea-Awhi_5375_OG9750_intClade_1
99.9/100
64.8/60
Osmo_Teretosporea-Awhi_19691_OG9750_intClade_1
Osmo_Pseudofungi-Hcat_10464_OG9750
0/57
Osmo_Pseudofungi-Hcat_15585_OG9750
99.7/100
Osmo_Pseudofungi-Hcat_1448_OG9750
Cp_Diaphoretickes-Vbra_8707_OG9750
87.7/62
Cp_Diaphoretickes-Cvel_2865_OG9750
0/23
Cp_Diaphoretickes-Cvel_15606_OG9750
77.9/59
Cp_Diaphoretickes-Cvel_6191_OG9750
99.9/100
Other_Other-Egra_20911_OG9750
0.3

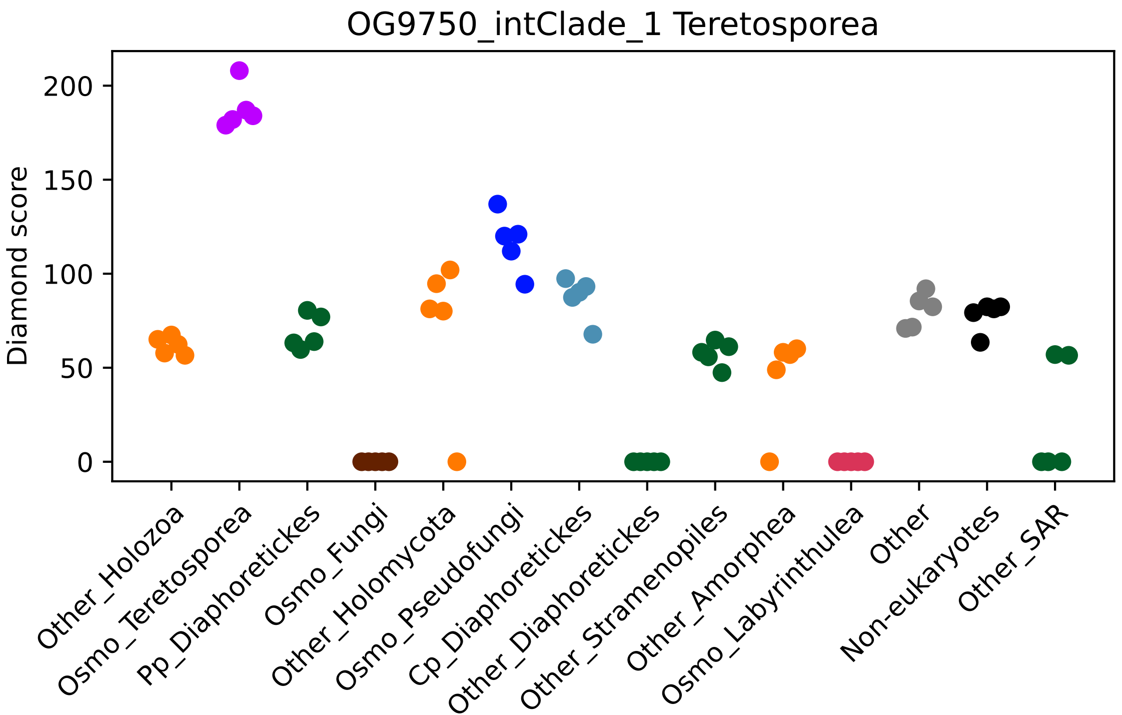
OG9750_intClade_1 Teretosporea
Diamond score
0
50
100
150
200
Other_Holozoa
Osmo_Teretosporea
Pp_Diaphoretickes
Osmo_Fungi
Other_Holomycota
Osmo_Pseudofungi
Cp_Diaphoretickes
Other_Diaphoretickes
Other_Stramenopiles
Other_Amorphea
Osmo_Labyrinthulea
Other
Non-eukaryotes
Other_SAR

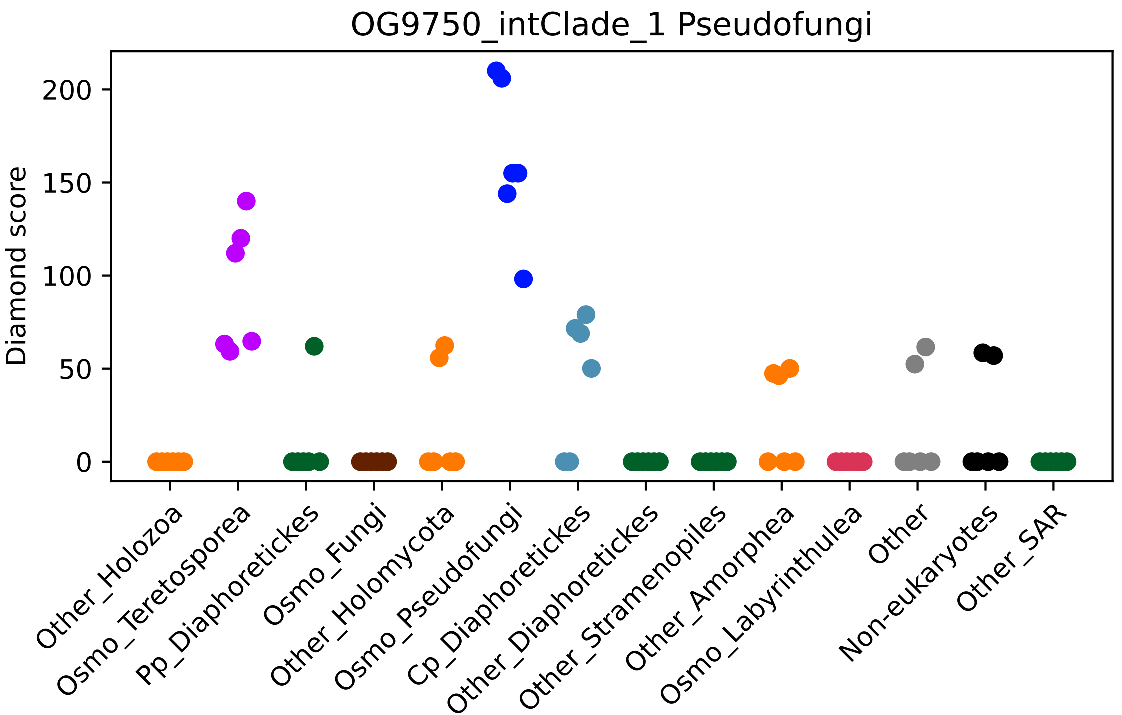
OG9750_intClade_1 Pseudofungi
Diamond score
0
50
100
150
200
Other_Holozoa
Osmo_Teretosporea
Pp_Diaphoretickes
Osmo_Fungi
Other_Holomycota
Osmo_Pseudofungi
Cp_Diaphoretickes
Other_Diaphoretickes
Other_Stramenopiles
Other_Amorphea
Osmo_Labyrinthulea
Other
Non-eukaryotes
Other_SAR

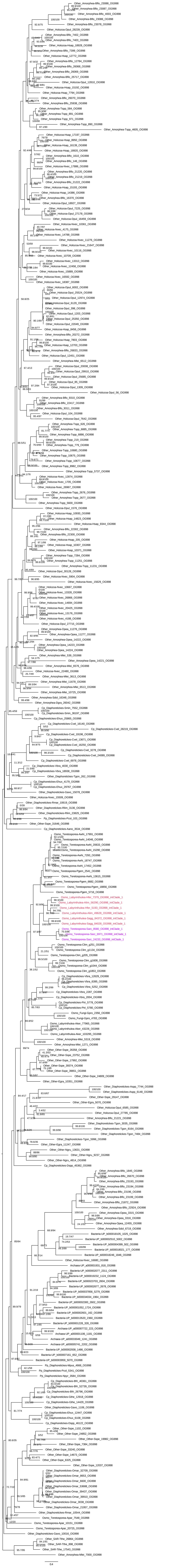

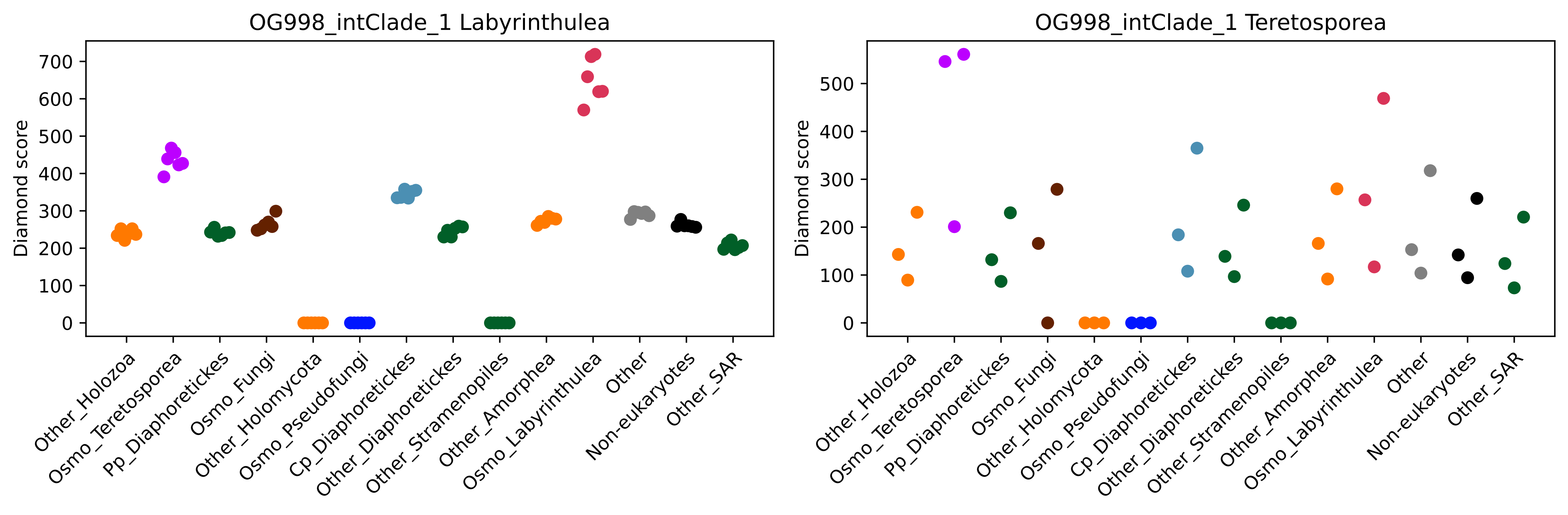

OG998_intClade_1 Labyrinthulea
Diamond score
0
100
200
300
400
500
600
700
Other_Holozoa
Osmo_Teretosporea
Pp_Diaphoretickes
Osmo_Fungi
Other_Holomycota
Osmo_Pseudofungi
Cp_Diaphoretickes
Other_Diaphoretickes
Other_Stramenopiles
Other_Amorphea
Osmo_Labyrinthulea
Other
Non-eukaryotes
Other_SAR
OG998_intClade_1 Teretosporea
Diamond score
0
100
200
300
400
500
Other_Holozoa
Osmo_Teretosporea
Pp_Diaphoretickes
Osmo_Fungi
Other_Holomycota
Osmo_Pseudofungi
Cp_Diaphoretickes
Other_Diaphoretickes
Other_Stramenopiles
Other_Amorphea
Osmo_Labyrinthulea
Other
Non-eukaryotes
Other_SAR
