# Supplementary material for: Signatures of gene transfer in the parallel evolution of osmotrophic specialization in eukaryotes
Source: Nat Ecol Evol. 2026 May 25;10(7):1342–55. doi: 10.1038/s41559-026-03054-w (PMC13345962; doi:10.1038/s41559-026-03054-w)
Supplement: Supplementary file 3 — Peer Review File [file 41559_2026_3054_MOESM3_ESM.pdf]

nature portfolio

Peer Review File

On the convergent origins of osmotrophic specialisation in eukaryotes

Corresponding Author: Dr Eduard Ocana-Pallares

Version 0:

Decision Letter:

20th August 2025

*Please ensure you delete the link to your author homepage in this e-mail if you wish to forward it to your co-authors.

Dear Edu,

Your Article, "Reticulate evolution and the rise of trophic complexity in eukaryotes" has now been seen by 3 reviewers. You will see from their comments copied below that while they find your work of considerable potential interest, they have raised quite substantial concerns that must be addressed. In light of these comments, we cannot accept the manuscript for publication, but would be very interested in considering a revised version that addresses these serious concerns.

In particular, Reviewer #1 has noted three major reservations related to the eukaryote phylogeny, limited taxon sampling, and reliability of inferring HGT events. Some of these thoughts are echoed in Reviewer #3's comments. We are happy to consider a revised version that addresses *all* issues raised by these reviewers, but I should note that we will aim to receive sign-off from Reviewer #1 before offering publication of the study.

We hope you will find the reviewers' comments useful as you decide how to proceed. If you wish to submit a substantially revised manuscript, please bear in mind that we will be reluctant to approach the reviewers again in the absence of major revisions.

If you choose to revise your manuscript taking into account all reviewer and editor comments, please highlight all changes in the manuscript text file [OPTIONAL: in Microsoft Word format].

We are committed to providing a fair and constructive peer-review process. Please do not hesitate to contact us if there are specific requests from the reviewers that you believe are technically impossible or unlikely to yield a meaningful outcome.

If revising your manuscript:

* Include a “Response to reviewers” document detailing, point-by-point, how you addressed each referee comment. If no action was taken to address a point, you must provide a compelling argument. This response will be sent back to the referees along with the revised manuscript.

* If you have not done so already we suggest that you begin to revise your manuscript so that it conforms to our Article format instructions at http://www.nature.com/natecolevol/info/final-submission. Refer also to any guidelines provided in this letter.

* Include a revised version of any required reporting checklist. It will be available to referees (and, potentially, statisticians) to aid in their evaluation if the manuscript goes back for peer review. A revised checklist is essential for re-review of the paper.

Please use the link below to submit a revised paper:

Link Redacted

Note: This URL links to your confidential home page and associated information about manuscripts you may have submitted, or that you are reviewing for us. If you wish to forward this email to co-authors, please delete the link to your homepage.

If you wish to submit a suitably revised manuscript we would hope to receive it within 6 months. If you cannot send it within

this time, please let us know. We will be happy to consider your revision so long as nothing similar has been accepted for publication at Nature Ecology & Evolution or published elsewhere.

Nature Ecology & Evolution is committed to improving transparency in authorship. As part of our efforts in this direction, we are now requesting that all authors identified as ‘corresponding author’ on published papers create and link their Open Researcher and Contributor Identifier (ORCID) with their account on the Manuscript Tracking System (MTS), prior to acceptance. This applies to primary research papers only. ORCID helps the scientific community achieve unambiguous attribution of all scholarly contributions. You can create and link your ORCID from the home page of the MTS by clicking on ‘Modify my Springer Nature account’. For more information please visit please visit www.springernature.com/orcid.

Please do not hesitate to contact me if you have any questions or would like to discuss the required revisions further.

Thank you for the opportunity to review your work.

[redacted]

Reviewer expertise:

Reviewer #1: microbial eukaryotes, phylogenomics

Reviewer #2: phylogenomics of eukaryotes, HGT

Reviewer #3: microbial eukaryotes, organelle evolution

Reviewers’ comments:

Reviewer #1 (Remarks to the Author):

Review of “Reticulate evolution and the rise of trophic complexity in eukaryotes”

This manuscript seeks to elucidate the evolutionary history of osmotrophy among eukaryotes. Using a set of phylogenomic methods, the authors investigate the toolkit used by a diversity of osmotrophic lineages, and also date the timing of the acquisition of these genes using molecular clock approaches. While I agree that this topic is one that has yet to receive sufficient study, I have numerous concerns with the manuscript as written and I see several important limitations to this study

**Inferences are based on a tree topology that is controversial.
The manuscript fails to discuss uncertainties in the topology of the eukaryotic tree of life, and particularly in the order of early events and the root. Instead the current paper, discussion of topology (e.g. lines 66-69, Fig 1a) relies on a single, controversial topology of the eukaryotic tree of life. Given that the root is as yet unknown, the inferences about order of events in evolution of osmotrophy are much more uncertain than presented here.

Specifically, the authors assume the validity of taxonomic groupings including Diaphoretickes and Amorphea, which may or may not be robust. This problem is perhaps articulated in a recent paper from another group in which they acknowledge: “We arbitrarily rooted our trees using the excavate clade Metamonada, including the relatively short branching Trimastix and Paratrimastix species (Figure 1). “ Torruella et al (2024) (https://pubmed.ncbi.nlm.nih.gov/39642877/). If the root is unknown, the validity of higher level taxa is questionable and hence the interpretation of osmotrophy requires more nuanced discussion.

Similarly, the manuscript asserts: “Our dataset does not include data from species from outside the Amorphea and Diaphoretickes divisions of eukaryotes. This is because these major eukaryotic divisions encompass all four osmogroups” - yet this interpretation again assumes the validity of higher level clades and a known location of the root. This logical framework also excludes the possibility that some genes have been coopted from other metabolisms. For this latter point, the authors might (re)read the following on misunderstandings of the “meiotic toolkit’ as many genes involved in meiosis have other functions:
https://pubmed.ncbi.nlm.nih.gov/31667871/

In sum, a more nuanced understanding of the eukaryotic tree of life is required to interpret the data presented here.

**Taxon sampling is very limited, and this is problematic in several ways:
The study relies on data from only 86 lineages! This is problematic.

Limited taxon sampling is likely to have a substantial impact on the inferences of ‘osmoclusters’ (e.g. Fig 2a and associated discussion). The idea of ‘background clusters’ rests on the diversity of non-osmotrophs, and many many lineages of eukaryotes are undersampled. Similarly, interpreting individual gene trees and timing of expansion of osmoclusters all

depends on taxon sampling. The reliance on such a limited set of species is surprising here given the wealth of data that have emerged on protists in the past decades.

Interpretation of osmotrophy as a distinct character is confounded by the diversity of trophic forms among eukaryotes, including mixotrophy - and many mixotrophic/phototrophic lineages are not sampled. Not sampling key lineages will decrease the power of the study, and weaken the interpretations.

The interpretation of the clock here is also problematic given the limited number of taxa sampled. Moreover, the manuscript would be improved with a greater discussion of the other events that likely occurred during the focal time period of 800-1000mya. Here the paper misses key articles such as:

https://pubmed.ncbi.nlm.nih.gov/37713454/
https://pubmed.ncbi.nlm.nih.gov/34949483/

**The work largely ignores known problems in interpreting horizontal gene transfer events.
It is surprising to see so little mention of caveats and cautions for interpreting HGTs. As documented elsewhere, interpretation of single gene trees requires considerable more finesse to account for gene loss and unstable tree topologies. Addressing these issues can readily be done by including more taxa and exploring alternative topologies (e.g. AU test). Moreover, greater acknowledgement of uncertainties and past literature would strengthen the manuscript.

Some references here:
https://pubmed.ncbi.nlm.nih.gov/33012528/
https://pubmed.ncbi.nlm.nih.gov/35731825/
https://pubmed.ncbi.nlm.nih.gov/40402828/
https://pubmed.ncbi.nlm.nih.gov/38263430/

Other issues
The logical flow of the last sentence of the abstract could be improved.

I am unconvinced that all osmotrophs are decomposers… many parasites are osmotrophs (e.g. tape worms)

The claim that 'canonical phagotrophy is also absent' (line 91) in osmotrophs seems a bit circular given definitions here

Similarly, the association of "multinucleated coenocytic/syncytial development" with osmotrophy misses that these features are also widespread among photosynthetic 'algae' and in many phagotrophic amoeboid groups

The logic here is unclear "Osmotrophy specialisation must require changes in gene content" (line 102) – this could be an exaptation, or a simple case of gene loss

Perhaps 'tone down' quotes from past literature as at times they feel forced and they may not be widely accessible. Here is just one example where meaning was unclear:
-If we had to replay life's tape as imagined by Stephen Jay Gould , but constraining eukaryotic evolution to a fully unidimensional process, with no reticulations, would major groups of eukaryotes that specialise in osmotrophy and photoautotrophy repeatedly evolved?

The quality of figures could be improved to make images more easily readable

The brevity of the discussion is surprising, and incorporating more literature (including those linked above) would strengthen the manuscript

Reviewer #2 (Remarks to the Author):

The authors examine the contribution of reticulate evolutionary trends in trophic variation across eukaryotes. They focus on "osmogroups" - four groups of eukaryotes specialized in osmotrophy on either side of the Diaphoretickes/Amorphea split. First, the authors performed molecular clock dating analysis to establish a timeframe for the evolution of eukaryotic osmotrophy between 800-1000Ma. Next, the authors identified 172 "osmoclusters" - gene clusters encompassing >3 osmogroups that likely evolved convergently on either side of the eukaryote split. Metabolic and enzymatic functions are over-represented in these osmoclusters, as well processes related to osmoregulation. The osmoclusters have a deep origin in eukaryotes, but the greatest diversity and copy number is seen from the origin of each osmogroup to the present. Using reconciliation analysis, the authors find that two waves of HGT of osmoclusters occurred: first from non-osmogroups to osmogroups during early osmogroup evolution, and later between osmogroups as they diversified. Other evidence of HGT was also observed separately between Fungi and Pseudofungi and between the two marine osmogroups, consistent with

previous findings of HGT between Fungi and Pseduofungi (particularly in oomycetes). These HGT events concern genes involved in metabolic processes.

Overall, this is a rigorous study of the evolutionary origins of osmotrophy in eukaryotes. The authors propose that reticulate evolution in the form of HGT shaped the evolution of osmotrophy similar to how endosymbiosis shaped the evolution of photoautotrophy. I think this is a reasonable assessment, given that many osmogroups arose at similar dates and at one point inhabited similar niches as evidenced in the fossil record. I agree with the authors' statement in the Discussion that reticulate evolution of trophic specialization across unrelated taxa is a more parsimonious explanation than de novo evolution for traits that are present in multiple eukaryotic lineages but are not ancestral to eukaryotes.

I think this study would be acceptable for publication pending minor alterations below.

Abstract Line 24 and Discussion Line 318: I would say "have received".

Main Text Line 46-51: I don't think it's correct at all to state that RE is a process by which species or branches of a phylogeny can "merge" either by endosymbiosis or HGT. This is a very informal description of its outcomes, at best, and this section should be revised.

Reviewer #3 (Remarks to the Author):

In this work, the authors identify genes that are enriched in the four large known clades of eukaryotic osmotrophs: fungi, teretosporeans, labyrinthulids, and oomycetes (pseudofungi). They then explore the origin of those genes and show that a considerable proportion of them were acquired by horizontal gene transfer (HGT), either from other osmotrophic eukaryotes (notably fungi) or, ultimately, from bacteria. They also apply molecular dating approaches to date the origin of those eukaryotic clades. The authors have done an impressive amount of work, providing additional and extensive support to the idea that those lineages of osmotrophic eukaryotes adapted to that nutrition mode by importing genes from fungi and, originally, from bacteria. However, as it appears now, the manuscript is obscured by confusion in some concepts and names, lack of linearity in argumentation, and the mixed nature of different sections. I think this is a valuable piece of work, but the manuscript needs considerable rewriting and streamlining to present clear hypotheses, results, and interpretation in a connected, linear manner.

My major comments are as follows:

- Some parts of this manuscript appear to be written for a PhD thesis rather than a research article. For instance, the introduction discusses some generalities about the importance of endosymbiosis but does not present the specific questions and hypotheses actually addressed in the present research. These are introduced along the way, sometimes in the methods section (e.g. line 413 and subsequent).

- Part of this confusion stems from the fact that, although the authors present and discuss data pertaining to osmotrophic lineages, they do not carry out a thorough comparison with photoautotrophic lineages. These are alluded to in different sections of the manuscript, but in an ambiguous and not very direct manner. Yet, many of the genes (orthoclusters) enriched in osmotrophs are also present or enriched in photoautotrophs. There is a (logical) hypothesis in the methods section about why this is so, but it is not properly addressed or commented on in the main text. At the same time, if the same genes are enriched in osmotrophs and photoautotrophs, can these genes be considered an "osmotrophic toolkit"? Furthermore, "osmoclusters" appear highly enriched in some photosynthetic lineages, notably ochrophytes (cf. Fig. 3A), but not in others (Archaeplastida). How can one differentiate between function and phylogeny as drivers of the observed patterns? Those genes may have been acquired at the base of stramenopiles and then inherited by ochrophytes, not because of their role in osmotrophy/phototrophy, but because they are neighboring groups. I feel that either there should be a full discussion about this, with comparative data and tests supporting the authors' ideas, or—perhaps more practically—the manuscript should focus on the phylogenetic side, i.e., on the acquisition of genes by HGT at the onset of those different osmotrophic lineages, which may have enhanced or facilitated the adaptation to osmotrophy, without necessarily being exclusive or diagnostic for that nutritional mode.

- The use of the term "osmogroup" (meaning osmotrophic eukaryotic groups) is extremely confusing and unnecessary. I recommend that the authors refer to these clades of eukaryotes as "osmotrophic eukaryotes/groups/lineages/clades." The root "troph," which is a key concept in the whole discussion, is lost, so the term alone does not convey the idea of osmotrophy. Furthermore, "osmogroup" (referring to organisms) can be easily confused with "osmoclusters" (referring to genes). This confusion is worsened by the existing term "orthogroups" (OGs – genes), which is used to name and number the osmoclusters. Please find a simple and unambiguous way of naming things. Sometimes, not having an acronym is better

- Some concepts are presented in a way that denotes misunderstanding or could lead to misunderstanding. For instance, in the abstract, osmotrophs are equated with decomposers. While osmotrophs are (generally) decomposers, the opposite is not necessarily true. Many decomposers are, for example, insects or worms, which degrade organic matter by phagocytosing it. Even the title of the article is a bit misleading in this sense: Reticulate evolution and the rise of trophic complexity. Do the authors consider osmotrophy a trophic mode of higher complexity? Arguably, phagotrophy—involving a developed cytoskeleton, membrane remodeling, and (also) degradation enzymes—can be considered more complex. True, that argument could potentially be applied to photosynthesis as well, but the results of this manuscript do not address that question. I would suggest focusing on osmotrophy for the sake of clarity.

- Whittaker – please, drop the reference to his work. Many ecologists and protistologists have rightfully discussed nutrition modes. However, despite the focus on nutrition, Whittaker's view on evolution was based on the assumption that evolution always proceeds towards increasing complexity and that simple means primitive (animals, plants and fungi being on top of all unicellular organisms). His evolutionary scheme was wrong, as demonstrated by molecular phylogeny. For instance, according to Figure 1 in Whittaker's cited paper, fungi derive directly from bacteria. Accordingly, their nutrition mode would directly derive from bacterial osmotrophy (vertical inheritance). Just the opposite of what you are saying, i.e. that fungi evolved from phagotrophic organisms and acquired genes from bacteria (horizontally) to convergently adapt to osmotrophy.

- Bacteria are alluded as gene donors. However, they are a black box referred as "non-eukaryotic donors". Which are the bacterial donors? This information is missing or so hidden that it is impossible to retrieve. Yet, this might provide interesting information. Many bacteria are osmotrophs, but not all. For instance, cyanobacteria (photosynthesizers) are known to have exchanged genes with fungi. Is there any preferential donor? It is a pity that a discussion on the prokaryotic donors of osmotrophy-related genes is completely absent

- Dating. This is not uninteresting, but potentially this part would rather benefit from being introduced later, close to the discussion.

- Reconciliation – many gene trees are poorly resolved. How to discriminate between poor signal and real HGT? A well-known problem deserving a discussion. How many "false positives"?

Version 1:

Decision Letter:

23rd January 2026

Dear Dr. Ocana-Pallares,

Thank you for submitting your revised manuscript "Reticulate evolution facilitated convergence towards specialised trophic modes across eukaryotes" (NATECOLEVOL-25051720A). It has now been seen again by the original reviewers and their comments are below. The reviewers find that the paper has improved in revision, and therefore we'll be happy in principle to publish it in Nature Ecology & Evolution, pending minor revisions to satisfy the reviewers' final requests and to comply with our editorial and formatting guidelines.

If the current version of your manuscript is in a PDF format, please email us a copy of the file in an editable format (Microsoft Word or LaTex)-- we can not proceed with PDFs at this stage.

If you have not done so already, please ensure that you also email us a completed copy of the Reporting summary :

Reporting summary: https://www.nature.com/documents/nr-reporting-summary.pdf

We are now performing detailed checks on your paper and will send you a checklist detailing our editorial and formatting requirements in about a week. Please do not upload the final materials or make any revisions until you receive this additional information from us.

Thank you again for your interest in Nature Ecology & Evolution. Please do not hesitate to contact me if you have any questions.

[redacted]

Reviewer #1 (Remarks to the Author):

Review of "Reticulate evolution and the rise of trophic complexity in eukaryotes"

This is a revision of a manuscript that seeks to elucidate the evolutionary history of osmotrophy among eukaryotes. Using a set of phylogenomic methods, the authors investigate the toolkit used by a diversity of osmotrophic lineages, and also date the timing of the acquisition of these genes using molecular clock approaches. I agree that this topic is one that has yet to receive sufficient study, and I am impressed by the extensive revisions in light of the first round of review. However, though the current manuscript is much improved, some important points still need to be addressed.

Defining shared clusters: I am a bit confused by the discussion of the clusters (lines 154-163), and I think that this can be addressed by rewriting here. In order for a gene family to be 'convergent adaptations', they need to be in at least 3 of the 4 osmotrophs AND absent for non-osmotrophs. (Obviously, widespread presence in osmotrophs and non-osmotrophs is consistent with the gene family being present in the last common ancestor of both groups). The second paragraph in this section mentions 'background clusters'- perhaps these might be described early, and perhaps renamed (e.g. 'ancestral

clusters', or at least the authors need to briefly defining what is meant by 'background'). I also think this section (and the entire manuscript) would be improved by acknowledging the possibility of gene loss contributing to the patterns for at least some gene families (see below).

Alternative hypotheses: The section entitled "Horizontal gene transfers in osmoclusters" certainly needs some discussion of gene loss, especially given reliance on data visualization for some inferences. A similar caveat deserves mention in the next section as well. And again in lines 474-483. And in the section on "Manually supervised identification of osmoclusters with putative HGT patterns" starting on line 774.

Writing:Overall, the writing could be improved a bit further to clarify logical flow. As just some examples, the inferences about Tonian origins could be removed from results – many/most readers won't know much (anything?) about the Tonian, and so perhaps this is best only in the discussion where greater context can be provided. Similarly, there are numerous overly-long paragraphs and other opportunities to clarify text (see examples below). The paragraph in the discussion from 425-444 seems to repeat too much of the methods/results.

This sentence seems highly speculative and could do with a bit of refining: "Since eukaryotes are usually larger than prokaryotes and also more prone to evolve multicellular forms, a progressive increase in the relative representation of eukaryotes during the Tonian may have predisposed the evolution of specialised osmotrophic forms that could exploit more efficiently the gradually increasing availability of polymerised and structured organic matter and associated macroscopic forms." (of course, cyanobacteria and bacterial biofilms can also get quite large). In fact, the language/logic of the rest of this paragraph would benefit from some revision (lines 390-408)

Figures:
Though improved over the last version, more could be done on the main figures.

Figure 1: The organismal images in figure 1 are disappointing: not particularly clear, no scale bars (this is unacceptable), and you have the opportunity to teach a bit about these poorly-known lineages. Please rethink. Also, the legend is inaccurate (no phylogeny in this version) and the description of figure 1B is likely not accessible to a naive reader. I suggest a complete rewrite of this legend (and I wonder if this is in part the result of putting time into writing a very long rebuttal that repeats details in revision – perhaps better to use some time in improving the main document).

Figure 3: rethink colours - the brown is hard to discern.

Figure 5: the number of events in 5A is difficult (impossible?) to discern. I suggest moving to something like numbers in circles, and perhaps moving 5B to supplement to make this important part of the clearer. In fact if 5A was bigger (maybe not a circle?), you could do more to teach the diversity of organisms, which would help broad readership.

Some minor comments
On language, the following seems a bit odd: "proved attractive" when referring to Whitaker (line 38). Where is the 'proving' demonstrated? In fact, I would rethink this first paragraph as it seems written for an in-crowd.

Perhaps define "reticulate evolution" briefly (in parentheses) after first use of this term (line 53). Similarly, some slightly more elaborated explanation of osmotrophy might be good early on. As is, the intro defines just as 'absorption (osmotrophy)', yet I imagine that phototrophs and heterotrophs still absorb some things?

I might also provide a bit more definition of these terms for non-specialists: Teretosporea, Pseudofungi and Labyrinthulea. And then start a new paragraph in line 7

Rethink the use of the word 'host' (line 112)

The paragraph on page 7 is quite long, perhaps break somewhere to improve logical flow. In fact, look for too-long paragraphs throughout to improve writing/logic.

In legend, mention that coloured branches in figure 3A are osmotrophs. Perhaps rethink these colors though as the brown and grey look very similar, particularly in figure 3C!

Line 357 - overrepresented compared to what? What is null here?

Line 377 - teach something about the Tonian, please - in just a short phrase. discussion is your opportunity to provide context for naive reader.

In rebuttal – another exam of poor writing: I am not sure what 'fairly unrelated' means.

Reviewer #3 (Remarks to the Author):

The authors have made a considerable effort to clarify the manuscript and enhance its readability. It is very much appreciated. They have responded to most of my questions and I recommend publication.

A few minor points:

- Please substitute the “don’t” by “do not” along the text.

- Line 210, “phagocytised”, phagocytosed?

- Lines 474-478: “In the absence of reticulations, would photosynthetic organisms or lineages specialised into osmotrophy have repeatedly evolved in eukaryotes? The fact that endosymbiosis is behind the origin of photosynthetic eukaryotes indicates that it is at least much easier to become a photosynthetic organism by means of species-level reticulations than by de novo evolving the photosynthetic machinery”. I still do not think that the two processes, eukaryotic photosynthesis and osmotrophy, are fully comparable. There is horizontal gene transfer in the evolution of both, but in the case of photosynthesis, transferred genes are not enough. Photosynthesis is still carried out in the old cyanobacterium that has become an organelle (the plastid). Nothing of the kind happened in osmotrophs. I hardly doubt that if you transferred all photosynthetic genes to a eukaryotic nucleus, the resulting eukaryotic cell could perform photosynthesis. You need a different compartment, and that historically happened by the internalization of another cell. This is a very different case, with limited analogy to the evolution of osmotrophy.

Open Access This Peer Review File is licensed under a Creative Commons Attribution 4.0 International License, which permits use, sharing, adaptation, distribution and reproduction in any medium or format, as long as you give appropriate credit to the original author(s) and the source, provide a link to the Creative Commons license, and indicate if changes were made.
In cases where reviewers are anonymous, credit should be given to 'Anonymous Referee' and the source.
The images or other third party material in this Peer Review File are included in the article’s Creative Commons license, unless indicated otherwise in a credit line to the material. If material is not included in the article’s Creative Commons license and your intended use is not permitted by statutory regulation or exceeds the permitted use, you will need to obtain permission directly from the copyright holder.
To view a copy of this license, visit https://creativecommons.org/licenses/by/4.0/

Reviewer expertise:

Reviewer #1: microbial eukaryotes, phylogenomics

Reviewer #2: phylogenomics of eukaryotes, HGT

Reviewer #3: microbial eukaryotes, organelle evolution

Reviewers' comments:

Reviewer #1 (Remarks to the Author):

Review of "Reticulate evolution and the rise of trophic complexity in eukaryotes"

This manuscript seeks to elucidate the evolutionary history of osmotrophy among eukaryotes. Using a set of phylogenomic methods, the authors investigate the toolkit used by a diversity of osmotrophic lineages, and also date the timing of the acquisition of these genes using molecular clock approaches. While I agree that this topic is one that has yet to receive sufficient study, I have numerous concerns with the manuscript as written and I see several important limitations to this study

We would like to thank the three reviewers for their insightful and constructive feedback. Their comments have been instrumental in strengthening the manuscript.

**Inferences are based on a tree topology that is controversial.

As a summary of what we will detail in the responses below, in response to Reviewer #1's concerns, we have validated our primary findings using an expanded dataset of 122 species. This expanded analysis confirms that the results that we reported are unlikely to have been driven by a species underrepresentation in our taxon sampling. Furthermore, to address topological uncertainty, our analyses were re-run under three alternative rooting scenarios for the eukaryotic phylogeny (Extended Data Figs. 3-4), all of which yielded consistent results.

In the following response points, we will provide separate answers to a series of Reviewer #1's concerns which are related to taxon sampling selection and the species topology used. To sum up, in the following responses, we will:

1) Provide a justification for the design of our original taxon sampling.
2) Provide an explanation on where the species tree corresponding to the original species sampling comes from.
3) Provide a justification on why the species tree for this original dataset can be rooted without uncertainty.
4) Explain how we have extended our original taxon sampling, in order to be able to test reviewer's concerns on lack of species representation.
5) Describe the results obtained under our re-analyses with an extended species sampling, which corroborated the results obtained with our original species selection.

The manuscript fails to discuss uncertainties in the topology of the eukaryotic tree of life, and particularly in the order of early events and the root. Instead the current paper, discussion of topology (e.g. lines 66-69, Fig 1a) relies on a single, controversial topology of the eukaryotic tree of life.

Here we provide a justification on where does the species tree employed for our original taxon sampling of 86 species comes from (discussion on whether there is uncertainty on the root for our phylogeny will be discussed in the next question point):

In the previous version of the manuscript, particularly in the Results section, we did not make sufficiently explicit the fact that the species topology that we used in our study has not been arbitrarily chosen. Instead, it corresponds to the most supported topology retrieved by performing a multigene concatenate-based phylogenetic inference (Methods section, lines 589-633 in MANUSCRIPT_clean.pdf). The maximum clade credibility tree recovered (Extended Data Fig. 5), which is the topology that we ended up selecting for our study (Fig. 3A), is highly consistent with the bibliography, either in the certainties (branches with posterior probabilities >95%) and in the few uncertainties retrieved (branches <95%).
We did a substantial effort to clarify some of the remaining topological uncertainties on the retrieved species topology. In particular, we performed gene tree-species tree reconciliations of all gene family clusters under a series of potential alternative topologies, each covering an alternative resolution for some of the poorly supported bipartitions, including a topological change within Basidiomycota, an alternative position for Haptophyta, and a topological change within Ochrophyta (Supplementary Table 9). Among the alternative tested topologies, the species tree that retrieved the best reconciliation likelihoods coincided with the maximum clade credibility tree recovered from the multigene concatenated analyses described above (Supplementary Table 9). We thus decided to use this topology for our analyses. We are now making a more explicit mention of these analyses in the Results section (lines 133-135), leaving the details for the Methods (lines 589-633).

Given that the root is as yet unknown, the inferences about order of events in evolution of osmotrophy are much more uncertain than presented here … Specifically, the authors assume the validity of taxonomic groupings including Diaphoretickes and Amorphea, which may or may not be robust. … This problem is perhaps articulated in a recent paper from another group in which they acknowledge: "We arbitrarily rooted our trees using the excavate clade Metamonada, including the relatively short branching Trimastix and Paratrimastix species (Figure 1). " Torruella et al (2024) (https://pubmed.ncbi.nlm.nih.gov/39642877/).

Here we provide a justification on how we rooted our species topology, based on the retrieved results and in concordance with the bibliography:

The species tree built recovered with full support (Extended Data Fig. 5) the clades including, on the one hand, Amoebozoa, Apusozoa, Holozoa and Holomycota (a clade which we referred to as Amorphea), and on the other hand, the clade including Stramenopiles, Alveolata, Rhizaria, Archaeplastida, Cryptophyta and Haptophyta (a clade which we referred to as Diaphoretickes). We referred to these clades as Amorphea and Diaphoretickes

because both terms are employed in the literature to describe the clades representing the last common ancestors of the taxonomic groups included in these two clades (e.g., Fig. 1 in https://doi.org/10.1111/jeu.12691 or Fig. 3 in https://doi.org/10.1016/j.cub.2024.10.075). Given our current understanding of the eukaryotic phylogeny, and given that no one is proposing that the root of eukaryotes should be within the clades that we referred to as Amorphea or Diaphoretickes, and that the monophyly of these two clades is well supported in our analyses and in the literature (i.e., no phylogenies are showing taxa from one of the two clades branching with taxa from the other group in any recently published phylogeny; e.g., https://doi.org/10.1016/j.cub.2024.10.075), we decided to place the root of the species phylogeny reconstructed with our original taxon sampling between the clades we refer to as Amorphea and Diaphoretickes.

Here we provide a justification on why the exclusion of species which are fairly unrelated to the osmotrophic groups, and which also have uncertain phylogenetic placements, was done in purpose also to facilitate an un ambiguous rooting of our species phylogeny, and why we considered that this shouldn't penalize the accuracy of our results (although we have tested it by extending our taxon sampling, as we will describe below):

In the original taxon sampling employed in this study (see responses below for details on our re-analysis with an extended taxon sampling), the exclusion of eukaryotic groups from outside the clades which we referred to as Amorphea and Diaphoretickes was done on purpose. We have clarified this in the Methods (lines 495-518).
First, because groups such as Metamonada and Discoba are fairly unrelated to the osmotrophic groups, we expected that their incorporation will add little value for this study. Moreover many of them are diverged parasitic species which can be expected to be poorly informative for ancestral gene content reconstruction.
Second, because many of the eukaryotic species branching outside Amorphea and Diaphoretickes are represented by species datasets of poor quality (either because they come from contaminated transcriptomes, or correspond to transcriptomes with a lot of sequence redundancy; we will discuss this below).
Having provided these justifications, we agree with the reviewer on the need to confirm that the exclusion of taxa from these groups taxa in our original taxon sampling have not led to artifactual results. This has been addressed by re-analysing our findings with an extended taxon sampling, as we will detail below.

If the root is unknown, the validity of higher level taxa is questionable and hence the interpretation of osmotrophy requires more nuanced discussion. … Similarly, the manuscript asserts: "Our dataset does not include data from species from outside the Amorphea and Diaphoretickes divisions of eukaryotes. This is because these major eukaryotic divisions encompass all four osmogroups" - yet this interpretation again assumes the validity of higher level clades and a known location of the root. …

While we agree on the need of extending the taxon sampling to test whether lack of species representation could have biased our results, we don't consider that the ambiguity on the earliest splits of the eukaryotic tree challenges our interpretation of the evolutionary history of osmotrophy specialisation. First, because our original taxon sampling already includes multiple lineages branching between each of the four osmotrophic groups in the eukaryotic phylogeny (Fig. 3). Further, and as we will argue below, the results obtained from our

re-analyses with an extended taxon sampling are consistent independently on choice of the root (Extended Data Fig. 3).

… This logical framework also excludes the possibility that some genes have been coopted from other metabolisms. For this latter point, the authors might (re)read the following on misunderstandings of the "meiotic toolkit' as many genes involved in meiosis have other functions:
https://pubmed.ncbi.nlm.nih.gov/31667871/

We appreciate the reviewer's insight regarding the role of gene co-option. We have revised the manuscript to better balance the narrative between gene acquisition and the co-option of ancestral genes. By making these roles more explicit, we believe the revised text provides a more comprehensive view of the evolutionary path toward osmotrophy. We now address this at lines 248–251: "*Consistent with their presence in distantly related lineages, osmoclusters have a deep origin in the tree of life (Fig. 3A). This suggests an important role of gene co-option of ancestral genes that later became important for osmotrophic function in these groups*"

In sum, a more nuanced understanding of the eukaryotic tree of life is required to interpret the data presented here.
**Taxon sampling is very limited, and this is problematic in several ways:
The study relies on data from only 86 lineages! This is problematic.

Justification on why the original taxon sampling employed included 86 chosen species, and how we have extended it to explore reviewer's concerns:

a) Justification on the choice of the original taxon sapling:

We have removed a schematic and highly simplified version of the eukaryotic tree of life, previously displayed as Fig. 1A, because it was an oversimplification of the eukaryotic diversity represented by our taxon sampling, which has now been extended as well. Species included in the original and in the extended sampling are available in Supplementary Table 6, with phylogenies shown in Fig. 3A and Extended Data Figs. 3-4, respectively.

To clarify our criteria to design our original taxon sampling. For our original taxon sampling of 86 species, we incorporated as much good quality genome data as possible for the lineages that branch proximal to the four osmotrophic groups. Then, in order to connect these four groups so that we can reconstruct the gene content changes that occurred since the last common ancestor of these groups to the current representatives of the osmotrophic groups, we also populated the lineages branching between them, in a zoom-in/zoom-out approach, giving more sampling priority to those branches that are more phylogenetically proximal to these groups. While doing so, we prioritized including taxa from as many groups as possible (phylogenetic diversity+good quality data), and we only excluded taxa that have a clear uncertain branching within eukaryotes, but only if that taxa is highly phylogenetically distant to the osmotrophic groups (e.g., Metamonada and Discoba). A justification on our choice for selecting the taxa for the original taxon sampling is now in the first Methods section (lines 495-518.

Two important factors that guided our original taxon sampling design were 1) availability of good quality genome data and 2) expected computational load. We would like to note that a

large-scale reconstruction of gene family evolution in a taxon sampling of 86 species while performing HGT-aware gene tree-species tree reconciliation, species tree reconstruction, and relaxed molecular clock analyses with robust Bayesian methods and by employing site-heterogeneous phylogenetic models for the species reconstruction and datations steps, takes weeks/months to run. We aimed to balance dataset size (prioritizing only sufficient quality species proteomes) and the computational time required to run the analyses when setting up our original taxon sampling, always prioritising the employment of state-of-the-art methods in every methodological step.

b) Justification on the procedure to extend our taxon sampling:

Having justified the design of our original taxon sampling, we considered essential to explore Reviewer #1's concern in order to discard the possibility that our results may have been driven to some extent by a potential underrepresentation of species in our dataset. We thus decided to reproduce our analyses with an extended sampling that incorporates 36 additional species for a total of 122 taxa, as we detail below, and in Methods section 'Re-analyses with an extended dataset' (lines 737-773).

To extend our taxon sampling, we incorporated data from EukProt, in particular from "The Comparative Set" (196 species chosen based on BUSCO completion and phylogenetic importance) (https://evocellbio.com/eukprot/). For the decision on which species to incorporate from this set, we first discarded data with evidence of contamination https://github.com/edu3716/EukprotV3_TCS_saferSubset/tree/main. From the non-discarded data, we prioritised incorporating taxa for those groups that were either not represented, or that have lesser representation in the original taxon sampling. Previous to that, we also discarded transcriptomic data with BUSCO metrics suggesting elevated rates of missing and/or duplicated sequences, since we expected this to be problematic at multiple levels (gene copy underrepresentation/overrepresentation in the osmocluster analyses, deflated/inflated gene counts in the ancestral gene content reconstruction analyses, missing/redundant sequences for gene tree reconstruction, inflated duplication and gene loss rates in the gene tree-species tree reconciliation analyses, etc.).

The detailed list of the original and extended taxon samplings can be found in the updated Supplementary Table 6. To sum it up here, in the original taxon sampling, in relation to the eukaryotic clade which we refer to Amorphea, beyond the taxa from the two osmotrophic groups from this clade (Fungi and Teretosporea), we incorporated 3 metazoans, 2 choanoflagellates, 3 filastereans, 1 microsporidian, 1 rozellid, 2 nucleariids, 1 apusomonad, 2 amoebozoans. For this part of the tree, the extended taxon sampling now incorporates +1 extra amoebozoan, 1 breviate (previously unrepresented), +2 choanoflagellates and +4 metazoans. In relation to the clade which we refer to as Diaphoretickes, beyond the taxa from the two osmotrophic groups from this clade (Pseudofungi and Labyrinthulea), the original taxon sampling incorporated 4 alveolates, 3 chlorophytes, 2 Cryptophyceae, 3 embryophytes, 2 glaucophytes, 2 haptophytes, 4 ochrophytes, 3 rhizarians, 1 Rhodelphidia, 3 rhodophytes, 3 other stramenopiles and 2 other streptophytes. For this part of the tree, the extended taxon sampling now also incorporates +2 extra alveolates, 1 Centroplasthelida (previously unrepresented), +5 chlorophytes, +1 Cryptophyceae, +1 haptophyte, +1 ochrophyte, +2 rhizarians, +1 Rhodelphidia, +1 rhodophyte, +1 stramenopile, +2 streptophytes. Apart from the taxa from Amorphea and Diaphoretickes, in order to address the reviewer's concern on the absence of taxa from outside both divisions, the extended

sampling includes 6 representatives from Discoba, 2 from Metamonada, and 1 from Malawimonadida, all of which were previously unrepresented.

Our taxon sampling, particularly after the extension, covers a broad range of the currently known diversity of major groups across eukaryotes, putting particular emphasis in those lineages that branch proximal to the osmotrophic groups, but also covering those more distant groups for which genomic data of sufficient quality is available. We also want to emphasize that when including taxa from each eukaryotic group, we prioritised including species as phylogenetically distant to each other as possible in order to cover the maximum diversity within the group.

Limited taxon sampling is likely to have a substantial impact on the inferences of 'osmoclusters' (e.g. Fig 2a and associated discussion). The idea of 'background clusters' rests on the diversity of non-osmotrophs, and many many lineages of eukaryotes are undersampled. Similarly, interpreting individual gene trees and timing of expansion of osmoclusters all depends on taxon sampling. The reliance on such a limited set of species is surprising here given the wealth of data that have emerged on protists in the past decades.

Justification on why data quality issues from some protist groups limits their usage for gene content reconstruction analyses:

We agree with the reviewer that the community has produced a lot of valuable genomic data for protists in the last decades, and that this has substantially improved the coverage of the eukaryotic phylogeny in terms of diversity. Indeed, the present study wouldn't have been possible without this community effort. Having said that, we believe it is important to emphasize that a substantial fraction of the protist data produced, particularly for the most poorly represented groups, is based on transcriptomic sequencing of protist cultures which most of the times included bacterial contaminant species, and sometimes also eukaryotic contaminants. This type data is not adequate for large-scale evolutionary reconstructions of gene contents like our study; it is problematic for many reasons (contamination, duplicates, partial gene contents, etc.). To exemplify the limitations of some protist transcriptomic data available, among the EukProt v3 TCS species set, we could not incorporate any species from the following groups due to issues of data quality issues (either contamination reports or to high BUSCO duplicates / BUSCO missing values): Hemimastigophora, Telonema, Ancoracysta, CRuMs and Ancyromonadida. Still, none of these groups is phylogenetically proximal to the osmotrophic groups, and the current representation of the phylogenetic diversity is now broad, particularly in the extended taxon sampling.

Explanation on the re-analyses done with our extended taxon sampling:

As mentioned above, because we considered it important to address reviewer's concerns on our original taxon sampling, we re-run all those analyses that were computationally feasible for a dataset of a size of the extended one (122 species). In particular, we re-run all analyses except the species tree reconstruction and the molecular clock, which would have otherwise been too time consuming. The expansion of the species tree was thus achieved by incorporating the new taxa into our original species tree based on the most supported phylogenetic relationships among the available literature (see references used in Supplementary Table 8). On the other hand, regarding the molecular clock, no valuable fossil

data that could be informative for a deep species phylogeny of eukaryotes is available for the extended taxa. Dating an already comprehensive phylogeny of the size of the original taxon sampling (86 species) by employing sophisticated Bayesian methods was already very time consuming, and taxon sampling expansion has very limited impact on molecular clock analyses unless it allows incorporating informative calibrations.

As it can be seen in Extended Data Figs. 3-4, the results retrieved with this extended sampling are fully consistent with the results obtained with the original taxon sampling. This serves as a control to discard that a taxon sampling underrepresentation could have driven the original results found and described in this study. Regarding osmocluster counts, based on a preliminary exploration, we found that repeating the statistical tests with the extended taxon sampling indeed increases the amount of osmoclusters retrieved by employing exactly the same methodological settings, probably because the extended list of the background set of species provide higher resolution to find overrepresented gene families in the osmotrophic groups. Still, we decided to operate with the osmoclusters retrieved from the original taxon sampling, as the extended taxon sampling is richer in transcriptomic data which is more likely to contain inaccurate gene counts. Finally, we have repeated our HGT screenings from scratch based on this extended sampling (Supplementary Information 3 and 4). Of the 175 HGT clades originally reported, only a very narrow number (9) have been discarded from that fact that we do not consider them sufficiently supported anymore after having incorporated the extended taxon sampling into the gene trees.

To sum up, gene content trajectory analyses have been repeated under the extended taxon sampling, testing three alternative roots for the eukaryotic phylogeny (Extended Data Figs. 3-4), and HGT screens have been repeated from scratch based on the extended taxon sampling (Supplementary Information 3 and 4).

Interpretation of osmotrophy as a distinct character is confounded by the diversity of trophic forms among eukaryotes, including mixotrophy - and many mixotrophic/phototrophic lineages are not sampled. Not sampling key lineages will decrease the power of the study, and weaken the interpretations.

Among the eukaryotic diversity represented in our taxon sampling, we cover the vast majority of algae groups (i.e., the vast majority of mixotrophic/phototrophic lineages): chlorophytes, streptophytes, rhodophytes, glaucophytes, cryptophytes (*Guillardia*), chlorarachniophytes, myzozoans (dinoflagellates and chromerids), and haptophytes. With the extended taxon sampling, we now also cover *Euglena* and *Paulinella*, two photosynthetic groups which were missing from the original taxon sampling. A large diversity of heterotrophic groups is also represented (Fig. 3A, Extended Data Fig. 3).

Regarding the complexity of establishing phenotypic categories based on the diversity of trophic modes among eukaryotes, we consider that phenotypic categorizations are valuable providing they are useful and have a biological justification. In this regard, we believe that it is reasonable to consider the four groups we studied as groups that specialised into osmotrophy (“osmotrophic groups”) given the listed series of features that allowed them to specialise into osmotrophy (lines 74-93). Moreover, we consider that this categorization (osmotrophic groups vs background species) was useful as it allowed us to reconstruct the evolutionary history of a series of lineages which clearly specialised into osmotrophy, finding

interesting patterns and trends concerning the convergence on a gene content level that accompanied this case of phenotypic convergence.

The interpretation of the clock here is also problematic given the limited number of taxa sampled. Moreover, the manuscript would be improved with a greater discussion of the other events that likely occurred during the focal time period of 800-1000mya. Here the paper misses key articles such as:

https://pubmed.ncbi.nlm.nih.gov/37713454/
https://pubmed.ncbi.nlm.nih.gov/34949483/

We thank the reviewer for the shared references, which allowed us to extend our discussion on the retrieved age estimates (we have actually moved this section from Results to the beginning of the Discussion, lines 379-408). In this Discussion section, we have also added a statement making explicit the fact that there is substantial uncertainty in the literature concerning the geological features of that period (e.g., it is unclear which were the oxygen levels of that time, and fossil record of eukaryotes from that period is scarce).

Regarding limitation of the clock analyses concerning taxon sampling size, running a relaxed molecular clock analysis like we did with 86 species, while employing site-heterogeneous phylogenetic models (e.g., CAT, which have been shown to be important for deep species phylogenies) is already very time consuming. Dating larger taxon samplings is possible, but at the expense of employing simplistic phylogenetic models. For example, two reference studies which dated a paneukaryotic phylogeny employed larger taxon samplings (109 species in Parfrey et al. 2011, PNAS, or 136 species in Strassert et al. 2022, Nat Comm). However, they could achieve this extended size compared to us by the fact of having used simplistic site-homogeneous models for the datation step. Moreover, the difference in size with our taxon sampling is due to the extended coverage of some groups, the overrepresentation of which is not priority for our study (e.g., ~20 Viridiplantae in Strassert et al., ~20 Excavata in Parfrey et al.). On the other hand, taxon sampling expansion has very limited impact on molecular clock analyses unless it allows incorporating informative calibrations, which is not the case for the taxa that we could incorporate in our extended sampling. In this regard, the limiting availability of an informative fossil record of eukaryotes from before the Cambrian period, and particularly from before the Neoproterozoic, is an important challenge for relaxed molecular clock datations of eukaryotes. We have incorporated an explicit mention to this limitation in the text (lines 379-408).

**The work largely ignores known problems in interpreting horizontal gene transfer events. It is surprising to see so little mention of caveats and cautions for interpreting HGTs. As documented elsewhere, interpretation of single gene trees requires considerable more finesse to account for gene loss and unstable tree topologies. Addressing these issues can readily be done by including more taxa and exploring alternative topologies (e.g. AU test). Moreover, greater acknowledgement of uncertainties and past literature would strengthen the manuscript.

Some references here:
https://pubmed.ncbi.nlm.nih.gov/33012528/
https://pubmed.ncbi.nlm.nih.gov/35731825/

https://pubmed.ncbi.nlm.nih.gov/40402828/
https://pubmed.ncbi.nlm.nih.gov/38263430/

In the Discussion, we now include a paragraph (lines 410-444) discussing with further detail the challenges of inferring HGT events as well as why an alternative history based solely on gene-loss+unstable tree topologies is unparsimonious enough to be rejected (lines 446-462), new Fig. 5). We thank the reviewer for the shared references. In the Discussion we now also added further mentions to previous studies on HGT in eukaryotes (lines 410-423).

The design of our methodology to explore for HGT is based on our expertise and on the methodology that is typically employed in the literature. On a first step, we followed the standard approach of inspecting gene trees to identify well-supported clades including distantly-related taxa. We agree with the reviewer that a good standard practice is to validate the found phylogenetic associations, knowing that gene tree reconstruction methods have a limited accuracy and can lead to artifactual tree topologies. While we have previously employed topological tests (https://doi.org/10.1371/journal.pgen.1007986), employing this approach systematically for a long list of cases in which the alternative topologies that should be tested are unclear due to paralog-rich and unrepresented species in the gene trees makes it a suboptimal approach. Further, topological tests are also affected by artifacts in the phylogenetic reconstructions, and are also sensitive to the potential lack or presence of erroneous sequences in the trees. To double-check the candidate HGTs found, what we did is to confirm that the phylogenetic associations between sequences from the distantly-related groups were also recovered based on an independent method. For that, we performed alignments between sequence representatives from the candidate HGT clades against the whole dataset which includes millions of eukaryotic and non-eukaryotic sequences. We only considered as positives those cases in which the phylogenetic association found in the gene trees were supported by the fact that the sequences involved also show high sequence similarity scores between them compared to alignments against other sequences. We carefully looked at every case one-by-one, and wrote a justification report for each potential HGT topology identified in the osmoclusters (Supplementary Information 3). Still, we acknowledge limitations of HGT inference, and in the abstract, we refer to our findings as “signatures that are highly compatible with horizontal gene transfer” and “gene tree topologies which are supportive of transfer events”.

For every HGT-suggestive topology, an alternative explanation based on the pervasive occurrence of duplications and losses and/or gene tree errors is always possible. Still, Fig. 5 shows that these alternative explanations are highly unparsimonious and hence unrealistic (lines 446-472). We have adapted the abstract to acknowledge these findings (lines 26-29): “*We report signatures that are highly compatible with horizontal gene transfer encompassing at least 20% of this toolkit, and show that evolutionary reconstructions of ancestral genomic content of these genes are only realistic by factoring in gene transfers*.”

We have also re-evaluated all cases of HGT based on the extended taxon sampling. This addition of further taxa in the gene trees has led to consistent numbers of found HGT topologies (166 HGT clades now, 175 before). Considering also the functional trends (overrepresentation of metabolism, Fig. 4D) and the overrepresentation of specific lineages in the HGTs found (Figs. 4A-C), these altogether made us confident that our approach

allowed us to identify real biological signals over the methodological noise that often challenges the identification of HGT events (Discussion, lines 446-472).

Other issues
The logical flow of the last sentence of the abstract could be improved.

We have modified the sentence: "*... With our results supporting a prevalent role of gene transfer in the evolutionary history of these groups, our work identifies an extended contribution of reticulate evolution in facilitating convergence towards specialised trophic modes across eukaryotes.*" (lines 32-35).

I am unconvinced that all osmotrophs are decomposers many parasites are osmotrophs (e.g. tape worms)

We agree with the reviewer that not all osmotrophs are decomposers, they can also be symbionts/parasites, etc., so the current version of the manuscript avoids using both terms as synonyms. "*In both terrestrial or aquatic environments, osmotrophs usually live as symbionts/parasites or as free-living decomposers, …*" (lines 109-111)

The claim that 'canonical phagotrophy is also absent' (line 91) in osmotrophs seems a bit circular given definitions here

To clarify this, we rephrased this point as "*ii) heterotrophy via specialised absorptive nutrition, with absence of canonical phagotrophy*" (lines XX-YY).

Similarly, the association of "multinucleated coenocytic/syncytial development" with osmotrophy misses that these features are also widespread among photosynthetic 'algae' and in many phagotrophic amoeboid groups

In the current version of the manuscript we have aimed to clarify this point "*While multinucleated development is neither specific nor strictly necessary for osmotrophy, it allows organisms with an expanding volume of cytoplasm to maintain a constant nuclear density*" (lines 84-87).

The logic here is unclear "Osmotrophy specialisation must require changes in gene content" (line 102) – this could be an exaptation, or a simple case of gene loss

This sentence has been removed as it was unnecessary for the paragraph.

Perhaps 'tone down' quotes from past literature as at times they feel forced and they may not be widely accessible. Here is just one example where meaning was unclear:
-If we had to replay life's tape as imagined by Stephen Jay Gould , but constraining eukaryotic evolution to a fully unidimensional process, with no reticulations, would major groups of eukaryotes that specialise in osmotrophy and photoautotrophy repeatedly evolved?

Thanks for pointing this out. We believe the current version of the text is more accessible now. For example, we have replaced the quoted sentence by "*In the absence of*

reticulations, would major osmotrophic or photoautotrophic groups of eukaryotes have repeatedly evolved? …" (lines 474-475).

The quality of figures could be improved to make images more easily readable

We have standardised colors between figures (using always the same color for each osmotrophic group), increased word size, streamlined axis labels, and improved resolution whenever possible. Furthermore, all figures and supplementaries have been uploaded in the Figshare repository so that they can be visualised with maximum resolution.

The brevity of the discussion is surprising, and incorporating more literature (including those linked above) would strengthen the manuscript

We believe the quality of the Discussion has significantly improved with extended sections of molecular clock (lines 379-408) and one new section about the limitations of HGT inference and discussing alternative explanations to HGT (lines 425-472). We would like to thank Reviewer #1 for the many valuable suggestions made.

Reviewer #2 (Remarks to the Author):

The authors examine the contribution of reticulate evolutionary trends in trophic variation across eukaryotes. They focus on “osmogroups” - four groups of eukaryotes specialized in osmotrophy on either side of the Diaphoretickes/Amorphea split. First, the authors performed molecular clock dating analysis to establish a timeframe for the evolution of eukaryotic osmotrophy between 800-1000Ma. Next, the authors identified 172 “osmoclusters” - gene clusters encompassing >3 osmogroups that likely evolved convergently on either side of the eukaryote split. Metabolic and enzymatic functions are over-represented in these osmoclusters, as well processes related to osmoregulation. The osmoclusters have a deep origin in eukaryotes, but the greatest diversity and copy number is seen from the origin of each osmogroup to the present. Using reconciliation analysis, the authors find that two waves of HGT of osmoclusters occurred: first from non-osmogroups to osmogroups during early osmogroup evolution, and later between osmogroups as they diversified. Other evidence of HGT was also observed separately between Fungi and Pseudofungi and between the two marine osmogroups, consistent with previous findings of HGT between Fungi and Pseduofungi (particularly in oomycetes). These HGT events concern genes involved in metabolic processes.

Overall, this is a rigorous study of the evolutionary origins of osmotrophy in eukaryotes. The authors propose that reticulate evolution in the form of HGT shaped the evolution of osmotrophy similar to how endosymbiosis shaped the evolution of photoautotrophy. I think this is a reasonable assessment, given that many osmogroups arose at similar dates and at one point inhabited similar niches as evidenced in the fossil record. I agree with the authors’ statement in the Discussion that reticulate evolution of trophic specialization across unrelated taxa is a more parsimonious explanation than de novo evolution for traits that are present in multiple eukaryotic lineages but are not ancestral to eukaryotes.

I think this study would be acceptable for publication pending minor alterations below.

We thank the reviewer for the suggestions made and for acknowledging the rigour and the value of our study.

Abstract Line 24 and Discussion Line 318: I would say “have received”.

We have fixed it: “*the multiple origins of osmotroph specialists have received little attention*” (lines 22-23).

Main Text Line 46-51: I don’t think it’s correct at all to state that RE is a process by which species or branches of a phylogeny can “merge” either by endosymbiosis or HGT. This is a very informal description of its outcomes, at best, and this section should be revised.

Thanks for pointing this out. We have gone through this paragraph in the text, aiming to include a more formal description of reticulate evolution (lines 53-65).

Reviewer #3 (Remarks to the Author):

In this work, the authors identify genes that are enriched in the four large known clades of eukaryotic osmotrophs: fungi, teretosporeans, labyrinthulids, and oomycetes (pseudofungi). They then explore the origin of those genes and show that a considerable proportion of them were acquired by horizontal gene transfer (HGT), either from other osmotrophic eukaryotes (notably fungi) or, ultimately, from bacteria. They also apply molecular dating approaches to date the origin of those eukaryotic clades. The authors have done an impressive amount of work, providing additional and extensive support to the idea that those lineages of osmotrophic eukaryotes adapted to that nutrition mode by importing genes from fungi and, originally, from bacteria. However, as it appears now, the manuscript is obscured by confusion in some concepts and names, lack of linearity in argumentation, and the mixed nature of different sections. I think this is a valuable piece of work, but the manuscript needs considerable rewriting and streamlining to present clear hypotheses, results, and interpretation in a connected, linear manner.

We would like to thank Reviewer #3 for the feedback shared and the suggestions made. As it will be detailed point-by-point below, we have made an effort to streamline the manuscript, unifying the previously first two sections as a formal Introduction section, which now presents more clearly the aims of this work. We also streamlined the Results section, moving our elaboration on the molecular clock results to the Discussion. Following suggestions by the reviewers, we also extended the Discussion by providing more details on the challenges of HGT inference and more references to previous works on HGT in eukaryotes.

My major comments are as follows:

- Some parts of this manuscript appear to be written for a PhD thesis rather than a research article. For instance, the introduction discusses some generalities about the importance of endosymbiosis but does not present the specific questions and hypotheses actually addressed in the present research. These are introduced along the way, sometimes in the methods section (e.g. line 413 and subsequent).

We have re-organized and thoroughly re-written the previous two first sections, joining them into a formal "Introduction" section. In the Introduction, we present important concepts and the background that the reader may need to follow this manuscript (we present the uncertainty concerning the convergent evolution of alternative trophic modes to phagotrophy in eukaryotes, including the gap of knowledge concerning the convergent evolution of osmotrophy specialisation; we then introduce the osmotrophic groups, as well as previous results which suggest HGT as potential mechanism that may have had a general contribution to the convergent evolution of these groups); concluding with lines 120-123 in which we explain the main approach and question addressed in this study "*To clarify the role of HGT and the evolutionary history of these groups, we used comparative genomics, phylogenomics and molecular clock methods to reconstruct and date the trajectory of gene content changes that accompanied the convergent evolution of osmotrophy specialisation in eukaryotes.*"

- Part of this confusion stems from the fact that, although the authors present and discuss data pertaining to osmotrophic lineages, they do not carry out a thorough comparison with photoautotrophic lineages. …

We hope in the current manuscript it becomes clear that the objective of our work is to clarify the gap of knowledge concerning the role of reticulate evolution (via HGT) in the evolutionary convergence of some eukaryotes towards osmotrophic specialisation (see improved Introduction, lines 53-128). We consider that the convergent evolution of photosynthetic eukaryotes has already been widely studied such as to confidently claim that reticulate evolution (via endosymbioses) also had a role in this case of phenotypic convergent evolution.

… These are alluded to in different sections of the manuscript, but in an ambiguous and not very direct manner. Yet, many of the genes (orthoclusters) enriched in osmotrophs are also present or enriched in photoautotrophs. There is a (logical) hypothesis in the methods section about why this is so, but it is not properly addressed or commented on in the main text. At the same time, if the same genes are enriched in osmotrophs and photoautotrophs, can these genes be considered an “osmotrophic toolkit”?

Regarding osmoclusters and the “osmotrophic toolkit”. After having re-considered it, the current version of this work only considers as “osmocluster”—and hence only studies the evolution of—those clusters that are strictly overrepresented in the osmotrophic groups, with the photosynthetic species being now also included the background set of species when doing the statistical tests. We believe choosing this *sensu stricto* definition for osmoclusters is the right choice and avoids the confusion that Reviewer #3 pointed out.

… Furthermore, “osmoclusters” appear highly enriched in some photosynthetic lineages, notably ochrophytes (cf. Fig. 3A), but not in others (Archaeplastida). How can one differentiate between function and phylogeny as drivers of the observed patterns? Those genes may have been acquired at the base of stramenopiles and then inherited by ochrophytes, not because of their role in osmotrophy/phototrophy, but because they are neighboring groups. I feel that either there should be a full discussion about this, with comparative data and tests supporting the authors’ ideas, or—perhaps more practically—the manuscript should focus on the phylogenetic side, i.e., on the acquisition of genes by HGT at the onset of those different osmotrophic lineages, which may have enhanced or facilitated the adaptation to osmotrophy, without necessarily being exclusive or diagnostic for that nutritional mode.

Before (but we have changed it, as described in the previous response), we allowed osmoclusters to be also overrepresented in the photosynthetic groups (by not including photosynthetic species in the background set of species during the statistical tests). We did so based on the expectations that some metabolic genes involved in anabolic functions are likely important for both photosynthetic organisms and for osmotrophic organisms, as we indeed identify and report in the current manuscript (lines 209-244). However, with the aim of following a more strict definition for osmoclusters, as we argued above, we are not including anymore these extra sets of gene family clusters among osmoclusters. In the current manuscript, we only refer to this additional set of clusters in a specific section of the

manuscript in which we explore extended metabolic features in osmotrophs compared to other heterotrophs (lines 209-244).

On whether osmoclusters may include false positives due to phylogenetic association rather than to phenotypic association, while we can't expect any approach to define osmoclusters to be perfect, the requirement that osmoclusters should be overrepresented at least in three groups, which implies that at least two groups are from different divisions of eukaryotes, is a strong filter to discard this potential confounding factor. Moreover, the clear functional trends that we have identified and we describe for osmoclusters in comparison to the background set of clusters is what we would expect for a set of clusters in which at least the vast majority of it can be confidently considered as convergent evolutionary changes that were important for the evolution of osmotrophy specialisation in eukaryotes. On the other hand, the fact that photosynthetic groups (Ochrophytes but also others) present slightly higher copy numbers of those gene families that we identified as osmoclusters compared to, for example, phagotrophs (although they still present much fewer copies than the osmotrophic groups, see Fig. 3) is not unexpected. Indeed, we expected similarities on a gene content level between photosynthetic eukaryotes and osmotroph specialists. We elaborate into this on (lines 209-244: … "*In limiting nutrient conditions, the ability to assimilate inorganic sources or simpler metabolites can provide an advantage over other competitors for public goods. As such, we expected osmotrophs to present an expanded anabolic section of the metabolism compared to phagotrophs. Because the same could be expected for photosynthetic eukaryotes, shared similarities may exist on a metabolic level between the osmotrophic groups and the photosynthetic groups …*"). Still, the scope of our work is to focus on those evolutionary changes that occurred specifically in the evolutionary path leading to the osmotrophic groups.

- The use of the term "osmogroup" (meaning osmotrophic eukaryotic groups) is extremely confusing and unnecessary. I recommend that the authors refer to these clades of eukaryotes as "osmotrophic eukaryotes/groups/lineages/clades." The root "troph," which is a key concept in the whole discussion, is lost, so the term alone does not convey the idea of osmotrophy. Furthermore, "osmogroup" (referring to organisms) can be easily confused with "osmoclusters" (referring to genes). This confusion is worsened by the existing term "orthogroups" (OGs – genes), which is used to name and number the osmoclusters. Please find a simple and unambiguous way of naming things. Sometimes, not having an acronym is better

Thanks for pointing this out. We have removed the abbreviation "osmogroup/s", and we use "osmotrophic group/s" instead.

- Some concepts are presented in a way that denotes misunderstanding or could lead to misunderstanding. For instance, in the abstract, osmotrophs are equated with decomposers. While osmotrophs are (generally) decomposers, the opposite is not necessarily true. Many decomposers are, for example, insects or worms, which degrade organic matter by phagocytosing it.

To avoid any chance of confusion or misunderstanding, we have modified those sentences which may have been misleading by employing the term decomposer when osmotroph was the right term (Introduction, lines 109-111: "*In both terrestrial or aquatic environments,*

osmotrophs usually live as symbionts/parasites or as free-living decomposers, often occupying three dimensional environments composed of complex polymerized and recalcitrant metabolic resources)”)

Even the title of the article is a bit misleading in this sense: Reticulate evolution and the rise of trophic complexity. Do the authors consider osmotrophy a trophic mode of higher complexity? Arguably, phagotrophy—involving a developed cytoskeleton, membrane remodeling, and (also) degradation enzymes—can be considered more complex. True, that argument could potentially be applied to photosynthesis as well, but the results of this manuscript do not address that question. I would suggest focusing on osmotrophy for the sake of clarity.

Thanks for pointing this out. To avoid any misunderstanding, we have changed the previous title for “*Reticulate evolution facilitated convergence towards specialised trophic modes across eukaryotes*”.

On why we generalize to “reticulate evolution” and “trophic modes” instead of specifically referring to “HGT” and “osmotrophy specialisation”: In our study we specifically focus on the evolution of the eukaryotic osmotrophs, true. While we didn’t study the evolution of photosynthetic groups in eukaryotes, there is plenty of work on the evolutionary history of these groups and the role of endosymbiosis in the multiple acquisitions of plastids by eukaryotes such that we can integrate our findings (HGT in osmotrophy) with these previous findings (endosymbiosis in photosynthetic organisms) to generalize about the contribution that reticulate evolution had in the diversification of eukaryotic trophic modes. We believe this is a nice conceptual framework from which to present our work to the community, but we agree, as previously pointed by the reviewer, that a major re-writing of the introduction was needed to clarify the aim and scope of this work (lines 38-129).

- Whittaker – please, drop the reference to his work. Many ecologists and protistologists have rightfully discussed nutrition modes. However, despite the focus on nutrition, Whittaker’s view on evolution was based on the assumption that evolution always proceeds towards increasing complexity and that simple means primitive (animals, plants and fungi being on top of all unicellular organisms). His evolutionary scheme was wrong, as demonstrated by molecular phylogeny. For instance, according to Figure 1 in Whittaker’s cited paper, fungi derive directly from bacteria. Accordingly, their nutrition mode would directly derive from bacterial osmotrophy (vertical inheritance). Just the opposite of what you are saying, i.e. that fungi evolved from phagotrophic organisms and acquired genes from bacteria (horizontally) to convergently adapt to osmotrophy.

We fully agree with the reviewer that the evolutionary interpretation of Robert Whittaker is nowadays completely obsolete. We agree with the reviewer that overreferencing this work can be problematic, and because of that, we have removed all previous references to this work in the Discussion and in the Abstract. In the current draft, we are only referencing this work once, in the beginning of the Introduction (lines 38-51), and for one particular reason. Despite being obsolete, the fact that a taxonomic classification system that has been in biology textbooks for decades misplaced in the same categories lineages that are distantly-related (as we nowadays know) to each other by the fact that they are strikingly similar from a phenotypic perspective (trophic modes), we believe this is very illustrative of

how remarkable are the cases of convergent evolution that we study and discuss in this work. Still, while mentioning this work in our manuscript, we now emphasise more clearly on the fact that, while intuitive, the idea of a taxonomic system based on these nutritional modes is not supported by species phylogenies, and that this system is nowadays obsolete (lines 38-51).

- Bacteria are alluded as gene donors. However, they are a black box referred as "non-eukaryotic donors". Which are the bacterial donors? This information is missing or so hidden that it is impossible to retrieve. Yet, this might provide interesting information. Many bacteria are osmotrophs, but not all. For instance, cyanobacteria (photosynthesizers) are known to have exchanged genes with fungi. Is there any preferential donor? It is a pity that a discussion on the prokaryotic donors of osmotrophy-related genes is completely absent

In order to make gene tree-species tree reconciliation analyses feasible, we employed a trick that we already used in some previous studies, which is to incorporate a "Non-eukaryotes" pseudobranch in the species tree, branching as a sister-group to the rest of eukaryotes. Reconciliation analyses are very time consuming. The advantage of our strategy is that it allows us to get information on non-eukaryotic lineage transfers to eukaryotes, which otherwise would not be possible under this methodology. The limitation is that we can't know specifically which lineage was the putative donor of each gene. Still, we agree with the reviewer that it would be interesting to offer the readers some insights on the non-eukaryotic sources of osmoclusters, acknowledging at the same time that this type of analyses of identifying potential prokaryotic sources of genes to eukaryotes are often limited by the phylogenetic signal available, bacterial pangenomes and by the high prevalence of HGT within bacteria. One way we have found to do this is to explore which non-eukaryotic species are more represented in the osmoclusters. Since the strategy to incorporate non-eukaryotic sequences into the clusters was a kind of best bi-directional hit approach between the eukaryotic sequences of the clusters and the large dataset of non-eukaryotic sequence (Methods, lines 646-661), we can expect the non-eukaryotic sequences incorporated into the osmoclusters to be those showing higher sequence similarity (a proxy of phylogenetic proximity) to the sequences represented within the osmoclusters. By mining NCBI taxonomy information, we counted how many times each taxonomic category was represented, proportionally, in the osmoclusters (Extended Data Fig. 2). We found Actinomycetes (15.9%), Gammaproteobacteria (15.2%) and Alphaproteobacteria (12.8%) as the three outstanding taxonomic classes among the non-eukaryotic sequences represented in the osmoclusters (Extended Data Fig. 2A). However, if we compare with the proportions obtained from random samplings of clusters from the background set, to identify which representations of bacterial classes are more unexpected, only Actinomycetes (+8.5%) stands out over the rest (Extended Data Fig. 2B). We have incorporated a mention of these findings in the Results section 'Origin and expansion of the osmoclusters' (lines 245-266).

- Dating. This is not uninteresting, but potentially this part would rather benefit from being introduced later, close to the discussion.

We have moved our elaboration on the results from the dating analyses to the onset of the Discussion section (lines 379-408). We believe this may help in making the reading of that Results section more straightforward. We still describe the Results of this analysis at the beginning of the Results section, because for us it feels natural talking about the ages of the

osmotrophic groups right after the introduction. First, we think it connects well with the general narrative flow (in the introduction we present the osmogroups groups, then in the results we start by showing that these case of phenotypic convergence most likely occurred in a similar age range, and then we jump to exploring whether this case of phenotypic convergence was followed by convergence on a genetic level, i.e., the osmoclusters, HGT analyses, etc.). The second reason is that we not only dated for the purpose of retrieving age information for these lineages, but then we also employed the retrieved ages to date the gene content changes of the osmoclusters in the path to the extant representatives of the osmotrophic groups. We believe that it is thus important to show first from where the ages employed to date the gene content trajectories come from.

- Reconciliation – many gene trees are poorly resolved. How to discriminate between poor signal and real HGT? A well-known problem deserving a discussion. How many “false positives”?

Thanks for pointing this out. We have included a section in the Discussion (lines 425-472) explaining challenges of HGT inference, how we have dealt with these challenges, and describe evidence that support that our approach captured true signal over the uncertainties and limits that can challenge HGT detection (e.g., a clear functional bias of the identified HGTs to metabolism, absence of randomness in the phylogenetic distribution of the found HGTs, Fig. 4).

Because noise in gene trees can inflate HGT counts, we have employed a conservative approach to identify gene transfer events. First, we only focused on candidate HGT topologies which correspond to highly supported bipartitions. Next, we manually evaluated all candidate HGTs, requiring validation by an independent methodology (sequence-similarity alignments), in order to avoid HGT inferences that could be misled by gene tree artifacts (Methods, 774-800).

This methodological approach has been set to maximize specificity over sensitivity. First, we have only focused on supported gene tree clades validated by sequence-similarity analyses described above. Furthermore, we only considered candidate HGT topologies involving phylogenetic associations between sequences from osmotrophic groups that belong to two distinct eukaryotic divisions, since these are less likely to be artefactual, and less likely to be explained by a pervasive occurrence of gene losses. Regarding this latter point, to increase the robustness of our results, we have incorporated a control analysis which shows that the evolutionary history of the osmoclusters is only realistic by factoring in gene transfer (Fig. 5, lines 446-472).

Regarding the usage of reconciliation for HGT, because of the challenges of overrelying on automated methods for HGT inference, we only employed reconciliation for those osmoclusters in which we previously identified, in the supervised analyses, topologies that are likely to be explained by HGT events (Supplementary Information 3). When there are topologies that are highly compatible with HGTs, but for which there may be uncertainty on the exact lineages that could have been involved in the role of HGT donors/receptors, probabilistic reconciliation methods such as ALE are useful because they deal with uncertainty in a probabilistic manner. ALE samples a distribution of reconciliation scenarios from which we can obtain the probability of pairs of donor/receptor lineages to have been

involved in a HGT, this while also taking into account the whole heterogeneity of the bootstrap samples obtained during the gene tree reconstruction step. By doing so, we could add up results from the reconciled osmoclusters (in which we previously identified potential HGT topologies) and aggregate the signal to get insights into an approximate distribution of osmogroup-to-osmogroup HGT signal over evolutionary time. We have added the details of this methodological choice described above in the Methods section (lines 684-700).

Reviewer #1 (Remarks to the Author):

Review of “Reticulate evolution and the rise of trophic complexity in eukaryotes”

This is a revision of a manuscript that seeks to elucidate the evolutionary history of osmotrophy among eukaryotes. Using a set of phylogenomic methods, the authors investigate the toolkit used by a diversity of osmotrophic lineages, and also date the timing of the acquisition of these genes using molecular clock approaches. I agree that this topic is one that has yet to receive sufficient study, and I am impressed by the extensive revisions in light of the first round of review. However, though the current manuscript is much improved, some important points still need to be addressed.

We thank Reviewer #1 for the discussion and suggestions made to improve our manuscript.

Defining shared clusters: I am a bit confused by the discussion of the clusters (lines 154-163), and I think that this can be addressed by rewriting here. In order for a gene family to be 'convergent adaptations', they need to be in at least 3 of the 4 osmotrophs AND absent for non-osmotrophs.

We have clarified it: “*We grouped extant protein sequences into clusters, used as proxies for gene families. Then we performed statistical tests to identify clusters overrepresented in copy number in the osmotrophic groups compared to the rest of species*” (lines 128-131)

(Obviously, widespread presence in osmotrophs and non-osmotrophs is consistent with the gene family being present in the last common ancestor of both groups). The second paragraph in this section mentions ‘background clusters’- perhaps these might be described early, and perhaps renamed (e.g. ‘ancestral clusters’, or at least the authors need to briefly defining what is meant by ‘background’).

We have replaced “background clusters” either by “other clusters” or by “non-osmoclusters” throughout the manuscript, which are more intuitive terms.

I also think this section (and the entire manuscript) would be improved by acknowledging the possibility of gene loss contributing to the patterns for at least some gene families (see below). Alternative hypotheses: The section entitled “Horizontal gene transfers in osmoclusters” certainly needs some discussion of gene loss, especially given reliance on data visualization for some inferences. A similar caveat deserves mention in the next section as well. And again in lines 474-483. And in the section on “Manually supervised identification of osmoclusters with putative HGT patterns” starting on line 774.

We agree with the reviewer on the need of including further mentions and discussions about gene loss throughout the manuscript. We are now doing so in the two Results section mentioned by the reviewer (lines 223-228, lines 272-275), in the Discussion (lines 401-419, 425-431) as well as to the corresponding methods section (lines 733-739, lines 759-762). E.g., In the section “Horizontal gene transfers in osmoclusters”, we now start with: “(lines 223-228): *Gene duplications and gene losses are major mechanisms driving gene content changes in eukaryotes. However, the old origin of the osmoclusters (Fig. 3A), combined with the reported increase in osmoclusters’ genomic diversity in the osmotrophic groups (Fig. 3B), suggests a potential*

role for HGT in the evolutionary history of these gene families. We therefore explored the role of HGT in the osmotrophic groups (but see Discussion for an alternative, HGT-independent evolutionary reconstruction of osmoclusters). ...". On the other hand, Fig. 5 has been updated to show a clearer contrast between our evolutionary reconstructions of osmoclusters with and without HGTs, and we explain why we find the no-HGT scenario less realistic (lines 401-419).

Writing:Overall, the writing could be improved a bit further to clarify logical flow. As just some examples, the inferences about Tonian origins could be removed from results – many/most readers won't know much (anything?) about the Tonian, and so perhaps this is best only in the discussion where greater context can be provided.

We have made a substantial effort to improve the writing over the manuscript. Regarding the specific section mentioned by the Reviewer, we have removed the mention to Tonian origins from the Results (now only in Discussion). Now: "... *osmotrophy specialisation may have evolved convergently in the eukaryotes during a similar geological period, most likely near the beginning of the last billion years, predating the emergence of crown-group metazoans and the embryophytes (Fig. 1, we elaborate on this in the Discussion section)*" (lines 122-125)

Similarly, there are numerous overly-long paragraphs and other opportunities to clarify text (see examples below). The paragraph in the discussion from 425-444 seems to repeat too much of the methods/results.

The manuscript has been revised in accordance with reviewers' recommendations, streamlining and improving the content of some paragraphs. Overly-long paragraphs have been split to enhance readability. On the other hand, we agree with the Reviewer that the former paragraph in lines 425-444 was repetitive. This has been improved (lines 380-399).

This sentence seems highly speculative and could do with a bit of refining: "Since eukaryotes are usually larger than prokaryotes and also more prone to evolve multicellular forms, a progressive increase in the relative representation of eukaryotes during the Tonian may have predisposed the evolution of specialised osmotrophic forms that could exploit more efficiently the gradually increasing availability of polymerised and structured organic matter and associated macroscopic forms." (of course, cyanobacteria and bacterial biofilms can also get quite large). In fact, the language/logic of the rest of this paragraph would benefit from some revision (lines 390-408)

We have streamlined the paragraph to improve its logic and readability. In doing so, we removed the quoted section entirely. We agree with the reviewer that the statement was speculative and, upon review, found it to be redundant to the final part of that same section in the Discussion (lines 356-360: *… Altogether, we hypothesize that these groups most likely emerged in aquatic environments enriched in organic matter and composed of communities which, despite still being dominated by prokaryotic forms (perhaps cyanobacterial mats), included an increasing representation of eukaryotes, some of which probably presented multicellular forms.*)

Figures:
Though improved over the last version, more could be done on the main figures.

Figure 1: The organismal images in figure 1 are disappointing: not particularly clear, no scale bars (this is unacceptable), and you have the opportunity to teach a bit about these poorly-known lineages. Please rethink.

The images in former panel 1A were provided by collaborators as we aimed to offer visual context for these lineages. As they served a purely illustrative purpose and are not relevant to our quantitative findings, we have removed them to address the reviewer's valid concerns regarding image quality and missing scale bars (which were unavailable for some of these third-party materials). As a result, we have extended the size of Fig. 1B, which now has higher resolution and is now full figure 1. We have extended the introduction and the list of references for readers interested in knowing more about these groups (lines 51-69).

Also, the legend is inaccurate (no phylogeny in this version) and the description of figure 1B is likely not accessible to a naive reader. I suggest a complete rewrite of this legend (and I wonder if this is in part the result of putting time into writing a very long rebuttal that repeats details in revision – perhaps better to use some time in improving the main document).

Fig. 1's caption has been made more accessible "Fig. 1. *Timing the origin of Teretosporea, Fungi, Labyrinthulea and Pseudofungi, four eukaryotic groups which experienced parallel transitions toward osmotrophic specialisation. Curves represent the proportion of post burn-in sampled chronograms from relaxed molecular clock analyses (n=32,379) in which the estimated age of a group is greater than or equal to the time indicated on the x-axis. …*".

Figure 3: rethink colours - the brown is hard to discern.

We have improved the color code used for each osmotrophic group.

Figure 5: the number of events in 5A is difficult (impossible?) to discern. I suggest moving to something like numbers in circles, and perhaps moving 5B to supplement to make this important part of the clearer. In fact if 5A was bigger (maybe not a circle?), you could do more to teach the diversity of organisms, which would help broad readership.

Our intention with Fig. 5 is to illustrate that the trajectory of osmocluster changes is less realistic when reconstructed with the vertical-only mode, i.e., without factoring in gene transfers. We have improved Fig. 5 for a better display of the contrasts between the trends retrieved with the HGT-yes vs vertical-only models. Figs. 5 now shows this comparison in a 2x2 panel showing the trends retrieved for osmocluster diversity (presence/absence) and osmocluster copy number for both approaches (HGT-yes vs vertical-only). The caption has also been modified to clarify the aim of the figure.

On the other hand, we have also introduced taxonomic information on the figure showing osmocluster content changes over the eukaryotic phylogeny for the extended taxon sampling (Extended Data Fig. 4). This will help broad readership to understand our species tree for the extended taxon sampling.

Some minor comments

On language, the following seems a bit odd: "proved attractive" when referring to Whitaker (line 38). Where is the ‘proving’ demonstrated? In fact, I would rethink this first paragraph as it seems written for an in-crowd.

The paragraph which included this sentence has been re-written, now providing a more direct introduction to osmotrophy and the osmotrophic groups (lines 41-49).

Perhaps define "reticulate evolution" briefly (in parentheses) after first use of this term (line 53).

Given Reviewer 3’s concerns about the analogy between endosymbiosis and HGT, we don’t put emphasis anymore on the concept of reticulate evolution. We have adapted the title, the first paragraph of the introduction, and the end of the discussion for a more direct and clear presentation of our work (“On the convergent origins of osmotrophic specialisation in eukaryotes”). This change has also allowed us to improve our introduction about osmotrophy as a feeding strategy and about the osmotrophic groups studied in this work (see below).

Similarly, some slightly more elaborated explanation of osmotrophy might be good early on.

The first paragraph of the introduction now provides a direct and immediate definition for osmotrophy, lines 41-49: “... *Osmotrophy is a form of heterotrophic nutrition based on the absorption of nutrients directly from the environment, often via extracellular digestion of complex molecules, without relying on phagocytosis. While this strategy is to some extent present in many organisms, some groups have independently evolved a series of adaptations that allow them to specialise in this trophic mode*”. Then the second paragraph continues with a presentation of the eukaryotic groups which specialised in osmotrophy.

As is, the intro defines just as 'absorption (osmotrophy)', yet I imagine that phototrophs and heterotrophs still absorb some things?

We have removed the oversimplified equivalence “absorption (osmotrophy)” while streamlining and improving the first paragraph (see above).

I might also provide a bit more definition of these terms for non-specialists: Teretosporea, Pseudofungi and Labyrinthulea.

The 2nd paragraph now provides a more detailed introduction to these groups (lines 51-59).

And then start a new paragraph in line 7

We have made an effort to streamline and split very large paragraphs into smaller ones.

Rethink the use of the word 'host' (line 112)

The current version of this sentence is “*Osmotrophs do not feed by internalising particles such as prey cells. Instead they are specialised in absorbing subunits directly from the environment for metabolism*” (lines 89-90).

The paragraph on page 7 is quite long, perhaps break somewhere to improve logical flow. In fact, look for too-long paragraphs throughout to improve writing/logic.

We have separated the paragraph in the sentence "*When exploring osmoclusters including this additional set of clusters …*" (line 180). We have improved the writing throughout the text.

In legend, mention that coloured branches in figure 3A are osmotrophs.

Fixed, thanks: "*Four colors other than black have been chosen to indicate the clades corresponding to the four osmotrophic groups (see legend)*"

Perhaps rethink these colors though as the brown and grey look very similar, particularly in figure 3C!

We have improved the colors for the osmotrophic groups in order to better distinguish the trends in the figures.

Line 357 - overrepresented compared to what? What is null here?

We have clarified this: Lines 306-307: "*Metabolism is overrepresented in the 163 gene families with HGT topologies compared to all other gene families sampled*"

Line 377 - teach something about the Tonian, please - in just a short phrase. discussion is your opportunity to provide context for naive reader.

We have integrated this sentence with the paragraph that follows it (lines 333), which provides information on major events during the Tonian period, and discusses them in the context of the possible origin of the osmotrophic groups during this period (lines 330-345).

In rebuttal – another exam of poor writing: I am not sure what 'fairly unrelated' means.

We apologize. By 'fairly unrelated', we meant 'distantly-related to' in a phylogenetic context.

Reviewer #3 (Remarks to the Author):

The authors have made a considerable effort to clarify the manuscript and enhance its readability. It is very much appreciated. They have responded to most of my questions and I recommend publication.

We thank Reviewer #3 for the feedback given during the revision process.

A few minor points:

- Please substitute the “don’t” by “do not” along the text.

Solved.

- Line 210, “phagocytised”, phagocytosed?

Solved.

- Lines 474-478: “In the absence of reticulations, would photosynthetic organisms or lineages specialised into osmotrophy have repeatedly evolved in eukaryotes? The fact that endosymbiosis is behind the origin of photosynthetic eukaryotes indicates that it is at least much easier to become a photosynthetic organism by means of species-level reticulations than by de novo evolving the photosynthetic machinery”. I still do not think that the two processes, eukaryotic photosynthesis and osmotrophy, are fully comparable. There is horizontal gene transfer in the evolution of both, but in the case of photosynthesis, transferred genes are not enough. Photosynthesis is still carried out in the old cyanobacterium that has become an organelle (the plastid). Nothing of the kind happened in osmotrophs. I hardly doubt that if you transferred all photosynthetic genes to a eukaryotic nucleus, the resulting eukaryotic cell could perform photosynthesis. You need a different compartment, and that historically happened by the internalization of another cell. This is a very different case, with limited analogy to the evolution of osmotrophy.

We thank Reviewer 3 for pointing this out. We agree that framing our work around 'reticulate evolution' and comparing plastid endosymbiosis to HGT introduced a problematic analogy that distracted from our core findings.

As a result, we have removed the final section of the Discussion referenced by the Reviewer. To ensure the paper focuses strictly on the transition toward osmotrophy specialisation, we have also updated the title to “On the convergent origins of osmotrophic specialisation in eukaryotes”. Removing the reticulate evolution framework has also allowed us to provide a much clearer presentation about osmotrophy and the osmotrophic groups in the Introduction (first and second paragraphs, lines 41-69).
